# Systematically exploring repurposing effects of antihypertensives

**DOI:** 10.1002/pds.5491

**Published:** 2022-06-21

**Authors:** Zach Shahn, Phoebe Spear, Helen Lu, Sharon Jiang, Suki Zhang, Neil Deshmukh, Shenbo Xu, Kenney Ng, Roy Welsch, Stan Finkelstein

**Affiliations:** ^1^ Division of Healthcare and Life Sciences IBM Research Armonk New York USA; ^2^ MIT‐IBM Watson AI Lab Cambridge Massachusetts USA; ^3^ Department of Epidemiology and Biostatistics CUNY School of Public Health New York City New York USA; ^4^ Department of Electrical Engineering and Computer Science MIT Cambridge Massachusetts USA; ^5^ Engineering Systems MIT Institute for Data Systems, and Society Cambridge Massachusetts USA; ^6^ Operations Research and Statistics MIT Sloan School of Management Cambridge Massachusetts USA; ^7^ Division of Clinical Informatics Beth Israel Deaconess Medical Center Boston Massachusetts USA

**Keywords:** antihypertensives, causal inference, drug repurposing, hierarchical models

## Abstract

With availability of voluminous sets of observational data, an empirical paradigm to screen for drug repurposing opportunities (i.e., beneficial effects of drugs on nonindicated outcomes) is feasible. In this article, we use a linked claims and electronic health record database to comprehensively explore repurposing effects of antihypertensive drugs. We follow a target trial emulation framework for causal inference to emulate randomized controlled trials estimating confounding adjusted effects of antihypertensives on each of 262 outcomes of interest. We then fit hierarchical models to the results as a form of postprocessing to account for multiple comparisons and to sift through the results in a principled way. Our motivation is twofold. We seek both to surface genuinely intriguing drug repurposing opportunities and to elucidate through a real application some study design decisions and potential biases that arise in this context.

**Keypoints**

- Large observational healthcare databases have the potential to help identify promising drug repurposing candidates.
- We illustrate a high throughput screening approach based on target trial emulation, followed by hierarchical modeling for postprocessing.
- We discuss biases that might arise in this setting and how they should inform study design decisions.

**Plain language summary**

We consider the problem of how to search for drug repurposing opportunities (i.e., beneficial effects of drugs on outcomes that they were not designed or approved for). We use a large observational healthcare database to comprehensively explore potential repurposing effects of antihypertensive drugs. In lieu of randomized trials to assess each candidate repurposing opportunity (which would be the gold standard of evidence, but would be infeasible due to resource and time constraints), we use statistical techniques to *emulate* the randomized trials we wish we could conduct assessing effects of antihypertensives on each of 262 outcomes of interest. We then use another statistical technique called “hierarchical modeling” to sift through the results and identify the most promising repurposing candidates in a principled way. Our motivation for conducting this analysis is twofold. We seek both to surface genuinely intriguing drug repurposing opportunities and to elucidate through a real application some study design decisions and potential biases that arise in this setting due to the inability of statistical adjustment techniques to truly correct for the vagaries of observational data.

## 1 INTRODUCTION

Drug development over the past three decades has been characterized by what has been called “rational drug design.” Insights into the mechanism of action of a particular substance to prevent or alleviate disease first appear in peer reviewed biological science literature. Then, medicinal chemists, mostly in pharmaceutical companies, search systematically for molecules with properties conducive to use in the human body and then engineer changes to the molecule to maximize efficacy and tolerability. Mechanism driven drug development is limited by the requirement to know a precise molecular pathway at the outset, but there is every reason to believe that drug candidates work in ways not previously considered. For example, the full complement of mechanisms that explain all the indications for aspirin are still not well understood. And off label uses are common for many medicines.

Now, with increasing availability of voluminous sets of observational data and modern analytics, an empirical paradigm is also feasible. Opportunities exist to conduct exploratory empirical research in a high throughput fashion and then to consider findings in the context of what is known about a drug from traditional hypothesis driven studies. The potential value of such an approach is amplified by the current interest in repurposing medications, especially ones that have been widely used. In this article, we use a linked claims and electronic health record (EHR) database to comprehensively explore repurposing effects (i.e., effects on nonindicated outcomes) of antihypertensive drugs.

Our motivation is twofold. We seek both to surface genuinely intriguing drug repurposing opportunities and to elucidate through a real application some study design decisions and biases that arise in this context.

Within the antihypertensive therapeutic class, drugs from five mechanistic classes are candidate first line therapies for hypertension. Guidelines^1^ offer some criteria on how to choose among them, but there is still considerable variation in care. Past randomized trials^2^ and observational studies^3^, ^4^ have explored the comparative effectiveness (at lowering blood pressure and preventing adverse cardiovascular events) and safety profiles of antihypertensives in order to guide treatment decisions. While safety profiles capture the unintended negative consequences of these drugs, it is also of interest to assess their unintended *beneficial* effects. Unintended beneficial effects can also inform treatment decisions, as well as indicate potential repurposing opportunities or suggest pathways for drug development. Scattered studies have looked at repurposing effects of antihypertensives (e.g., Reference 5 considers effects on dementia), but not in a systematic fashion.

We follow a target trial emulation framework^6^, ^7^ to emulate five‐arm (one arm for each first line mechanistic class) randomized controlled trials estimating confounding adjusted effects of antihypertensives on each of 262 outcomes of interest. We then fit hierarchical models to the results as a form of postprocessing to account for multiple comparisons and to sift through the results in a principled way. Finally, we take stock of what we have learned, both about the repurposing potential of antihypertensives and the challenges of screening for repurposing opportunities.

## 2 METHODS

Ideally, any comparison of the effects of drugs on outcomes of interest would be based on a randomized trial. Multiple trials comparing antihypertensives have been conducted,^2^ but they did not study the full range of outcomes we are interested in. Therefore, we attempt to estimate effects of interest using observational data, following a target trial emulation framework.^6^, ^7^ We specify protocols for the pragmatic trials we wish we could conduct, and then construct cohorts and perform statistical analyses to generate effect estimates that, under certain causal assumptions, approximate results that would be observed in the trials.

### 2.1 Target trial protocols

We specified the protocols for our target trials as follows:

*Inclusion criteria*: To be eligible for inclusion in the study, a patient must have confirmed hypertension [defined as a systolic blood pressure (SBP) reading exceeding 140 mmHg or a diastolic blood pressure (DBP) reading exceeding 90 mmHg), be over 50 years old, be about to start an antihypertensive for the first time, and have no prior occurrence of the outcome of interest.

*Treatment arms*: Patients are randomly assigned to start an antihypertensive from one and only one of five first line mechanistic classes: angiotensin converting enzyme (ACE) inhibitors, angiotensin receptor blockers (ARBs), thiazide diuretics, beta‐adrenergic blocking agents (beta blockers), or calcium channel blockers. The choice of the specific drug within the class corresponding to the subject's treatment arm is left to the prescribing physician's discretion.

*Baseline*: Randomization and treatment initiation occur at the first time that all inclusion criteria are met.

*Follow‐up period*: Patients are followed until the earliest of occurrence of the outcome of interest, death, or loss to follow‐up.

### 2.2 Trial emulation

Using observational data, we sought to estimate intention to treat (ITT) effects from trials with the protocols above. In this context, ITT effects are effects of initiating an antihypertensive class but potentially adding another class, switching classes, or discontinuing antihypertensives altogether postbaseline according to usual care.

#### 2.2.1 Data

We used linked claims and EHR data from the IBM LCED database comprising data on over 6.5 million patients from across the United States who appear in both the MarketScan claims database^8^ and the Explorys EHR database.^9^ EHR data has the limitation that out of network care is not captured, while claims data has the shortcoming that most lab values and exam findings are not captured. The linked claims/EHR database greatly mitigates each of these limitations. It contains claims for diagnoses, prescriptions, and procedures that occur out of network as long as they were covered by insurance included in the database. It also contains in‐network lab results and exam findings.

#### 2.2.2 Cohort construction

For each outcome, we constructed a cohort of patients meeting target trial inclusion criteria from Section 2.1 corresponding to that outcome. (The only difference between cohorts for different outcomes is that different patients were excluded for having a previous outcome occurrence in their medical history.) Baseline was taken to be the first date that a prescription for an antihypertensive from one of the five classes of interest was filled. Patients were excluded if they had ever filled any antihypertensive prescription (from any class) previously, or if they simultaneously initiated drugs from multiple classes. Disease outcomes were grouped according to the Agency for Healthcare Research and Quality Clinical Classifications Software (CCS) that aggregates the International Classification of Diseases (ICD) diagnostic codes into clinically sensible groupings.^10^ Patients were excluded if any ICD code corresponding to the outcome CCS code appeared before baseline. Patients were also excluded if they had less than 1 year of continuous observation with insurance coverage in the database prior to baseline. This was to ensure that we could reliably confirm that they were new antihypertensive users who had never experienced the outcome and so that we could collect reliable data on confounders. Follow‐up was until the earliest of occurrence of the outcome of interest, death, or a break in continuous insurance coverage for any reason (i.e., loss to follow‐up).

#### 2.2.3 Inverse probability weighting to adjust for confounding and informative censoring

The baseline confounding variables we adjusted for included age, sex, calendar year, diabetes, prior stroke, prior acute myocardial infarction (AMI), prior heart failure, chronic kidney disease (as defined in Reference 11), most recent SBP recording, most recent DBP recording, and most recent BMI recording. These confounders comprise all variables mentioned anywhere in the American Heart Association guidelines^1^ for selecting a first line antihypertensive, plus calendar year to adjust for changing prescribing patterns.

For each antihypertensive class/outcome pair, we estimated the counterfactual cumulative incidence curve for the outcome had the full cohort been assigned to that class, adjusting for the confounding variables listed above using inverse probability weighting.^12^, ^13^ This required fitting four models for each class/outcome pair.

*Model 1 (treatment model)*: We fit a logistic regression model for probability of receiving an antihypertensive from the class of interest (as opposed to any other class, given membership in the cohort) conditional on the confounders.

*Model 2 (censoring model, denominator)*: We fit a Cox hazard model for loss to follow‐up (i.e., end of continuous insurance coverage) at time *t* given baseline confounders and treatment assignment.

*Model 3 (censoring model, numerator)*: We fit another Cox hazard model for loss to follow‐up at time *t* conditional only on treatment assignment.

From these three models we computed stabilized weights as follows. Let *a*_*i*_ indicate whether subject *i* was prescribed the antihypertensive class of interest (yes = 1) and *x*_*i*_ denote the values of subject *i*'s confounder variables. Let ${\hat{\pi }}_{t}\left(x_{i}\right)$ denote subject *i*'s estimated probability of receiving the treatment of interest given *x*_*i*_ as computed using Model 1. Let ${\hat{\pi }}_{c}^{\mathrm{den}}\left(x_{i},a_{i}\right)$ denote subject *i*'s estimated probability of not being censored for at least as long as they were actually observed in the data conditional on their covariates *x*_*i*_ and treatment assignment *a*_*i*_ as computed using Model 2. Let ${\hat{\pi }}_{c}^{\mathrm{num}}\left(a_{i}\right)$ denote subject *i*'s estimated probability of not being censored for at least as long as they were actually observed in the data conditional only on their treatment assignment *a*_*i*_ as computed using Model 3. Finally, let *p*_*a*_ denote the sample proportion of subjects prescribed the class of interest in the cohort. We then compute stabilized weights:

$$w_{i}=\frac{{\hat{\pi }}_{c}^{\mathrm{num}}\left(a_{i}\right)}{{\hat{\pi }}_{c}^{\mathrm{den}}\left(x_{i},a_{i}\right)}\times \frac{a_{i}p_{a}+\left(1-a_{i}\right)\left(1-p_{a}\right)}{a_{i}{\hat{\pi }}_{t}\left(x_{i}\right)+\left(1-a_{i}\right)\left(1-{\hat{\pi }}_{t}\left(x_{i}\right)\right)}.$$

*Model 4 (weighted outcome Cox model)*: Finally, we fit a weighted Cox proportional hazards model for the outcome, weighting each subject by *w*_*i*_ (above) with treatment assignment as the only predictor.

From Model 4, we compute the estimated counterfactual cumulative incidence curve. We estimated covariance matrices of the incidence curve estimates and corresponding confidence intervals for all outcomes of interest via bootstrap. Our analysis plan called for capping extreme weights, but the maximum stabilized weight in our analysis was 31 so in practice no cap was implemented.

### 2.3 Hierarchical modeling

We fit hierarchical models to our counterfactual incidence curve and bootstrap covariance matrix estimates to account for multiple comparisons, share strength across analyses, and use posterior probabilities to help interpret results.^14^, ^15^ Let $\delta_{i}^{j}$ denote the true counterfactual 1 year incidence rate for outcome *j* under antihypertensive treatment *i* with *j* indexing the 262 outcomes of interest and *i* indexing the five antihypertensive mechanistic classes of interest. Let ${\hat{\delta }}_{i}^{j}$ denote the corresponding estimates of the 1 year incidence rate for outcome *j* and treatment *i*. Because our estimators are regular and asymptotically linear, under the assumptions that we sufficiently adjusted for confounding and correctly specified models, we have that our estimates come from a jointly Gaussian sampling distribution centered at the true counterfactual rates, that is

(1) $${\hat{\delta }}_{1}^{j},\ldots ,{\hat{\delta }}_{5}^{j}∼N\left(\left[\delta_{1}^{j},\ldots ,\delta_{5}^{j}\right],\sum_{j}\right).$$

Given estimates ${\hat{\delta }}_{i}^{j}$ and a prior distribution on the counterfactual rate parameters $\delta_{i}^{j}$, we might obtain posterior distributions for $\delta_{i}^{j}$ taking our estimates as the “data.” We consider several models with alternative prior distributions on the $\delta_{i}^{j}$ parameters that pool information and shrink the $\delta_{i}^{j}$ parameters toward a common mean in different ways and to varying degrees.

**No Pooling** Under this model, we put independent weakly informative priors on each rate $\delta_{i}^{j}$:

$${\hat{\delta }}_{1}^{j},\ldots ,{\hat{\delta }}_{5}^{j}∼N\left(\left[\delta_{1}^{j},\ldots ,\delta_{5}^{j}\right],{\hat{\sum }}_{j}\right)$$

$$\delta_{i}^{j}∼\text{Uniform}\left(0,1\right)\;\text{for}\;\mathrm{all}\;i,j.$$

Here, we follow Gelman et al^14^ in making the simplifying modeling assumption that our bootstrap estimate ${\hat{\sum }}_{j}$ is the true covariance ∑_*j*_. Under this model, no rate estimate for any treatment/outcome pair ${\hat{\delta }}_{i}^{j}$ has any impact on the posterior distribution of the rate for any other treatment/outcome pair $\delta_{i^{\prime }}^{j^{\prime }}$. This is why it is called a “no pooling” model.

**Single Outcome Pooling** Under this model, we posit that the five rates for each outcome are drawn from a common distribution with a common outcome specific mean.

$${\hat{\delta }}_{1}^{j},\ldots ,{\hat{\delta }}_{5}^{j}∼N\left(\left[\delta_{1}^{j},\ldots ,\delta_{5}^{j}\right],{\hat{\sum }}_{j}\right)$$

$$\delta_{i}^{j}∼N\left({µ}_{j},\sigma_{j}\right)$$

$${µ}_{j}∼\text{Uniform}\left(0,0.3\right);\sigma_{j}∼\text{Uniform}\left(0,0.2\right)$$

Under this model, rate estimates for the same outcome are pooled together, since the assumption that they are generated by a common distribution makes outlier rates a priori less likely. Suppose for outcome *j* a particular treatment has a counterfactual rate estimate that is far from the other treatments, but the other treatments have rate estimates that are close together. Then the cluster of rate estimates that are close together will pull the estimate of *σ*_*j*_ closer to 0. A smaller *σ*_*j*_ will in turn pull the posterior of the true rate under the treatment with the disparate estimated rate closer to *μ*_*j*_, the common mean of the distribution postulated to have generated all the true rates. In this way, the single outcome pooling model mitigates the issue of multiple comparisons within outcomes. However, rate estimates for one outcome have no impact on posterior rates for other outcomes under this model.

**All Outcome Pooling** Under this model, we posit that there is a base rate corresponding to each outcome, and for each treatment/outcome pair the ratio of the counterfactual incidence rate to the base rate is drawn from a common distribution centered at 1.

$${\hat{\delta }}_{1}^{j},\ldots ,{\hat{\delta }}_{5}^{j}∼N\left(\left[\delta_{1}^{j},\ldots ,\delta_{5}^{j}\right],{\hat{\sum }}_{j}\right)$$

$$\alpha_{i}^{j}∼N\left(1,\sigma \right)$$

$${µ}_{j}∼\text{Uniform}\left(0,0.3\right);\sigma ∼\text{Uniform}\left(0,0.2\right)$$

$$\delta_{\mathrm{ij}}=\mu_{j}\alpha_{i}^{j}$$

Under this model, if most treatments do not impact most outcomes, then the posterior distribution for *σ* will be small. This will pull the posteriors of all $\delta_{i}^{j}$ toward their outcome specific base rates *μ*_*j*_ for all *i* and *j*. In this way, the all outcome pooling model mitigates the issue of multiple comparisons both within and across outcomes.

## 3 RESULTS

Our systematic approach produced estimated counterfactual cumulative incidence curves under each of five treatments for 262 outcomes along with bootstrap covariance estimates. Furthermore, we generated posterior joint distributions of the 1‐year incidence rate (one time point along the cumulative incidence curve) under the no pooling, single outcome pooling, and all outcome pooling models specified above. Our full results are available in the supplementary materials.

We used a multipronged strategy to sift through and interpret key takeaways from all of these estimates. For critical efficacy outcomes (AMI, stroke, and heart failure), we examined results in full. For repurposing outcomes, we looked for drug class/outcome pairs where the outcome incidence rate under the drug class “stood out from the pack” in accordance to criteria we formalize below. Beyond the results produced by this automated and somewhat principled approach, we also highlighted other results that are of interest because, once additional context was brought to bear, they seemed to represent either promising opportunities or potential sources of bias in the data.

### 3.1 Cohort description

Baseline characteristics of the study cohort are shown in Table 1. 12 555 patients met inclusion criteria for at least one target trial. We note that there were significant differences between prebaseline disease rates across study arms. Patients on thiazide diuretics were healthiest, having the lowest rates of prior AMI, diabetes, heart failure, kidney disease, and stroke. Patients on beta blockers had the highest rates of prebaseline adverse cardiovascular events (AMI, heart failure, and stroke), while patients on ACE inhibitors and ARBs had higher baseline rates of diabetes. Age, blood pressure, and BMI were similar across treatment arms at baseline. Table 2 contains the inverse probability of treatment weighted means of each baseline covariate in Table 1. That these means are similar across treatment arms indicates that we successfully adjusted for observed confounding by the variables in Table 1.

**TABLE 1:** Baseline characteristics of study cohort

	Overall	ACE	ARB	Beta Blocker	CCB	Thiazide Diuretic
Characteristics	(n = 12 555)	(n = 5093)	(n = 1375)	(n = 2661)	(n = 1701)	(n = 1725)
sex = Female, n (%)	6551 (52.2)	2447 (48.0)	687 (50.0)	1395 (52.4)	884 (52.0)	1138 (66.0)
AMI, n (%)	319 (2.5)	79 (1.6)	19 (1.4)	164 (6.2)	44 (2.6)	13 (0.8)
Diabetes, n (%)	2278 (18.1)	1129 (22.2)	283 (20.6)	441 (16.6)	256 (15.0)	169 (9.8)
Heart failure, n (%)	467 (3.7)	126 (2.5)	35 (2.5)	208 (7.8)	75 (4.4)	23 (1.3)
Stroke, n (%)	1085 (8.6)	353 (6.9)	102 (7.4)	329 (12.4)	203 (11.9)	98 (5.7)
CKD, n (%)	374 (3.0)	109 (2.1)	43 (3.1)	112 (4.2)	73 (4.3)	37 (2.1)
BMI, mean (SD)	28.4 (9.6)	28.7 (9.7)	28.8 (10.2)	27.2 (9.4)	28.0 (8.9)	29.4 (9.6)
Diastolic BP, mean (SD)	87.1 (11.5)	88.1 (11.0)	87.7 (11.5)	85.0 (11.5)	86.5 (12.4)	87.2 (11.6)
Systolic BP, mean (SD)	154.1 (14.3)	154.5 (14.5)	154.0 (14.4)	152.6 (13.3)	155.4 (14.7)	154.0 (14.5)
Age, median [Q1,Q3]	61.0 [56.0,67.0]	60.0 [55.0,65.0]	60.0 [55.0,66.0]	62.0 [57.0,71.0]	62.0 [56.0,71.0]	60.0 [55.0,65.0]

*Note*: Summaries are given for the overall cohort and for each treatment assignment arm.

Abbreviations: AMI, acute myocardial infarction; CKD, chronic kidney disease; BMI, body mass index; BP, blood pressure; eGFR, estimated glomerular filtration rate.

**TABLE 2:** Inverse probability of treatment weighted baseline characteristics of the study cohort

Characteristics	ACE	ARB	Beta Blocker	CCB	Thiazide Diuretic
Sex = Female (%)	52.3	52.2	52.7	51.8	51.4
AMI (%)	2.7	2.4	2.6	2.7	2.8
Diabetes (%)	17.9	18.8	18.4	18.0	19.0
Heart failure (%)	3.8	3.6	3.8	4.0	4.6
Stroke (%)	8.8	8.7	9.1	8.7	9.2
CKD (%)	3.1	3.0	3.1	3.0	2.8
BMI (mean)	28.4	28.4	28.3	28.4	28.6
Diastolic BP (mean)	87.1	87.2	87.0	87.2	87.2
Systolic BP (mean)	154.2	154.1	154.1	154.2	154.4
Age (mean)	62.9	62.7	63.0	62.8	63.0

*Note*: For a given baseline variable, similar weighted rates or means across study arms suggests adequate adjustment for confounding by that variable.

Abbreviations: AMI, acute myocardial infarction; CKD, chronic kidney disease; BMI, body mass index; BP, blood pressure; eGFR, estimated glomerular filtration rate.

### 3.2 Comparative effectiveness results

**FIGURE 1:**
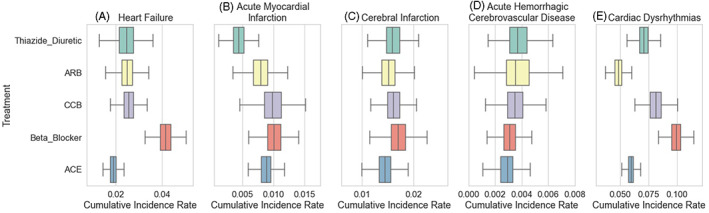
Comparative effectiveness results of the antihypertensive treatments for A, heart failure, B, acute myocardial infarction, C, ischemic stroke, D, hemorrhagic stroke, and E, cardiac dysrhythmias. The cumulative incidence rate is measured at 1 year after baseline. The results use the single outcome pooling method

ACE inhibitors were estimated to lead to the lowest rates of heart failure onset and beta blockers the highest rates of heart failure onset relative to the other antihypertensives (Figure 1A). Thiazides were estimated to be most effective at preventing acute myocardial infarction (Figure 1B). There were no significant differences between medications in estimated effect on ischemic or hemorrhagic stroke (Figure 1C,D). ARB and ACE were estimated to lead to the lowest rates of cardiac dysrhythmia onset and beta blockers the highest rates of cardiac dysrhythmia onset relative to the other antihypertensives (Figure 1E).

### 3.3 Repurposing results

For the No Pooling, Single Outcome Pooling, and All Outcome Pooling models, we identified drug class/outcome pairs such that: (A) there was a high posterior probability that the 1‐year counterfactual incidence of the outcome was lowest for that drug; and (B) the ratio between the posterior mean 1‐year incidences under that drug and the drug with the second lowest estimated counterfactual 1 year cumulative incidence was ≤0.8. Table 3 shows the nine outcomes meeting these criteria when effect estimates and posterior probabilities were computed under the No Pooling model. The starred (*) outcomes also met these criteria under the Single Outcome Pooling model. Outcomes with daggers (**) further met the criteria under the All Outcome Pooling model.

**TABLE 3:** Screening raw results for unexpected beneficial effects and repurposing opportunities under the no pooling model

Outcome	Thiazide	ARB	CCB	Beta Blocker	ACE	RR (*p*)
Acute myocardial infarction*	**0.4** (0.2,0.6)	0.8 (0.4,1.2)	1.1 (0.6,1.5)	1.1 (0.7,1.4)	0.9 (0.7,1.1)	0.52 (*p* = 0.96)
Pulmonary heart disease*	1.2 (0.5,1.9)	**0.4** (0.2,0.7)	1.3 (0.9,1.8)	1.2 (0.8,1.5)	0.9 (0.6,1.1)	0.52 (*p* = 0.97)
Aneurysms	**0.5** (0.2,0.8)	1.0 (0.6,1.5)	1.0 (0.6,1.4)	1.1 (0.7,1.4)	0.9 (0.6,1.2)	0.55 (*p* = 0.94)
Varicose Veins*,**	1.6 (1.1,2.2)	1.3 (0.8,1.9)	1.1 (0.7,1.5)	1.3 (0.9,1.6)	**0.7** (0.5,0.9)	0.65 (*p* = 0.93)
Regional enteritis, ulcerative colitis	0.9 (0.4,1.3)	**0.4** (0.1,0.7)	0.8 (0.4,1.2)	1.2 (0.8,1.6)	0.8 (0.6,1.0)	0.55 (*p* = 0.91)
Schizophrenia, psychosis*	0.8 (0.4,1.3)	**0.3** (0.1,0.7)	1.1 (0.4,1.2)	1.4 (0.8,1.6)	0.7 (0.6,1.0)	0.55 (*p* = 0.94)
Gastrointestinal hemorrhage	3.0 (2.2,3.7)	**2.0** (1.4,2.5)	3.3 (2.4,4.1)	2.9 (2.3,3.6)	2.6 (2.2,3.1)	0.77 (*p* = 0.92)
Paralysis*,**	0.7 (0.3,1.1)	**0.2** (0.1,0.4)	0.8 (0.4,1.1)	0.7 (0.4,1.0)	0.6 (0.4,0.8)	0.38 (*p* = 0.97)
Symptoms of mental and substance use conditions	1.6 (0.9,2.4)	1.6 (1.0,2.1)	1.4 (0.9,1.9)	1.4 (1.0,1.8)	**0.9** (0.7,1.1)	0.66 (*p* = 0.92)

*Note*: Numbers are percents. RR is risk ratio of lowest to second lowest rate. *p* is pseudo‐posterior probability that the lowest estimated rate is truly the lowest. The bold faced value in each row corresponds to the drug (column) estimated to have a protective effect for the condition corresponding to that row.

* Under the single outcome pooling model.

** Under the all outcome pooling model.

## 4 DISCUSSION

### 4.1 Comparative effectiveness

Agreement between our comparative effectiveness results and past studies was mixed. Regarding stroke prevention, the literature is fairly consistent and our results confirm that there are no significant differences between antihypertensive classes. Our finding that ACE inhibitors were most effective at preventing heart failure contradicts the ALLHAT randomized trial,^2^ which found that thiazide diuretics were most effective in a high cardiovascular risk population. It also contradicts two observational studies by the OHDSI group^3^, ^4^ which, respectively, found that thiazide diuretics were superior and that ARBS and ACE inhibitors did not significantly differ from each other. That beta blockers lead to higher incidence of heart failure onset (despite being effective at treating existing heart failure) is consistent with past studies including ALLHAT.^2^ The finding that thiazide diuretics prevent AMI relative to other classes again contradicts the ALLHAT trial,^2^ where no significant differences across treatments were found for this outcome. However, the OHDSI group^3^ did also find that thiazide diuretics were superior at preventing AMI.

### 4.2 Design considerations for repurposing screening studies

There are pros and cons to the active comparator design when searching for repurposing opportunities. A drawback is that ACE inhibitors and ARBs work through closely related pathways, so comparing them to each other could in theory mask shared effects. For example, both classes are known to prevent cardiac dysrhythmias,^16^ and indeed both are estimated to prevent cardiac dysrhythmias in our analysis compared with the other classes (Figure 1E). If not for ACE inhibitors, ARBs would more clearly stand out from the pack for this outcome.

A benefit of the active comparator design is that comparatively beneficial effects on new outcomes are unlikely to be indirect effects of the drug's indicated purpose (reducing blood pressure), which is shared by all drugs being compared. Thus, it is more likely in an active comparator design that the mechanism of action behind an observed repurposing effect might also apply in patients without the indication for the drug. However, it is still possible (though not a priori likely) that even if the mechanism of action for a repurposing effect is not primarily mediated by treatment of the drug's indication, effects would only appear in a population where the indication (hypertension in this instance) was present.

Another study design decision was to consider mechanistic classes as opposed to specific drugs as the treatments in our analyses. The primary motivation for this choice was sample size. If a class did have a repurposing effect, we might not have the power to detect it in the individual drugs in the class. Of course, if an individual drug has a repurposing effect not shared across its mechanistic class, we risk diluting and failing to detect that effect at the class level. Since drugs with similar mechanisms of action are likely to have similar repurposing effects, we believed the benefits of larger treatment arms in our RCT emulations outweighed the risk.

Looking at prevention as opposed to treatment of disease also has pluses and minuses. The main drawback is of course that one might hope to find cases in which a drug can be used for treatment of a condition postonset, but we are only directly assessing preventive effects. For example, beta blockers are estimated to lead to increased incidence of heart failure compared with other antihypertensives in our cohort of patients who have never had a heart failure diagnosis in the past. And indeed beta blockers are known to precipitate heart failure (which we can take as validation of our results). But beta blockers are also effective treatments for chronic heart failure post onset. This is an extreme example of how conflating prevention of onset with potential for treatment after onset can yield misleading results.

But assessing postonset effects on a range of conditions can be challenging because it requires defining and extracting from the data separate relevant outcomes marking disease progression for each condition. It can also be challenging to adjust for measures of disease progression as potential confounders. These challenges of course do not arise if we restrict our cohort to a preonset population.

Furthermore, a treatment estimated to have a preventive effect on an outcome in our study design might also be a promising candidate to treat that outcome postonset. This is because ‘prevention’ in our setting really means prevention of *diagnosis*. The mechanism for prevention of diagnosis will likely often be early postonset but predetection treatment. For example, a drug that delays cancer diagnosis may do so by slowing tumor growth, which would be desirable postdiagnosis as well.

Another study design decision was to select a therapeutic class and search across outcomes as opposed to selecting outcomes and searching across a wide range of drugs as Laifenfeld et al.^17^ did. One reason we made this decision is that it is more straightforward to identify a sufficient confounding adjustment set that can be used for the same treatments across outcomes than vice versa. The main drivers of treatment decisions can be ascertained by consulting guidelines and medical expertise, and they suffice for confounding adjustment for any outcome. The full set of variables prognostic for an outcome is much harder to identify, making it very difficult to construct an outcome‐specific adjustment set that would perform reasonably well across a wide range of treatments. VanderWeele^18^ makes similar arguments in favor of outcome wide analysis.

Finally, our high throughput approach required us to use crude outcome definitions. Outcome occurrences were defined by the appearance of ICD codes alone, when additional criteria such as hospitalization could have significantly improved accuracy in many cases. Furthermore, we grouped outcomes into categories defined by CCS codes. These groupings were designed to be clinically meaningful but can be suboptimal for exploring repurposing effects. Some degree of outcome grouping is required for high throughput screening to have adequately powered studies, but it is difficult to reason a priori about which groupings are appropriate, and the groupings can both impact results and lead to challenges in interpretation. For example, our analyses estimated that ACE inhibitors prevent onset of the CCS code labeled “anxiety and fear related disorders” (to a degree that just missed our strict selection criteria). This category includes a host of phobias (e.g., ICD codes for agoraphobia and arachnophobia) as well as anxiety disorders (e.g., ICD codes for generalized anxiety disorder and panic disorder). Estimating the effect of ACE inhibitors on a more granular level, we found that the effect was almost entirely driven by anxiety disorders, with very low incidence of phobias in any treatment arm of the cohort.

### 4.3 Observations on bias patterns in repurposing screening studies

When we search for causal signals in observational data, we will inevitably dredge up some relationships driven by confounding or measurement error. This is particularly likely to occur for outcomes with known relationships to the drugs under study (e.g., side effects or indications). We consider a few examples below.

ACE‐inhibitors are known to lower both red and white blood cell counts as a side effect. However, in our analyses, ACE inhibitors were found to be protective against white blood cell diseases, immune disorders, and nutritional anemia. There are several possible explanations for these results. The white blood cell and immune effects could be due to confounding if clinicians could tell from information not in our data that certain patients were at risk for low white blood cell counts and therefore did not prescribe ACE inhibitors. It is even possible that many instances of these outcomes were already present prior to drug initiation but only appeared in our data afterward (leading us to fail to exclude such cases from the cohort as intended and instead wrongly count them as new occurrences of the outcome). The apparent protective effects of ACE inhibitors could be due to fewer unrecorded (but known to physicians) prebaseline instances of these outcomes in the ACE cohort (since patients known to have low white blood cell counts would not be given ACE inhibitors). Alternatively, ACE inhibitors may have had real protective effects against disorders that result from too *many* white blood cells or too *strong* an immune responses, which are lumped into the same CSS categories as diseases from too few white blood cells or too weak immune response. Of course, investigation of outcomes at a finer granularity could test this latter hypothesis.

Regarding nutritional anemia, it is possible that anemia cases in patients taking ACE inhibitors are assumed to be due to the known side effect of the medication and therefore not coded as nutritional. ACE inhibitors would appear protective against nutritional anemia when in reality ACE inhibitors only affect its coding. This would be an example of outcome measurement error associated with treatment assignment biasing an effect estimate.

Patterns like these might be expected to appear frequently for known side effects. As further examples, ACE inhibitors were also found to be protective for “other specified joint diseases” even though joint pain is a known side effect, and thiazides were estimated to be protective for diabetes, also a known side effect.^19^ However, we note that when assessing repurposing opportunities on outcomes that are not *known* side effects (or indications) of any of the drugs, the danger of such biases driving results is greatly reduced. This is because considerations pertaining to such outcomes do not typically relate to treatment decisions, reducing the likelihood of strong confounding. For evidence that strong confounding was not widespread in our repurposing effect estimates, we refer the reader to forest plots of effect estimates across all treatments and outcomes in Appendix 3. Because true repurposing effects are rare, we would expect credible intervals for most repurposing effect estimates to contain no effect if strong confounding is not widespread. Indeed, this is what we see. See Appendix 3 for further details.

### 4.4 Future work

Future work could improve on the screening procedure we implemented in multiple ways. First, we used CCS outcome groupings and mechanistic class treatment groupings to avoid treatment arms with too few outcome occurrences to make precise effect estimates. Multilevel hierarchical models properly implemented should enable strength sharing across drugs and outcomes to mitigate this problem, which might in turn enable detection of more narrowly defined repurposing effects, that is, effects of specific drugs on well‐defined outcomes. Second, all our results depended for their validity on the absence of strong unobserved confounding. Other methods for effect estimation, such as instrumental variables^5^, ^20^ or the recently developed proximal inference,^21^, ^22^ depend on alternative assumptions. Triangulating results from multiple methods (each depending on different distinct assumptions) could significantly improve reliability.

## 5 CONCLUSION

We have illustrated a principled approach to high throughput screening for drug repurposing opportunities using observational data. While our study design decisions entailed tradeoffs and we identified several clear instances of bias, we also identified some intriguing opportunities. For example, we found some suggestive evidence of efficacy of ACE inhibitors against mood disorders. A cursory literature search reveals physiological arguments that the renin‐angiotensin system (on which ACE inhibitors act) plays an important role in mood disorders, as well as case studies dating back to the 1980s of ACE inhibitors appearing to treat major depression.^23^, ^24^, ^25^, ^26^ Another cursory literature search surfaced that angiotensin sustains paralysis inducing brain inflammation in mice,^27^ possibly lending some credence to our finding that ARBs might prevent paralysis. Perhaps these lines of thought deserve additional attention? Certainly, triangulation would make the case more compelling (future work). But the more general point is that perhaps empirical evidence from observational studies can fruitfully help to stimulate thought and direct expert attention towards promising directions at little cost.

## CONFLICT OF INTEREST

The authors declare no conflict of interest.

## ETHICS STATEMENT

No ethics committee approval was required for this study.

## Supporting information


**Appendix S1** Supporting Information
