# Supplementary material for: Systematically exploring repurposing effects of antihypertensives
Source: Pharmacoepidemiol Drug Saf. 2022 Jun 21;31(9):944–52. doi: 10.1002/pds.5491 (PMC9545793; doi:10.1002/pds.5491)

Guide to Supplementary Materials for "Systematically Exploring Repurposing Effects of Antihypertensives"

While the main text highlights some interesting findings, the supplementary materials include the full results of our analyses. The bootstrap estimates for counterfactual incidence rates for each outcome under each treatment can be found at github.com/zshahn/antihypertensive_repurposing/bootstrap_results/. R code for fitting the Single Outcome Pooling Model to these bootstrap estimates and summarizing the results is at github.com/zshahn/antihypertensive_repurposing/single_outcome_model.R, and R code for fitting the All Outcome Pooling Model and summarizing results is at github.com/zshahn/antihypertensive_repurposing/all_outcome_pooling_model.R. A spreadsheet with descriptors of all outcome CCS codes and the ICD codes comprising them can be found at github.com/zshahn/antihypertensive_repurposing/dx_to_ccsr_mapping_applicable.csv.

Appendix 1 (starting on page 2): We include plots of posterior distributions of counterfactual incidence ratios under the All Outcome Pooling model for each outcome and under each treatment. Each plot corresponds to a single target trial and outcome. The outcomes are presented in alphabetical order of their CSS codes.

Appendix 2 (starting on page 266): We include plots of posterior distributions of counterfactual incidence ratios under the Single Outcome Pooling model defined in Section 2.3 for each outcome and under each treatment. Each plot corresponds to a single target trial and outcome. The outcomes are presented in alphabetical order of their CSS codes.

Appendix 3 (starting on page 530): We include forest plots corresponding to each anti-hypertensive class. Each forest plot contains 95% posterior credible intervals for one year counterfactual incidence rate ratios under the All Outcome Pooling Model specified in Section 2.3 for a single treatment across all outcomes. Each counterfactual IRR in a plot is the ratio between the estimated counterfactual incidence under the drug of interest (in the filename and the title of the plot) and the average of the estimated counterfactual incidences under the other four treatments. Scrolling down each plot indicates that significant results (i.e. credible intervals excluding 1) are relatively rare. To the degree that the reader believes that repurposing effects and side effects comprise a small proportion of all drug-outcome pairs, these plots can be construed as an approximate graphical negative control analysis, reassuring us that our methodology does not produce a large number of false positive repurposing effects. A large proportion of the positive results are known side effects, known repurposing effects, or biased estimates of known side effects as discussed in Section 4 of the main text.

APPENDIX 1

Summary Plots of Counterfactual Cumulative Incidence Rate Estimates Under the All Outcome Pooling Model From Section 2.3

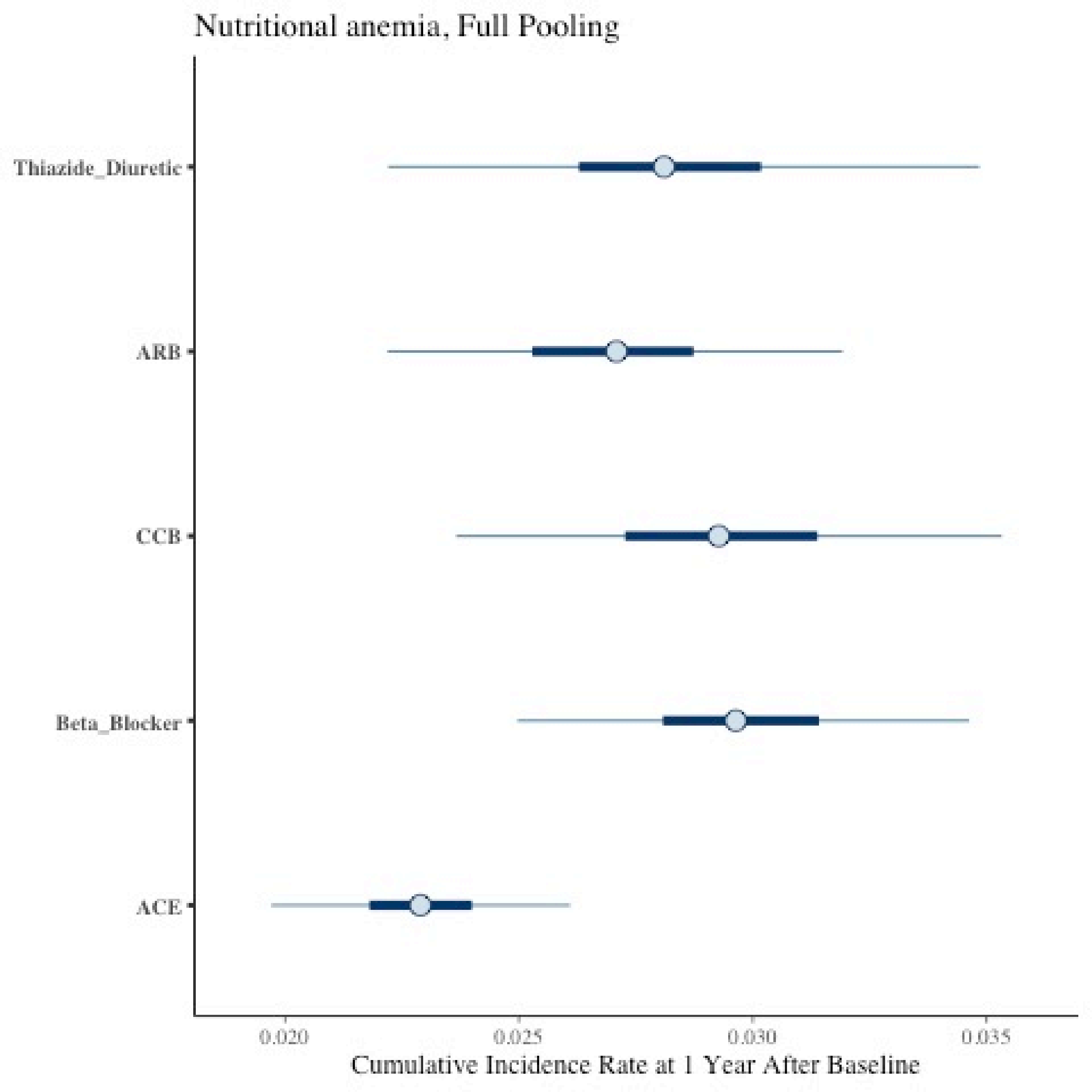
Nutritional anemia, Full Pooling
Thiazide_Diuretic
ARB
CCB
Beta_Blocker
ACE
0.020
0.025
0.030
0.035
Cumulative Incidence Rate at 1 Year After Baseline

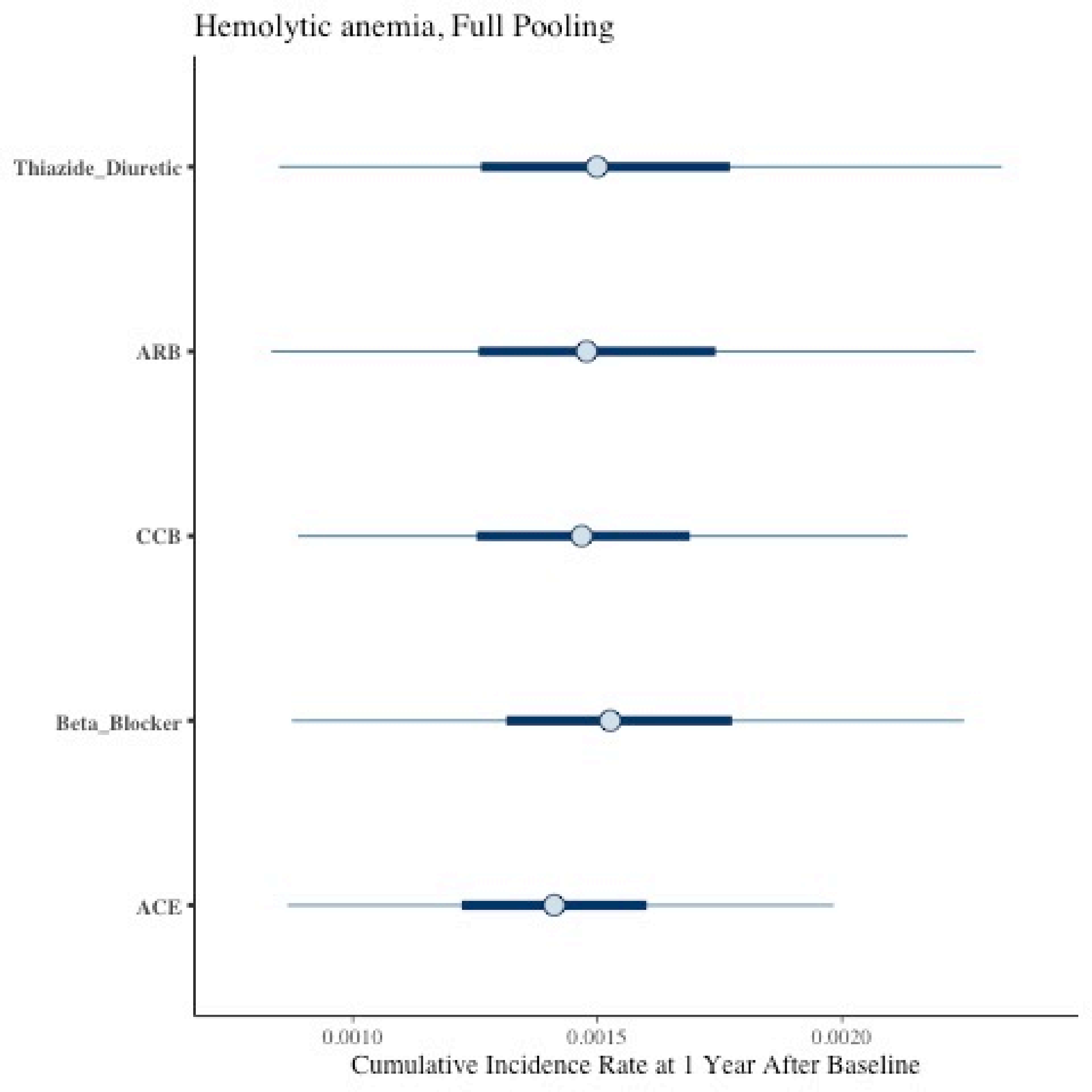

Hemolytic anemia, Full Pooling
Thiazide_Diuretic
ARB
CCB
Beta_Blocker
ACE
0.0010
0.0015
0.0020
Cumulative Incidence Rate at 1 Year After Baseline

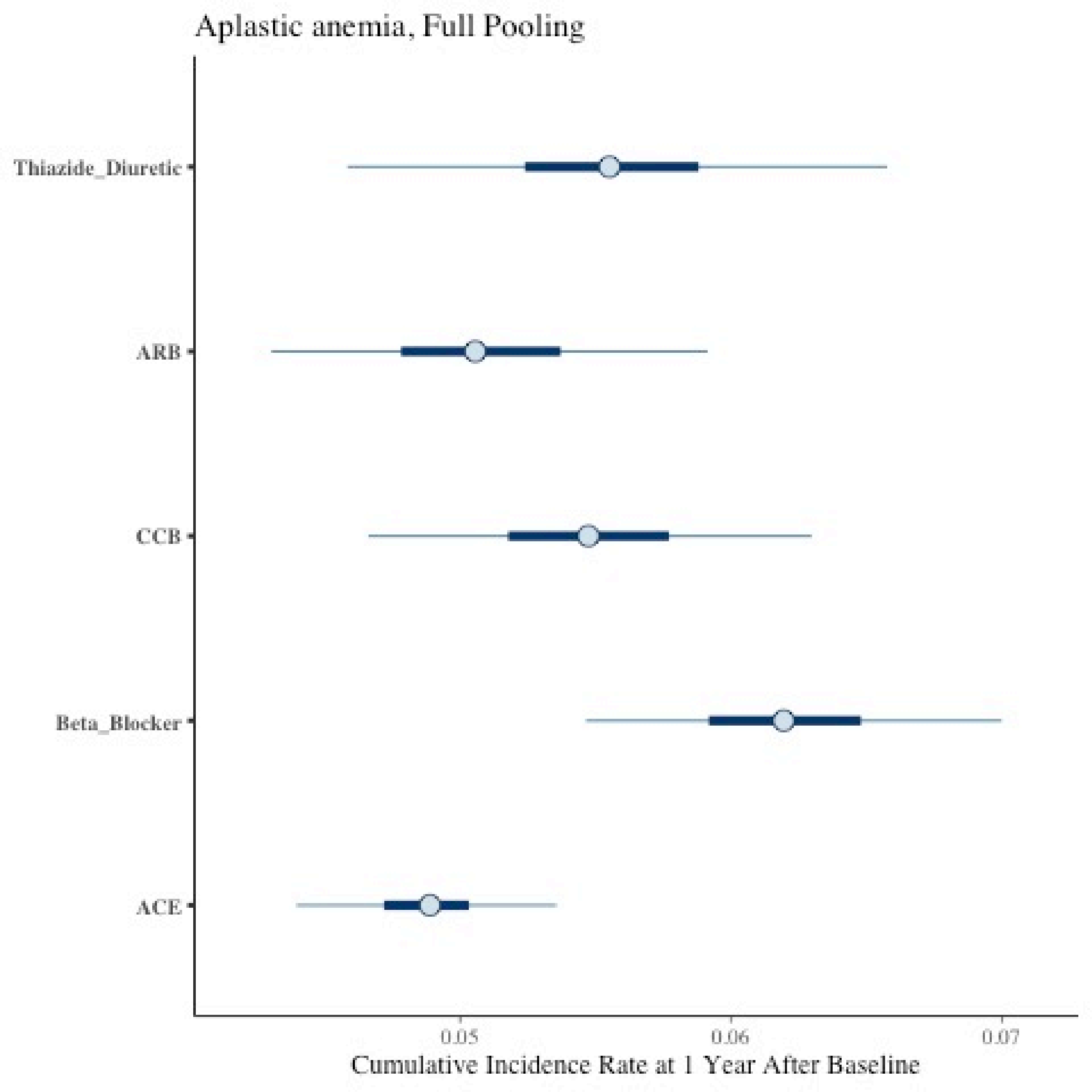

Aplastic anemia, Full Pooling
Thiazide_Diuretic
ARB
CCB
Beta_Blocker
ACE
0.05
0.06
0.07
Cumulative Incidence Rate at 1 Year After Baseline

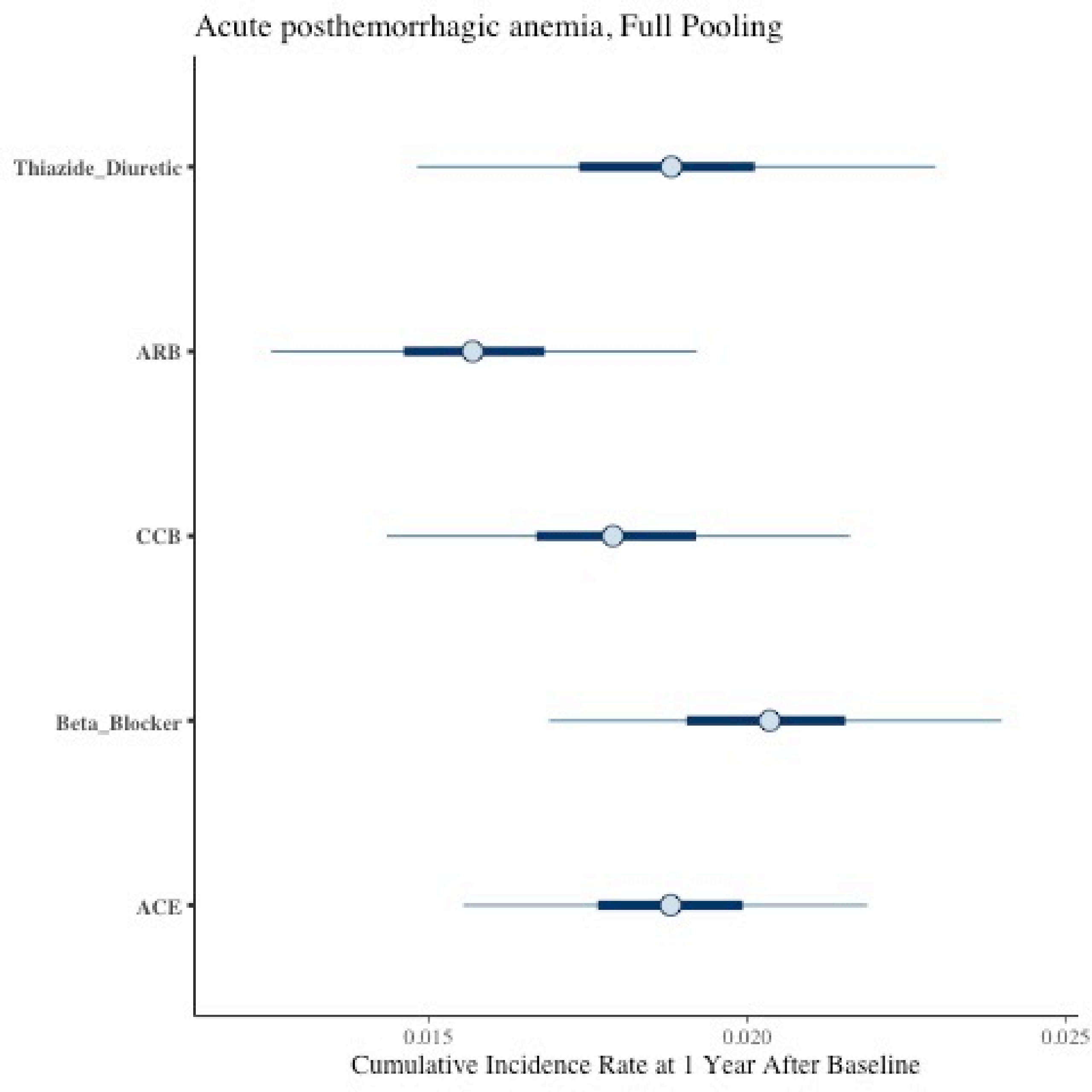

Acute posthemorrhagic anemia, Full Pooling
Thiazide_Diuretic
ARB
CCB
Beta_Blocker
ACE
0.015
0.020
0.025
Cumulative Incidence Rate at 1 Year After Baseline

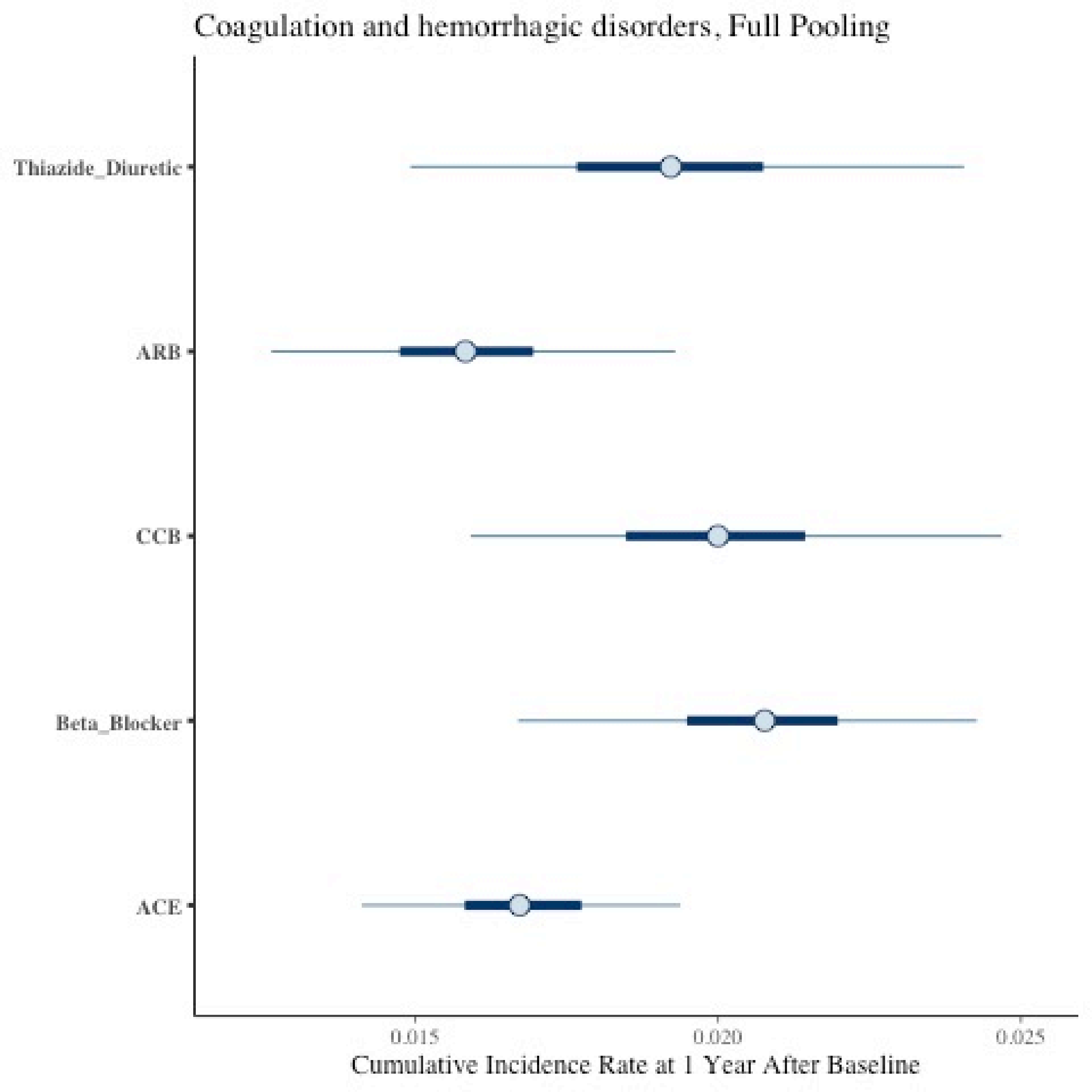
Coagulation and hemorrhagic disorders, Full Pooling
Thiazide_Diuretic
ARB
CCB
Beta_Blocker
ACE
0.015
0.020
0.025
Cumulative Incidence Rate at 1 Year After Baseline

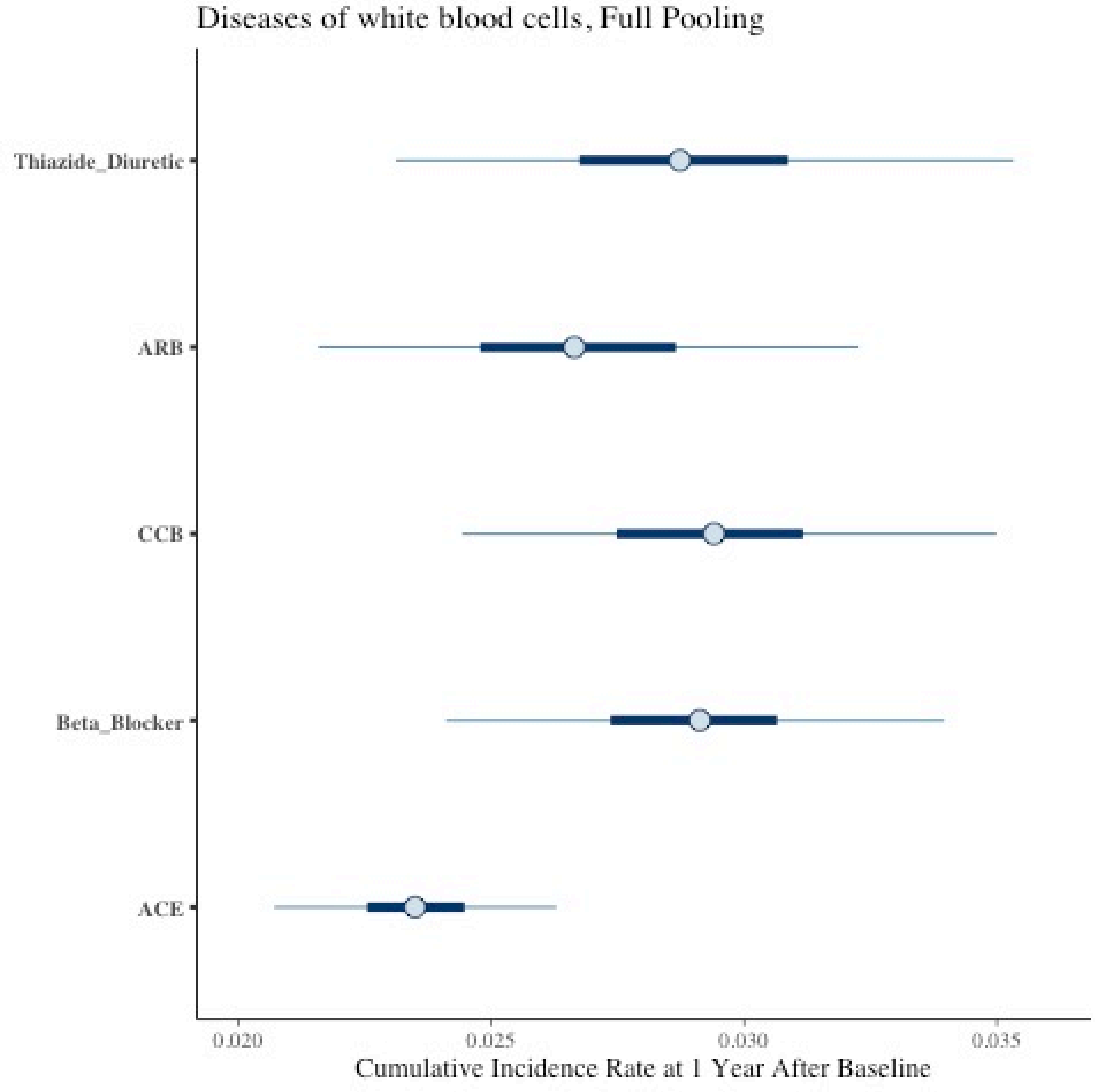

Diseases of white blood cells, Full Pooling
Thiazide_Diuretic
ARB
CCB
Beta_Blocker
ACE
0.020
0.025
0.030
0.035
Cumulative Incidence Rate at 1 Year After Baseline

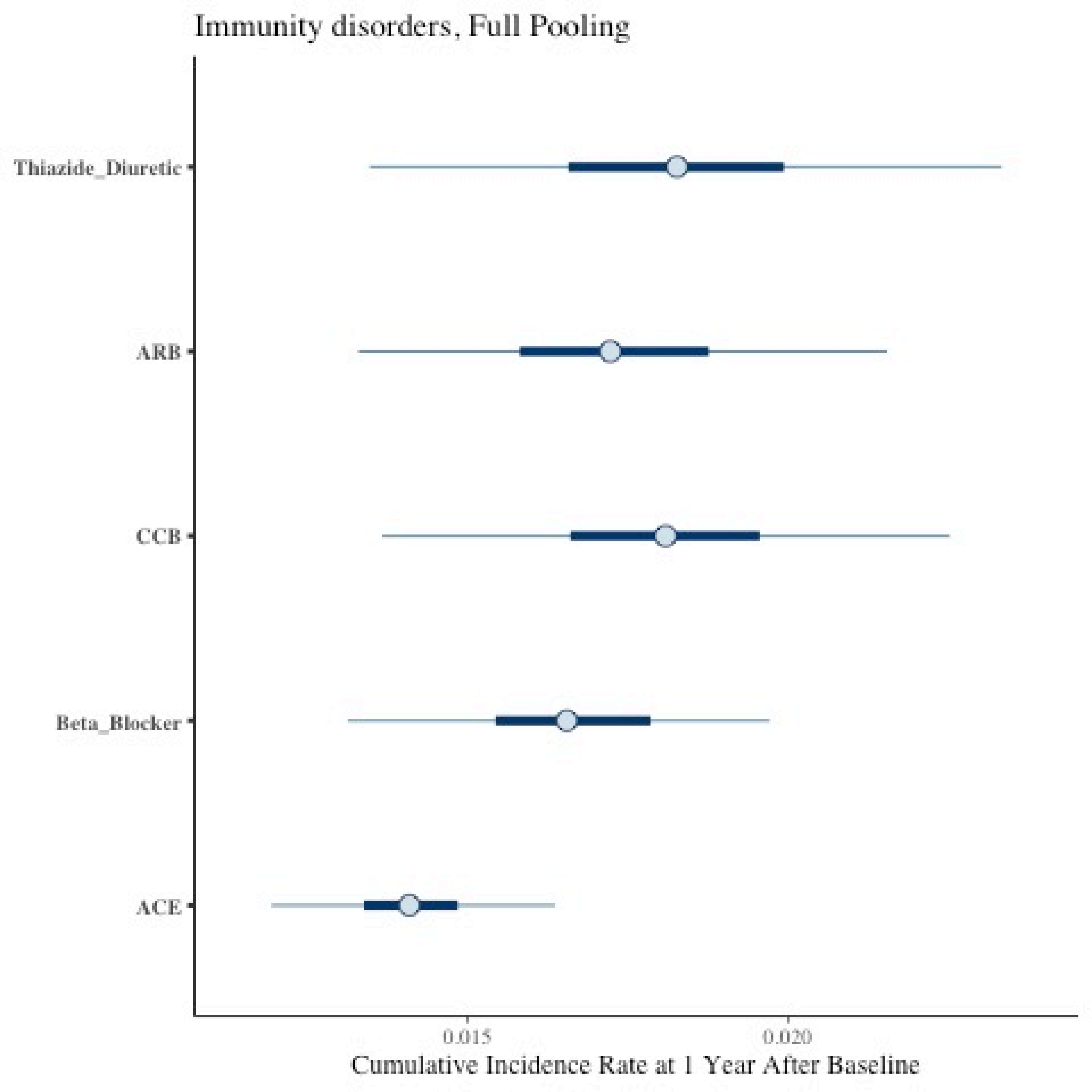

Immunity disorders, Full Pooling
Thiazide_Diuretic
ARB
CCB
Beta_Blocker
ACE
0.015
0.020
Cumulative Incidence Rate at 1 Year After Baseline

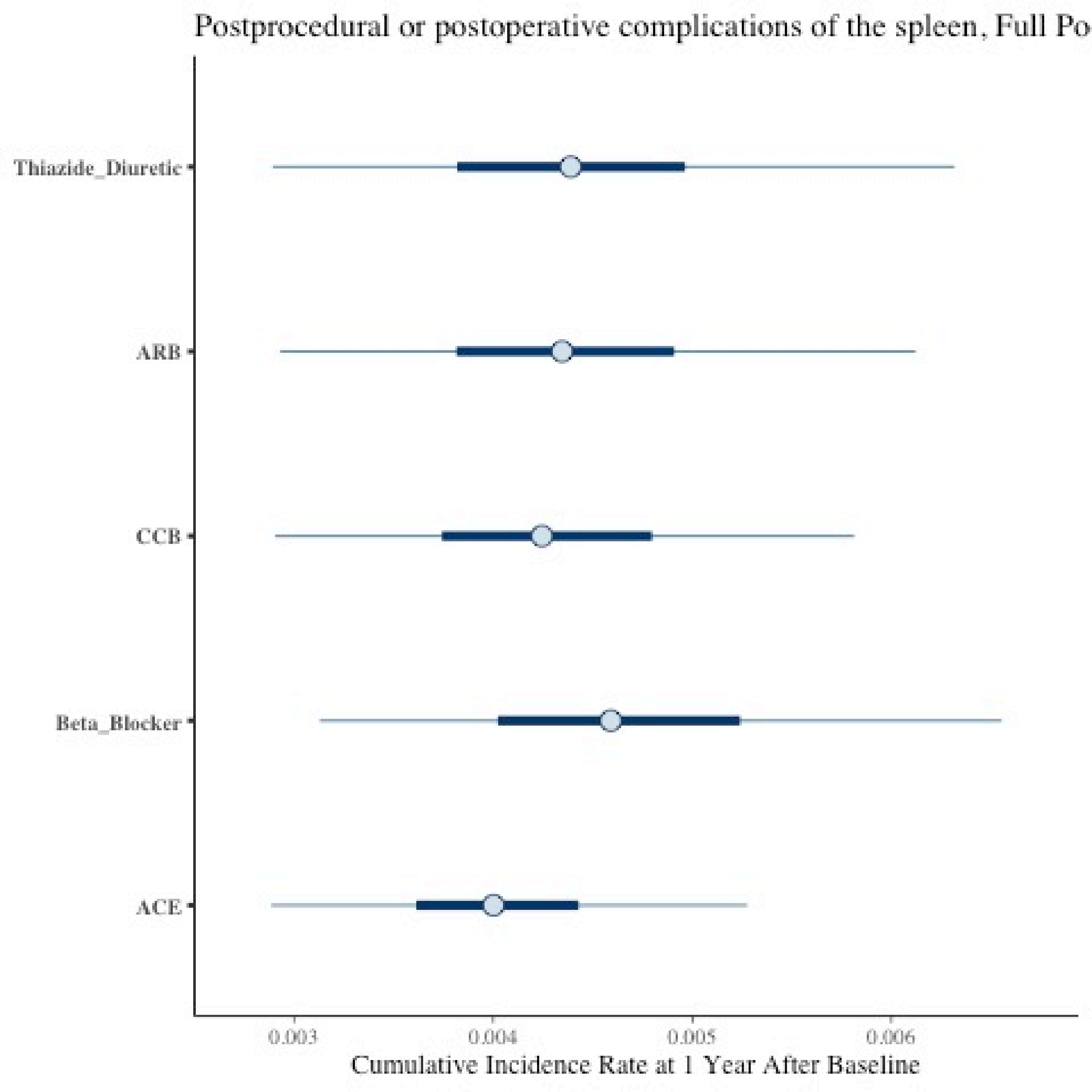

Postprocedural or postoperative complications of the spleen, Full Po
Thiazide_Diuretic
ARB
CCB
Beta_Blocker
ACE
0.003
0.004
0.005
0.006
Cumulative Incidence Rate at 1 Year After Baseline

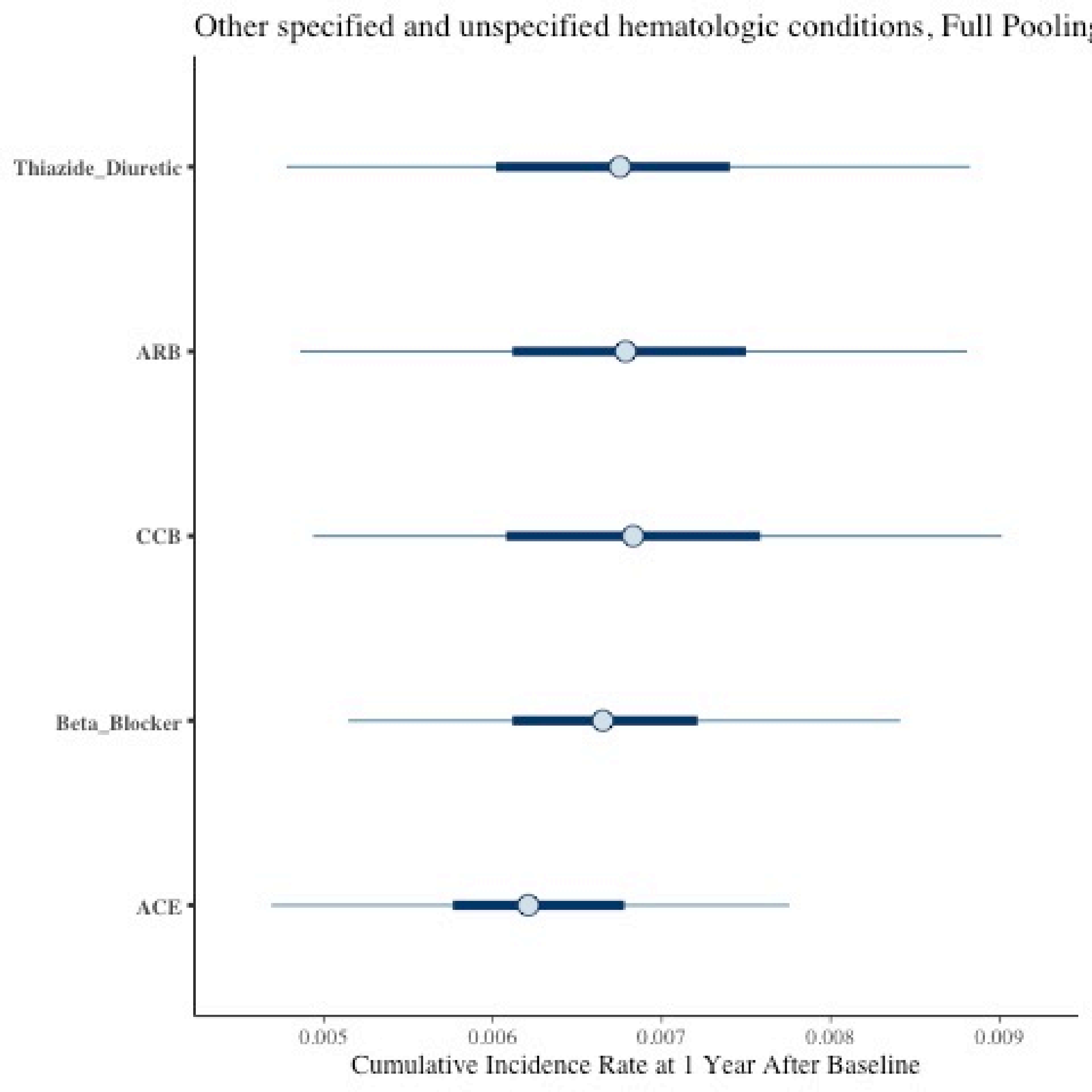

Other specified and unspecified hematologic conditions, Full Pooling
Thiazide_Diuretic
ARB
CCB
Beta_Blocker
ACE
0.005
0.006
0.007
0.008
0.009
Cumulative Incidence Rate at 1 Year After Baseline

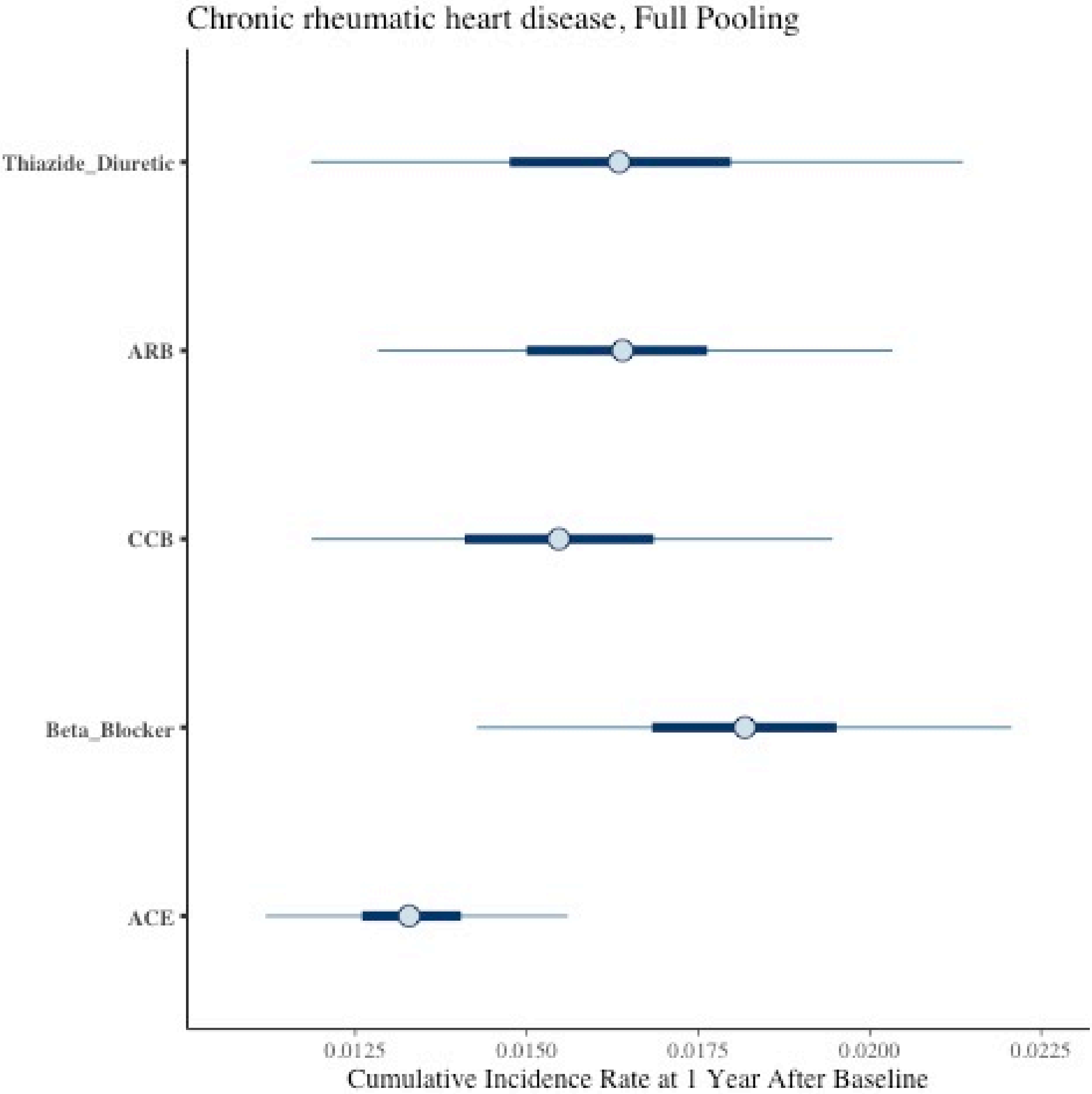

Chronic rheumatic heart disease, Full Pooling
Thiazide_Diuretic
ARB
CCB
Beta_Blocker
ACE
0.0125
0.0150
0.0175
0.0200
0.0225
Cumulative Incidence Rate at 1 Year After Baseline

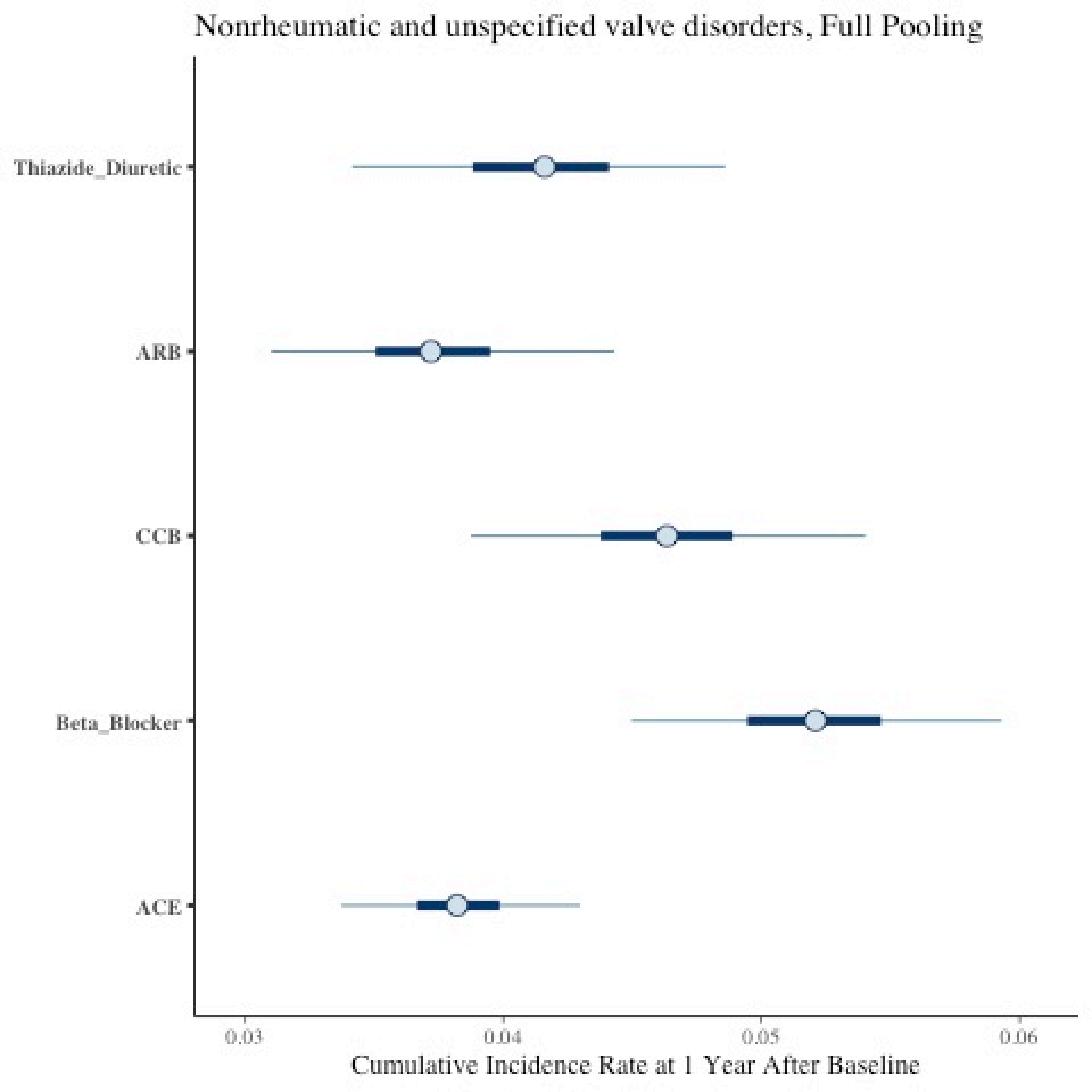

Nonrheumatic and unspecified valve disorders, Full Pooling
Thiazide_Diuretic
ARB
CCB
Beta_Blocker
ACE
0.03
0.04
0.05
0.06
Cumulative Incidence Rate at 1 Year After Baseline

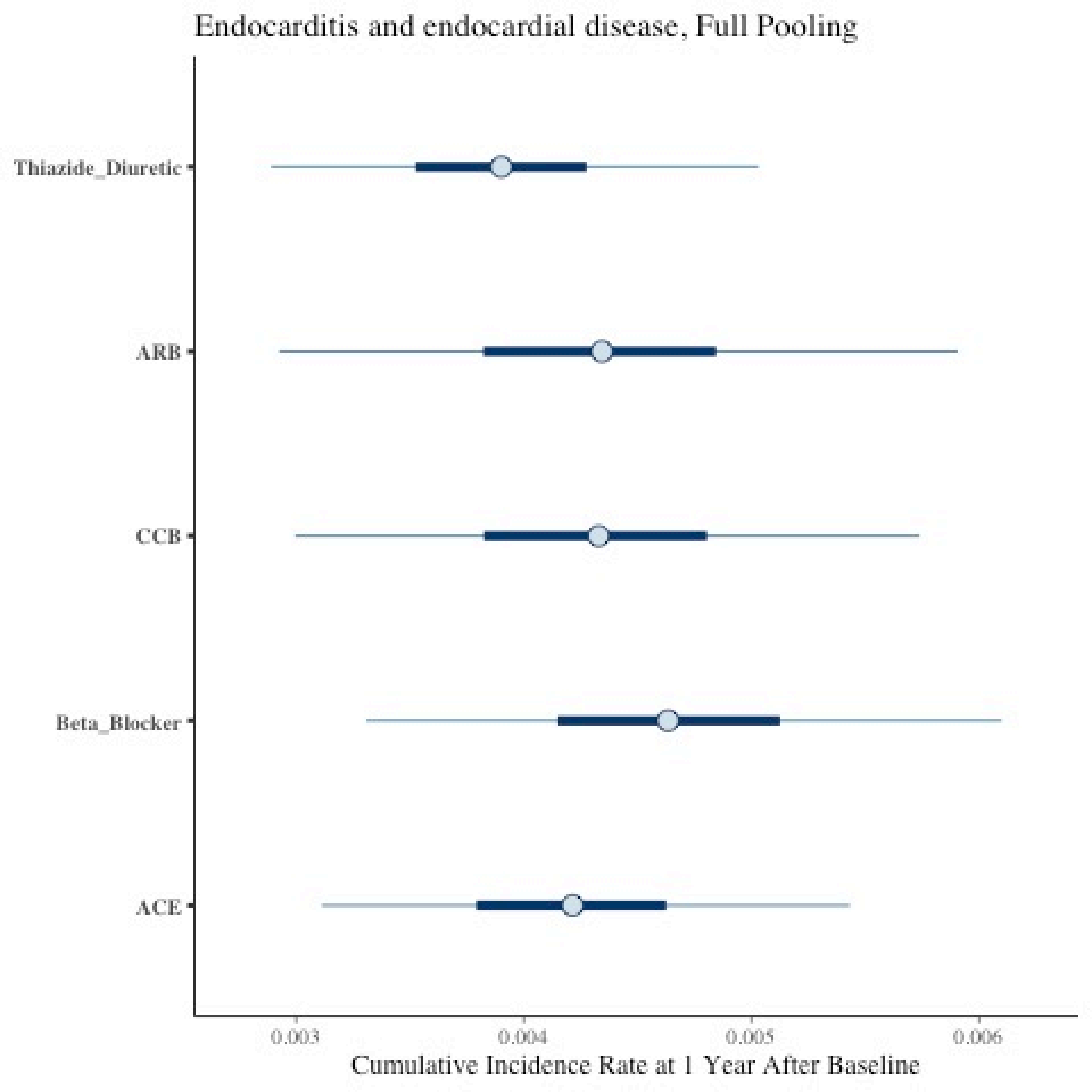

Endocarditis and endocardial disease, Full Pooling
Thiazide_Diuretic
ARB
CCB
Beta_Blocker
ACE
0.003
0.004
0.005
0.006
Cumulative Incidence Rate at 1 Year After Baseline

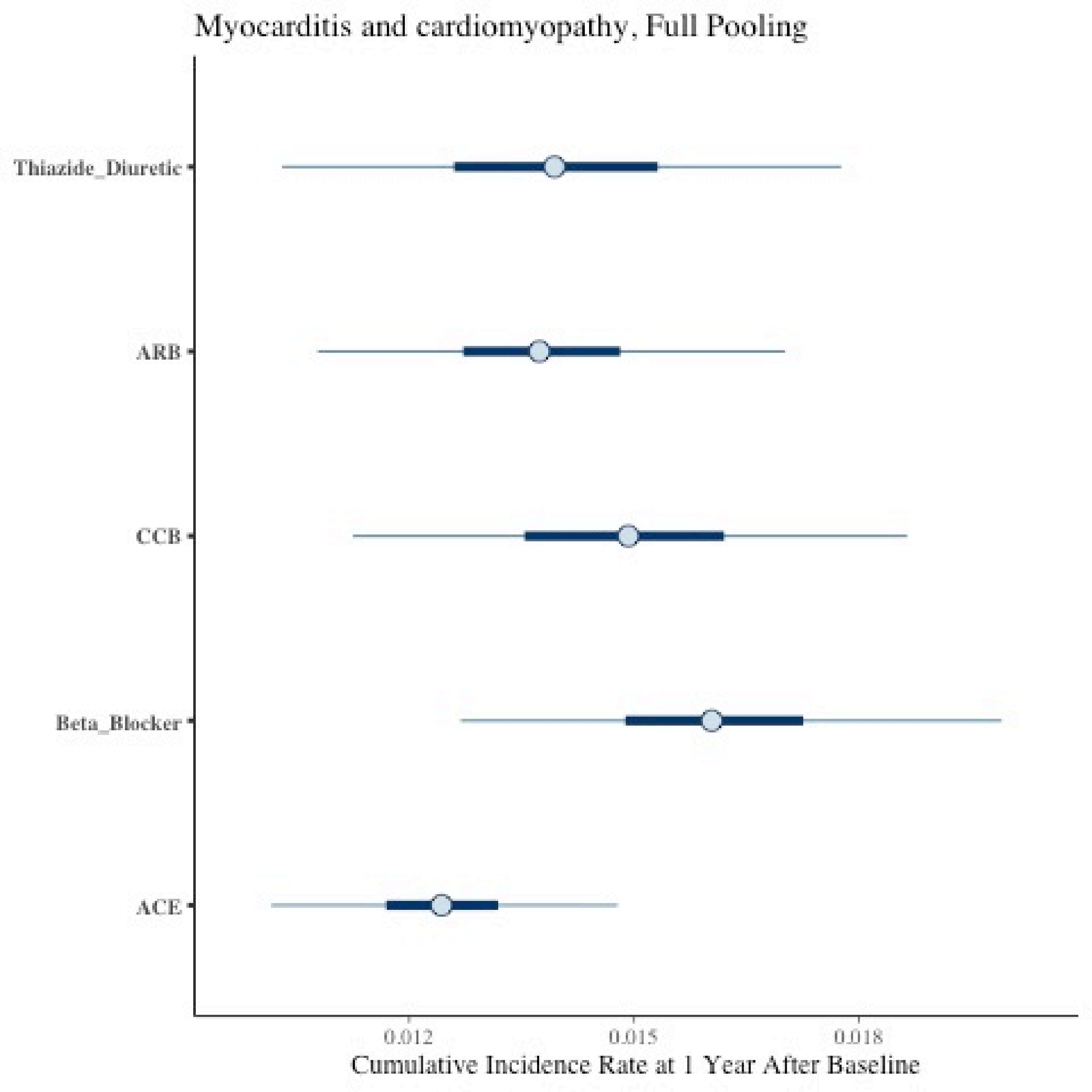
Myocarditis and cardiomyopathy, Full Pooling
Thiazide_Diuretic
ARB
CCB
Beta_Blocker
ACE
0.012
0.015
0.018
Cumulative Incidence Rate at 1 Year After Baseline

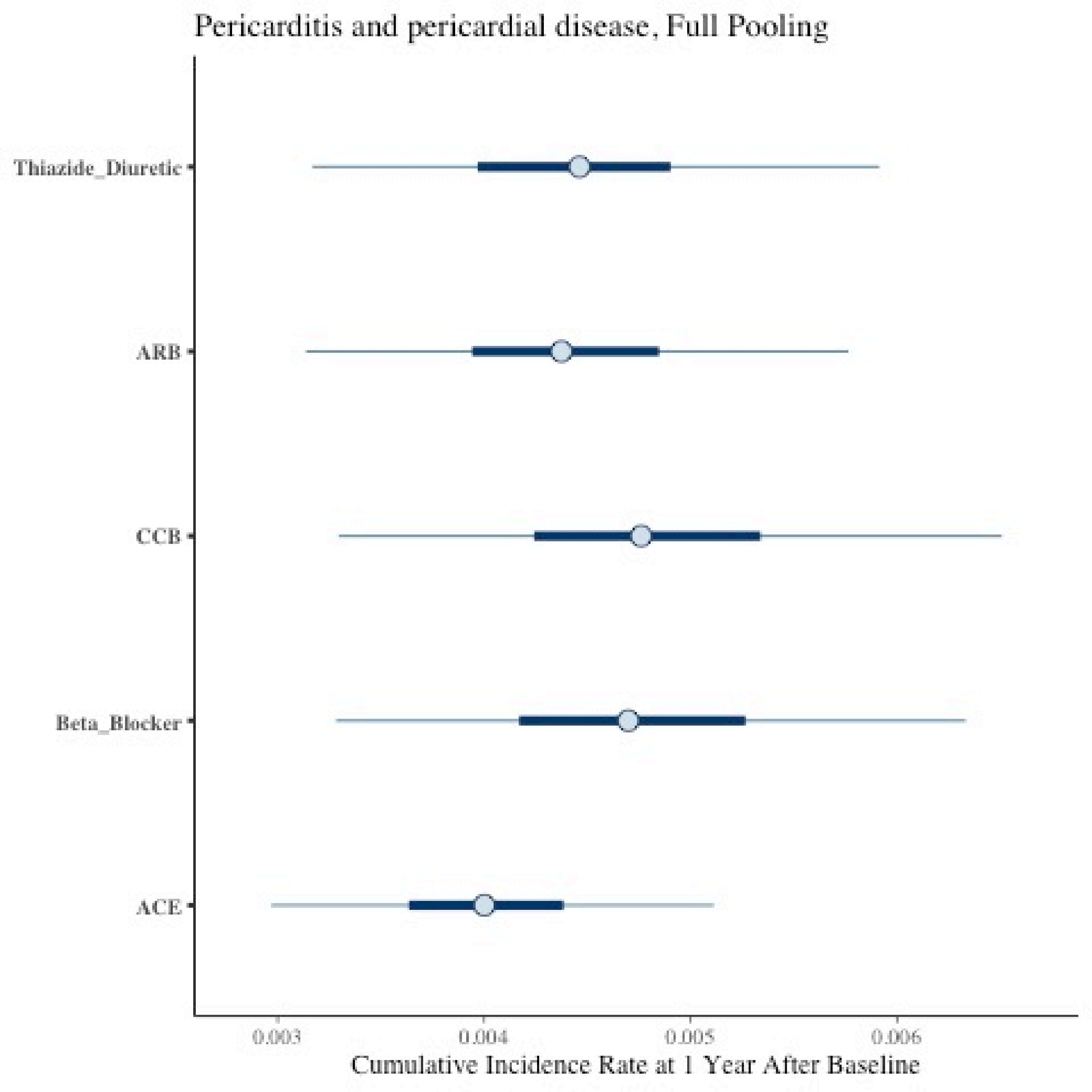
Pericarditis and pericardial disease, Full Pooling
Thiazide_Diuretic
ARB
CCB
Beta_Blocker
ACE
0.003
0.004
0.005
0.006
Cumulative Incidence Rate at 1 Year After Baseline

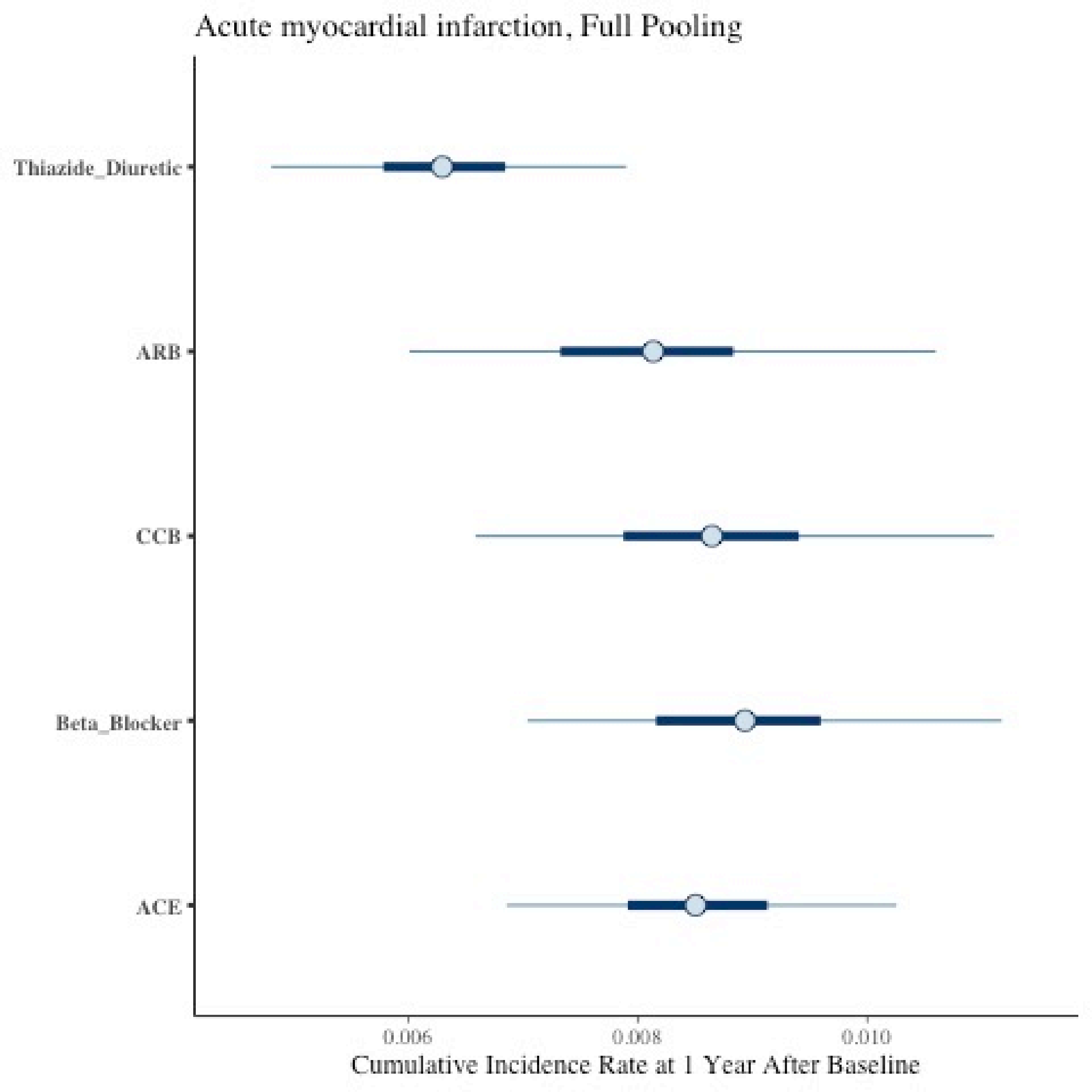

Acute myocardial infarction, Full Pooling
Thiazide_Diuretic
ARB
CCB
Beta_Blocker
ACE
0.006
0.008
0.010
Cumulative Incidence Rate at 1 Year After Baseline

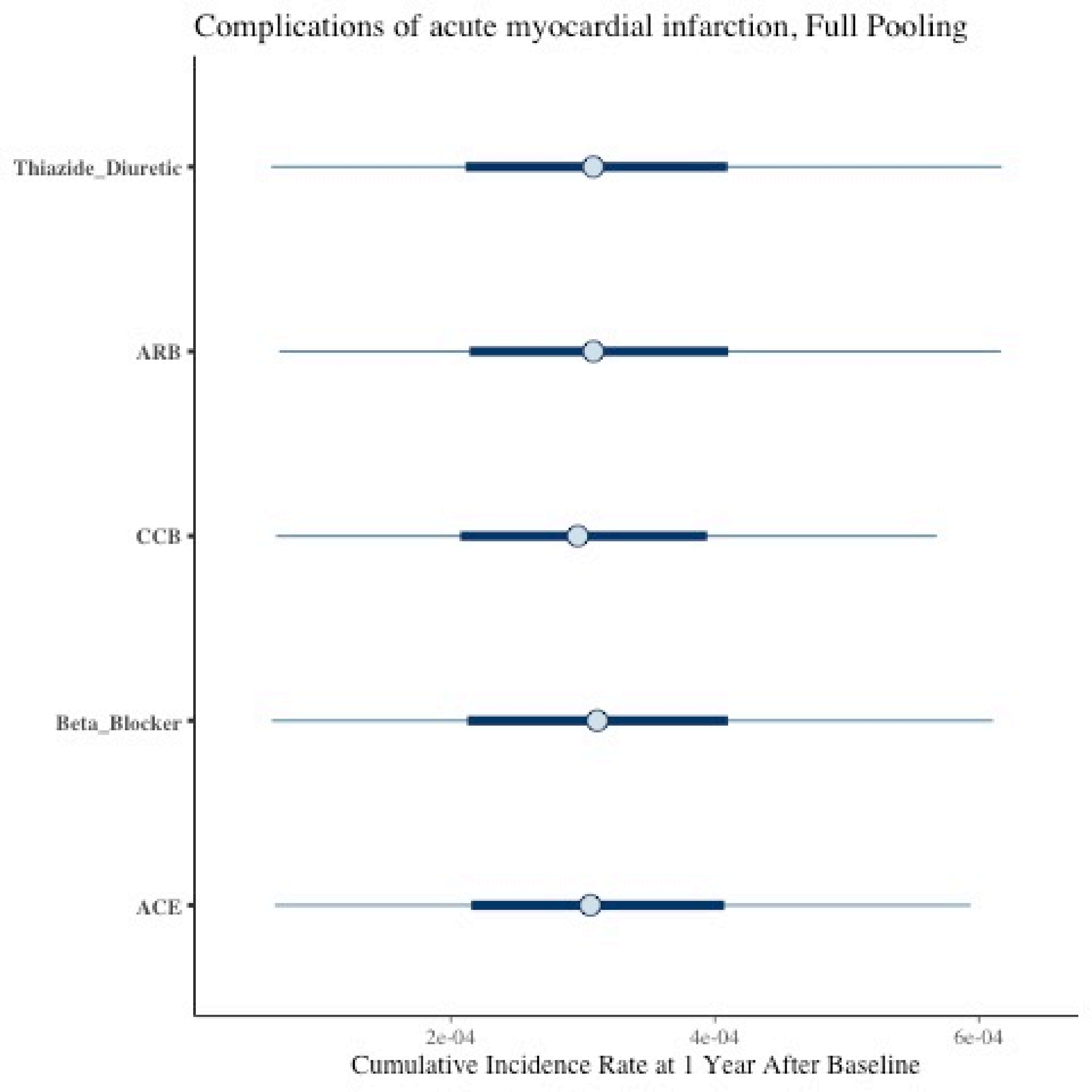

Complications of acute myocardial infarction, Full Pooling
Thiazide_Diuretic
ARB
CCB
Beta_Blocker
ACE
2e-04
4e-04
6e-04
Cumulative Incidence Rate at 1 Year After Baseline

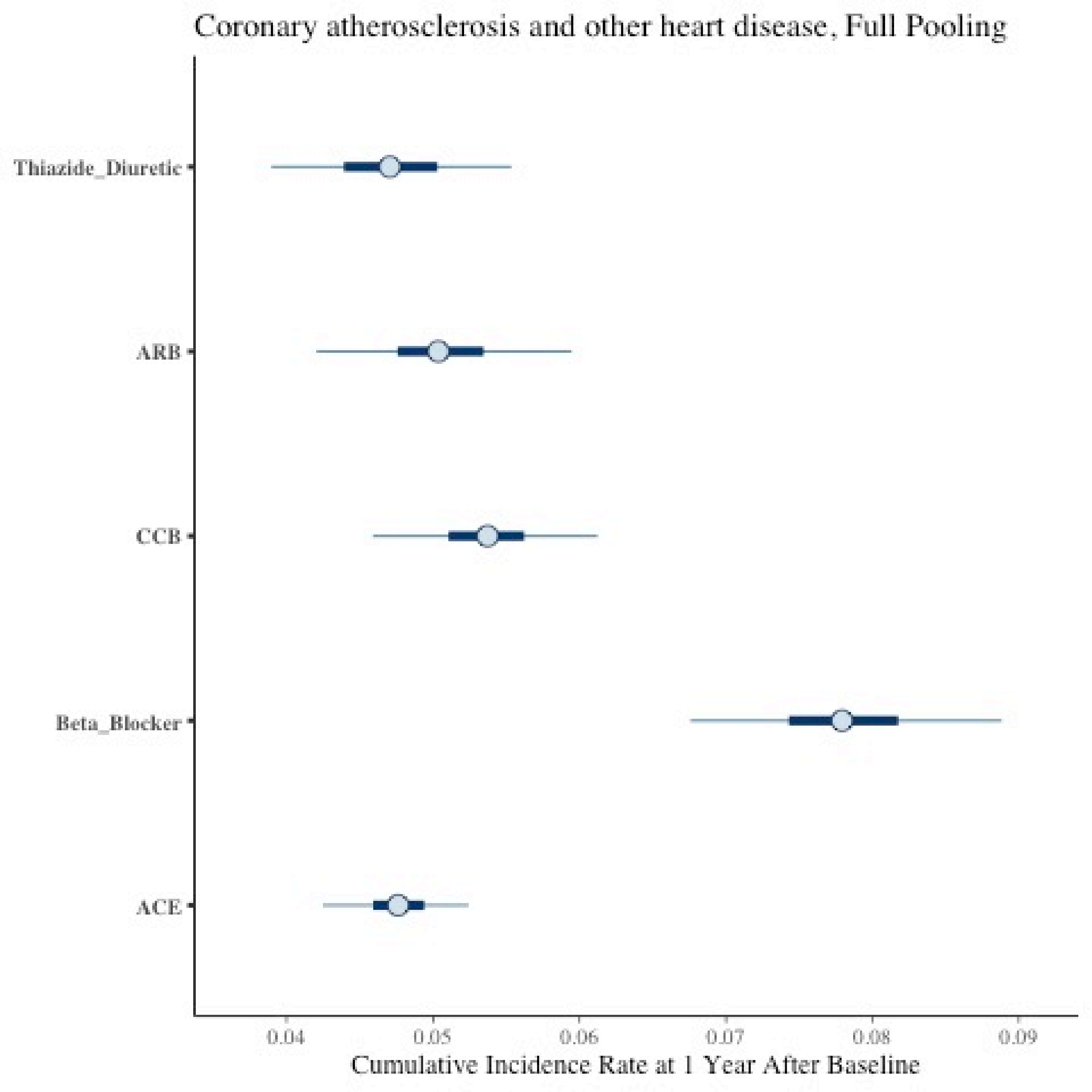
Coronary atherosclerosis and other heart disease, Full Pooling
Thiazide_Diuretic
ARB
CCB
Beta_Blocker
ACE
0.04
0.05
0.06
0.07
0.08
0.09
Cumulative Incidence Rate at 1 Year After Baseline

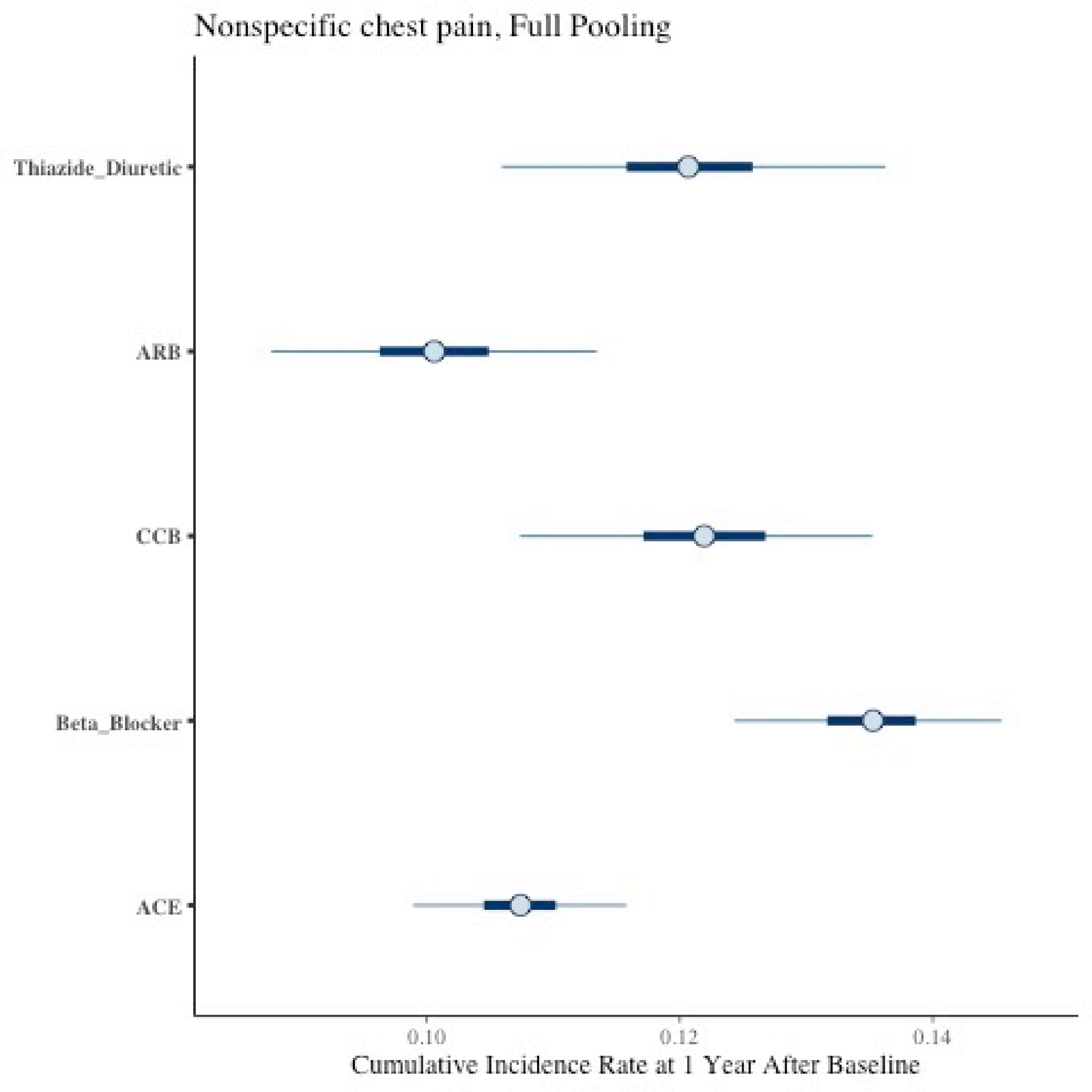

Nonspecific chest pain, Full Pooling
Thiazide_Diuretic
ARB
CCB
Beta_Blocker
ACE
0.10
0.12
0.14
Cumulative Incidence Rate at 1 Year After Baseline

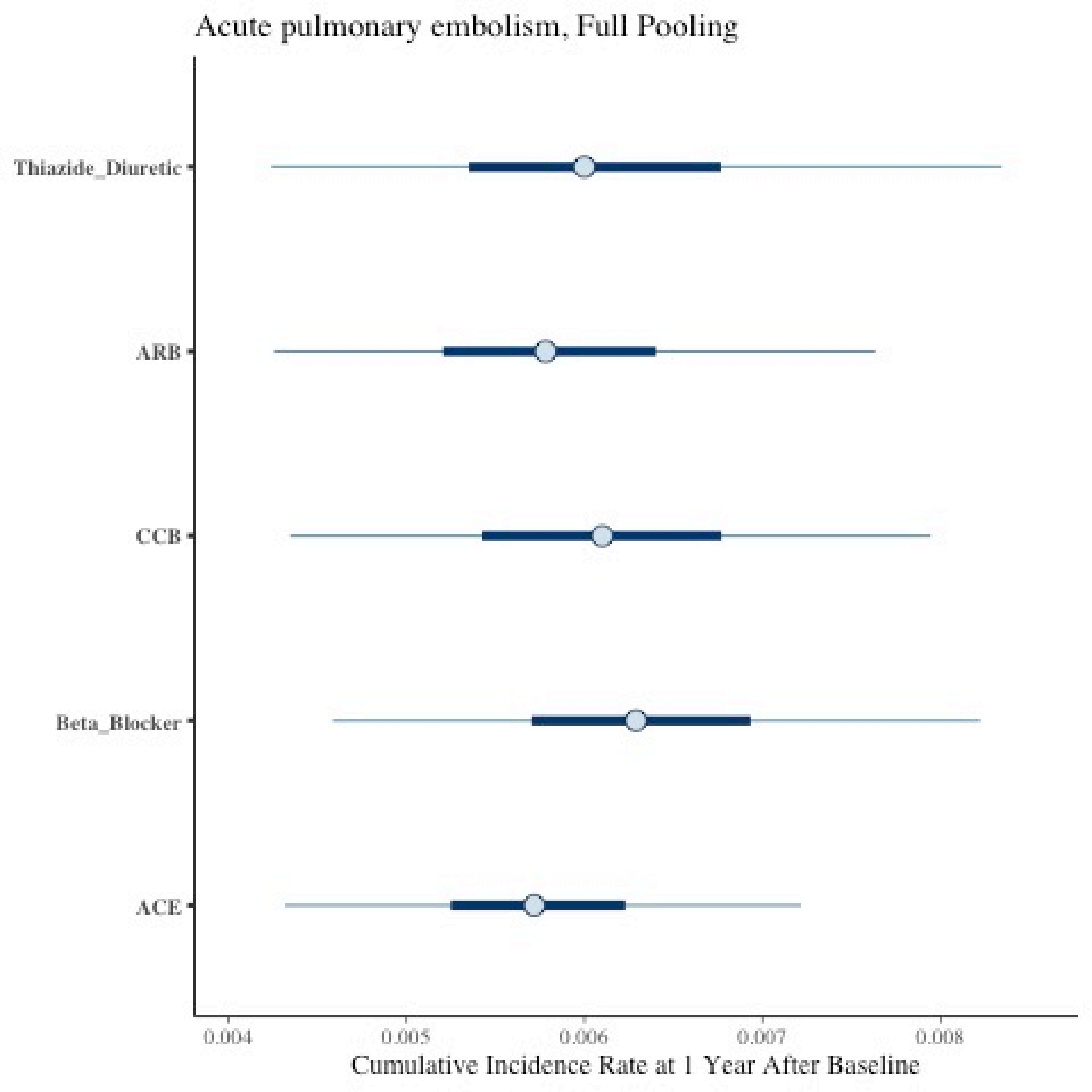

Acute pulmonary embolism, Full Pooling
Thiazide_Diuretic
ARB
CCB
Beta_Blocker
ACE
0.004
0.005
0.006
0.007
0.008
Cumulative Incidence Rate at 1 Year After Baseline

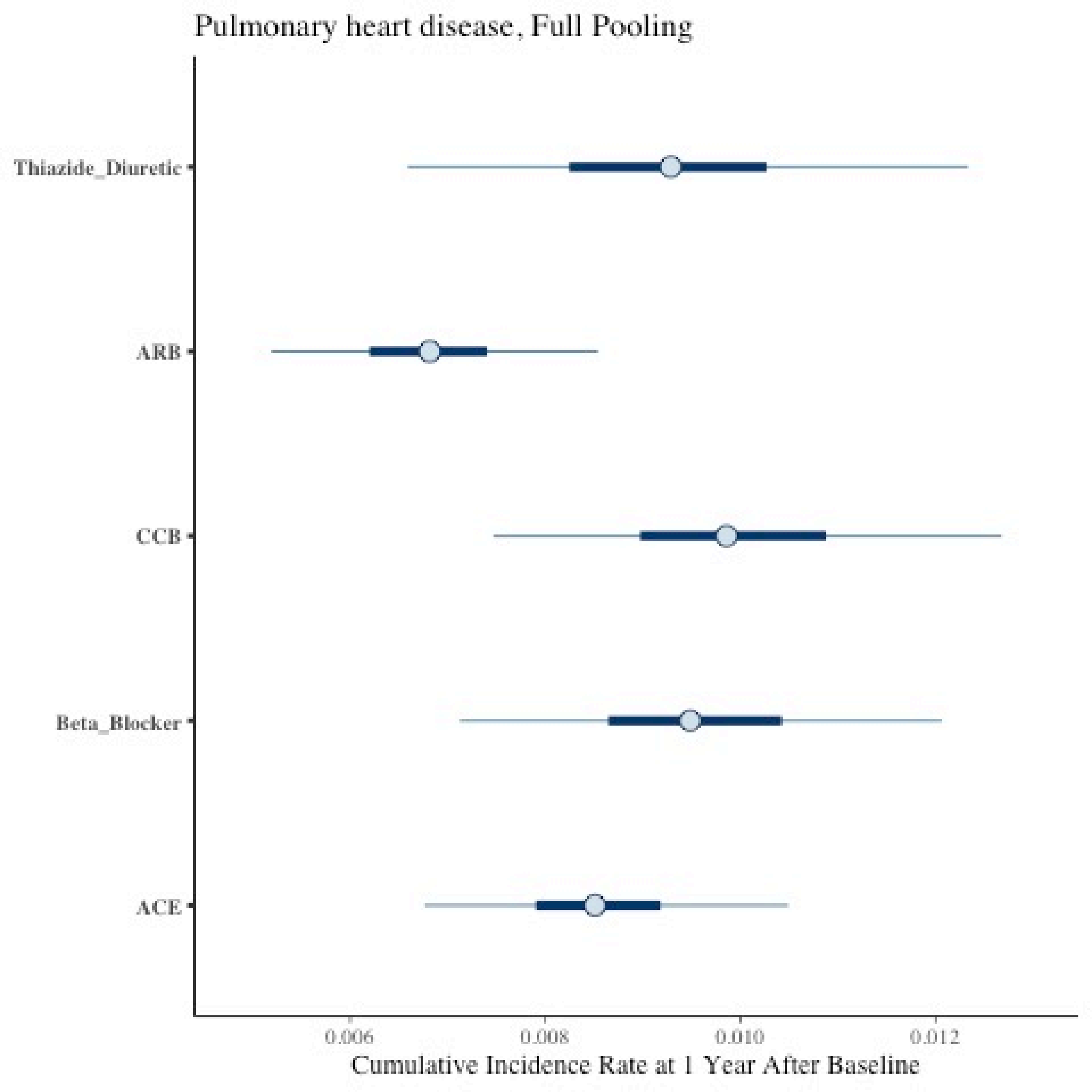

Pulmonary heart disease, Full Pooling
Thiazide_Diuretic
ARB
CCB
Beta_Blocker
ACE
0.006
0.008
0.010
0.012
Cumulative Incidence Rate at 1 Year After Baseline

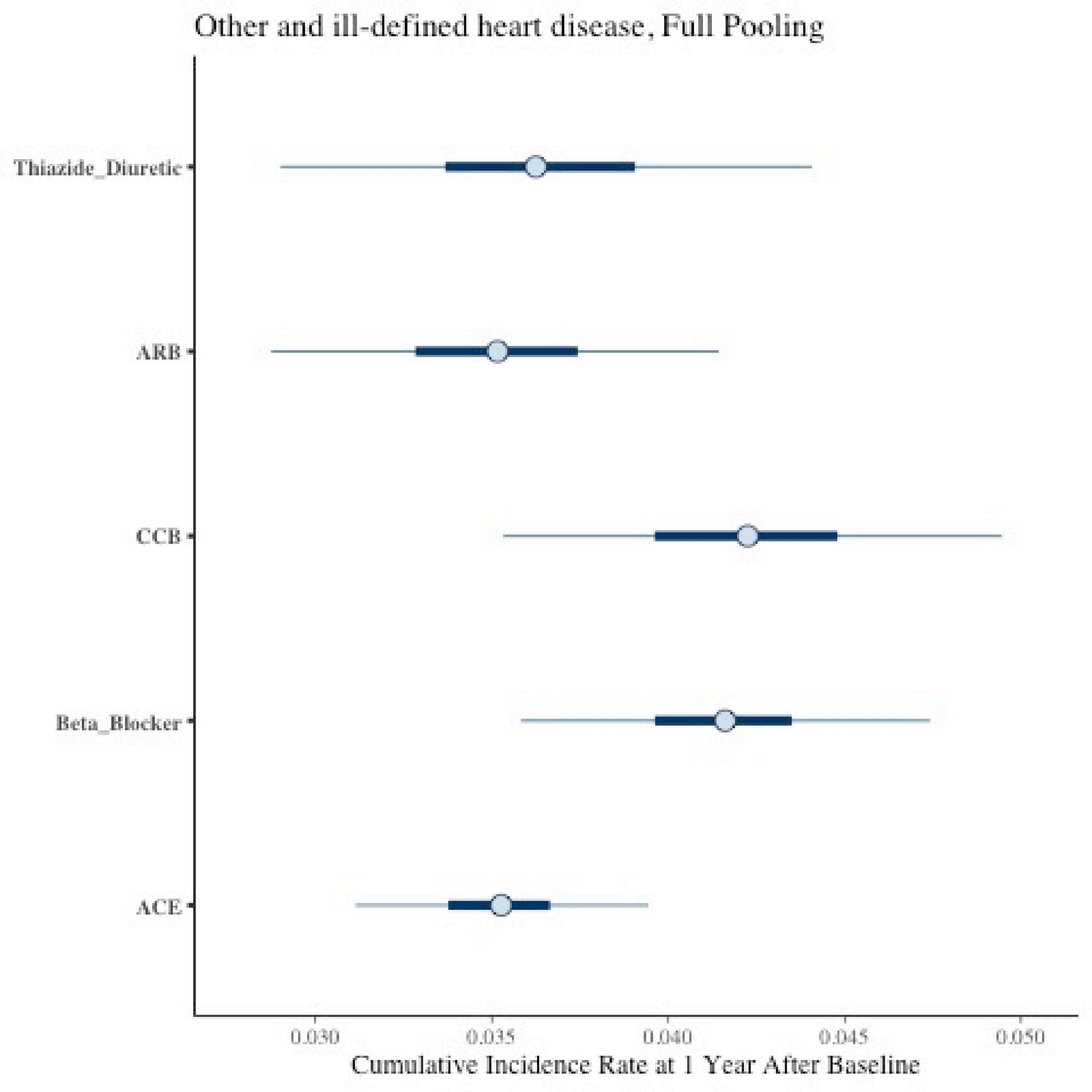

Other and ill-defined heart disease, Full Pooling
Thiazide_Diuretic
ARB
CCB
Beta_Blocker
ACE
0.030
0.035
0.040
0.045
0.050
Cumulative Incidence Rate at 1 Year After Baseline

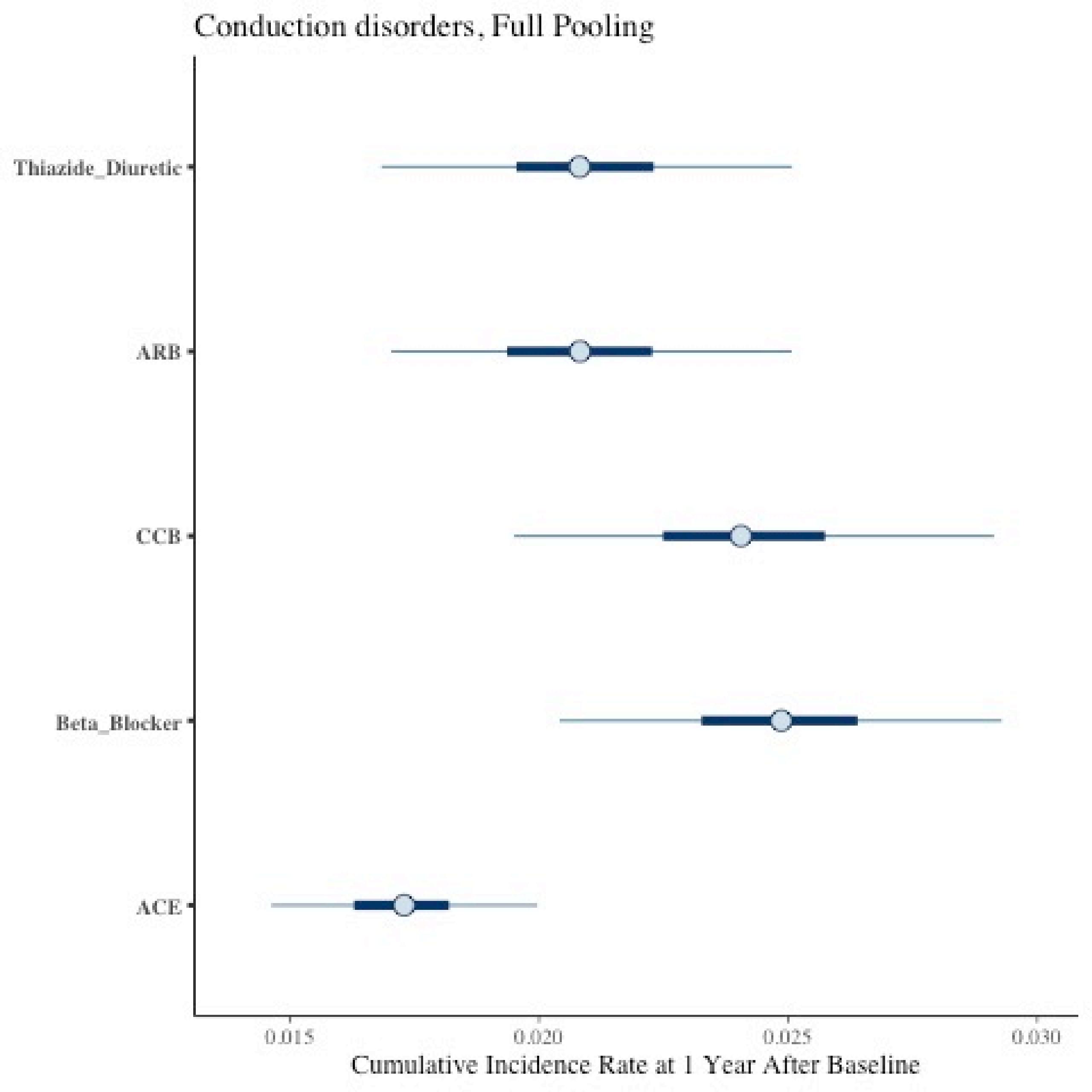

Conduction disorders, Full Pooling
Thiazide_Diuretic
ARB
CCB
Beta_Blocker
ACE
0.015
0.020
0.025
0.030
Cumulative Incidence Rate at 1 Year After Baseline

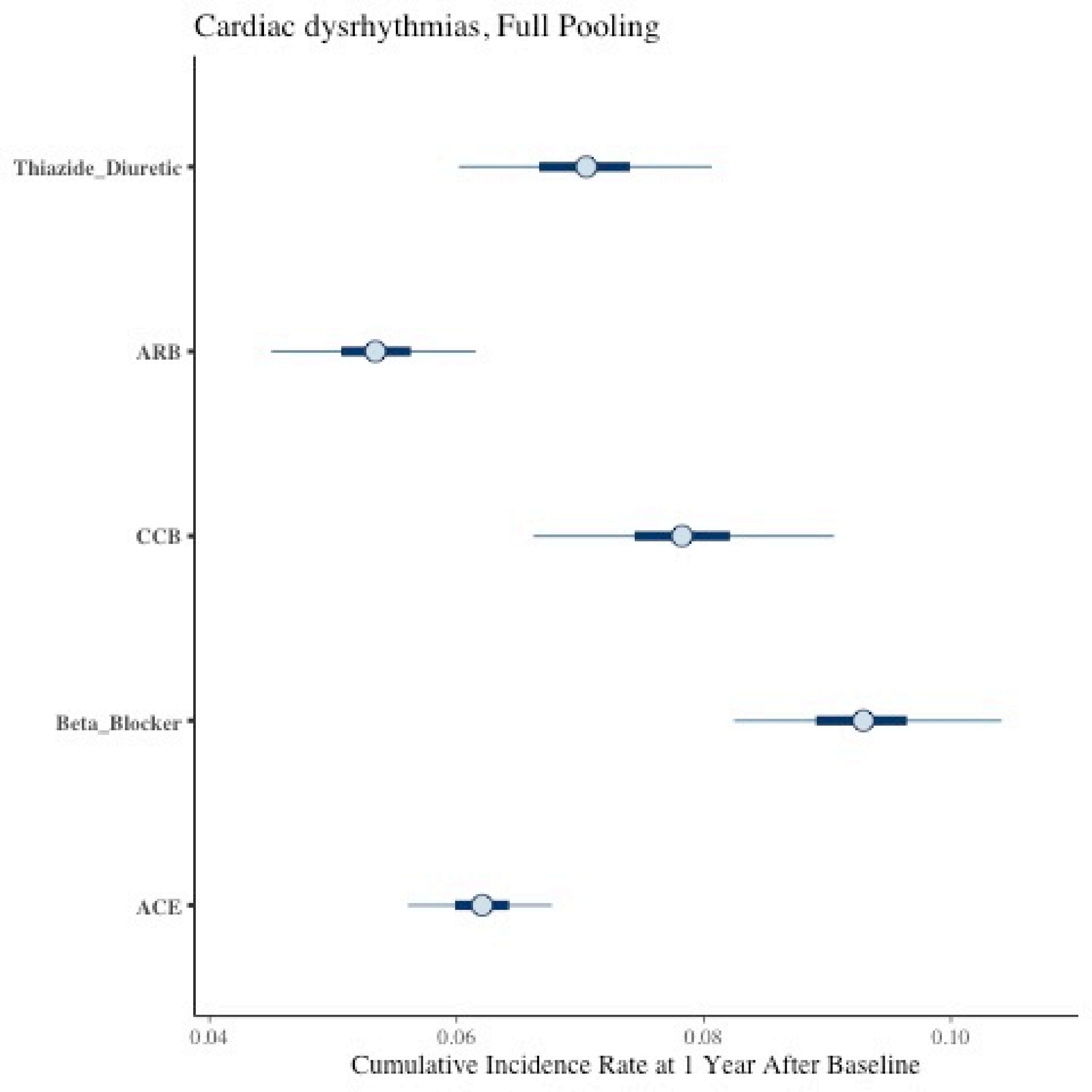

Cardiac dysrhythmias, Full Pooling
Thiazide_Diuretic
ARB
CCB
Beta_Blocker
ACE
0.04
0.06
0.08
0.10
Cumulative Incidence Rate at 1 Year After Baseline

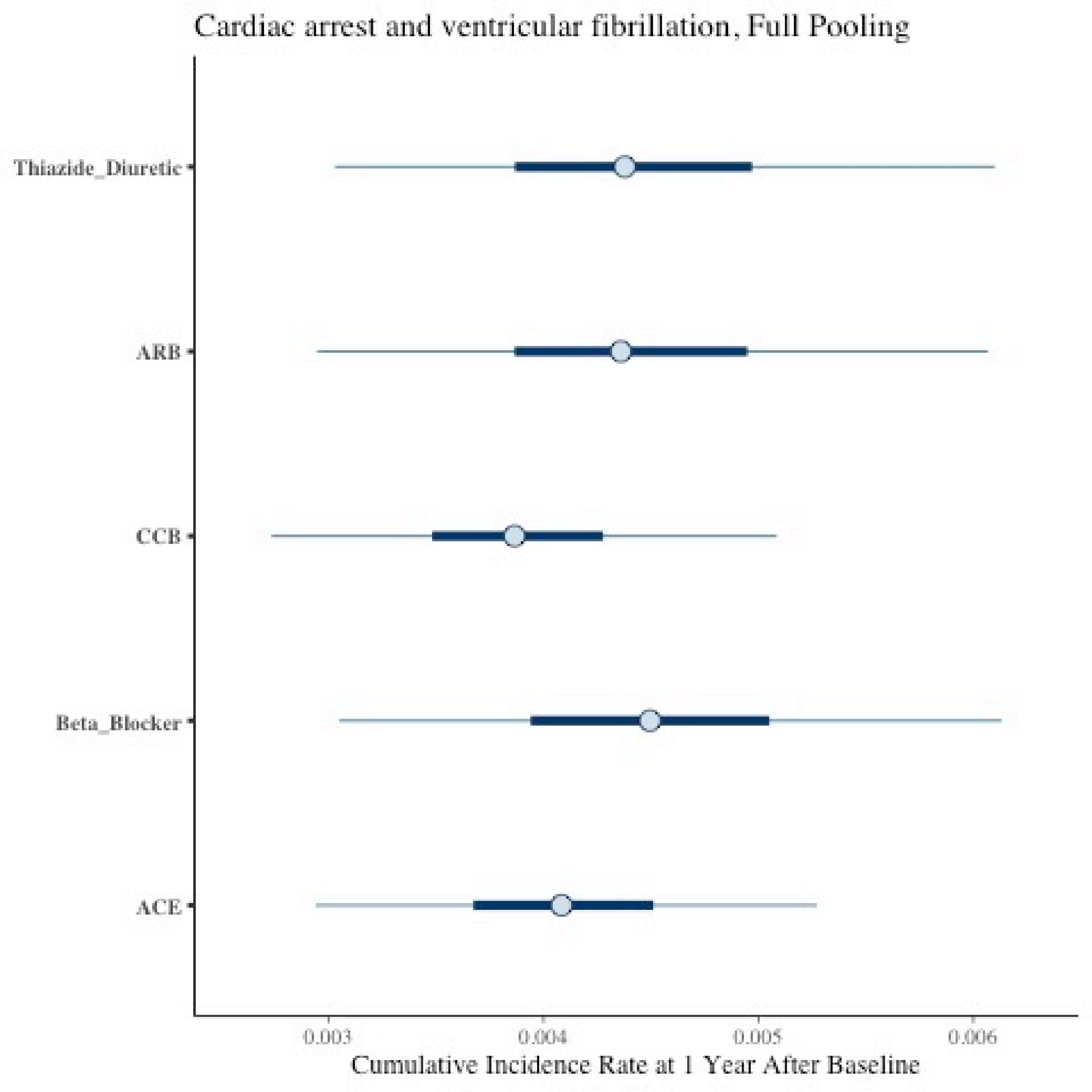

Cardiac arrest and ventricular fibrillation, Full Pooling
Thiazide_Diuretic
ARB
CCB
Beta_Blocker
ACE
0.003
0.004
0.005
0.006
Cumulative Incidence Rate at 1 Year After Baseline

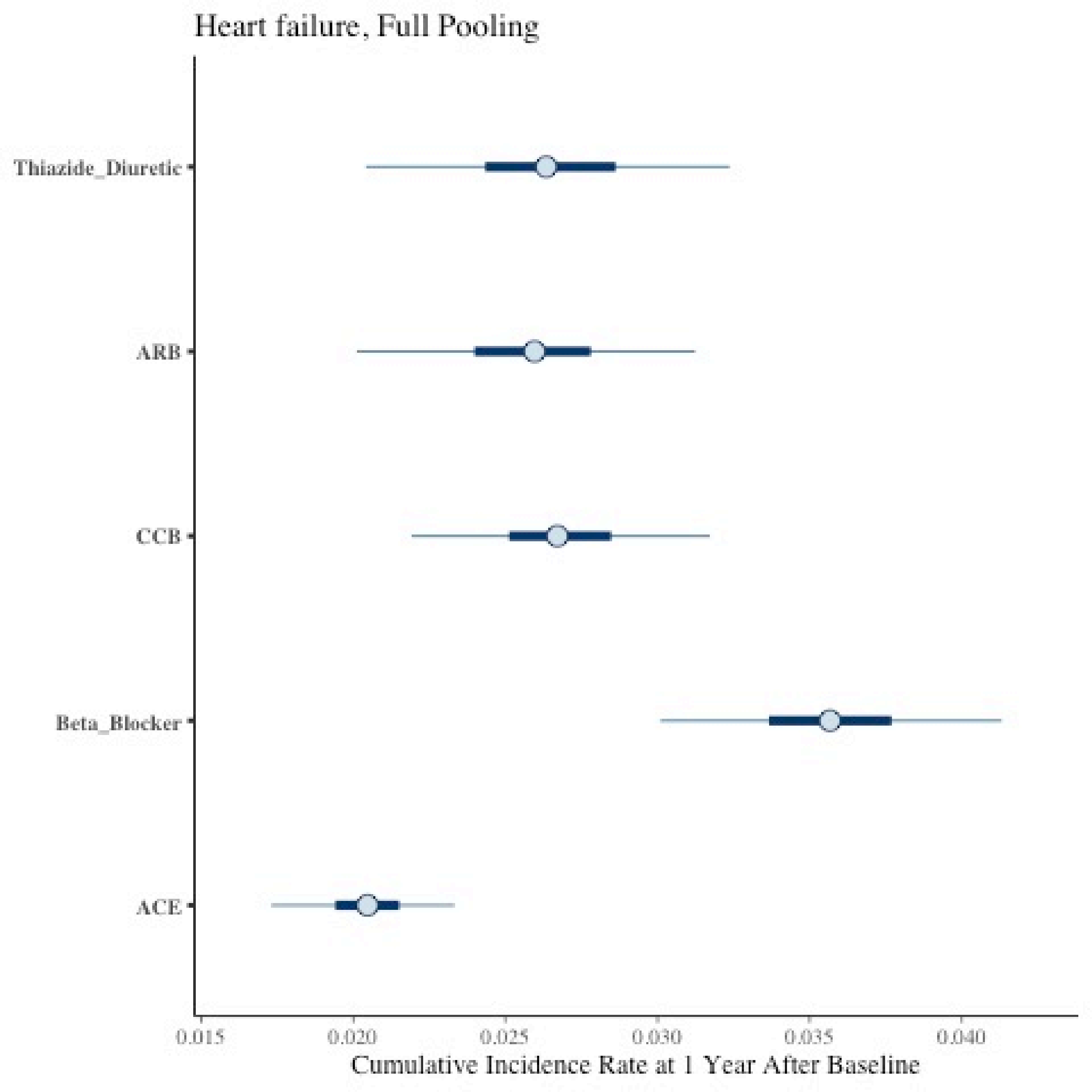

Heart failure, Full Pooling
Thiazide_Diuretic
ARB
CCB
Beta_Blocker
ACE
0.015
0.020
0.025
0.030
0.035
0.040
Cumulative Incidence Rate at 1 Year After Baseline

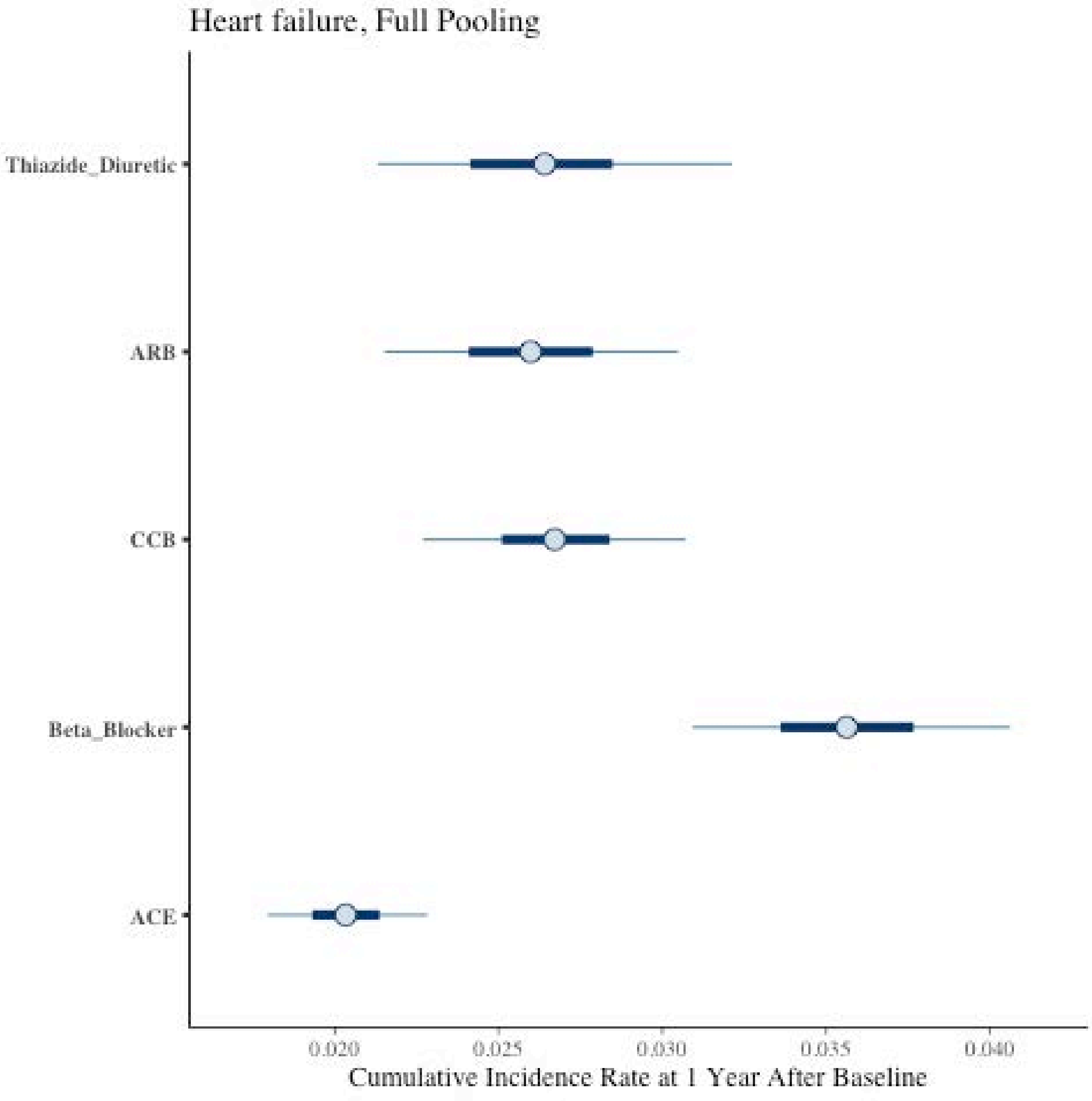
Heart failure, Full Pooling
Thiazide_Diuretic
ARB
CCB
Beta_Blocker
ACE
0.020
0.025
0.030
0.035
0.040
Cumulative Incidence Rate at 1 Year After Baseline

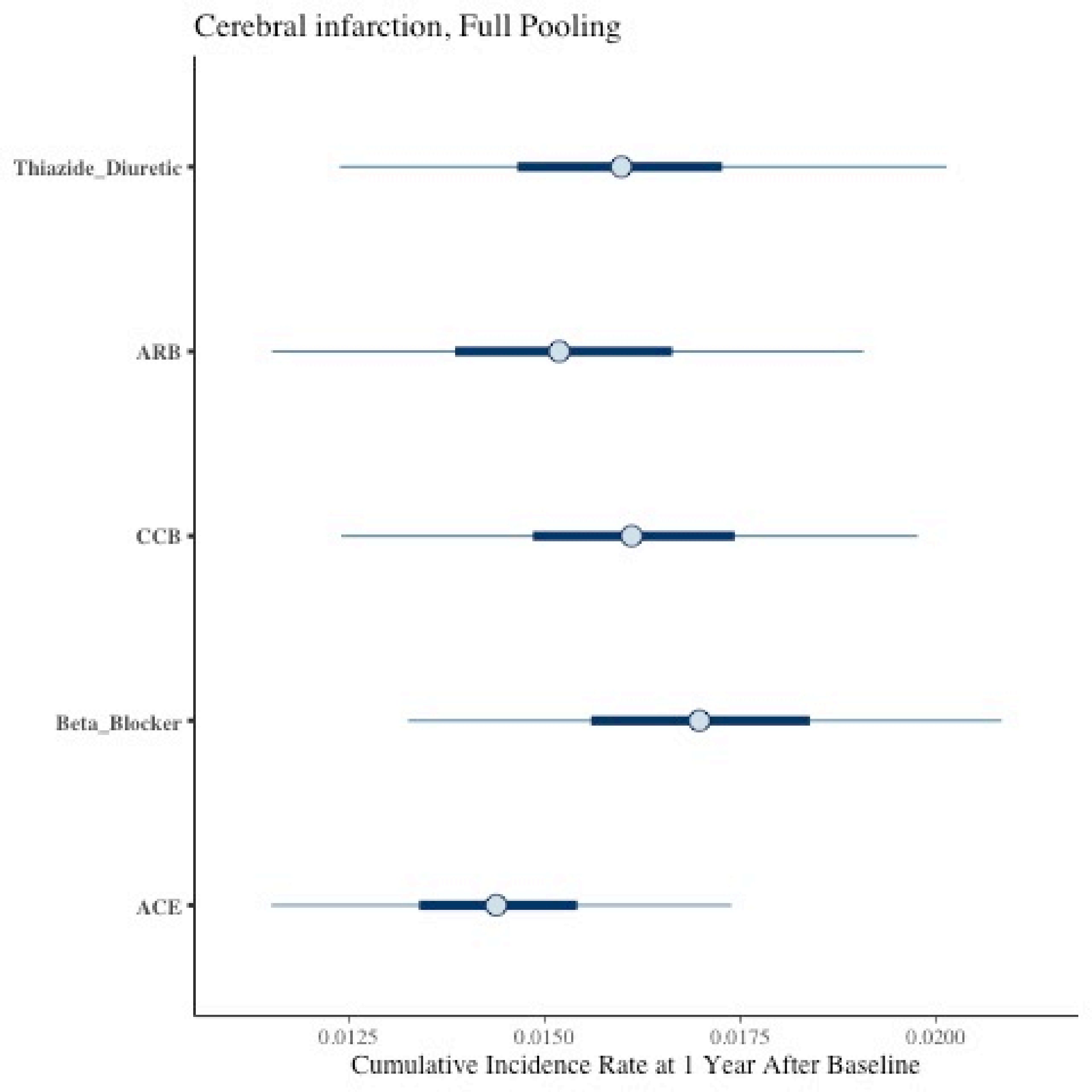

Cerebral infarction, Full Pooling
Thiazide_Diuretic
ARB
CCB
Beta_Blocker
ACE
0.0125
0.0150
0.0175
0.0200
Cumulative Incidence Rate at 1 Year After Baseline

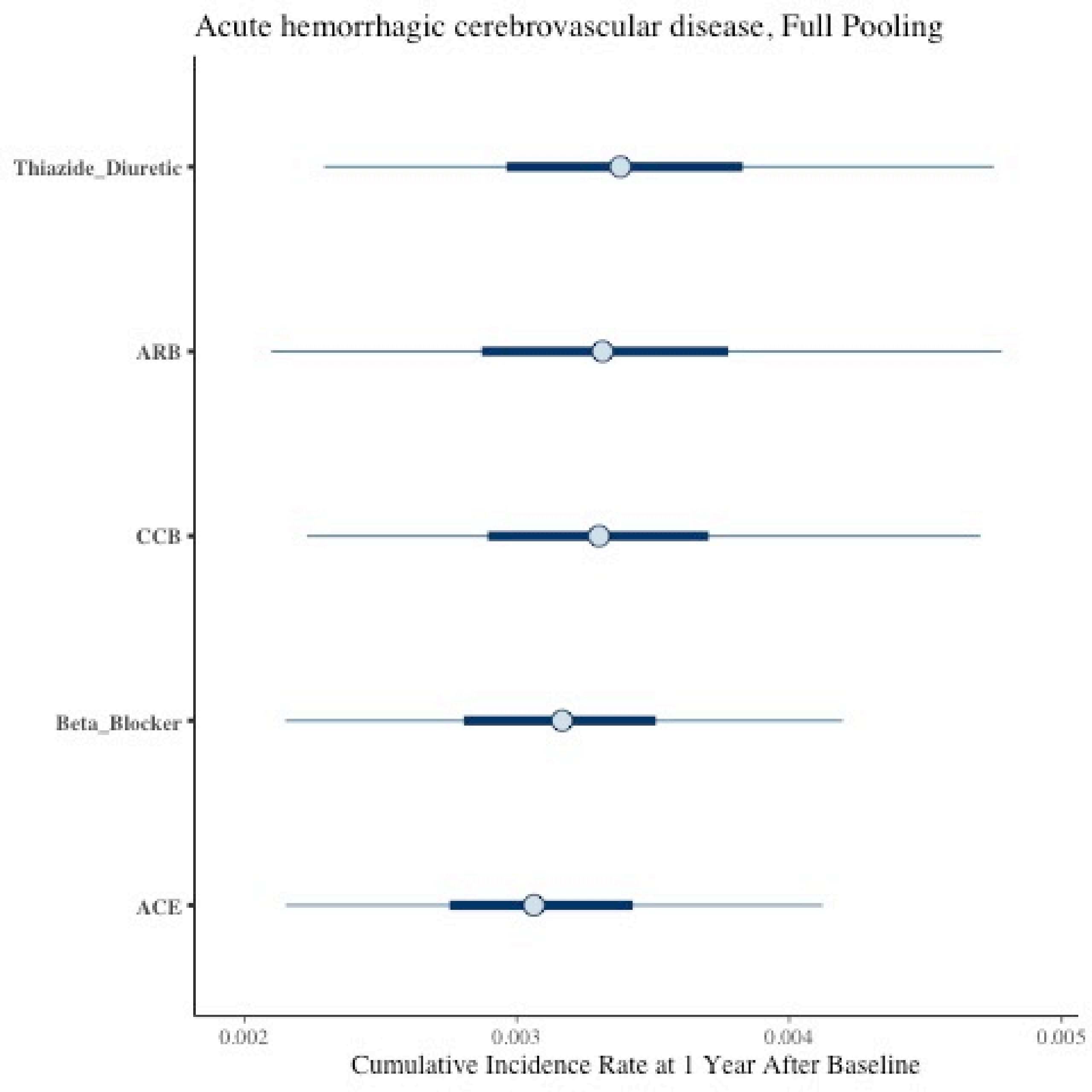
Acute hemorrhagic cerebrovascular disease, Full Pooling
Thiazide_Diuretic
ARB
CCB
Beta_Blocker
ACE
0.002
0.003
0.004
0.005
Cumulative Incidence Rate at 1 Year After Baseline

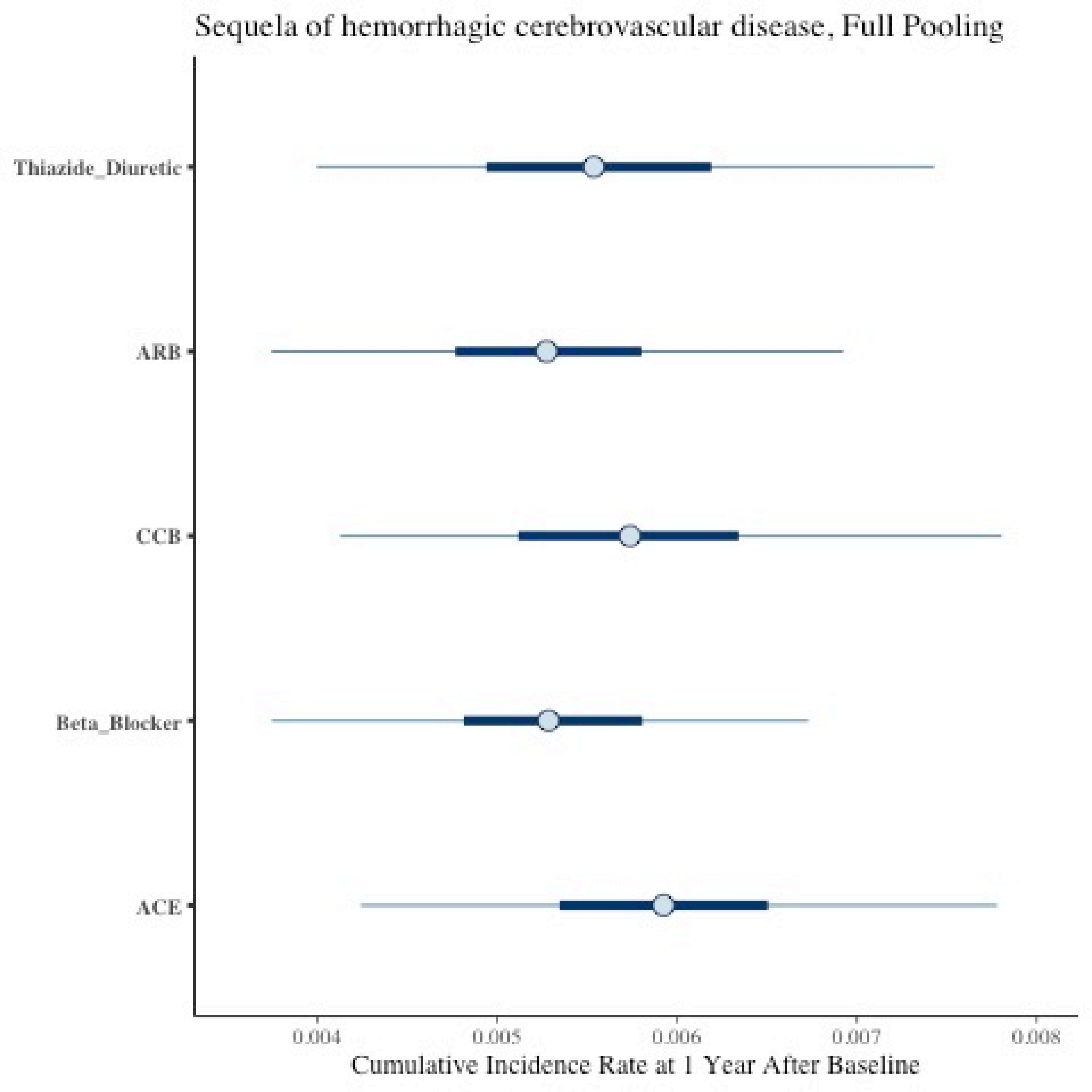
Sequela of hemorrhagic cerebrovascular disease, Full Pooling
Thiazide_Diuretic
ARB
CCB
Beta_Blocker
ACE
0.004
0.005
0.006
0.007
0.008
Cumulative Incidence Rate at 1 Year After Baseline

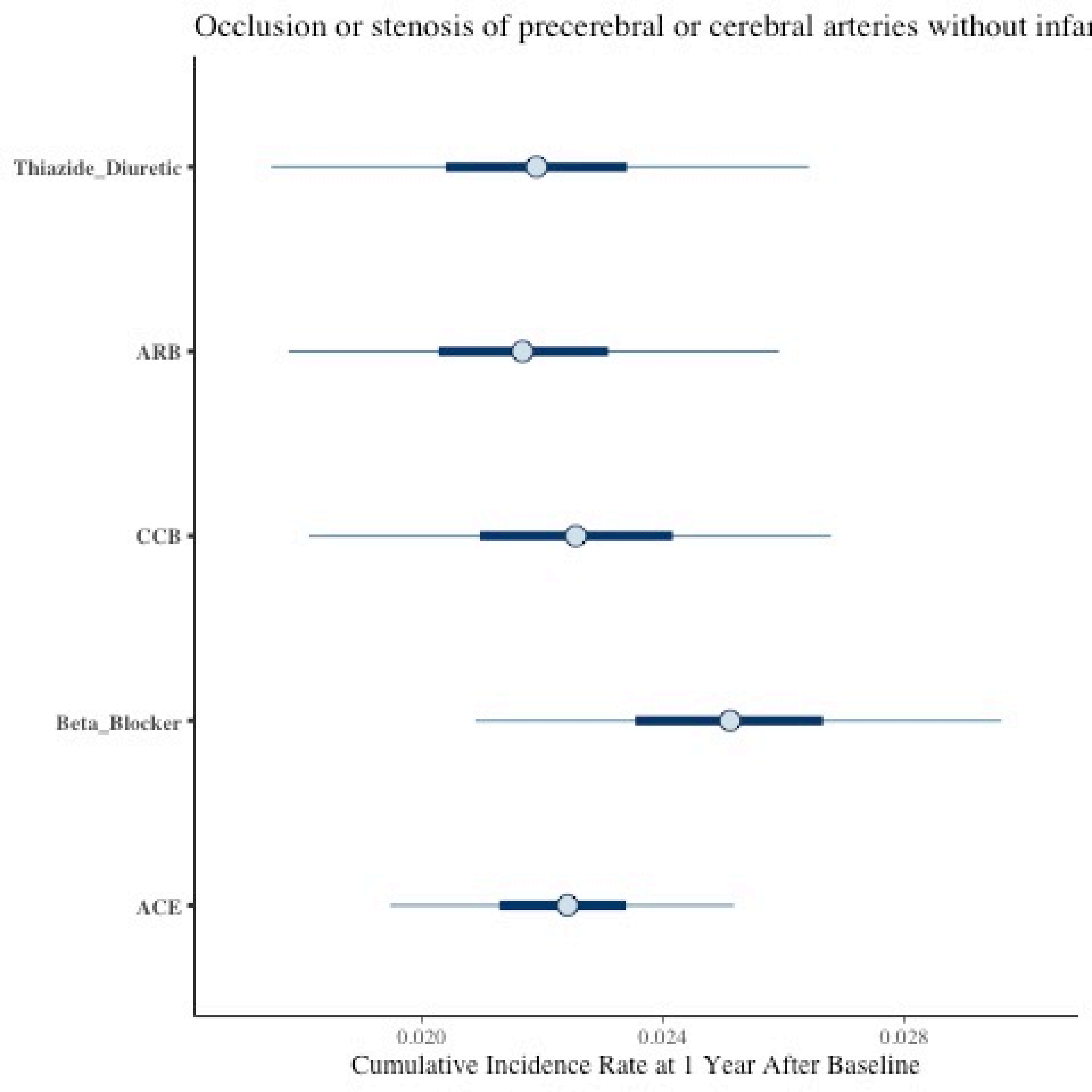

Occlusion or stenosis of precerebral or cerebral arteries without infar
Thiazide_Diuretic
ARB
CCB
Beta_Blocker
ACE
0.020
0.024
0.028
Cumulative Incidence Rate at 1 Year After Baseline

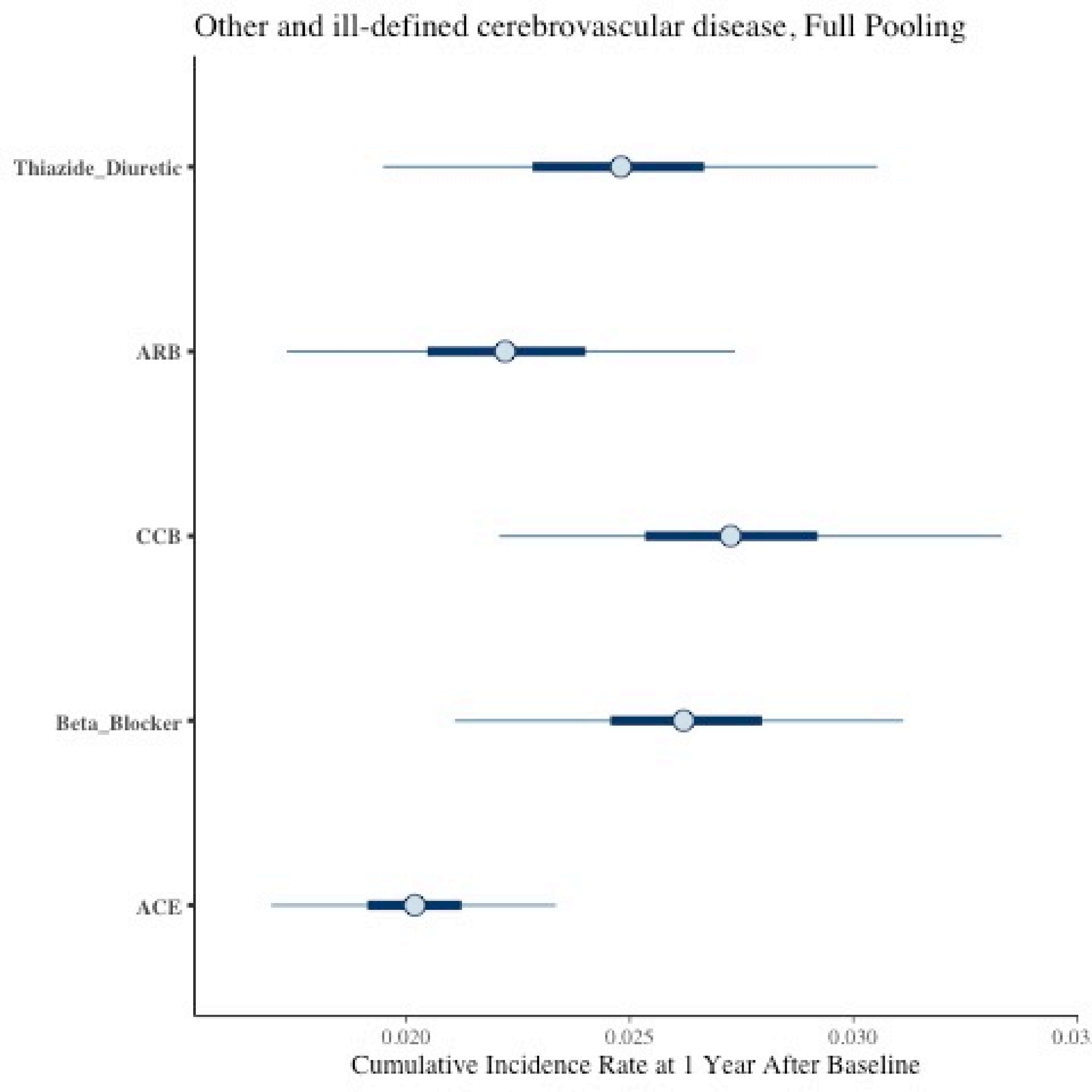

Other and ill-defined cerebrovascular disease, Full Pooling
Thiazide_Diuretic
ARB
CCB
Beta_Blocker
ACE
0.020
0.025
0.030
Cumulative Incidence Rate at 1 Year After Baseline

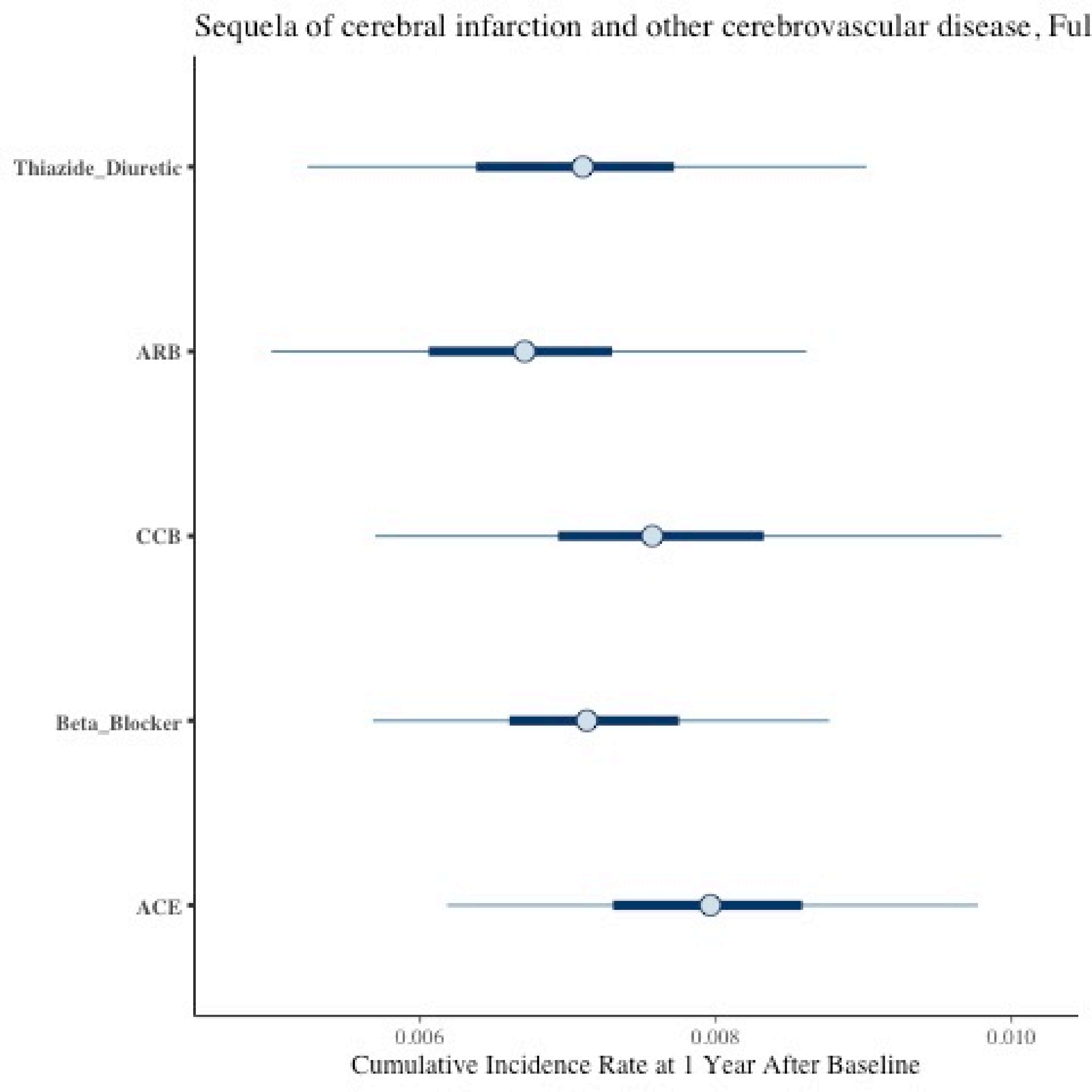
Sequela of cerebral infarction and other cerebrovascular disease, Ful
Thiazide_Diuretic
ARB
CCB
Beta_Blocker
ACE
0.006
0.008
0.010
Cumulative Incidence Rate at 1 Year After Baseline

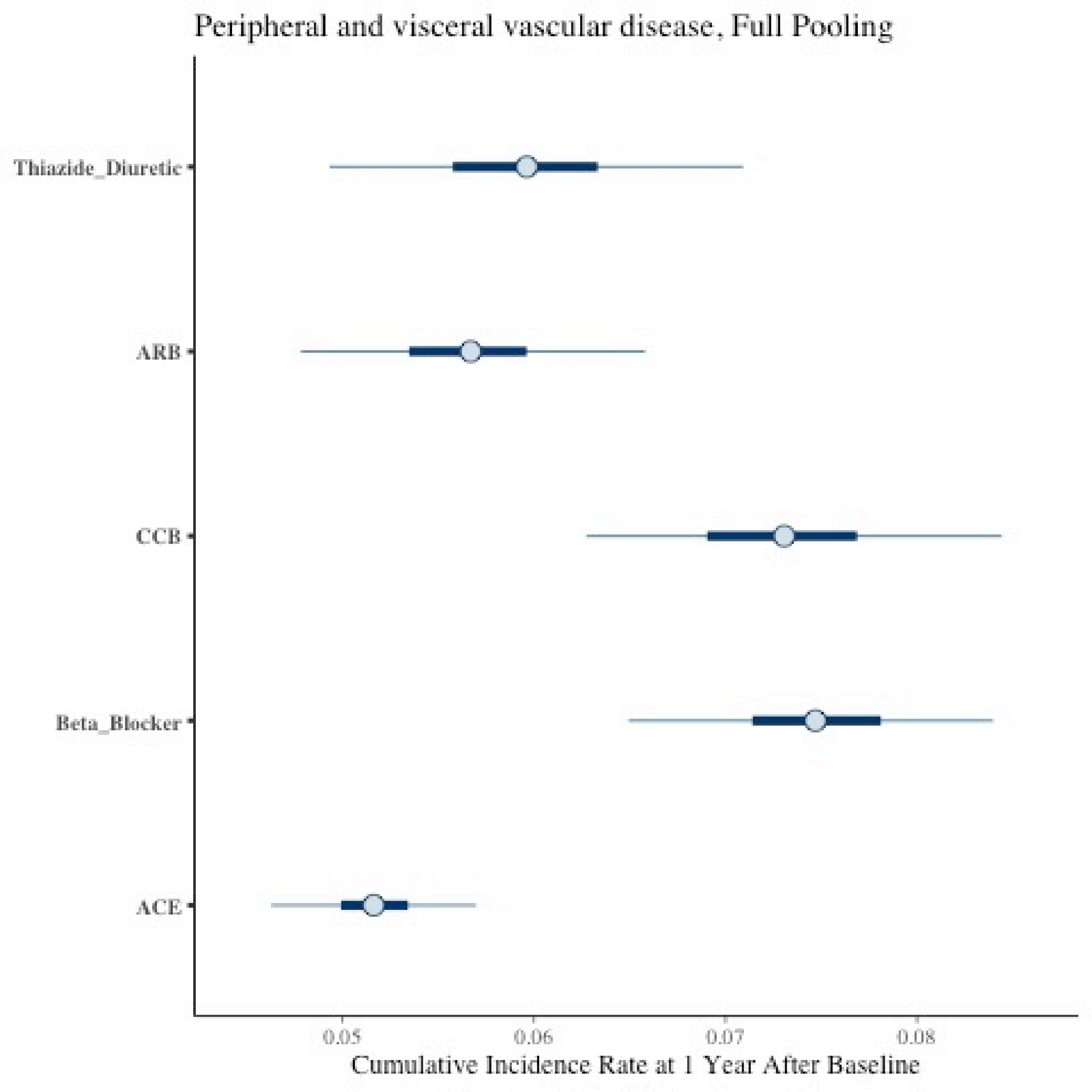

Peripheral and visceral vascular disease, Full Pooling
Thiazide_Diuretic
ARB
CCB
Beta_Blocker
ACE
0.05
0.06
0.07
0.08
Cumulative Incidence Rate at 1 Year After Baseline

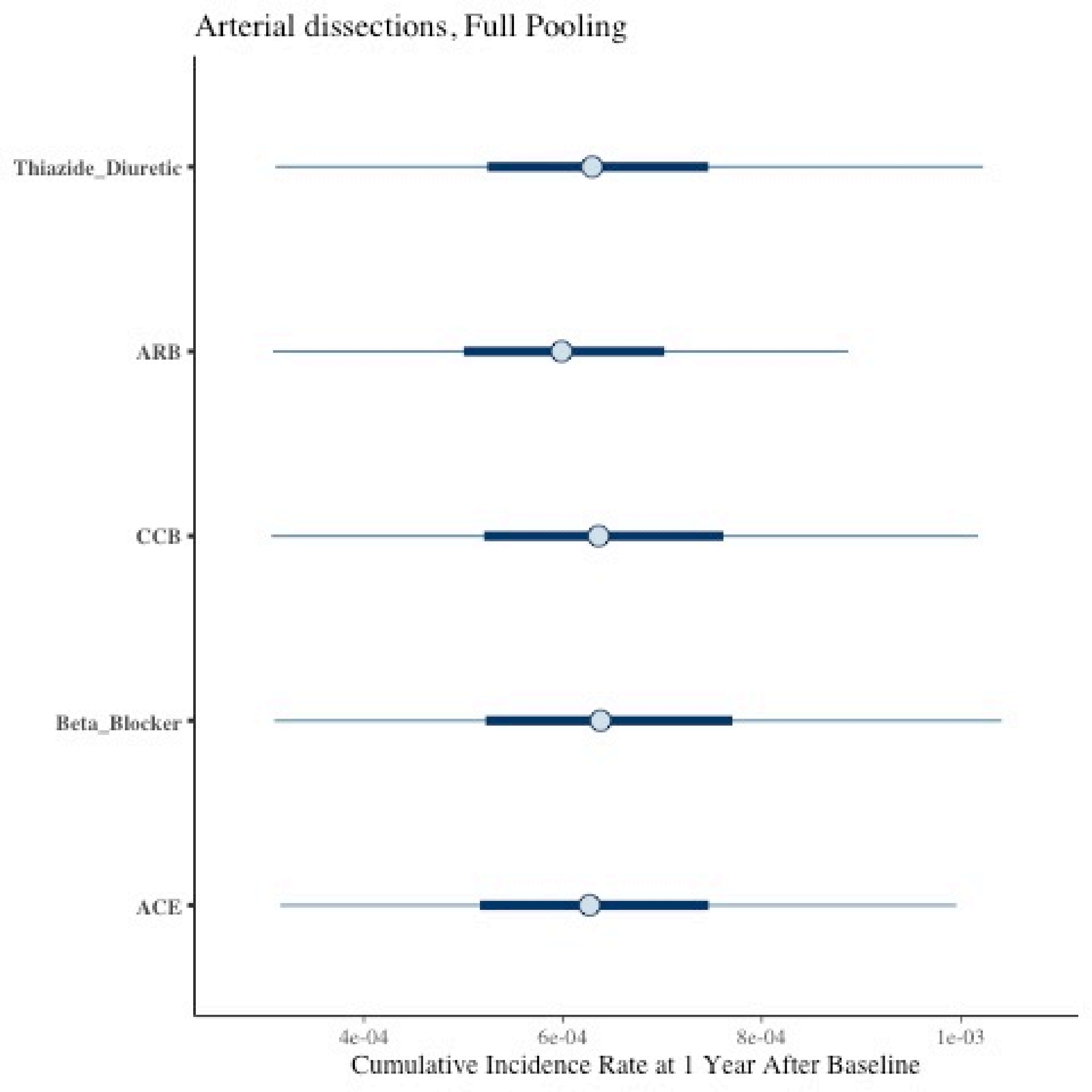

Arterial dissections, Full Pooling
Thiazide_Diuretic
ARB
CCB
Beta_Blocker
ACE
4e-04
6e-04
8e-04
1e-03
Cumulative Incidence Rate at 1 Year After Baseline

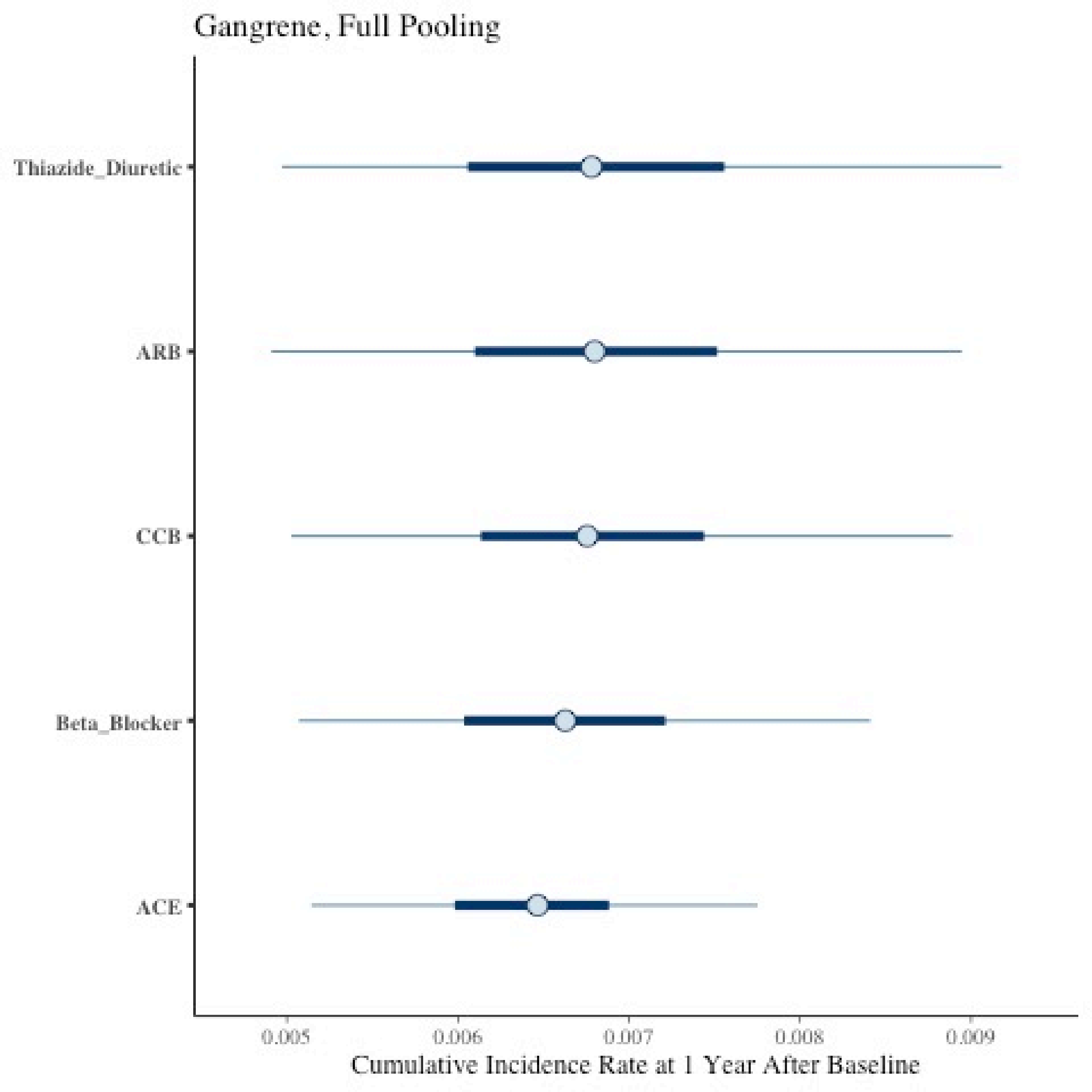

Gangrene, Full Pooling
Thiazide_Diuretic
ARB
CCB
Beta_Blocker
ACE
0.005
0.006
0.007
0.008
0.009
Cumulative Incidence Rate at 1 Year After Baseline

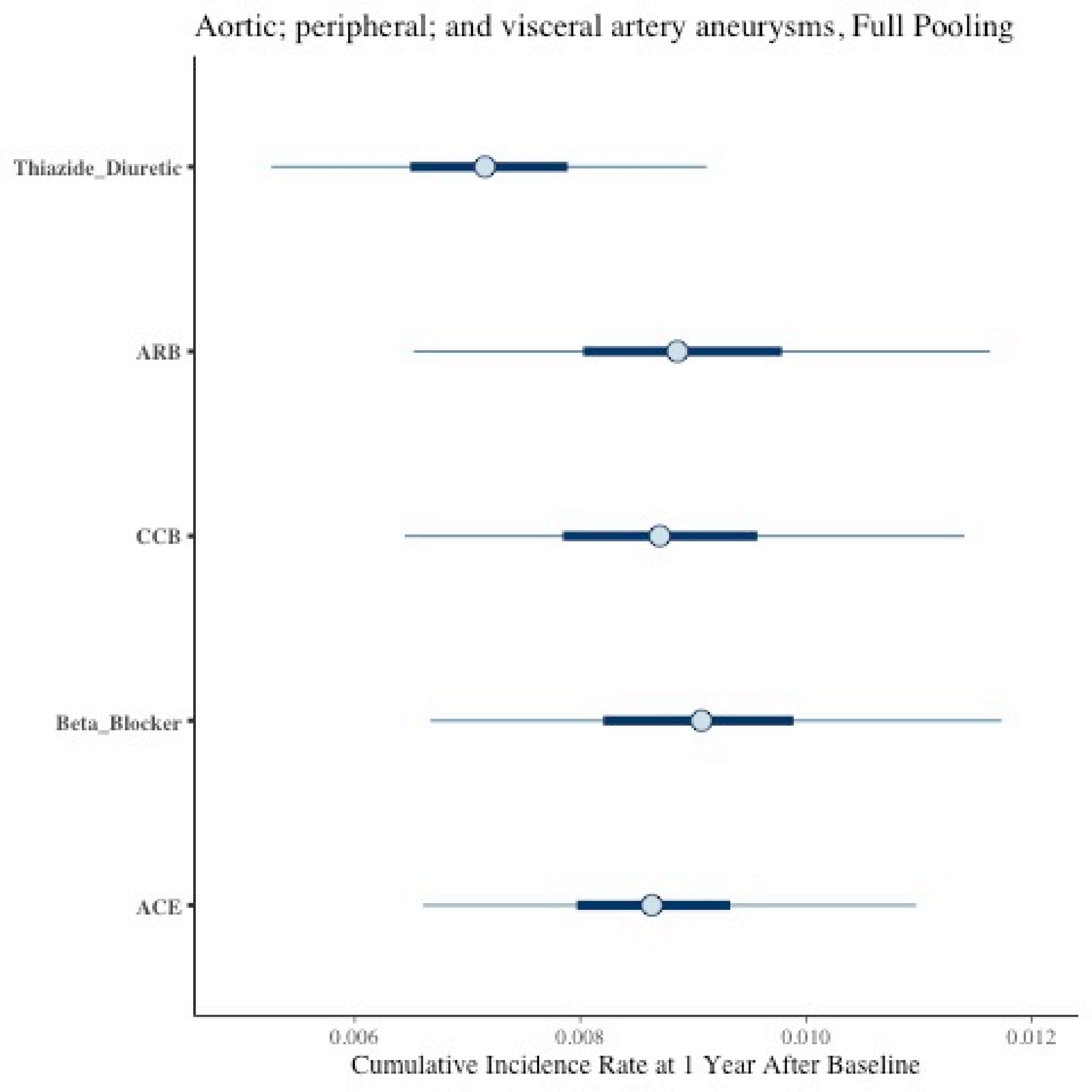

Aortic; peripheral; and visceral artery aneurysms, Full Pooling
Thiazide_Diuretic
ARB
CCB
Beta_Blocker
ACE
0.006
0.008
0.010
0.012
Cumulative Incidence Rate at 1 Year After Baseline

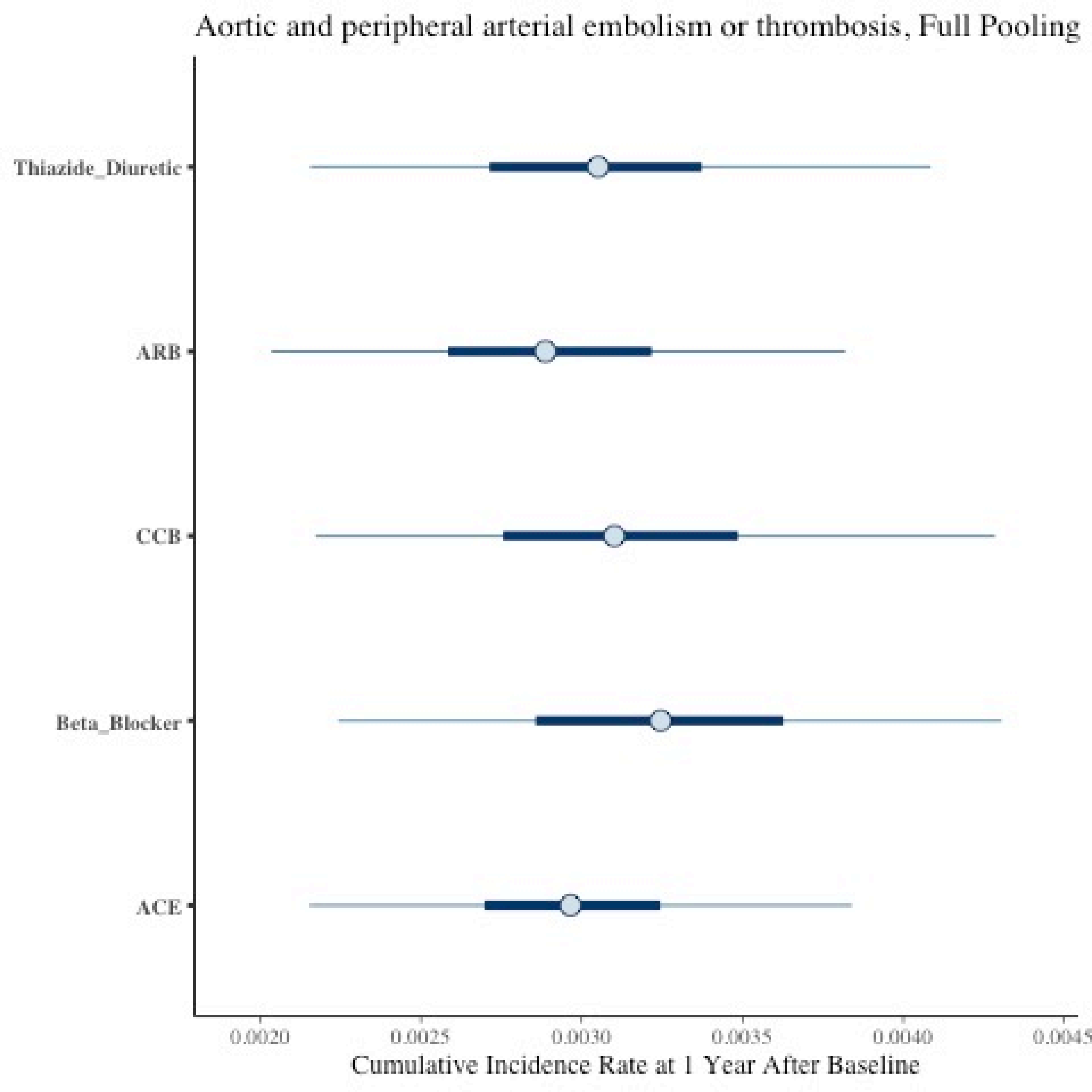

Aortic and peripheral arterial embolism or thrombosis, Full Pooling
Thiazide_Diuretic
ARB
CCB
Beta_Blocker
ACE
0.0020
0.0025
0.0030
0.0035
0.0040
0.0045
Cumulative Incidence Rate at 1 Year After Baseline

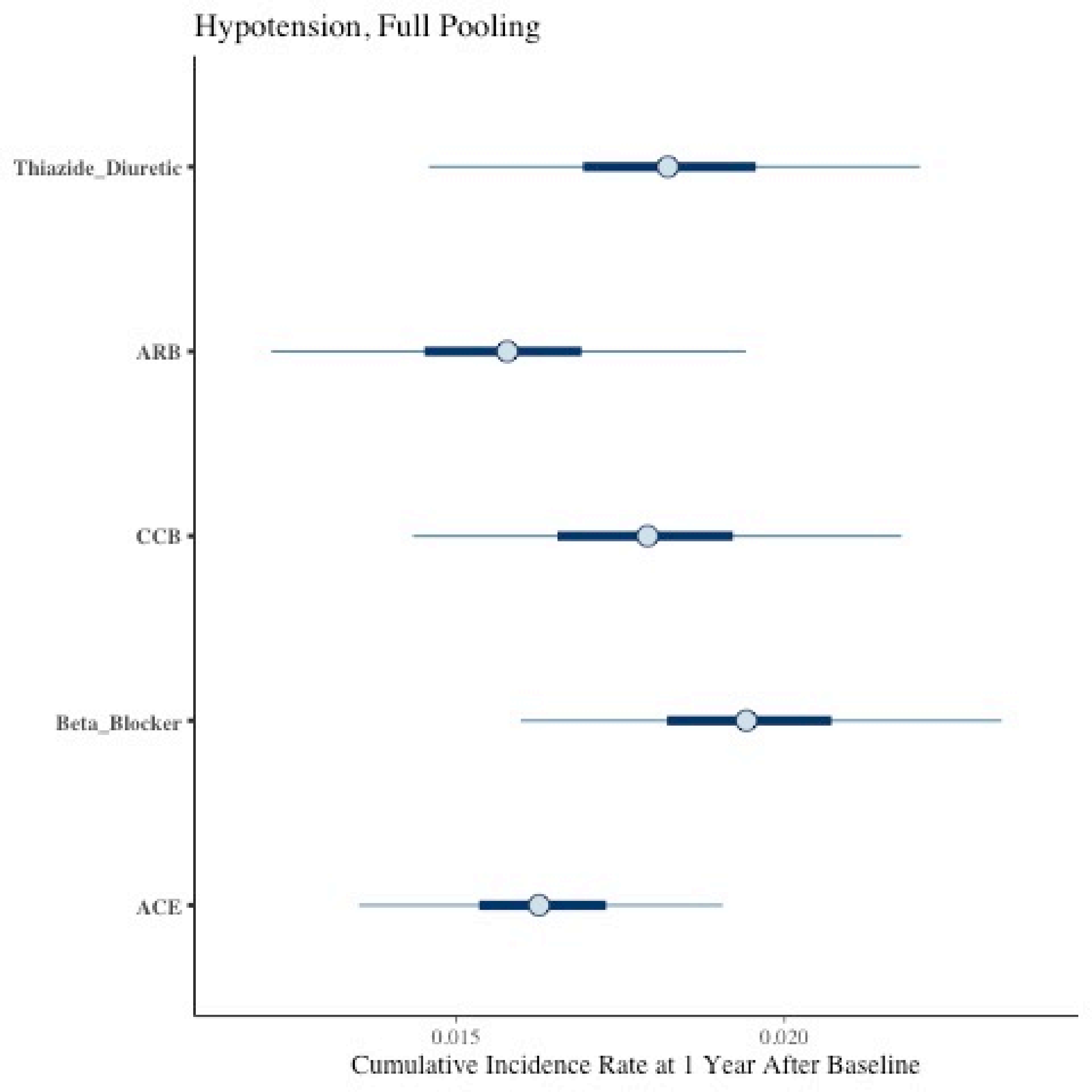

Hypotension, Full Pooling
Thiazide_Diuretic
ARB
CCB
Beta_Blocker
ACE
0.015
0.020
Cumulative Incidence Rate at 1 Year After Baseline

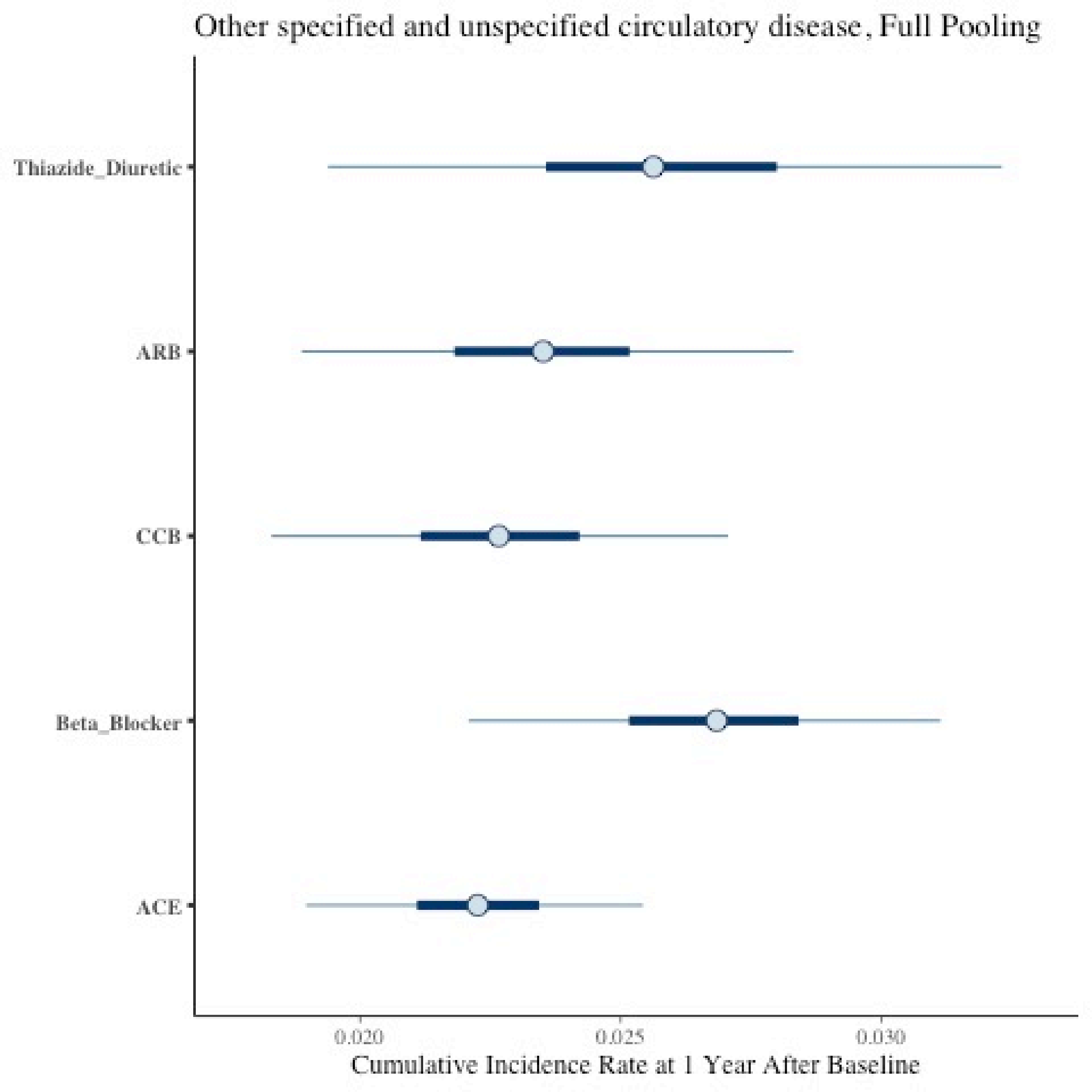

Other specified and unspecified circulatory disease, Full Pooling
Thiazide_Diuretic
ARB
CCB
Beta_Blocker
ACE
0.020
0.025
0.030
Cumulative Incidence Rate at 1 Year After Baseline

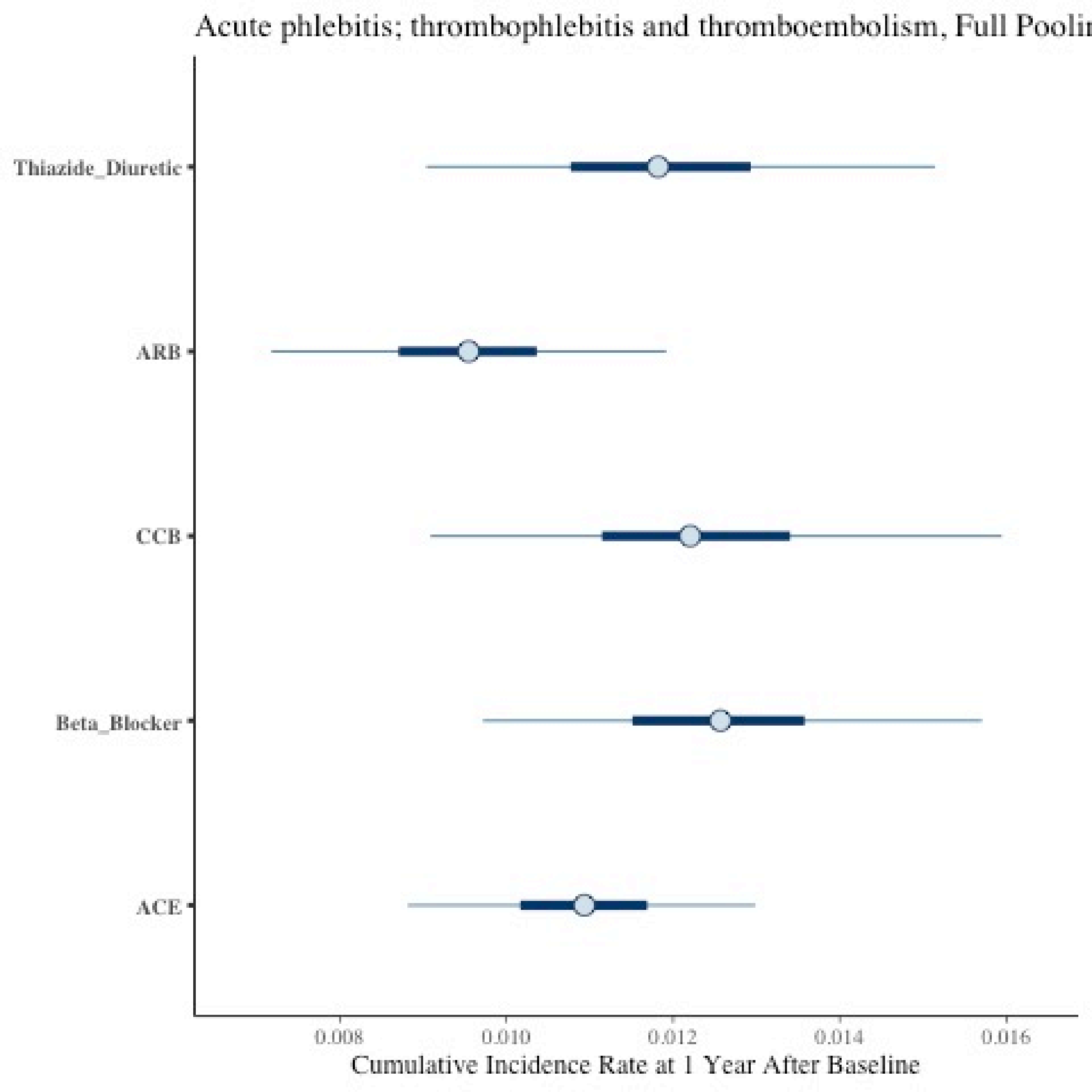

Acute phlebitis; thrombophlebitis and thromboembolism, Full Poolin
Thiazide_Diuretic
ARB
CCB
Beta_Blocker
ACE
0.008
0.010
0.012
0.014
0.016
Cumulative Incidence Rate at 1 Year After Baseline

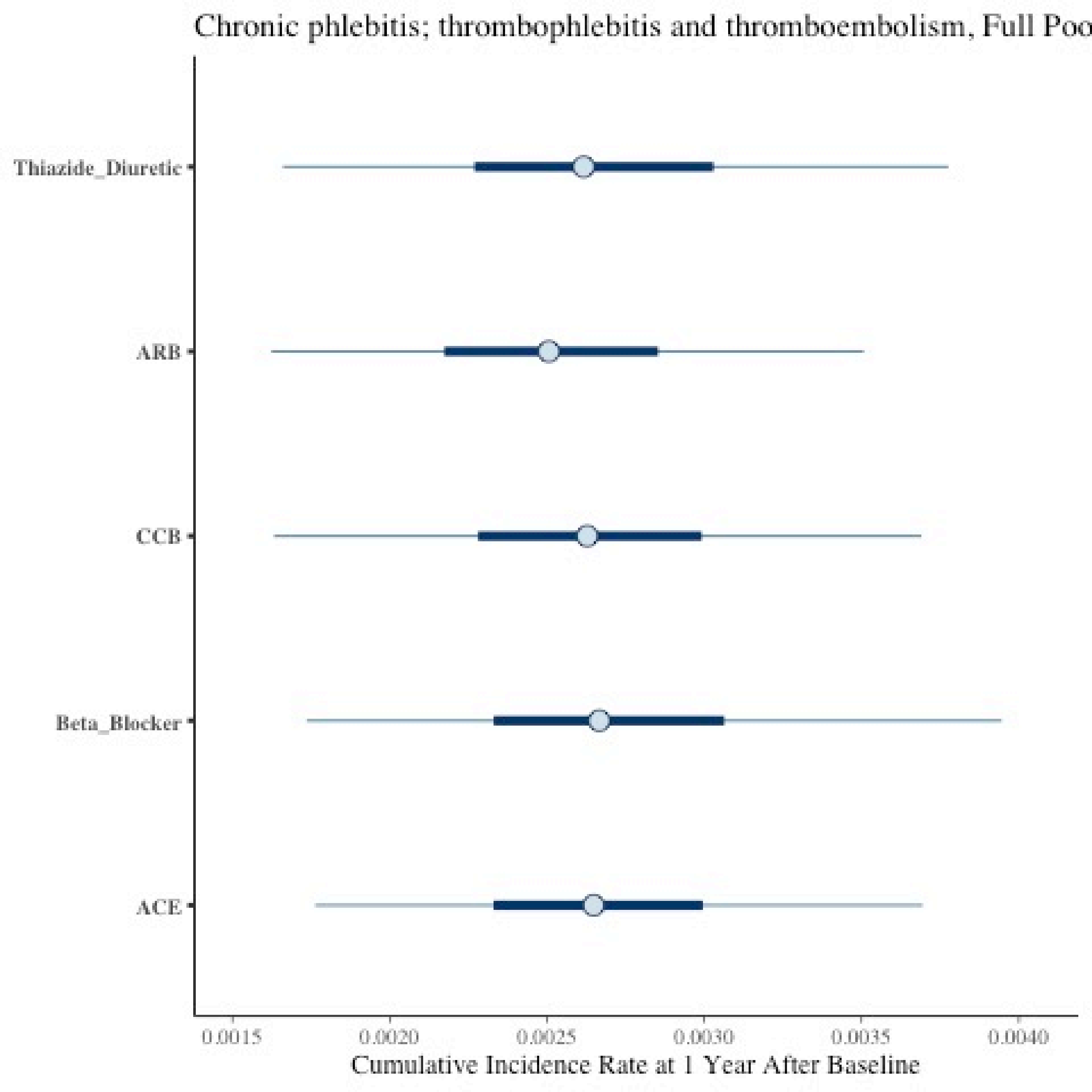
Chronic phlebitis; thrombophlebitis and thromboembolism, Full Poo
Thiazide_Diuretic
ARB
CCB
Beta_Blocker
ACE
0.0015
0.0020
0.0025
0.0030
0.0035
0.0040
Cumulative Incidence Rate at 1 Year After Baseline

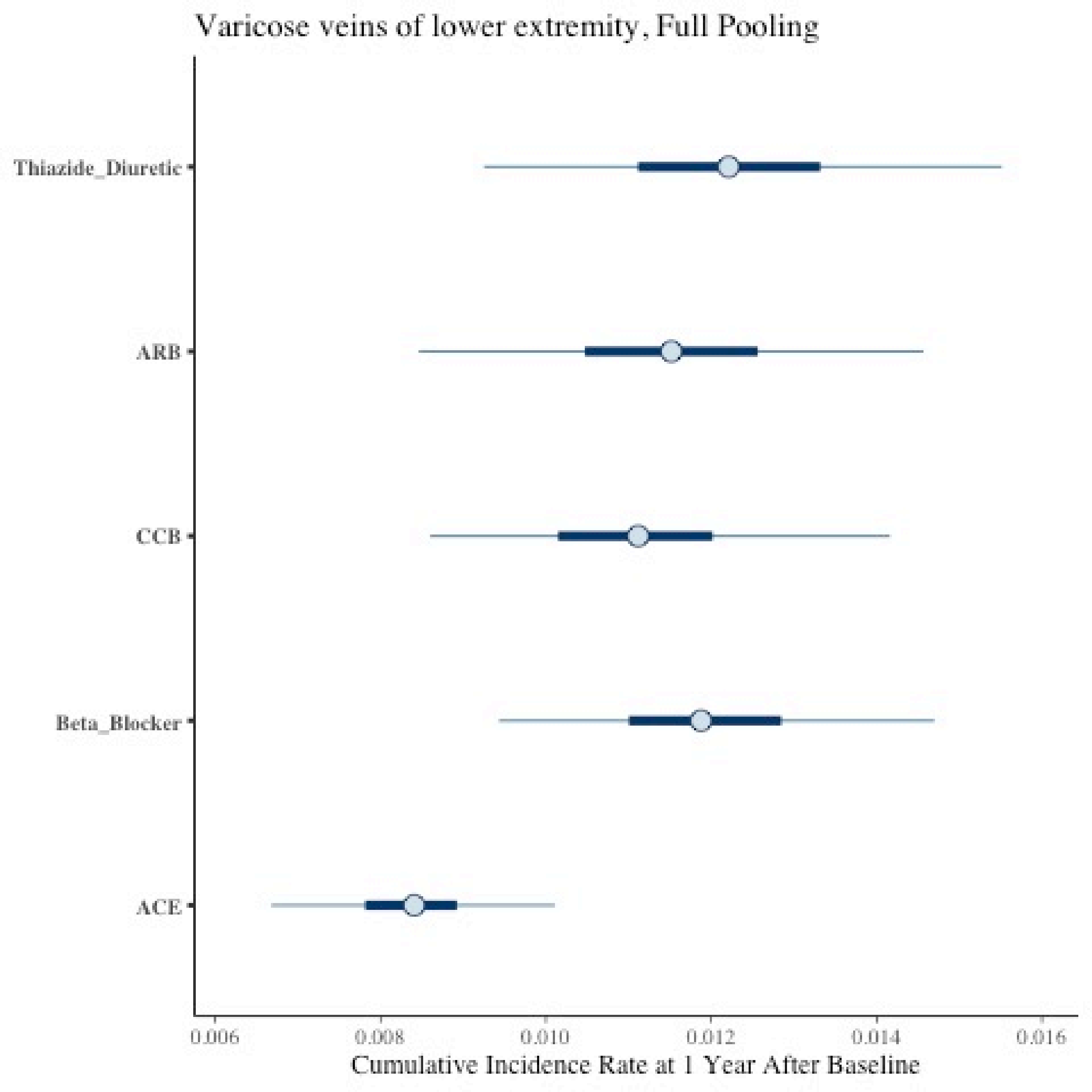

Varicose veins of lower extremity, Full Pooling
Thiazide_Diuretic
ARB
CCB
Beta_Blocker
ACE
0.006
0.008
0.010
0.012
0.014
0.016
Cumulative Incidence Rate at 1 Year After Baseline

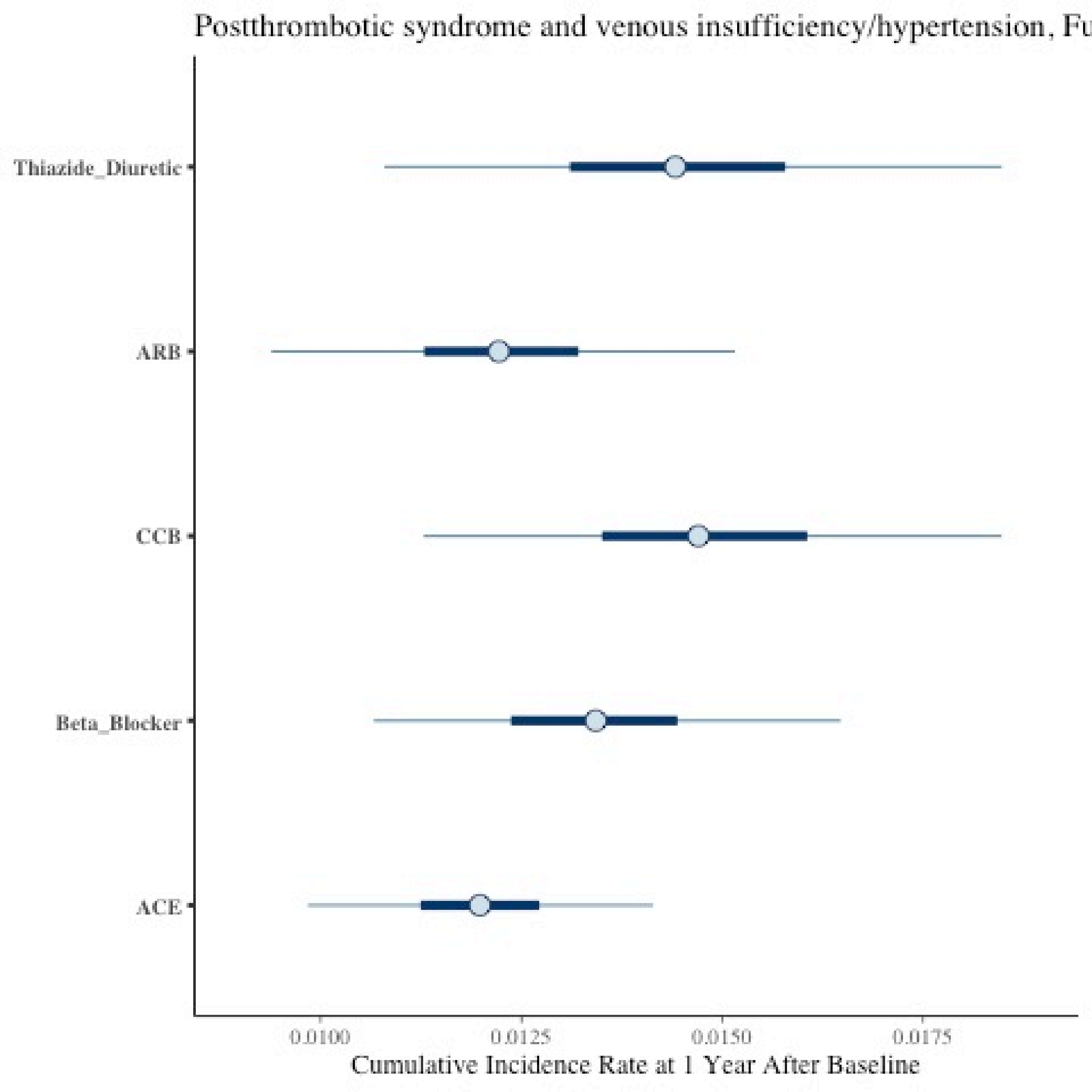

Postthrombotic syndrome and venous insufficiency/hypertension, Fu
Thiazide_Diuretic
ARB
CCB
Beta_Blocker
ACE
0.0100
0.0125
0.0150
0.0175
Cumulative Incidence Rate at 1 Year After Baseline

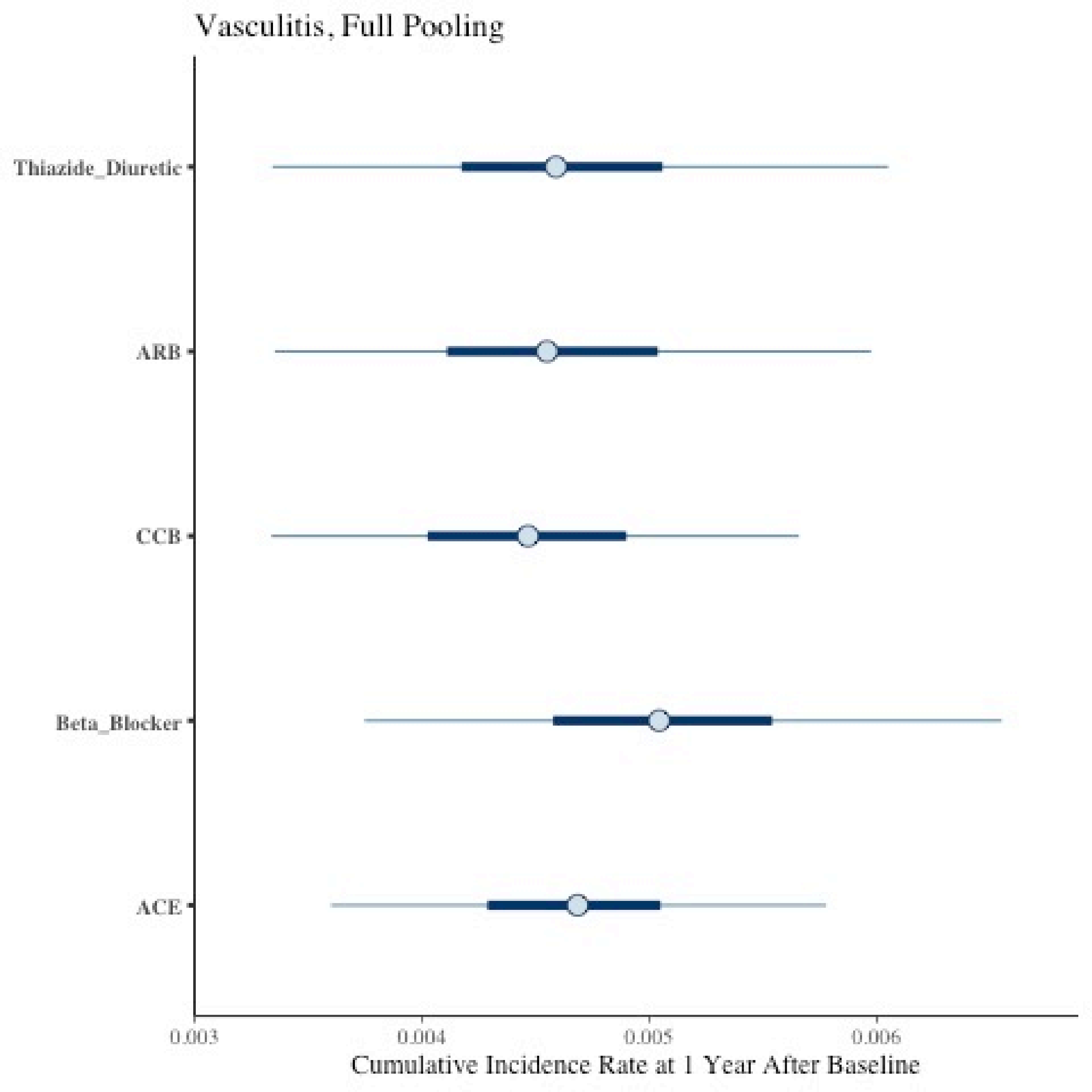

Vasculitis, Full Pooling
Thiazide_Diuretic
ARB
CCB
Beta_Blocker
ACE
0.003
0.004
0.005
0.006
Cumulative Incidence Rate at 1 Year After Baseline

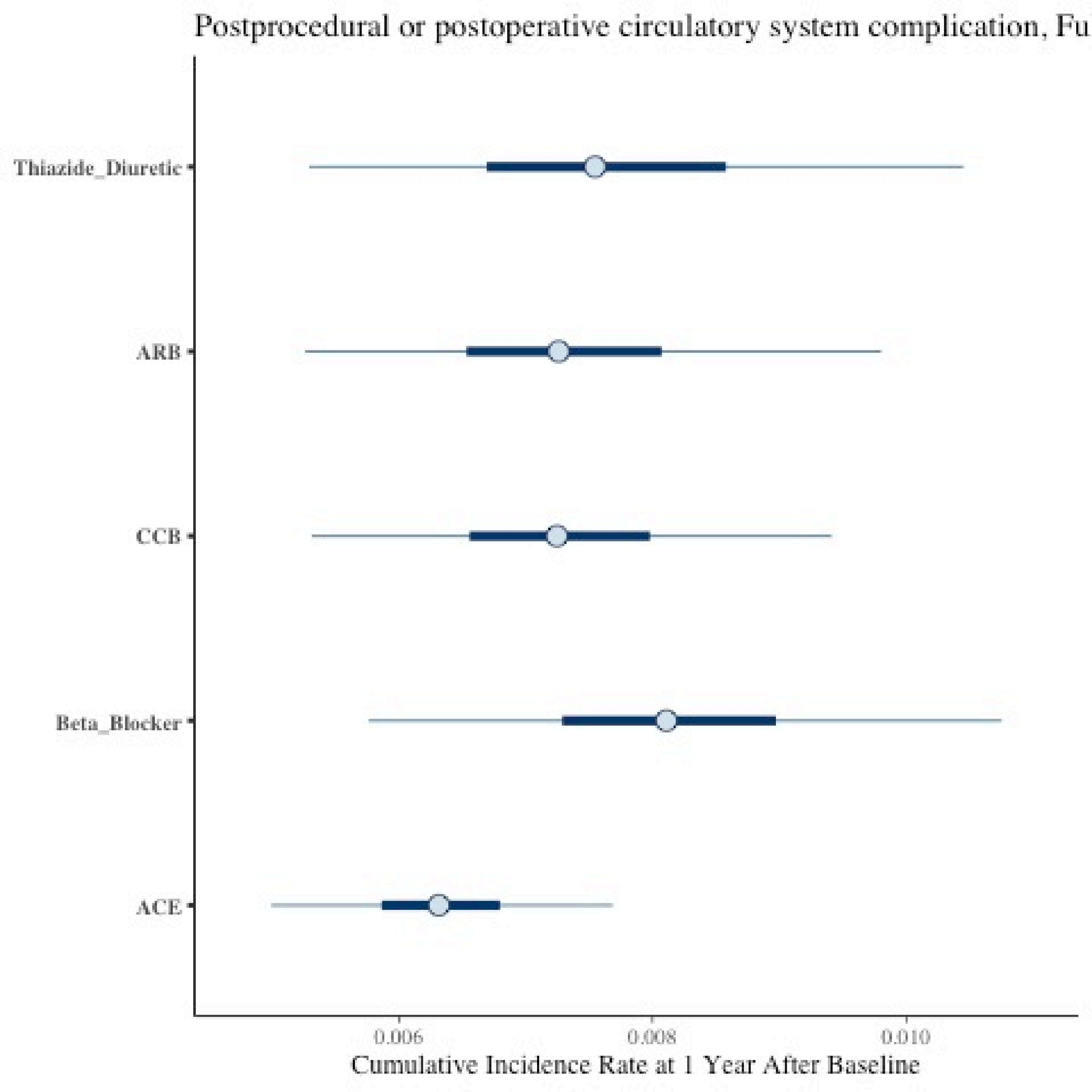

Postprocedural or postoperative circulatory system complication, Fu
Thiazide_Diuretic
ARB
CCB
Beta_Blocker
ACE
0.006
0.008
0.010
Cumulative Incidence Rate at 1 Year After Baseline

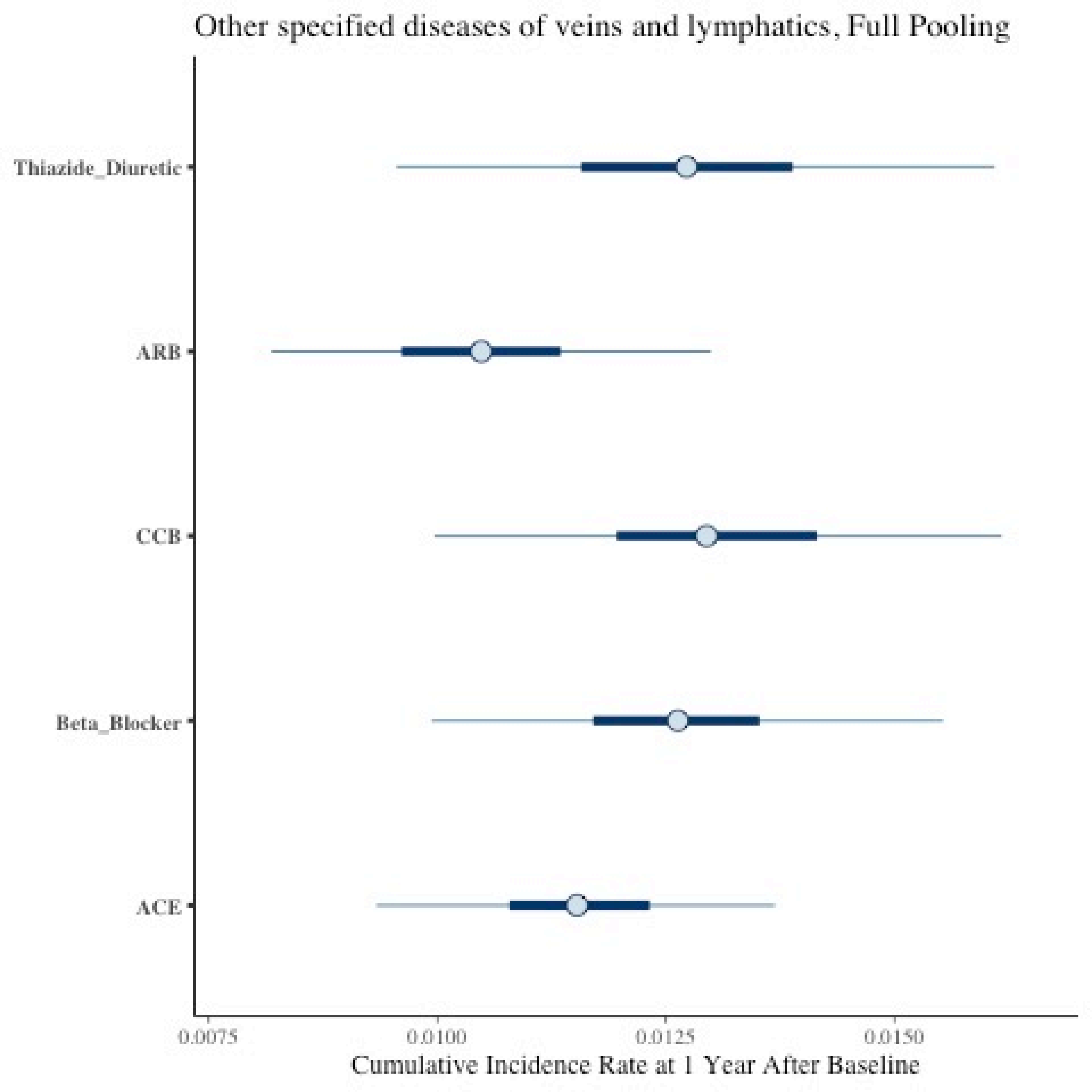

Other specified diseases of veins and lymphatics, Full Pooling
Thiazide_Diuretic
ARB
CCB
Beta_Blocker
ACE
0.0075
0.0100
0.0125
0.0150
Cumulative Incidence Rate at 1 Year After Baseline

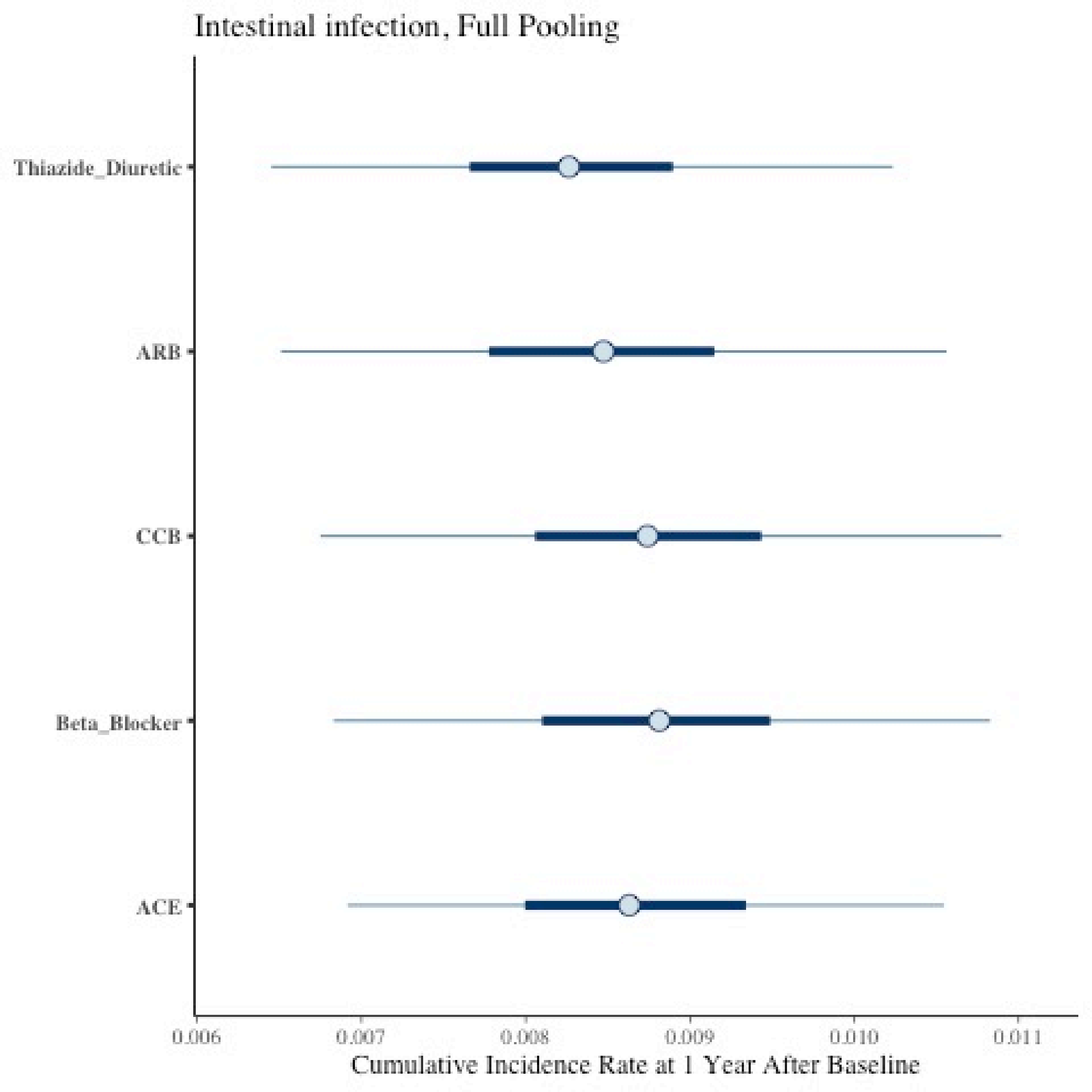

Intestinal infection, Full Pooling
Thiazide_Diuretic
ARB
CCB
Beta_Blocker
ACE
0.006
0.007
0.008
0.009
0.010
0.011
Cumulative Incidence Rate at 1 Year After Baseline

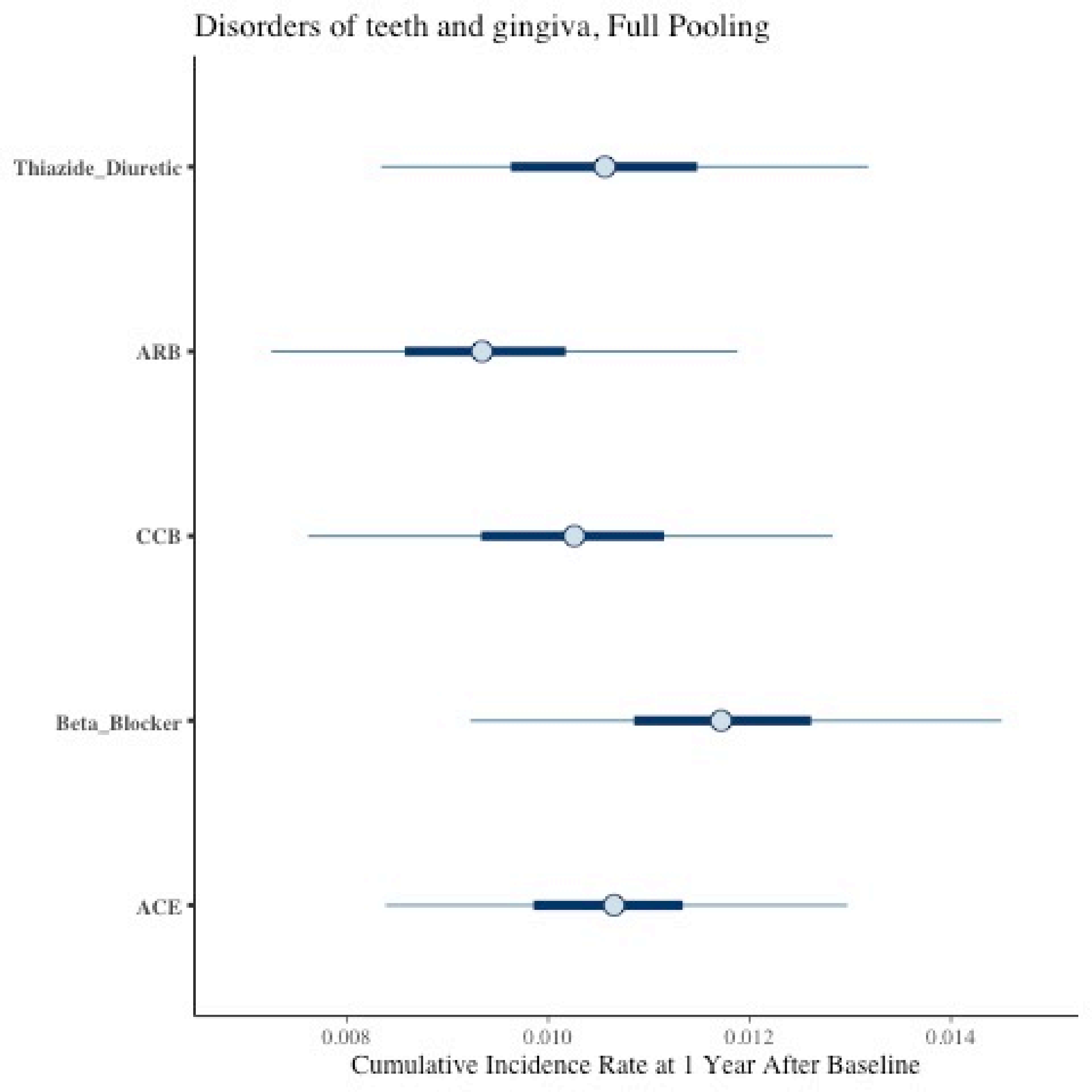

Disorders of teeth and gingiva, Full Pooling
Thiazide_Diuretic
ARB
CCB
Beta_Blocker
ACE
0.008
0.010
0.012
0.014
Cumulative Incidence Rate at 1 Year After Baseline

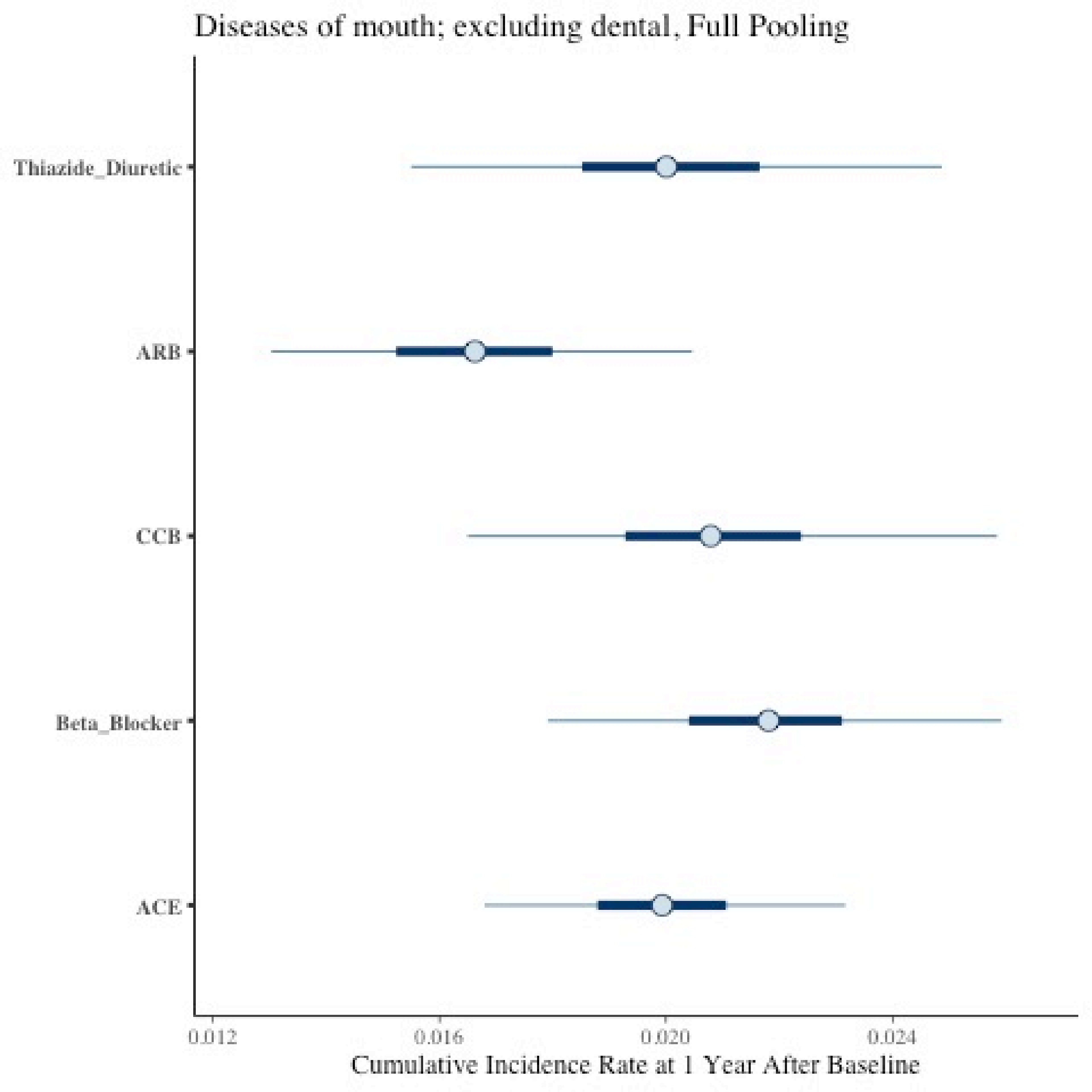
Diseases of mouth; excluding dental, Full Pooling
Thiazide_Diuretic
ARB
CCB
Beta_Blocker
ACE
0.012
0.016
0.020
0.024
Cumulative Incidence Rate at 1 Year After Baseline

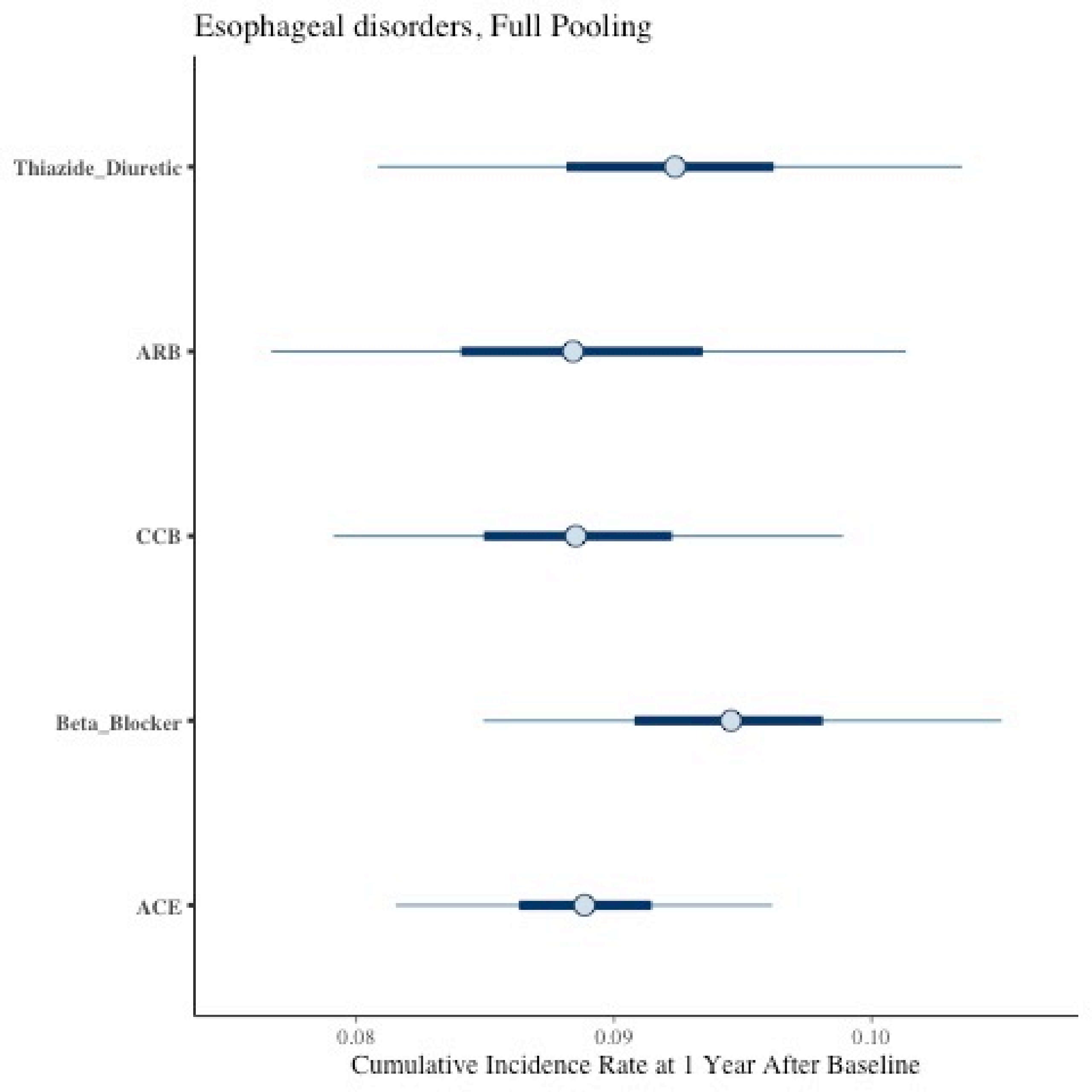

Esophageal disorders, Full Pooling
Thiazide_Diuretic
ARB
CCB
Beta_Blocker
ACE
0.08
0.09
0.10
Cumulative Incidence Rate at 1 Year After Baseline

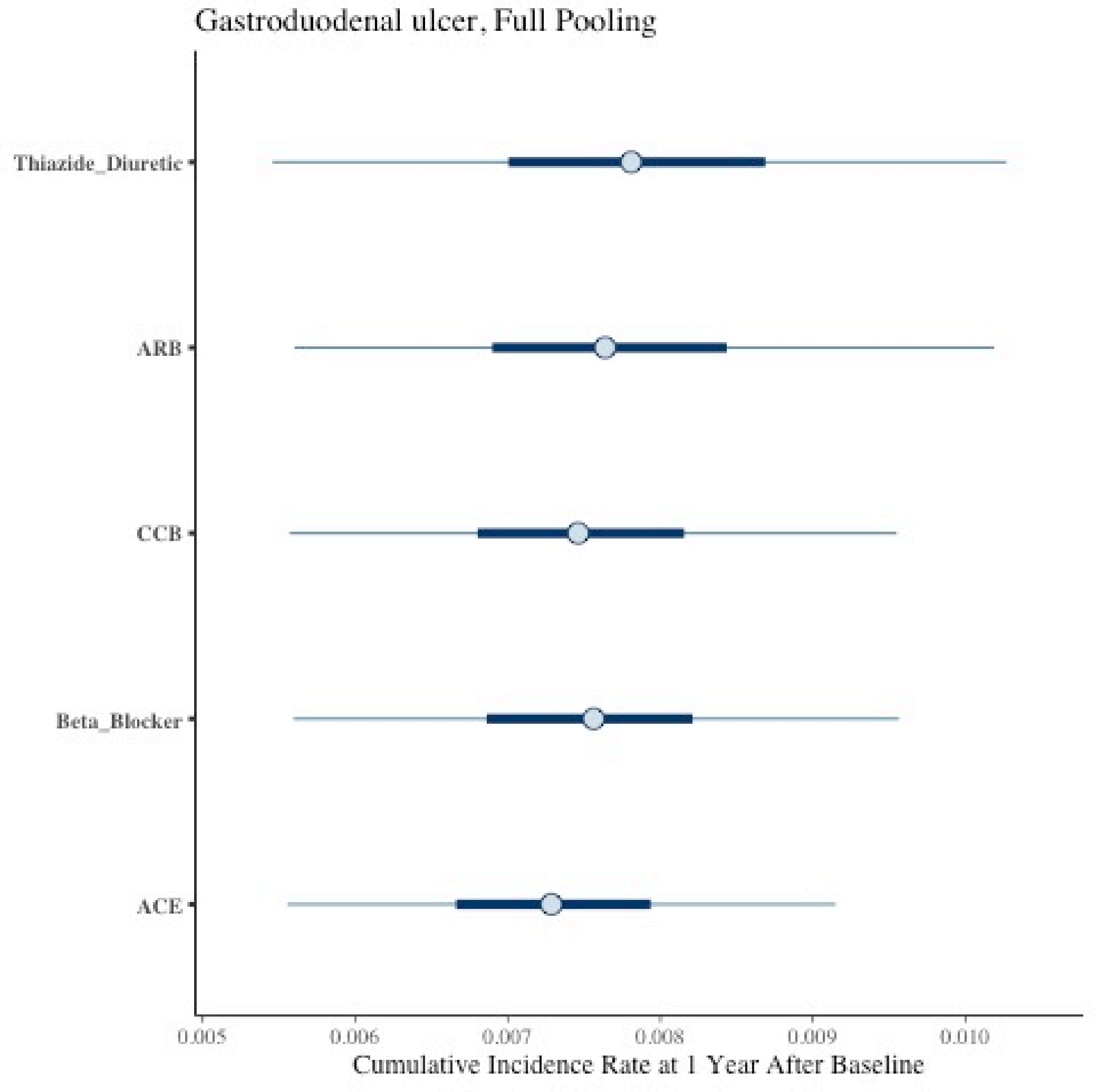

Gastroduodenal ulcer, Full Pooling
Thiazide_Diuretic
ARB
CCB
Beta_Blocker
ACE
0.005
0.006
0.007
0.008
0.009
0.010
Cumulative Incidence Rate at 1 Year After Baseline

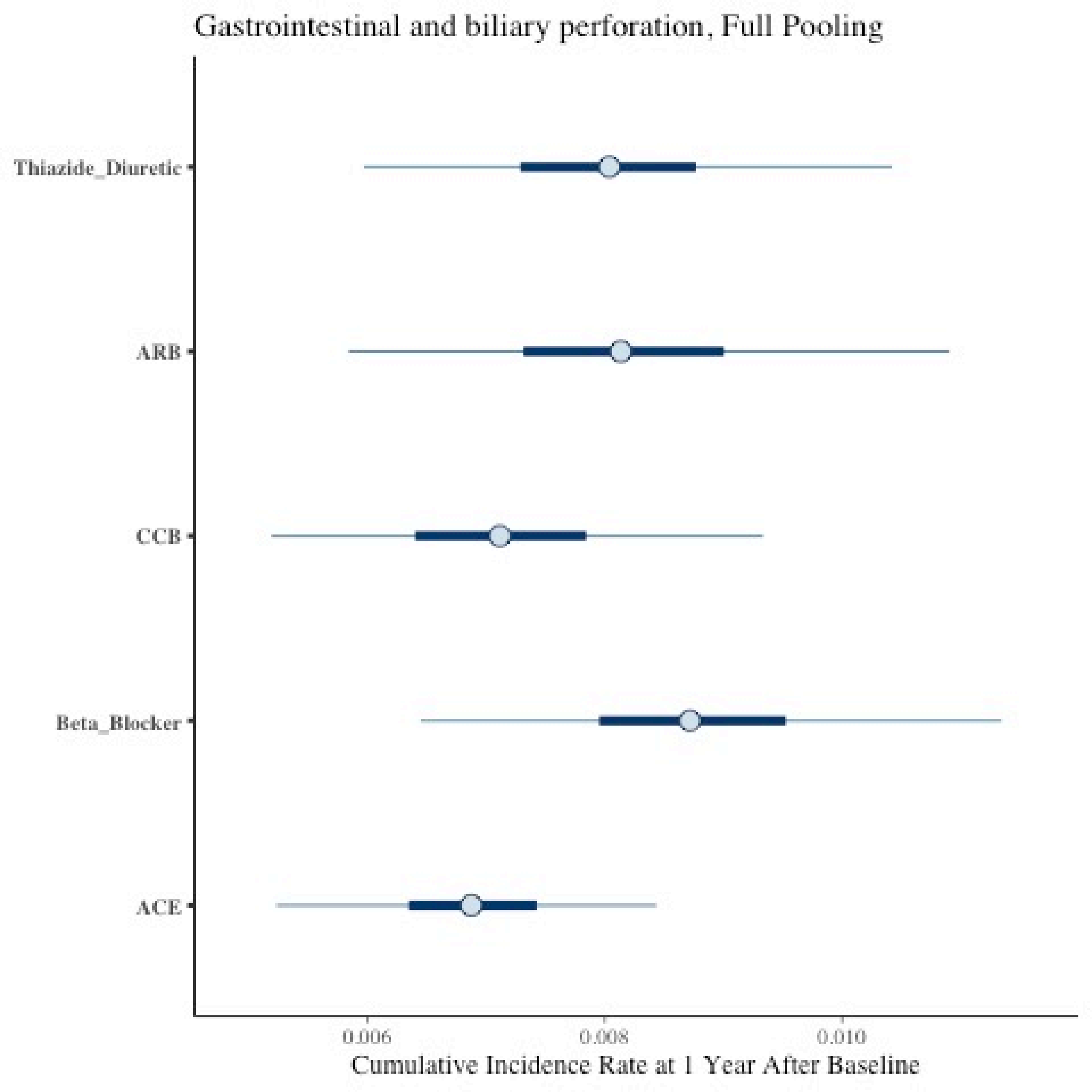

Gastrointestinal and biliary perforation, Full Pooling
Thiazide_Diuretic
ARB
CCB
Beta_Blocker
ACE
0.006
0.008
0.010
Cumulative Incidence Rate at 1 Year After Baseline

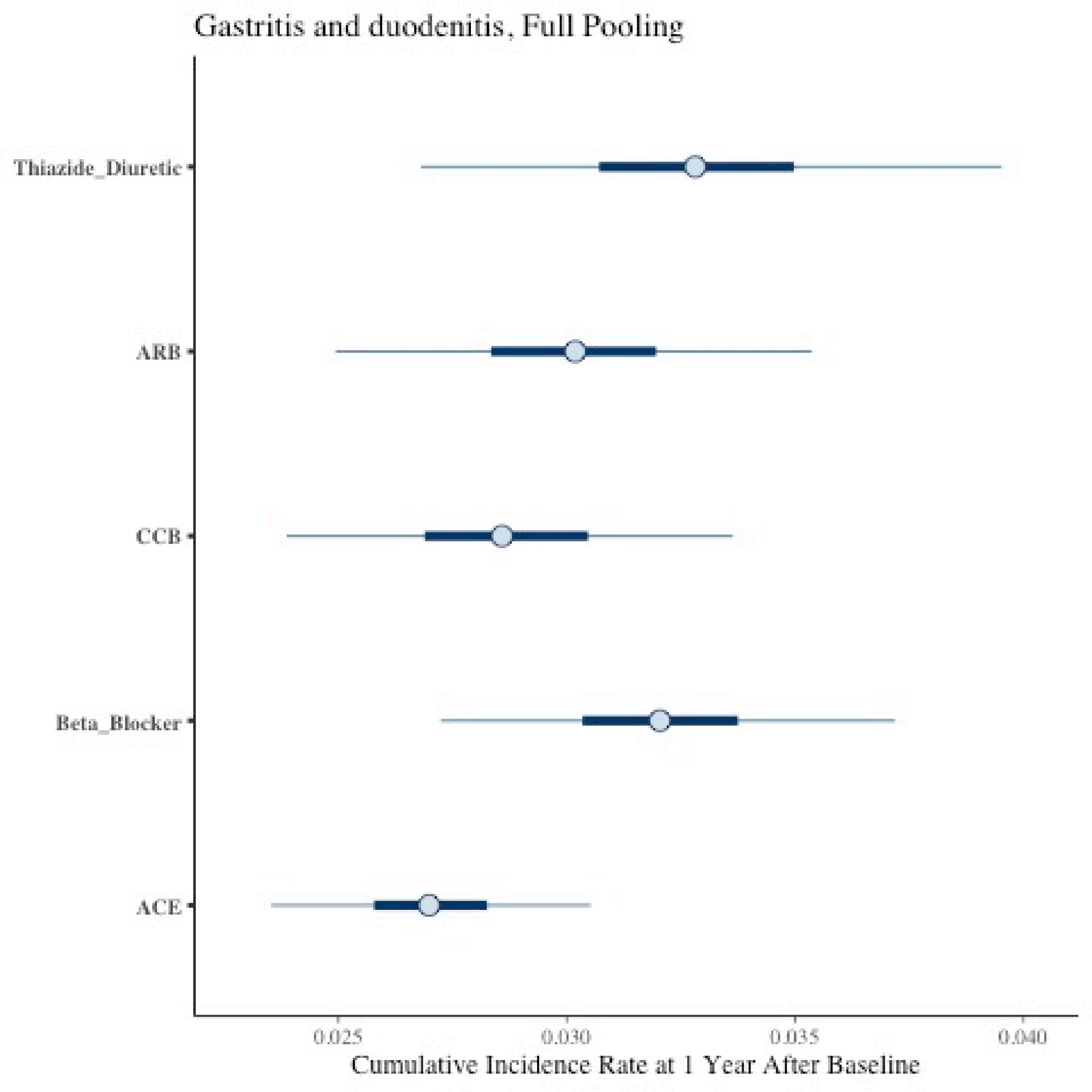

Gastritis and duodenitis, Full Pooling
Thiazide_Diuretic
ARB
CCB
Beta_Blocker
ACE
0.025
0.030
0.035
0.040
Cumulative Incidence Rate at 1 Year After Baseline

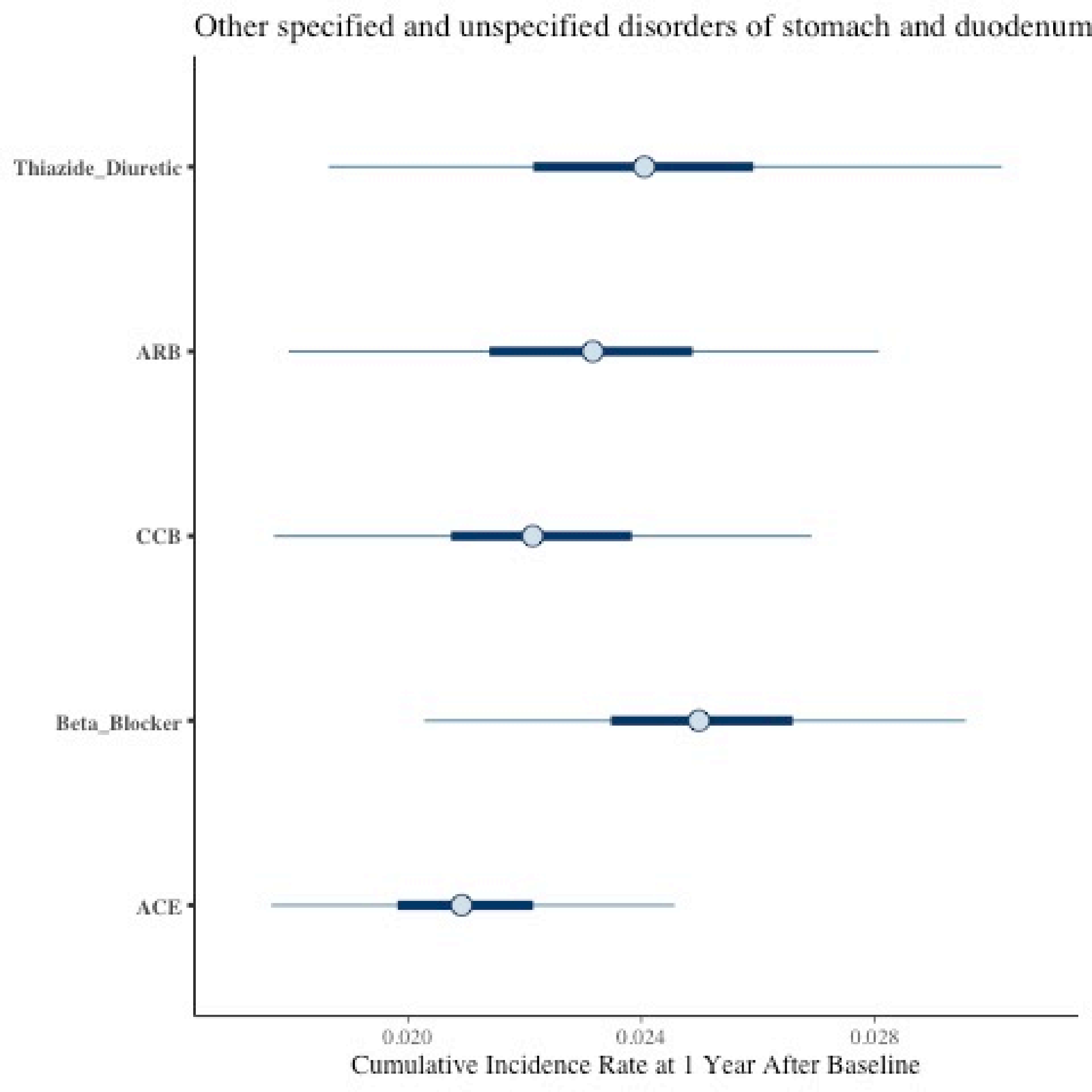

Other specified and unspecified disorders of stomach and duodenum
Thiazide_Diuretic
ARB
CCB
Beta_Blocker
ACE
0.020
0.024
0.028
Cumulative Incidence Rate at 1 Year After Baseline

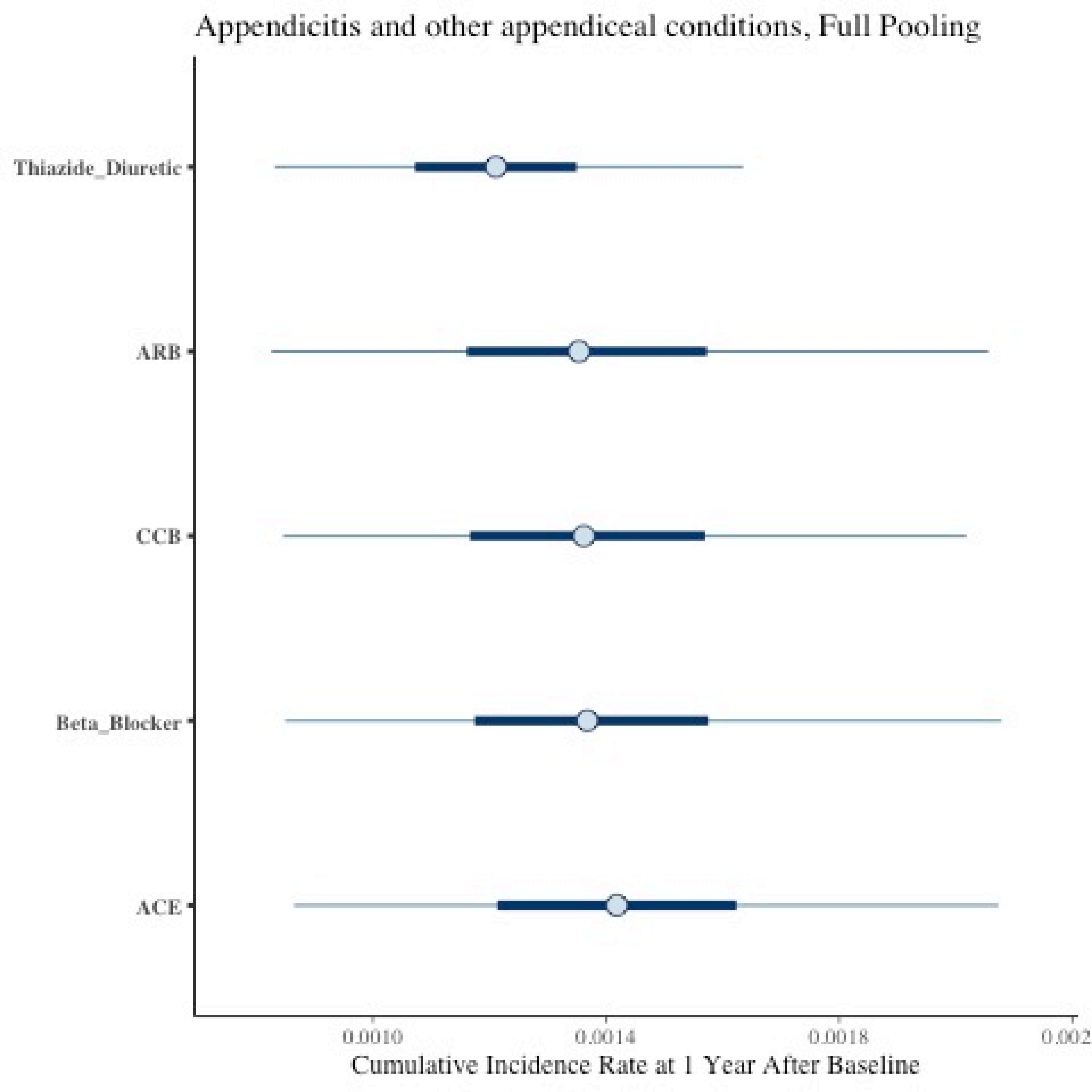

Appendicitis and other appendiceal conditions, Full Pooling
Thiazide_Diuretic
ARB
CCB
Beta_Blocker
ACE
0.0010
0.0014
0.0018
0.002
Cumulative Incidence Rate at 1 Year After Baseline

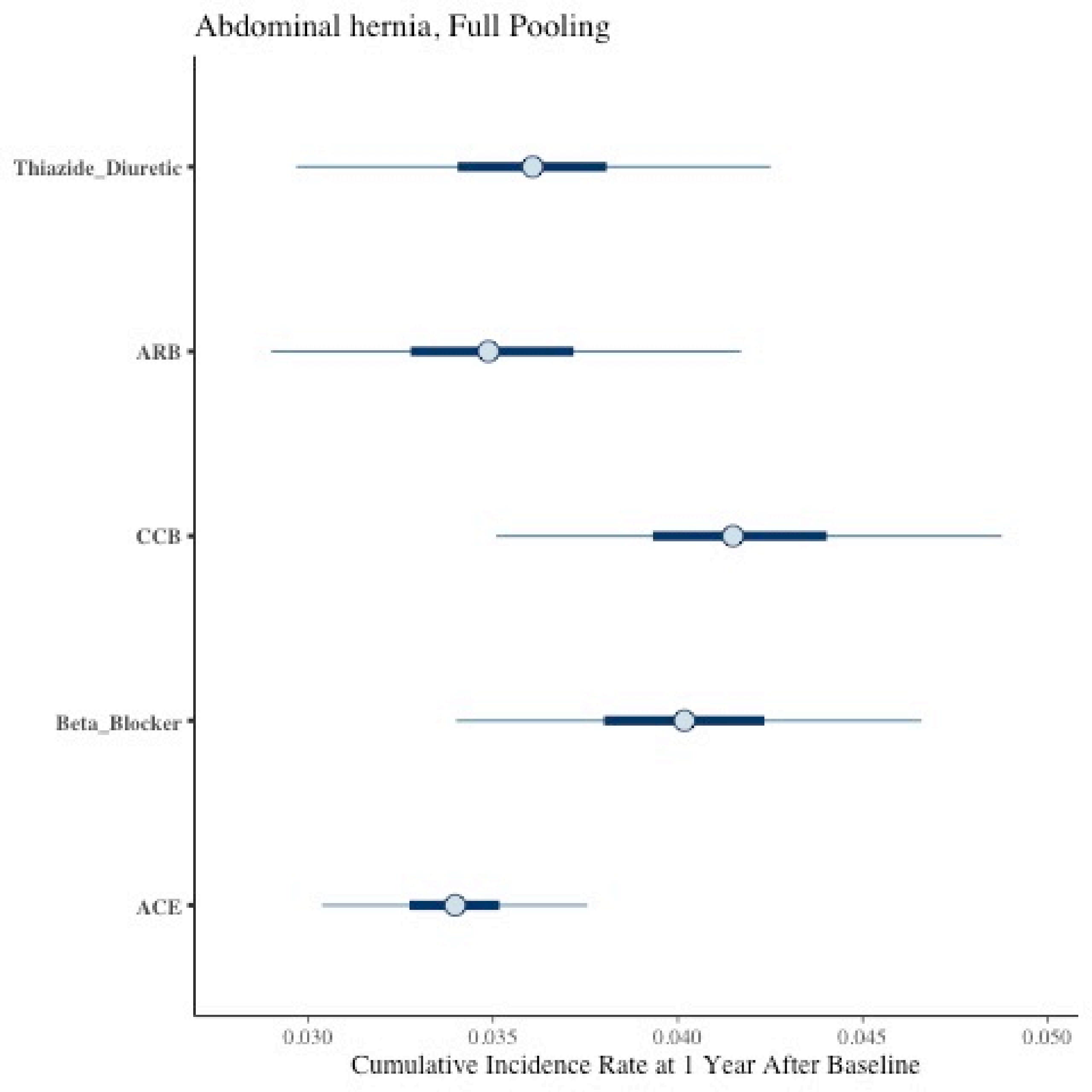
Abdominal hernia, Full Pooling
Thiazide_Diuretic
ARB
CCB
Beta_Blocker
ACE
0.030
0.035
0.040
0.045
0.050
Cumulative Incidence Rate at 1 Year After Baseline

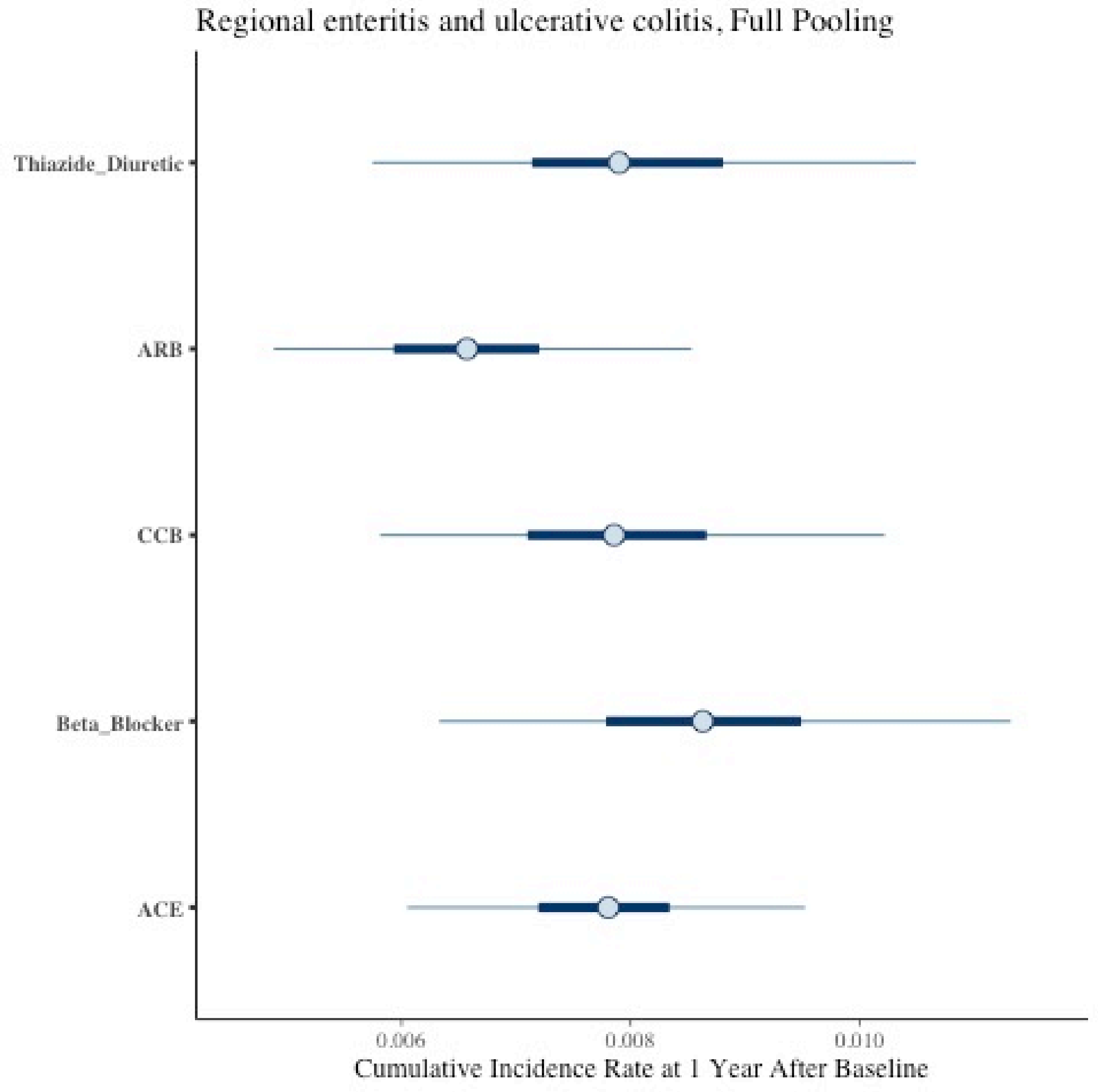

Regional enteritis and ulcerative colitis, Full Pooling
Thiazide_Diuretic
ARB
CCB
Beta_Blocker
ACE
0.006
0.008
0.010
Cumulative Incidence Rate at 1 Year After Baseline

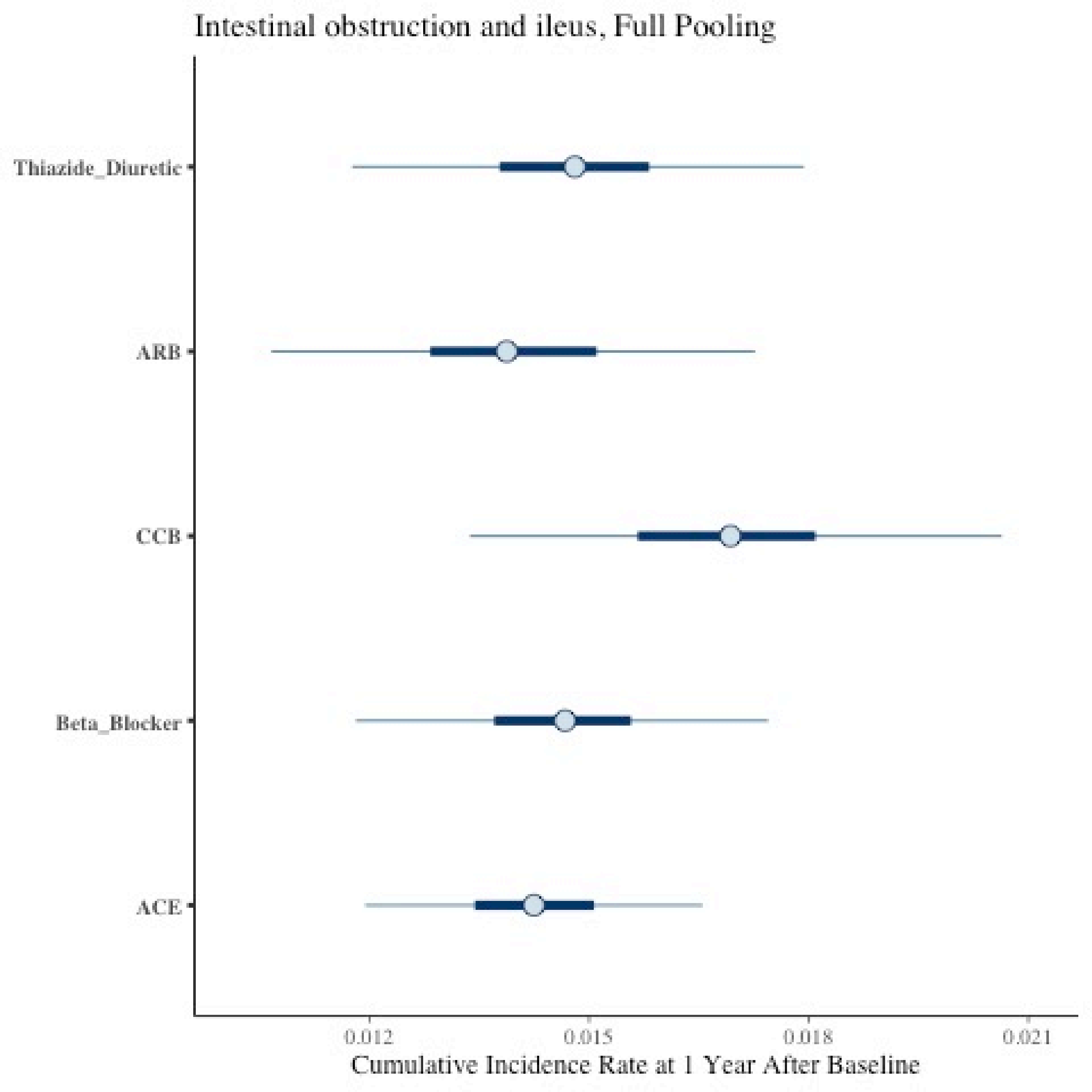
Intestinal obstruction and ileus, Full Pooling
Thiazide_Diuretic
ARB
CCB
Beta_Blocker
ACE
0.012
0.015
0.018
0.021
Cumulative Incidence Rate at 1 Year After Baseline

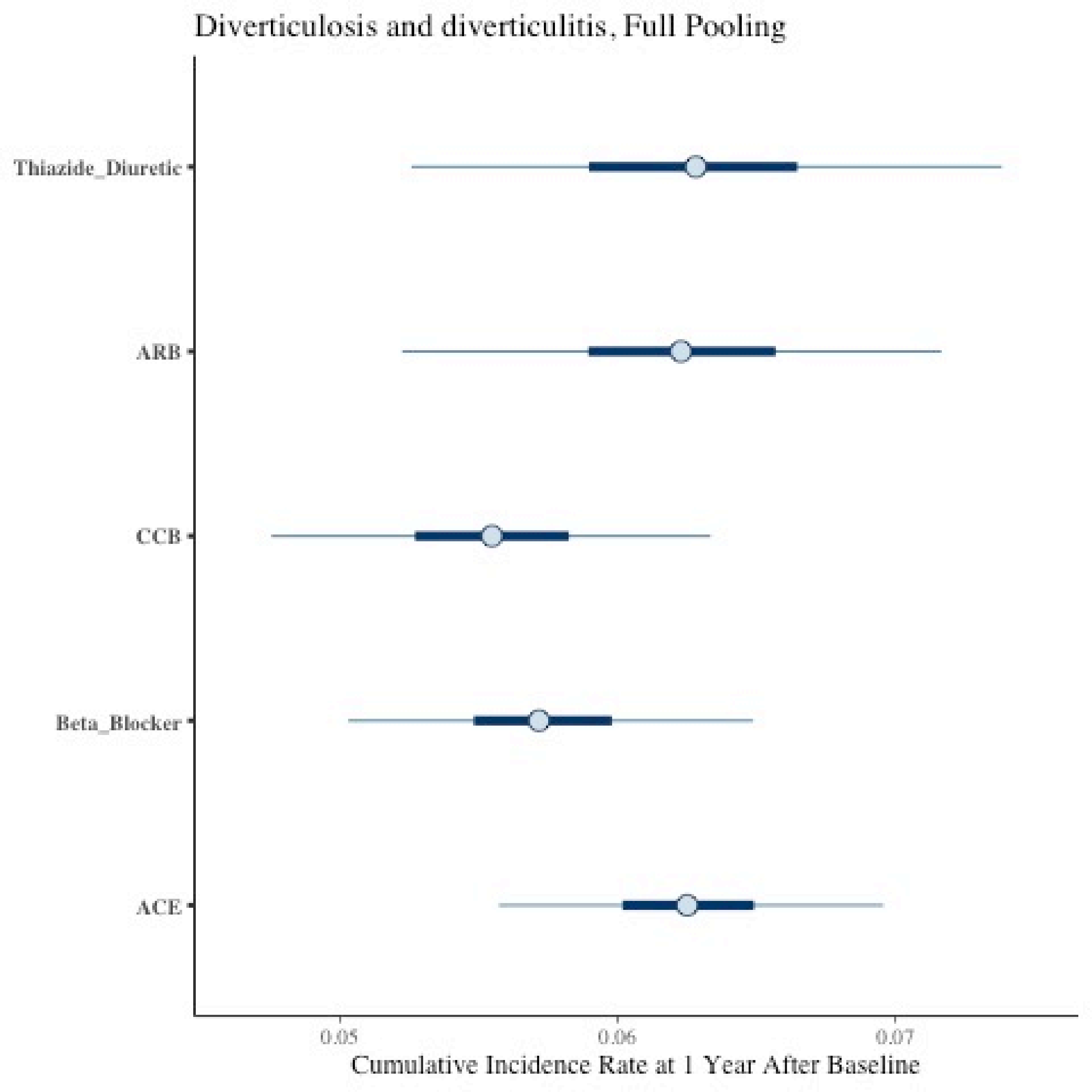

Diverticulosis and diverticulitis, Full Pooling
Thiazide_Diuretic
ARB
CCB
Beta_Blocker
ACE
0.05
0.06
0.07
Cumulative Incidence Rate at 1 Year After Baseline

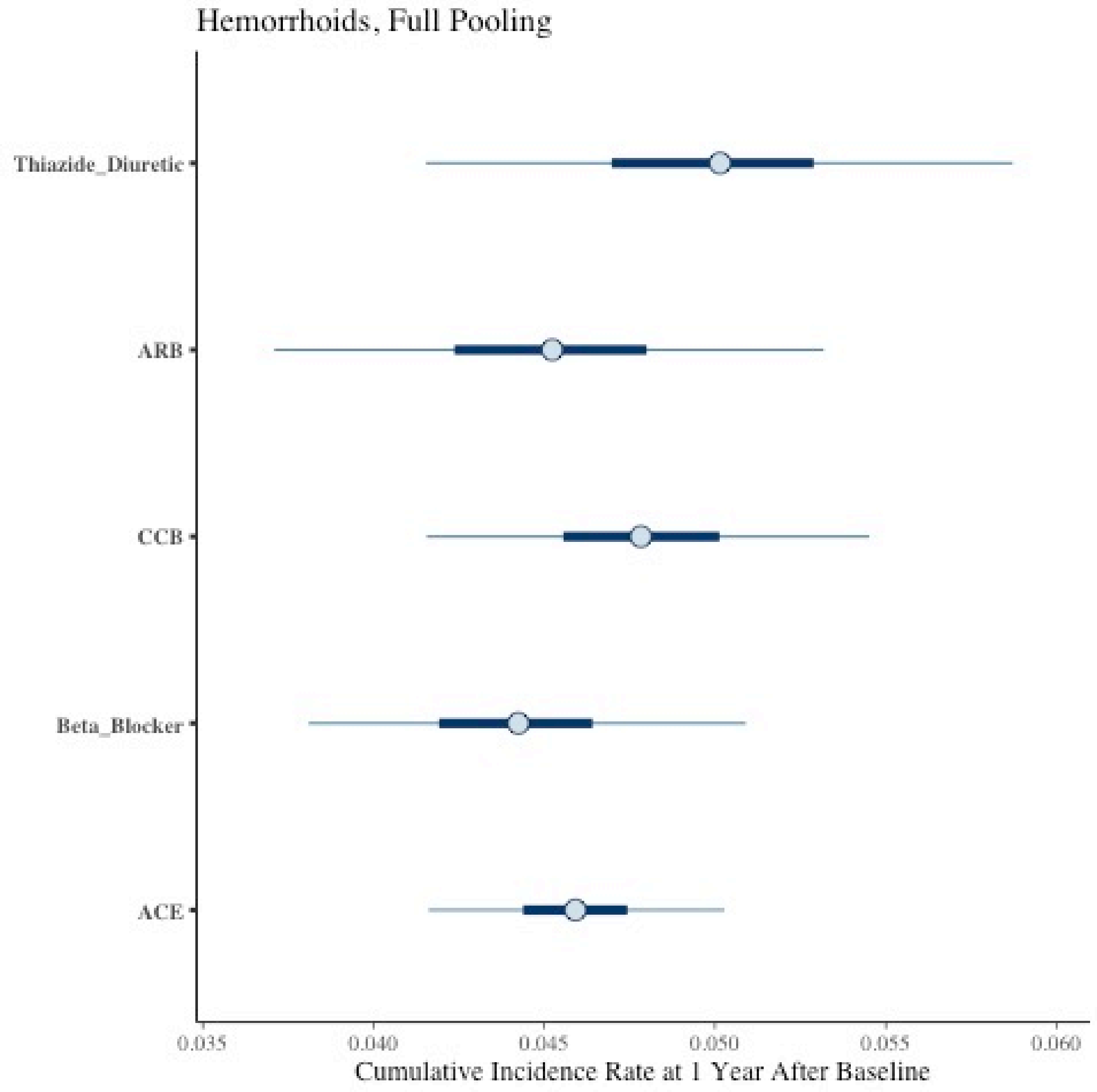

Hemorrhoids, Full Pooling
Thiazide_Diuretic
ARB
CCB
Beta_Blocker
ACE
0.035
0.040
0.045
0.050
0.055
0.060
Cumulative Incidence Rate at 1 Year After Baseline

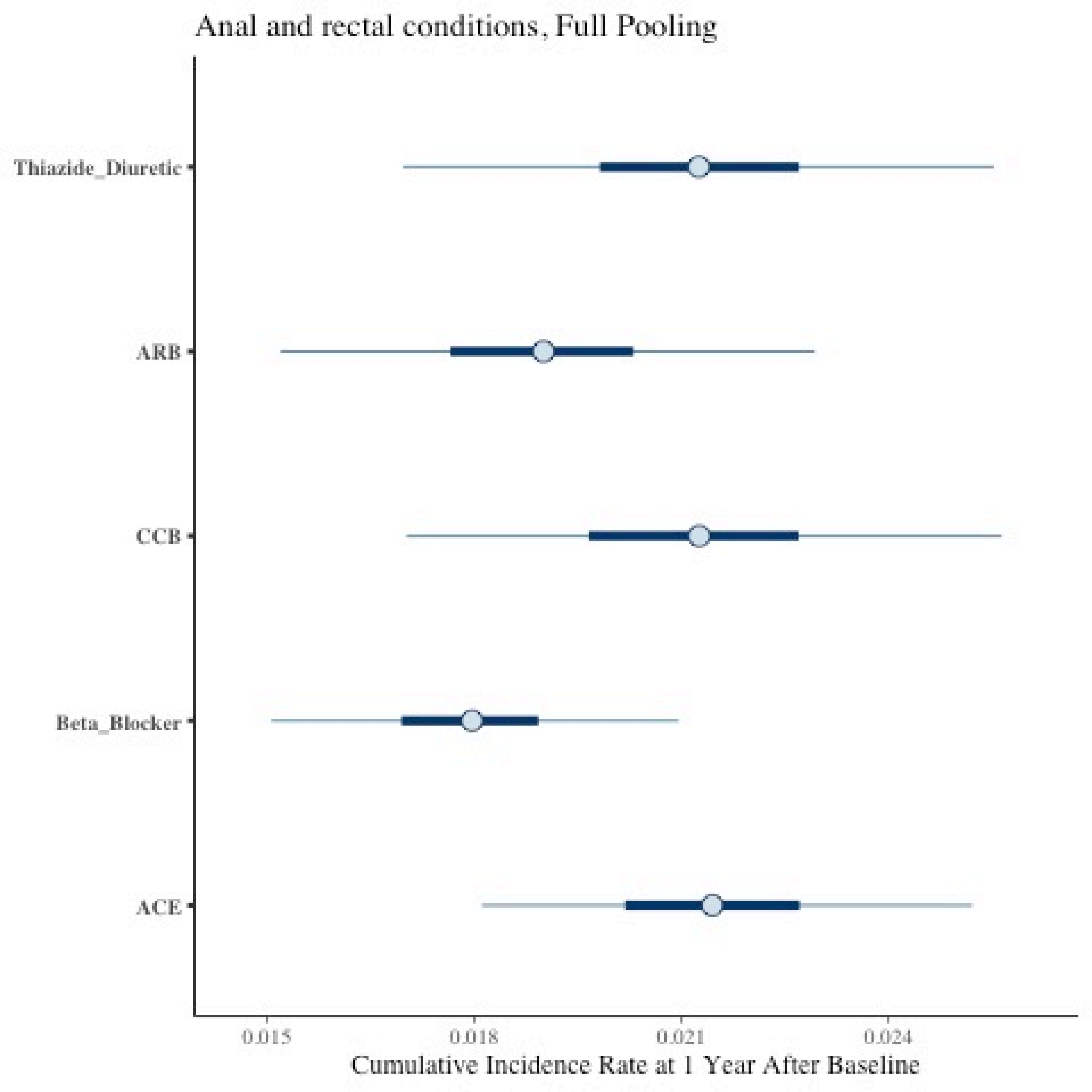
Anal and rectal conditions, Full Pooling
Thiazide_Diuretic
ARB
CCB
Beta_Blocker
ACE
0.015
0.018
0.021
0.024
Cumulative Incidence Rate at 1 Year After Baseline

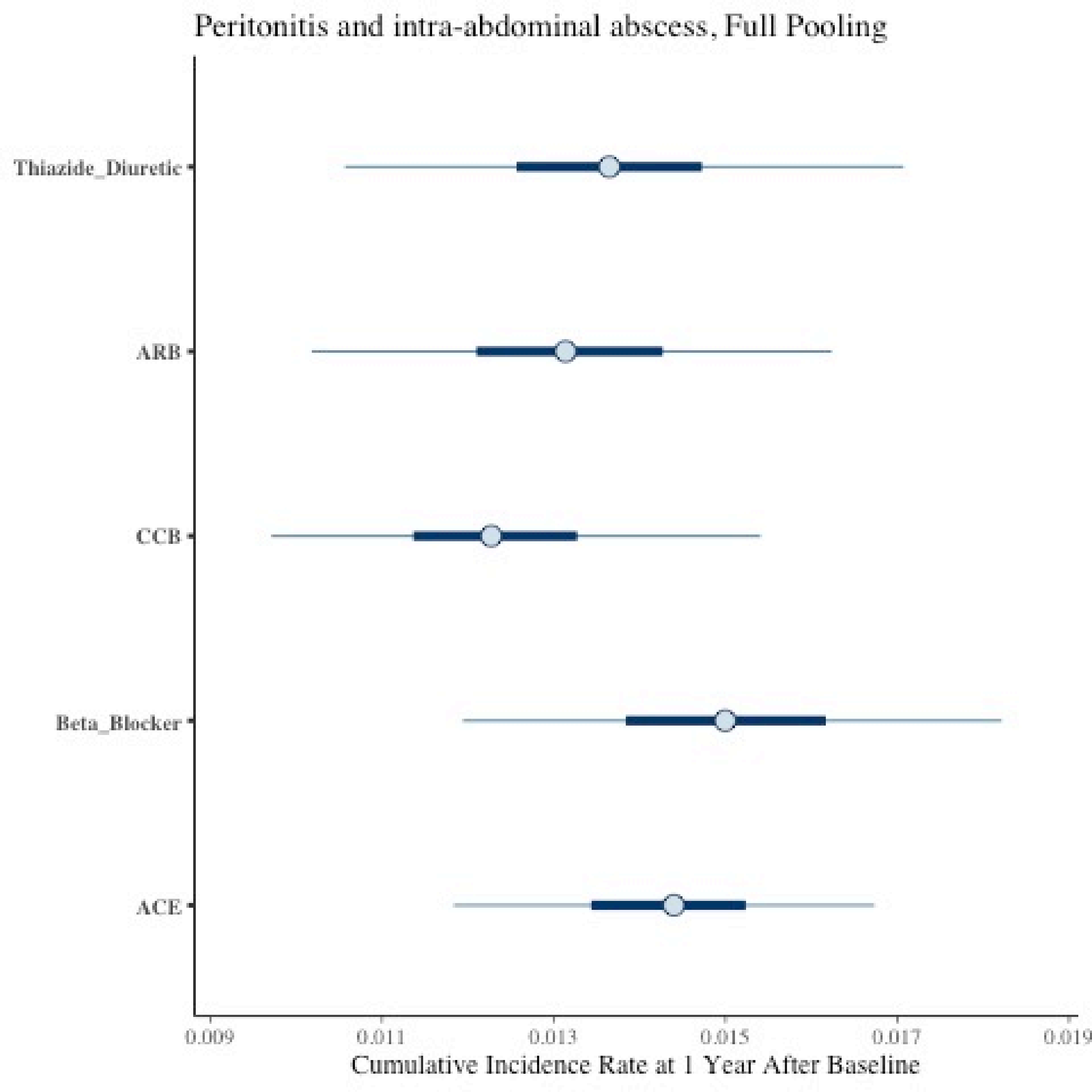

Peritonitis and intra-abdominal abscess, Full Pooling
Thiazide_Diuretic
ARB
CCB
Beta_Blocker
ACE
0.009
0.011
0.013
0.015
0.017
0.019
Cumulative Incidence Rate at 1 Year After Baseline

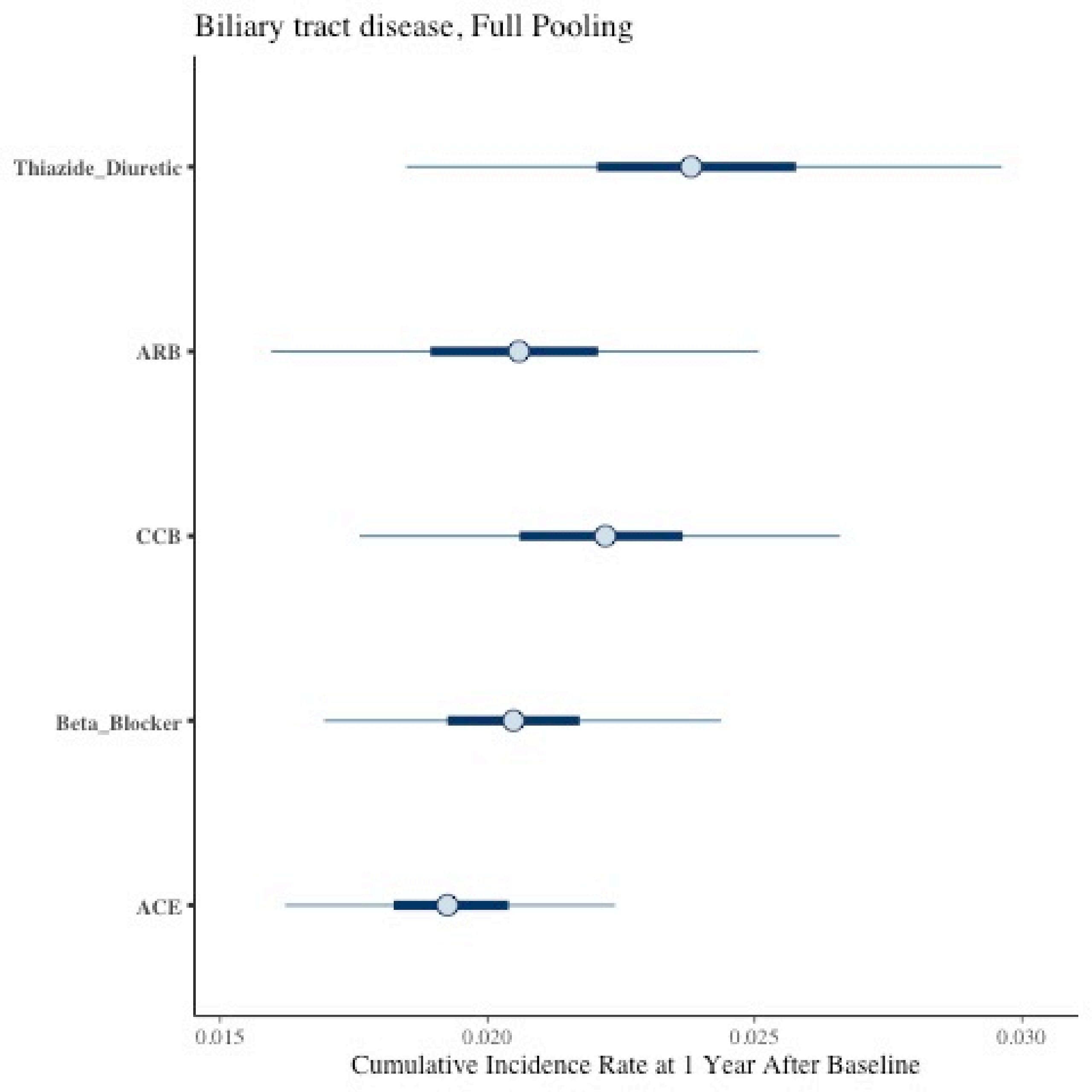

Biliary tract disease, Full Pooling
Thiazide_Diuretic
ARB
CCB
Beta_Blocker
ACE
0.015
0.020
0.025
0.030
Cumulative Incidence Rate at 1 Year After Baseline

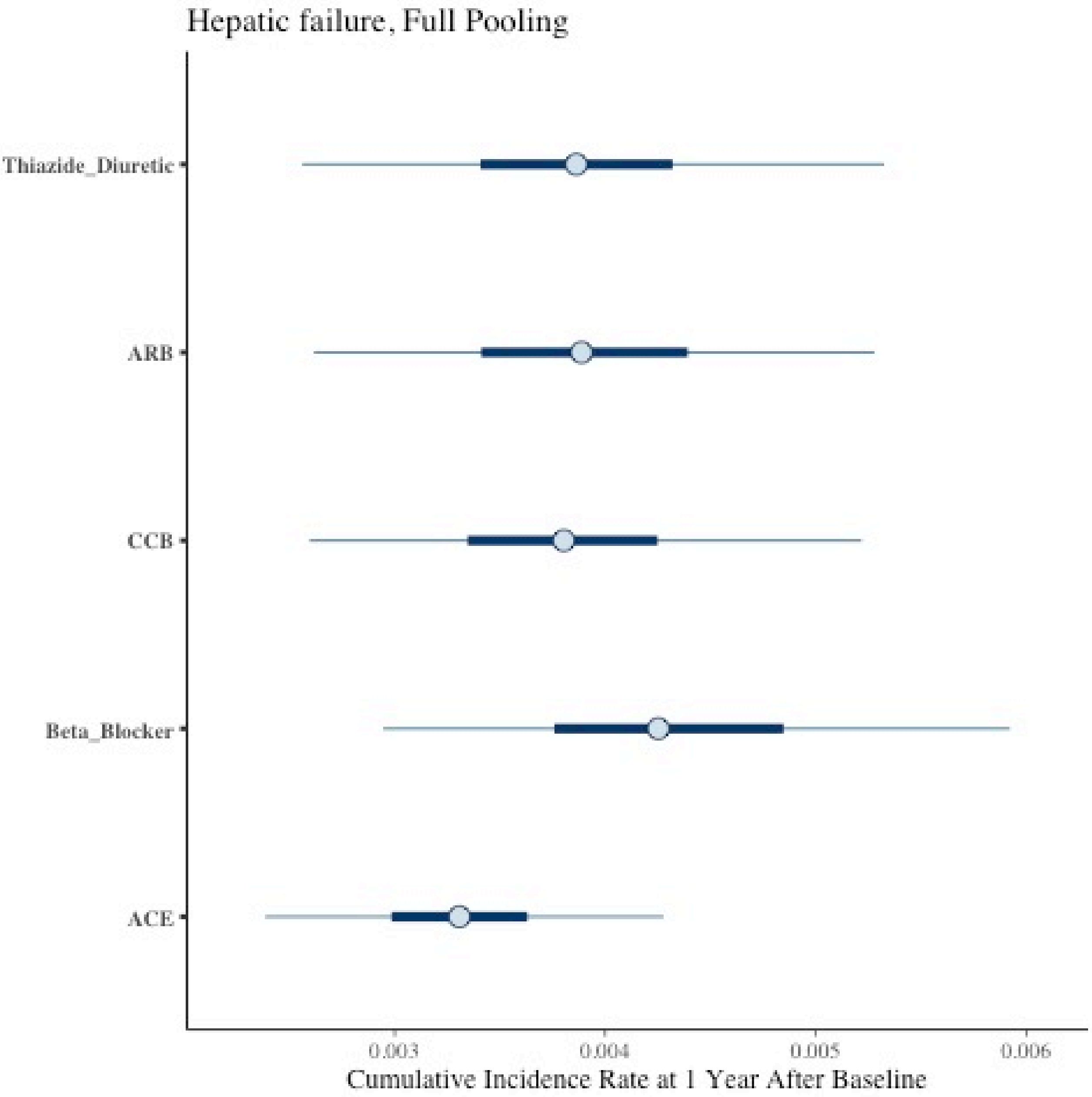

Hepatic failure, Full Pooling
Thiazide_Diuretic
ARB
CCB
Beta_Blocker
ACE
0.003
0.004
0.005
0.006
Cumulative Incidence Rate at 1 Year After Baseline

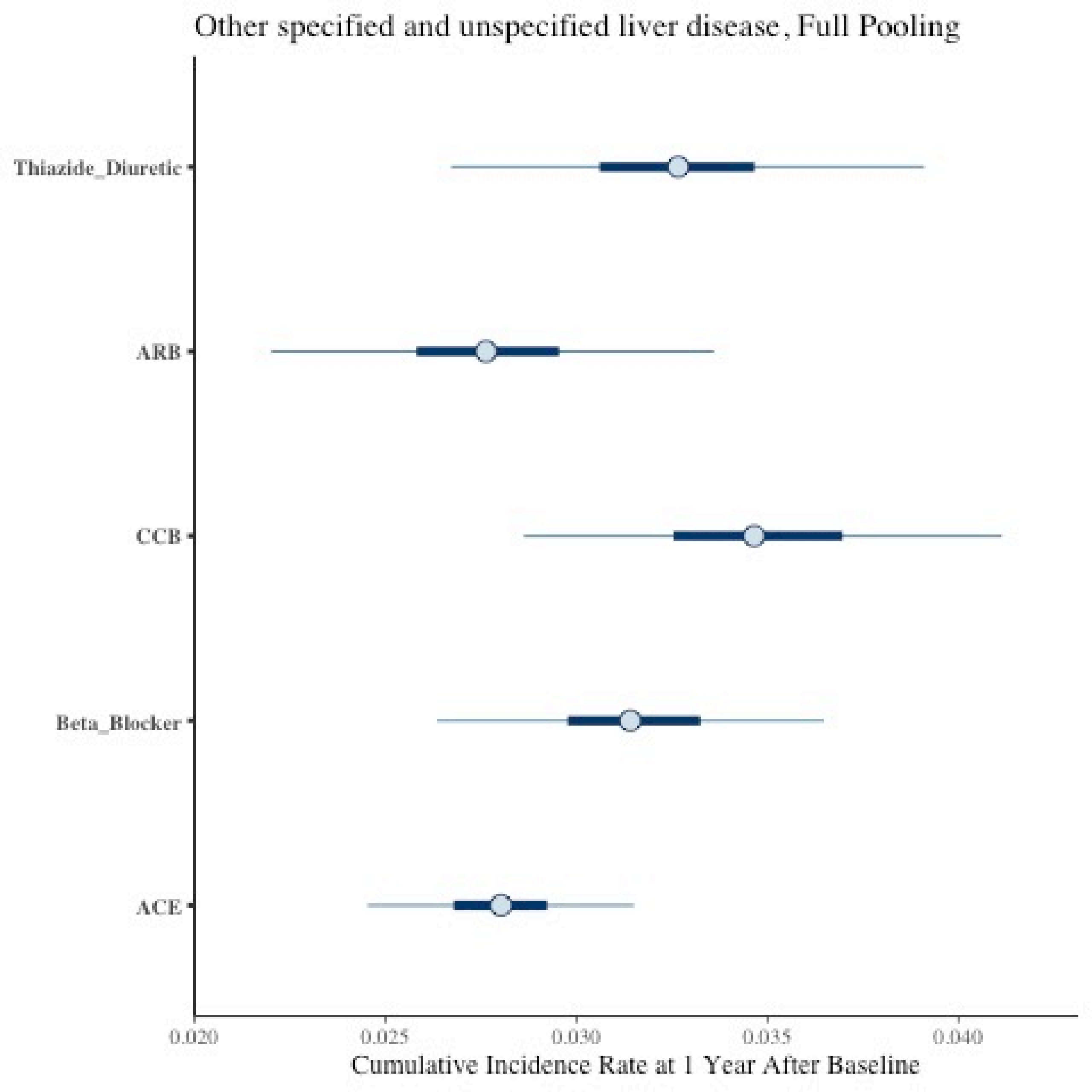

Other specified and unspecified liver disease, Full Pooling
Thiazide_Diuretic
ARB
CCB
Beta_Blocker
ACE
0.020
0.025
0.030
0.035
0.040
Cumulative Incidence Rate at 1 Year After Baseline

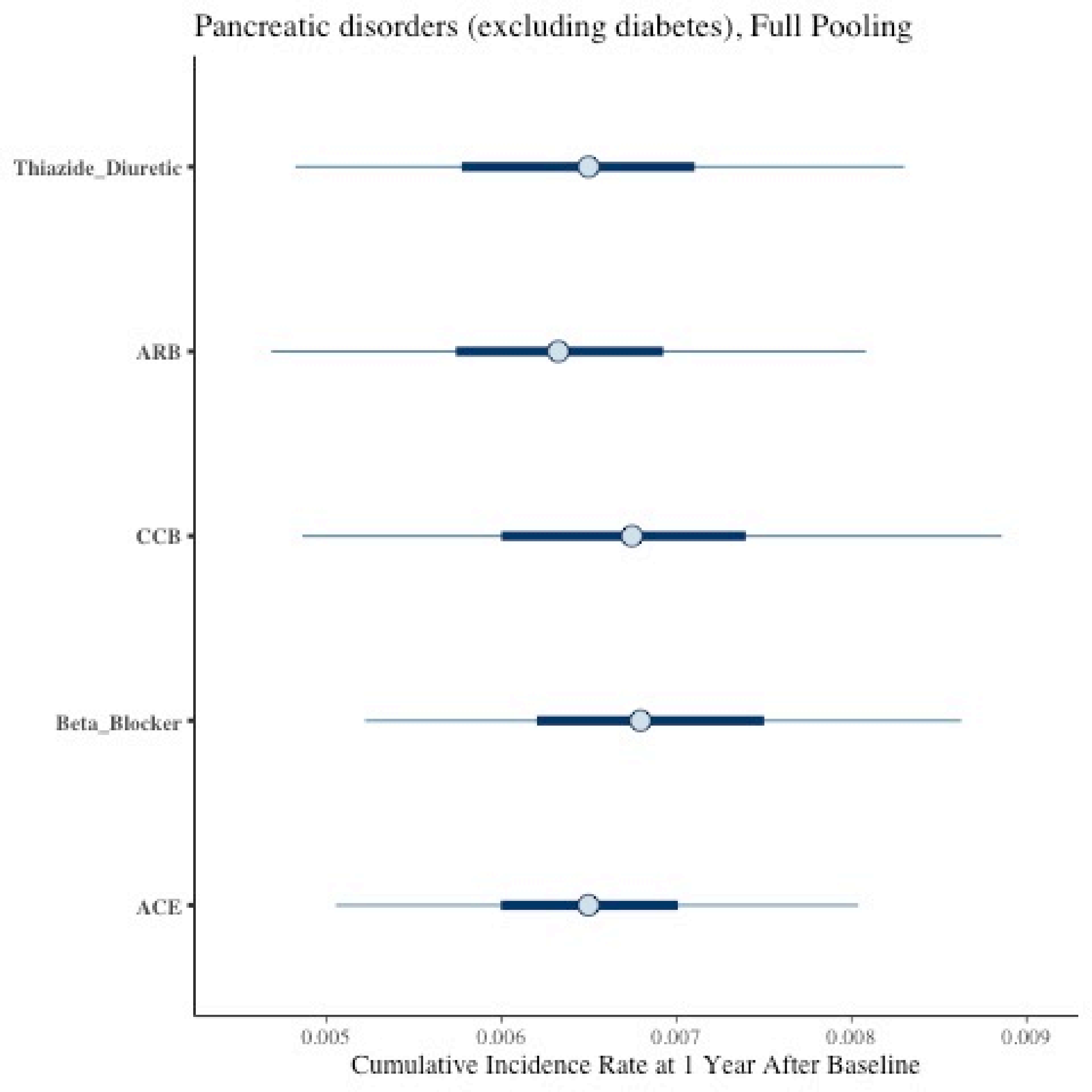

Pancreatic disorders (excluding diabetes), Full Pooling
Thiazide_Diuretic
ARB
CCB
Beta_Blocker
ACE
0.005
0.006
0.007
0.008
0.009
Cumulative Incidence Rate at 1 Year After Baseline

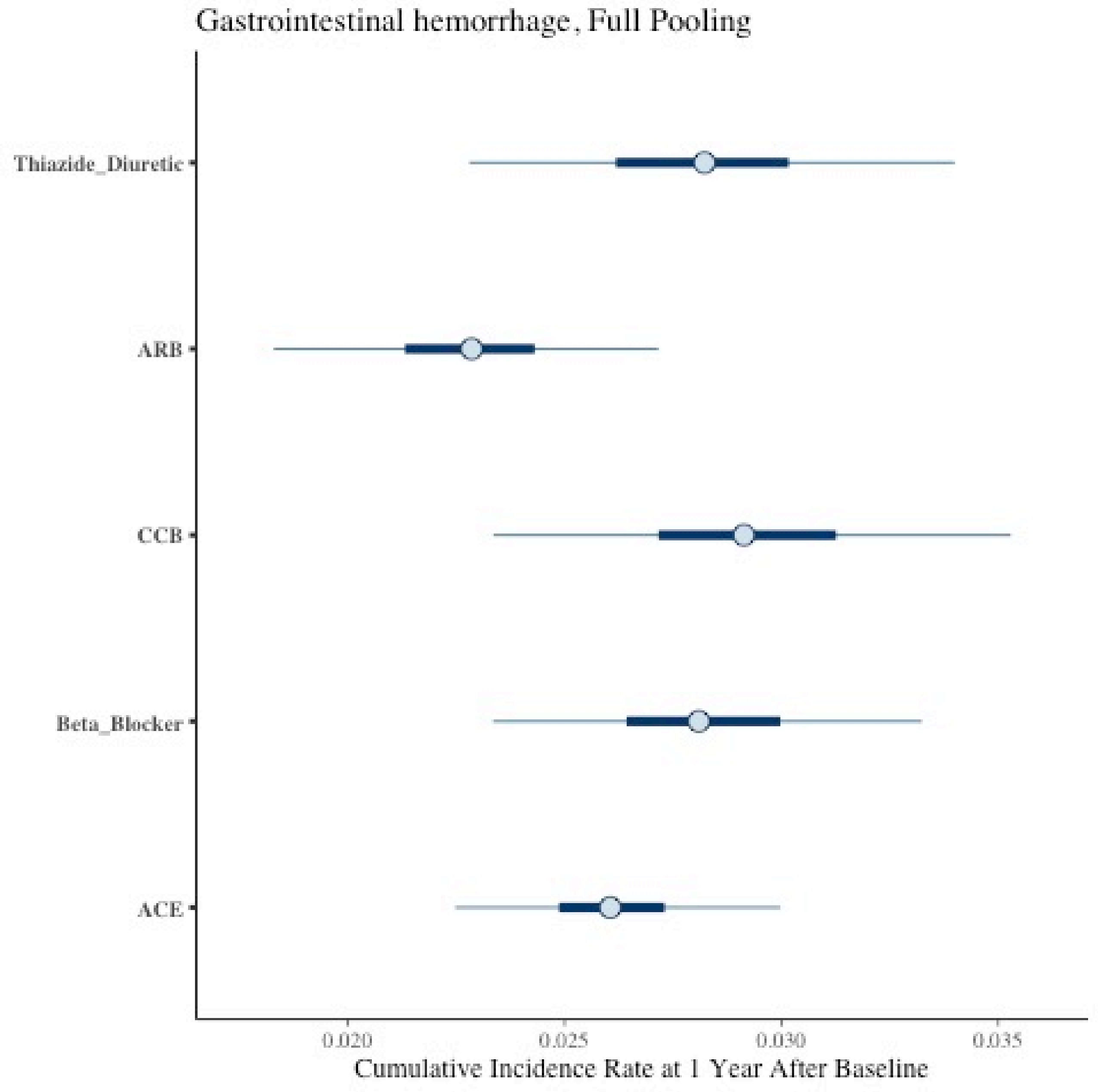

Gastrointestinal hemorrhage, Full Pooling
Thiazide_Diuretic
ARB
CCB
Beta_Blocker
ACE
0.020
0.025
0.030
0.035
Cumulative Incidence Rate at 1 Year After Baseline

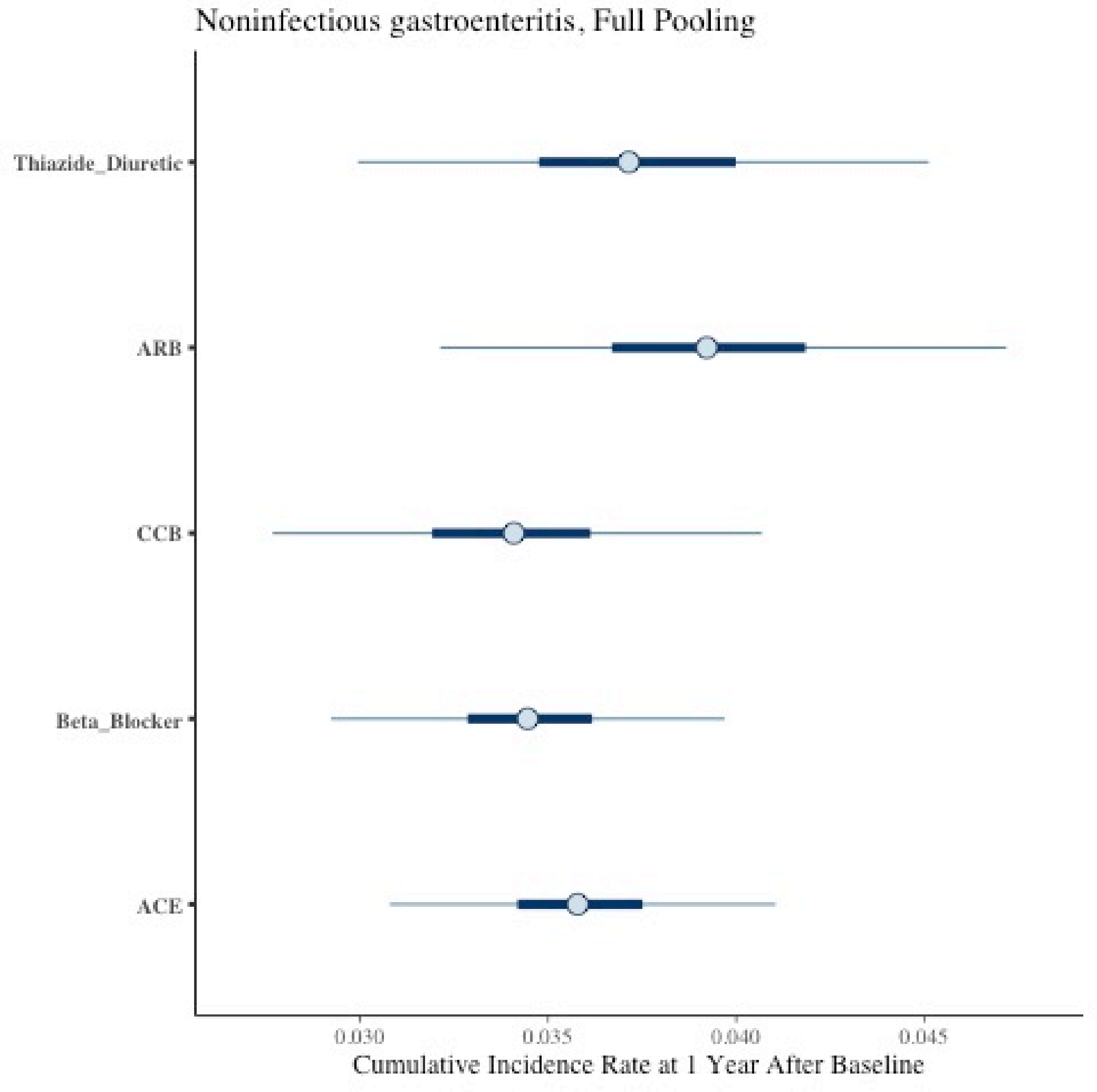

Noninfectious gastroenteritis, Full Pooling
Thiazide_Diuretic
ARB
CCB
Beta_Blocker
ACE
0.030
0.035
0.040
0.045
Cumulative Incidence Rate at 1 Year After Baseline

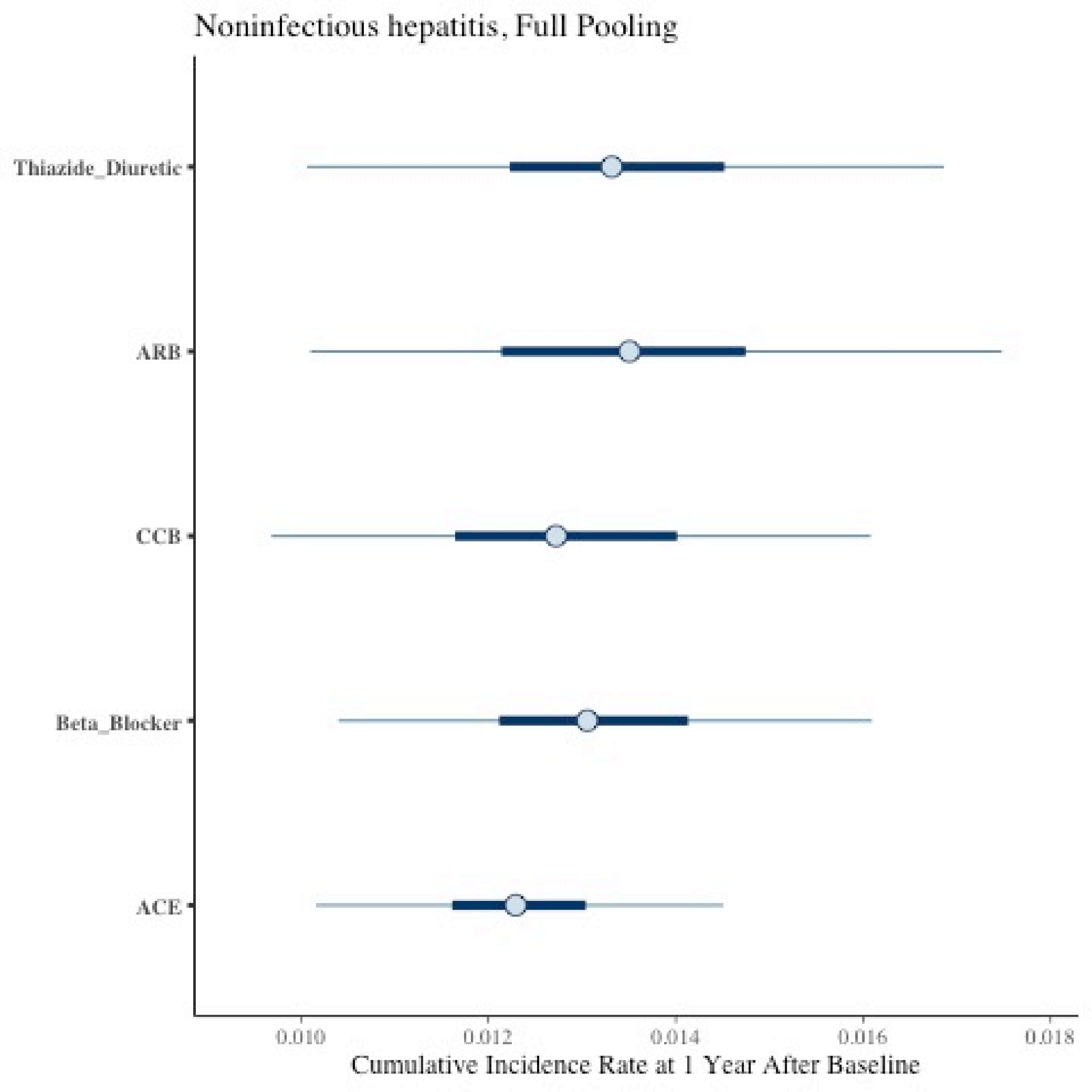

Noninfectious hepatitis, Full Pooling
Thiazide_Diuretic
ARB
CCB
Beta_Blocker
ACE
0.010
0.012
0.014
0.016
0.018
Cumulative Incidence Rate at 1 Year After Baseline

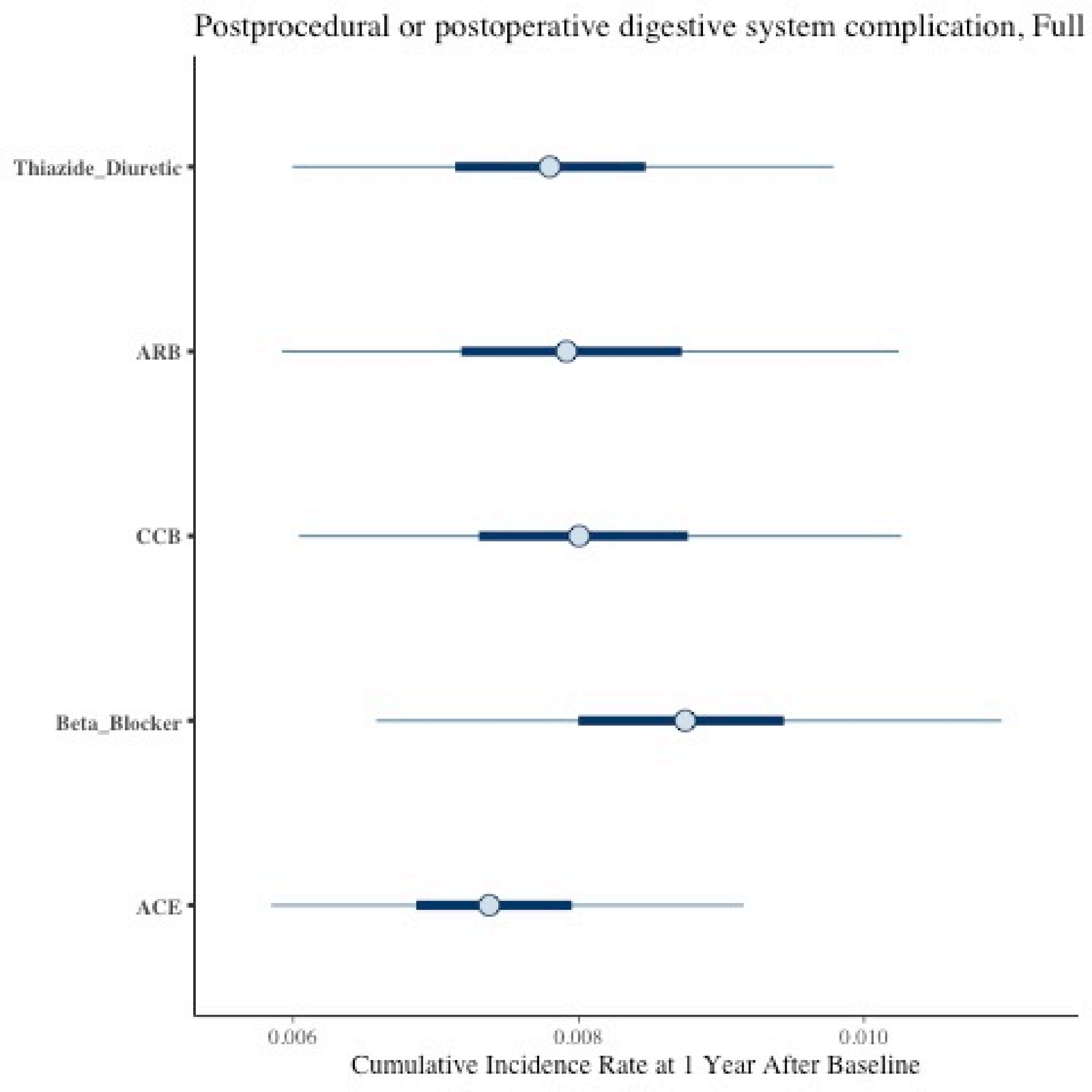

Postprocedural or postoperative digestive system complication, Full
Thiazide_Diuretic
ARB
CCB
Beta_Blocker
ACE
0.006
0.008
0.010
Cumulative Incidence Rate at 1 Year After Baseline

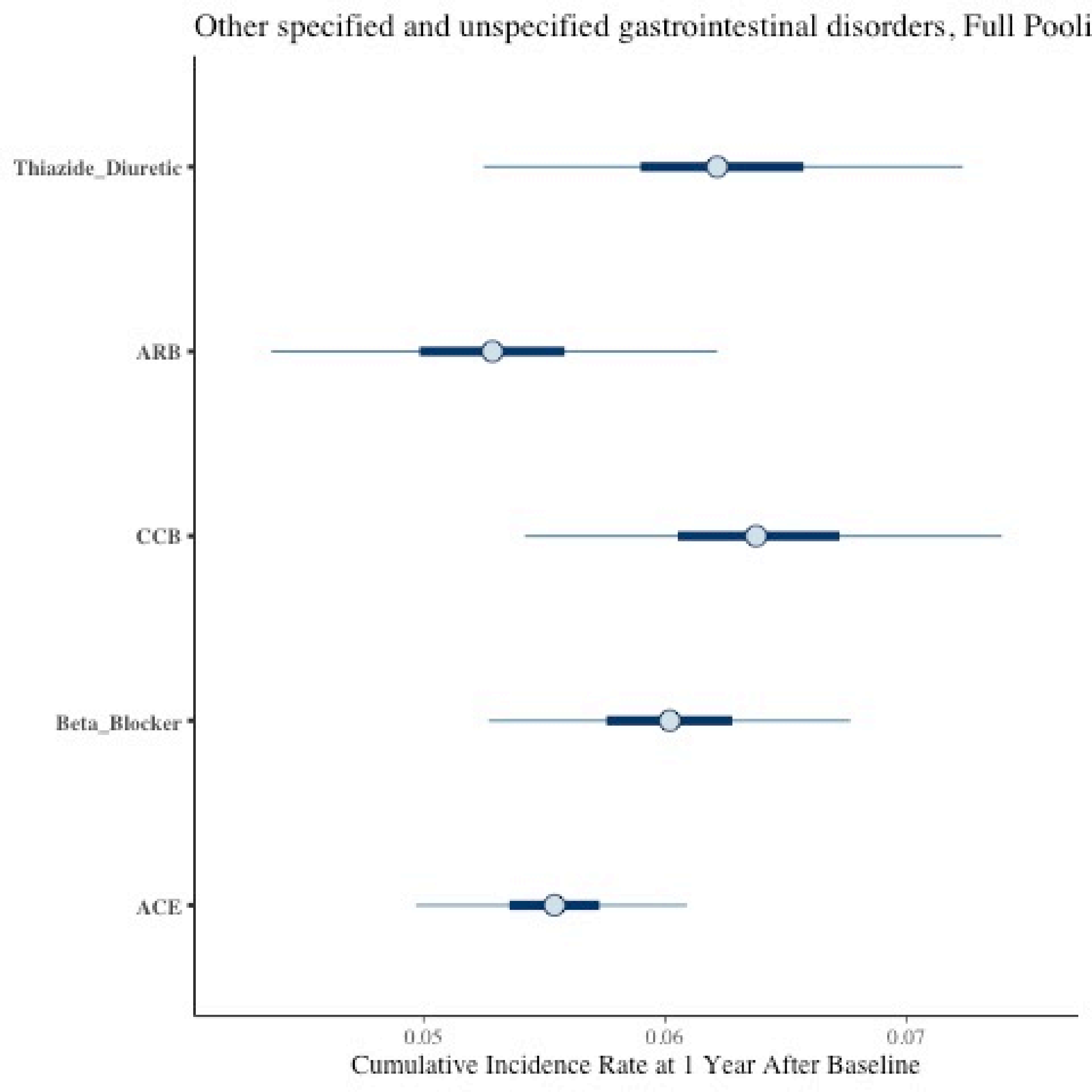

Other specified and unspecified gastrointestinal disorders, Full Pooli
Thiazide_Diuretic
ARB
CCB
Beta_Blocker
ACE
0.05
0.06
0.07
Cumulative Incidence Rate at 1 Year After Baseline

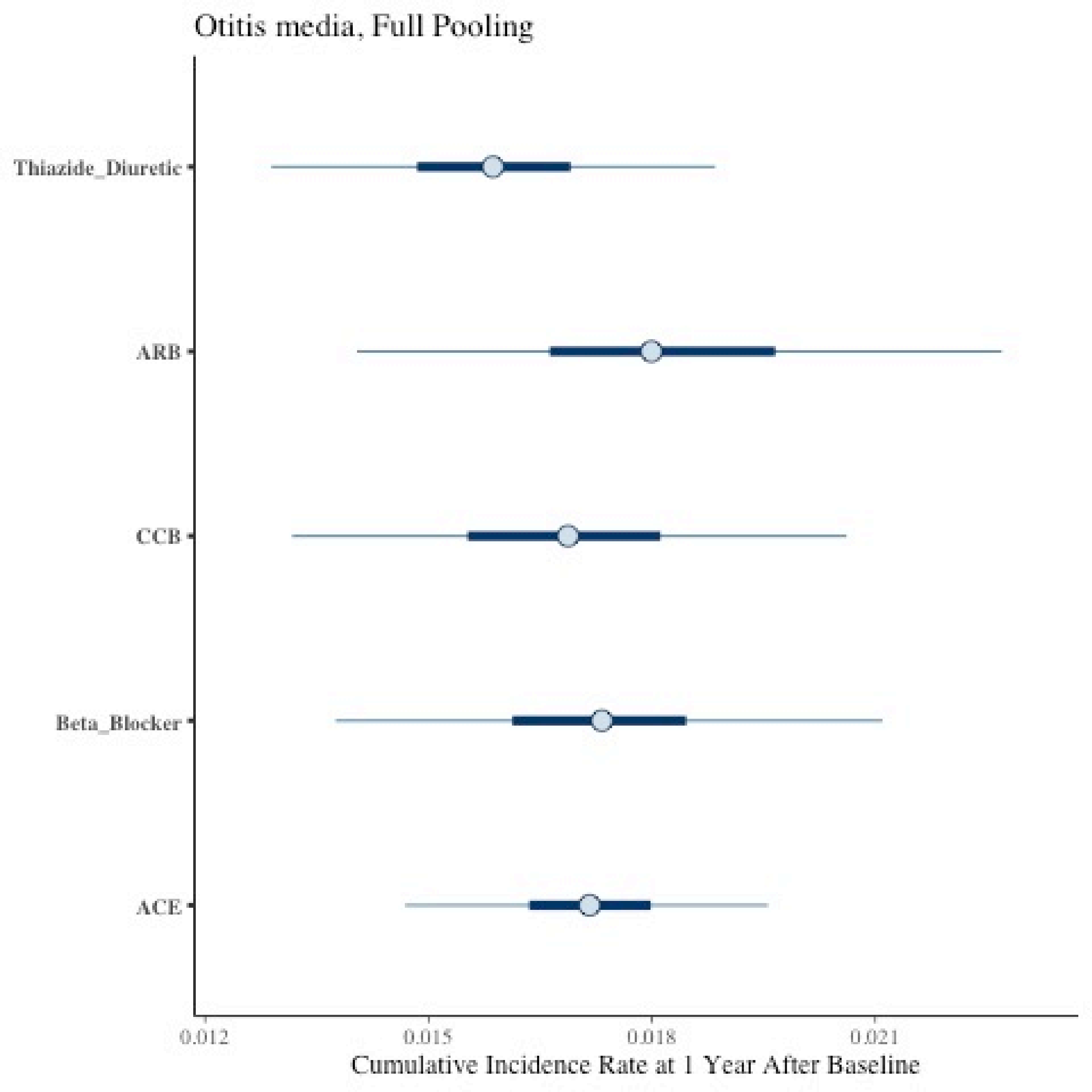

Otitis media, Full Pooling
Thiazide_Diuretic
ARB
CCB
Beta_Blocker
ACE
0.012
0.015
0.018
0.021
Cumulative Incidence Rate at 1 Year After Baseline

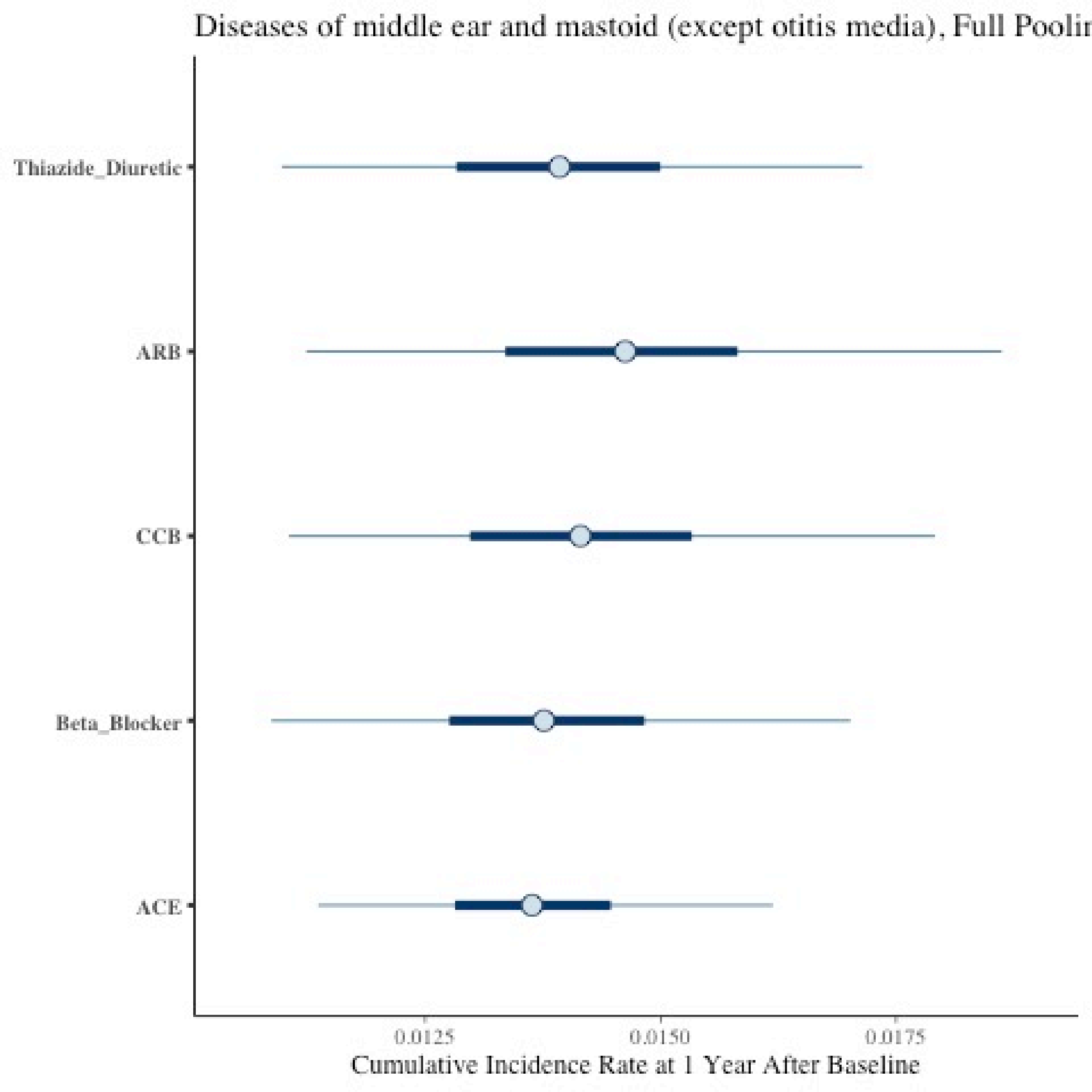

Diseases of middle ear and mastoid (except otitis media), Full Poolin
Thiazide_Diuretic
ARB
CCB
Beta_Blocker
ACE
0.0125
0.0150
0.0175
Cumulative Incidence Rate at 1 Year After Baseline

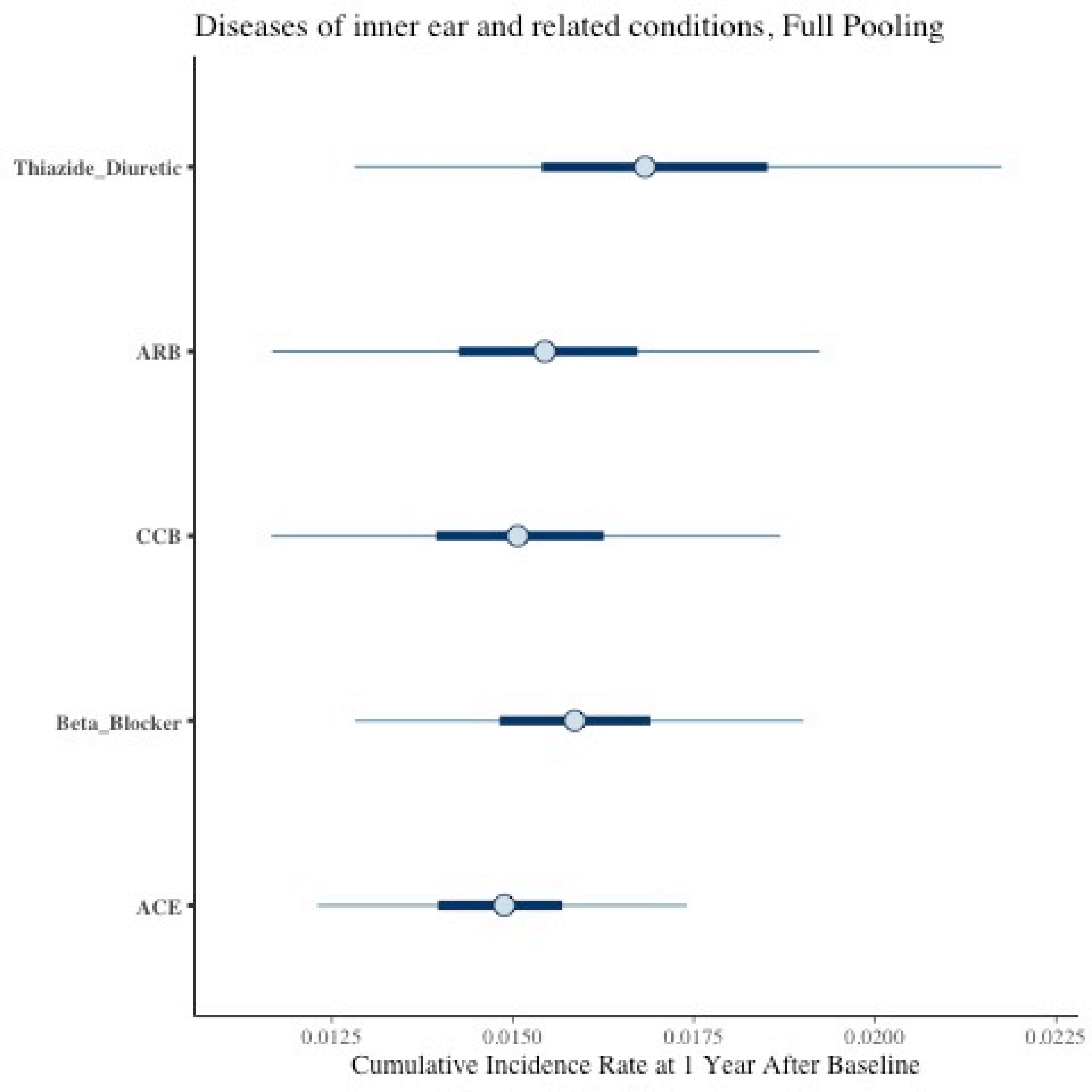
Diseases of inner ear and related conditions, Full Pooling
Thiazide_Diuretic
ARB
CCB
Beta_Blocker
ACE
0.0125
0.0150
0.0175
0.0200
0.0225
Cumulative Incidence Rate at 1 Year After Baseline

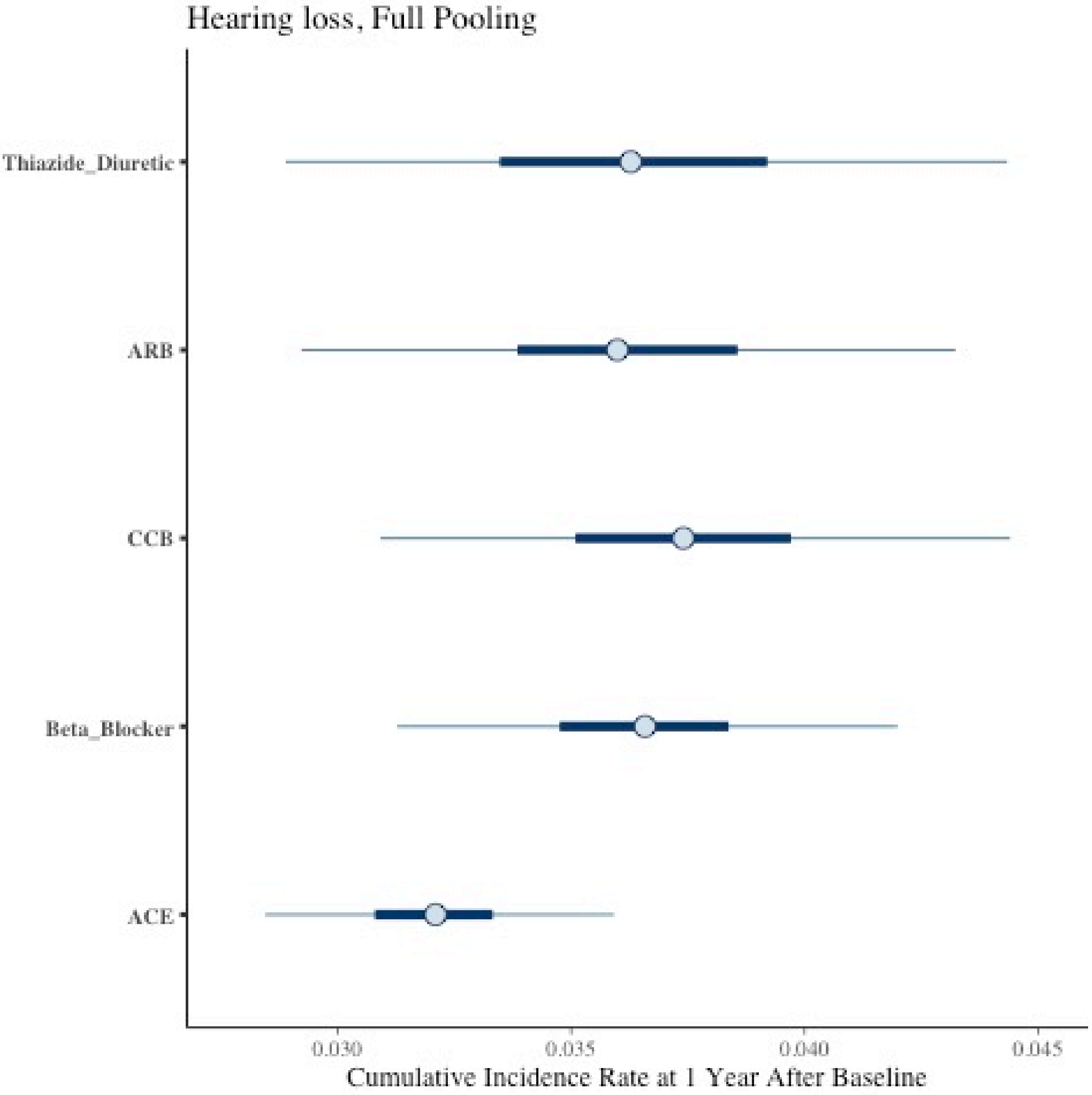

Hearing loss, Full Pooling
Thiazide_Diuretic
ARB
CCB
Beta_Blocker
ACE
0.030
0.035
0.040
0.045
Cumulative Incidence Rate at 1 Year After Baseline

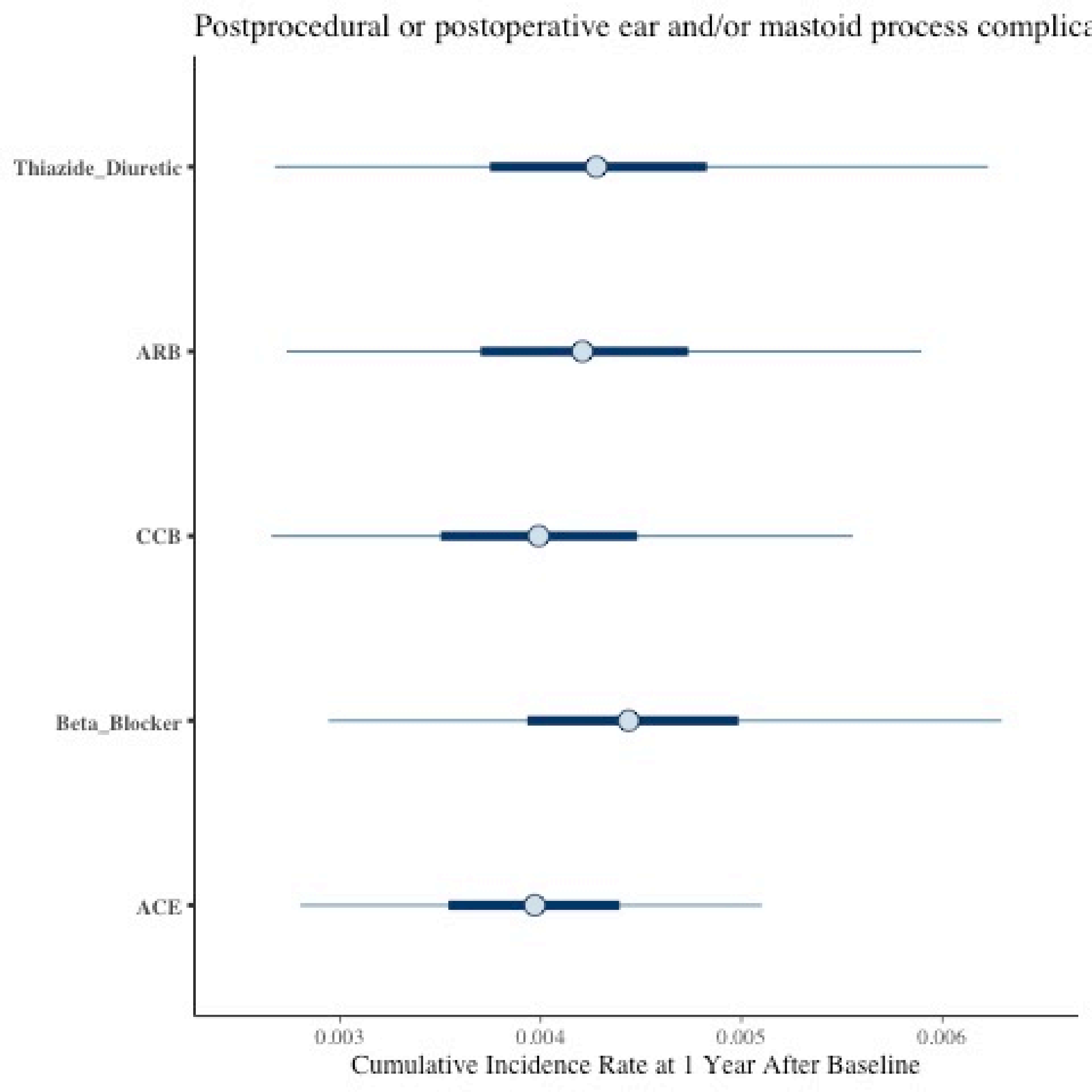

Postprocedural or postoperative ear and/or mastoid process complica
Thiazide_Diuretic
ARB
CCB
Beta_Blocker
ACE
0.003
0.004
0.005
0.006
Cumulative Incidence Rate at 1 Year After Baseline

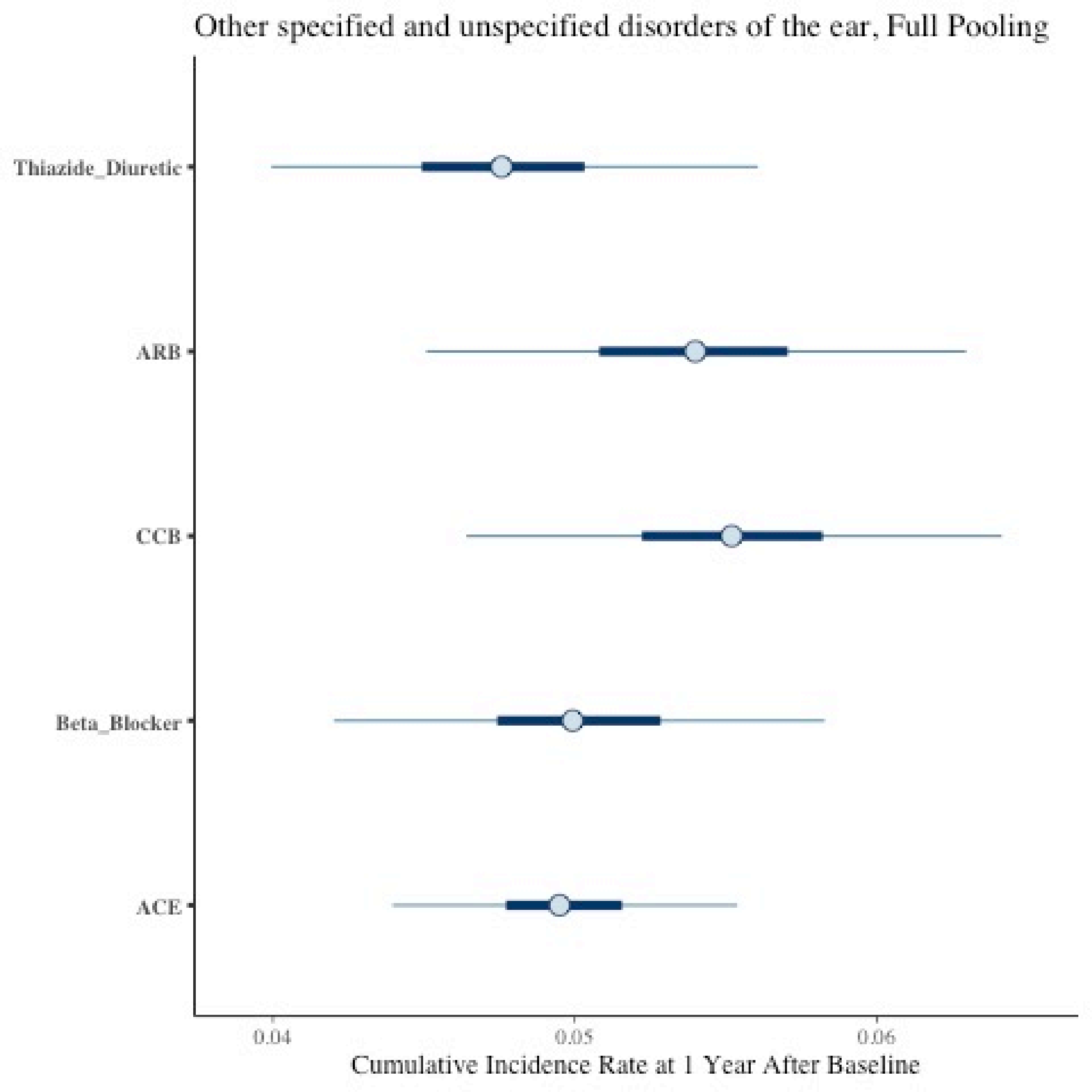

Other specified and unspecified disorders of the ear, Full Pooling
Thiazide_Diuretic
ARB
CCB
Beta_Blocker
ACE
0.04
0.05
0.06
Cumulative Incidence Rate at 1 Year After Baseline

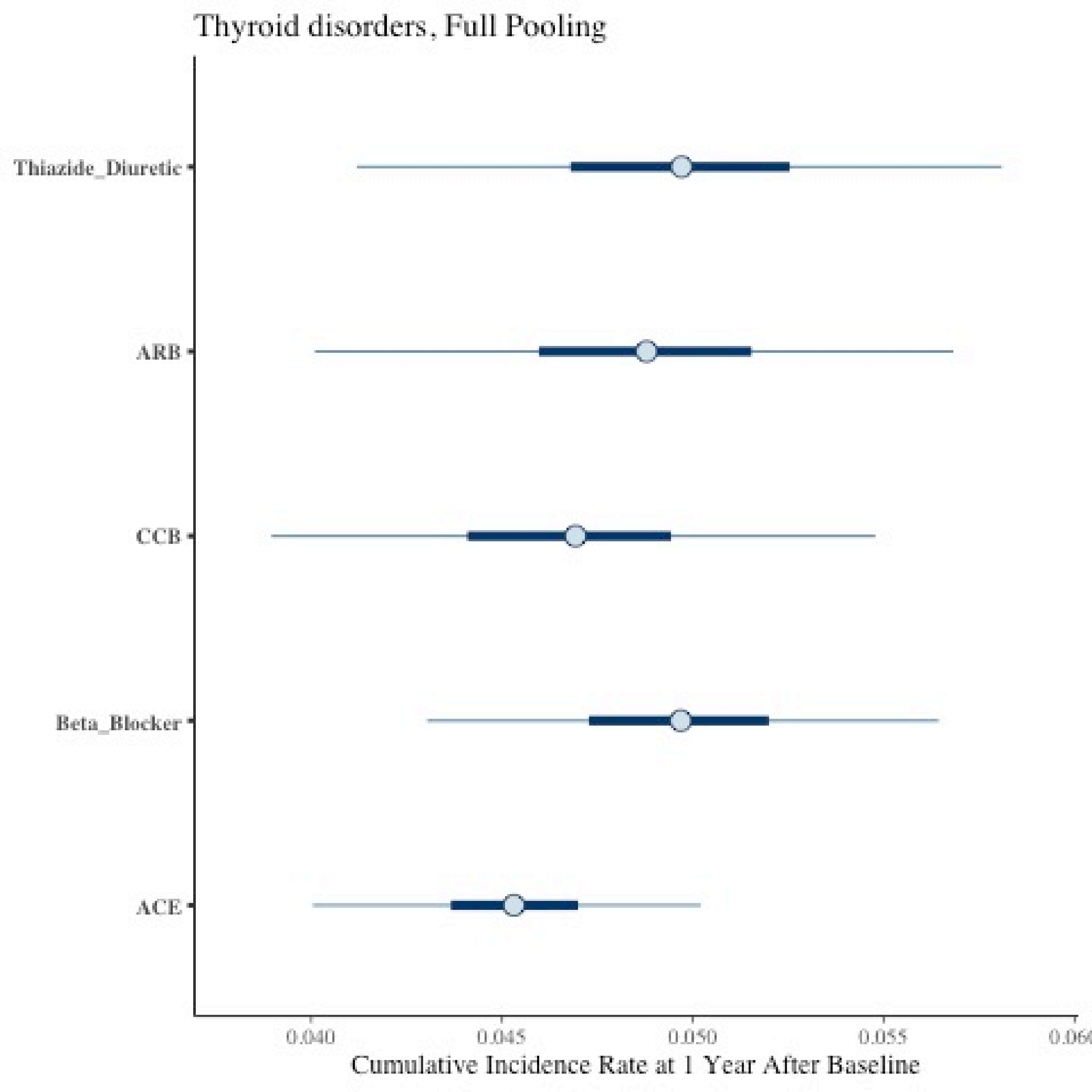

Thyroid disorders, Full Pooling
Thiazide_Diuretic
ARB
CCB
Beta_Blocker
ACE
0.040
0.045
0.050
0.055
0.060
Cumulative Incidence Rate at 1 Year After Baseline

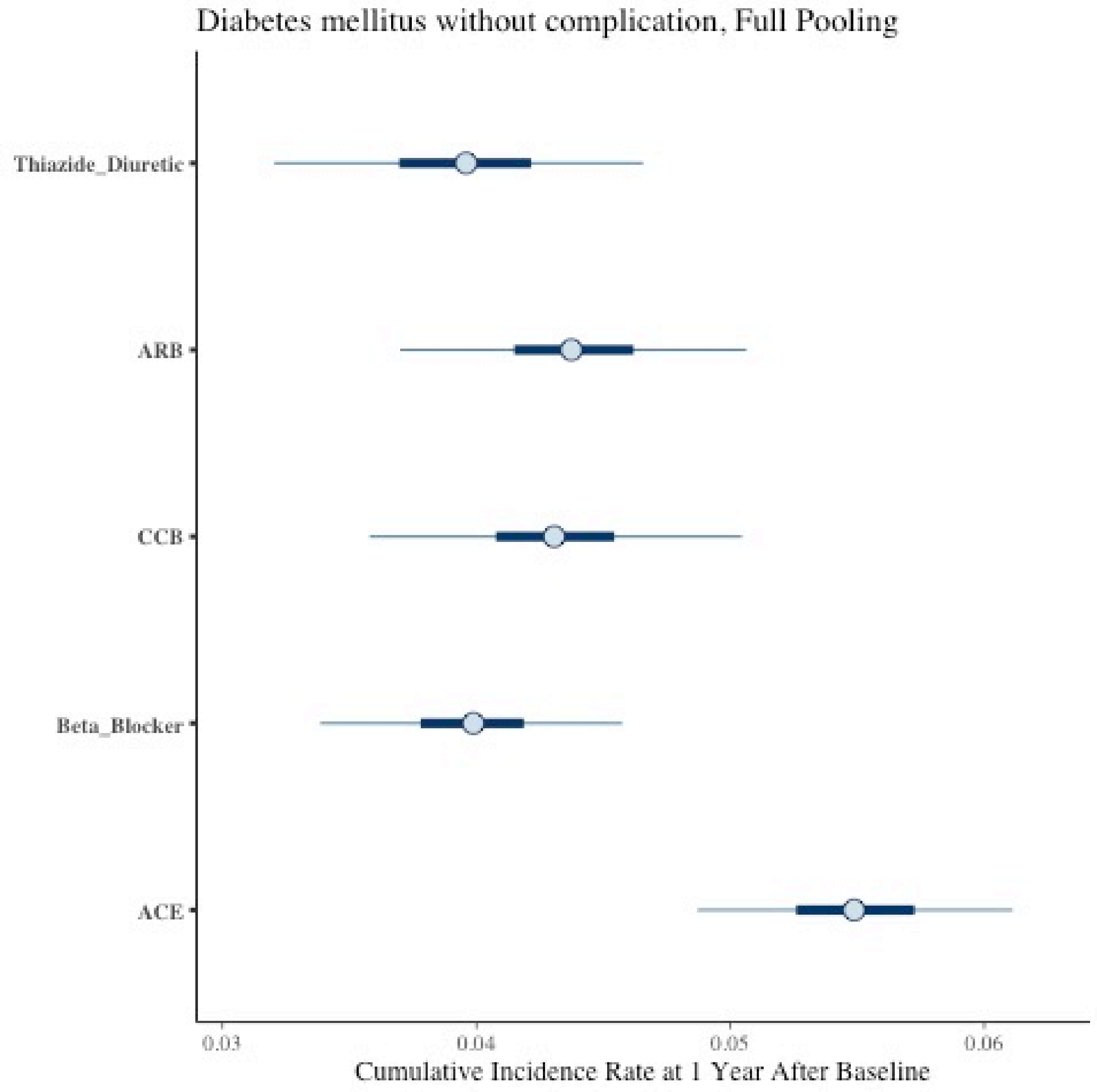

Diabetes mellitus without complication, Full Pooling
Thiazide_Diuretic
ARB
CCB
Beta_Blocker
ACE
0.03
0.04
0.05
0.06
Cumulative Incidence Rate at 1 Year After Baseline

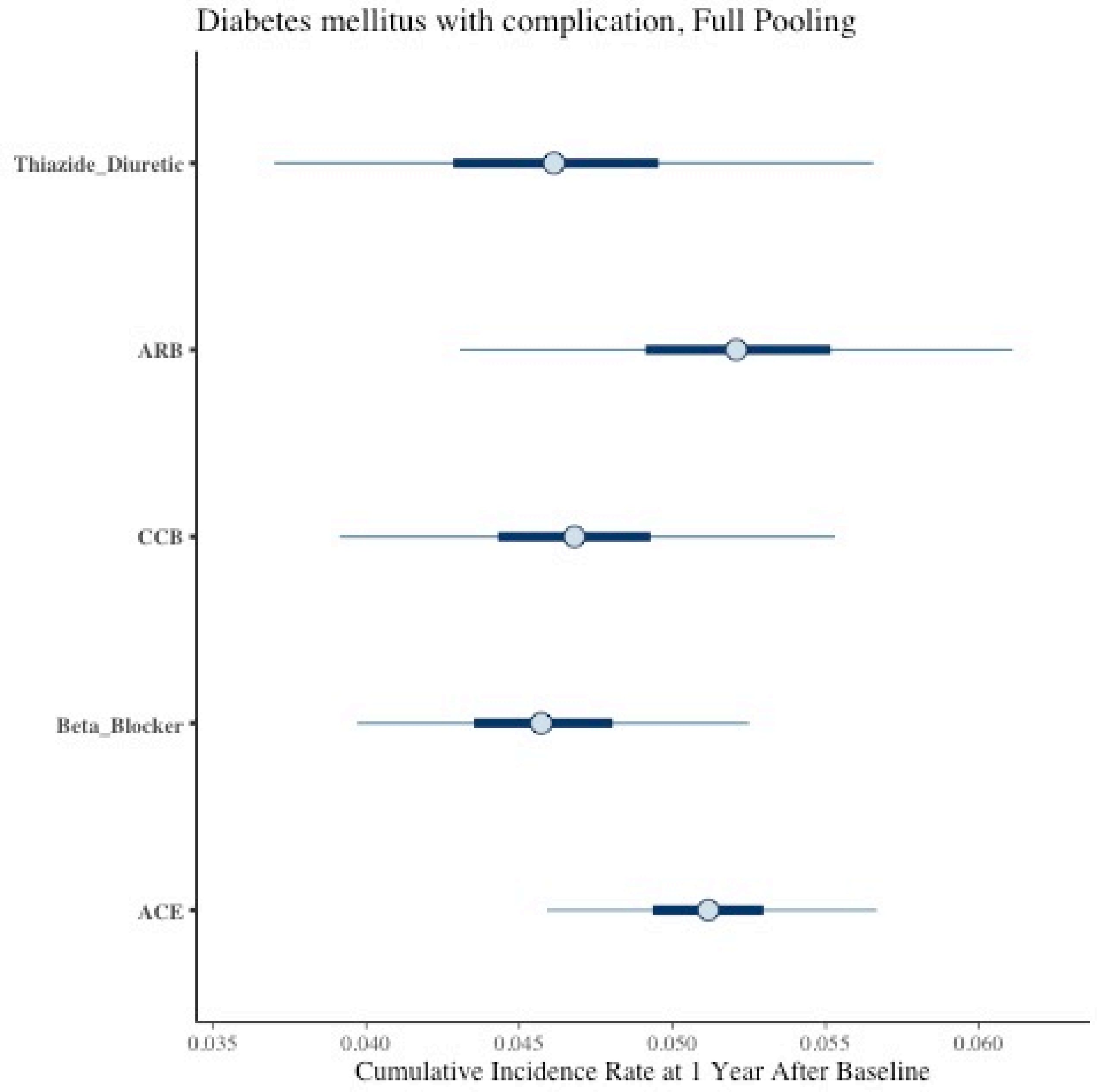
Diabetes mellitus with complication, Full Pooling
Thiazide_Diuretic
ARB
CCB
Beta_Blocker
ACE
0.035
0.040
0.045
0.050
0.055
0.060
Cumulative Incidence Rate at 1 Year After Baseline

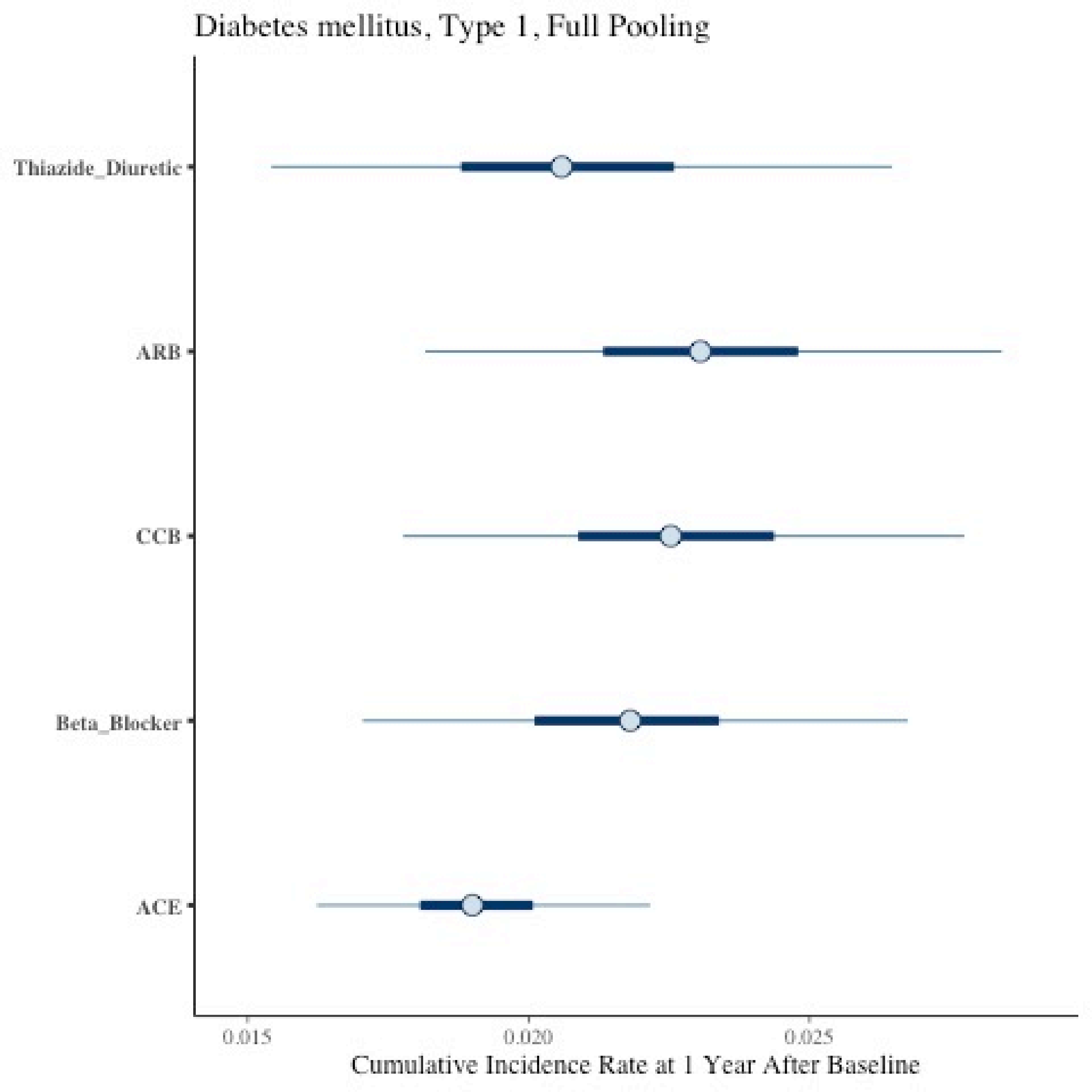
Diabetes mellitus, Type 1, Full Pooling
Thiazide_Diuretic
ARB
CCB
Beta_Blocker
ACE
0.015
0.020
0.025
Cumulative Incidence Rate at 1 Year After Baseline

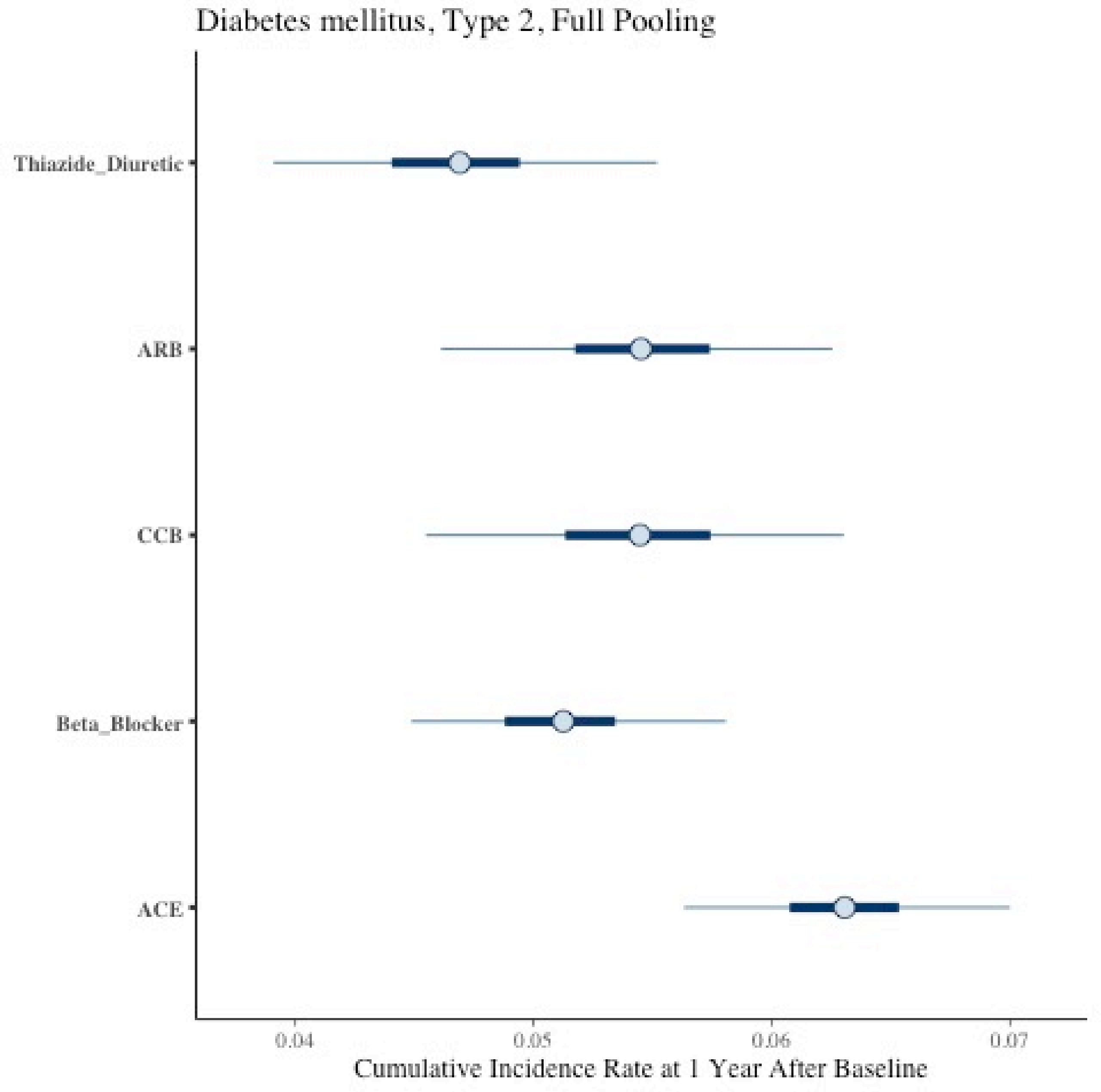

Diabetes mellitus, Type 2, Full Pooling
Thiazide_Diuretic
ARB
CCB
Beta_Blocker
ACE
0.04
0.05
0.06
0.07
Cumulative Incidence Rate at 1 Year After Baseline

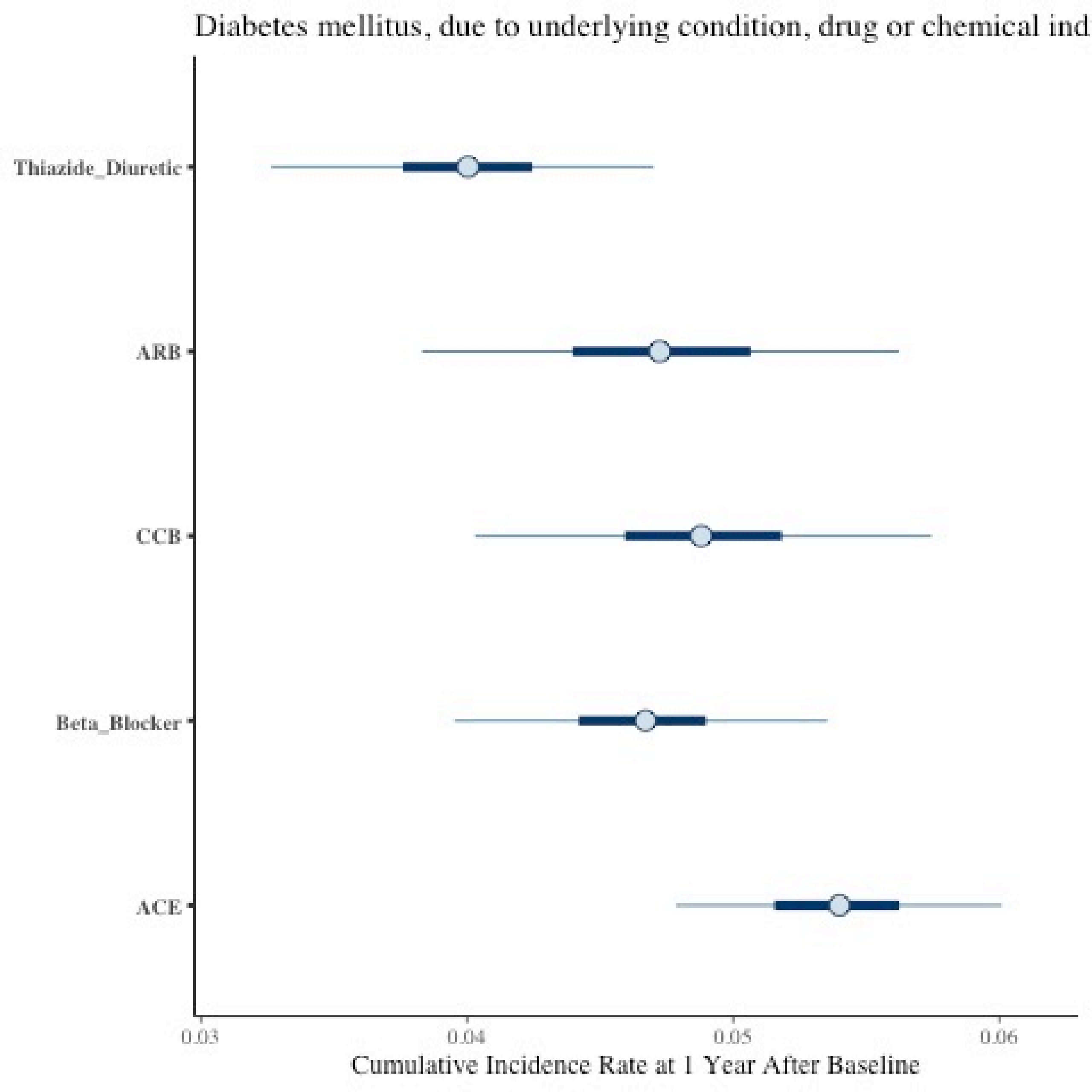

Diabetes mellitus, due to underlying condition, drug or chemical ind
Thiazide_Diuretic
ARB
CCB
Beta_Blocker
ACE
0.03
0.04
0.05
0.06
Cumulative Incidence Rate at 1 Year After Baseline

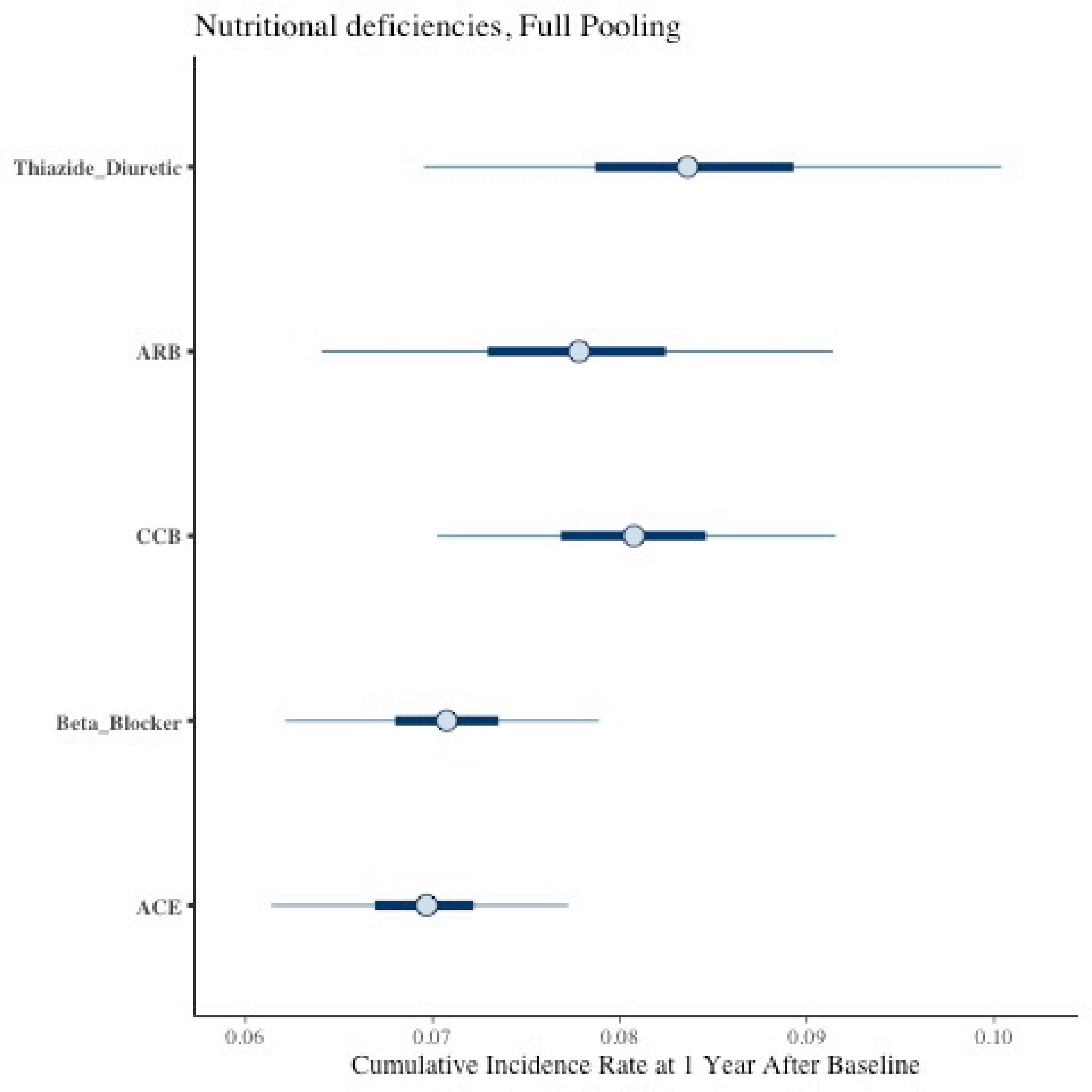
Nutritional deficiencies, Full Pooling
Thiazide_Diuretic
ARB
CCB
Beta_Blocker
ACE
0.06
0.07
0.08
0.09
0.10
Cumulative Incidence Rate at 1 Year After Baseline

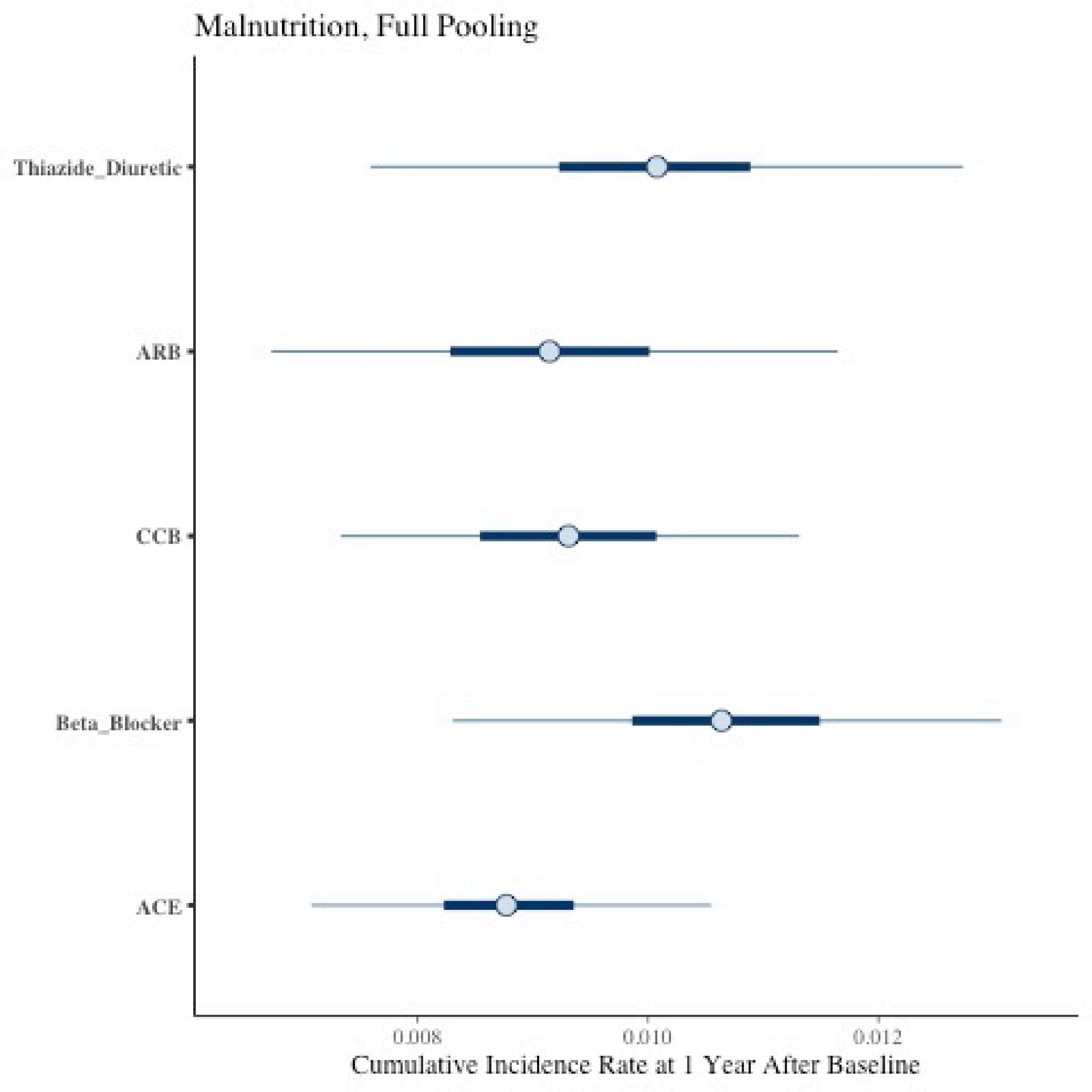

Malnutrition, Full Pooling
Thiazide_Diuretic
ARB
CCB
Beta_Blocker
ACE
0.008
0.010
0.012
Cumulative Incidence Rate at 1 Year After Baseline

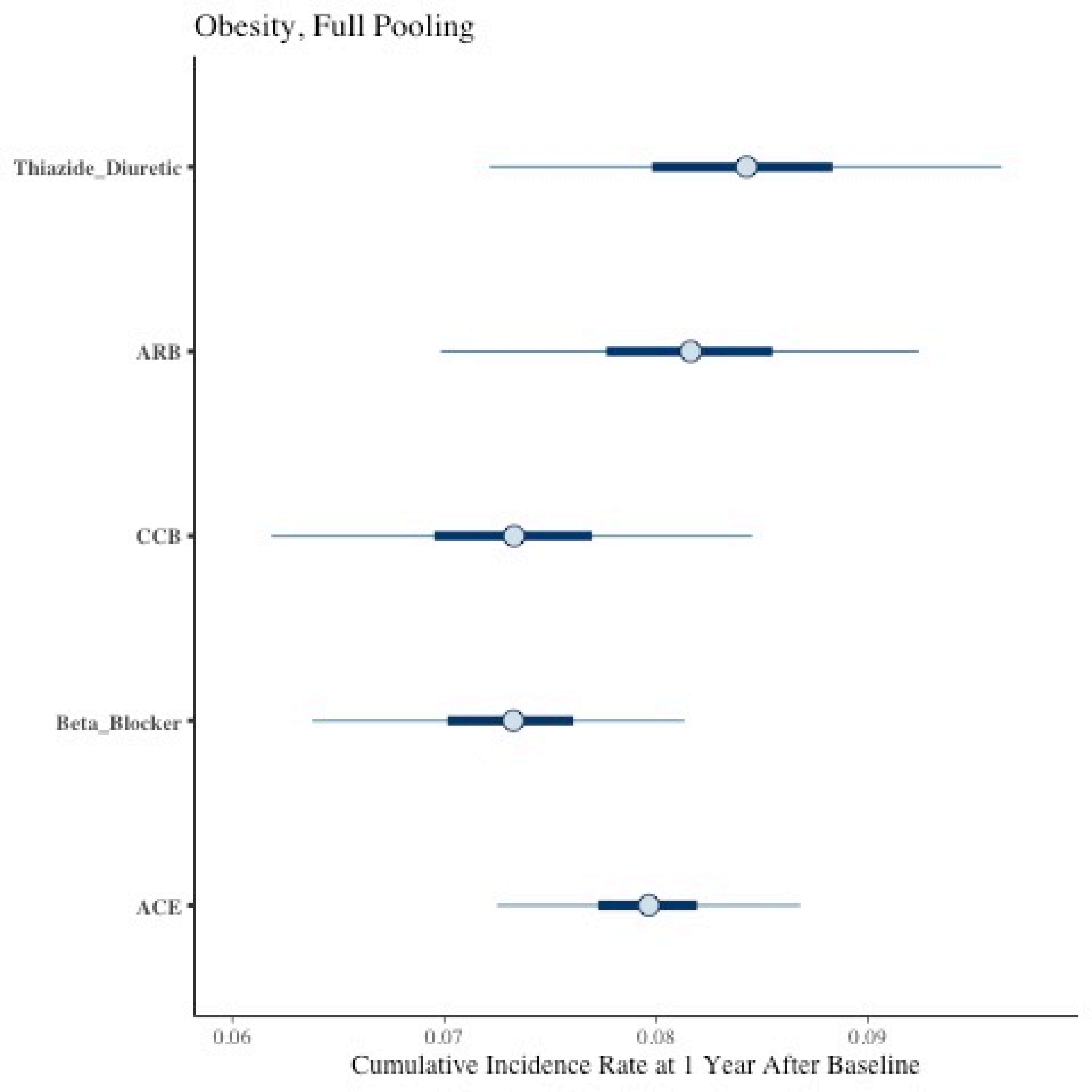

Obesity, Full Pooling
Thiazide_Diuretic
ARB
CCB
Beta_Blocker
ACE
0.06
0.07
0.08
0.09
Cumulative Incidence Rate at 1 Year After Baseline

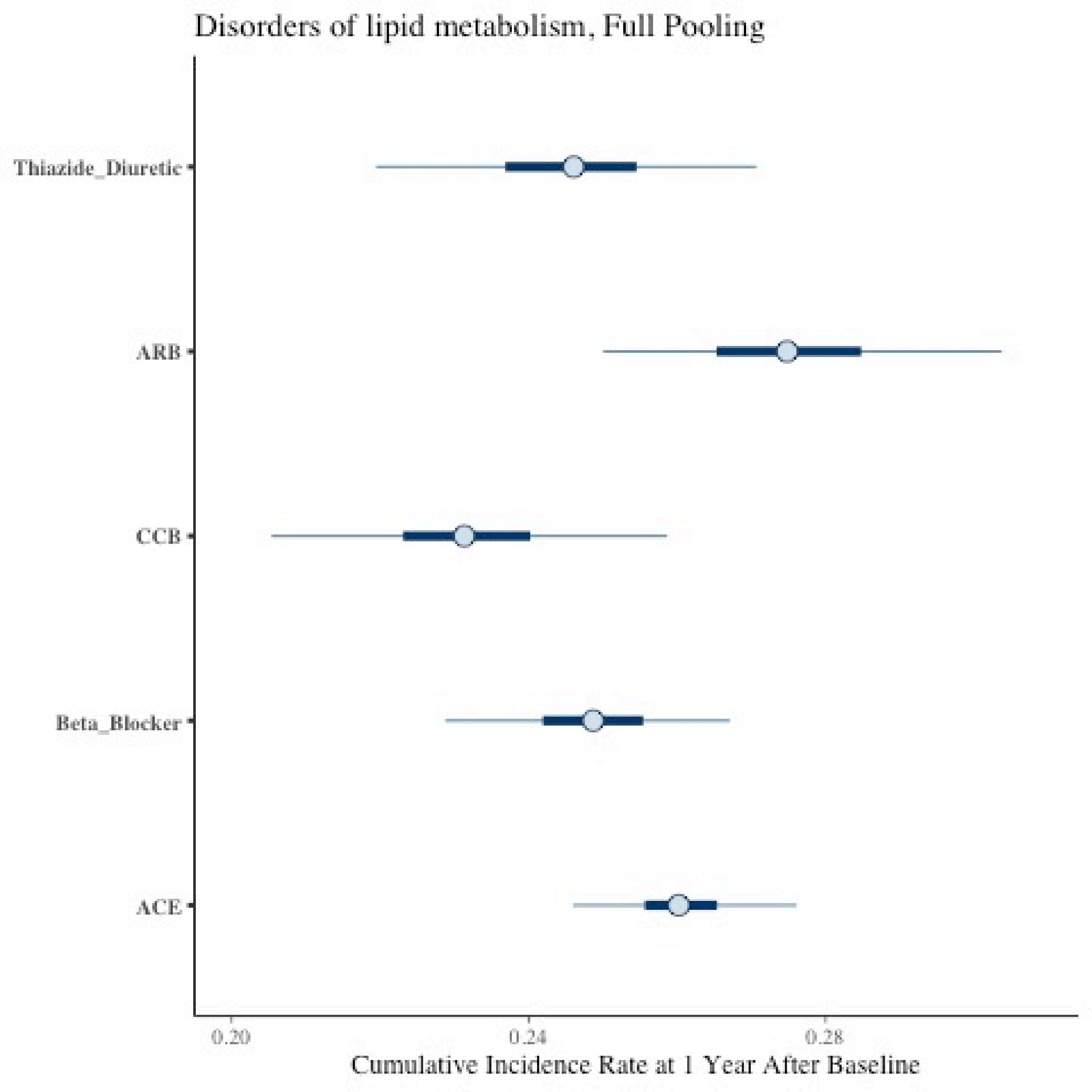

Disorders of lipid metabolism, Full Pooling
Thiazide_Diuretic
ARB
CCB
Beta_Blocker
ACE
0.20
0.24
0.28
Cumulative Incidence Rate at 1 Year After Baseline

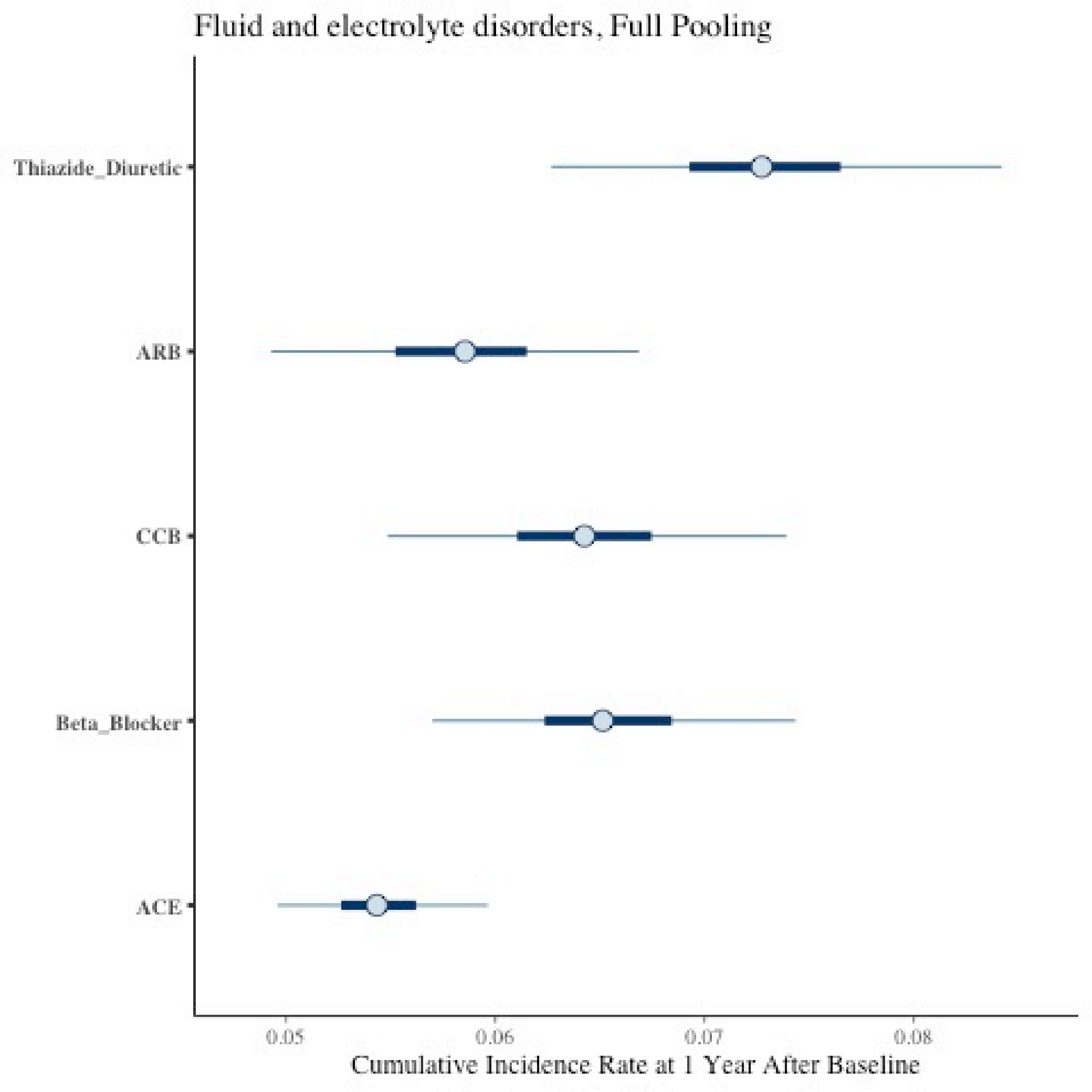

Fluid and electrolyte disorders, Full Pooling
Thiazide_Diuretic
ARB
CCB
Beta_Blocker
ACE
0.05
0.06
0.07
0.08
Cumulative Incidence Rate at 1 Year After Baseline

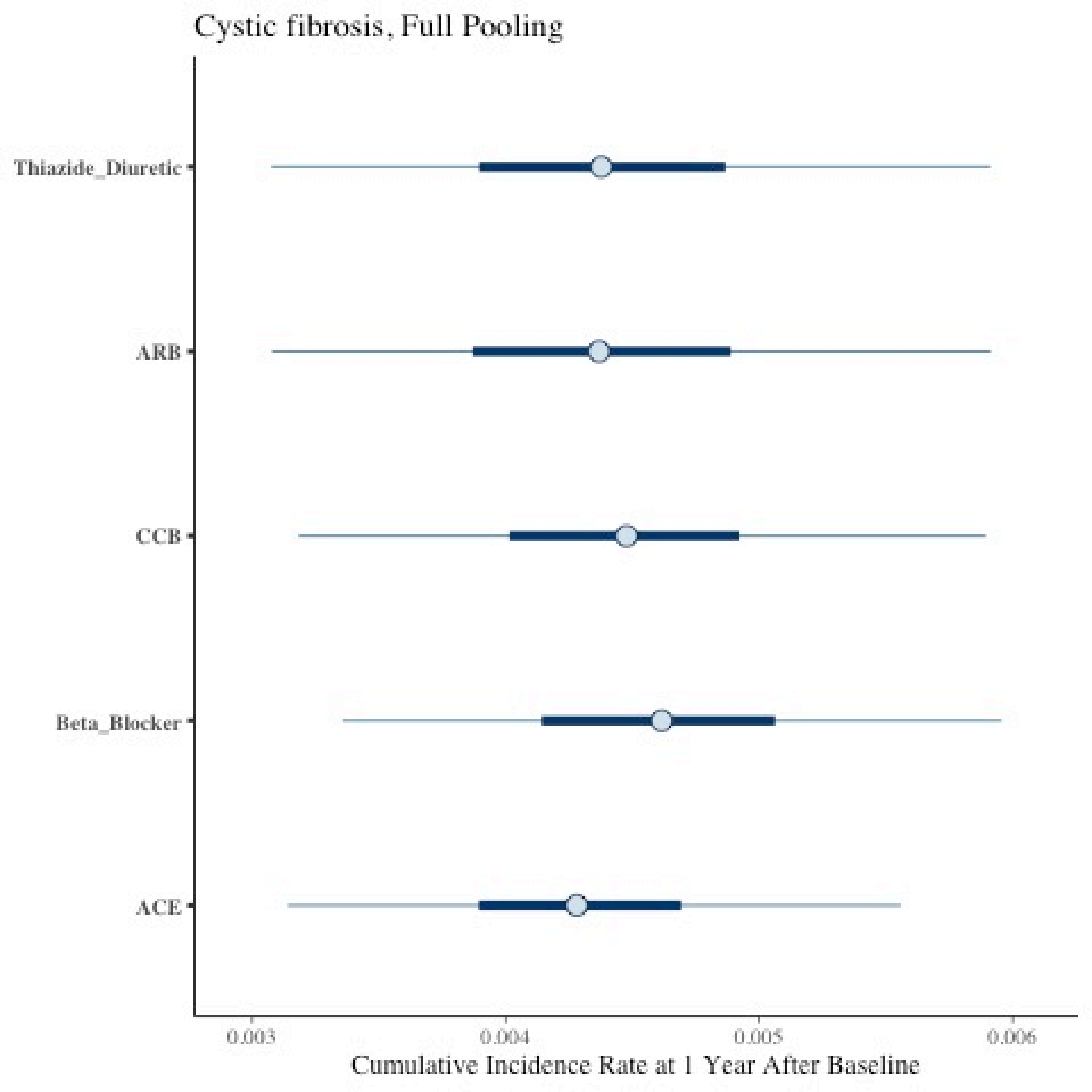
Cystic fibrosis, Full Pooling
Thiazide_Diuretic
ARB
CCB
Beta_Blocker
ACE
0.003
0.004
0.005
0.006
Cumulative Incidence Rate at 1 Year After Baseline

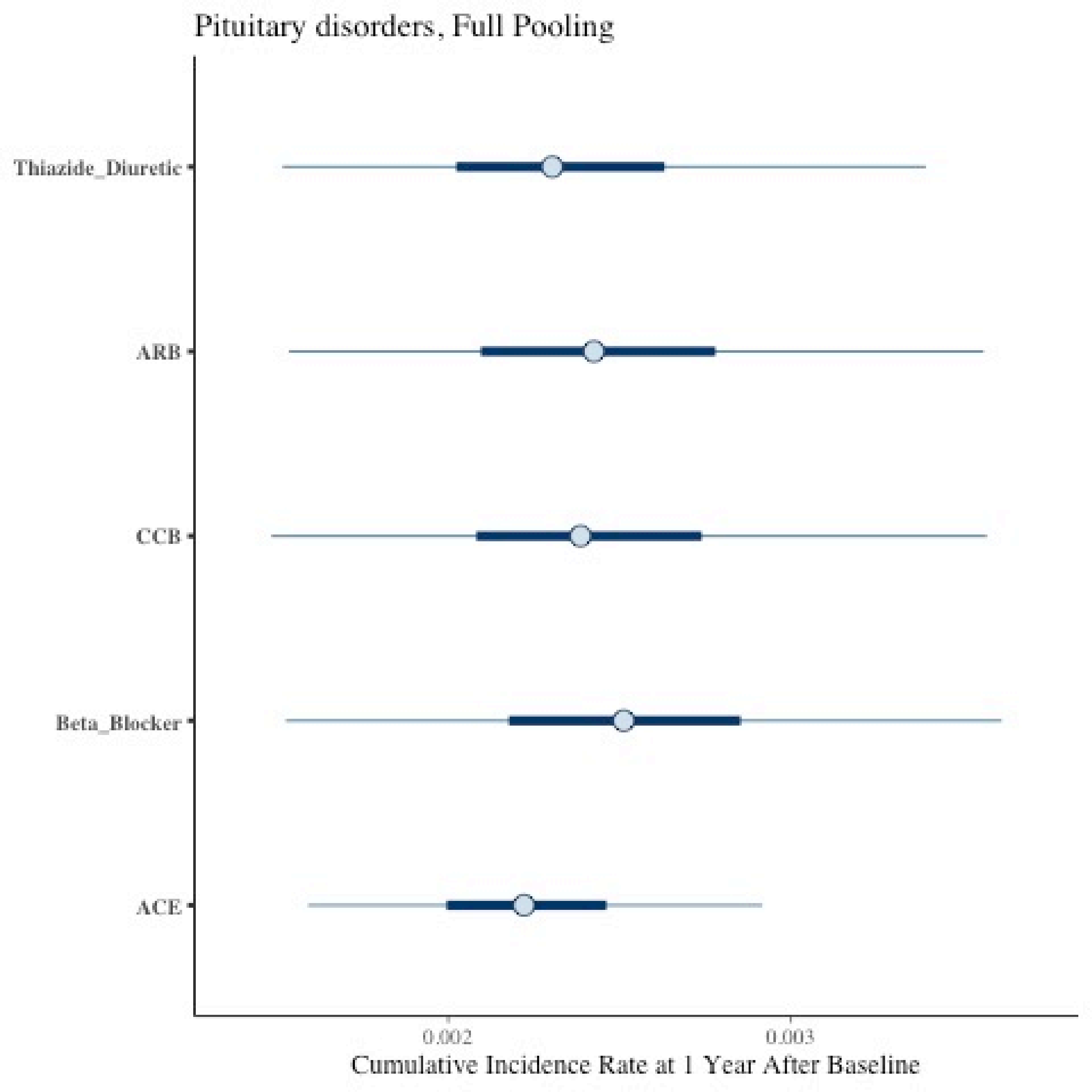

Pituitary disorders, Full Pooling
Thiazide_Diuretic
ARB
CCB
Beta_Blocker
ACE
0.002
0.003
Cumulative Incidence Rate at 1 Year After Baseline

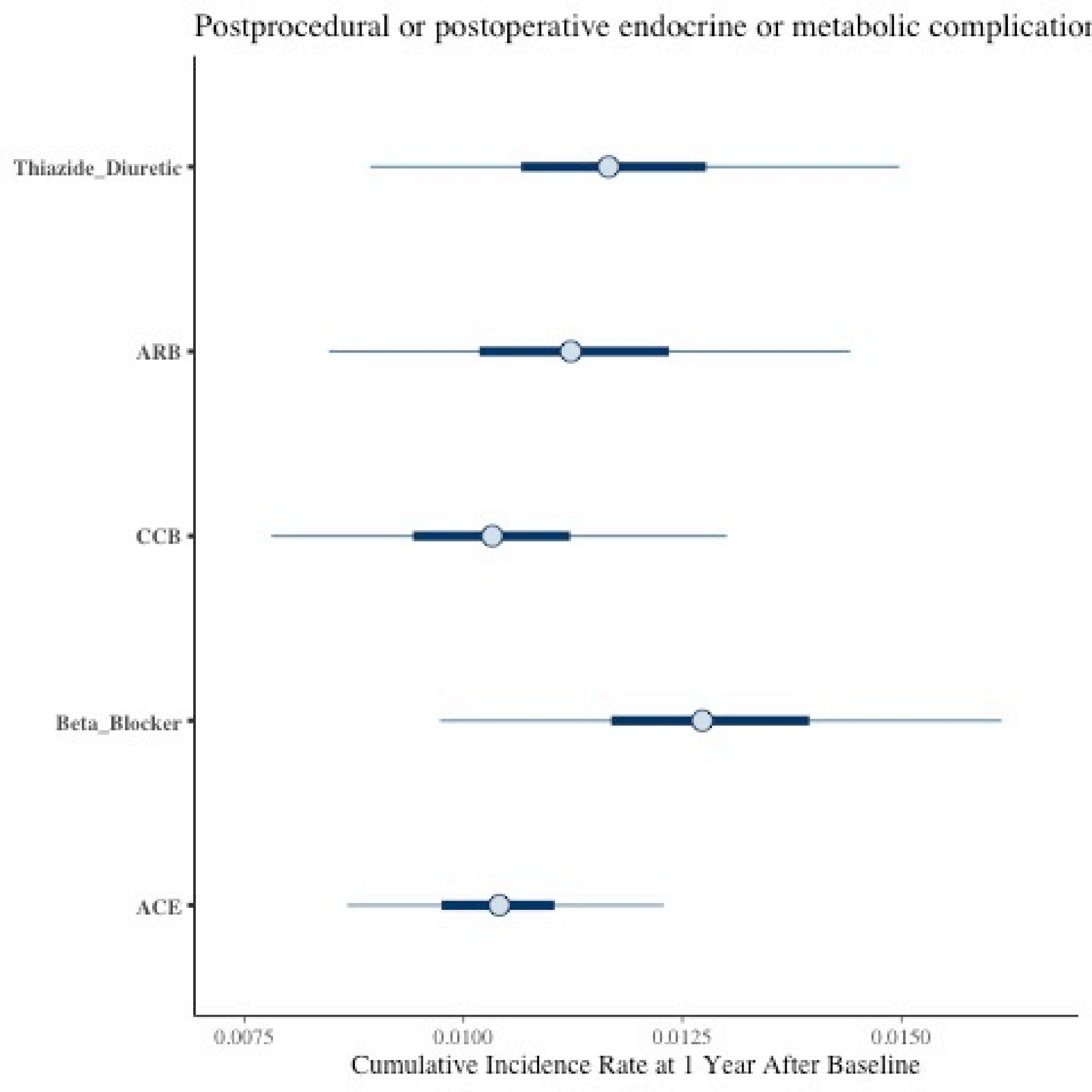

Postprocedural or postoperative endocrine or metabolic complication
Thiazide_Diuretic
ARB
CCB
Beta_Blocker
ACE
0.0075
0.0100
0.0125
0.0150
Cumulative Incidence Rate at 1 Year After Baseline

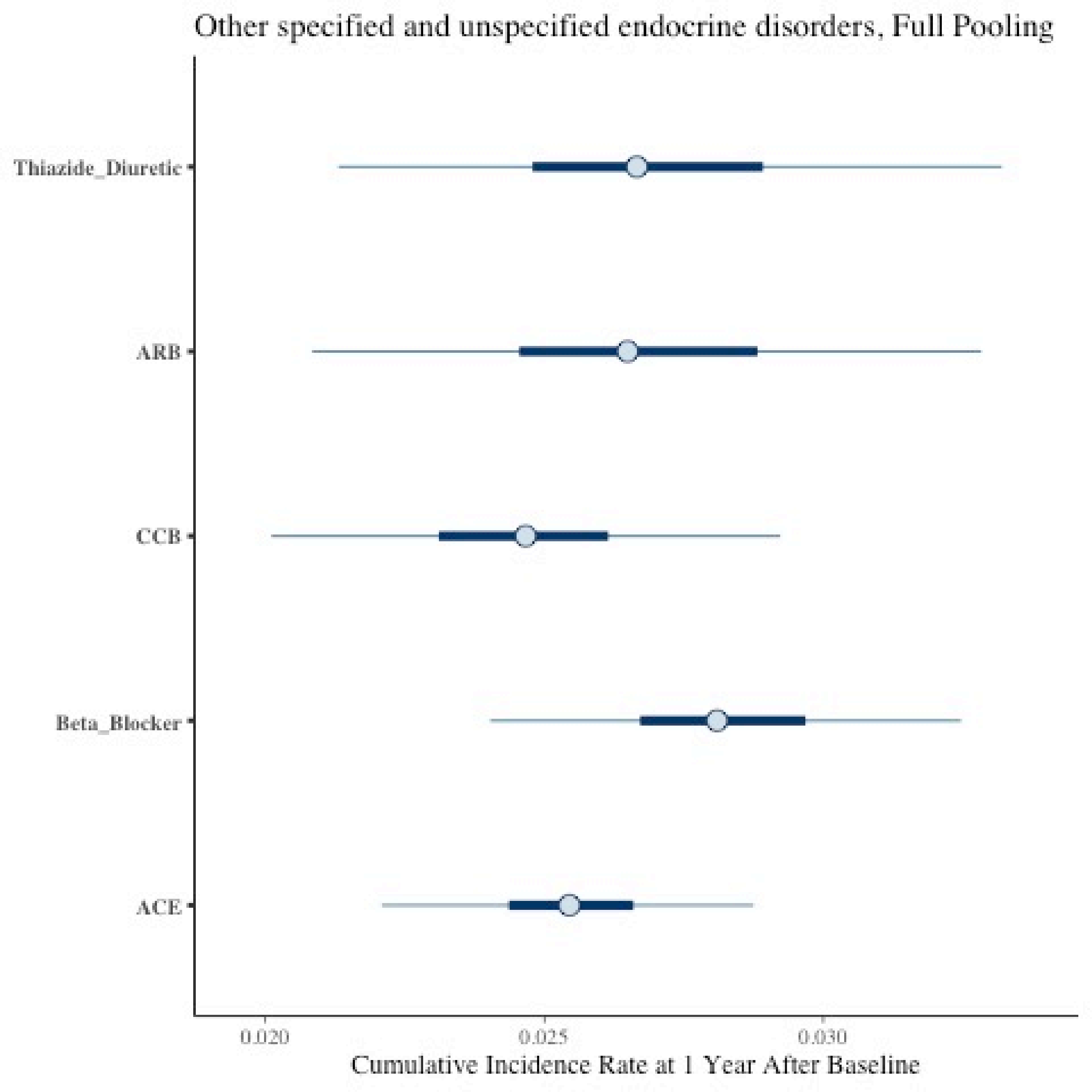

Other specified and unspecified endocrine disorders, Full Pooling
Thiazide_Diuretic
ARB
CCB
Beta_Blocker
ACE
0.020
0.025
0.030
Cumulative Incidence Rate at 1 Year After Baseline

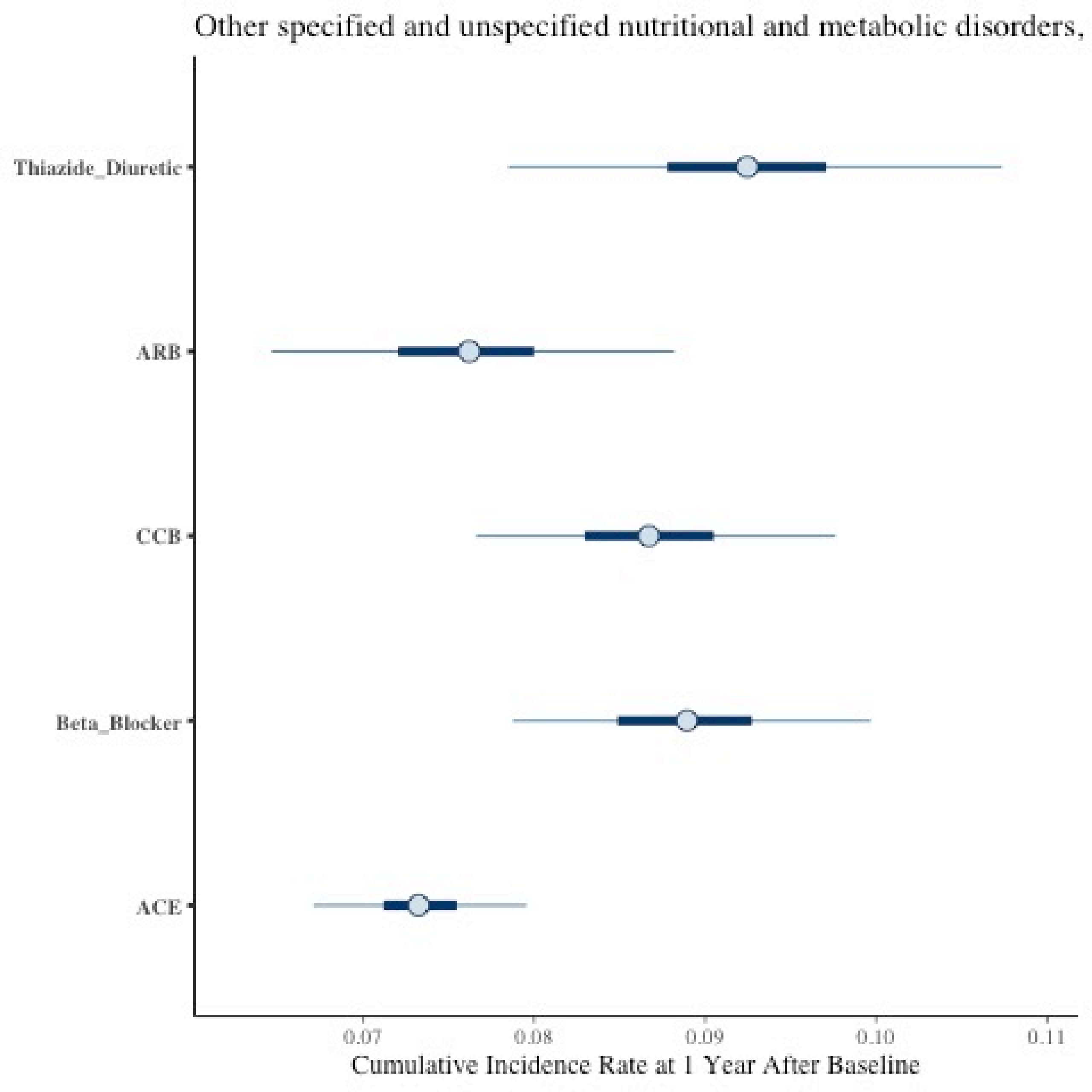

Other specified and unspecified nutritional and metabolic disorders,
Thiazide_Diuretic
ARB
CCB
Beta_Blocker
ACE
0.07
0.08
0.09
0.10
0.11
Cumulative Incidence Rate at 1 Year After Baseline

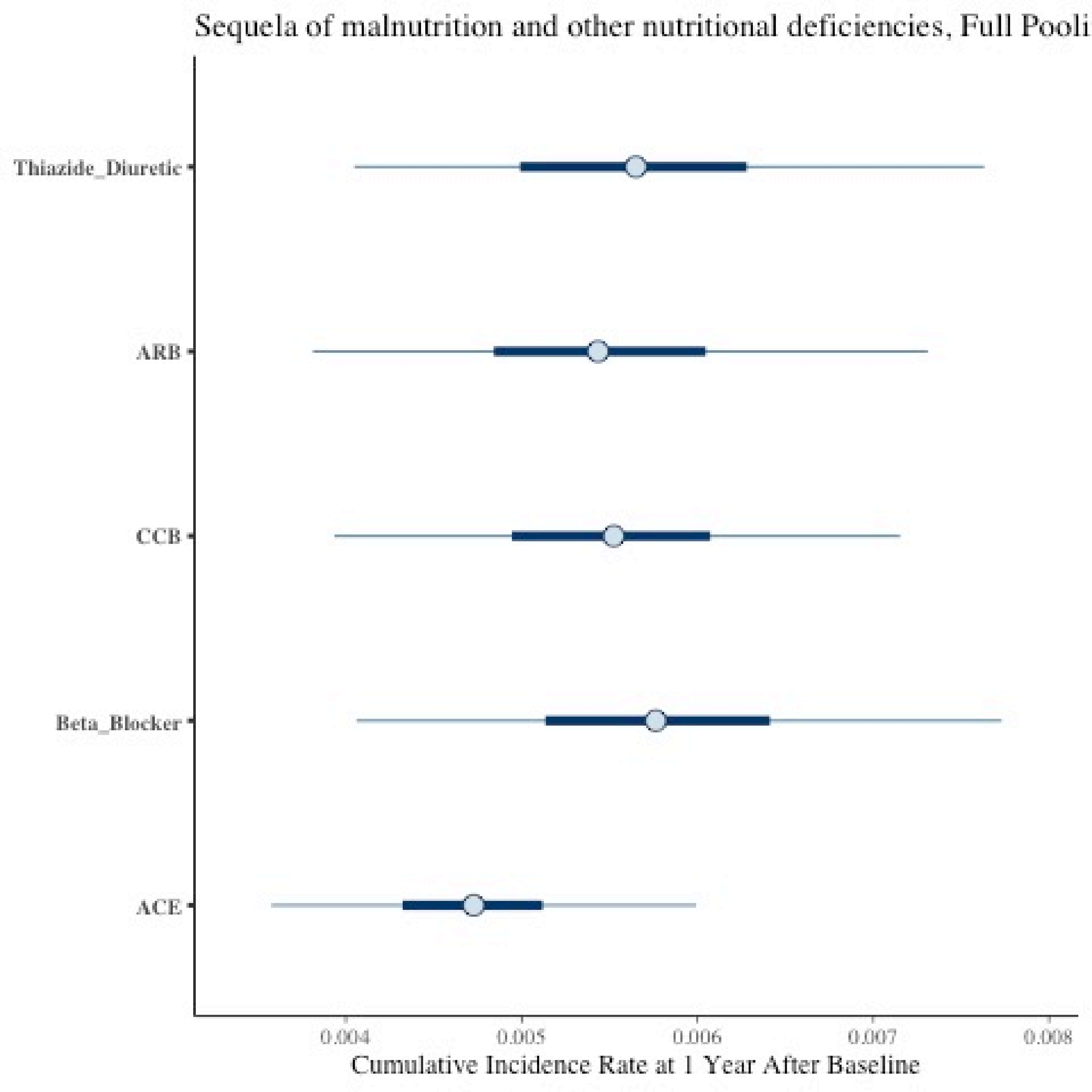
Sequela of malnutrition and other nutritional deficiencies, Full Pooli
Thiazide_Diuretic
ARB
CCB
Beta_Blocker
ACE
0.004
0.005
0.006
0.007
0.008
Cumulative Incidence Rate at 1 Year After Baseline

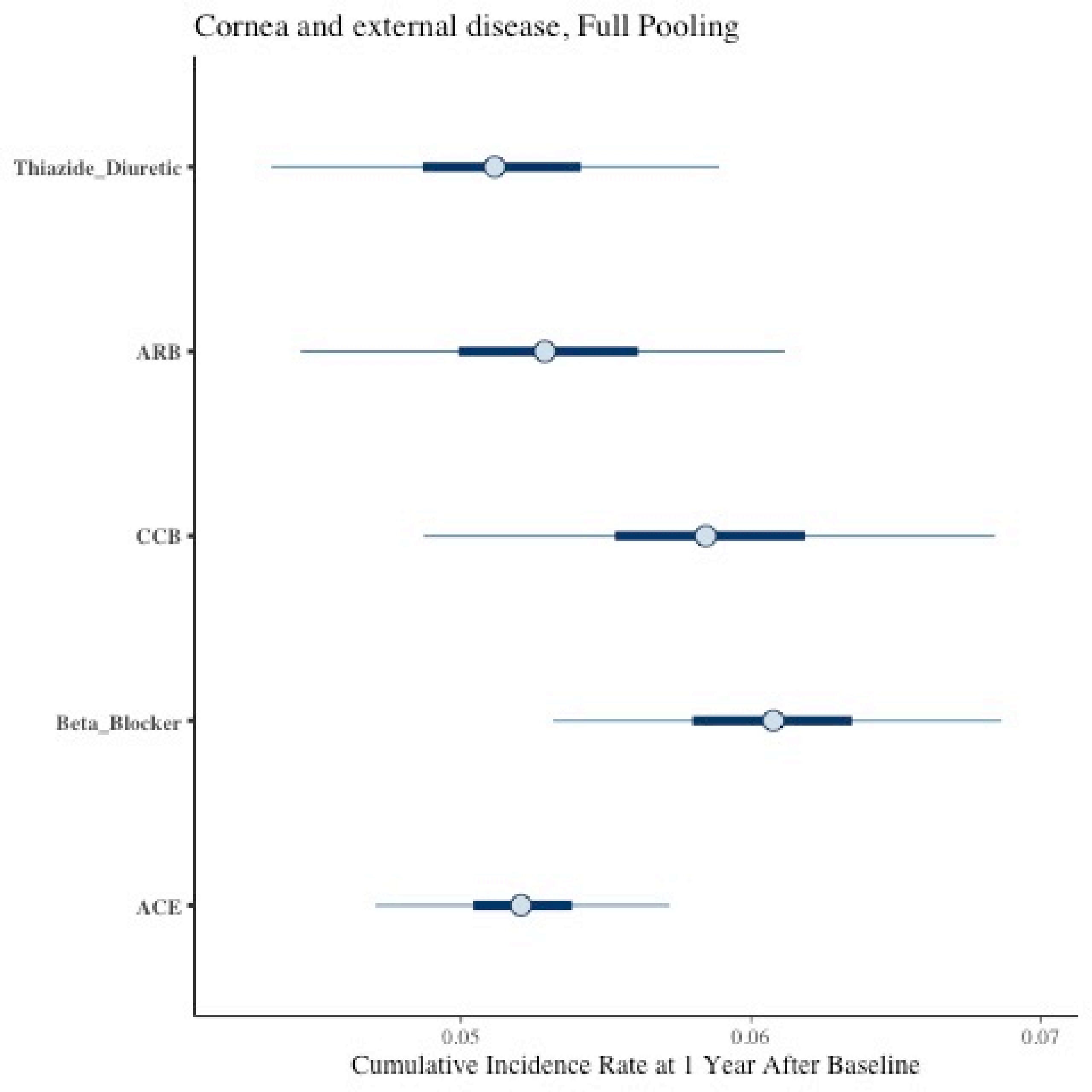
Cornea and external disease, Full Pooling
Thiazide_Diuretic
ARB
CCB
Beta_Blocker
ACE
0.05
0.06
0.07
Cumulative Incidence Rate at 1 Year After Baseline

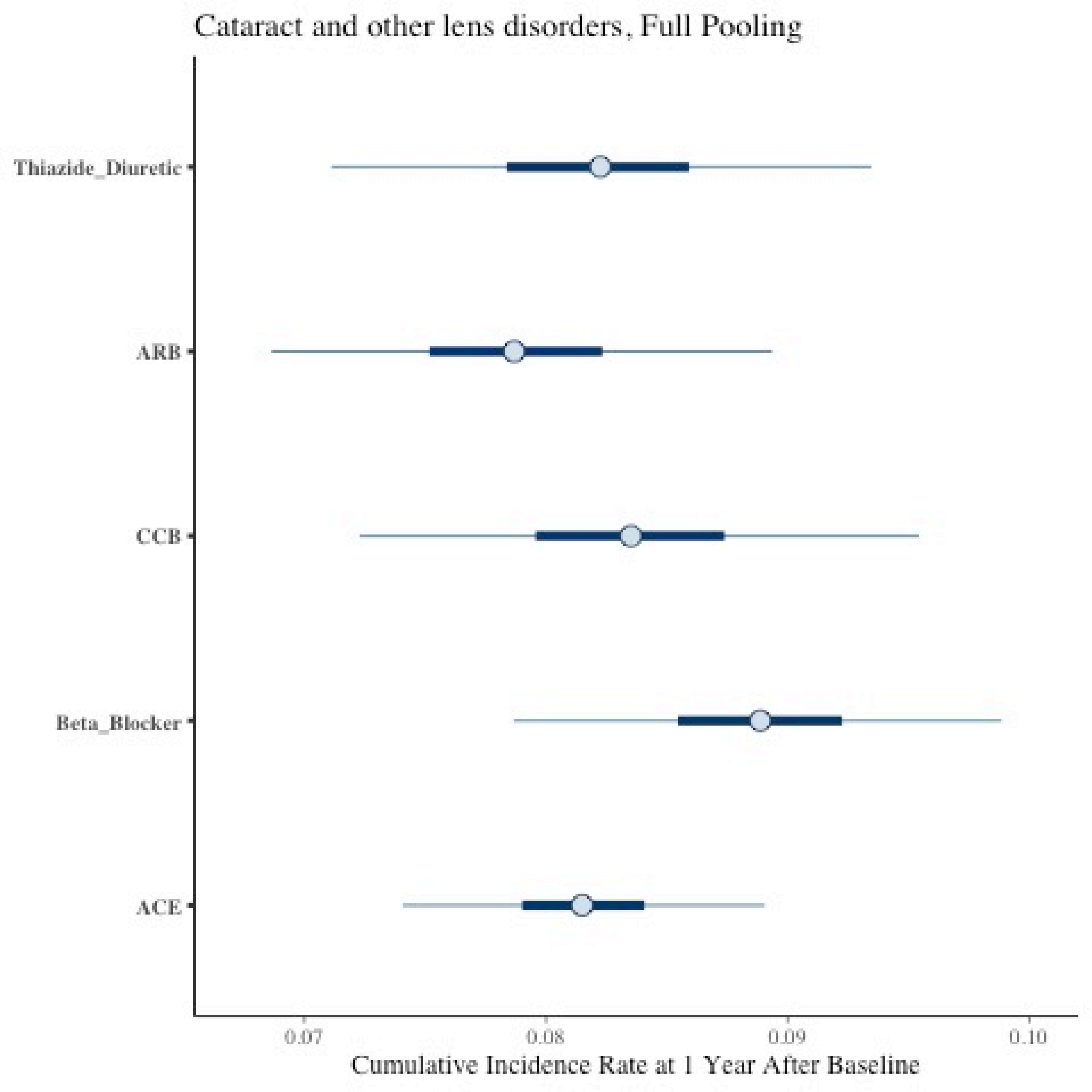

Cataract and other lens disorders, Full Pooling
Thiazide_Diuretic
ARB
CCB
Beta_Blocker
ACE
0.07
0.08
0.09
0.10
Cumulative Incidence Rate at 1 Year After Baseline

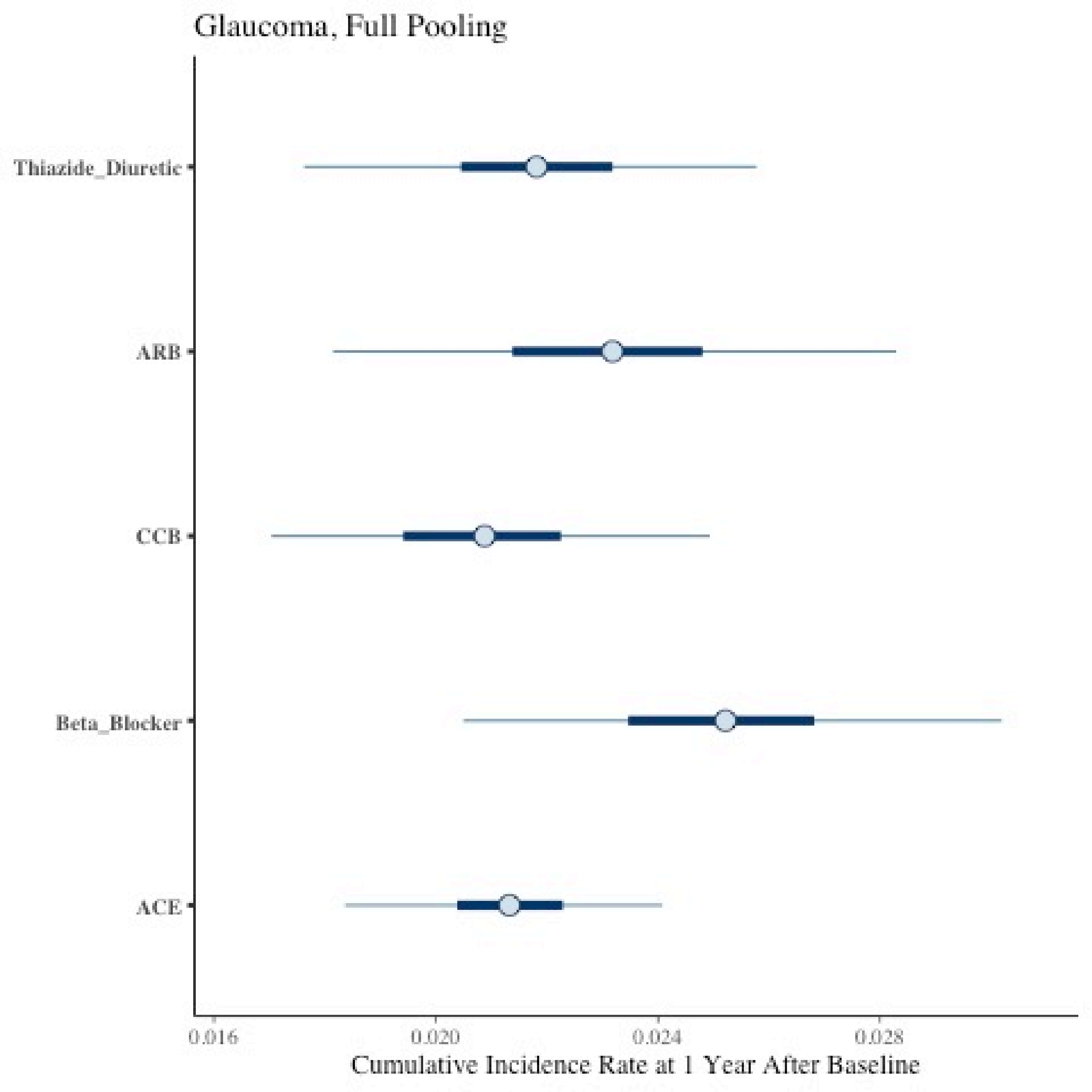

Glaucoma, Full Pooling
Thiazide_Diuretic
ARB
CCB
Beta_Blocker
ACE
0.016
0.020
0.024
0.028
Cumulative Incidence Rate at 1 Year After Baseline

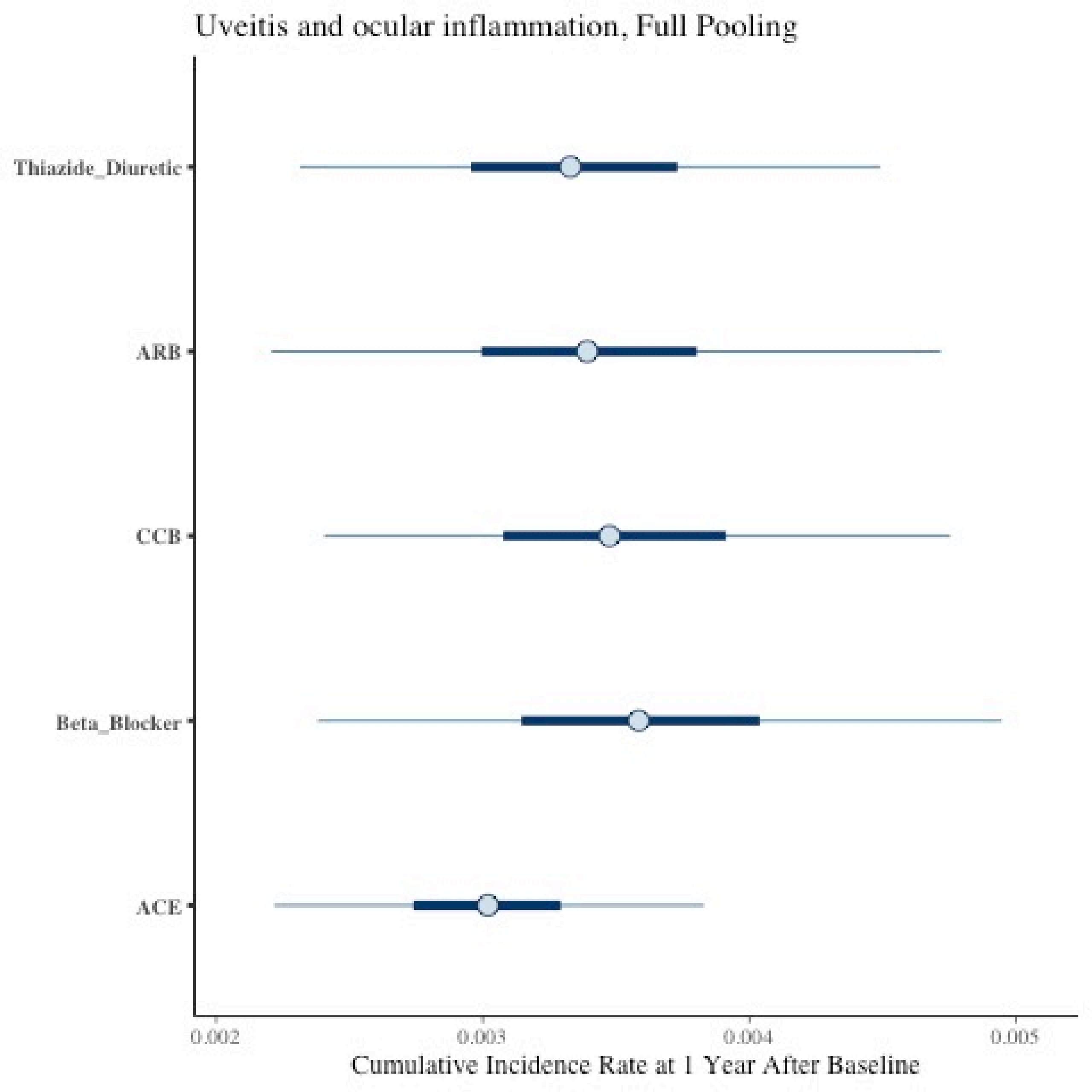
Uveitis and ocular inflammation, Full Pooling
Thiazide_Diuretic
ARB
CCB
Beta_Blocker
ACE
0.002
0.003
0.004
0.005
Cumulative Incidence Rate at 1 Year After Baseline

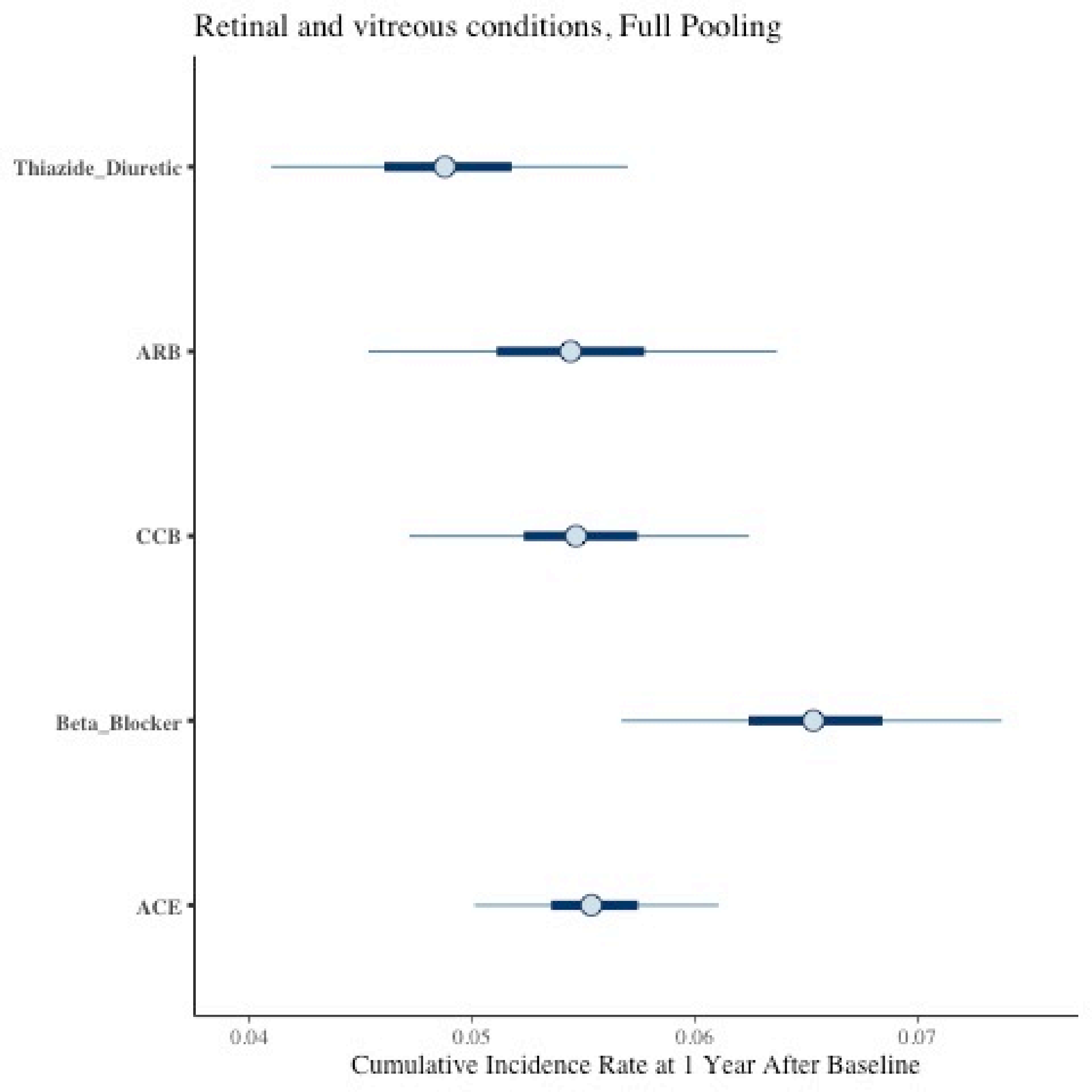
Retinal and vitreous conditions, Full Pooling
Thiazide_Diuretic
ARB
CCB
Beta_Blocker
ACE
0.04
0.05
0.06
0.07
Cumulative Incidence Rate at 1 Year After Baseline

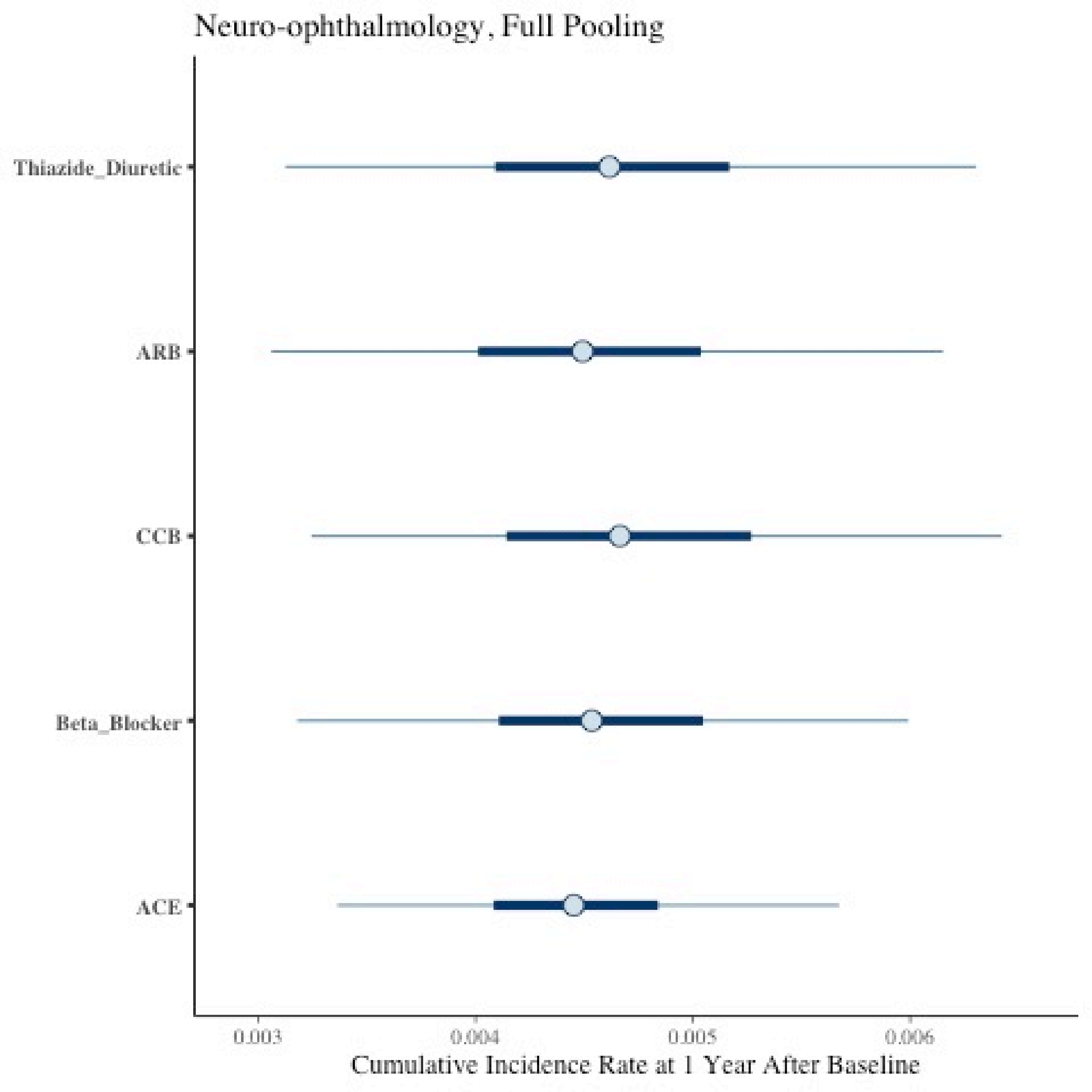

Neuro-ophthalmology, Full Pooling
Thiazide_Diuretic
ARB
CCB
Beta_Blocker
ACE
0.003
0.004
0.005
0.006
Cumulative Incidence Rate at 1 Year After Baseline

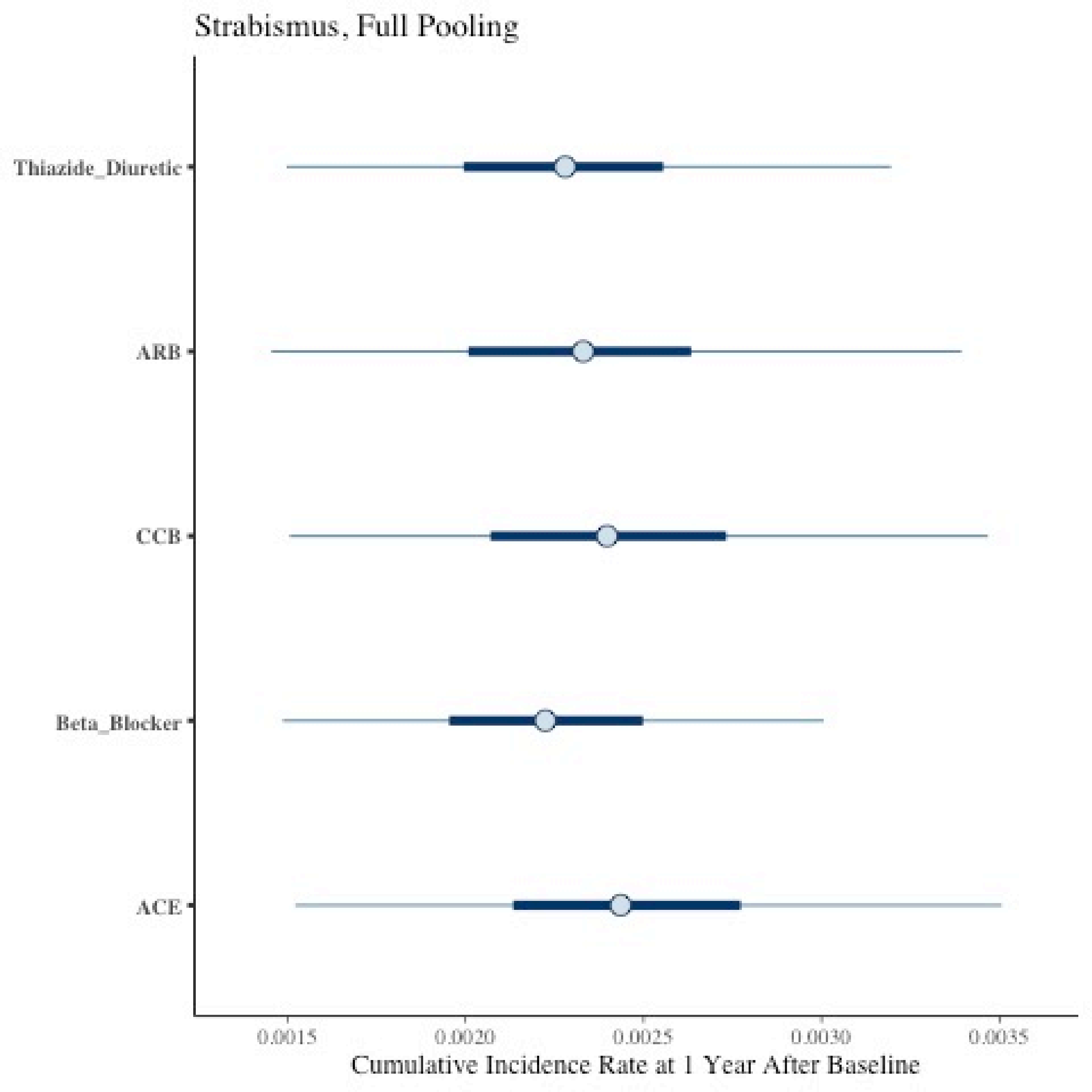

Strabismus, Full Pooling
Thiazide_Diuretic
ARB
CCB
Beta_Blocker
ACE
0.0015
0.0020
0.0025
0.0030
0.0035
Cumulative Incidence Rate at 1 Year After Baseline

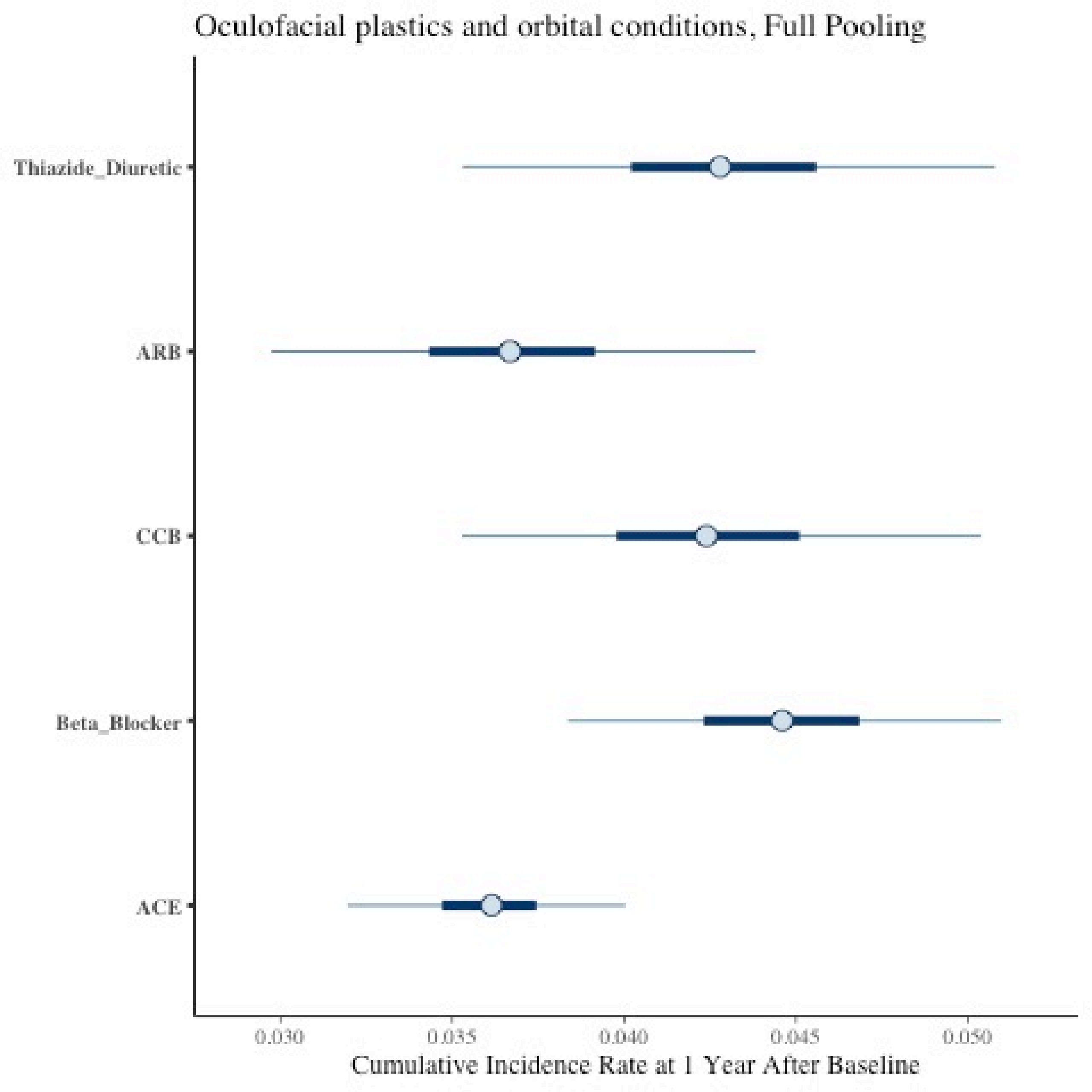

Oculofacial plastics and orbital conditions, Full Pooling
Thiazide_Diuretic
ARB
CCB
Beta_Blocker
ACE
0.030
0.035
0.040
0.045
0.050
Cumulative Incidence Rate at 1 Year After Baseline

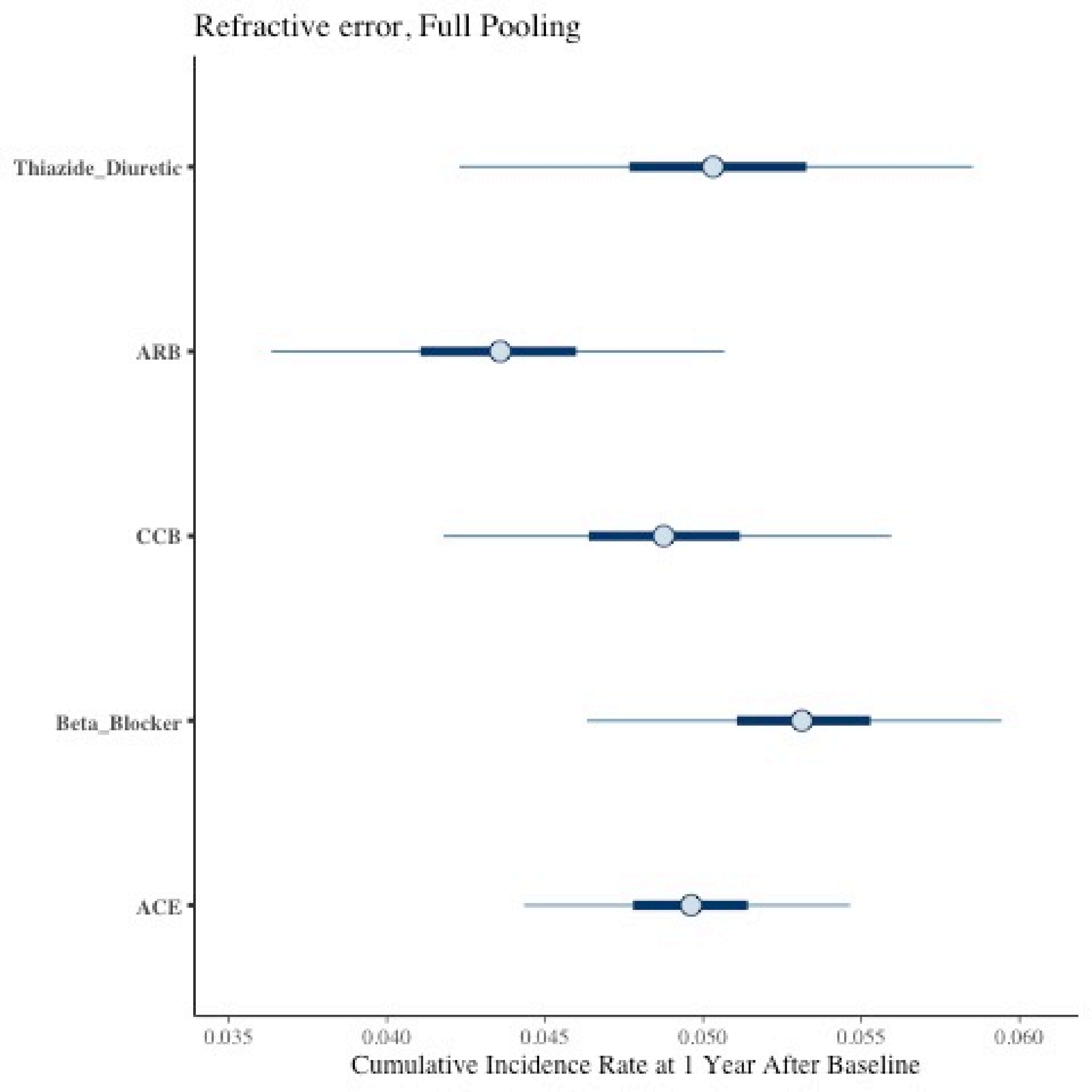

Refractive error, Full Pooling
Thiazide_Diuretic
ARB
CCB
Beta_Blocker
ACE
0.035
0.040
0.045
0.050
0.055
0.060
Cumulative Incidence Rate at 1 Year After Baseline

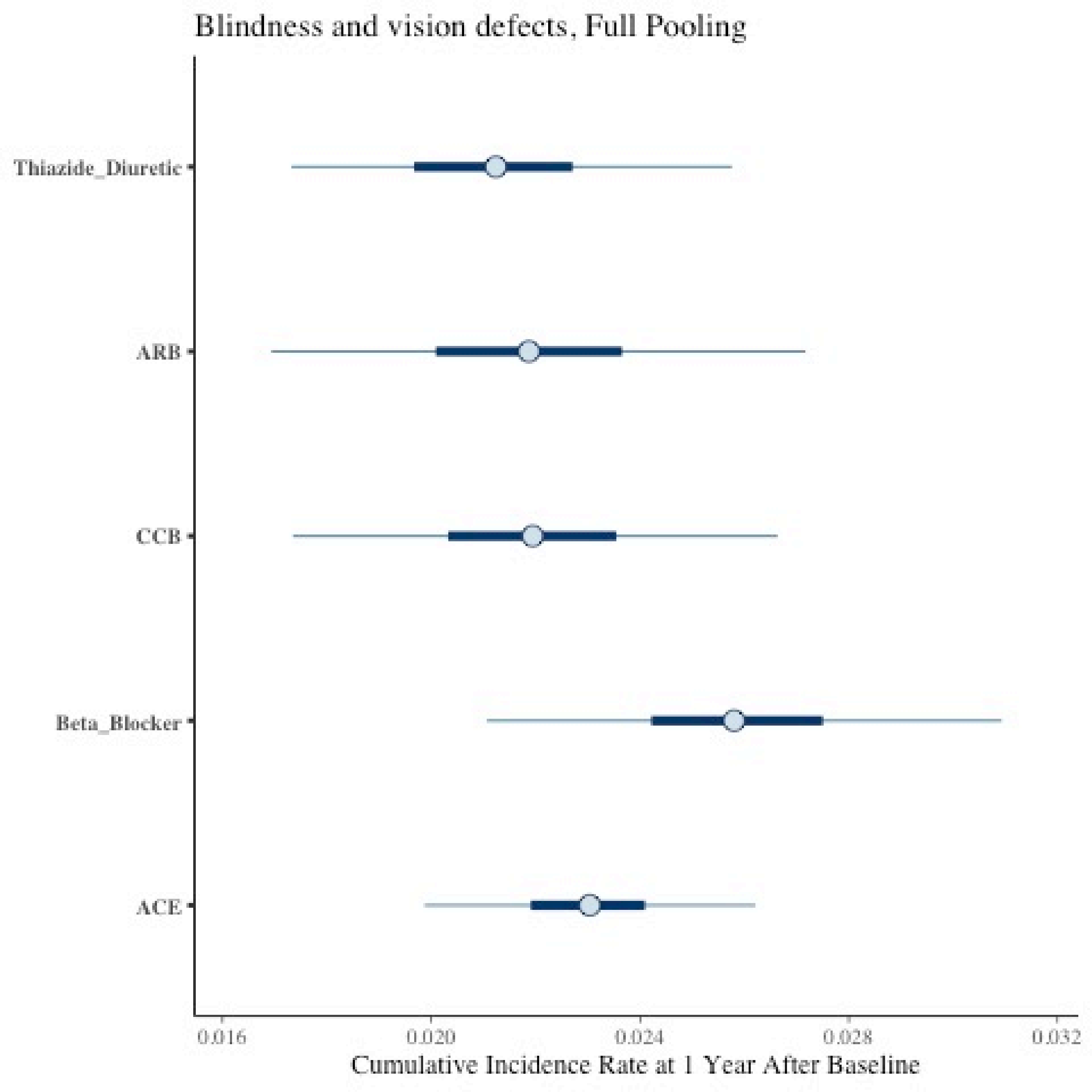
Blindness and vision defects, Full Pooling
Thiazide_Diuretic
ARB
CCB
Beta_Blocker
ACE
0.016
0.020
0.024
0.028
0.032
Cumulative Incidence Rate at 1 Year After Baseline

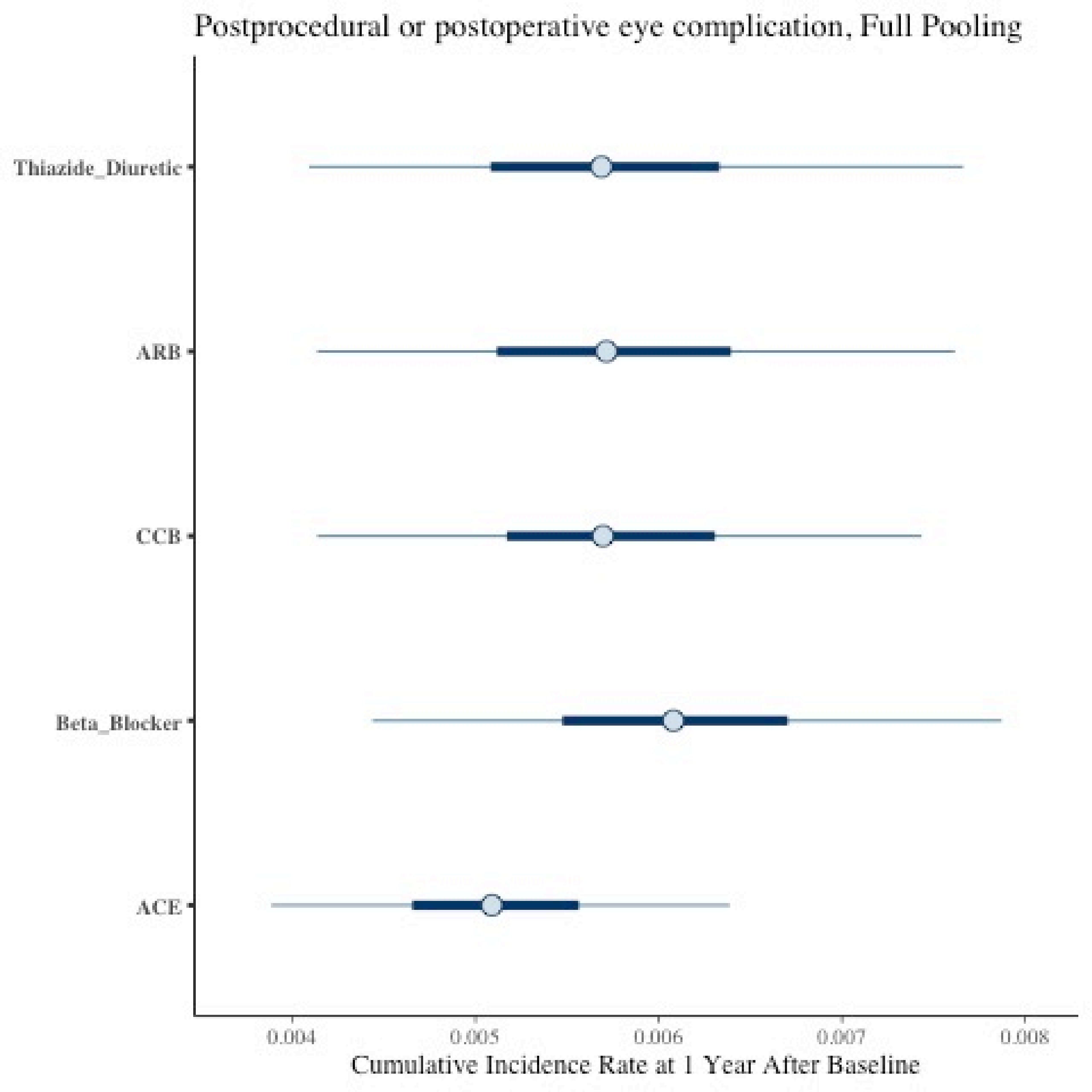

Postprocedural or postoperative eye complication, Full Pooling
Thiazide_Diuretic
ARB
CCB
Beta_Blocker
ACE
0.004
0.005
0.006
0.007
0.008
Cumulative Incidence Rate at 1 Year After Baseline

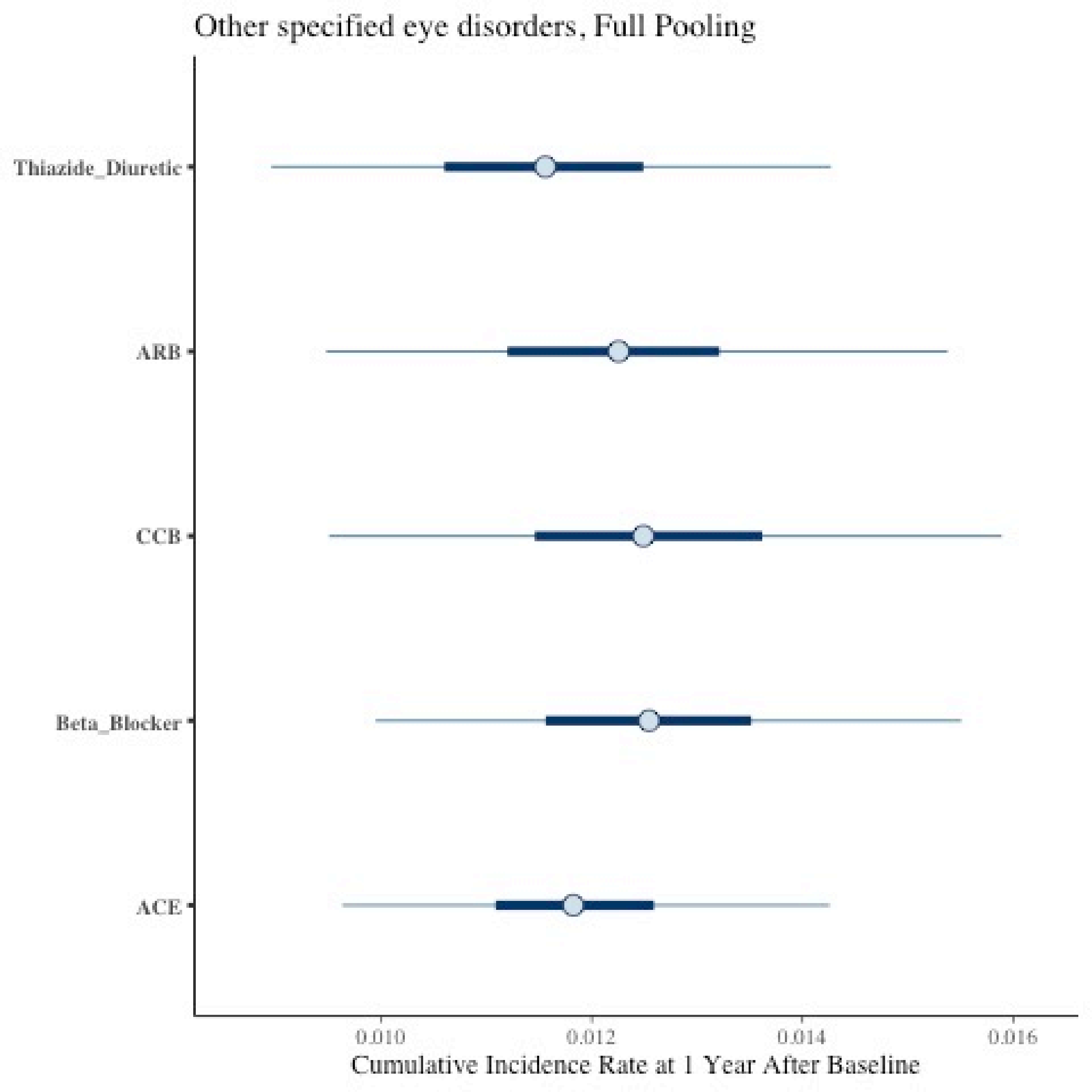

Other specified eye disorders, Full Pooling
Thiazide_Diuretic
ARB
CCB
Beta_Blocker
ACE
0.010
0.012
0.014
0.016
Cumulative Incidence Rate at 1 Year After Baseline

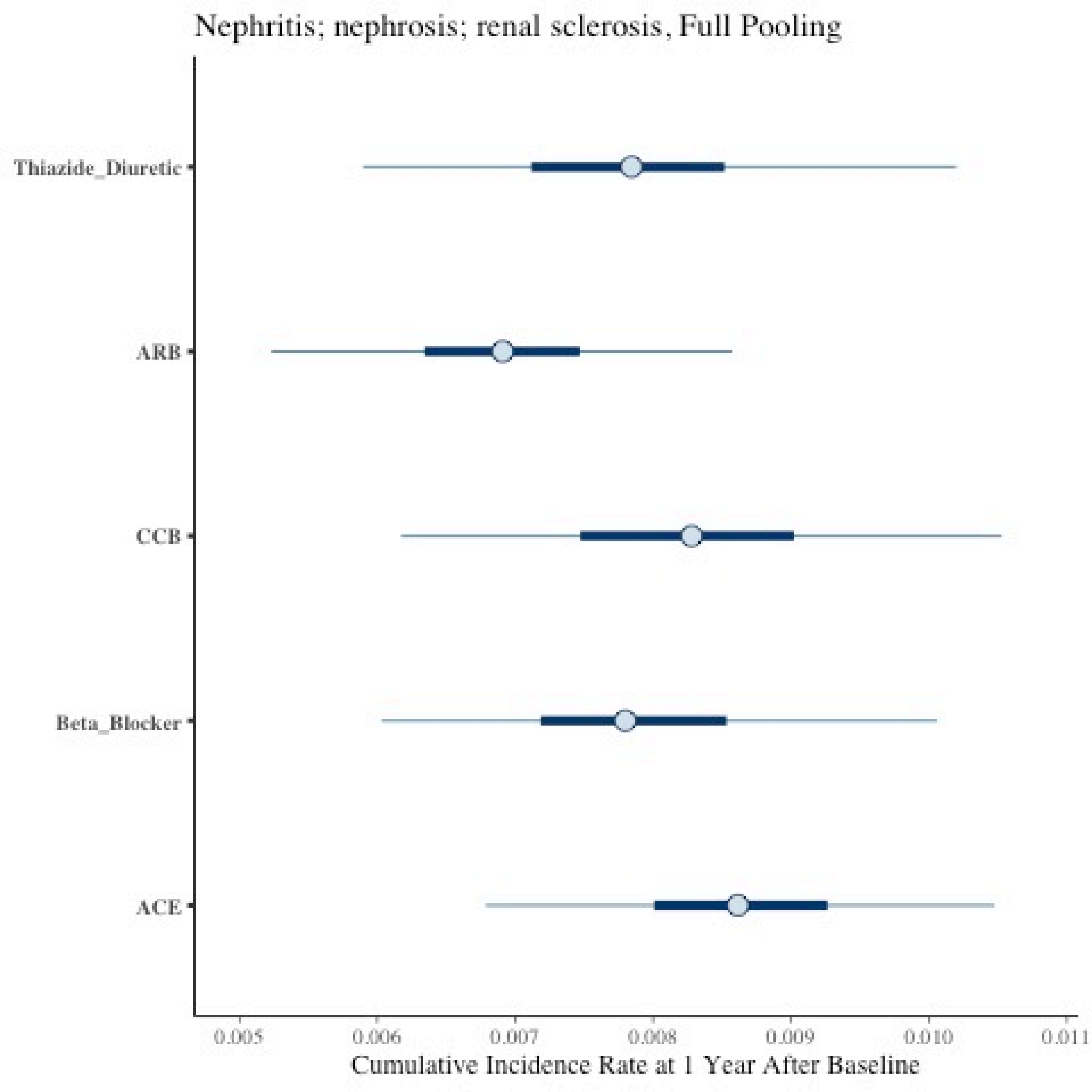
Nephritis; nephrosis; renal sclerosis, Full Pooling
Thiazide_Diuretic
ARB
CCB
Beta_Blocker
ACE
0.005
0.006
0.007
0.008
0.009
0.010
0.011
Cumulative Incidence Rate at 1 Year After Baseline

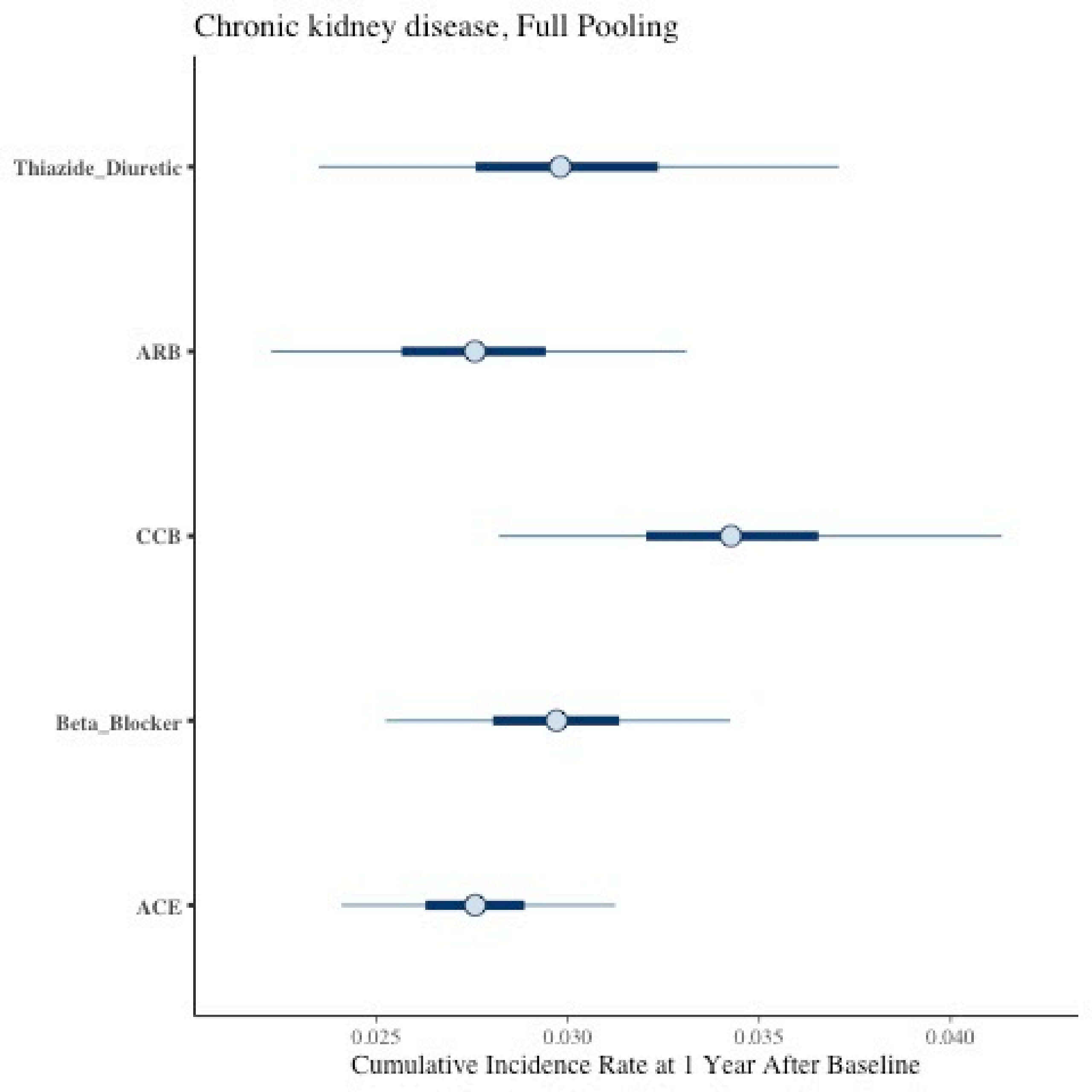

Chronic kidney disease, Full Pooling
Thiazide_Diuretic
ARB
CCB
Beta_Blocker
ACE
0.025
0.030
0.035
0.040
Cumulative Incidence Rate at 1 Year After Baseline

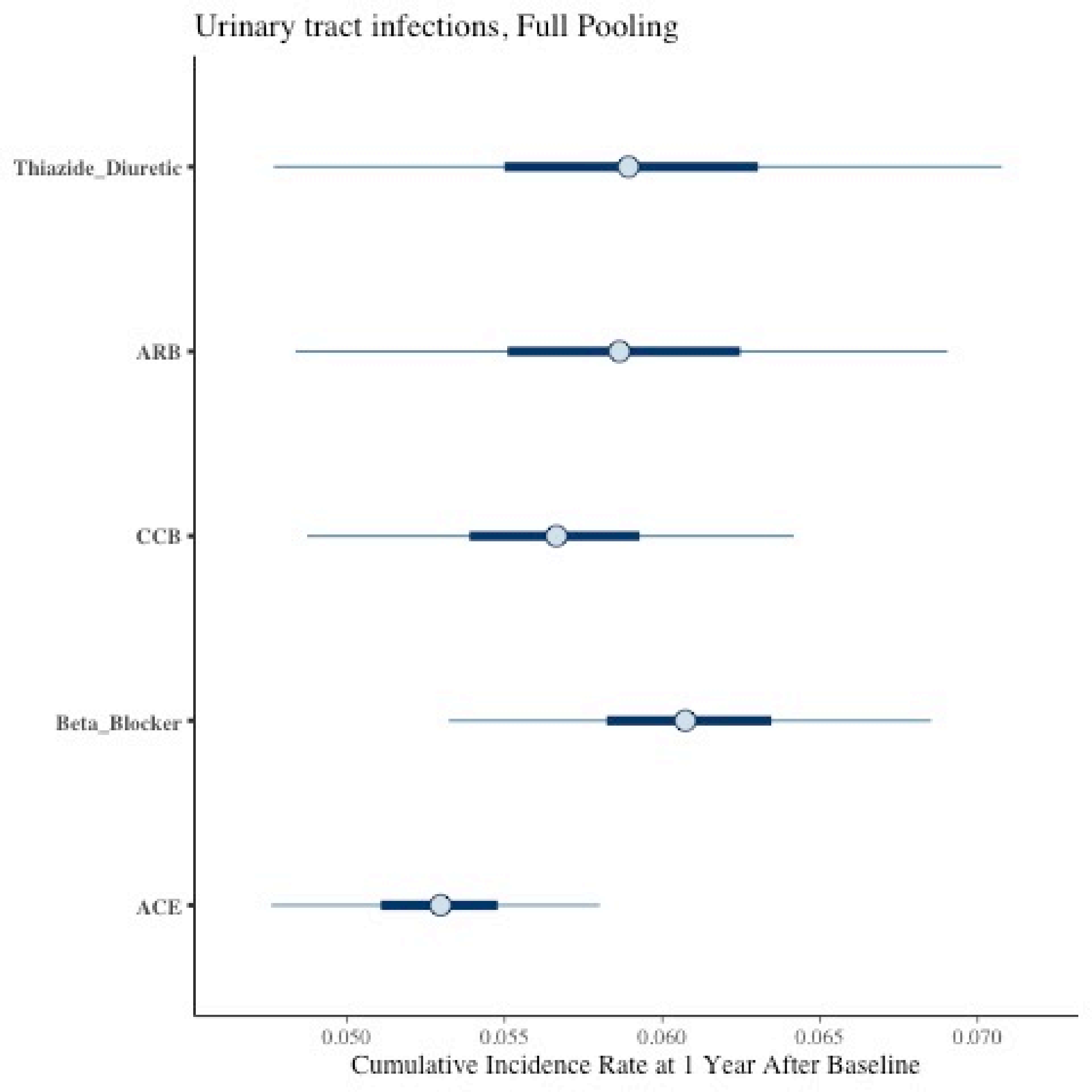

Urinary tract infections, Full Pooling
Thiazide_Diuretic
ARB
CCB
Beta_Blocker
ACE
0.050
0.055
0.060
0.065
0.070
Cumulative Incidence Rate at 1 Year After Baseline

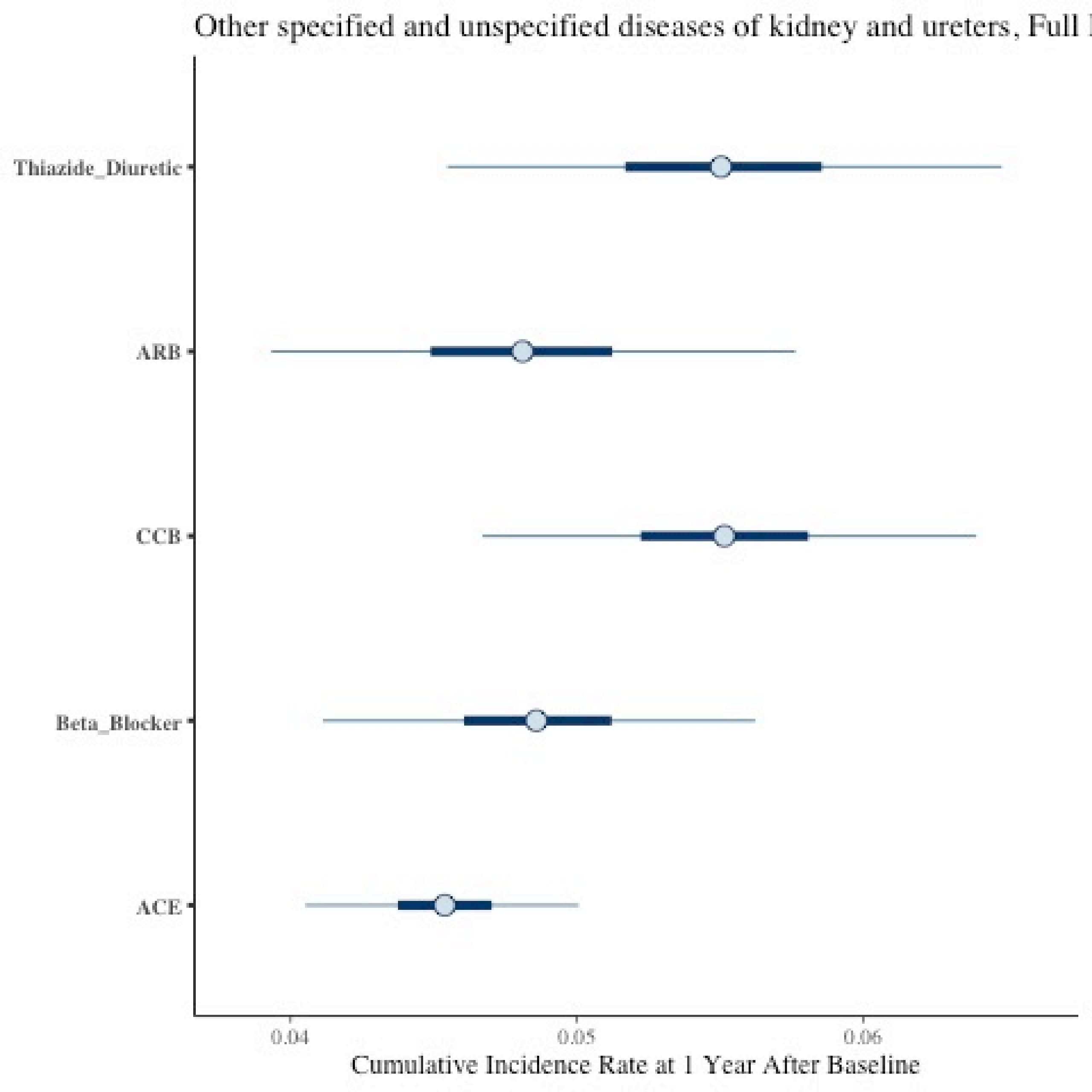

Other specified and unspecified diseases of kidney and ureters, Full
Thiazide_Diuretic
ARB
CCB
Beta_Blocker
ACE
0.04
0.05
0.06
Cumulative Incidence Rate at 1 Year After Baseline

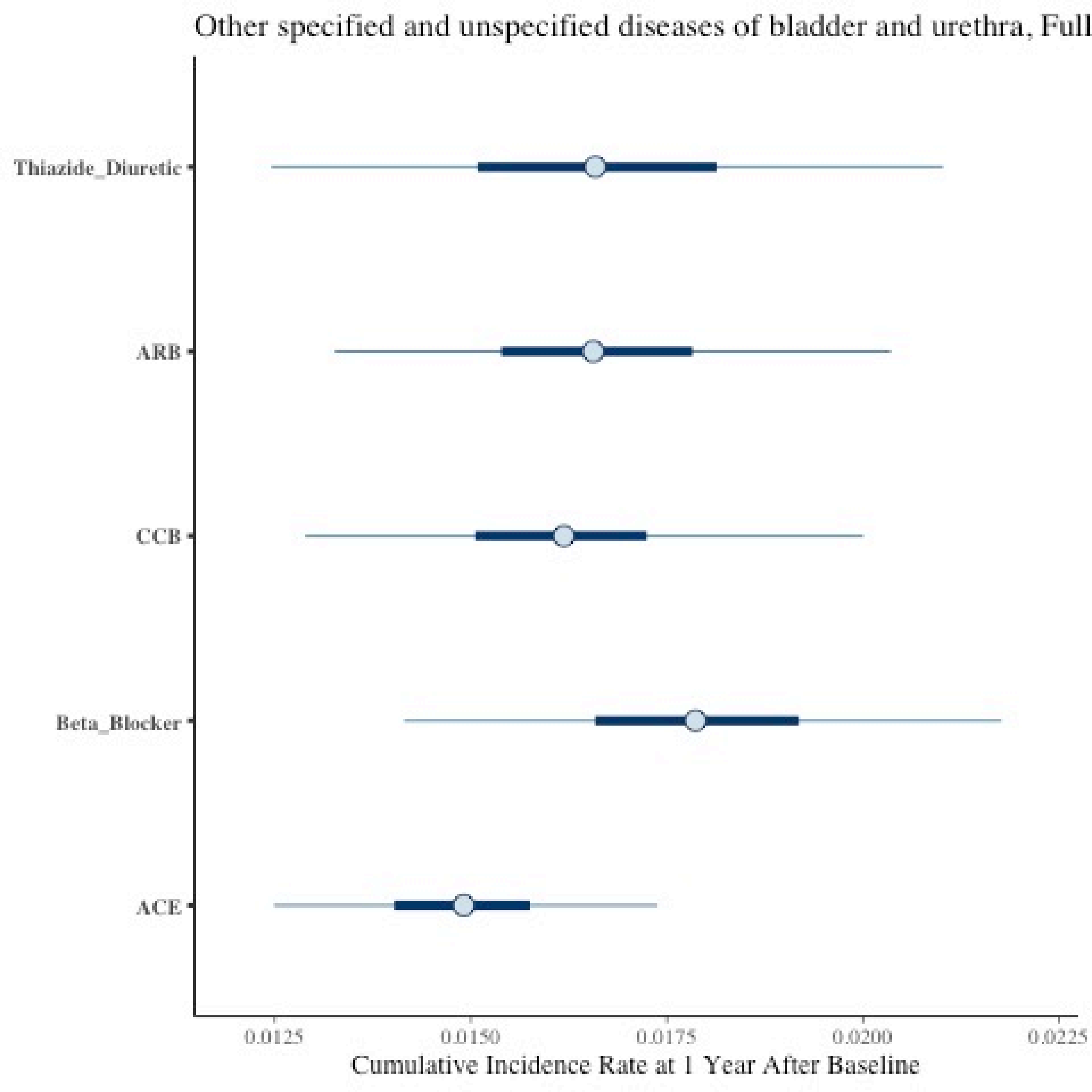

Other specified and unspecified diseases of bladder and urethra, Full
Thiazide_Diuretic
ARB
CCB
Beta_Blocker
ACE
0.0125
0.0150
0.0175
0.0200
0.0225
Cumulative Incidence Rate at 1 Year After Baseline

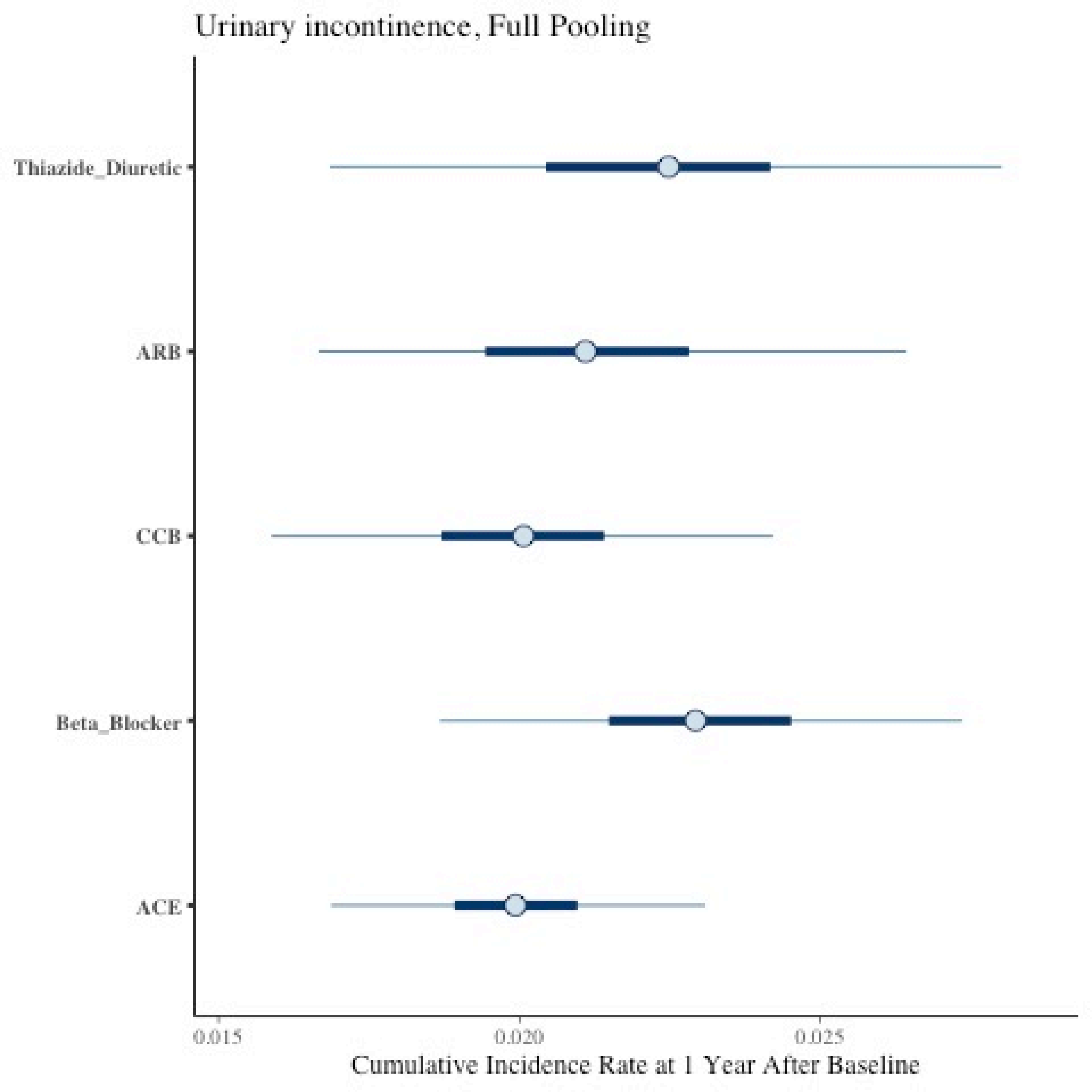

Urinary incontinence, Full Pooling
Thiazide_Diuretic
ARB
CCB
Beta_Blocker
ACE
0.015
0.020
0.025
Cumulative Incidence Rate at 1 Year After Baseline

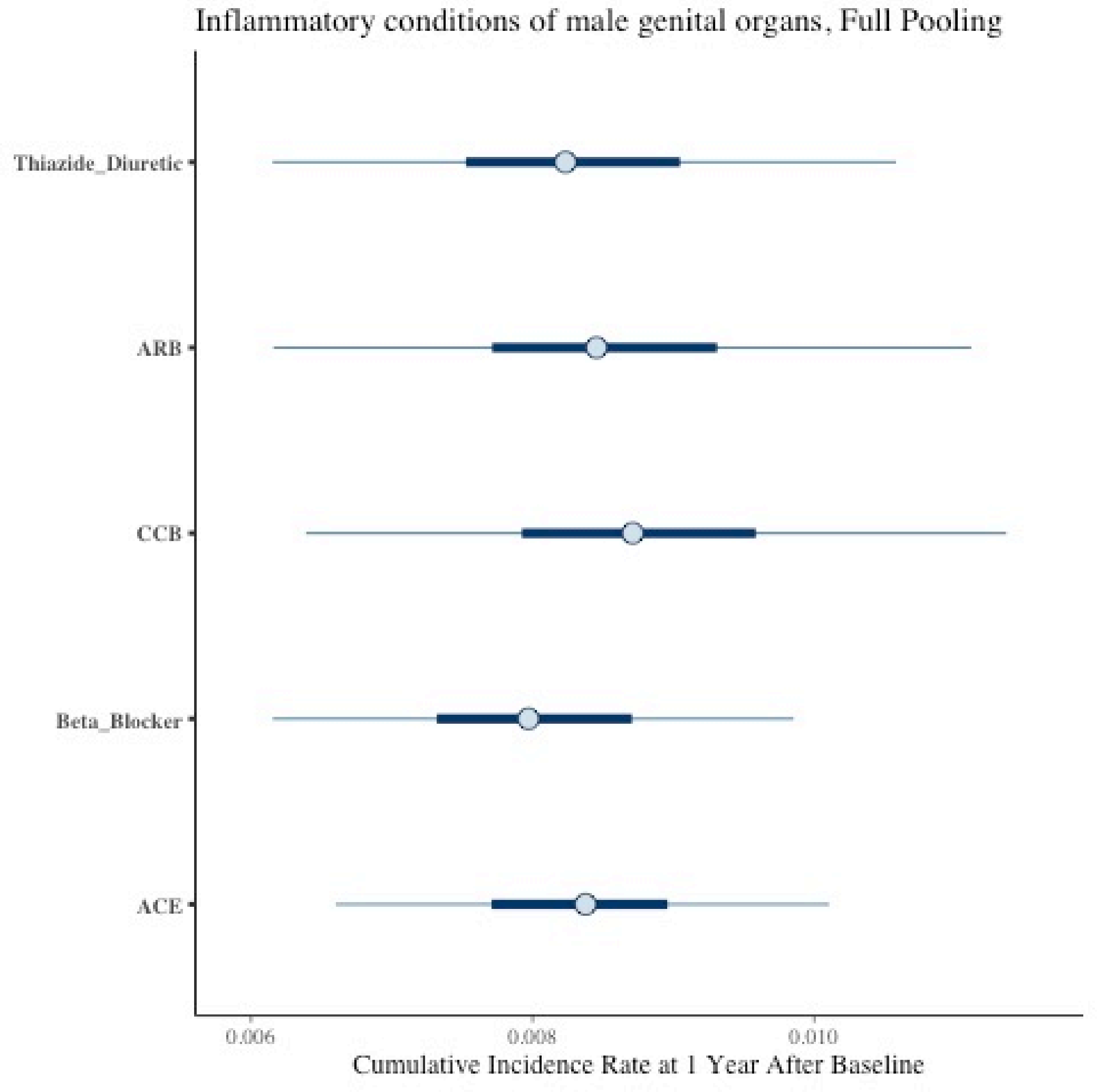

Inflammatory conditions of male genital organs, Full Pooling
Thiazide_Diuretic
ARB
CCB
Beta_Blocker
ACE
0.006
0.008
0.010
Cumulative Incidence Rate at 1 Year After Baseline

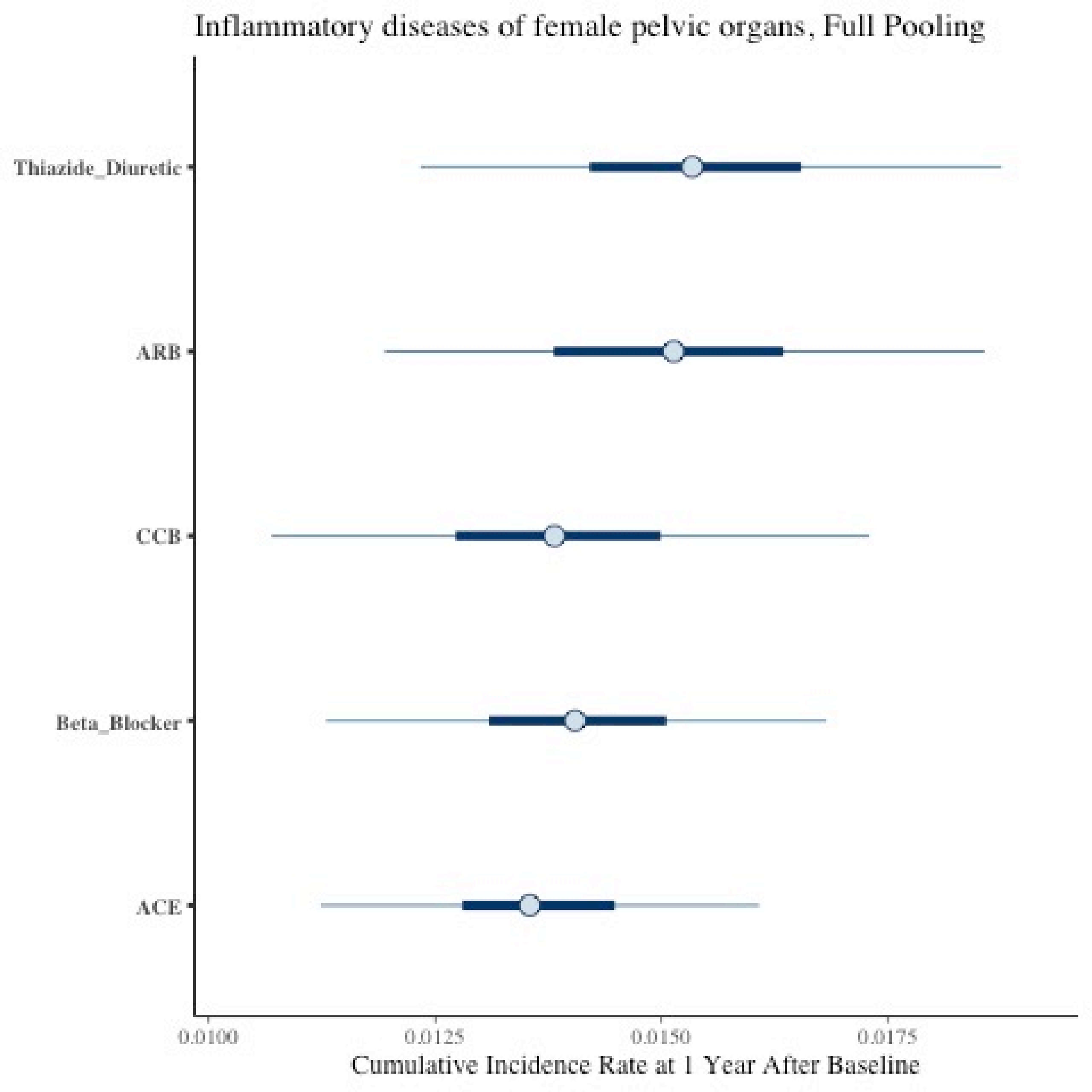
Inflammatory diseases of female pelvic organs, Full Pooling
Thiazide_Diuretic
ARB
CCB
Beta_Blocker
ACE
0.0100
0.0125
0.0150
0.0175
Cumulative Incidence Rate at 1 Year After Baseline

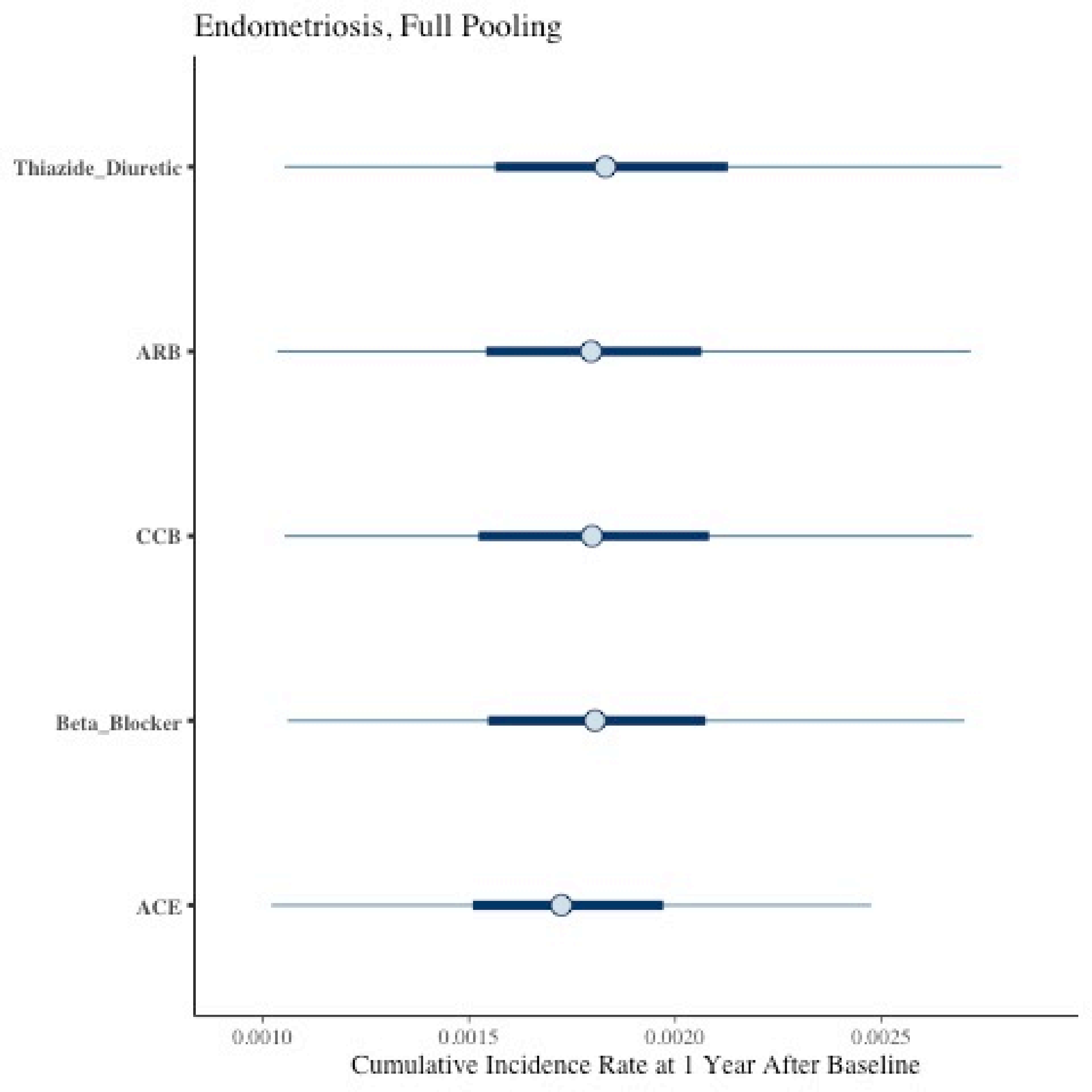
Endometriosis, Full Pooling
Thiazide_Diuretic
ARB
CCB
Beta_Blocker
ACE
0.0010
0.0015
0.0020
0.0025
Cumulative Incidence Rate at 1 Year After Baseline

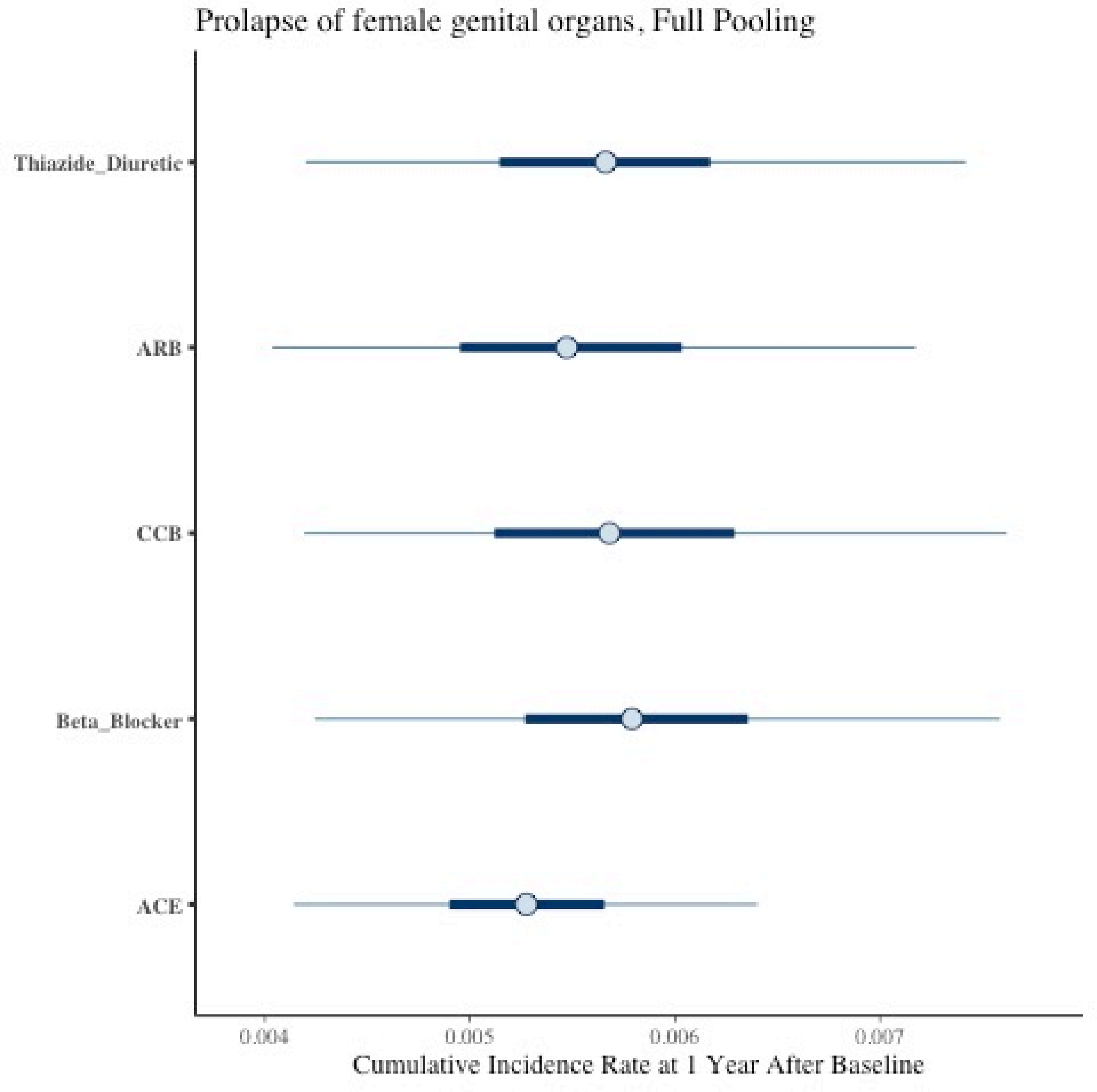

Prolapse of female genital organs, Full Pooling
Thiazide_Diuretic
ARB
CCB
Beta_Blocker
ACE
0.004
0.005
0.006
0.007
Cumulative Incidence Rate at 1 Year After Baseline

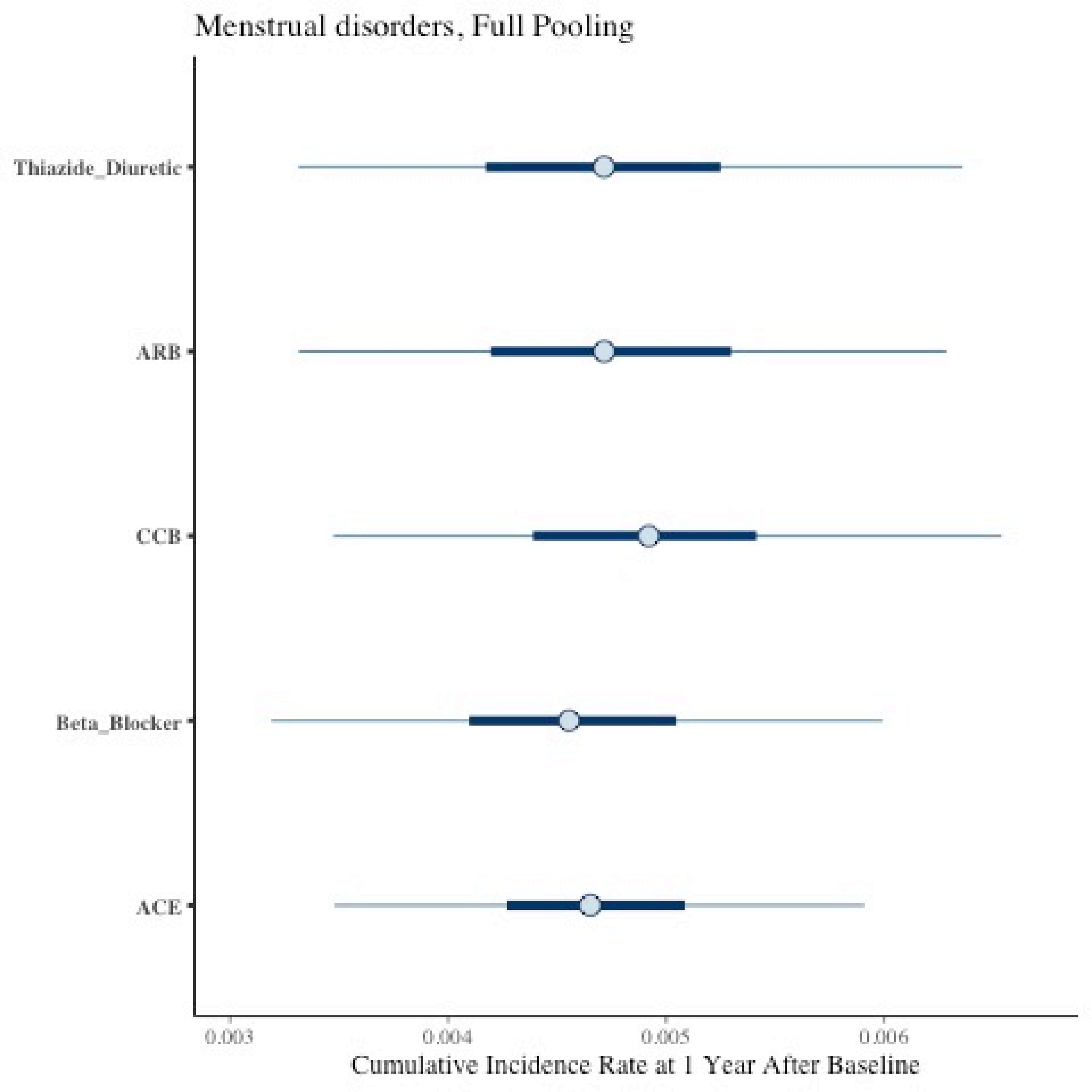

Menstrual disorders, Full Pooling
Thiazide_Diuretic
ARB
CCB
Beta_Blocker
ACE
0.003
0.004
0.005
0.006
Cumulative Incidence Rate at 1 Year After Baseline

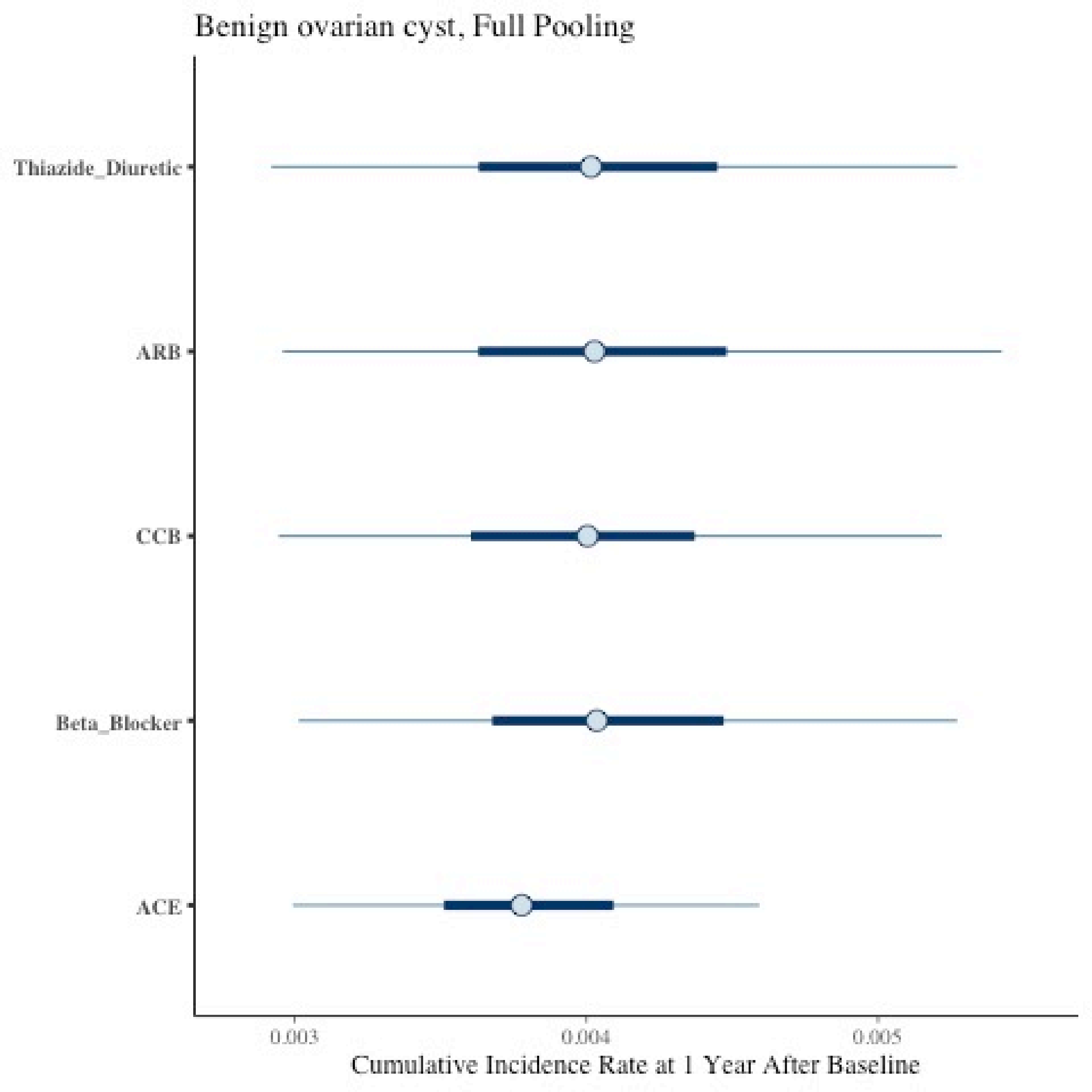

Benign ovarian cyst, Full Pooling
Thiazide_Diuretic
ARB
CCB
Beta_Blocker
ACE
0.003
0.004
0.005
Cumulative Incidence Rate at 1 Year After Baseline

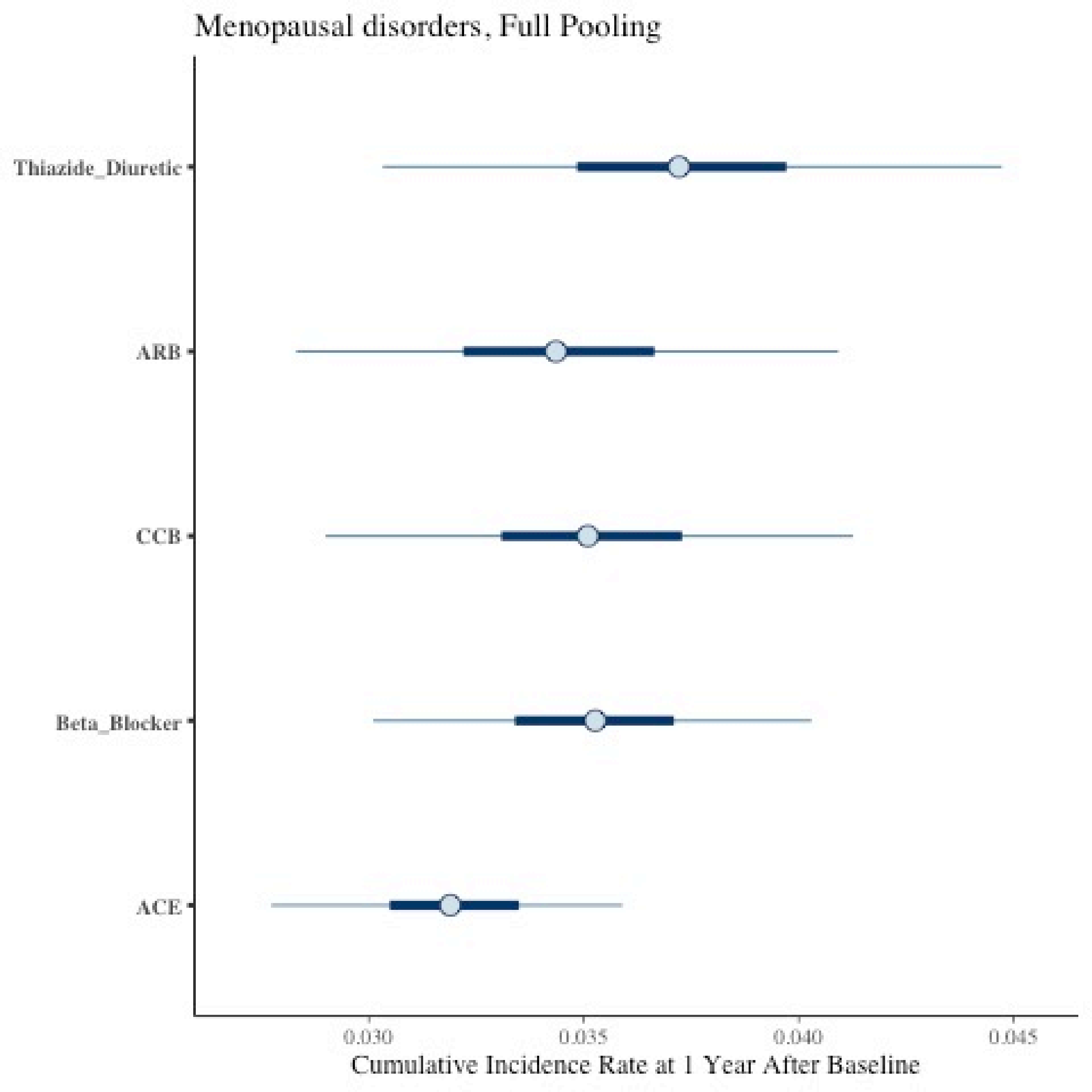

Menopausal disorders, Full Pooling
Thiazide_Diuretic
ARB
CCB
Beta_Blocker
ACE
0.030
0.035
0.040
0.045
Cumulative Incidence Rate at 1 Year After Baseline

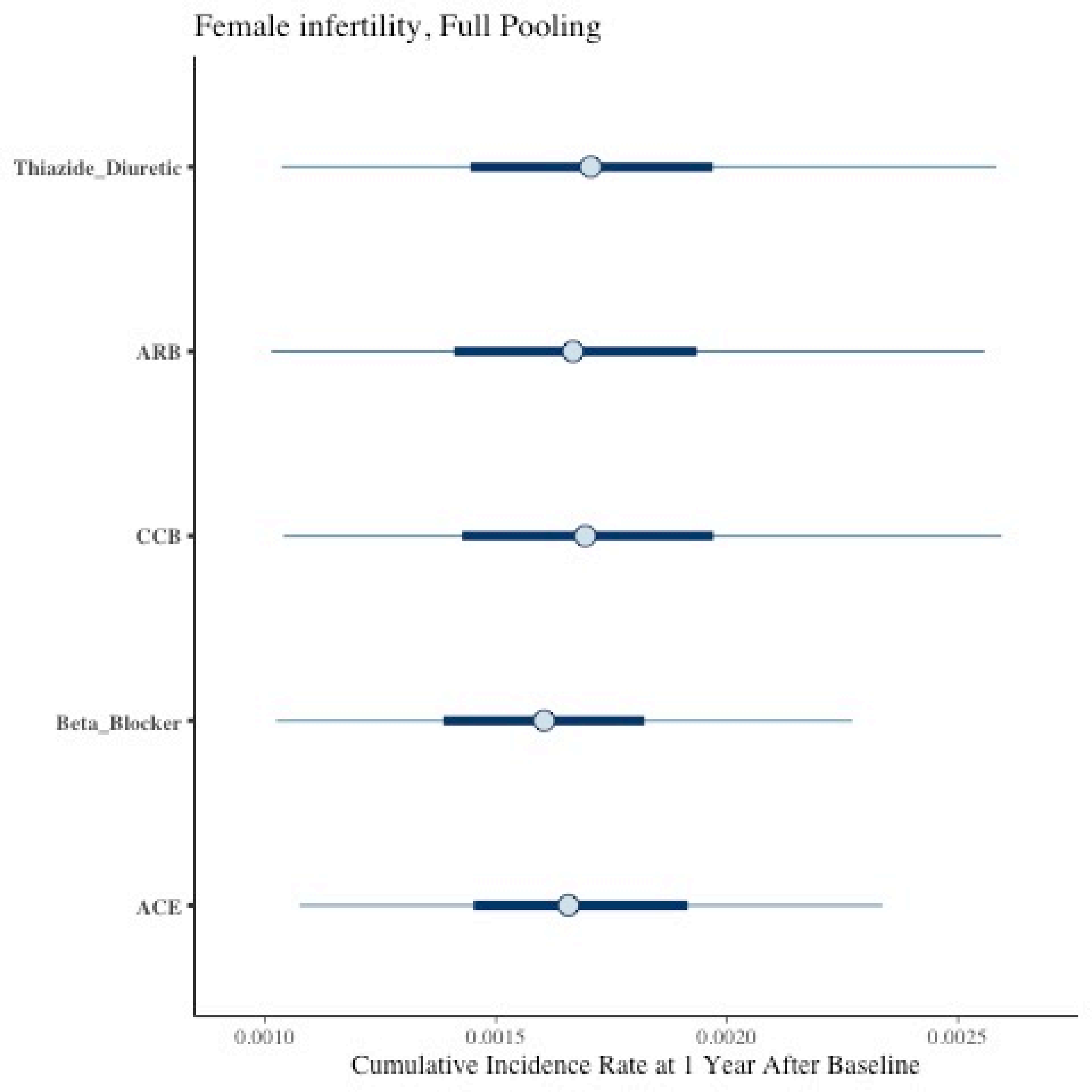
Female infertility, Full Pooling
Thiazide_Diuretic
ARB
CCB
Beta_Blocker
ACE
0.0010
0.0015
0.0020
0.0025
Cumulative Incidence Rate at 1 Year After Baseline

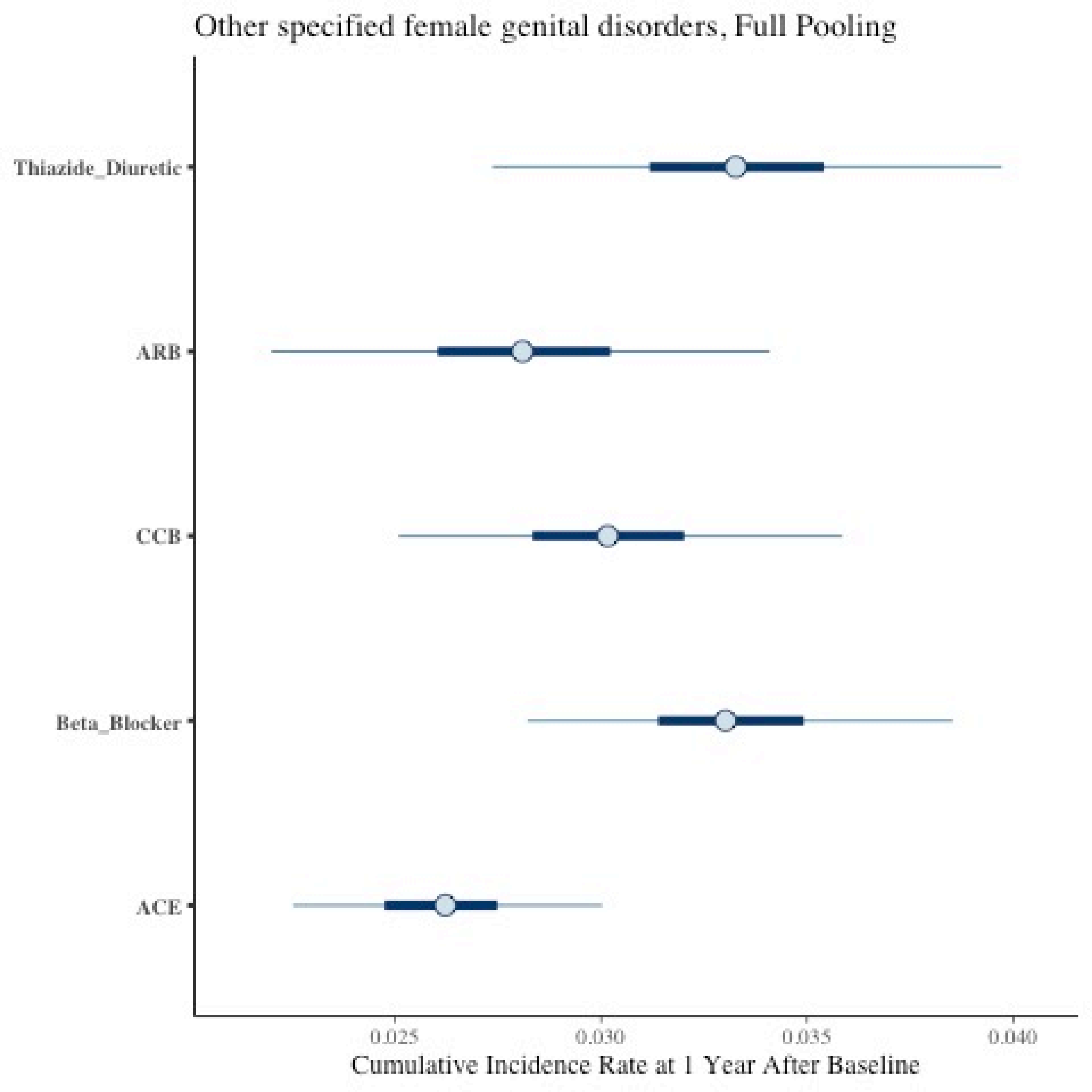

Other specified female genital disorders, Full Pooling
Thiazide_Diuretic
ARB
CCB
Beta_Blocker
ACE
0.025
0.030
0.035
0.040
Cumulative Incidence Rate at 1 Year After Baseline

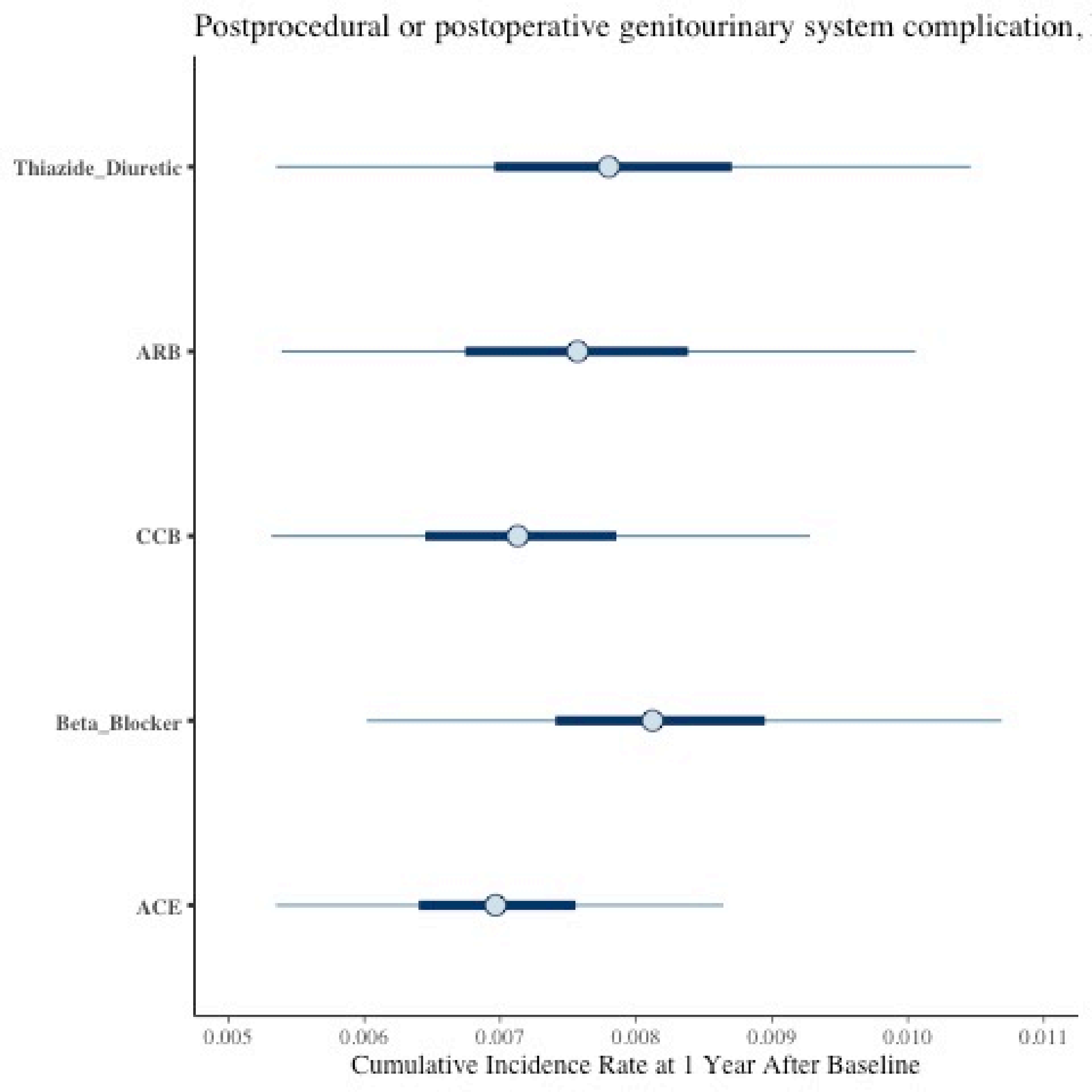

Postprocedural or postoperative genitourinary system complication,
Thiazide_Diuretic
ARB
CCB
Beta_Blocker
ACE
0.005
0.006
0.007
0.008
0.009
0.010
0.011
Cumulative Incidence Rate at 1 Year After Baseline

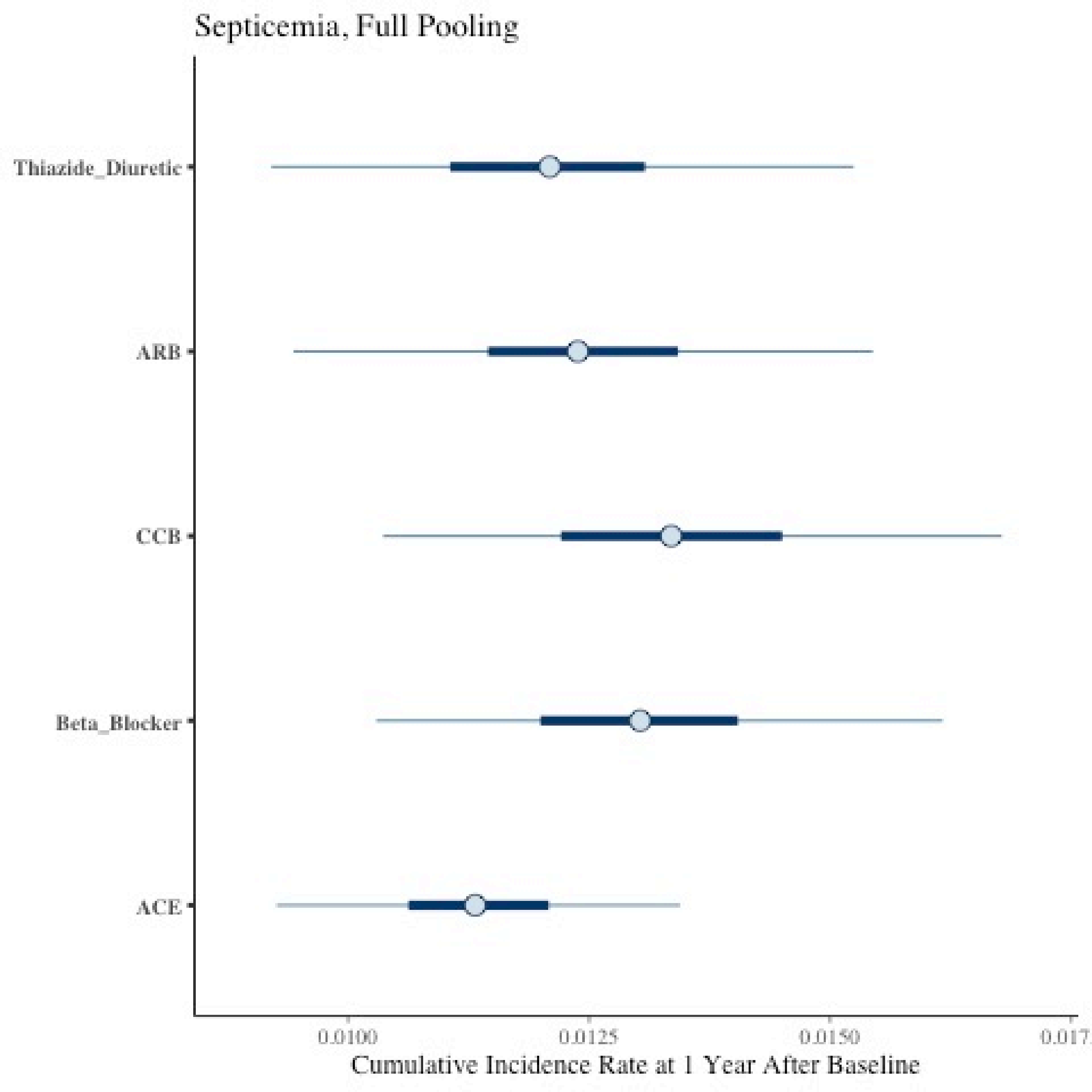
Septicemia, Full Pooling
Thiazide_Diuretic
ARB
CCB
Beta_Blocker
ACE
0.0100
0.0125
0.0150
0.017
Cumulative Incidence Rate at 1 Year After Baseline

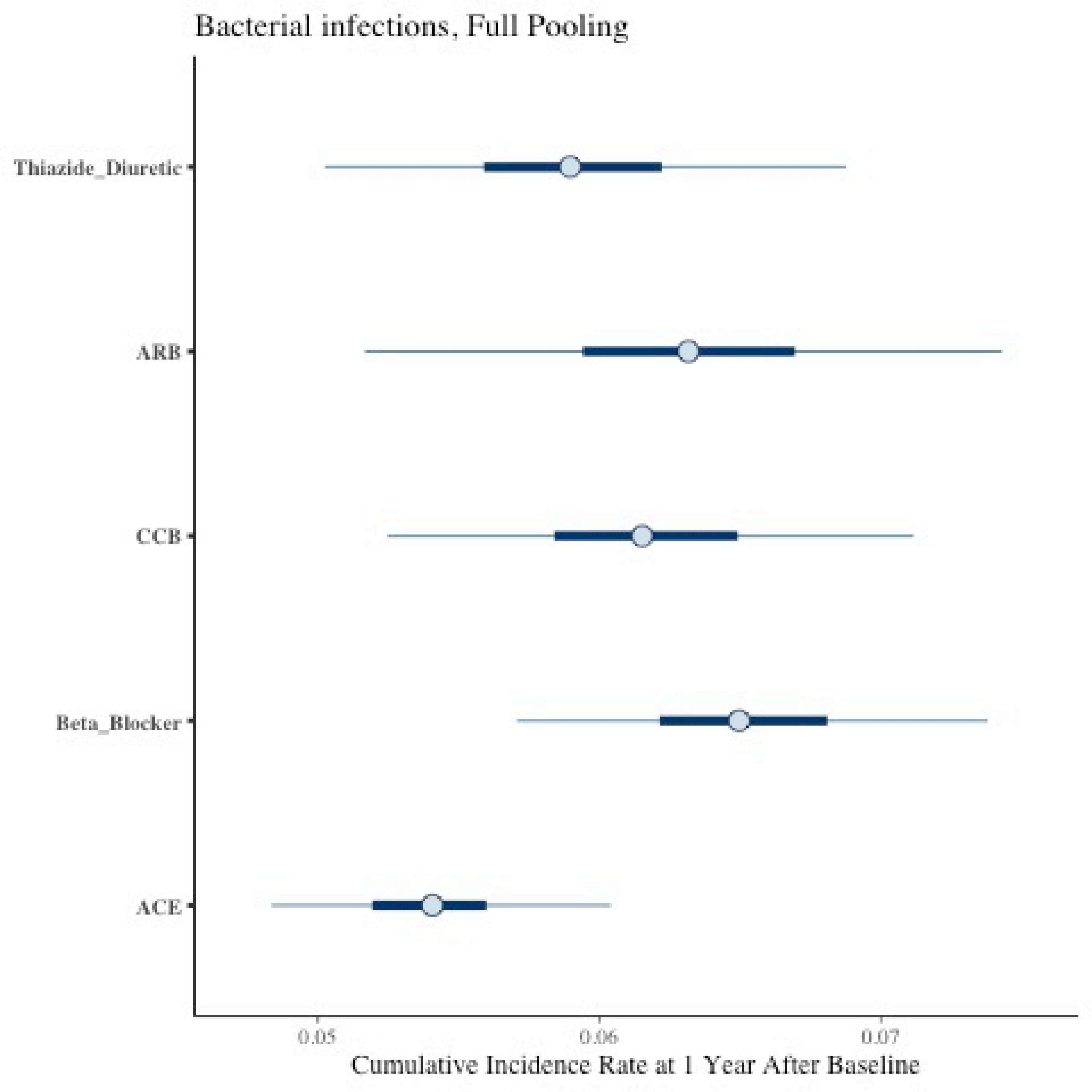

Bacterial infections, Full Pooling
Thiazide_Diuretic
ARB
CCB
Beta_Blocker
ACE
0.05
0.06
0.07
Cumulative Incidence Rate at 1 Year After Baseline

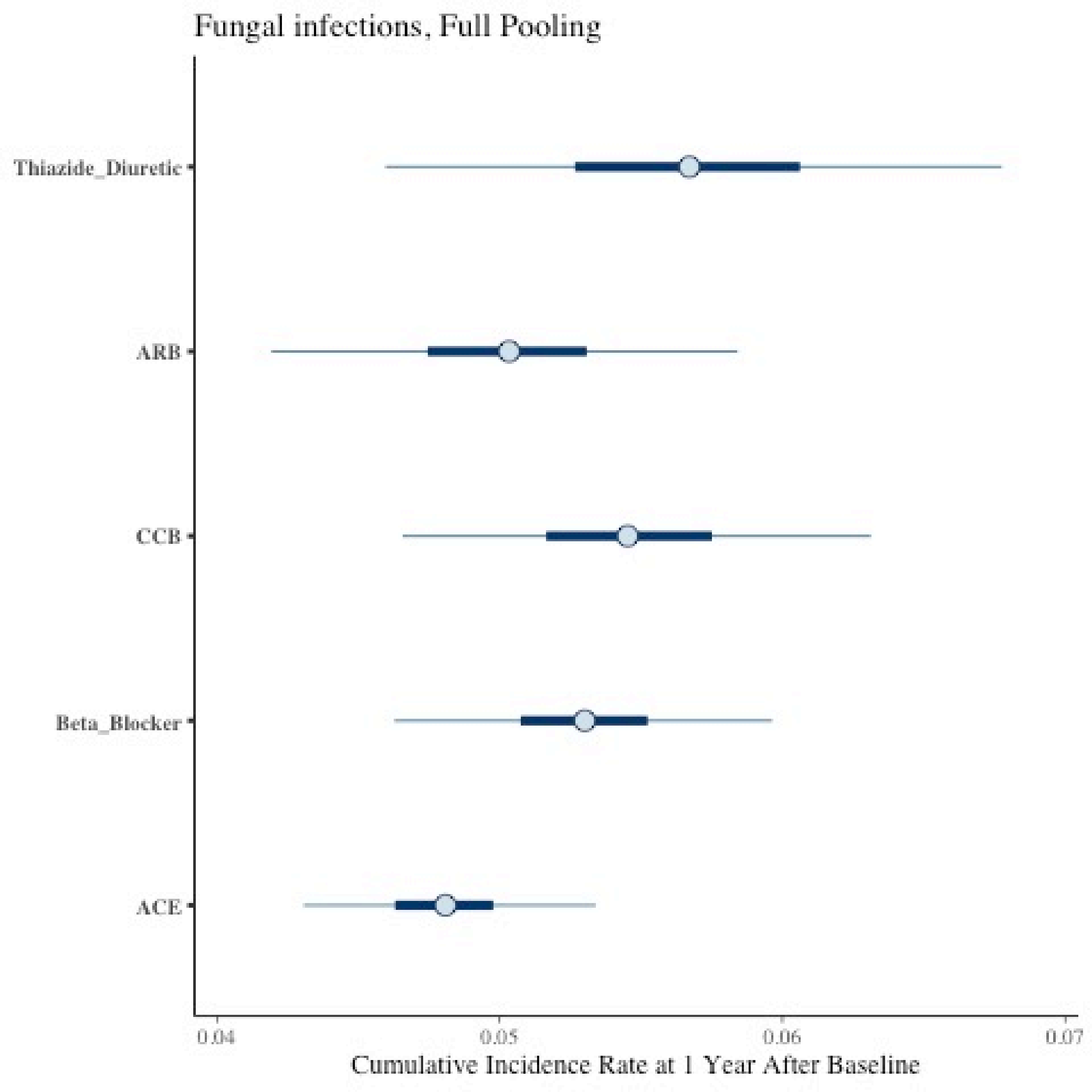

Fungal infections, Full Pooling
Thiazide_Diuretic
ARB
CCB
Beta_Blocker
ACE
0.04
0.05
0.06
0.07
Cumulative Incidence Rate at 1 Year After Baseline

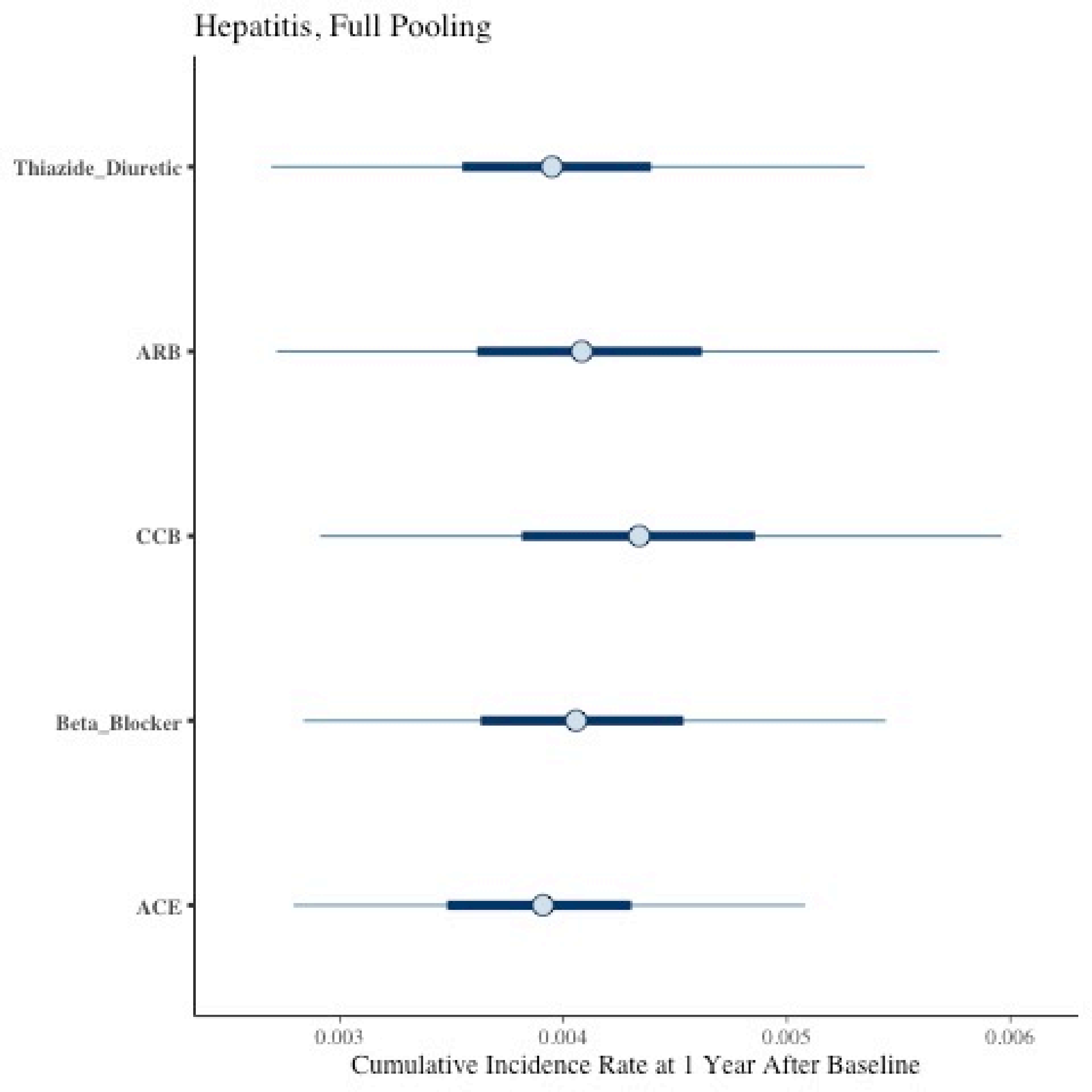
Hepatitis, Full Pooling
Thiazide_Diuretic
ARB
CCB
Beta_Blocker
ACE
0.003
0.004
0.005
0.006
Cumulative Incidence Rate at 1 Year After Baseline

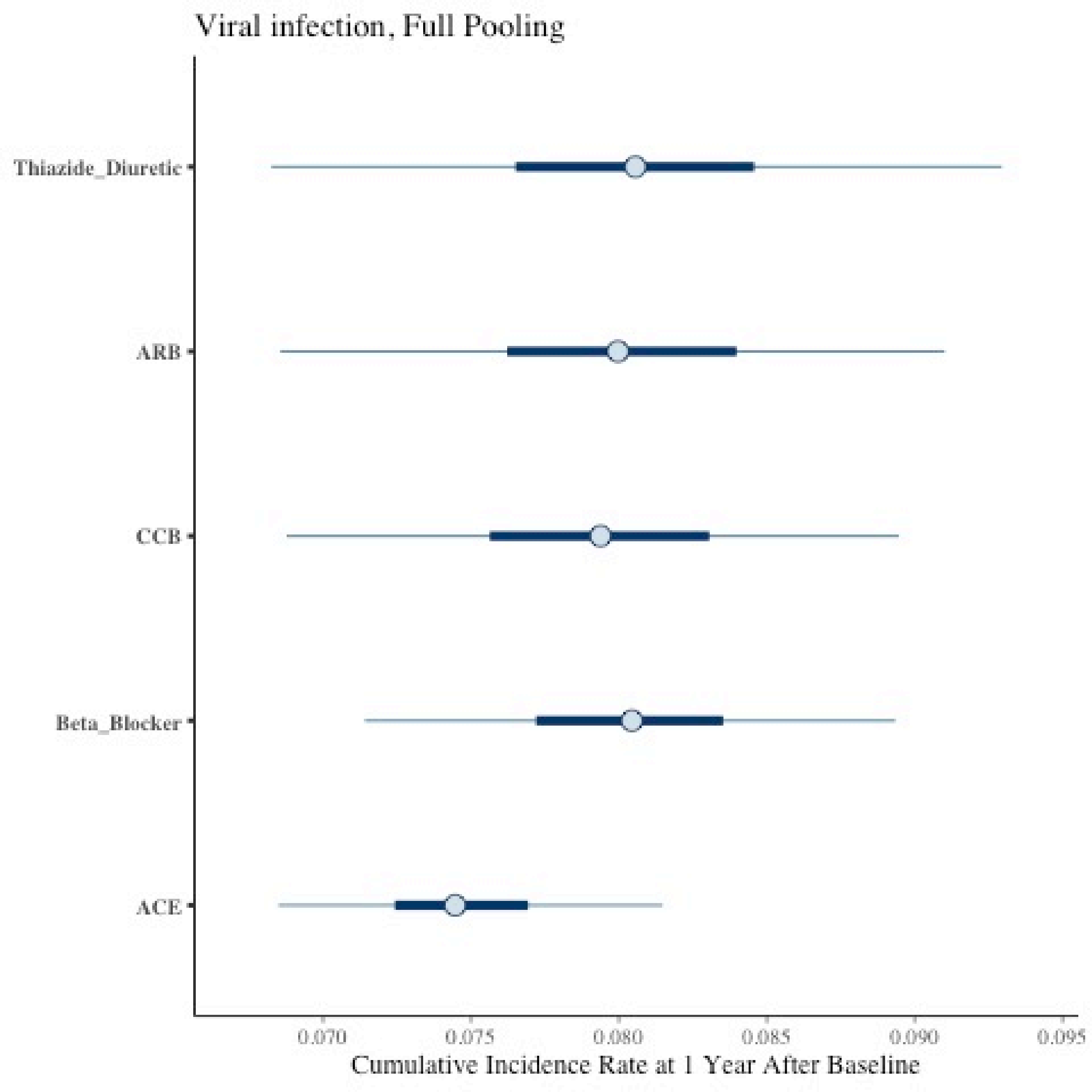

Viral infection, Full Pooling
Thiazide_Diuretic
ARB
CCB
Beta_Blocker
ACE
0.070
0.075
0.080
0.085
0.090
0.095
Cumulative Incidence Rate at 1 Year After Baseline

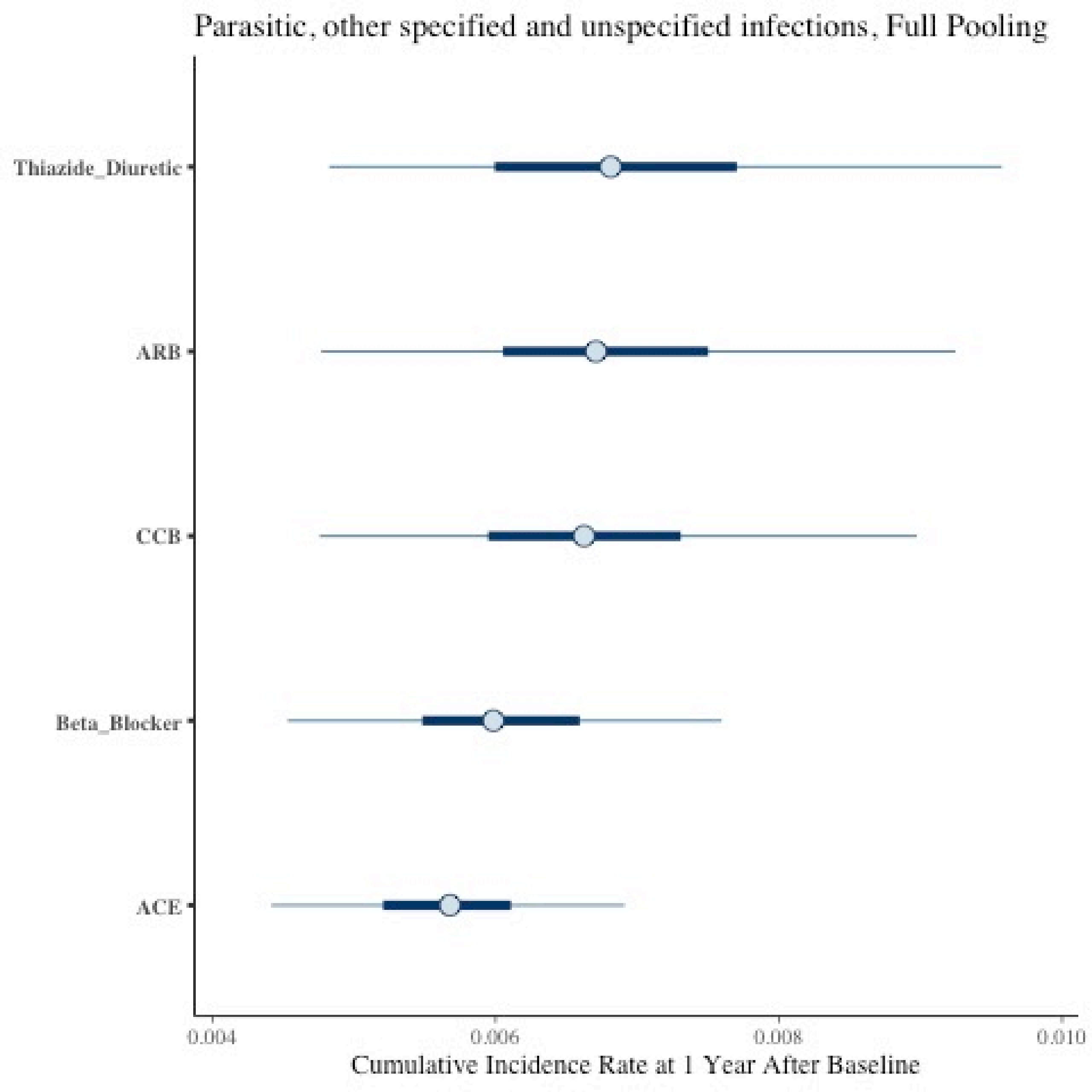

Parasitic, other specified and unspecified infections, Full Pooling
Thiazide_Diuretic
ARB
CCB
Beta_Blocker
ACE
0.004
0.006
0.008
0.010
Cumulative Incidence Rate at 1 Year After Baseline

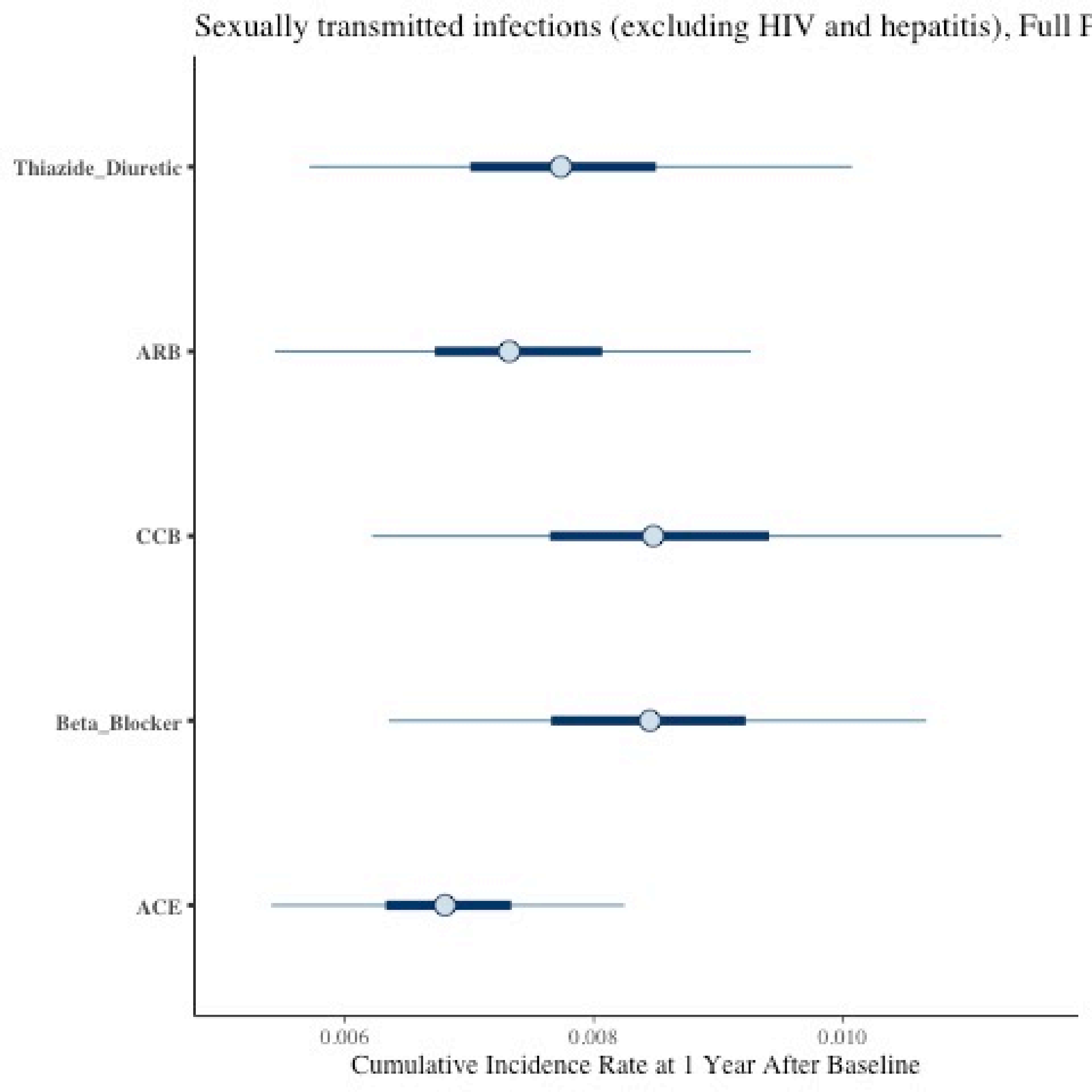
Sexually transmitted infections (excluding HIV and hepatitis), Full P
Thiazide_Diuretic
ARB
CCB
Beta_Blocker
ACE
0.006
0.008
0.010
Cumulative Incidence Rate at 1 Year After Baseline

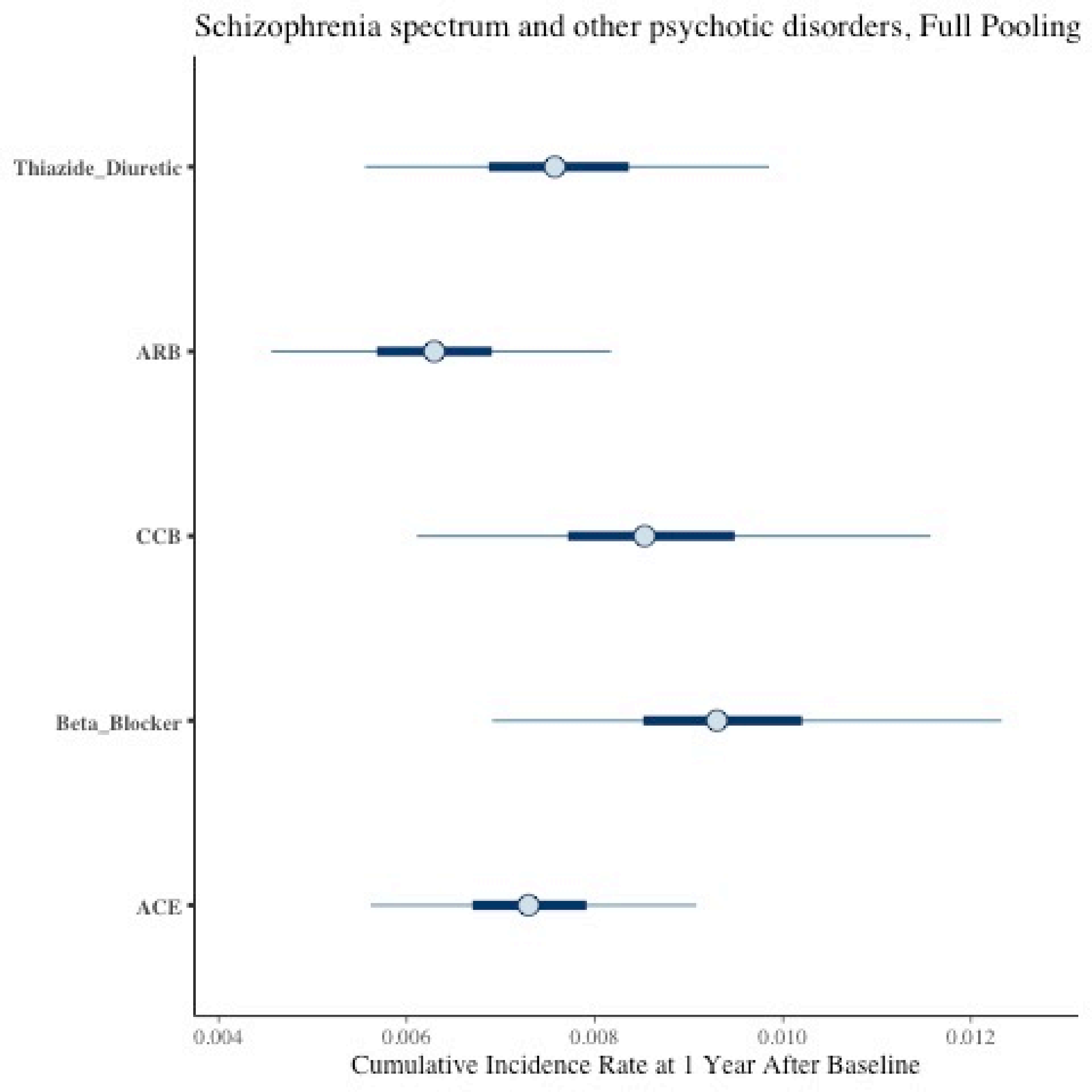

Schizophrenia spectrum and other psychotic disorders, Full Pooling
Thiazide_Diuretic
ARB
CCB
Beta_Blocker
ACE
0.004
0.006
0.008
0.010
0.012
Cumulative Incidence Rate at 1 Year After Baseline

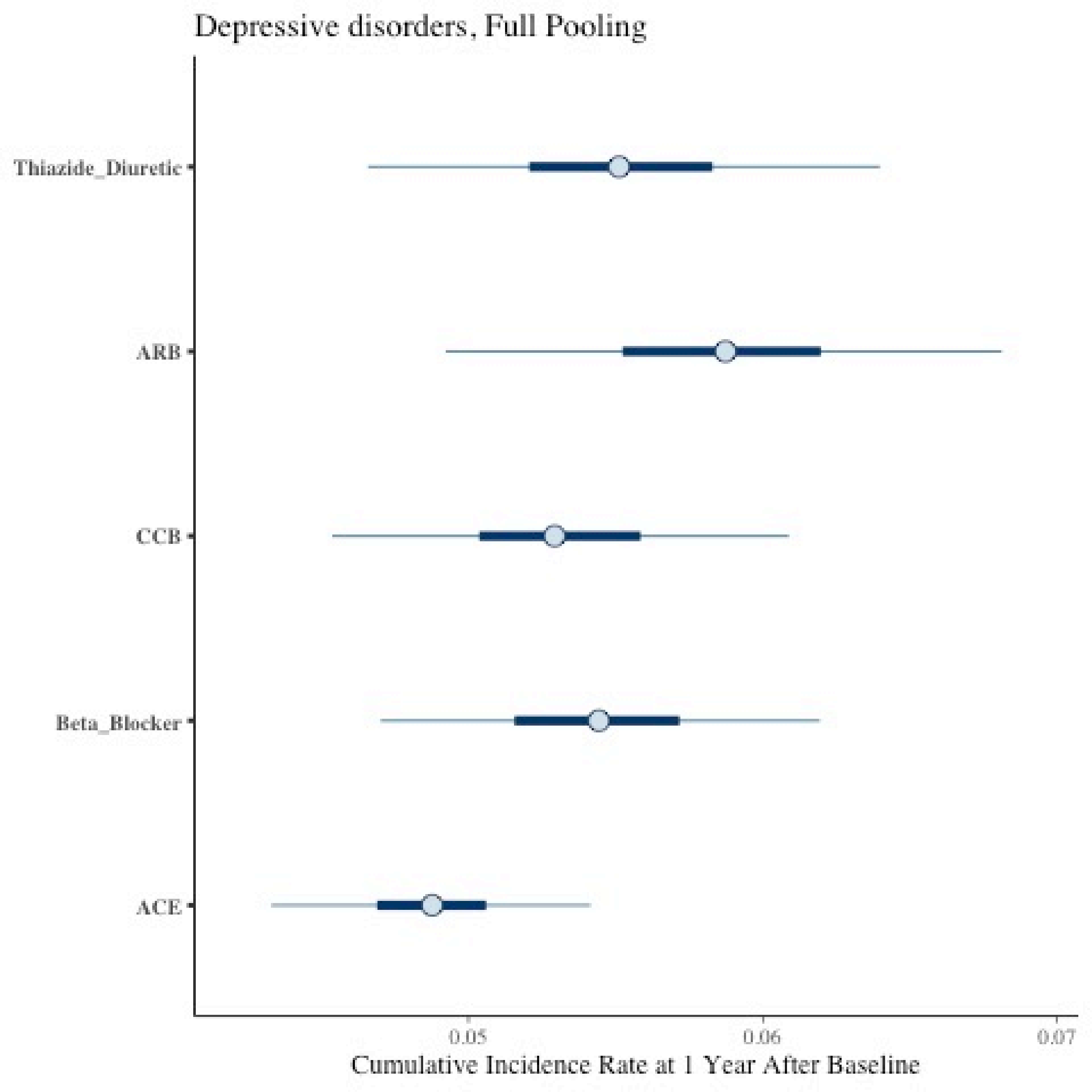

Depressive disorders, Full Pooling
Thiazide_Diuretic
ARB
CCB
Beta_Blocker
ACE
0.05
0.06
0.07
Cumulative Incidence Rate at 1 Year After Baseline

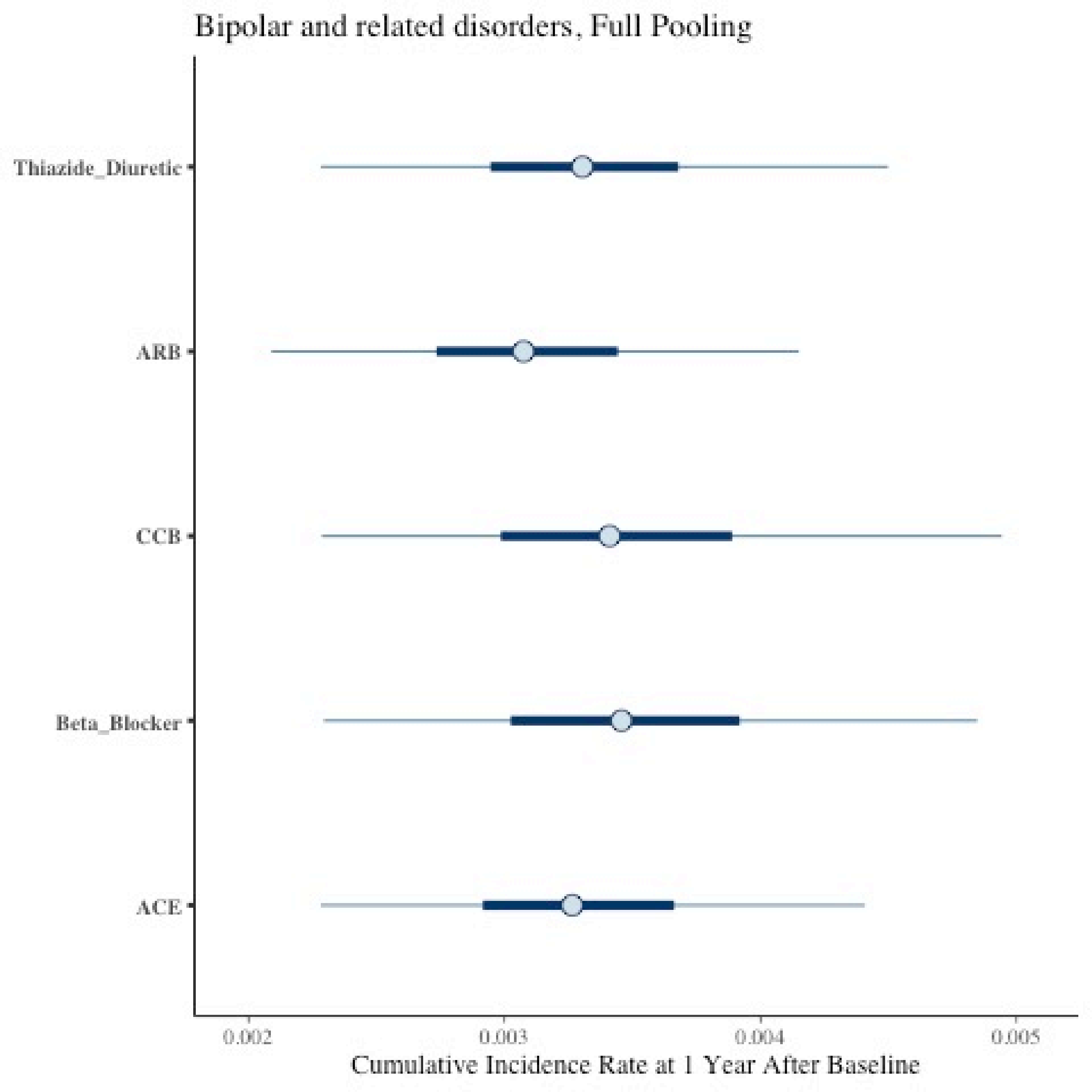

Bipolar and related disorders, Full Pooling
Thiazide_Diuretic
ARB
CCB
Beta_Blocker
ACE
0.002
0.003
0.004
0.005
Cumulative Incidence Rate at 1 Year After Baseline

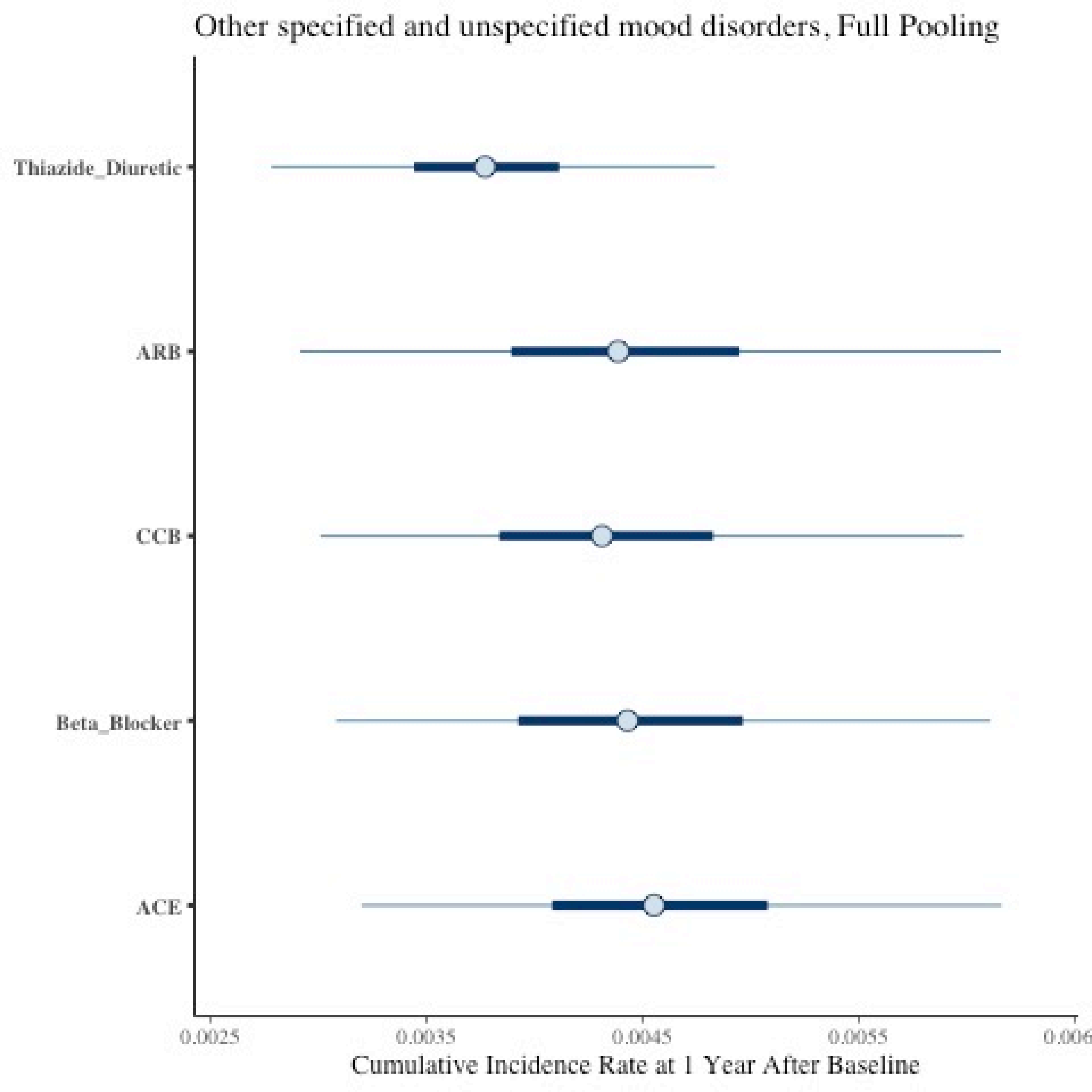
Other specified and unspecified mood disorders, Full Pooling
Thiazide_Diuretic
ARB
CCB
Beta_Blocker
ACE
0.0025
0.0035
0.0045
0.0055
0.006
Cumulative Incidence Rate at 1 Year After Baseline

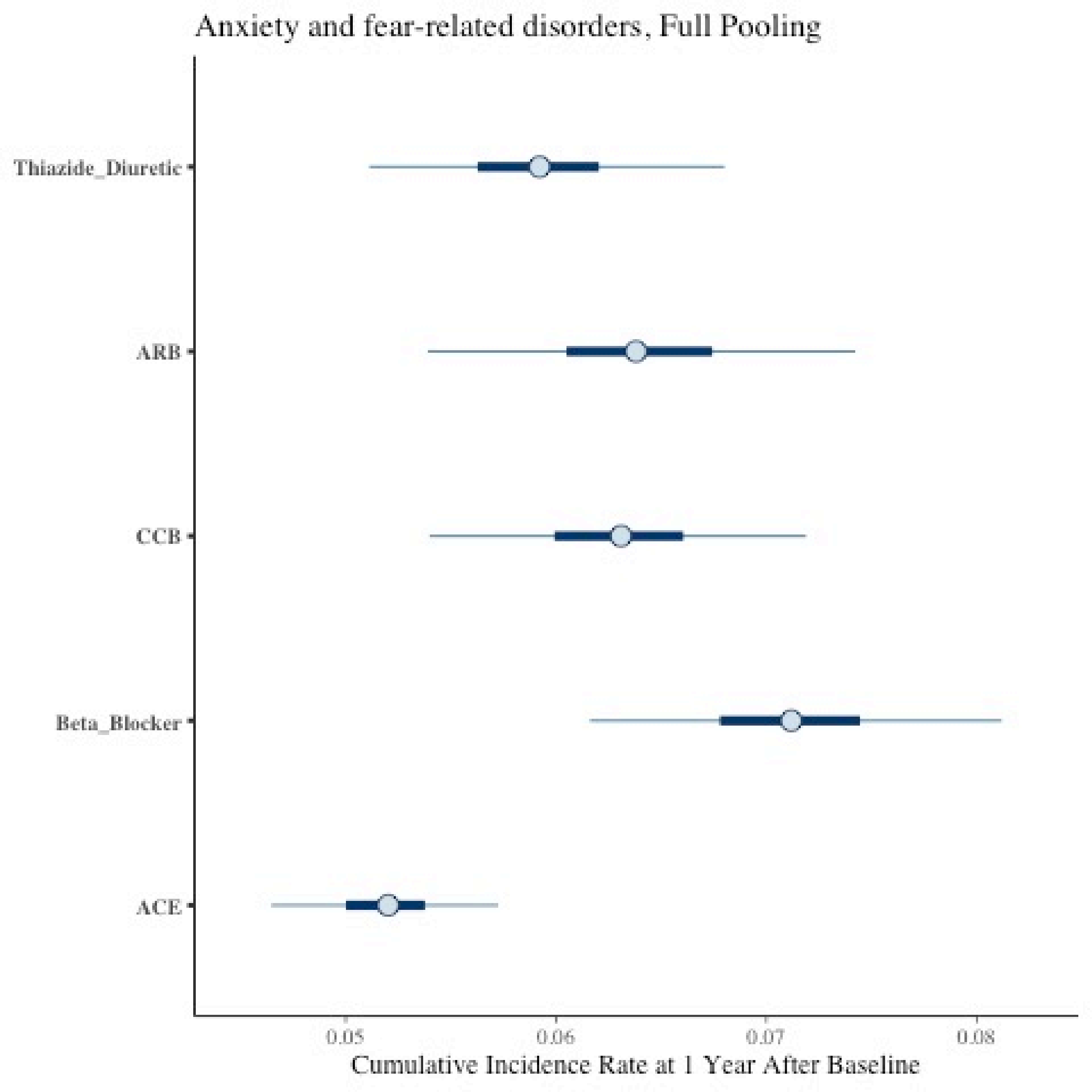

Anxiety and fear-related disorders, Full Pooling
Thiazide_Diuretic
ARB
CCB
Beta_Blocker
ACE
0.05
0.06
0.07
0.08
Cumulative Incidence Rate at 1 Year After Baseline

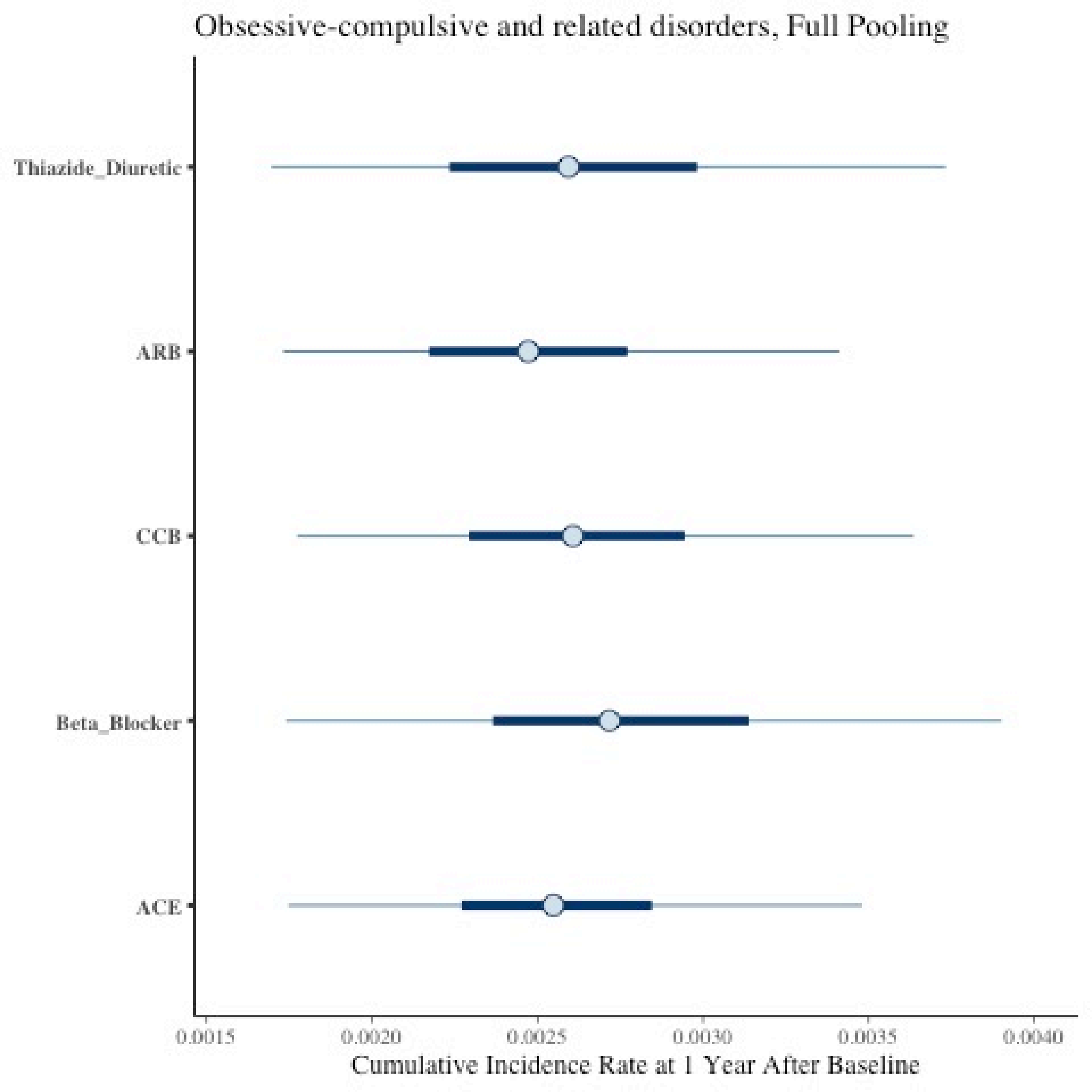
Obsessive-compulsive and related disorders, Full Pooling
Thiazide_Diuretic
ARB
CCB
Beta_Blocker
ACE
0.0015
0.0020
0.0025
0.0030
0.0035
0.0040
Cumulative Incidence Rate at 1 Year After Baseline

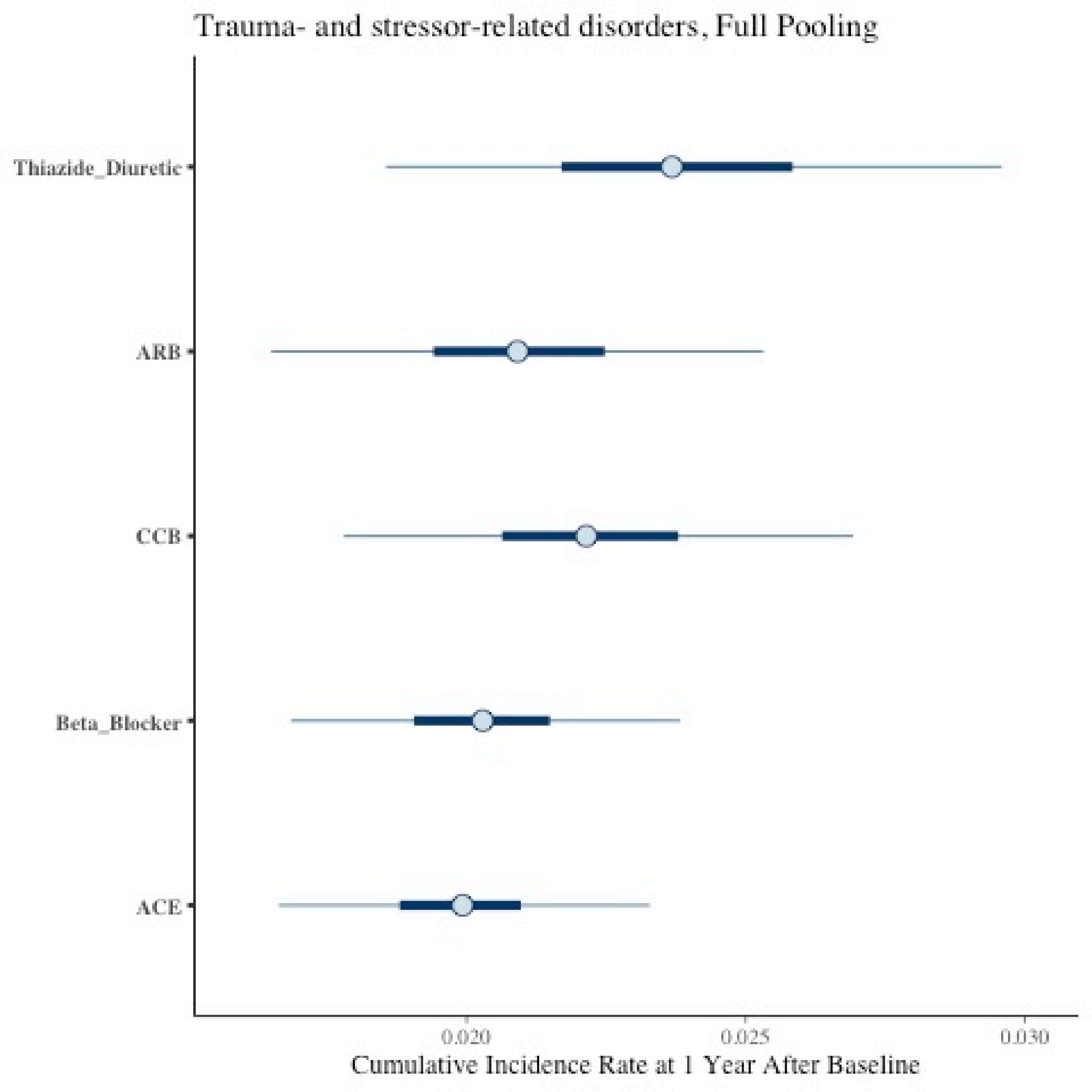

Trauma- and stressor-related disorders, Full Pooling
Thiazide_Diuretic
ARB
CCB
Beta_Blocker
ACE
0.020
0.025
0.030
Cumulative Incidence Rate at 1 Year After Baseline

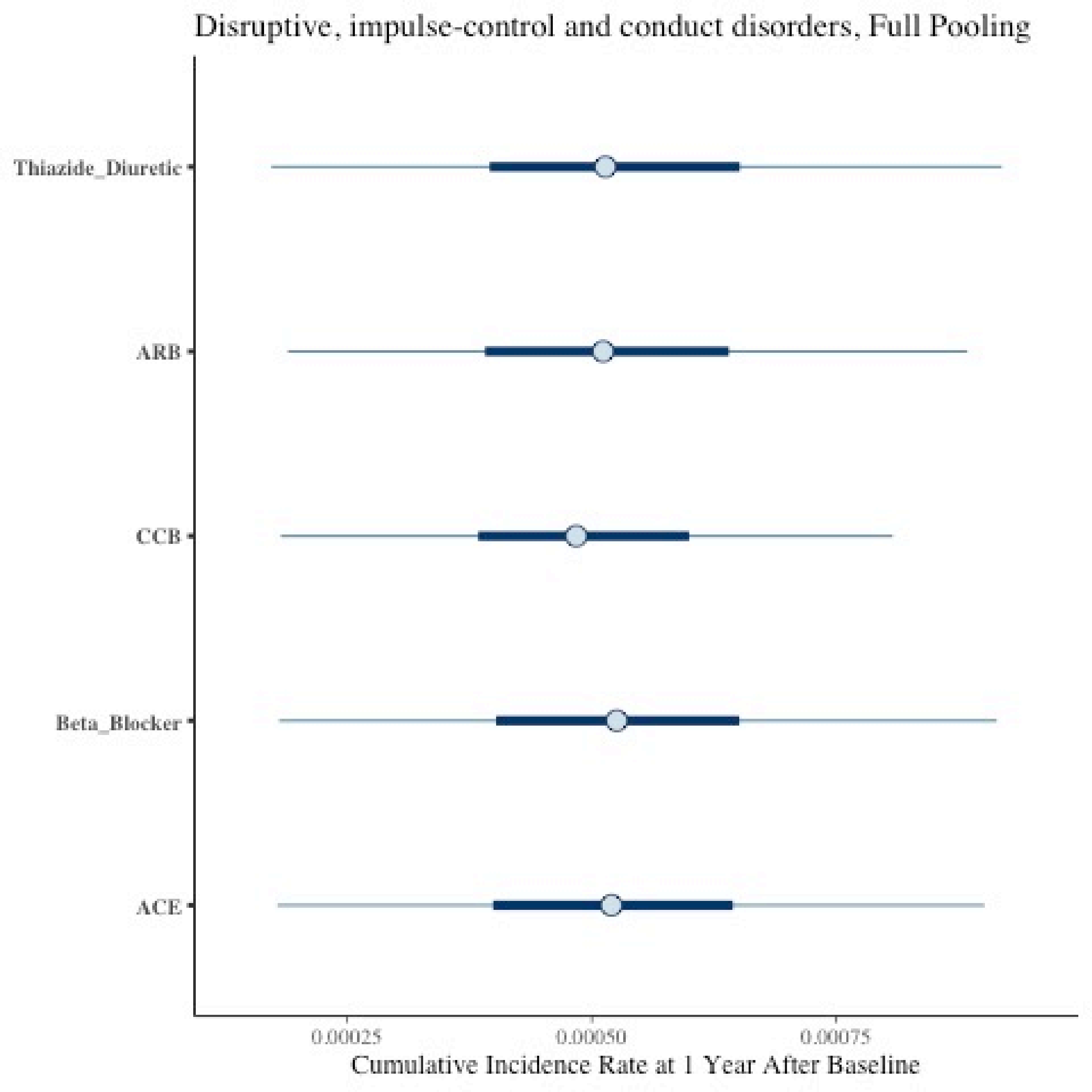

Disruptive, impulse-control and conduct disorders, Full Pooling
Thiazide_Diuretic
ARB
CCB
Beta_Blocker
ACE
0.00025
0.00050
0.00075
Cumulative Incidence Rate at 1 Year After Baseline

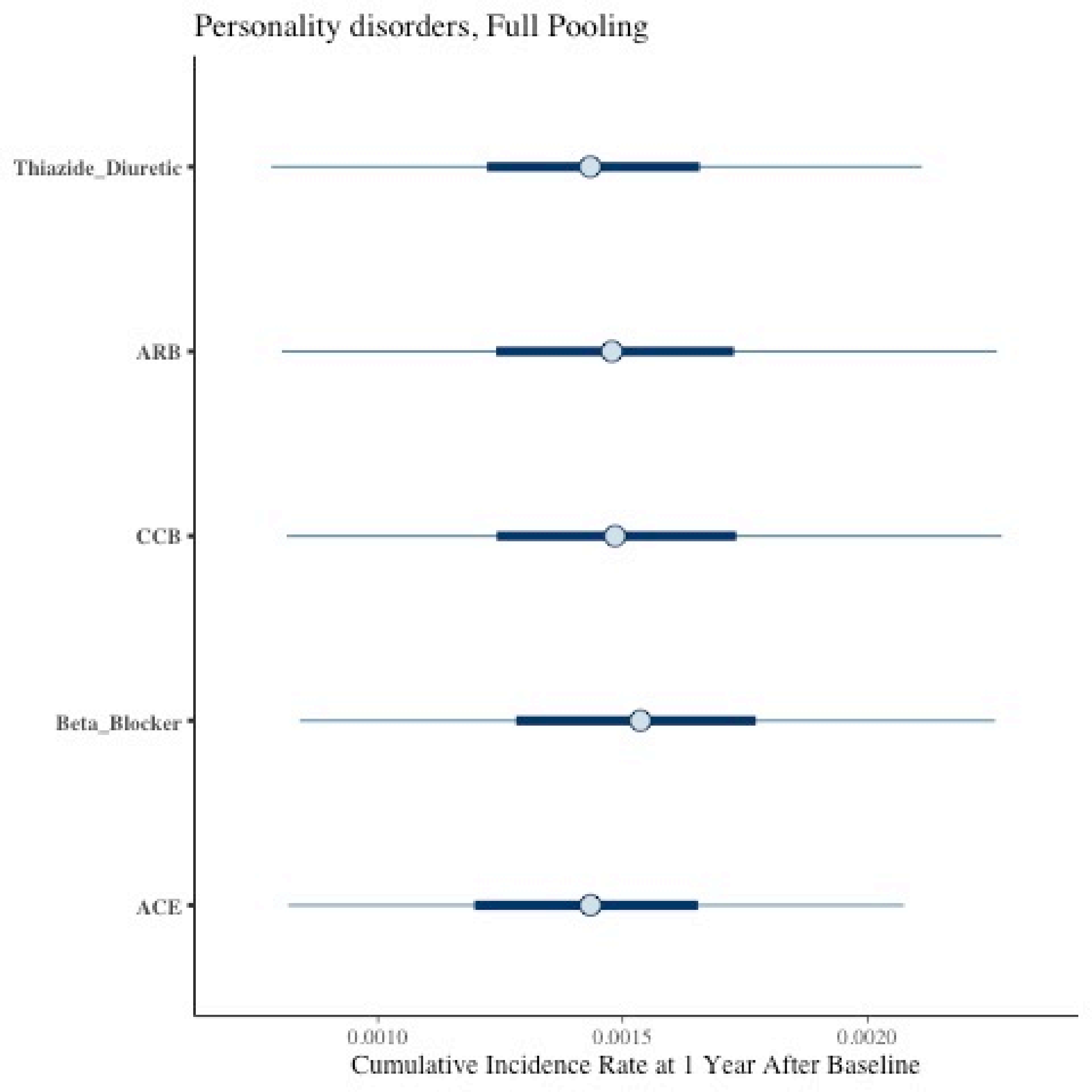

Personality disorders, Full Pooling
Thiazide_Diuretic
ARB
CCB
Beta_Blocker
ACE
0.0010
0.0015
0.0020
Cumulative Incidence Rate at 1 Year After Baseline

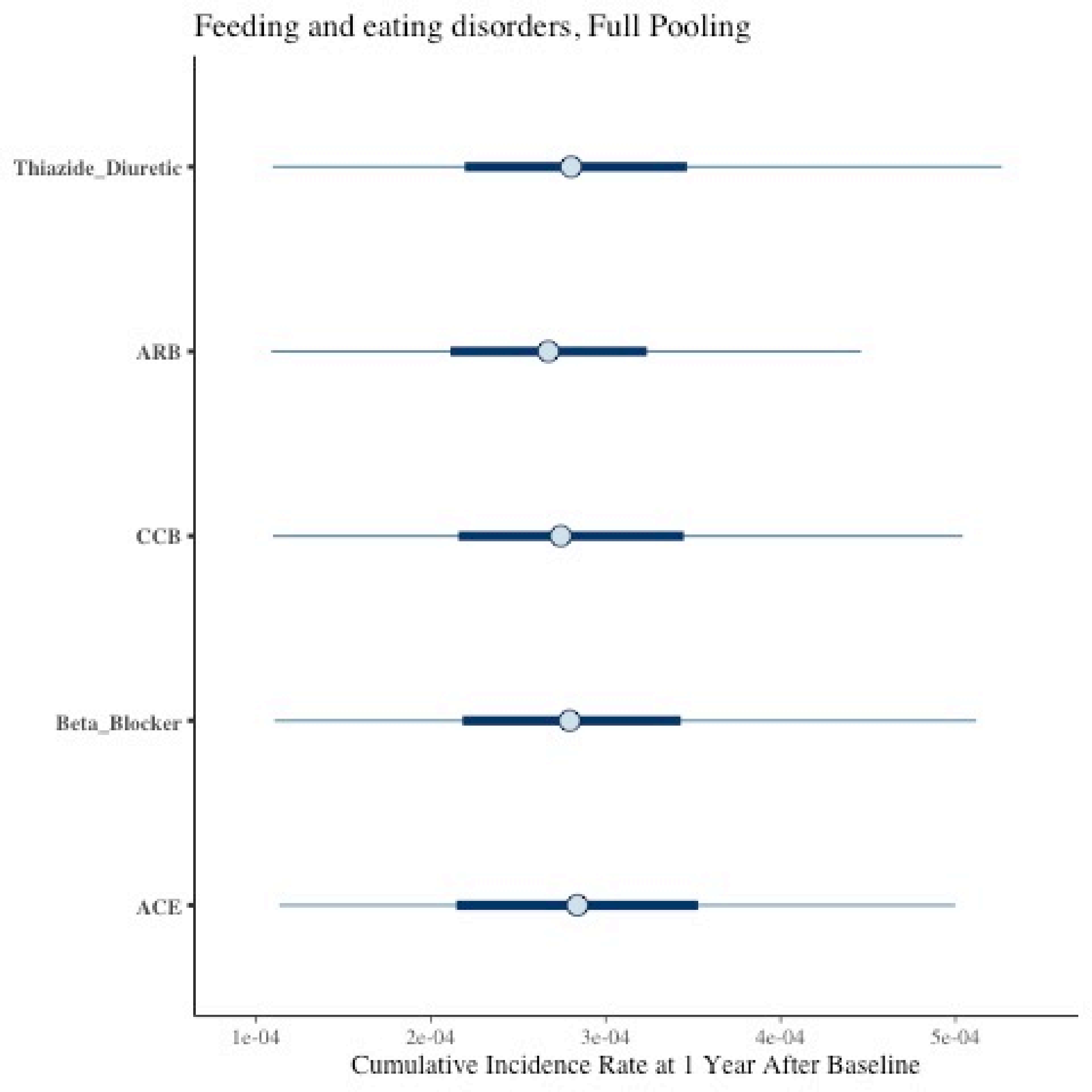
Feeding and eating disorders, Full Pooling
Thiazide_Diuretic
ARB
CCB
Beta_Blocker
ACE
1e-04
2e-04
3e-04
4e-04
5e-04
Cumulative Incidence Rate at 1 Year After Baseline

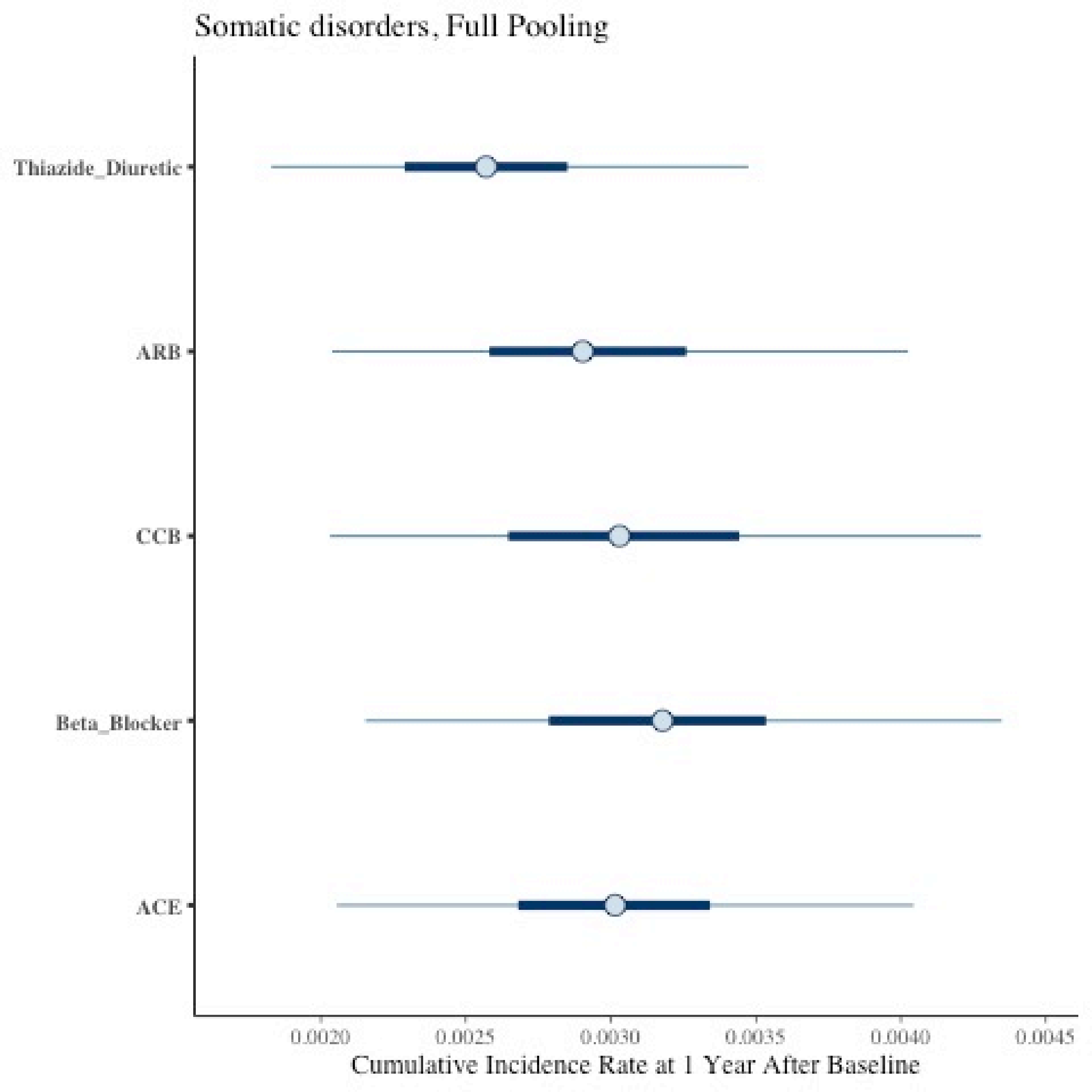

Somatic disorders, Full Pooling
Thiazide_Diuretic
ARB
CCB
Beta_Blocker
ACE
0.0020
0.0025
0.0030
0.0035
0.0040
0.0045
Cumulative Incidence Rate at 1 Year After Baseline

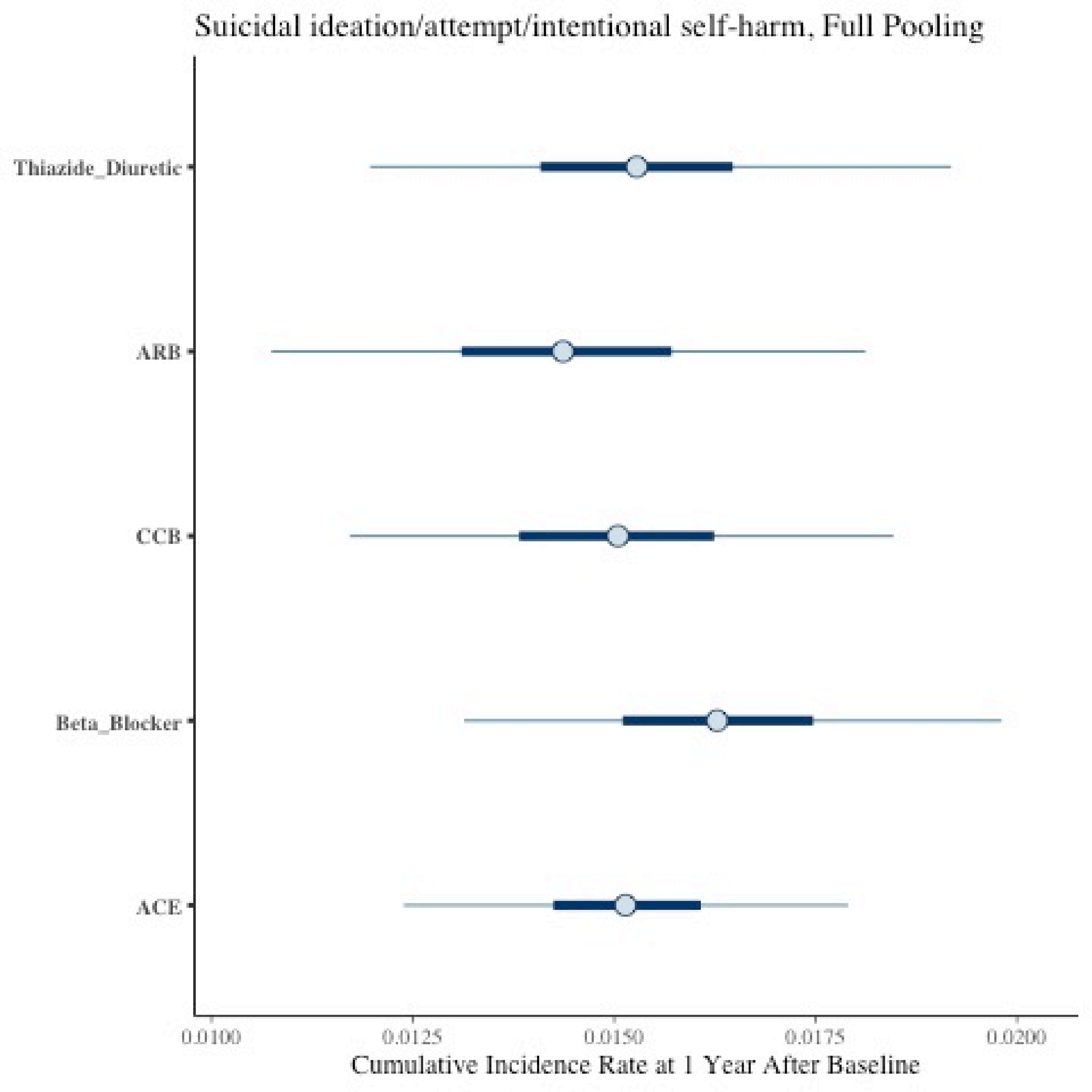
Suicidal ideation/attempt/intentional self-harm, Full Pooling
Thiazide_Diuretic
ARB
CCB
Beta_Blocker
ACE
0.0100
0.0125
0.0150
0.0175
0.0200
Cumulative Incidence Rate at 1 Year After Baseline

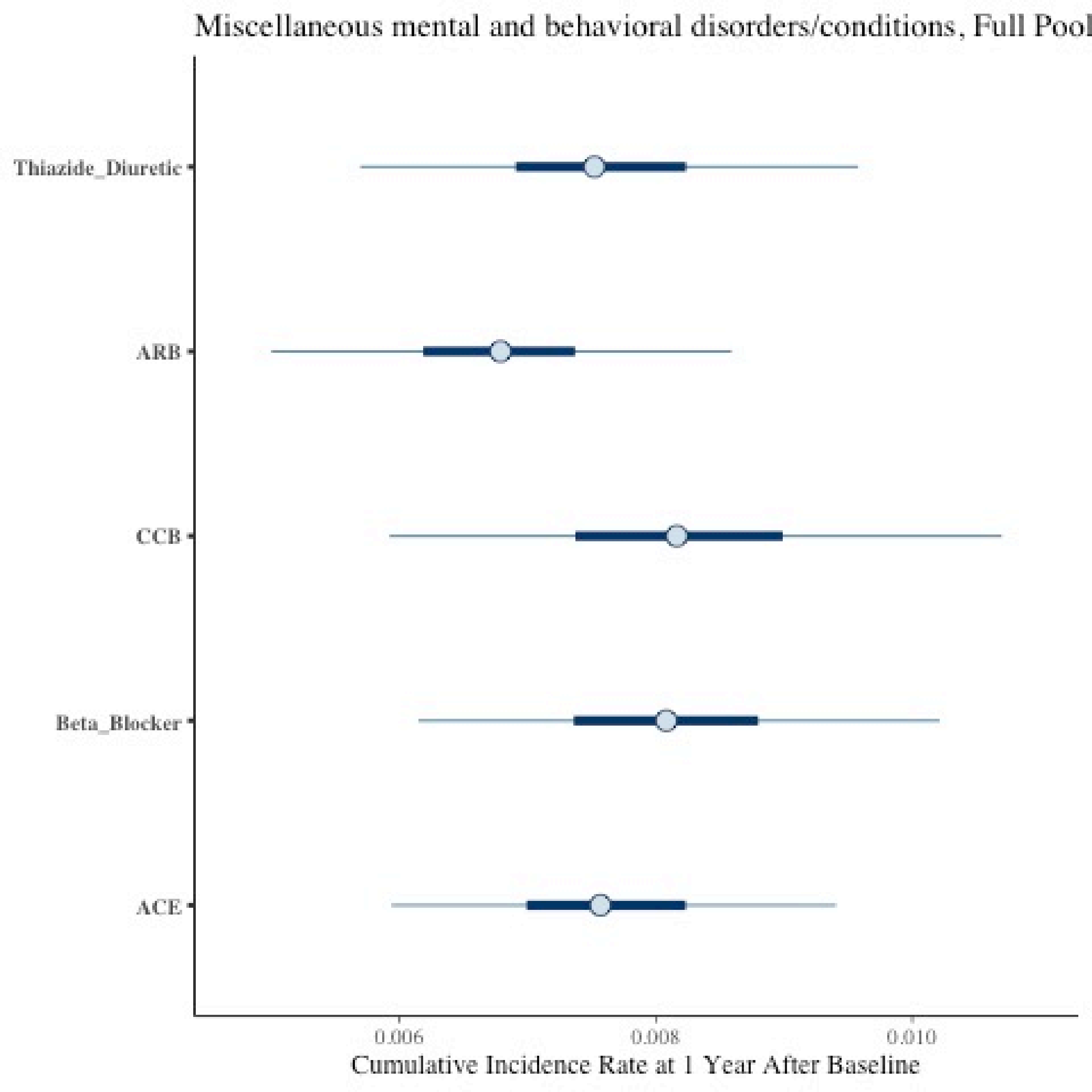

Miscellaneous mental and behavioral disorders/conditions, Full Pool
Thiazide_Diuretic
ARB
CCB
Beta_Blocker
ACE
0.006
0.008
0.010
Cumulative Incidence Rate at 1 Year After Baseline

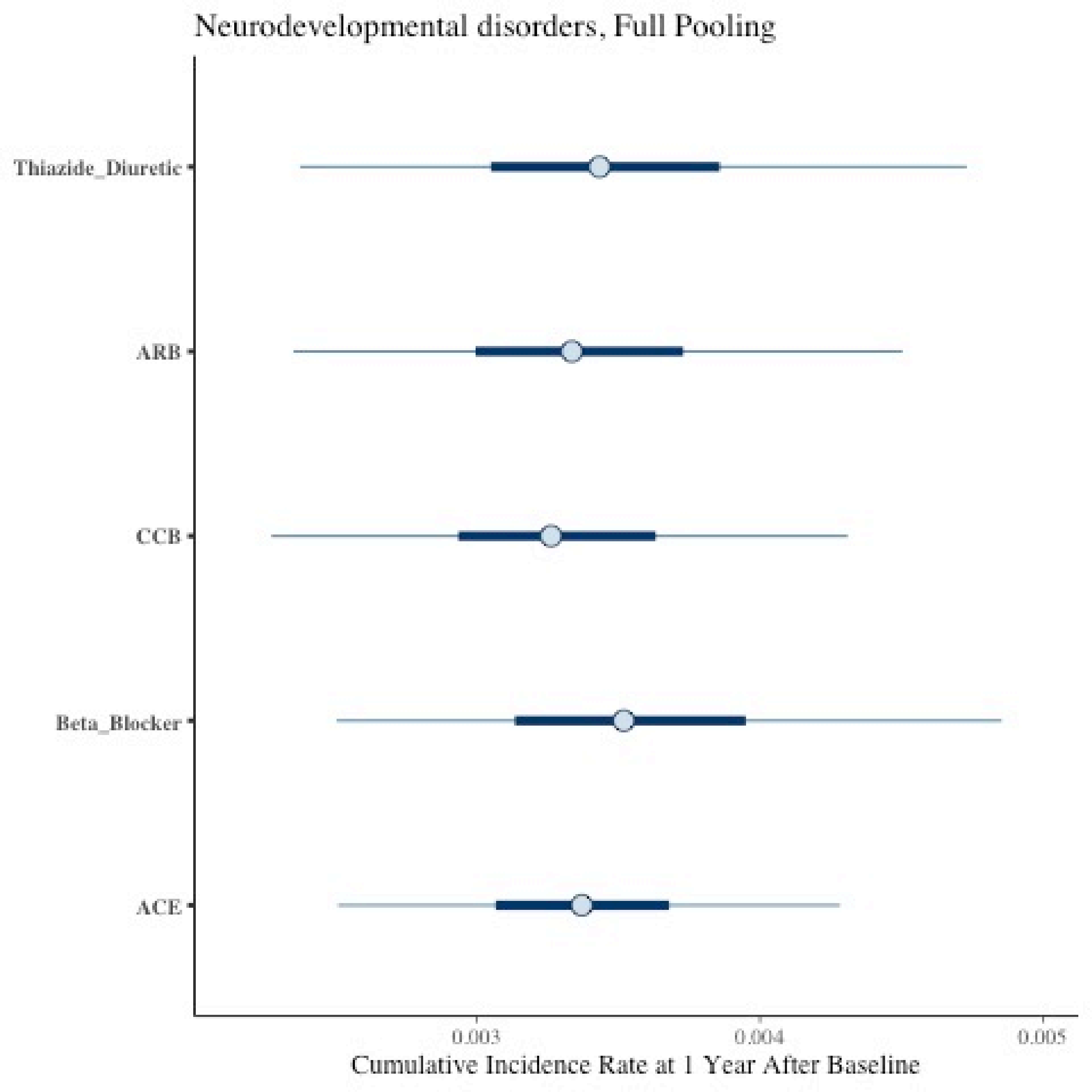

Neurodevelopmental disorders, Full Pooling
Thiazide_Diuretic
ARB
CCB
Beta_Blocker
ACE
0.003
0.004
0.005
Cumulative Incidence Rate at 1 Year After Baseline

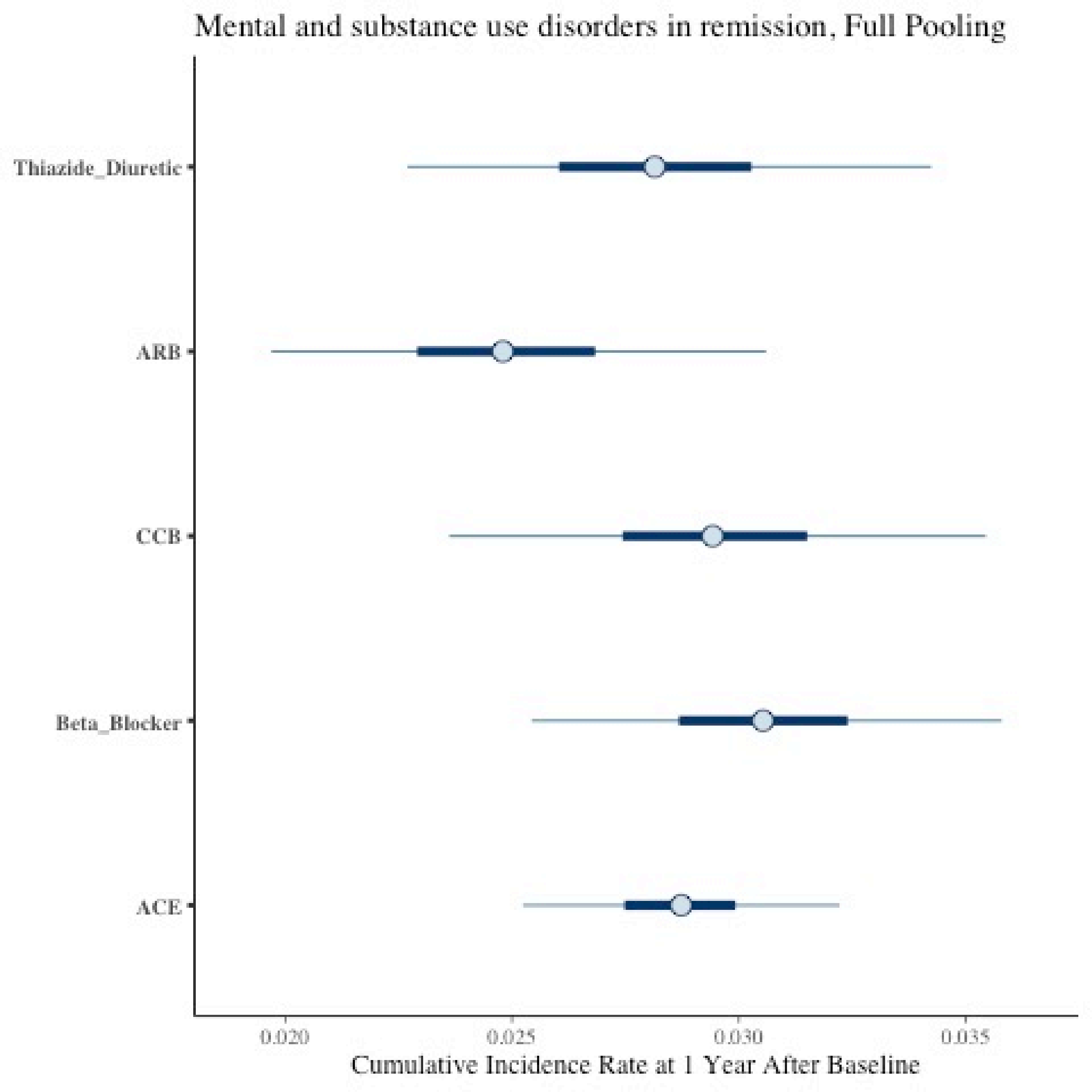

Mental and substance use disorders in remission, Full Pooling
Thiazide_Diuretic
ARB
CCB
Beta_Blocker
ACE
0.020
0.025
0.030
0.035
Cumulative Incidence Rate at 1 Year After Baseline

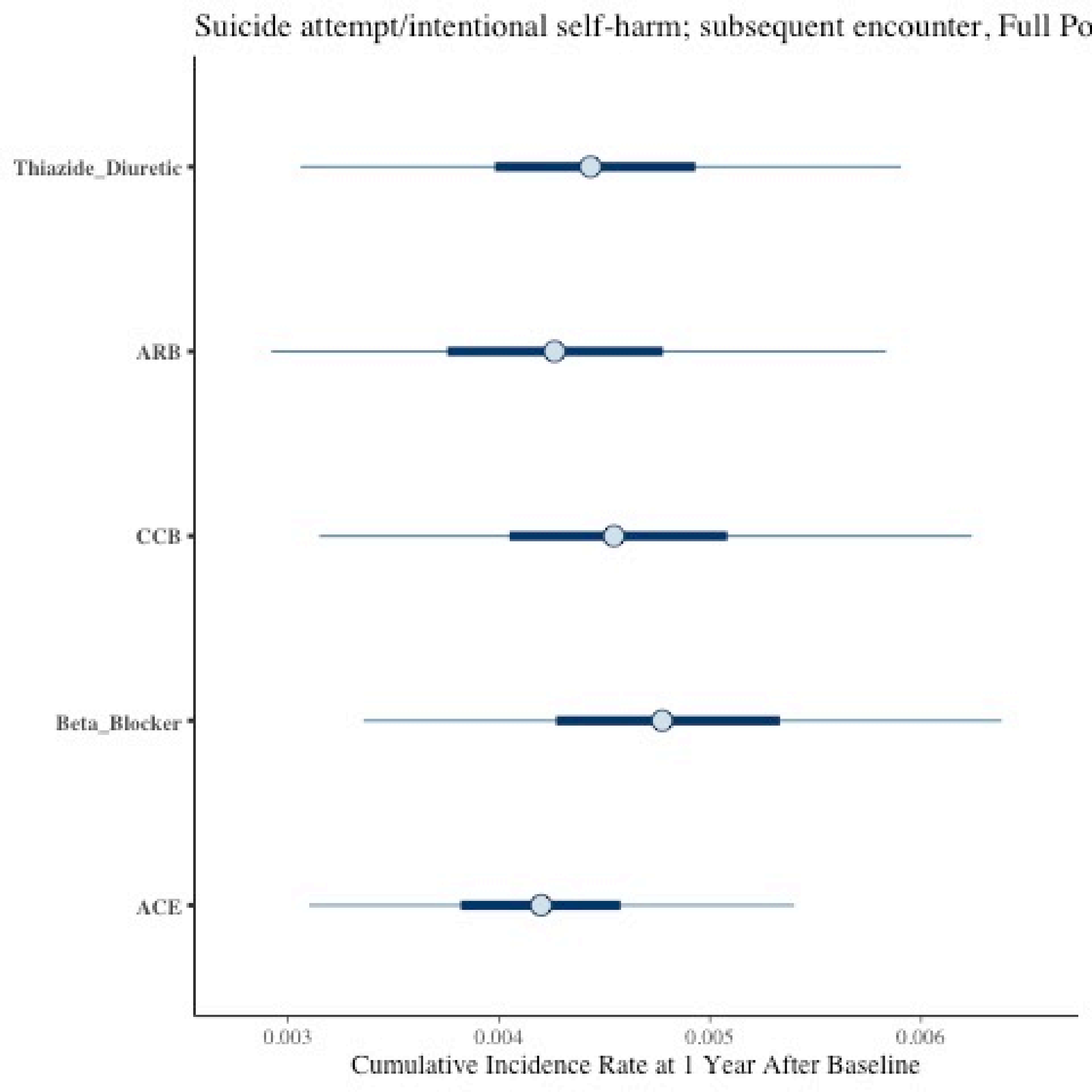

Suicide attempt/intentional self-harm; subsequent encounter, Full Po
Thiazide_Diuretic
ARB
CCB
Beta_Blocker
ACE
0.003
0.004
0.005
0.006
Cumulative Incidence Rate at 1 Year After Baseline

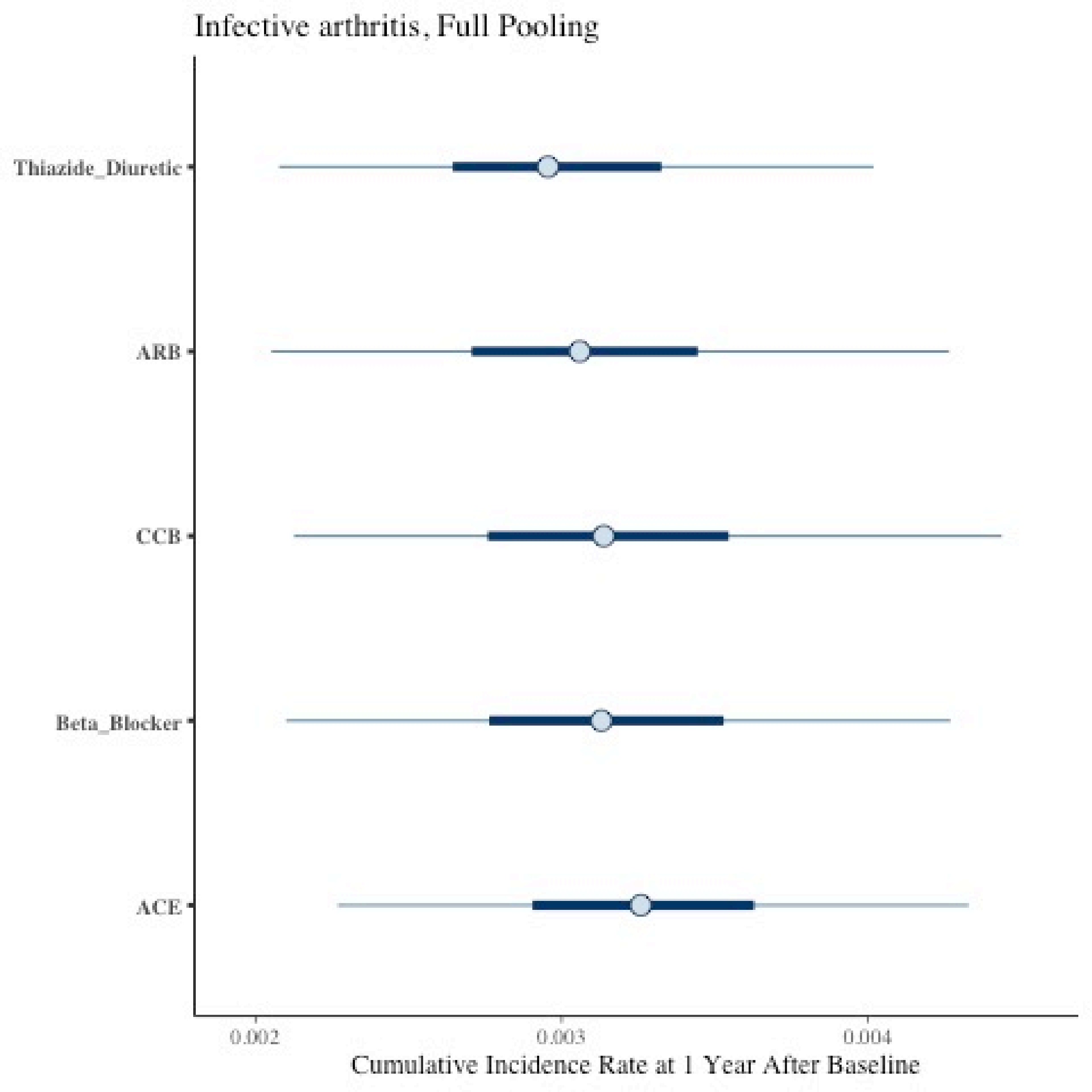

Infective arthritis, Full Pooling
Thiazide_Diuretic
ARB
CCB
Beta_Blocker
ACE
0.002
0.003
0.004
Cumulative Incidence Rate at 1 Year After Baseline

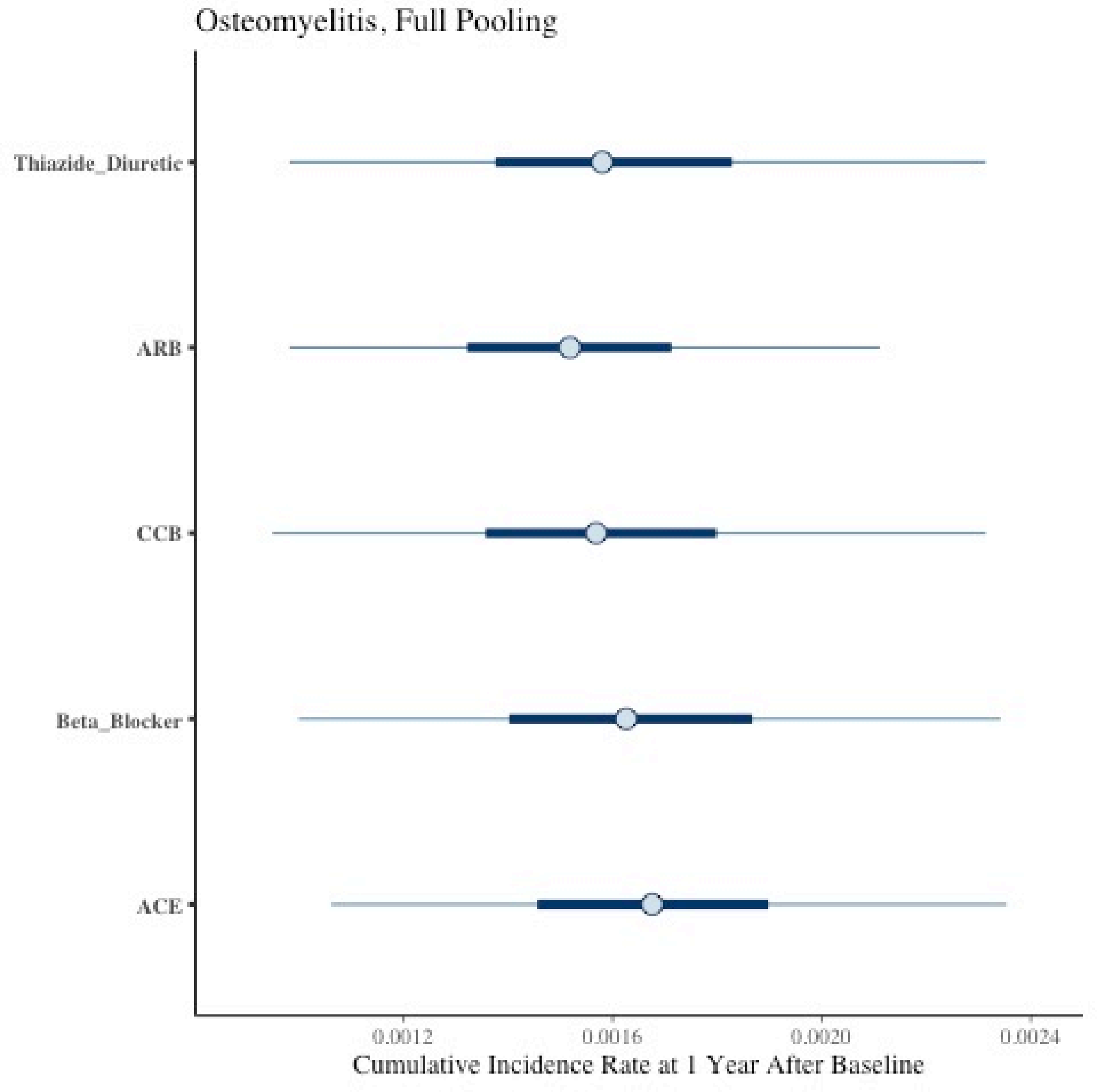

Osteomyelitis, Full Pooling
Thiazide_Diuretic
ARB
CCB
Beta_Blocker
ACE
0.0012
0.0016
0.0020
0.0024
Cumulative Incidence Rate at 1 Year After Baseline

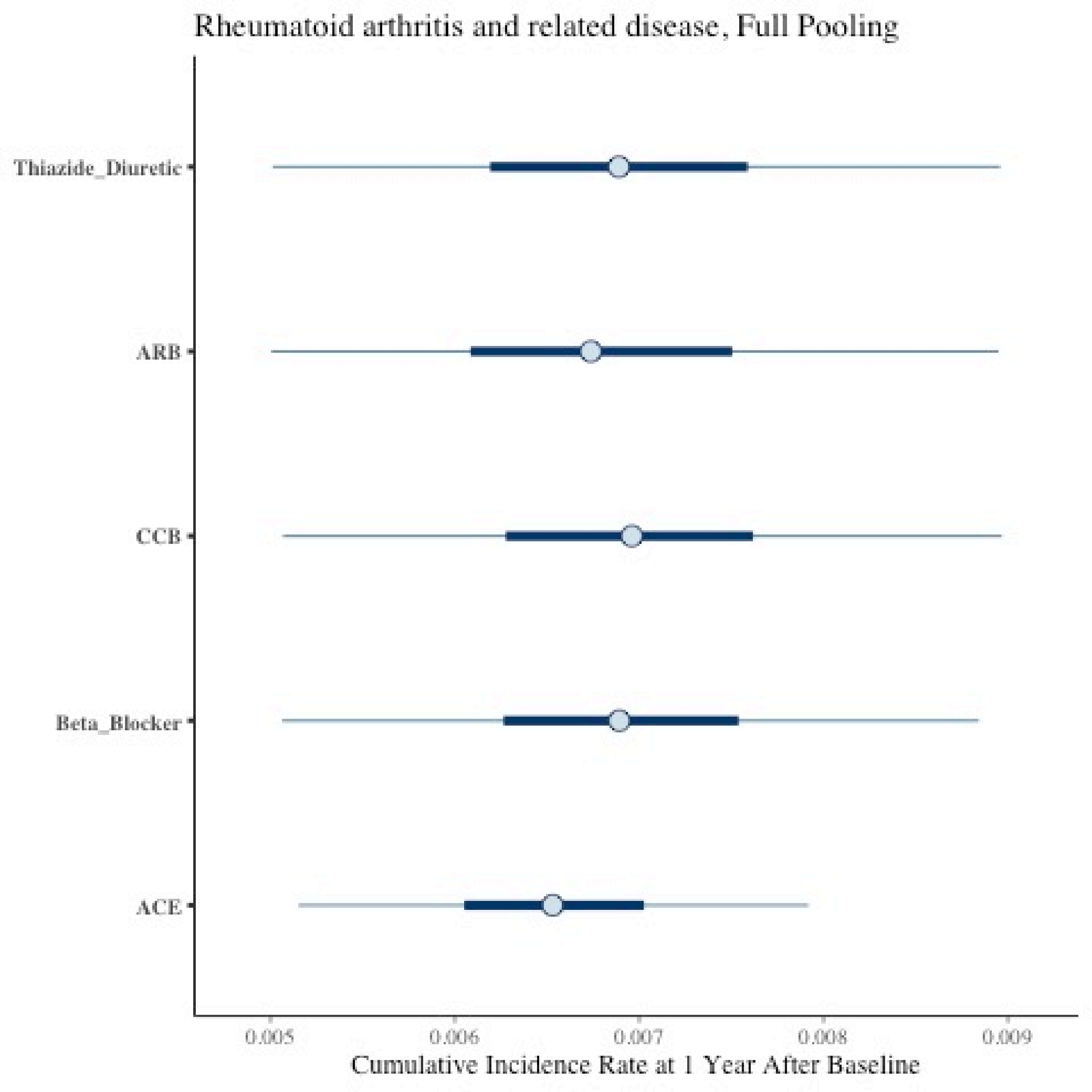

Rheumatoid arthritis and related disease, Full Pooling
Thiazide_Diuretic
ARB
CCB
Beta_Blocker
ACE
0.005
0.006
0.007
0.008
0.009
Cumulative Incidence Rate at 1 Year After Baseline

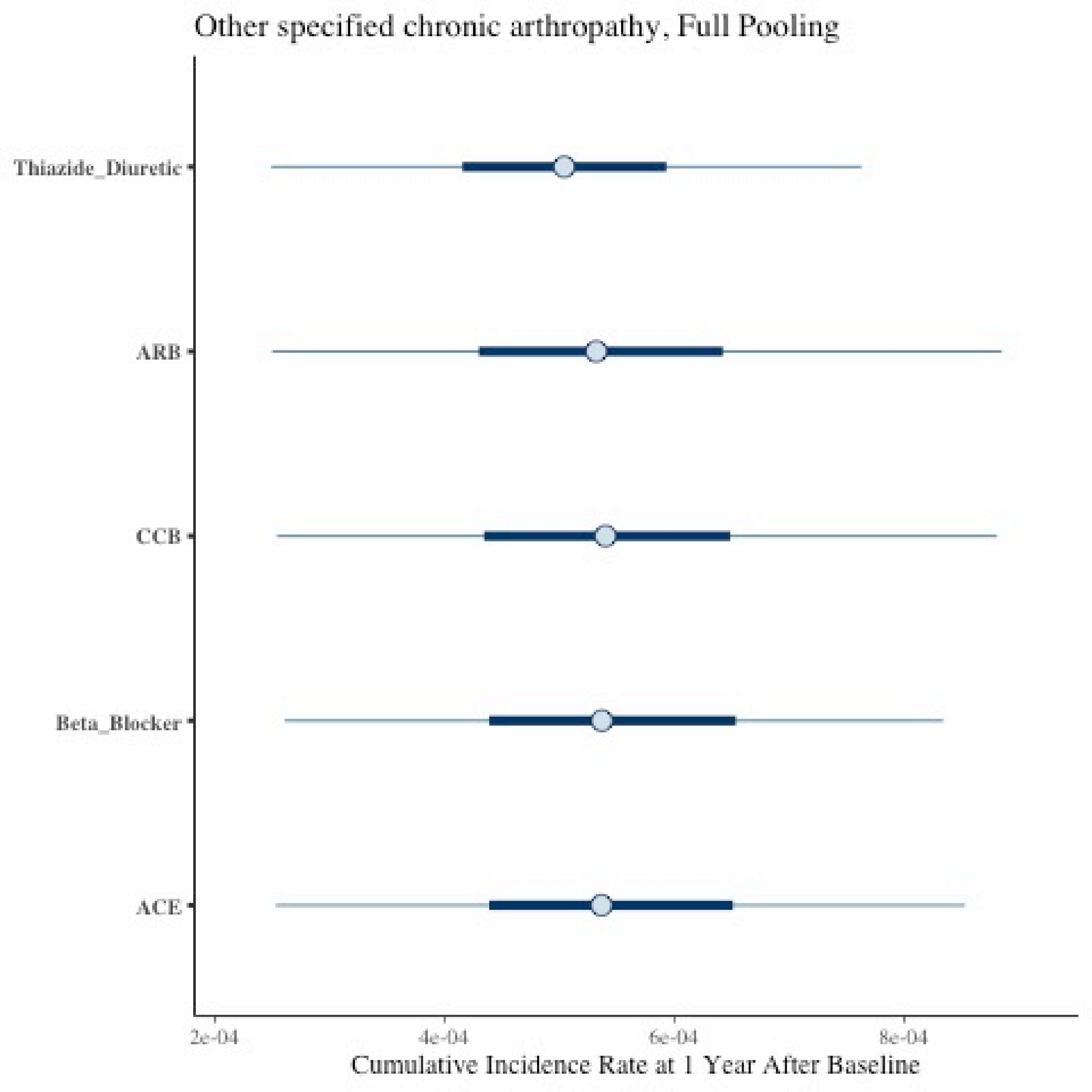

Other specified chronic arthropathy, Full Pooling
Thiazide_Diuretic
ARB
CCB
Beta_Blocker
ACE
2e-04
4e-04
6e-04
8e-04
Cumulative Incidence Rate at 1 Year After Baseline

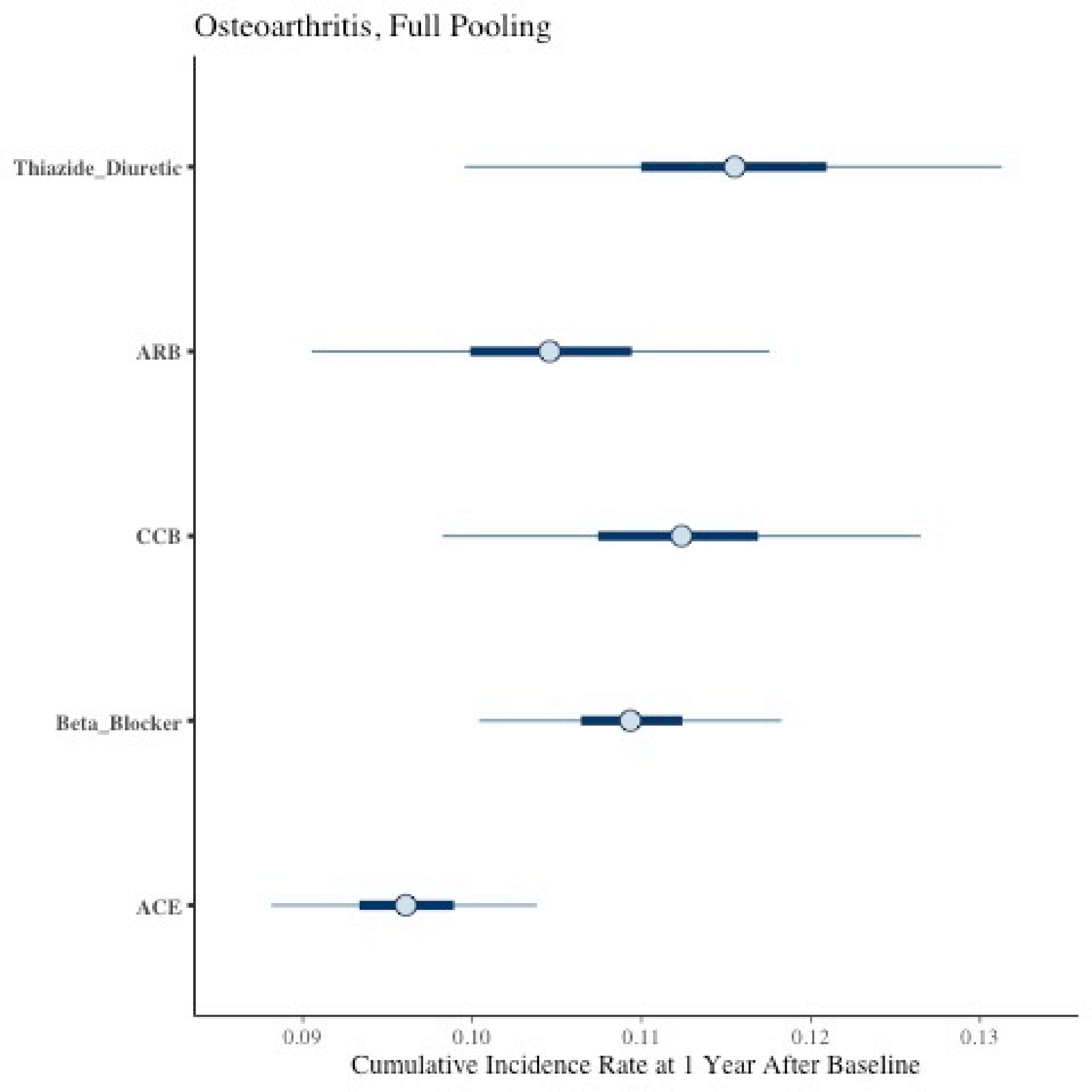

Osteoarthritis, Full Pooling
Thiazide_Diuretic
ARB
CCB
Beta_Blocker
ACE
0.09
0.10
0.11
0.12
0.13
Cumulative Incidence Rate at 1 Year After Baseline

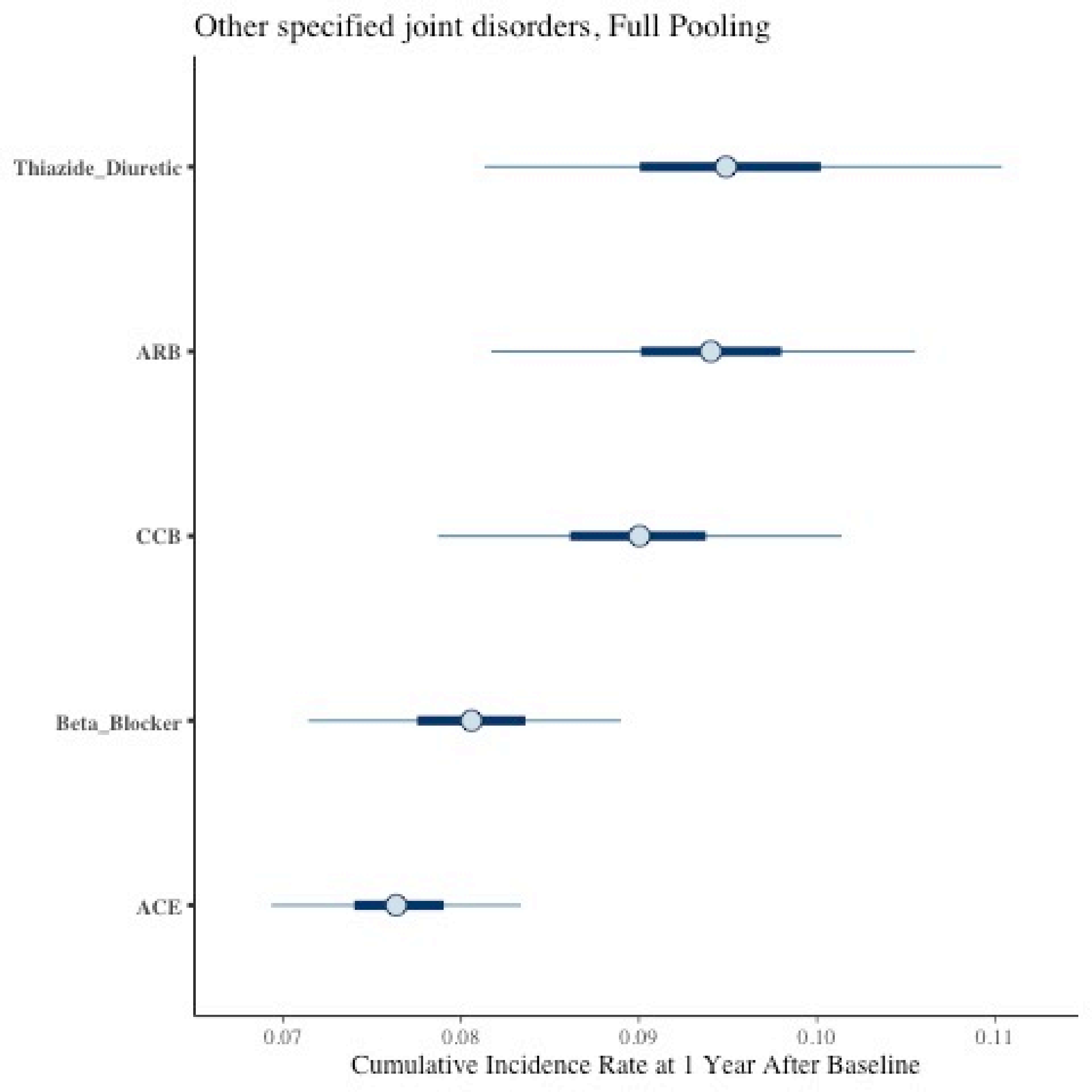

Other specified joint disorders, Full Pooling
Thiazide_Diuretic
ARB
CCB
Beta_Blocker
ACE
0.07
0.08
0.09
0.10
0.11
Cumulative Incidence Rate at 1 Year After Baseline

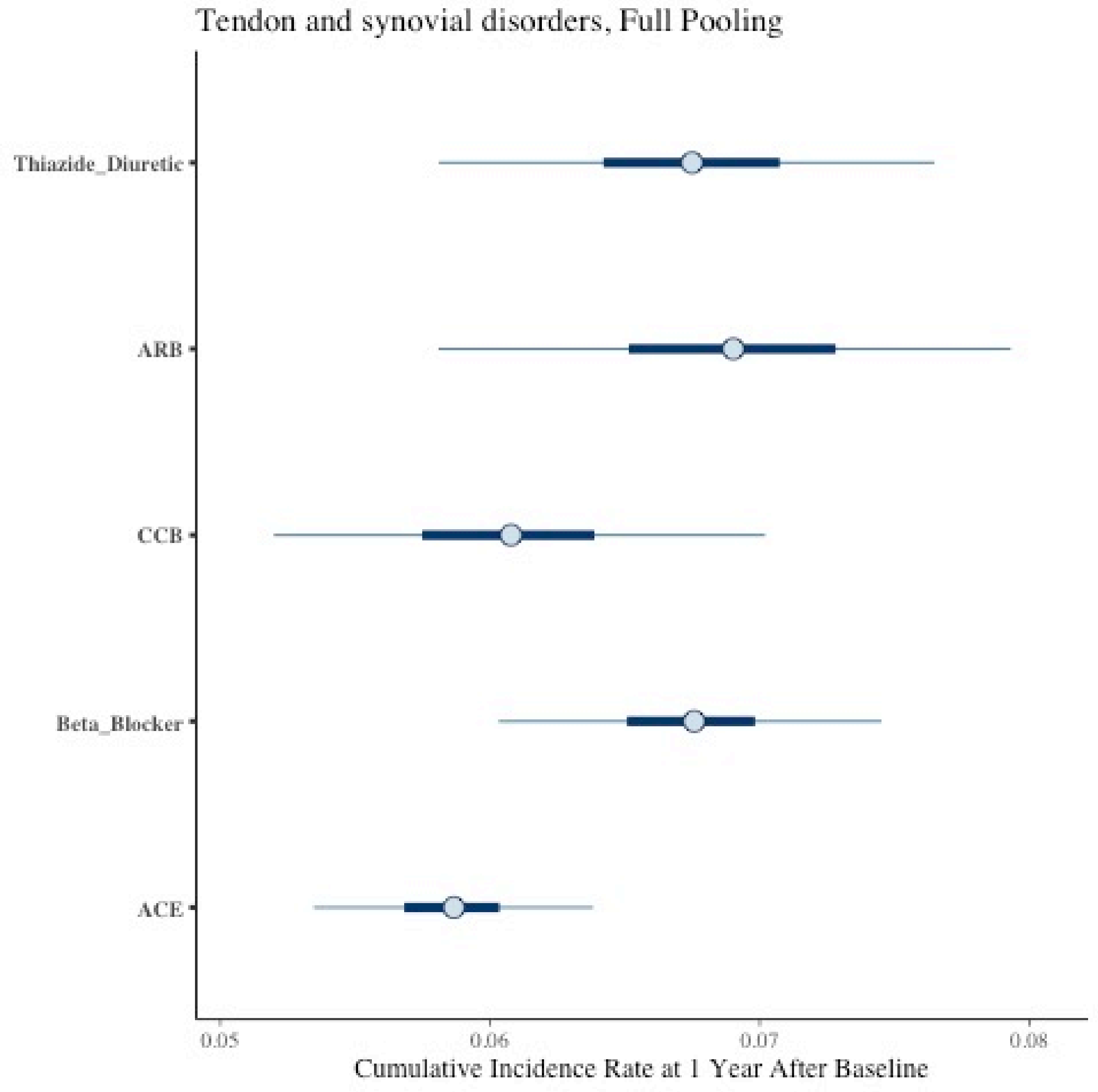
Tendon and synovial disorders, Full Pooling
Thiazide_Diuretic
ARB
CCB
Beta_Blocker
ACE
0.05
0.06
0.07
0.08
Cumulative Incidence Rate at 1 Year After Baseline

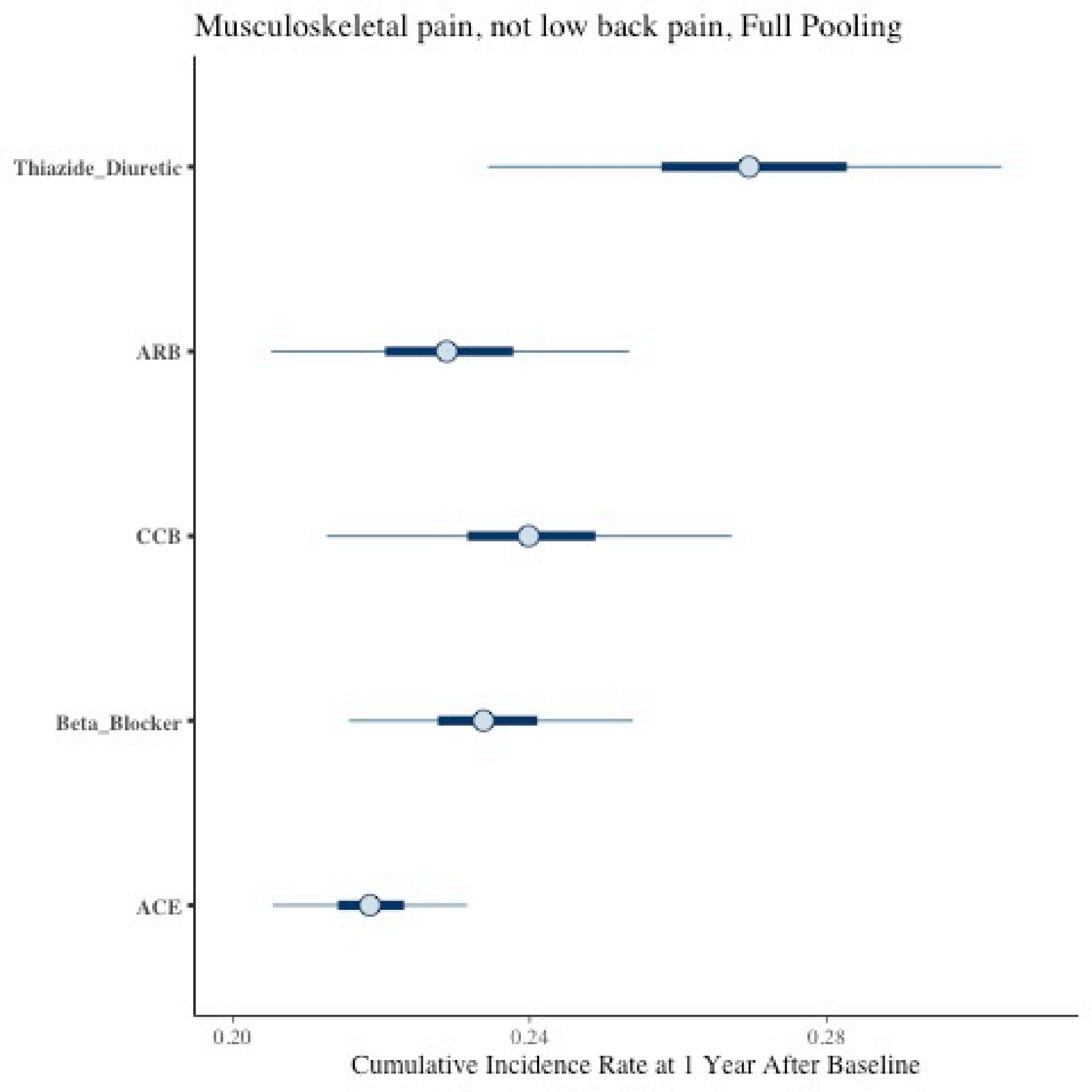

Musculoskeletal pain, not low back pain, Full Pooling
Thiazide_Diuretic
ARB
CCB
Beta_Blocker
ACE
0.20
0.24
0.28
Cumulative Incidence Rate at 1 Year After Baseline

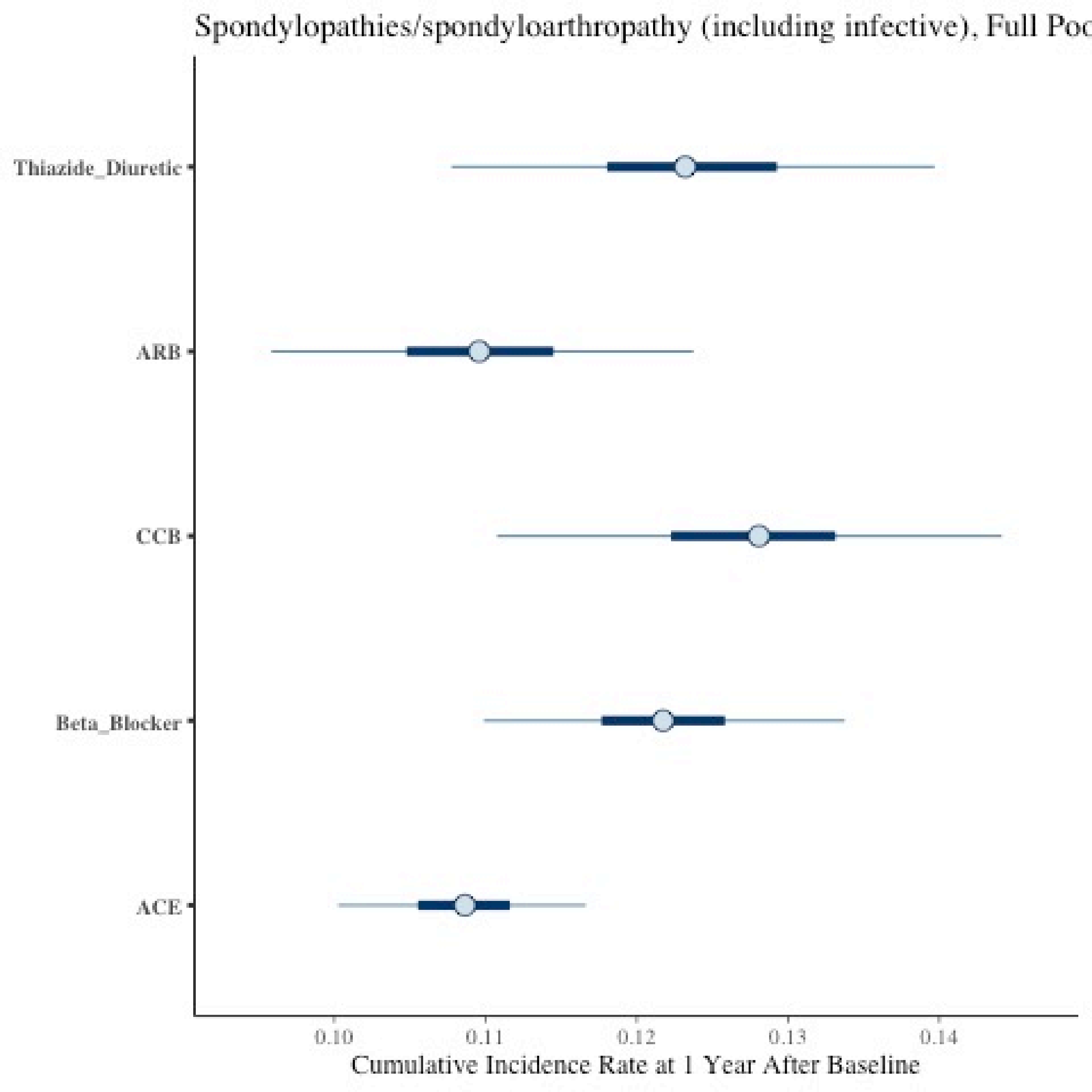
Spondylopathies/spondyloarthropathy (including infective), Full Poo
Thiazide_Diuretic
ARB
CCB
Beta_Blocker
ACE
0.10
0.11
0.12
0.13
0.14
Cumulative Incidence Rate at 1 Year After Baseline

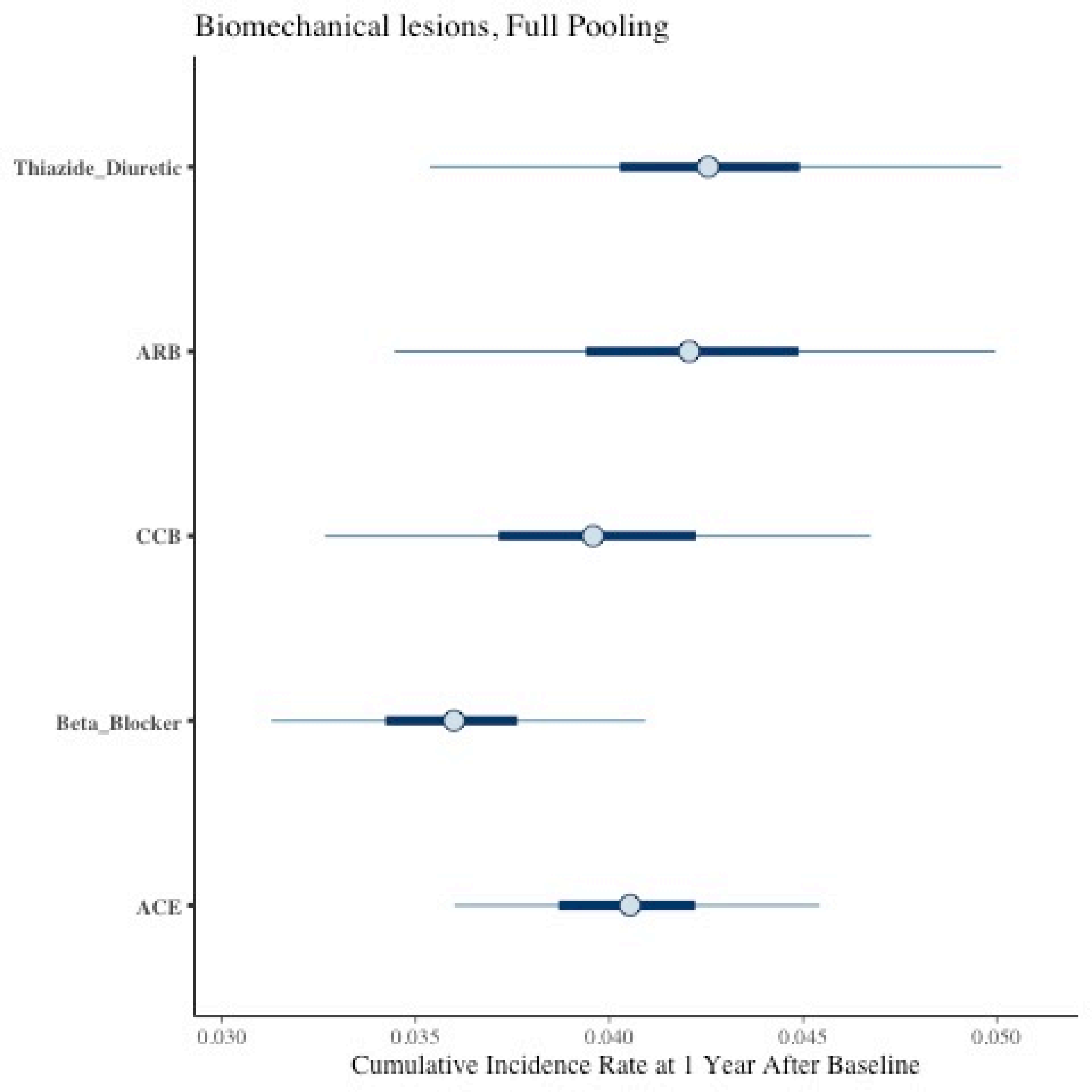
Biomechanical lesions, Full Pooling
Thiazide_Diuretic
ARB
CCB
Beta_Blocker
ACE
0.030
0.035
0.040
0.045
0.050
Cumulative Incidence Rate at 1 Year After Baseline

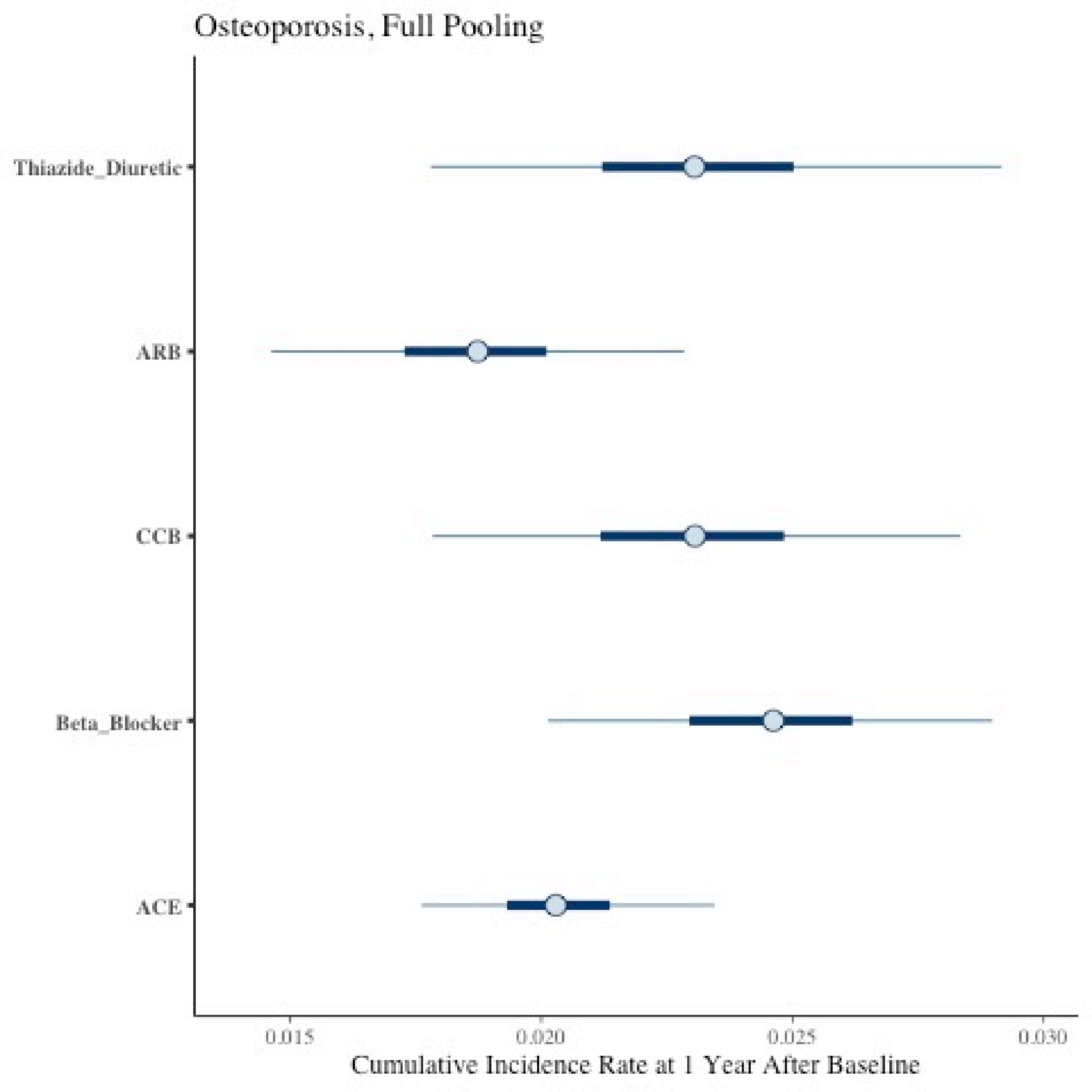
Osteoporosis, Full Pooling
Thiazide_Diuretic
ARB
CCB
Beta_Blocker
ACE
0.015
0.020
0.025
0.030
Cumulative Incidence Rate at 1 Year After Baseline

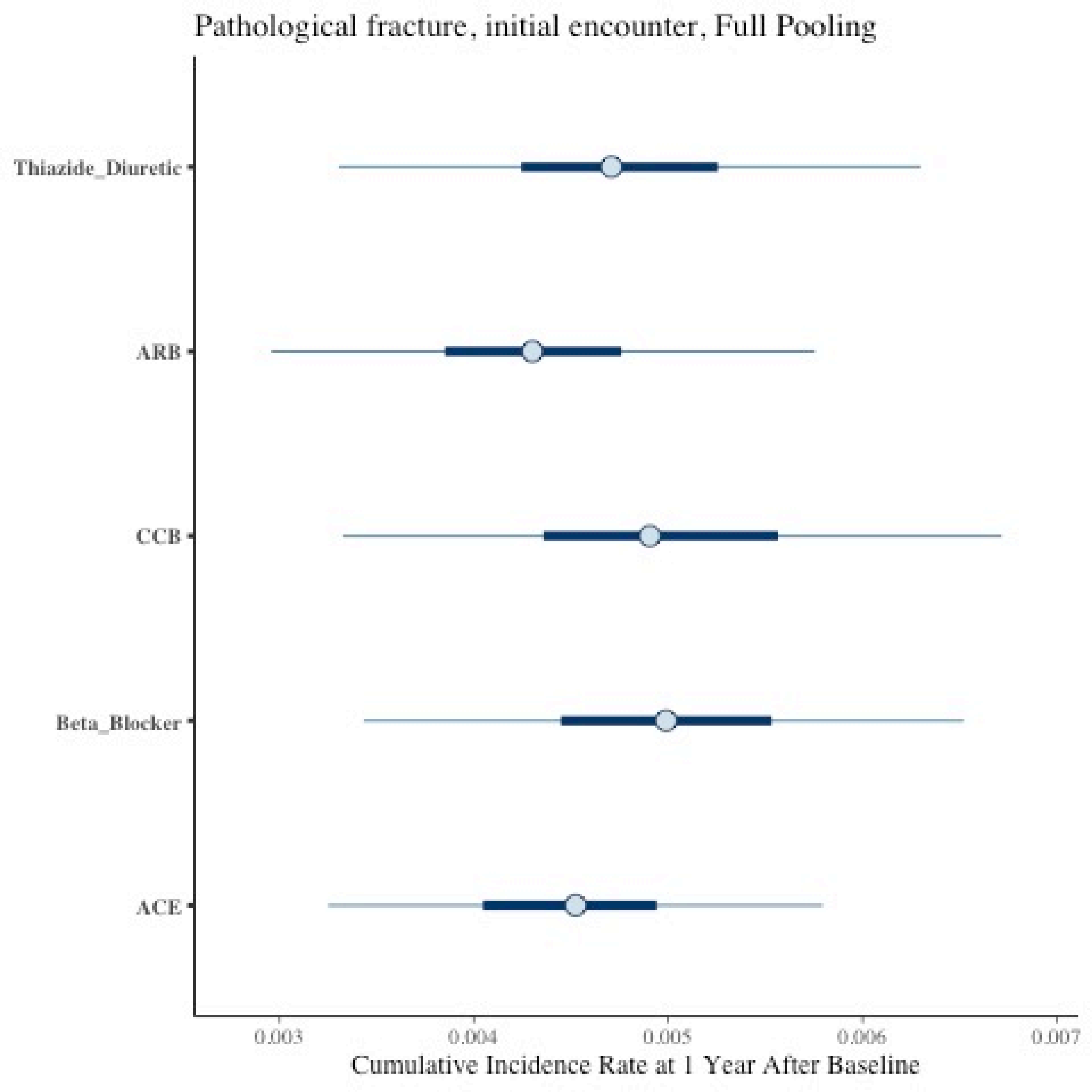

Pathological fracture, initial encounter, Full Pooling
Thiazide_Diuretic
ARB
CCB
Beta_Blocker
ACE
0.003
0.004
0.005
0.006
0.007
Cumulative Incidence Rate at 1 Year After Baseline

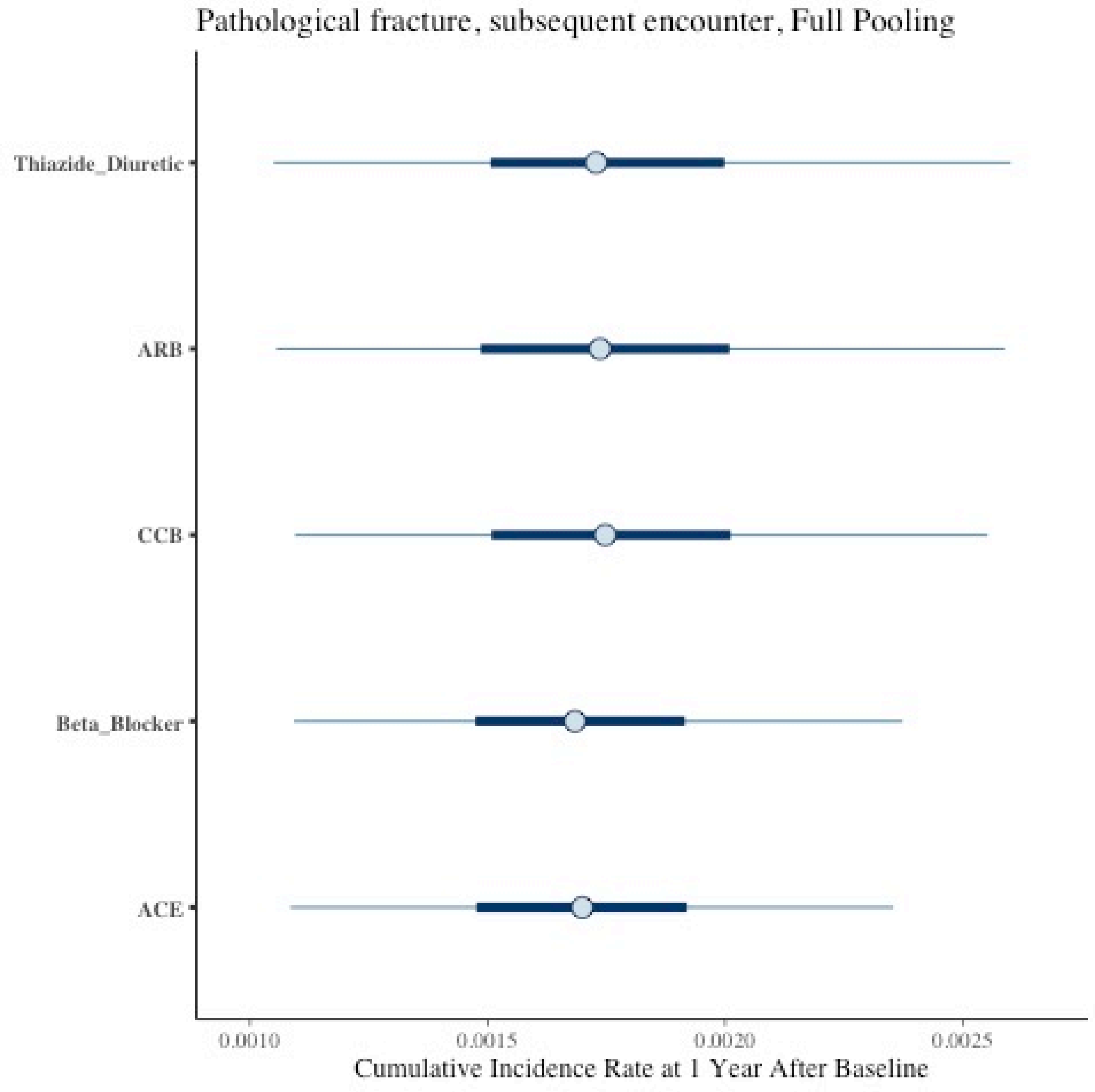

Pathological fracture, subsequent encounter, Full Pooling
Thiazide_Diuretic
ARB
CCB
Beta_Blocker
ACE
0.0010
0.0015
0.0020
0.0025
Cumulative Incidence Rate at 1 Year After Baseline

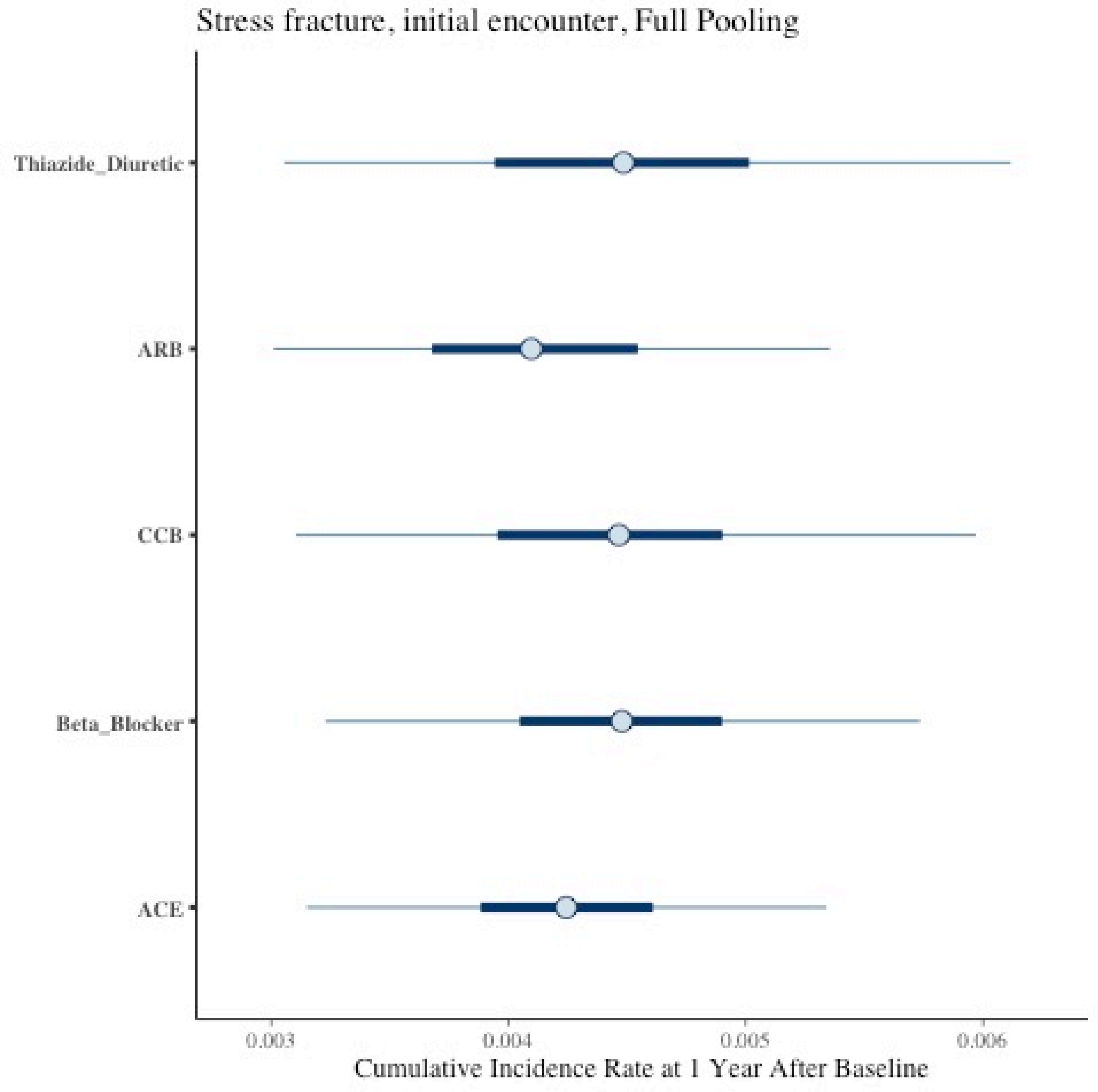
Stress fracture, initial encounter, Full Pooling
Thiazide_Diuretic
ARB
CCB
Beta_Blocker
ACE
0.003
0.004
0.005
0.006
Cumulative Incidence Rate at 1 Year After Baseline

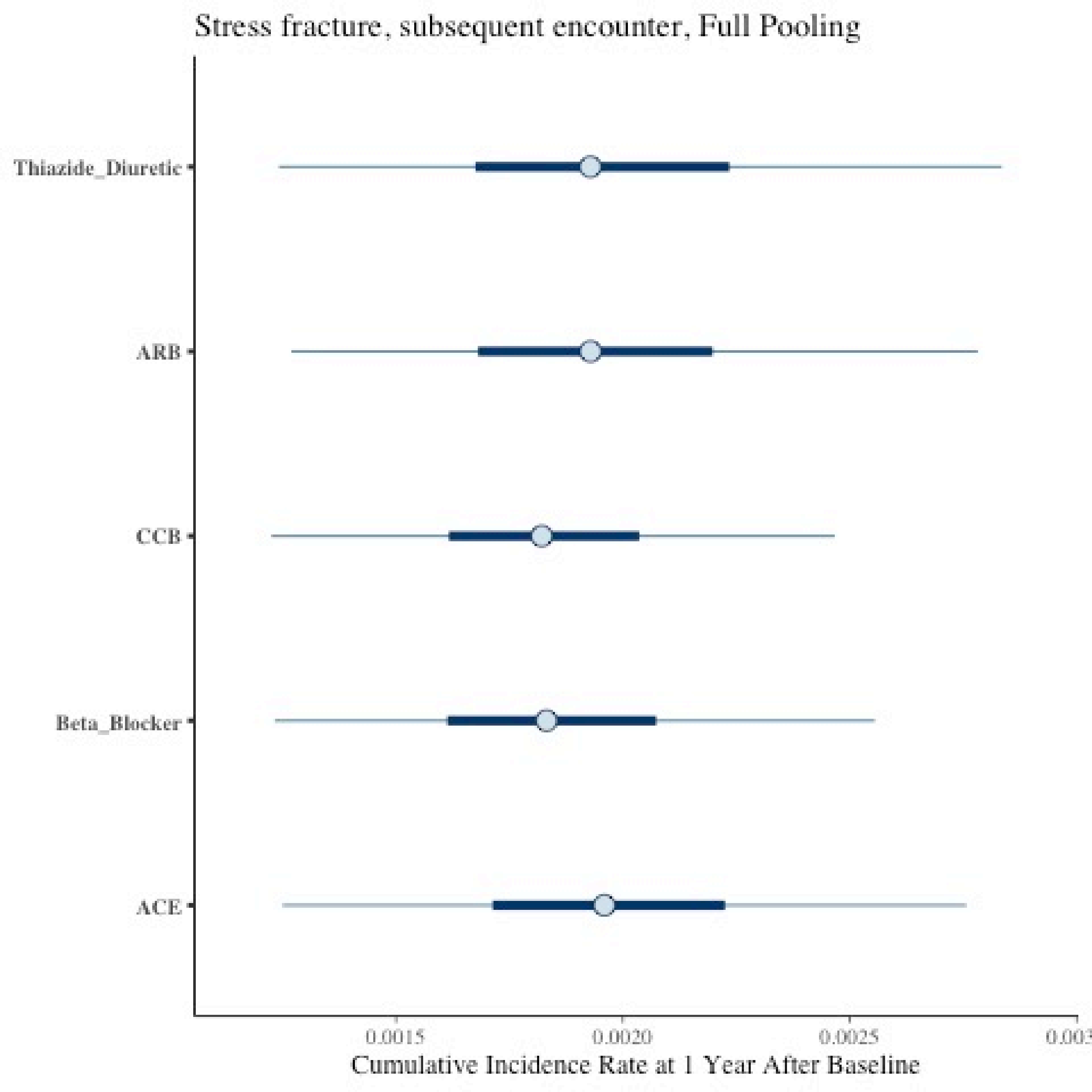

Stress fracture, subsequent encounter, Full Pooling
Thiazide_Diuretic
ARB
CCB
Beta_Blocker
ACE
0.0015
0.0020
0.0025
0.003
Cumulative Incidence Rate at 1 Year After Baseline

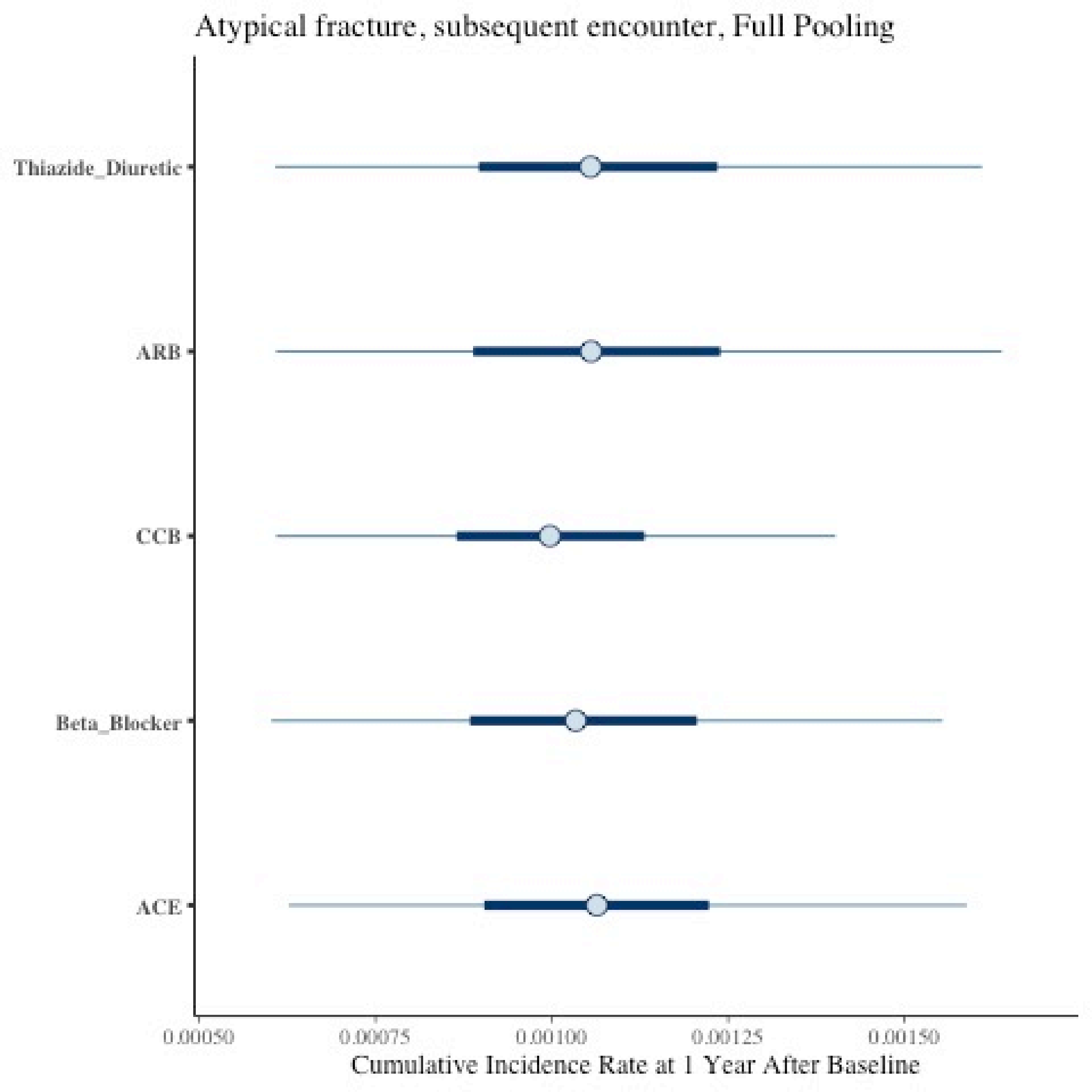

Atypical fracture, subsequent encounter, Full Pooling
Thiazide_Diuretic
ARB
CCB
Beta_Blocker
ACE
0.00050
0.00075
0.00100
0.00125
0.00150
Cumulative Incidence Rate at 1 Year After Baseline

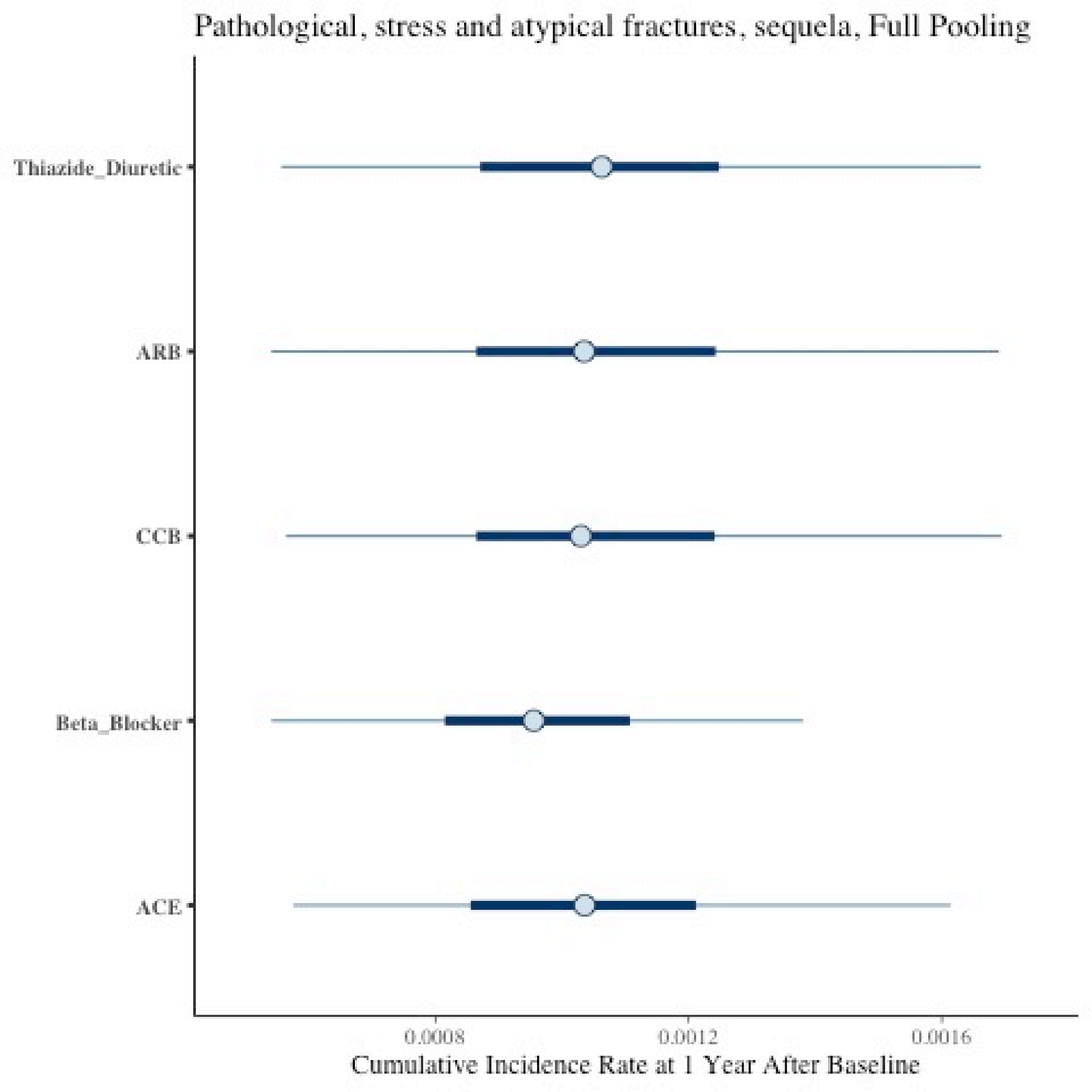
Pathological, stress and atypical fractures, sequela, Full Pooling
Thiazide_Diuretic
ARB
CCB
Beta_Blocker
ACE
0.0008
0.0012
0.0016
Cumulative Incidence Rate at 1 Year After Baseline

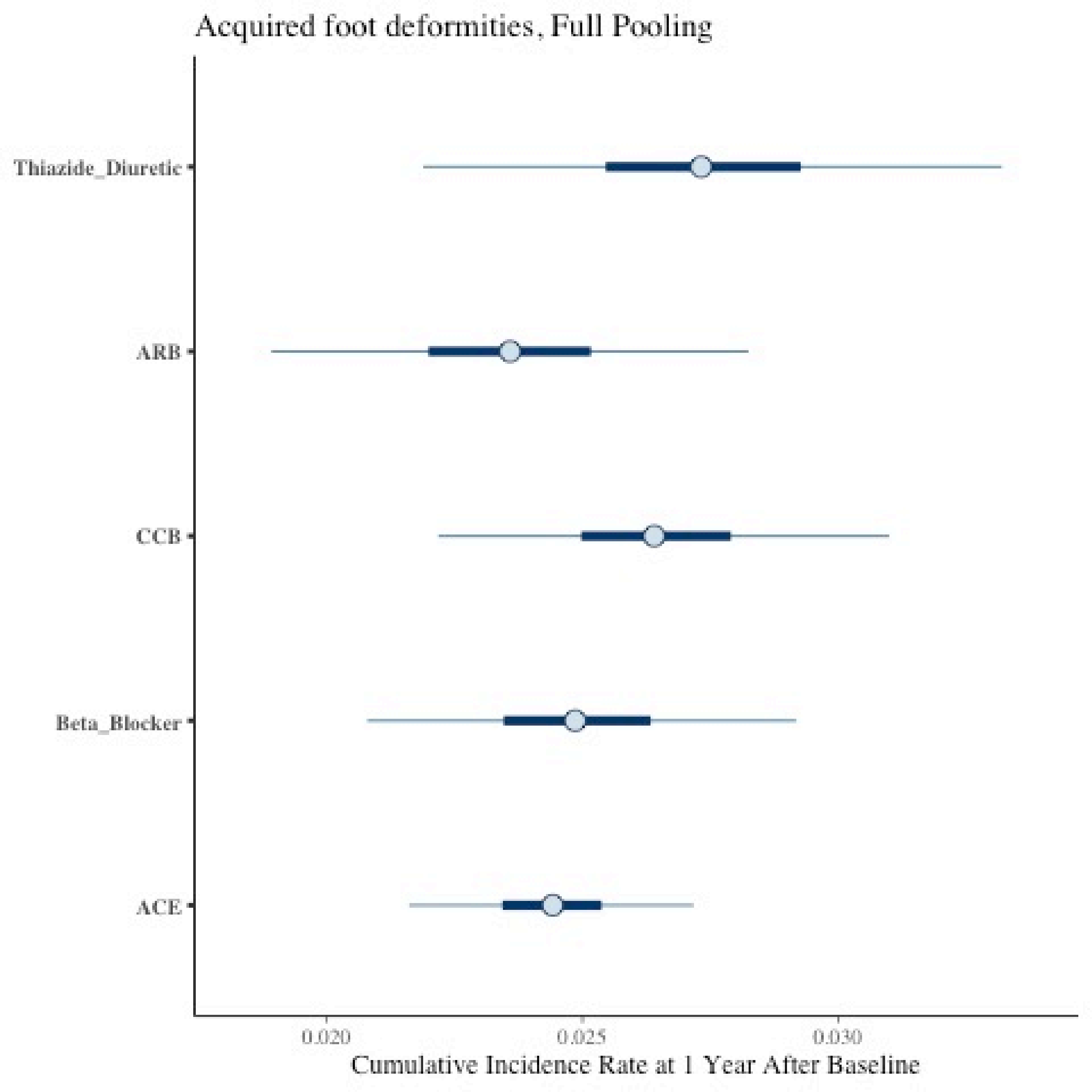
Acquired foot deformities, Full Pooling
Thiazide_Diuretic
ARB
CCB
Beta_Blocker
ACE
0.020
0.025
0.030
Cumulative Incidence Rate at 1 Year After Baseline

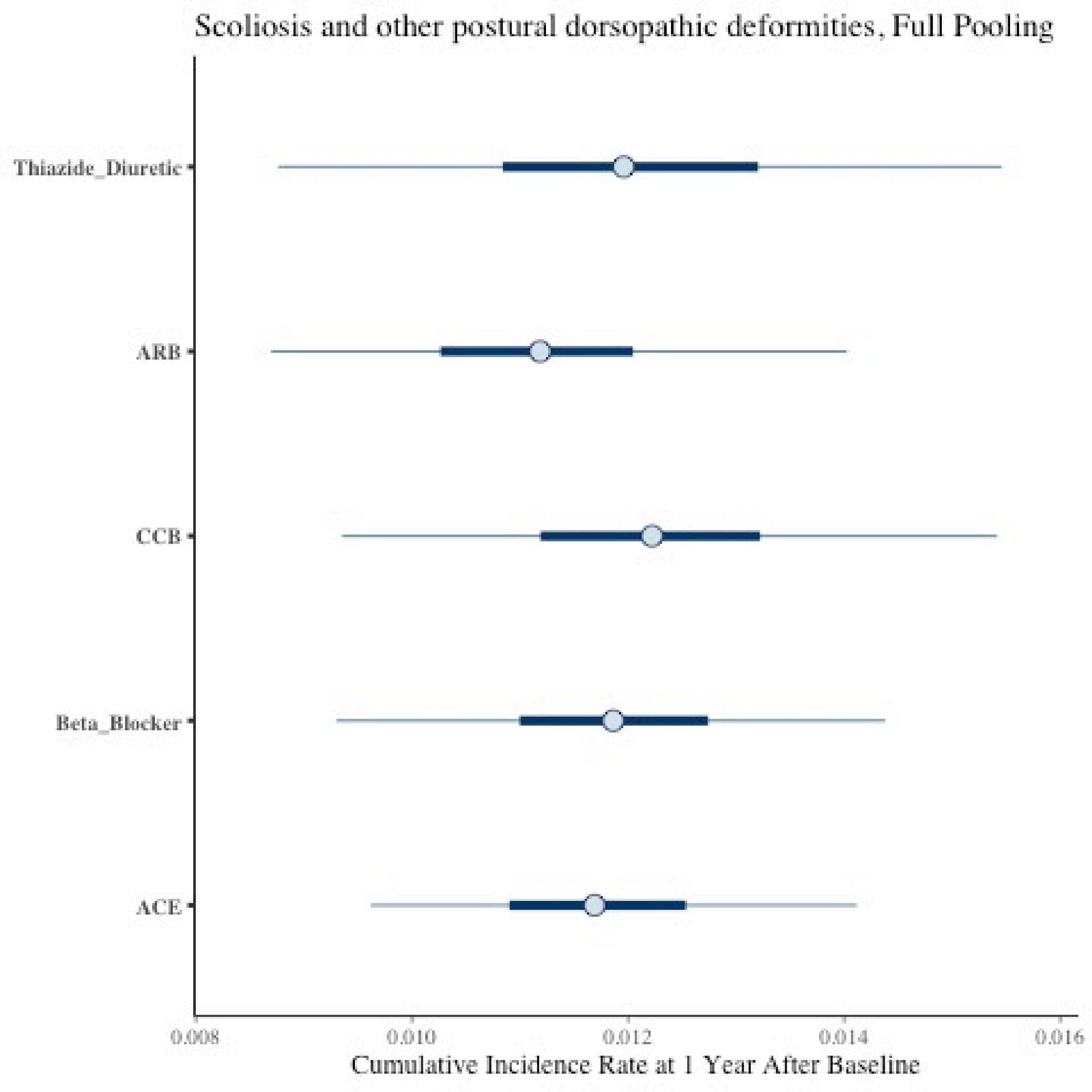

Scoliosis and other postural dorsopathic deformities, Full Pooling
Thiazide_Diuretic
ARB
CCB
Beta_Blocker
ACE
0.008
0.010
0.012
0.014
0.016
Cumulative Incidence Rate at 1 Year After Baseline

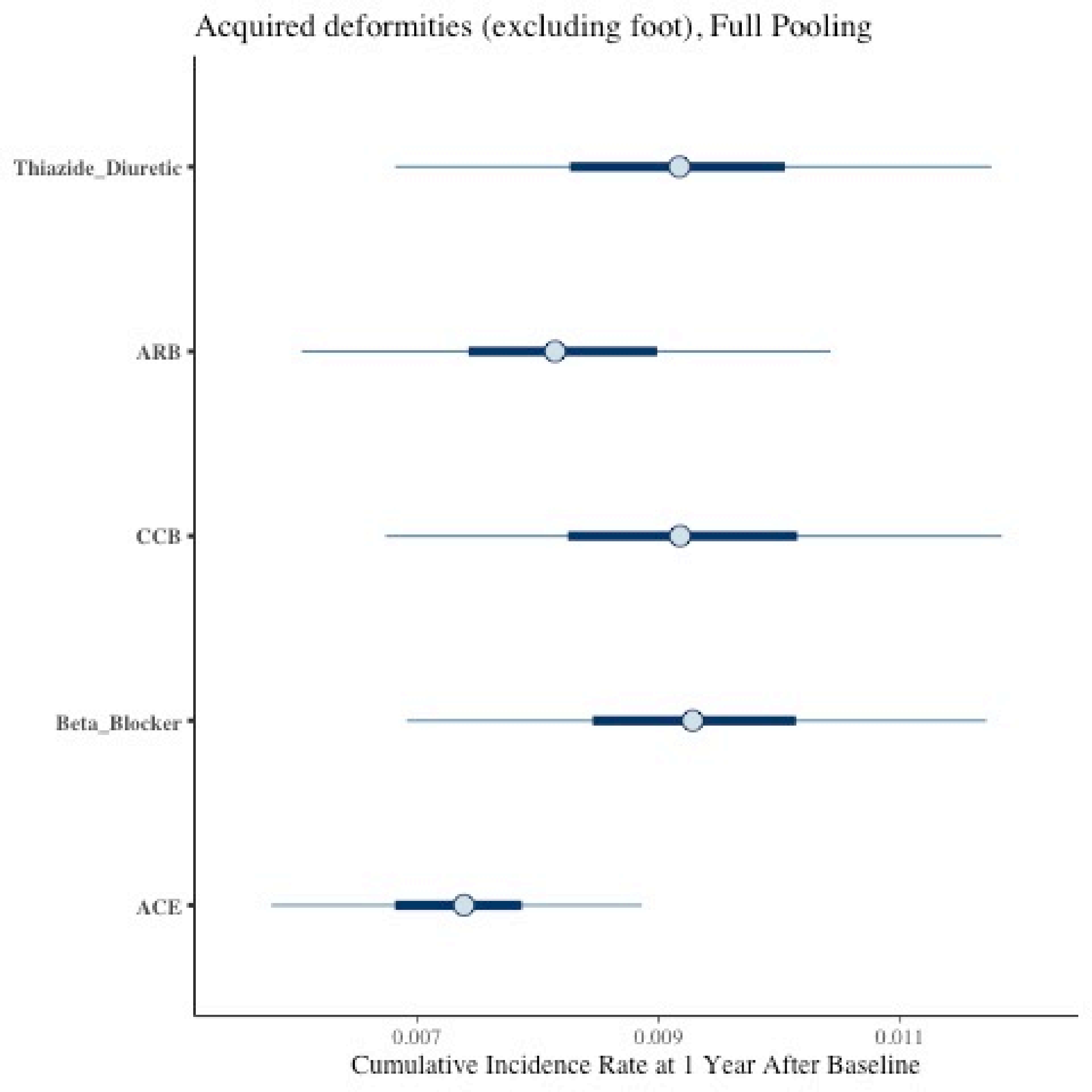

Acquired deformities (excluding foot), Full Pooling
Thiazide_Diuretic
ARB
CCB
Beta_Blocker
ACE
0.007
0.009
0.011
Cumulative Incidence Rate at 1 Year After Baseline

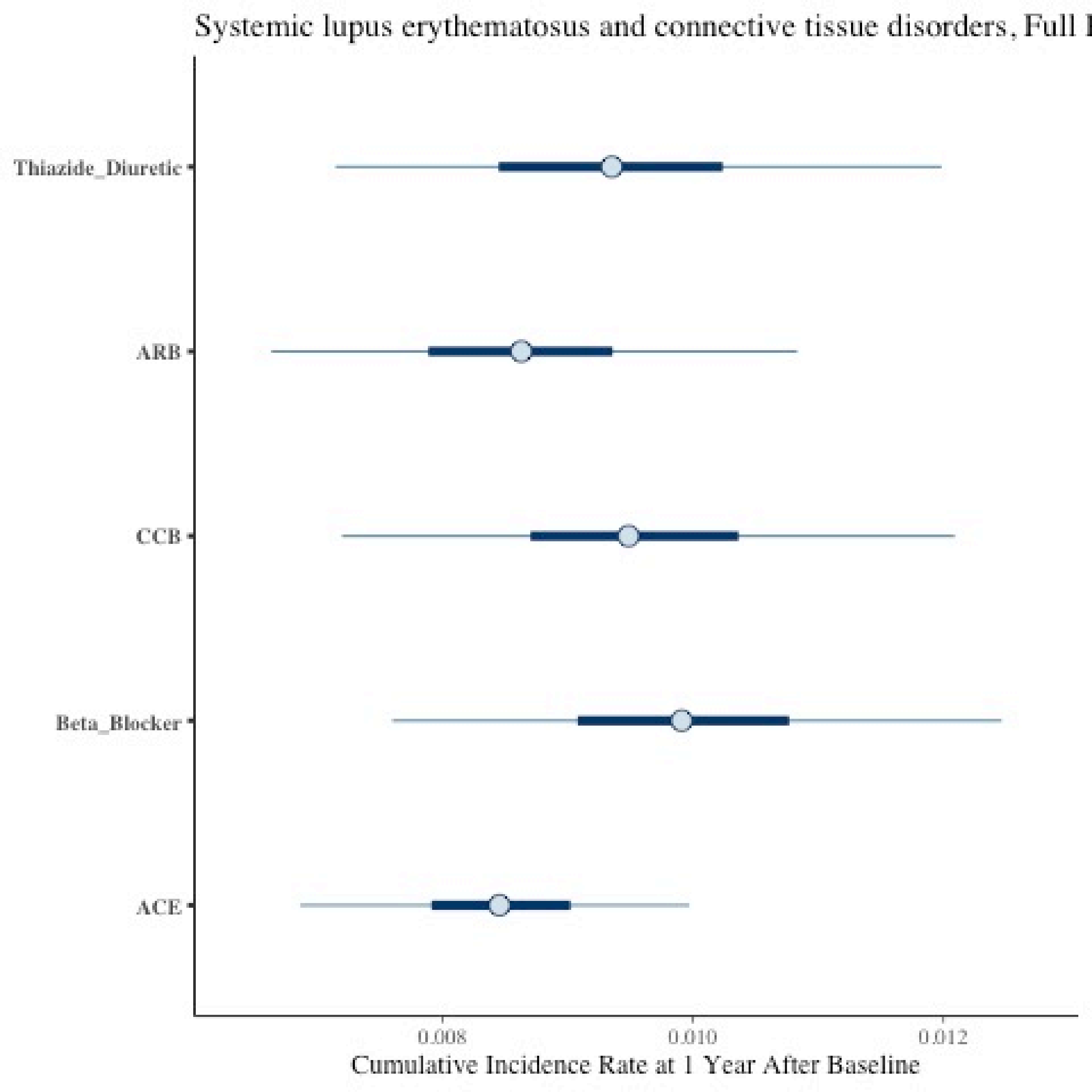

Systemic lupus erythematosus and connective tissue disorders, Full I
Thiazide_Diuretic
ARB
CCB
Beta_Blocker
ACE
0.008
0.010
0.012
Cumulative Incidence Rate at 1 Year After Baseline

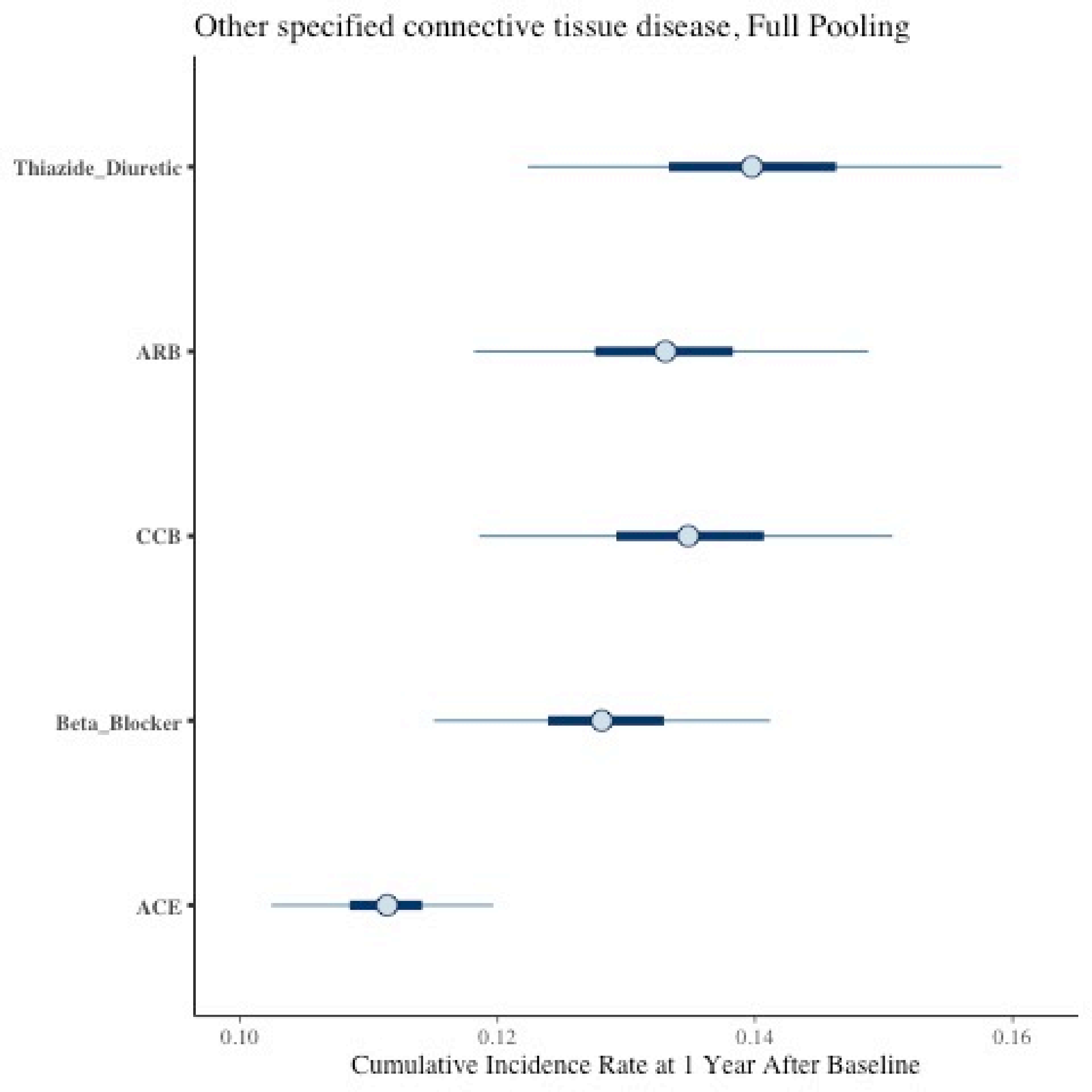

Other specified connective tissue disease, Full Pooling
Thiazide_Diuretic
ARB
CCB
Beta_Blocker
ACE
0.10
0.12
0.14
0.16
Cumulative Incidence Rate at 1 Year After Baseline

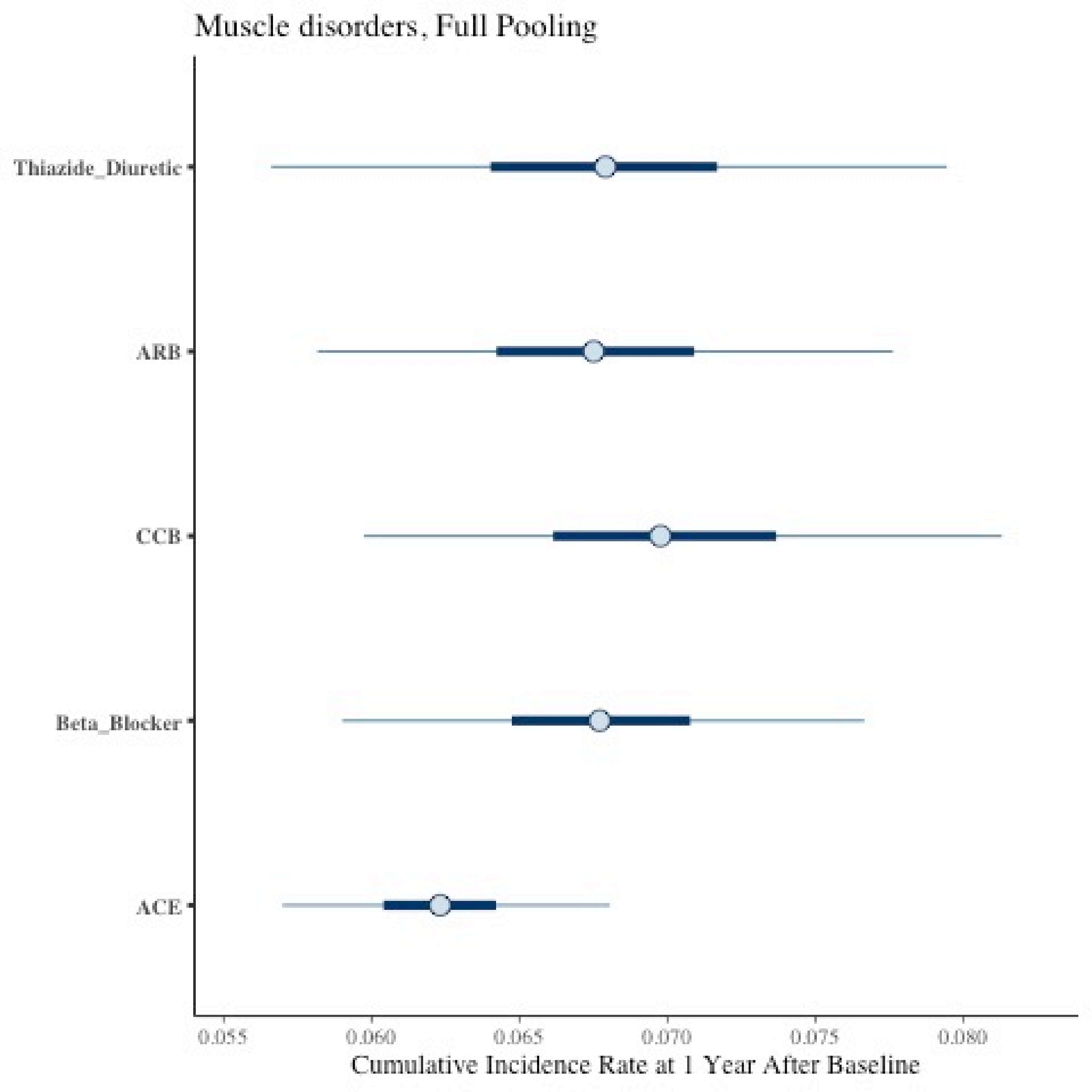

Muscle disorders, Full Pooling
Thiazide_Diuretic
ARB
CCB
Beta_Blocker
ACE
0.055
0.060
0.065
0.070
0.075
0.080
Cumulative Incidence Rate at 1 Year After Baseline

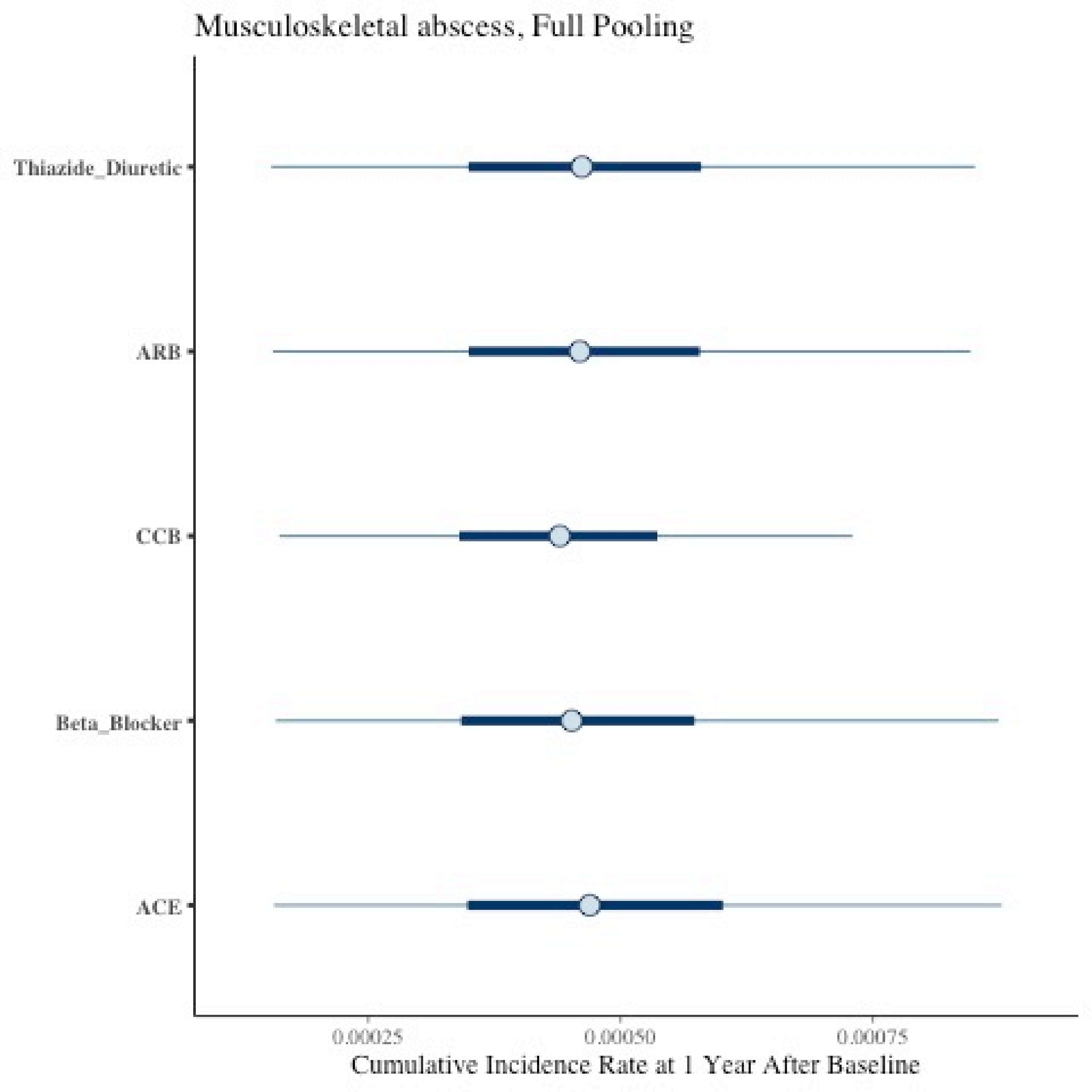
Musculoskeletal abscess, Full Pooling
Thiazide_Diuretic
ARB
CCB
Beta_Blocker
ACE
0.00025
0.00050
0.00075
Cumulative Incidence Rate at 1 Year After Baseline

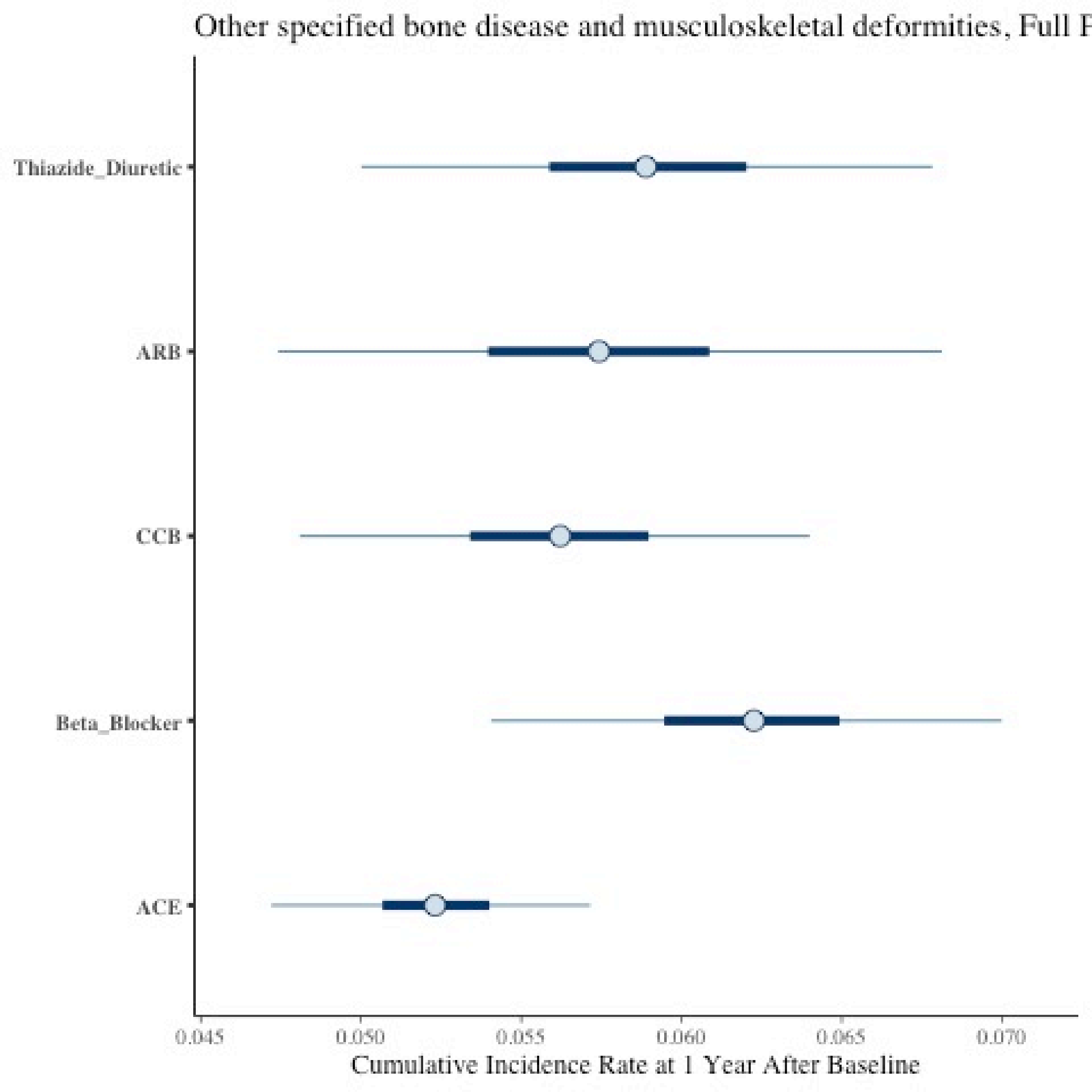

Other specified bone disease and musculoskeletal deformities, Full P
Thiazide_Diuretic
ARB
CCB
Beta_Blocker
ACE
0.045
0.050
0.055
0.060
0.065
0.070
Cumulative Incidence Rate at 1 Year After Baseline

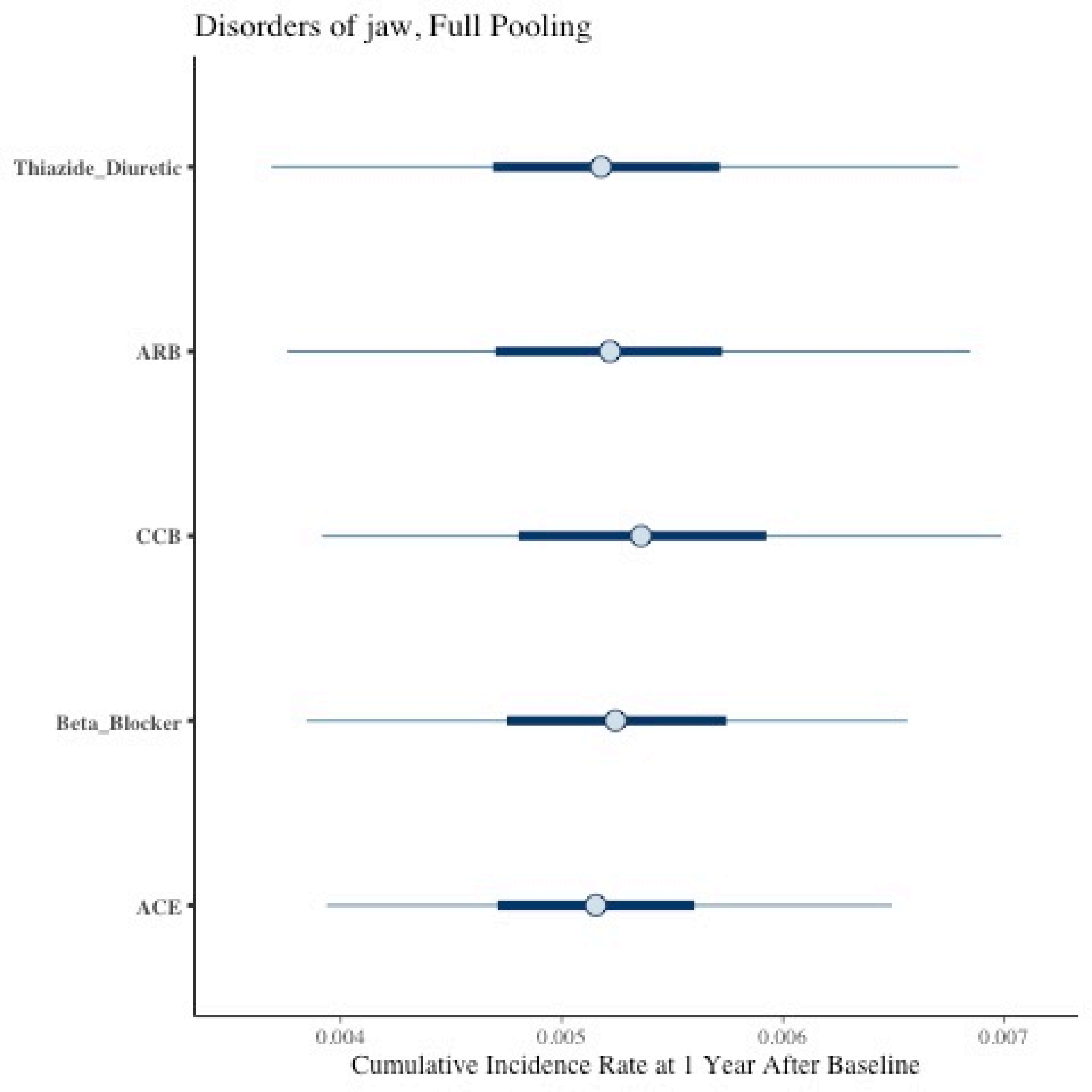

Disorders of jaw, Full Pooling
Thiazide_Diuretic
ARB
CCB
Beta_Blocker
ACE
0.004
0.005
0.006
0.007
Cumulative Incidence Rate at 1 Year After Baseline

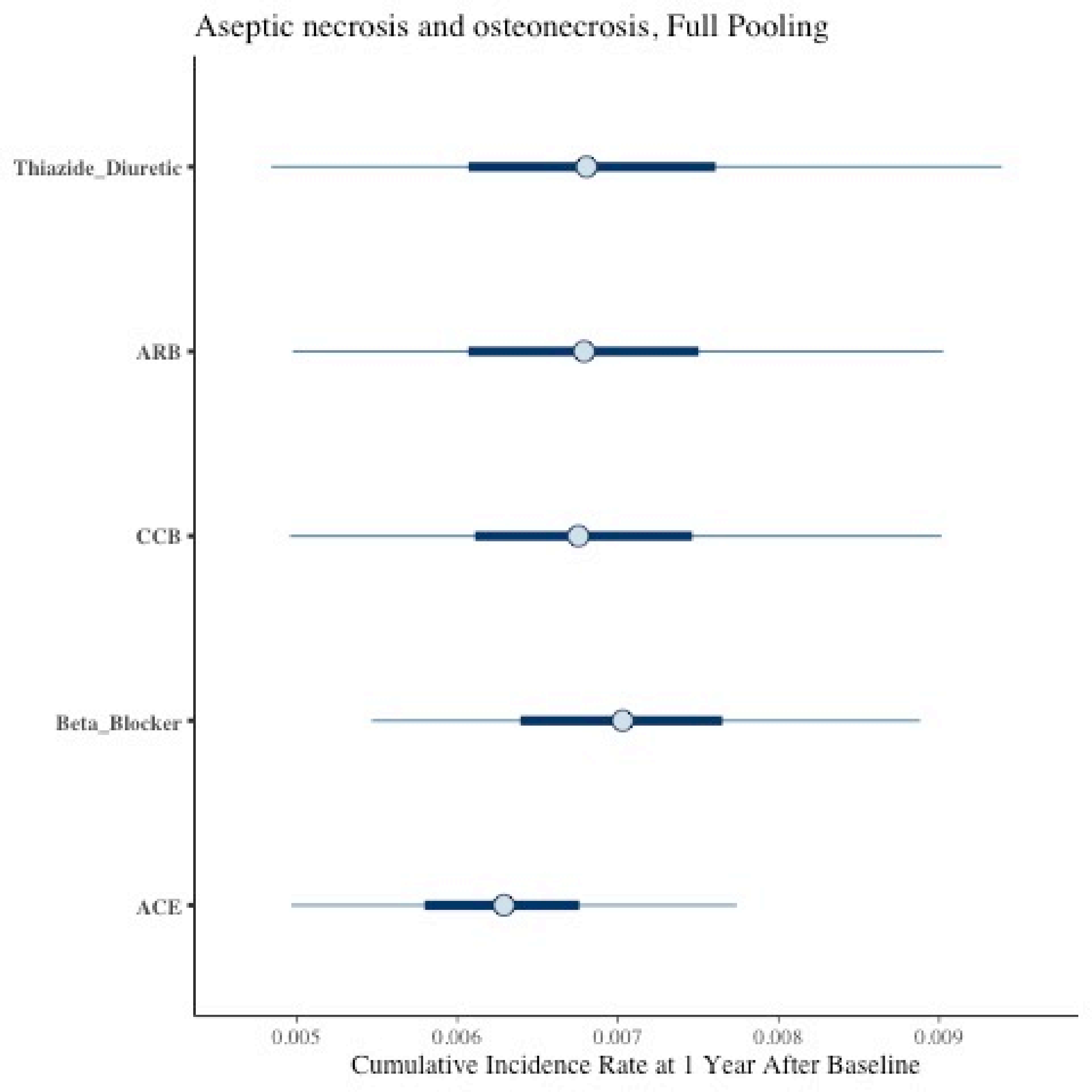
Aseptic necrosis and osteonecrosis, Full Pooling
Thiazide_Diuretic
ARB
CCB
Beta_Blocker
ACE
0.005
0.006
0.007
0.008
0.009
Cumulative Incidence Rate at 1 Year After Baseline

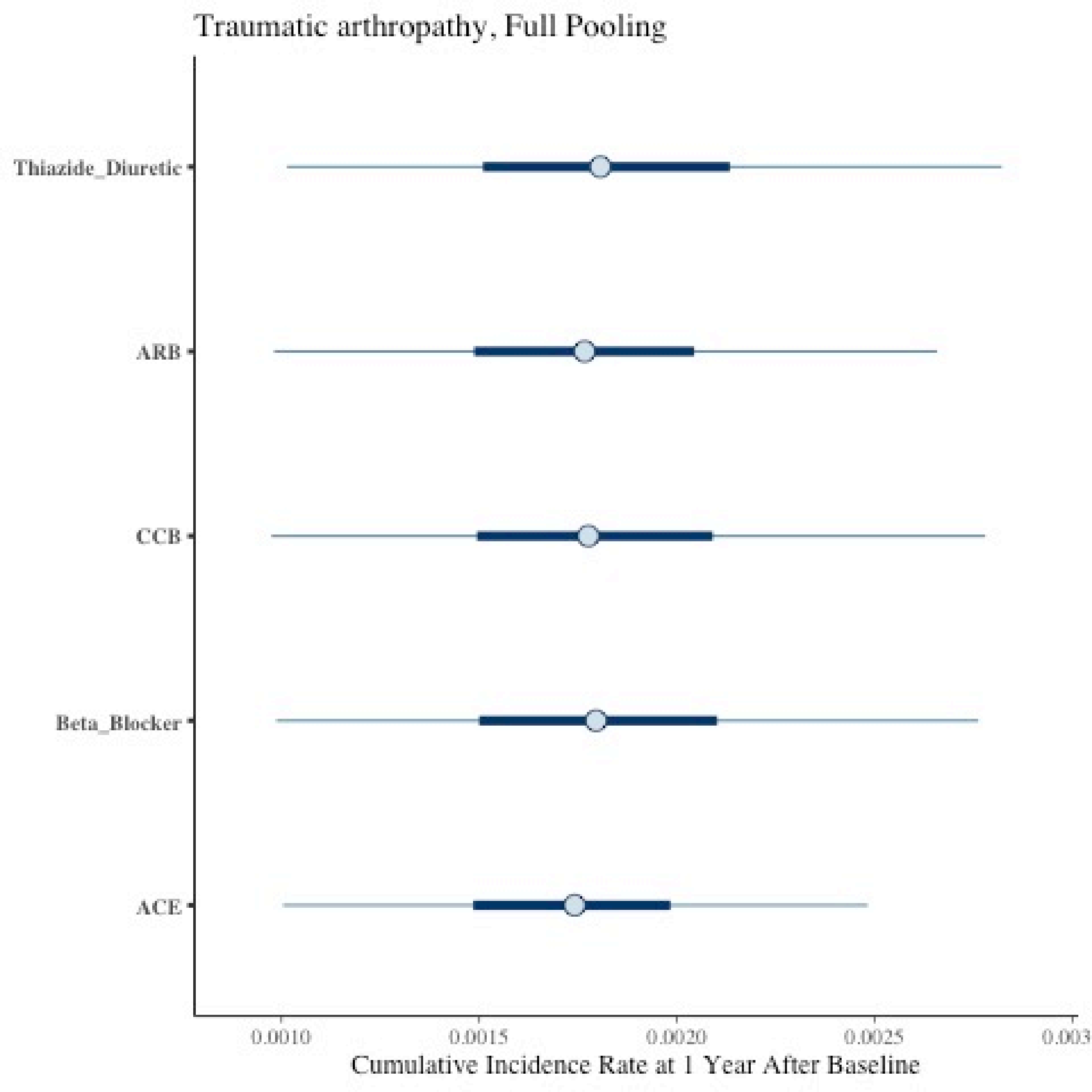

Traumatic arthropathy, Full Pooling
Thiazide_Diuretic
ARB
CCB
Beta_Blocker
ACE
0.0010
0.0015
0.0020
0.0025
0.003
Cumulative Incidence Rate at 1 Year After Baseline

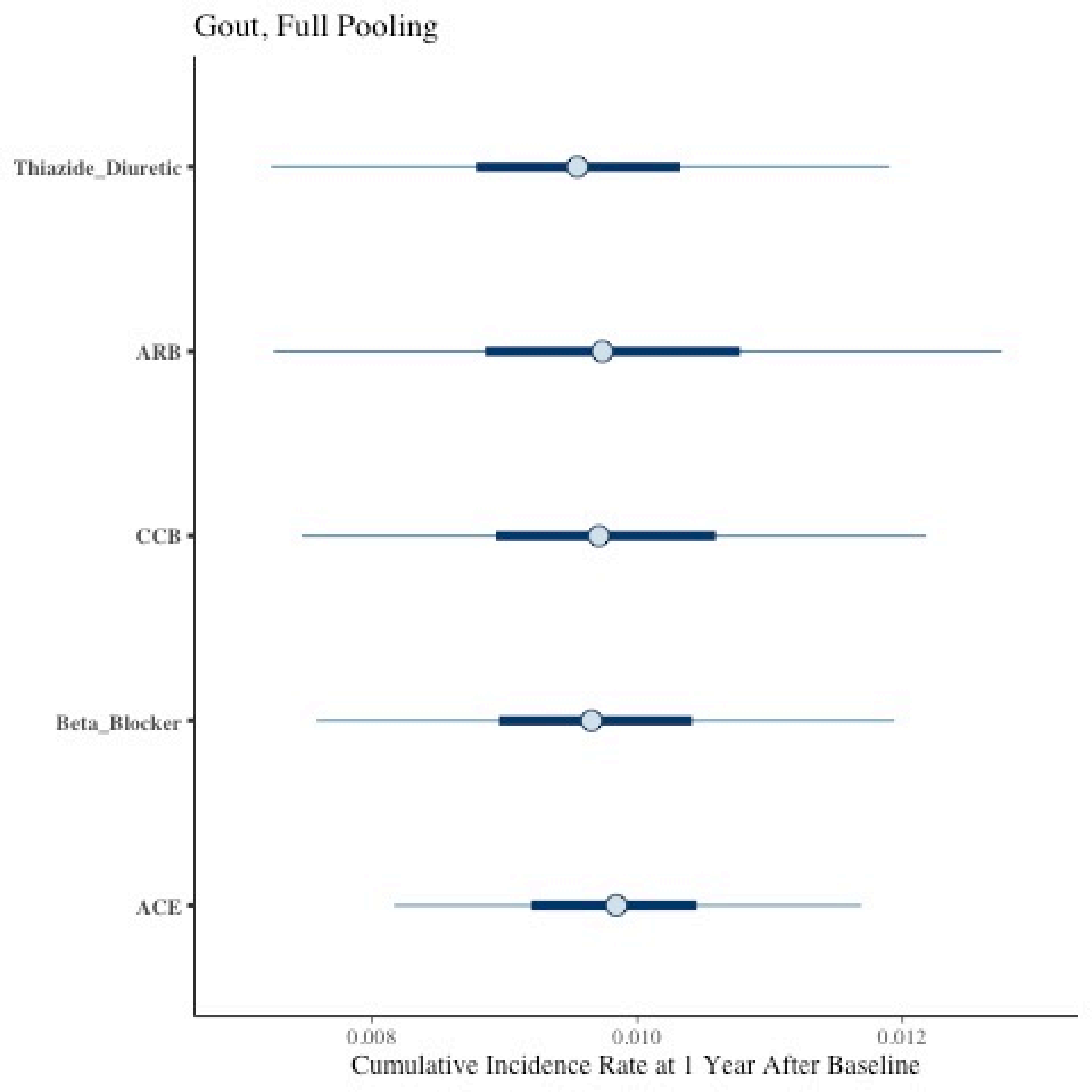

Gout, Full Pooling
Thiazide_Diuretic
ARB
CCB
Beta_Blocker
ACE
0.008
0.010
0.012
Cumulative Incidence Rate at 1 Year After Baseline

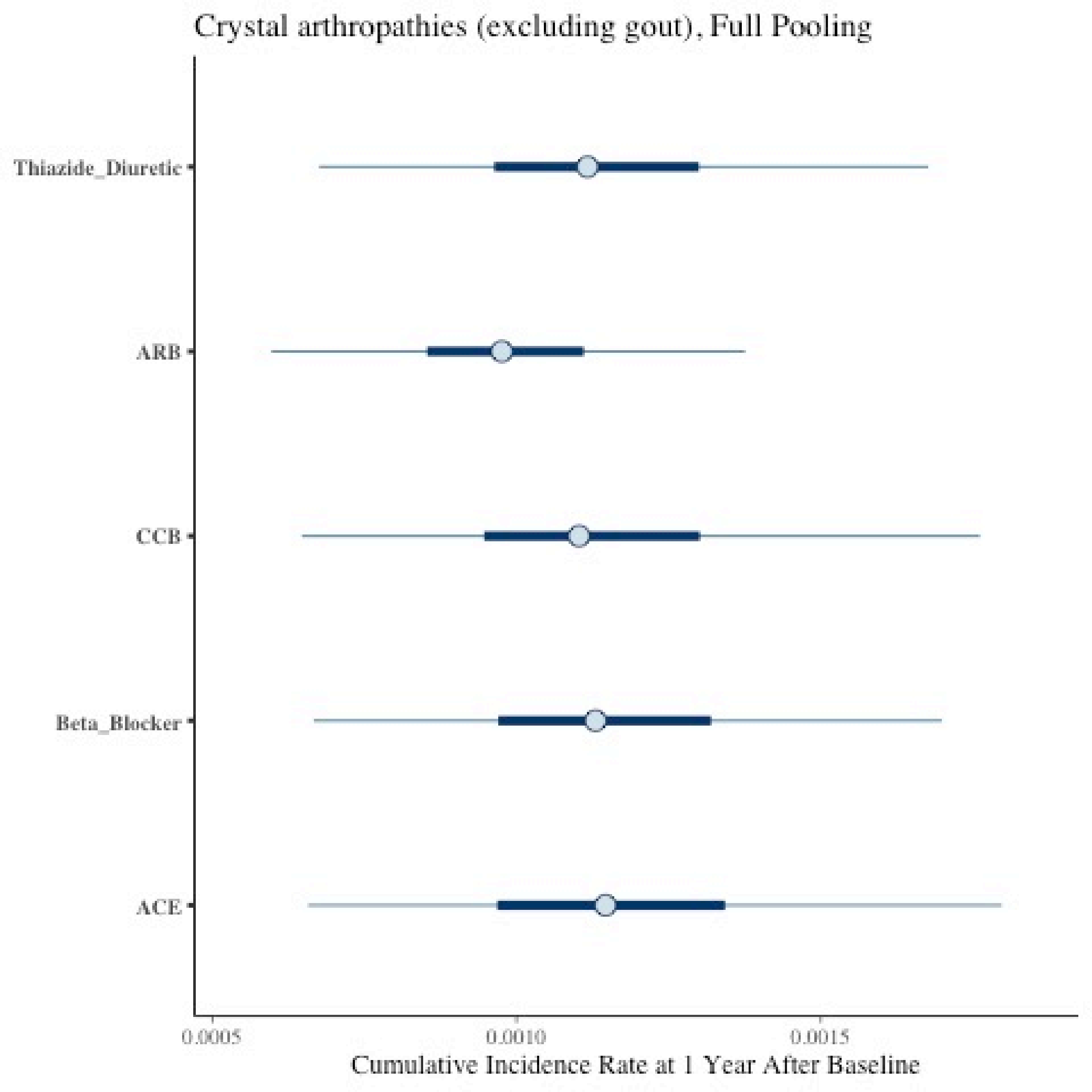

Crystal arthropathies (excluding gout), Full Pooling
Thiazide_Diuretic
ARB
CCB
Beta_Blocker
ACE
0.0005
0.0010
0.0015
Cumulative Incidence Rate at 1 Year After Baseline

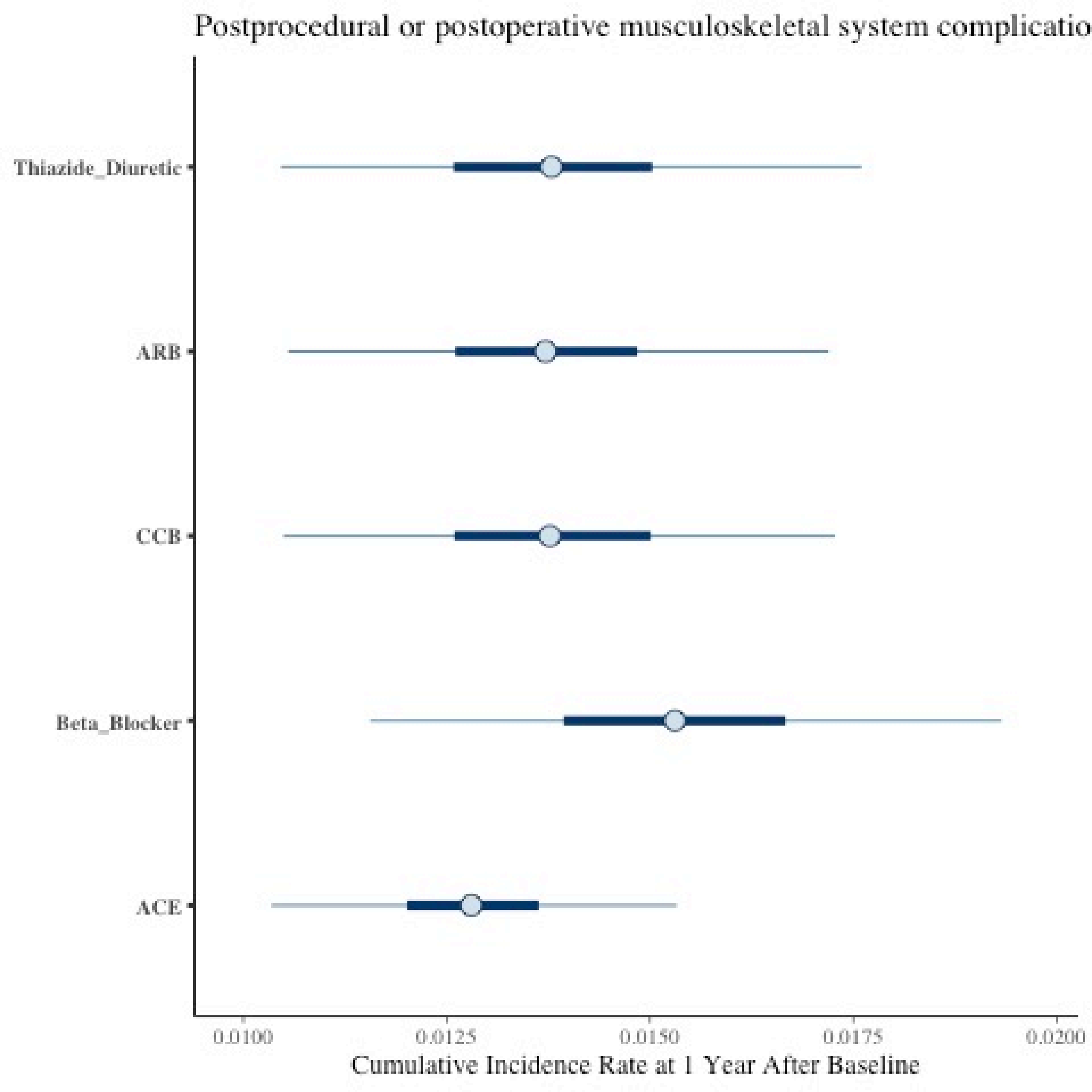

Postprocedural or postoperative musculoskeletal system complicatio
Thiazide_Diuretic
ARB
CCB
Beta_Blocker
ACE
0.0100
0.0125
0.0150
0.0175
0.0200
Cumulative Incidence Rate at 1 Year After Baseline

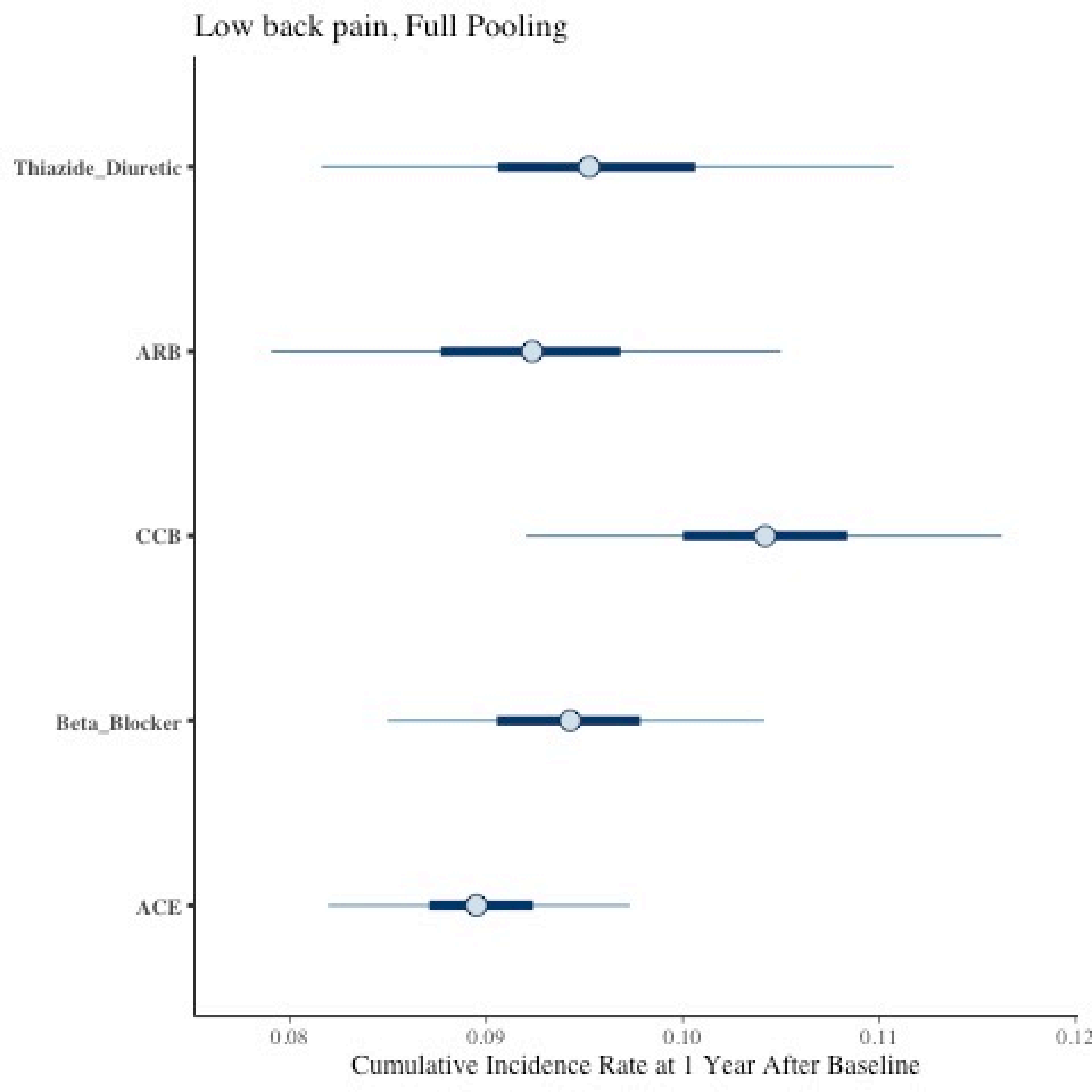

Low back pain, Full Pooling
Thiazide_Diuretic
ARB
CCB
Beta_Blocker
ACE
0.08
0.09
0.10
0.11
0.12
Cumulative Incidence Rate at 1 Year After Baseline

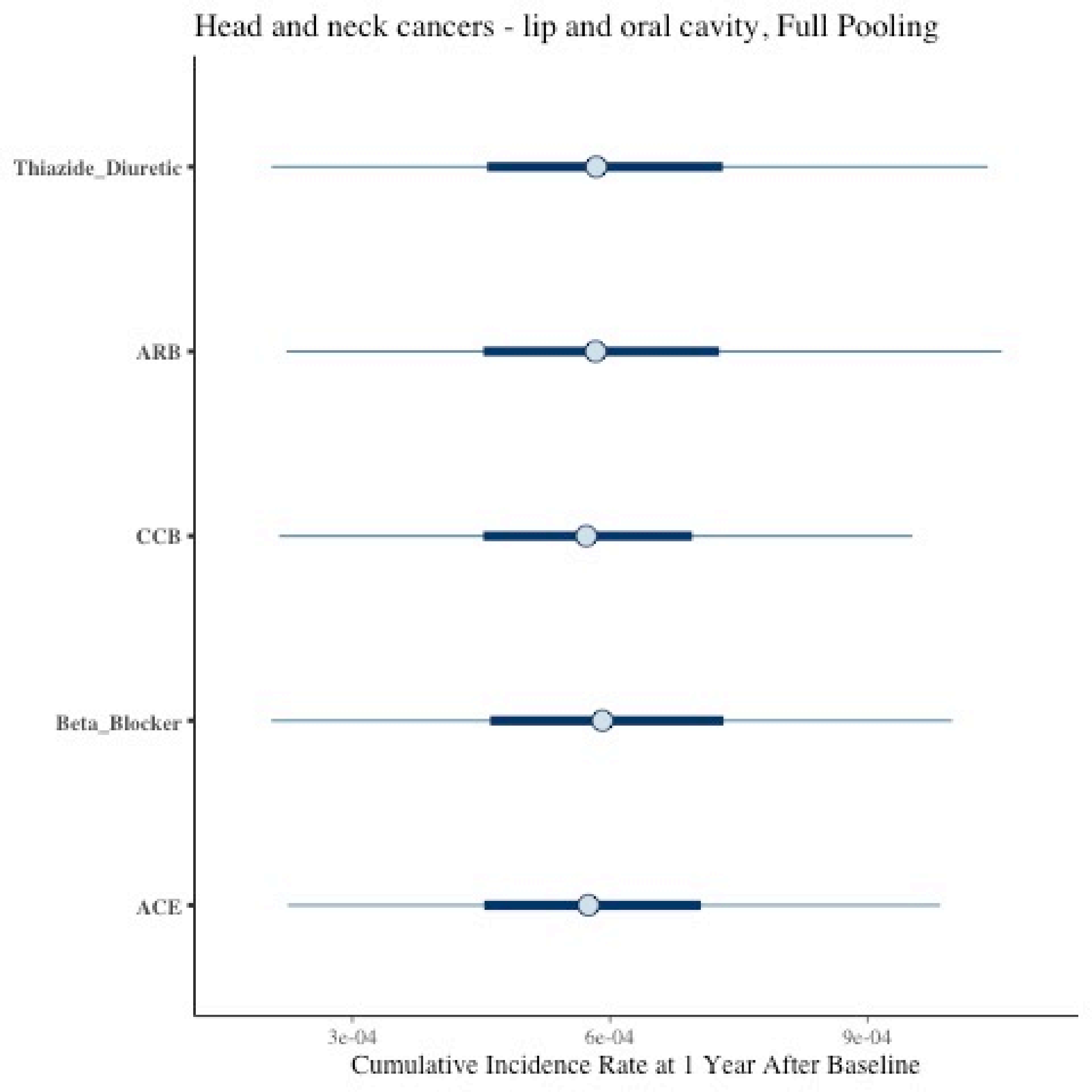

Head and neck cancers - lip and oral cavity, Full Pooling
Thiazide_Diuretic
ARB
CCB
Beta_Blocker
ACE
3e-04
6e-04
9e-04
Cumulative Incidence Rate at 1 Year After Baseline

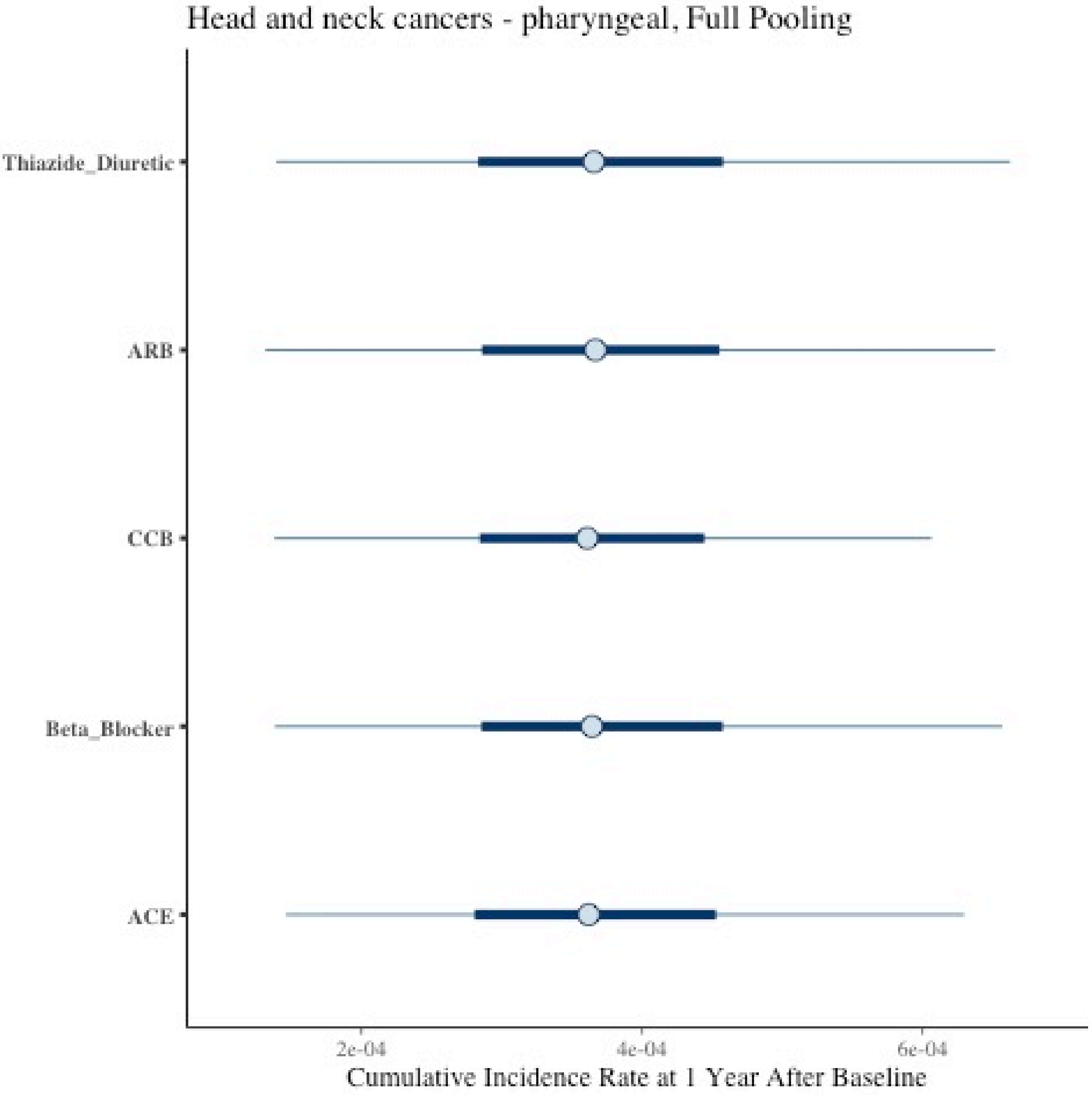

Head and neck cancers - pharyngeal, Full Pooling
Thiazide_Diuretic
ARB
CCB
Beta_Blocker
ACE
2e-04
4e-04
6e-04
Cumulative Incidence Rate at 1 Year After Baseline

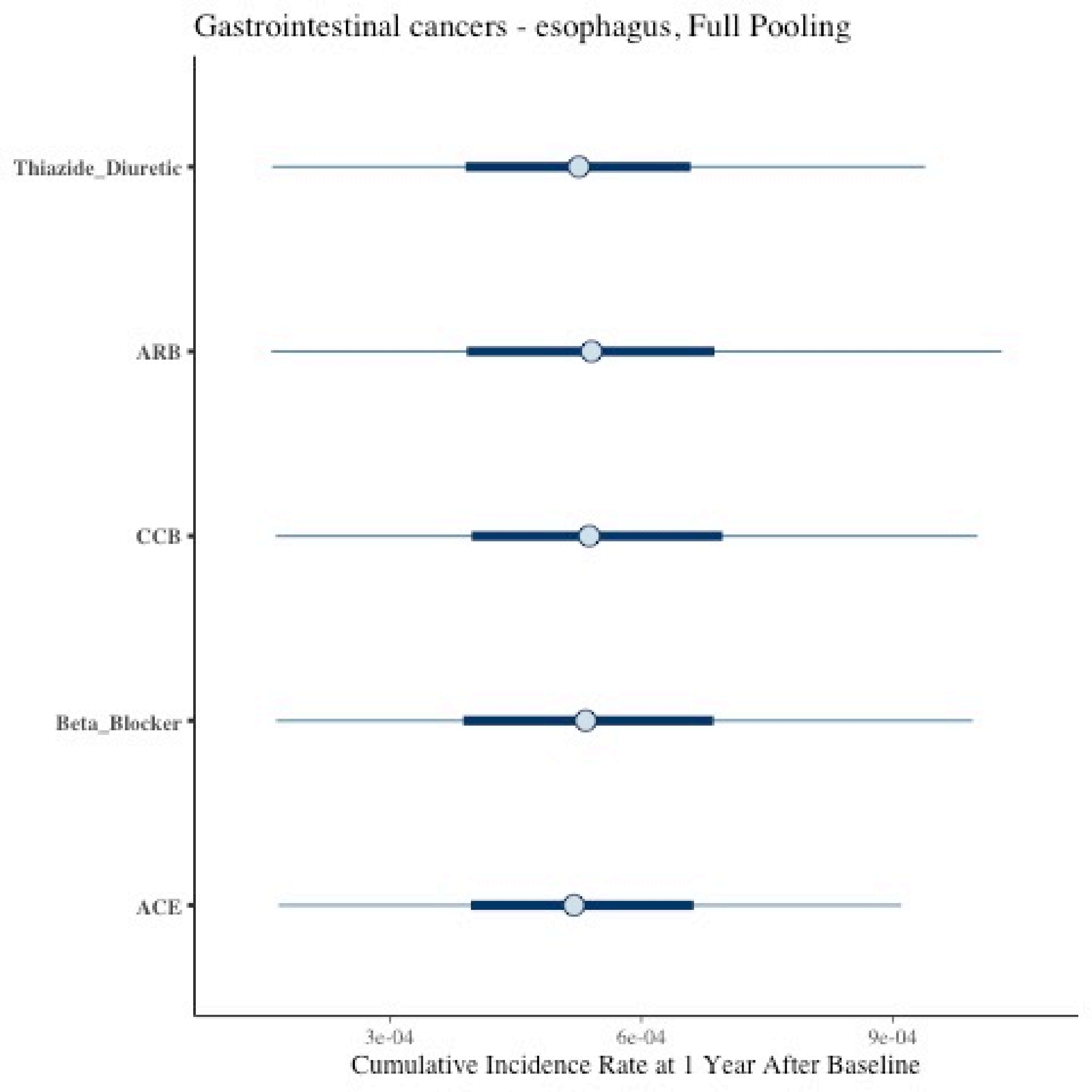

Gastrointestinal cancers - esophagus, Full Pooling
Thiazide_Diuretic
ARB
CCB
Beta_Blocker
ACE
3e-04
6e-04
9e-04
Cumulative Incidence Rate at 1 Year After Baseline

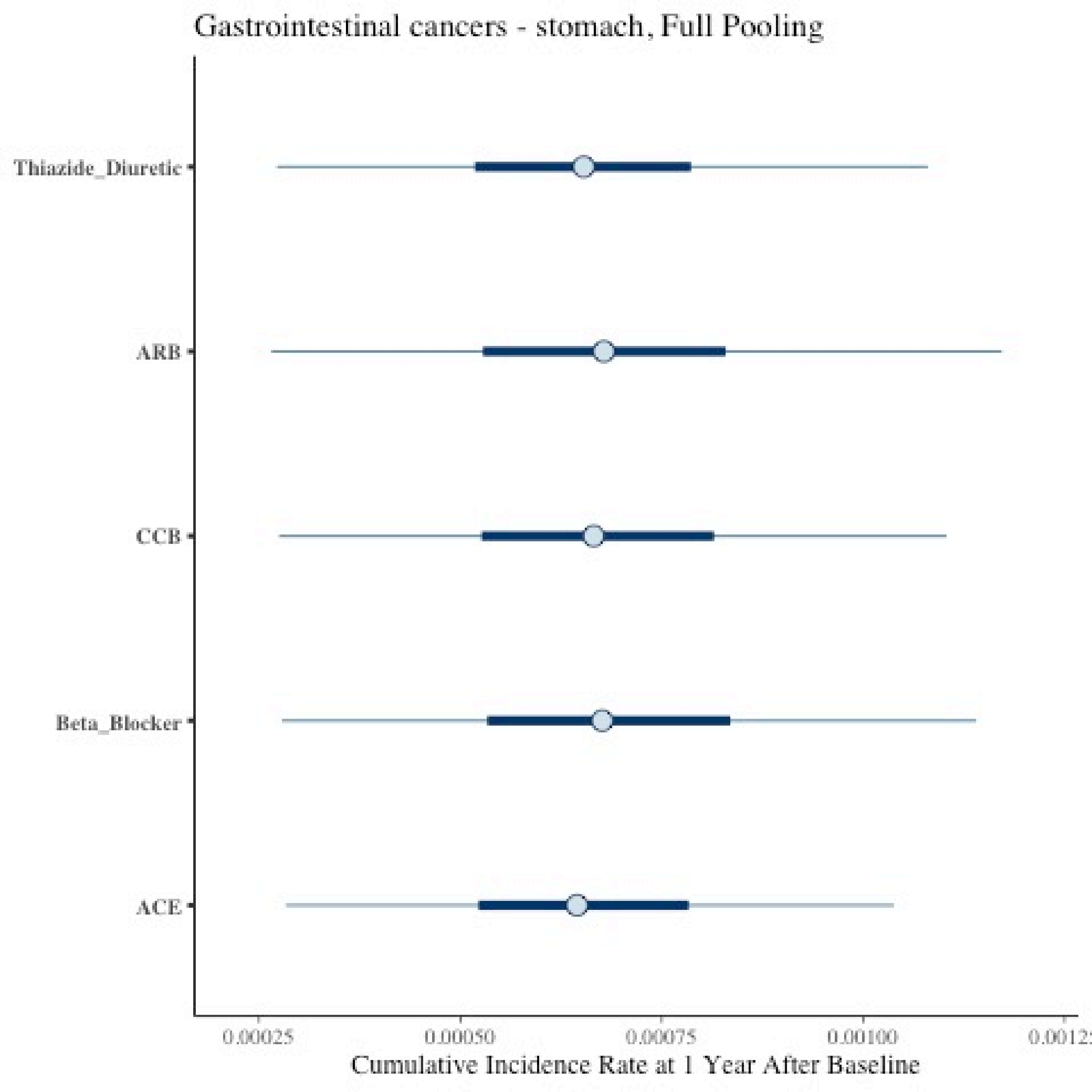

Gastrointestinal cancers - stomach, Full Pooling
Thiazide_Diuretic
ARB
CCB
Beta_Blocker
ACE
0.00025
0.00050
0.00075
0.00100
Cumulative Incidence Rate at 1 Year After Baseline

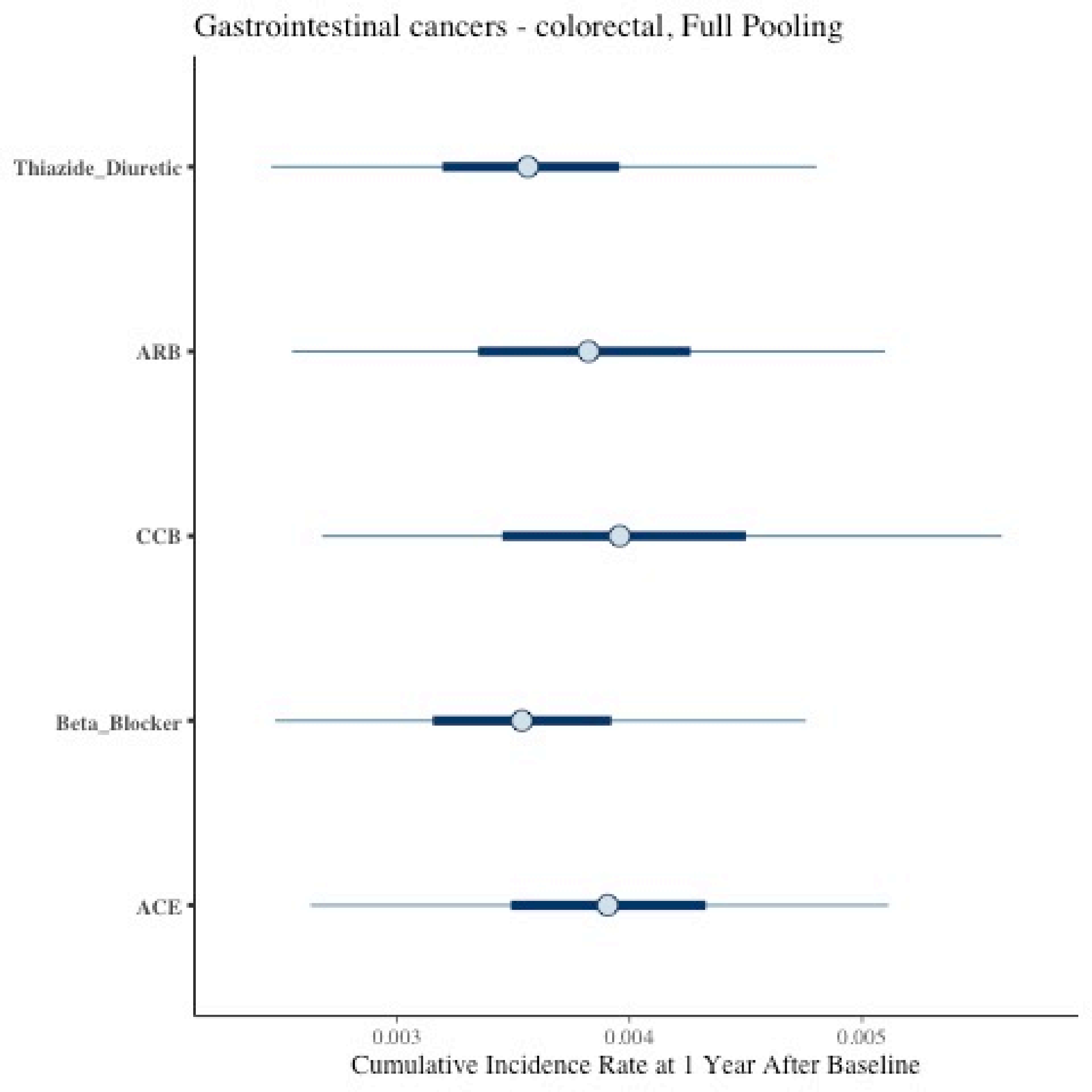

Gastrointestinal cancers - colorectal, Full Pooling
Thiazide_Diuretic
ARB
CCB
Beta_Blocker
ACE
0.003
0.004
0.005
Cumulative Incidence Rate at 1 Year After Baseline

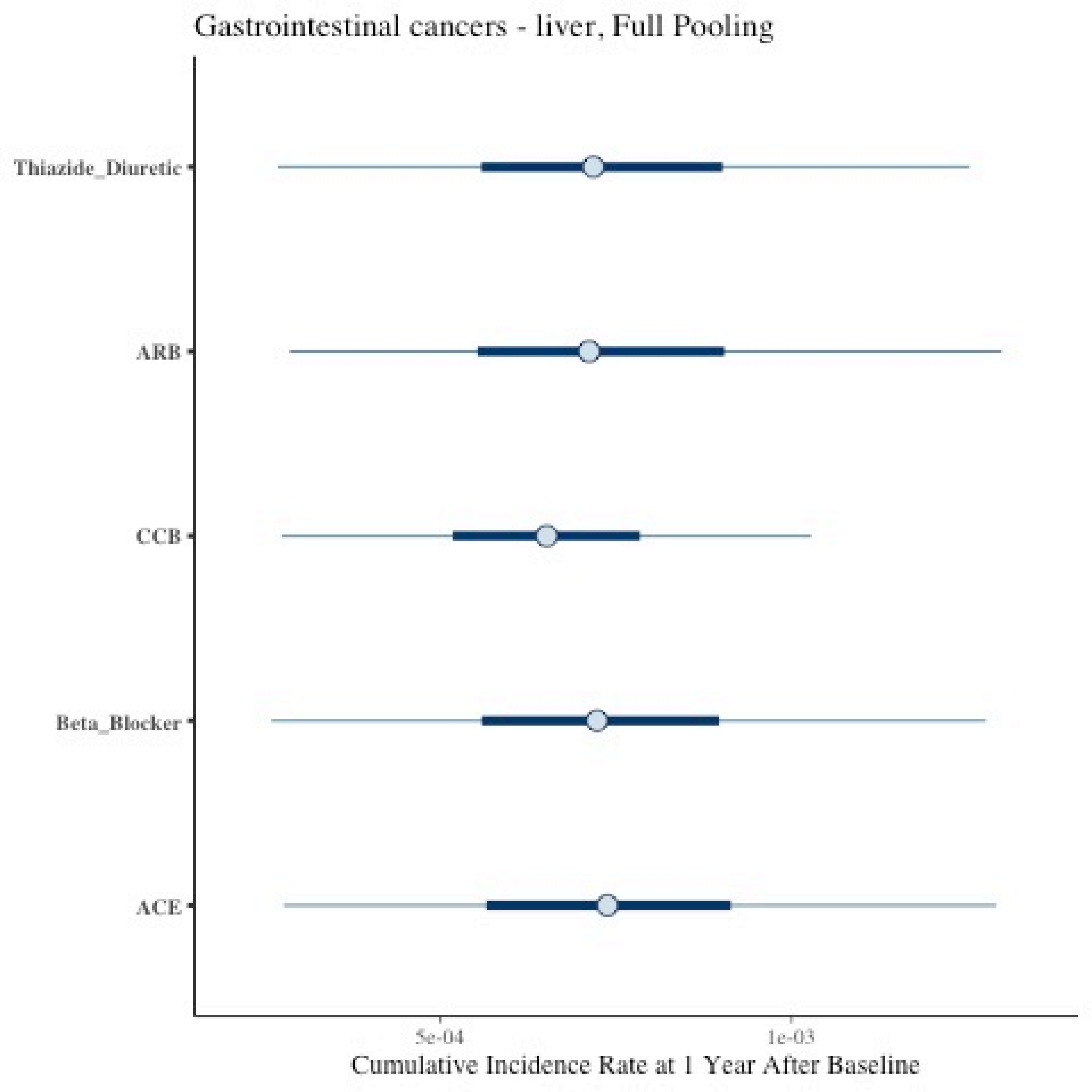
Gastrointestinal cancers - liver, Full Pooling
Thiazide_Diuretic
ARB
CCB
Beta_Blocker
ACE
5e-04
1e-03
Cumulative Incidence Rate at 1 Year After Baseline

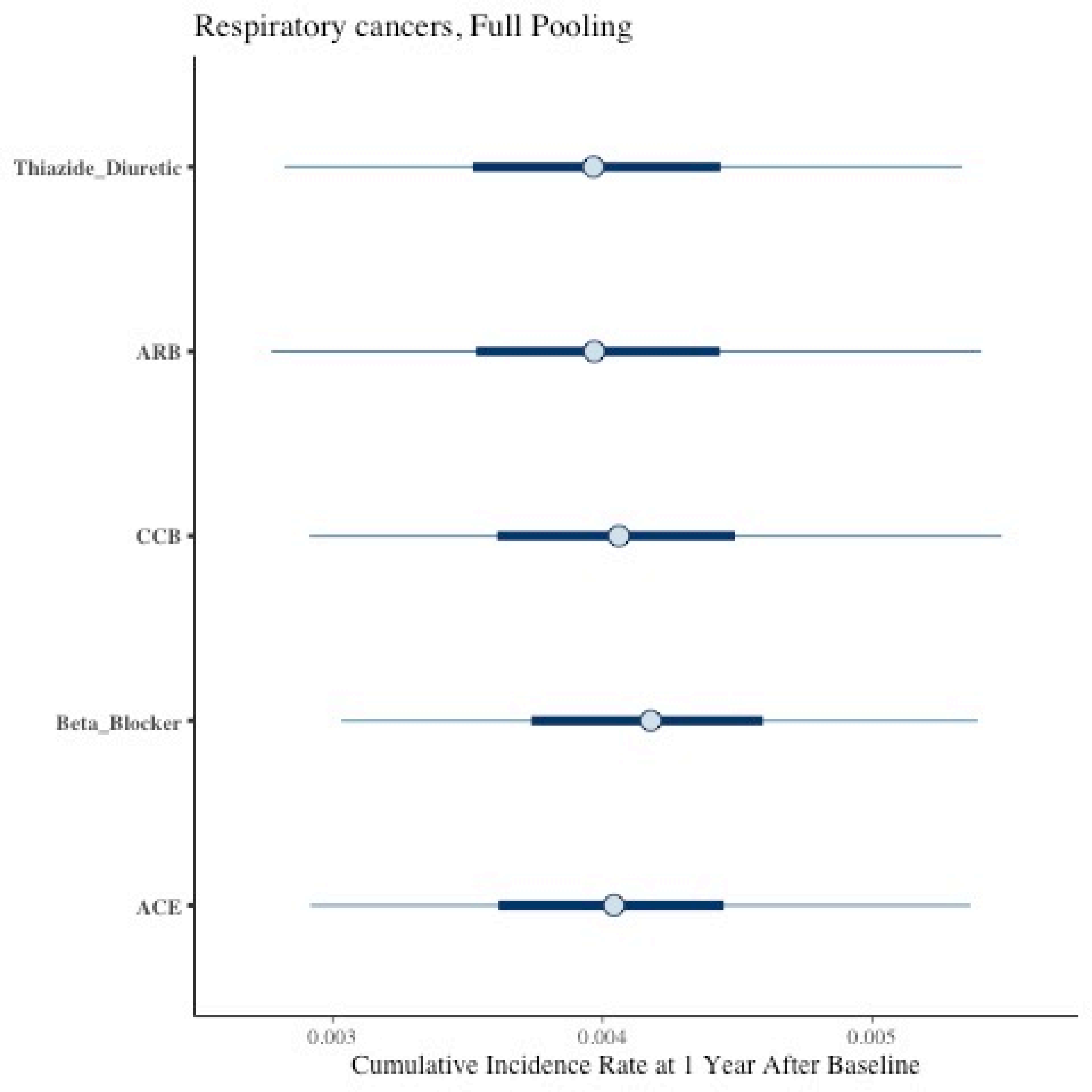
Respiratory cancers, Full Pooling
Thiazide_Diuretic
ARB
CCB
Beta_Blocker
ACE
0.003
0.004
0.005
Cumulative Incidence Rate at 1 Year After Baseline

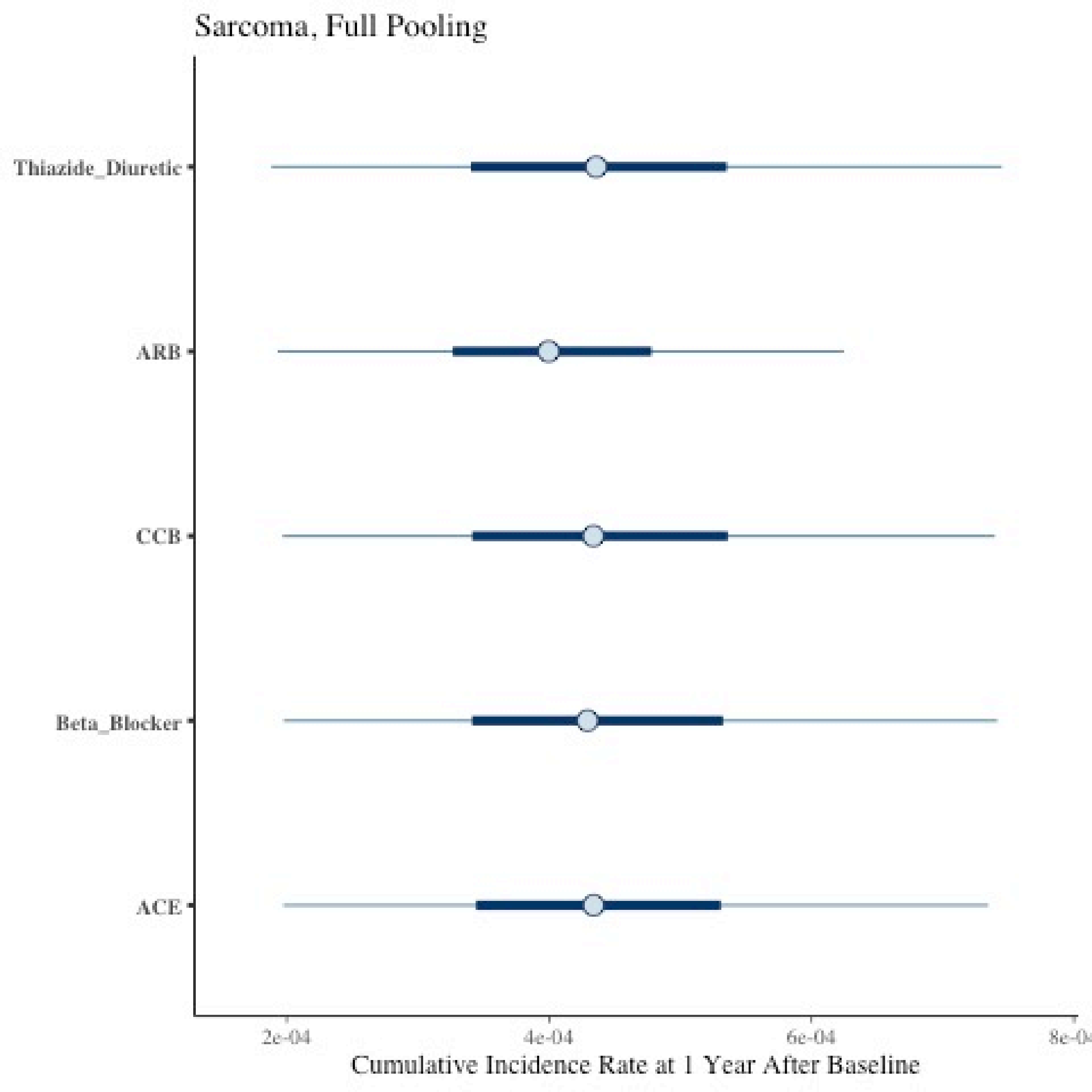

Sarcoma, Full Pooling
Thiazide_Diuretic
ARB
CCB
Beta_Blocker
ACE
2e-04
4e-04
6e-04
Cumulative Incidence Rate at 1 Year After Baseline

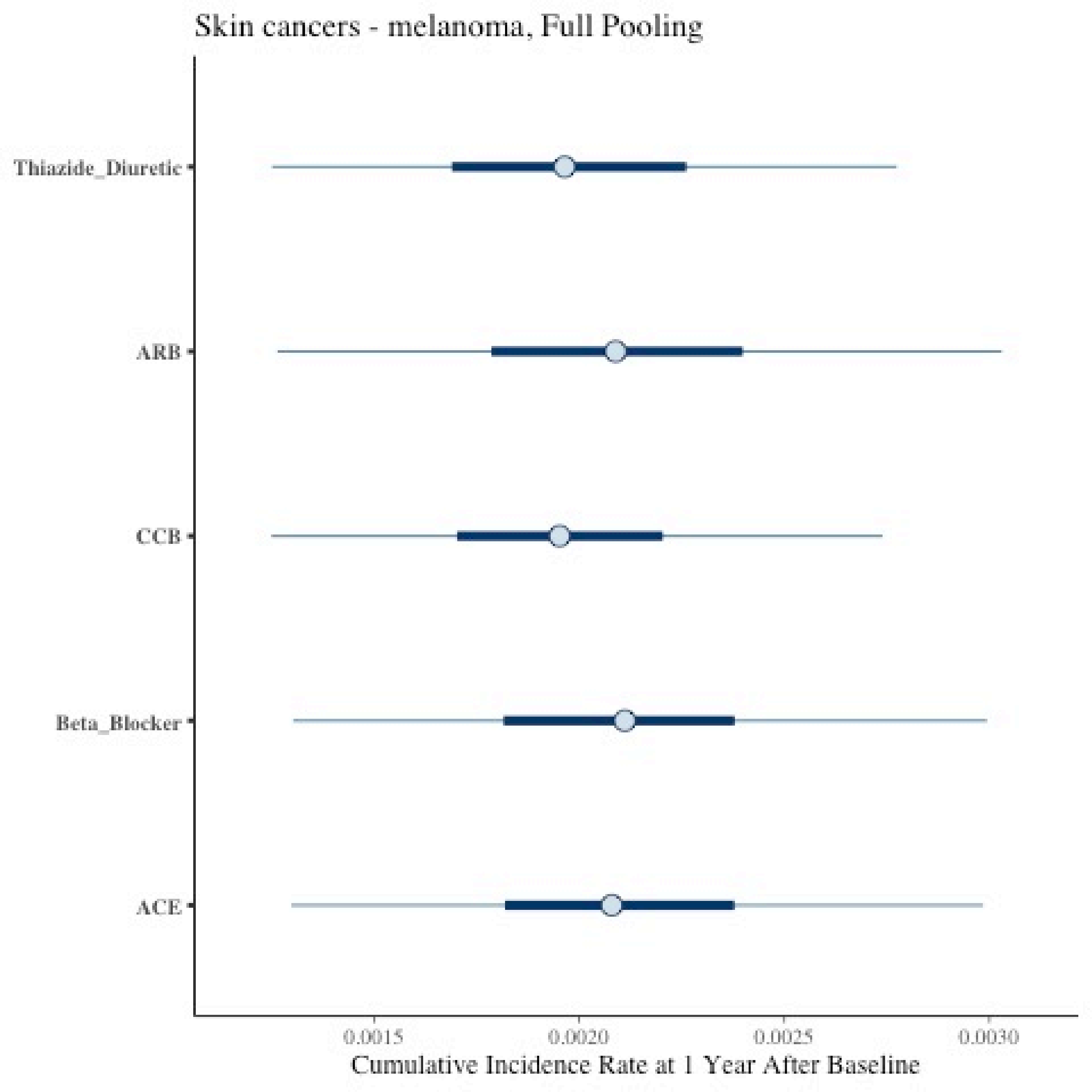
Skin cancers - melanoma, Full Pooling
Thiazide_Diuretic
ARB
CCB
Beta_Blocker
ACE
0.0015
0.0020
0.0025
0.0030
Cumulative Incidence Rate at 1 Year After Baseline

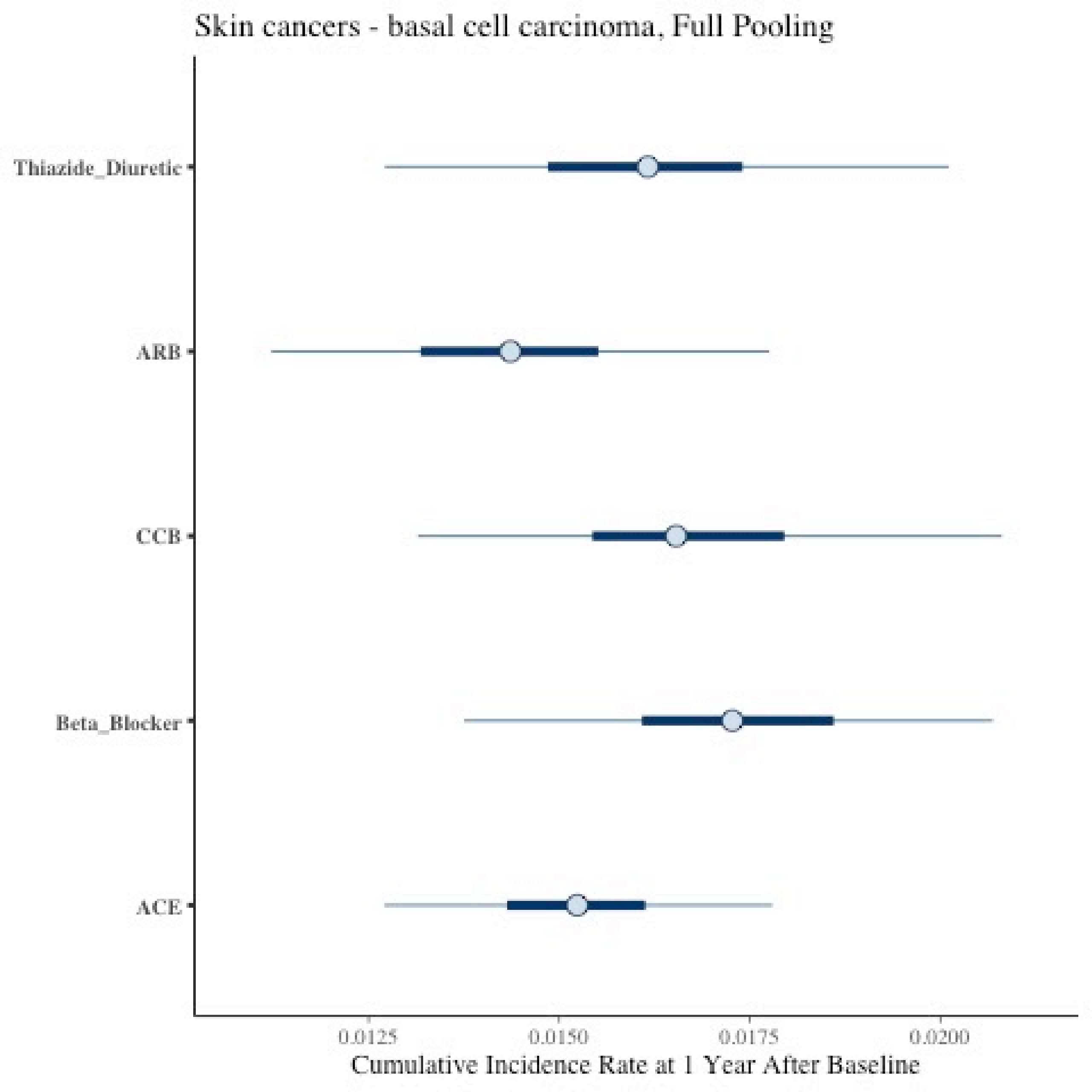

Skin cancers - basal cell carcinoma, Full Pooling
Thiazide_Diuretic
ARB
CCB
Beta_Blocker
ACE
0.0125
0.0150
0.0175
0.0200
Cumulative Incidence Rate at 1 Year After Baseline

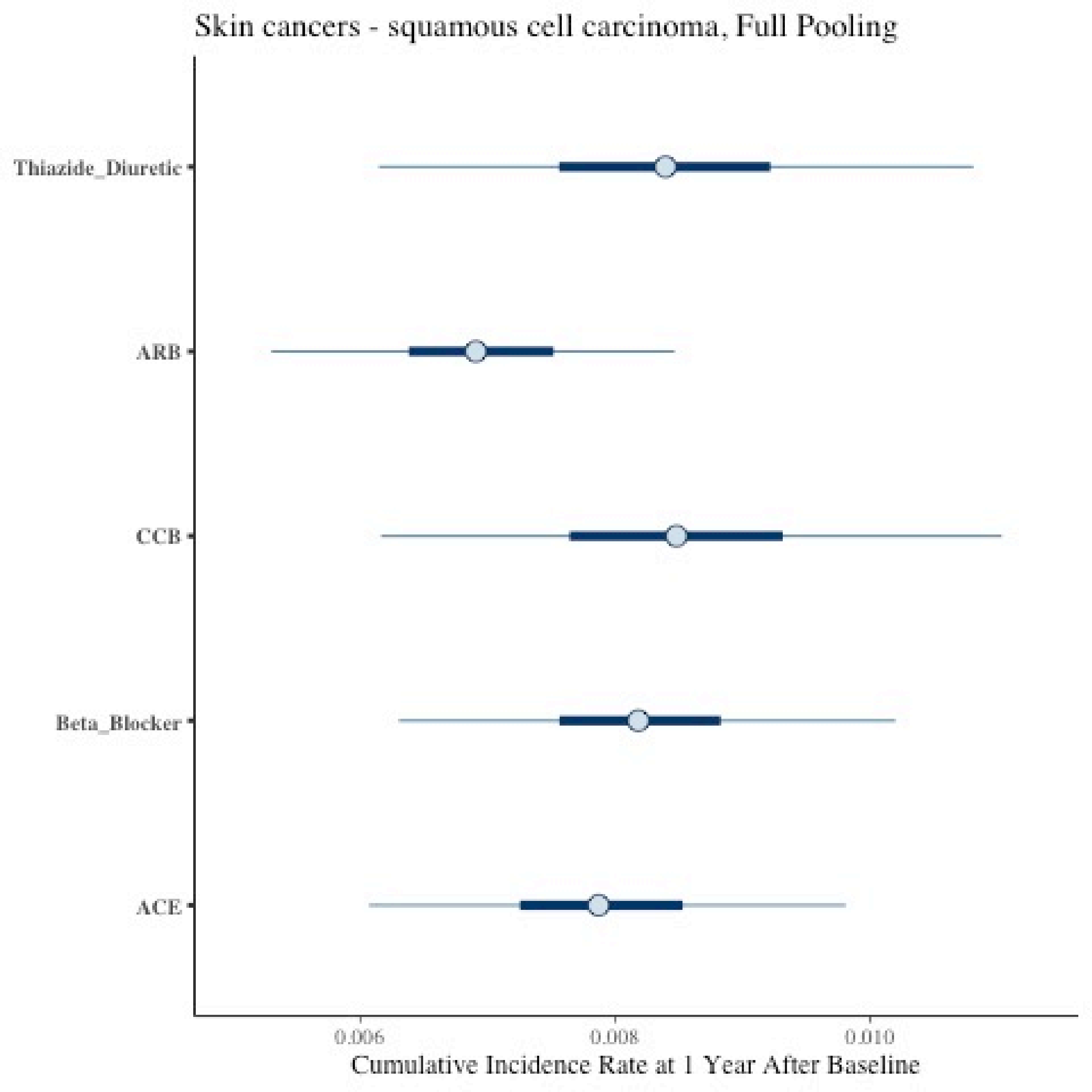

Skin cancers - squamous cell carcinoma, Full Pooling
Thiazide_Diuretic
ARB
CCB
Beta_Blocker
ACE
0.006
0.008
0.010
Cumulative Incidence Rate at 1 Year After Baseline

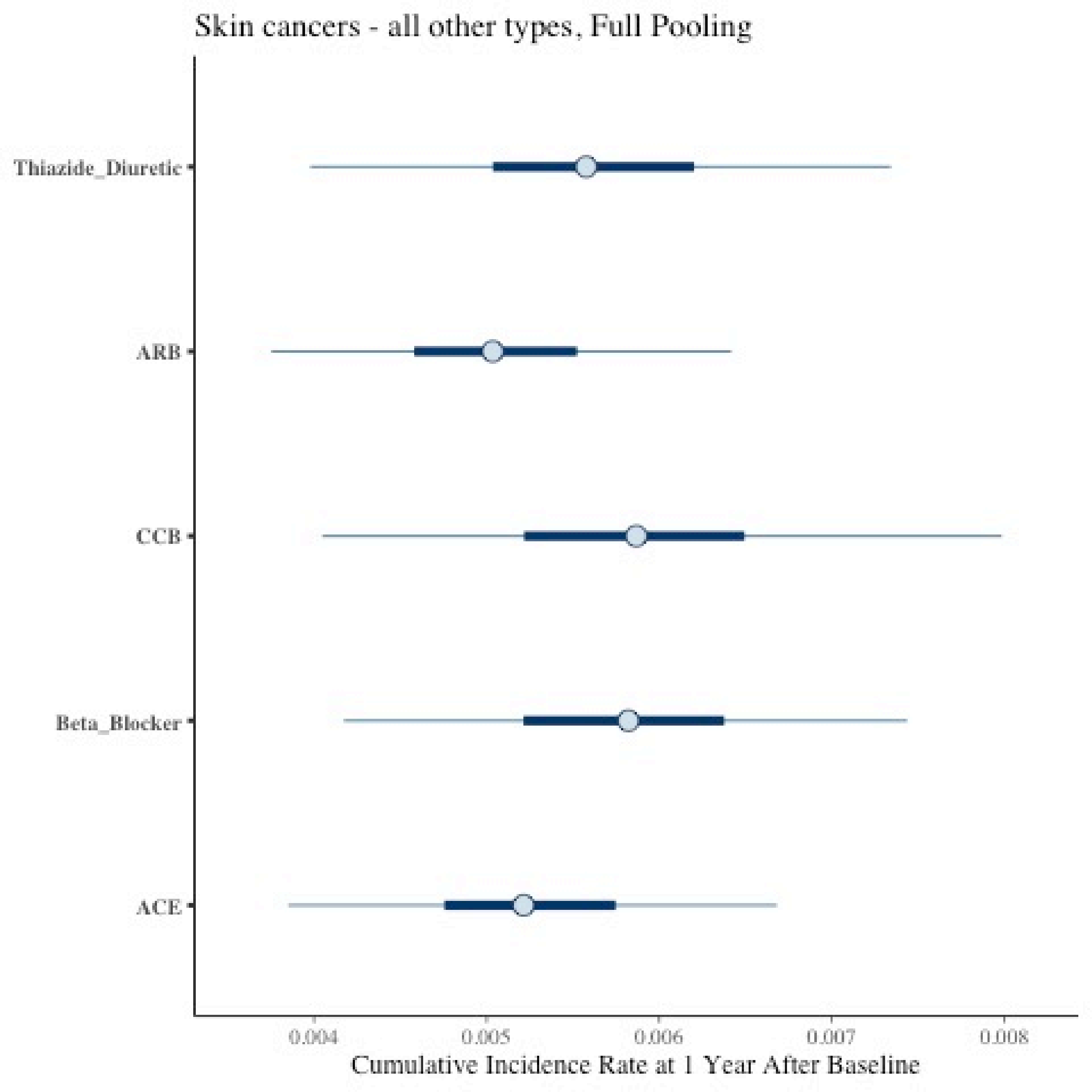

Skin cancers - all other types, Full Pooling
Thiazide_Diuretic
ARB
CCB
Beta_Blocker
ACE
0.004
0.005
0.006
0.007
0.008
Cumulative Incidence Rate at 1 Year After Baseline

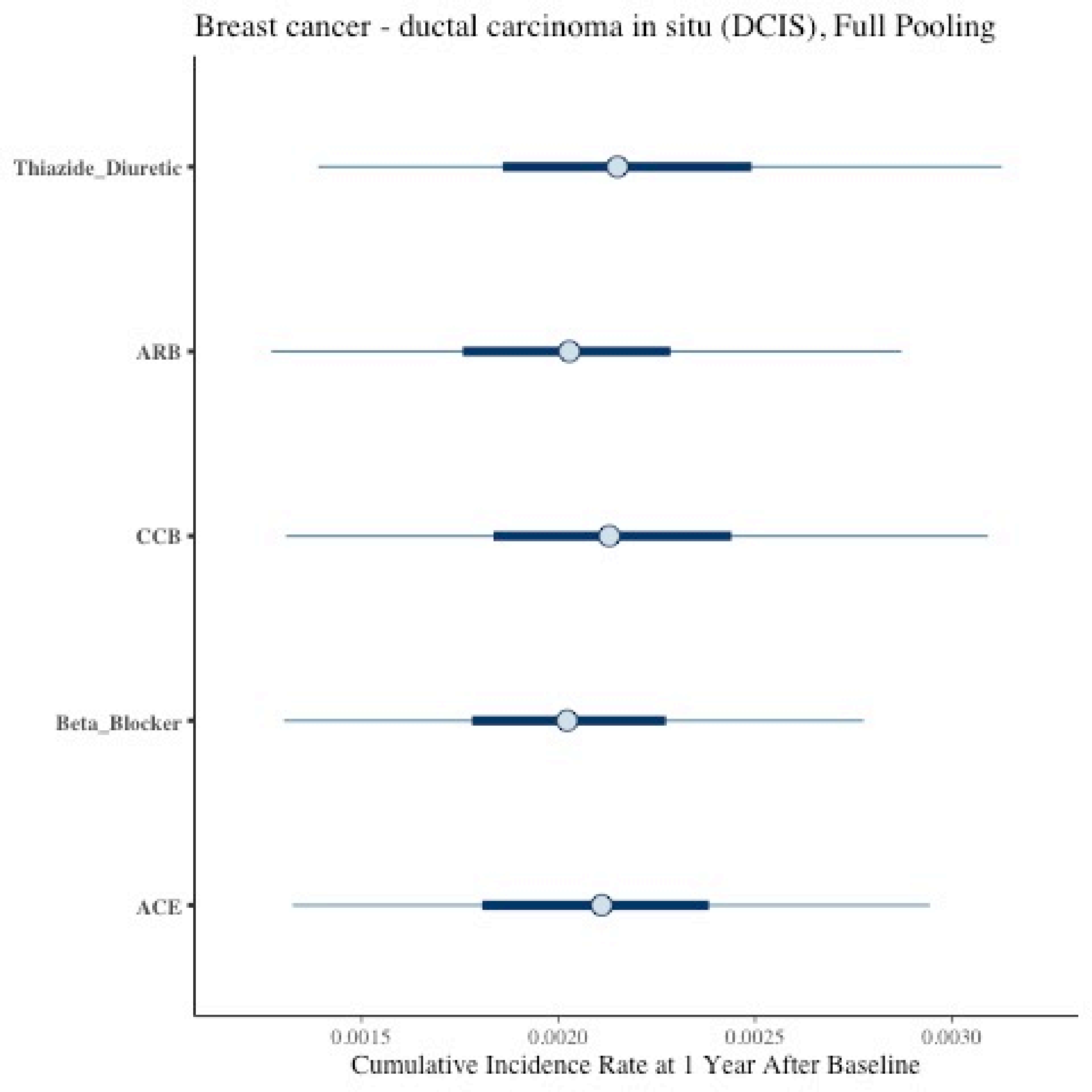

Breast cancer - ductal carcinoma in situ (DCIS), Full Pooling
Thiazide_Diuretic
ARB
CCB
Beta_Blocker
ACE
0.0015
0.0020
0.0025
0.0030
Cumulative Incidence Rate at 1 Year After Baseline

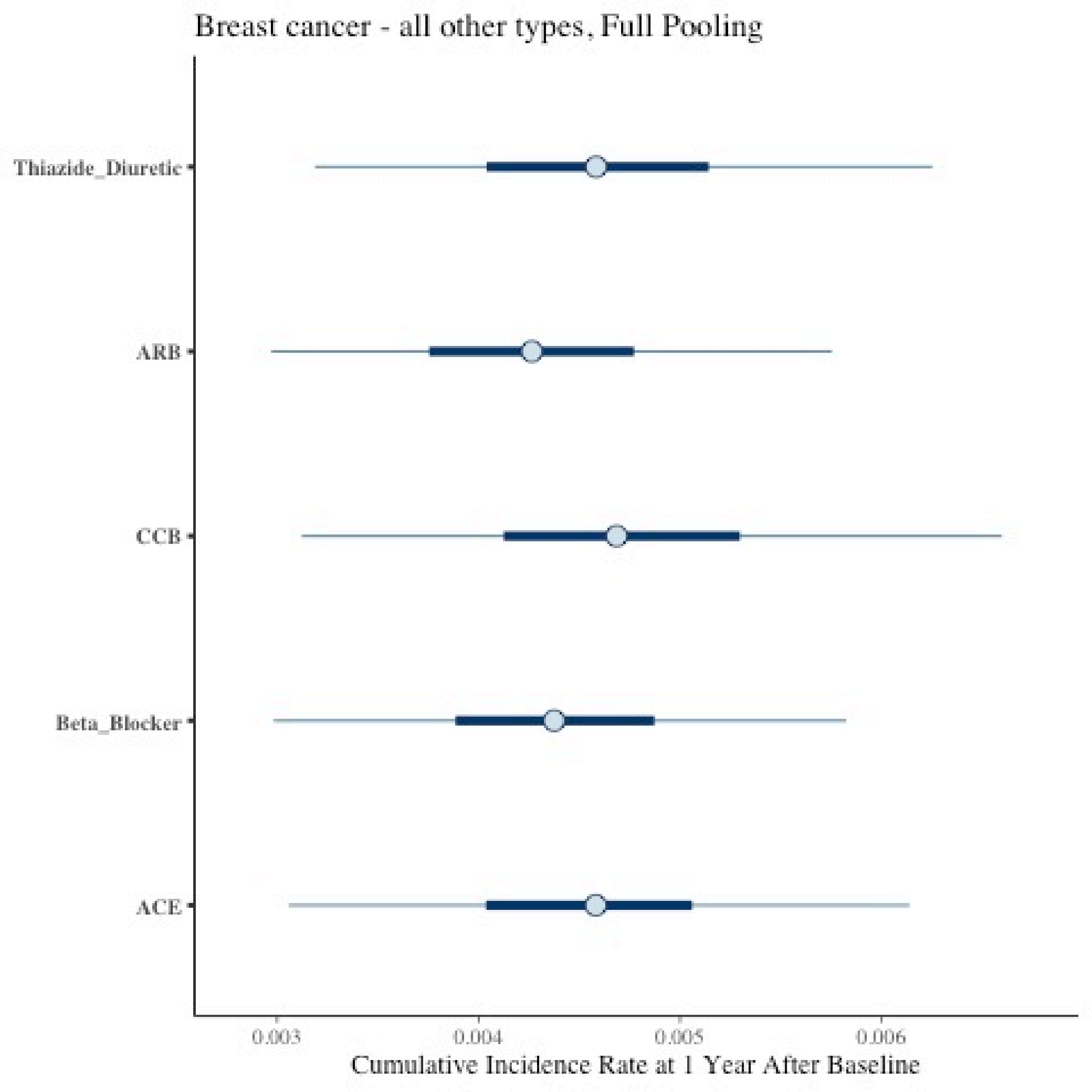
Breast cancer - all other types, Full Pooling
Thiazide_Diuretic
ARB
CCB
Beta_Blocker
ACE
0.003
0.004
0.005
0.006
Cumulative Incidence Rate at 1 Year After Baseline

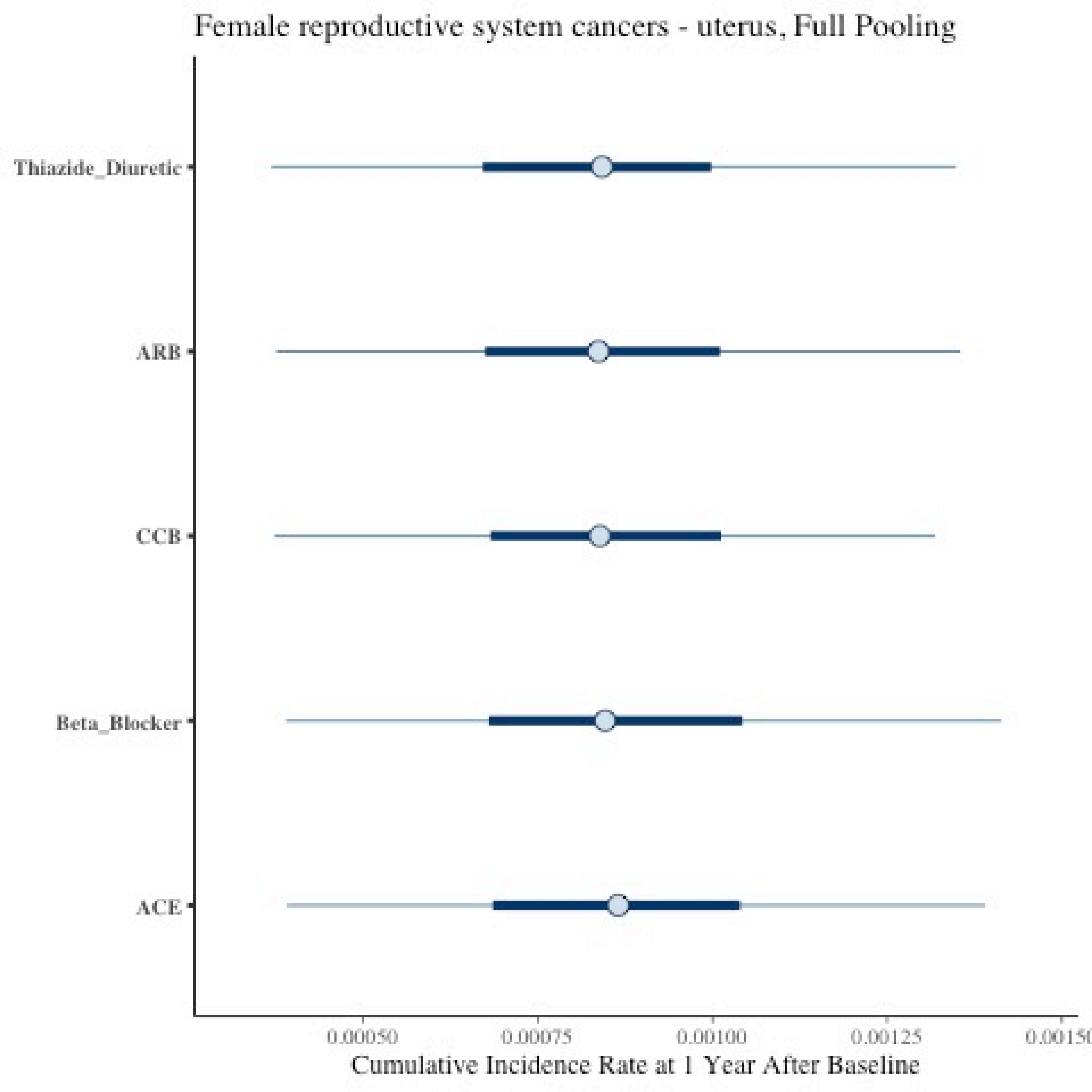

Female reproductive system cancers - uterus, Full Pooling
Thiazide_Diuretic
ARB
CCB
Beta_Blocker
ACE
0.00050
0.00075
0.00100
0.00125
0.00150
Cumulative Incidence Rate at 1 Year After Baseline

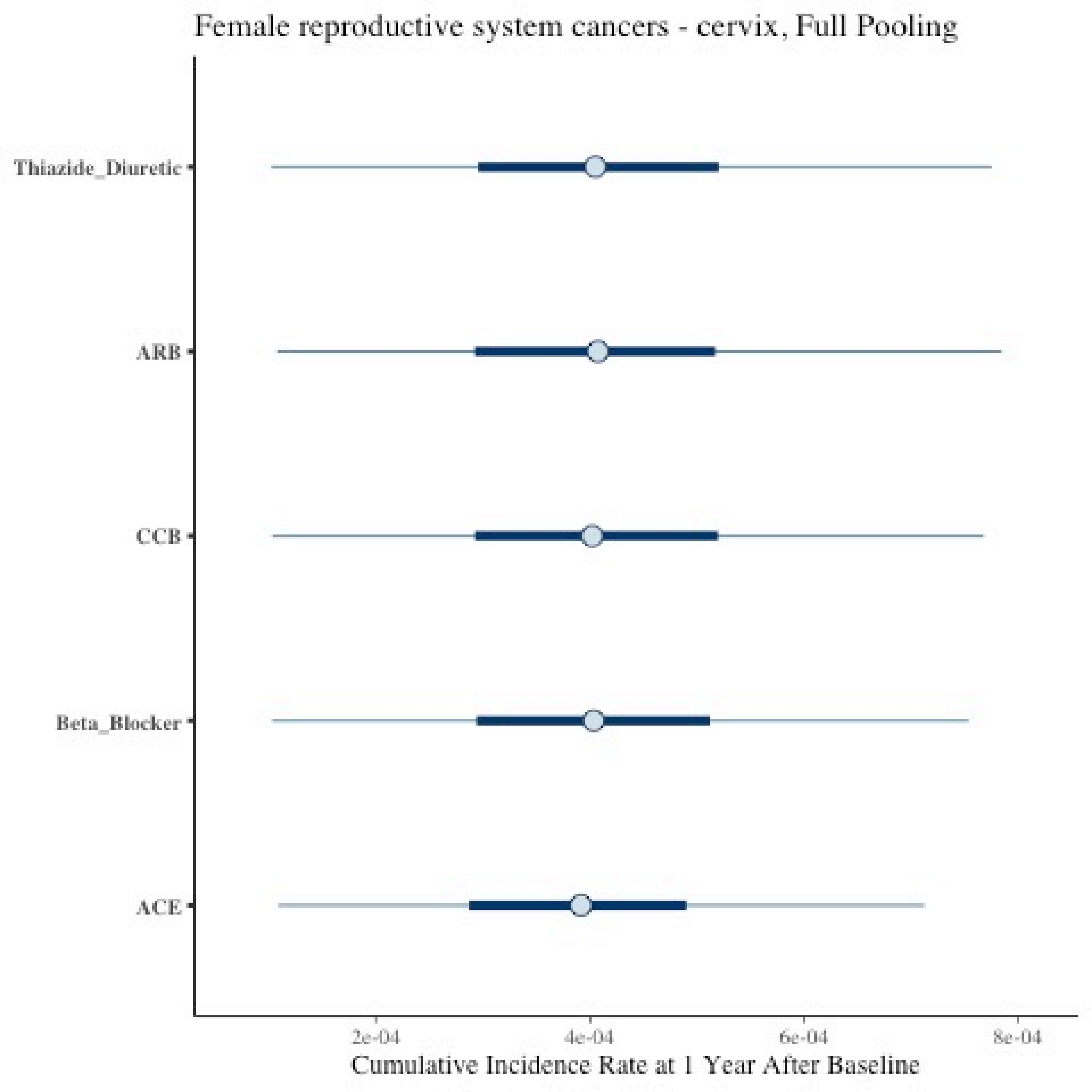

Female reproductive system cancers - cervix, Full Pooling
Thiazide_Diuretic
ARB
CCB
Beta_Blocker
ACE
2e-04
4e-04
6e-04
8e-04
Cumulative Incidence Rate at 1 Year After Baseline

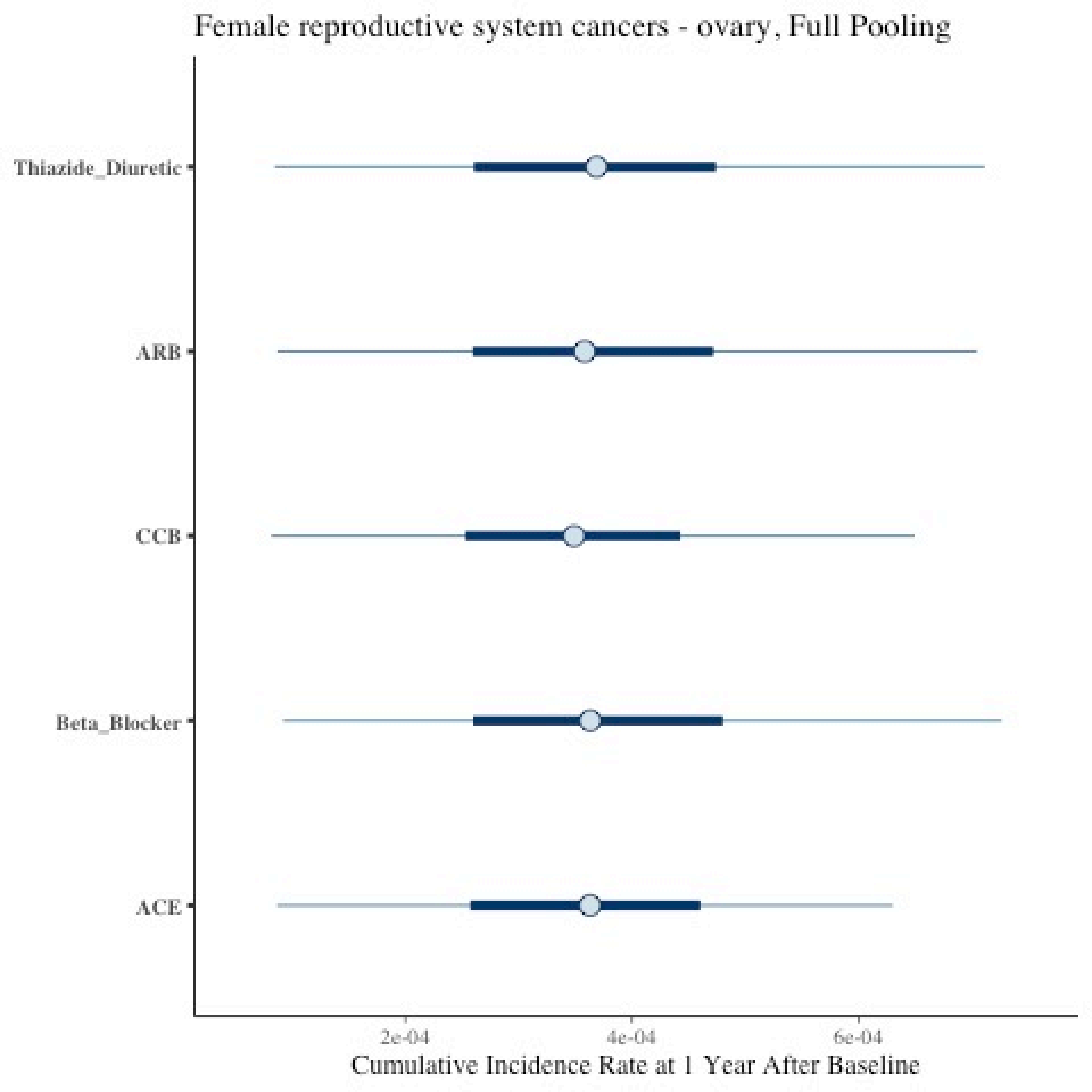

Female reproductive system cancers - ovary, Full Pooling
Thiazide_Diuretic
ARB
CCB
Beta_Blocker
ACE
2e-04
4e-04
6e-04
Cumulative Incidence Rate at 1 Year After Baseline

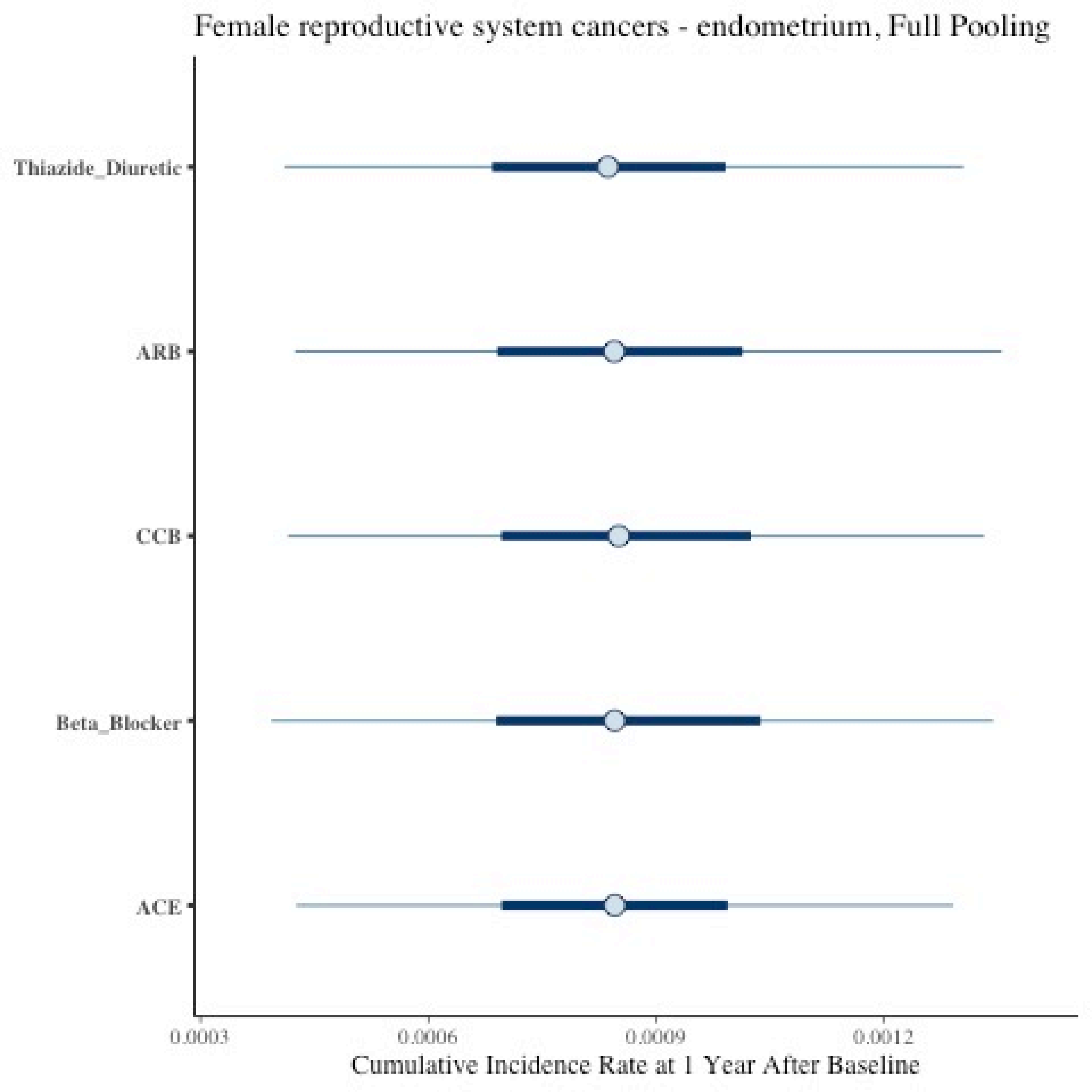

Female reproductive system cancers - endometrium, Full Pooling
Thiazide_Diuretic
ARB
CCB
Beta_Blocker
ACE
0.0003
0.0006
0.0009
0.0012
Cumulative Incidence Rate at 1 Year After Baseline

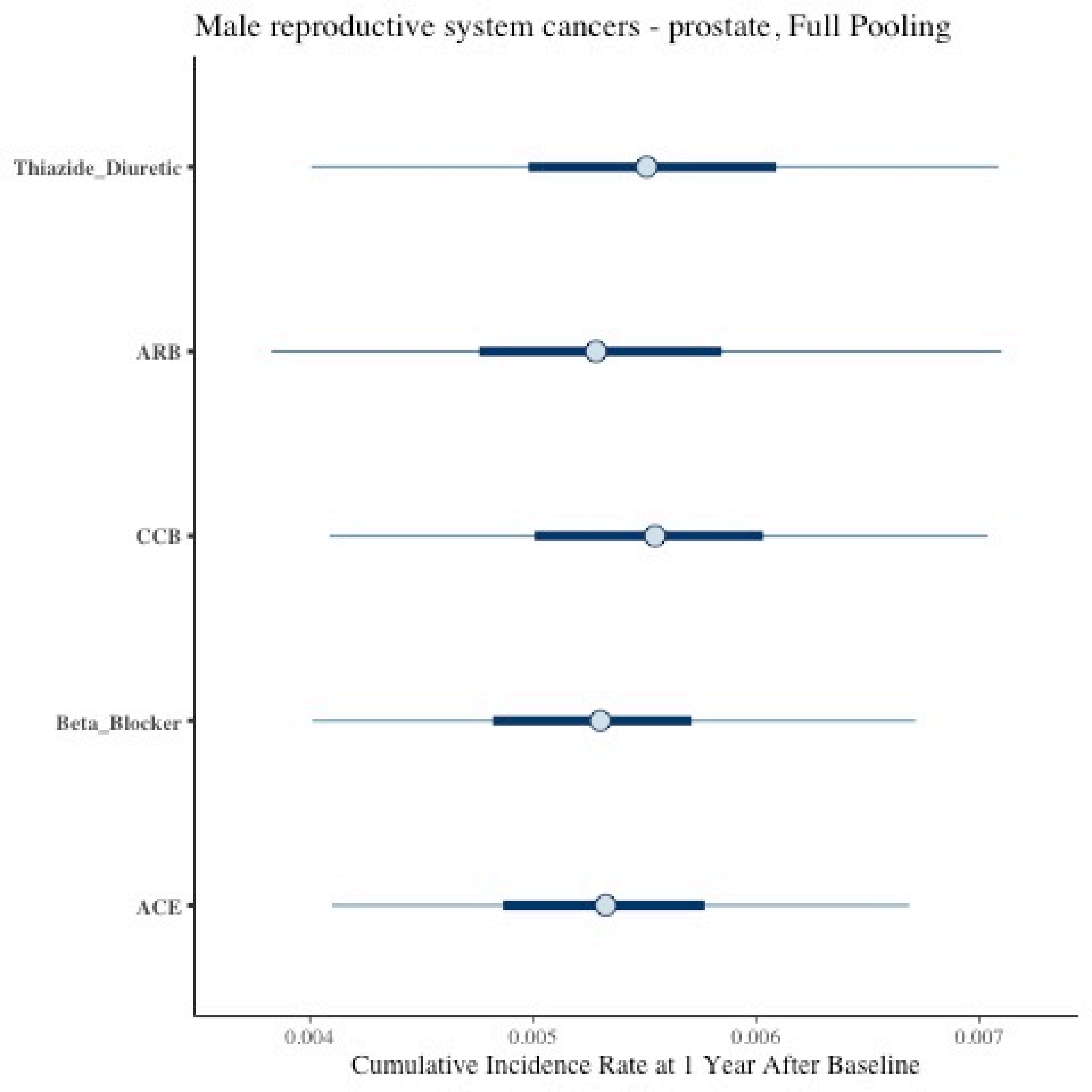
Male reproductive system cancers - prostate, Full Pooling
Thiazide_Diuretic
ARB
CCB
Beta_Blocker
ACE
0.004
0.005
0.006
0.007
Cumulative Incidence Rate at 1 Year After Baseline

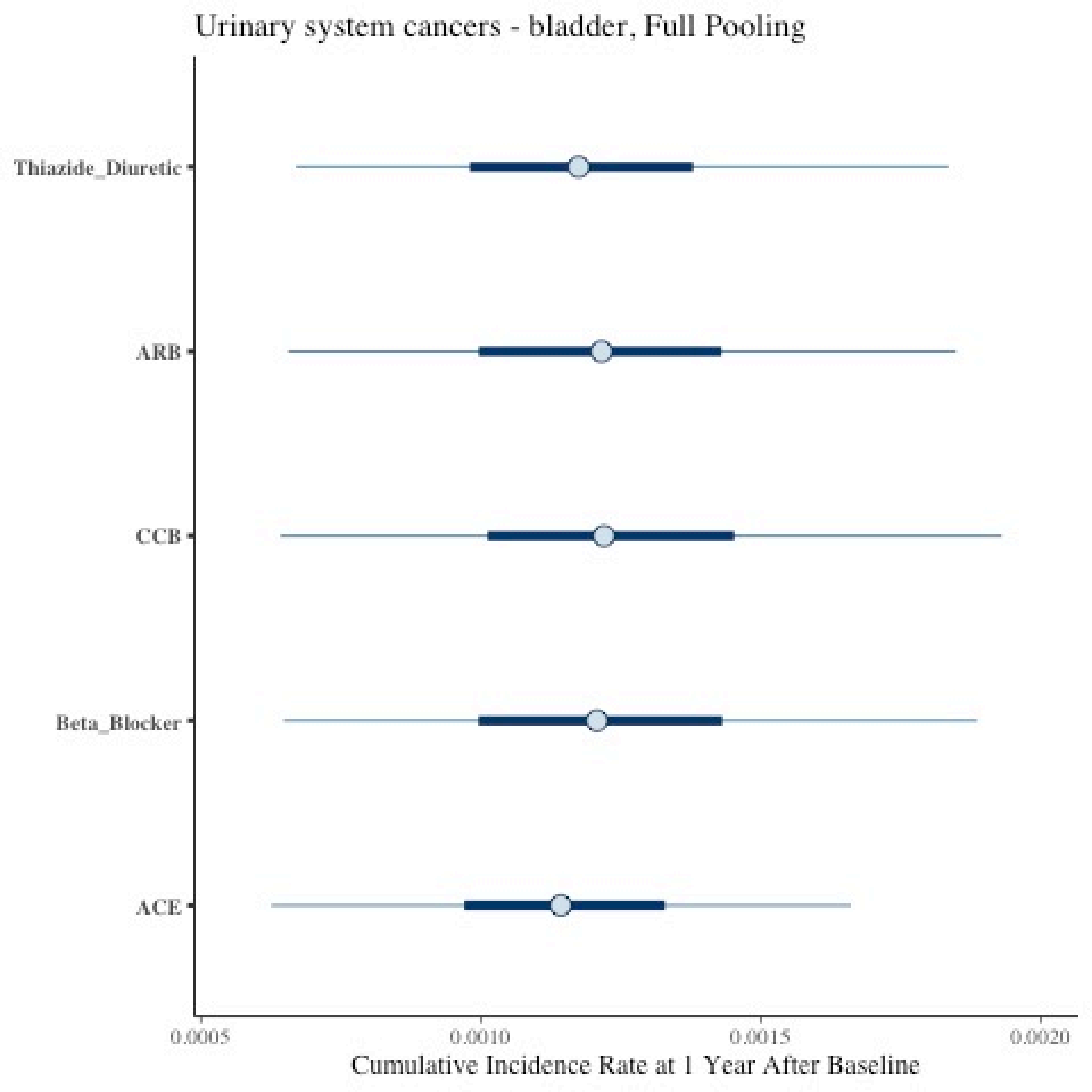

Urinary system cancers - bladder, Full Pooling
Thiazide_Diuretic
ARB
CCB
Beta_Blocker
ACE
0.0005
0.0010
0.0015
0.0020
Cumulative Incidence Rate at 1 Year After Baseline

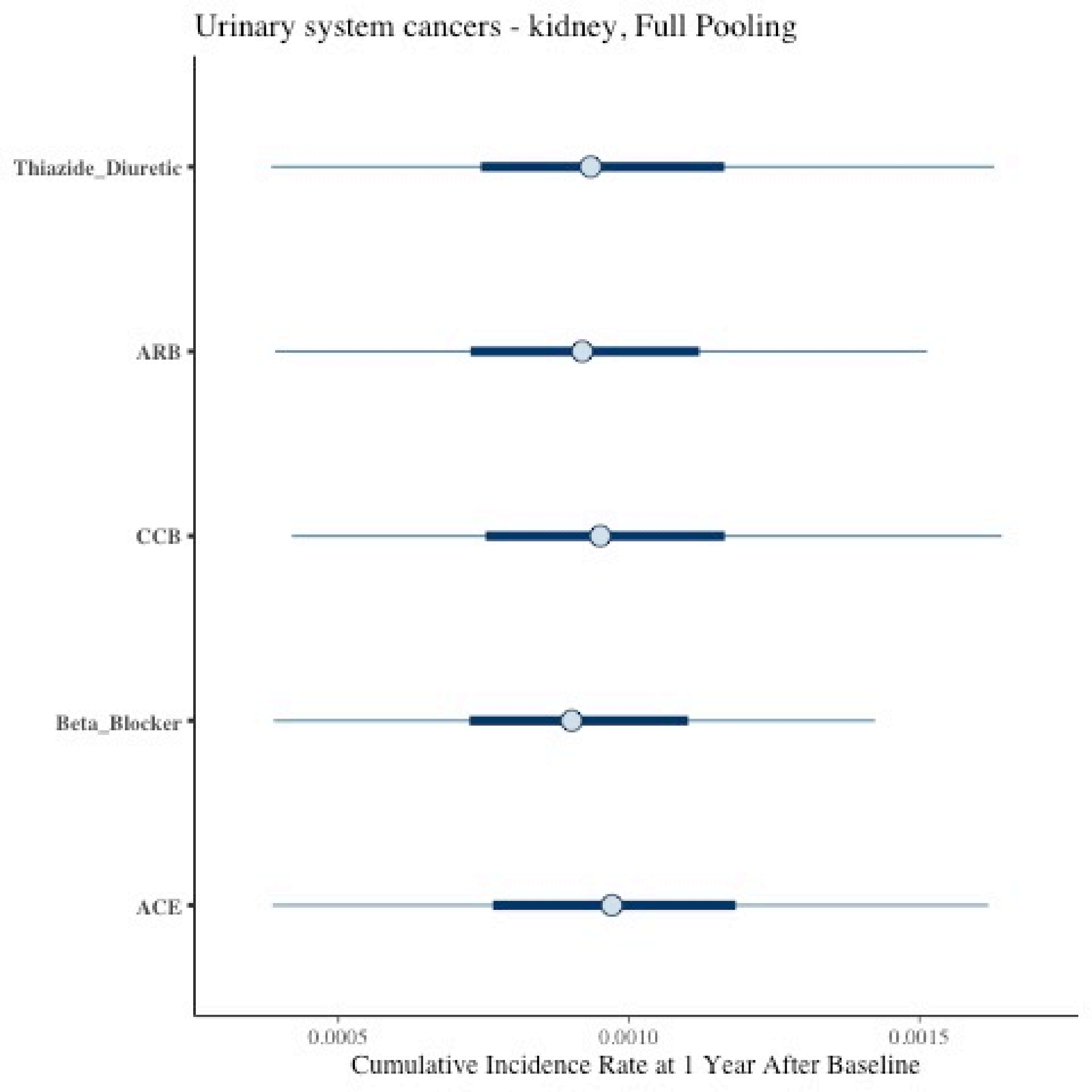
Urinary system cancers - kidney, Full Pooling
Thiazide_Diuretic
ARB
CCB
Beta_Blocker
ACE
0.0005
0.0010
0.0015
Cumulative Incidence Rate at 1 Year After Baseline

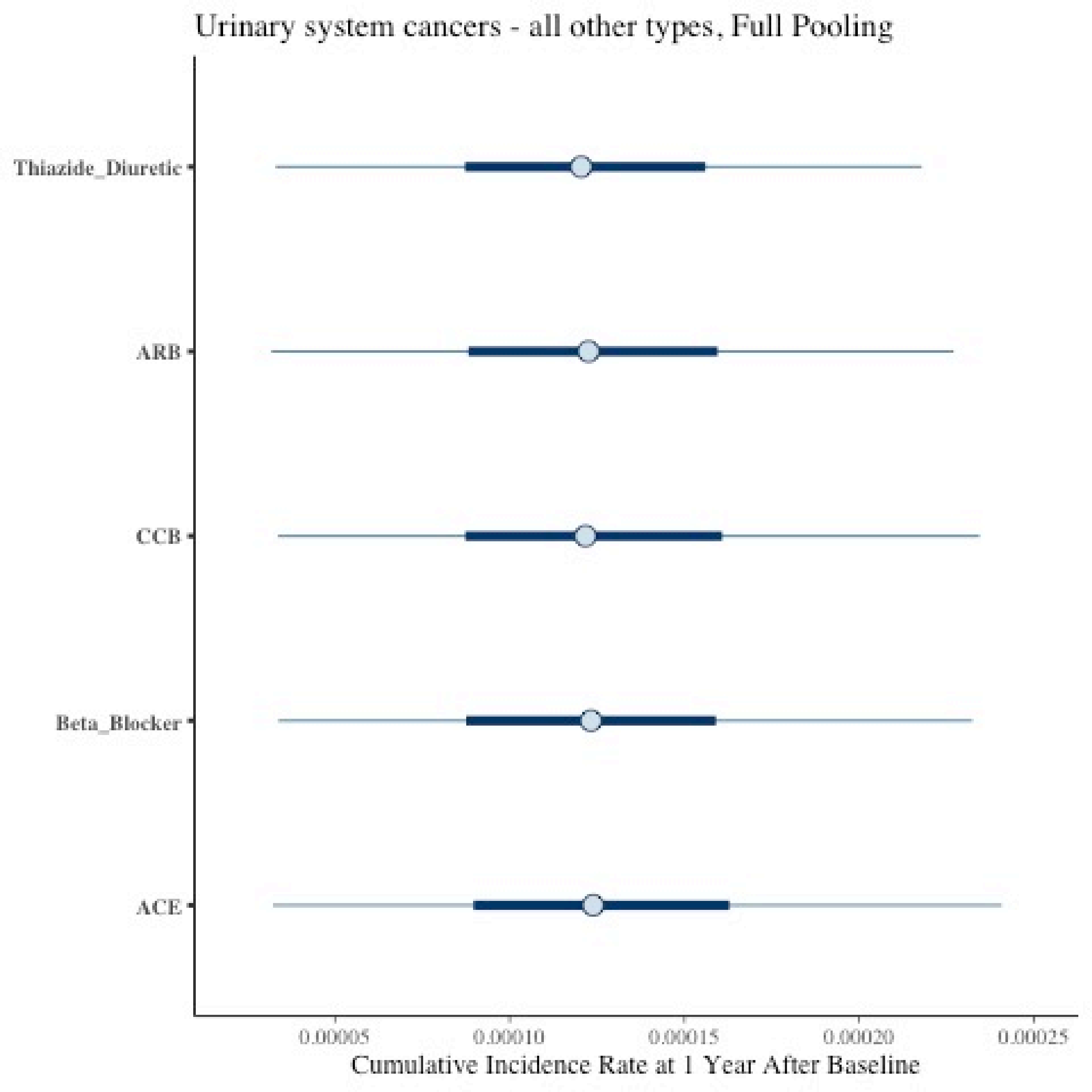
Urinary system cancers - all other types, Full Pooling
Thiazide_Diuretic
ARB
CCB
Beta_Blocker
ACE
0.00005
0.00010
0.00015
0.00020
0.00025
Cumulative Incidence Rate at 1 Year After Baseline

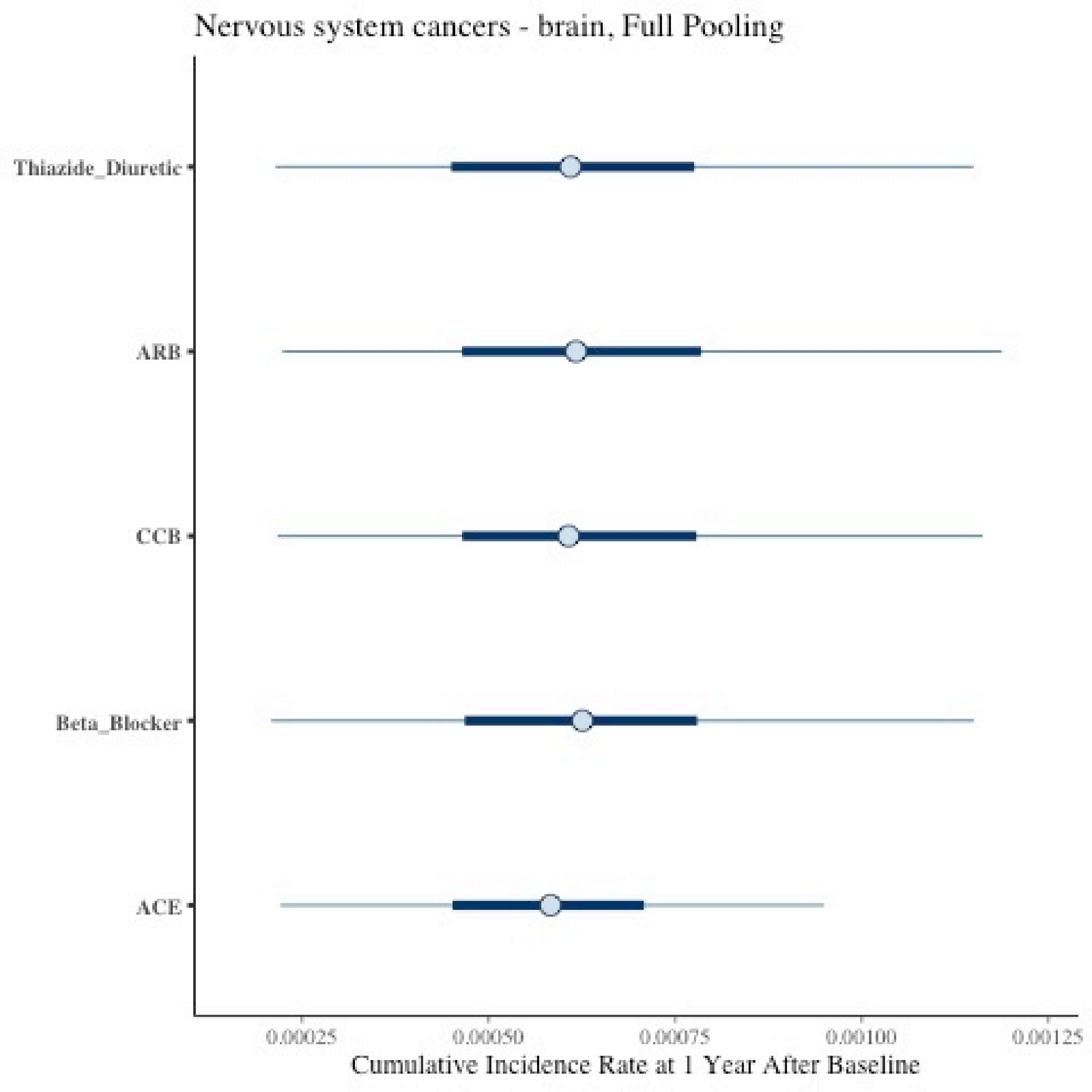

Nervous system cancers - brain, Full Pooling
Thiazide_Diuretic
ARB
CCB
Beta_Blocker
ACE
0.00025
0.00050
0.00075
0.00100
0.00125
Cumulative Incidence Rate at 1 Year After Baseline

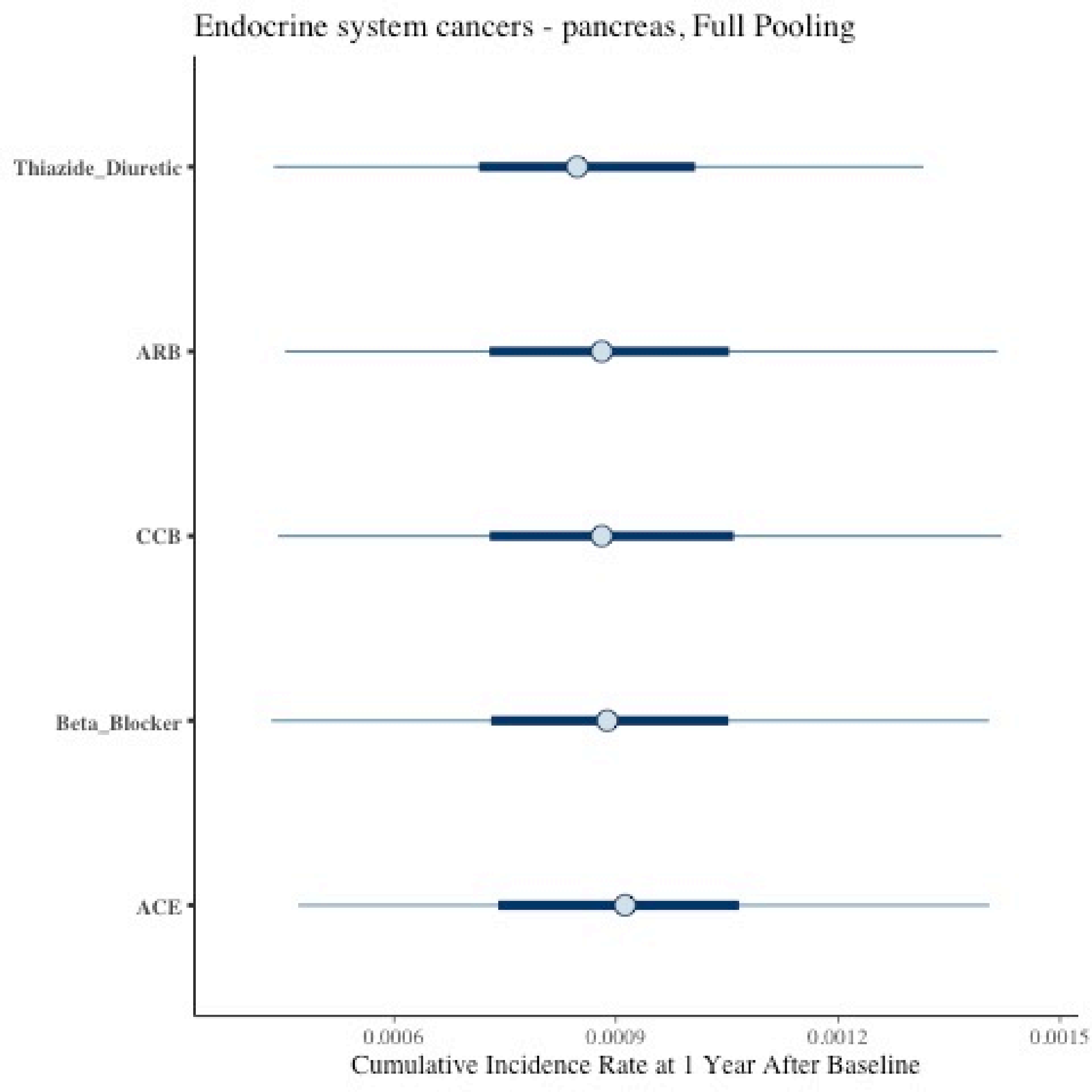

Endocrine system cancers - pancreas, Full Pooling
Thiazide_Diuretic
ARB
CCB
Beta_Blocker
ACE
0.0006
0.0009
0.0012
0.0015
Cumulative Incidence Rate at 1 Year After Baseline

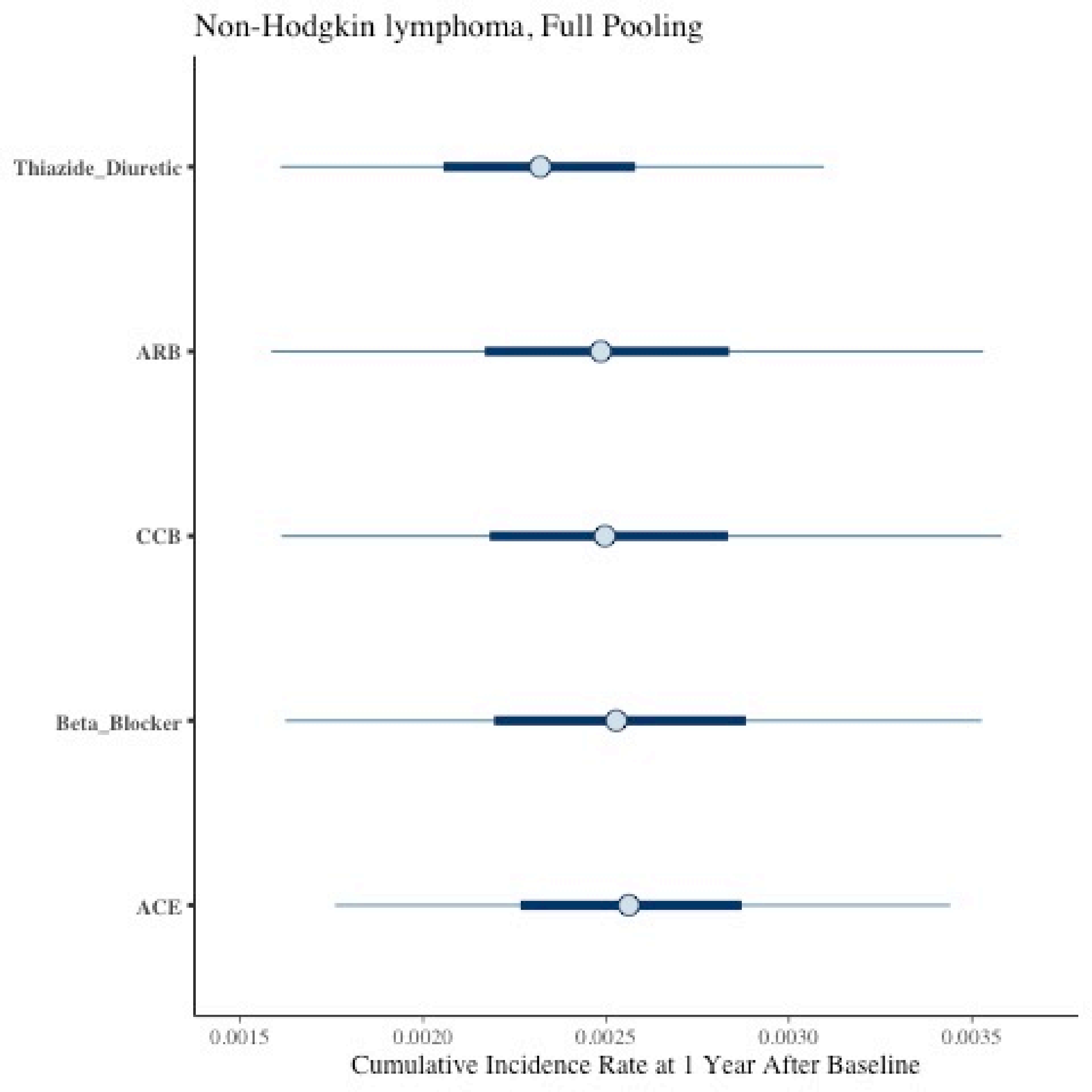

Non-Hodgkin lymphoma, Full Pooling
Thiazide_Diuretic
ARB
CCB
Beta_Blocker
ACE
0.0015
0.0020
0.0025
0.0030
0.0035
Cumulative Incidence Rate at 1 Year After Baseline

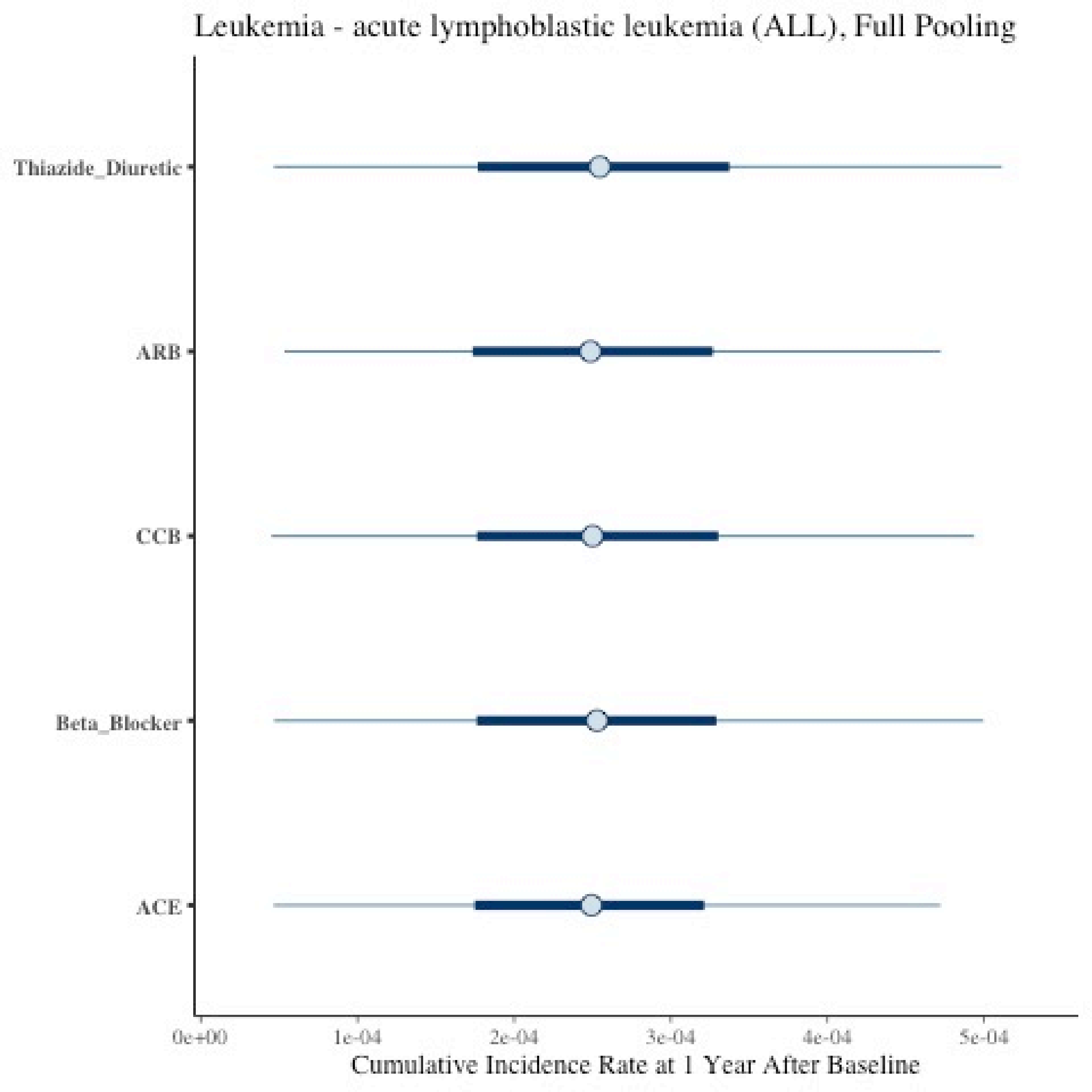

Leukemia - acute lymphoblastic leukemia (ALL), Full Pooling
Thiazide_Diuretic
ARB
CCB
Beta_Blocker
ACE
0e+00
1e-04
2e-04
3e-04
4e-04
5e-04
Cumulative Incidence Rate at 1 Year After Baseline

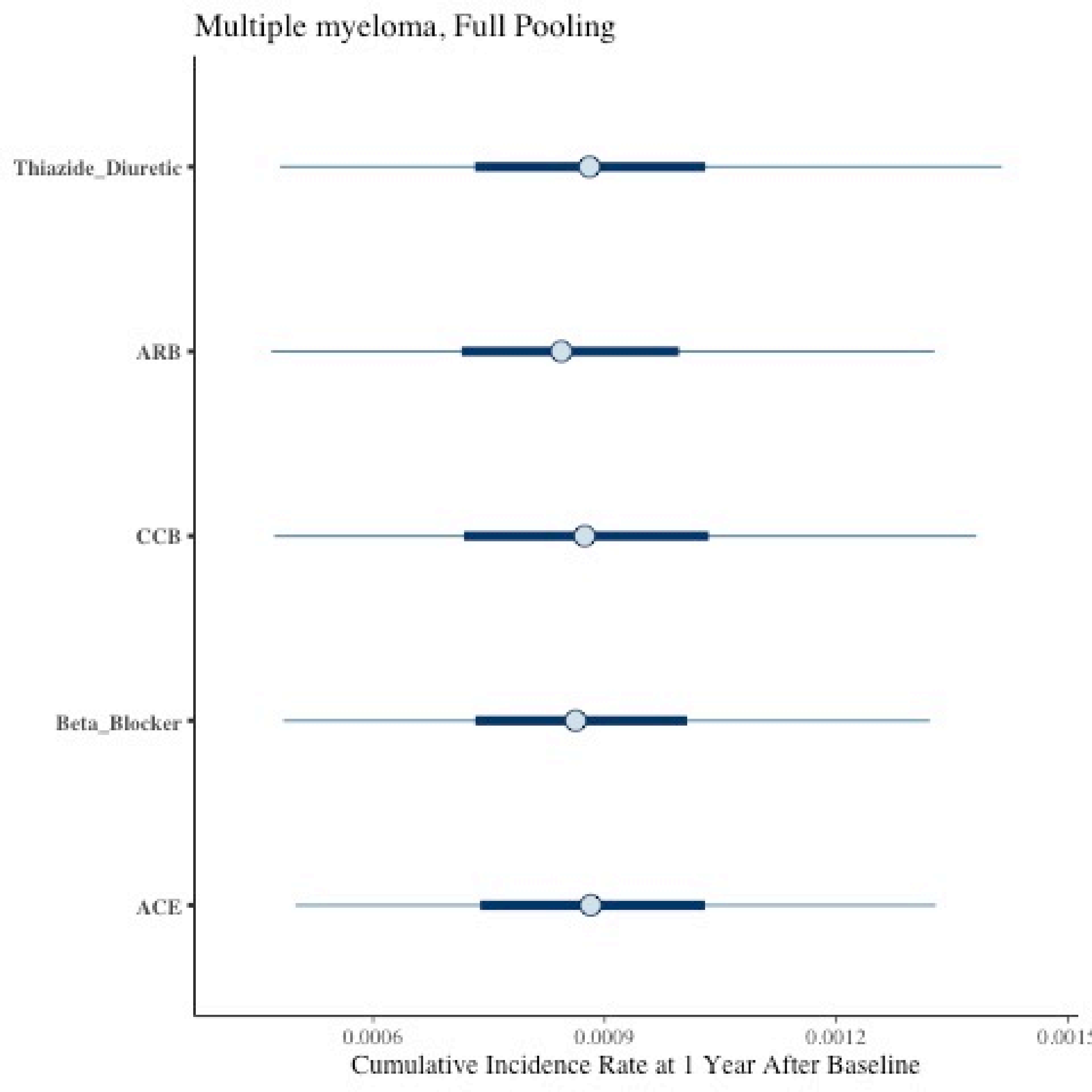

Multiple myeloma, Full Pooling
Thiazide_Diuretic
ARB
CCB
Beta_Blocker
ACE
0.0006
0.0009
0.0012
Cumulative Incidence Rate at 1 Year After Baseline

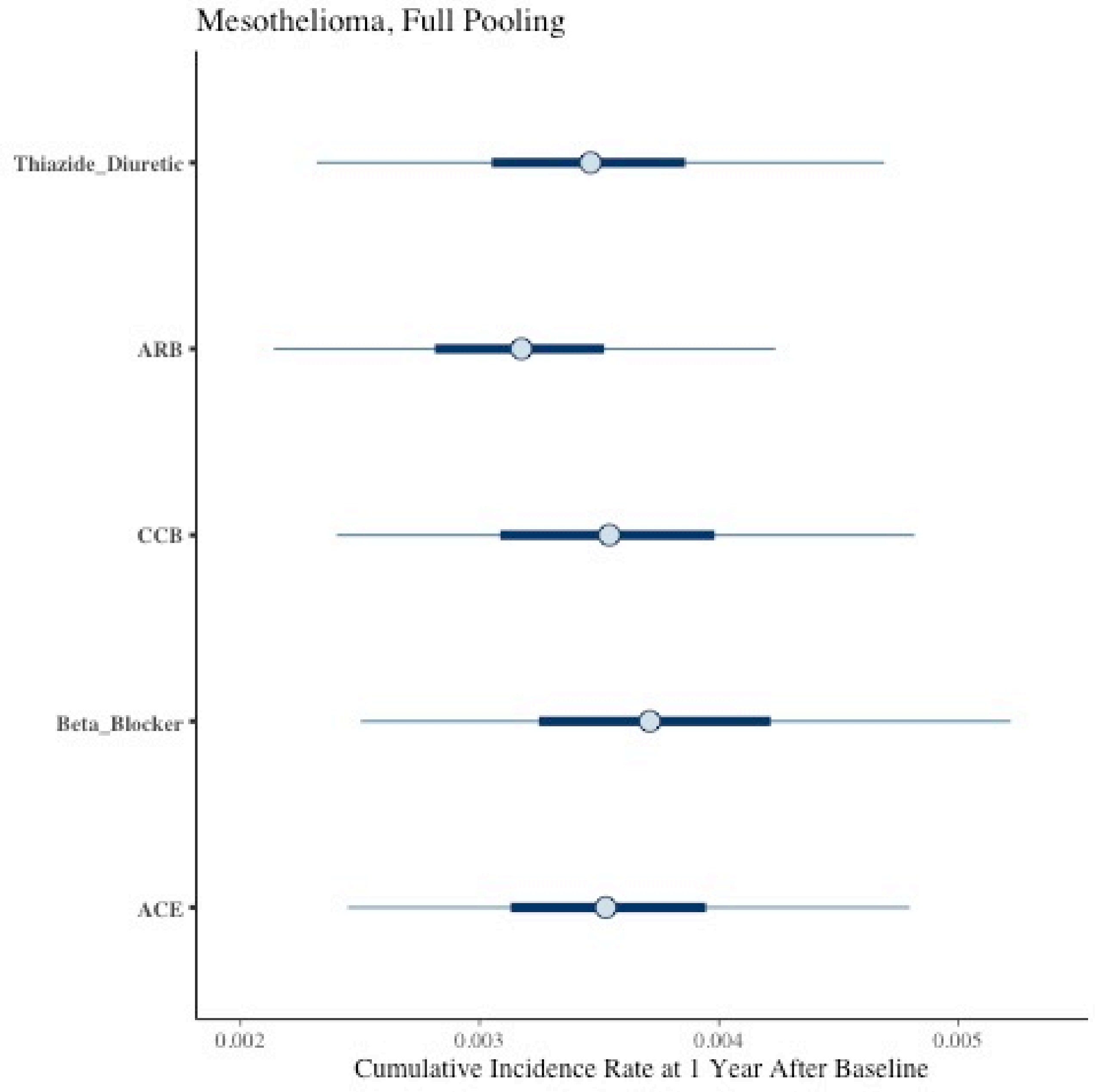

Mesothelioma, Full Pooling
Thiazide_Diuretic
ARB
CCB
Beta_Blocker
ACE
0.002
0.003
0.004
0.005
Cumulative Incidence Rate at 1 Year After Baseline

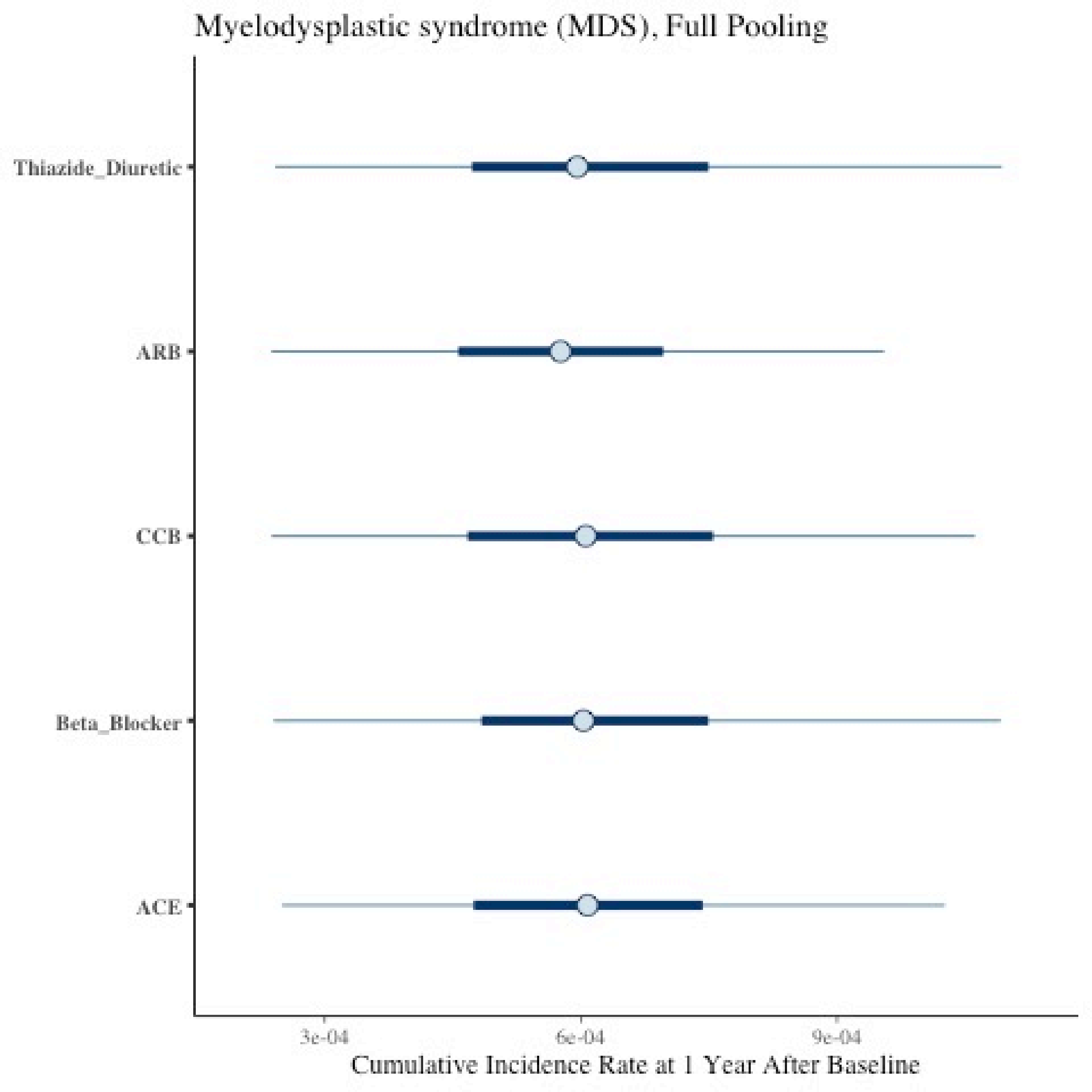

Myelodysplastic syndrome (MDS), Full Pooling
Thiazide_Diuretic
ARB
CCB
Beta_Blocker
ACE
3e-04
6e-04
9e-04
Cumulative Incidence Rate at 1 Year After Baseline

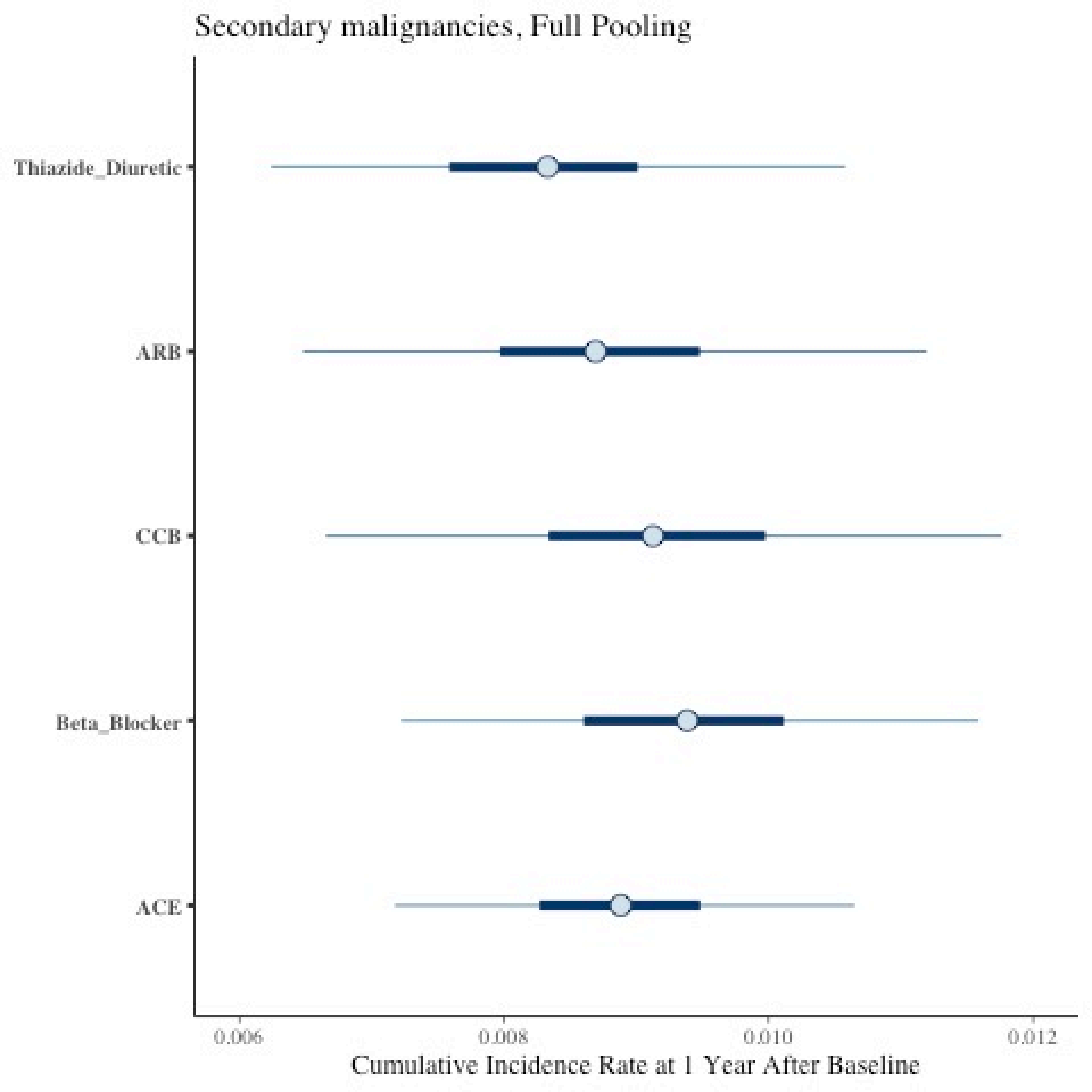
Secondary malignancies, Full Pooling
Thiazide_Diuretic
ARB
CCB
Beta_Blocker
ACE
0.006
0.008
0.010
0.012
Cumulative Incidence Rate at 1 Year After Baseline

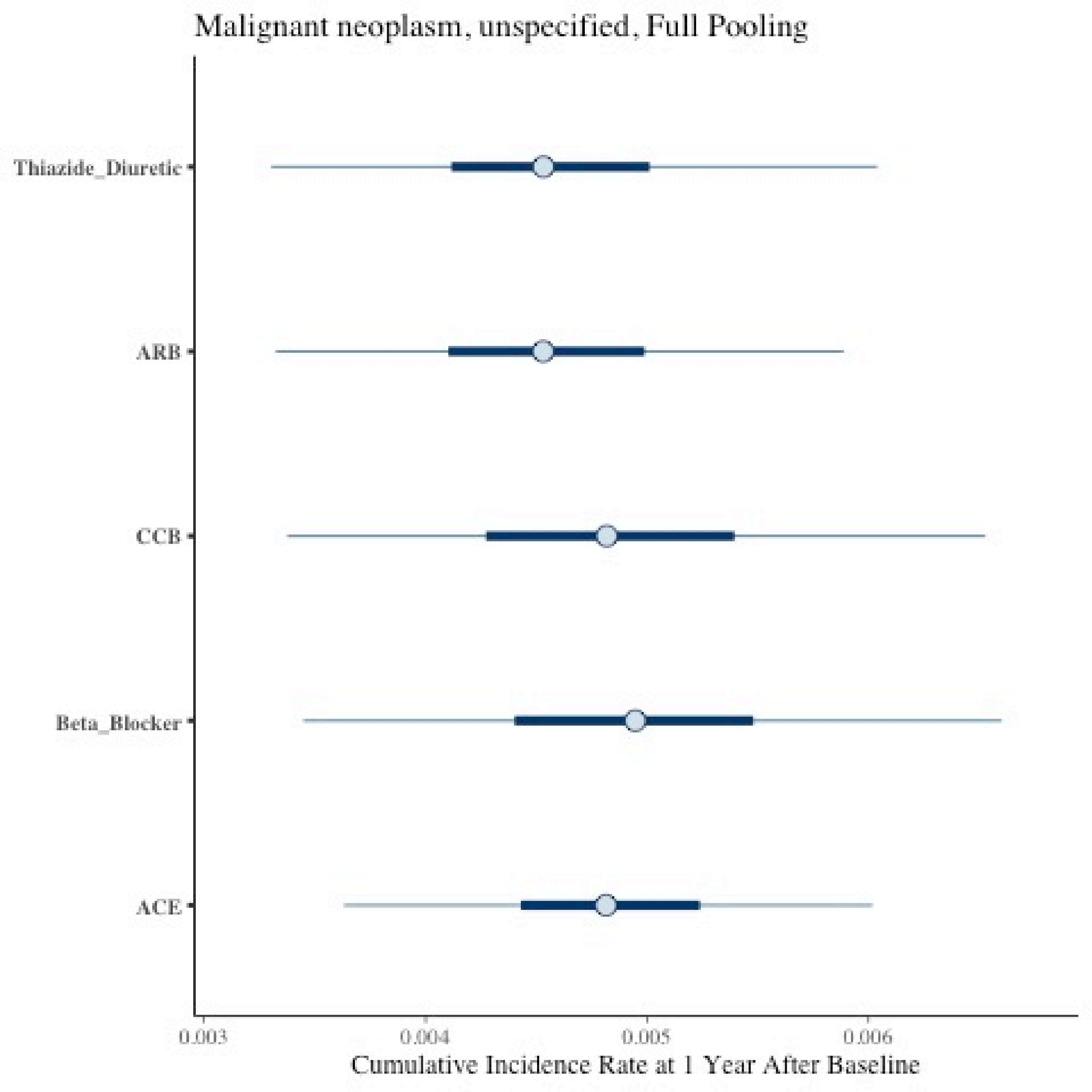

Malignant neoplasm, unspecified, Full Pooling
Thiazide_Diuretic
ARB
CCB
Beta_Blocker
ACE
0.003
0.004
0.005
0.006
Cumulative Incidence Rate at 1 Year After Baseline

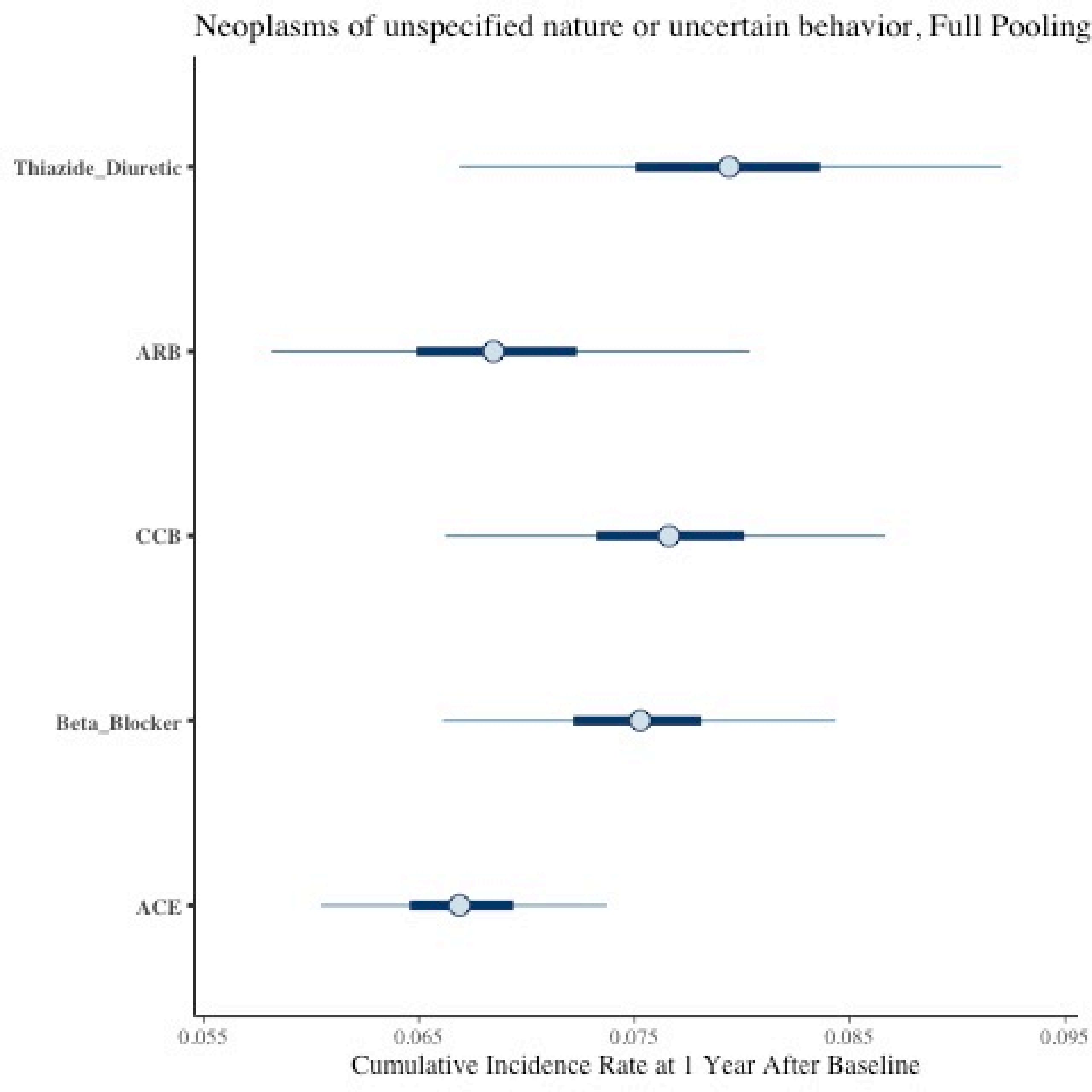
Neoplasms of unspecified nature or uncertain behavior, Full Pooling
Thiazide_Diuretic
ARB
CCB
Beta_Blocker
ACE
0.055
0.065
0.075
0.085
0.095
Cumulative Incidence Rate at 1 Year After Baseline

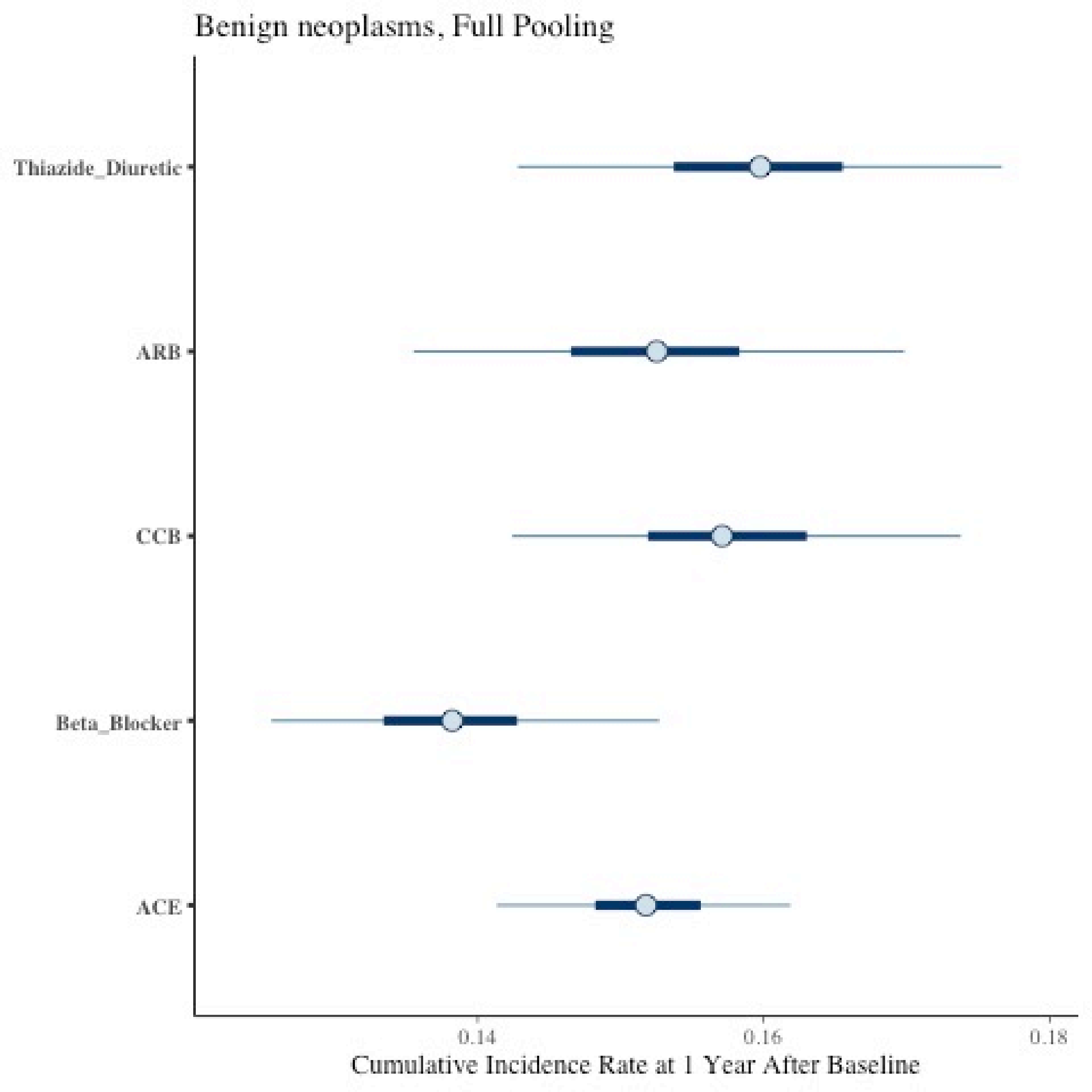

Benign neoplasms, Full Pooling
Thiazide_Diuretic
ARB
CCB
Beta_Blocker
ACE
0.14
0.16
0.18
Cumulative Incidence Rate at 1 Year After Baseline

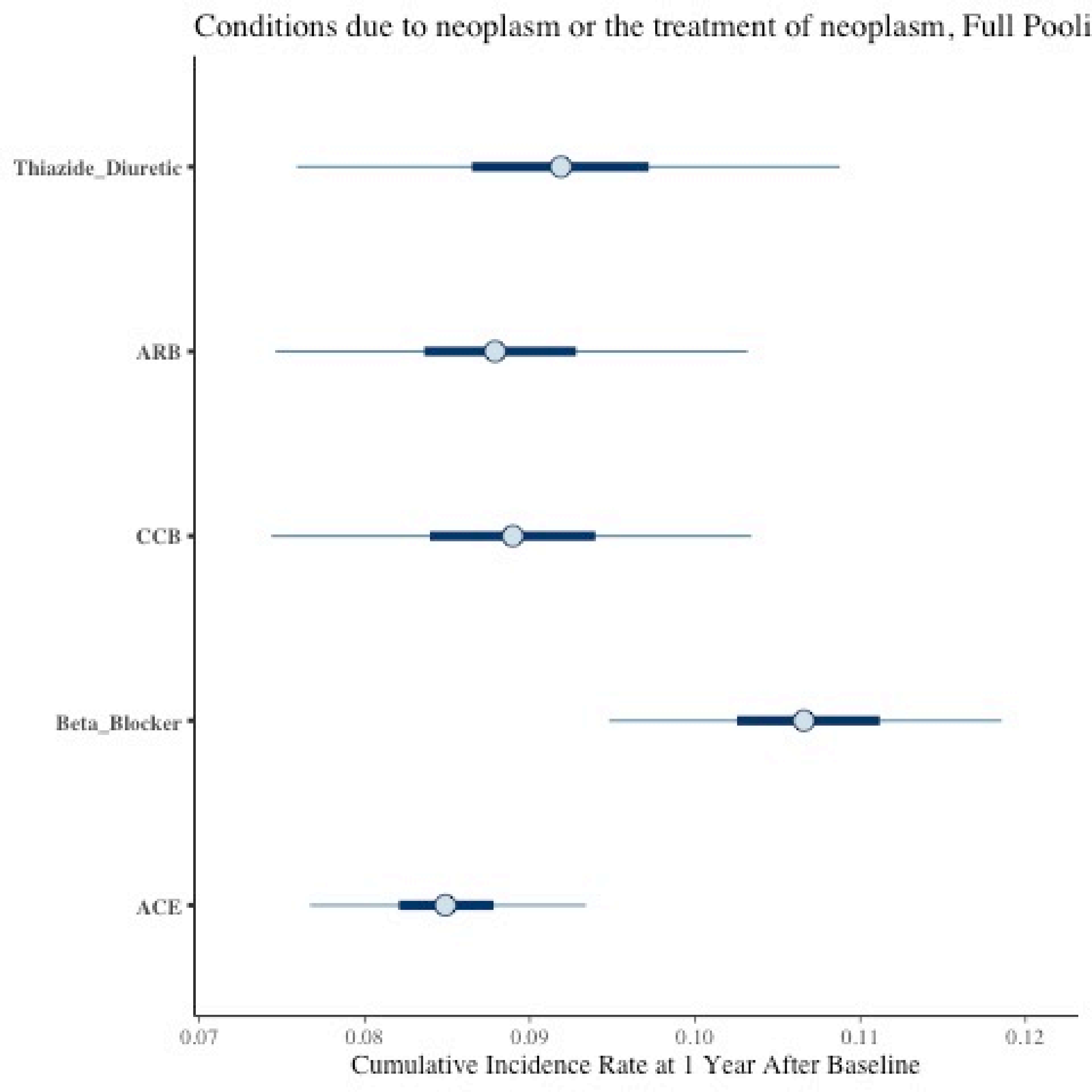

Conditions due to neoplasm or the treatment of neoplasm, Full Pooli
Thiazide_Diuretic
ARB
CCB
Beta_Blocker
ACE
0.07
0.08
0.09
0.10
0.11
0.12
Cumulative Incidence Rate at 1 Year After Baseline

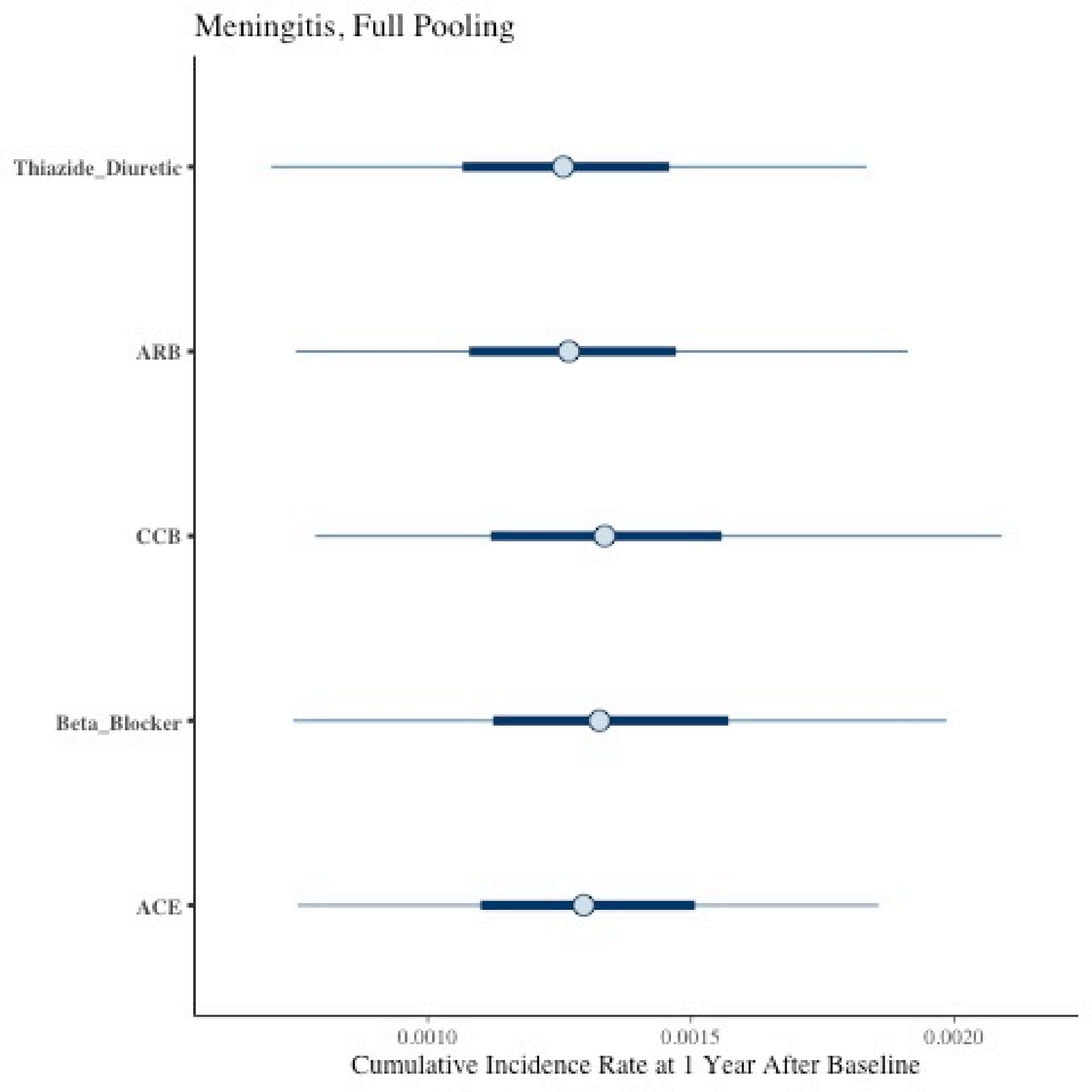

Meningitis, Full Pooling
Thiazide_Diuretic
ARB
CCB
Beta_Blocker
ACE
0.0010
0.0015
0.0020
Cumulative Incidence Rate at 1 Year After Baseline

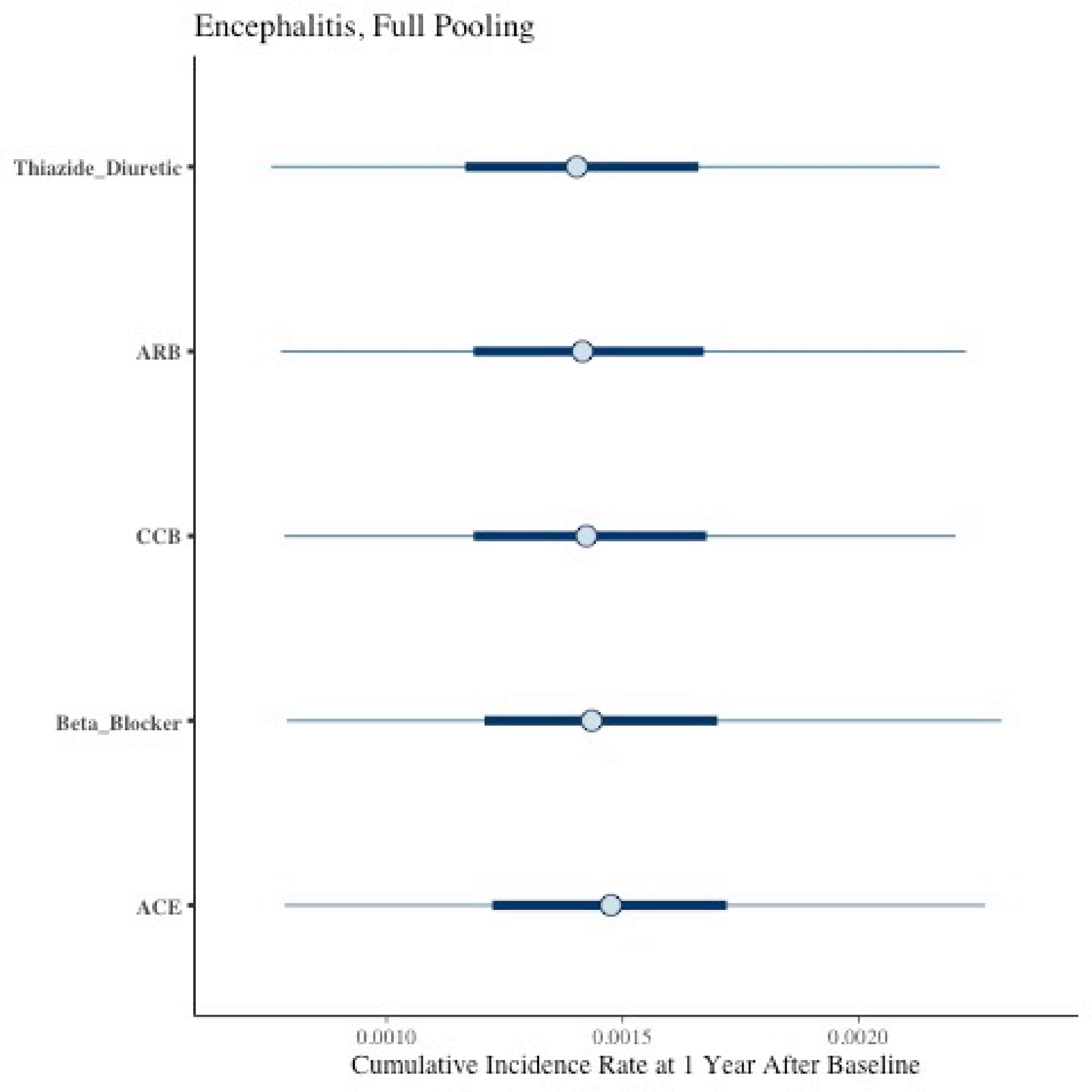

Encephalitis, Full Pooling
Thiazide_Diuretic
ARB
CCB
Beta_Blocker
ACE
0.0010
0.0015
0.0020
Cumulative Incidence Rate at 1 Year After Baseline

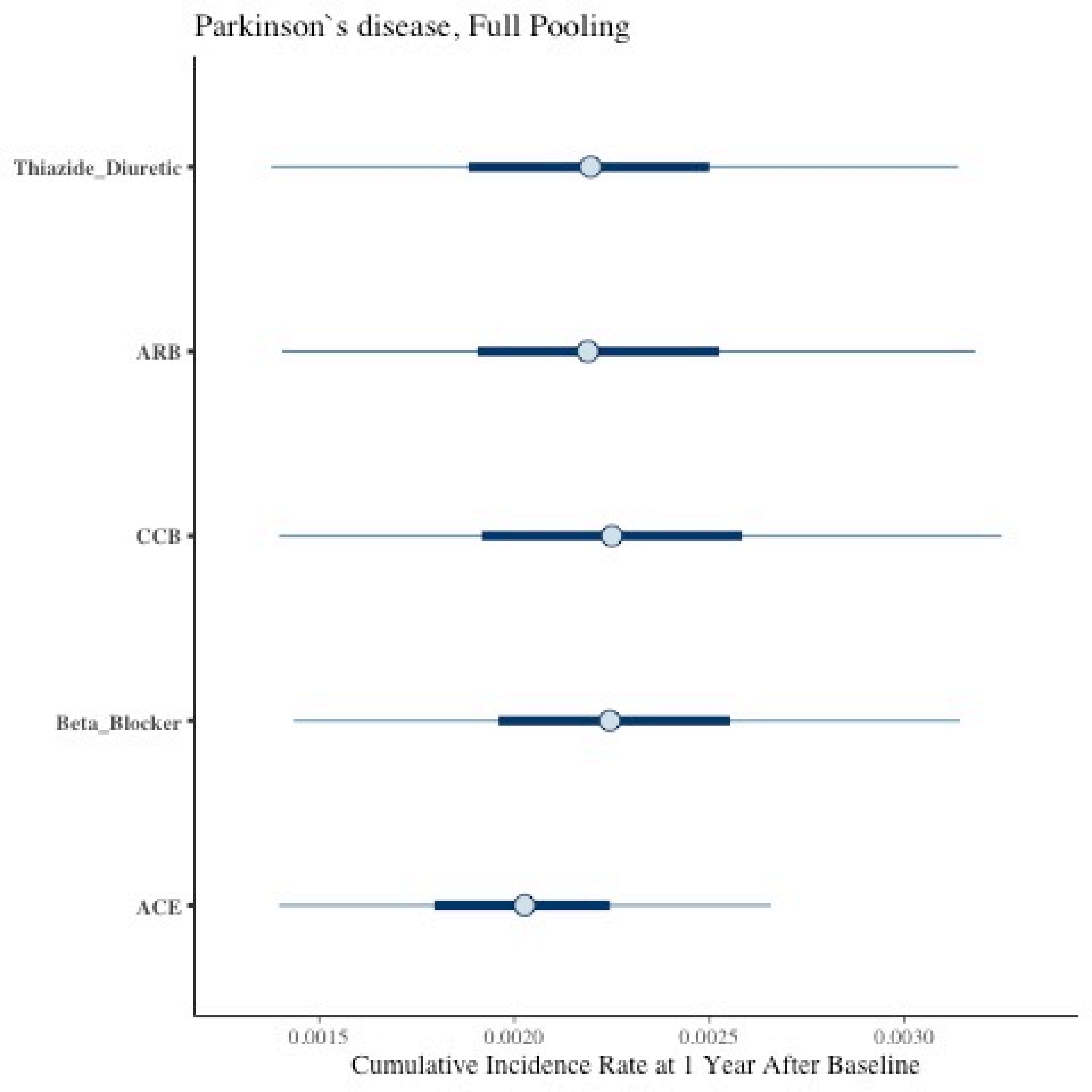
Parkinson`s disease, Full Pooling
Thiazide_Diuretic
ARB
CCB
Beta_Blocker
ACE
0.0015
0.0020
0.0025
0.0030
Cumulative Incidence Rate at 1 Year After Baseline

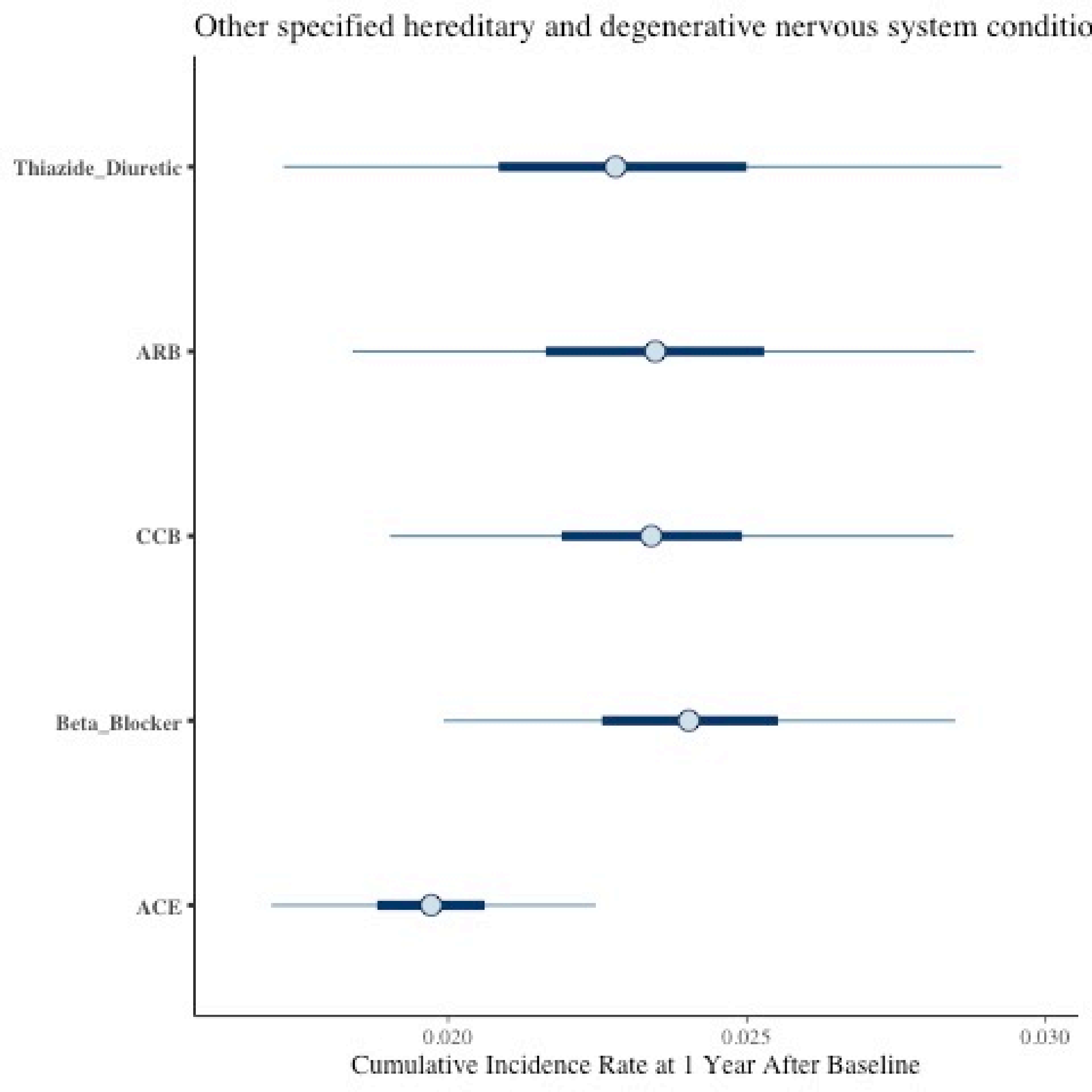
Other specified hereditary and degenerative nervous system conditio
Thiazide_Diuretic
ARB
CCB
Beta_Blocker
ACE
0.020
0.025
0.030
Cumulative Incidence Rate at 1 Year After Baseline

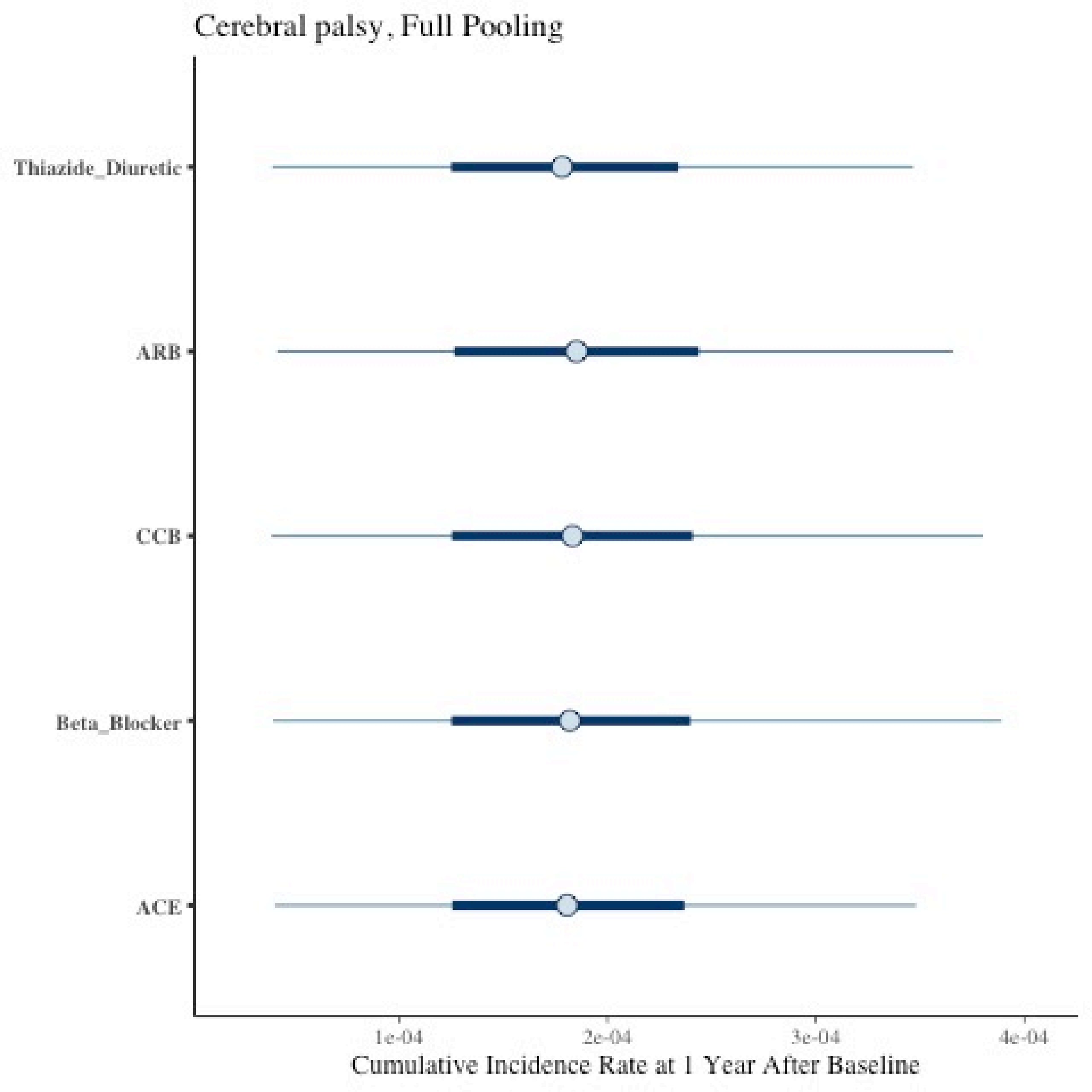

Cerebral palsy, Full Pooling
Thiazide_Diuretic
ARB
CCB
Beta_Blocker
ACE
1e-04
2e-04
3e-04
4e-04
Cumulative Incidence Rate at 1 Year After Baseline

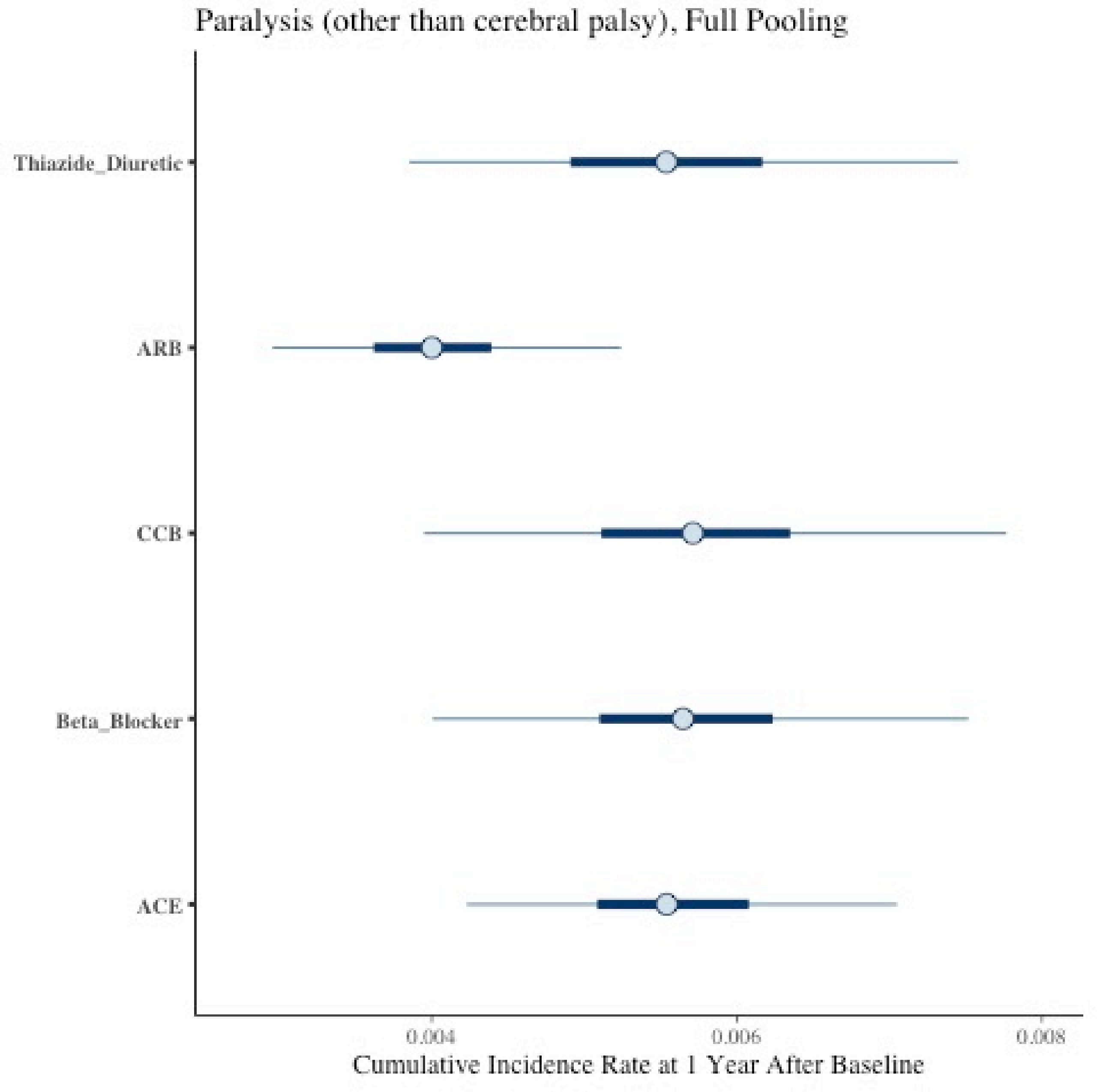

Paralysis (other than cerebral palsy), Full Pooling
Thiazide_Diuretic
ARB
CCB
Beta_Blocker
ACE
0.004
0.006
0.008
Cumulative Incidence Rate at 1 Year After Baseline

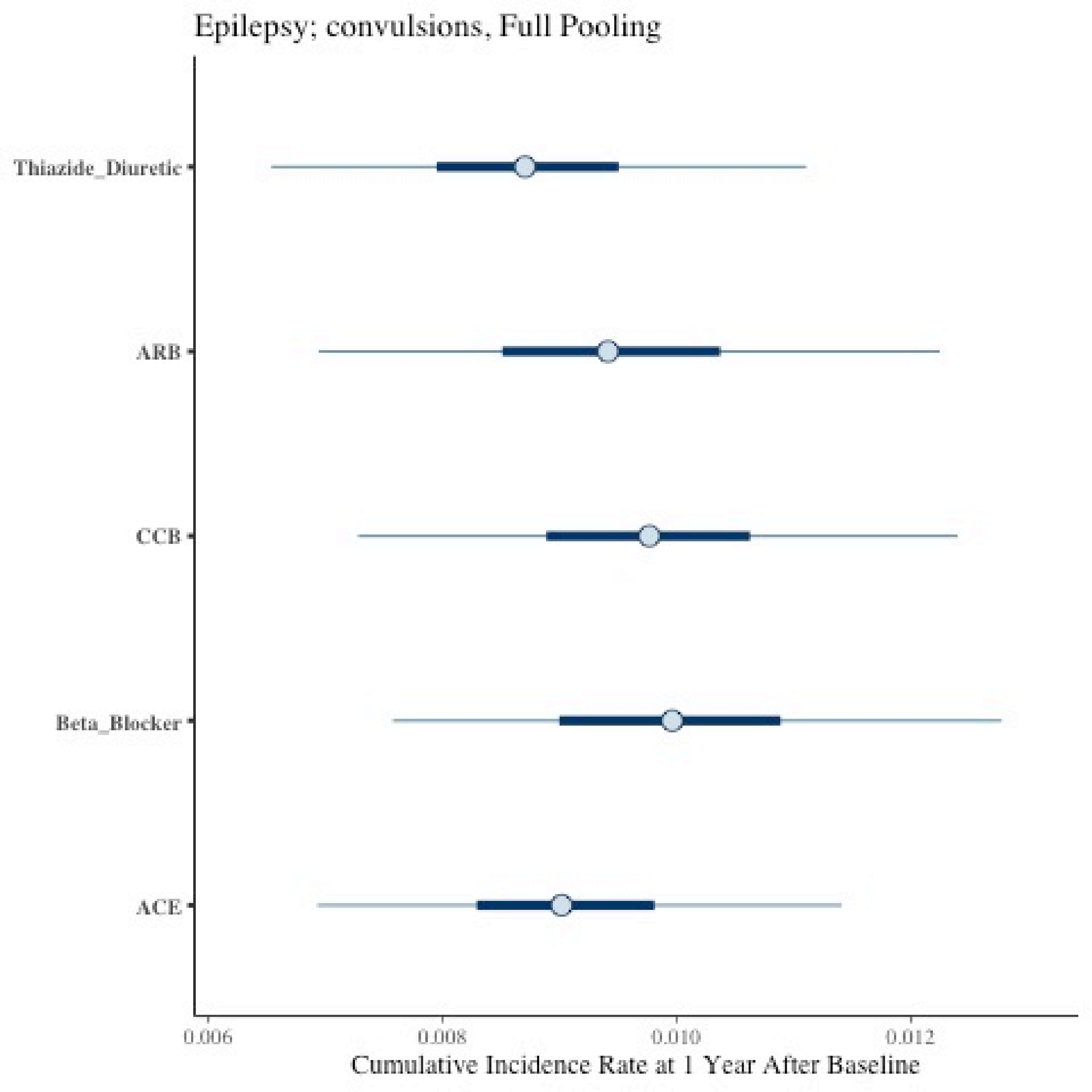

Epilepsy; convulsions, Full Pooling
Thiazide_Diuretic
ARB
CCB
Beta_Blocker
ACE
0.006
0.008
0.010
0.012
Cumulative Incidence Rate at 1 Year After Baseline

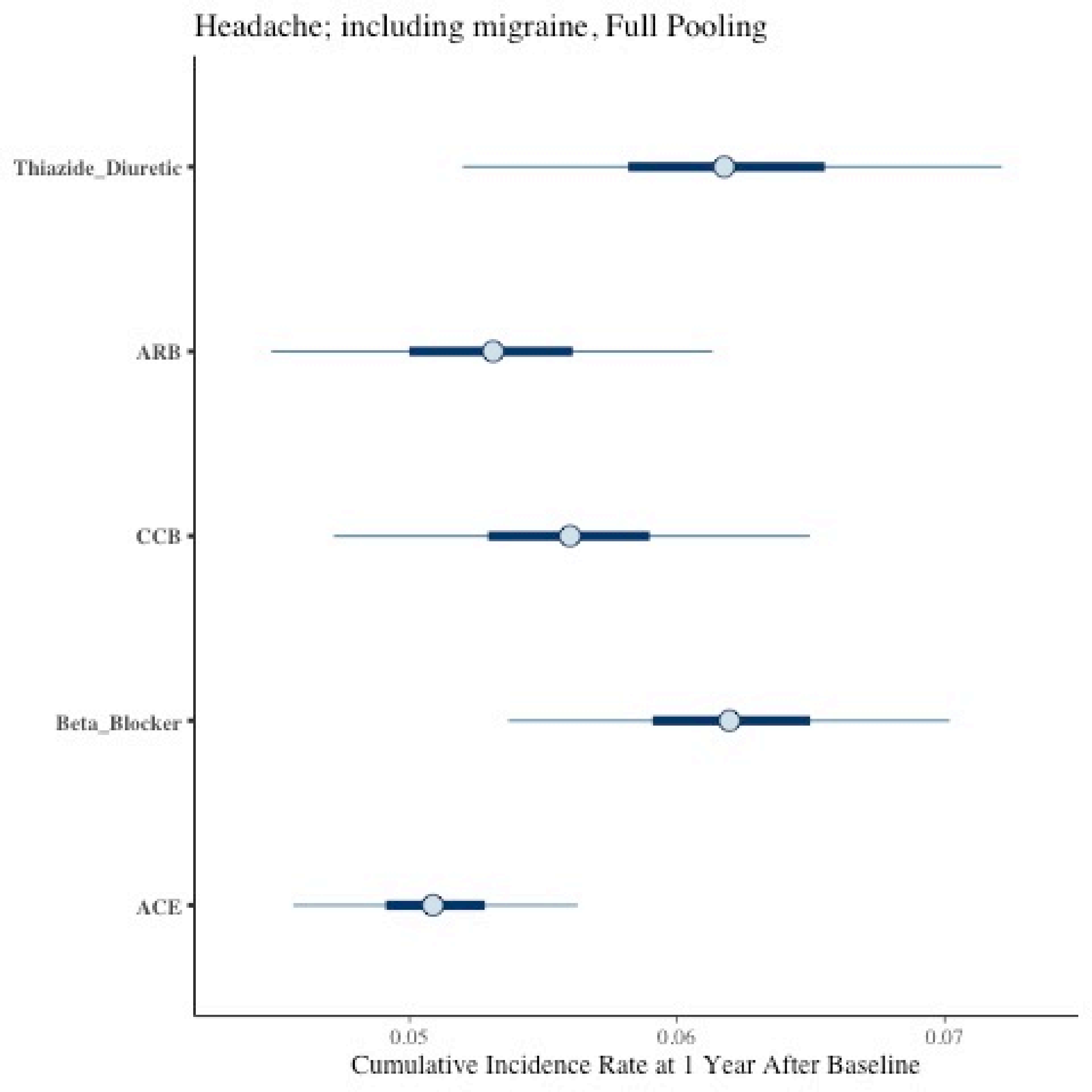

Headache; including migraine, Full Pooling
Thiazide_Diuretic
ARB
CCB
Beta_Blocker
ACE
0.05
0.06
0.07
Cumulative Incidence Rate at 1 Year After Baseline

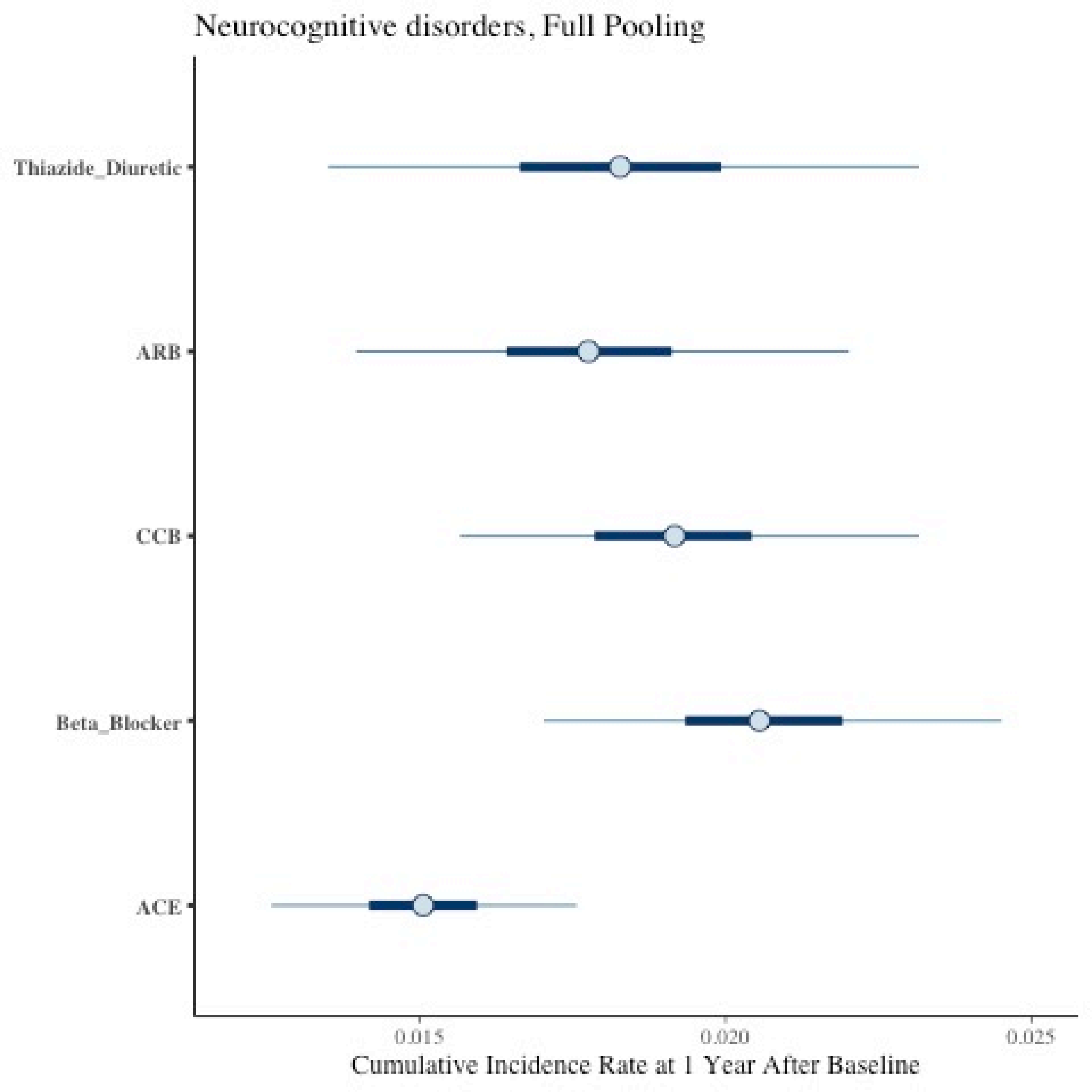

Neurocognitive disorders, Full Pooling
Thiazide_Diuretic
ARB
CCB
Beta_Blocker
ACE
0.015
0.020
0.025
Cumulative Incidence Rate at 1 Year After Baseline

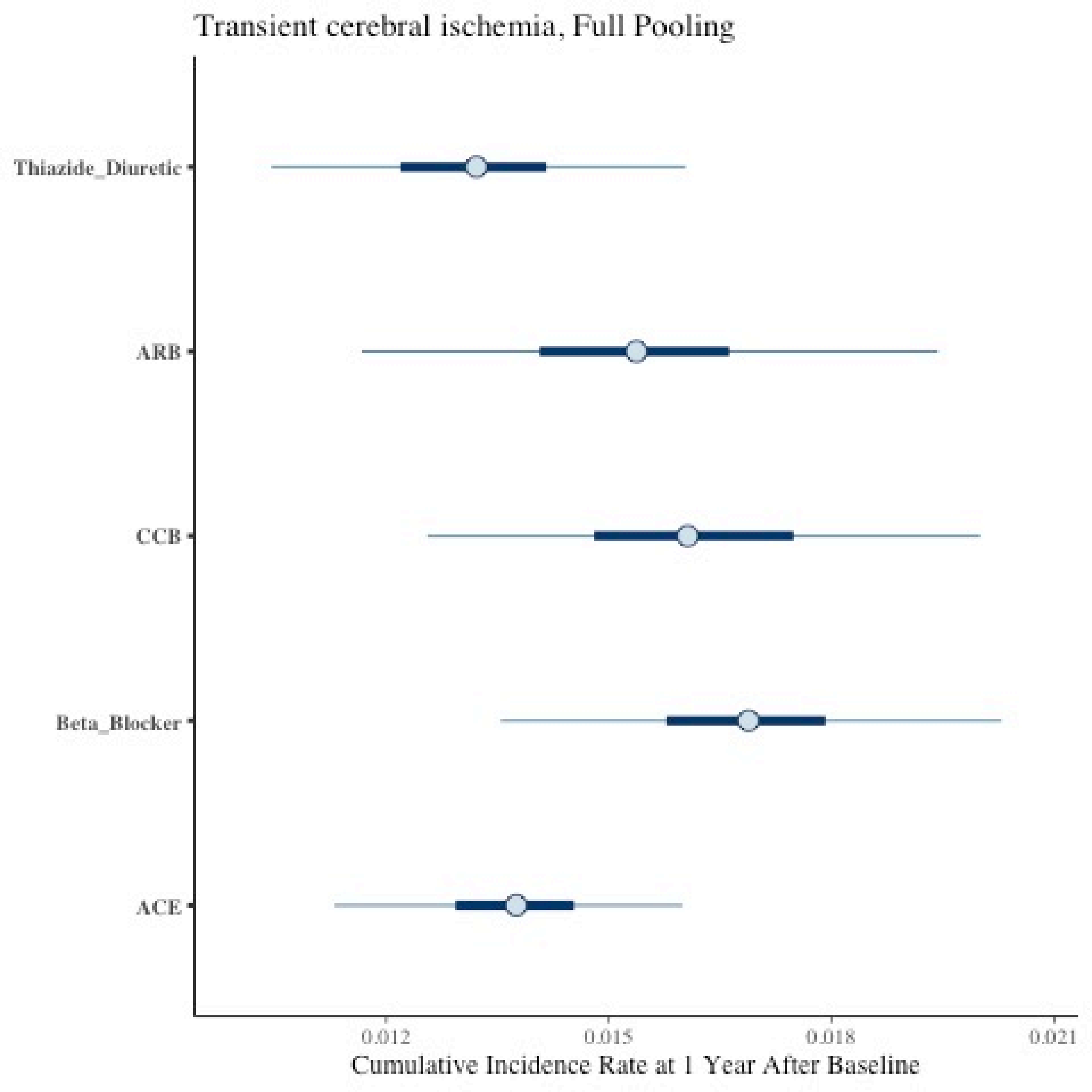

Transient cerebral ischemia, Full Pooling
Thiazide_Diuretic
ARB
CCB
Beta_Blocker
ACE
0.012
0.015
0.018
0.021
Cumulative Incidence Rate at 1 Year After Baseline

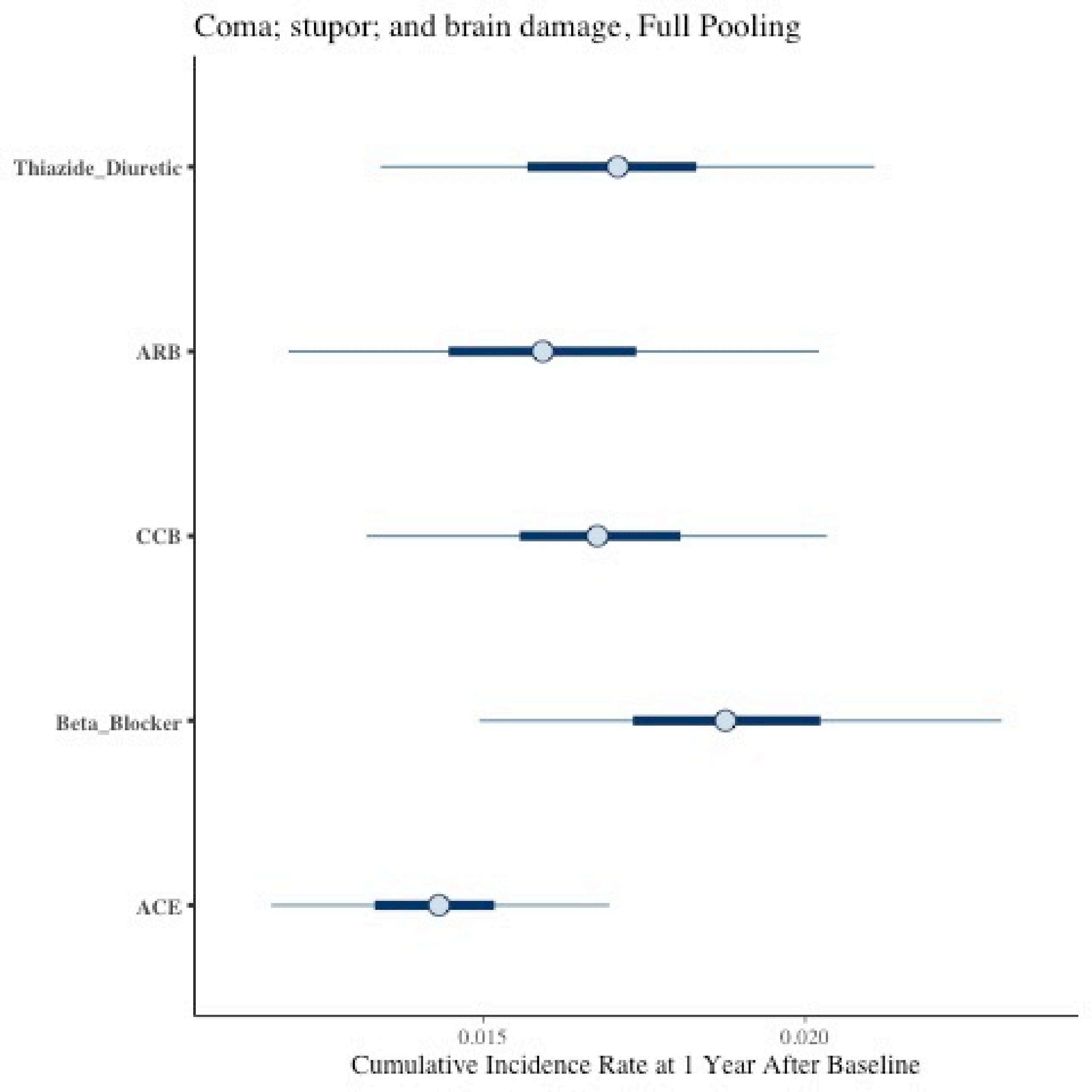
Coma; stupor; and brain damage, Full Pooling
Thiazide_Diuretic
ARB
CCB
Beta_Blocker
ACE
0.015
0.020
Cumulative Incidence Rate at 1 Year After Baseline

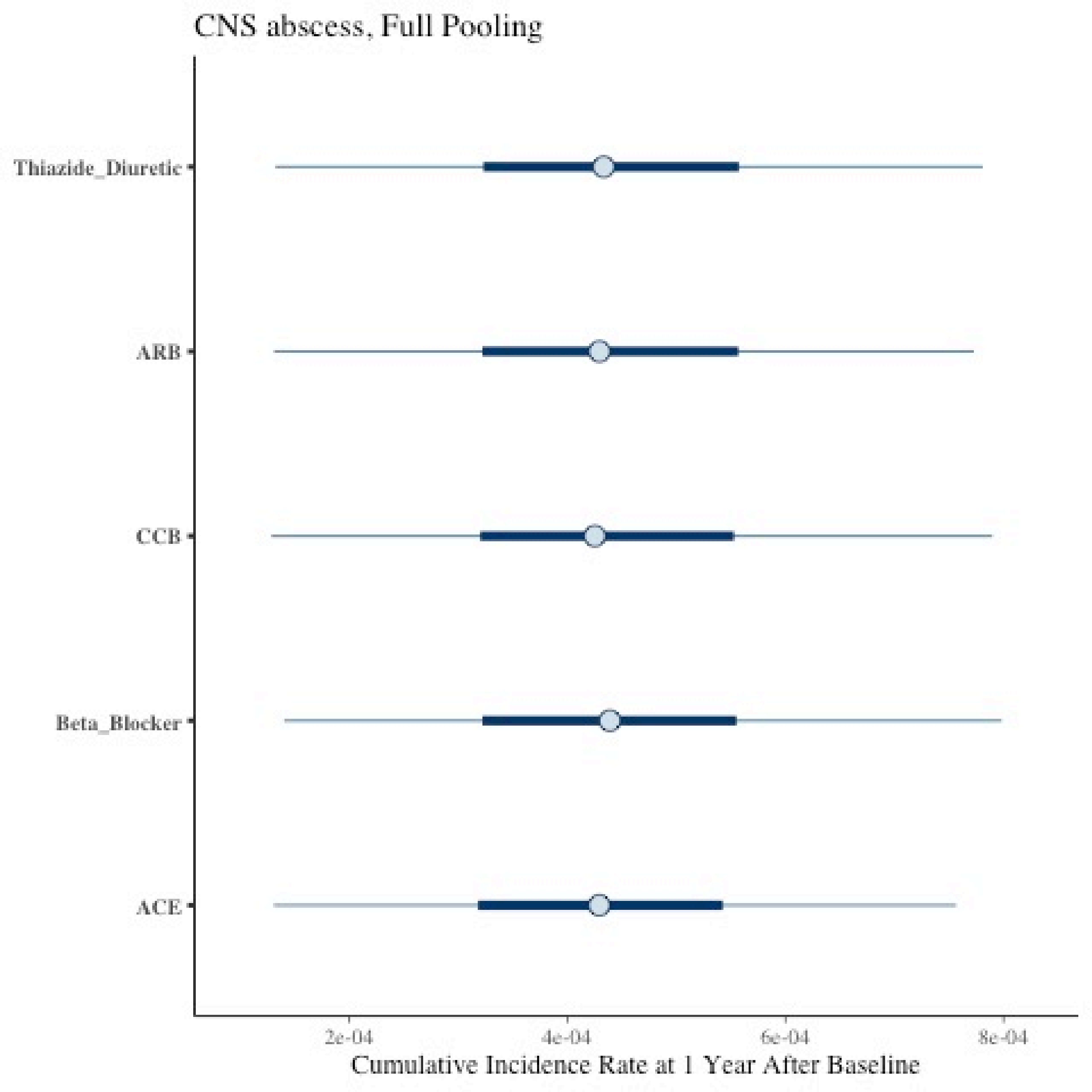

CNS abscess, Full Pooling
Thiazide_Diuretic
ARB
CCB
Beta_Blocker
ACE
2e-04
4e-04
6e-04
8e-04
Cumulative Incidence Rate at 1 Year After Baseline

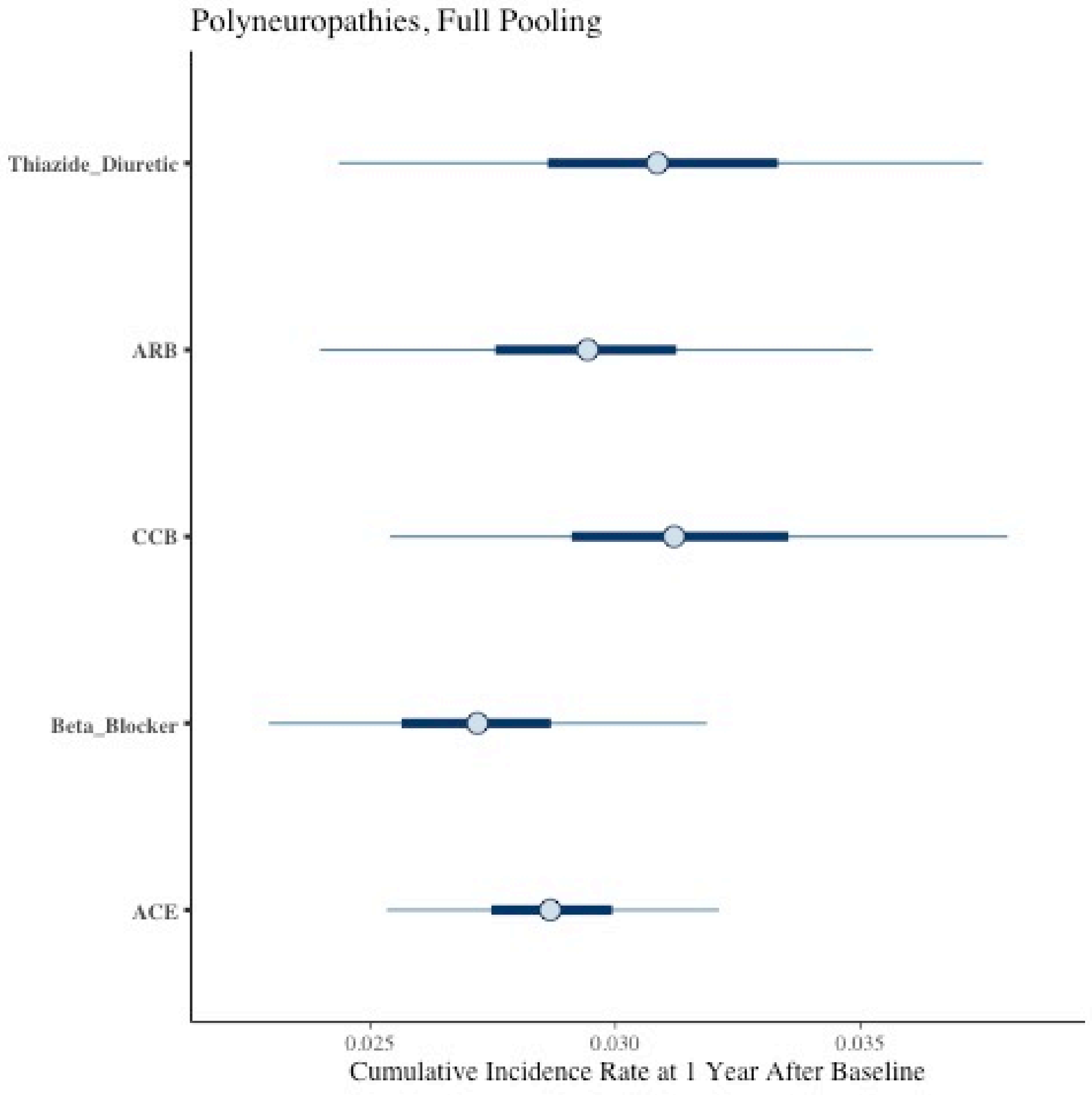
Polyneuropathies, Full Pooling
Thiazide_Diuretic
ARB
CCB
Beta_Blocker
ACE
0.025
0.030
0.035
Cumulative Incidence Rate at 1 Year After Baseline

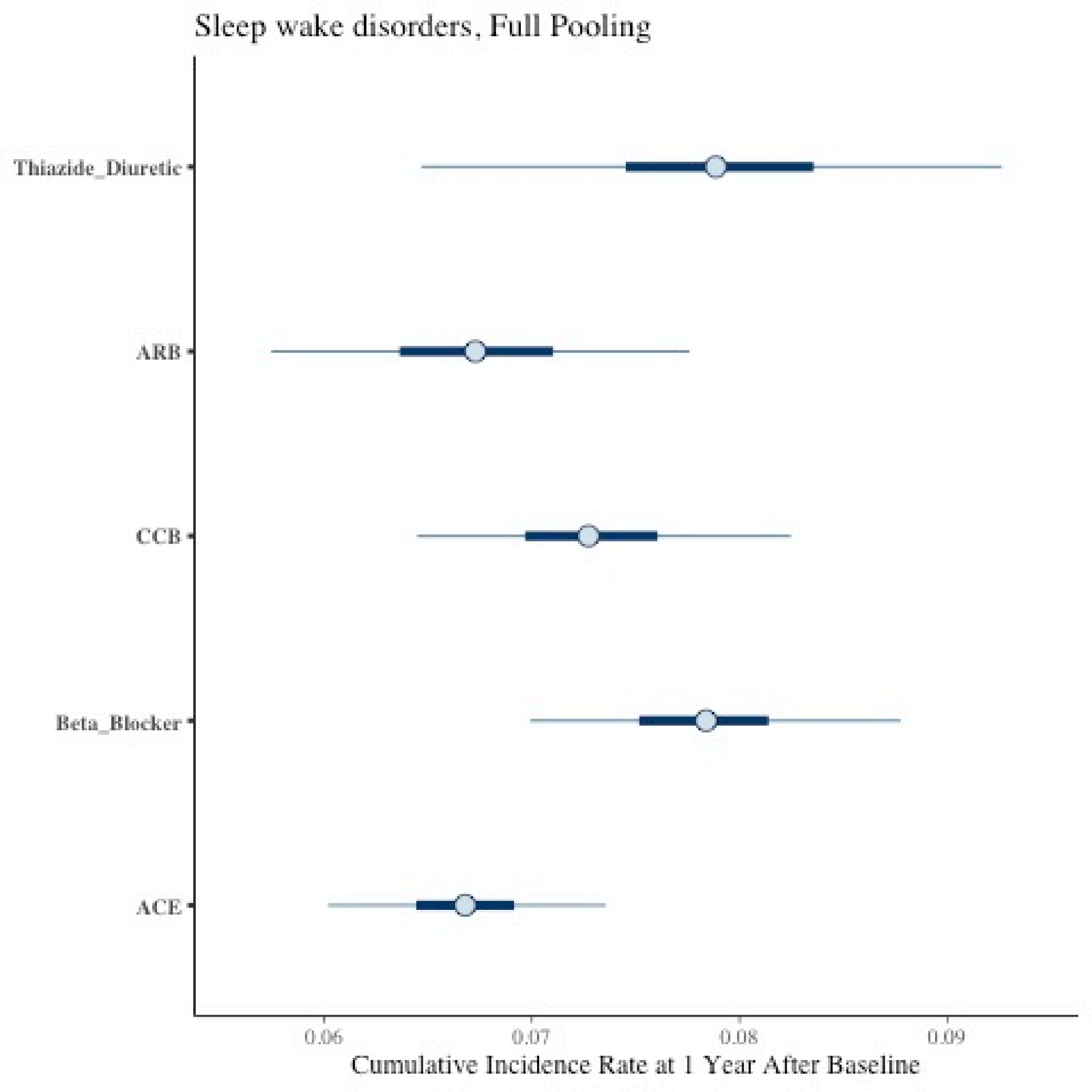

Sleep wake disorders, Full Pooling
Thiazide_Diuretic
ARB
CCB
Beta_Blocker
ACE
0.06
0.07
0.08
0.09
Cumulative Incidence Rate at 1 Year After Baseline

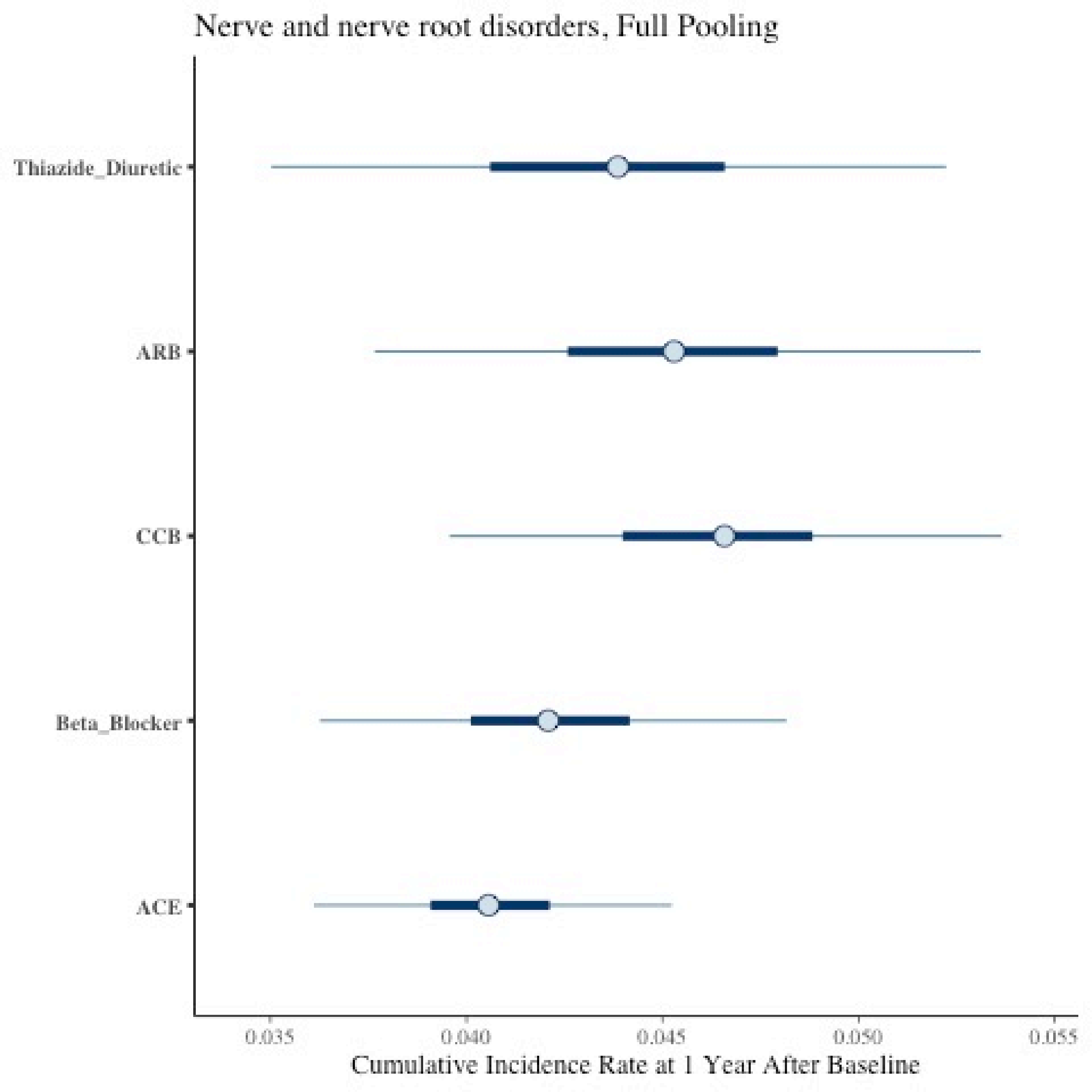
Nerve and nerve root disorders, Full Pooling
Thiazide_Diuretic
ARB
CCB
Beta_Blocker
ACE
0.035
0.040
0.045
0.050
0.055
Cumulative Incidence Rate at 1 Year After Baseline

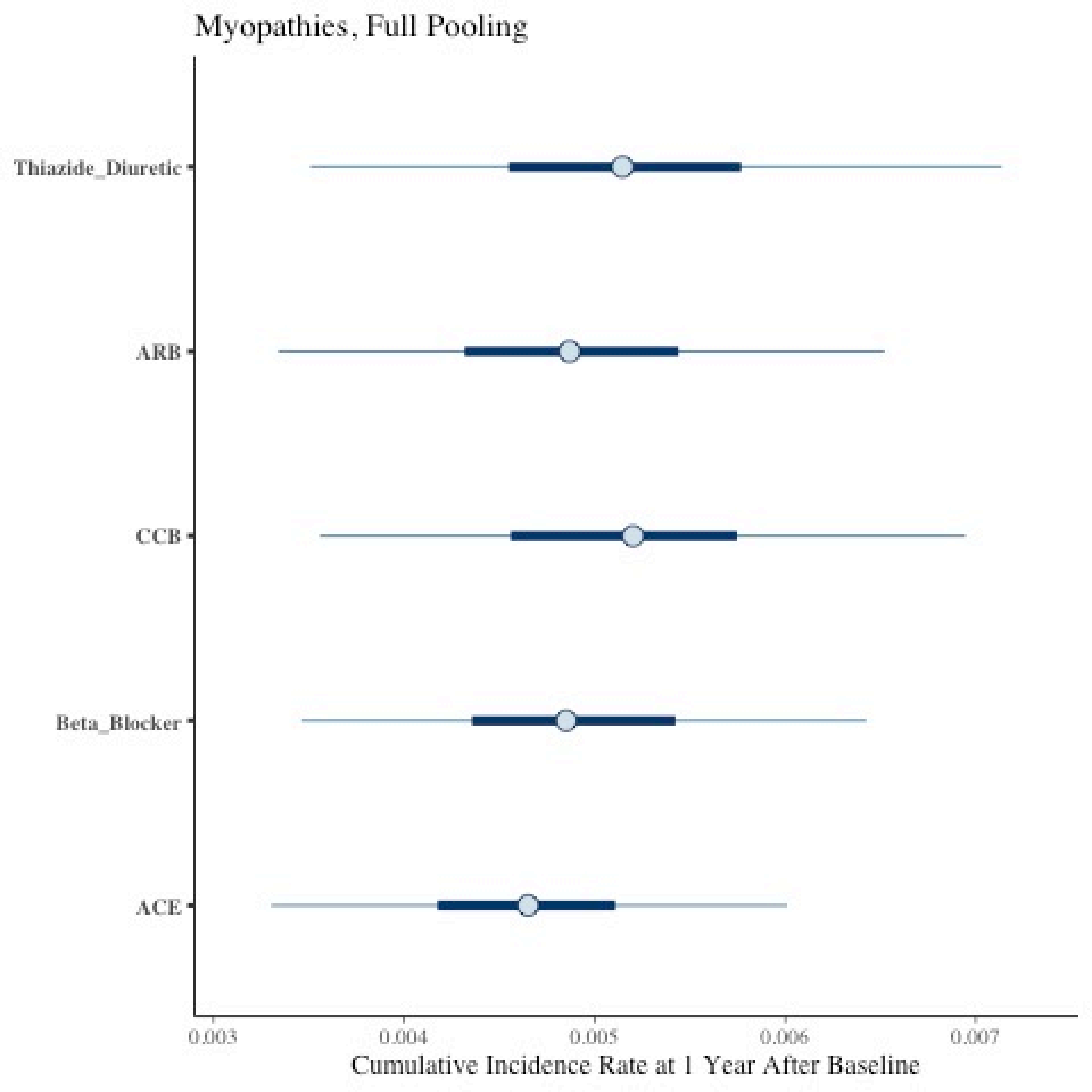
Myopathies, Full Pooling
Thiazide_Diuretic
ARB
CCB
Beta_Blocker
ACE
0.003
0.004
0.005
0.006
0.007
Cumulative Incidence Rate at 1 Year After Baseline

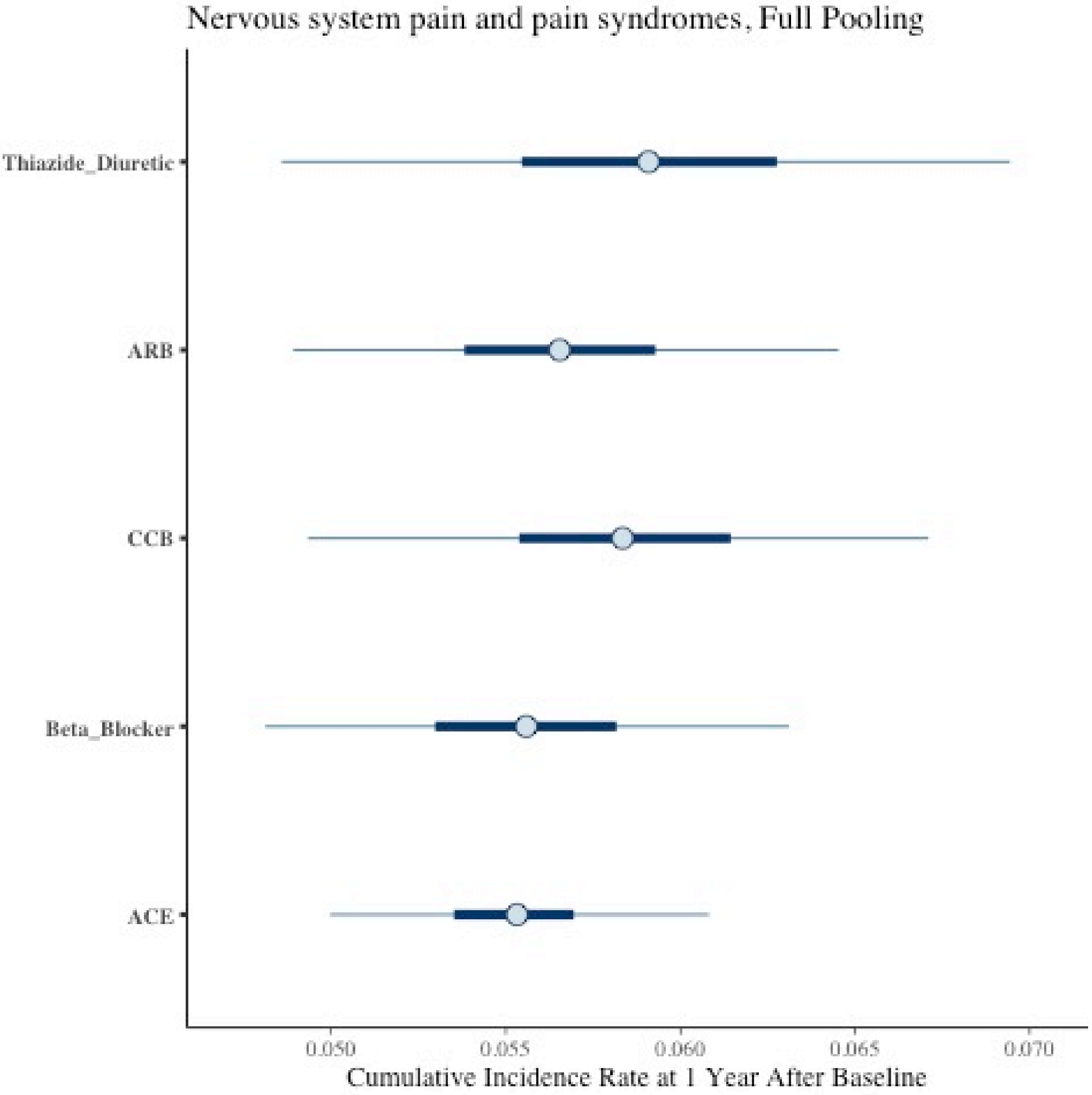

Nervous system pain and pain syndromes, Full Pooling
Thiazide_Diuretic
ARB
CCB
Beta_Blocker
ACE
0.050
0.055
0.060
0.065
0.070
Cumulative Incidence Rate at 1 Year After Baseline

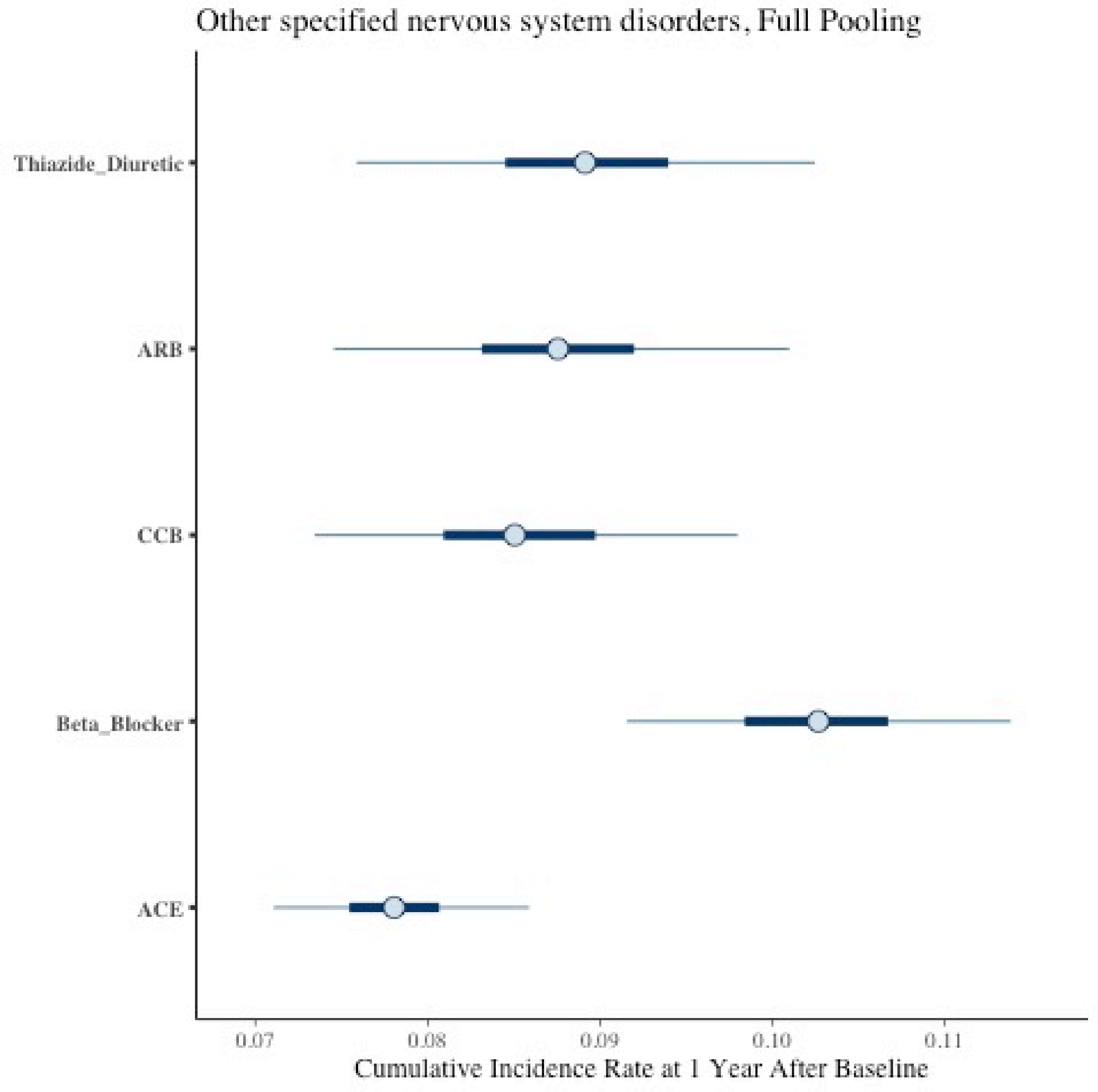

Other specified nervous system disorders, Full Pooling
Thiazide_Diuretic
ARB
CCB
Beta_Blocker
ACE
0.07
0.08
0.09
0.10
0.11
Cumulative Incidence Rate at 1 Year After Baseline

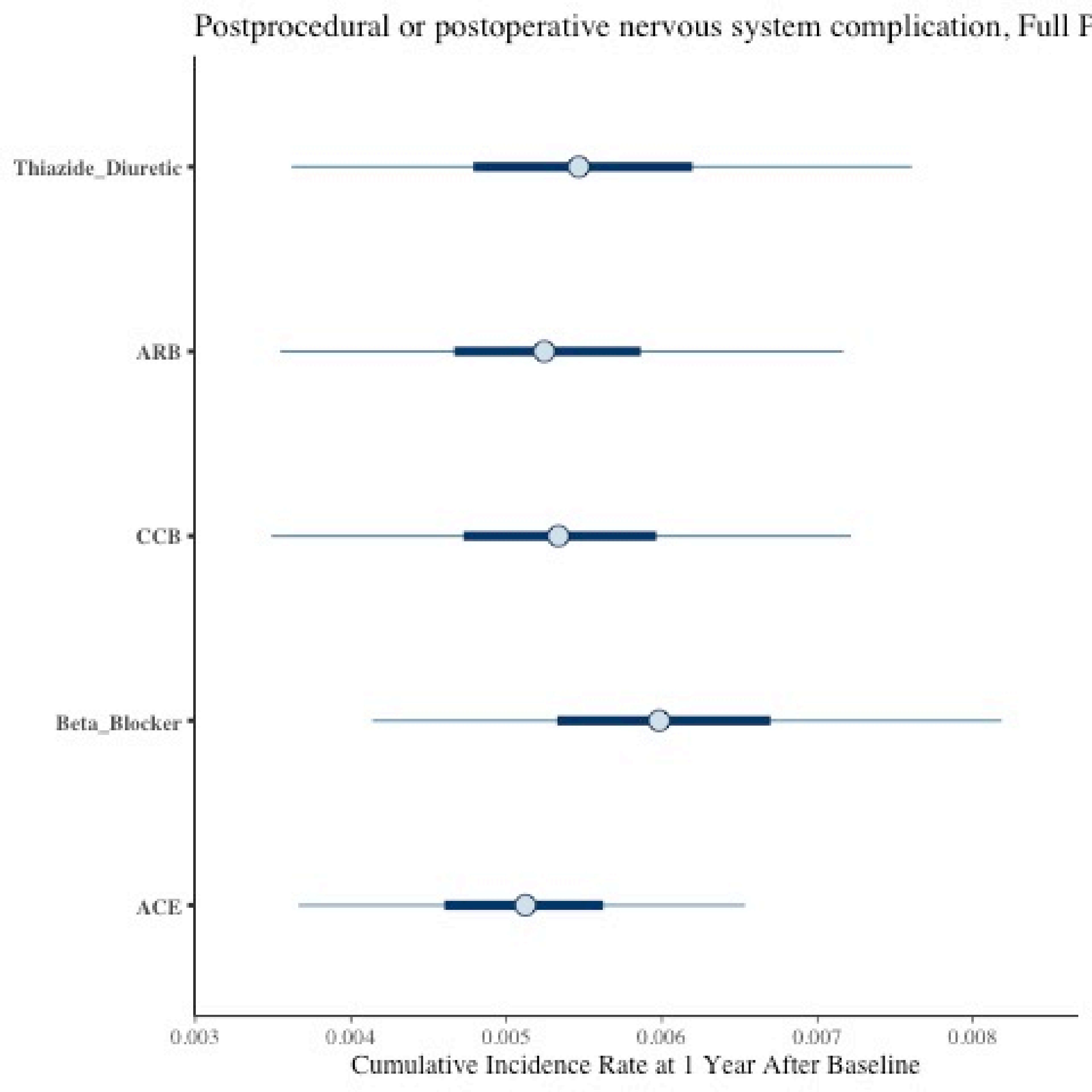

Postprocedural or postoperative nervous system complication, Full P
Thiazide_Diuretic
ARB
CCB
Beta_Blocker
ACE
0.003
0.004
0.005
0.006
0.007
0.008
Cumulative Incidence Rate at 1 Year After Baseline

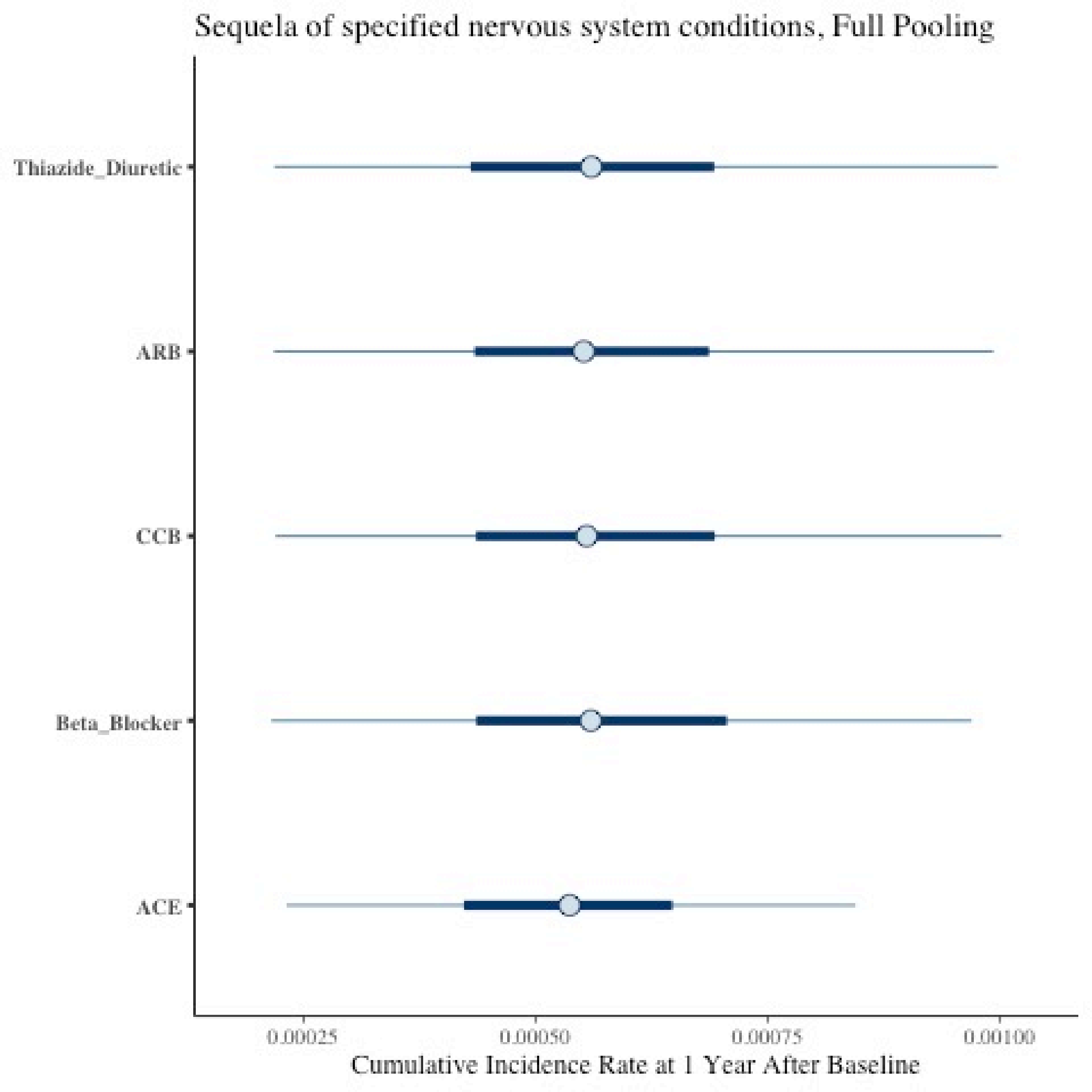

Sequela of specified nervous system conditions, Full Pooling
Thiazide_Diuretic
ARB
CCB
Beta_Blocker
ACE
0.00025
0.00050
0.00075
0.00100
Cumulative Incidence Rate at 1 Year After Baseline

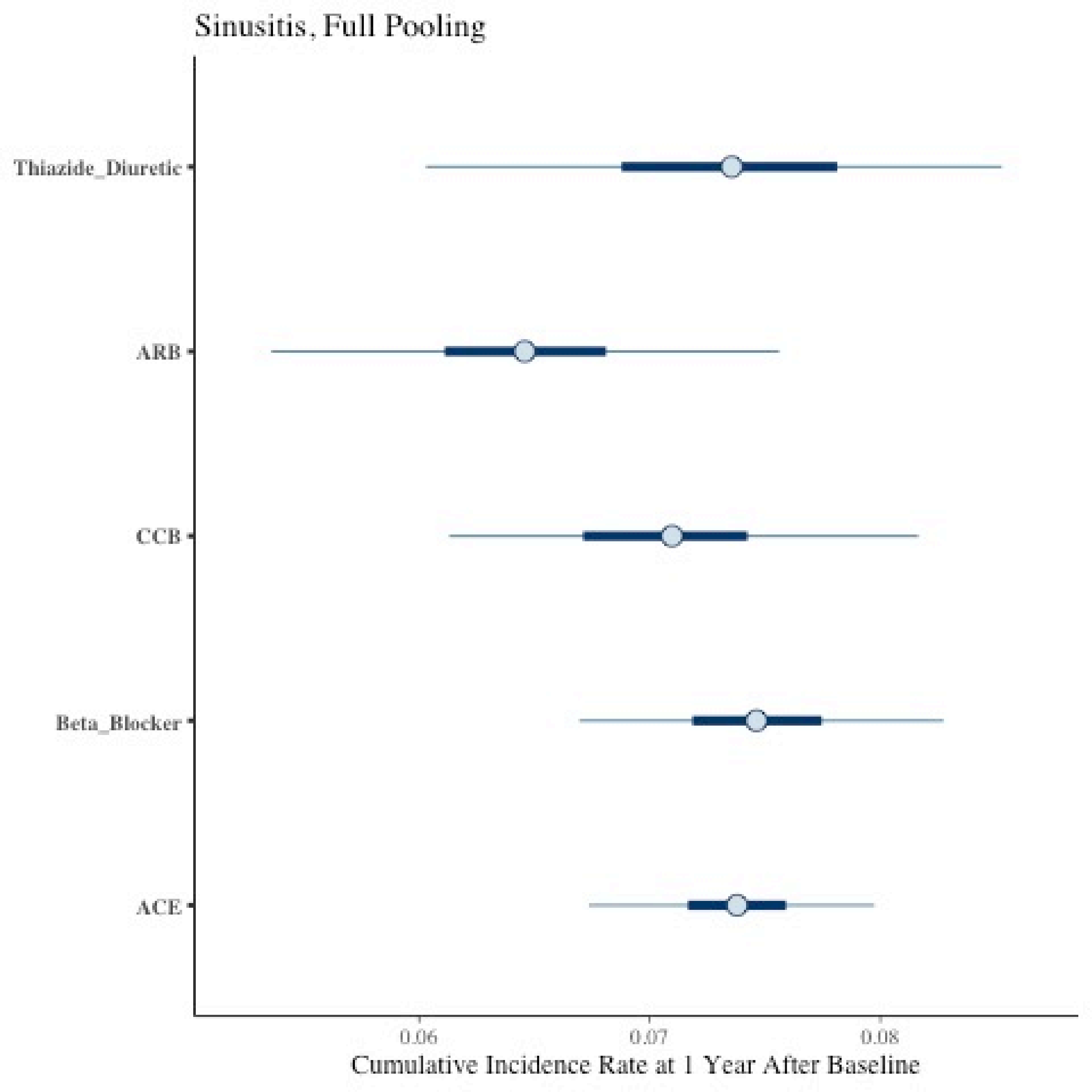

Sinusitis, Full Pooling
Thiazide_Diuretic
ARB
CCB
Beta_Blocker
ACE
0.06
0.07
0.08
Cumulative Incidence Rate at 1 Year After Baseline

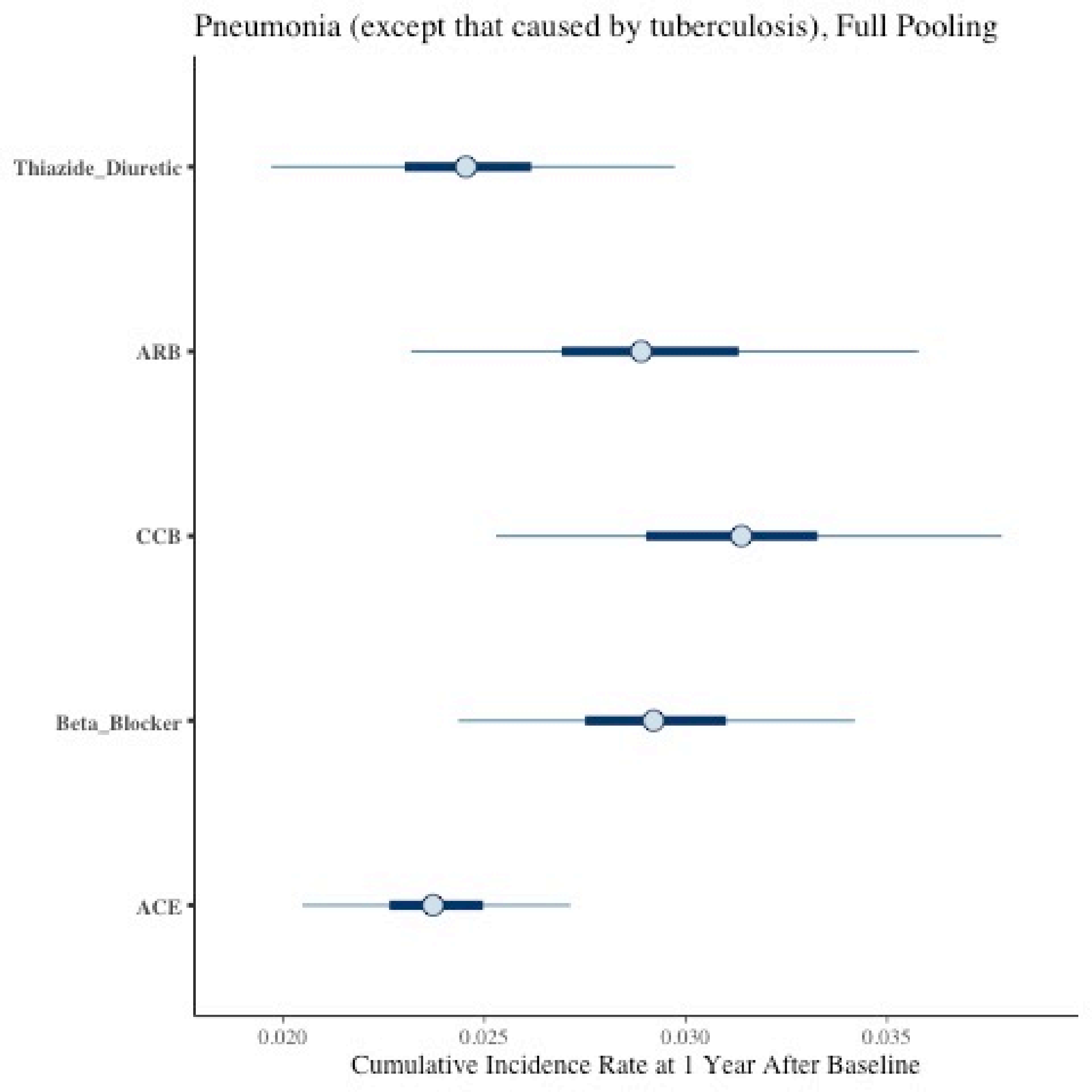

Pneumonia (except that caused by tuberculosis), Full Pooling
Thiazide_Diuretic
ARB
CCB
Beta_Blocker
ACE
0.020
0.025
0.030
0.035
Cumulative Incidence Rate at 1 Year After Baseline

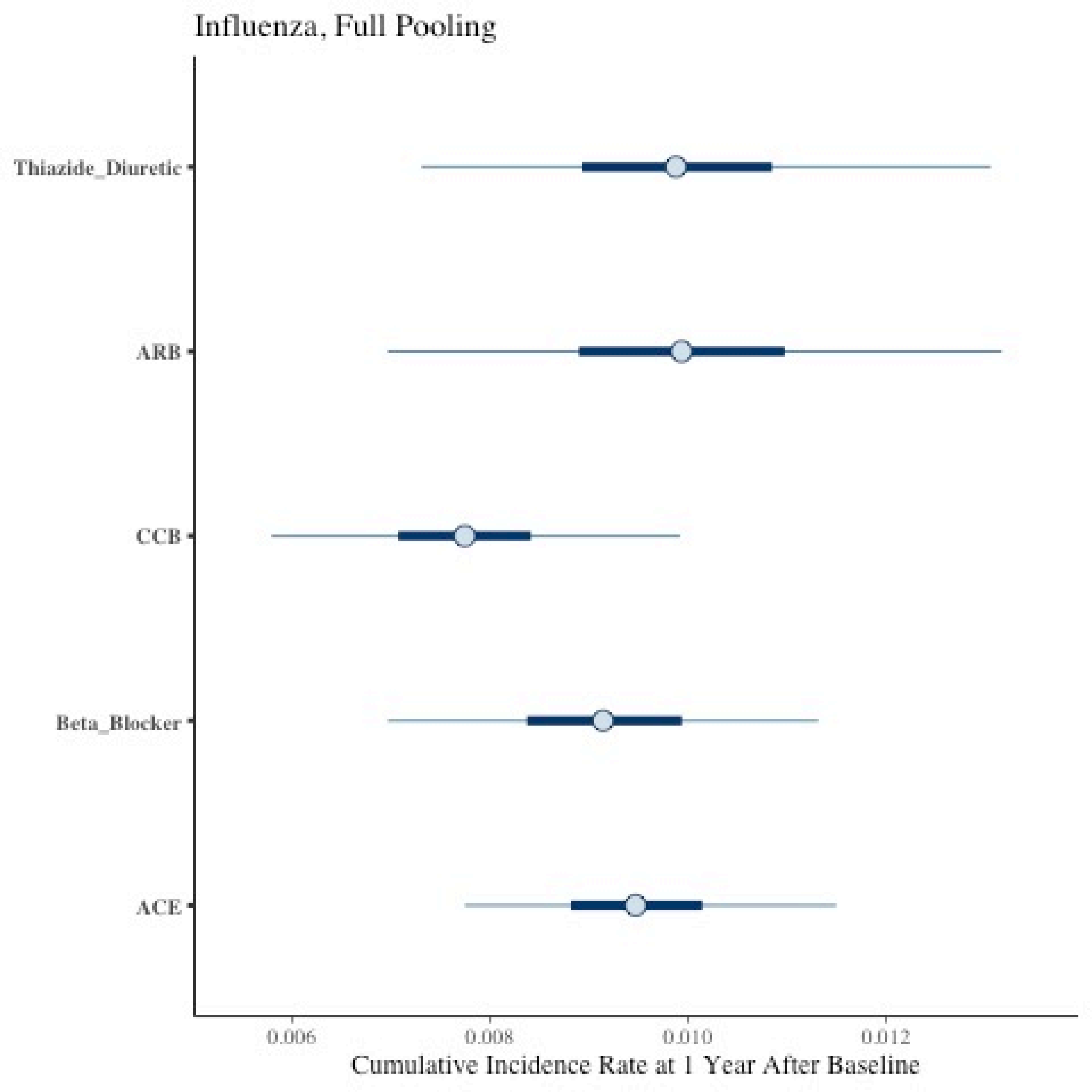
Influenza, Full Pooling
Thiazide_Diuretic
ARB
CCB
Beta_Blocker
ACE
0.006
0.008
0.010
0.012
Cumulative Incidence Rate at 1 Year After Baseline

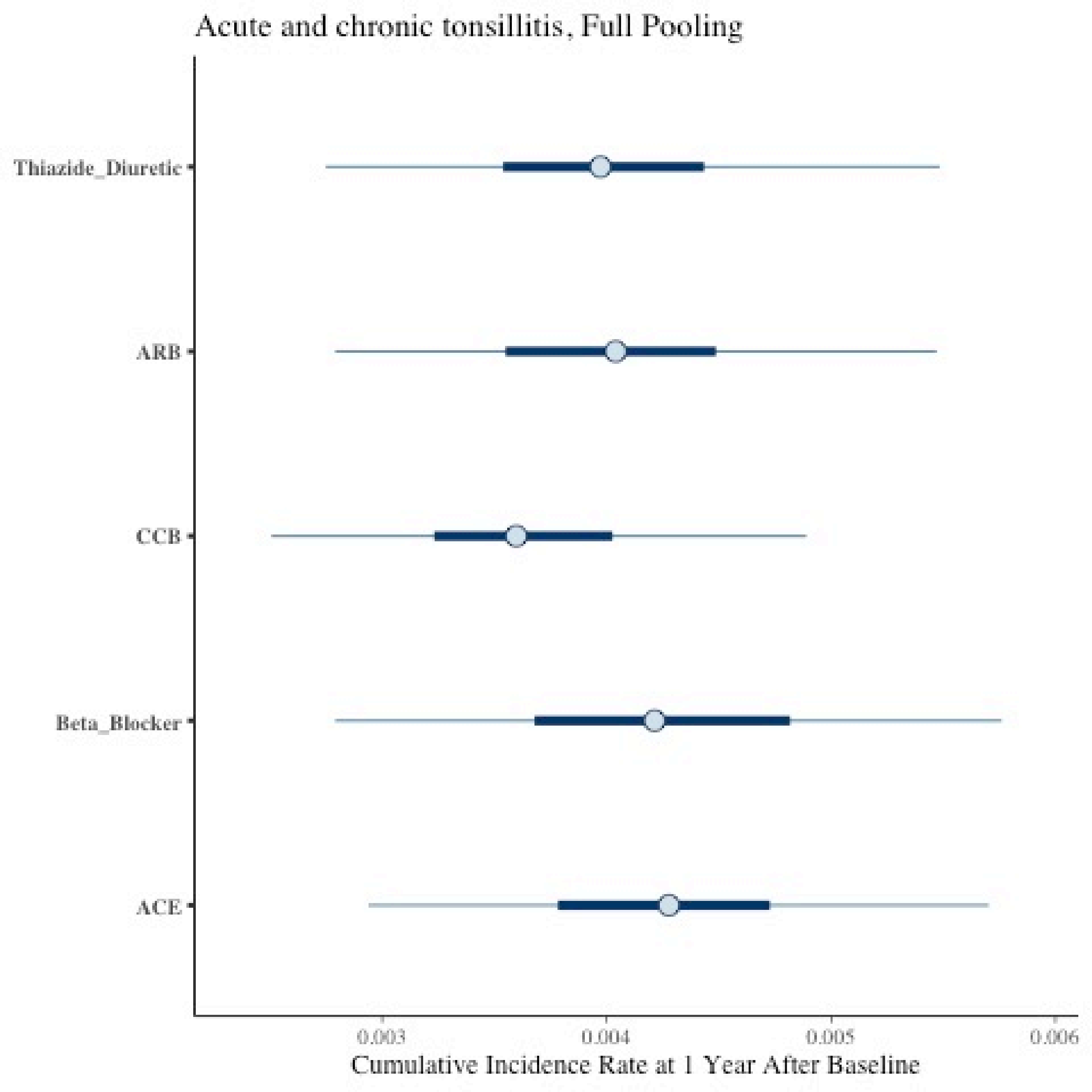

Acute and chronic tonsillitis, Full Pooling
Thiazide_Diuretic
ARB
CCB
Beta_Blocker
ACE
0.003
0.004
0.005
0.006
Cumulative Incidence Rate at 1 Year After Baseline

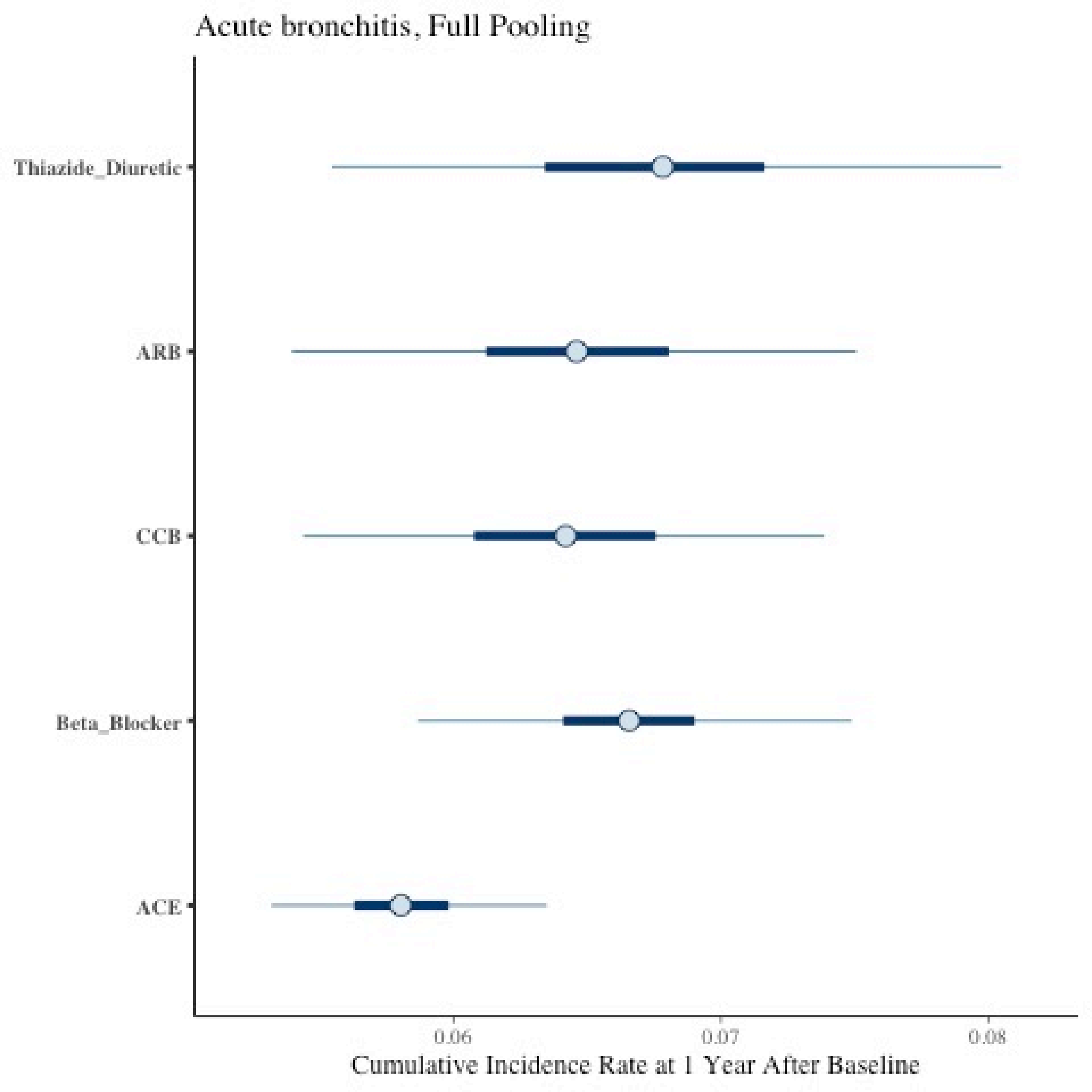

Acute bronchitis, Full Pooling
Thiazide_Diuretic
ARB
CCB
Beta_Blocker
ACE
0.06
0.07
0.08
Cumulative Incidence Rate at 1 Year After Baseline

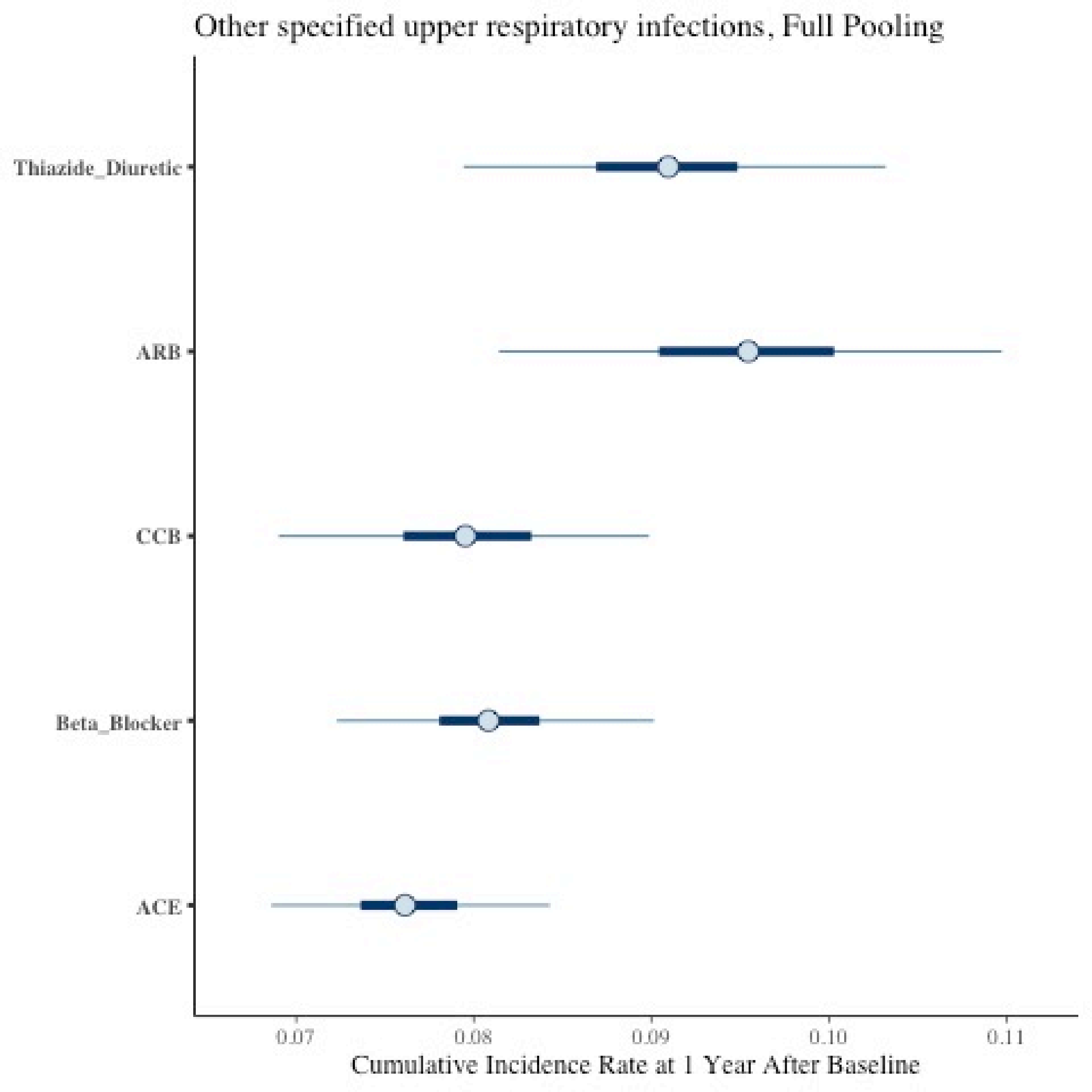

Other specified upper respiratory infections, Full Pooling
Thiazide_Diuretic
ARB
CCB
Beta_Blocker
ACE
0.07
0.08
0.09
0.10
0.11
Cumulative Incidence Rate at 1 Year After Baseline

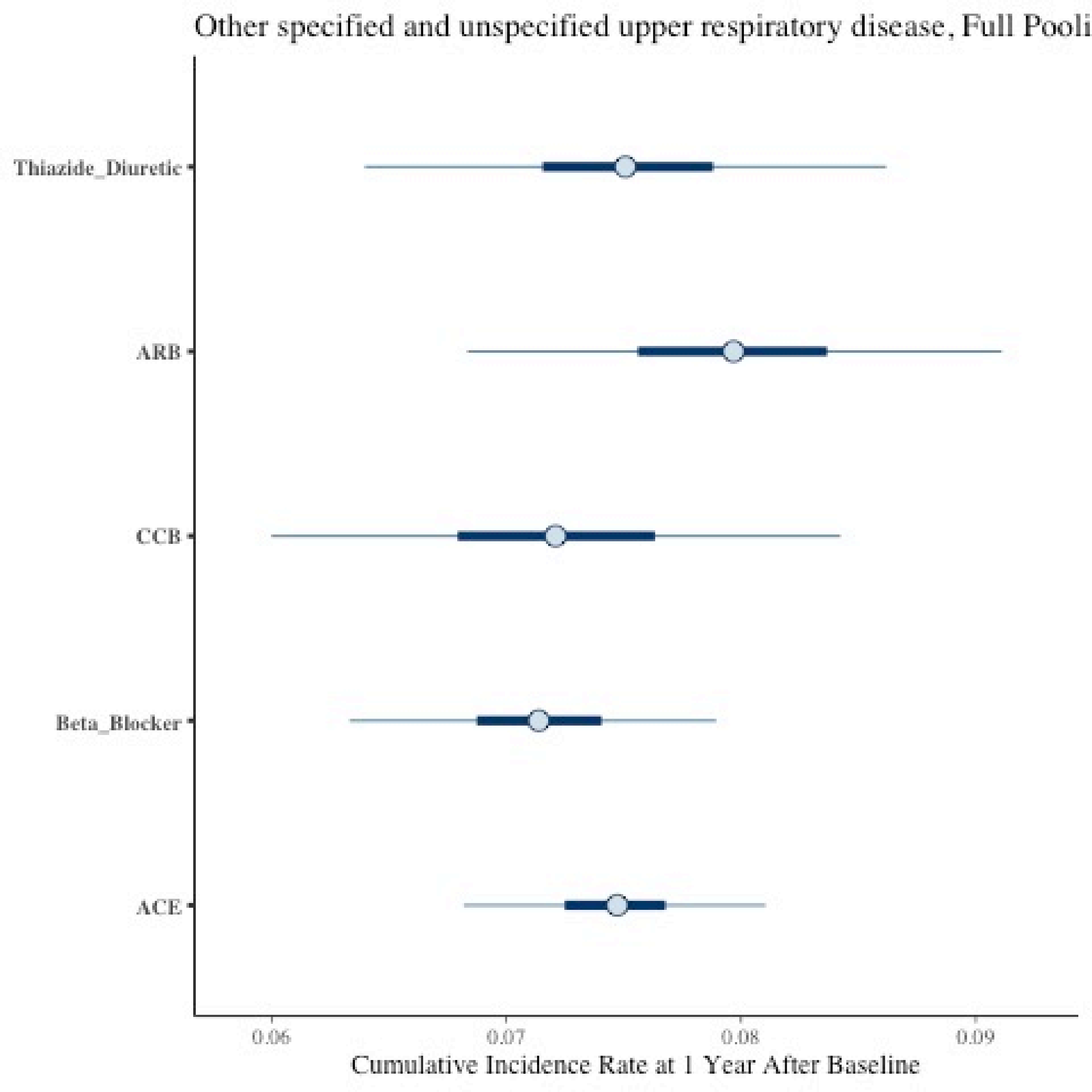

Other specified and unspecified upper respiratory disease, Full Pooli
Thiazide_Diuretic
ARB
CCB
Beta_Blocker
ACE
0.06
0.07
0.08
0.09
Cumulative Incidence Rate at 1 Year After Baseline

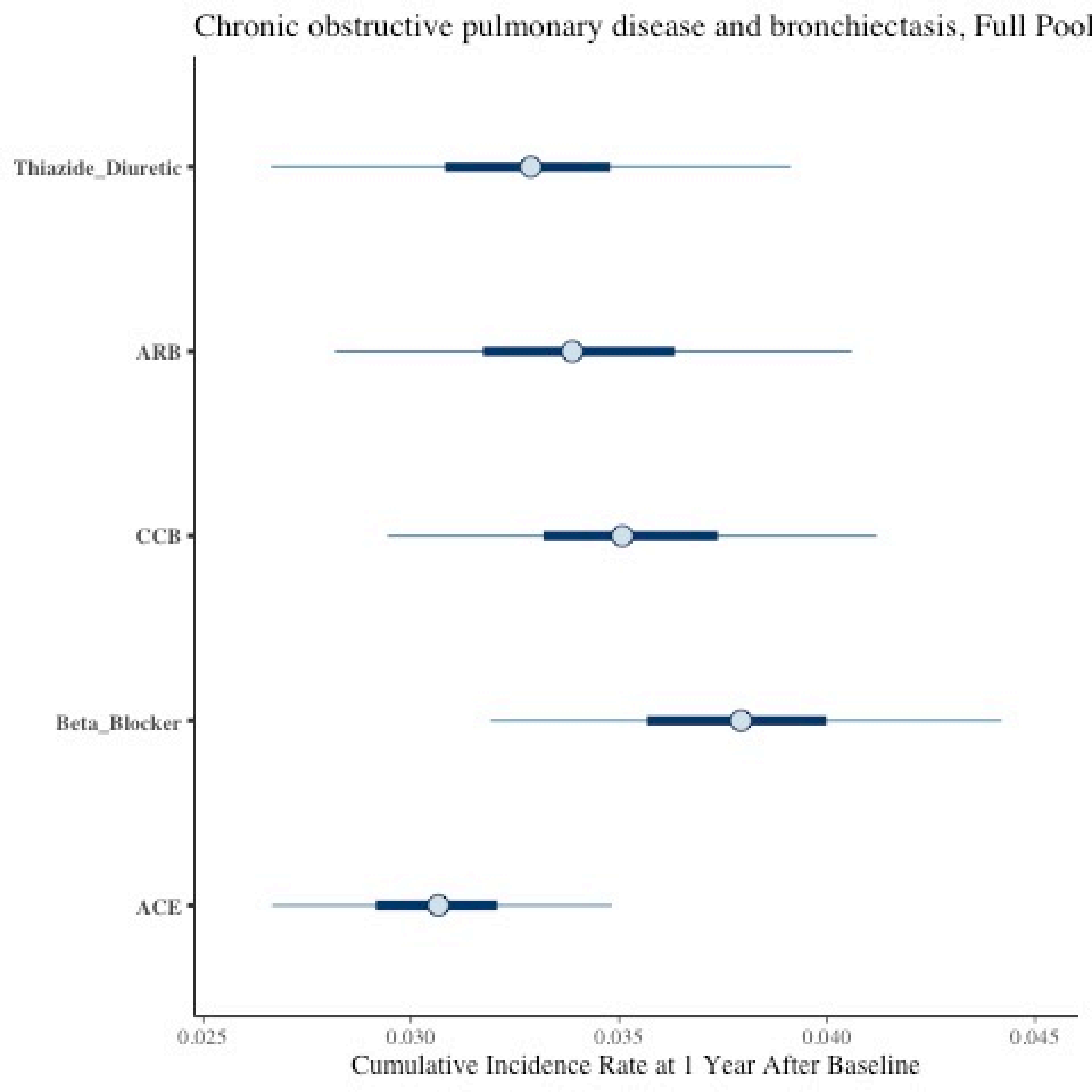

Chronic obstructive pulmonary disease and bronchiectasis, Full Pool
Thiazide_Diuretic
ARB
CCB
Beta_Blocker
ACE
0.025
0.030
0.035
0.040
0.045
Cumulative Incidence Rate at 1 Year After Baseline

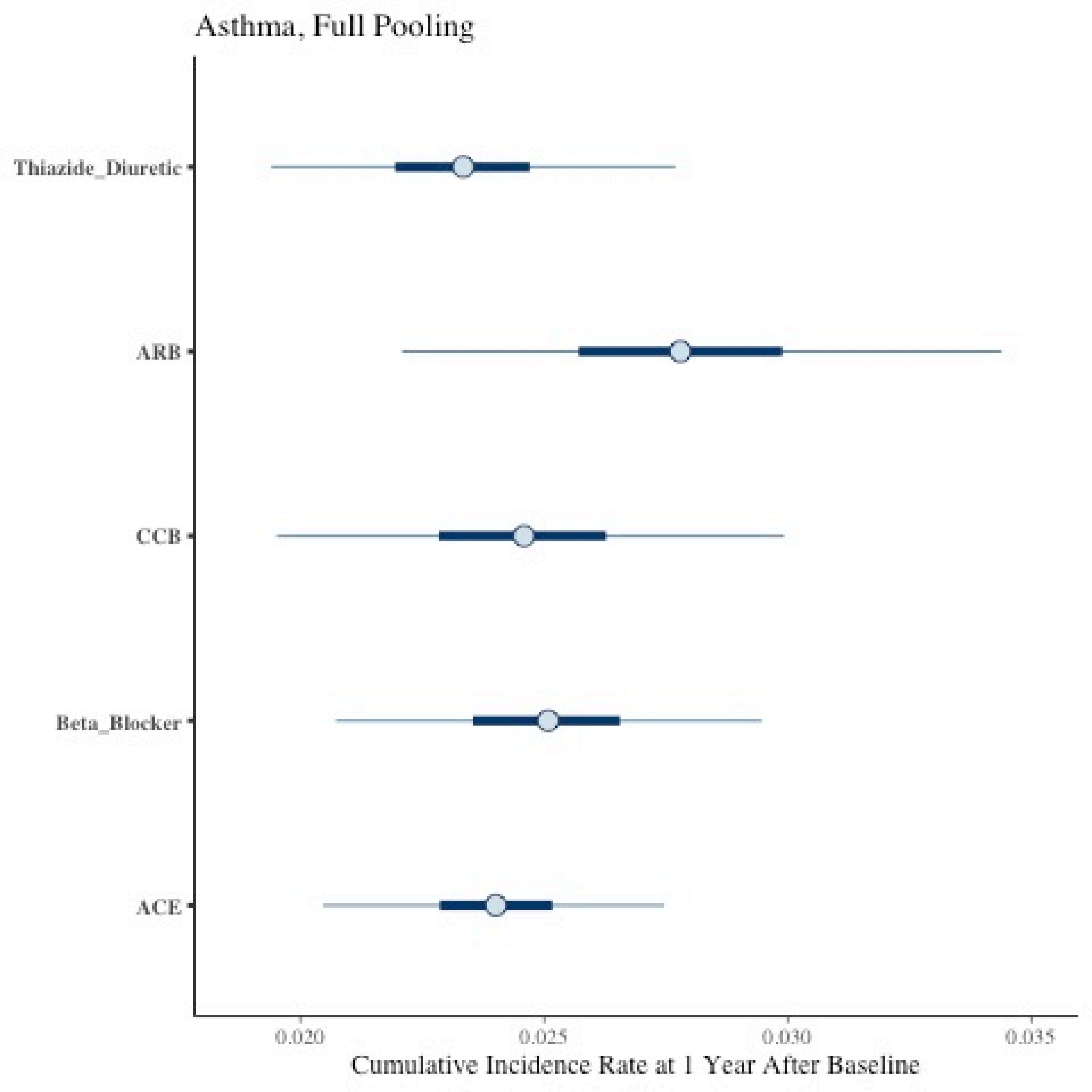

Asthma, Full Pooling
Thiazide_Diuretic
ARB
CCB
Beta_Blocker
ACE
0.020
0.025
0.030
0.035
Cumulative Incidence Rate at 1 Year After Baseline

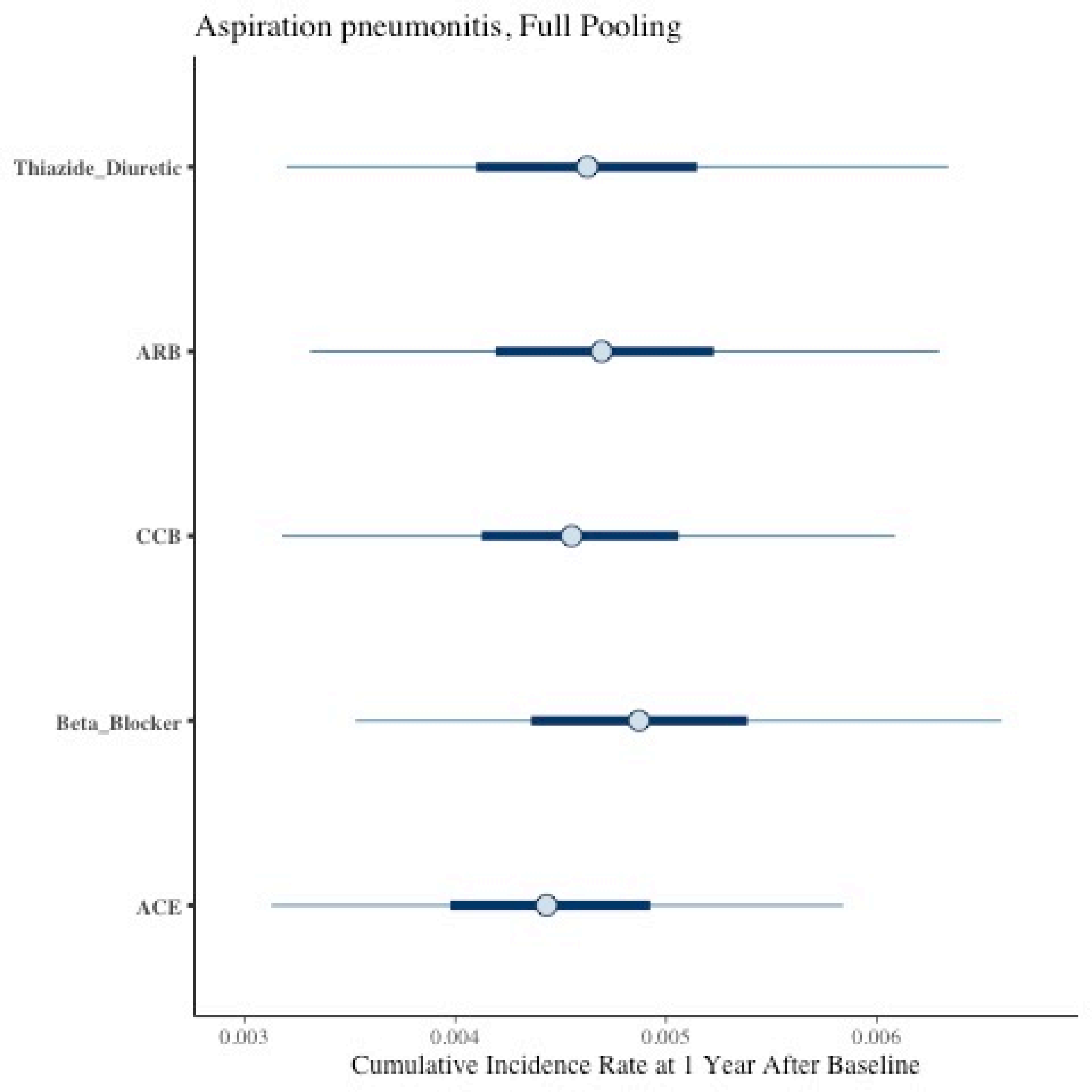
Aspiration pneumonitis, Full Pooling
Thiazide_Diuretic
ARB
CCB
Beta_Blocker
ACE
0.003
0.004
0.005
0.006
Cumulative Incidence Rate at 1 Year After Baseline

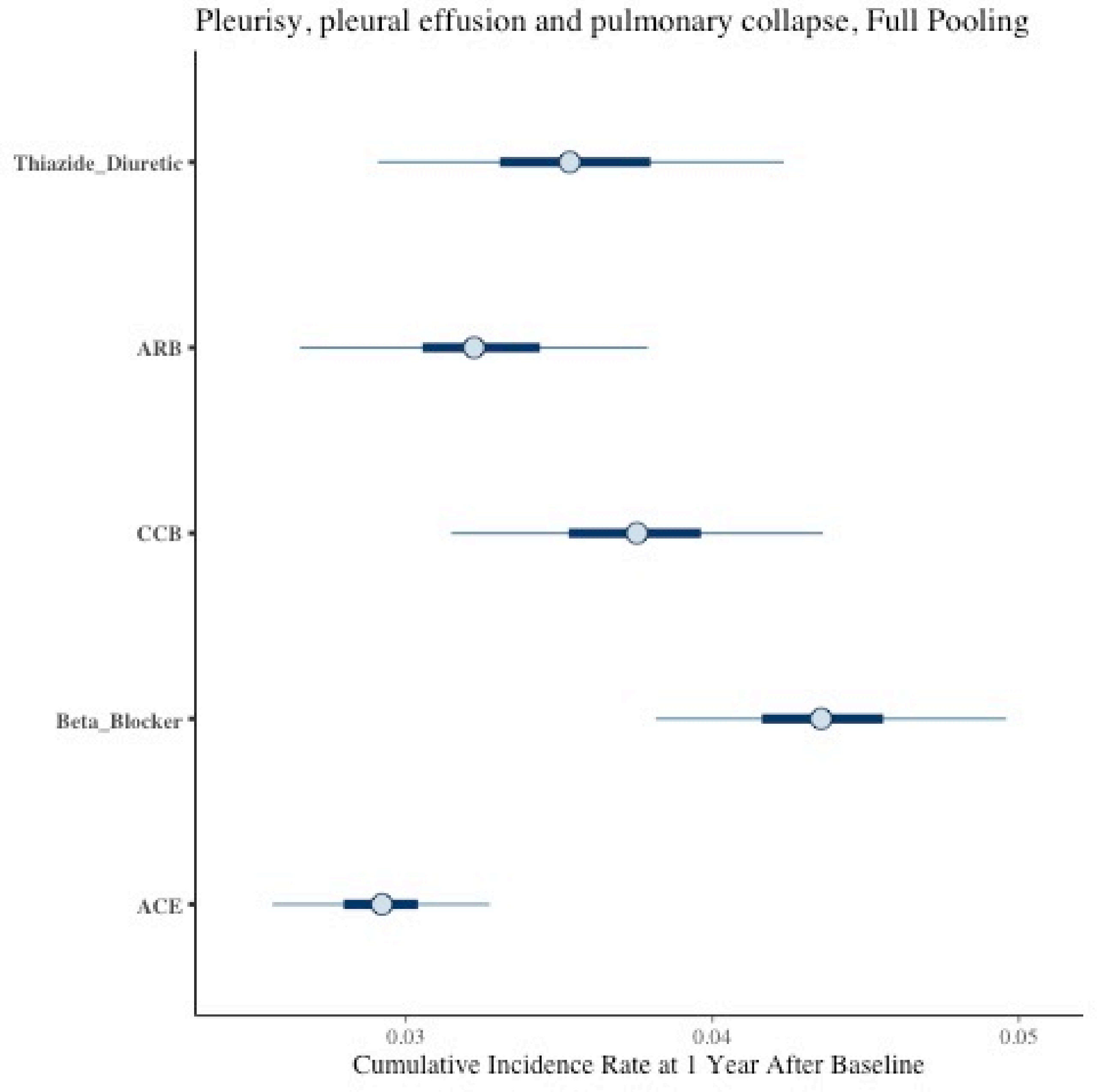

Pleurisy, pleural effusion and pulmonary collapse, Full Pooling
Thiazide_Diuretic
ARB
CCB
Beta_Blocker
ACE
0.03
0.04
0.05
Cumulative Incidence Rate at 1 Year After Baseline

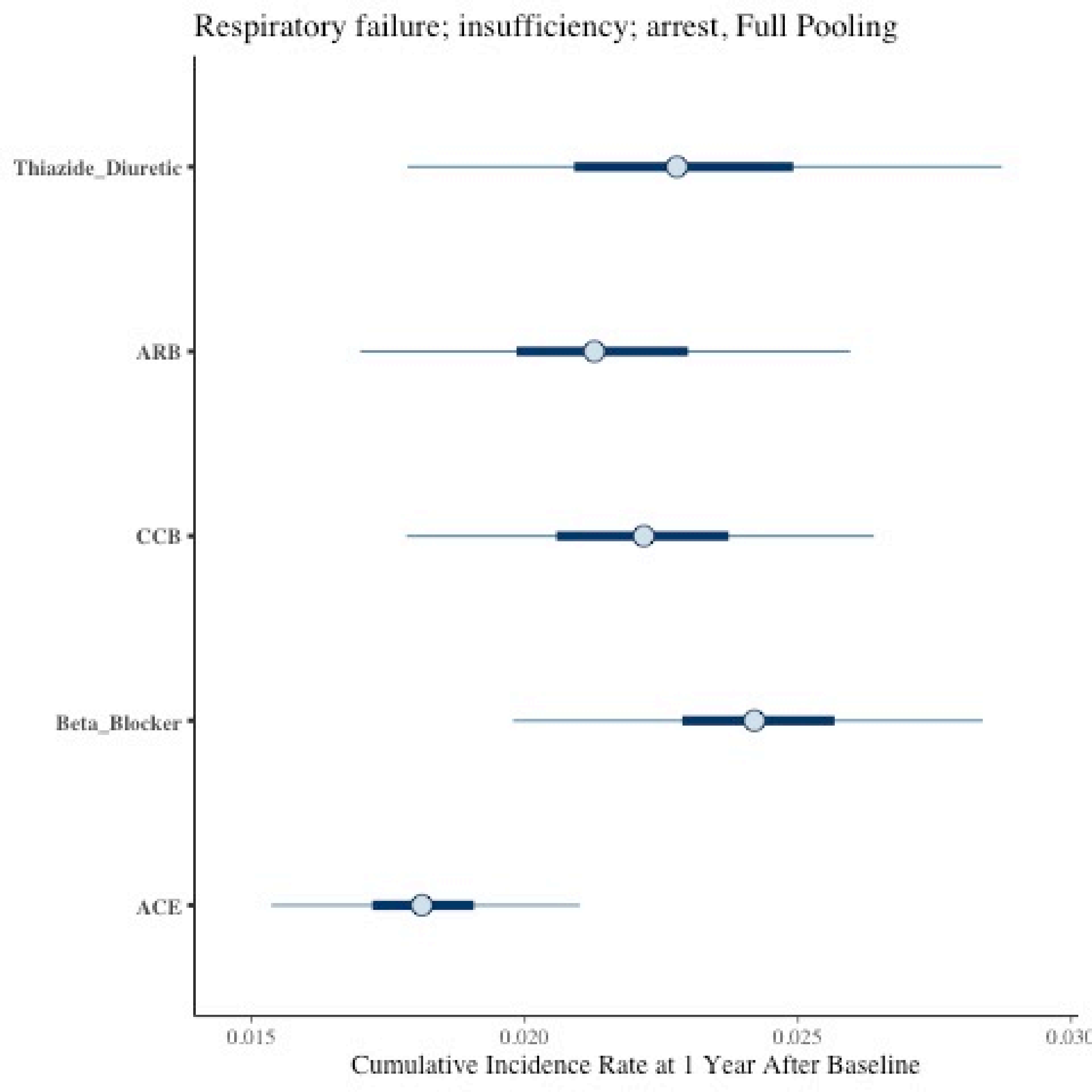

Respiratory failure; insufficiency; arrest, Full Pooling
Thiazide_Diuretic
ARB
CCB
Beta_Blocker
ACE
0.015
0.020
0.025
0.030
Cumulative Incidence Rate at 1 Year After Baseline

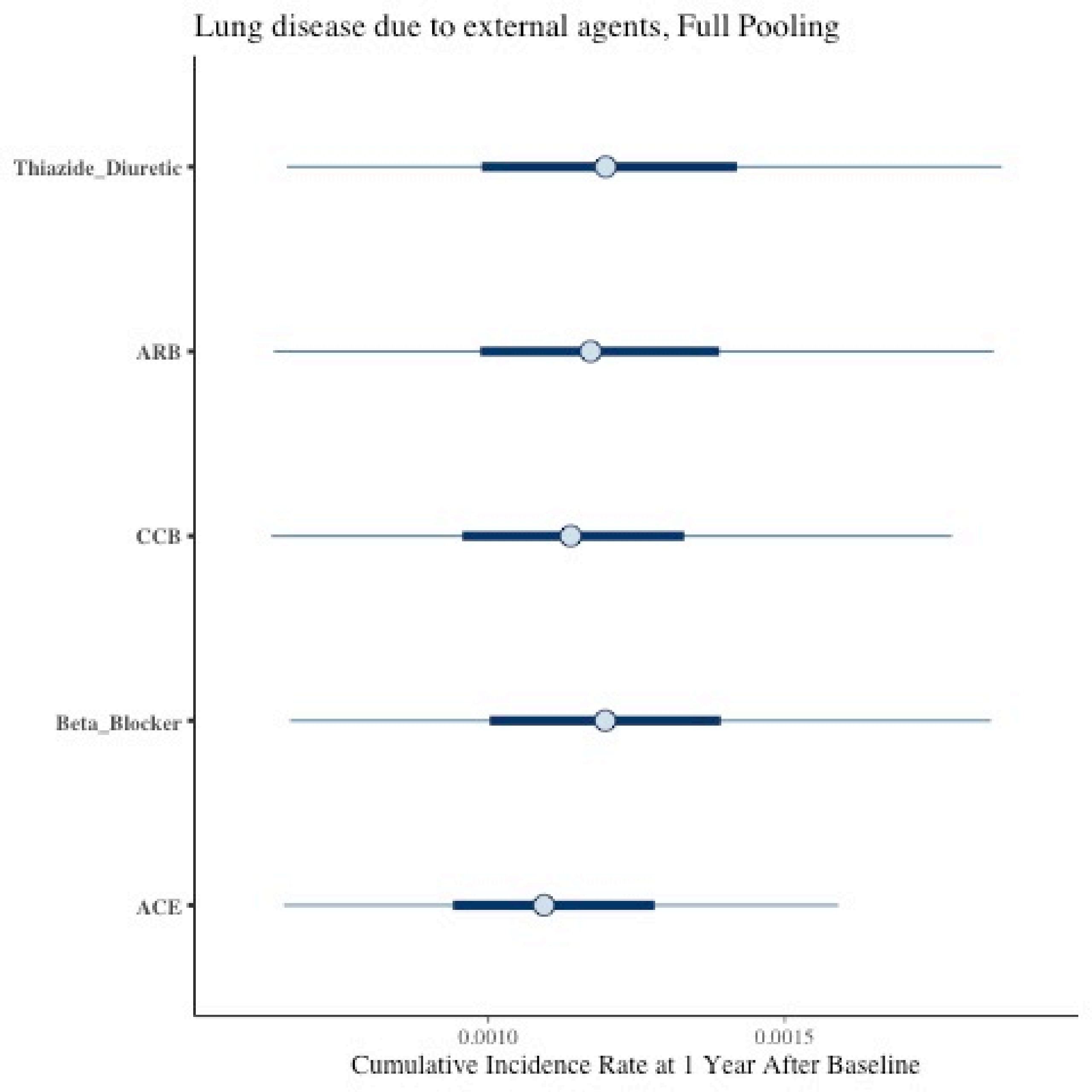

Lung disease due to external agents, Full Pooling
Thiazide_Diuretic
ARB
CCB
Beta_Blocker
ACE
0.0010
0.0015
Cumulative Incidence Rate at 1 Year After Baseline

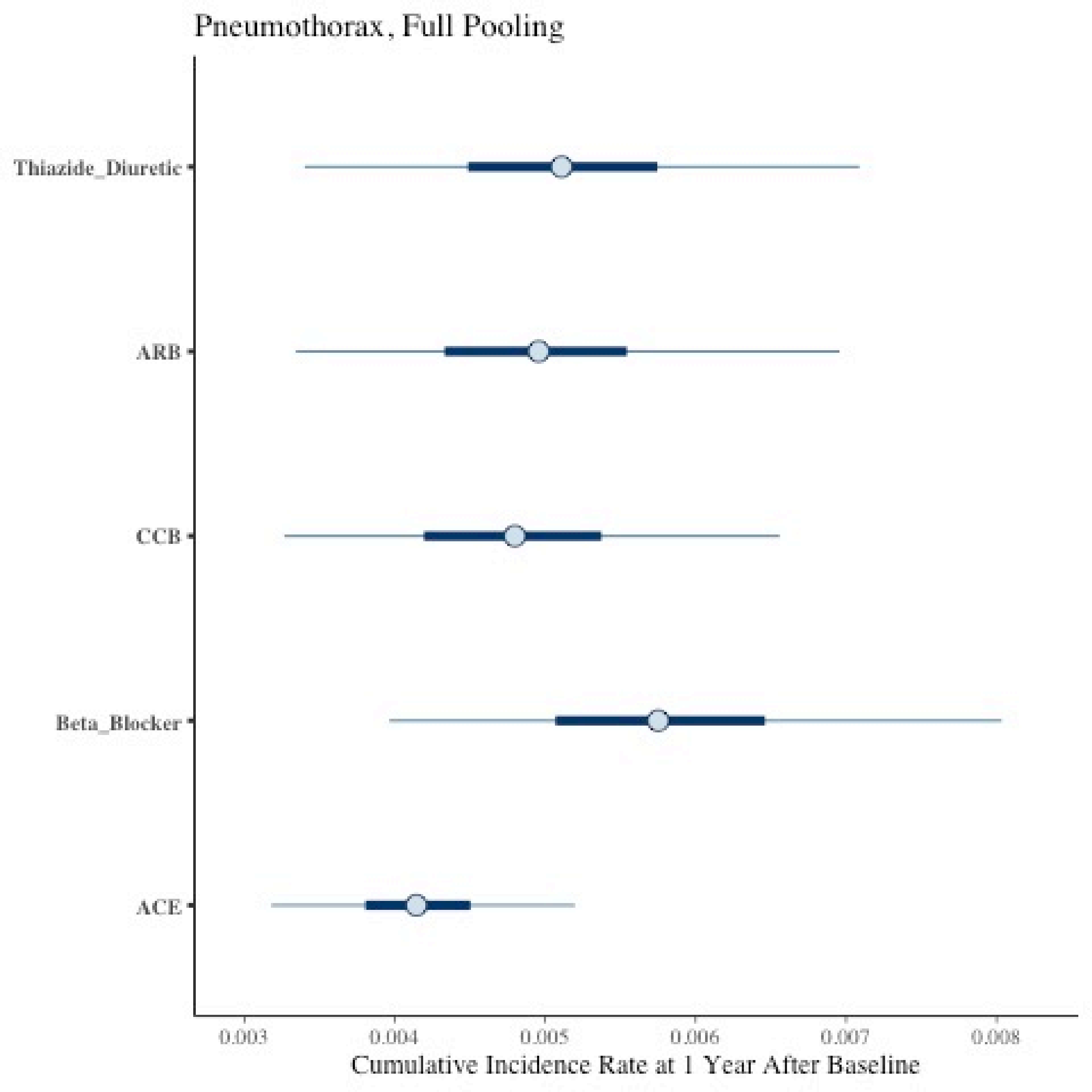

Pneumothorax, Full Pooling
Thiazide_Diuretic
ARB
CCB
Beta_Blocker
ACE
0.003
0.004
0.005
0.006
0.007
0.008
Cumulative Incidence Rate at 1 Year After Baseline

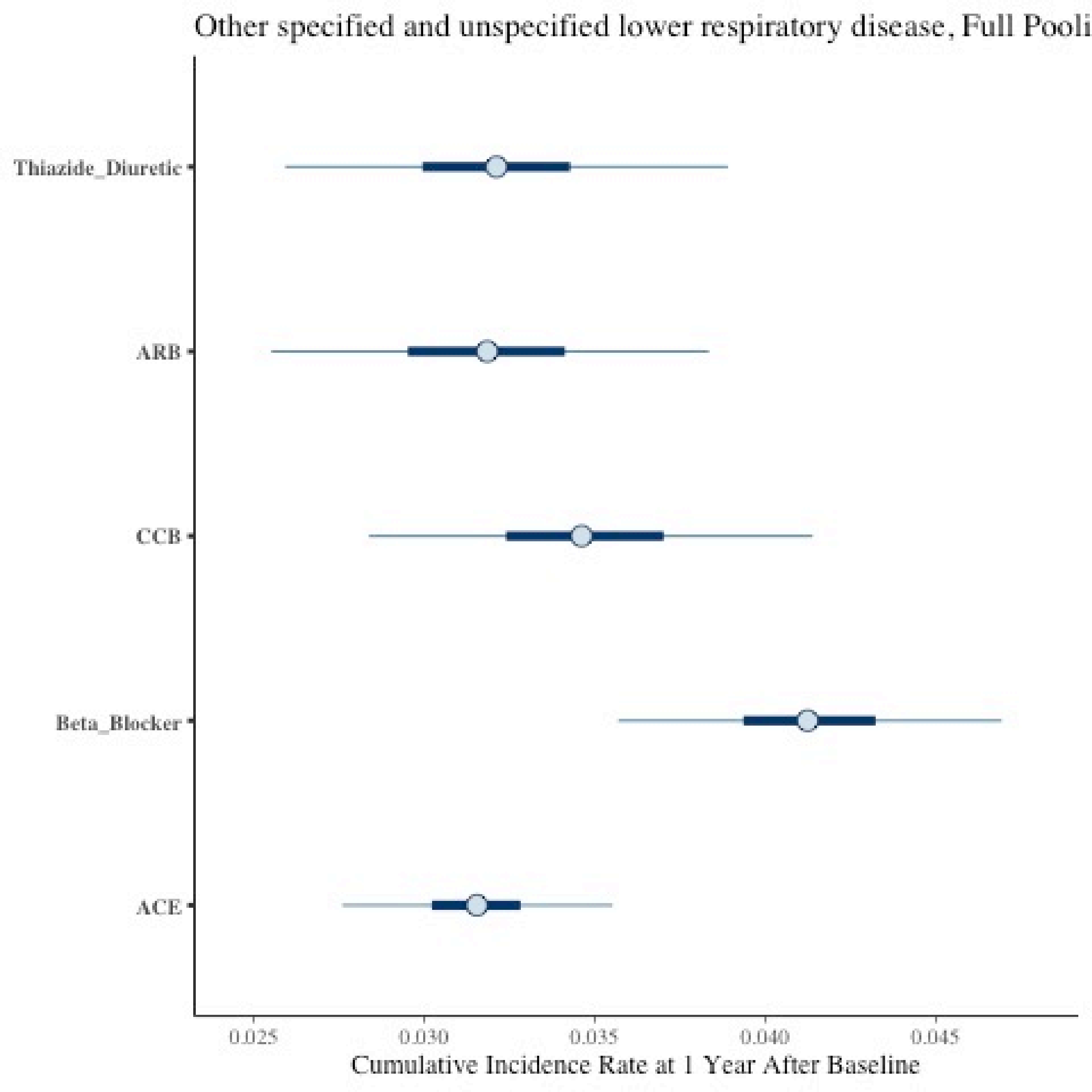

Other specified and unspecified lower respiratory disease, Full Pooli
Thiazide_Diuretic
ARB
CCB
Beta_Blocker
ACE
0.025
0.030
0.035
0.040
0.045
Cumulative Incidence Rate at 1 Year After Baseline

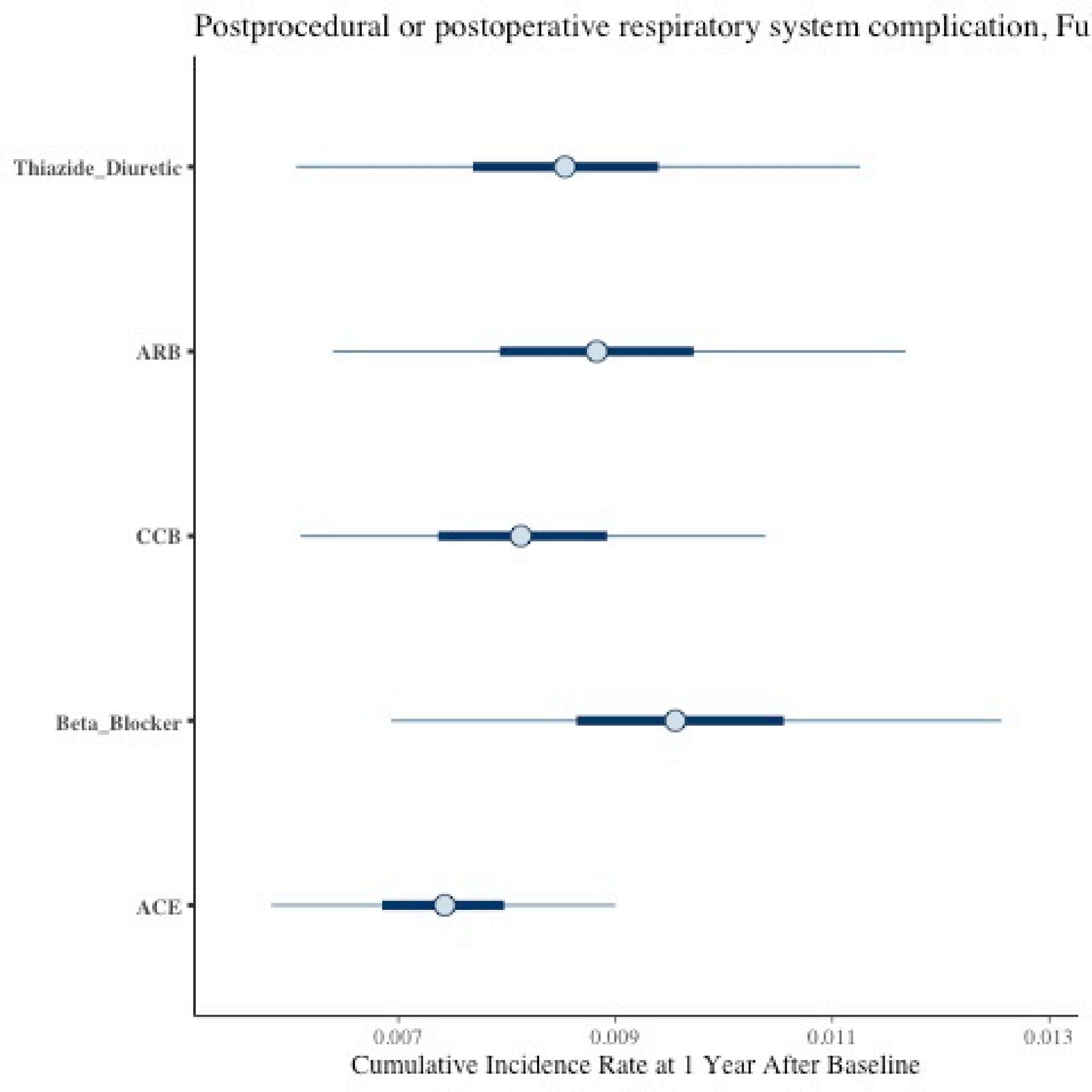

Postprocedural or postoperative respiratory system complication, Fu
Thiazide_Diuretic
ARB
CCB
Beta_Blocker
ACE
0.007
0.009
0.011
0.013
Cumulative Incidence Rate at 1 Year After Baseline

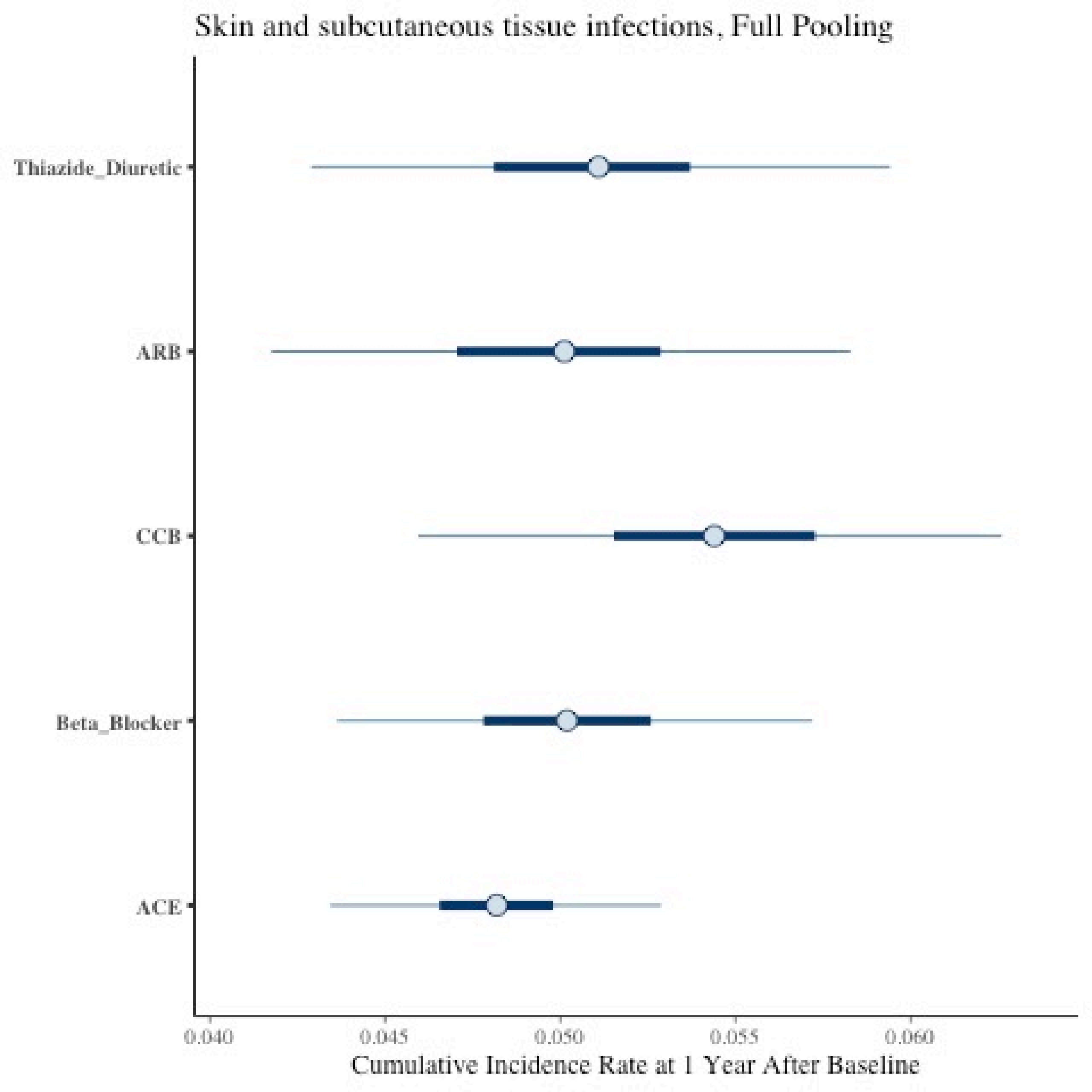
Skin and subcutaneous tissue infections, Full Pooling
Thiazide_Diuretic
ARB
CCB
Beta_Blocker
ACE
0.040
0.045
0.050
0.055
0.060
Cumulative Incidence Rate at 1 Year After Baseline

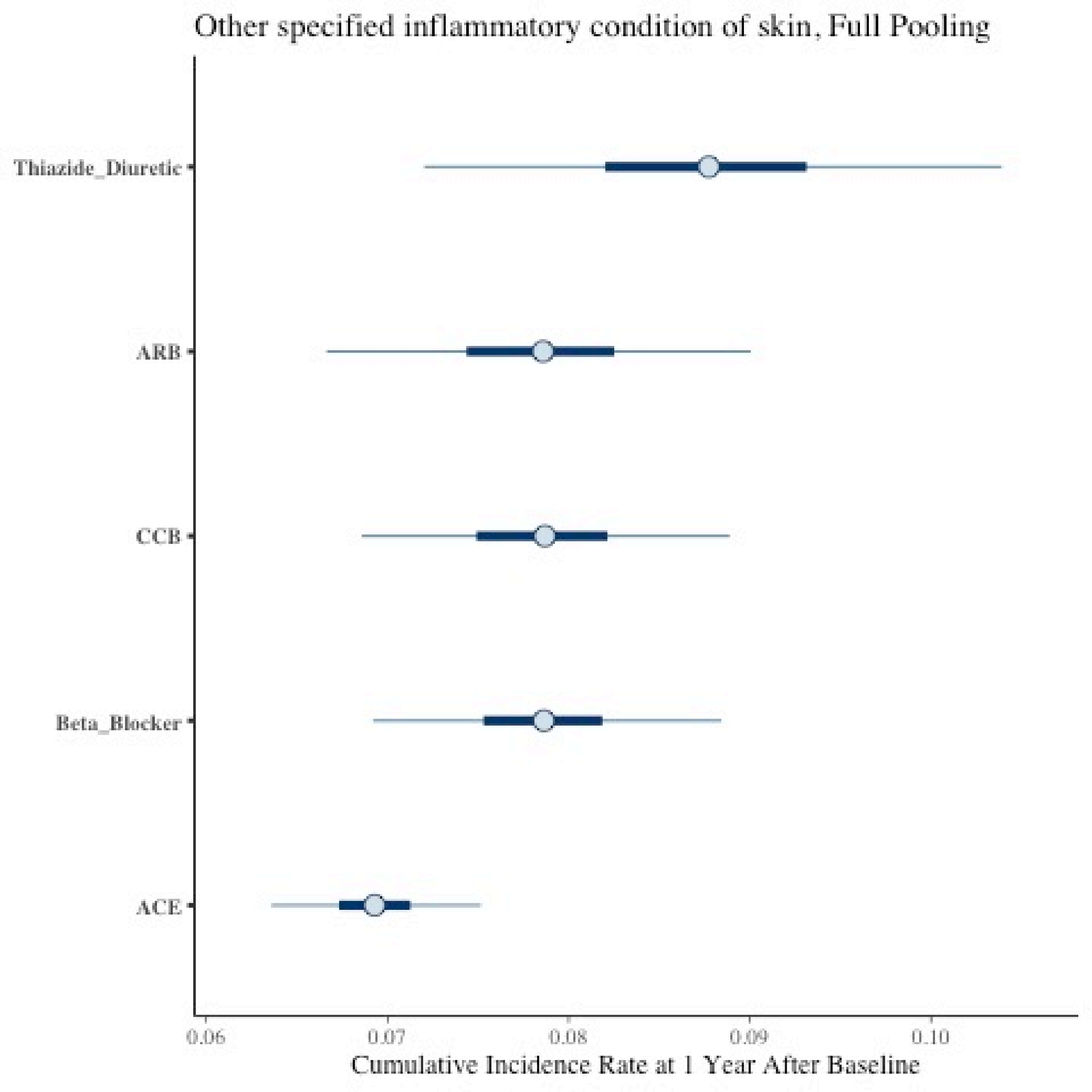

Other specified inflammatory condition of skin, Full Pooling
Thiazide_Diuretic
ARB
CCB
Beta_Blocker
ACE
0.06
0.07
0.08
0.09
0.10
Cumulative Incidence Rate at 1 Year After Baseline

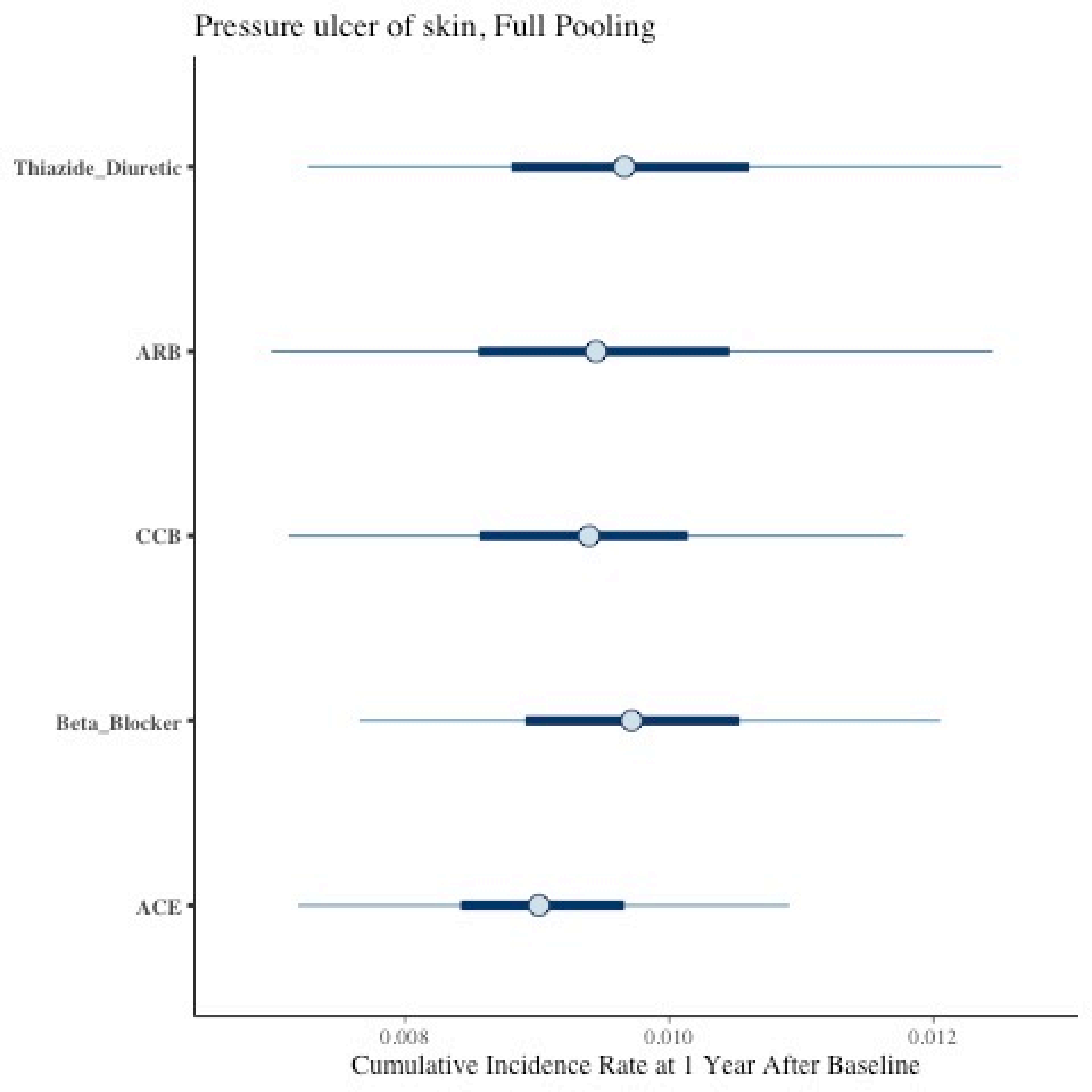

Pressure ulcer of skin, Full Pooling
Thiazide_Diuretic
ARB
CCB
Beta_Blocker
ACE
0.008
0.010
0.012
Cumulative Incidence Rate at 1 Year After Baseline

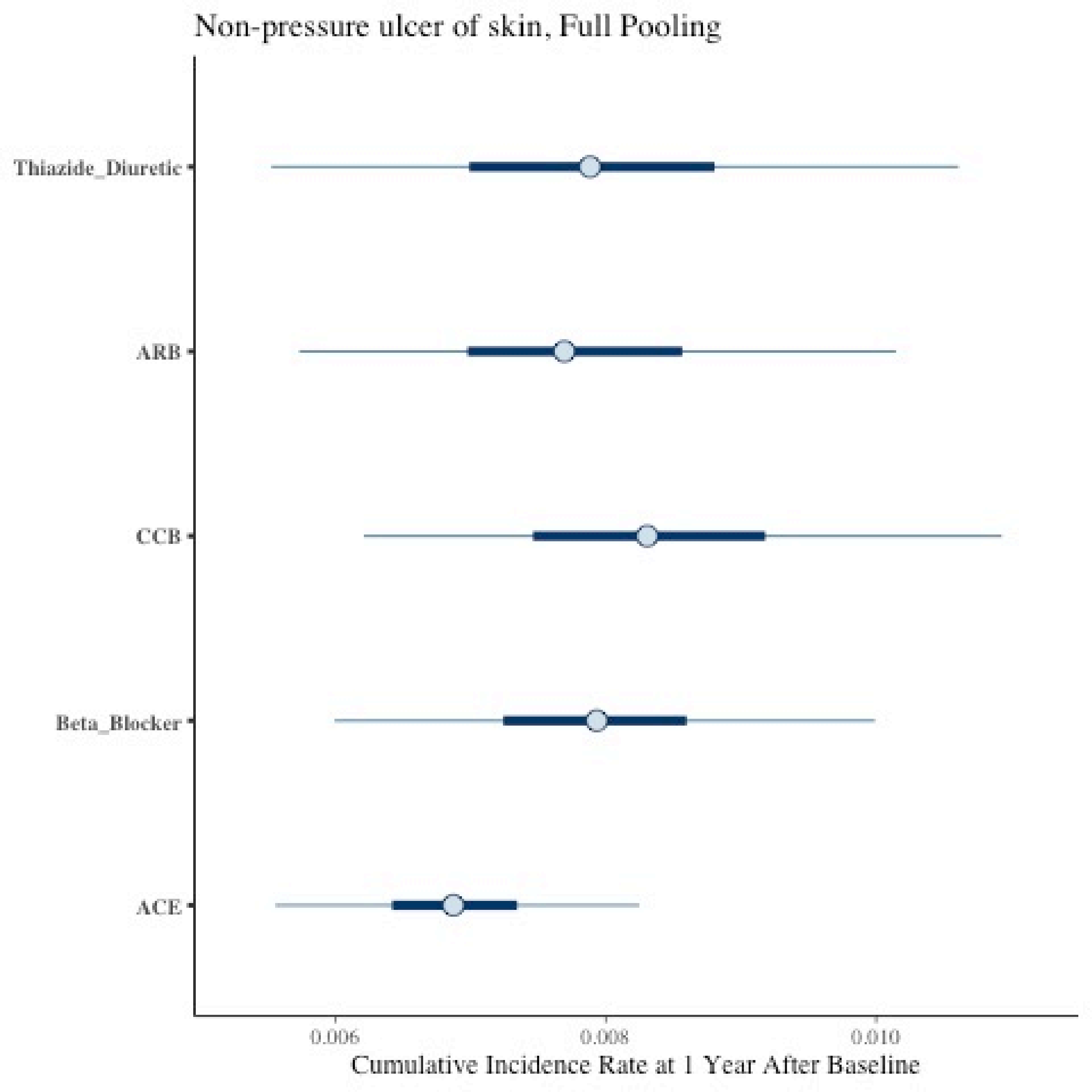
Non-pressure ulcer of skin, Full Pooling
Thiazide_Diuretic
ARB
CCB
Beta_Blocker
ACE
0.006
0.008
0.010
Cumulative Incidence Rate at 1 Year After Baseline

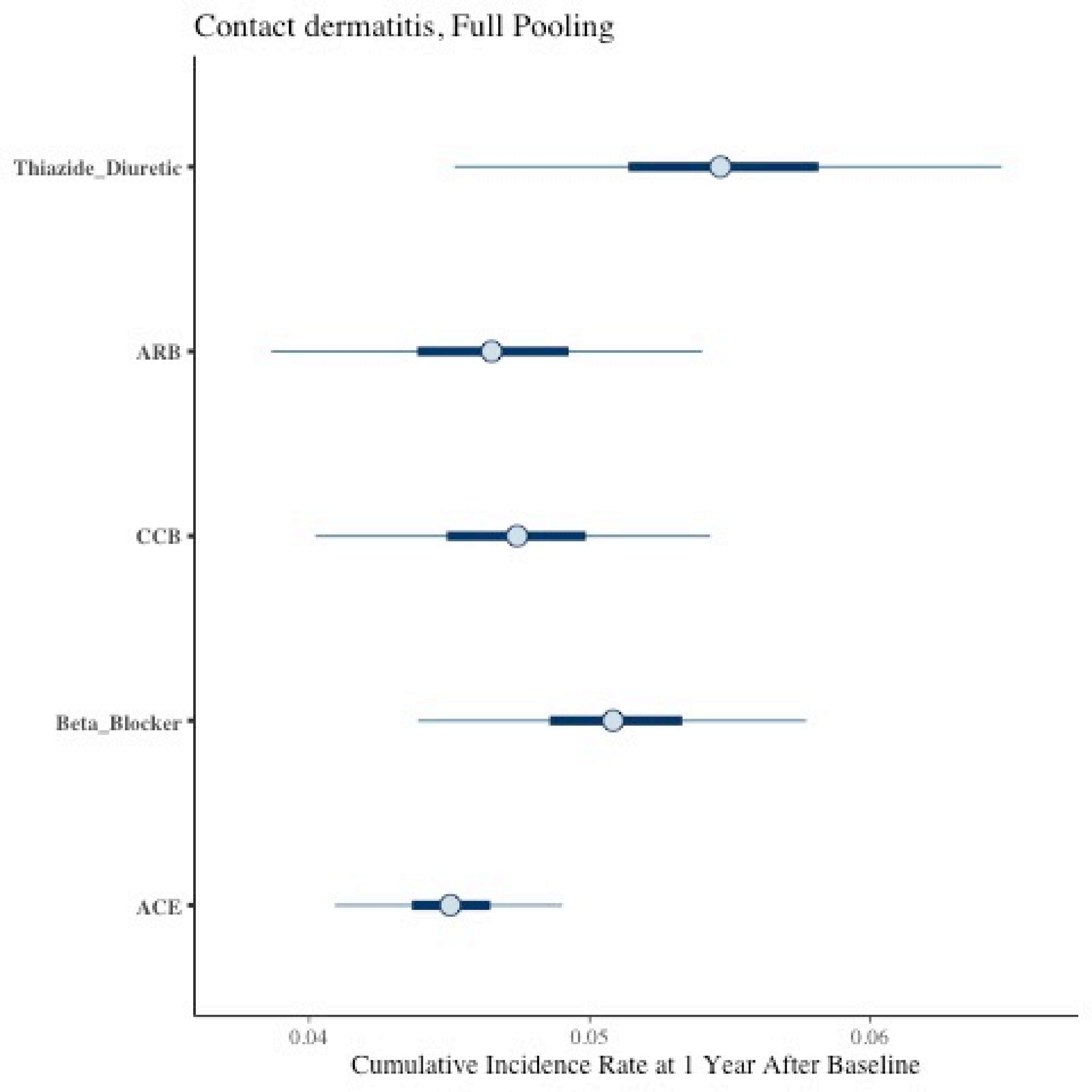

Contact dermatitis, Full Pooling
Thiazide_Diuretic
ARB
CCB
Beta_Blocker
ACE
0.04
0.05
0.06
Cumulative Incidence Rate at 1 Year After Baseline

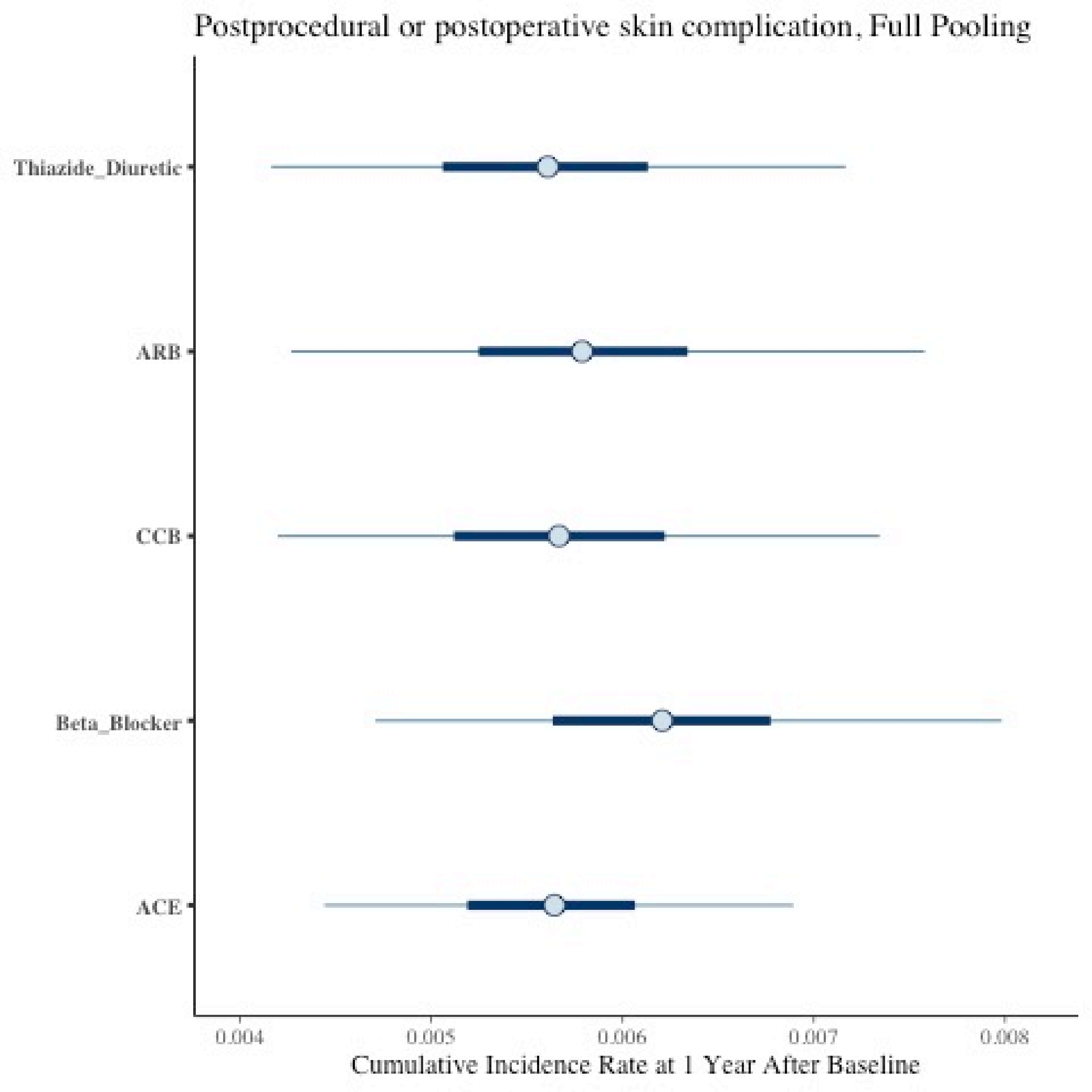
Postprocedural or postoperative skin complication, Full Pooling
Thiazide_Diuretic
ARB
CCB
Beta_Blocker
ACE
0.004
0.005
0.006
0.007
0.008
Cumulative Incidence Rate at 1 Year After Baseline

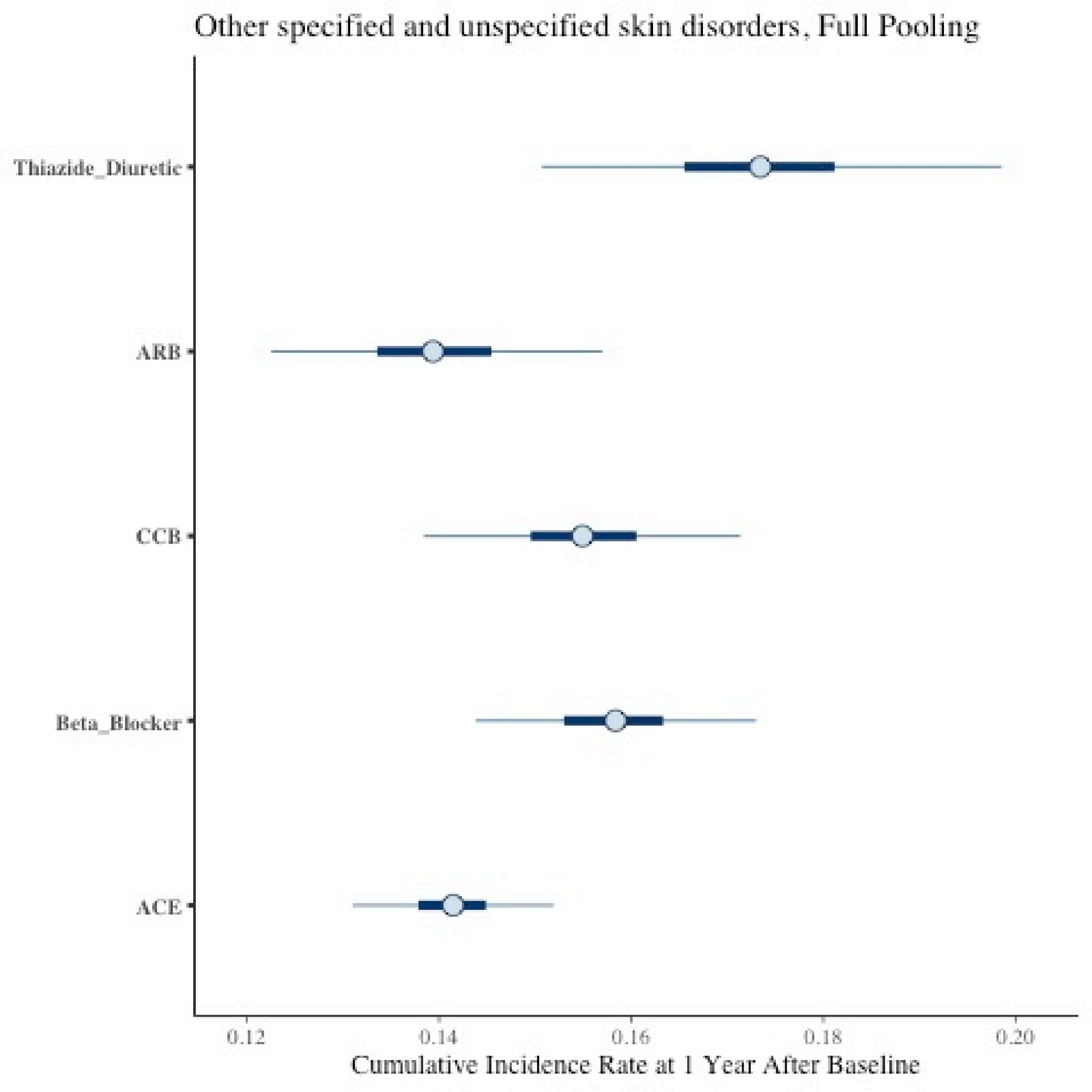

Other specified and unspecified skin disorders, Full Pooling
Thiazide_Diuretic
ARB
CCB
Beta_Blocker
ACE
0.12
0.14
0.16
0.18
0.20
Cumulative Incidence Rate at 1 Year After Baseline

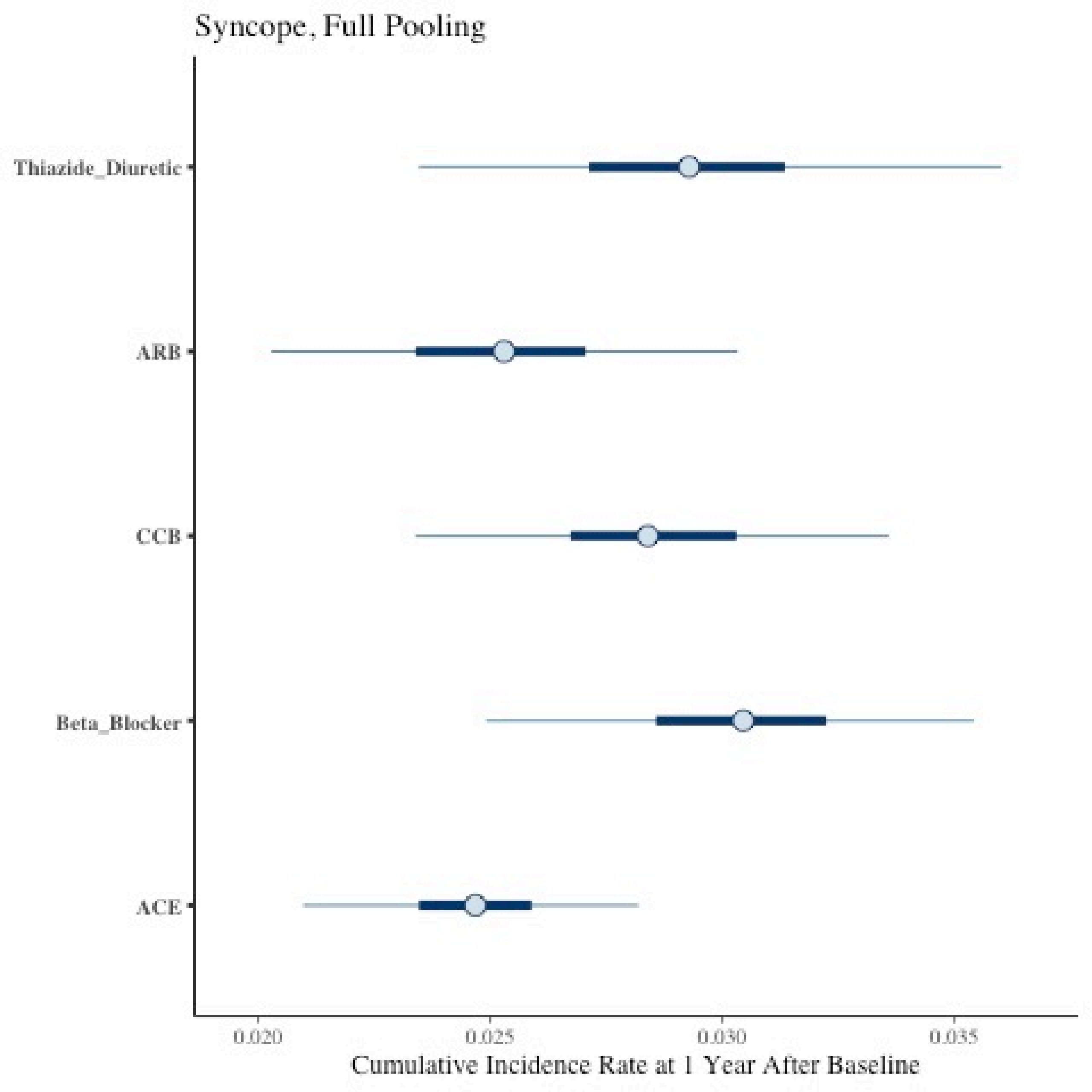

Syncope, Full Pooling
Thiazide_Diuretic
ARB
CCB
Beta_Blocker
ACE
0.020
0.025
0.030
0.035
Cumulative Incidence Rate at 1 Year After Baseline

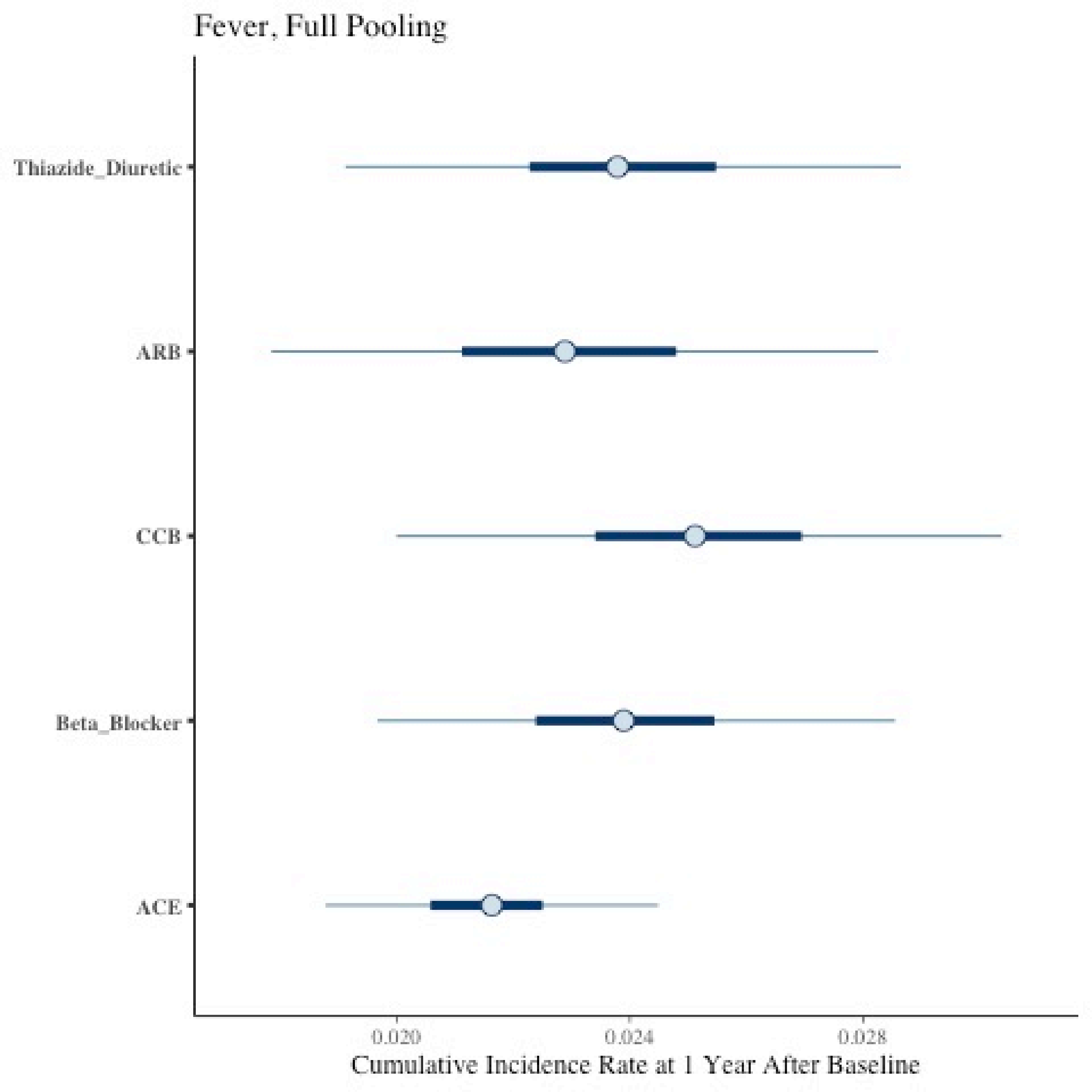

Fever, Full Pooling
Thiazide_Diuretic
ARB
CCB
Beta_Blocker
ACE
0.020
0.024
0.028
Cumulative Incidence Rate at 1 Year After Baseline

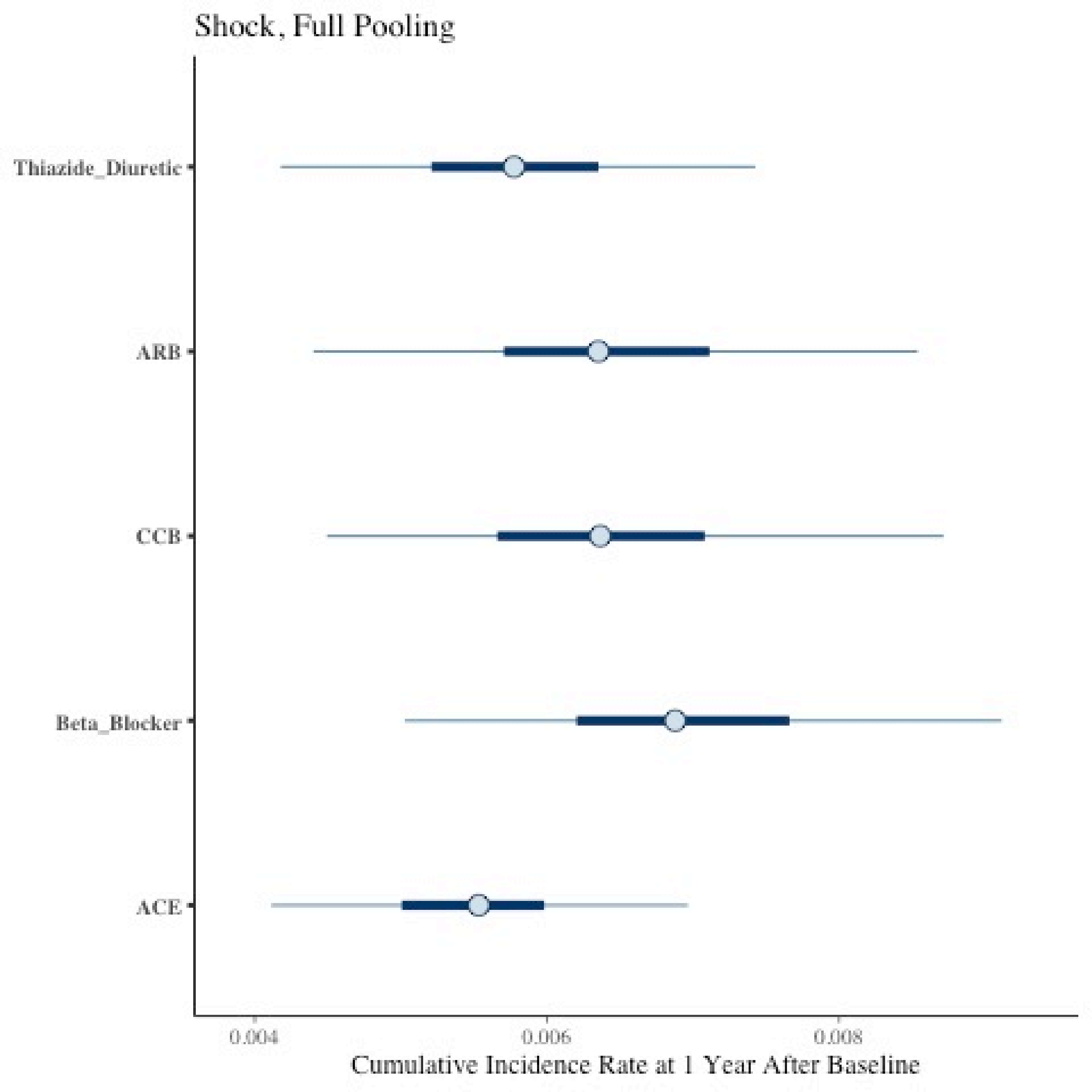

Shock, Full Pooling
Thiazide_Diuretic
ARB
CCB
Beta_Blocker
ACE
0.004
0.006
0.008
Cumulative Incidence Rate at 1 Year After Baseline

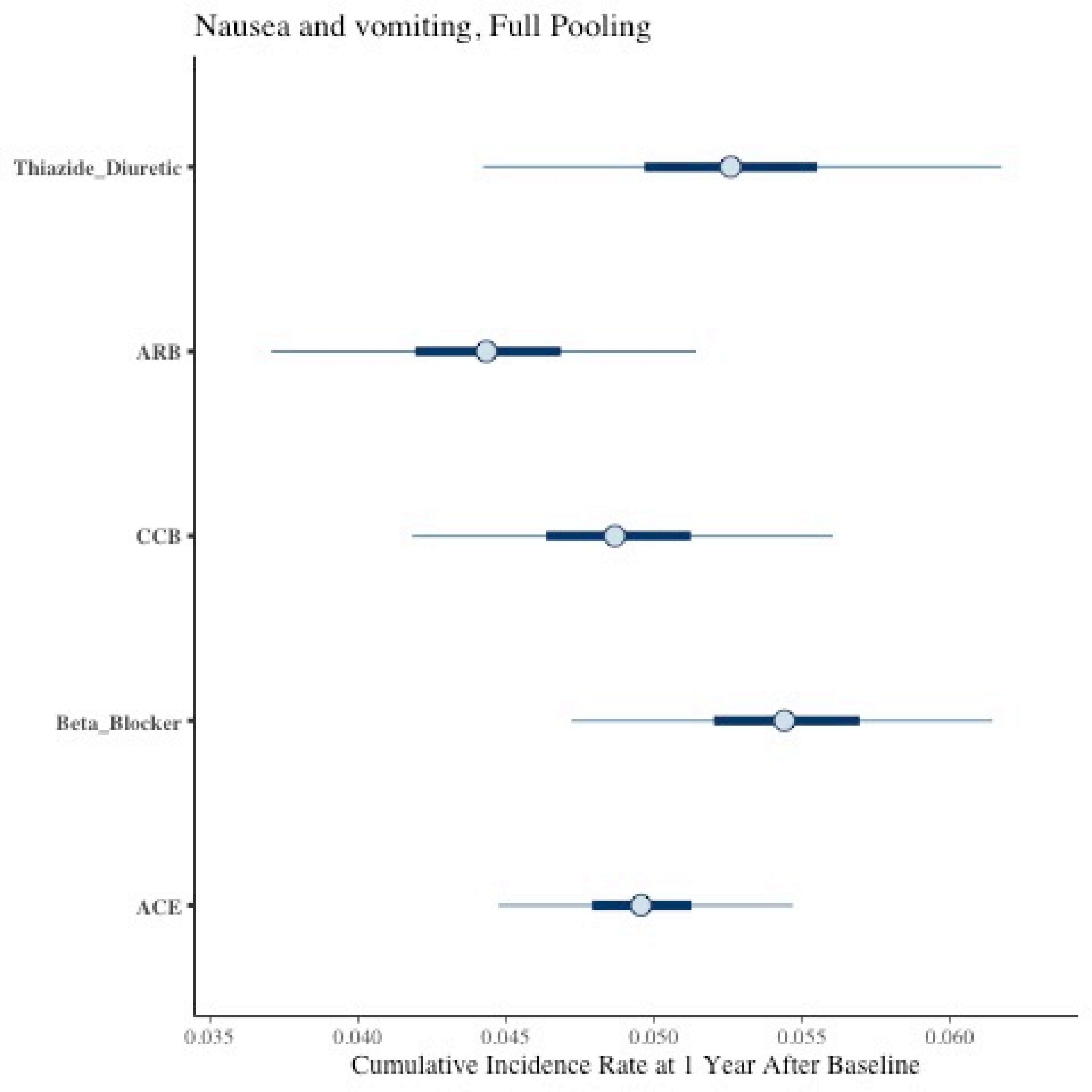

Nausea and vomiting, Full Pooling
Thiazide_Diuretic
ARB
CCB
Beta_Blocker
ACE
0.035
0.040
0.045
0.050
0.055
0.060
Cumulative Incidence Rate at 1 Year After Baseline

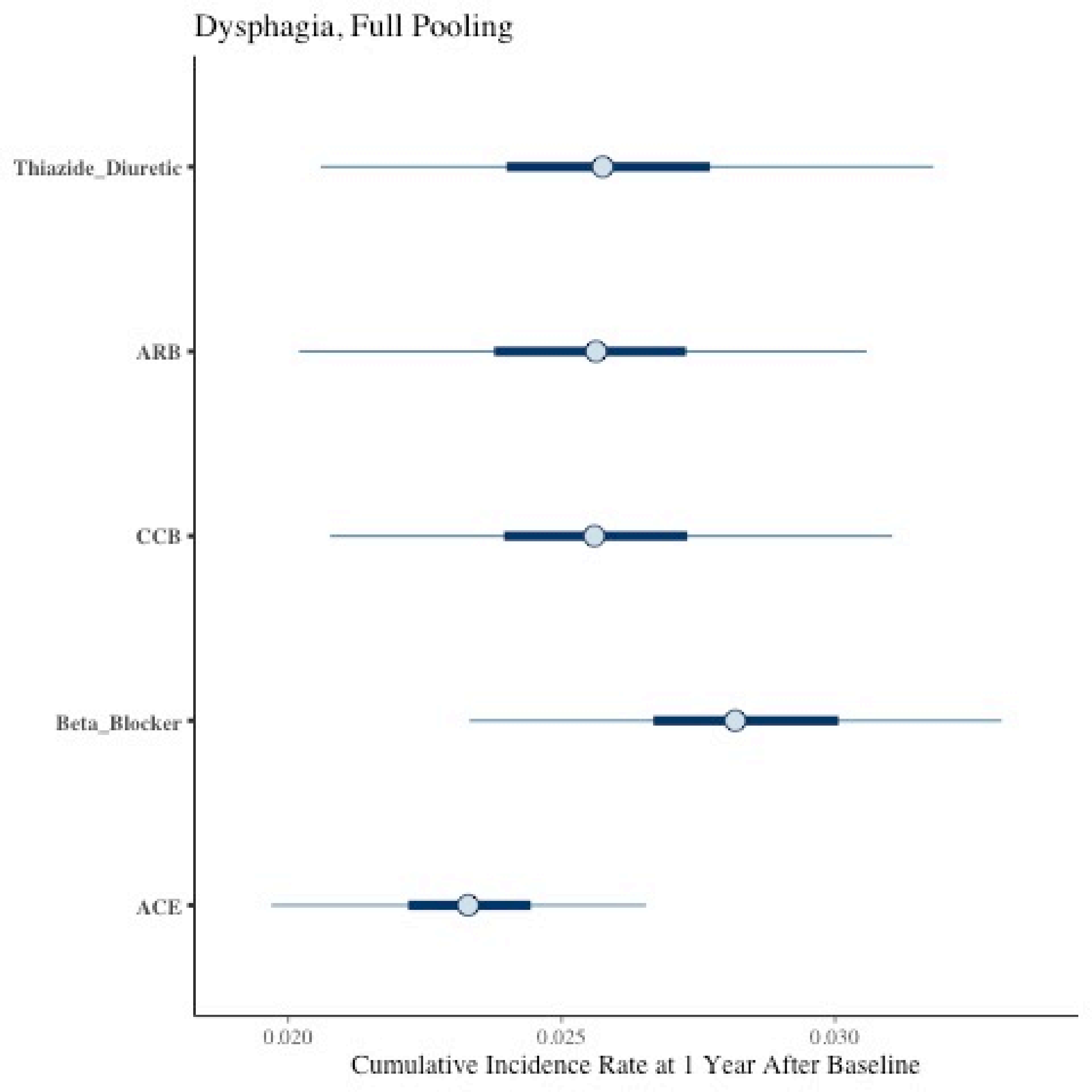

Dysphagia, Full Pooling
Thiazide_Diuretic
ARB
CCB
Beta_Blocker
ACE
0.020
0.025
0.030
Cumulative Incidence Rate at 1 Year After Baseline

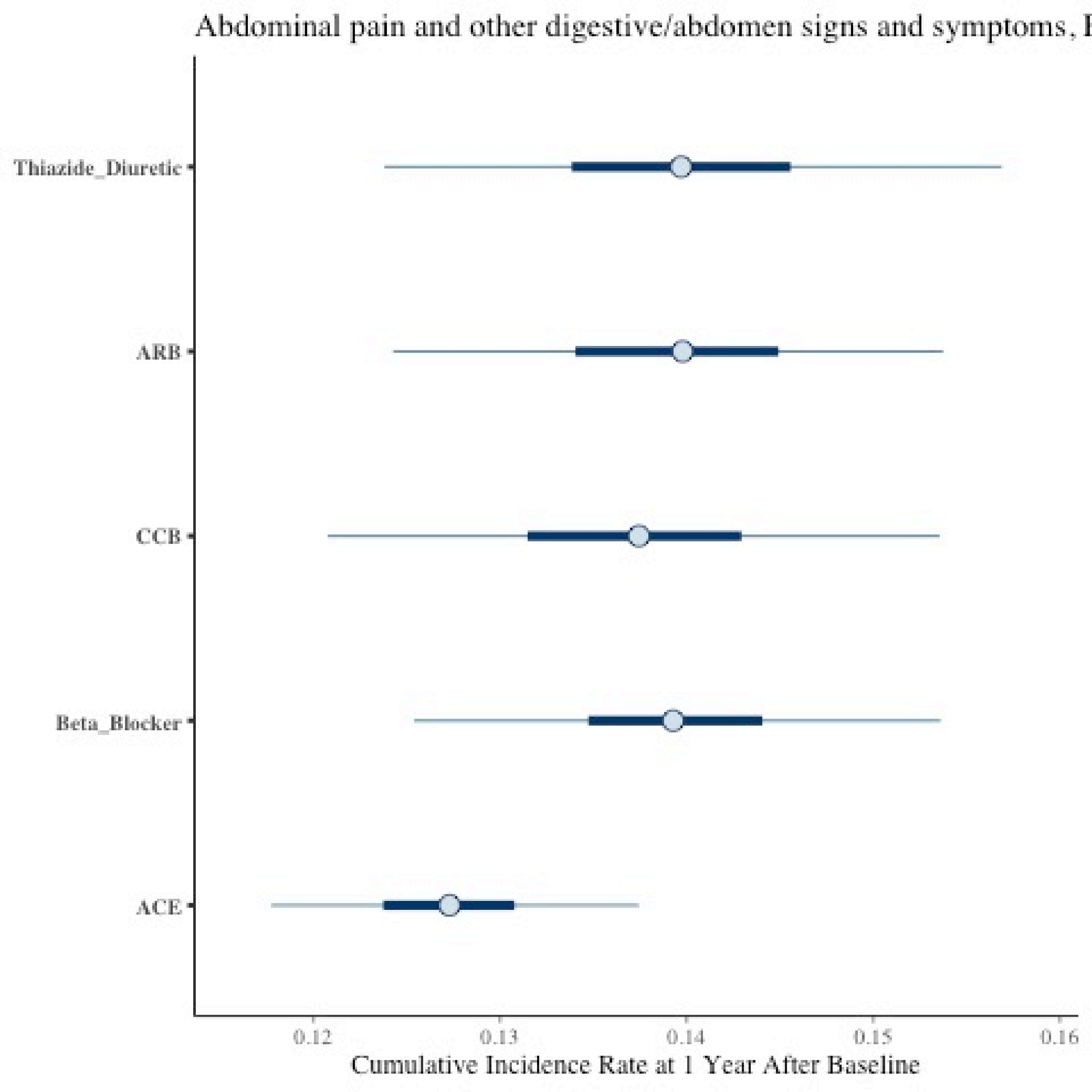

Abdominal pain and other digestive/abdomen signs and symptoms, F
Thiazide_Diuretic
ARB
CCB
Beta_Blocker
ACE
0.12
0.13
0.14
0.15
0.16
Cumulative Incidence Rate at 1 Year After Baseline

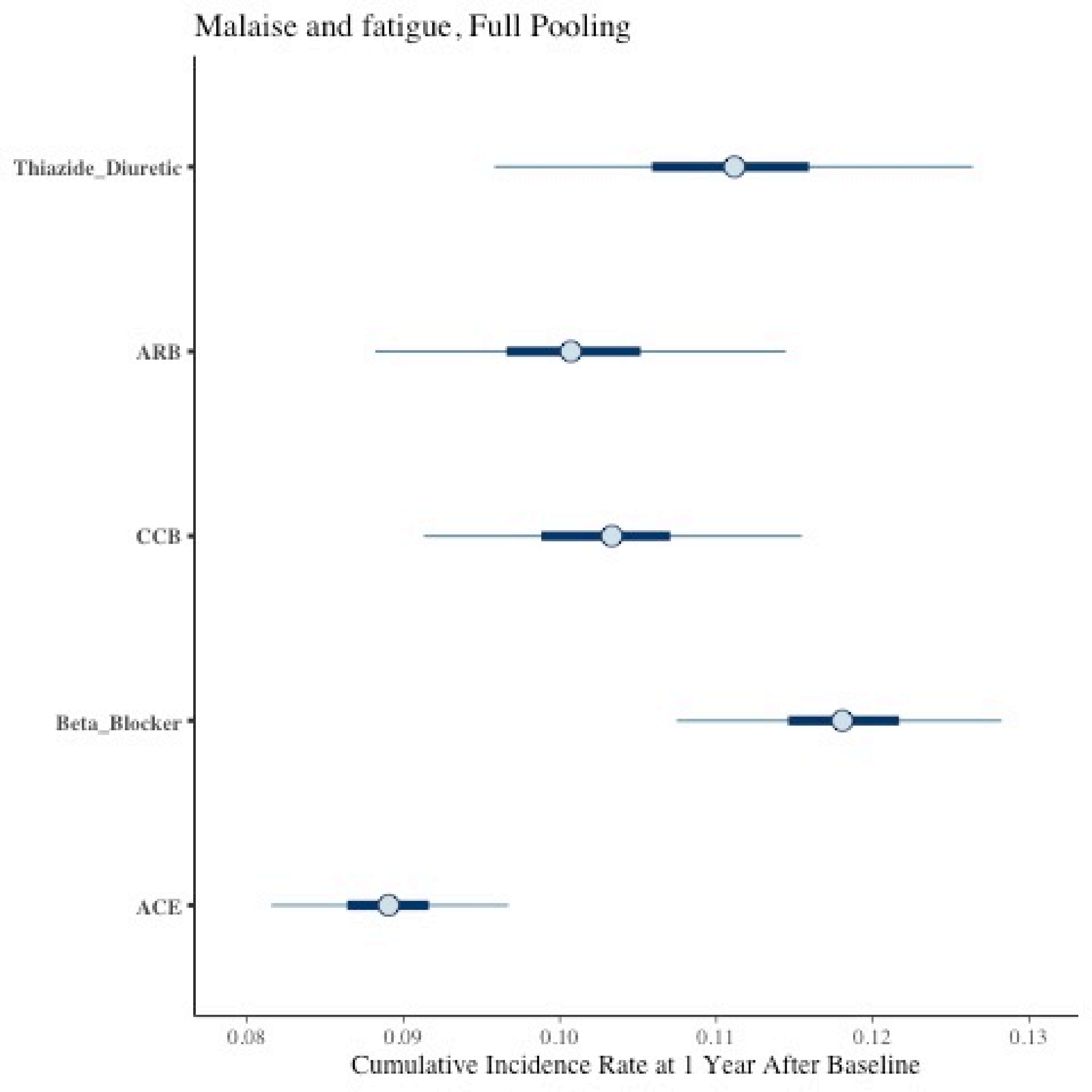

Malaise and fatigue, Full Pooling
Thiazide_Diuretic
ARB
CCB
Beta_Blocker
ACE
0.08
0.09
0.10
0.11
0.12
0.13
Cumulative Incidence Rate at 1 Year After Baseline

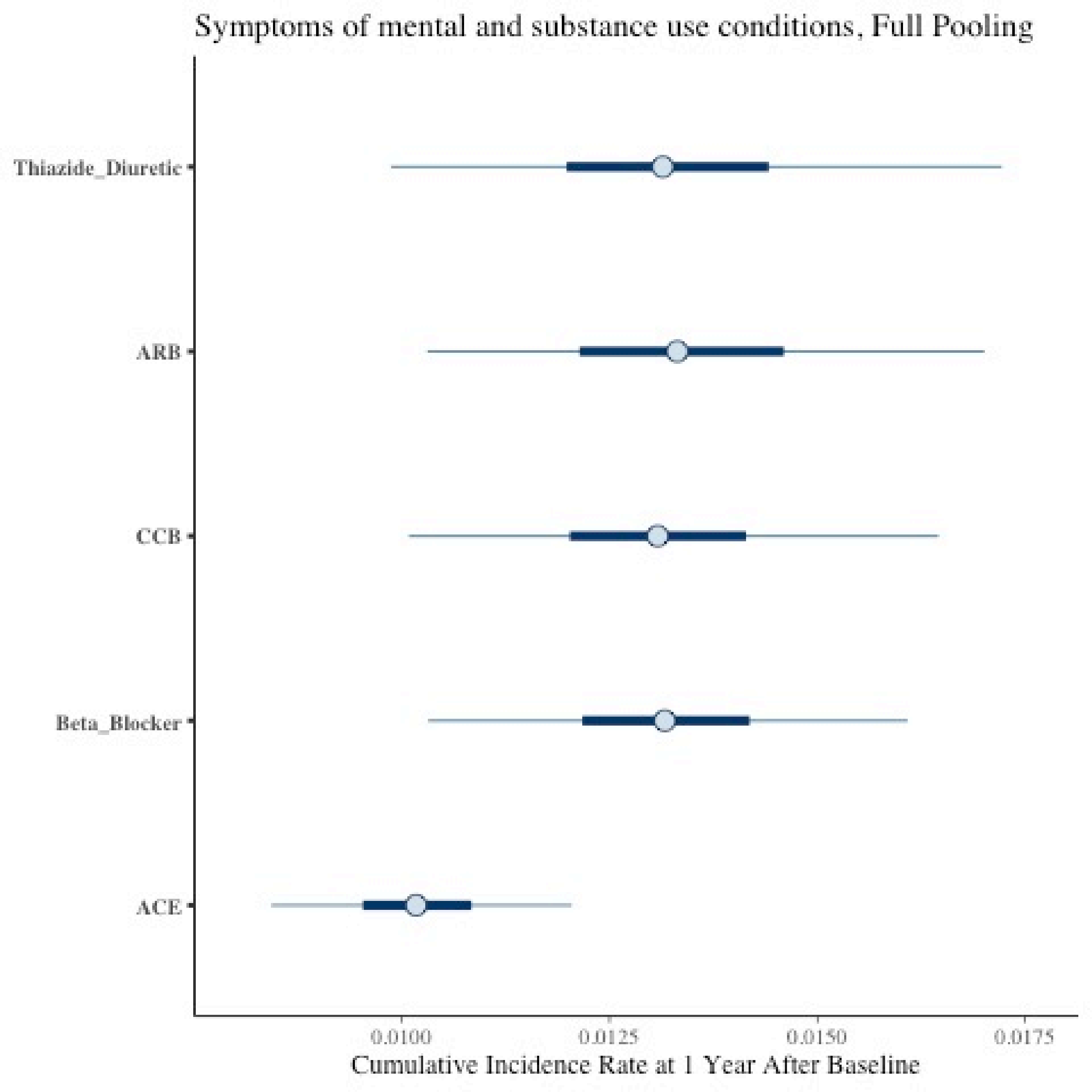

Symptoms of mental and substance use conditions, Full Pooling
Thiazide_Diuretic
ARB
CCB
Beta_Blocker
ACE
0.0100
0.0125
0.0150
0.0175
Cumulative Incidence Rate at 1 Year After Baseline

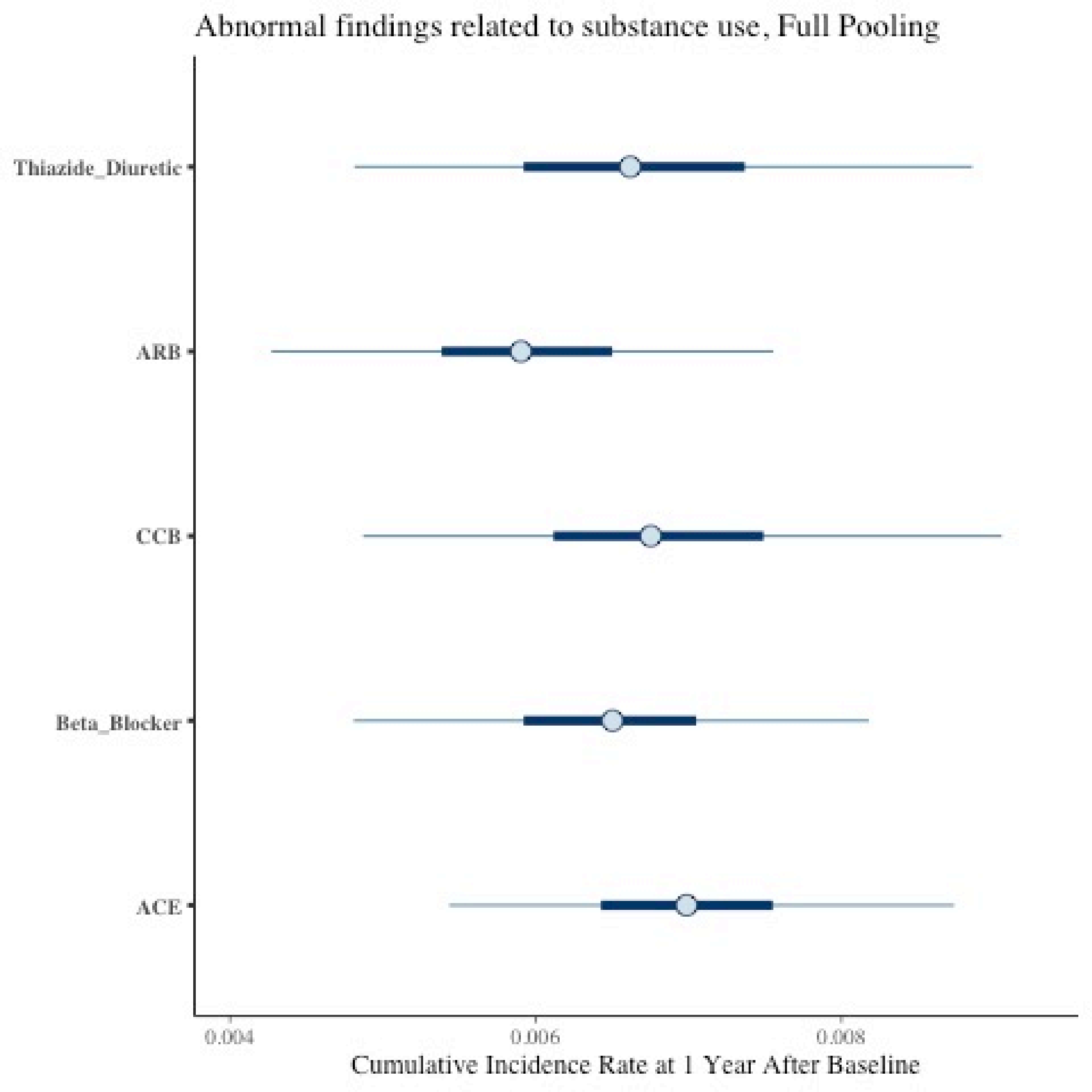
Abnormal findings related to substance use, Full Pooling
Thiazide_Diuretic
ARB
CCB
Beta_Blocker
ACE
0.004
0.006
0.008
Cumulative Incidence Rate at 1 Year After Baseline

APPENDIX 2

Summary Plots of Counterfactual Cumulative Incidence Rate Estimates Under the Single Outcome Pooling Model From Section 2.3

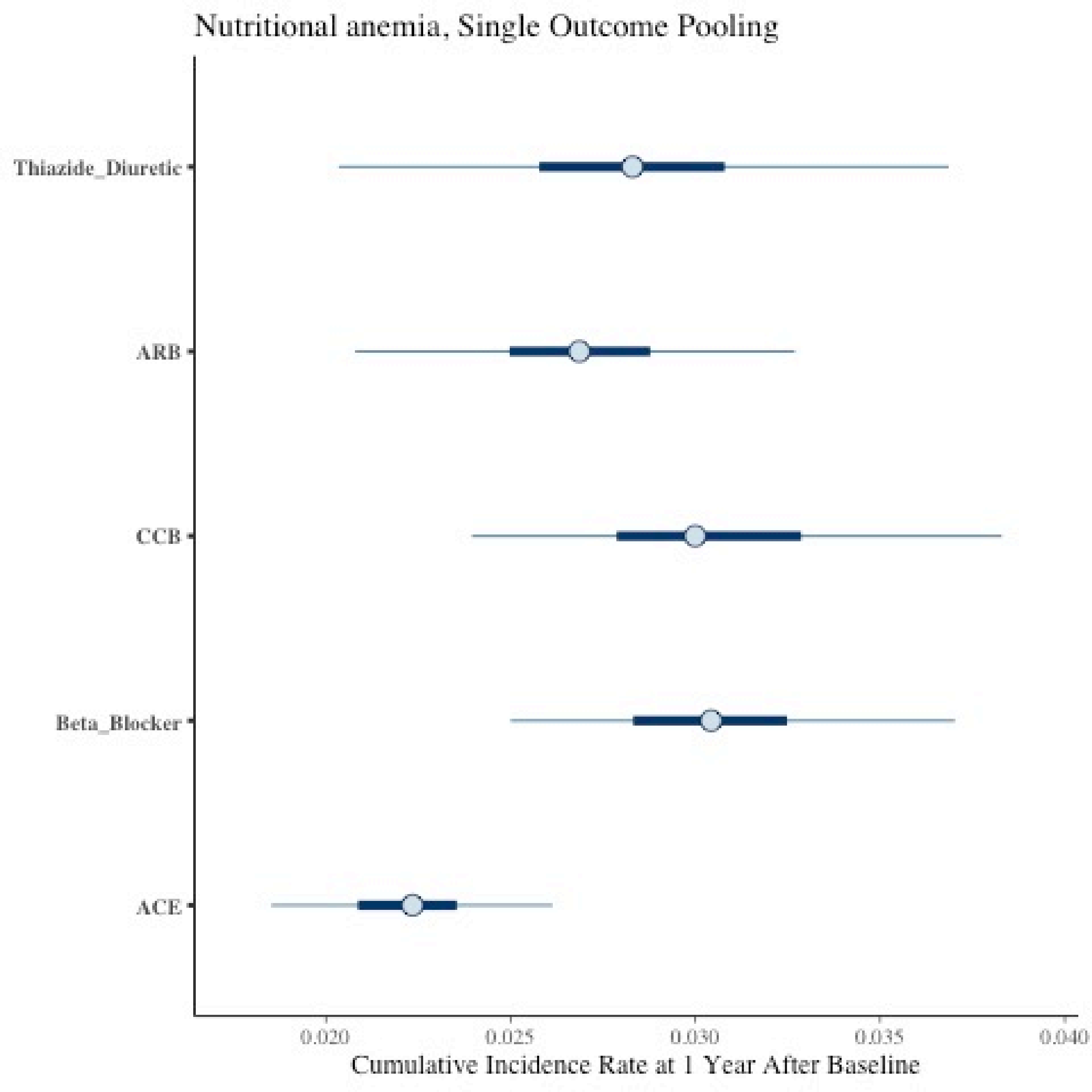

Nutritional anemia, Single Outcome Pooling
Thiazide_Diuretic
ARB
CCB
Beta_Blocker
ACE
0.020
0.025
0.030
0.035
0.040
Cumulative Incidence Rate at 1 Year After Baseline

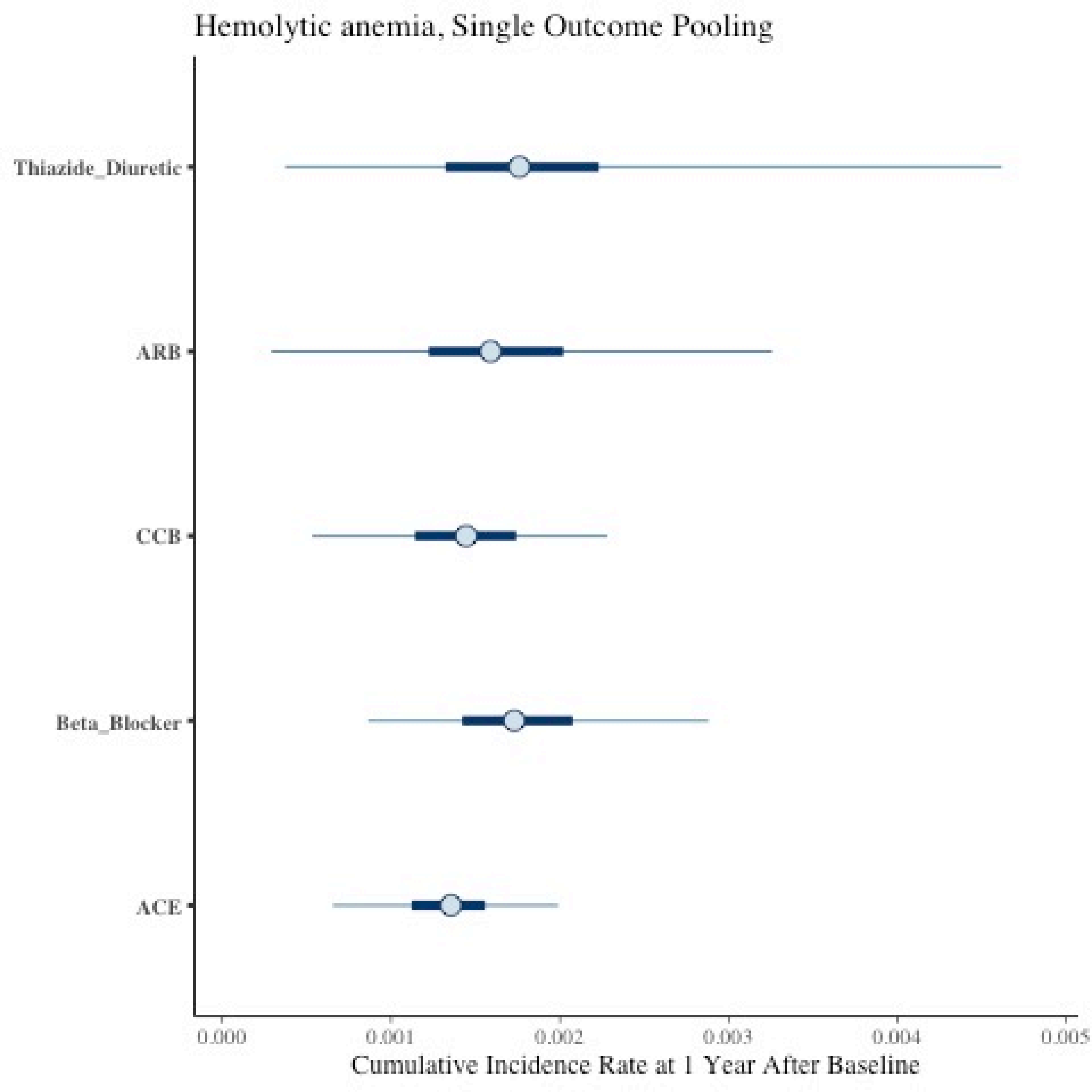

Hemolytic anemia, Single Outcome Pooling
Thiazide_Diuretic
ARB
CCB
Beta_Blocker
ACE
0.000
0.001
0.002
0.003
0.004
0.005
Cumulative Incidence Rate at 1 Year After Baseline

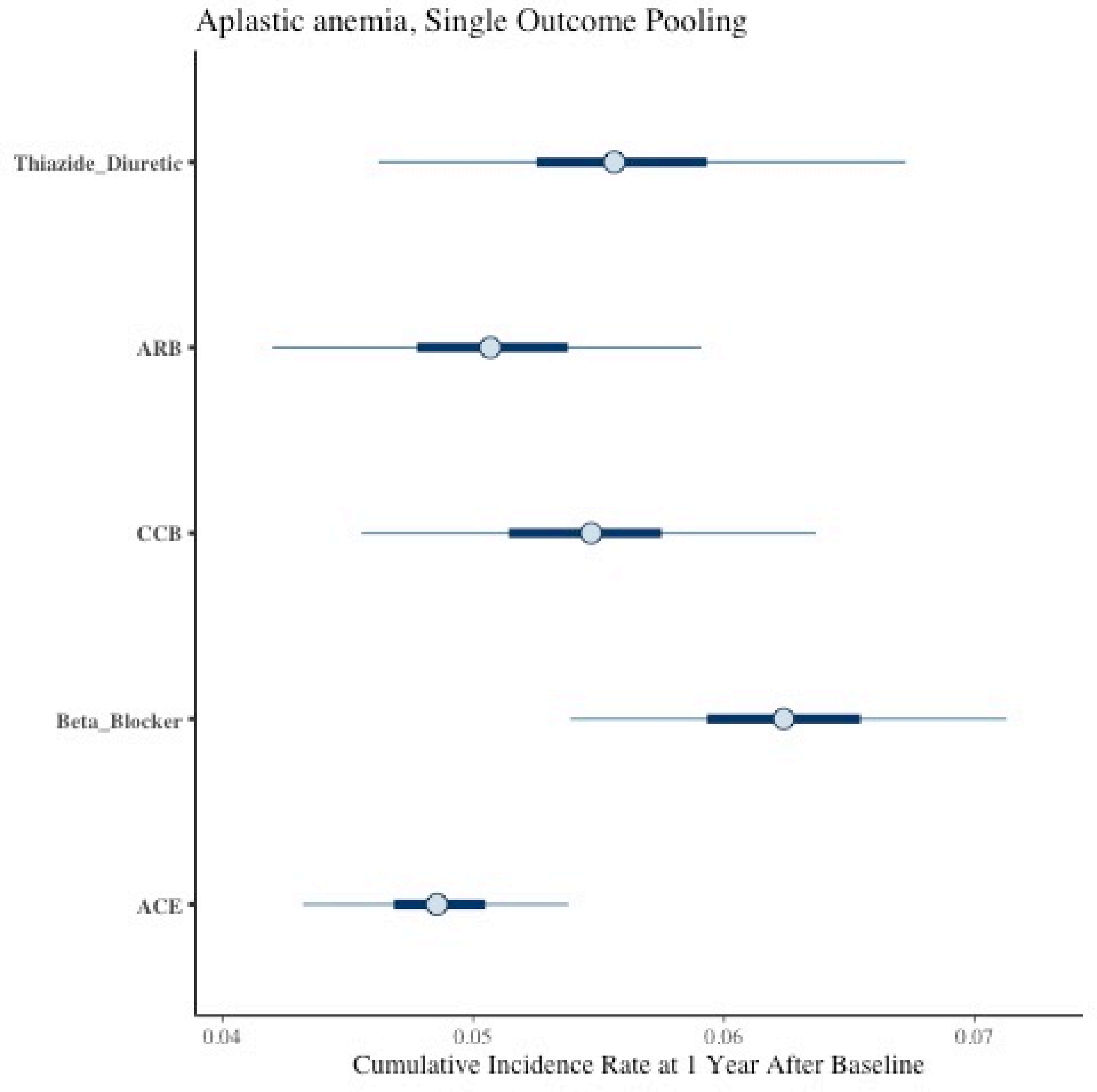
Aplastic anemia, Single Outcome Pooling
Thiazide_Diuretic
ARB
CCB
Beta_Blocker
ACE
0.04
0.05
0.06
0.07
Cumulative Incidence Rate at 1 Year After Baseline

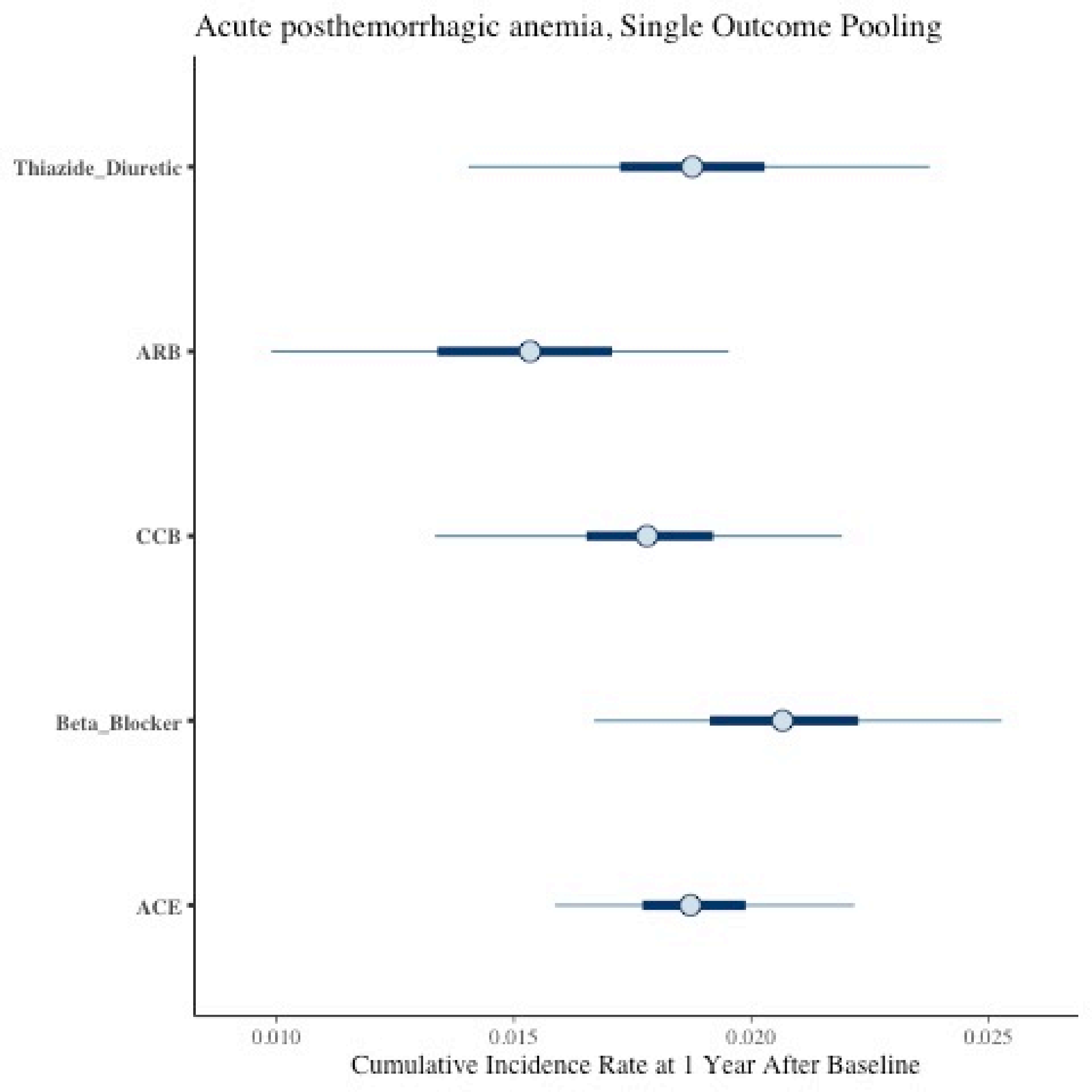

Acute posthemorrhagic anemia, Single Outcome Pooling
Thiazide_Diuretic
ARB
CCB
Beta_Blocker
ACE
0.010
0.015
0.020
0.025
Cumulative Incidence Rate at 1 Year After Baseline

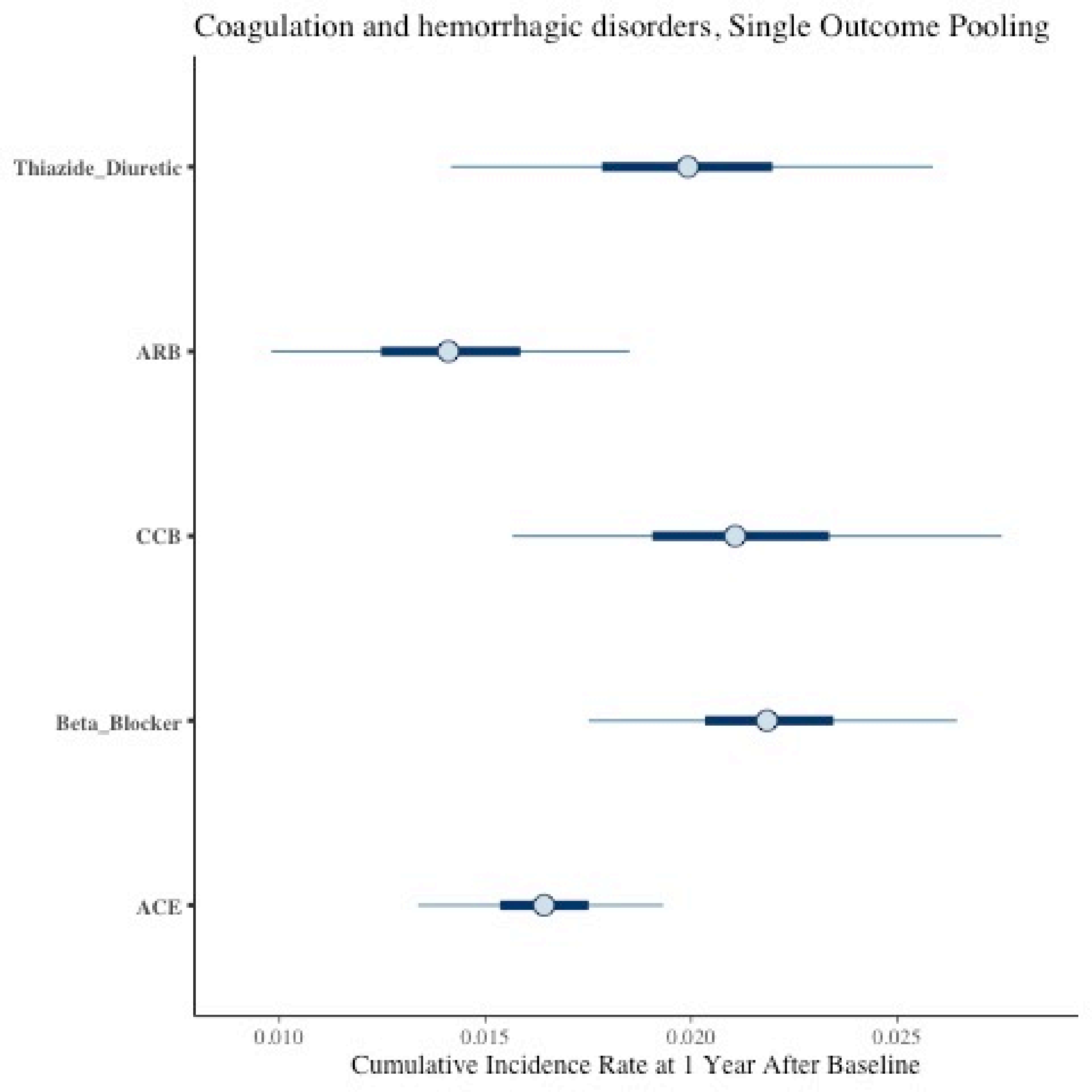

Coagulation and hemorrhagic disorders, Single Outcome Pooling
Thiazide_Diuretic
ARB
CCB
Beta_Blocker
ACE
0.010
0.015
0.020
0.025
Cumulative Incidence Rate at 1 Year After Baseline

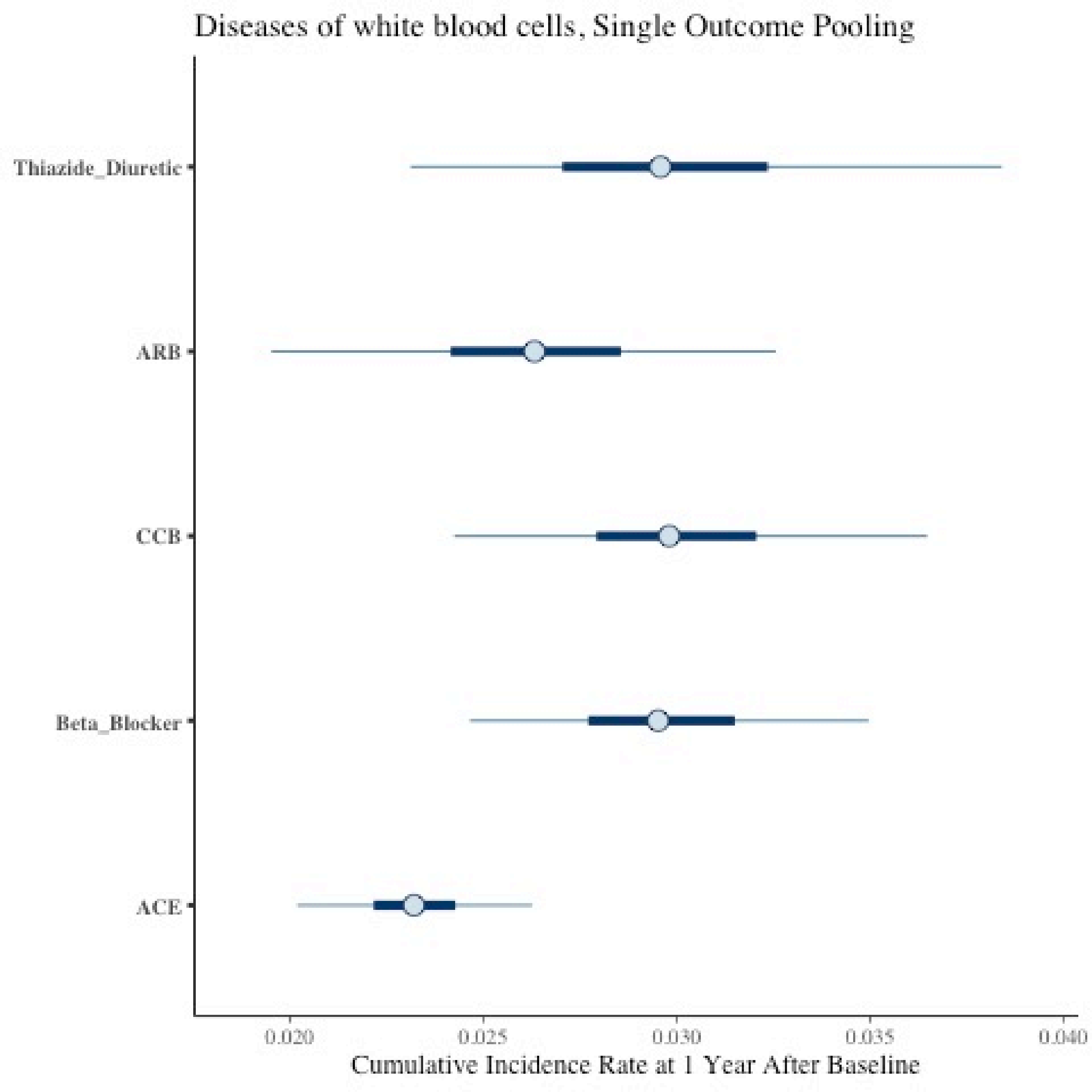

Diseases of white blood cells, Single Outcome Pooling
Thiazide_Diuretic
ARB
CCB
Beta_Blocker
ACE
0.020
0.025
0.030
0.035
0.040
Cumulative Incidence Rate at 1 Year After Baseline

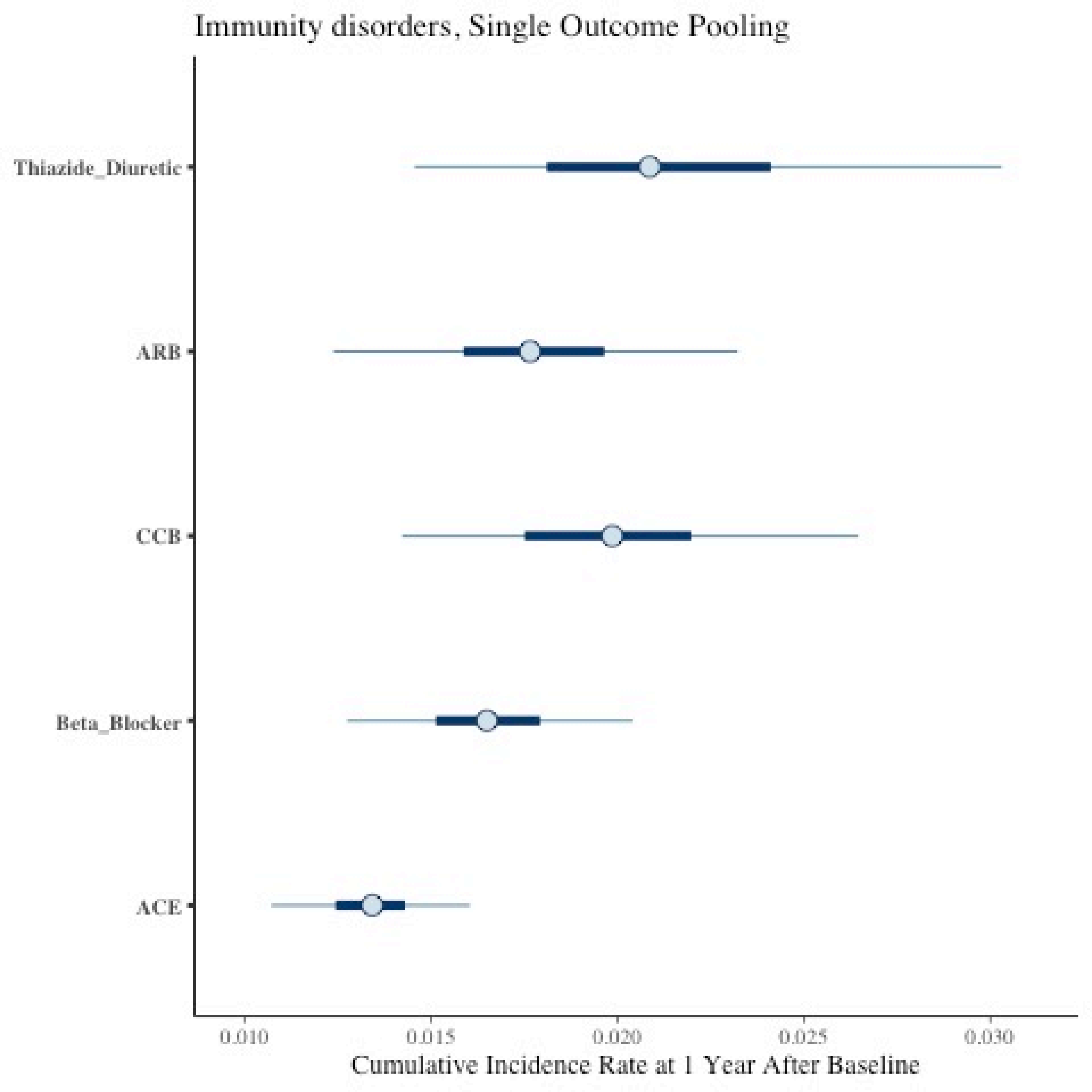

Immunity disorders, Single Outcome Pooling
Thiazide_Diuretic
ARB
CCB
Beta_Blocker
ACE
0.010
0.015
0.020
0.025
0.030
Cumulative Incidence Rate at 1 Year After Baseline

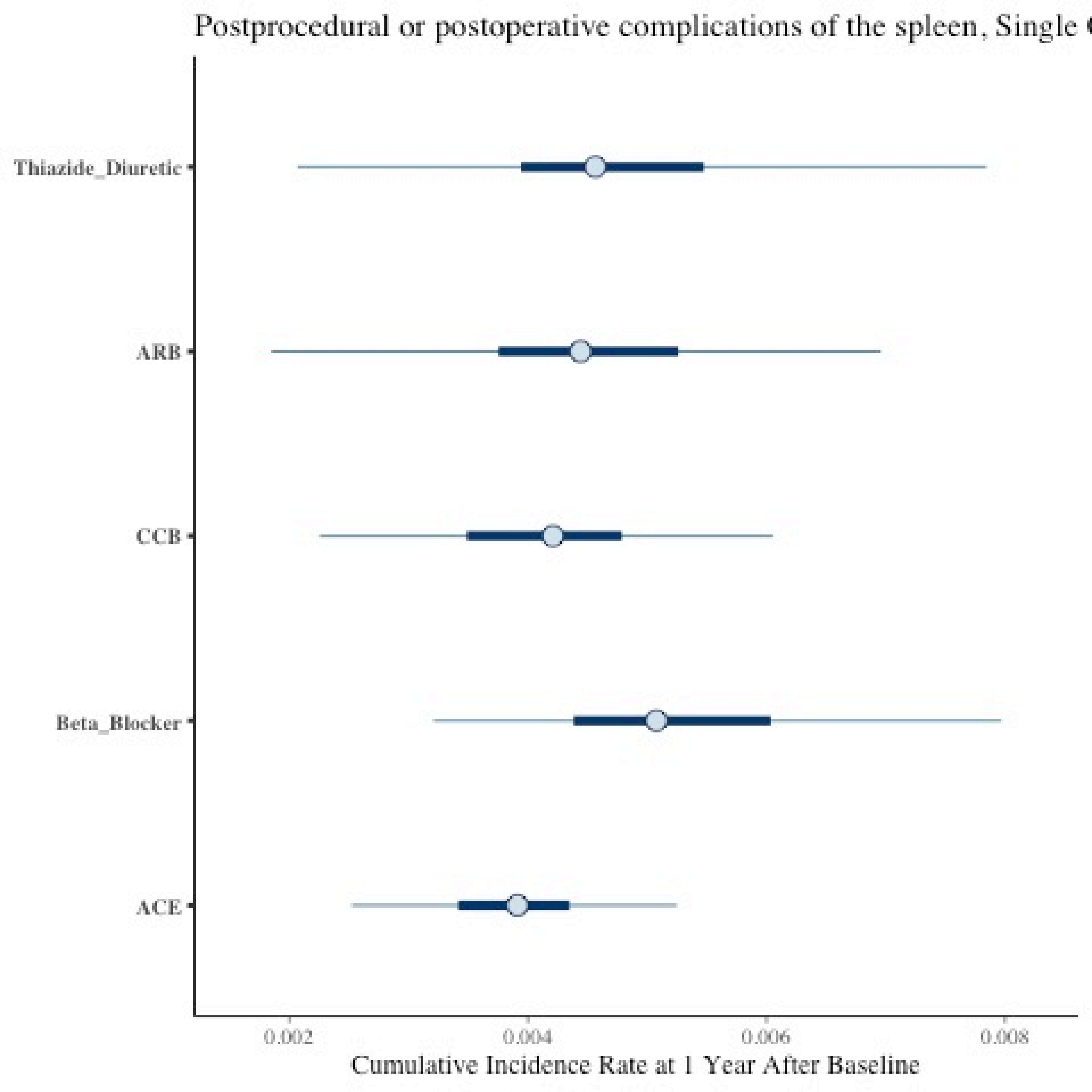

Postprocedural or postoperative complications of the spleen, Single
Thiazide_Diuretic
ARB
CCB
Beta_Blocker
ACE
0.002
0.004
0.006
0.008
Cumulative Incidence Rate at 1 Year After Baseline

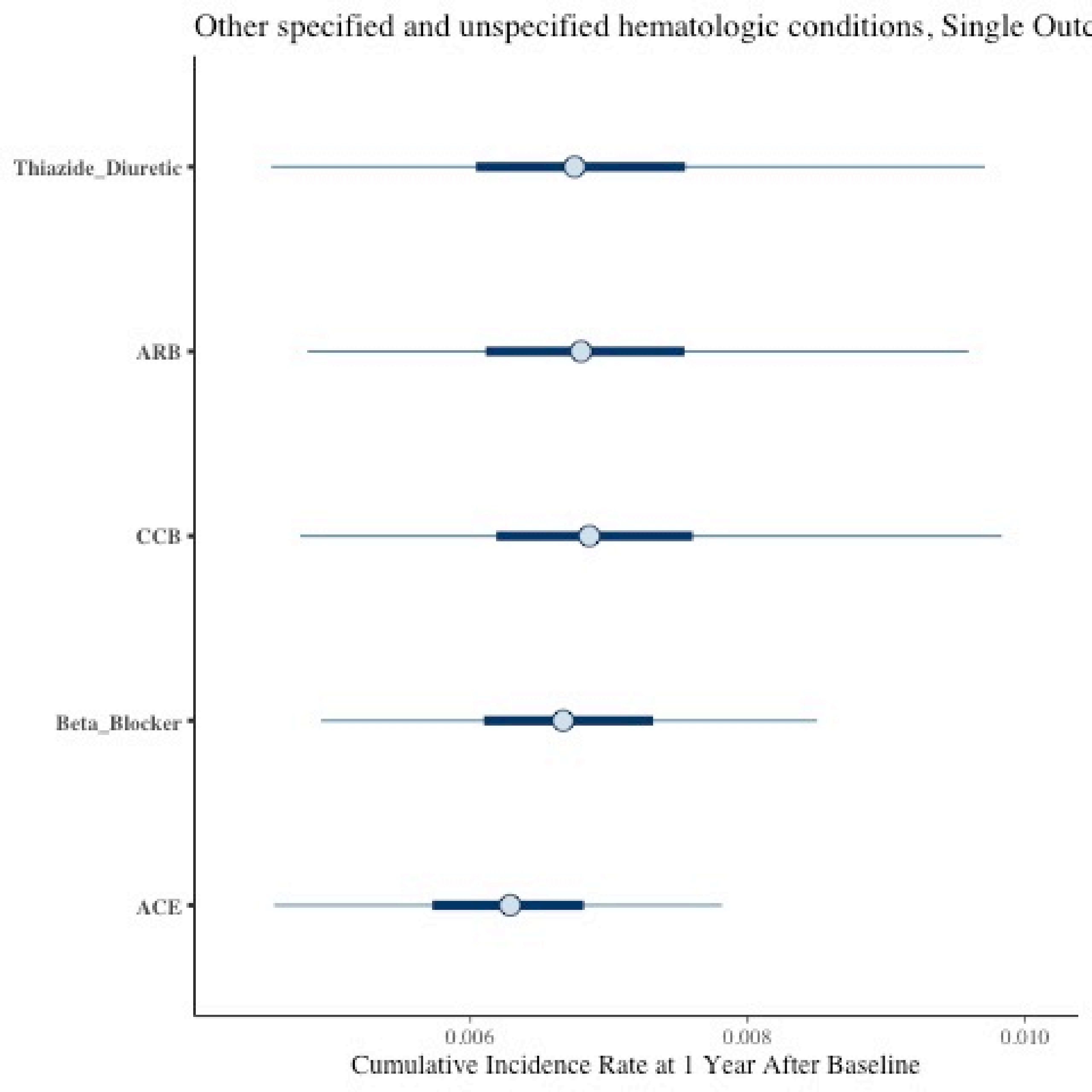

Other specified and unspecified hematologic conditions, Single Outc
Thiazide_Diuretic
ARB
CCB
Beta_Blocker
ACE
0.006
0.008
0.010
Cumulative Incidence Rate at 1 Year After Baseline

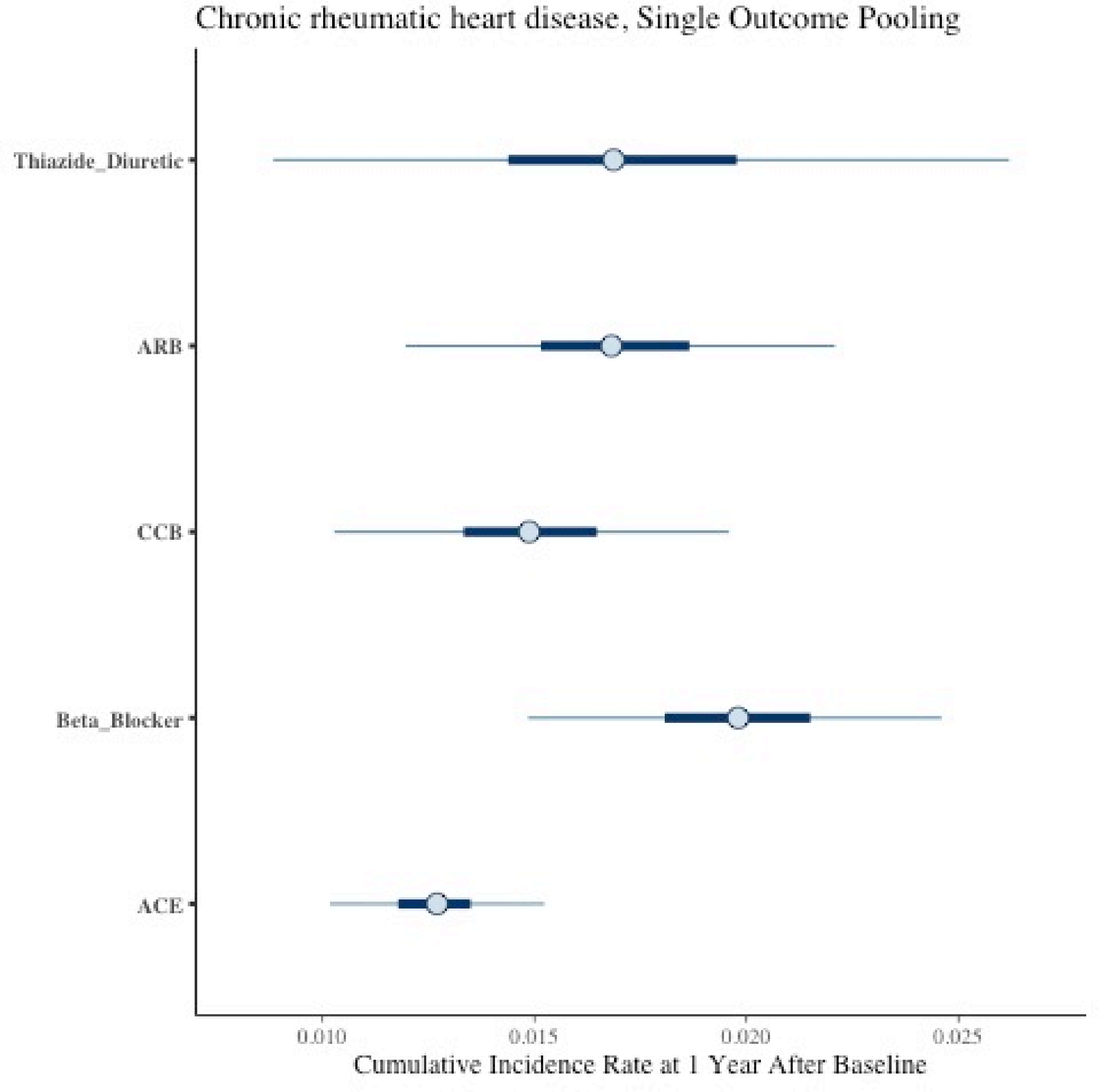

Chronic rheumatic heart disease, Single Outcome Pooling
Thiazide_Diuretic
ARB
CCB
Beta_Blocker
ACE
0.010
0.015
0.020
0.025
Cumulative Incidence Rate at 1 Year After Baseline

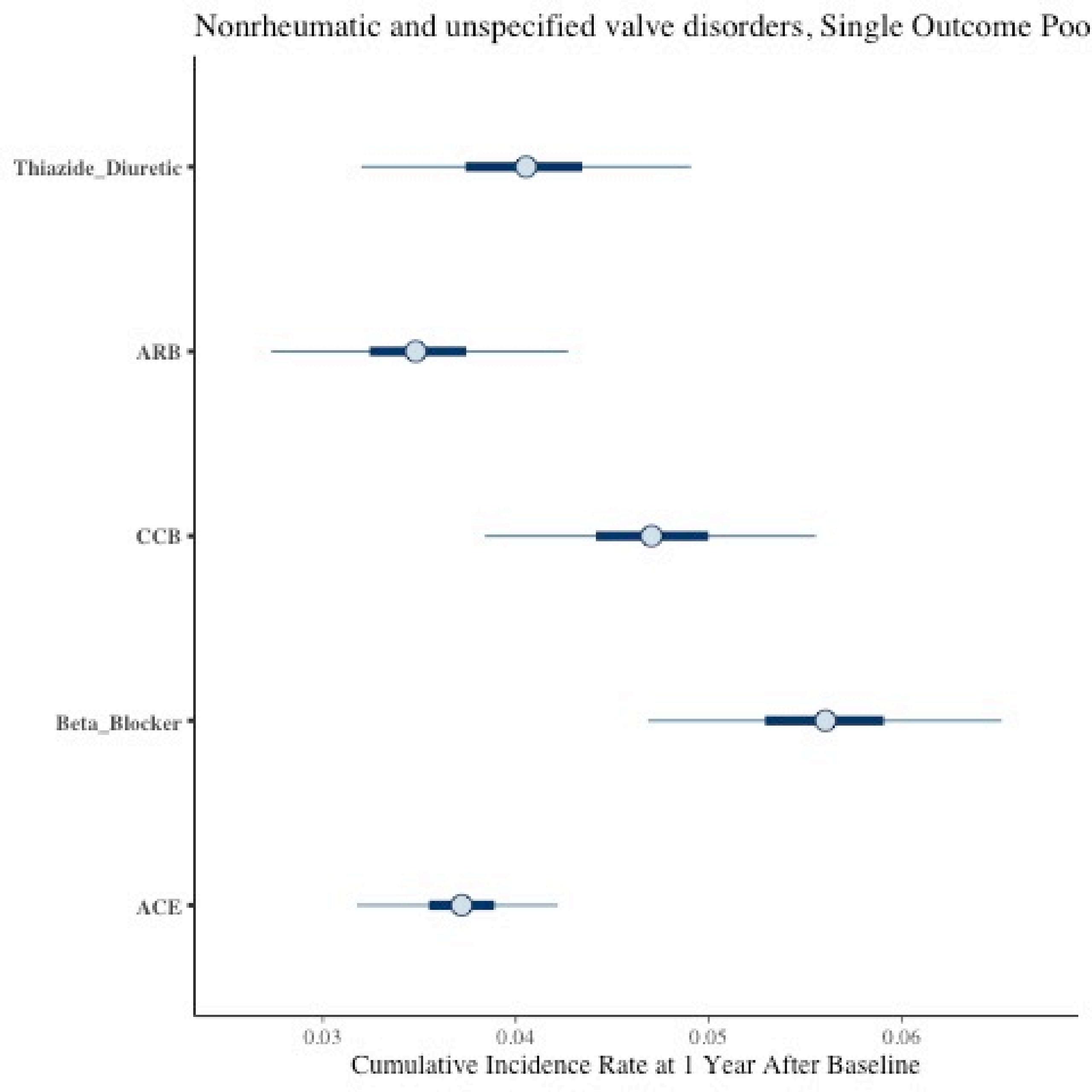

Nonrheumatic and unspecified valve disorders, Single Outcome Poo
Thiazide_Diuretic
ARB
CCB
Beta_Blocker
ACE
0.03
0.04
0.05
0.06
Cumulative Incidence Rate at 1 Year After Baseline

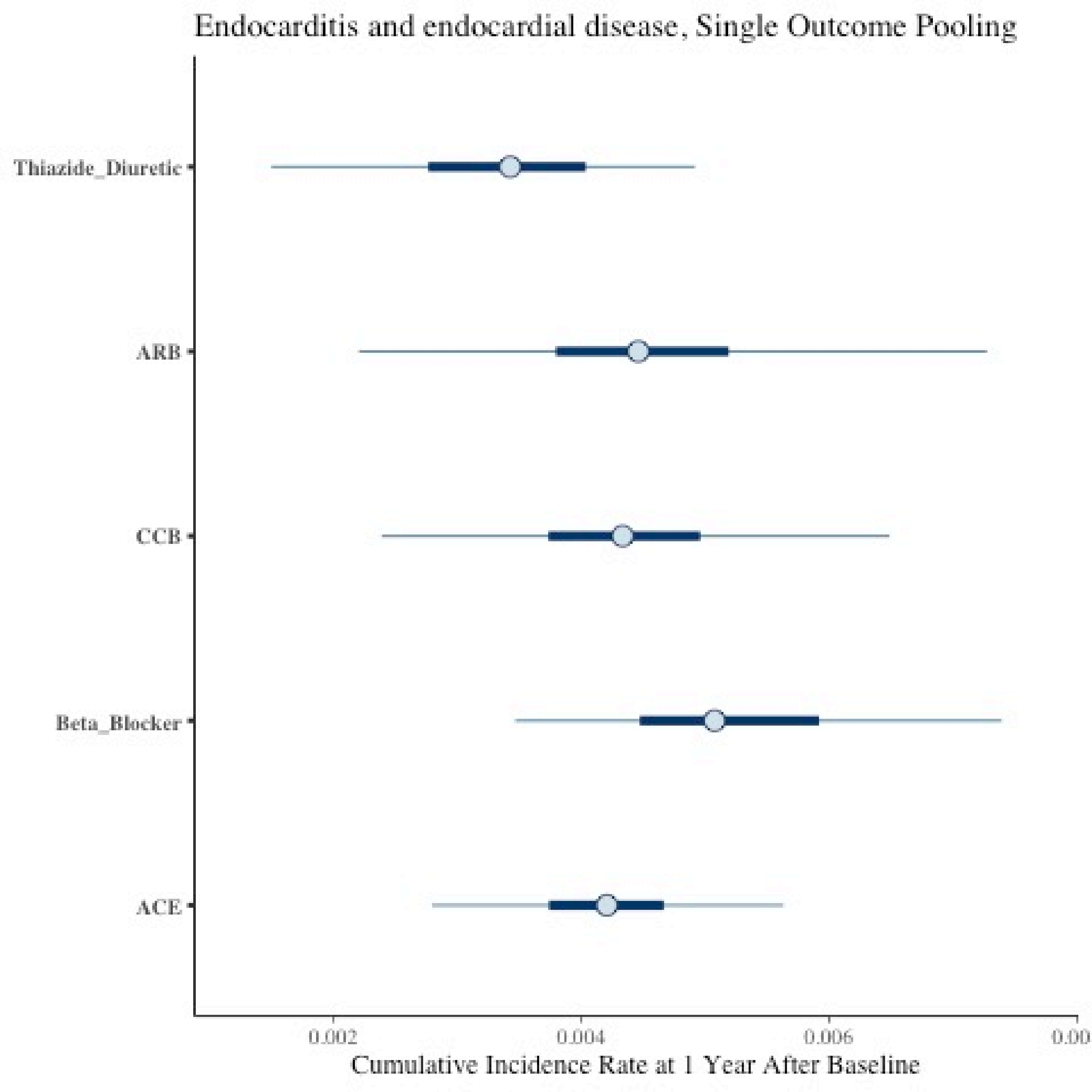

Endocarditis and endocardial disease, Single Outcome Pooling
Thiazide_Diuretic
ARB
CCB
Beta_Blocker
ACE
0.002
0.004
0.006
Cumulative Incidence Rate at 1 Year After Baseline

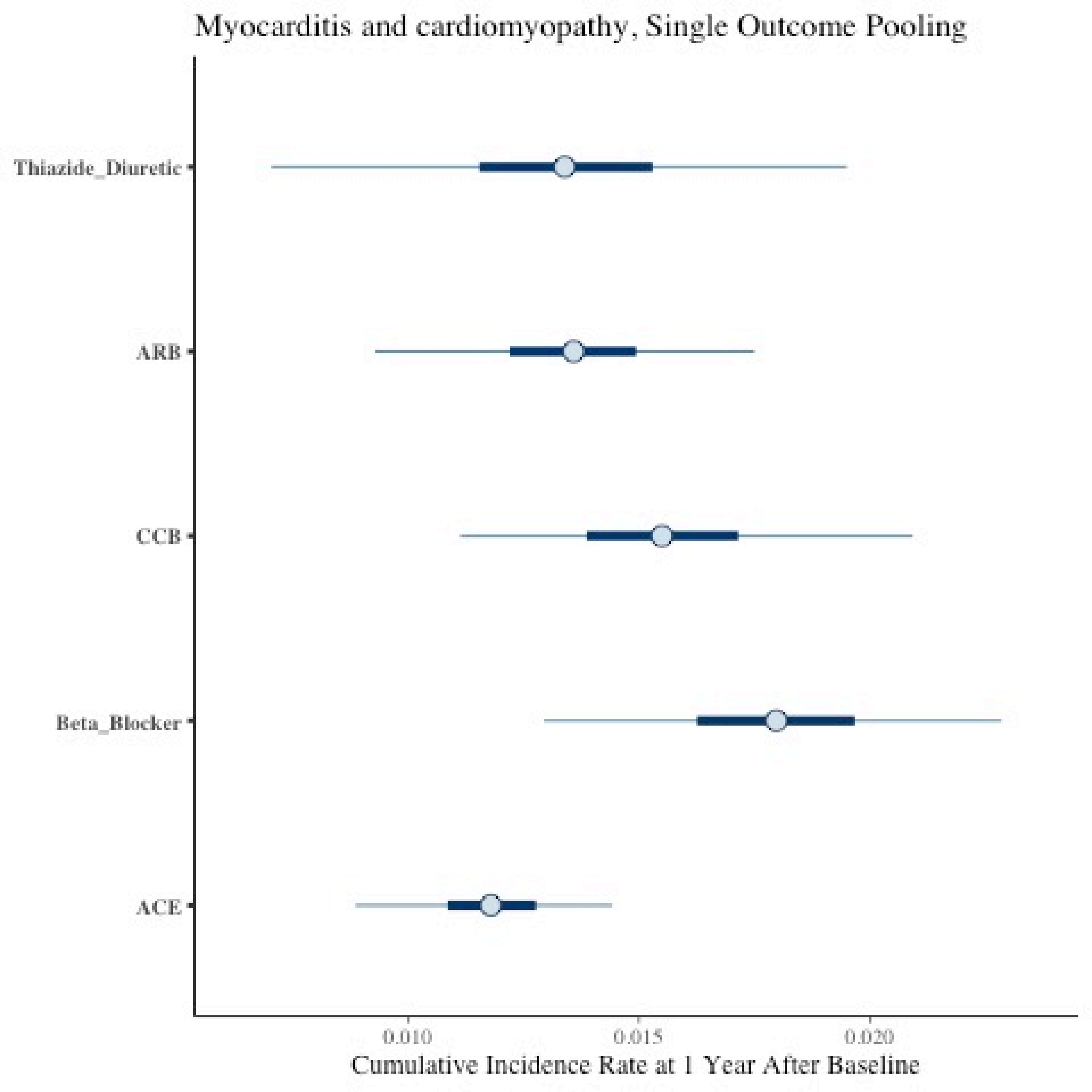

Myocarditis and cardiomyopathy, Single Outcome Pooling
Thiazide_Diuretic
ARB
CCB
Beta_Blocker
ACE
0.010
0.015
0.020
Cumulative Incidence Rate at 1 Year After Baseline

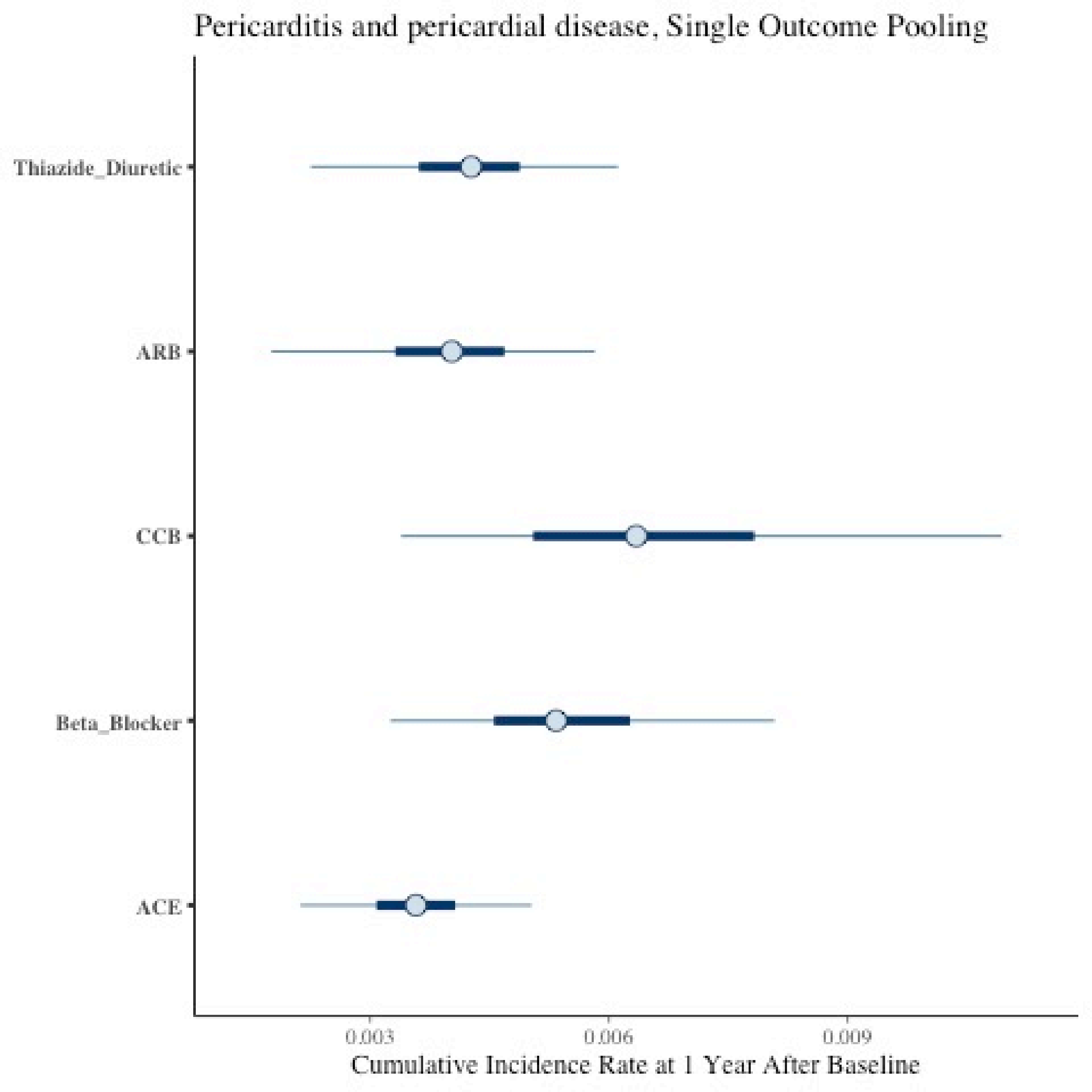

Pericarditis and pericardial disease, Single Outcome Pooling
Thiazide_Diuretic
ARB
CCB
Beta_Blocker
ACE
0.003
0.006
0.009
Cumulative Incidence Rate at 1 Year After Baseline

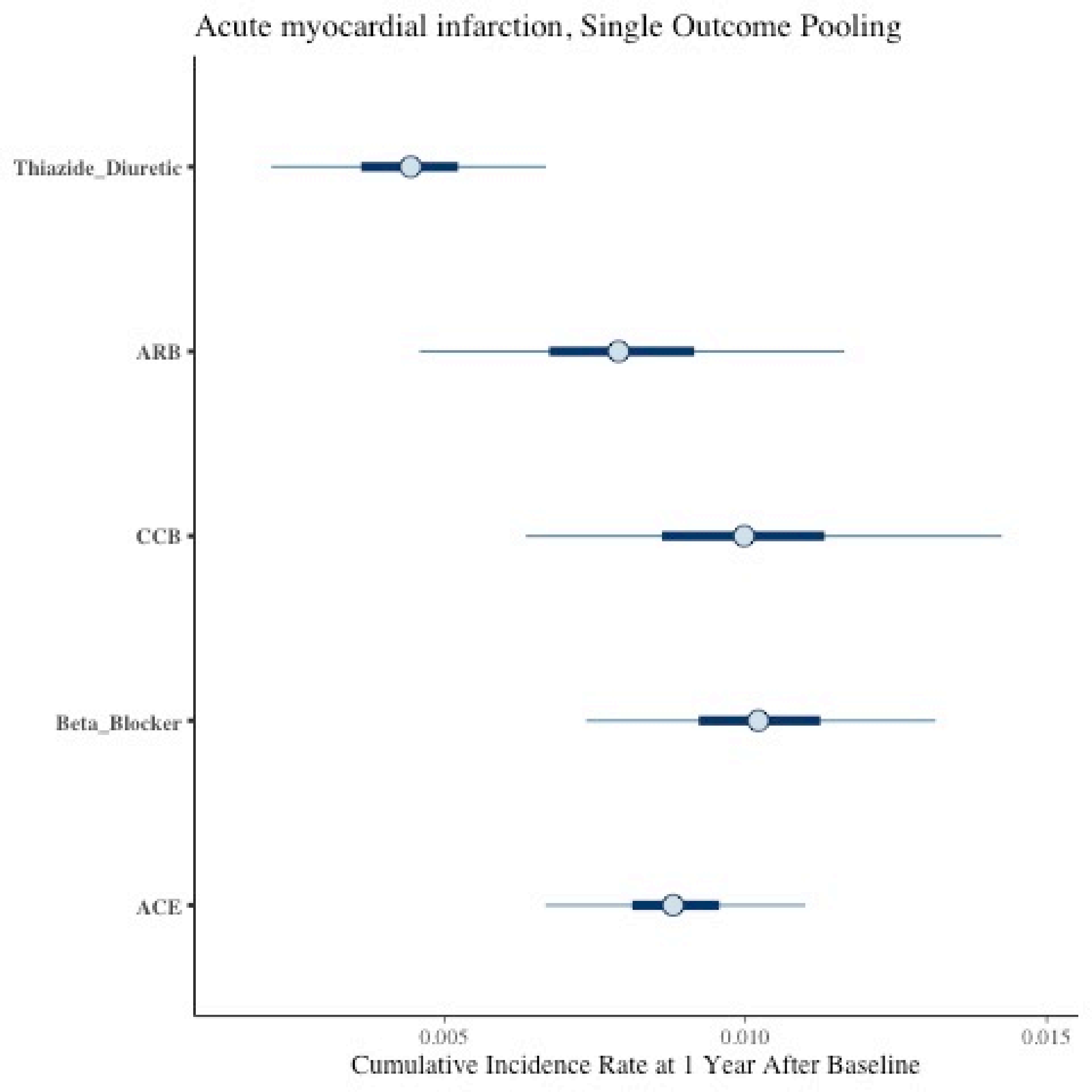

Acute myocardial infarction, Single Outcome Pooling
Thiazide_Diuretic
ARB
CCB
Beta_Blocker
ACE
0.005
0.010
0.015
Cumulative Incidence Rate at 1 Year After Baseline

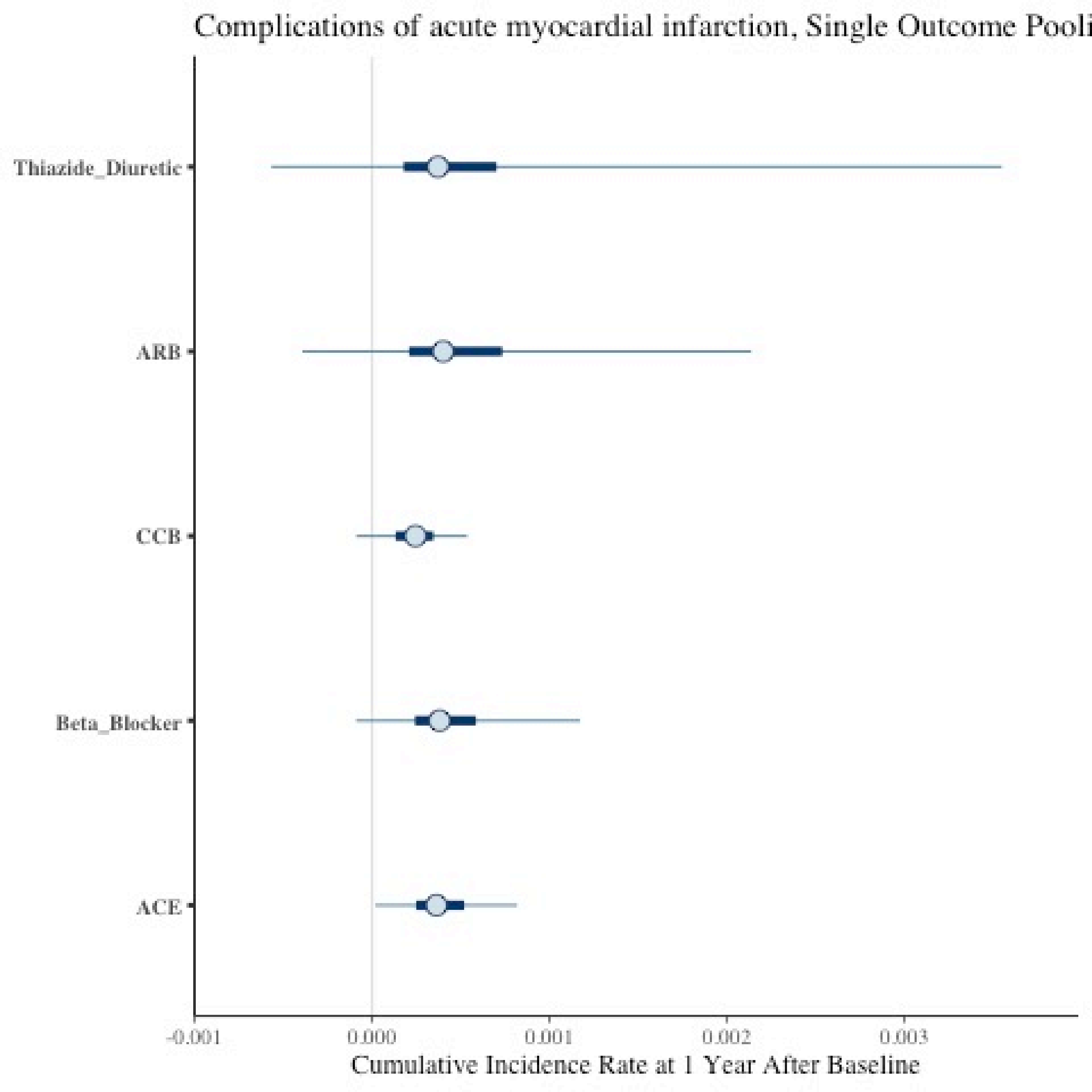

Complications of acute myocardial infarction, Single Outcome Pooli
Thiazide_Diuretic
ARB
CCB
Beta_Blocker
ACE
-0.001
0.000
0.001
0.002
0.003
Cumulative Incidence Rate at 1 Year After Baseline

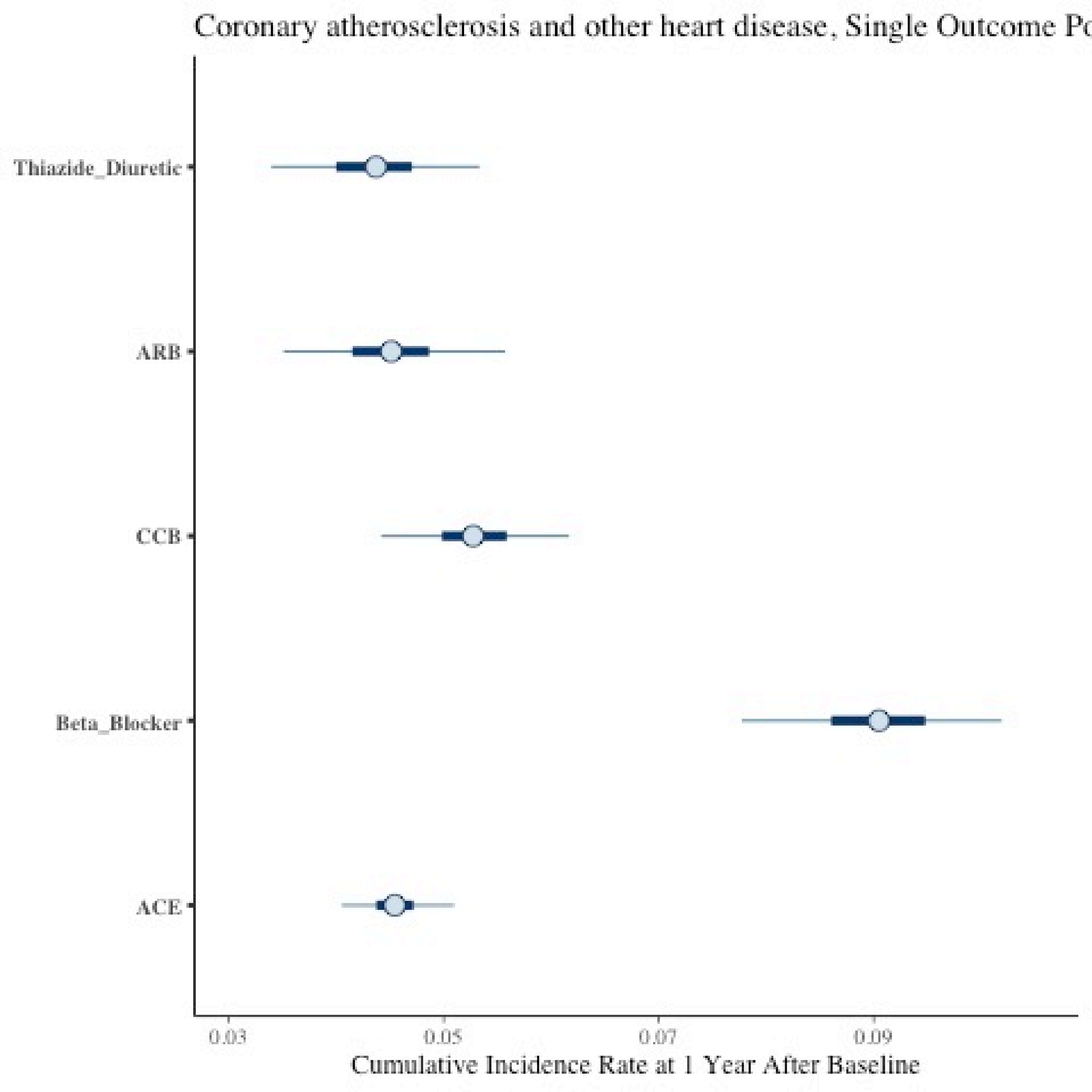

Coronary atherosclerosis and other heart disease, Single Outcome Po
Thiazide_Diuretic
ARB
CCB
Beta_Blocker
ACE
0.03
0.05
0.07
0.09
Cumulative Incidence Rate at 1 Year After Baseline

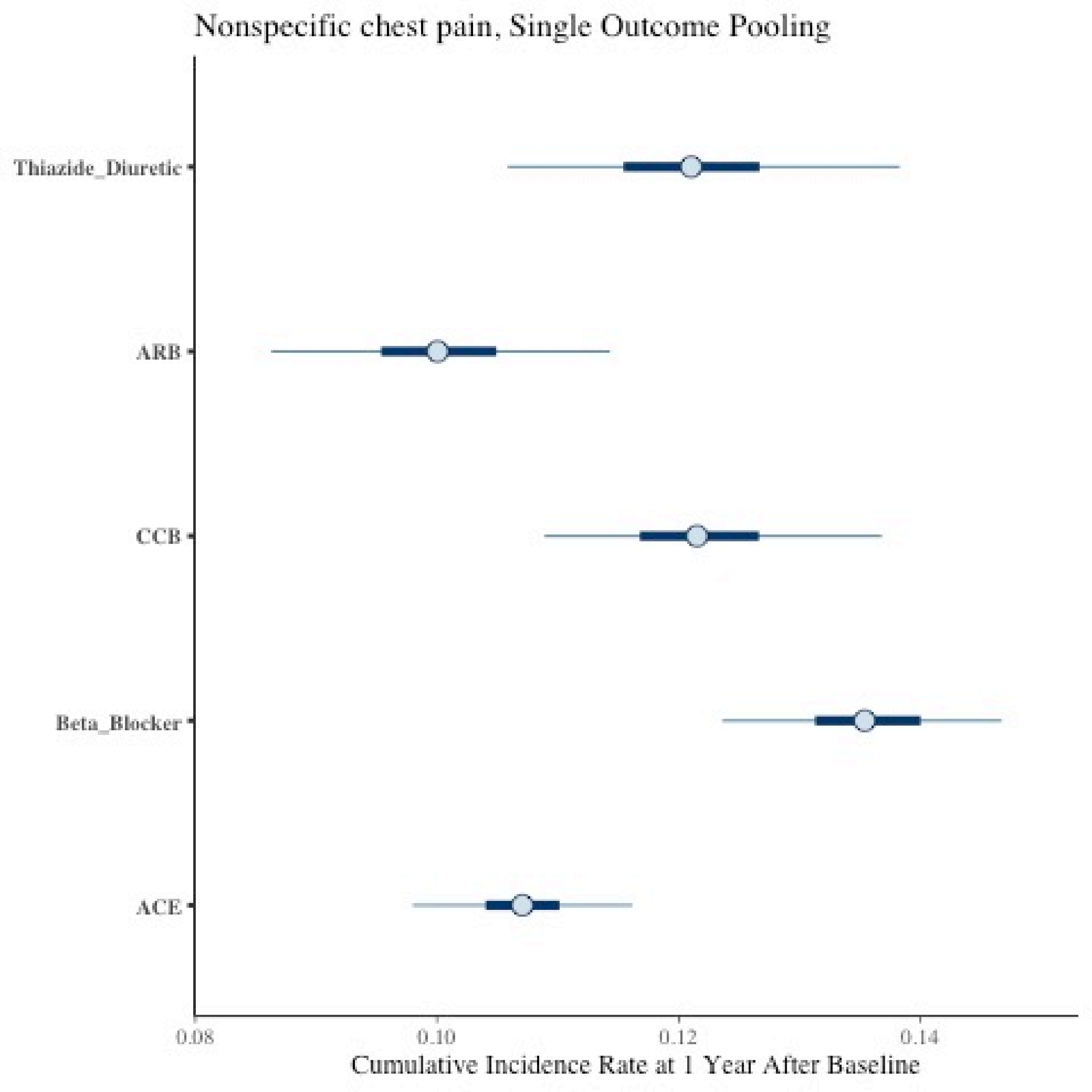
Nonspecific chest pain, Single Outcome Pooling
Thiazide_Diuretic
ARB
CCB
Beta_Blocker
ACE
0.08
0.10
0.12
0.14
Cumulative Incidence Rate at 1 Year After Baseline

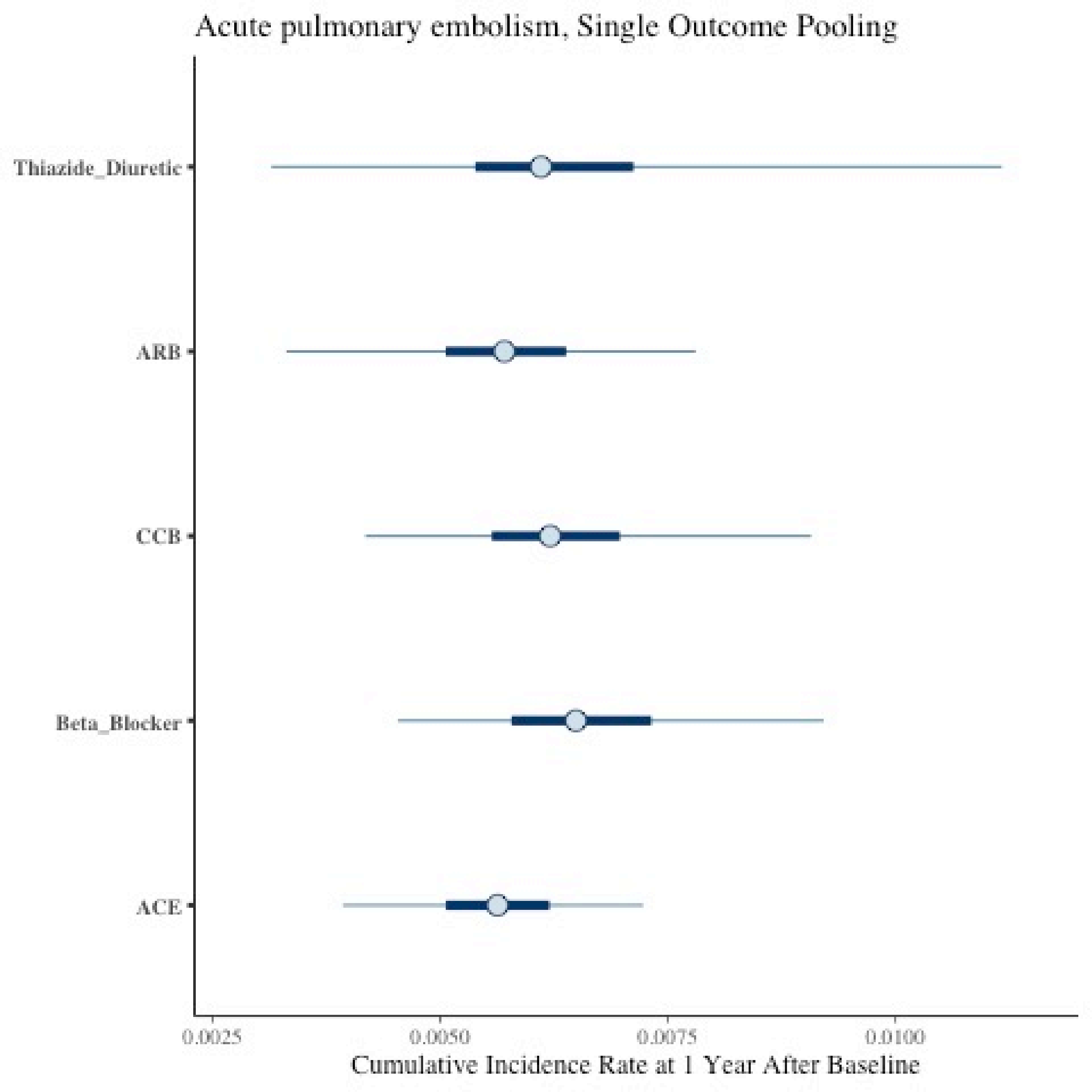

Acute pulmonary embolism, Single Outcome Pooling
Thiazide_Diuretic
ARB
CCB
Beta_Blocker
ACE
0.0025
0.0050
0.0075
0.0100
Cumulative Incidence Rate at 1 Year After Baseline

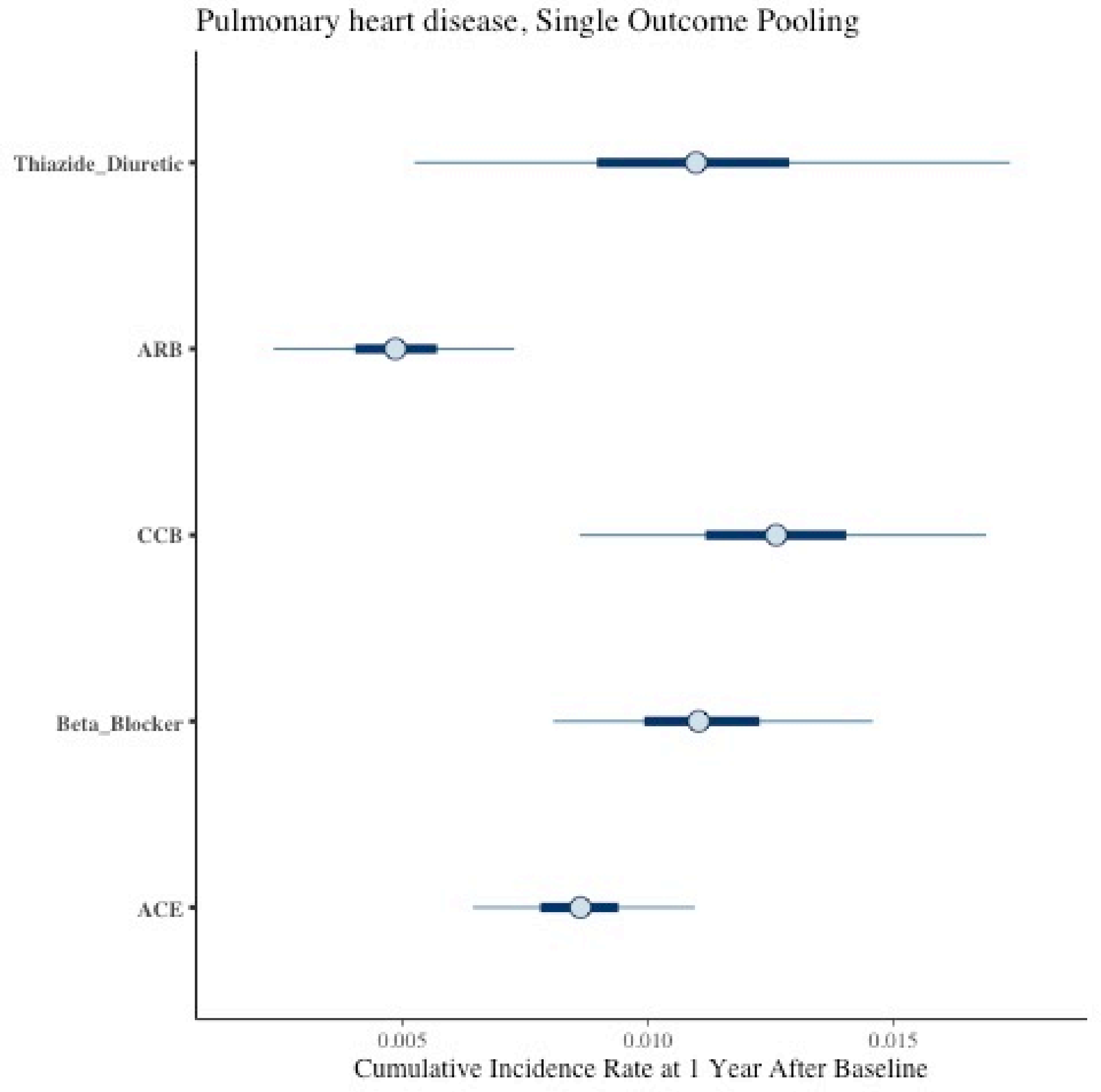

Pulmonary heart disease, Single Outcome Pooling
Thiazide_Diuretic
ARB
CCB
Beta_Blocker
ACE
0.005
0.010
0.015
Cumulative Incidence Rate at 1 Year After Baseline

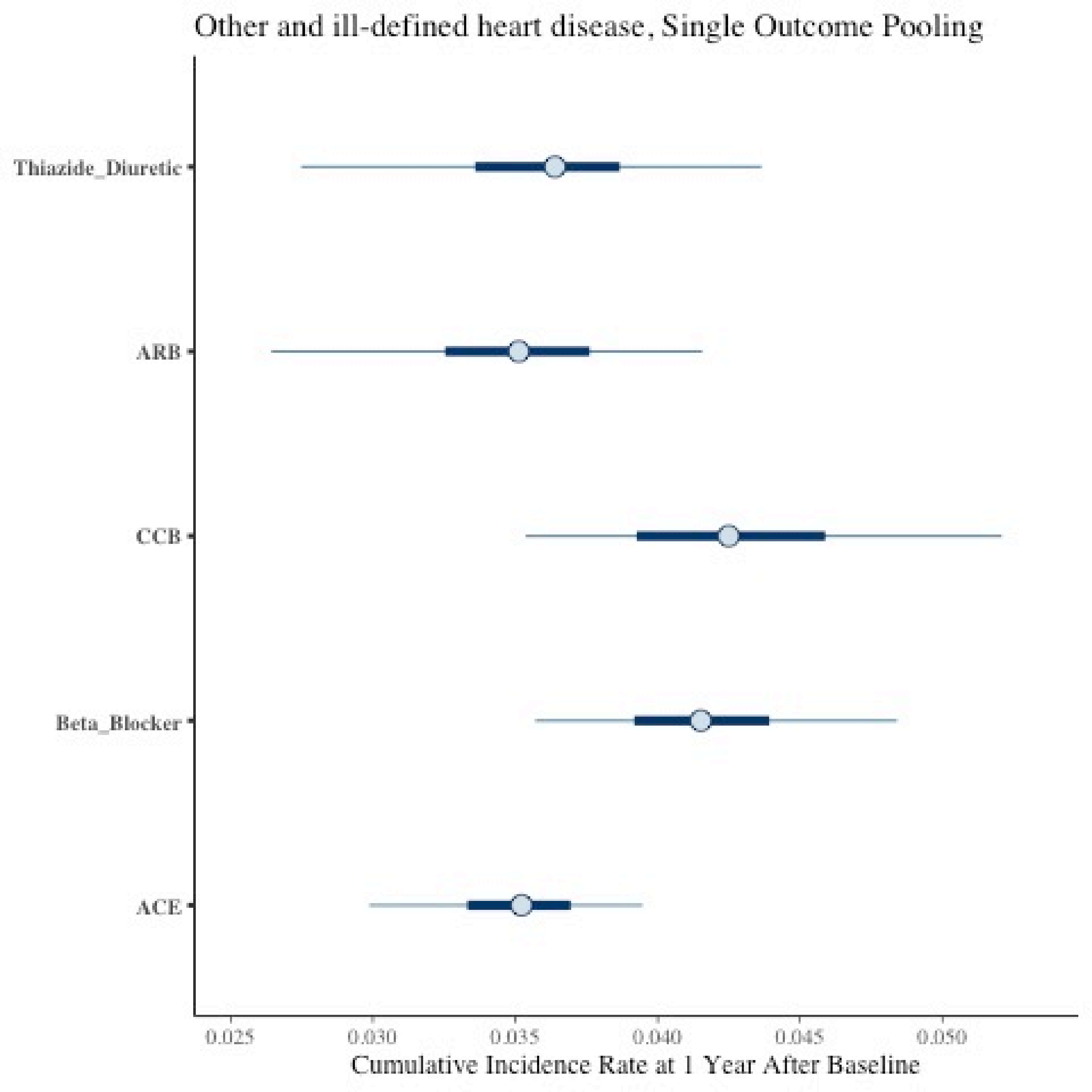

Other and ill-defined heart disease, Single Outcome Pooling
Thiazide_Diuretic
ARB
CCB
Beta_Blocker
ACE
0.025
0.030
0.035
0.040
0.045
0.050
Cumulative Incidence Rate at 1 Year After Baseline

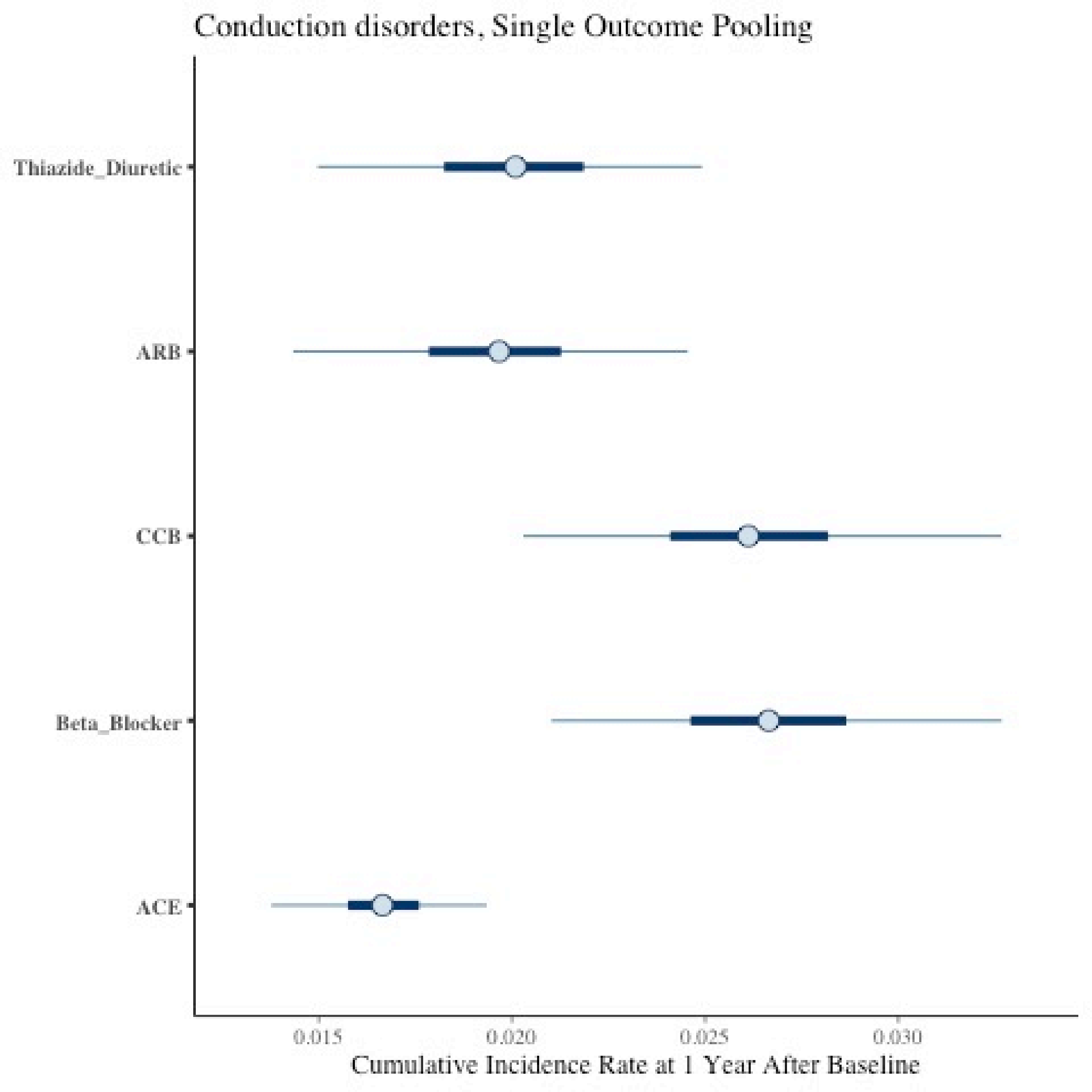
Conduction disorders, Single Outcome Pooling
Thiazide_Diuretic
ARB
CCB
Beta_Blocker
ACE
0.015
0.020
0.025
0.030
Cumulative Incidence Rate at 1 Year After Baseline

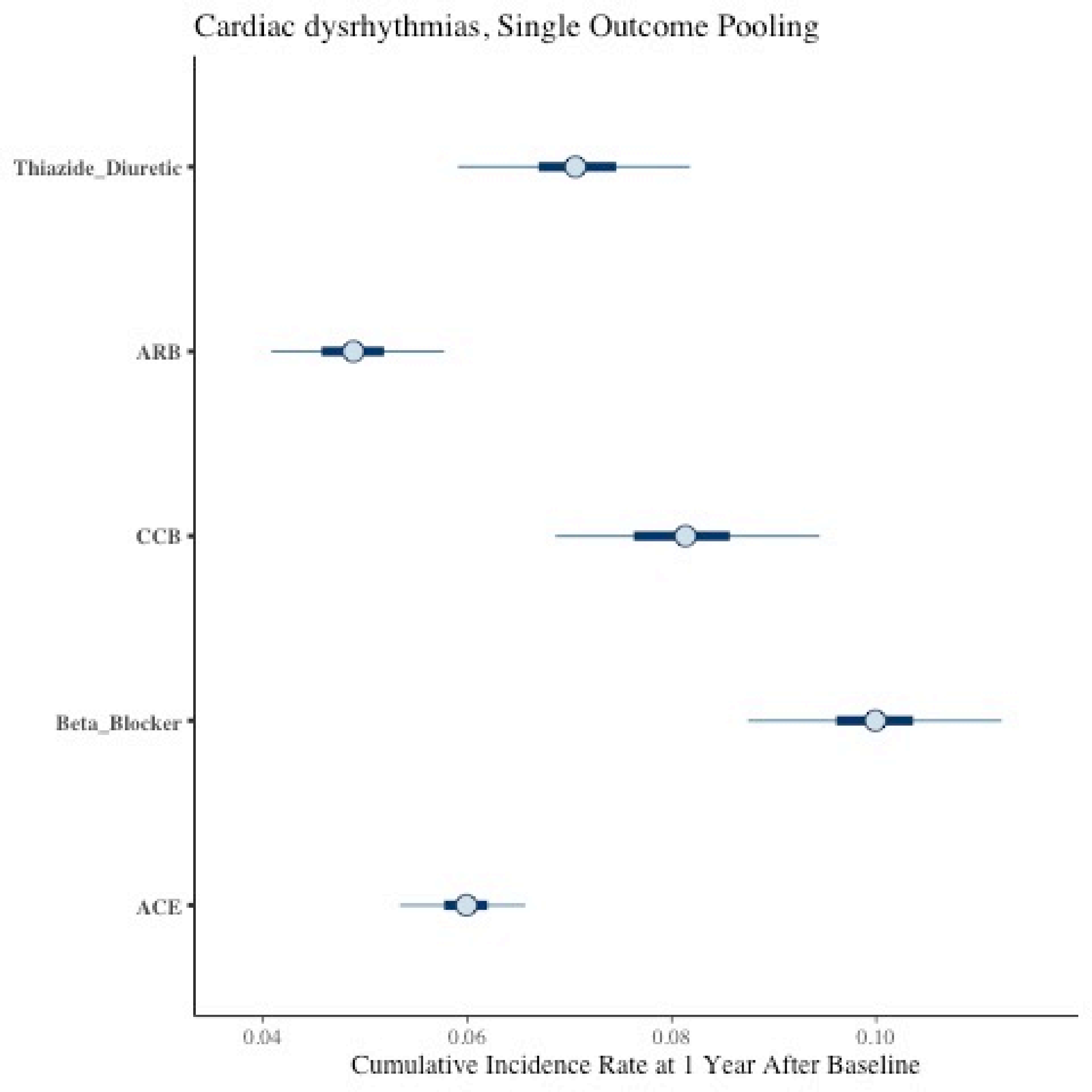
Cardiac dysrhythmias, Single Outcome Pooling
Thiazide_Diuretic
ARB
CCB
Beta_Blocker
ACE
0.04
0.06
0.08
0.10
Cumulative Incidence Rate at 1 Year After Baseline

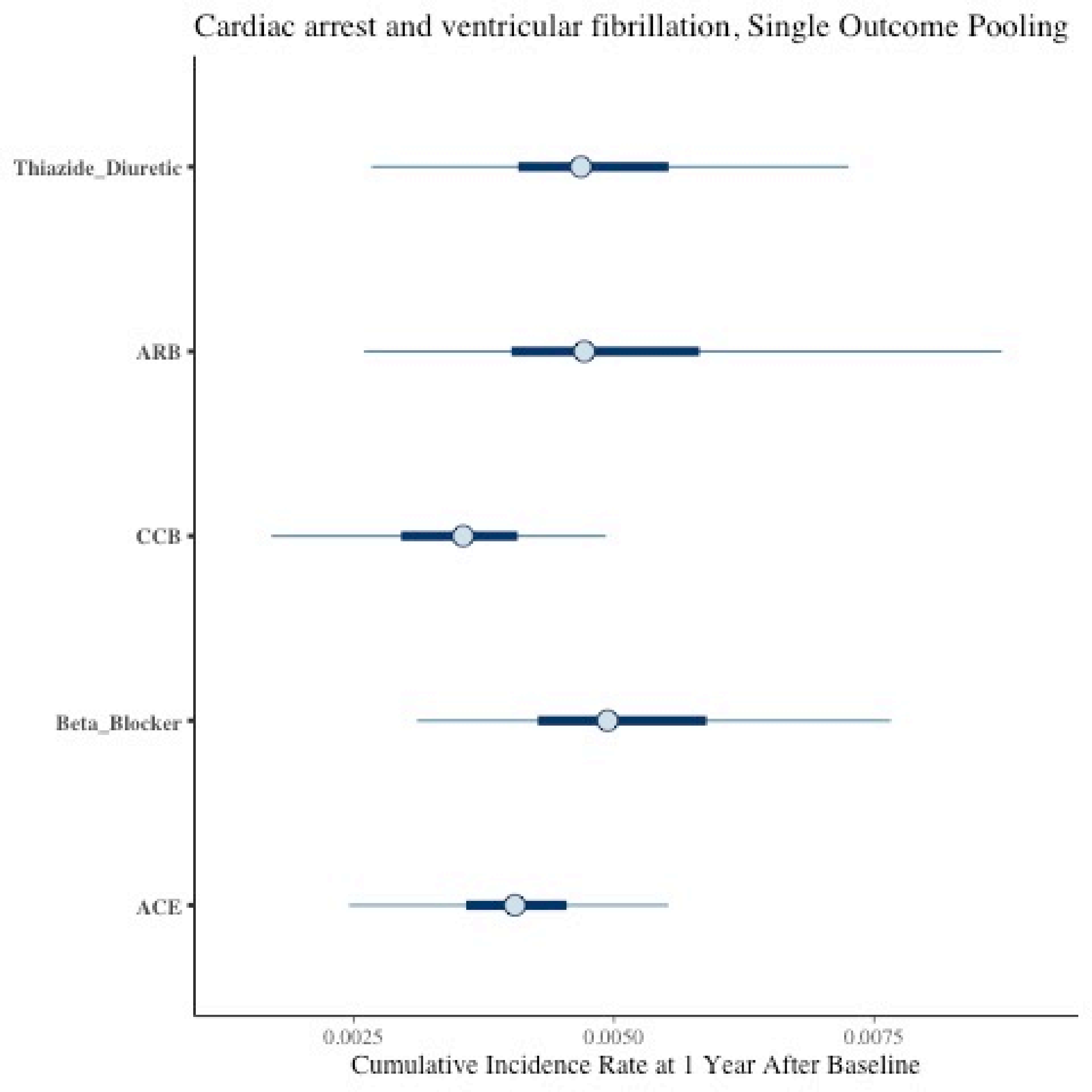

Cardiac arrest and ventricular fibrillation, Single Outcome Pooling
Thiazide_Diuretic
ARB
CCB
Beta_Blocker
ACE
0.0025
0.0050
0.0075
Cumulative Incidence Rate at 1 Year After Baseline

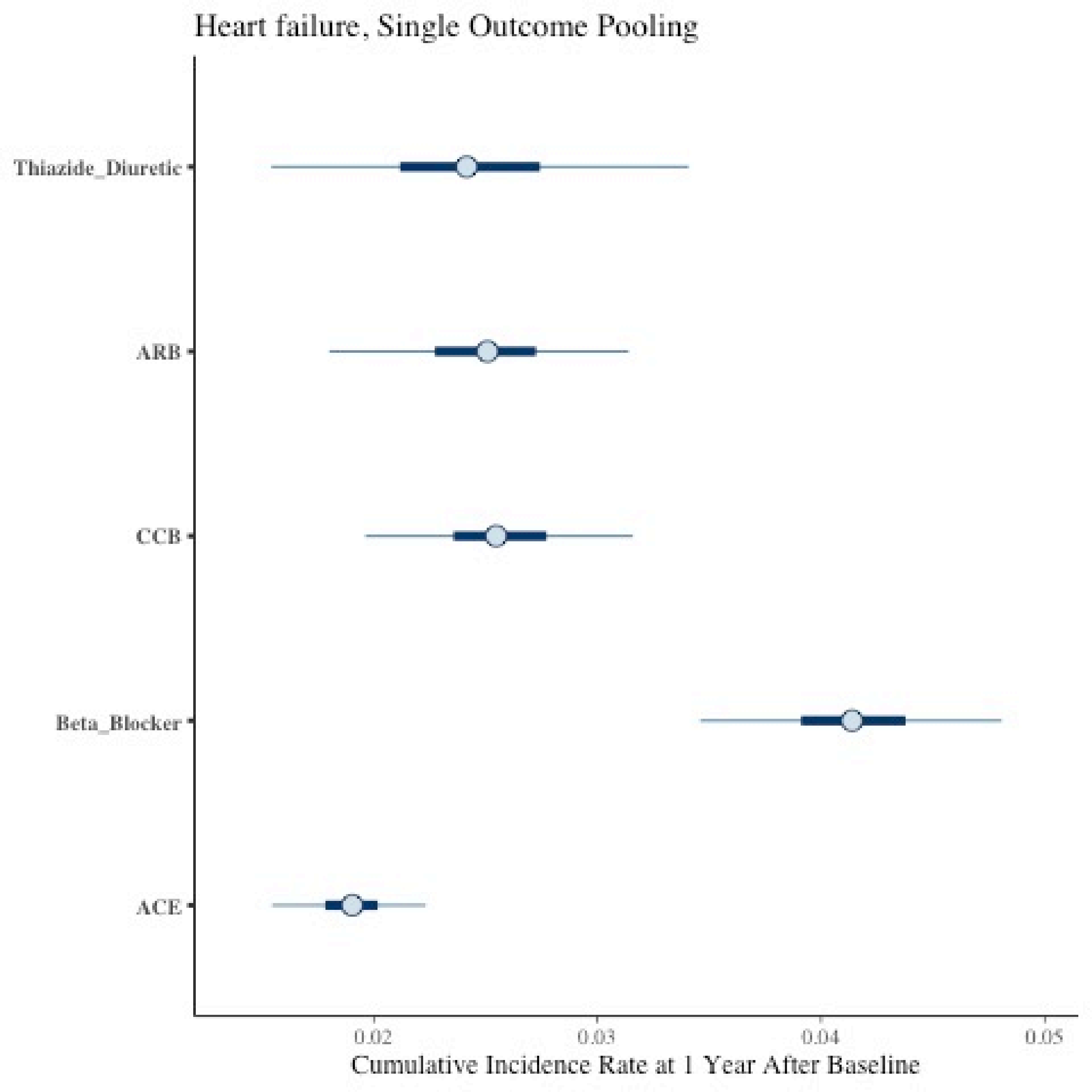
Heart failure, Single Outcome Pooling
Thiazide_Diuretic
ARB
CCB
Beta_Blocker
ACE
0.02
0.03
0.04
0.05
Cumulative Incidence Rate at 1 Year After Baseline

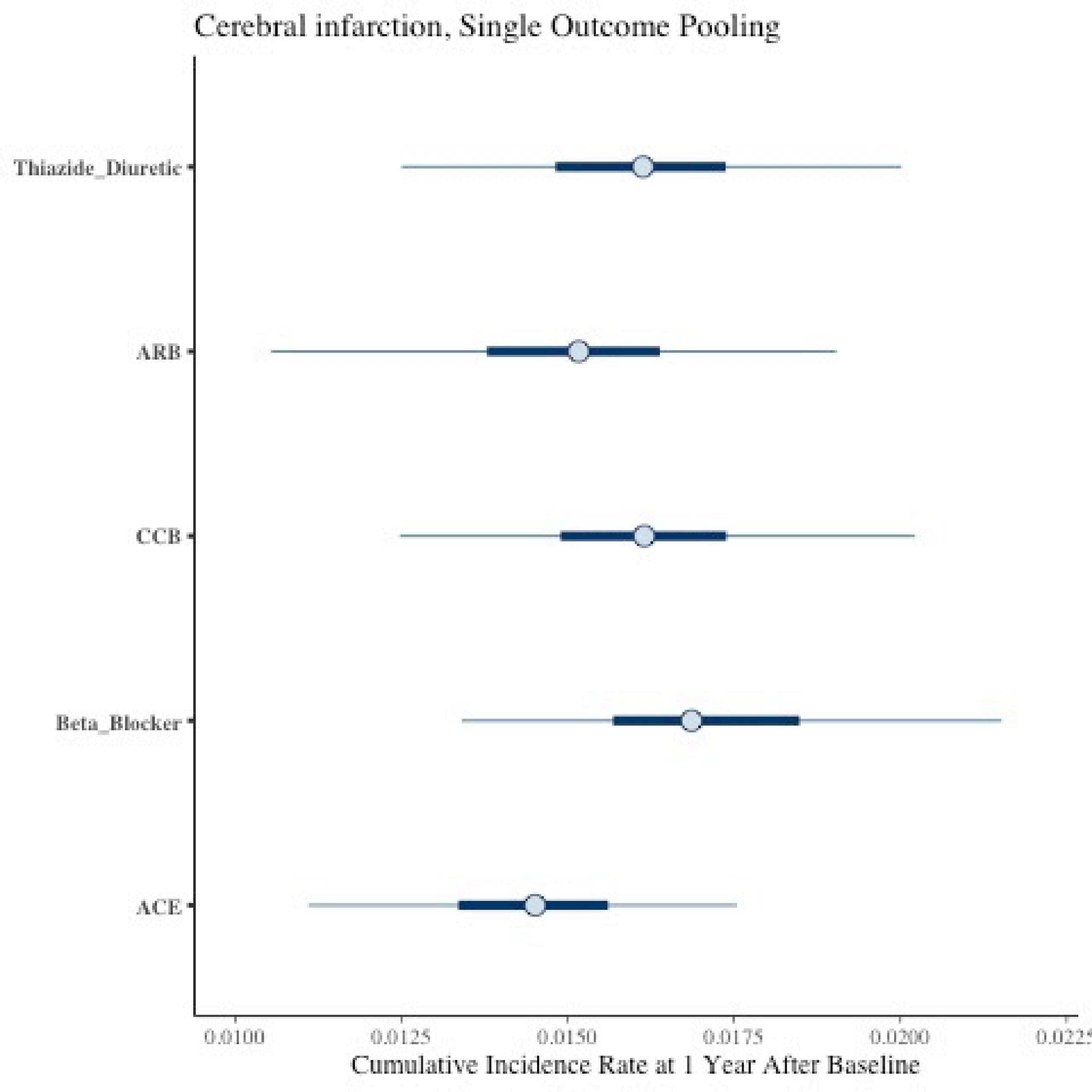
Cerebral infarction, Single Outcome Pooling
Thiazide_Diuretic
ARB
CCB
Beta_Blocker
ACE
0.0100
0.0125
0.0150
0.0175
0.0200
0.0225
Cumulative Incidence Rate at 1 Year After Baseline

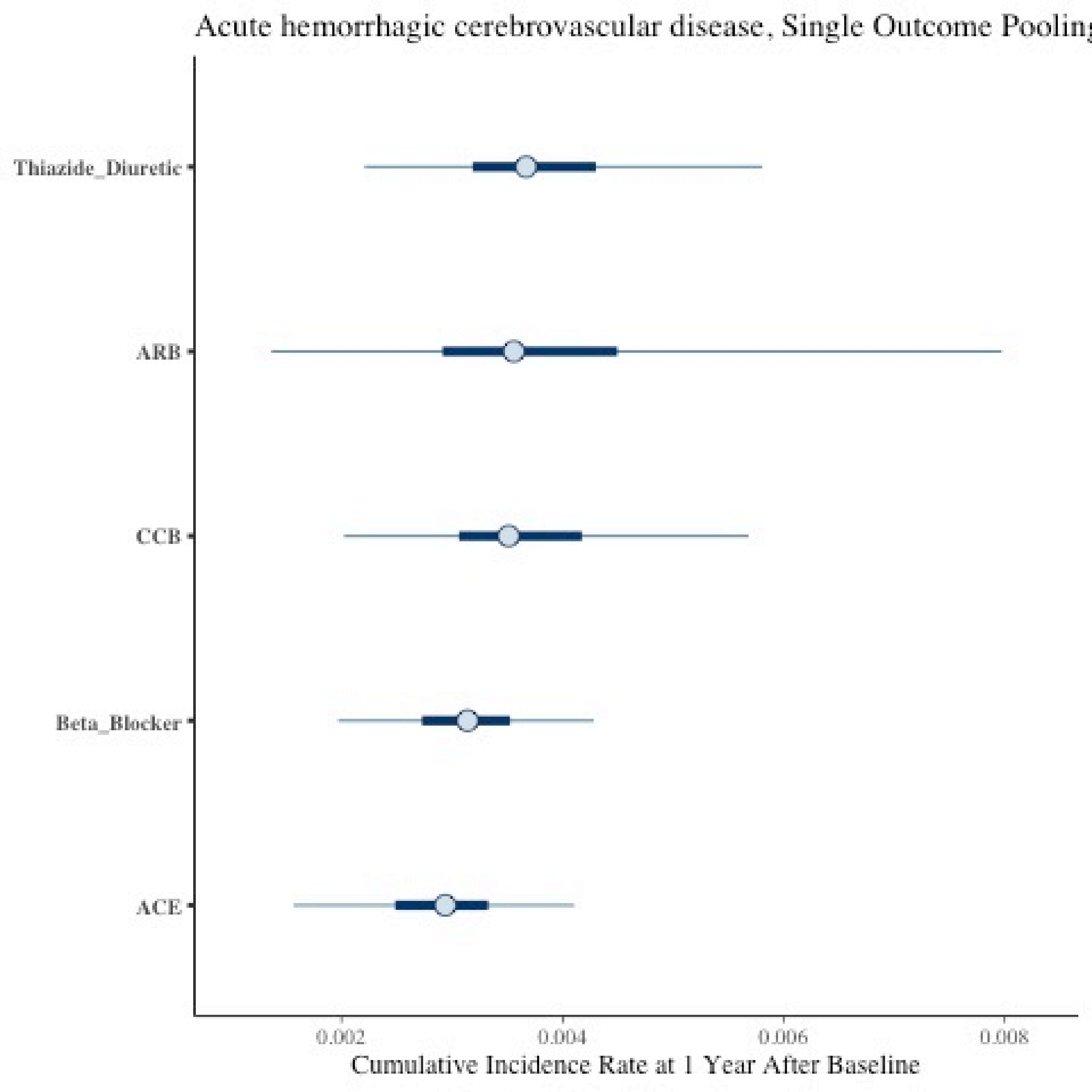

Acute hemorrhagic cerebrovascular disease, Single Outcome Pooling
Thiazide_Diuretic
ARB
CCB
Beta_Blocker
ACE
0.002
0.004
0.006
0.008
Cumulative Incidence Rate at 1 Year After Baseline

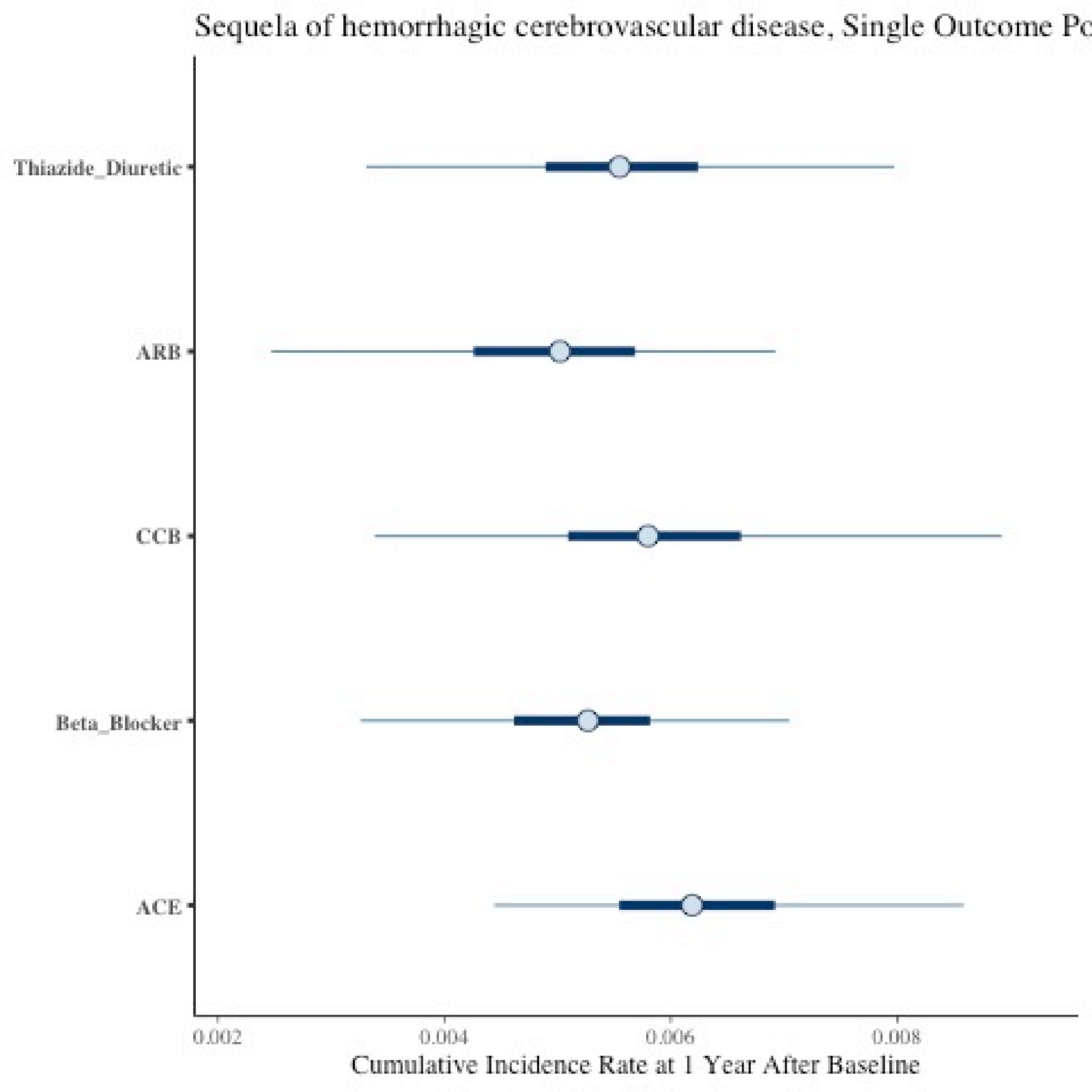

Sequela of hemorrhagic cerebrovascular disease, Single Outcome Po
Thiazide_Diuretic
ARB
CCB
Beta_Blocker
ACE
0.002
0.004
0.006
0.008
Cumulative Incidence Rate at 1 Year After Baseline

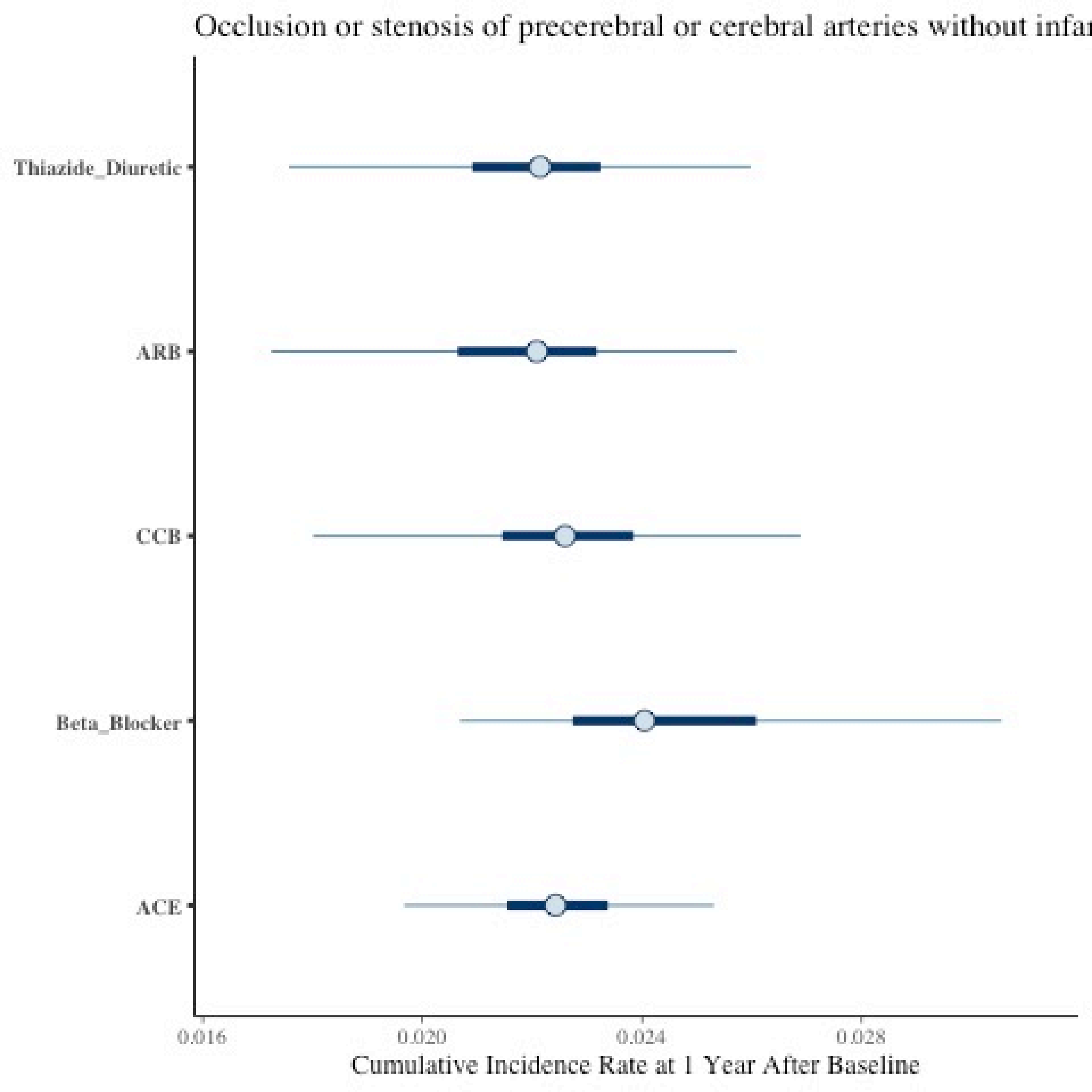

Occlusion or stenosis of precerebral or cerebral arteries without infar
Thiazide_Diuretic
ARB
CCB
Beta_Blocker
ACE
0.016
0.020
0.024
0.028
Cumulative Incidence Rate at 1 Year After Baseline

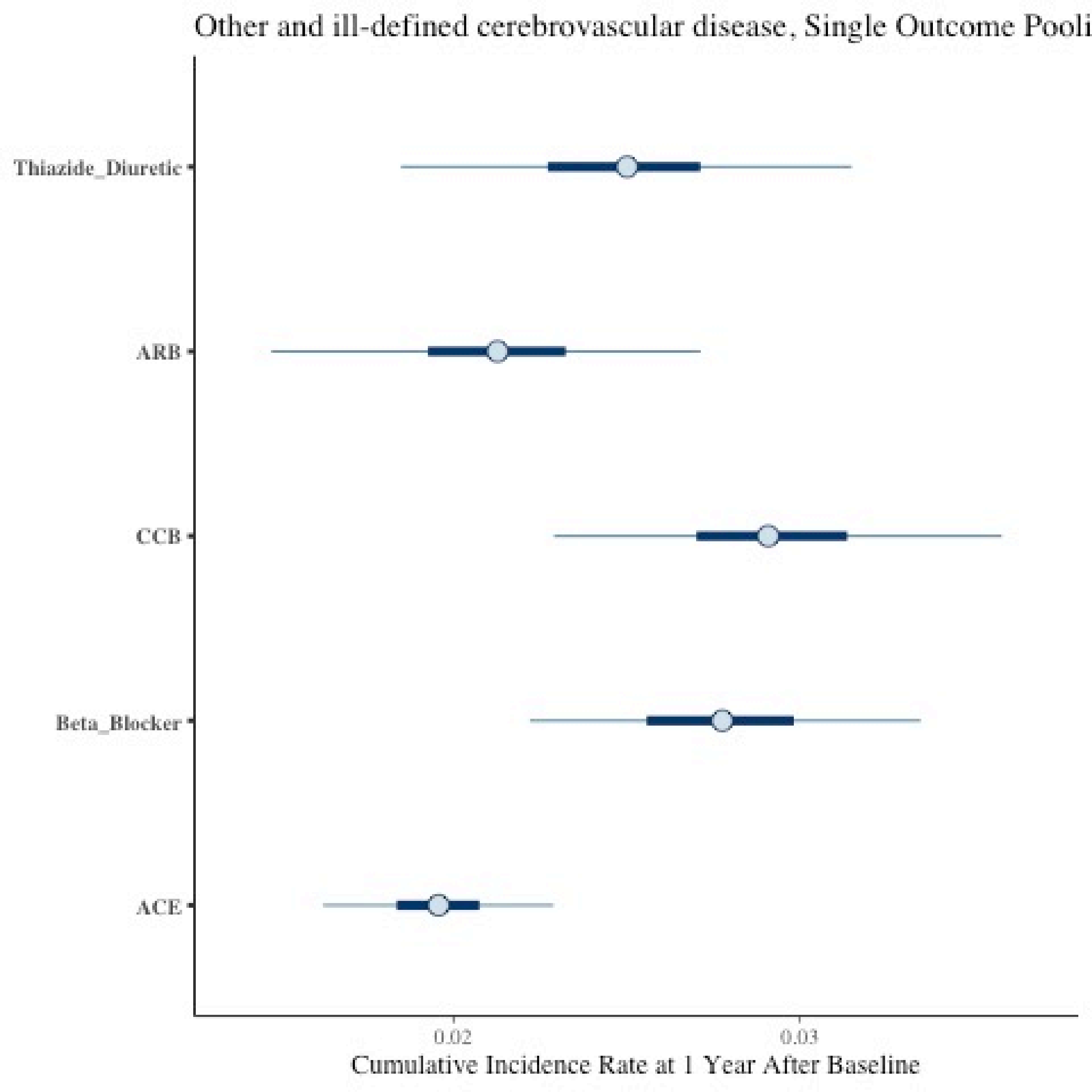

Other and ill-defined cerebrovascular disease, Single Outcome Pooli
Thiazide_Diuretic
ARB
CCB
Beta_Blocker
ACE
0.02
0.03
Cumulative Incidence Rate at 1 Year After Baseline

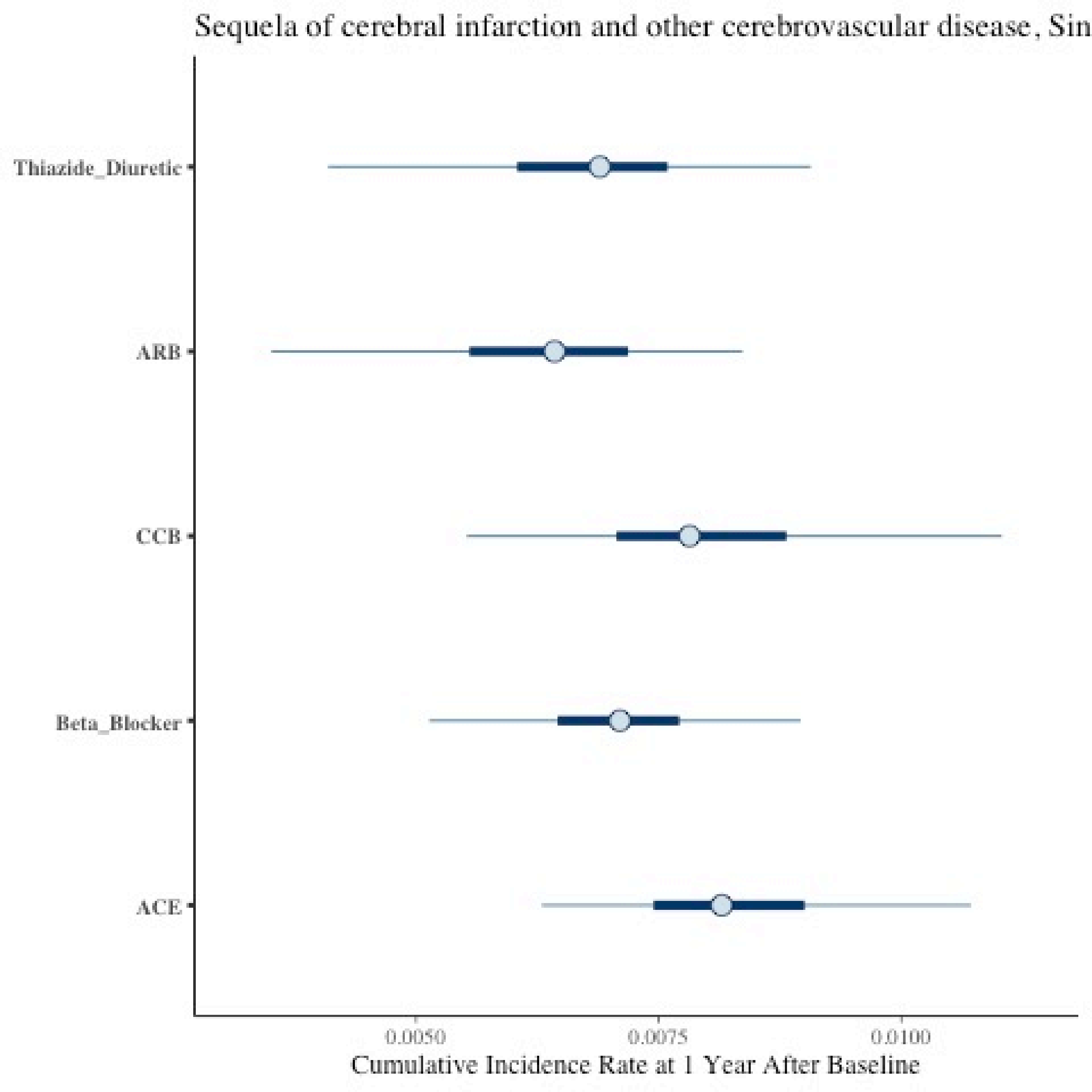

Sequela of cerebral infarction and other cerebrovascular disease, Sin
Thiazide_Diuretic
ARB
CCB
Beta_Blocker
ACE
0.0050
0.0075
0.0100
Cumulative Incidence Rate at 1 Year After Baseline

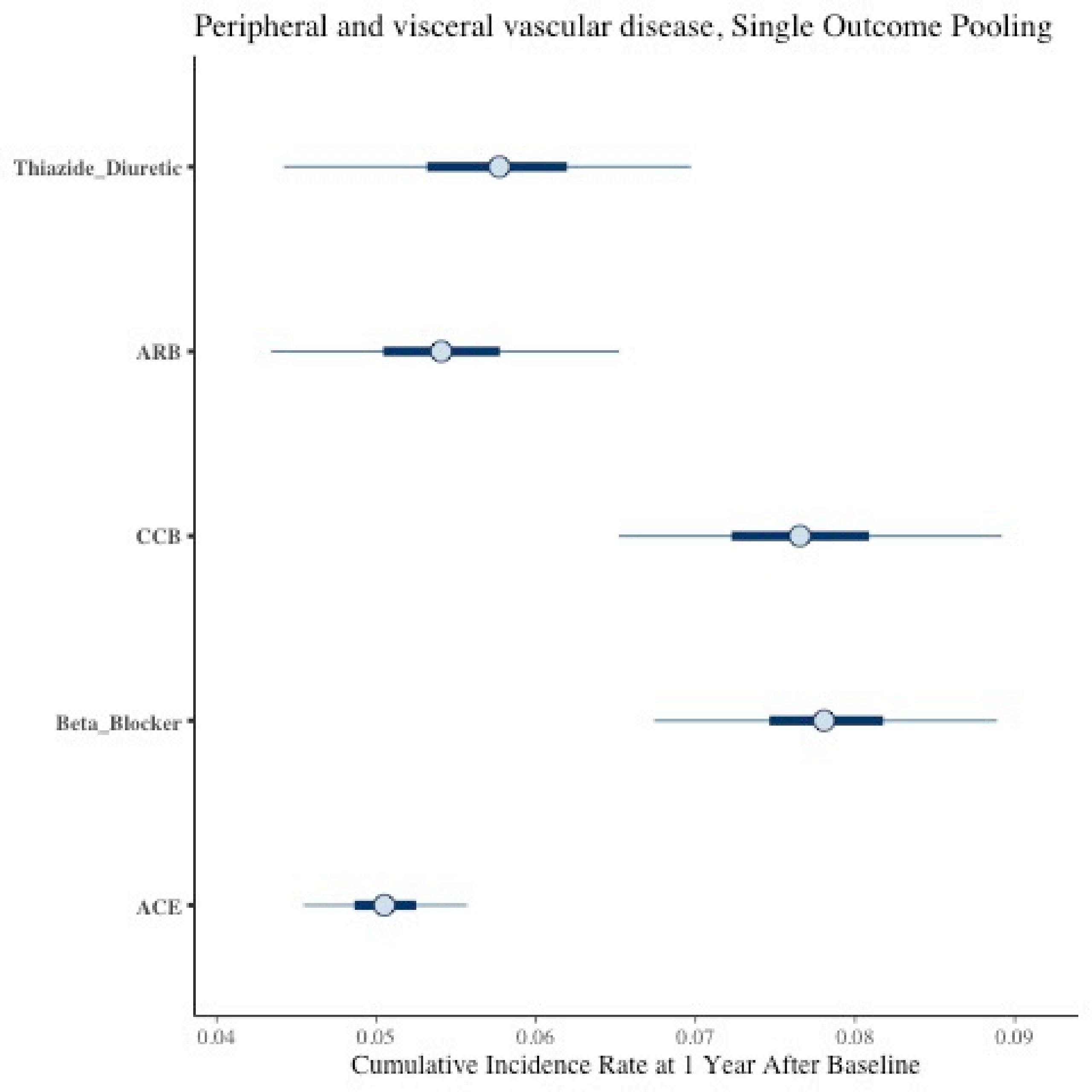
Peripheral and visceral vascular disease, Single Outcome Pooling
Thiazide_Diuretic
ARB
CCB
Beta_Blocker
ACE
0.04
0.05
0.06
0.07
0.08
0.09
Cumulative Incidence Rate at 1 Year After Baseline

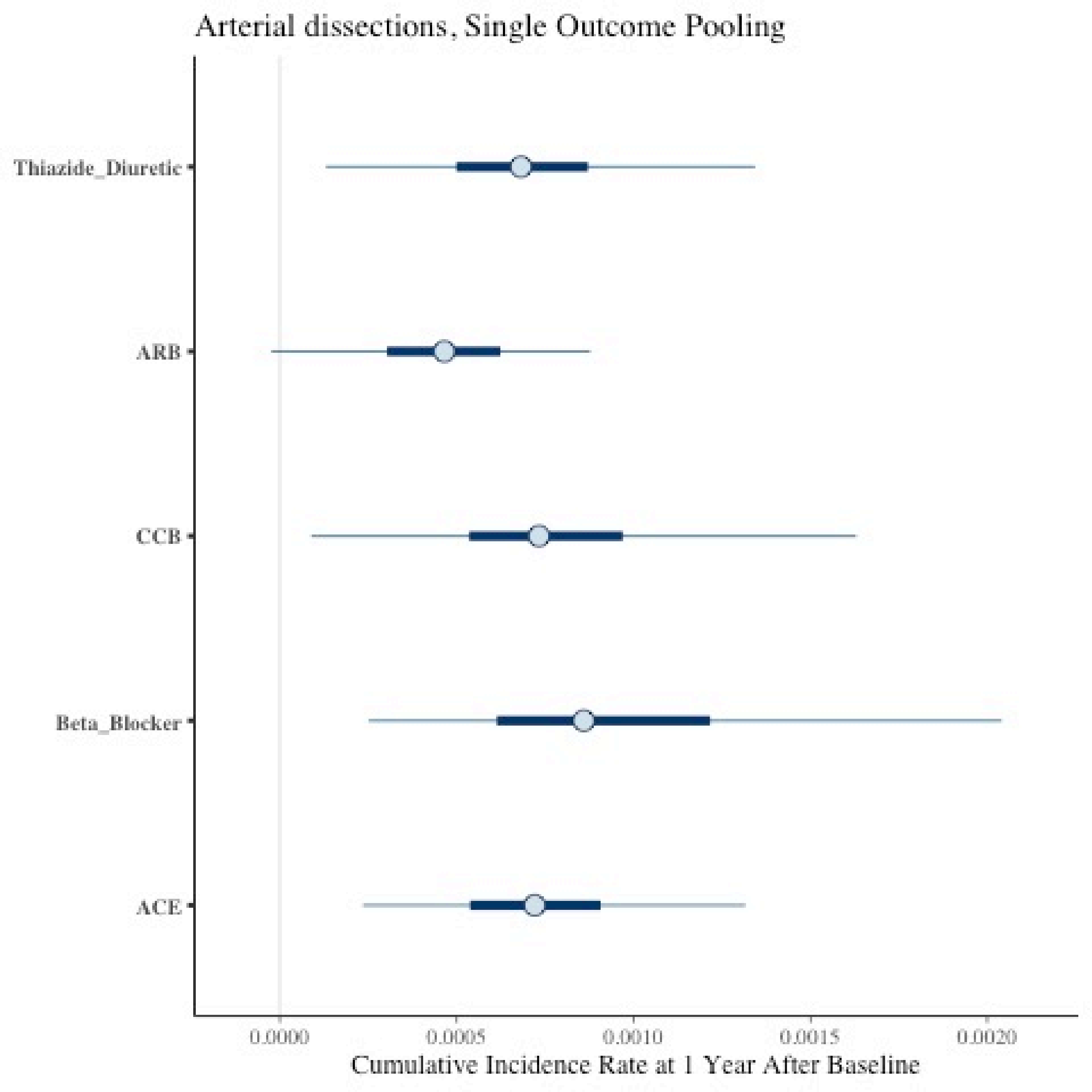
Arterial dissections, Single Outcome Pooling
Thiazide_Diuretic
ARB
CCB
Beta_Blocker
ACE
0.0000
0.0005
0.0010
0.0015
0.0020
Cumulative Incidence Rate at 1 Year After Baseline

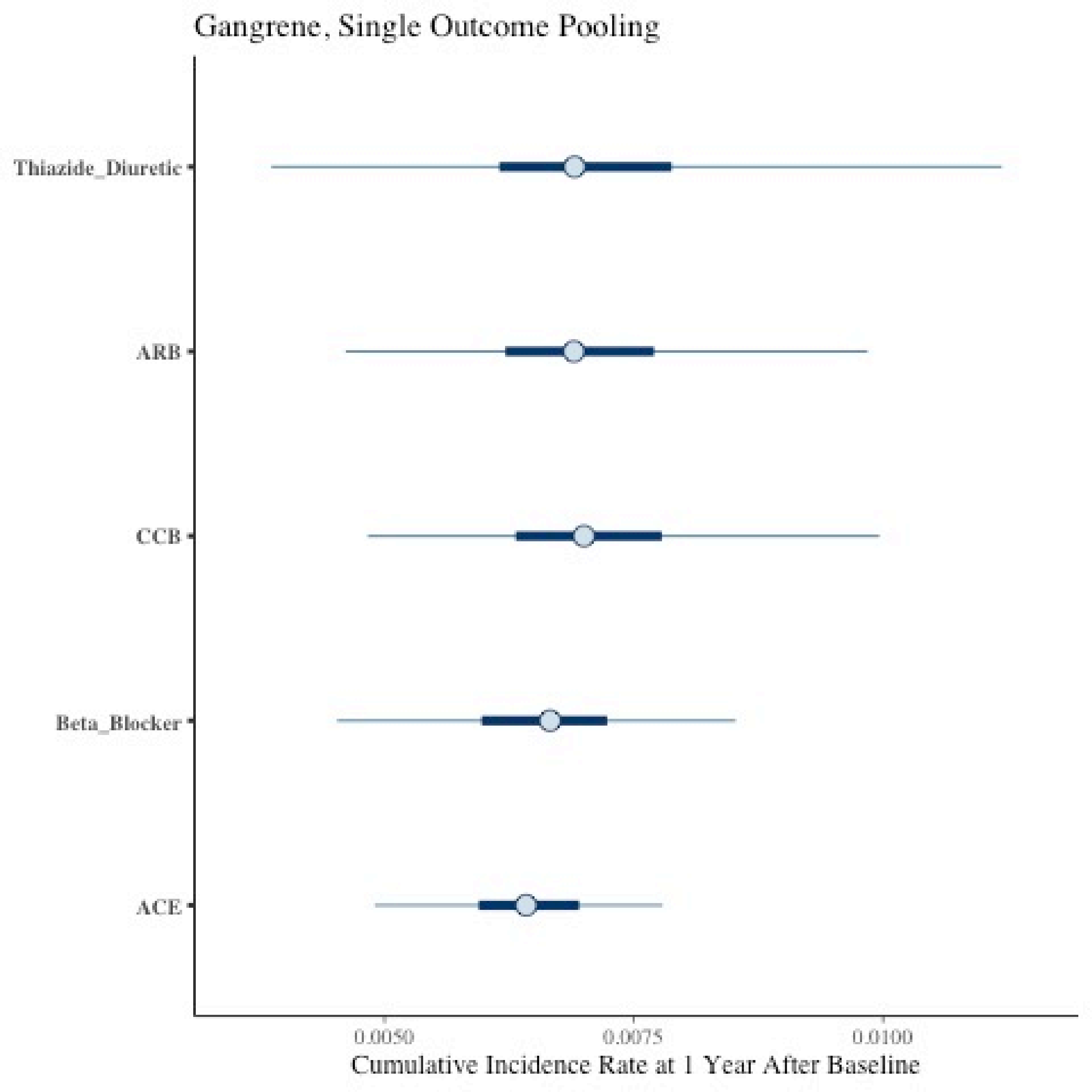
Gangrene, Single Outcome Pooling
Thiazide_Diuretic
ARB
CCB
Beta_Blocker
ACE
0.0050
0.0075
0.0100
Cumulative Incidence Rate at 1 Year After Baseline

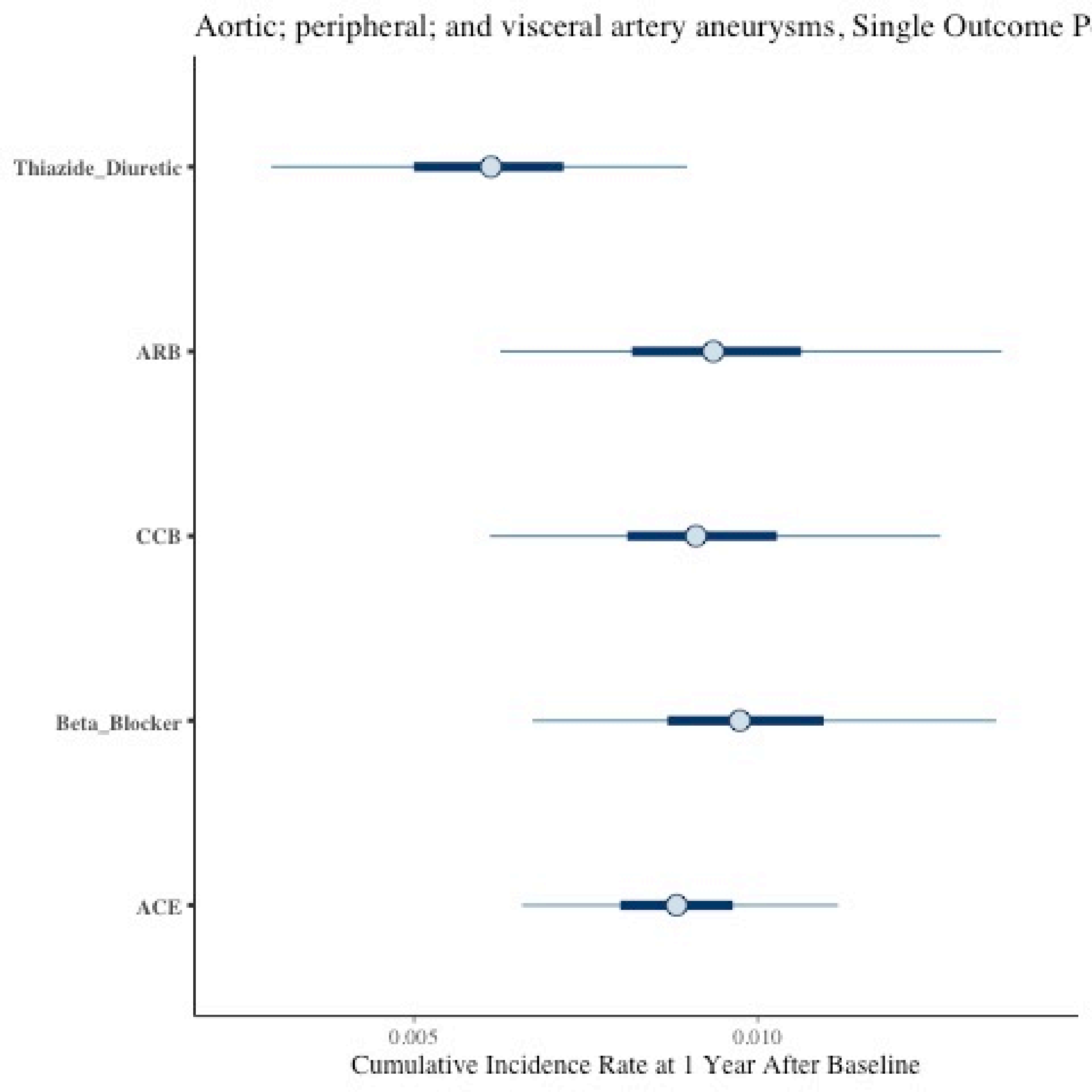

Aortic; peripheral; and visceral artery aneurysms, Single Outcome P
Thiazide_Diuretic
ARB
CCB
Beta_Blocker
ACE
0.005
0.010
Cumulative Incidence Rate at 1 Year After Baseline

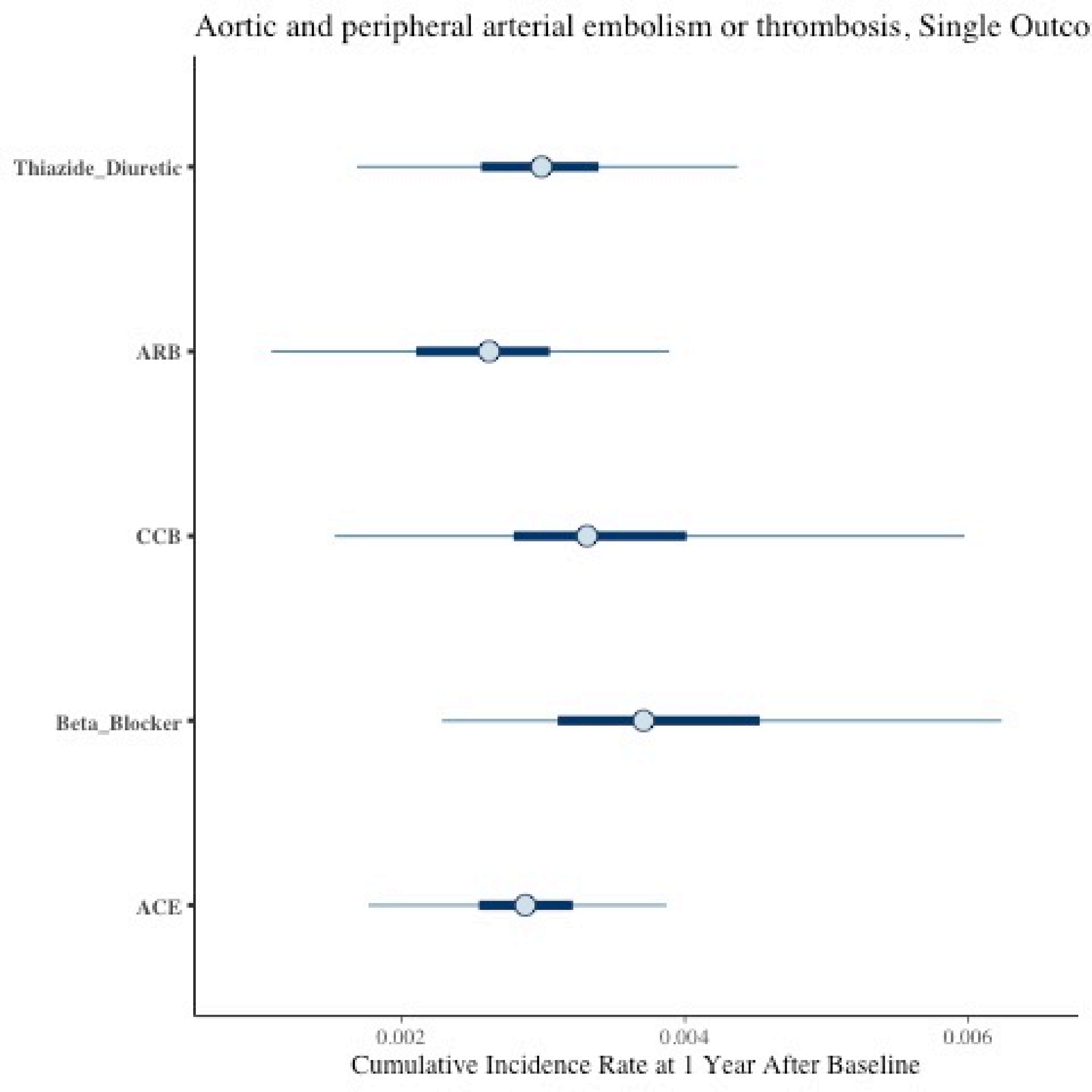
Aortic and peripheral arterial embolism or thrombosis, Single Outco
Thiazide_Diuretic
ARB
CCB
Beta_Blocker
ACE
0.002
0.004
0.006
Cumulative Incidence Rate at 1 Year After Baseline

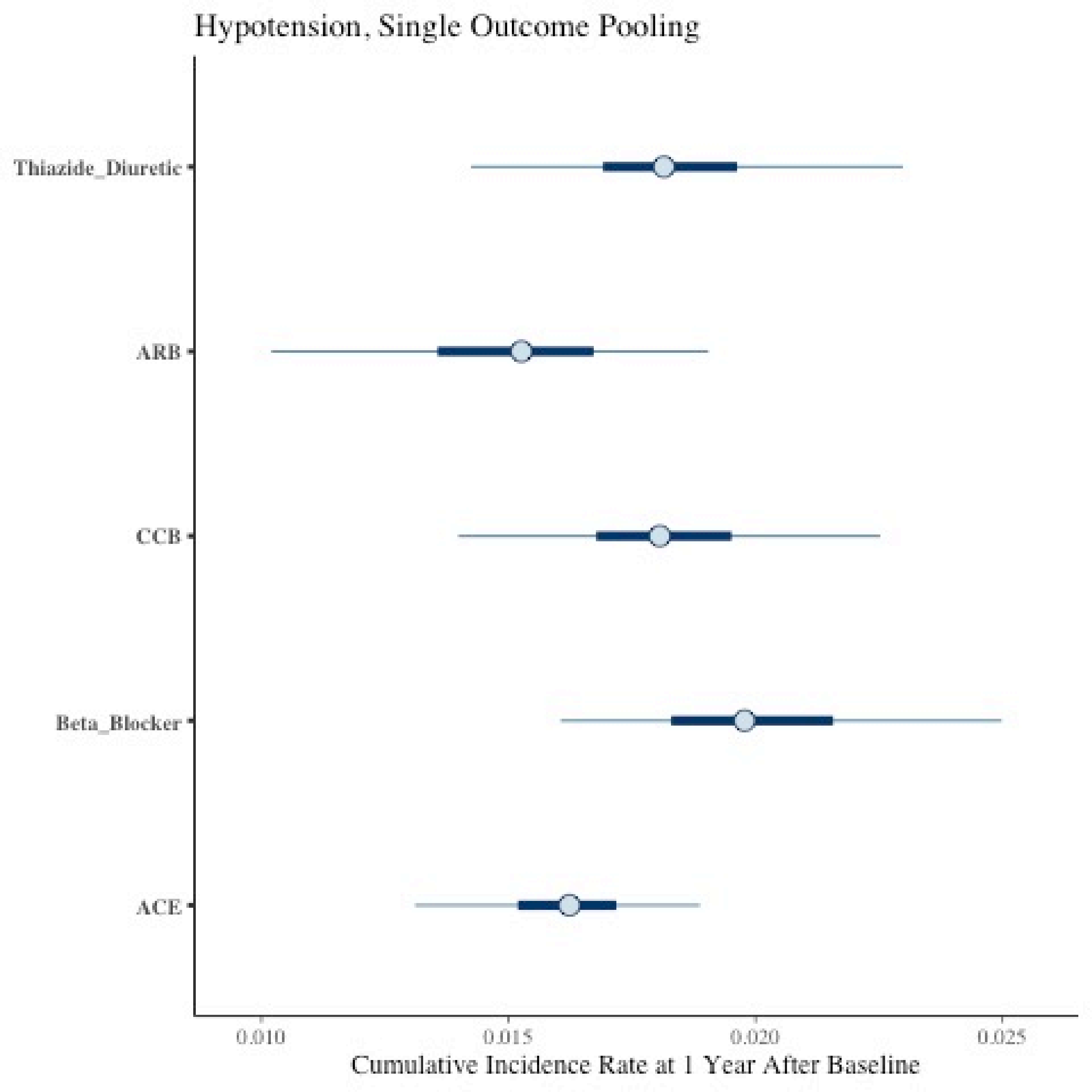
Hypotension, Single Outcome Pooling
Thiazide_Diuretic
ARB
CCB
Beta_Blocker
ACE
0.010
0.015
0.020
0.025
Cumulative Incidence Rate at 1 Year After Baseline

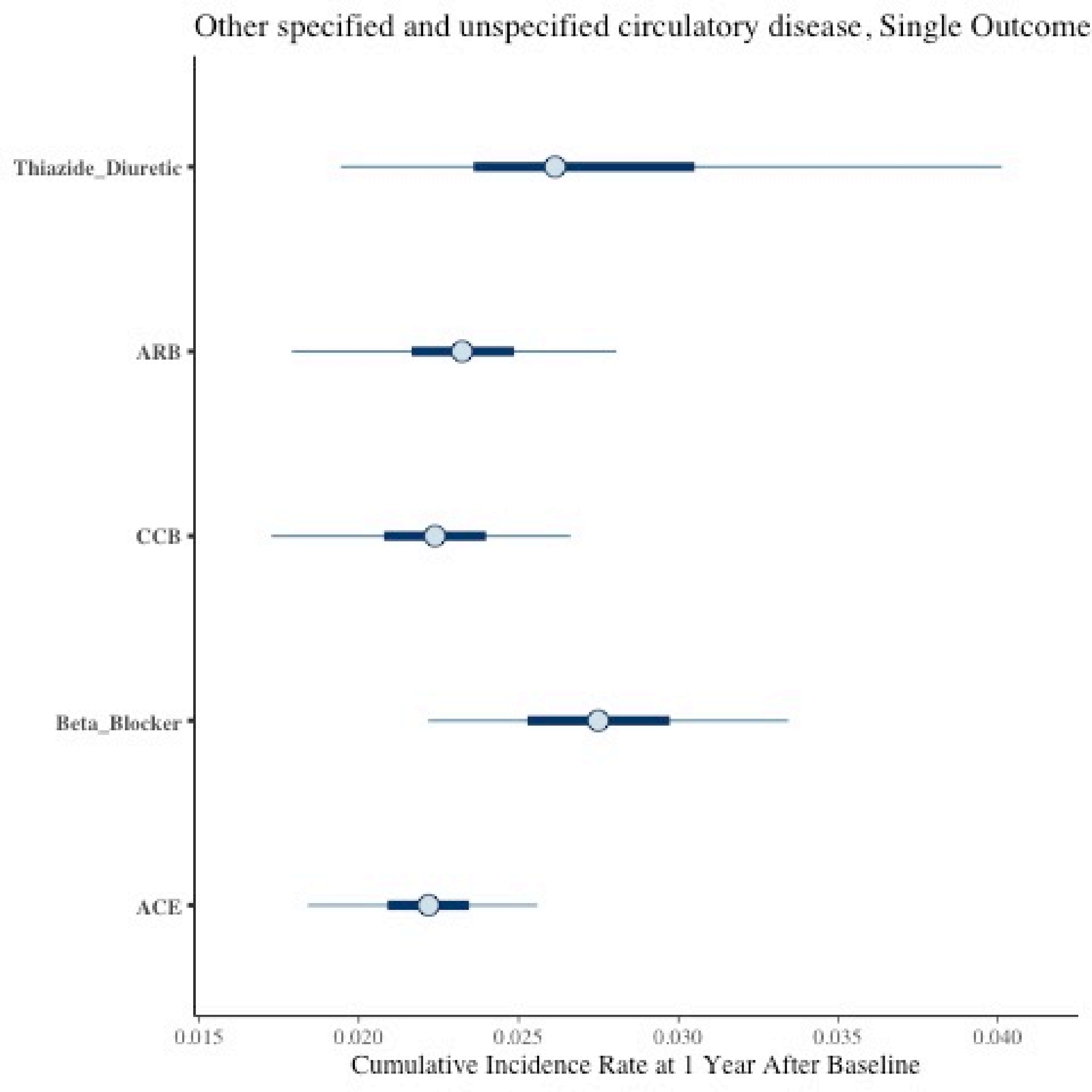
Other specified and unspecified circulatory disease, Single Outcome
Thiazide_Diuretic
ARB
CCB
Beta_Blocker
ACE
0.015
0.020
0.025
0.030
0.035
0.040
Cumulative Incidence Rate at 1 Year After Baseline

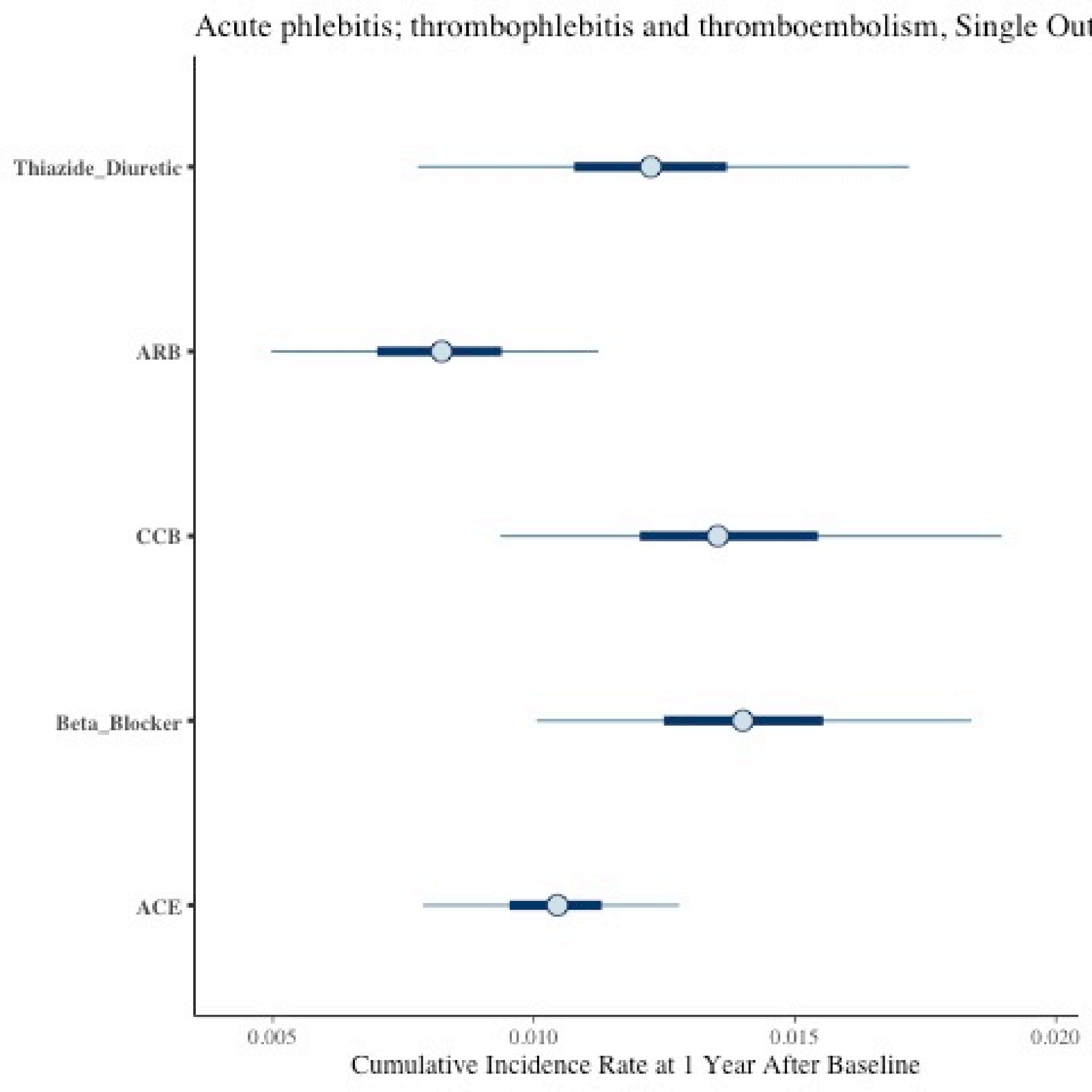

Acute phlebitis; thrombophlebitis and thromboembolism, Single Out
Thiazide_Diuretic
ARB
CCB
Beta_Blocker
ACE
0.005
0.010
0.015
0.020
Cumulative Incidence Rate at 1 Year After Baseline

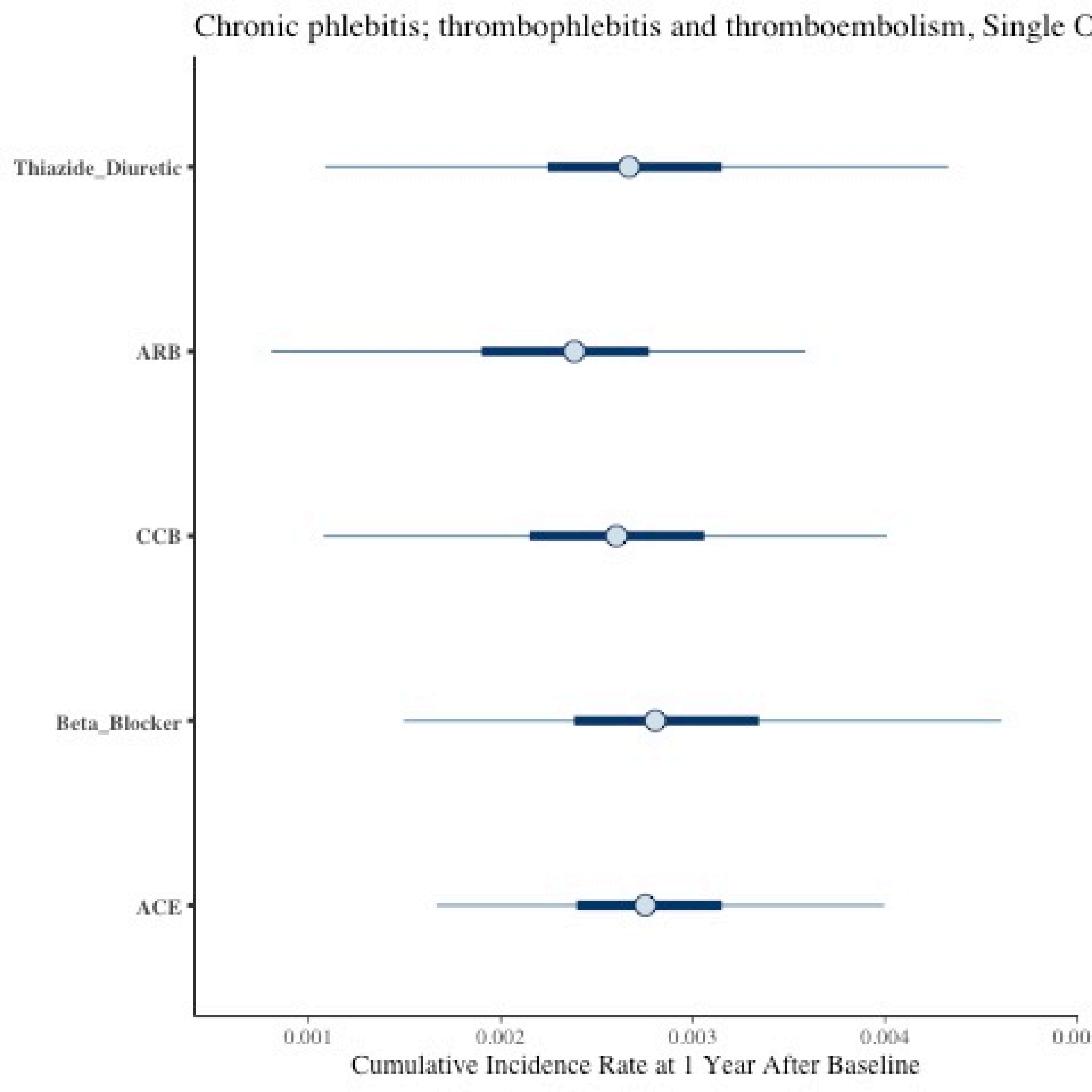

Chronic phlebitis; thrombophlebitis and thromboembolism, Single C
Thiazide_Diuretic
ARB
CCB
Beta_Blocker
ACE
0.001
0.002
0.003
0.004
0.00
Cumulative Incidence Rate at 1 Year After Baseline

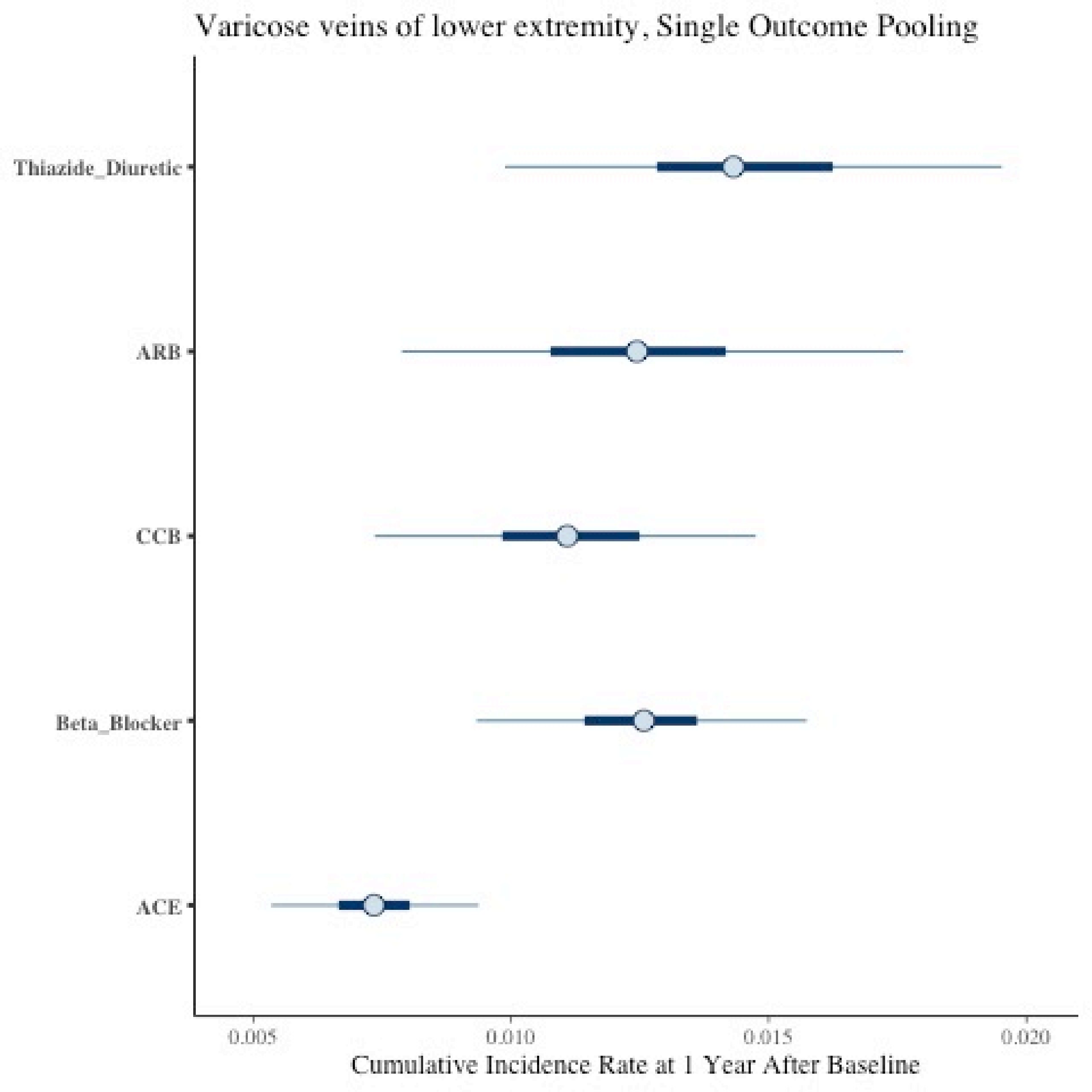

Varicose veins of lower extremity, Single Outcome Pooling
Thiazide_Diuretic
ARB
CCB
Beta_Blocker
ACE
0.005
0.010
0.015
0.020
Cumulative Incidence Rate at 1 Year After Baseline

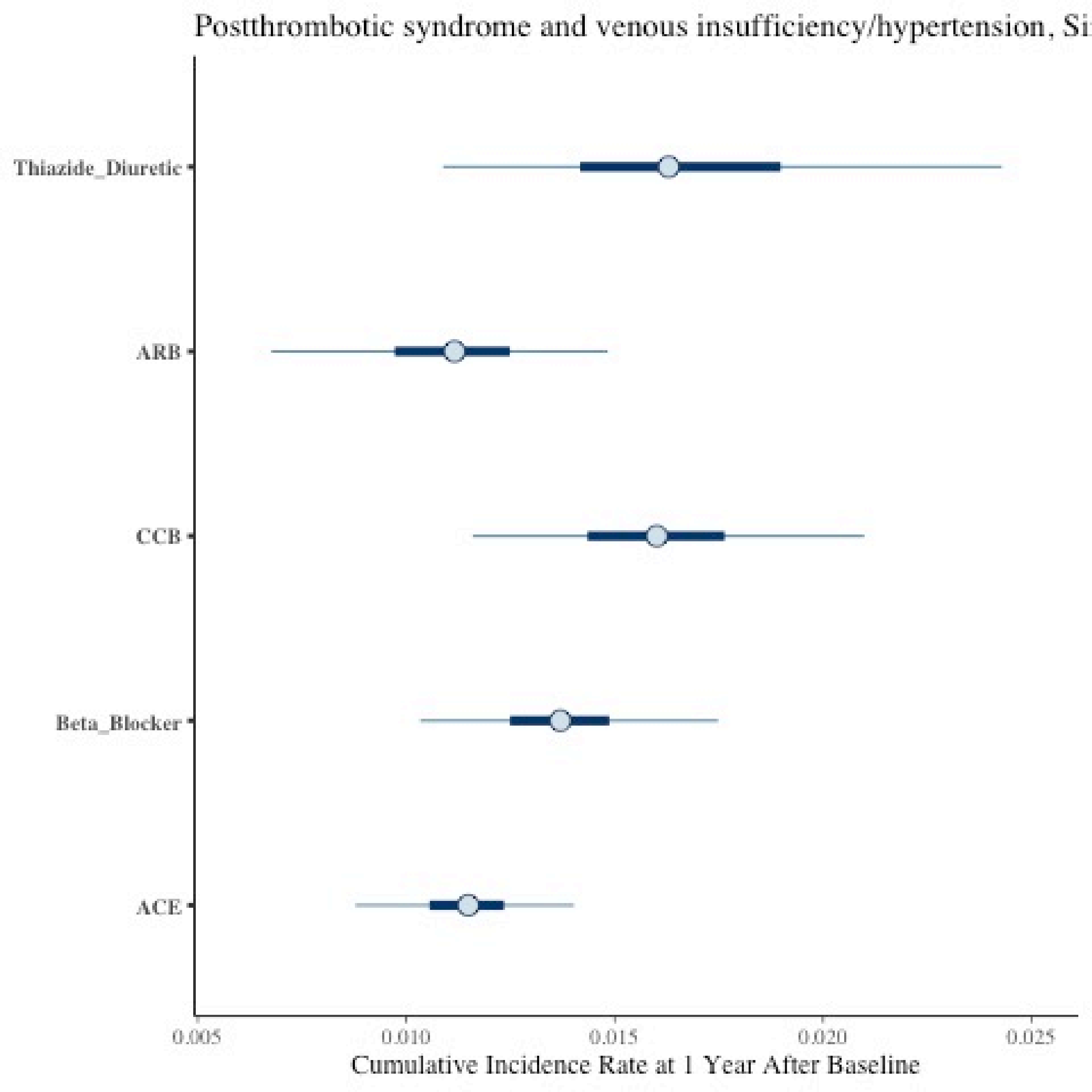

Postthrombotic syndrome and venous insufficiency/hypertension, Si
Thiazide_Diuretic
ARB
CCB
Beta_Blocker
ACE
0.005
0.010
0.015
0.020
0.025
Cumulative Incidence Rate at 1 Year After Baseline

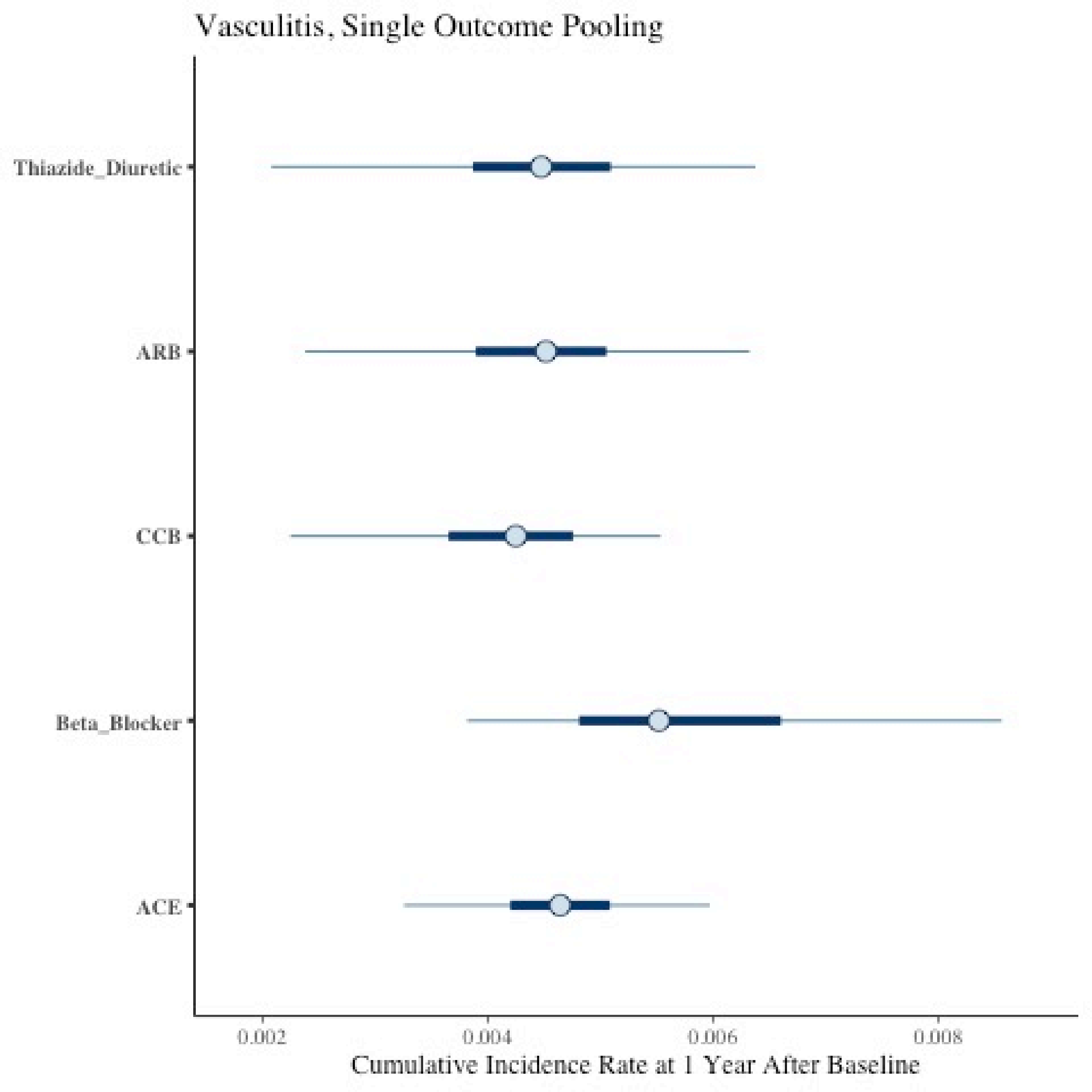

Vasculitis, Single Outcome Pooling
Thiazide_Diuretic
ARB
CCB
Beta_Blocker
ACE
0.002
0.004
0.006
0.008
Cumulative Incidence Rate at 1 Year After Baseline

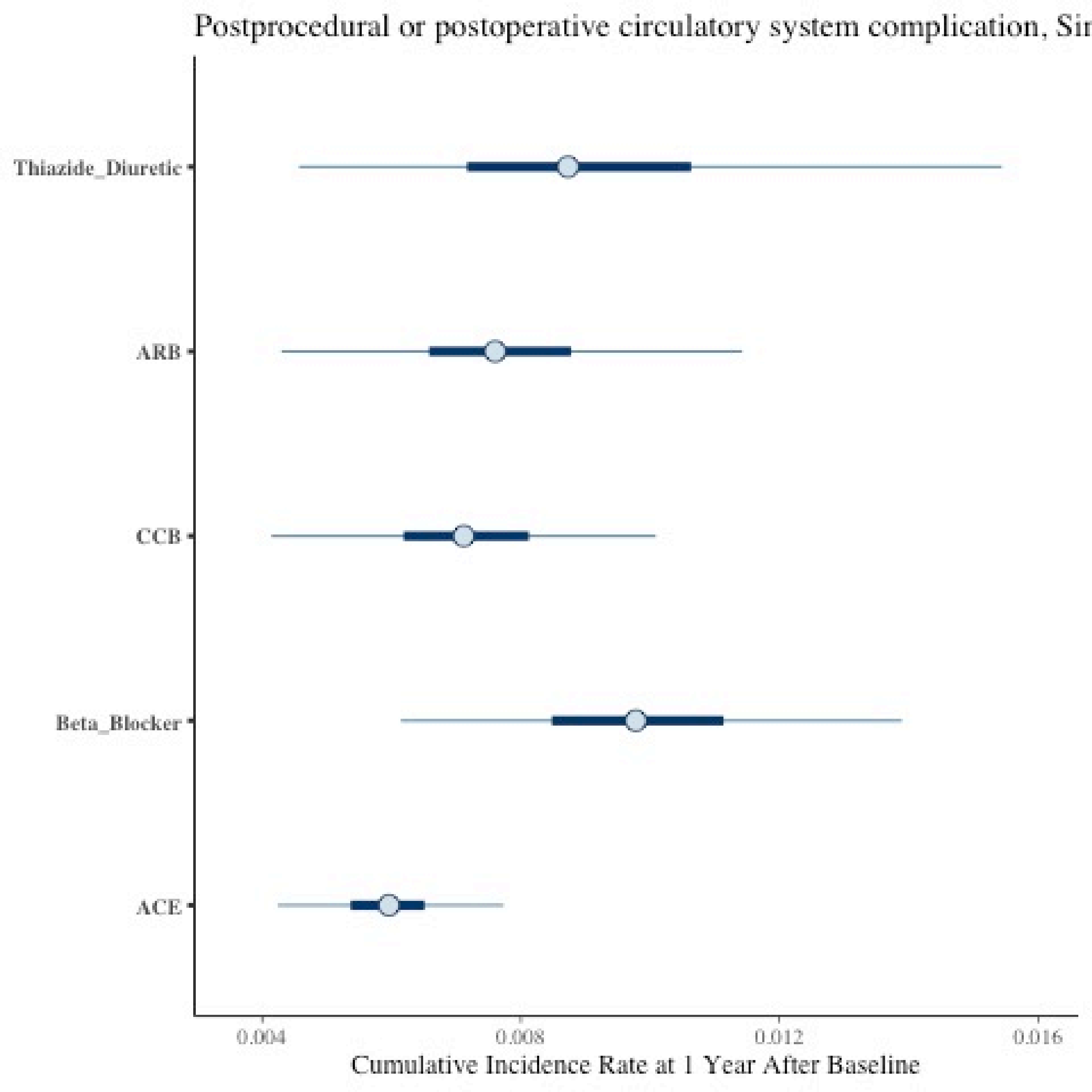

Postprocedural or postoperative circulatory system complication, Si
Thiazide_Diuretic
ARB
CCB
Beta_Blocker
ACE
0.004
0.008
0.012
0.016
Cumulative Incidence Rate at 1 Year After Baseline

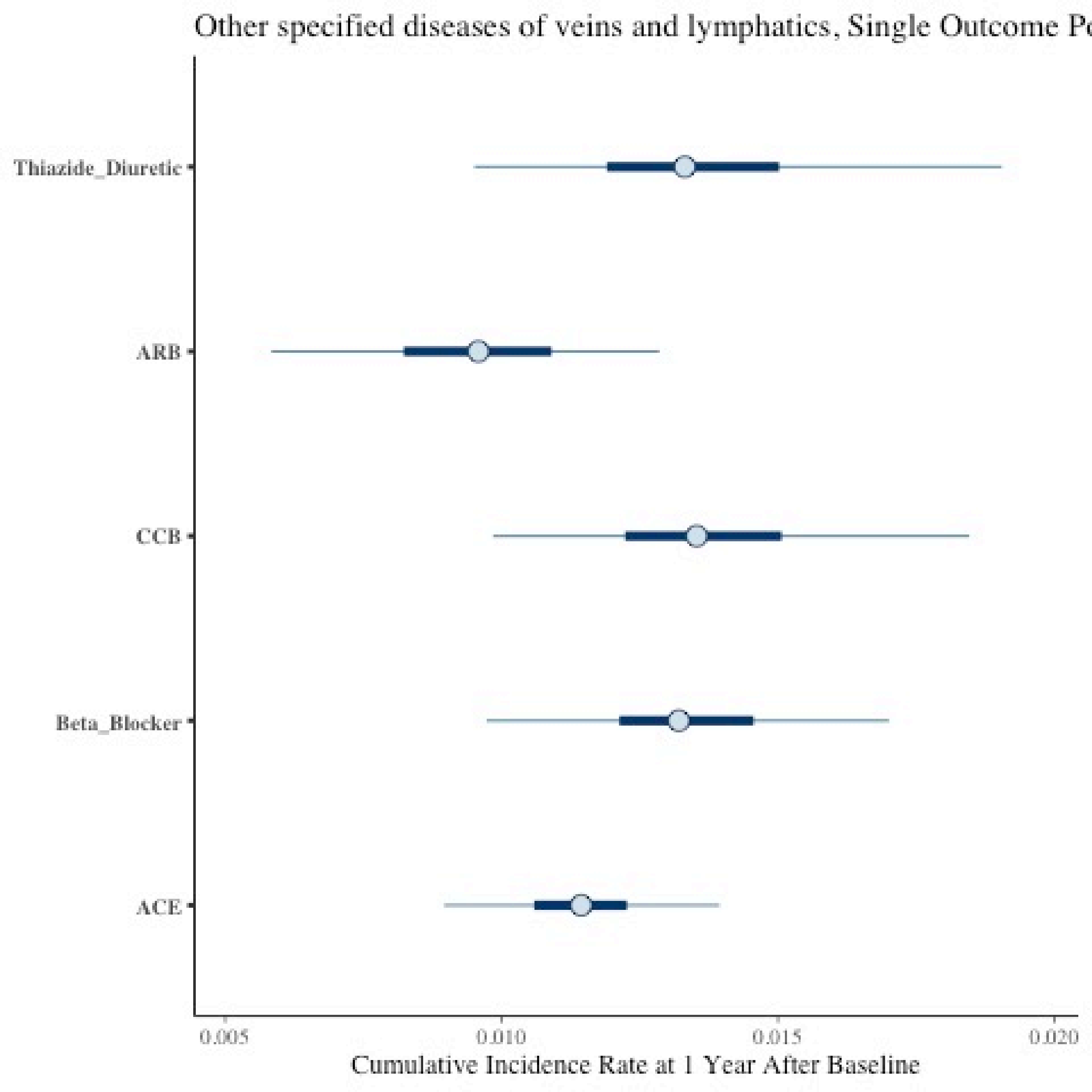
Other specified diseases of veins and lymphatics, Single Outcome Po
Thiazide_Diuretic
ARB
CCB
Beta_Blocker
ACE
0.005
0.010
0.015
0.020
Cumulative Incidence Rate at 1 Year After Baseline

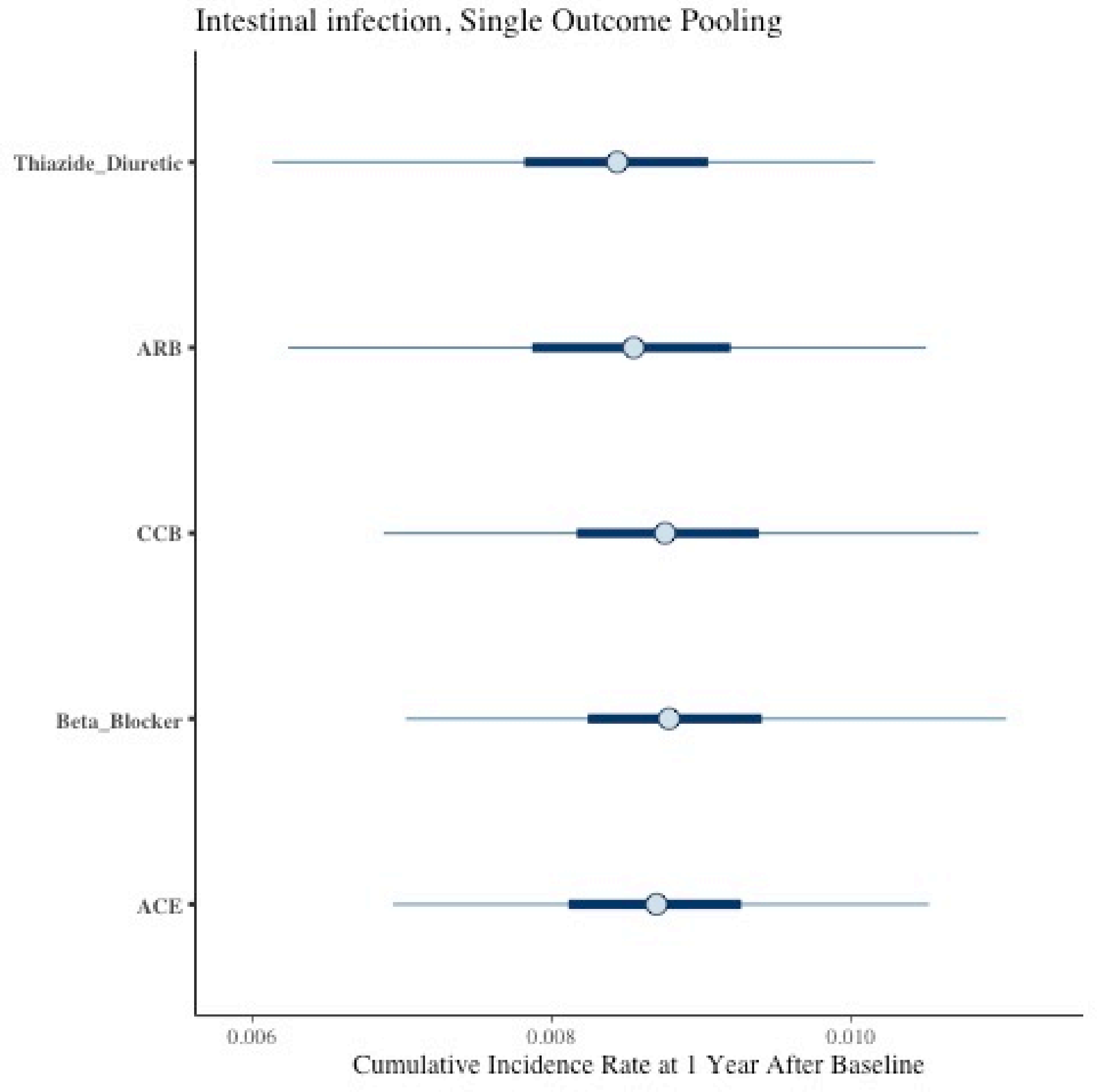
Intestinal infection, Single Outcome Pooling
Thiazide_Diuretic
ARB
CCB
Beta_Blocker
ACE
0.006
0.008
0.010
Cumulative Incidence Rate at 1 Year After Baseline

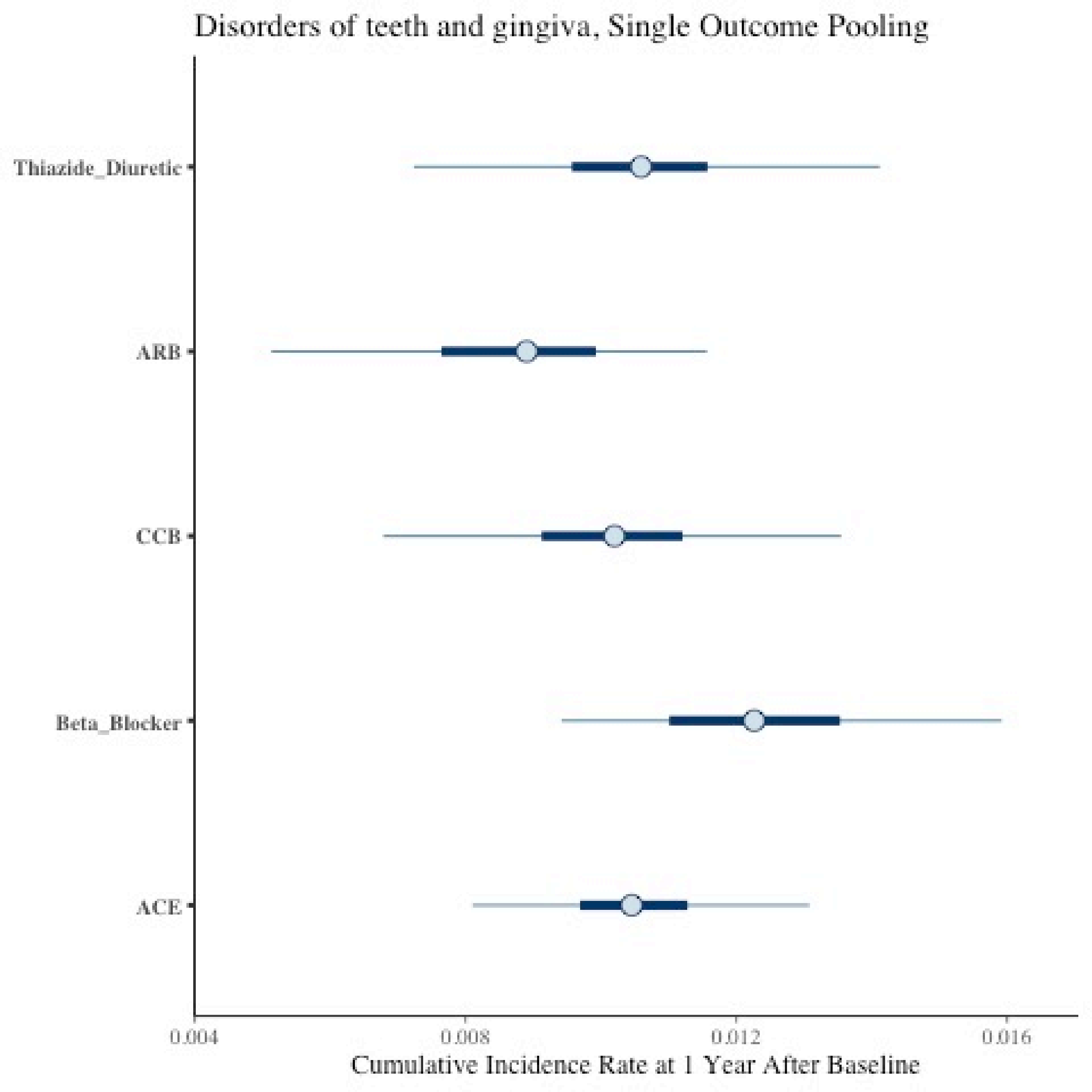
Disorders of teeth and gingiva, Single Outcome Pooling
Thiazide_Diuretic
ARB
CCB
Beta_Blocker
ACE
0.004
0.008
0.012
0.016
Cumulative Incidence Rate at 1 Year After Baseline

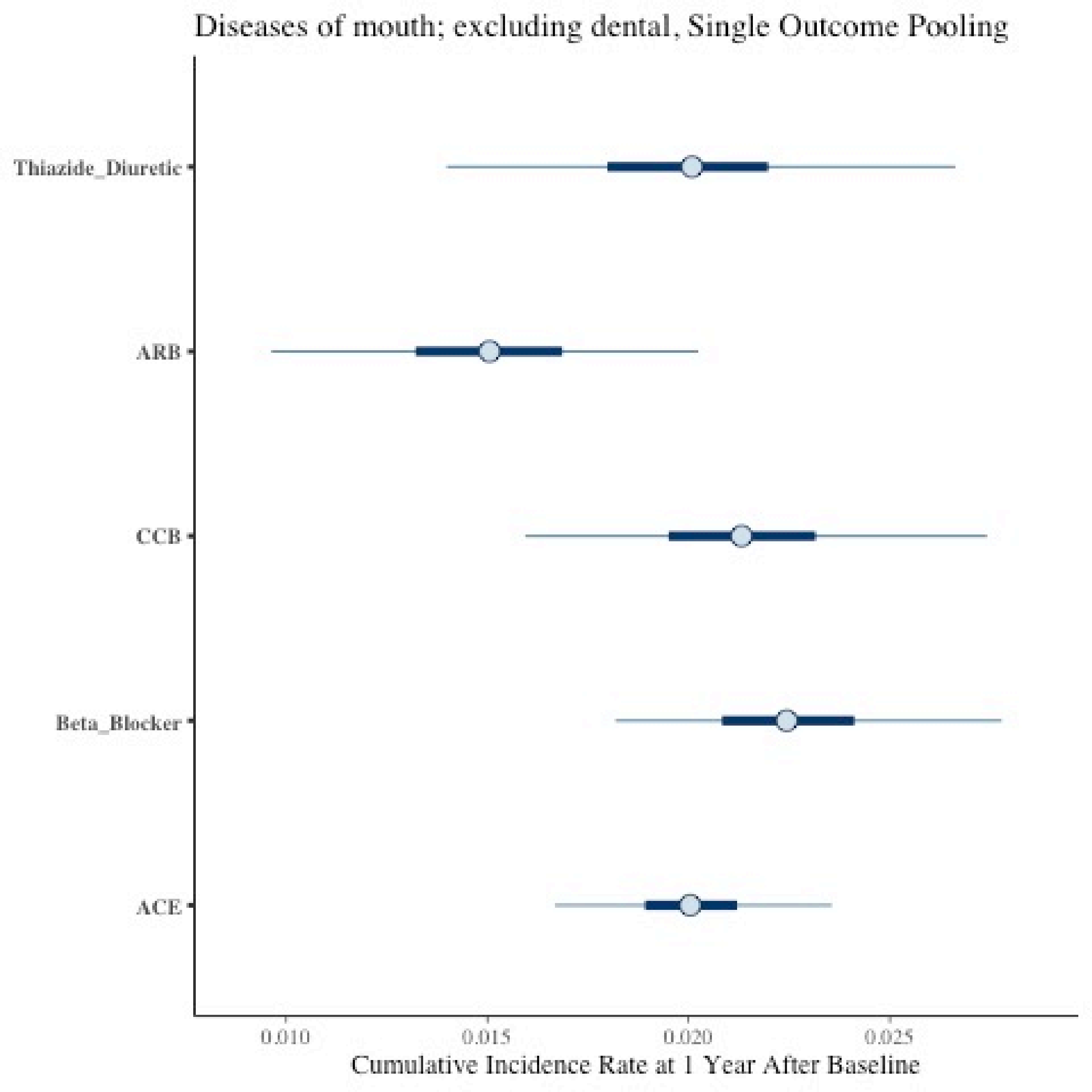
Diseases of mouth; excluding dental, Single Outcome Pooling
Thiazide_Diuretic
ARB
CCB
Beta_Blocker
ACE
0.010
0.015
0.020
0.025
Cumulative Incidence Rate at 1 Year After Baseline

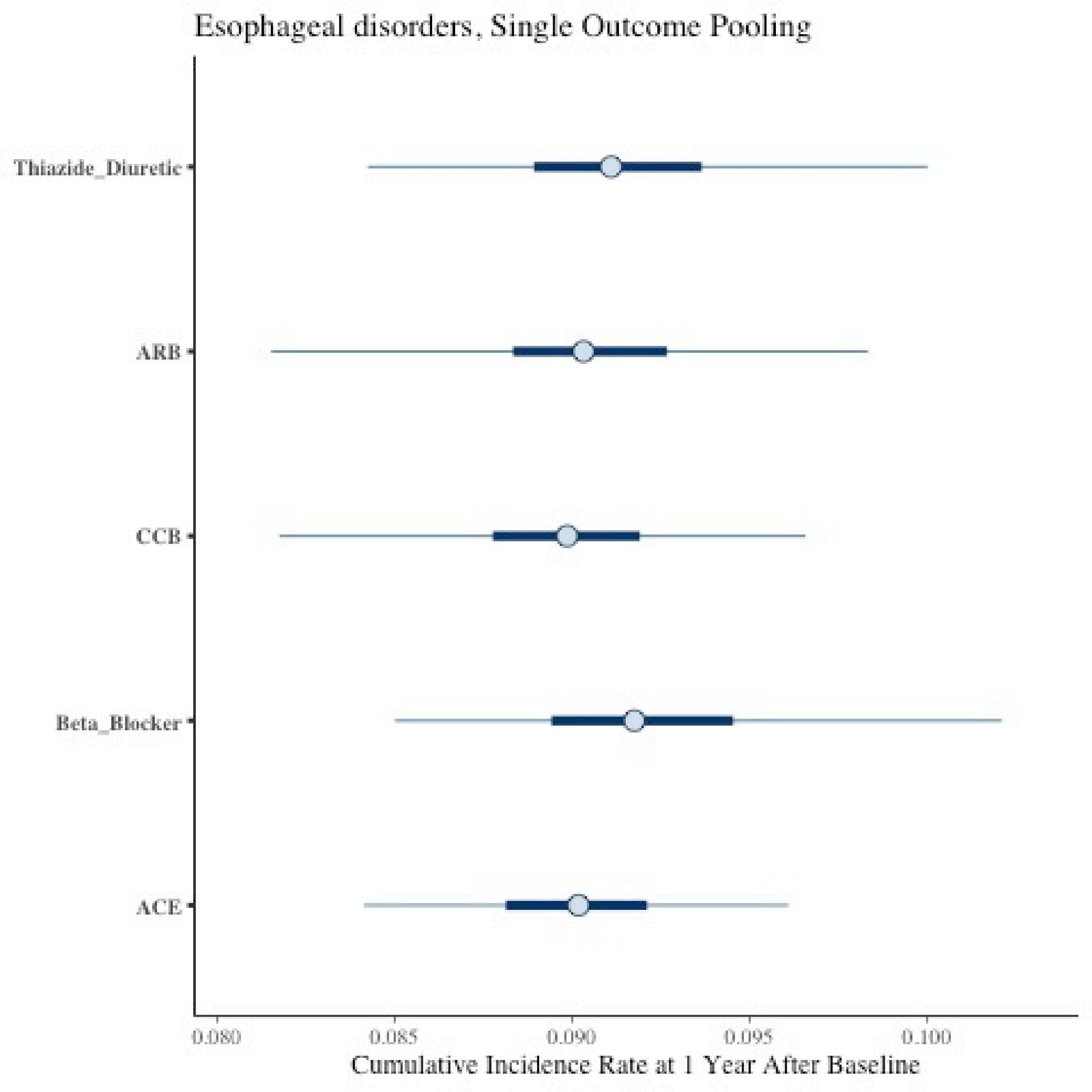

Esophageal disorders, Single Outcome Pooling
Thiazide_Diuretic
ARB
CCB
Beta_Blocker
ACE
0.080
0.085
0.090
0.095
0.100
Cumulative Incidence Rate at 1 Year After Baseline

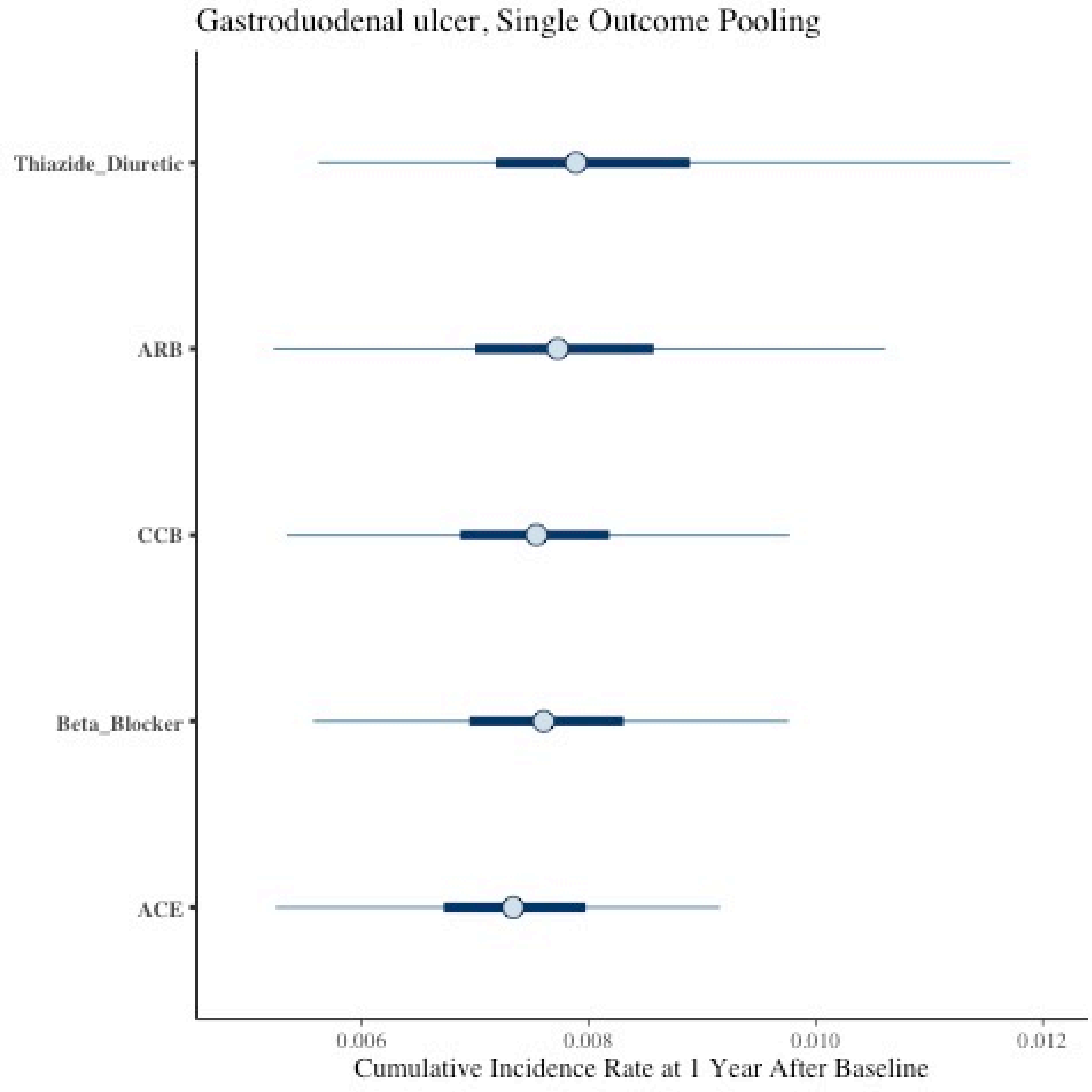
Gastroduodenal ulcer, Single Outcome Pooling
Thiazide_Diuretic
ARB
CCB
Beta_Blocker
ACE
0.006
0.008
0.010
0.012
Cumulative Incidence Rate at 1 Year After Baseline

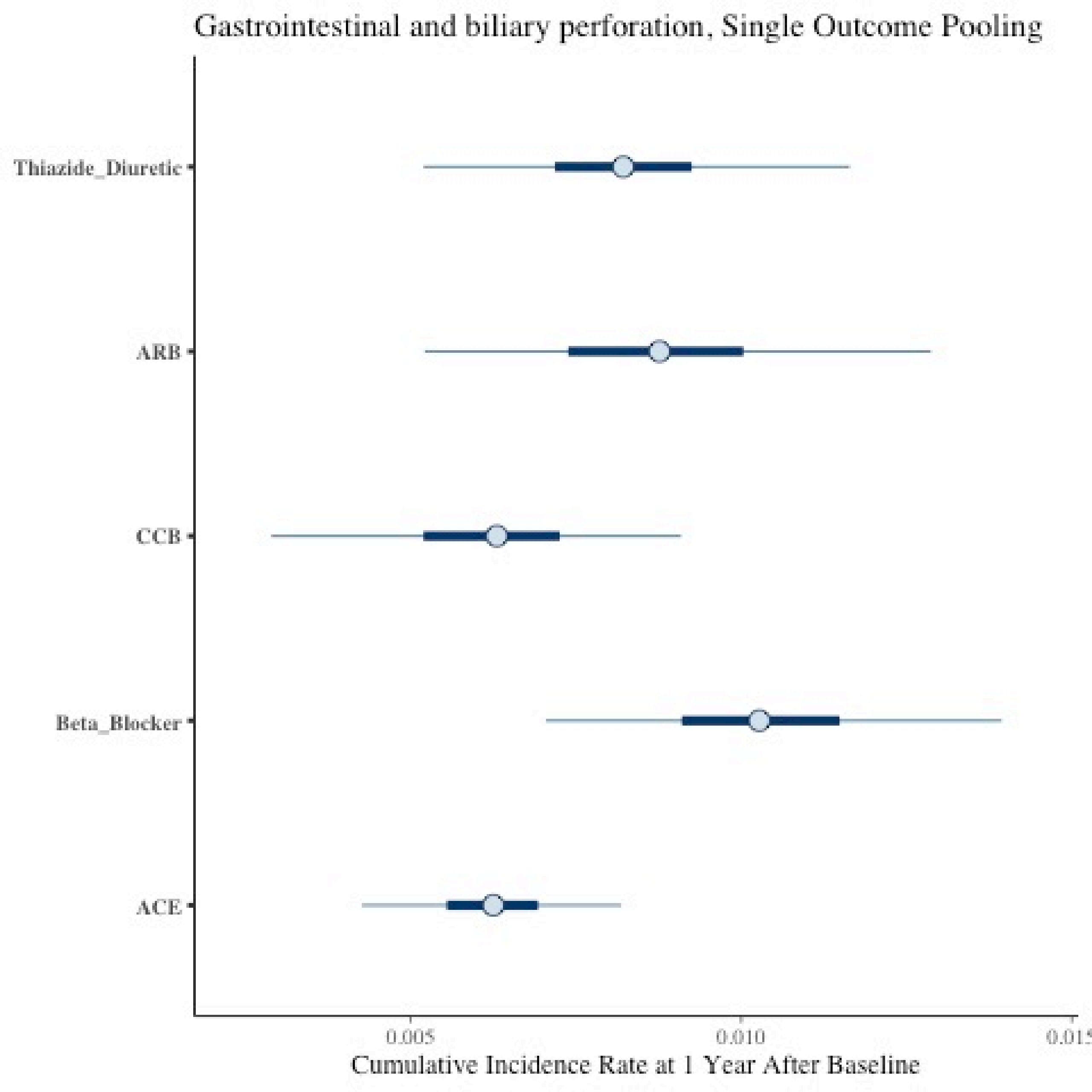

Gastrointestinal and biliary perforation, Single Outcome Pooling
Thiazide_Diuretic
ARB
CCB
Beta_Blocker
ACE
0.005
0.010
0.015
Cumulative Incidence Rate at 1 Year After Baseline

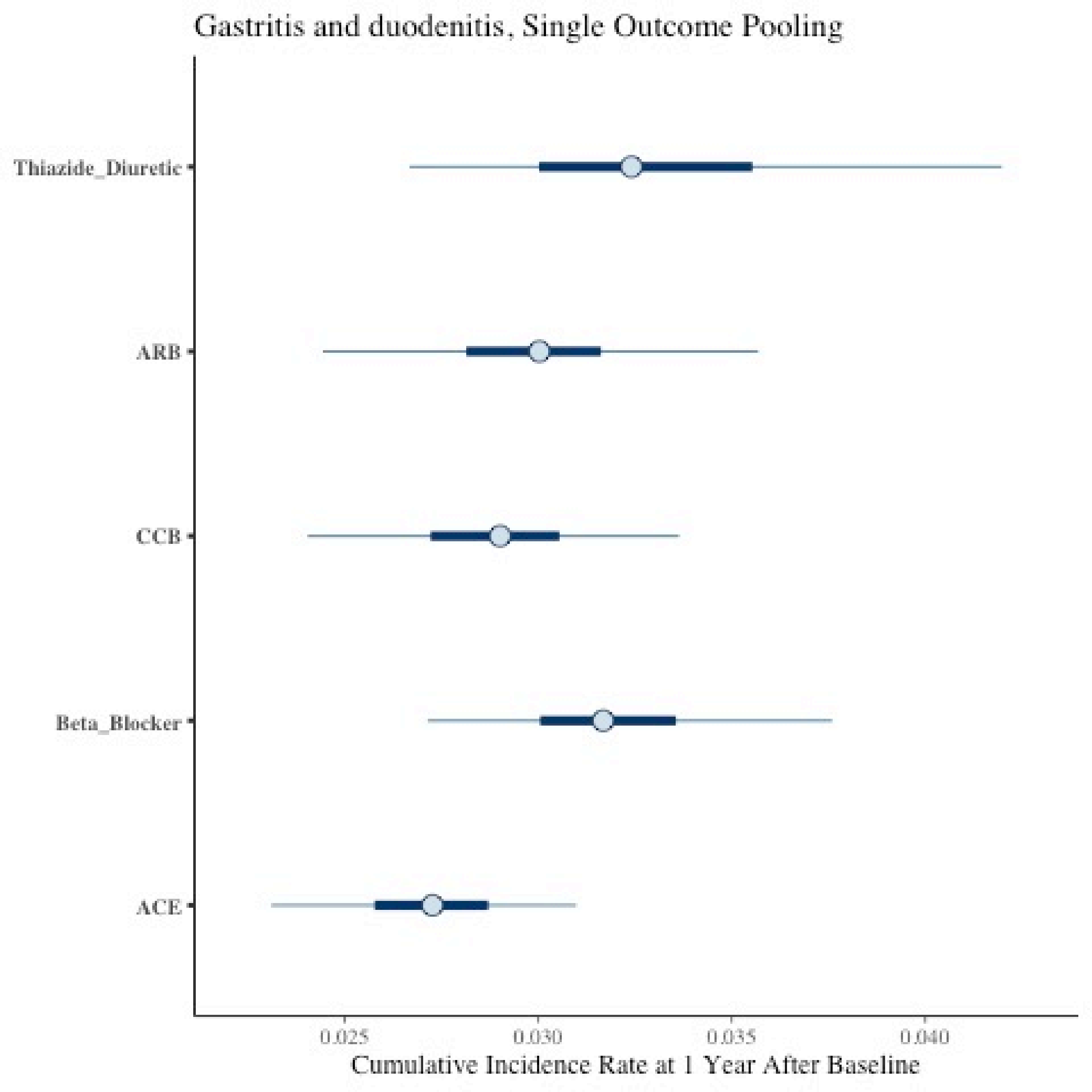
Gastritis and duodenitis, Single Outcome Pooling
Thiazide_Diuretic
ARB
CCB
Beta_Blocker
ACE
0.025
0.030
0.035
0.040
Cumulative Incidence Rate at 1 Year After Baseline

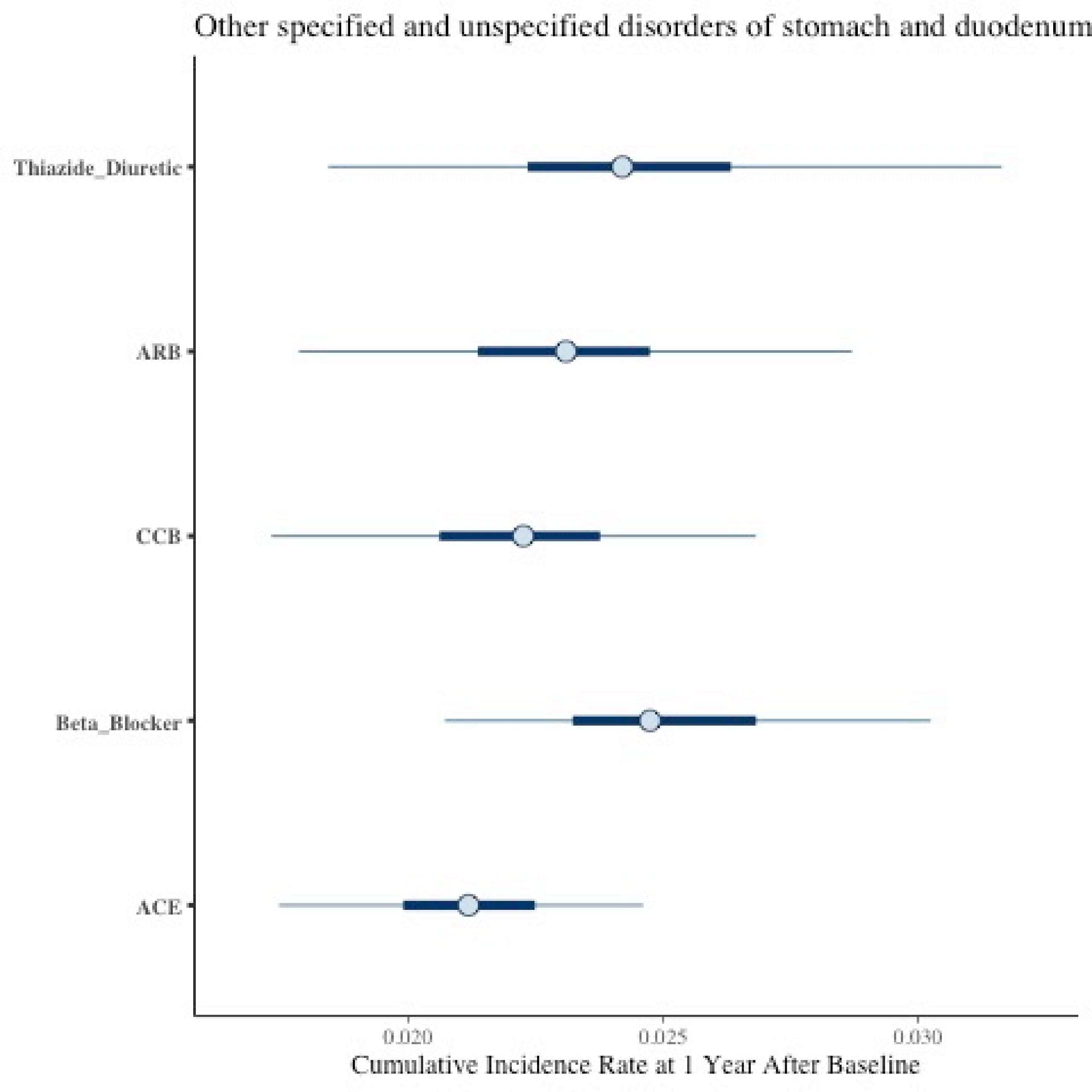

Other specified and unspecified disorders of stomach and duodenum
Thiazide_Diuretic
ARB
CCB
Beta_Blocker
ACE
0.020
0.025
0.030
Cumulative Incidence Rate at 1 Year After Baseline

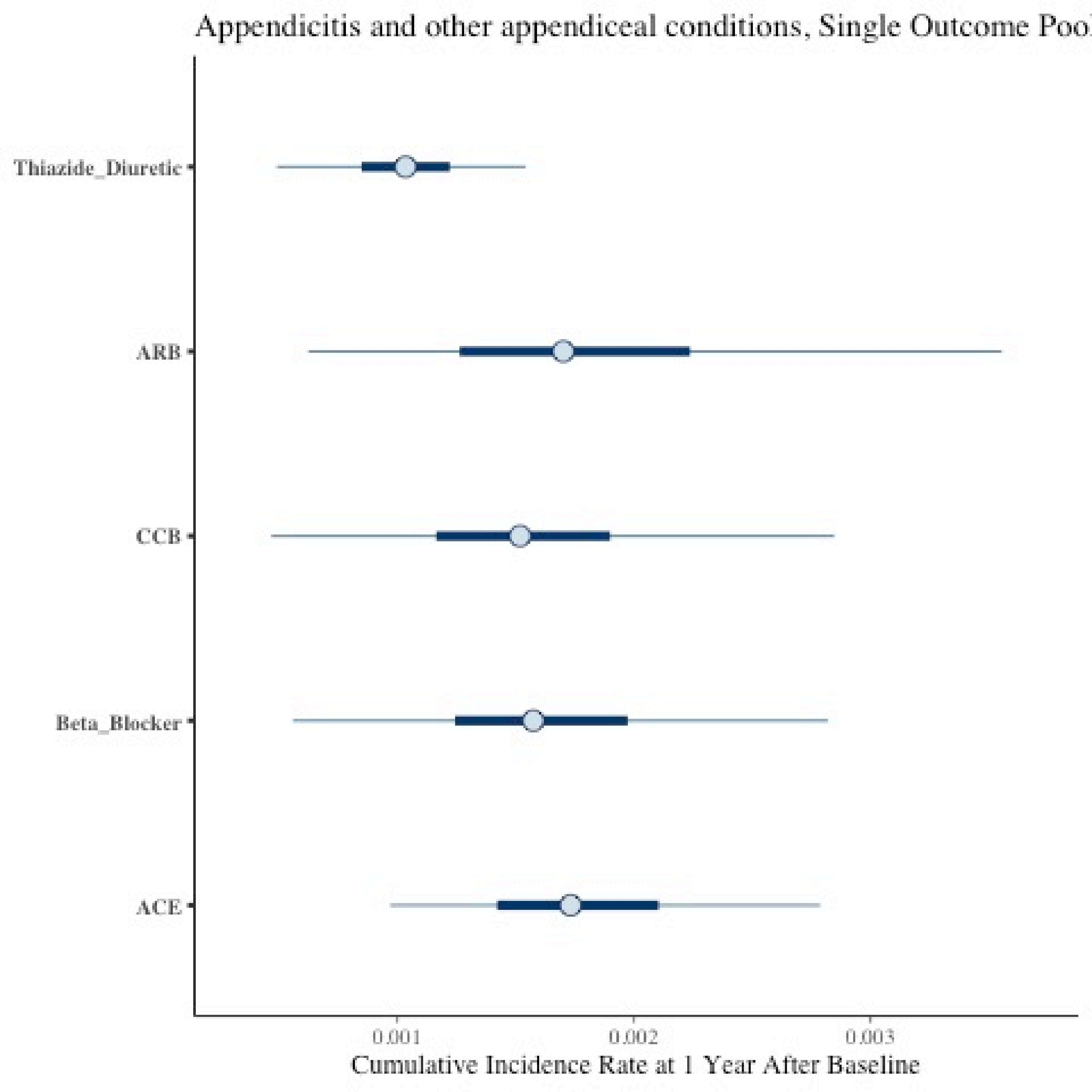

Appendicitis and other appendiceal conditions, Single Outcome Poo
Thiazide_Diuretic
ARB
CCB
Beta_Blocker
ACE
0.001
0.002
0.003
Cumulative Incidence Rate at 1 Year After Baseline

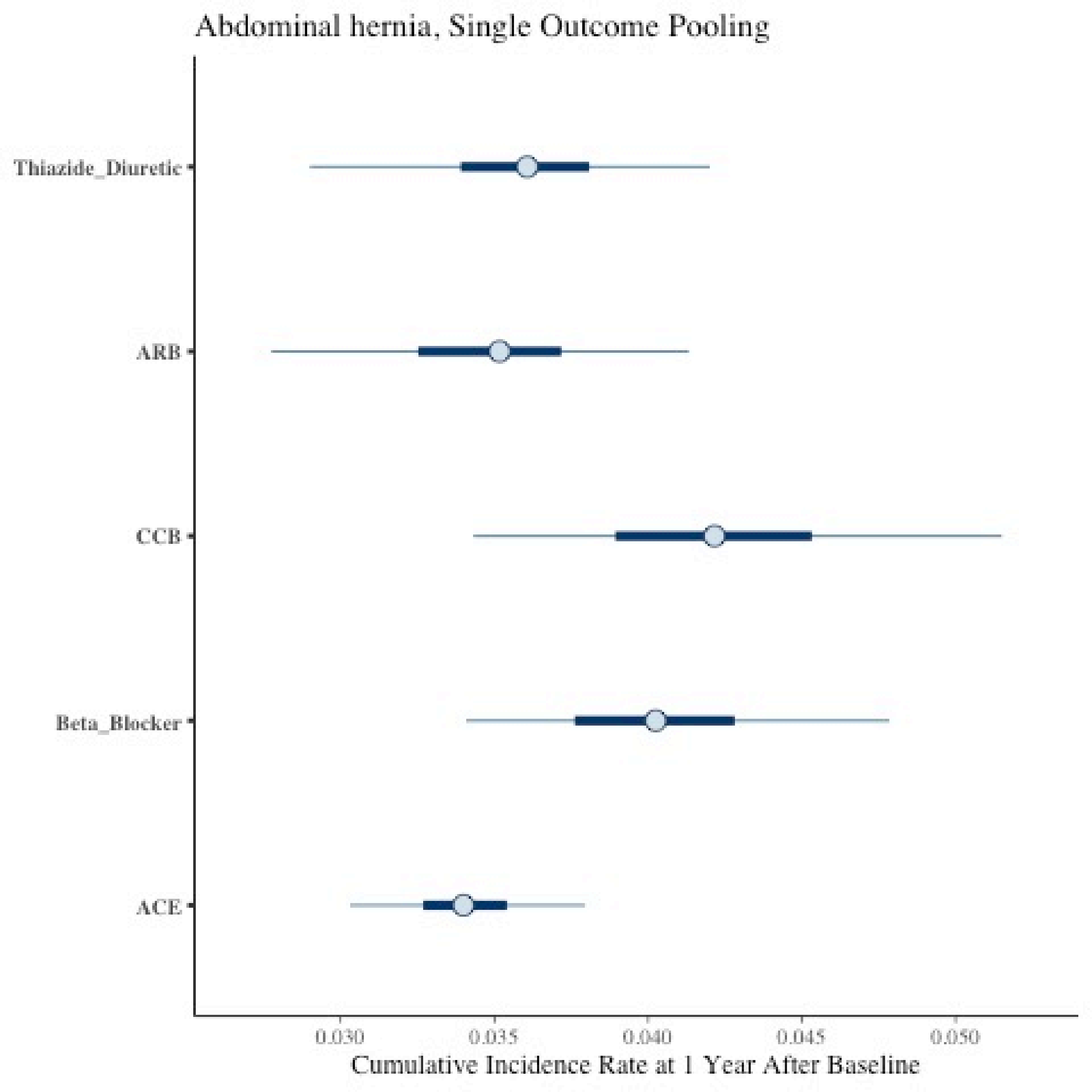

Abdominal hernia, Single Outcome Pooling
Thiazide_Diuretic
ARB
CCB
Beta_Blocker
ACE
0.030
0.035
0.040
0.045
0.050
Cumulative Incidence Rate at 1 Year After Baseline

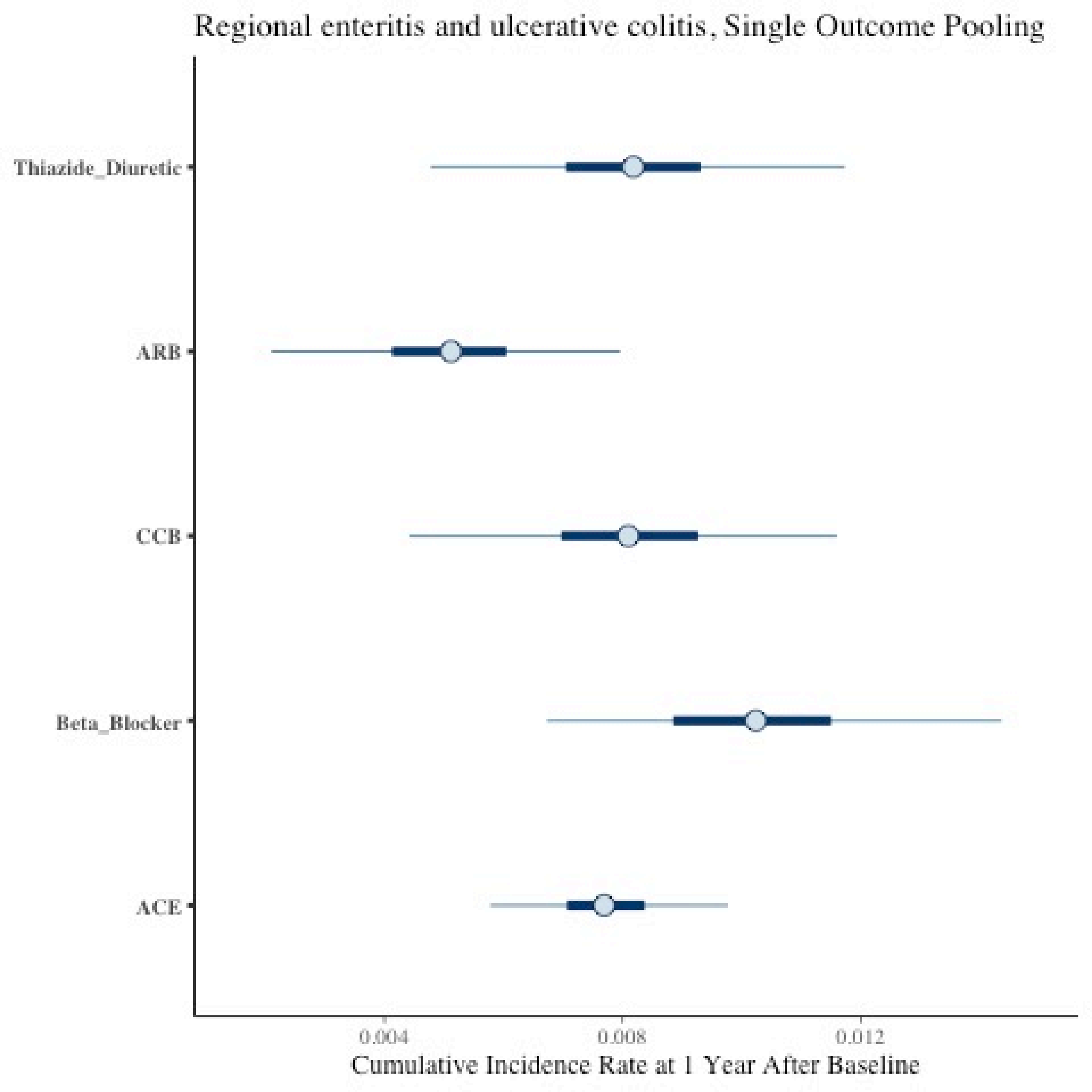
Regional enteritis and ulcerative colitis, Single Outcome Pooling
Thiazide_Diuretic
ARB
CCB
Beta_Blocker
ACE
0.004
0.008
0.012
Cumulative Incidence Rate at 1 Year After Baseline

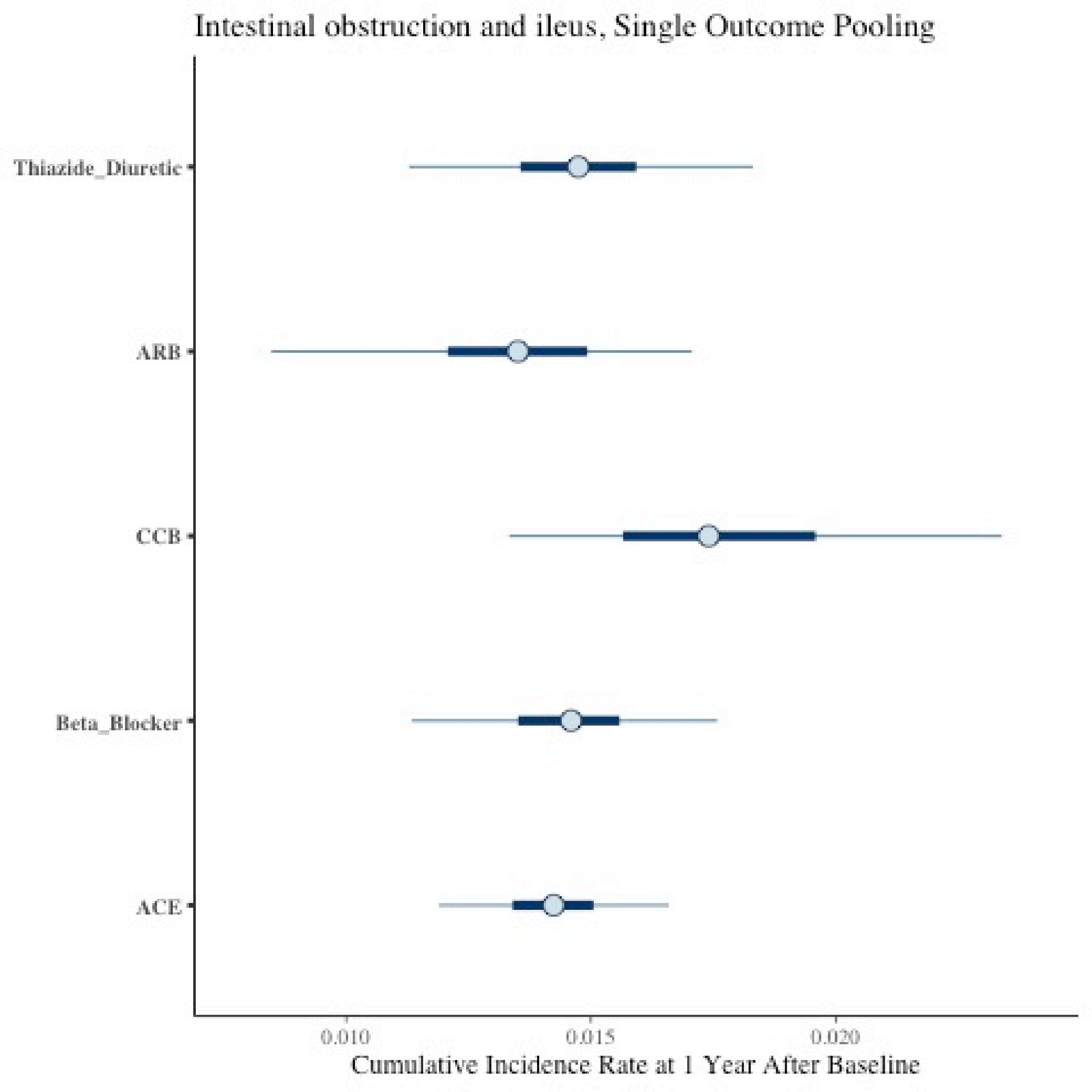

Intestinal obstruction and ileus, Single Outcome Pooling
Thiazide_Diuretic
ARB
CCB
Beta_Blocker
ACE
0.010
0.015
0.020
Cumulative Incidence Rate at 1 Year After Baseline

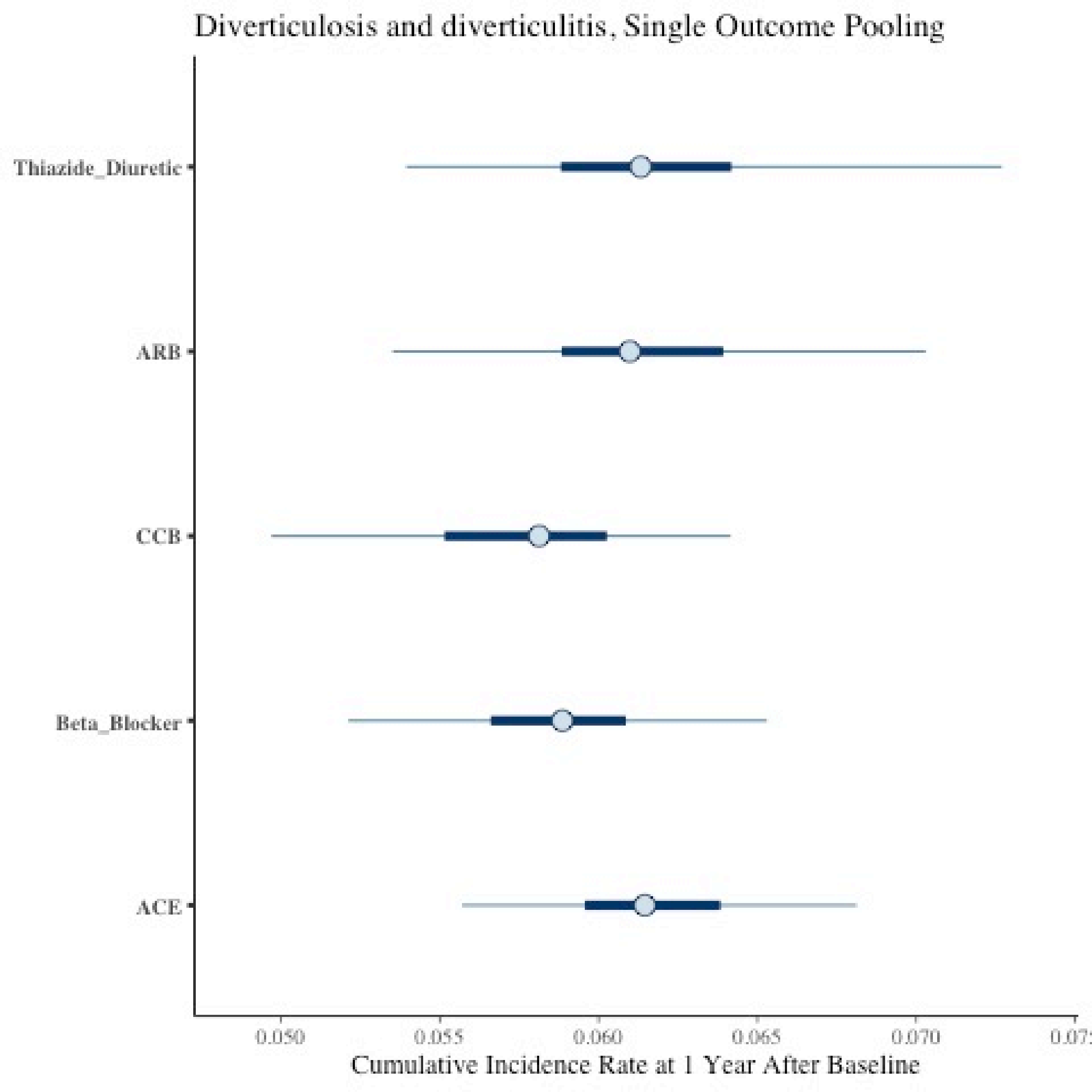

Diverticulosis and diverticulitis, Single Outcome Pooling
Thiazide_Diuretic
ARB
CCB
Beta_Blocker
ACE
0.050
0.055
0.060
0.065
0.070
Cumulative Incidence Rate at 1 Year After Baseline

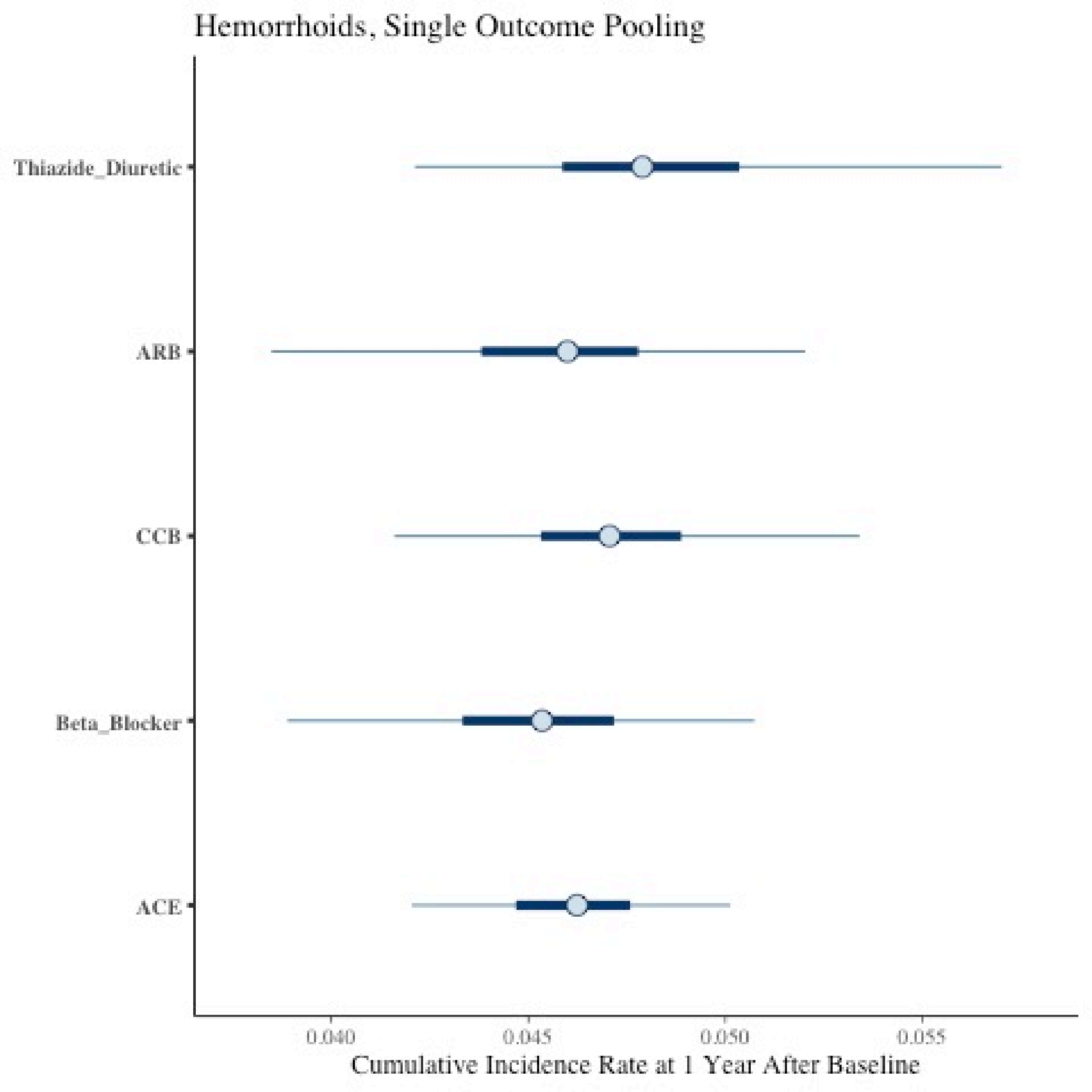

Hemorrhoids, Single Outcome Pooling
Thiazide_Diuretic
ARB
CCB
Beta_Blocker
ACE
0.040
0.045
0.050
0.055
Cumulative Incidence Rate at 1 Year After Baseline

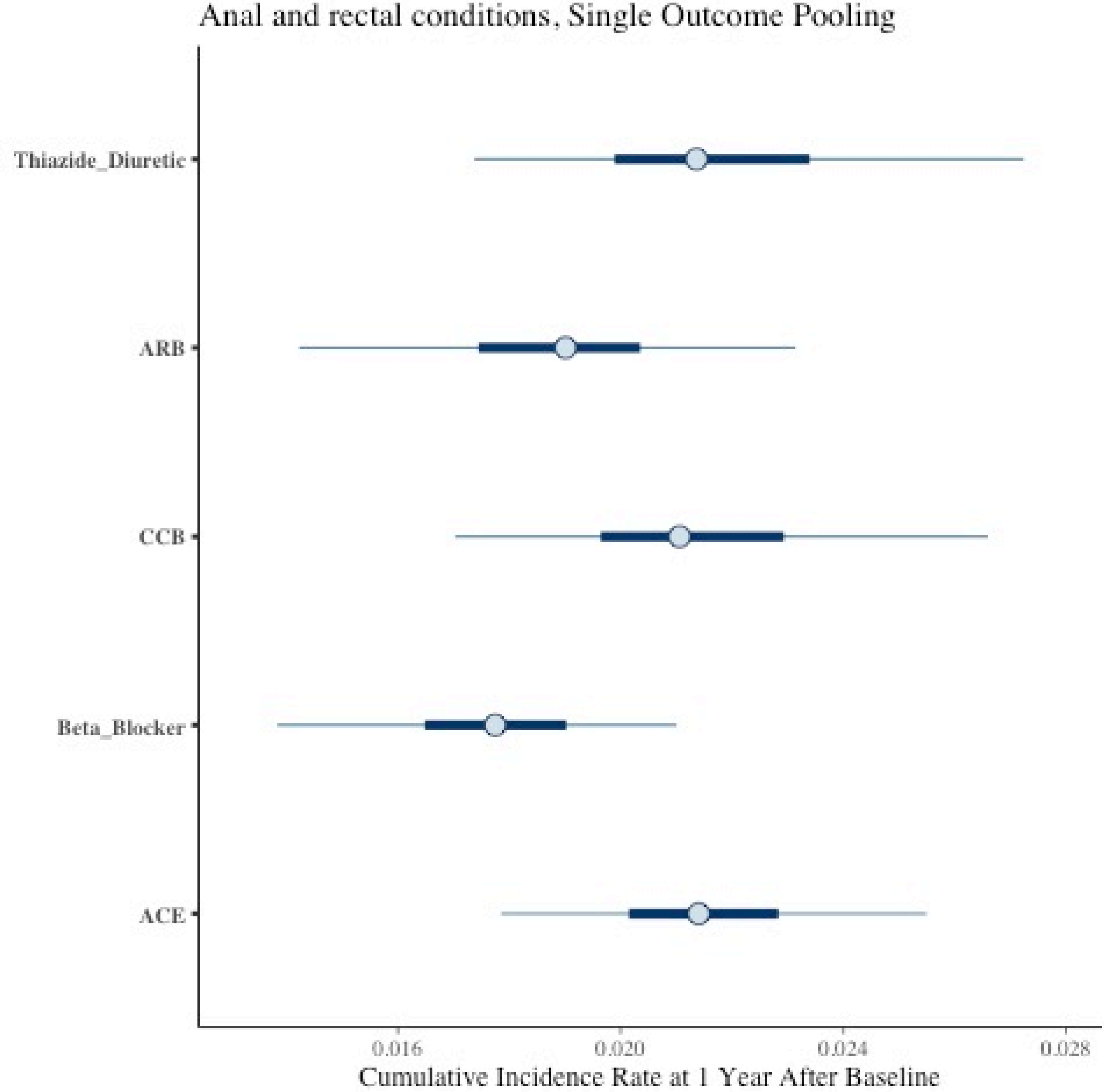

Anal and rectal conditions, Single Outcome Pooling
Thiazide_Diuretic
ARB
CCB
Beta_Blocker
ACE
0.016
0.020
0.024
0.028
Cumulative Incidence Rate at 1 Year After Baseline

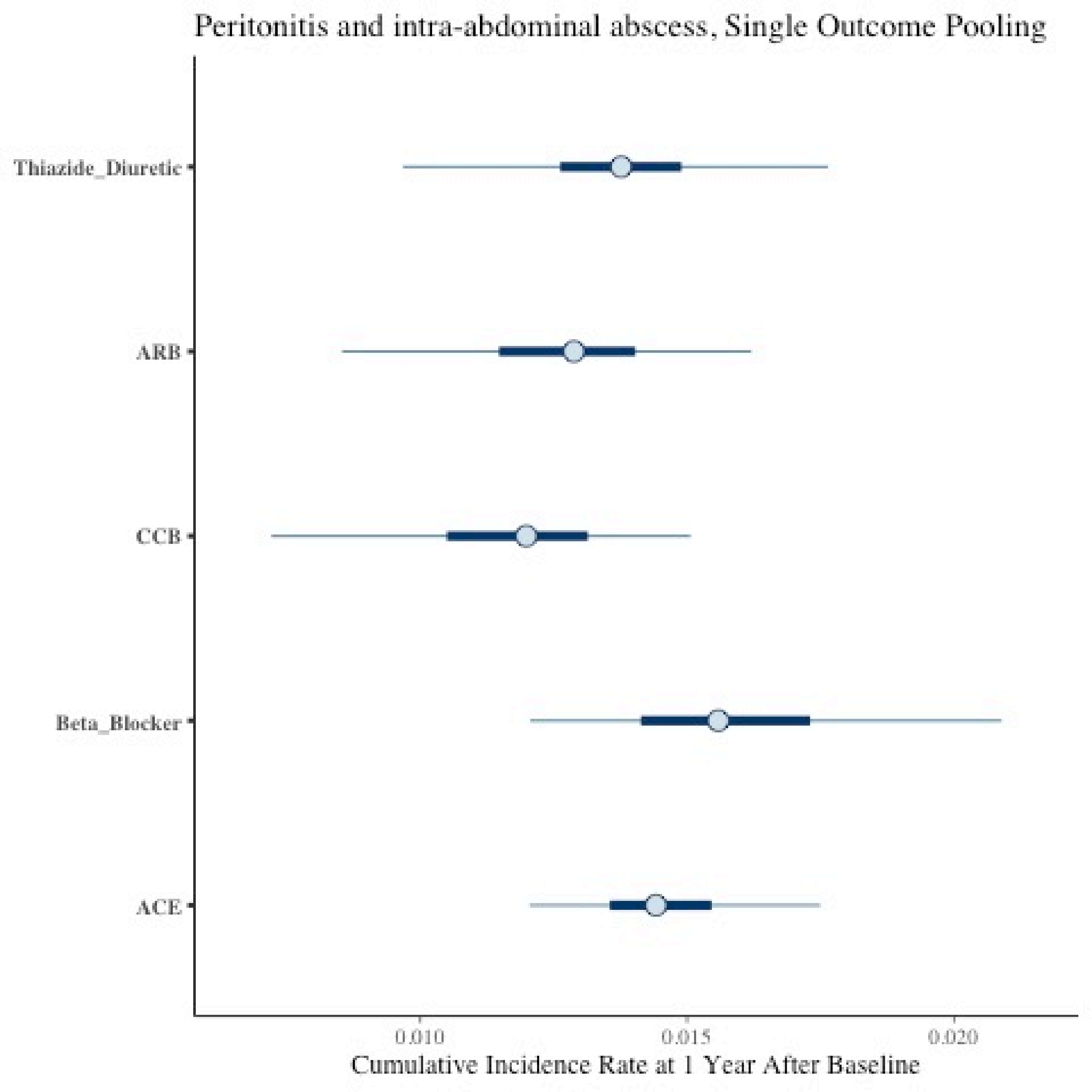

Peritonitis and intra-abdominal abscess, Single Outcome Pooling
Thiazide_Diuretic
ARB
CCB
Beta_Blocker
ACE
0.010
0.015
0.020
Cumulative Incidence Rate at 1 Year After Baseline

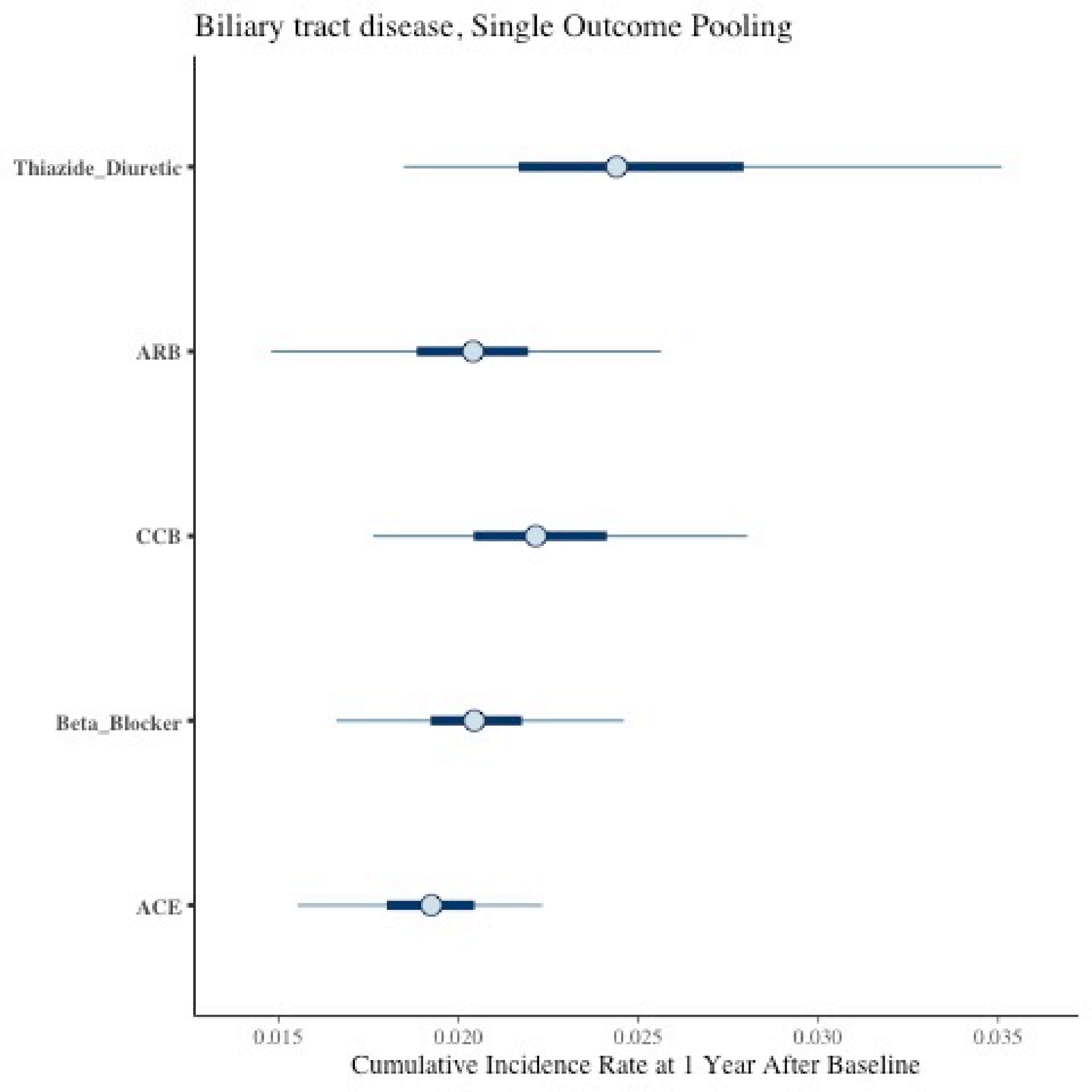

Biliary tract disease, Single Outcome Pooling
Thiazide_Diuretic
ARB
CCB
Beta_Blocker
ACE
0.015
0.020
0.025
0.030
0.035
Cumulative Incidence Rate at 1 Year After Baseline

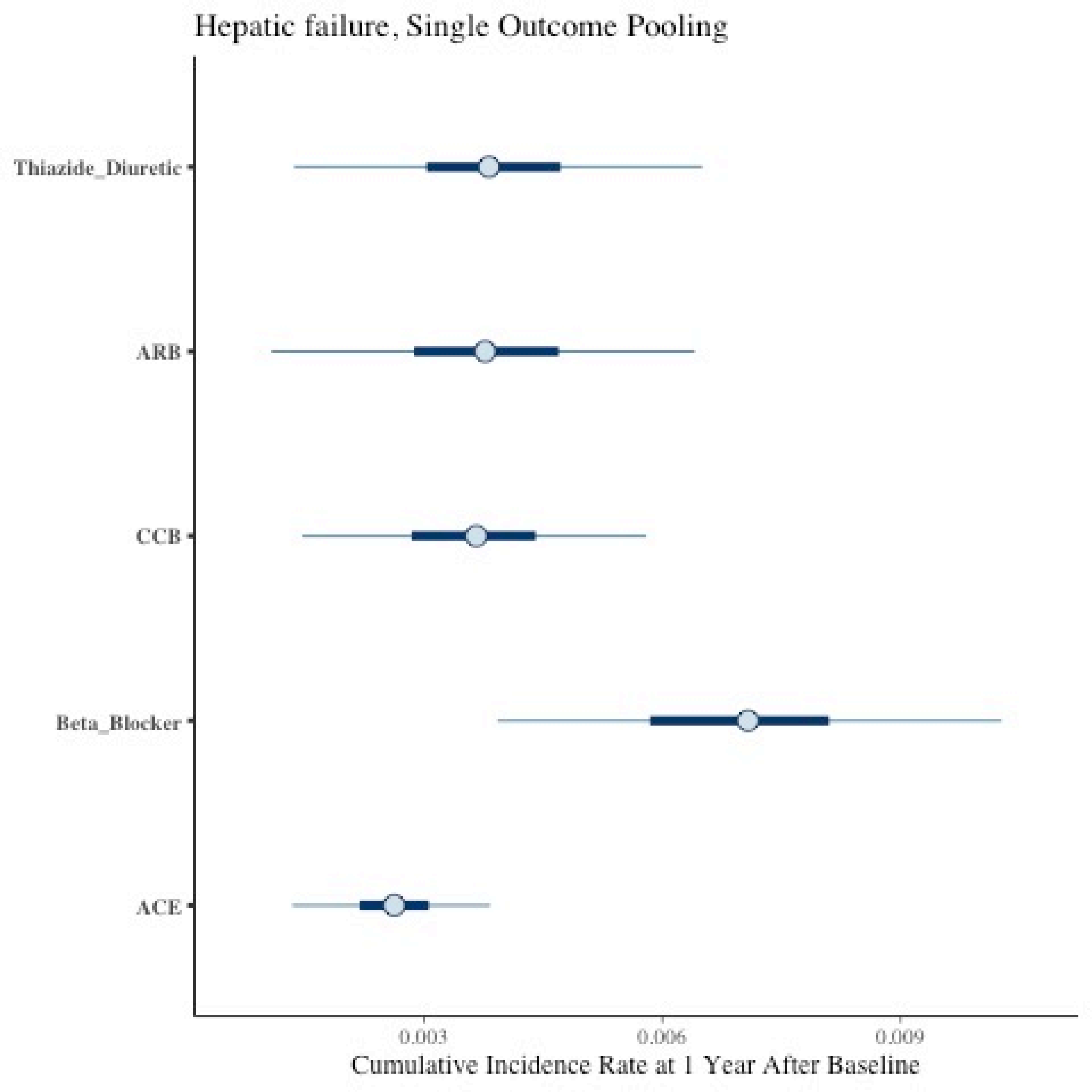
Hepatic failure, Single Outcome Pooling
Thiazide_Diuretic
ARB
CCB
Beta_Blocker
ACE
0.003
0.006
0.009
Cumulative Incidence Rate at 1 Year After Baseline

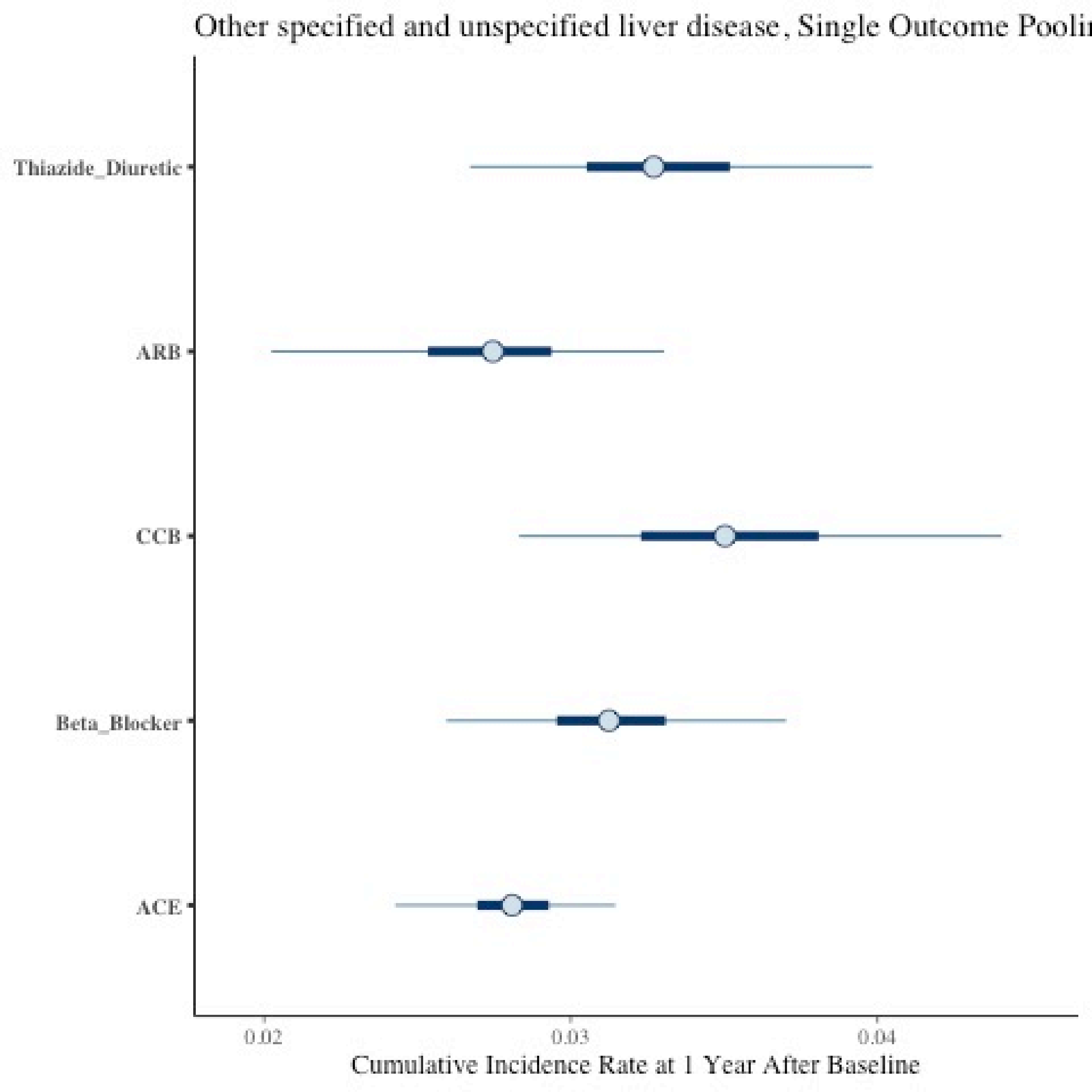

Other specified and unspecified liver disease, Single Outcome Pooli
Thiazide_Diuretic
ARB
CCB
Beta_Blocker
ACE
0.02
0.03
0.04
Cumulative Incidence Rate at 1 Year After Baseline

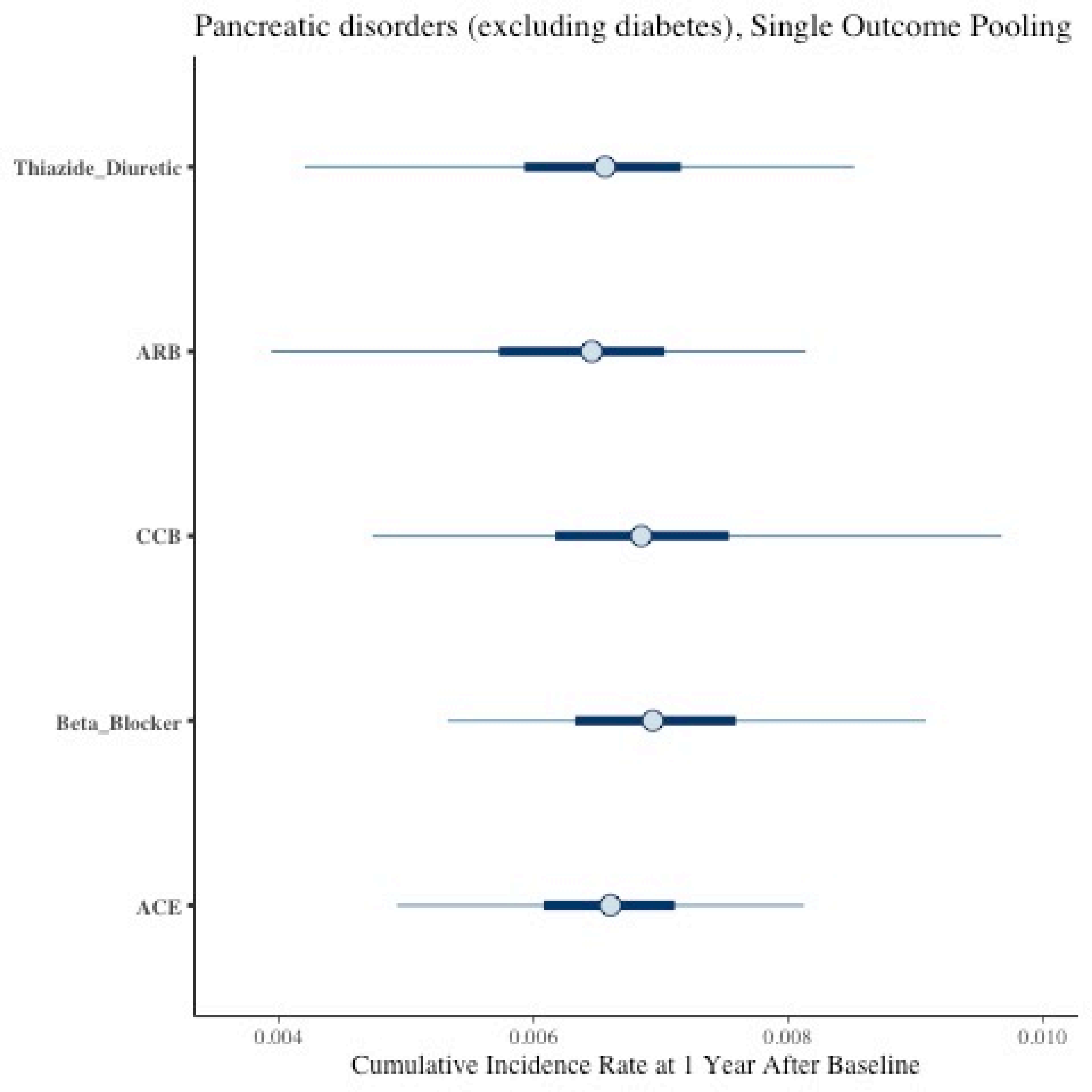

Pancreatic disorders (excluding diabetes), Single Outcome Pooling
Thiazide_Diuretic
ARB
CCB
Beta_Blocker
ACE
0.004
0.006
0.008
0.010
Cumulative Incidence Rate at 1 Year After Baseline

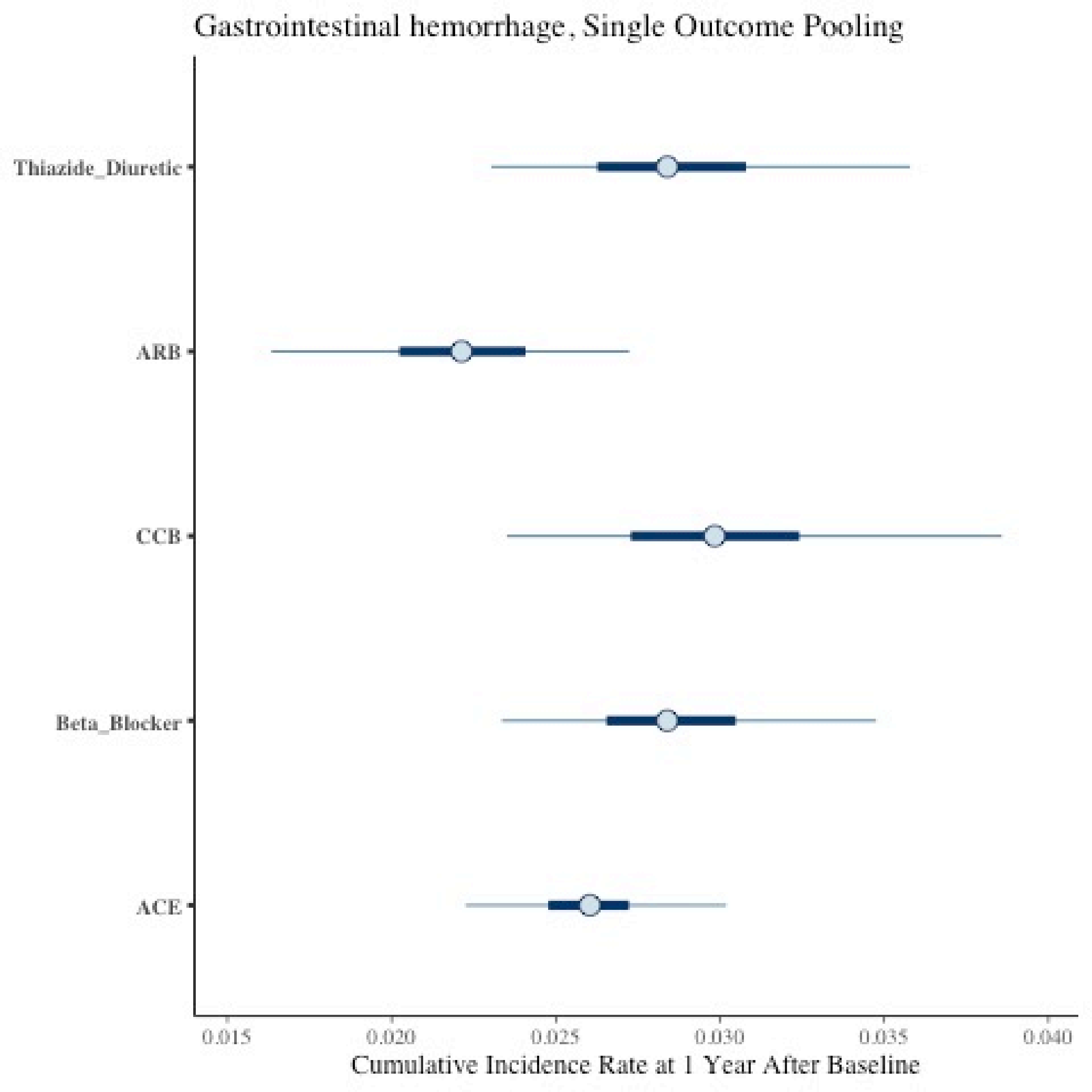
Gastrointestinal hemorrhage, Single Outcome Pooling
Thiazide_Diuretic
ARB
CCB
Beta_Blocker
ACE
0.015
0.020
0.025
0.030
0.035
0.040
Cumulative Incidence Rate at 1 Year After Baseline

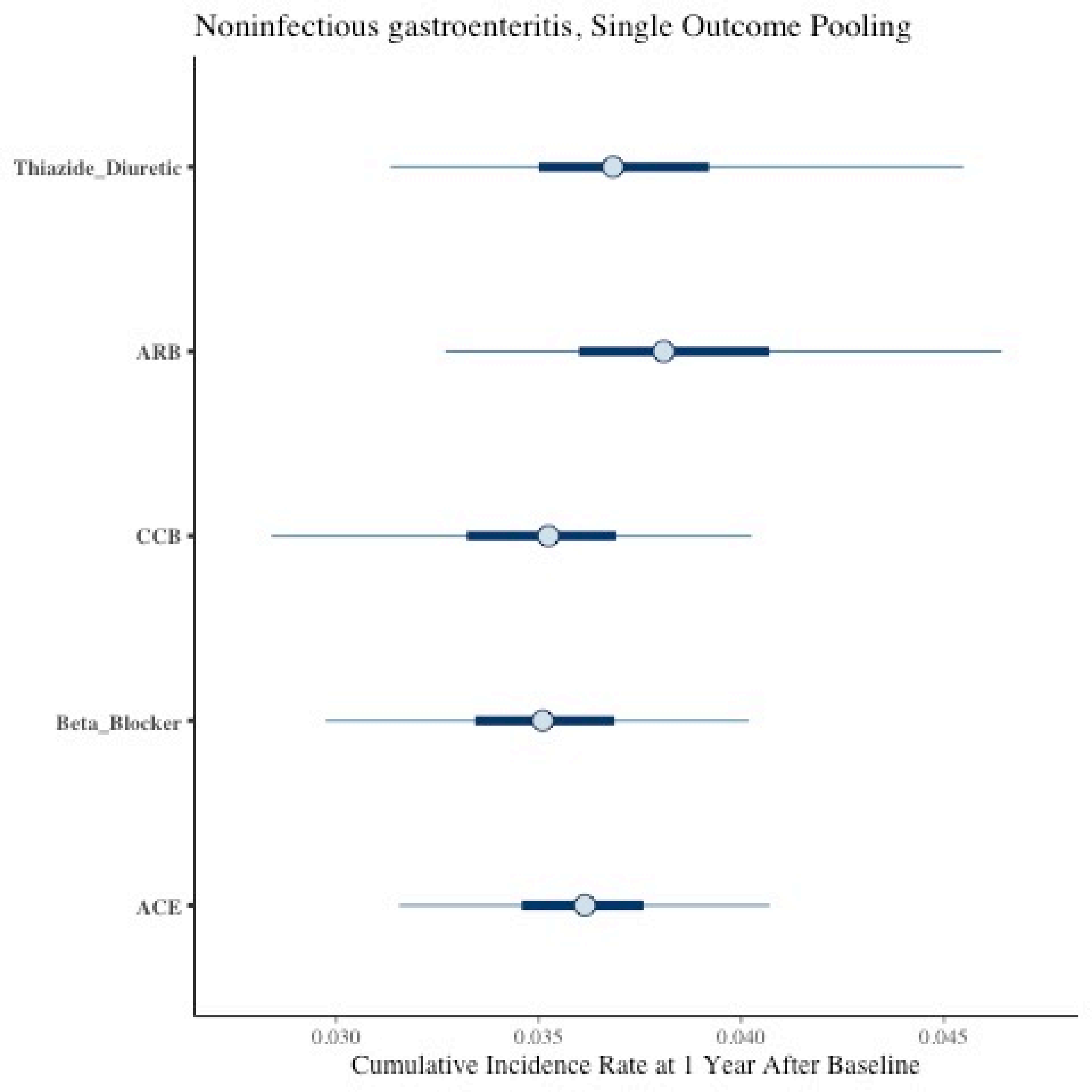

Noninfectious gastroenteritis, Single Outcome Pooling
Thiazide_Diuretic
ARB
CCB
Beta_Blocker
ACE
0.030
0.035
0.040
0.045
Cumulative Incidence Rate at 1 Year After Baseline

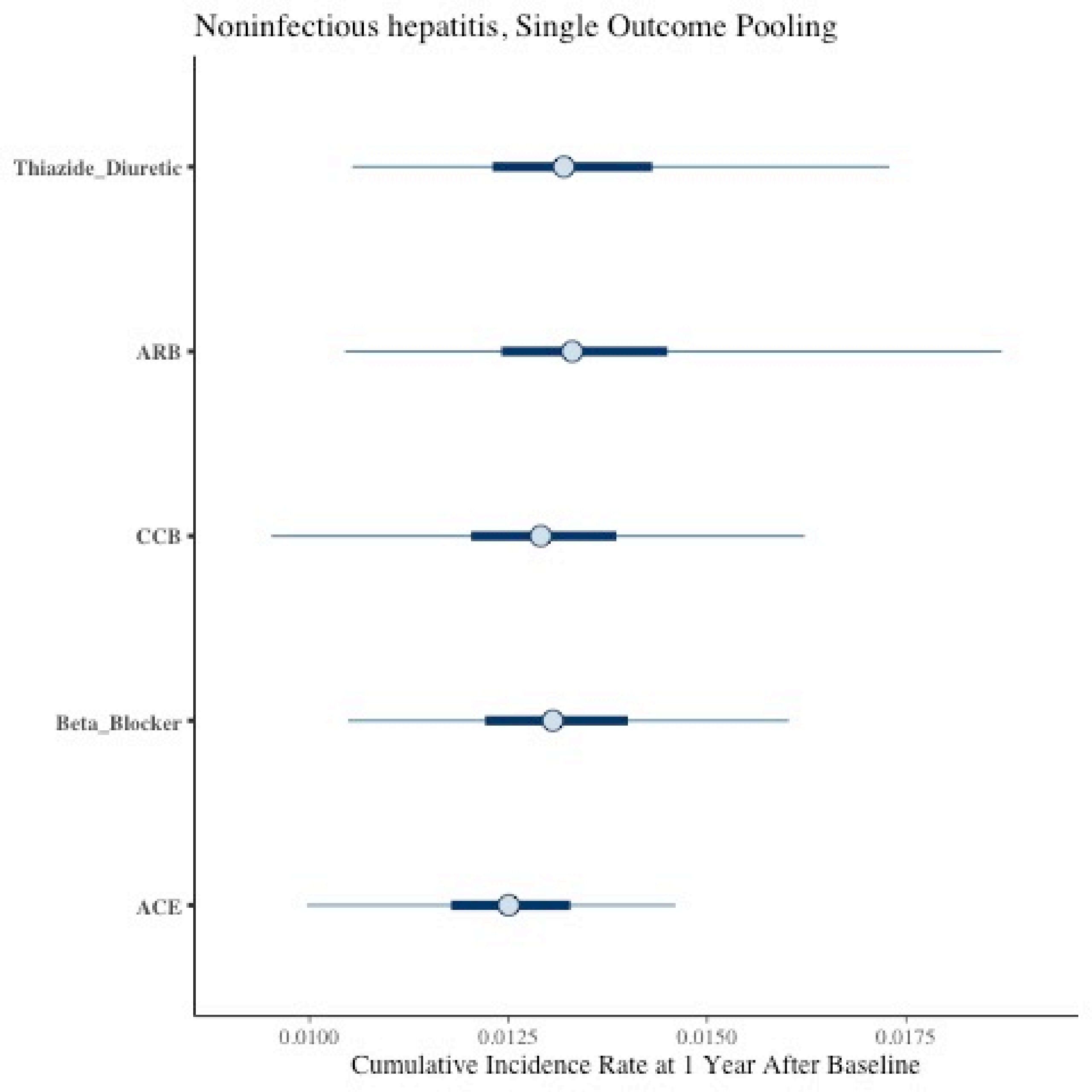
Noninfectious hepatitis, Single Outcome Pooling
Thiazide_Diuretic
ARB
CCB
Beta_Blocker
ACE
0.0100
0.0125
0.0150
0.0175
Cumulative Incidence Rate at 1 Year After Baseline

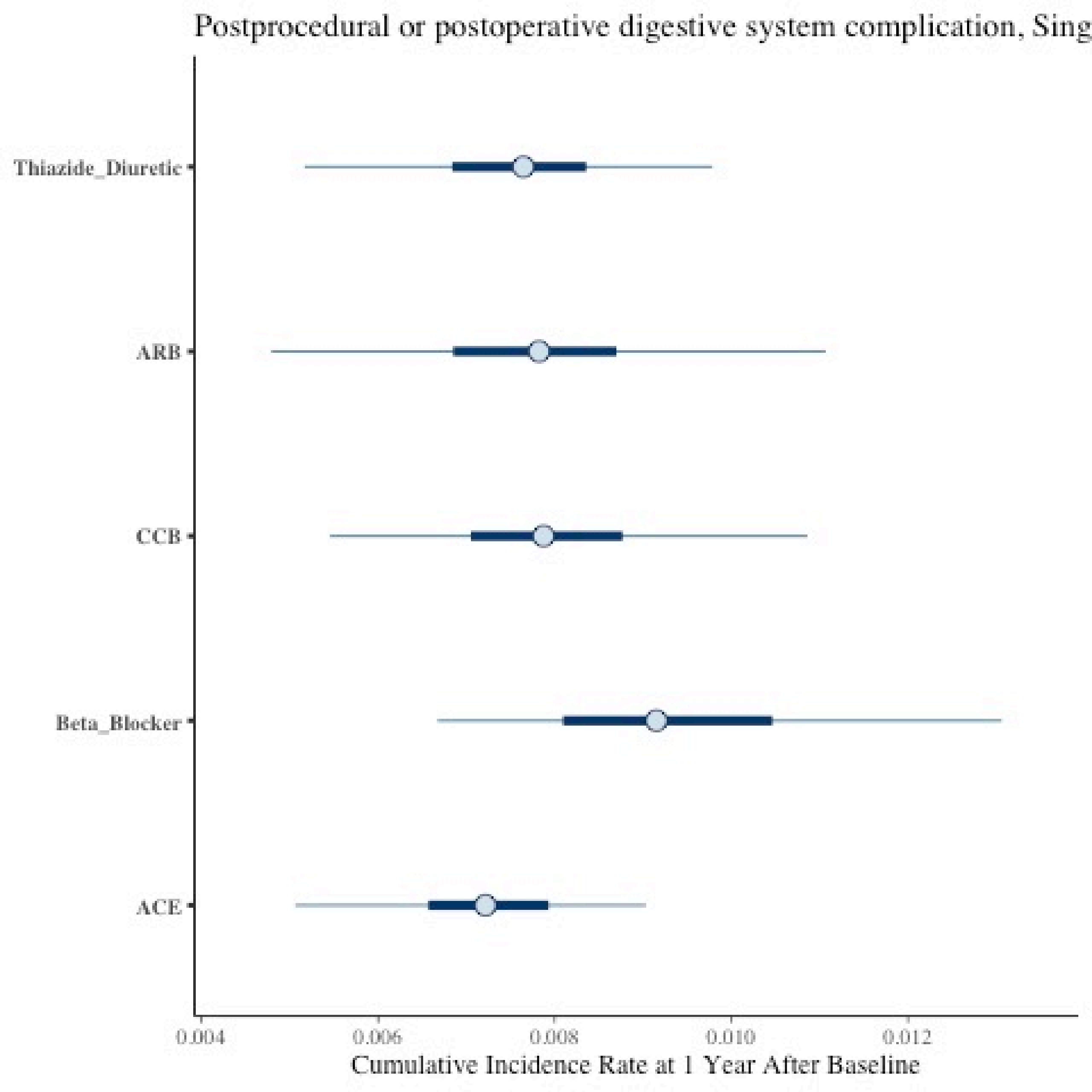
Postprocedural or postoperative digestive system complication, Sing
Thiazide_Diuretic
ARB
CCB
Beta_Blocker
ACE
0.004
0.006
0.008
0.010
0.012
Cumulative Incidence Rate at 1 Year After Baseline

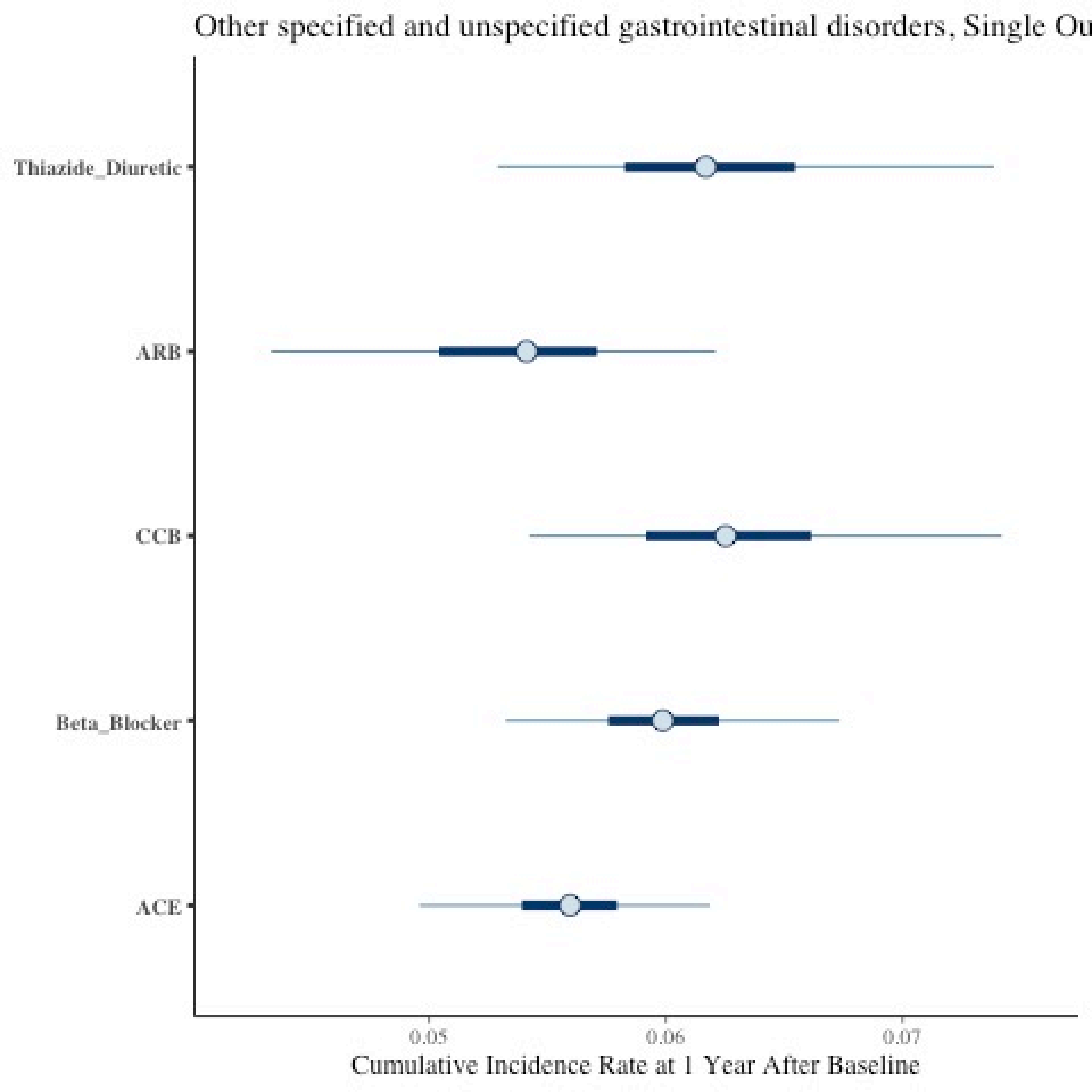

Other specified and unspecified gastrointestinal disorders, Single Ou
Thiazide_Diuretic
ARB
CCB
Beta_Blocker
ACE
0.05
0.06
0.07
Cumulative Incidence Rate at 1 Year After Baseline

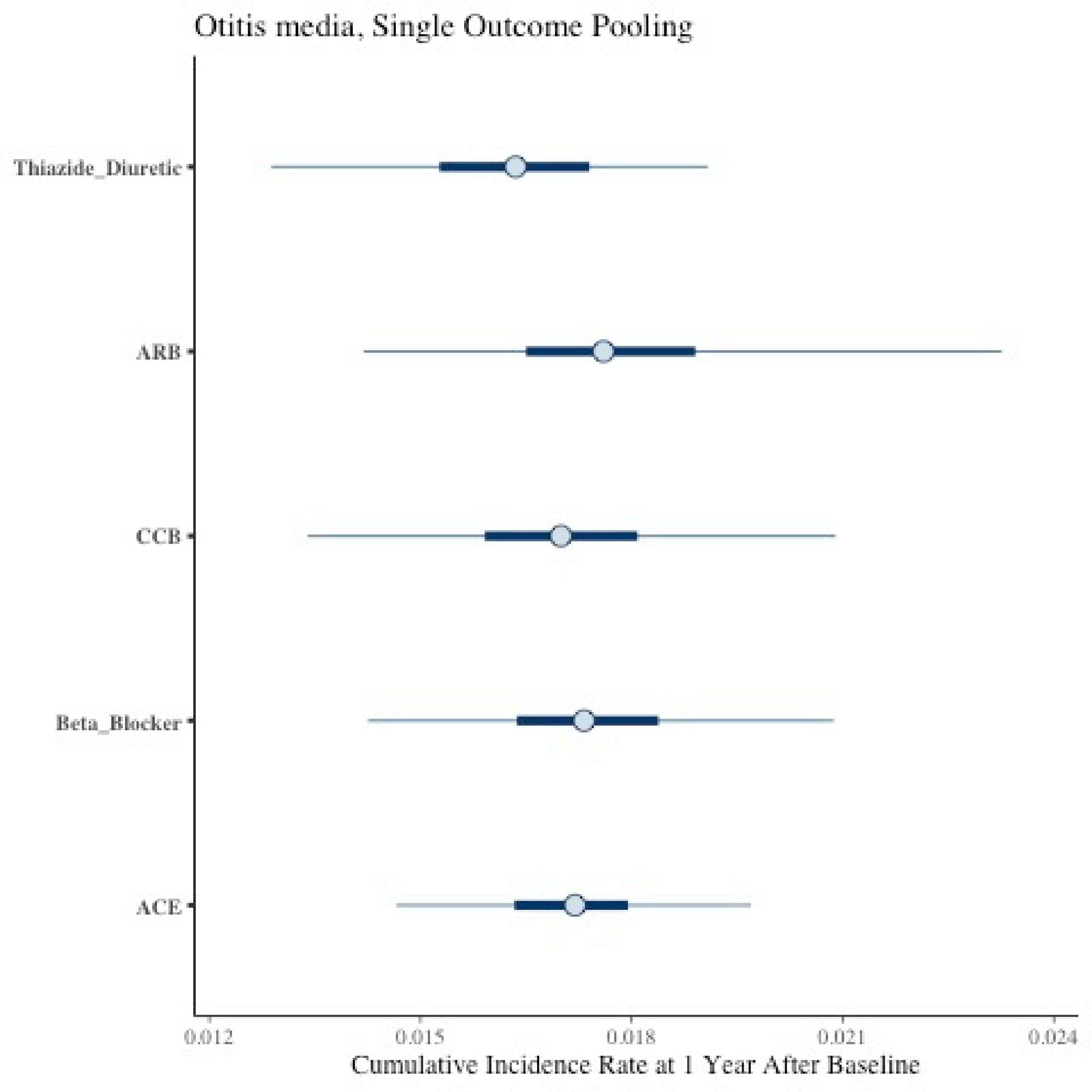

Otitis media, Single Outcome Pooling
Thiazide_Diuretic
ARB
CCB
Beta_Blocker
ACE
0.012
0.015
0.018
0.021
0.024
Cumulative Incidence Rate at 1 Year After Baseline

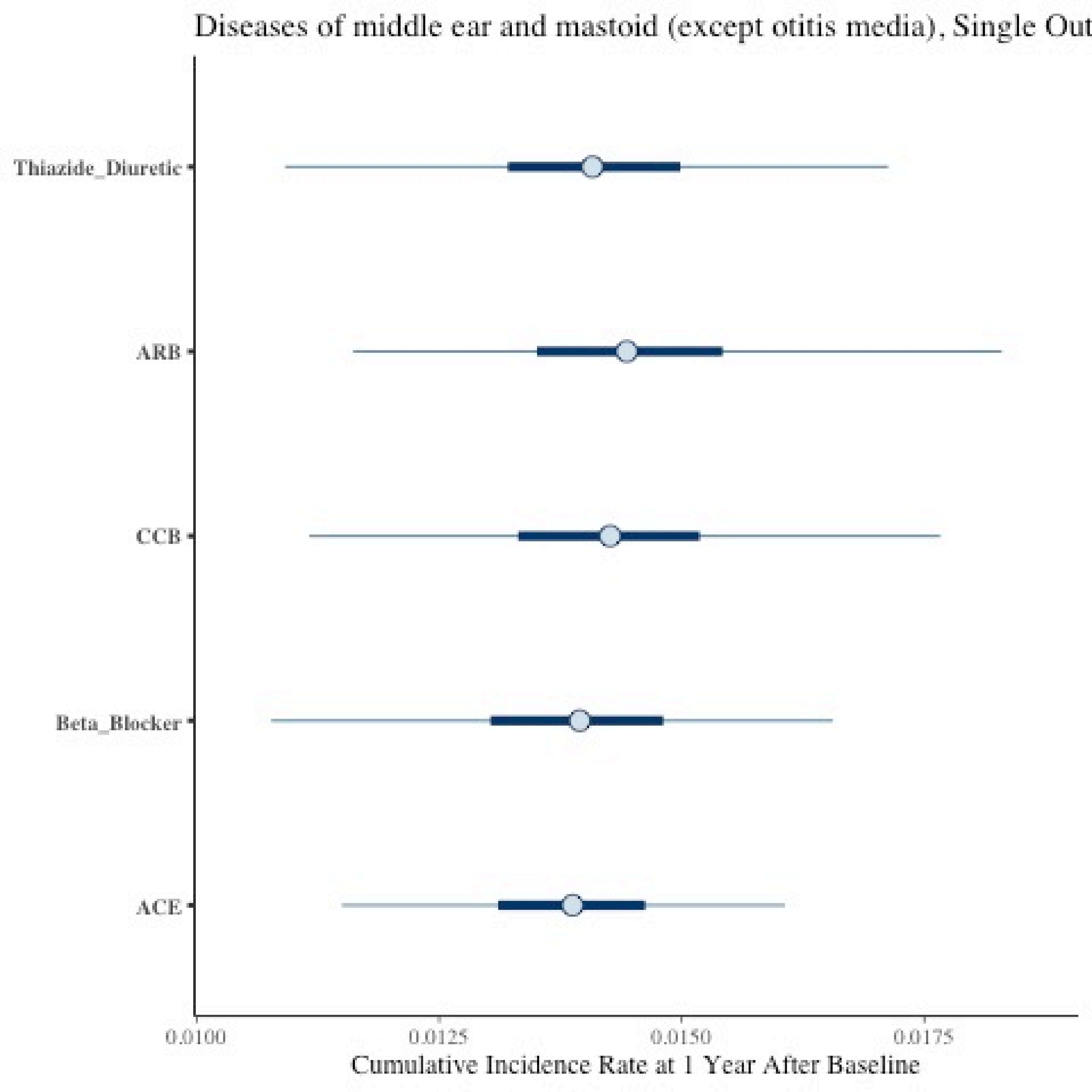
Diseases of middle ear and mastoid (except otitis media), Single Out
Thiazide_Diuretic
ARB
CCB
Beta_Blocker
ACE
0.0100
0.0125
0.0150
0.0175
Cumulative Incidence Rate at 1 Year After Baseline

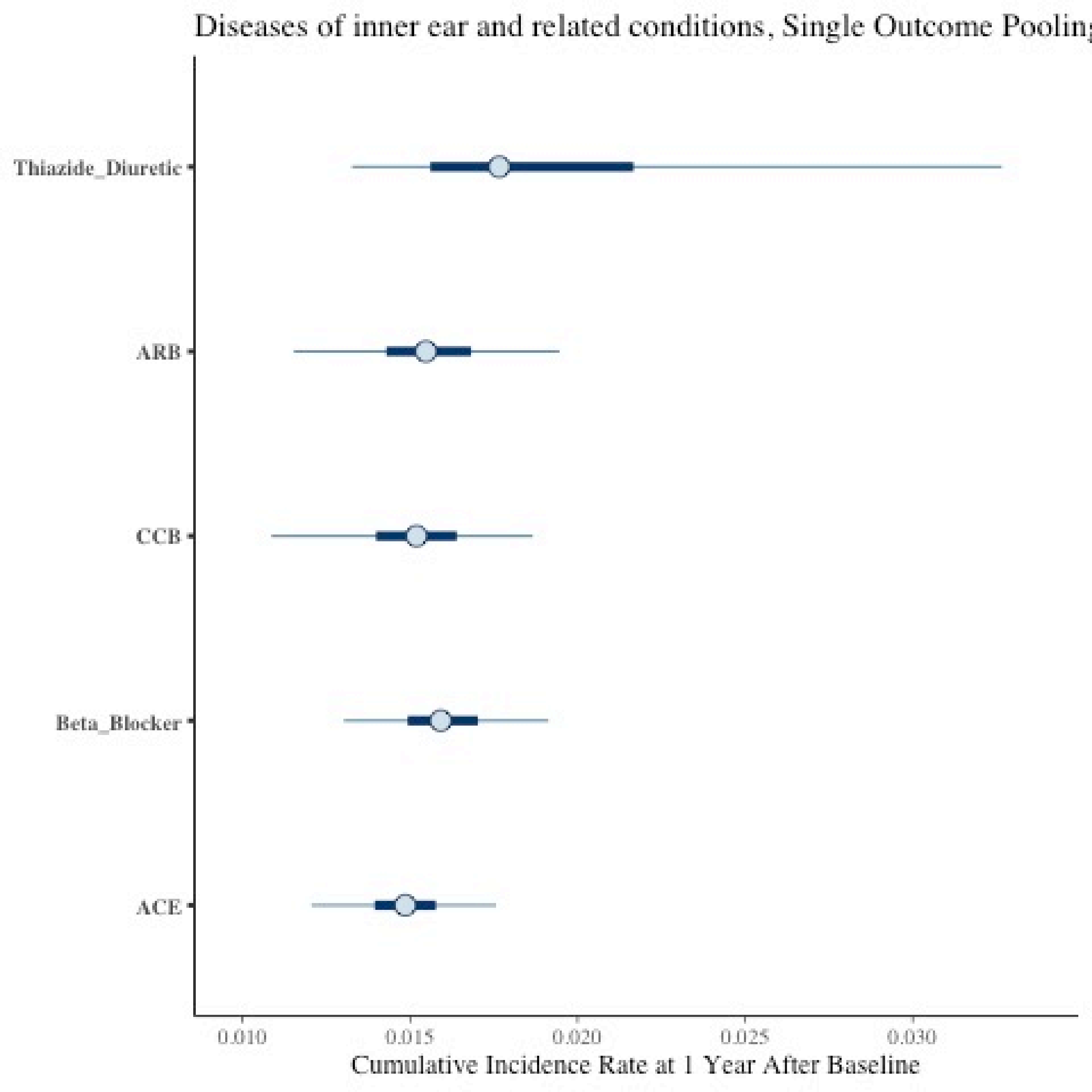

Diseases of inner ear and related conditions, Single Outcome Pooling
Thiazide_Diuretic
ARB
CCB
Beta_Blocker
ACE
0.010
0.015
0.020
0.025
0.030
Cumulative Incidence Rate at 1 Year After Baseline

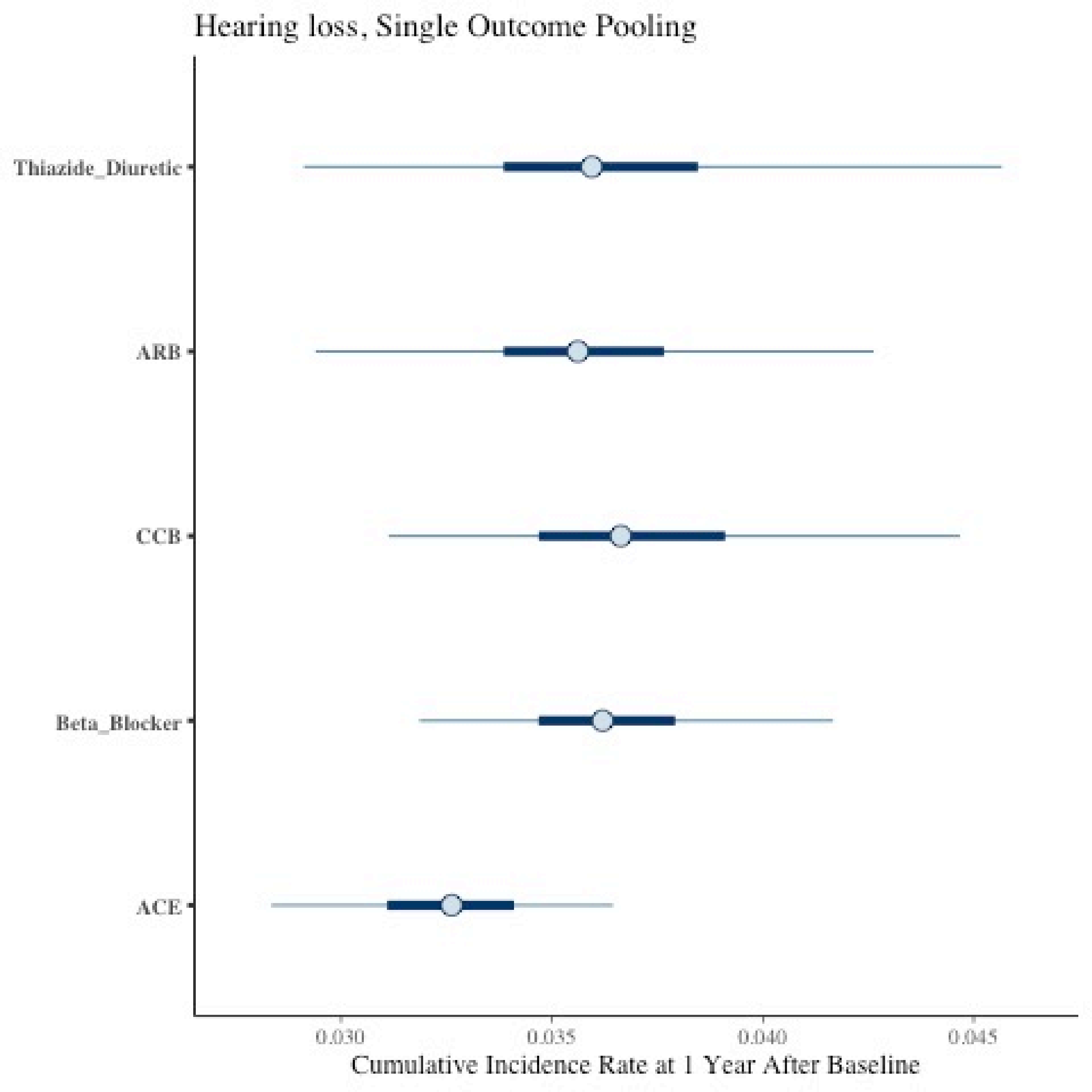
Hearing loss, Single Outcome Pooling
Thiazide_Diuretic
ARB
CCB
Beta_Blocker
ACE
0.030
0.035
0.040
0.045
Cumulative Incidence Rate at 1 Year After Baseline

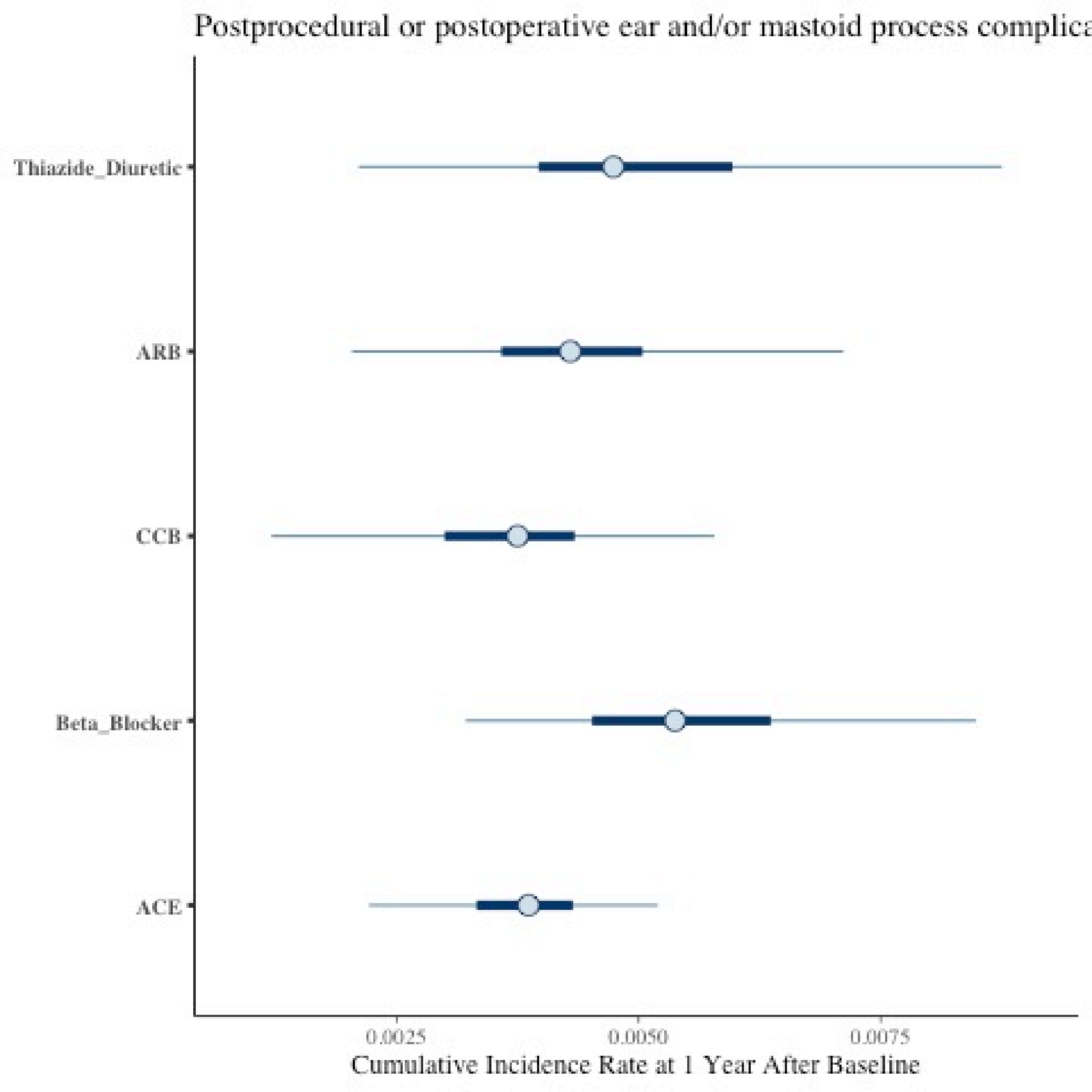

Postprocedural or postoperative ear and/or mastoid process complica
Thiazide_Diuretic
ARB
CCB
Beta_Blocker
ACE
0.0025
0.0050
0.0075
Cumulative Incidence Rate at 1 Year After Baseline

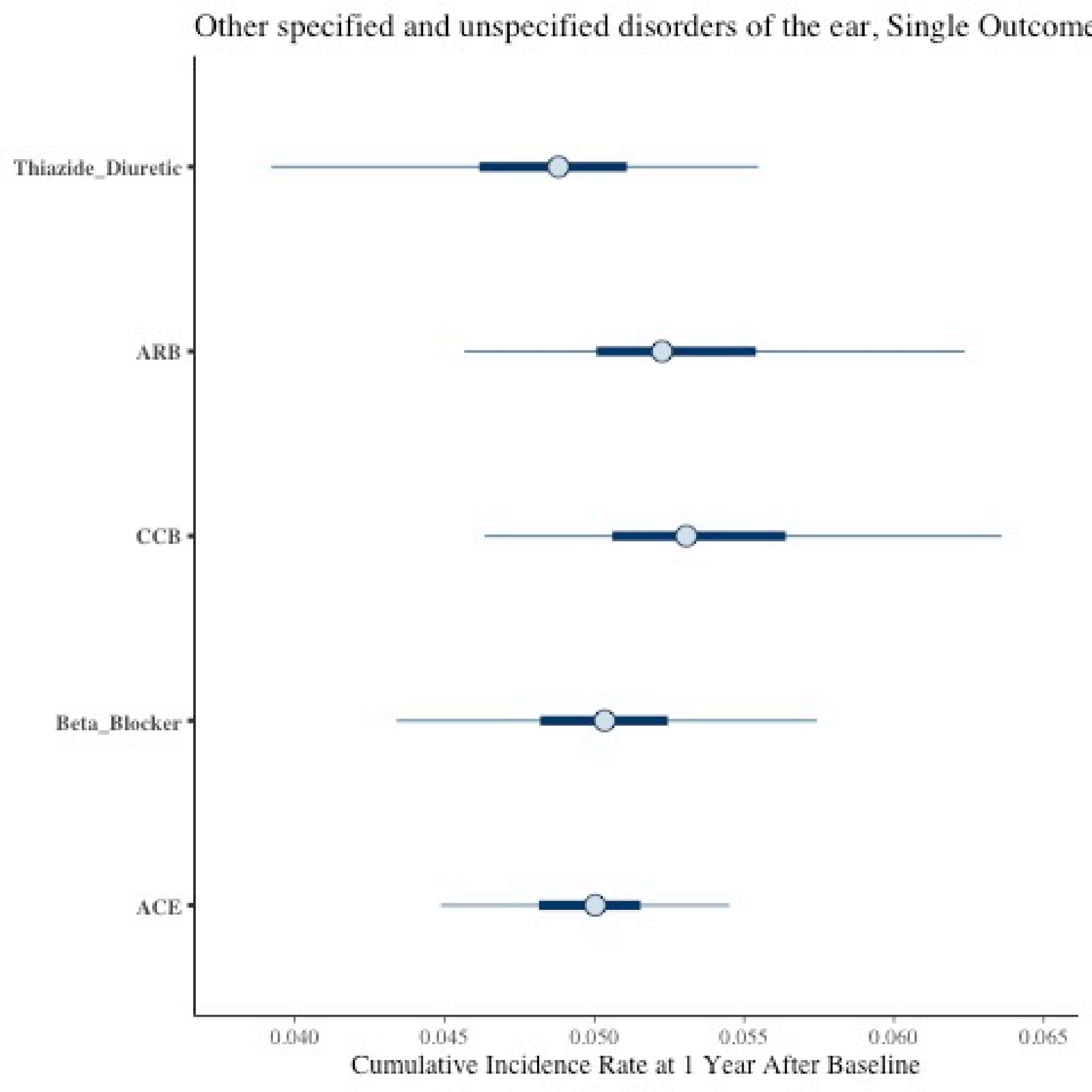

Other specified and unspecified disorders of the ear, Single Outcome
Thiazide_Diuretic
ARB
CCB
Beta_Blocker
ACE
0.040
0.045
0.050
0.055
0.060
0.065
Cumulative Incidence Rate at 1 Year After Baseline

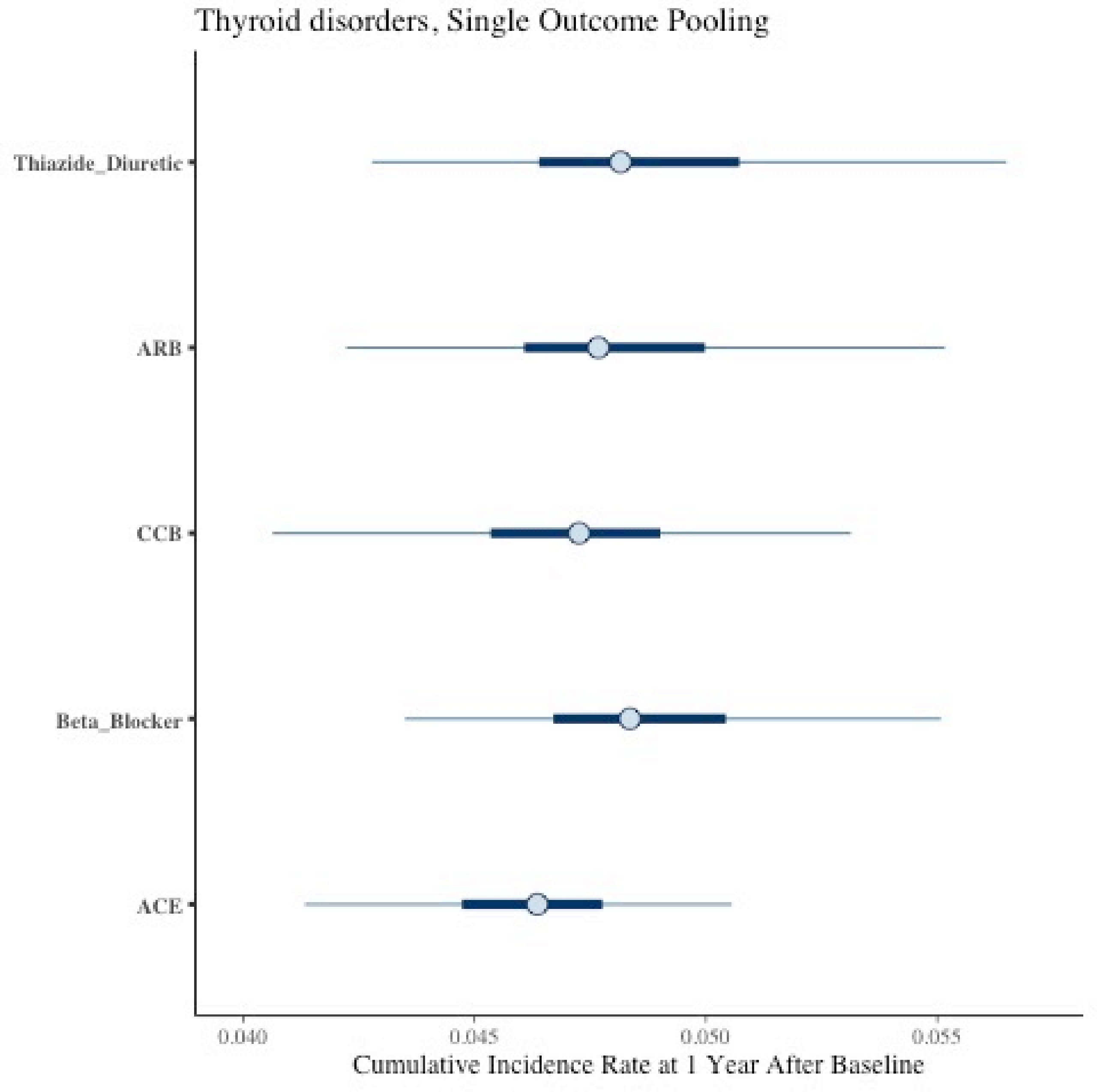

Thyroid disorders, Single Outcome Pooling
Thiazide_Diuretic
ARB
CCB
Beta_Blocker
ACE
0.040
0.045
0.050
0.055
Cumulative Incidence Rate at 1 Year After Baseline

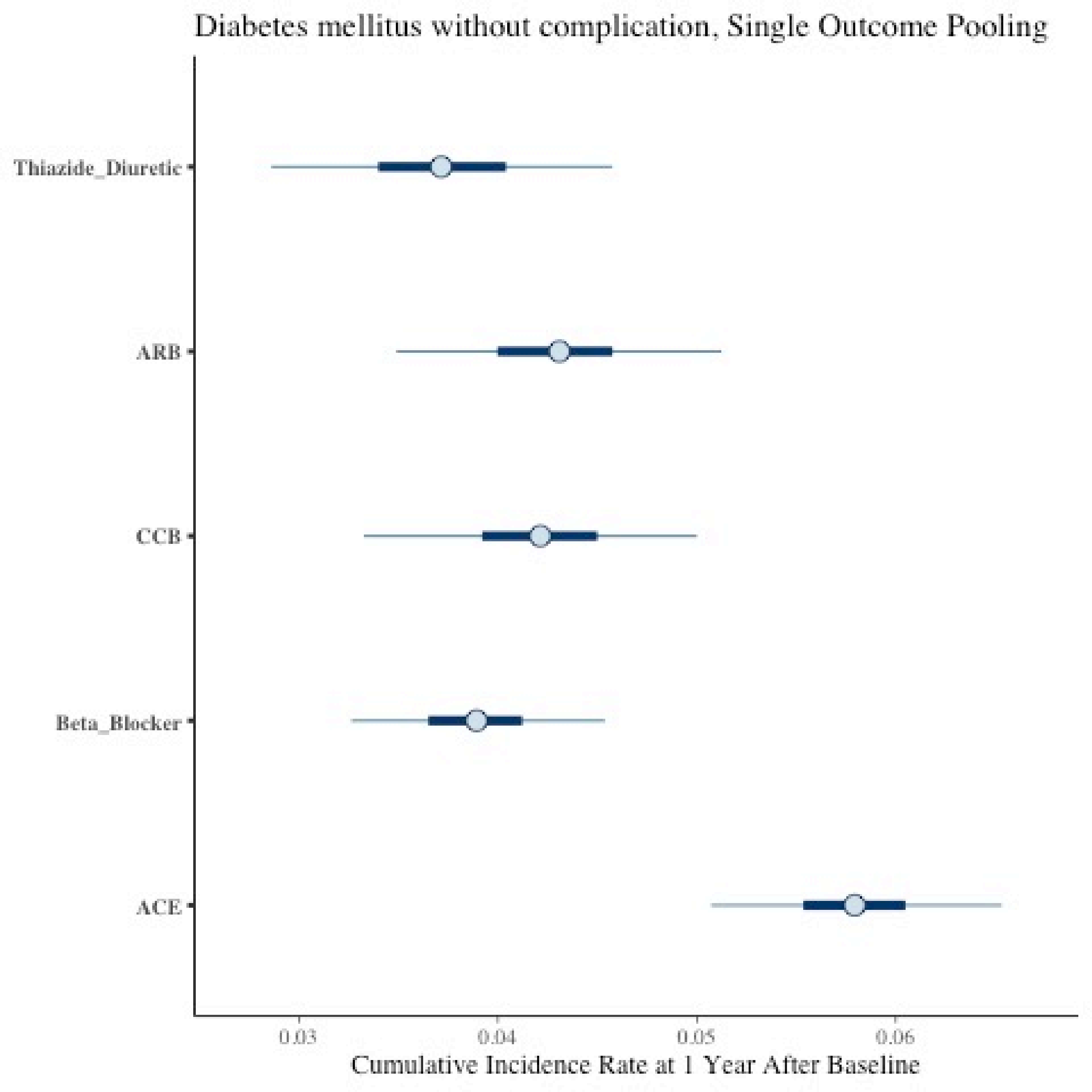

Diabetes mellitus without complication, Single Outcome Pooling
Thiazide_Diuretic
ARB
CCB
Beta_Blocker
ACE
0.03
0.04
0.05
0.06
Cumulative Incidence Rate at 1 Year After Baseline

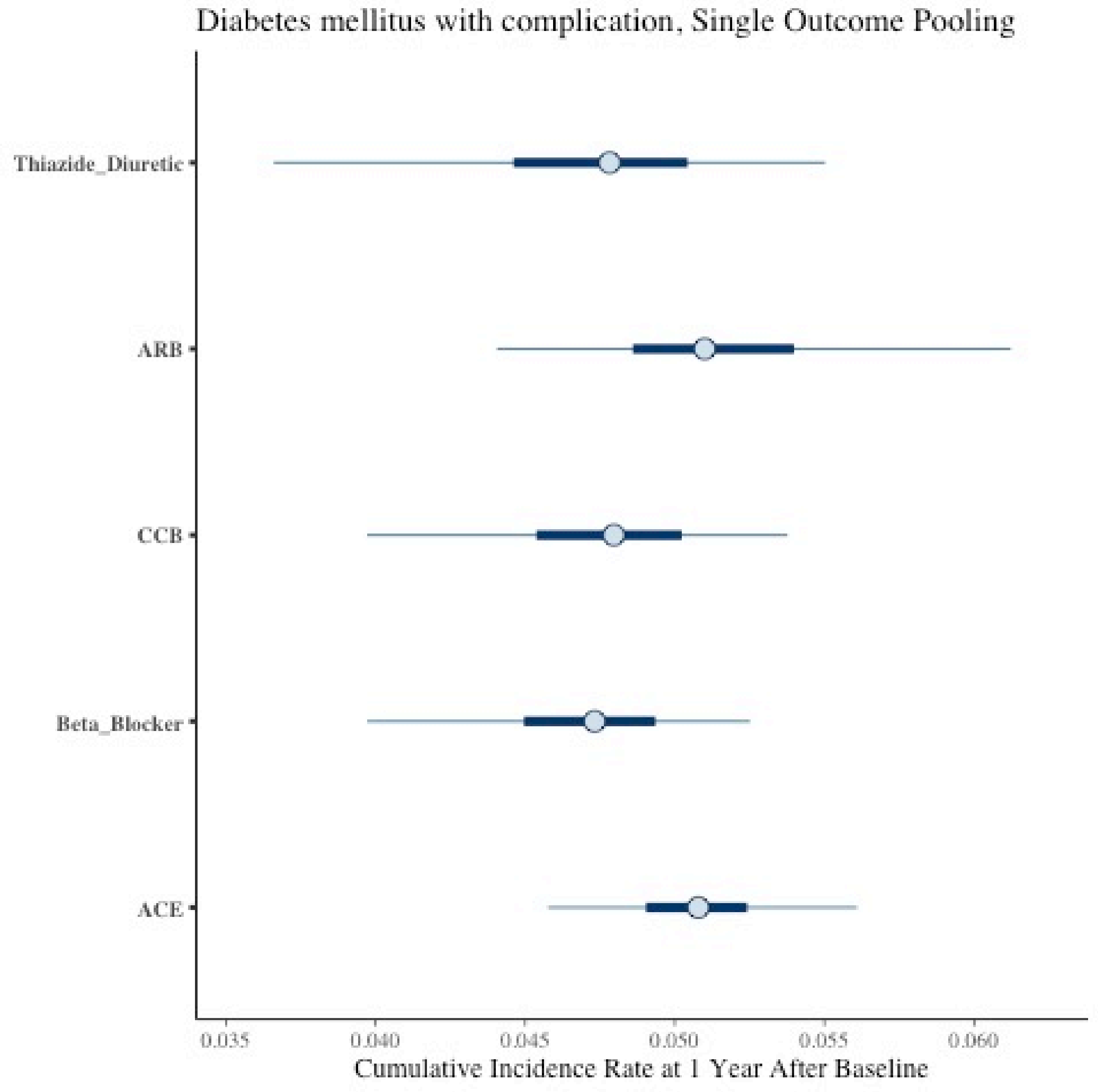

Diabetes mellitus with complication, Single Outcome Pooling
Thiazide_Diuretic
ARB
CCB
Beta_Blocker
ACE
0.035
0.040
0.045
0.050
0.055
0.060
Cumulative Incidence Rate at 1 Year After Baseline

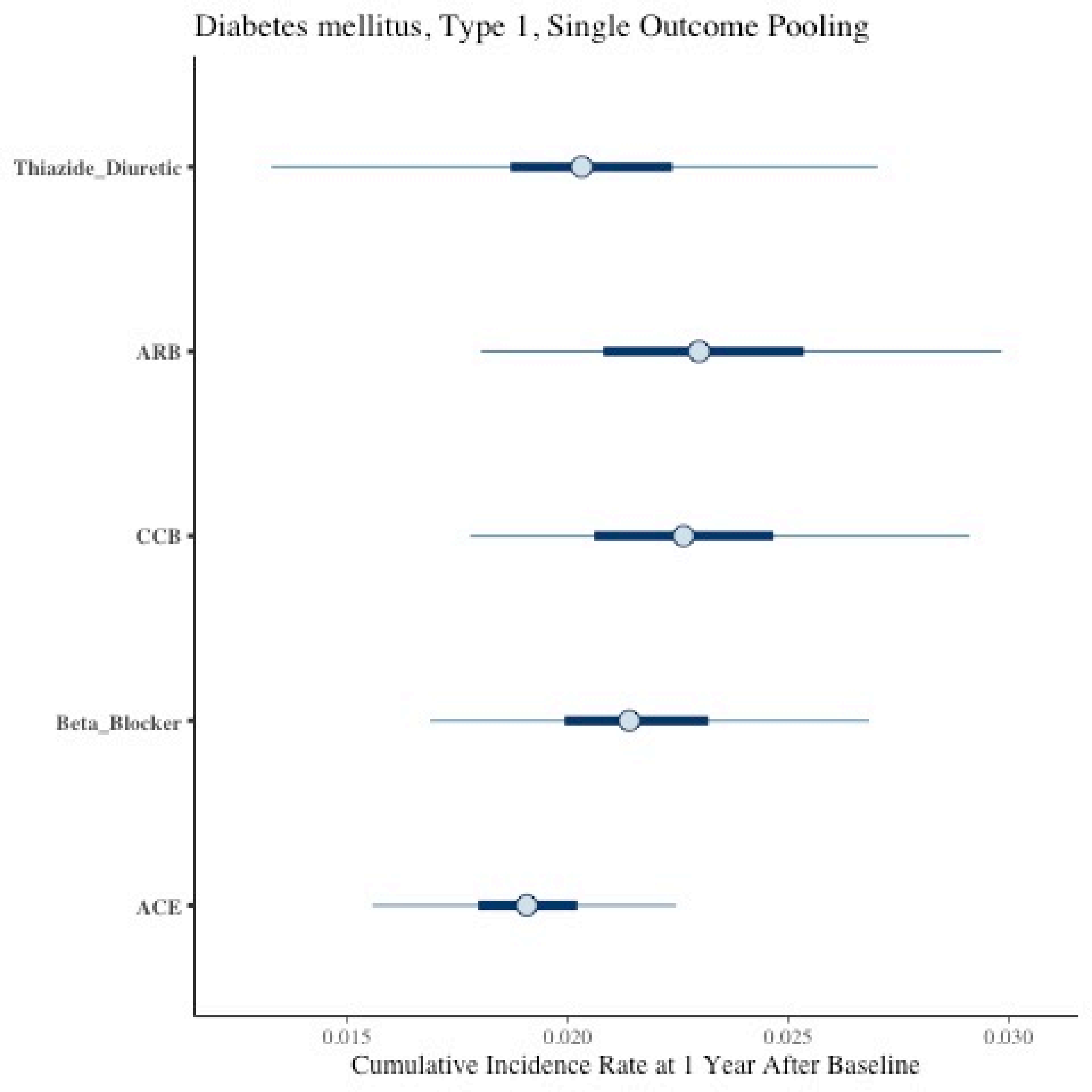

Diabetes mellitus, Type 1, Single Outcome Pooling
Thiazide_Diuretic
ARB
CCB
Beta_Blocker
ACE
0.015
0.020
0.025
0.030
Cumulative Incidence Rate at 1 Year After Baseline

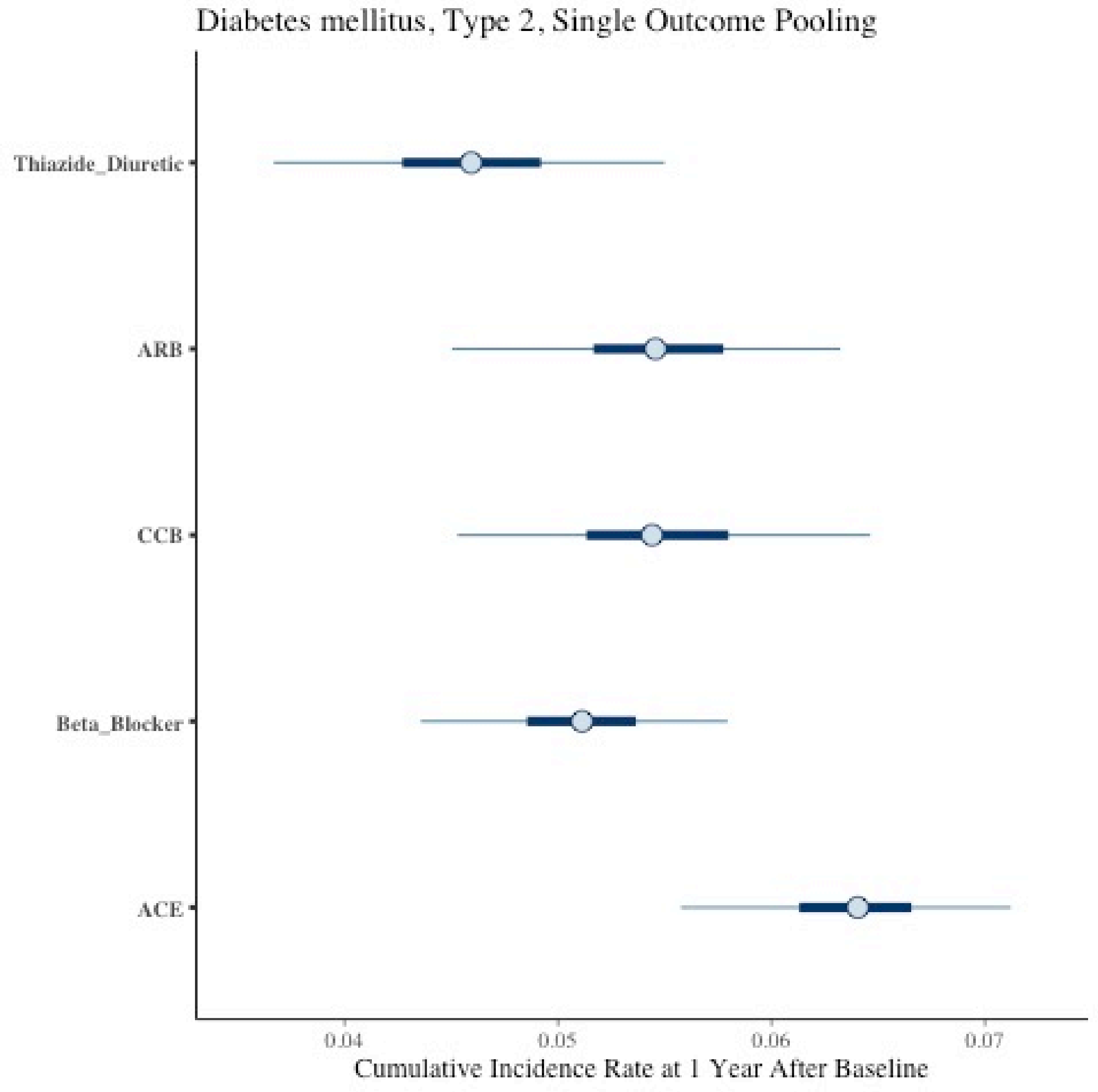

Diabetes mellitus, Type 2, Single Outcome Pooling
Thiazide_Diuretic
ARB
CCB
Beta_Blocker
ACE
0.04
0.05
0.06
0.07
Cumulative Incidence Rate at 1 Year After Baseline

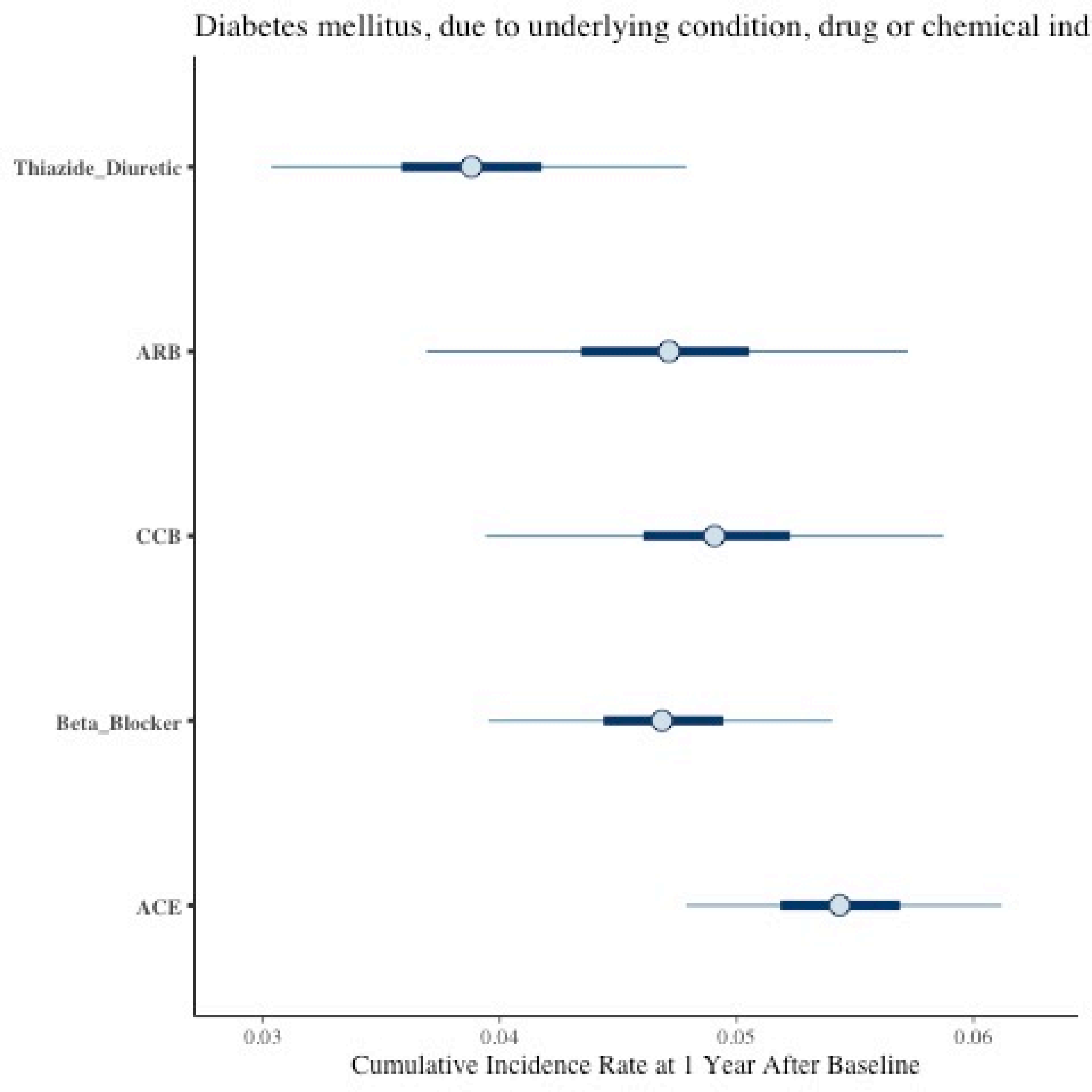

Diabetes mellitus, due to underlying condition, drug or chemical ind
Thiazide_Diuretic
ARB
CCB
Beta_Blocker
ACE
0.03
0.04
0.05
0.06
Cumulative Incidence Rate at 1 Year After Baseline

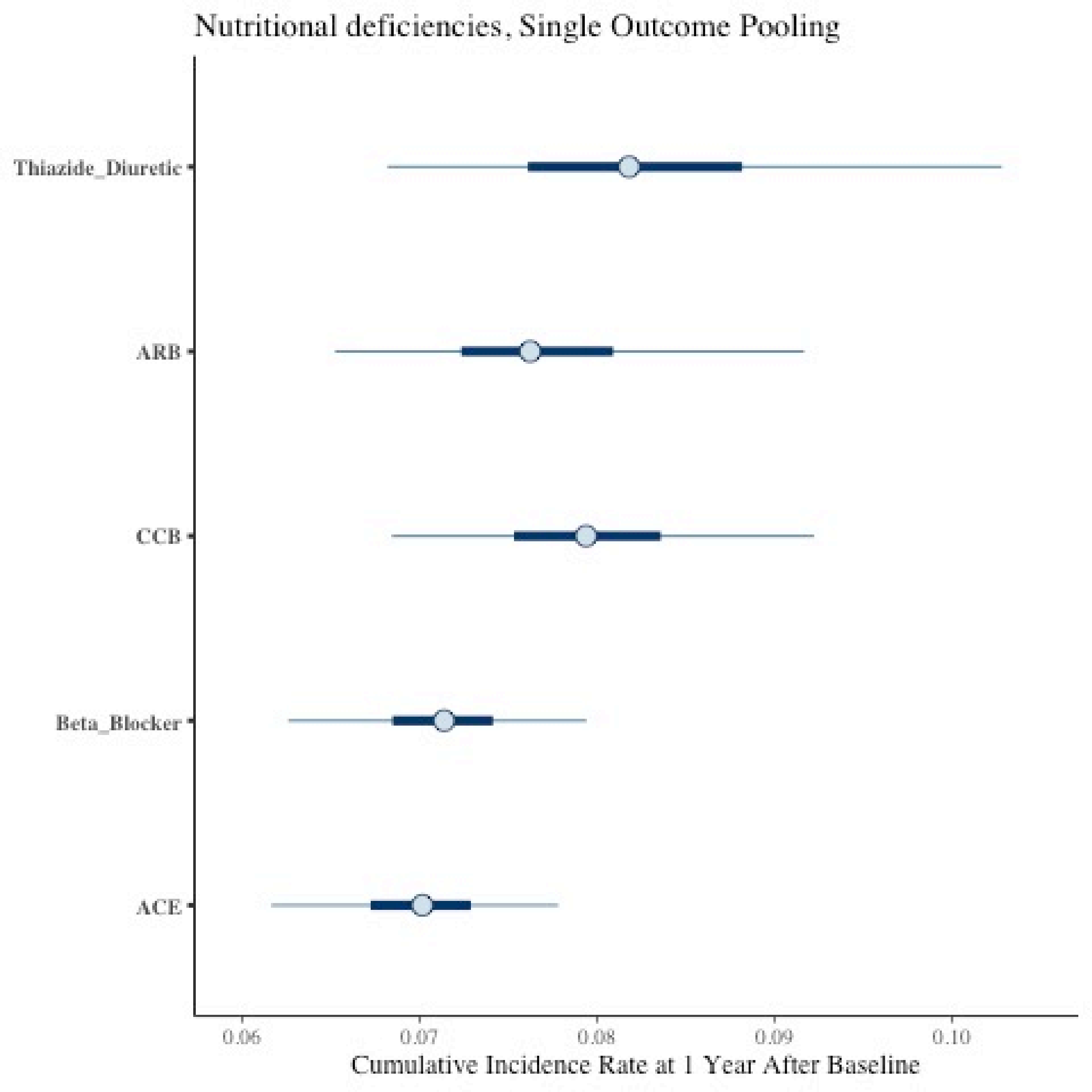

Nutritional deficiencies, Single Outcome Pooling
Thiazide_Diuretic
ARB
CCB
Beta_Blocker
ACE
0.06
0.07
0.08
0.09
0.10
Cumulative Incidence Rate at 1 Year After Baseline

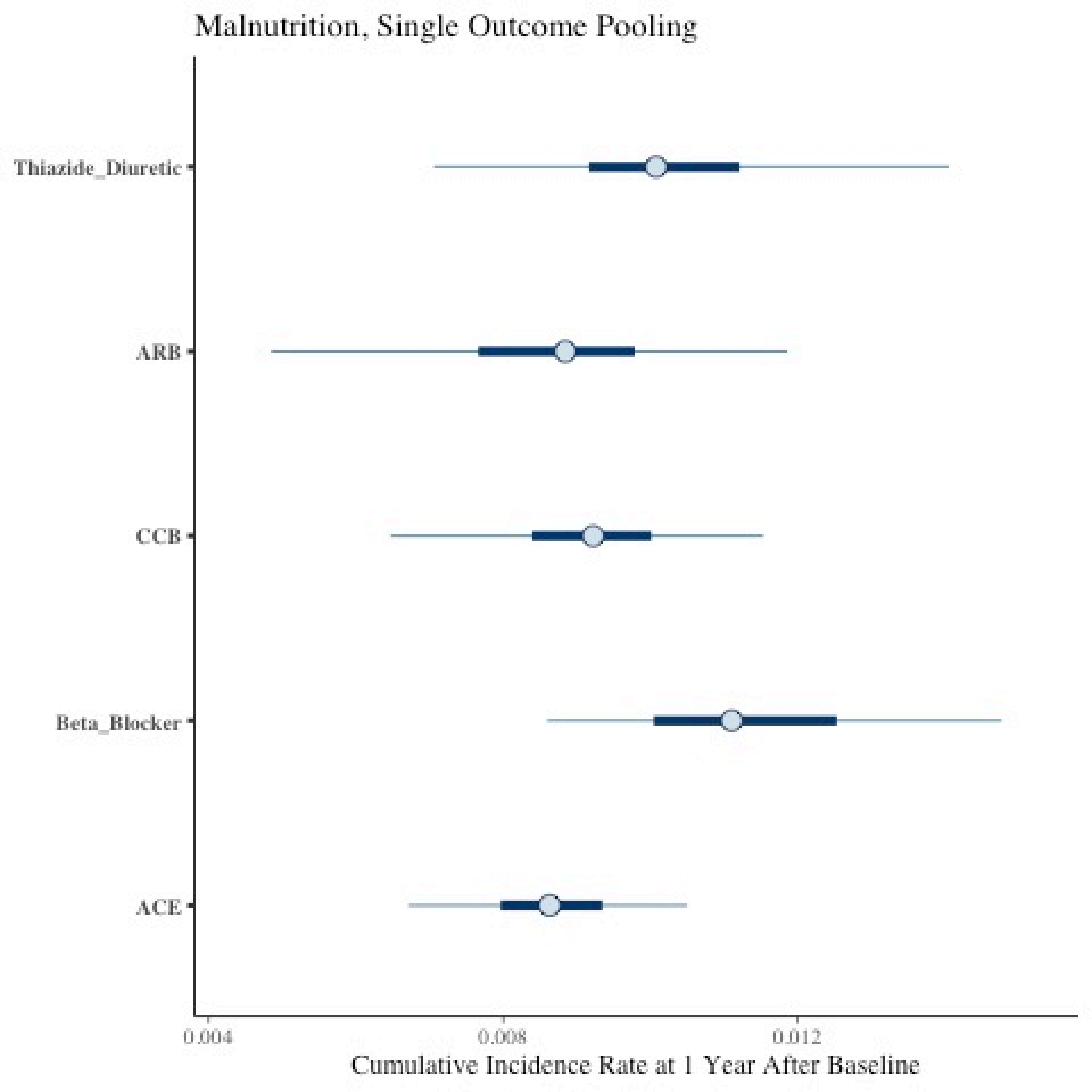

Malnutrition, Single Outcome Pooling
Thiazide_Diuretic
ARB
CCB
Beta_Blocker
ACE
0.004
0.008
0.012
Cumulative Incidence Rate at 1 Year After Baseline

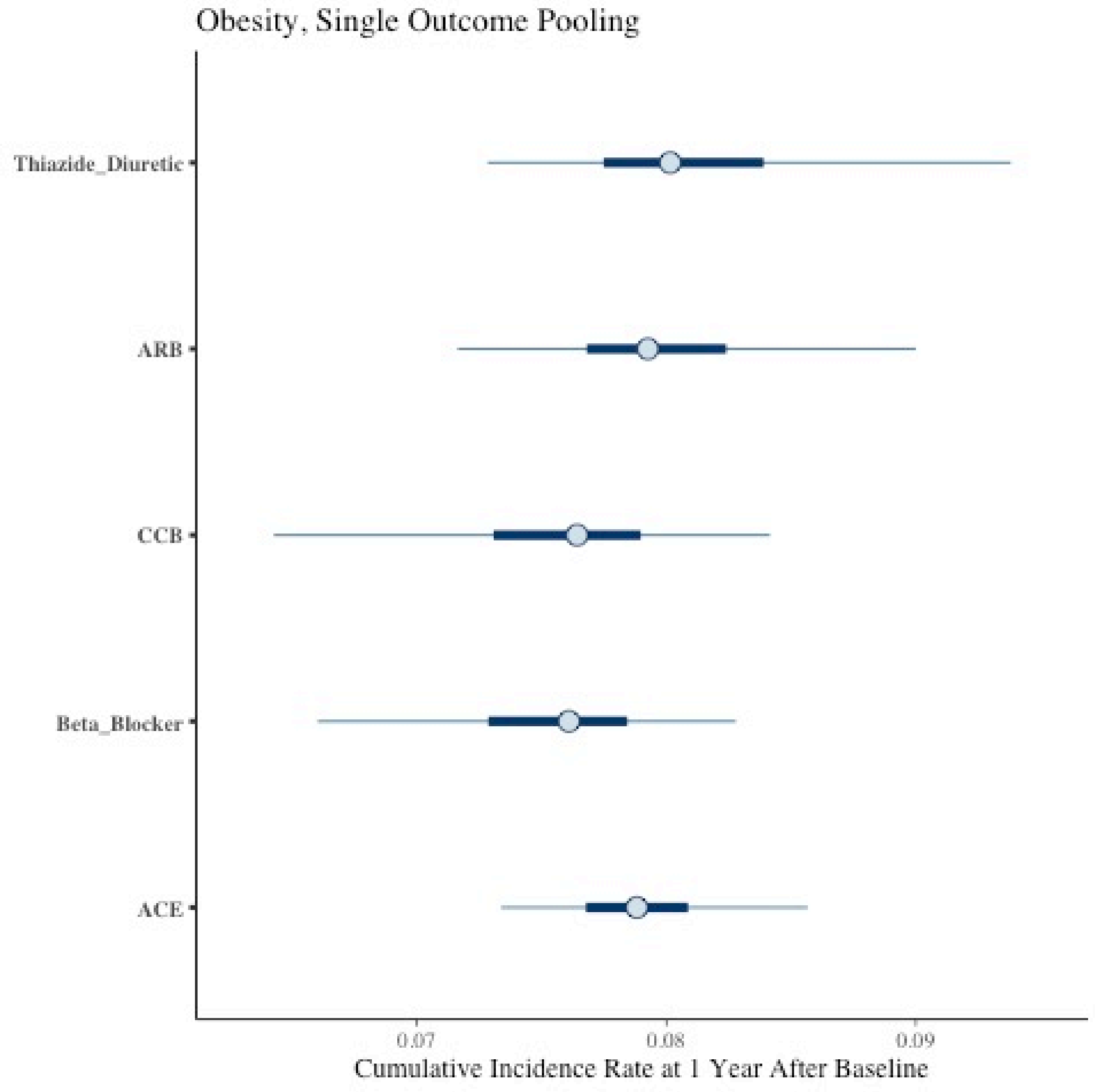

Obesity, Single Outcome Pooling
Thiazide_Diuretic
ARB
CCB
Beta_Blocker
ACE
0.07
0.08
0.09
Cumulative Incidence Rate at 1 Year After Baseline

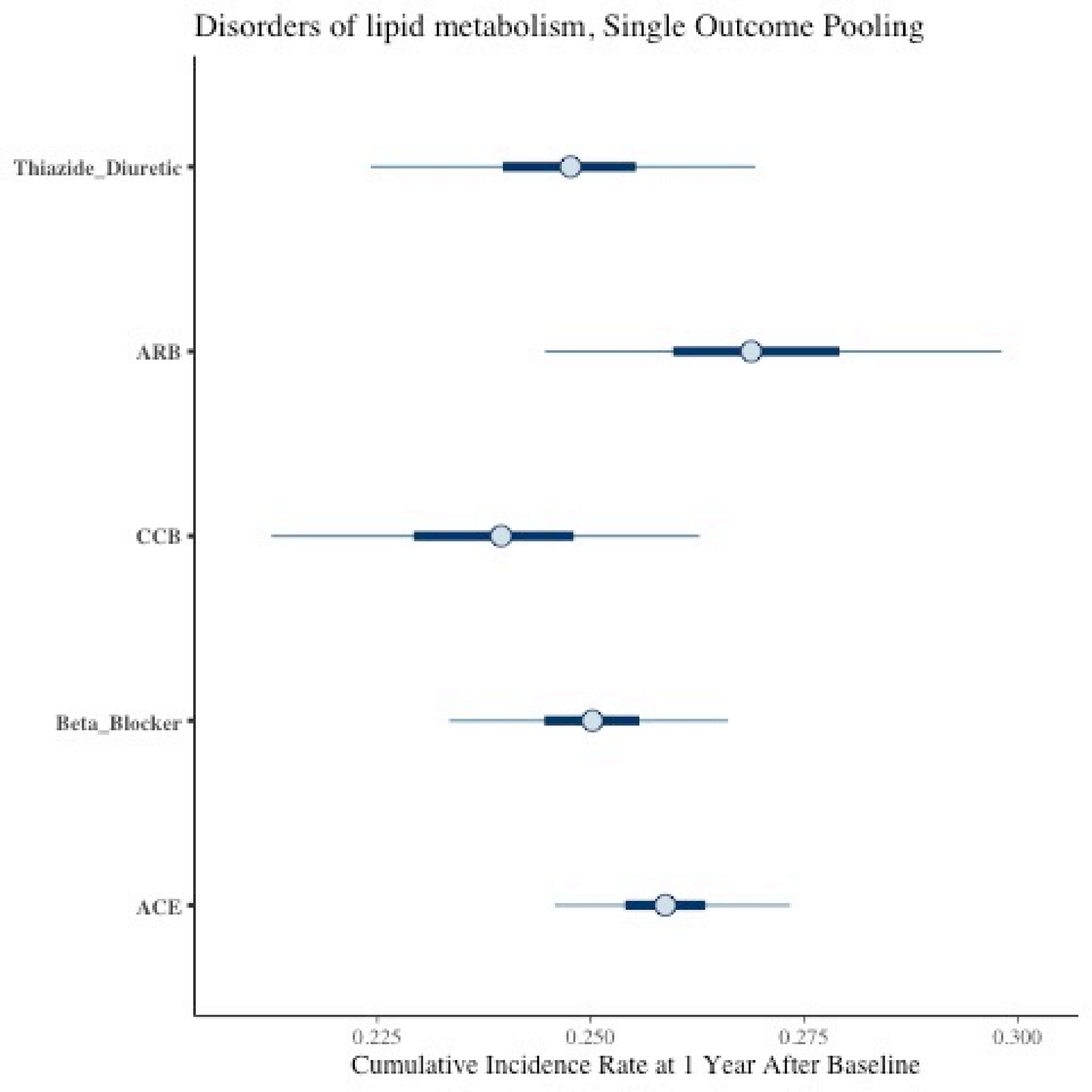

Disorders of lipid metabolism, Single Outcome Pooling
Thiazide_Diuretic
ARB
CCB
Beta_Blocker
ACE
0.225
0.250
0.275
0.300
Cumulative Incidence Rate at 1 Year After Baseline

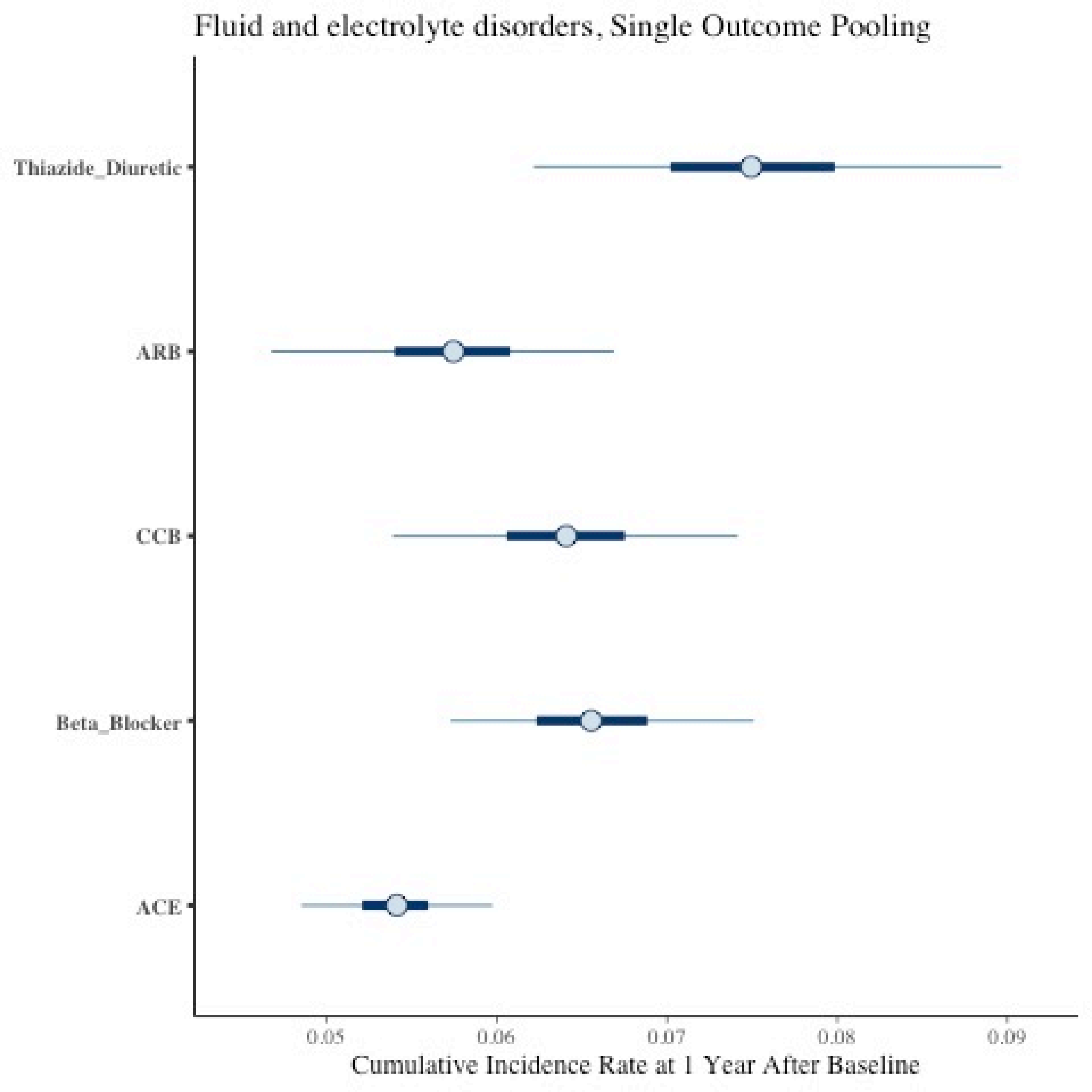

Fluid and electrolyte disorders, Single Outcome Pooling
Thiazide_Diuretic
ARB
CCB
Beta_Blocker
ACE
0.05
0.06
0.07
0.08
0.09
Cumulative Incidence Rate at 1 Year After Baseline

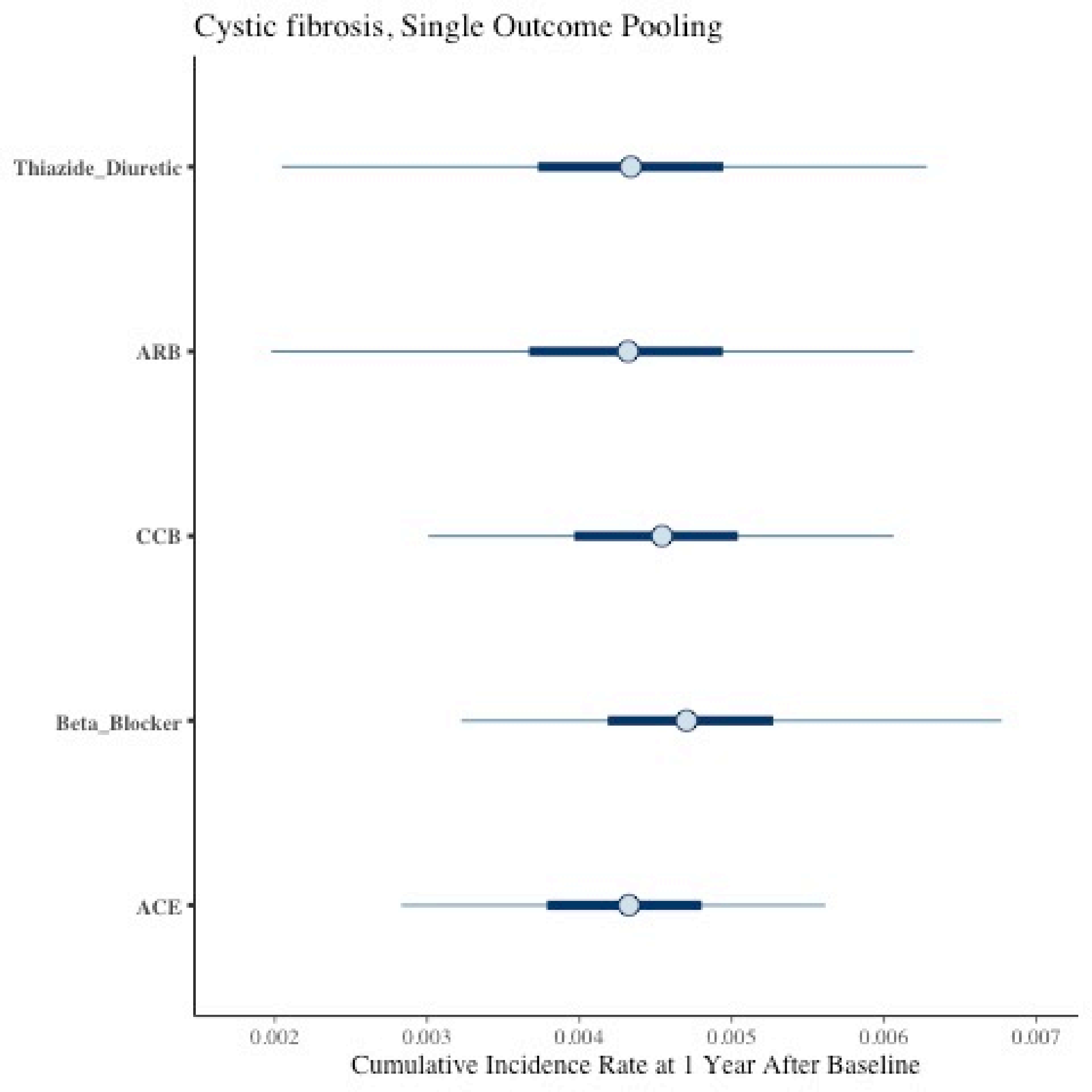
Cystic fibrosis, Single Outcome Pooling
Thiazide_Diuretic
ARB
CCB
Beta_Blocker
ACE
0.002
0.003
0.004
0.005
0.006
0.007
Cumulative Incidence Rate at 1 Year After Baseline

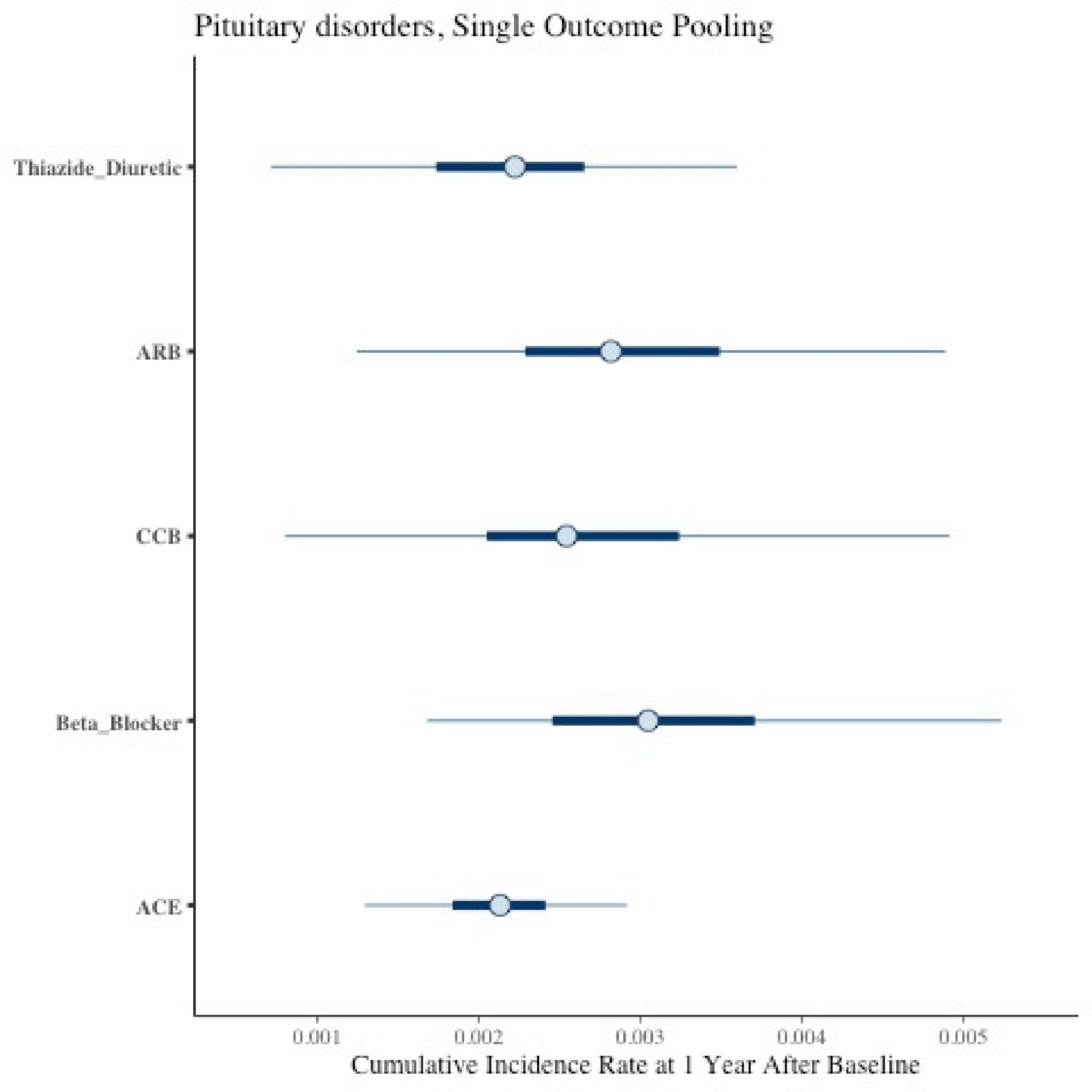

Pituitary disorders, Single Outcome Pooling
Thiazide_Diuretic
ARB
CCB
Beta_Blocker
ACE
0.001
0.002
0.003
0.004
0.005
Cumulative Incidence Rate at 1 Year After Baseline

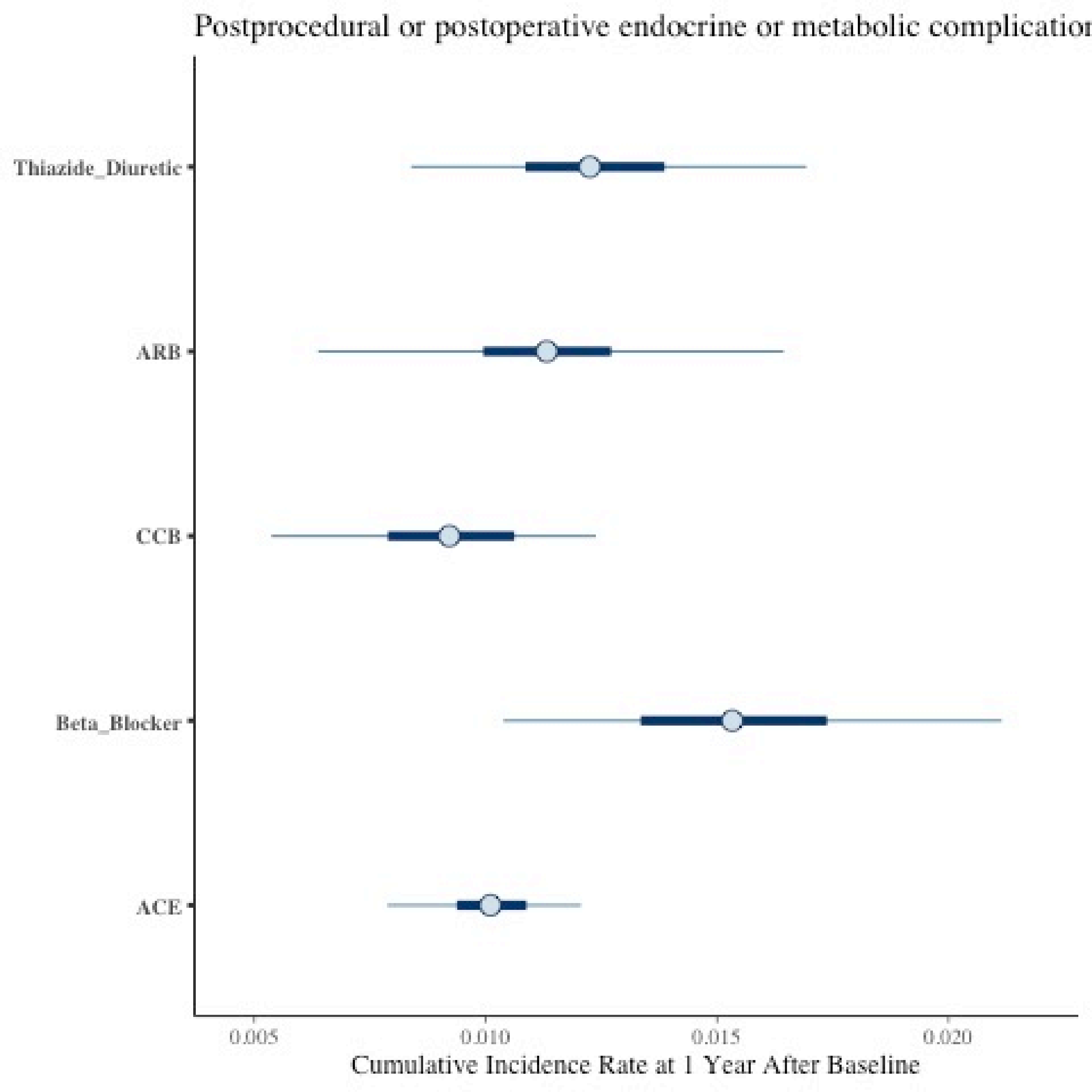
Postprocedural or postoperative endocrine or metabolic complication
Thiazide_Diuretic
ARB
CCB
Beta_Blocker
ACE
0.005
0.010
0.015
0.020
Cumulative Incidence Rate at 1 Year After Baseline

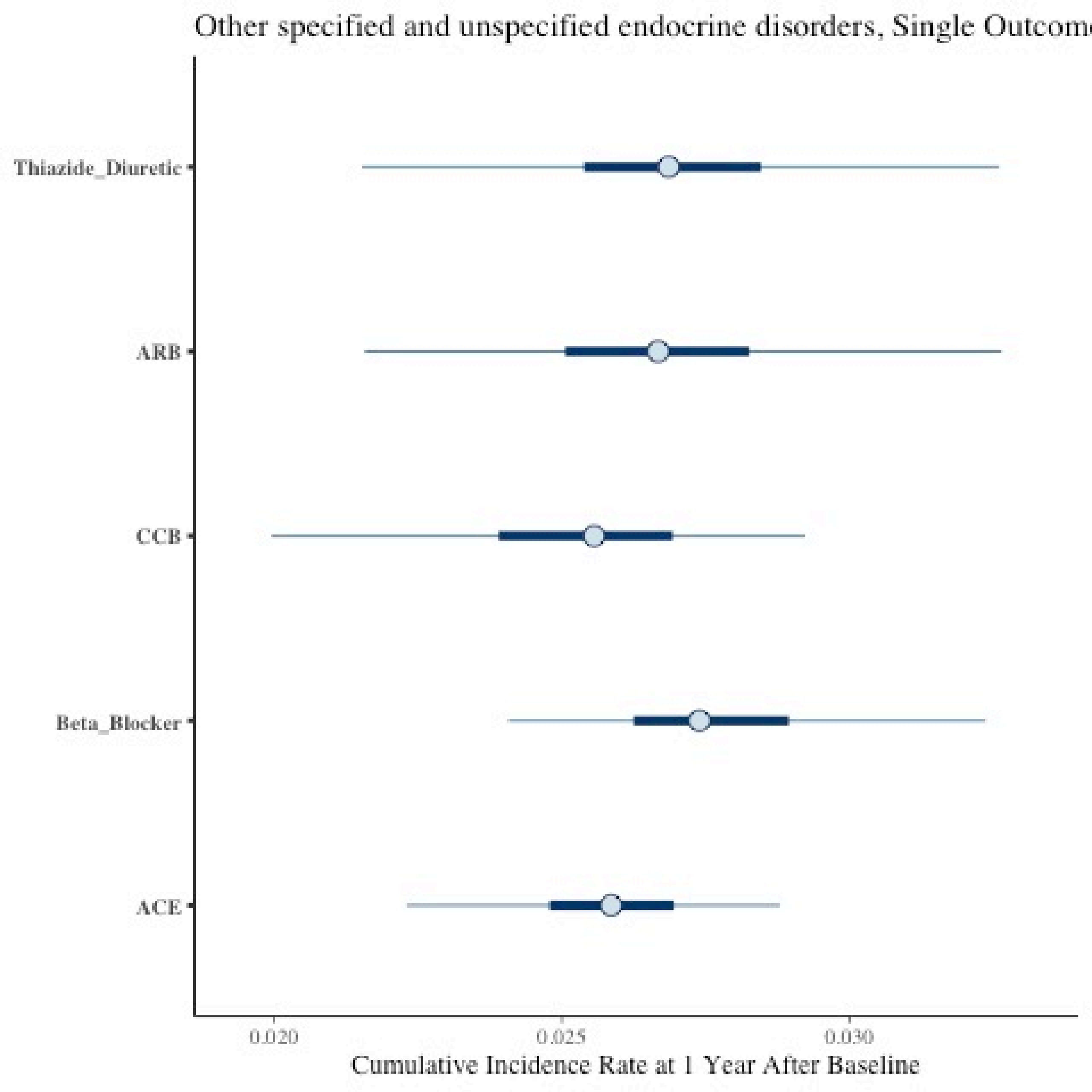
Other specified and unspecified endocrine disorders, Single Outcome
Thiazide_Diuretic
ARB
CCB
Beta_Blocker
ACE
0.020
0.025
0.030
Cumulative Incidence Rate at 1 Year After Baseline

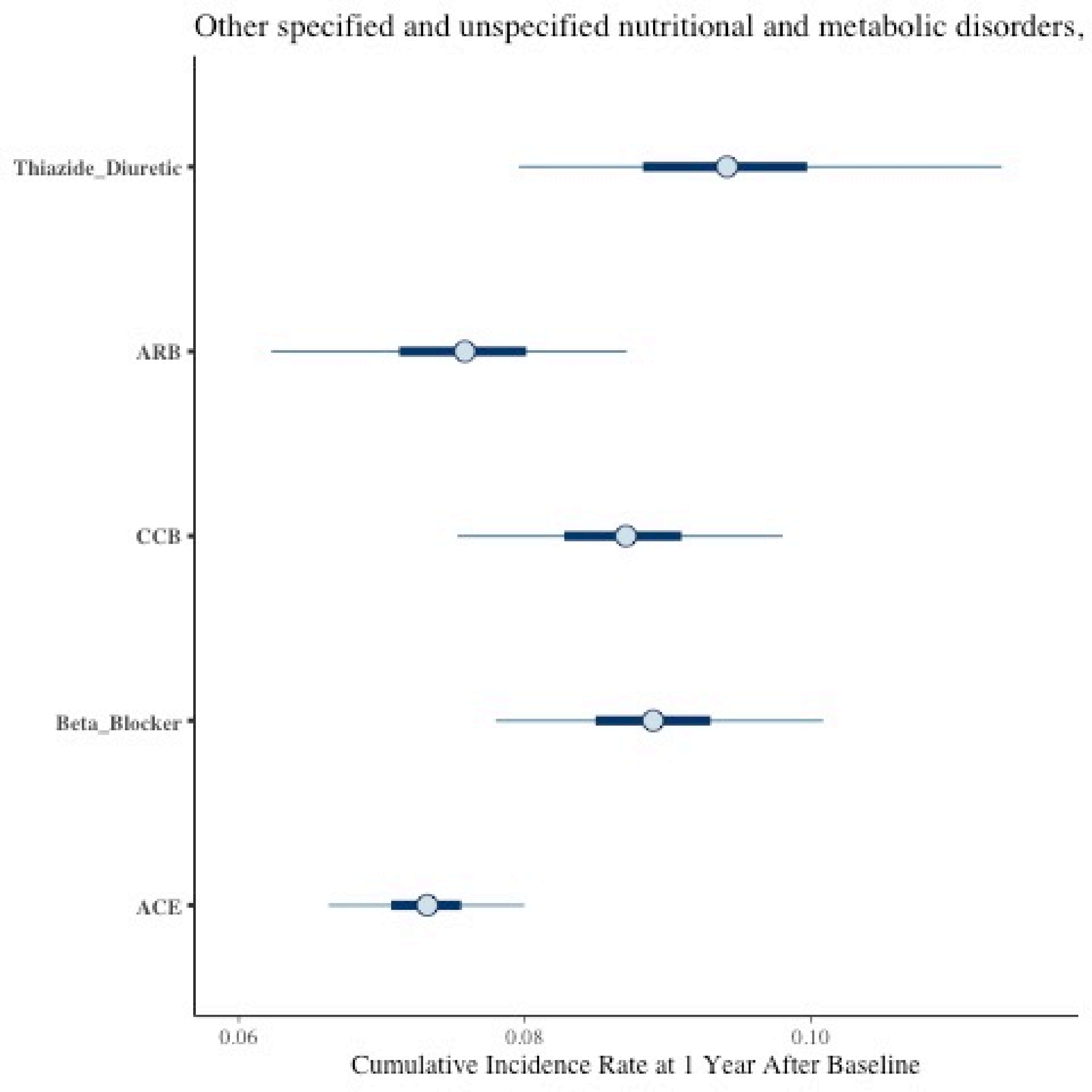
Other specified and unspecified nutritional and metabolic disorders,
Thiazide_Diuretic
ARB
CCB
Beta_Blocker
ACE
0.06
0.08
0.10
Cumulative Incidence Rate at 1 Year After Baseline

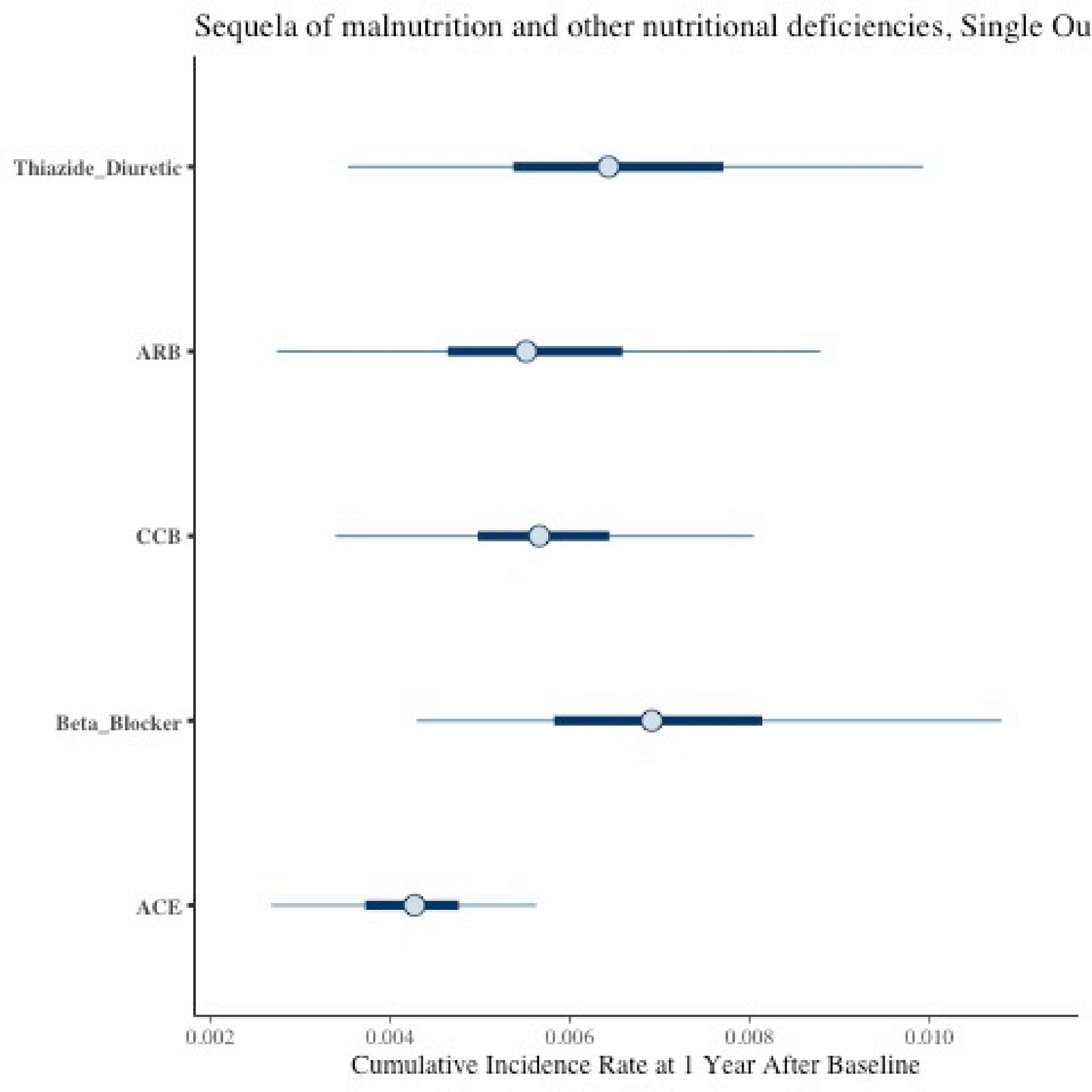

Sequela of malnutrition and other nutritional deficiencies, Single Ou
Thiazide_Diuretic
ARB
CCB
Beta_Blocker
ACE
0.002
0.004
0.006
0.008
0.010
Cumulative Incidence Rate at 1 Year After Baseline

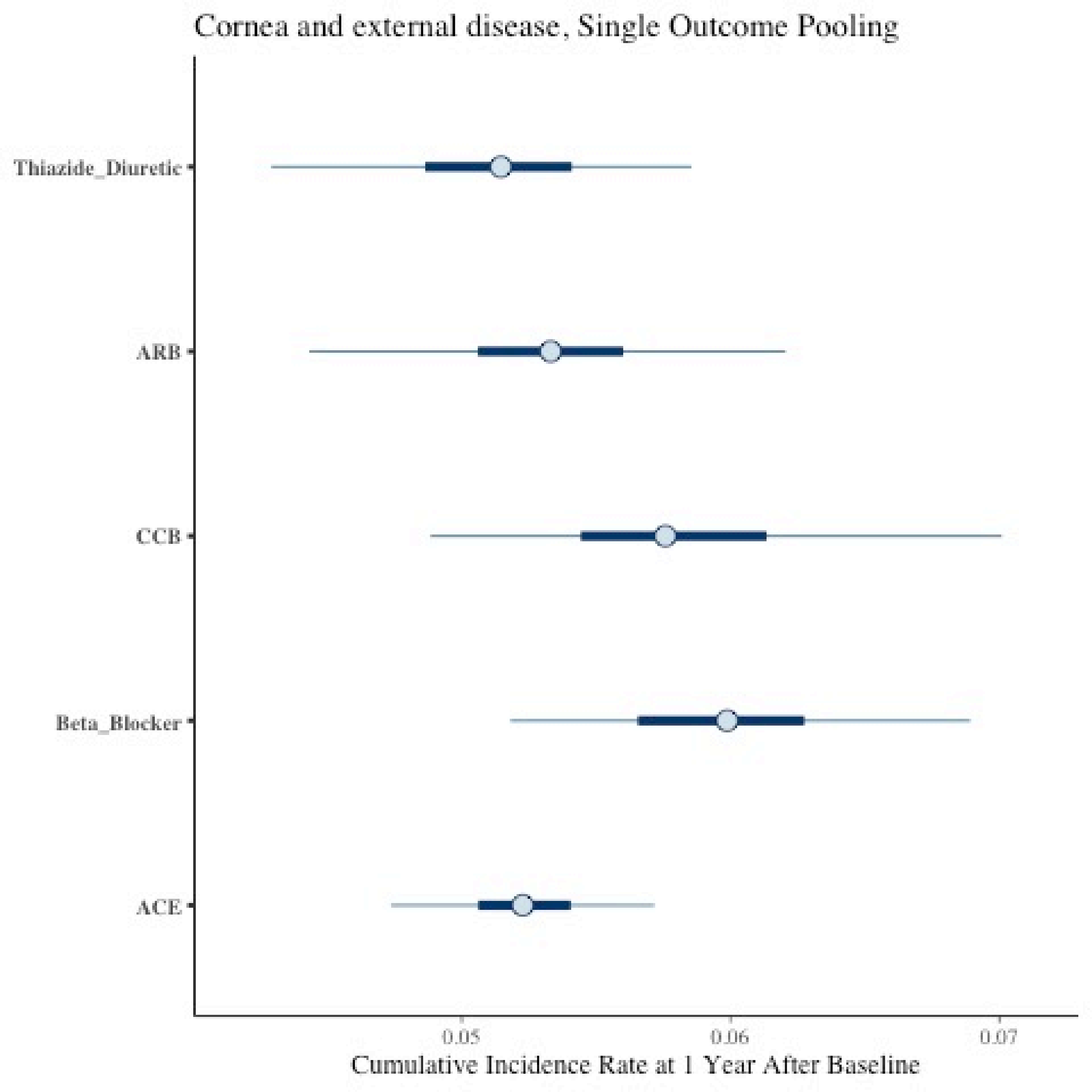

Cornea and external disease, Single Outcome Pooling
Thiazide_Diuretic
ARB
CCB
Beta_Blocker
ACE
0.05
0.06
0.07
Cumulative Incidence Rate at 1 Year After Baseline

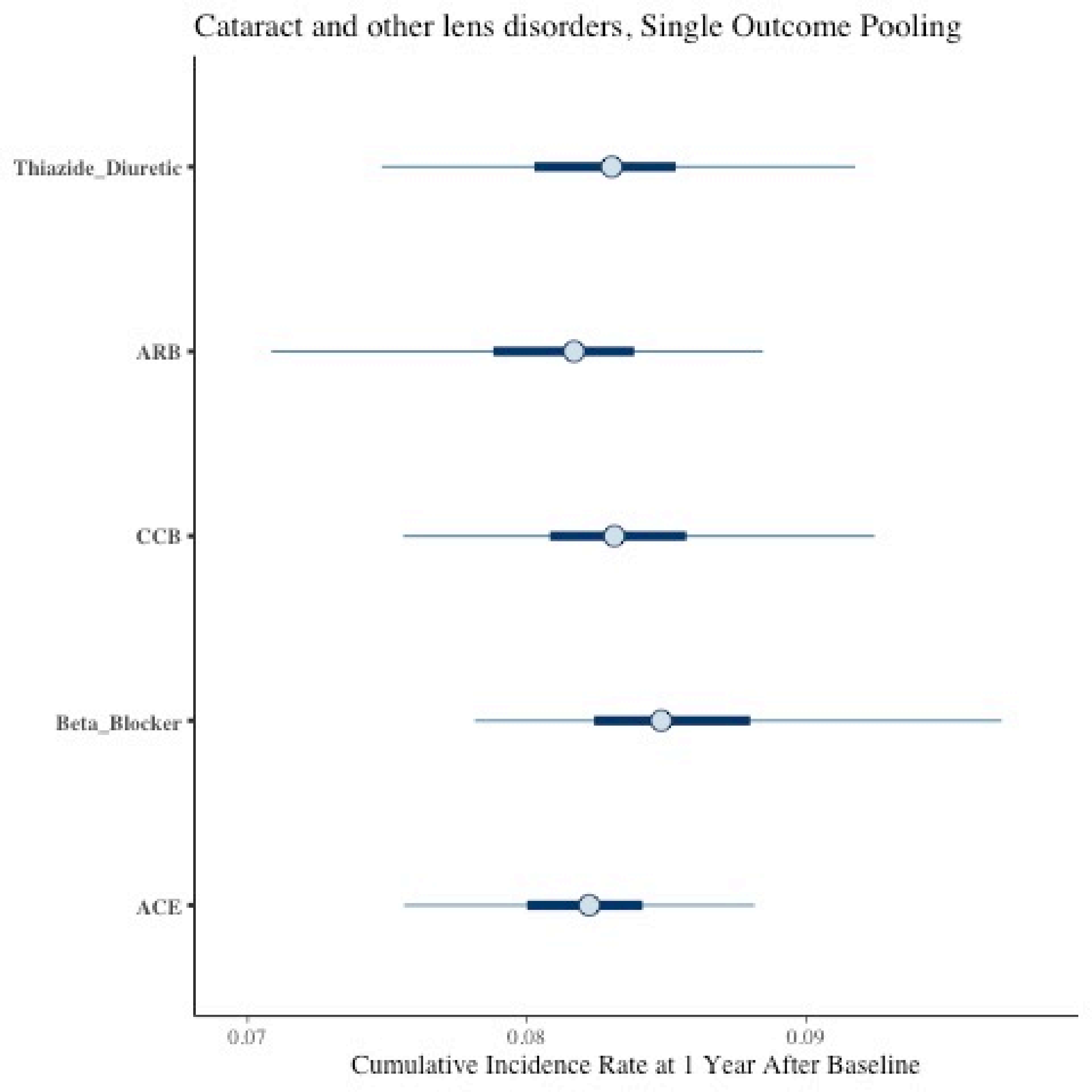

Cataract and other lens disorders, Single Outcome Pooling
Thiazide_Diuretic
ARB
CCB
Beta_Blocker
ACE
0.07
0.08
0.09
Cumulative Incidence Rate at 1 Year After Baseline

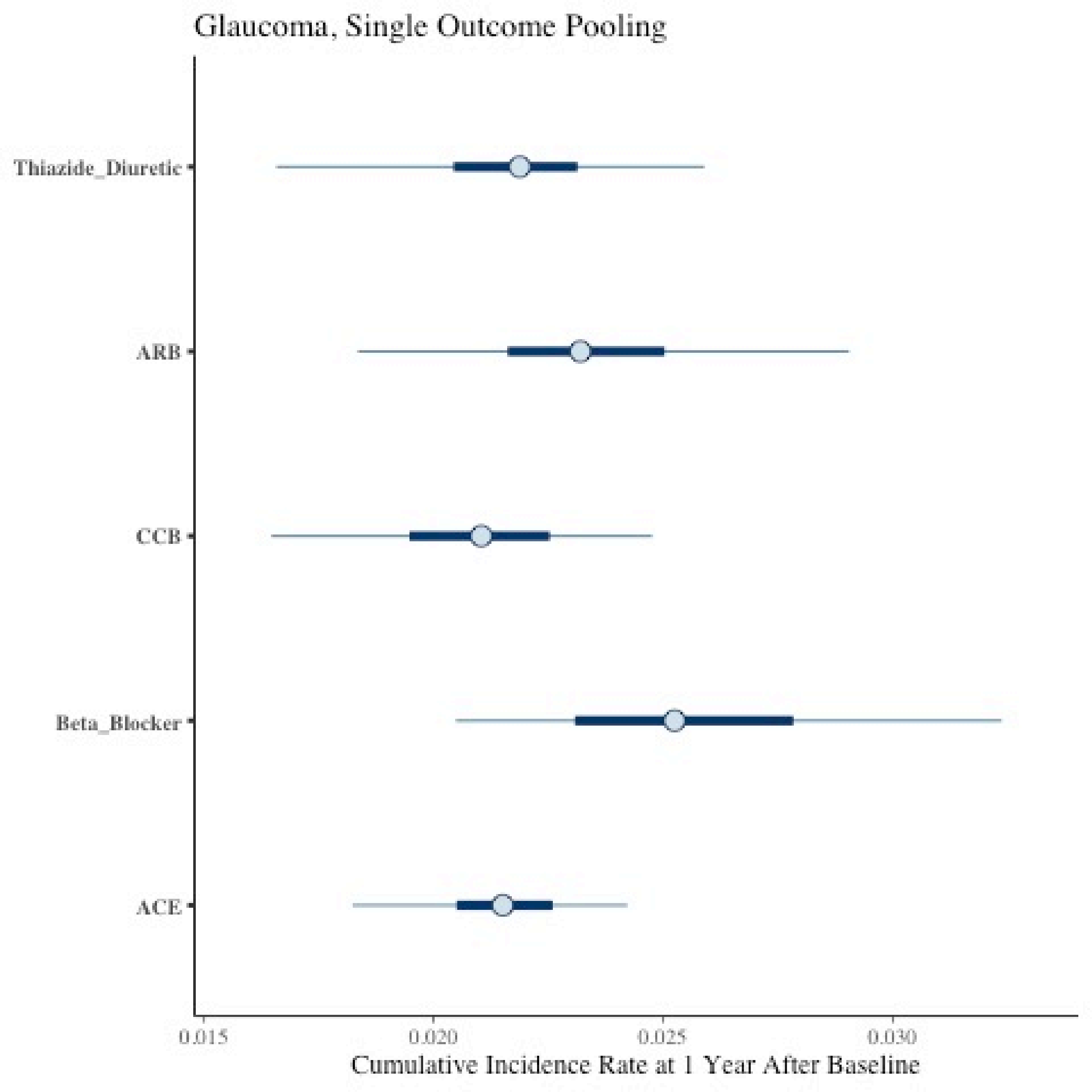
Glaucoma, Single Outcome Pooling
Thiazide_Diuretic
ARB
CCB
Beta_Blocker
ACE
0.015
0.020
0.025
0.030
Cumulative Incidence Rate at 1 Year After Baseline

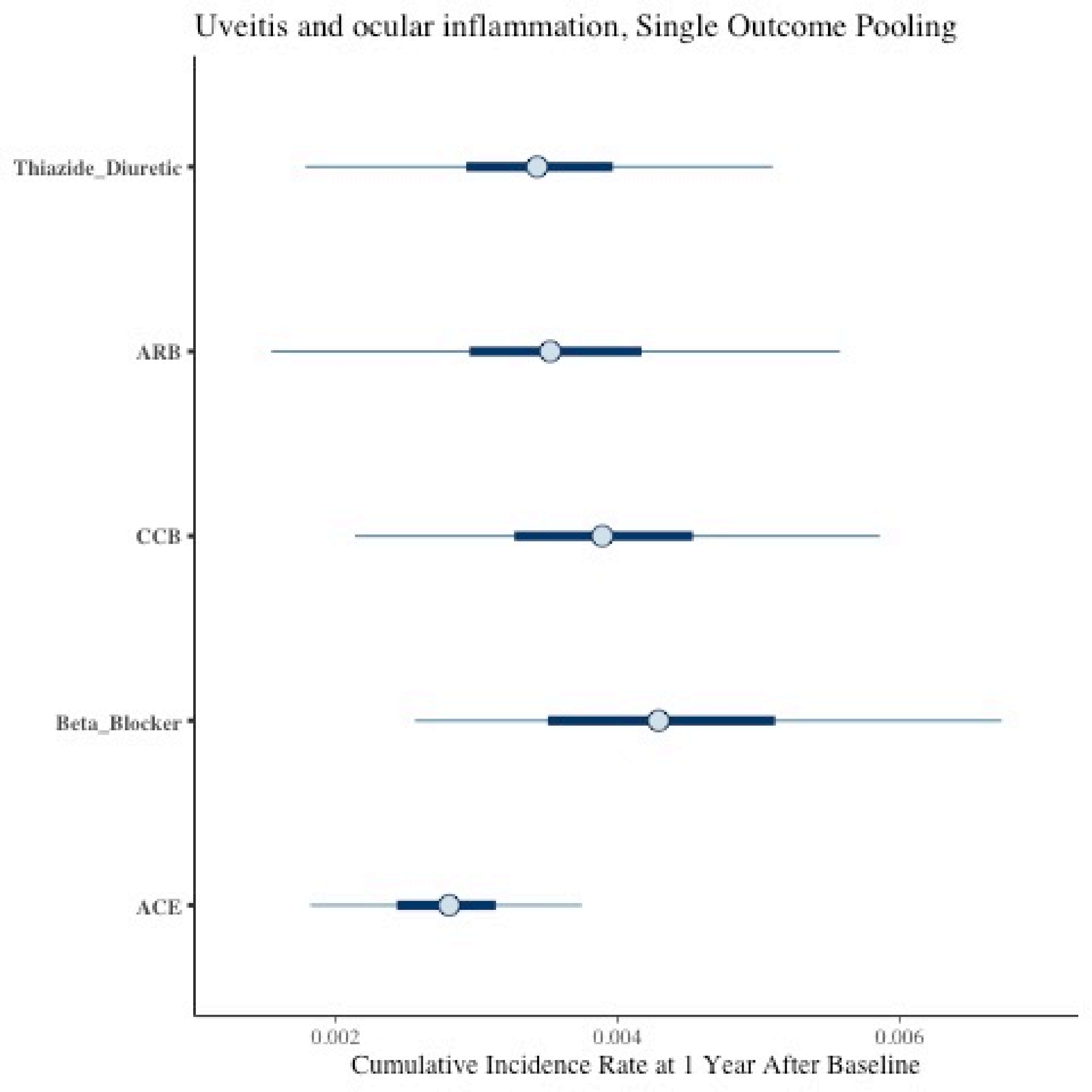
Uveitis and ocular inflammation, Single Outcome Pooling
Thiazide_Diuretic
ARB
CCB
Beta_Blocker
ACE
0.002
0.004
0.006
Cumulative Incidence Rate at 1 Year After Baseline

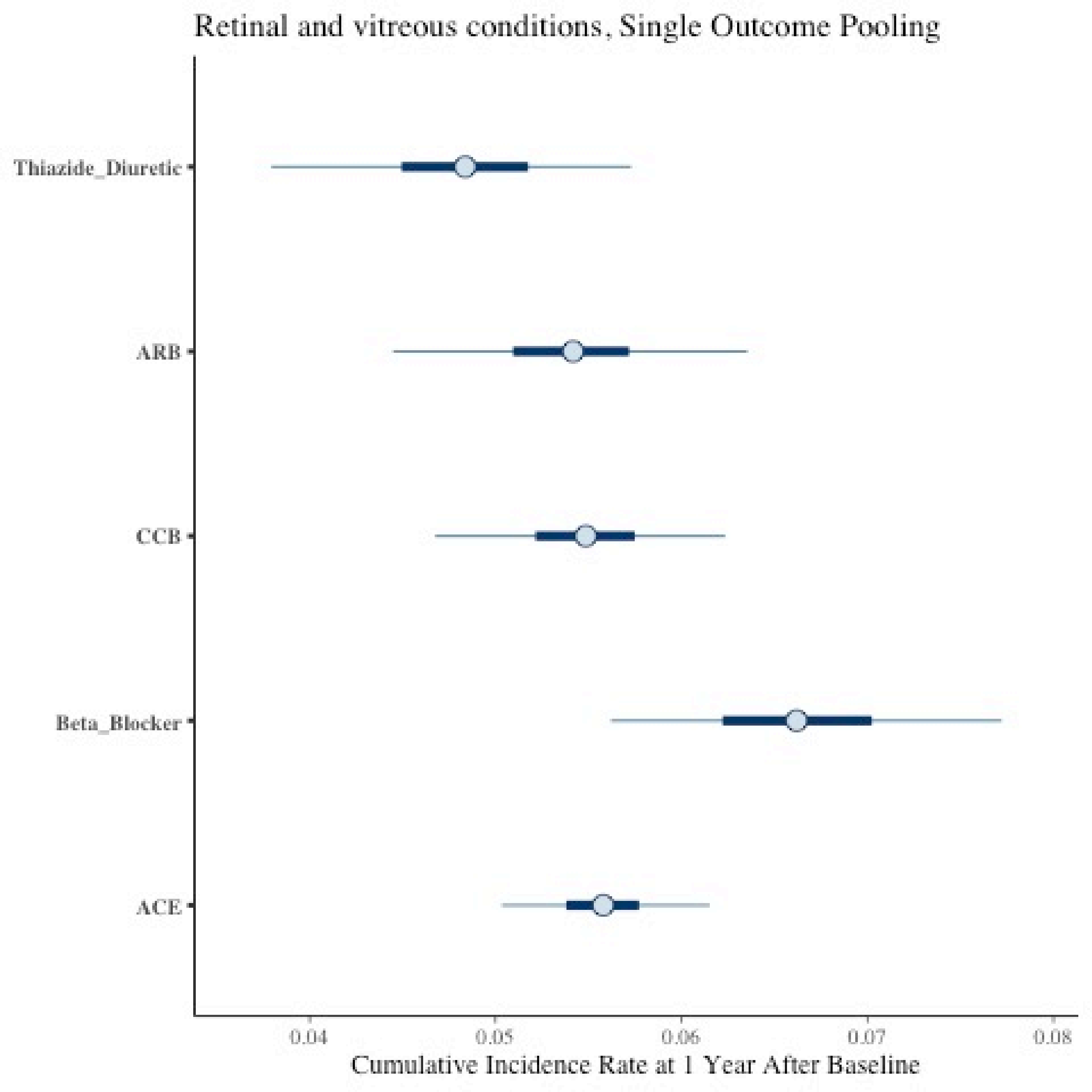
Retinal and vitreous conditions, Single Outcome Pooling
Thiazide_Diuretic
ARB
CCB
Beta_Blocker
ACE
0.04
0.05
0.06
0.07
0.08
Cumulative Incidence Rate at 1 Year After Baseline

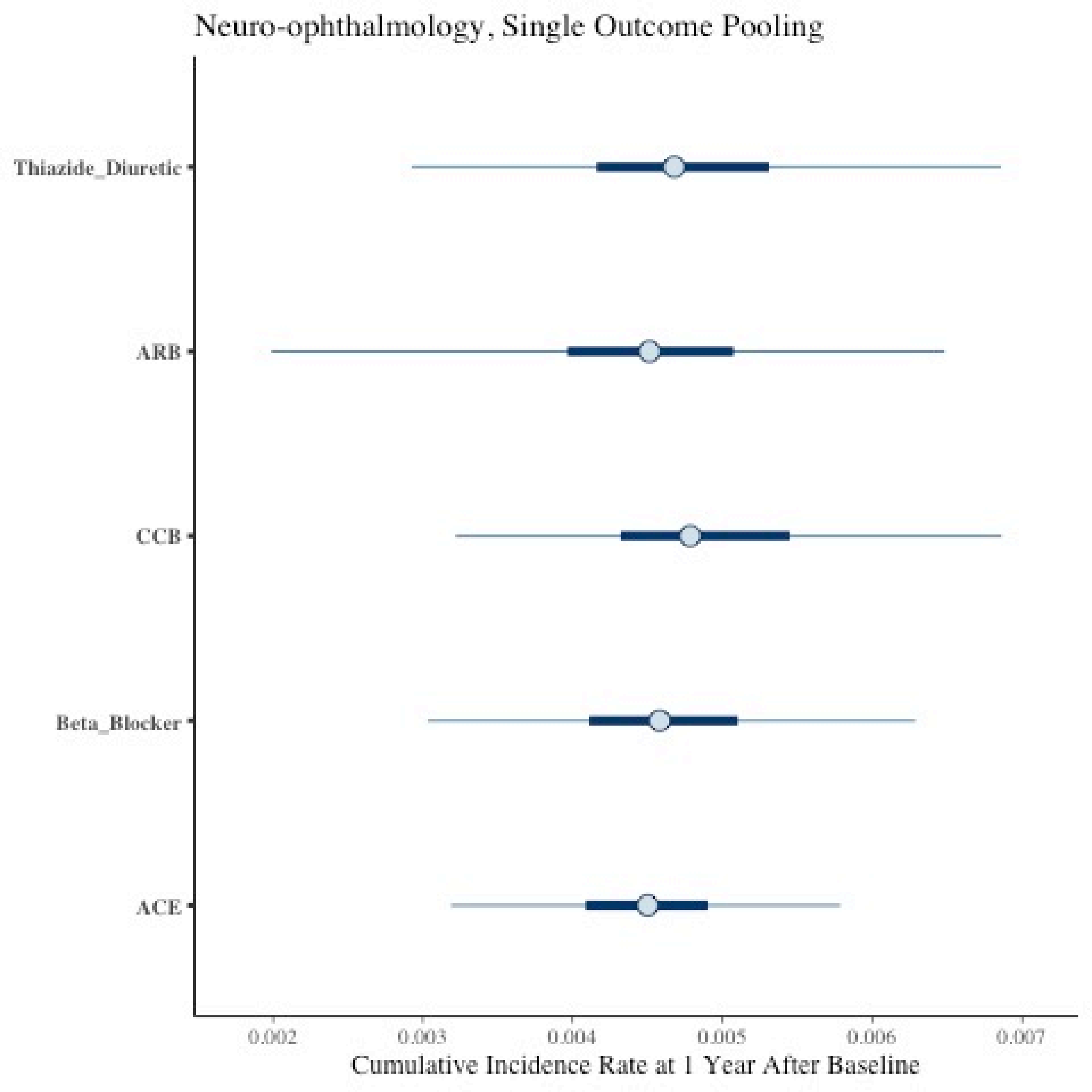
Neuro-ophthalmology, Single Outcome Pooling
Thiazide_Diuretic
ARB
CCB
Beta_Blocker
ACE
0.002
0.003
0.004
0.005
0.006
0.007
Cumulative Incidence Rate at 1 Year After Baseline

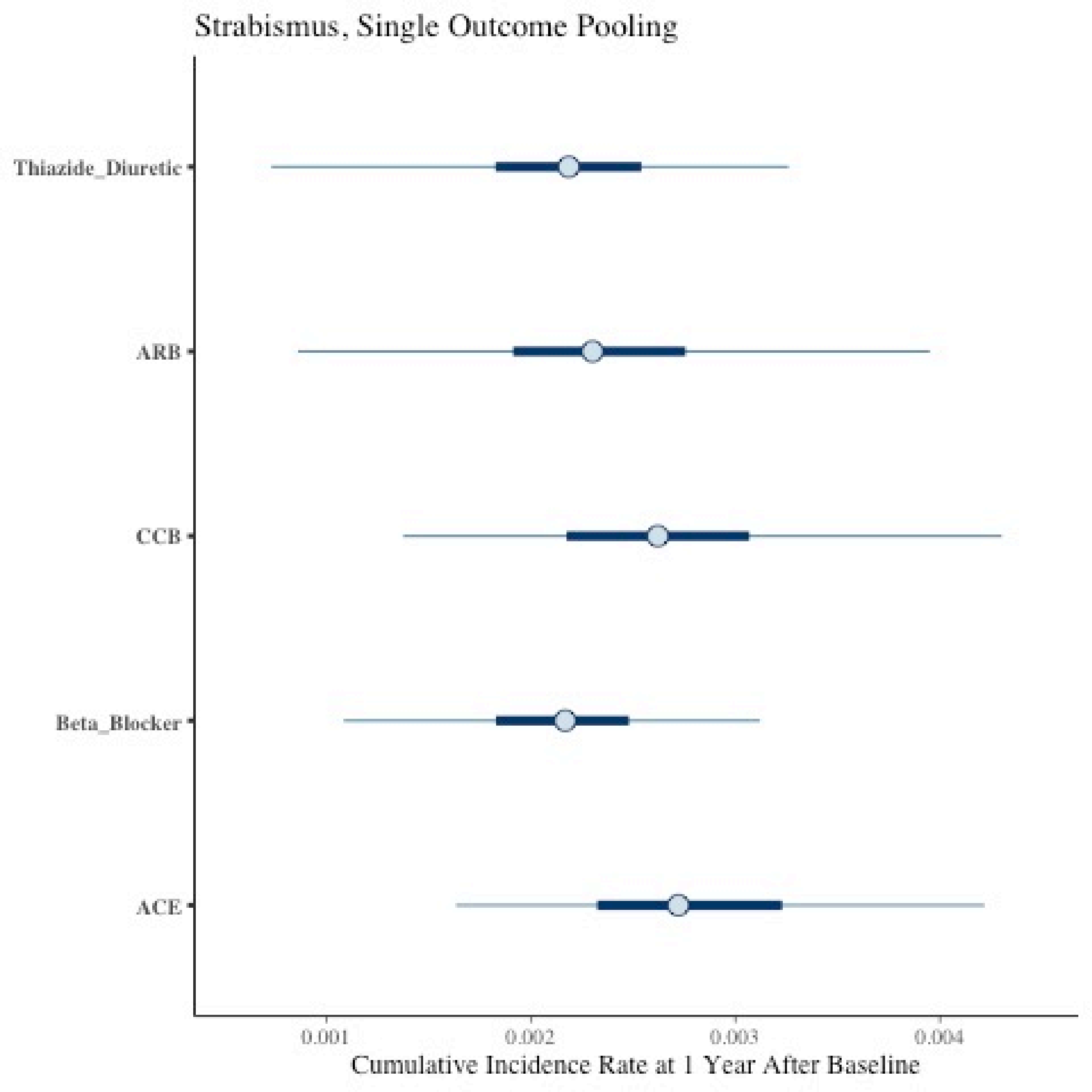

Strabismus, Single Outcome Pooling
Thiazide_Diuretic
ARB
CCB
Beta_Blocker
ACE
0.001
0.002
0.003
0.004
Cumulative Incidence Rate at 1 Year After Baseline

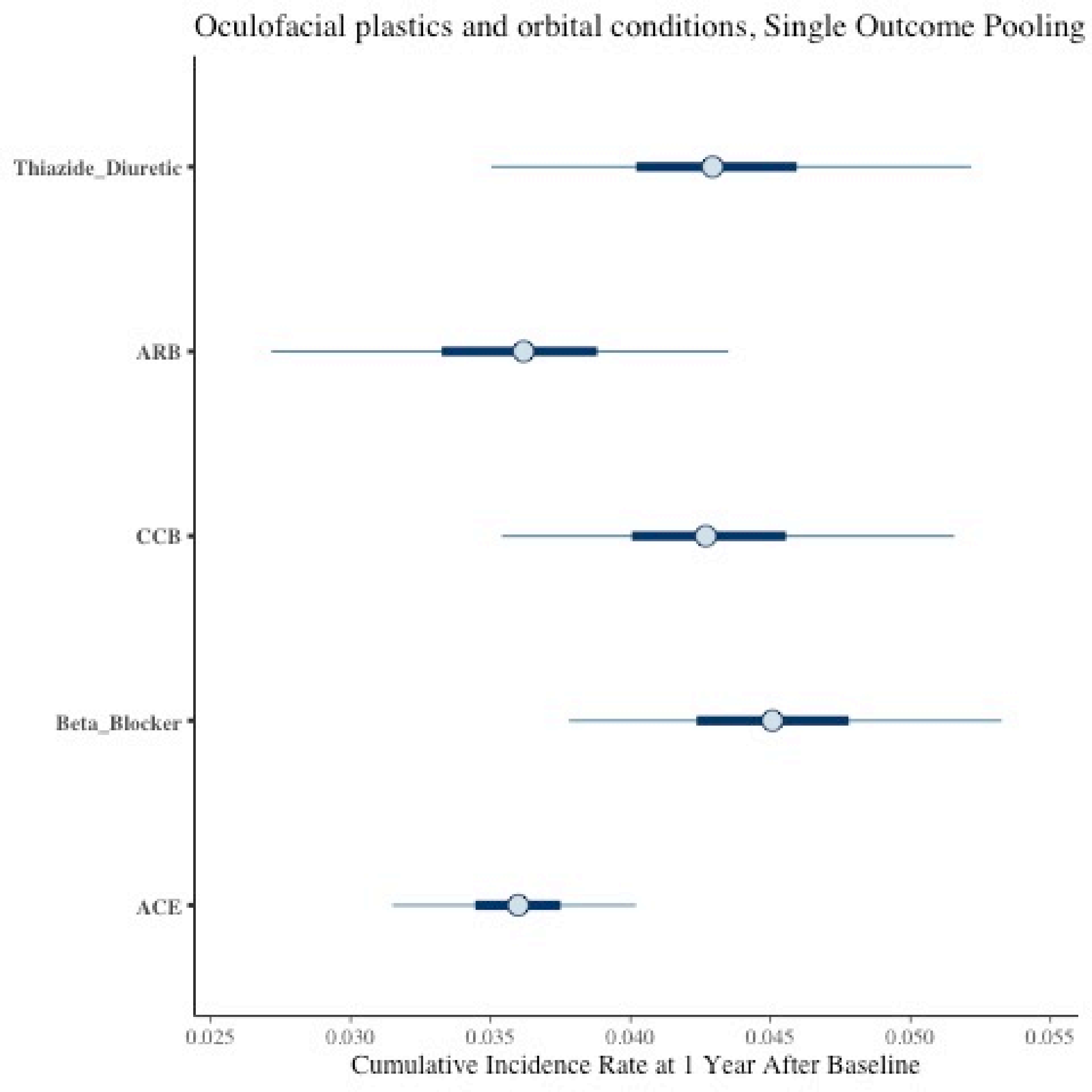

Oculofacial plastics and orbital conditions, Single Outcome Pooling
Thiazide_Diuretic
ARB
CCB
Beta_Blocker
ACE
0.025
0.030
0.035
0.040
0.045
0.050
0.055
Cumulative Incidence Rate at 1 Year After Baseline

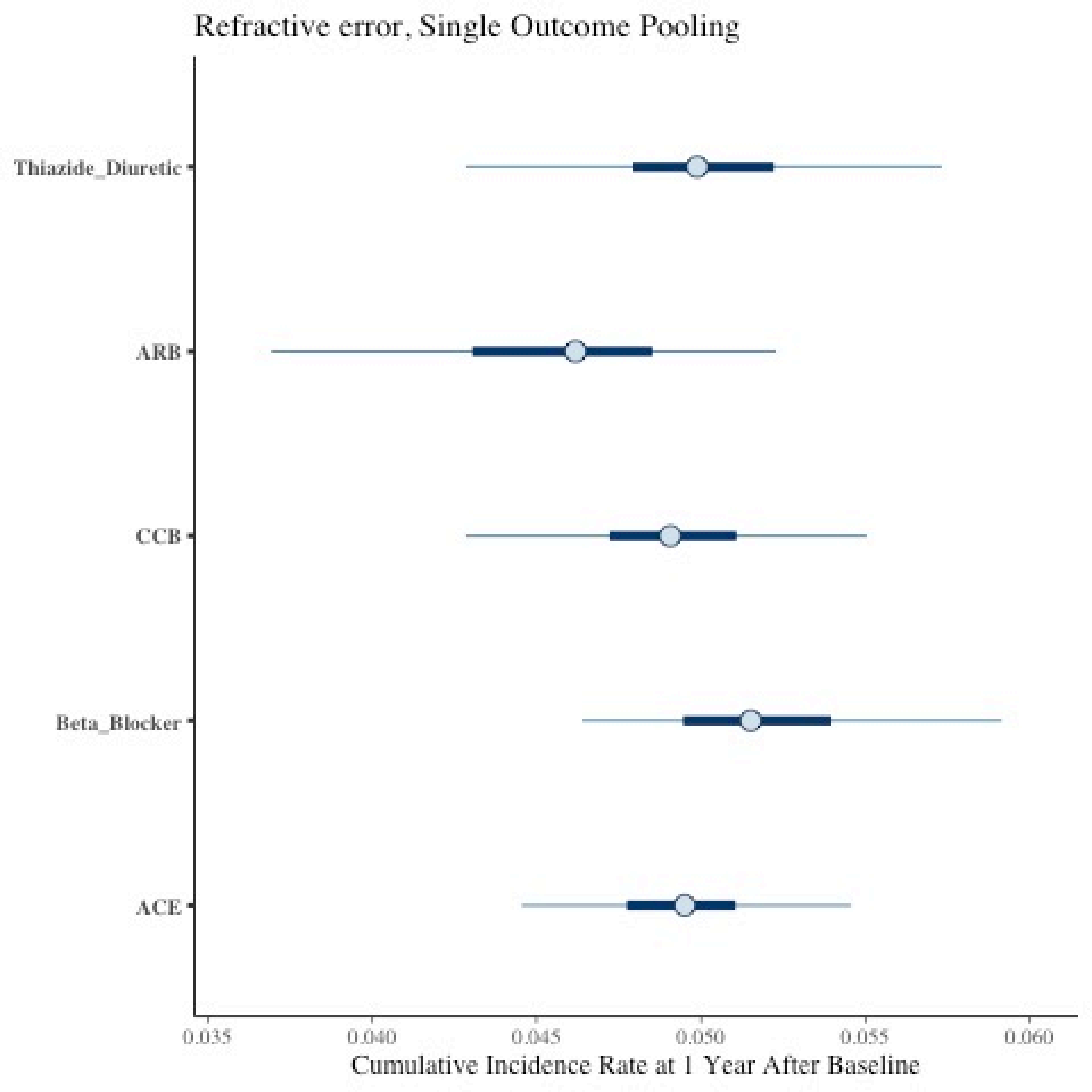

Refractive error, Single Outcome Pooling
Thiazide_Diuretic
ARB
CCB
Beta_Blocker
ACE
0.035
0.040
0.045
0.050
0.055
0.060
Cumulative Incidence Rate at 1 Year After Baseline

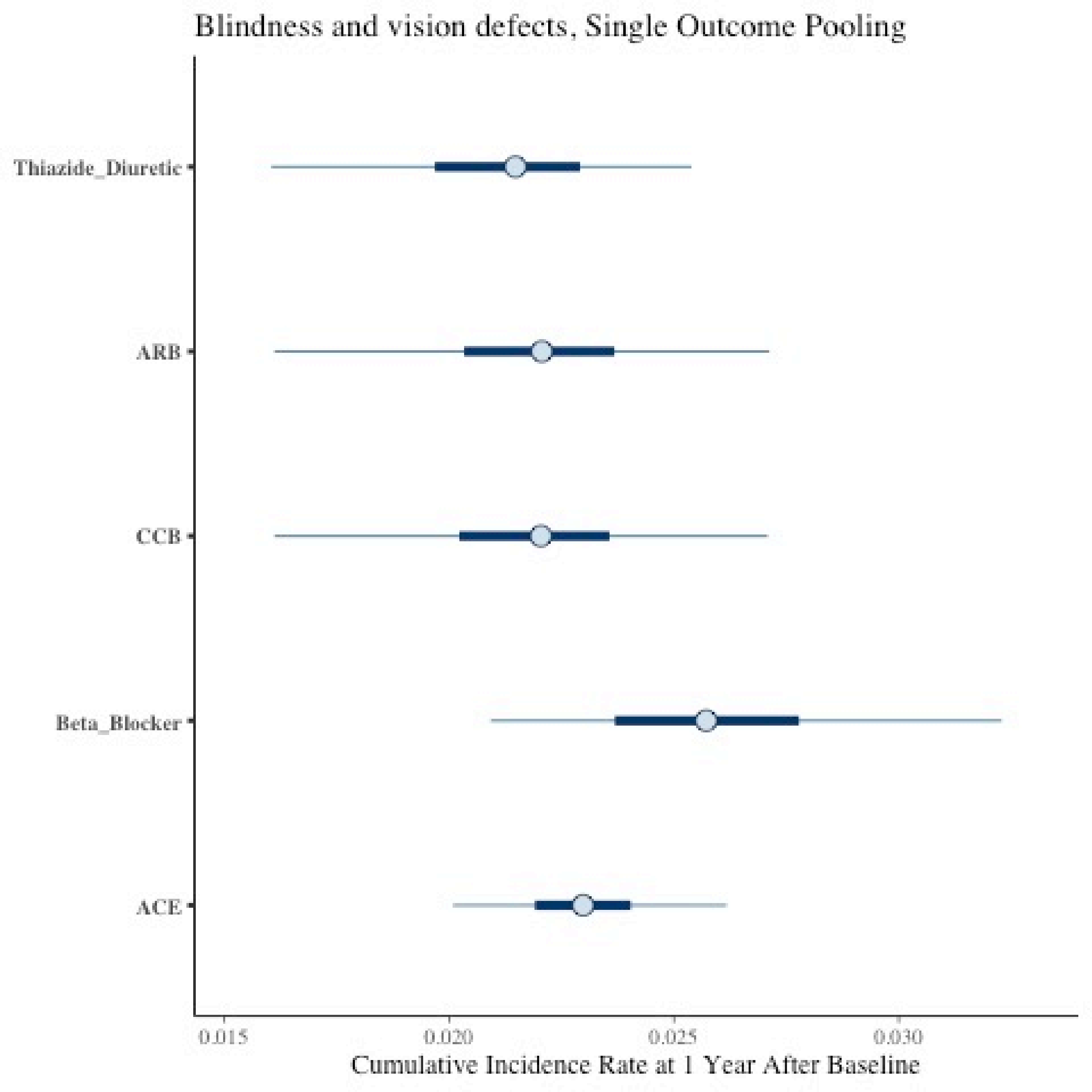
Blindness and vision defects, Single Outcome Pooling
Thiazide_Diuretic
ARB
CCB
Beta_Blocker
ACE
0.015
0.020
0.025
0.030
Cumulative Incidence Rate at 1 Year After Baseline

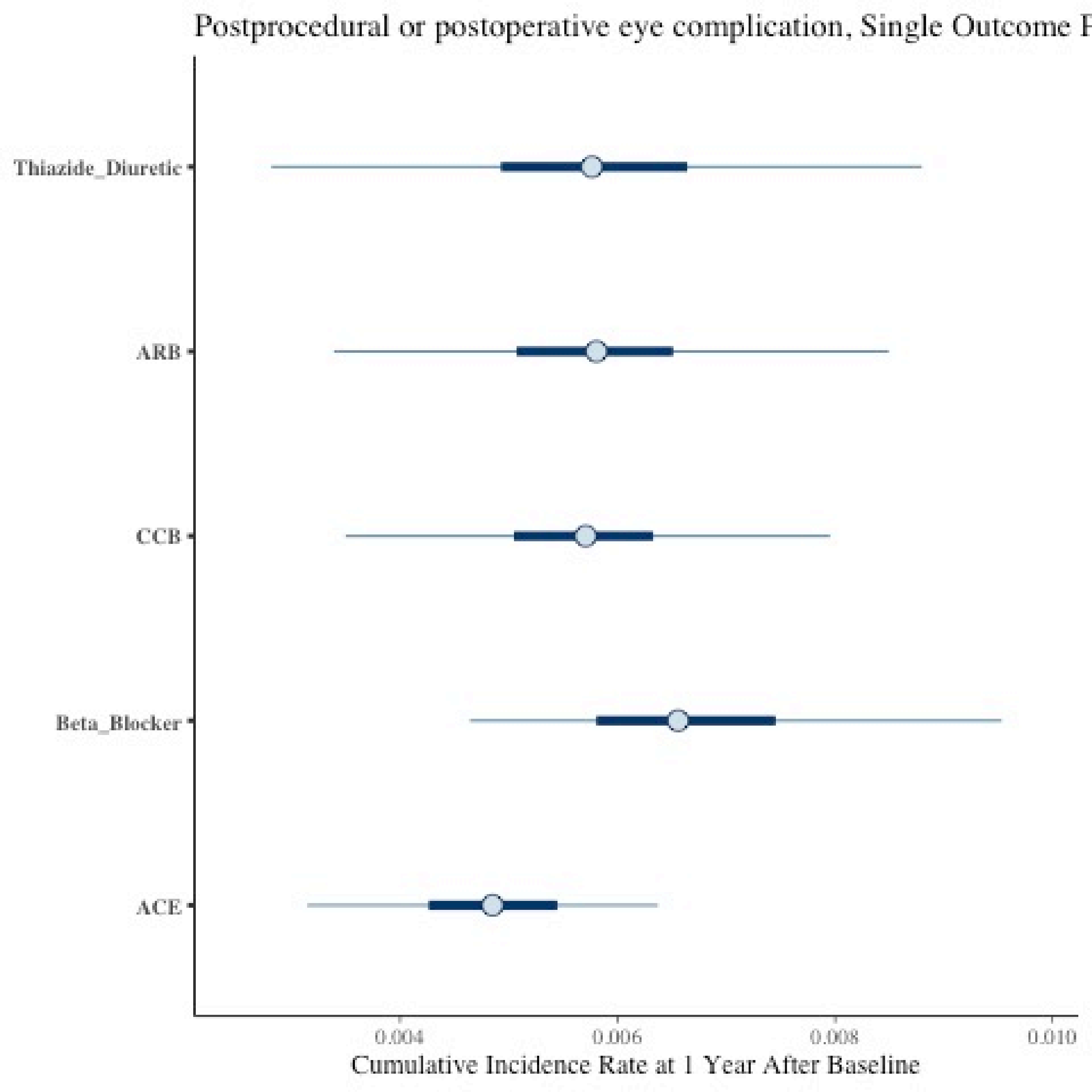

Postprocedural or postoperative eye complication, Single Outcome F
Thiazide_Diuretic
ARB
CCB
Beta_Blocker
ACE
0.004
0.006
0.008
0.010
Cumulative Incidence Rate at 1 Year After Baseline

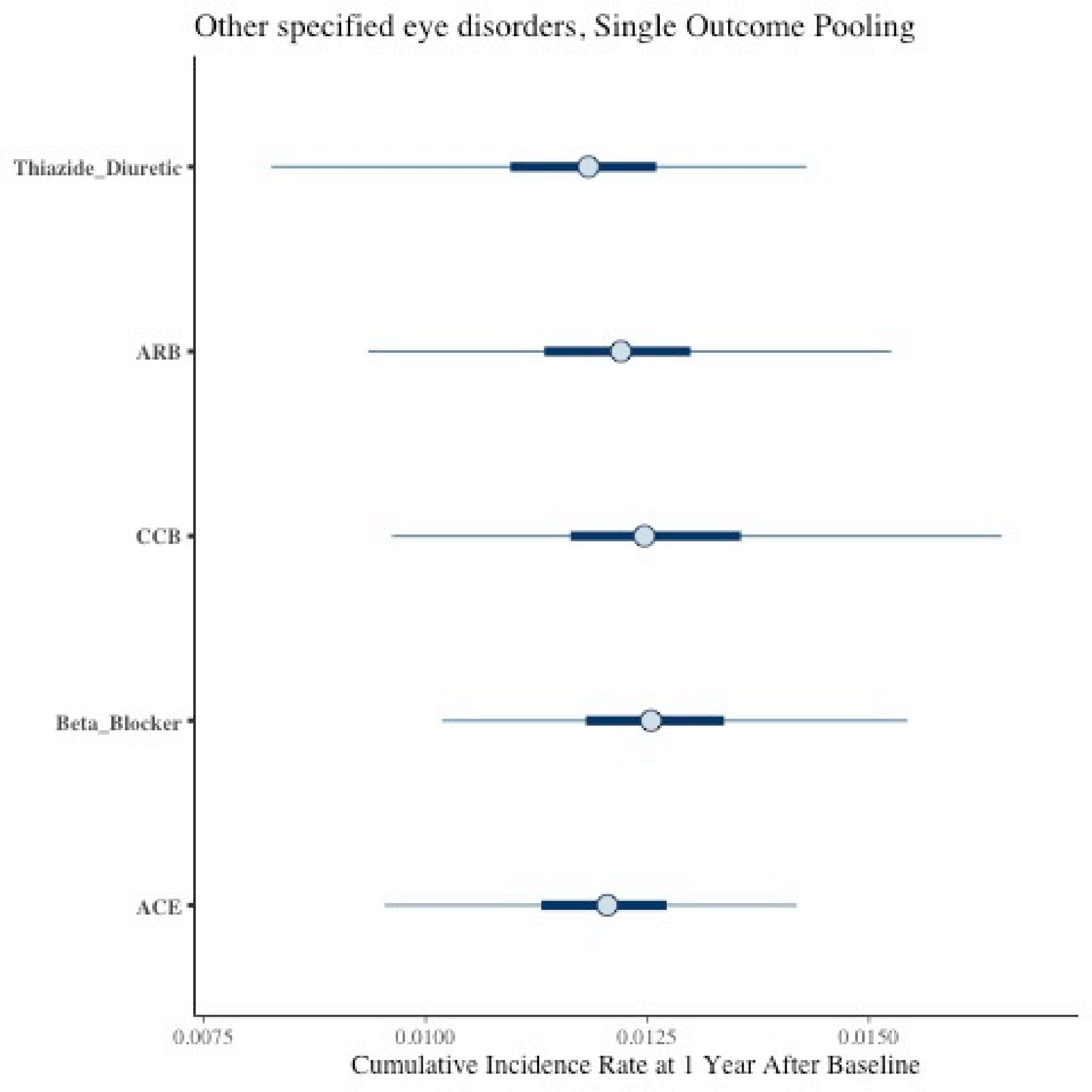

Other specified eye disorders, Single Outcome Pooling
Thiazide_Diuretic
ARB
CCB
Beta_Blocker
ACE
0.0075
0.0100
0.0125
0.0150
Cumulative Incidence Rate at 1 Year After Baseline

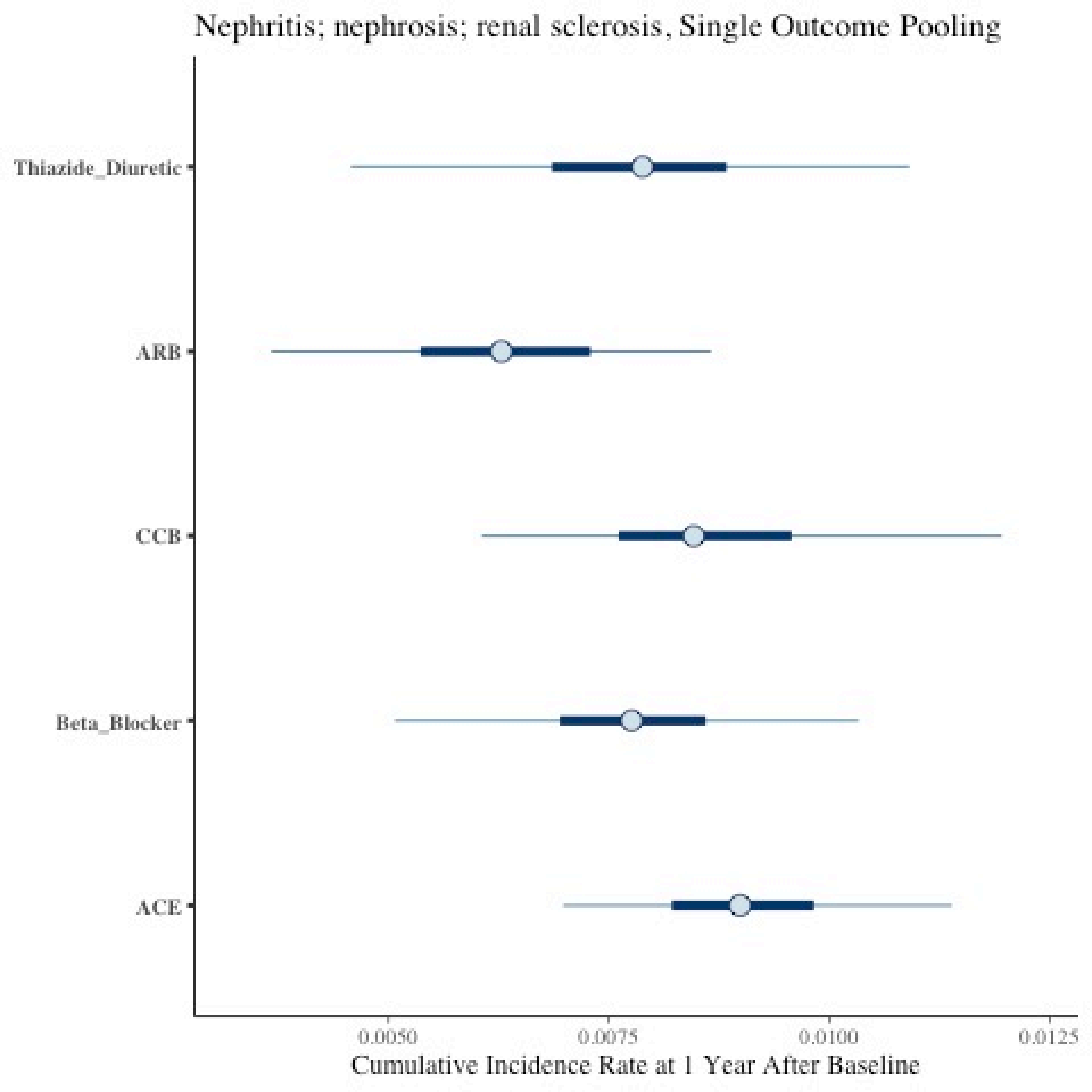
Nephritis; nephrosis; renal sclerosis, Single Outcome Pooling
Thiazide_Diuretic
ARB
CCB
Beta_Blocker
ACE
0.0050
0.0075
0.0100
0.0125
Cumulative Incidence Rate at 1 Year After Baseline

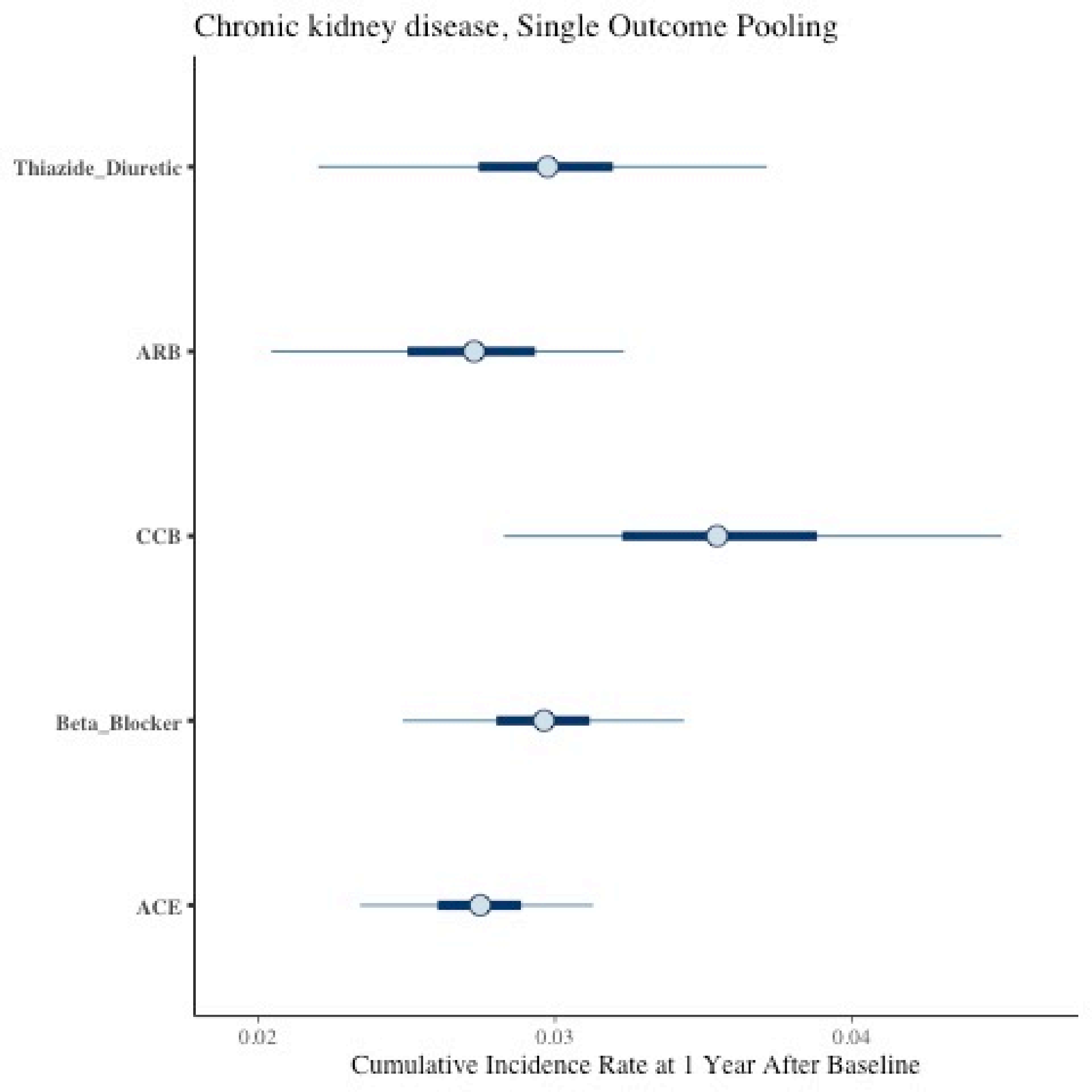

Chronic kidney disease, Single Outcome Pooling
Thiazide_Diuretic
ARB
CCB
Beta_Blocker
ACE
0.02
0.03
0.04
Cumulative Incidence Rate at 1 Year After Baseline

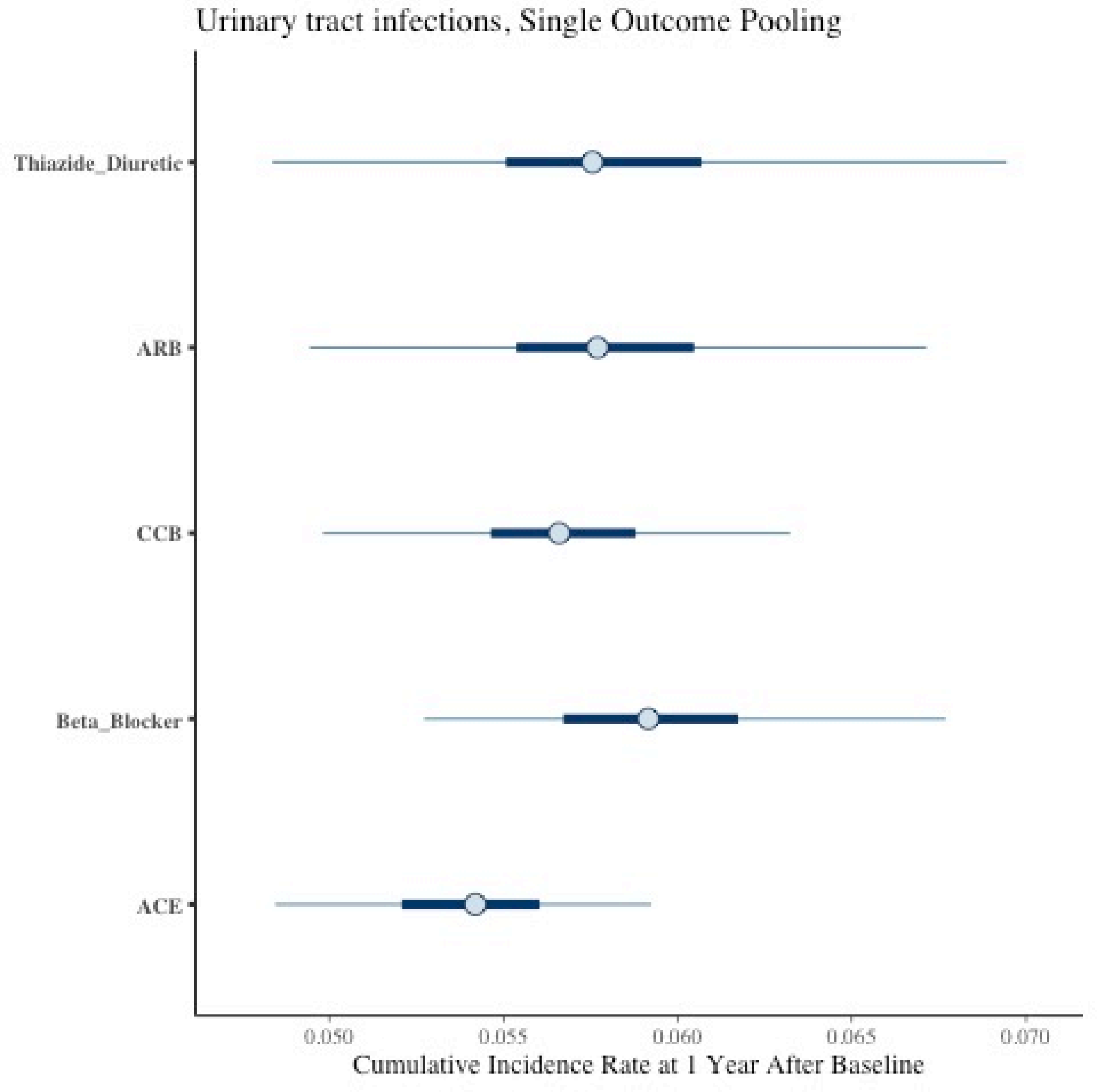

Urinary tract infections, Single Outcome Pooling
Thiazide_Diuretic
ARB
CCB
Beta_Blocker
ACE
0.050
0.055
0.060
0.065
0.070
Cumulative Incidence Rate at 1 Year After Baseline

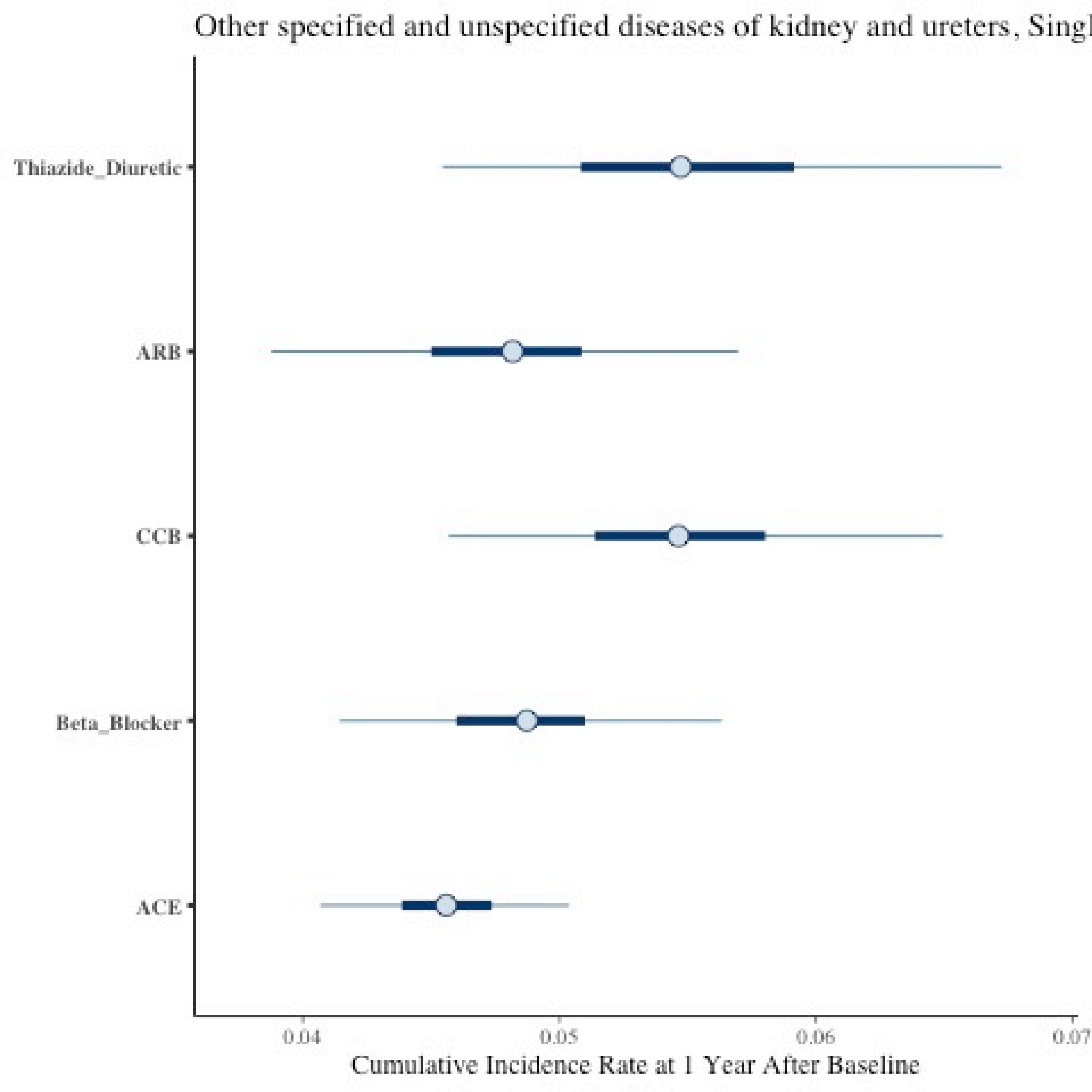

Other specified and unspecified diseases of kidney and ureters, Singl
Thiazide_Diuretic
ARB
CCB
Beta_Blocker
ACE
0.04
0.05
0.06
0.07
Cumulative Incidence Rate at 1 Year After Baseline

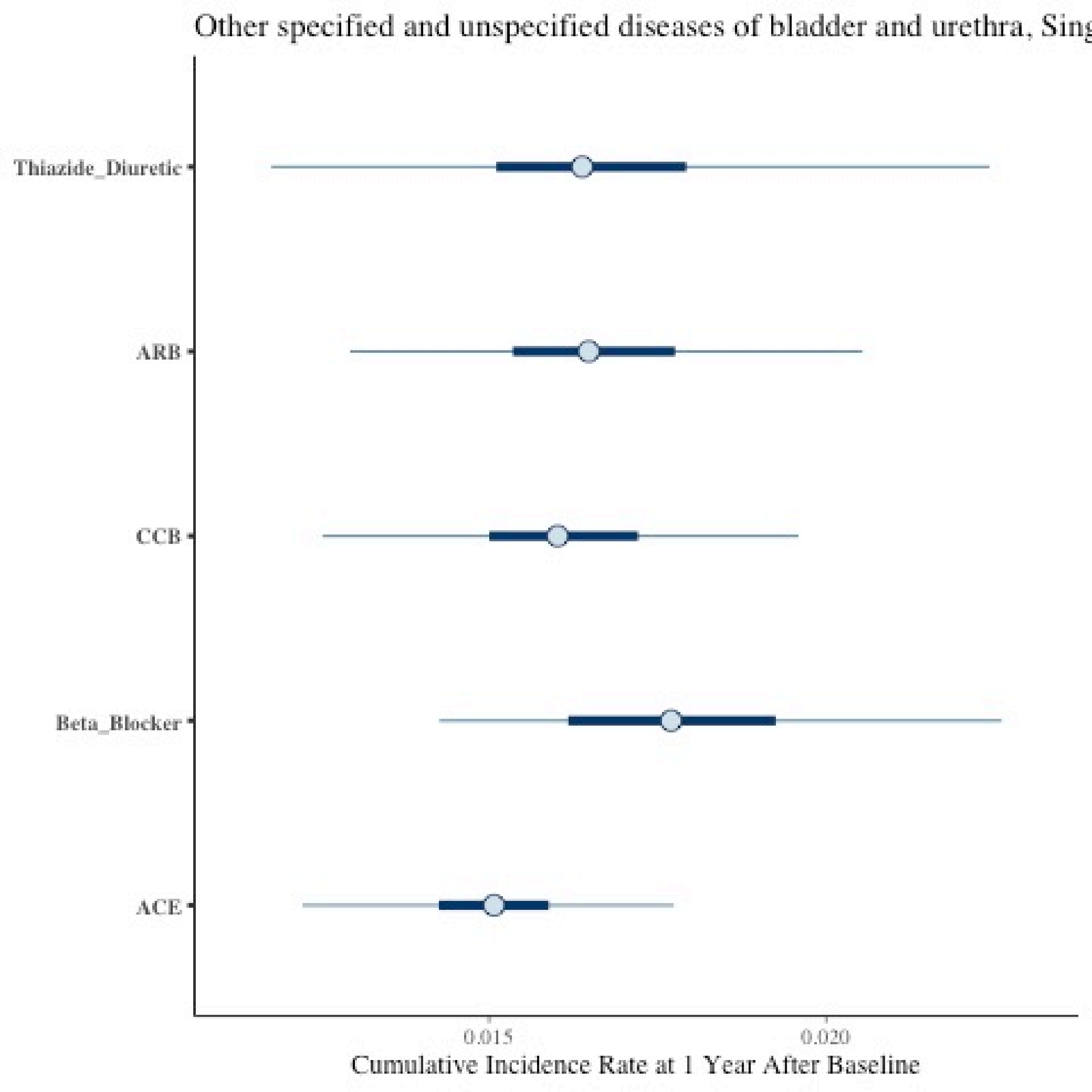
Other specified and unspecified diseases of bladder and urethra, Sing
Thiazide_Diuretic
ARB
CCB
Beta_Blocker
ACE
0.015
0.020
Cumulative Incidence Rate at 1 Year After Baseline

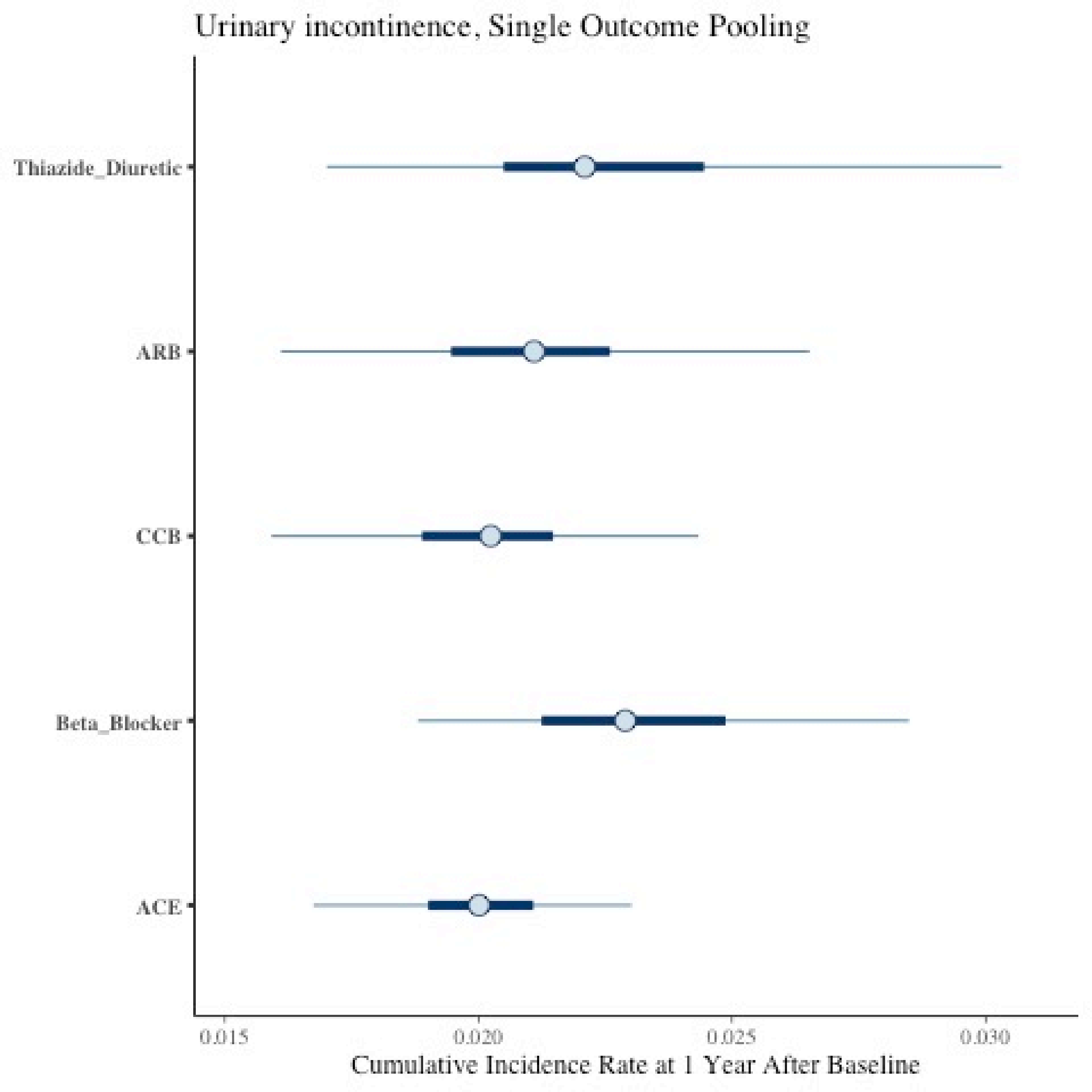

Urinary incontinence, Single Outcome Pooling
Thiazide_Diuretic
ARB
CCB
Beta_Blocker
ACE
0.015
0.020
0.025
0.030
Cumulative Incidence Rate at 1 Year After Baseline

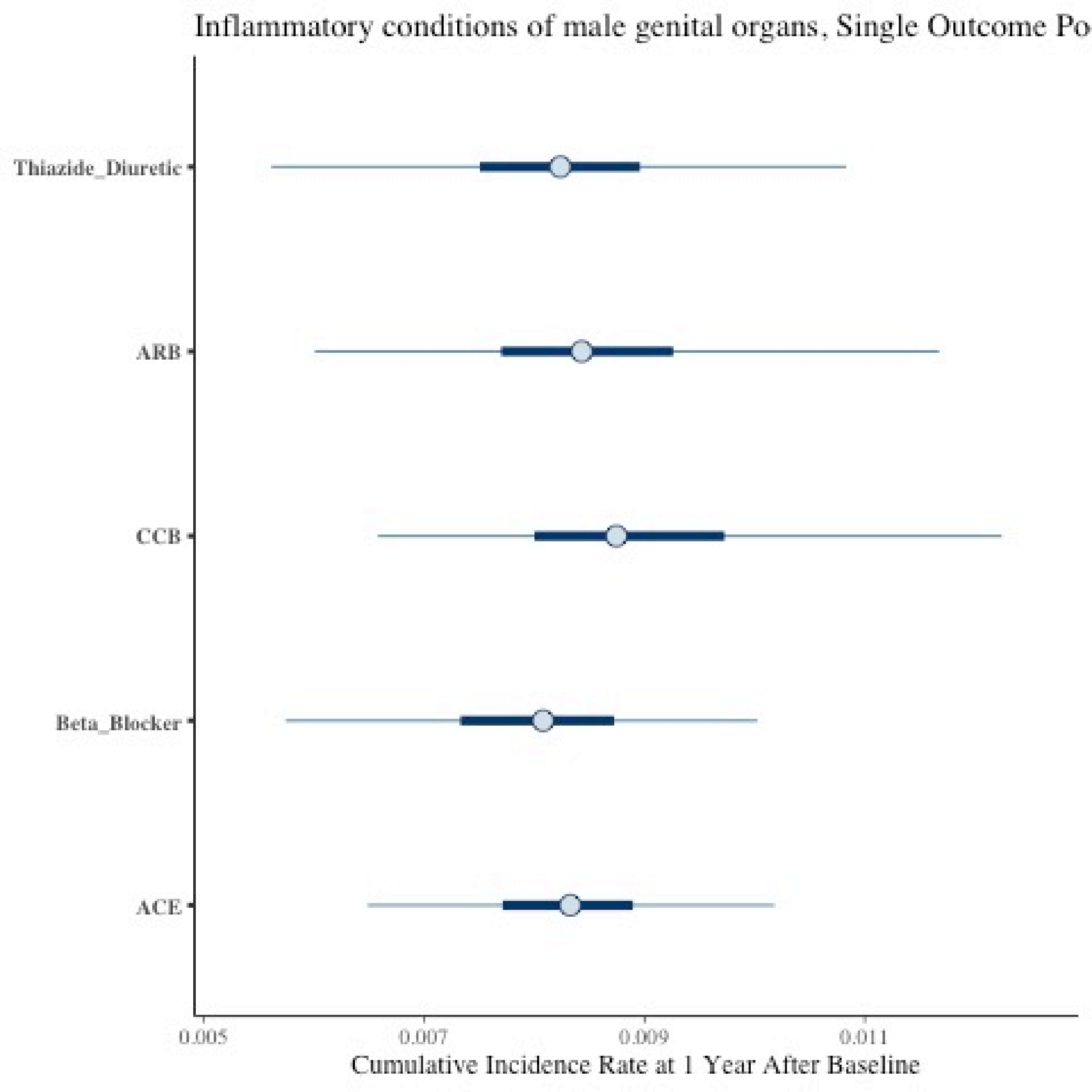
Inflammatory conditions of male genital organs, Single Outcome Po
Thiazide_Diuretic
ARB
CCB
Beta_Blocker
ACE
0.005
0.007
0.009
0.011
Cumulative Incidence Rate at 1 Year After Baseline

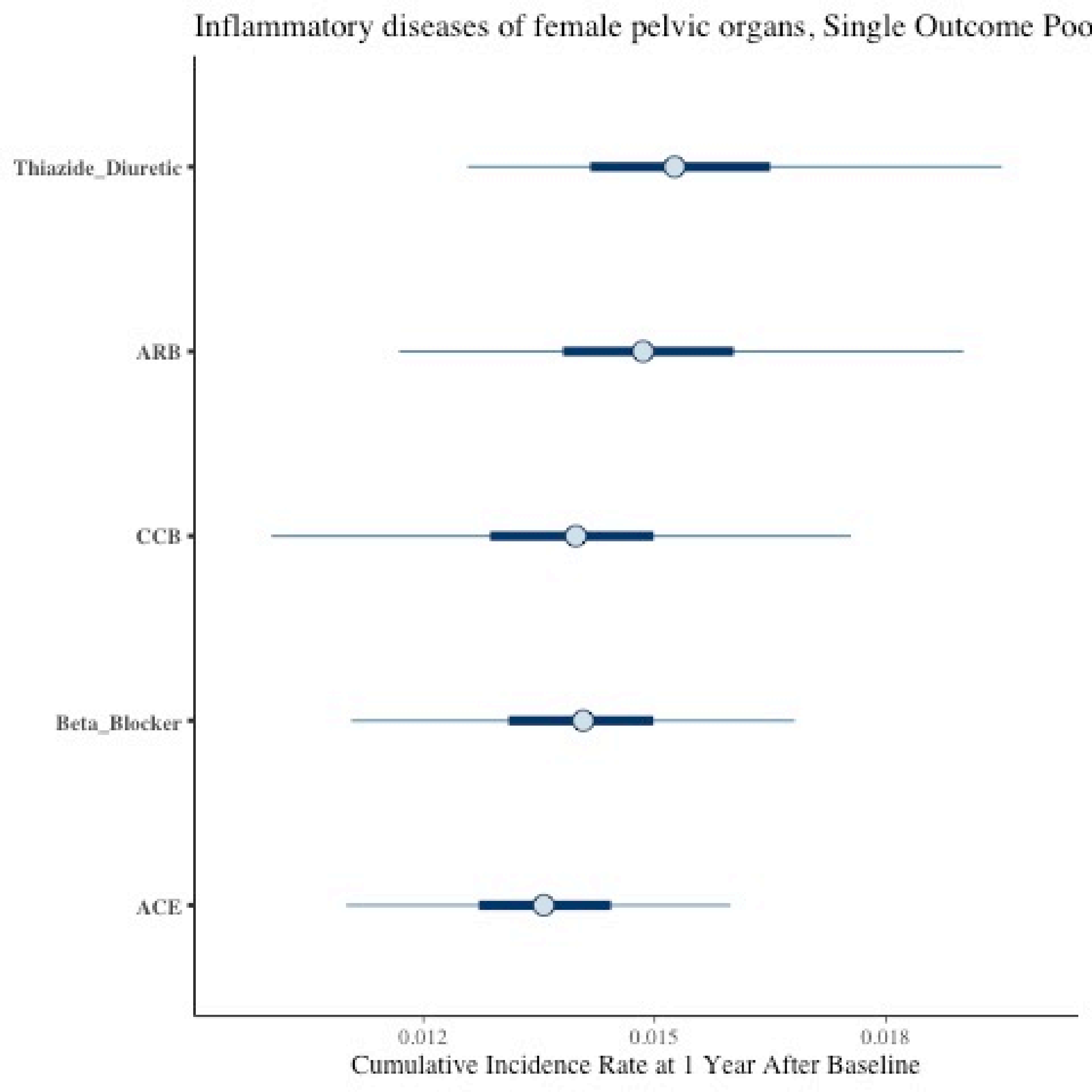
Inflammatory diseases of female pelvic organs, Single Outcome Poo
Thiazide_Diuretic
ARB
CCB
Beta_Blocker
ACE
0.012
0.015
0.018
Cumulative Incidence Rate at 1 Year After Baseline

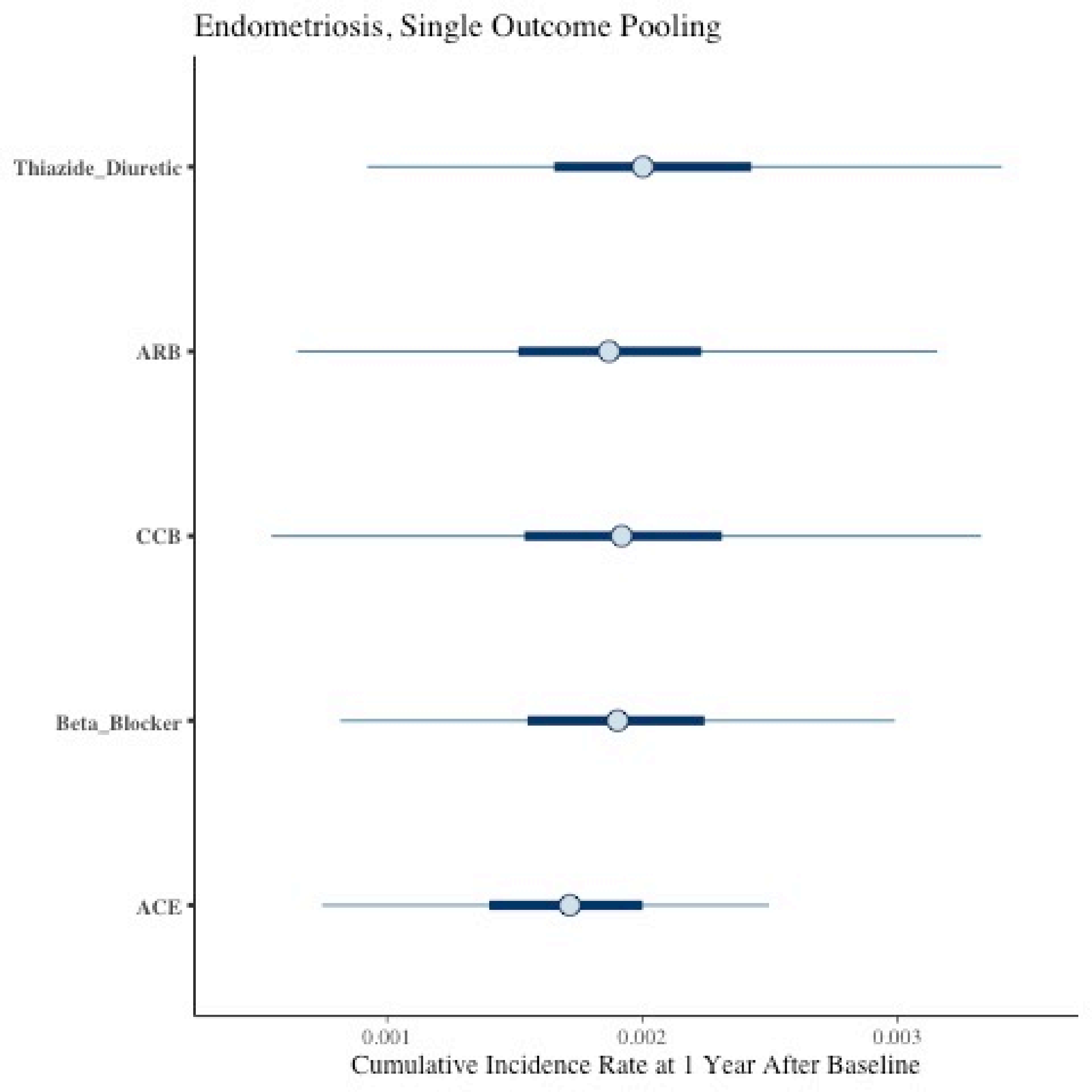
Endometriosis, Single Outcome Pooling
Thiazide_Diuretic
ARB
CCB
Beta_Blocker
ACE
0.001
0.002
0.003
Cumulative Incidence Rate at 1 Year After Baseline

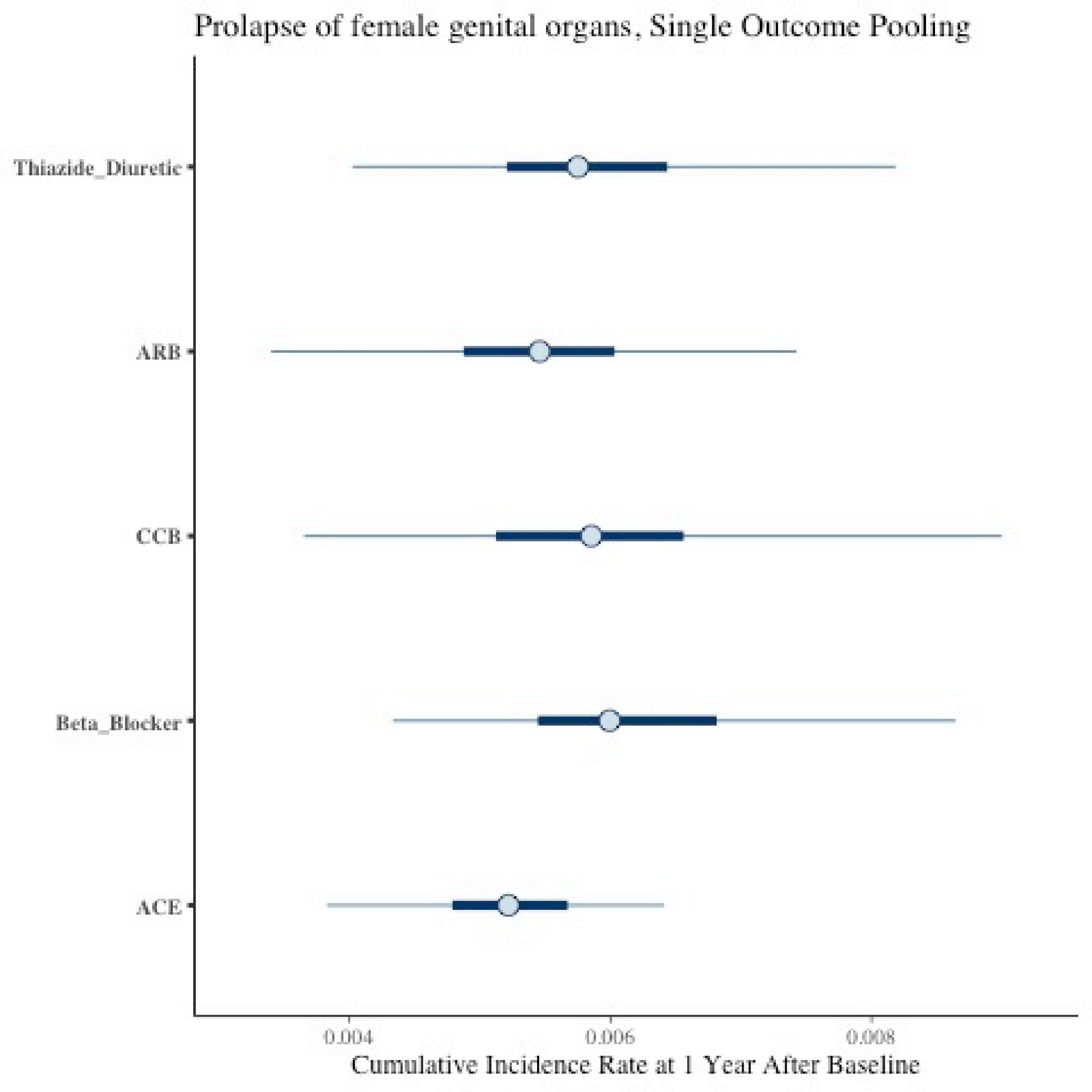

Prolapse of female genital organs, Single Outcome Pooling
Thiazide_Diuretic
ARB
CCB
Beta_Blocker
ACE
0.004
0.006
0.008
Cumulative Incidence Rate at 1 Year After Baseline

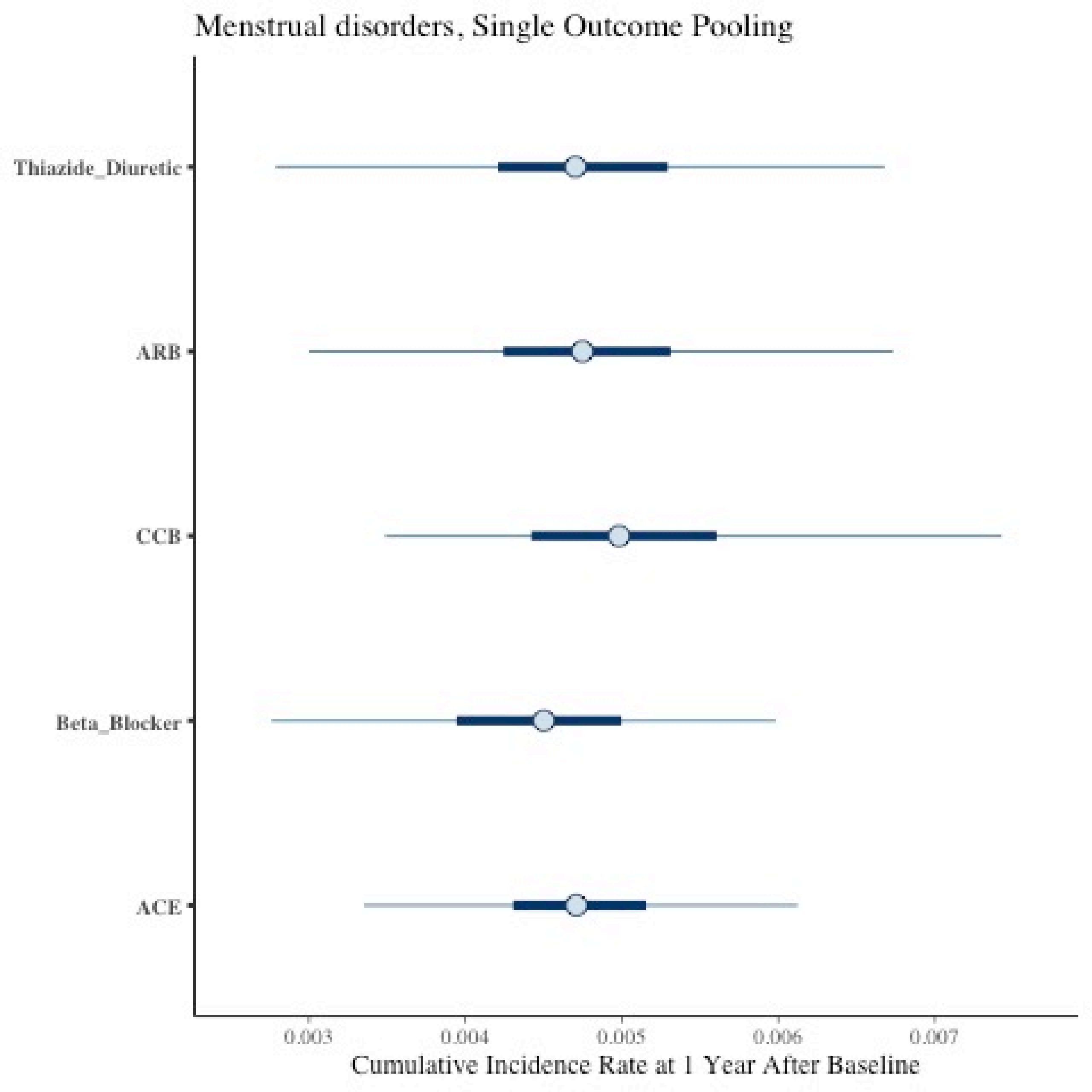

Menstrual disorders, Single Outcome Pooling
Thiazide_Diuretic
ARB
CCB
Beta_Blocker
ACE
0.003
0.004
0.005
0.006
0.007
Cumulative Incidence Rate at 1 Year After Baseline

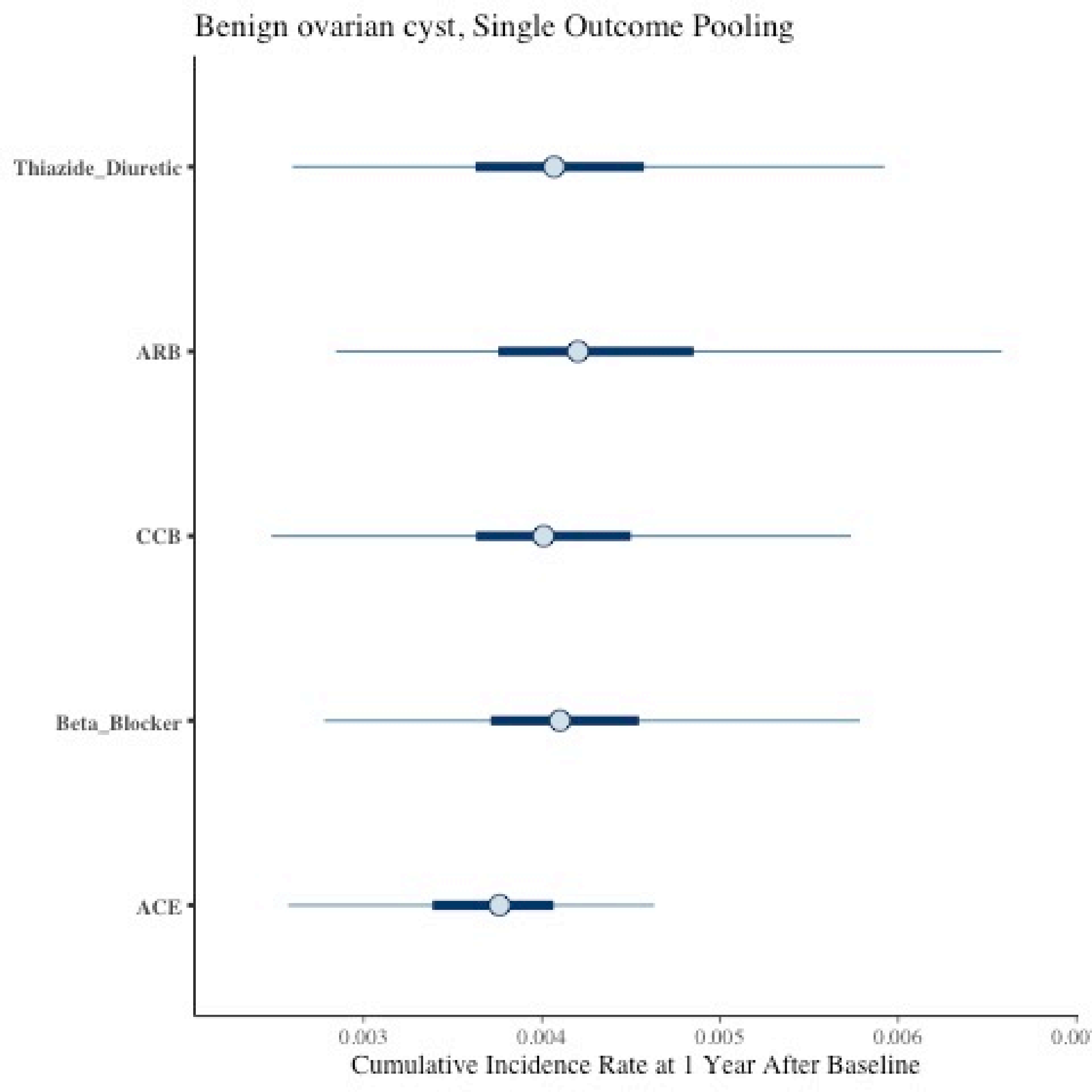

Benign ovarian cyst, Single Outcome Pooling
Thiazide_Diuretic
ARB
CCB
Beta_Blocker
ACE
0.003
0.004
0.005
0.006
Cumulative Incidence Rate at 1 Year After Baseline

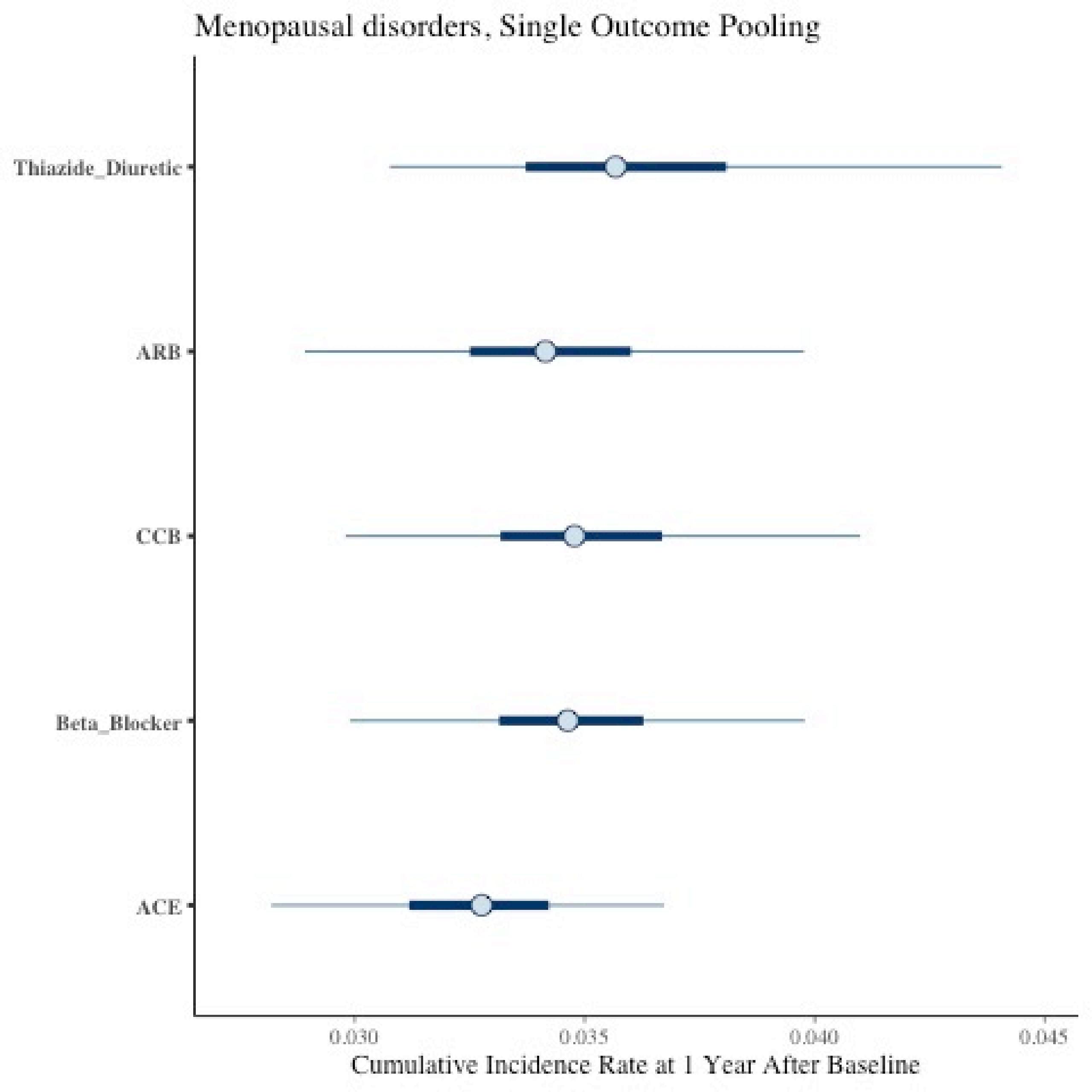

Menopausal disorders, Single Outcome Pooling
Thiazide_Diuretic
ARB
CCB
Beta_Blocker
ACE
0.030
0.035
0.040
0.045
Cumulative Incidence Rate at 1 Year After Baseline

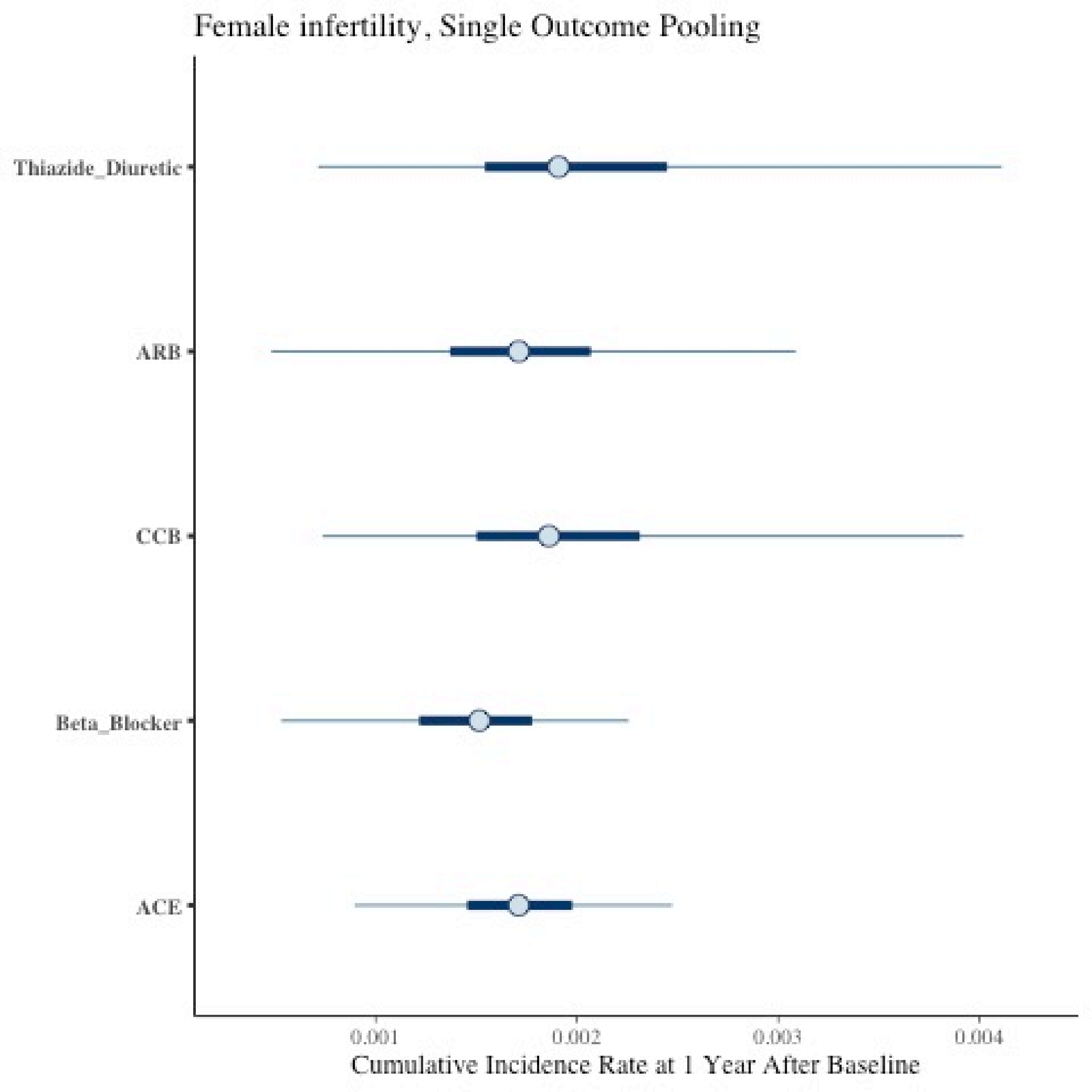
Female infertility, Single Outcome Pooling
Thiazide_Diuretic
ARB
CCB
Beta_Blocker
ACE
0.001
0.002
0.003
0.004
Cumulative Incidence Rate at 1 Year After Baseline

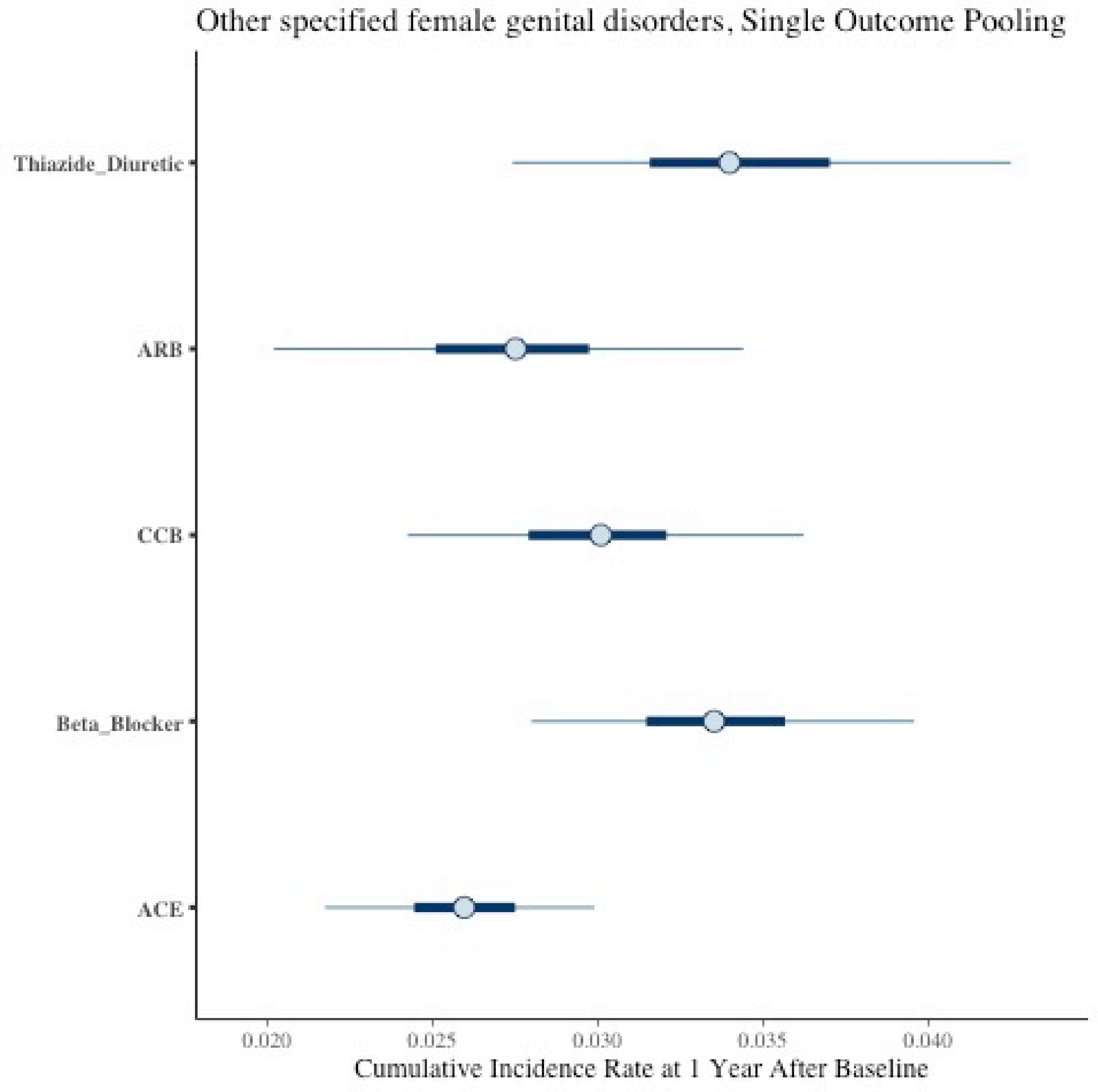

Other specified female genital disorders, Single Outcome Pooling
Thiazide_Diuretic
ARB
CCB
Beta_Blocker
ACE
0.020
0.025
0.030
0.035
0.040
Cumulative Incidence Rate at 1 Year After Baseline

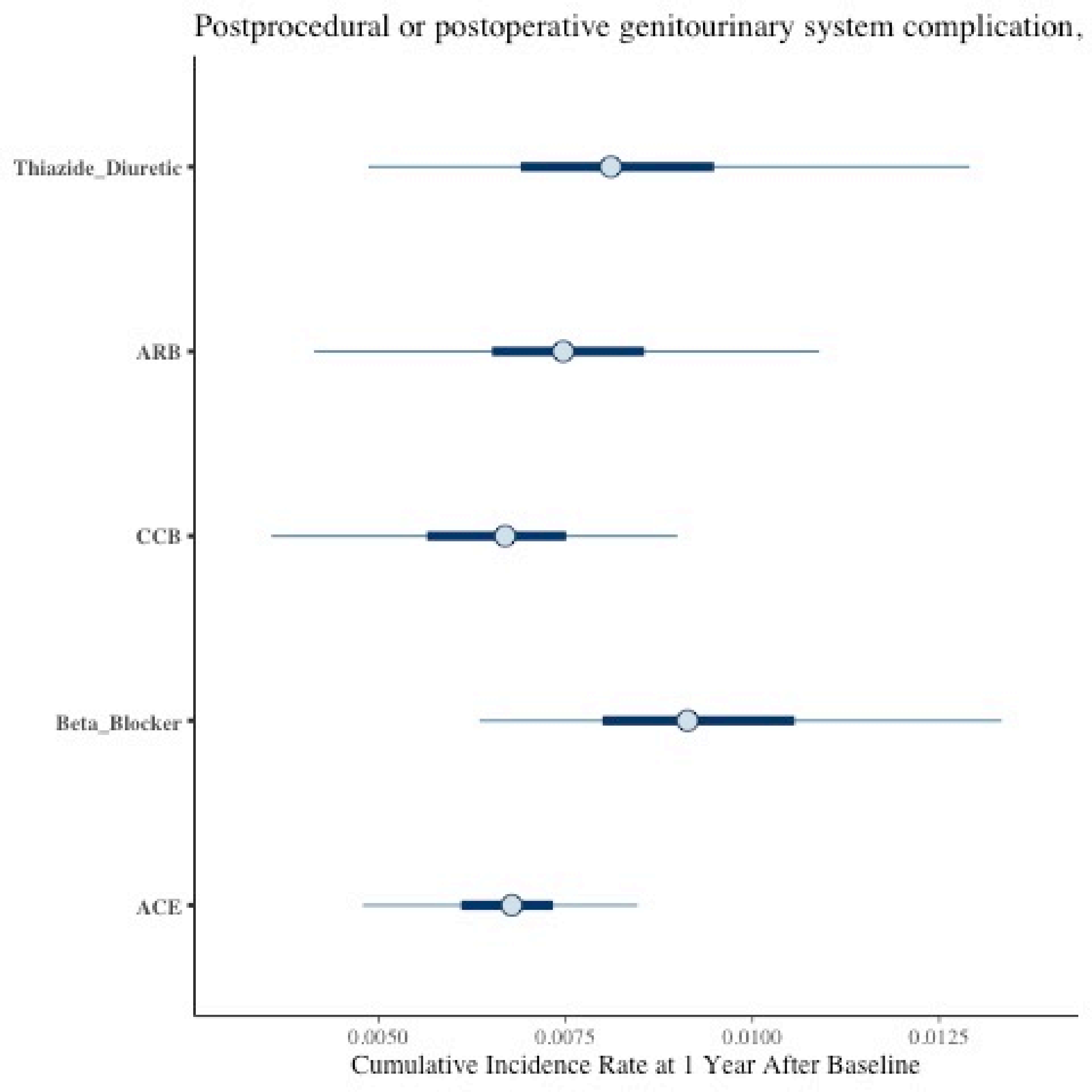
Postprocedural or postoperative genitourinary system complication,
Thiazide_Diuretic
ARB
CCB
Beta_Blocker
ACE
0.0050
0.0075
0.0100
0.0125
Cumulative Incidence Rate at 1 Year After Baseline

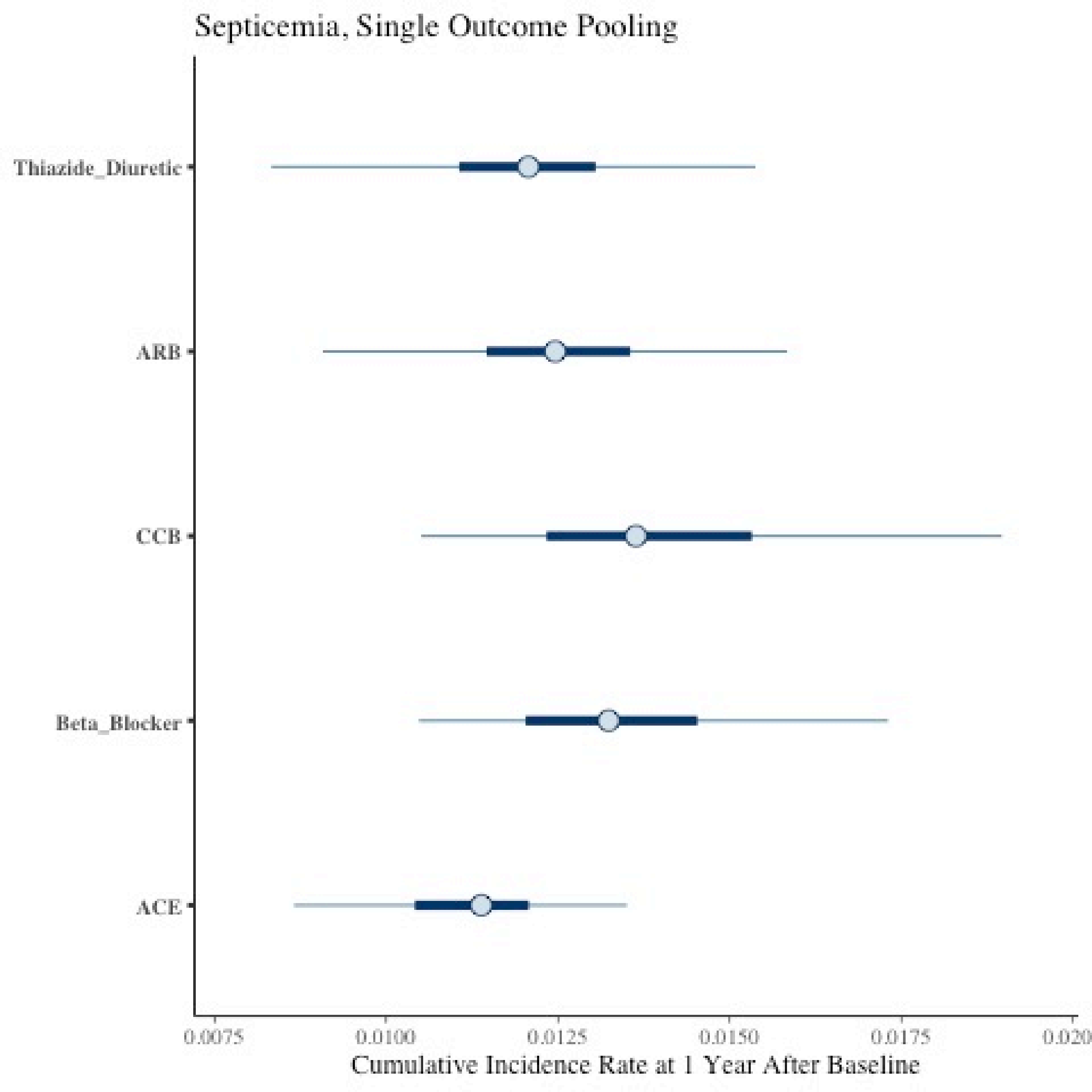
Septicemia, Single Outcome Pooling
Thiazide_Diuretic
ARB
CCB
Beta_Blocker
ACE
0.0075
0.0100
0.0125
0.0150
0.0175
0.020
Cumulative Incidence Rate at 1 Year After Baseline

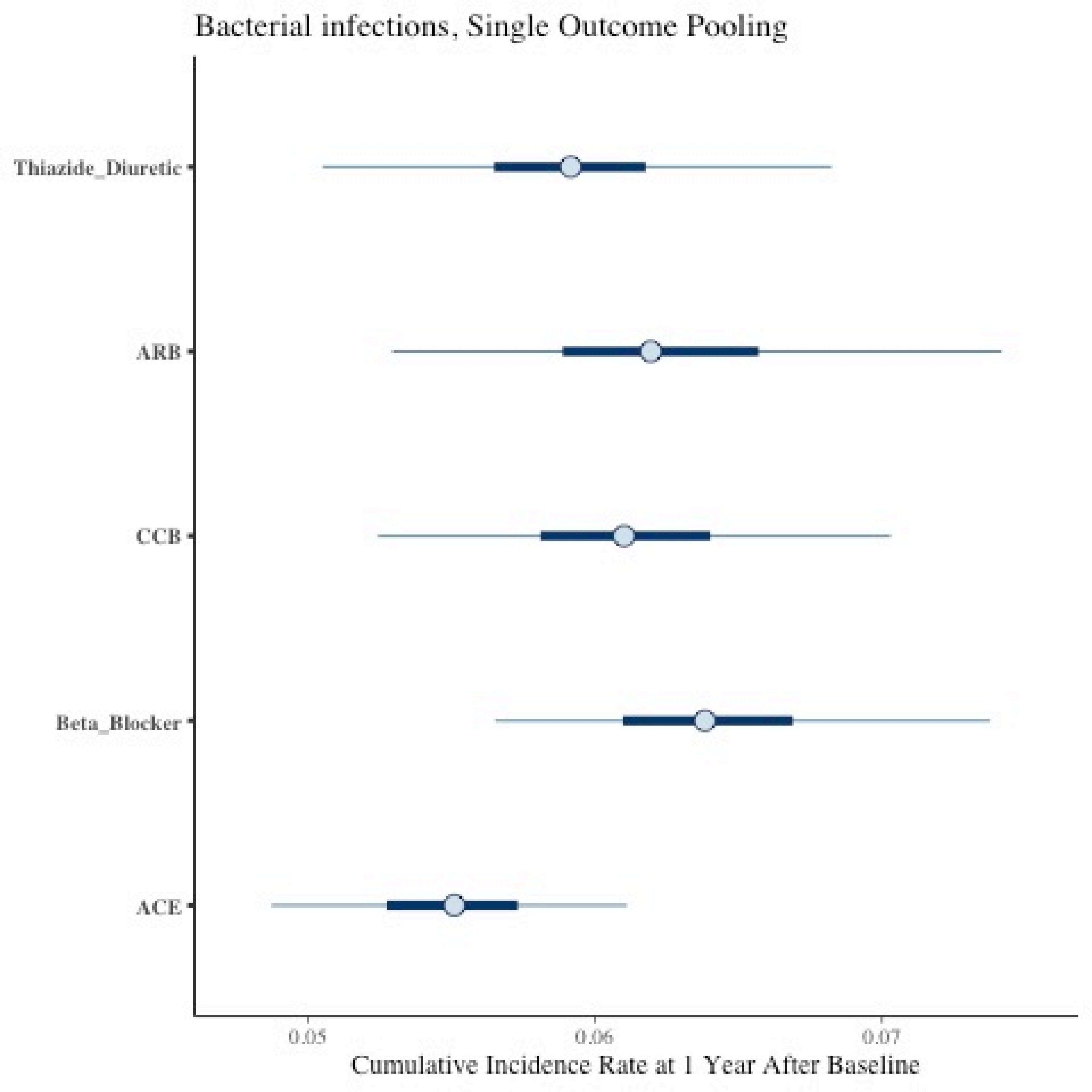

Bacterial infections, Single Outcome Pooling
Thiazide_Diuretic
ARB
CCB
Beta_Blocker
ACE
0.05
0.06
0.07
Cumulative Incidence Rate at 1 Year After Baseline

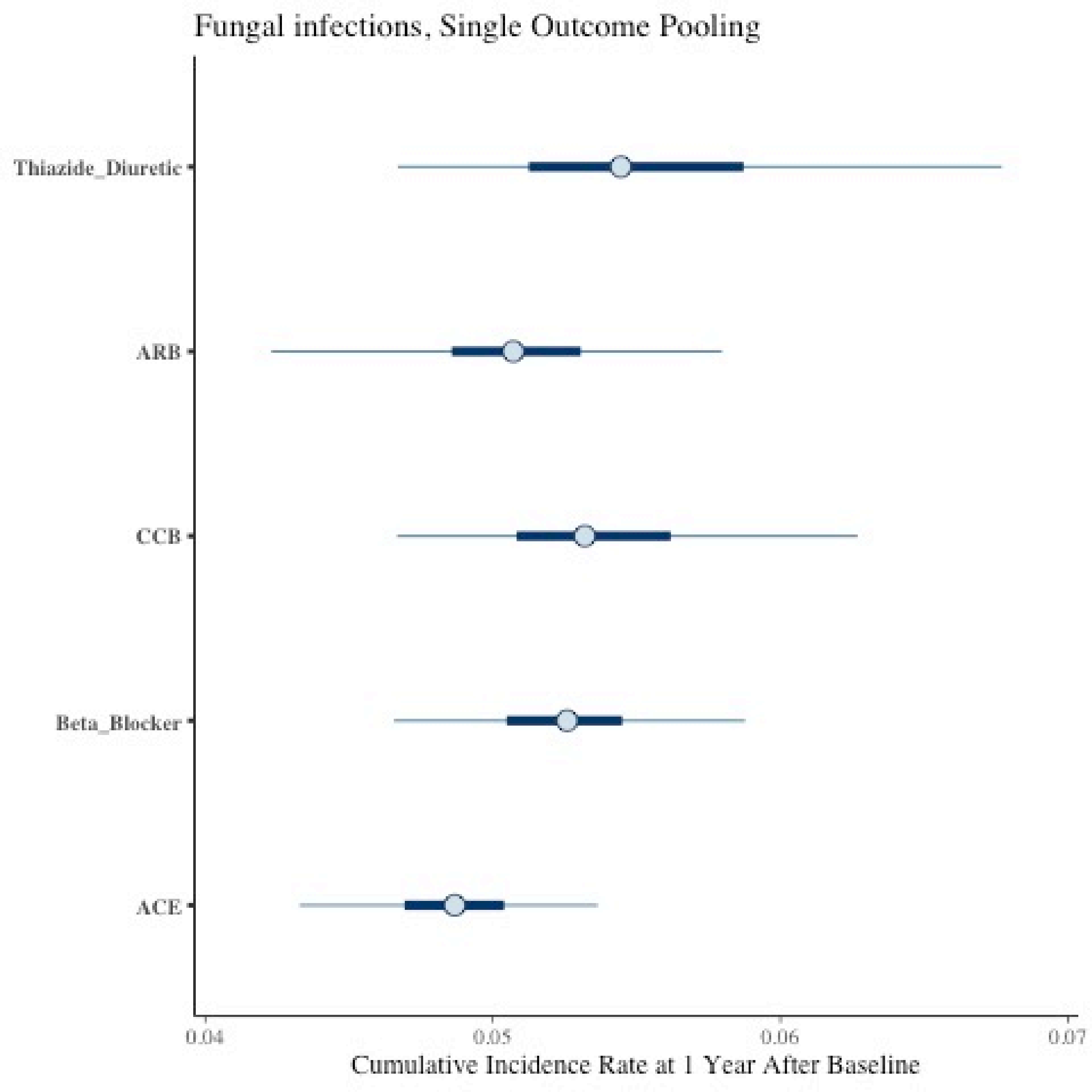

Fungal infections, Single Outcome Pooling
Thiazide_Diuretic
ARB
CCB
Beta_Blocker
ACE
0.04
0.05
0.06
0.07
Cumulative Incidence Rate at 1 Year After Baseline

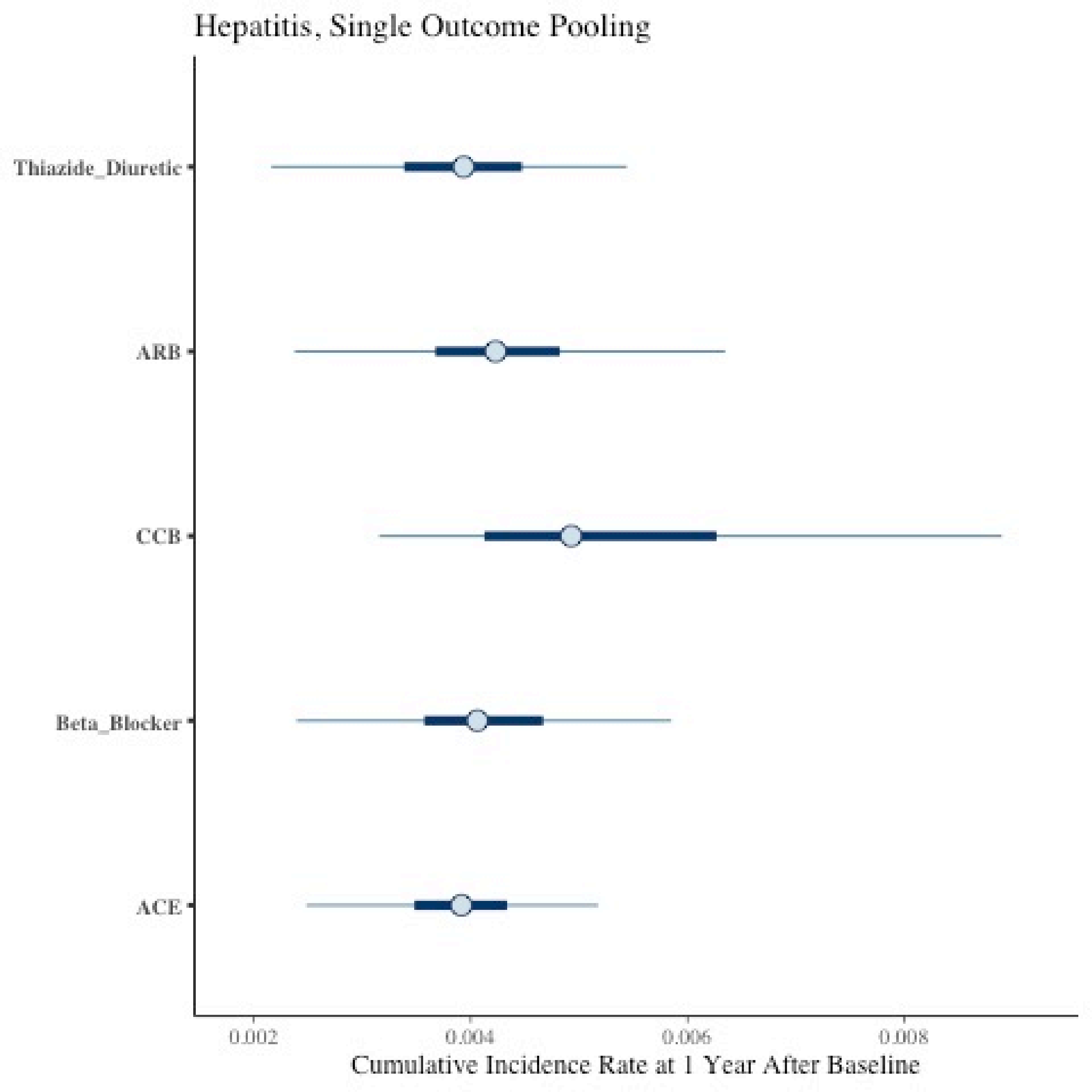

Hepatitis, Single Outcome Pooling
Thiazide_Diuretic
ARB
CCB
Beta_Blocker
ACE
0.002
0.004
0.006
0.008
Cumulative Incidence Rate at 1 Year After Baseline

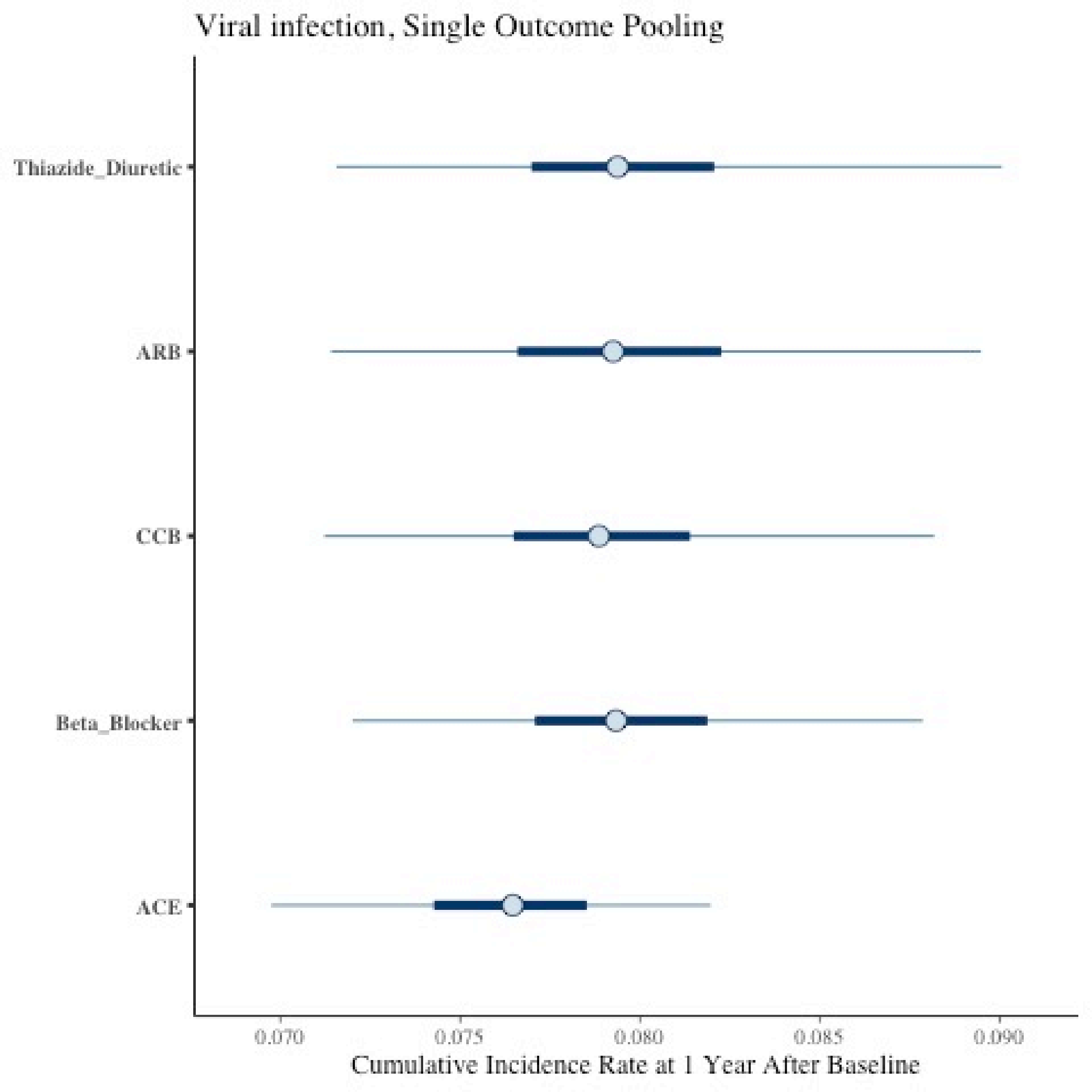

Viral infection, Single Outcome Pooling
Thiazide_Diuretic
ARB
CCB
Beta_Blocker
ACE
0.070
0.075
0.080
0.085
0.090
Cumulative Incidence Rate at 1 Year After Baseline

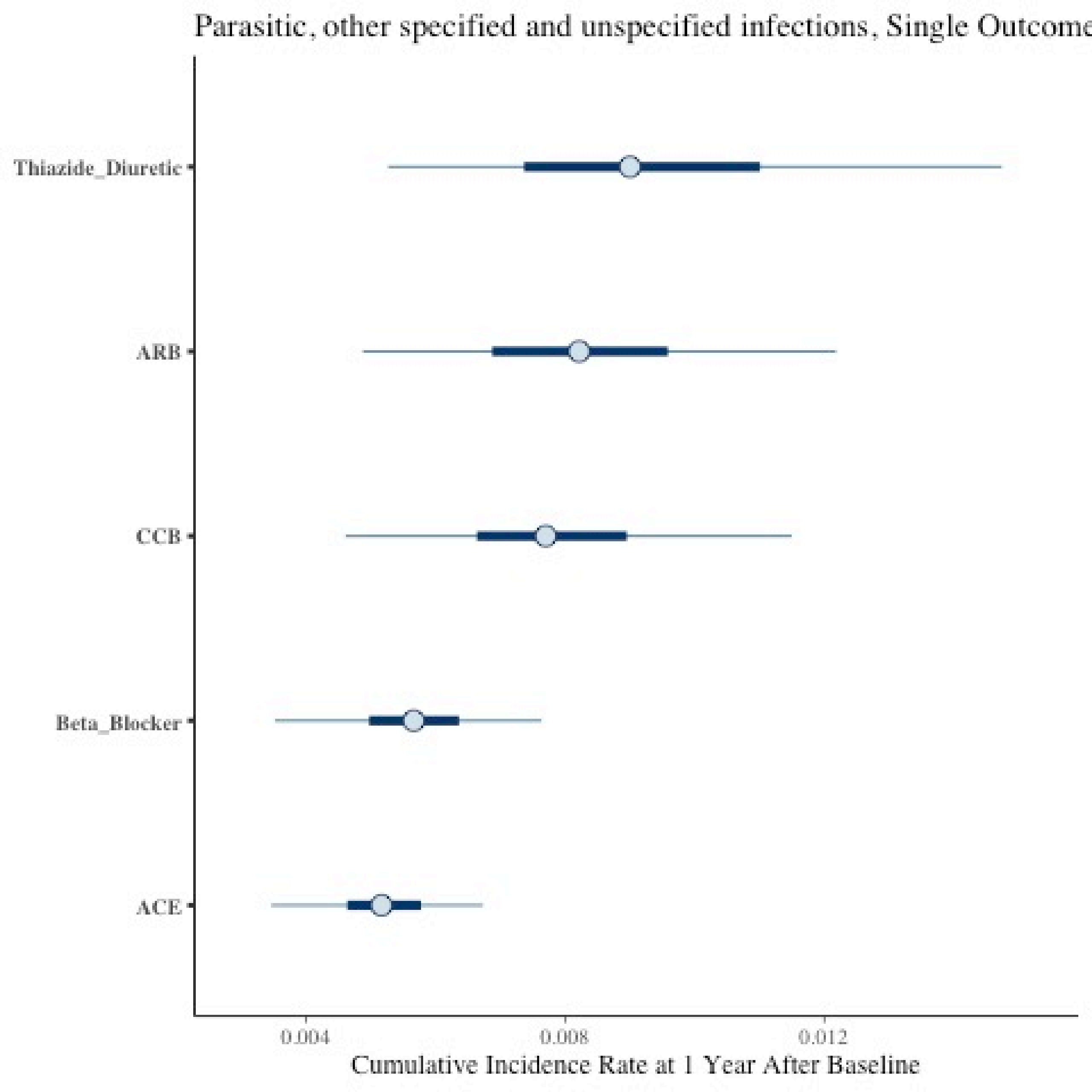

Parasitic, other specified and unspecified infections, Single Outcome
Thiazide_Diuretic
ARB
CCB
Beta_Blocker
ACE
0.004
0.008
0.012
Cumulative Incidence Rate at 1 Year After Baseline

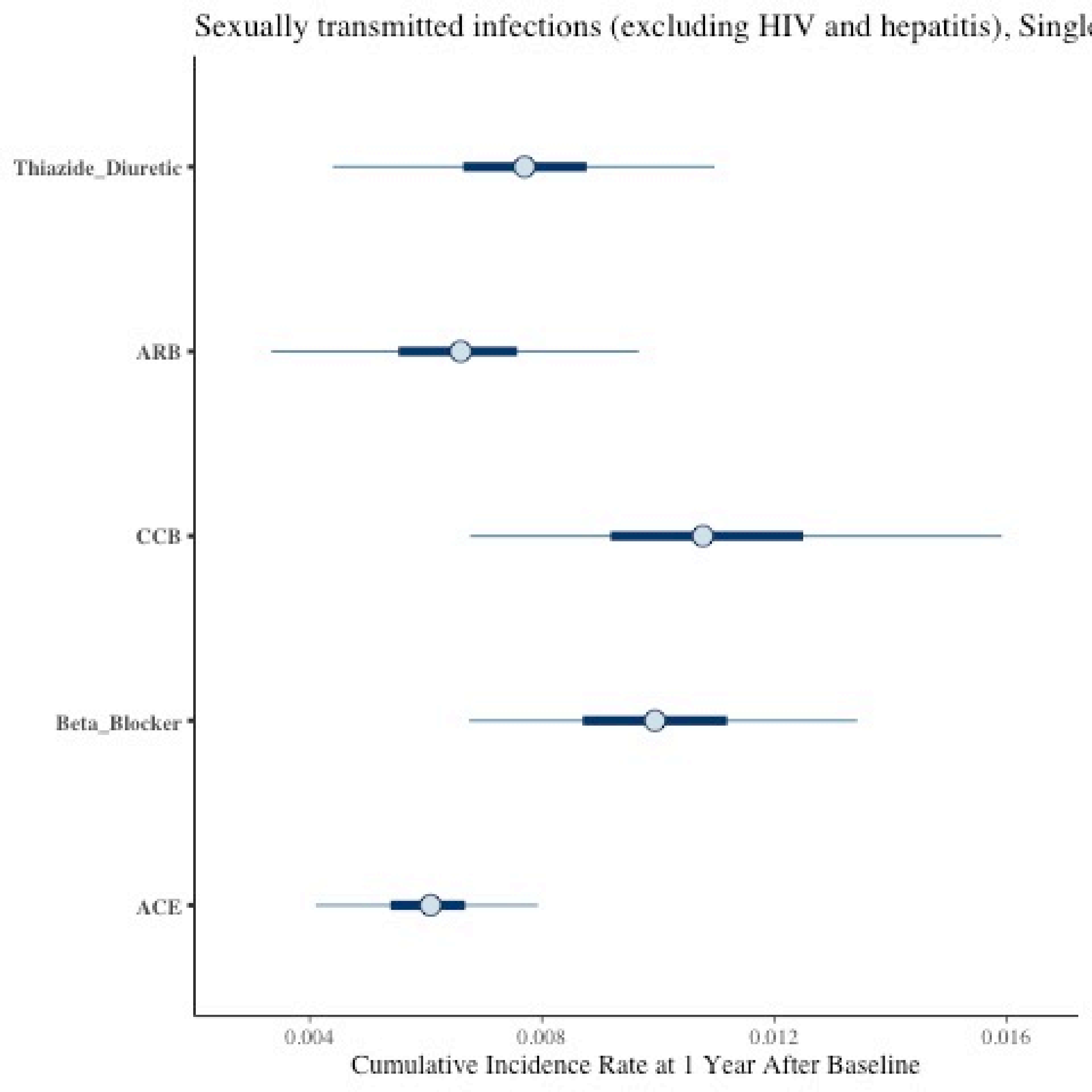

Sexually transmitted infections (excluding HIV and hepatitis), Singl
Thiazide_Diuretic
ARB
CCB
Beta_Blocker
ACE
0.004
0.008
0.012
0.016
Cumulative Incidence Rate at 1 Year After Baseline

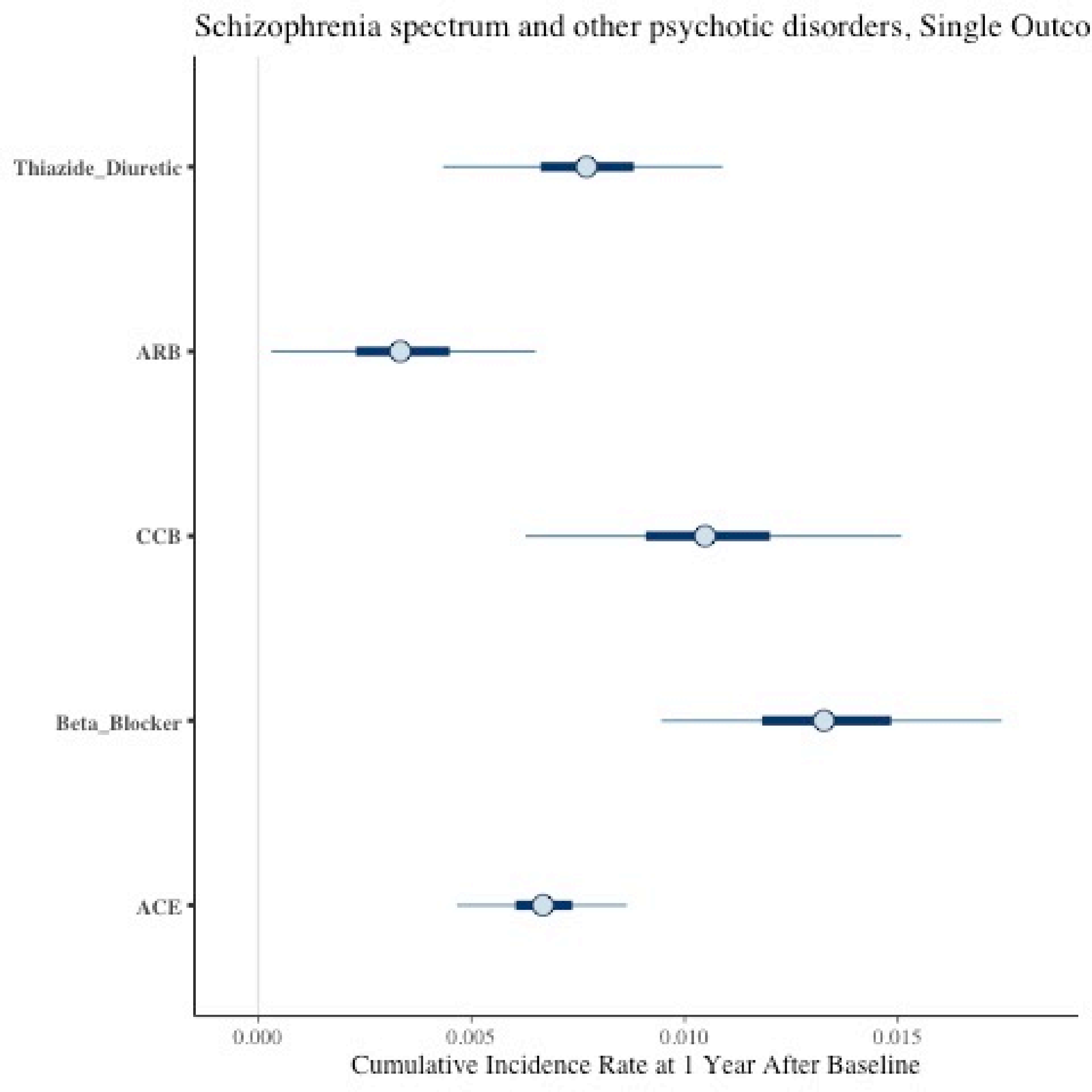

Schizophrenia spectrum and other psychotic disorders, Single Outco
Thiazide_Diuretic
ARB
CCB
Beta_Blocker
ACE
0.000
0.005
0.010
0.015
Cumulative Incidence Rate at 1 Year After Baseline

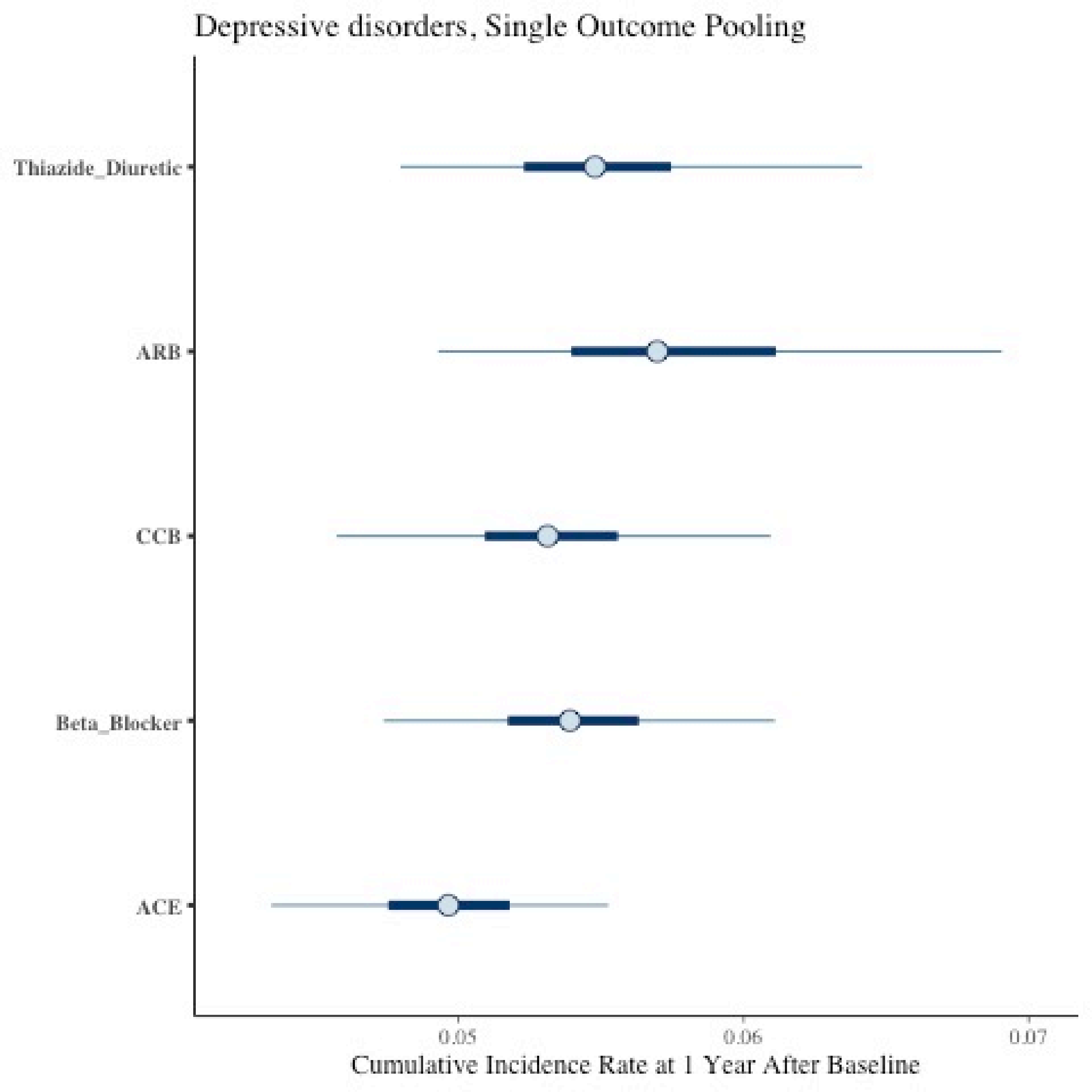
Depressive disorders, Single Outcome Pooling
Thiazide_Diuretic
ARB
CCB
Beta_Blocker
ACE
0.05
0.06
0.07
Cumulative Incidence Rate at 1 Year After Baseline

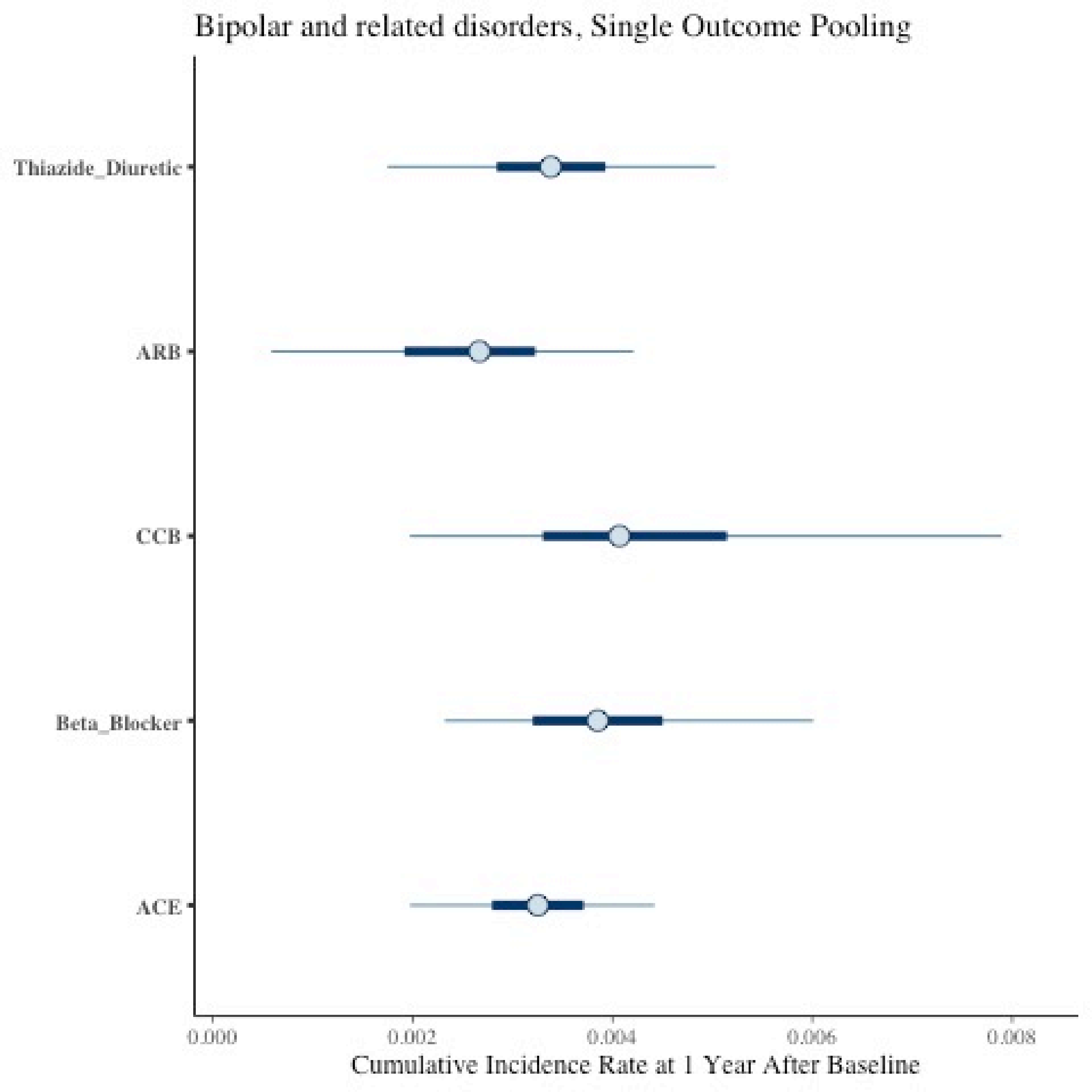

Bipolar and related disorders, Single Outcome Pooling
Thiazide_Diuretic
ARB
CCB
Beta_Blocker
ACE
0.000
0.002
0.004
0.006
0.008
Cumulative Incidence Rate at 1 Year After Baseline

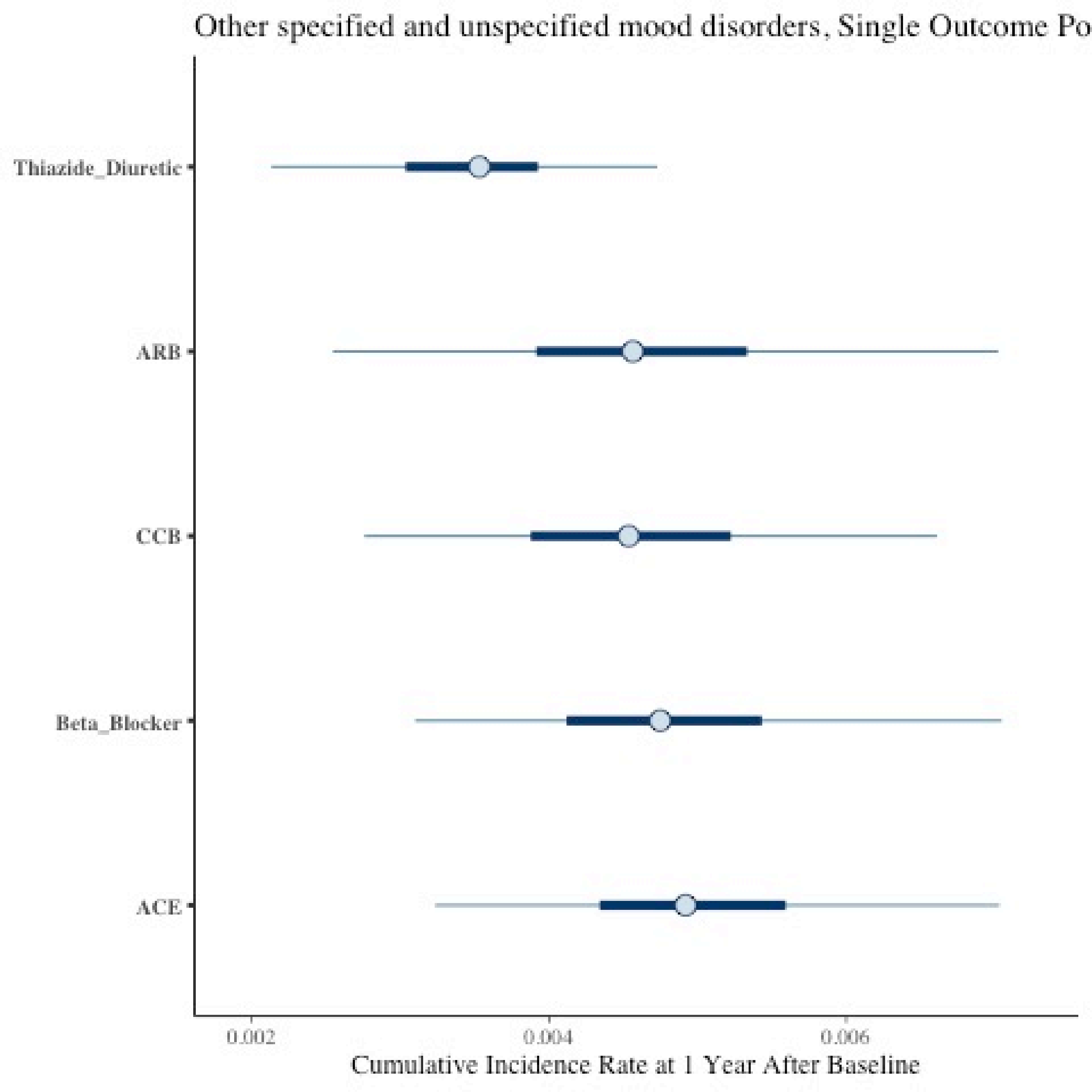
Other specified and unspecified mood disorders, Single Outcome Po
Thiazide_Diuretic
ARB
CCB
Beta_Blocker
ACE
0.002
0.004
0.006
Cumulative Incidence Rate at 1 Year After Baseline

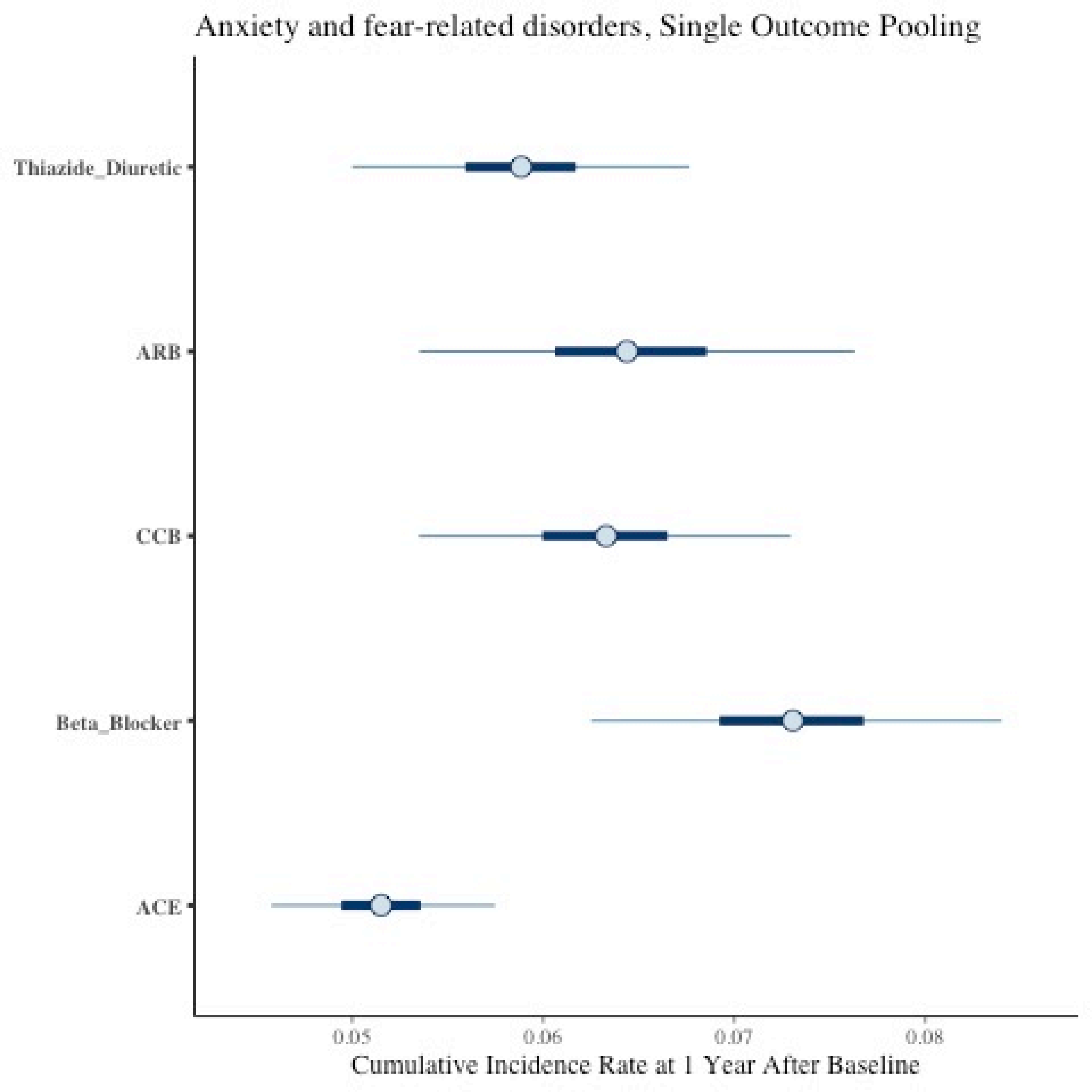
Anxiety and fear-related disorders, Single Outcome Pooling
Thiazide_Diuretic
ARB
CCB
Beta_Blocker
ACE
0.05
0.06
0.07
0.08
Cumulative Incidence Rate at 1 Year After Baseline

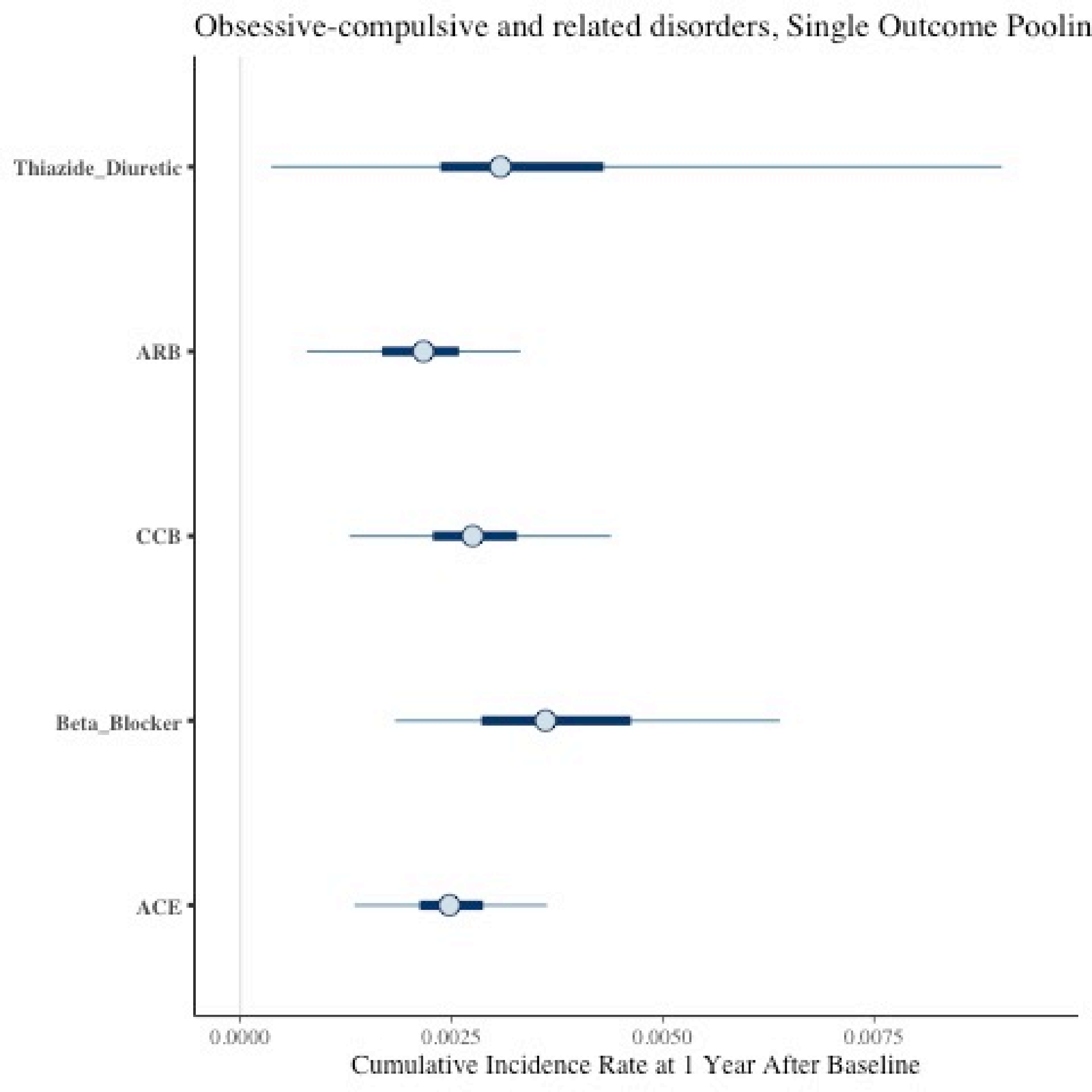
Obsessive-compulsive and related disorders, Single Outcome Poolin
Thiazide_Diuretic
ARB
CCB
Beta_Blocker
ACE
0.0000
0.0025
0.0050
0.0075
Cumulative Incidence Rate at 1 Year After Baseline

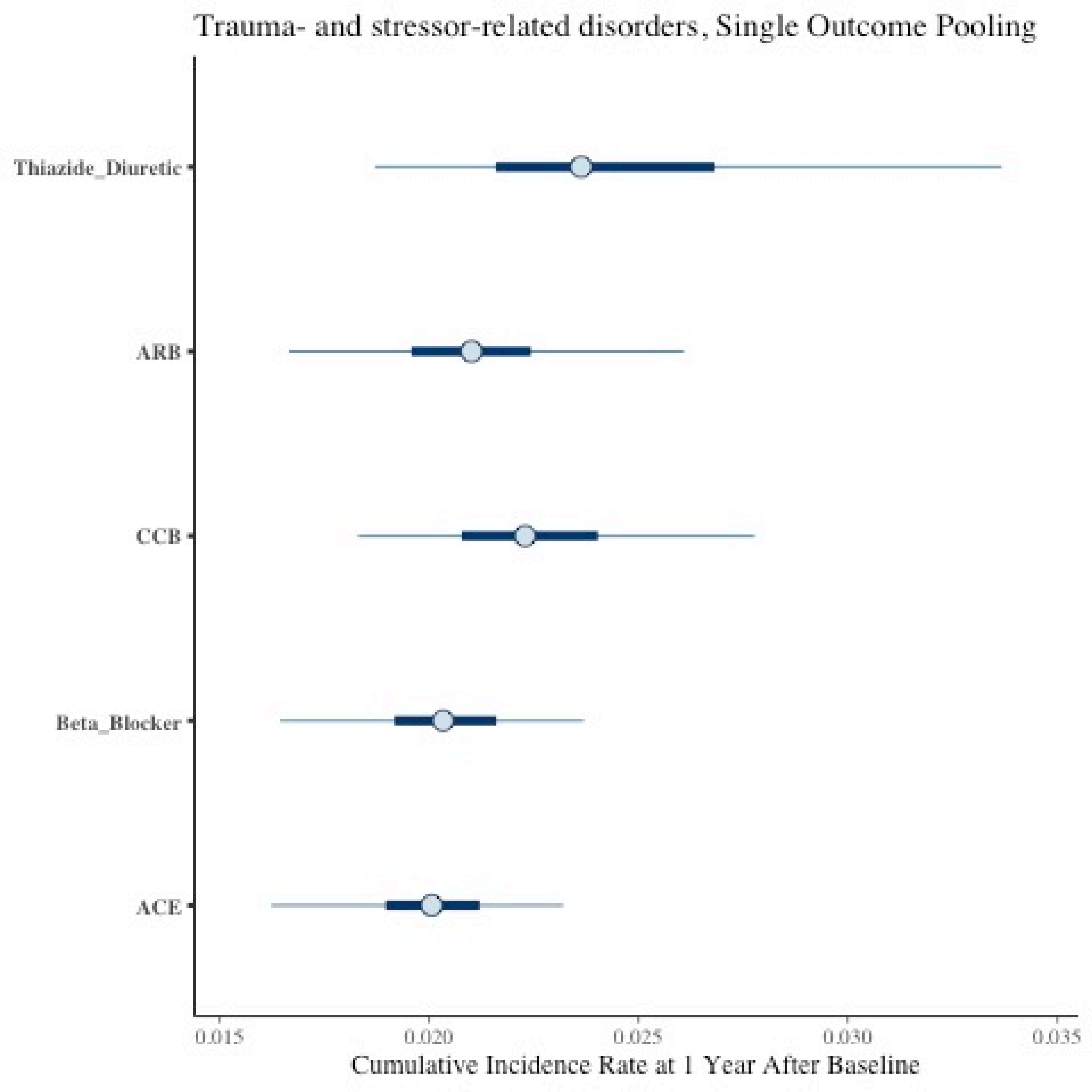

Trauma- and stressor-related disorders, Single Outcome Pooling
Thiazide_Diuretic
ARB
CCB
Beta_Blocker
ACE
0.015
0.020
0.025
0.030
0.035
Cumulative Incidence Rate at 1 Year After Baseline

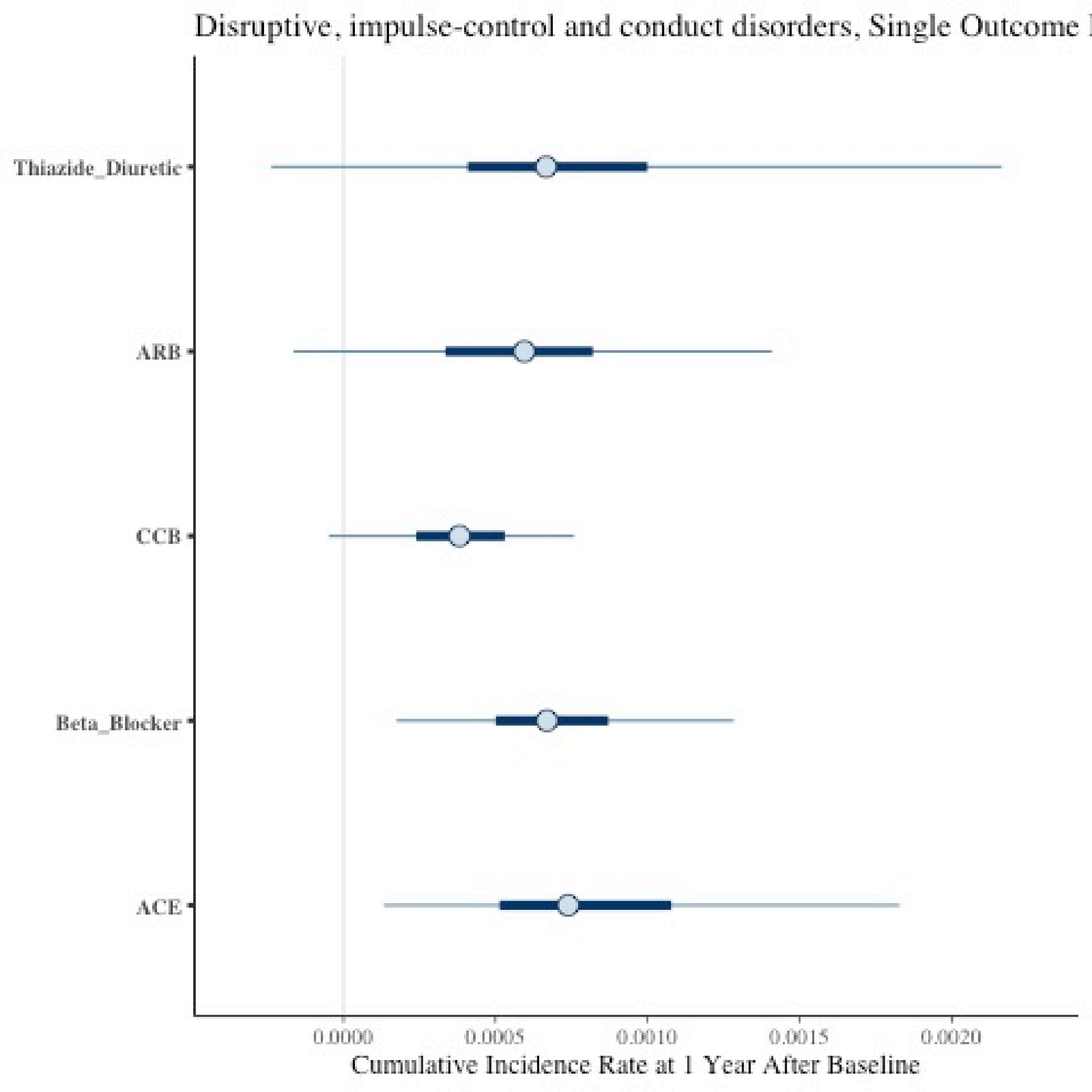
Disruptive, impulse-control and conduct disorders, Single Outcome
Thiazide_Diuretic
ARB
CCB
Beta_Blocker
ACE
0.0000
0.0005
0.0010
0.0015
0.0020
Cumulative Incidence Rate at 1 Year After Baseline

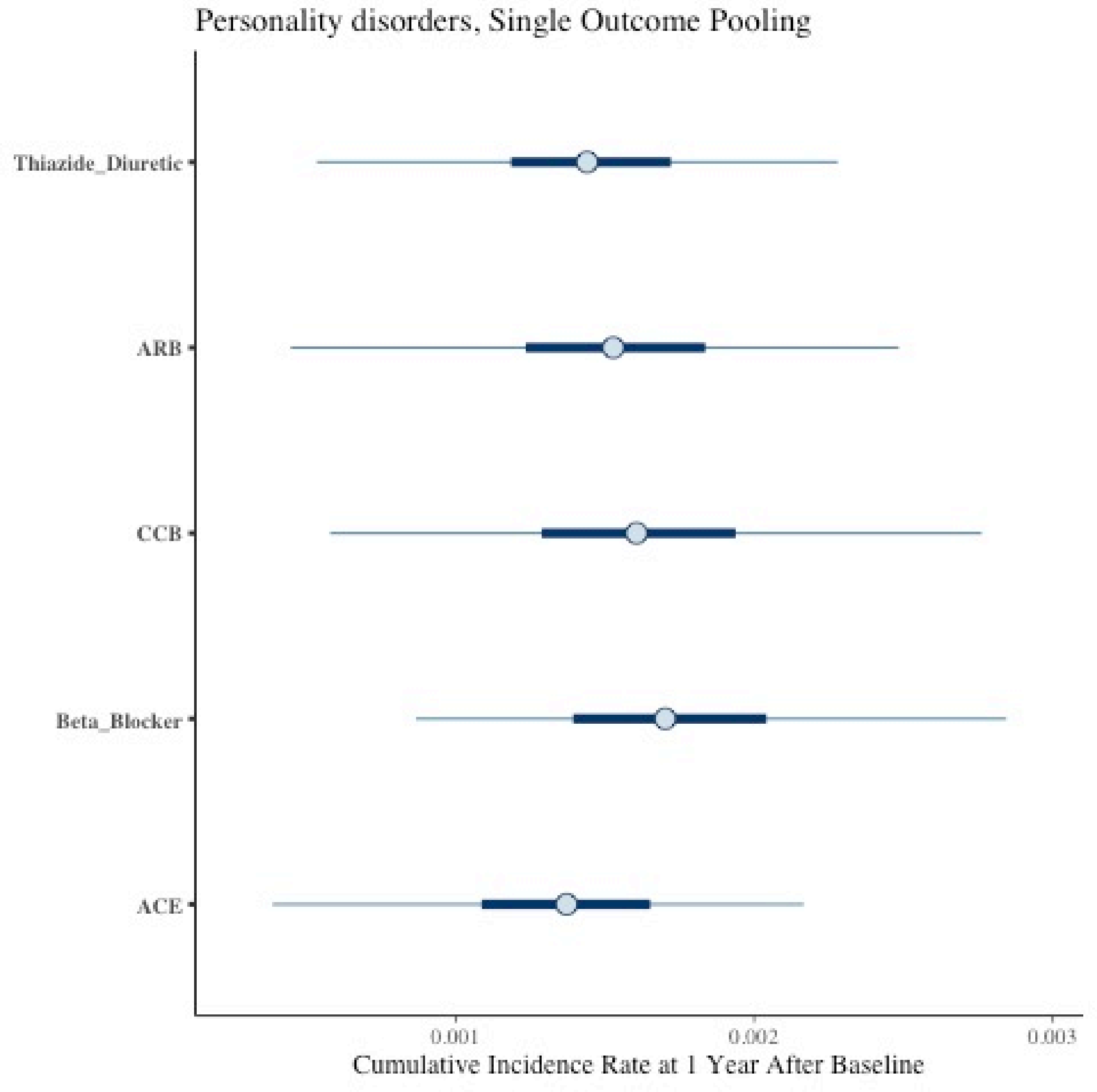
Personality disorders, Single Outcome Pooling
Thiazide_Diuretic
ARB
CCB
Beta_Blocker
ACE
0.001
0.002
0.003
Cumulative Incidence Rate at 1 Year After Baseline

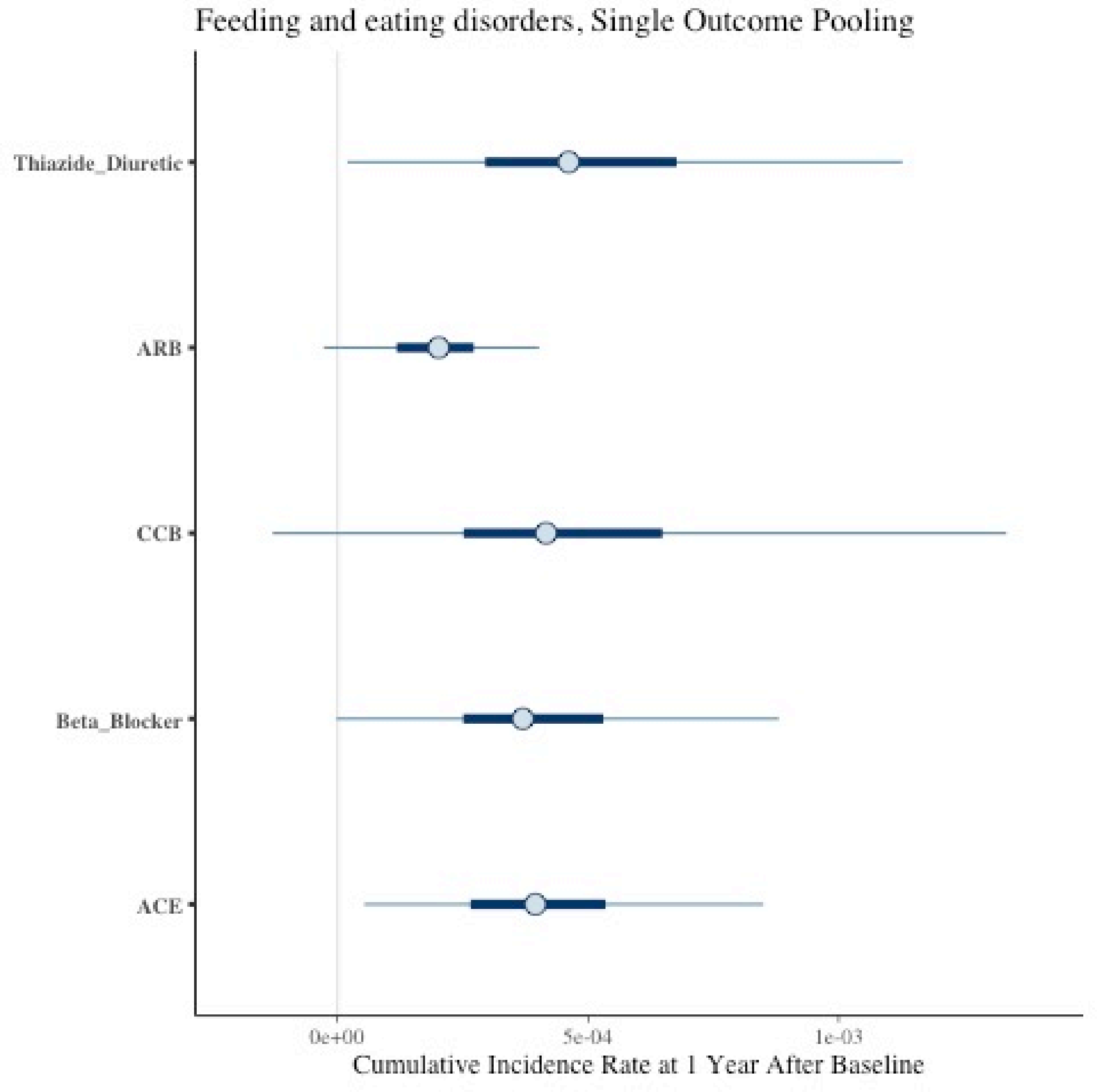
Feeding and eating disorders, Single Outcome Pooling
Thiazide_Diuretic
ARB
CCB
Beta_Blocker
ACE
0e+00
5e-04
1e-03
Cumulative Incidence Rate at 1 Year After Baseline

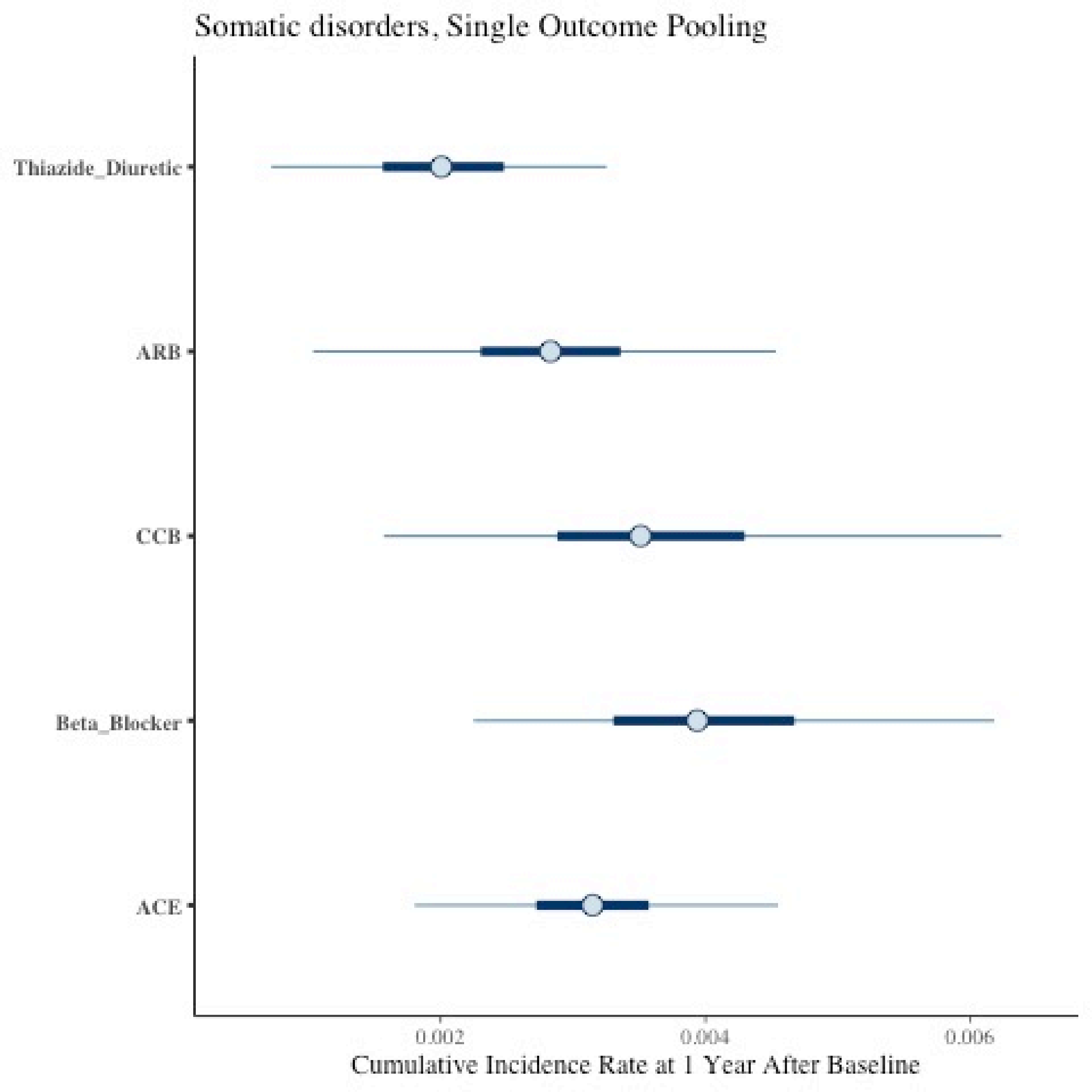

Somatic disorders, Single Outcome Pooling
Thiazide_Diuretic
ARB
CCB
Beta_Blocker
ACE
0.002
0.004
0.006
Cumulative Incidence Rate at 1 Year After Baseline

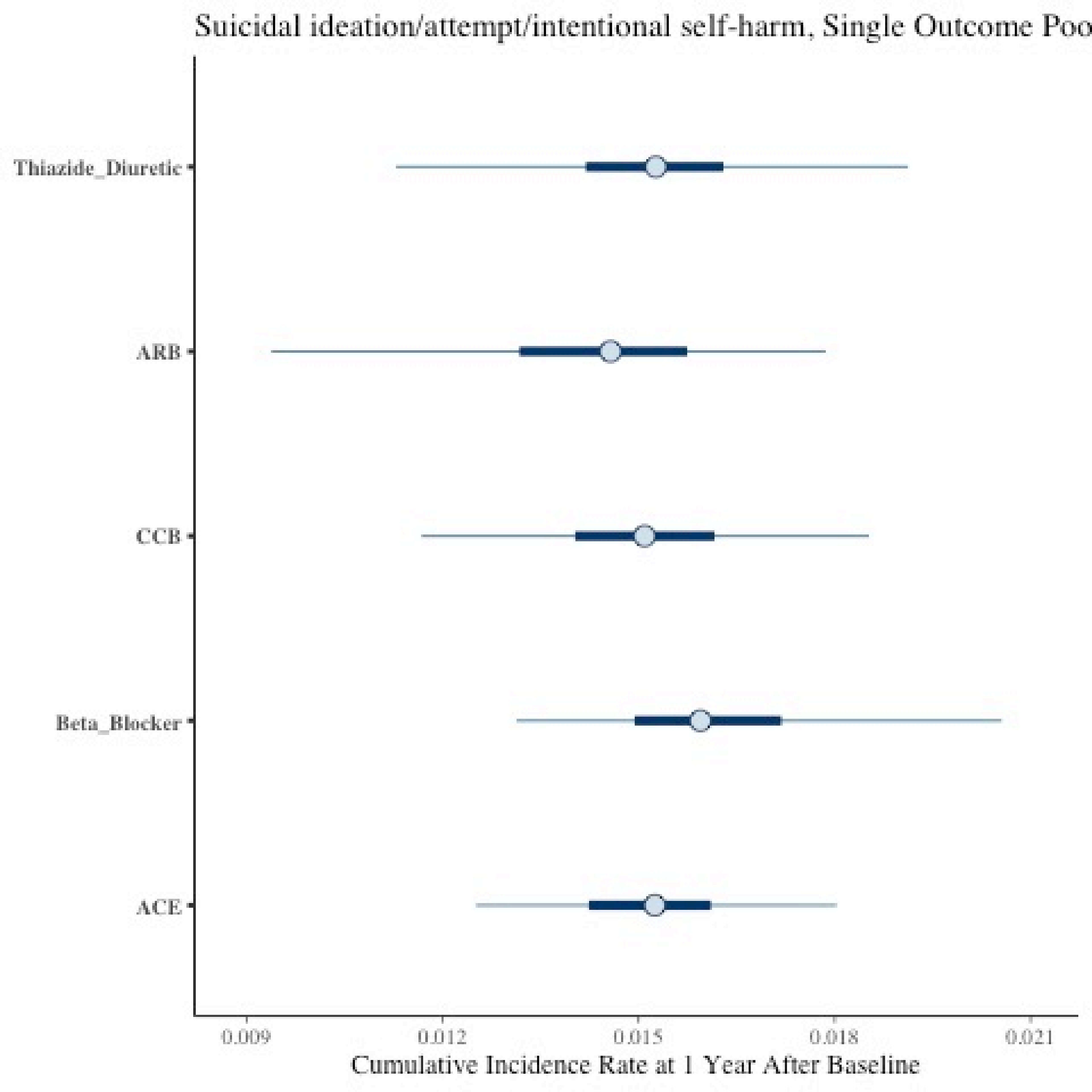

Suicidal ideation/attempt/intentional self-harm, Single Outcome Poo
Thiazide_Diuretic
ARB
CCB
Beta_Blocker
ACE
0.009
0.012
0.015
0.018
0.021
Cumulative Incidence Rate at 1 Year After Baseline

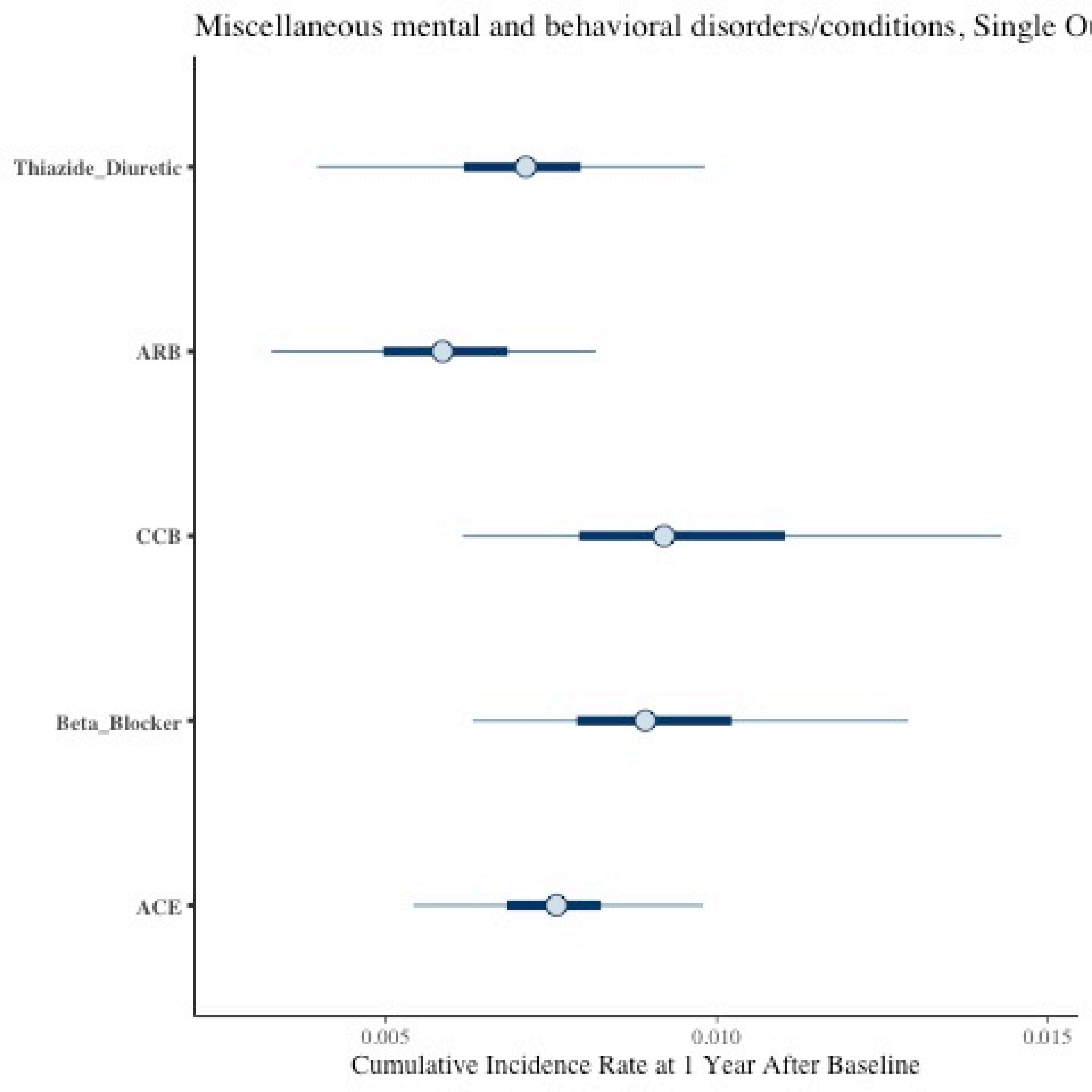

Miscellaneous mental and behavioral disorders/conditions, Single O
Thiazide_Diuretic
ARB
CCB
Beta_Blocker
ACE
0.005
0.010
0.015
Cumulative Incidence Rate at 1 Year After Baseline

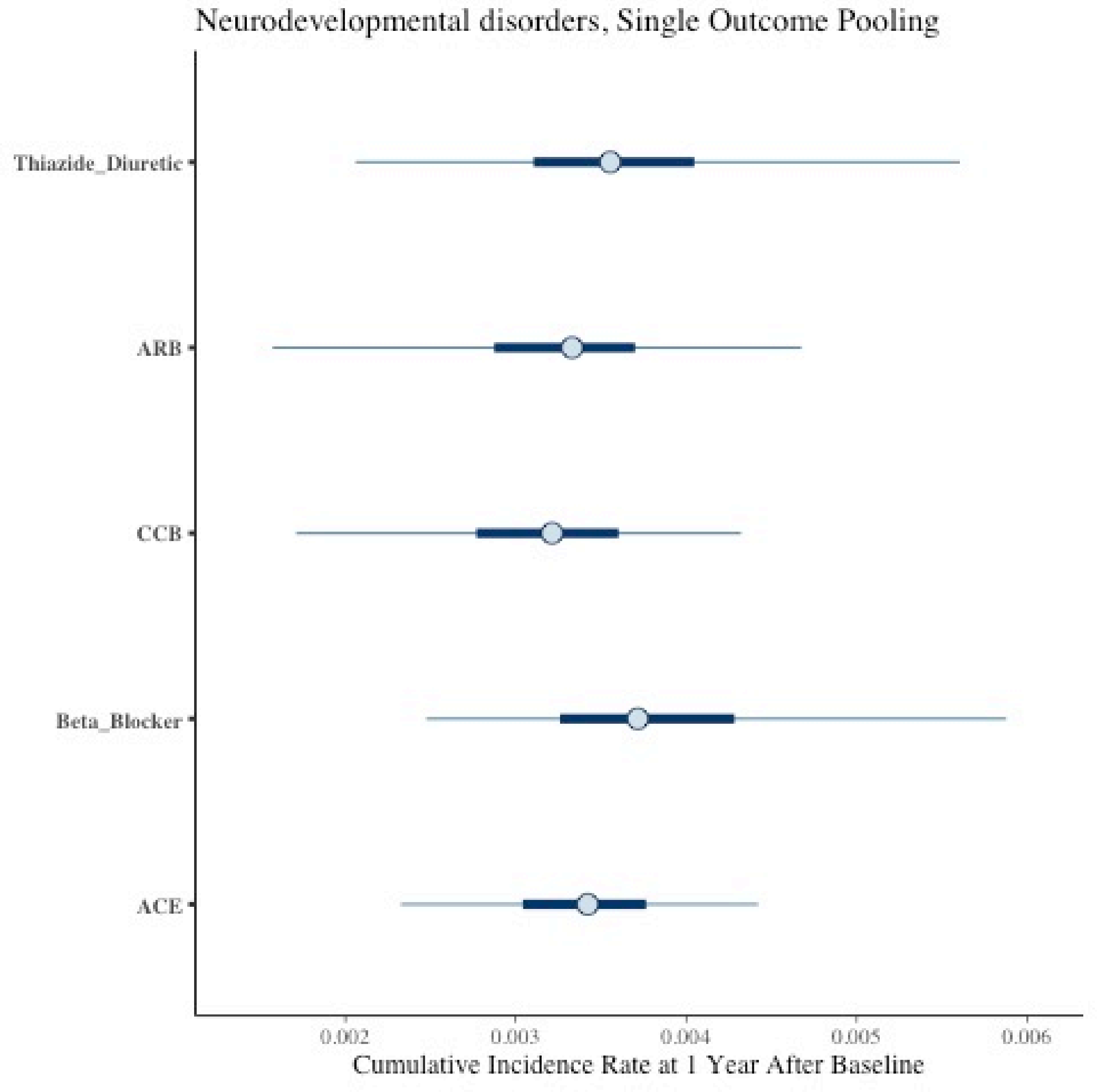
Neurodevelopmental disorders, Single Outcome Pooling
Thiazide_Diuretic
ARB
CCB
Beta_Blocker
ACE
0.002
0.003
0.004
0.005
0.006
Cumulative Incidence Rate at 1 Year After Baseline

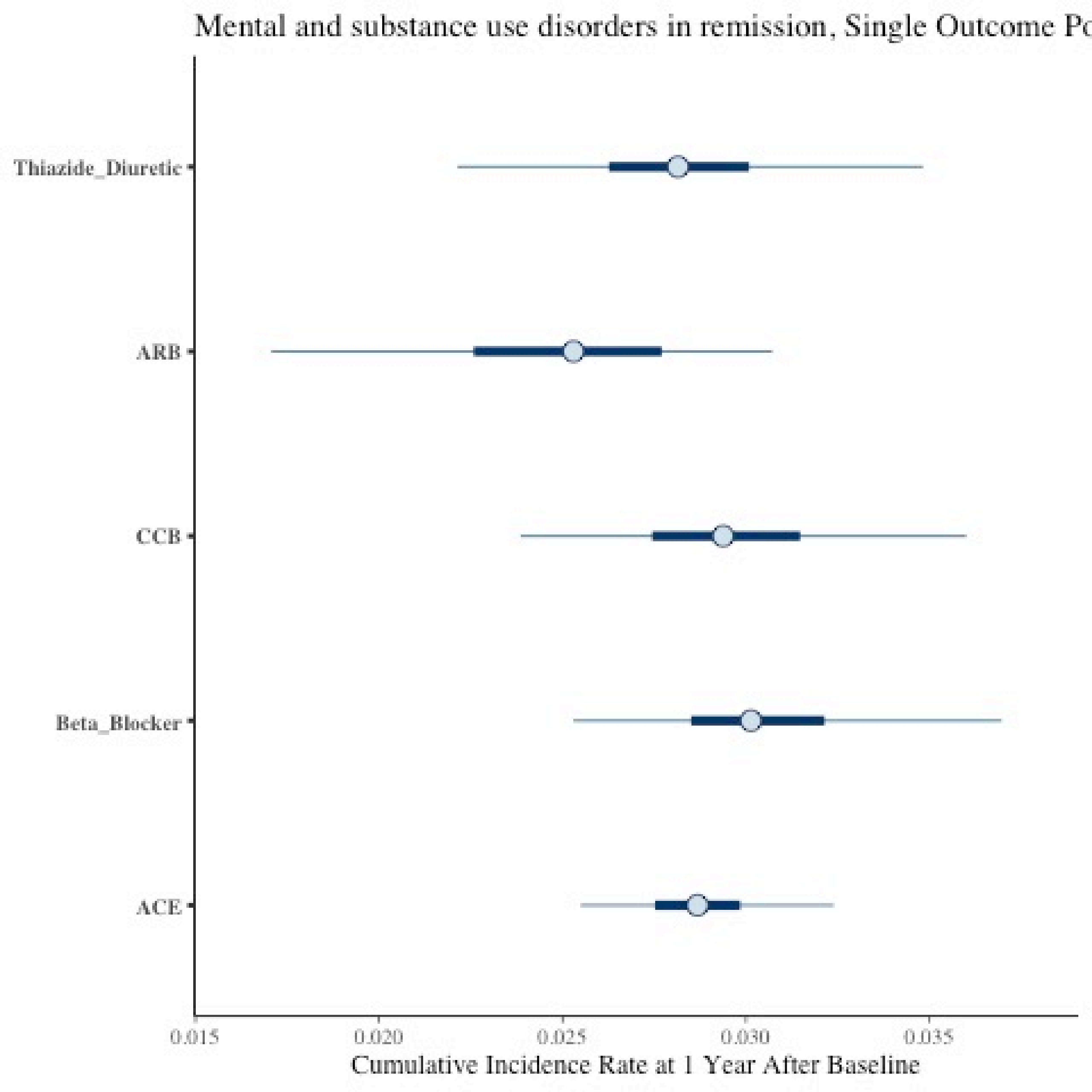
Mental and substance use disorders in remission, Single Outcome Po
Thiazide_Diuretic
ARB
CCB
Beta_Blocker
ACE
0.015
0.020
0.025
0.030
0.035
Cumulative Incidence Rate at 1 Year After Baseline

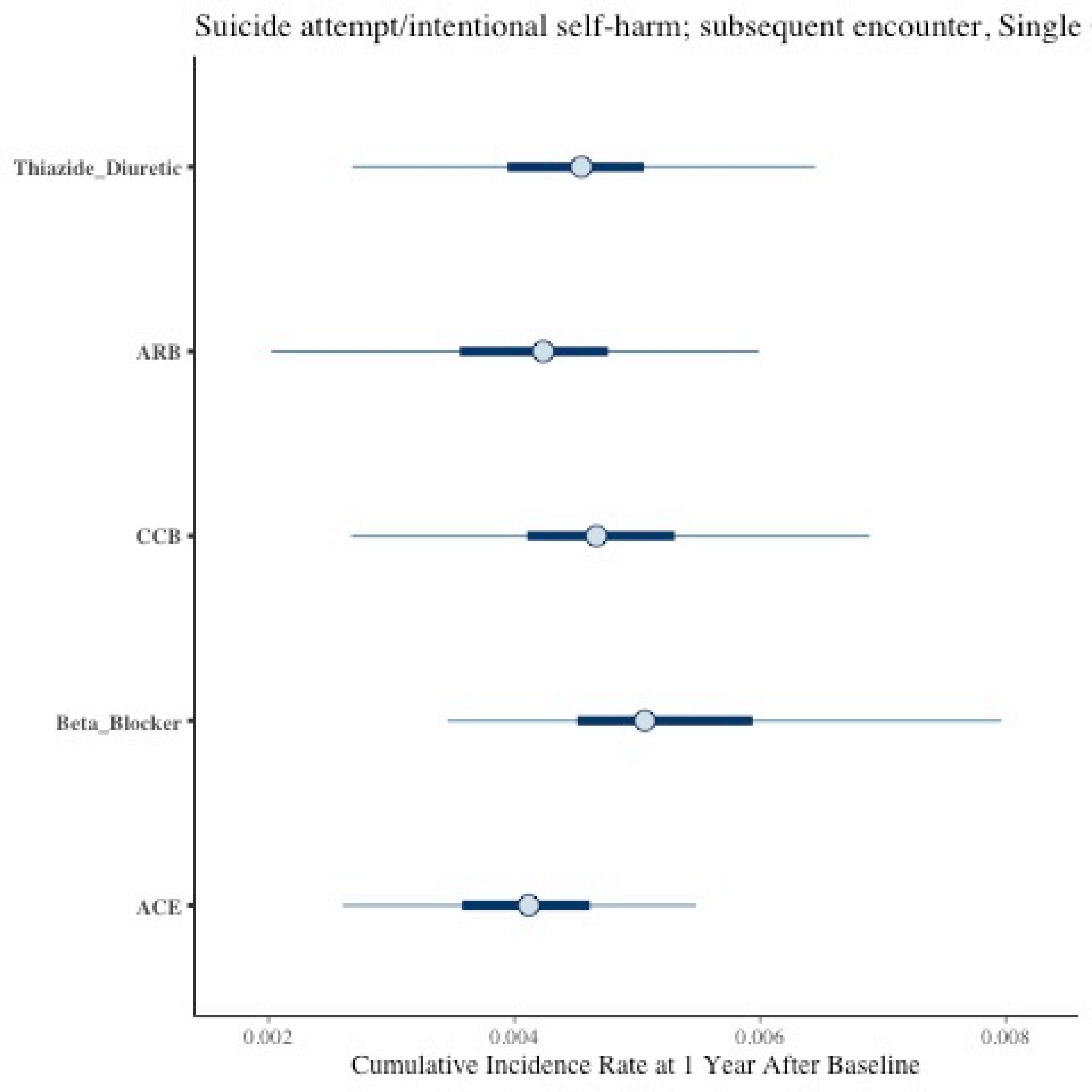
Suicide attempt/intentional self-harm; subsequent encounter, Single
Thiazide_Diuretic
ARB
CCB
Beta_Blocker
ACE
0.002
0.004
0.006
0.008
Cumulative Incidence Rate at 1 Year After Baseline

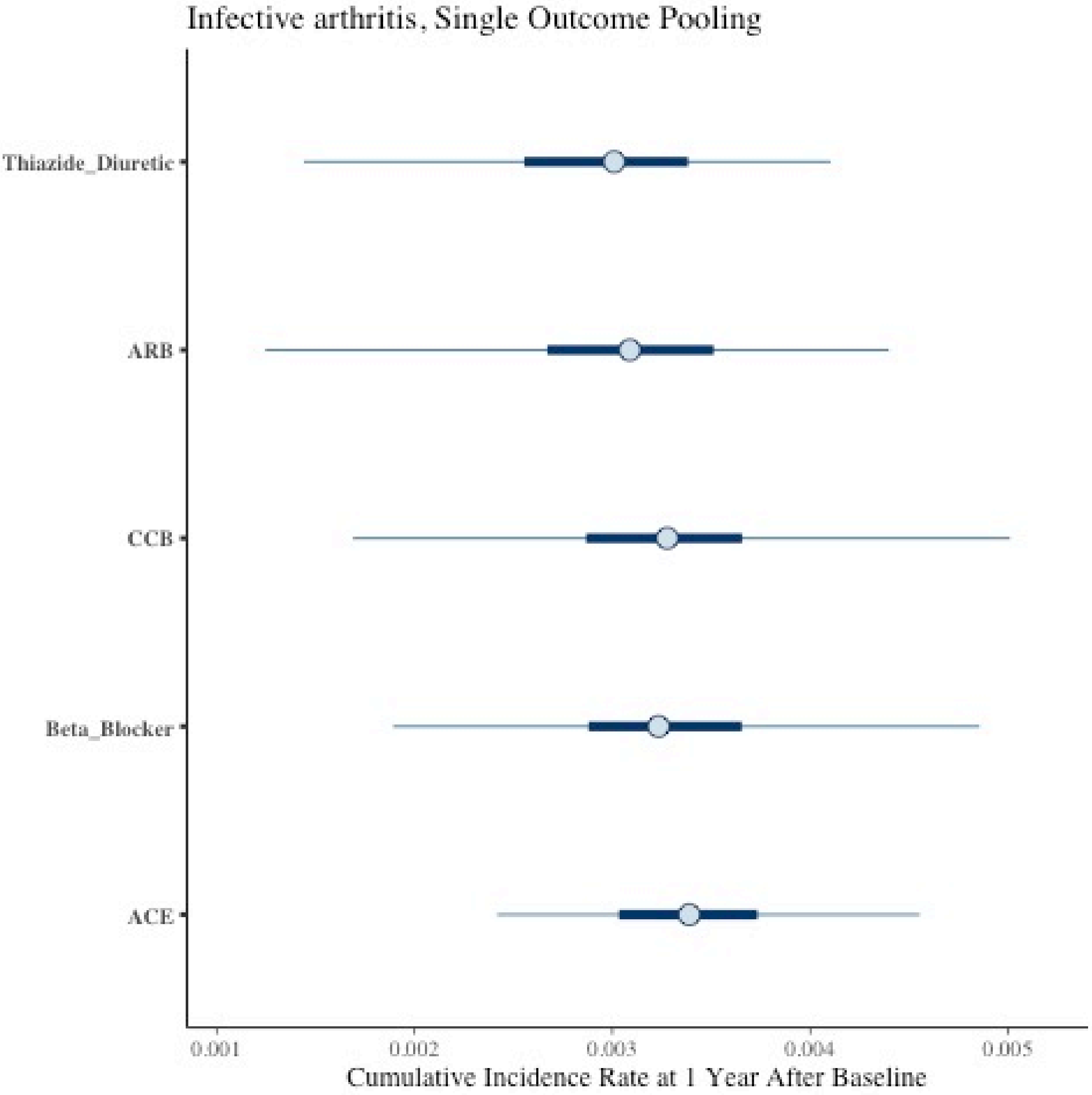

Infective arthritis, Single Outcome Pooling
Thiazide_Diuretic
ARB
CCB
Beta_Blocker
ACE
0.001
0.002
0.003
0.004
0.005
Cumulative Incidence Rate at 1 Year After Baseline

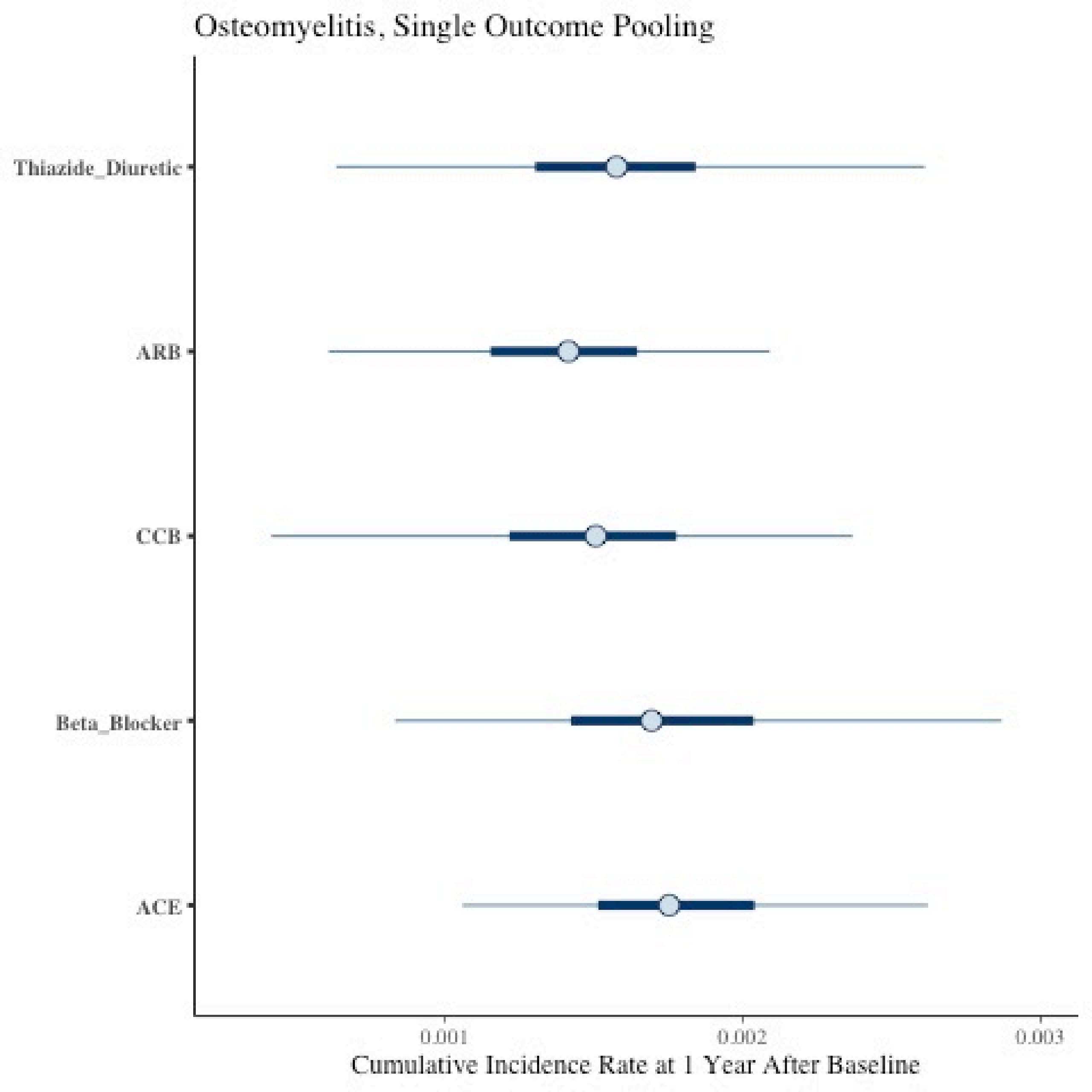
Osteomyelitis, Single Outcome Pooling
Thiazide_Diuretic
ARB
CCB
Beta_Blocker
ACE
0.001
0.002
0.003
Cumulative Incidence Rate at 1 Year After Baseline

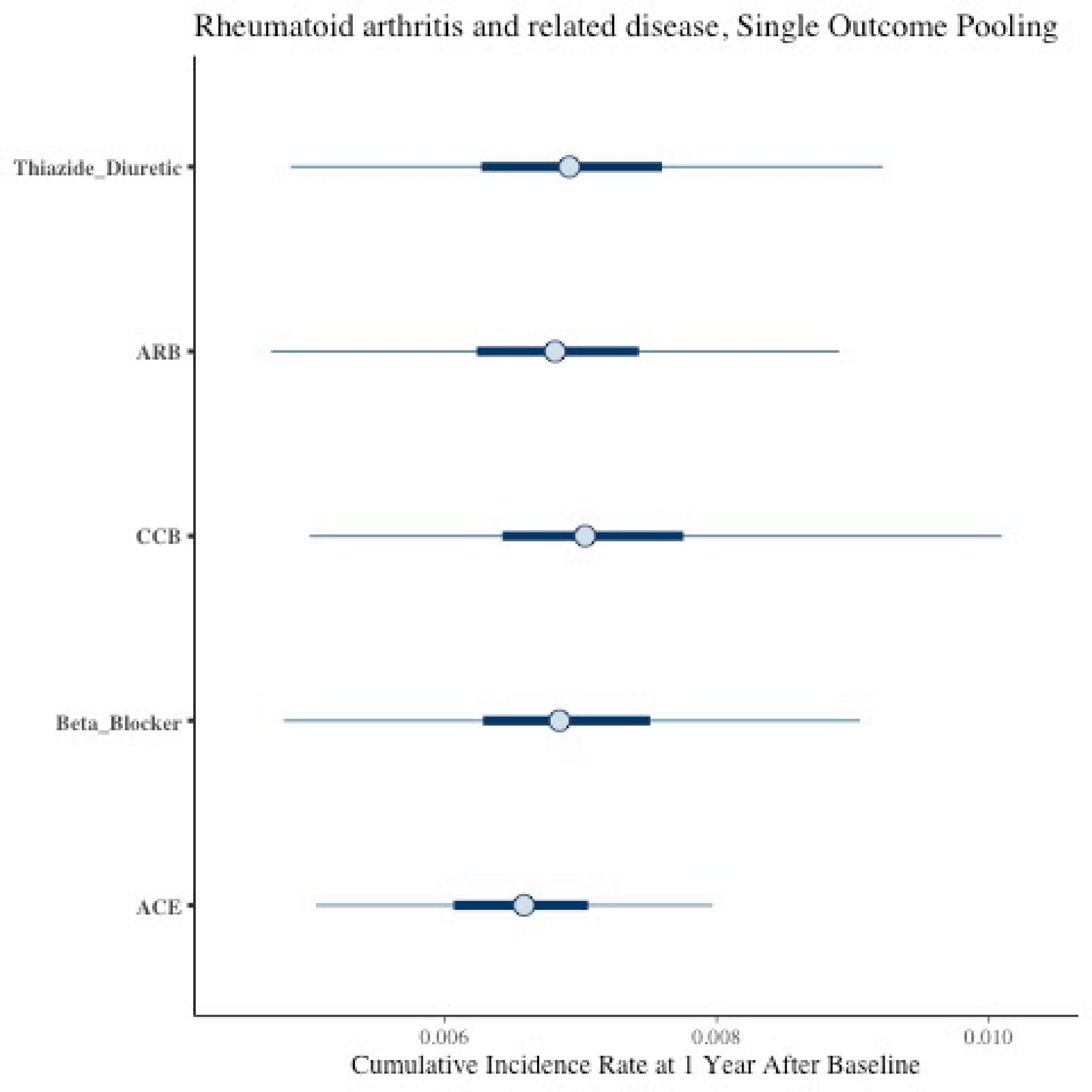
Rheumatoid arthritis and related disease, Single Outcome Pooling
Thiazide_Diuretic
ARB
CCB
Beta_Blocker
ACE
0.006
0.008
0.010
Cumulative Incidence Rate at 1 Year After Baseline

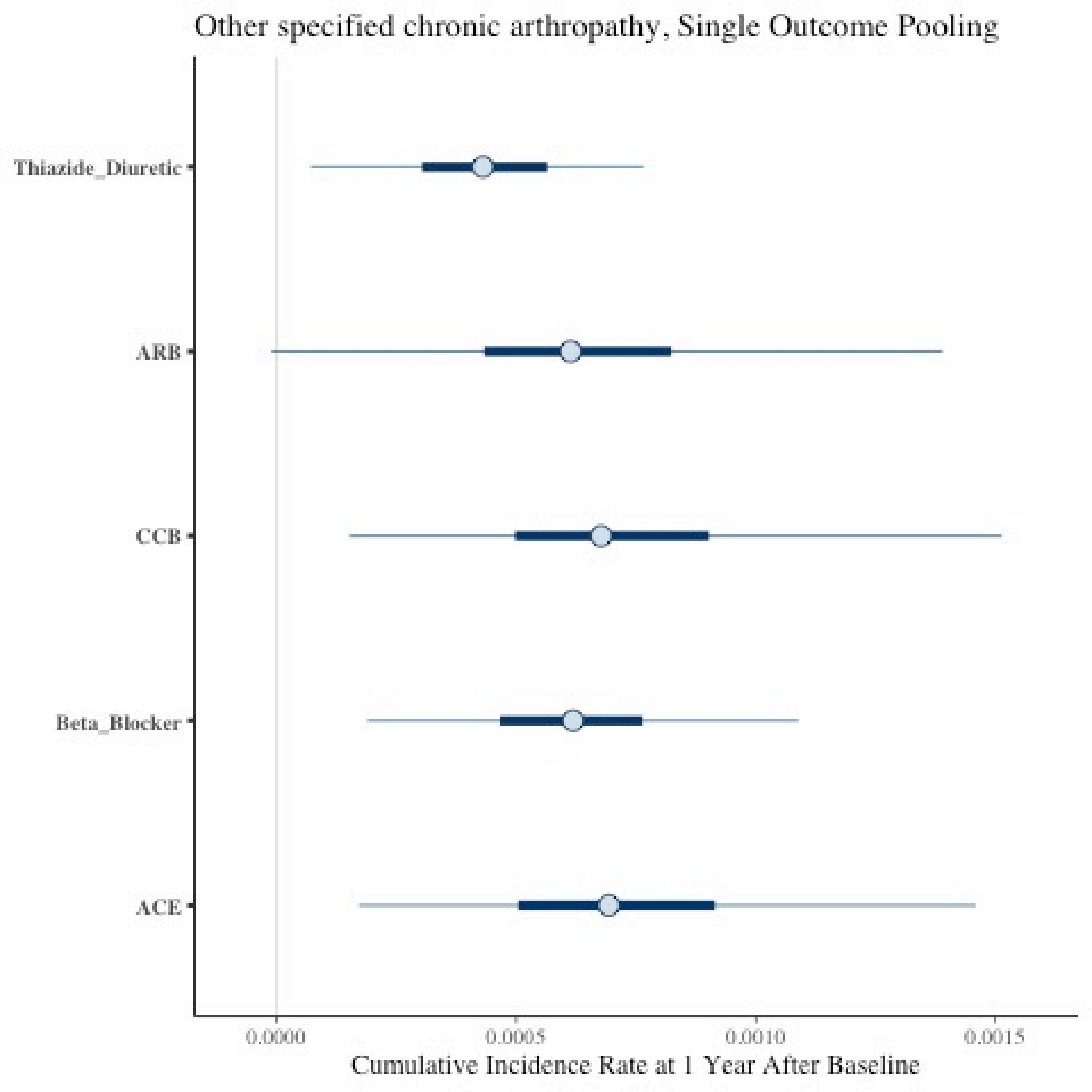

Other specified chronic arthropathy, Single Outcome Pooling
Thiazide_Diuretic
ARB
CCB
Beta_Blocker
ACE
0.0000
0.0005
0.0010
0.0015
Cumulative Incidence Rate at 1 Year After Baseline

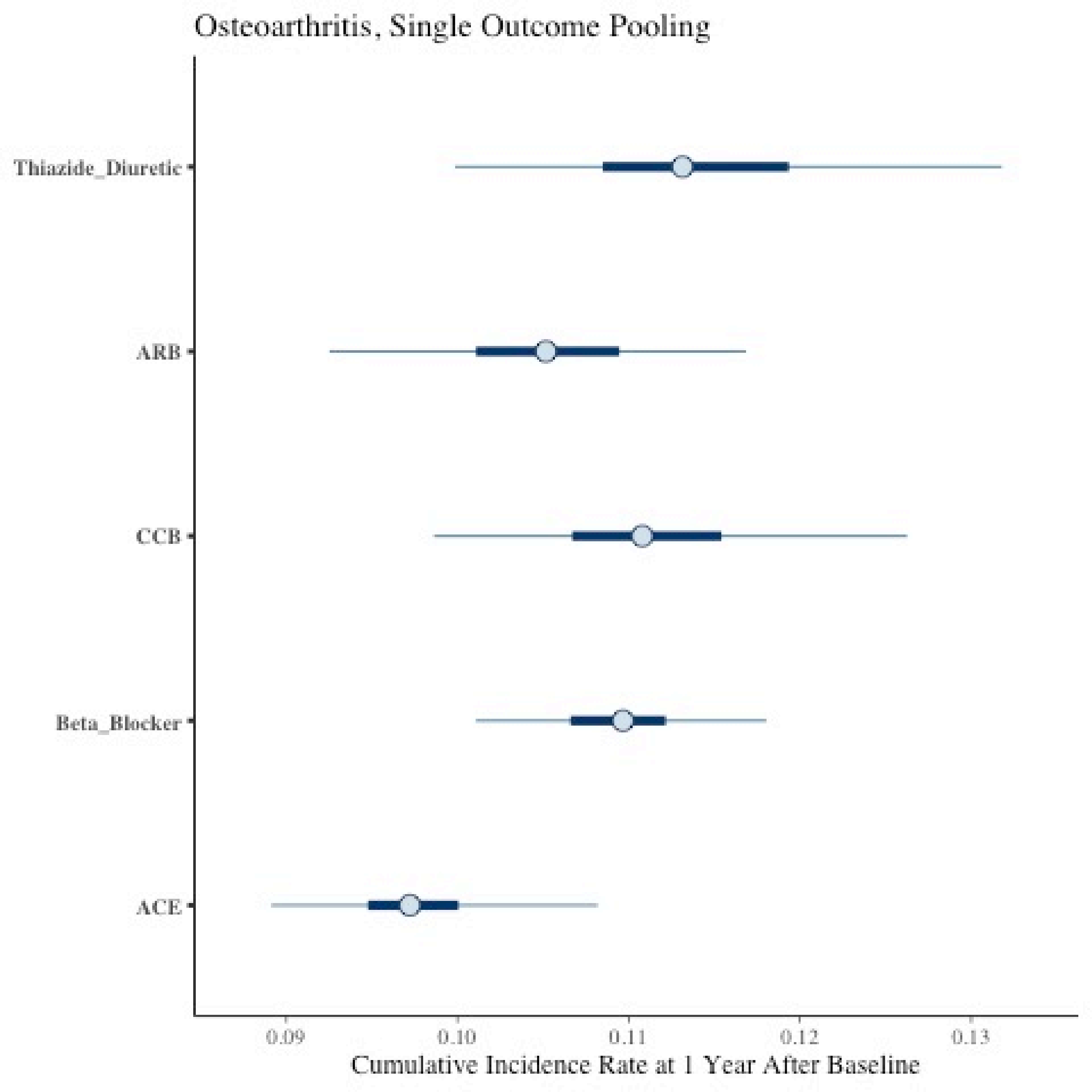

Osteoarthritis, Single Outcome Pooling
Thiazide_Diuretic
ARB
CCB
Beta_Blocker
ACE
0.09
0.10
0.11
0.12
0.13
Cumulative Incidence Rate at 1 Year After Baseline

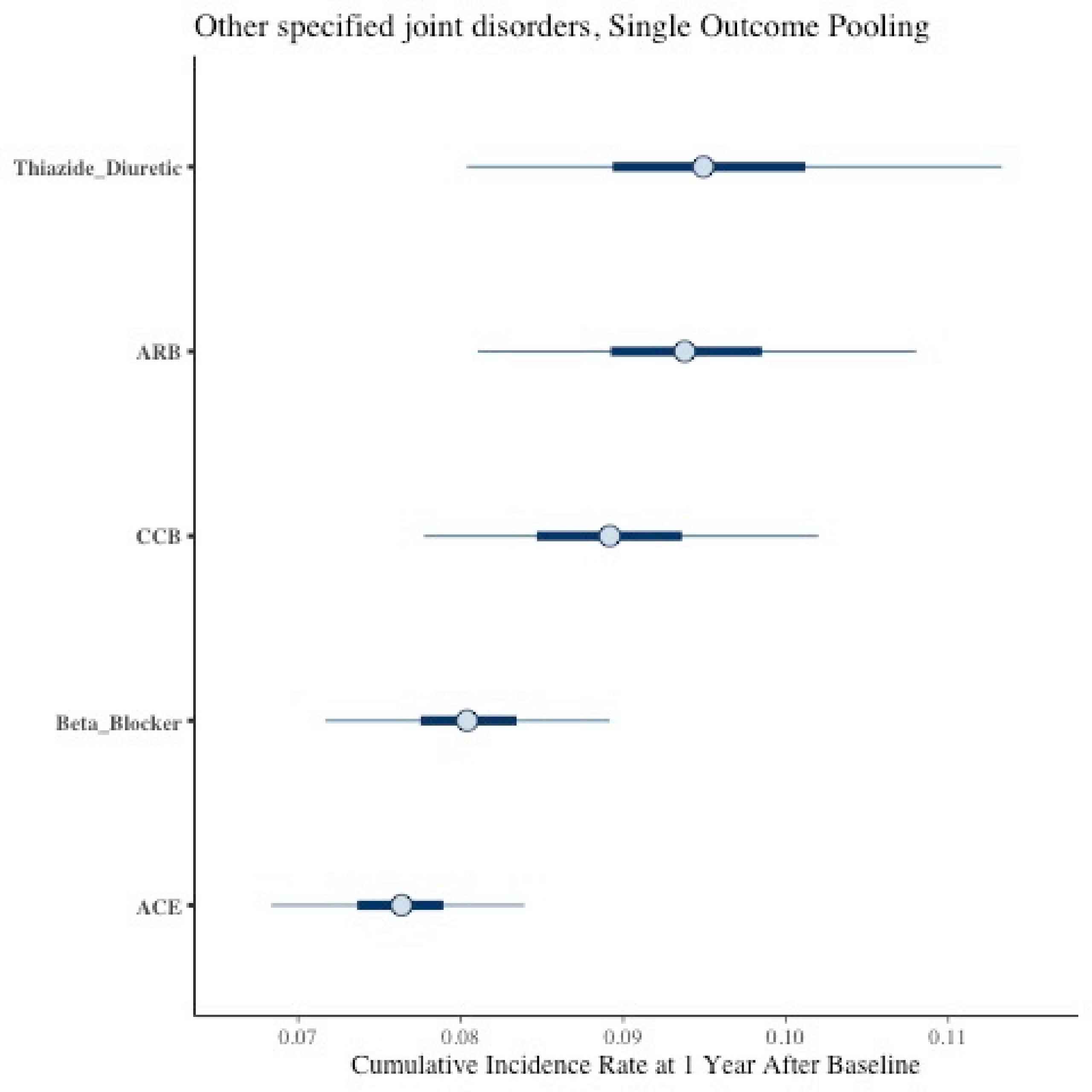

Other specified joint disorders, Single Outcome Pooling
Thiazide_Diuretic
ARB
CCB
Beta_Blocker
ACE
0.07
0.08
0.09
0.10
0.11
Cumulative Incidence Rate at 1 Year After Baseline

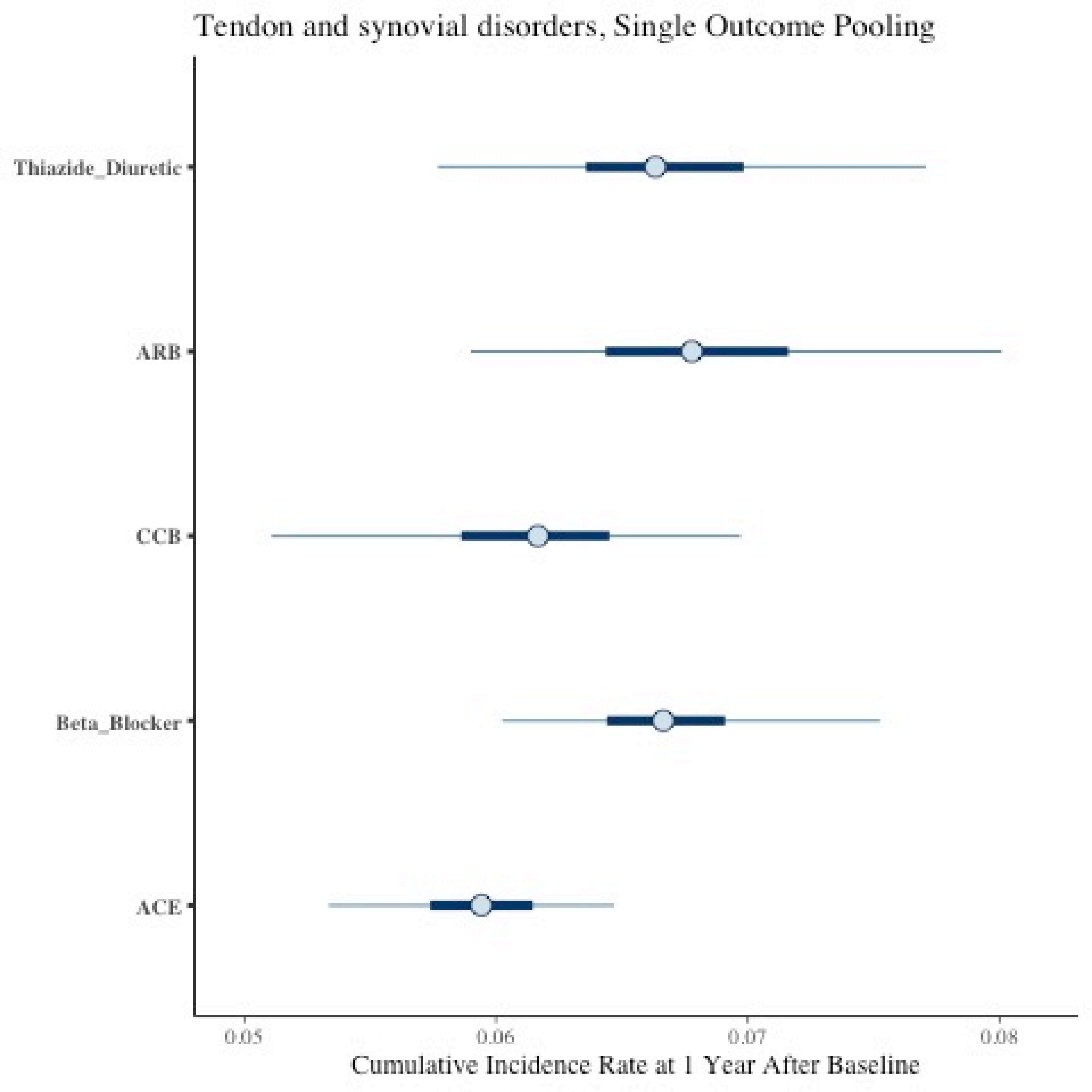

Tendon and synovial disorders, Single Outcome Pooling
Thiazide_Diuretic
ARB
CCB
Beta_Blocker
ACE
0.05
0.06
0.07
0.08
Cumulative Incidence Rate at 1 Year After Baseline

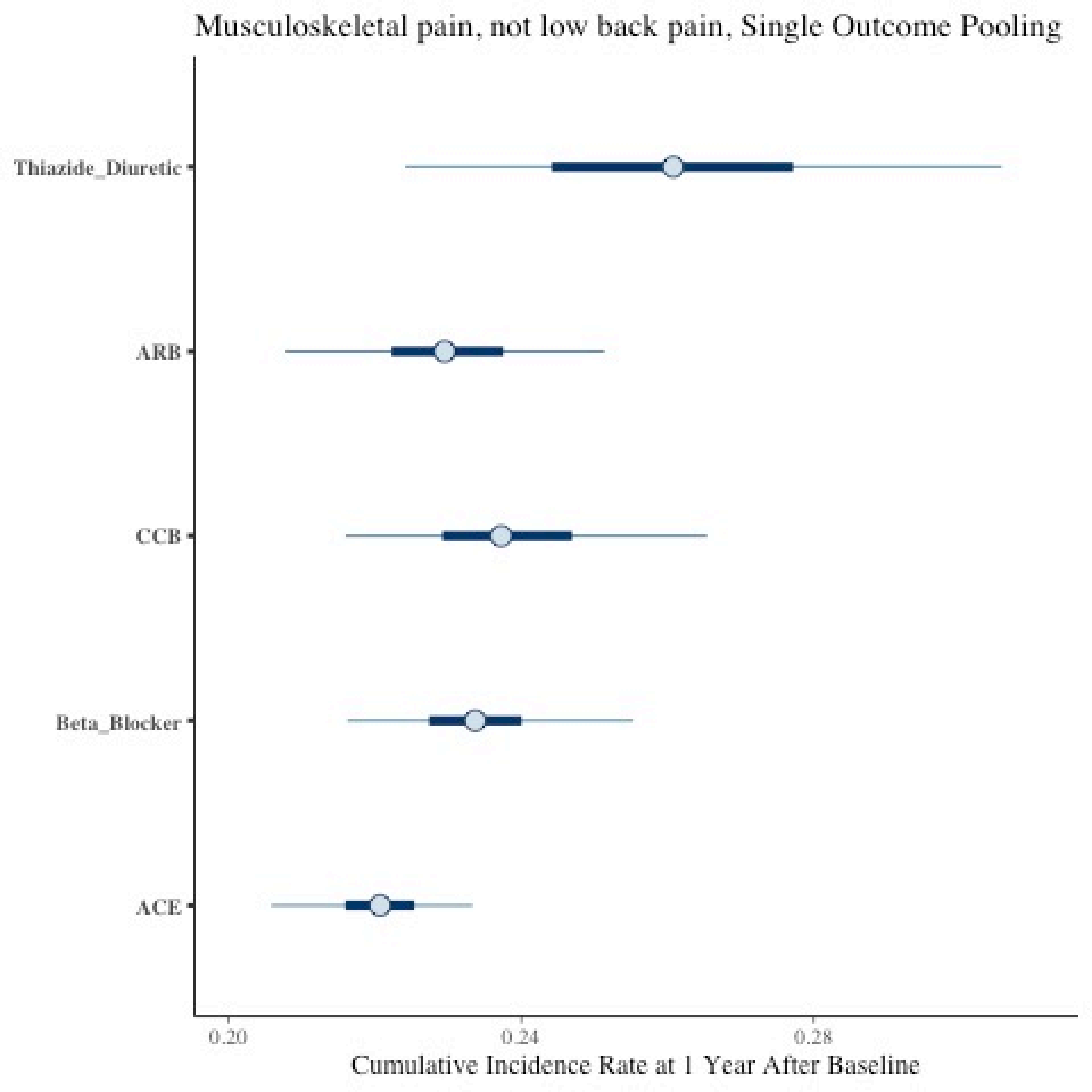
Musculoskeletal pain, not low back pain, Single Outcome Pooling
Thiazide_Diuretic
ARB
CCB
Beta_Blocker
ACE
0.20
0.24
0.28
Cumulative Incidence Rate at 1 Year After Baseline

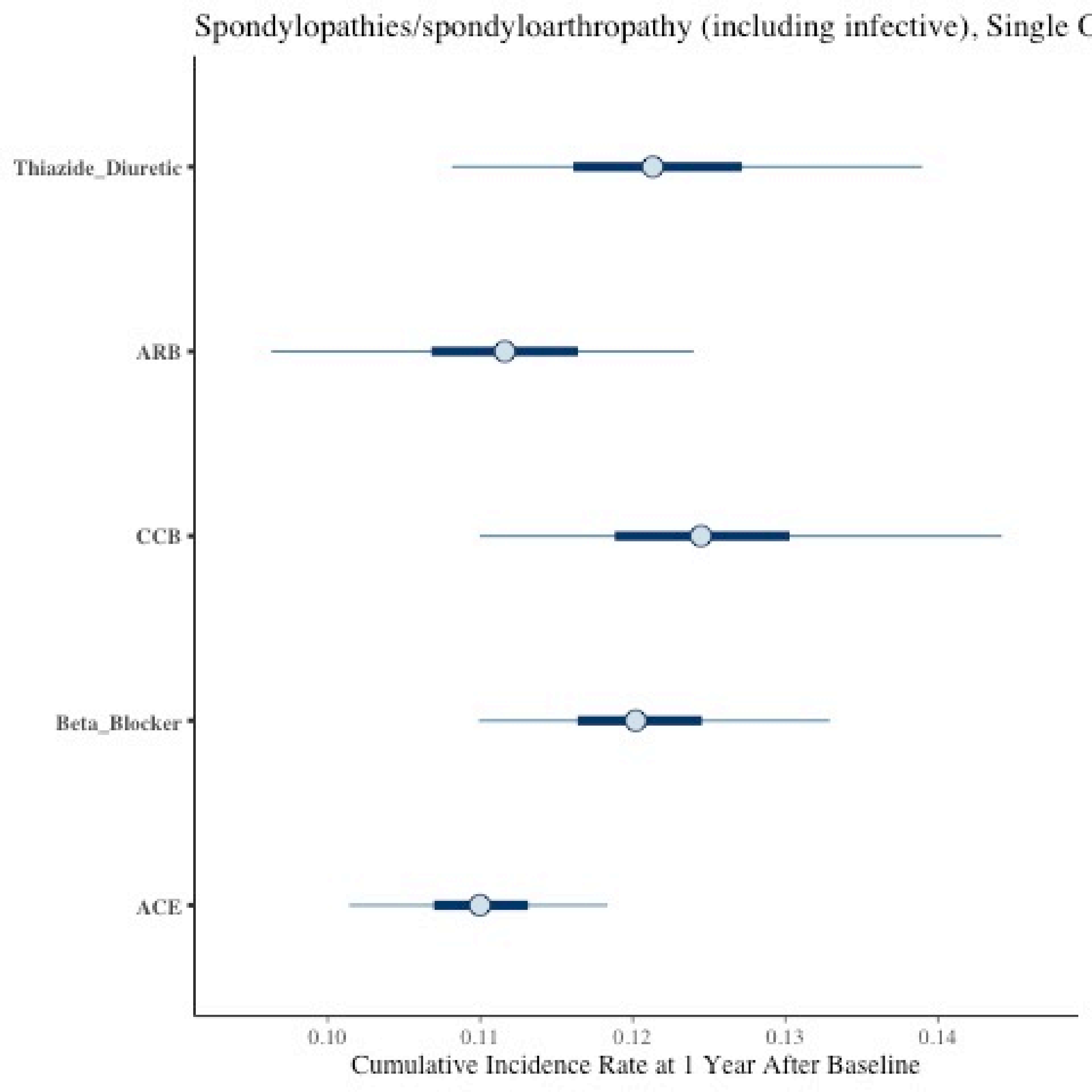

Spondylopathies/spondyloarthropathy (including infective), Single C
Thiazide_Diuretic
ARB
CCB
Beta_Blocker
ACE
0.10
0.11
0.12
0.13
0.14
Cumulative Incidence Rate at 1 Year After Baseline

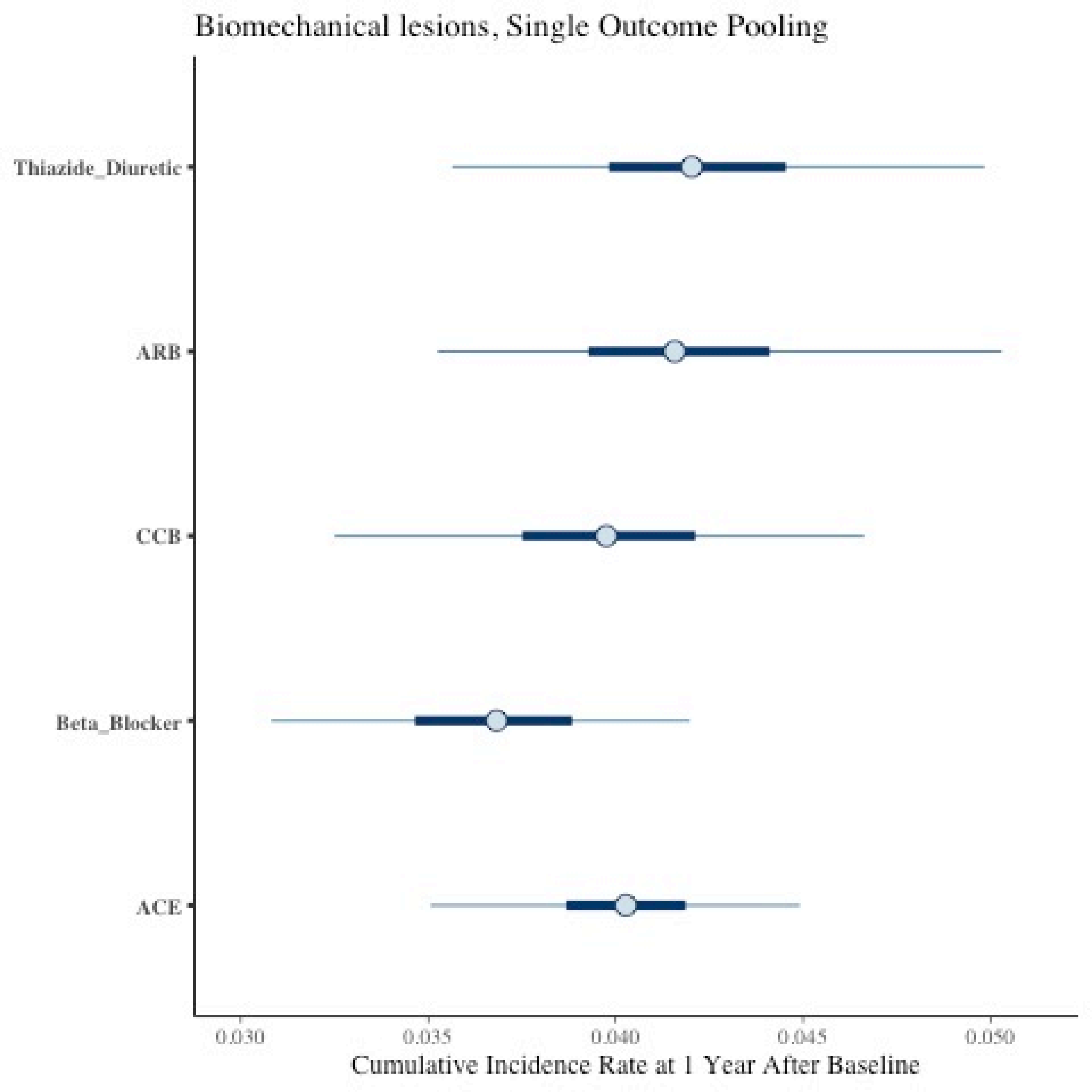
Biomechanical lesions, Single Outcome Pooling
Thiazide_Diuretic
ARB
CCB
Beta_Blocker
ACE
0.030
0.035
0.040
0.045
0.050
Cumulative Incidence Rate at 1 Year After Baseline

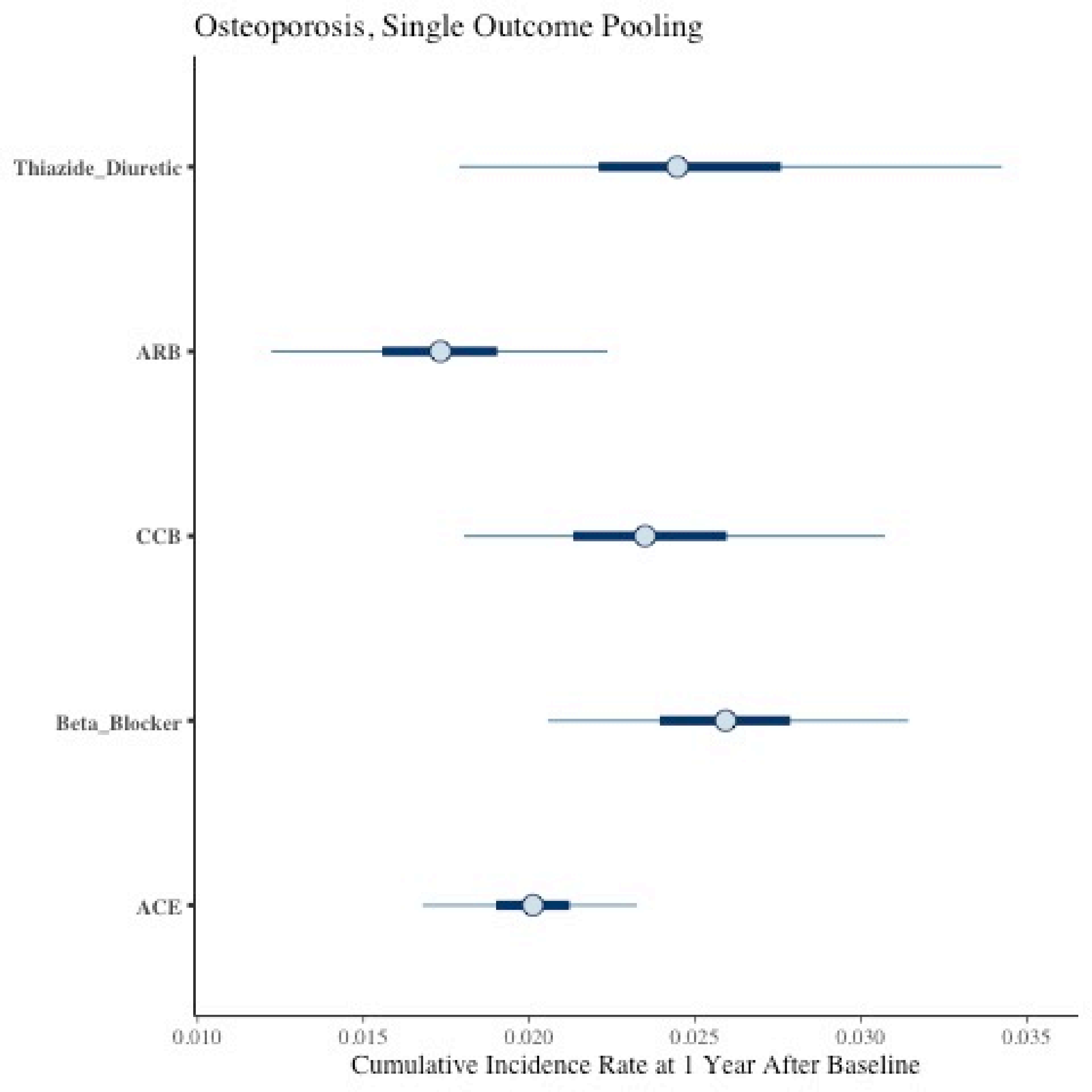

Osteoporosis, Single Outcome Pooling
Thiazide_Diuretic
ARB
CCB
Beta_Blocker
ACE
0.010
0.015
0.020
0.025
0.030
0.035
Cumulative Incidence Rate at 1 Year After Baseline

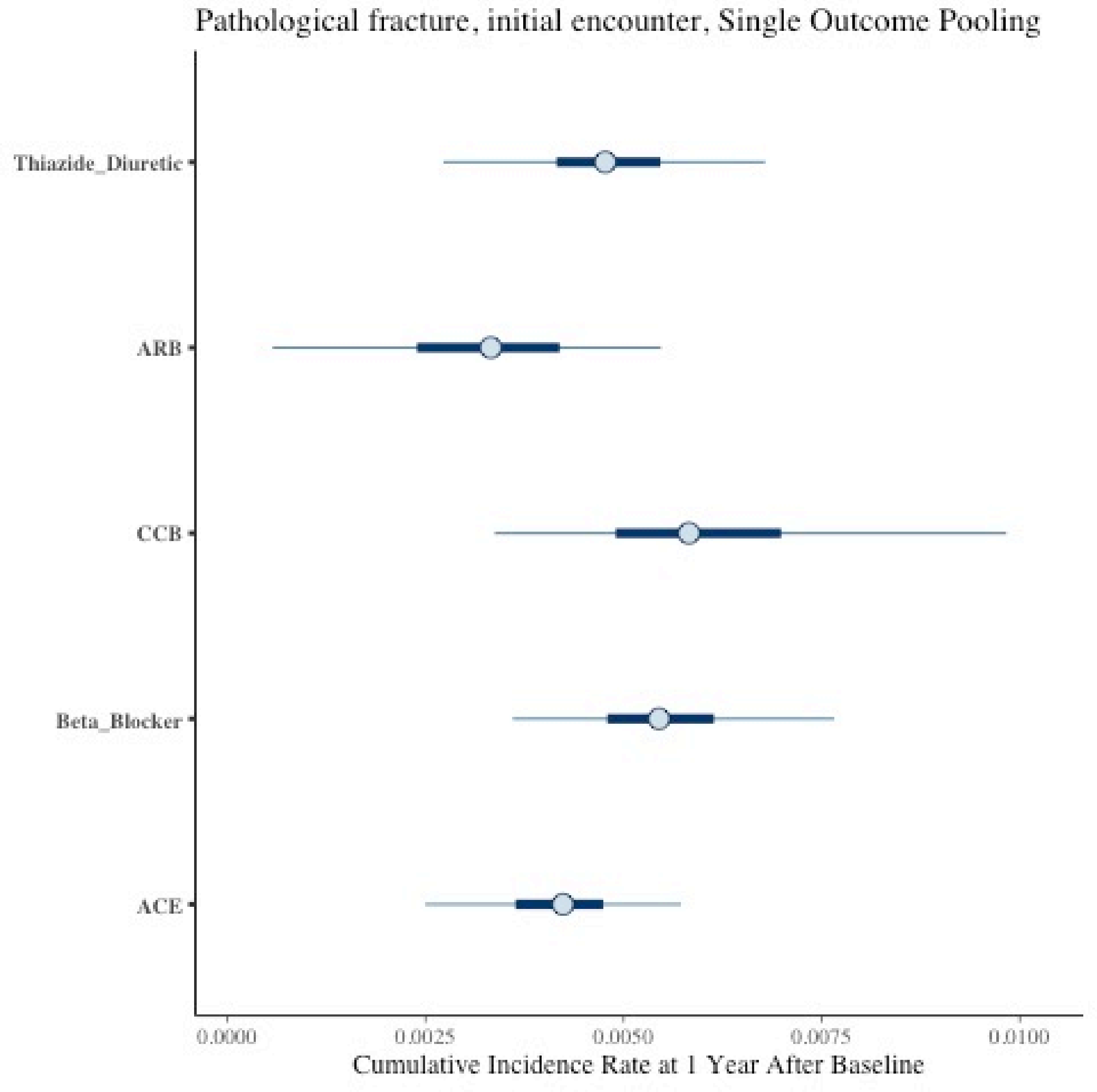

Pathological fracture, initial encounter, Single Outcome Pooling
Thiazide_Diuretic
ARB
CCB
Beta_Blocker
ACE
0.0000
0.0025
0.0050
0.0075
0.0100
Cumulative Incidence Rate at 1 Year After Baseline

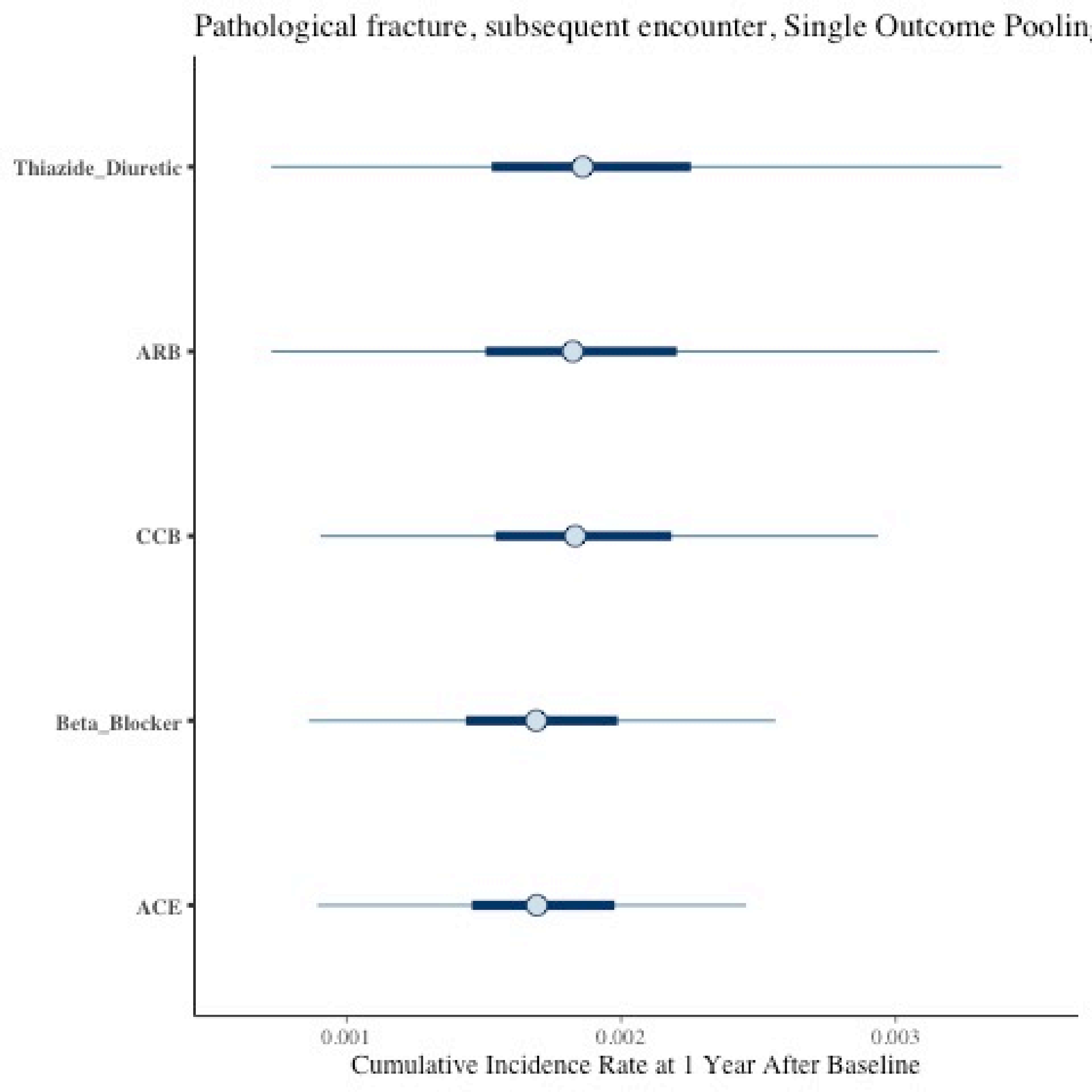

Pathological fracture, subsequent encounter, Single Outcome Poolin
Thiazide_Diuretic
ARB
CCB
Beta_Blocker
ACE
0.001
0.002
0.003
Cumulative Incidence Rate at 1 Year After Baseline

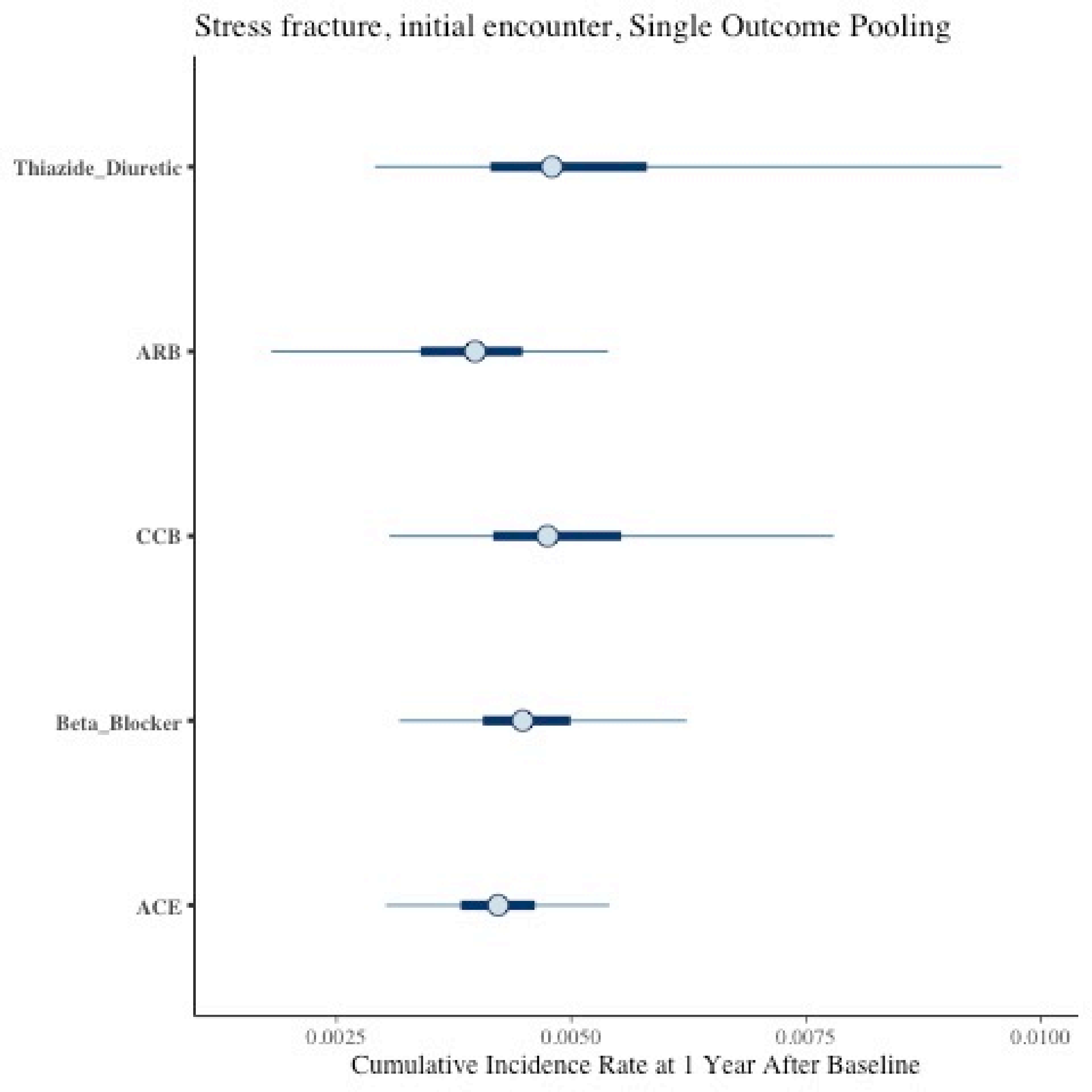

Stress fracture, initial encounter, Single Outcome Pooling
Thiazide_Diuretic
ARB
CCB
Beta_Blocker
ACE
0.0025
0.0050
0.0075
0.0100
Cumulative Incidence Rate at 1 Year After Baseline

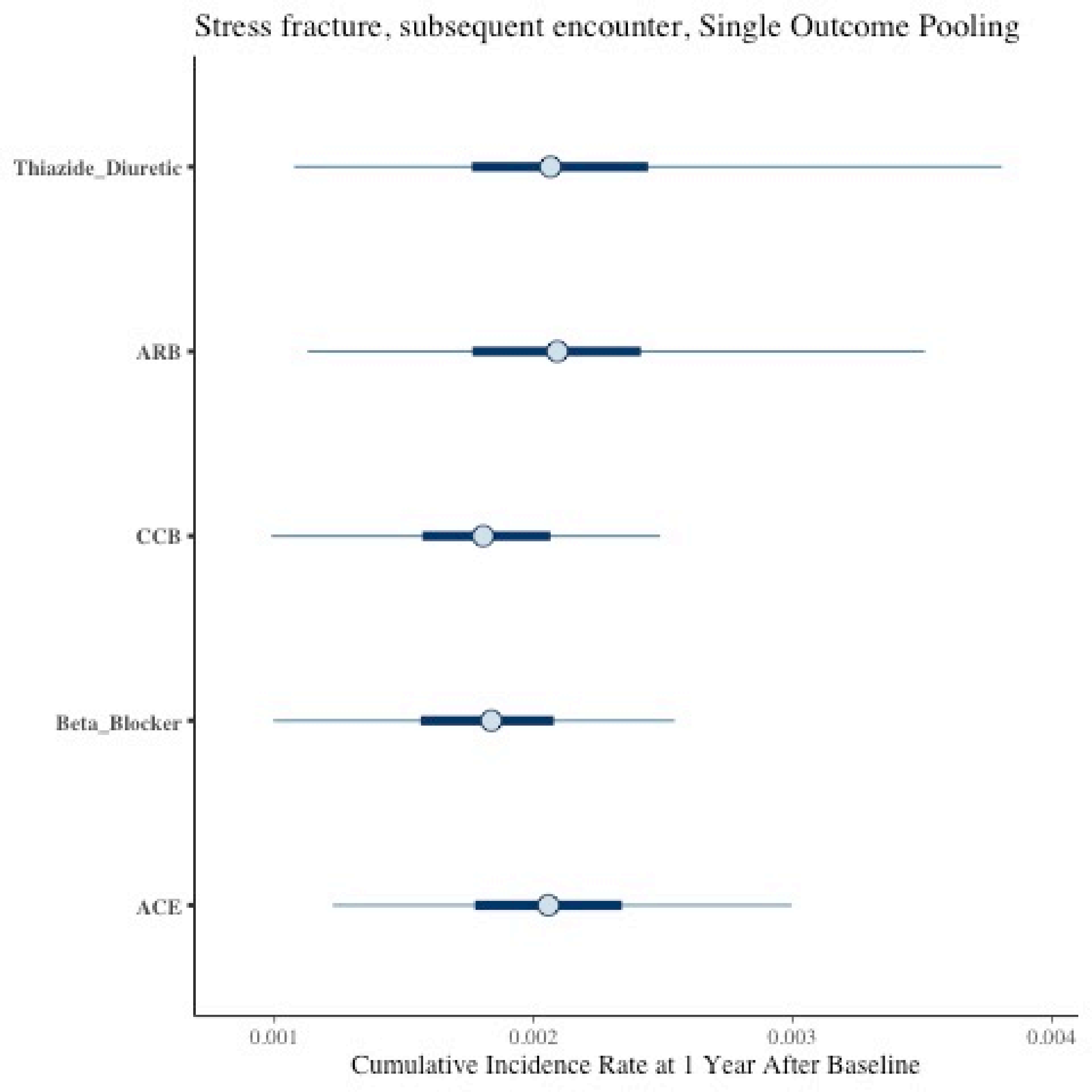

Stress fracture, subsequent encounter, Single Outcome Pooling
Thiazide_Diuretic
ARB
CCB
Beta_Blocker
ACE
0.001
0.002
0.003
0.004
Cumulative Incidence Rate at 1 Year After Baseline

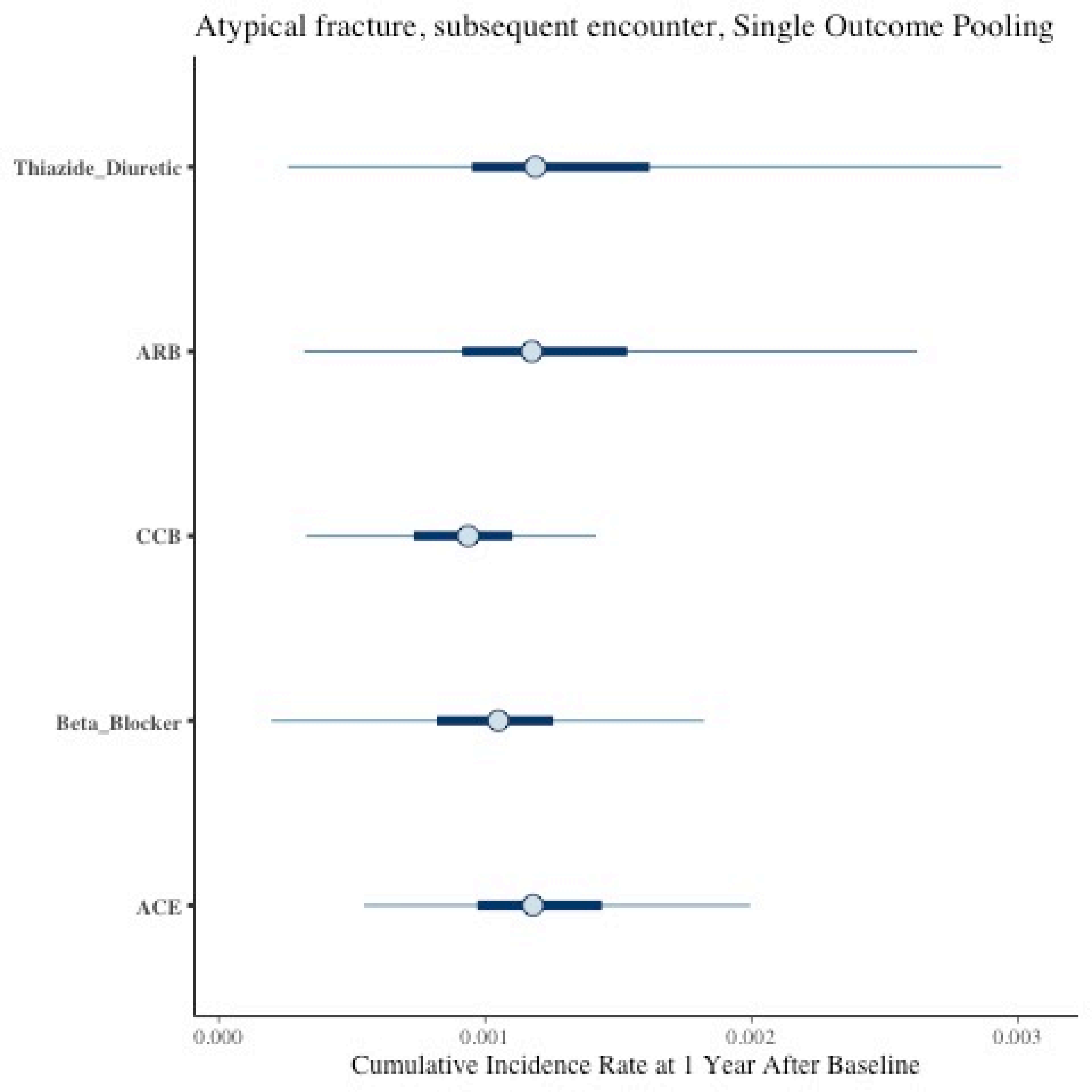
Atypical fracture, subsequent encounter, Single Outcome Pooling
Thiazide_Diuretic
ARB
CCB
Beta_Blocker
ACE
0.000
0.001
0.002
0.003
Cumulative Incidence Rate at 1 Year After Baseline

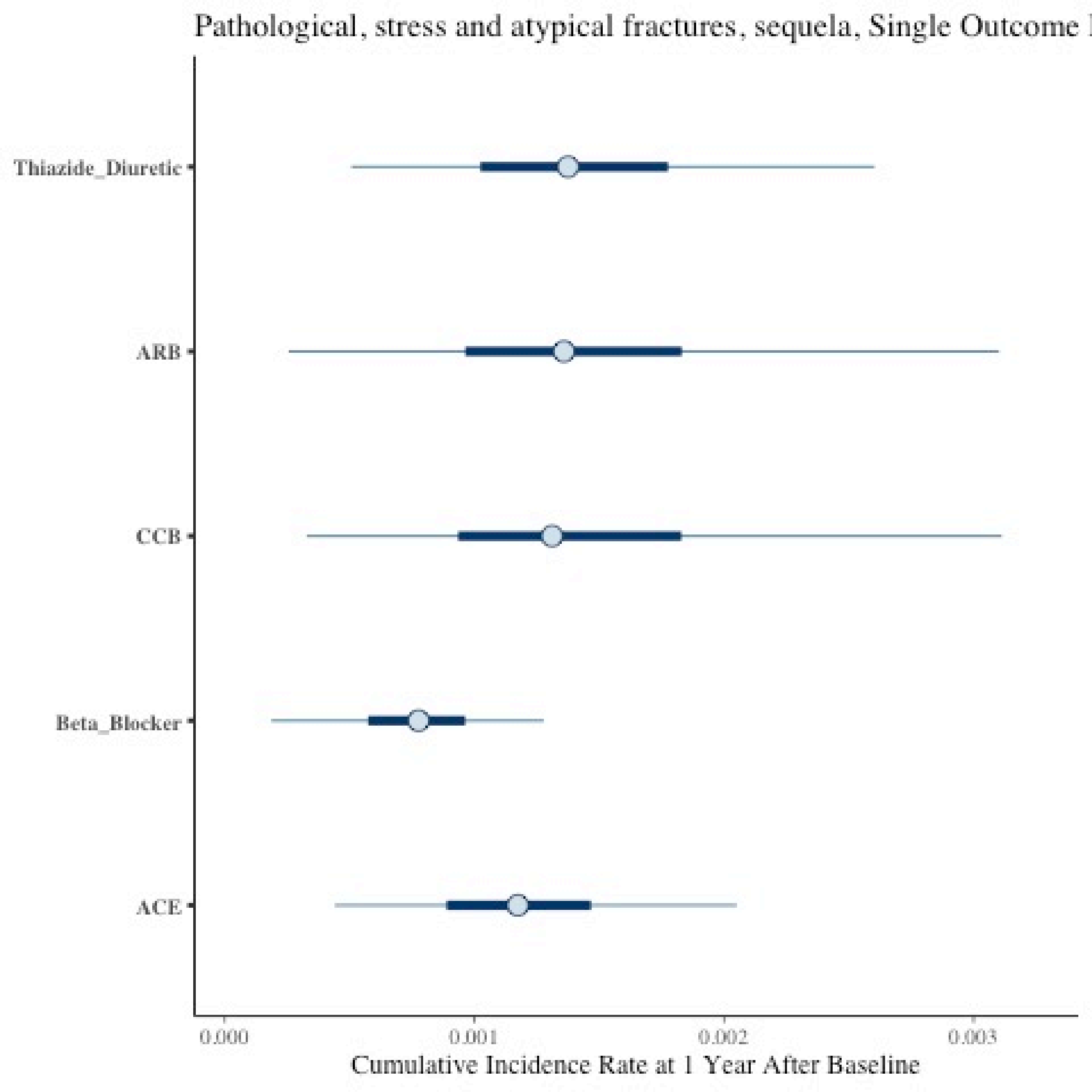

Pathological, stress and atypical fractures, sequela, Single Outcome
Thiazide_Diuretic
ARB
CCB
Beta_Blocker
ACE
0.000
0.001
0.002
0.003
Cumulative Incidence Rate at 1 Year After Baseline

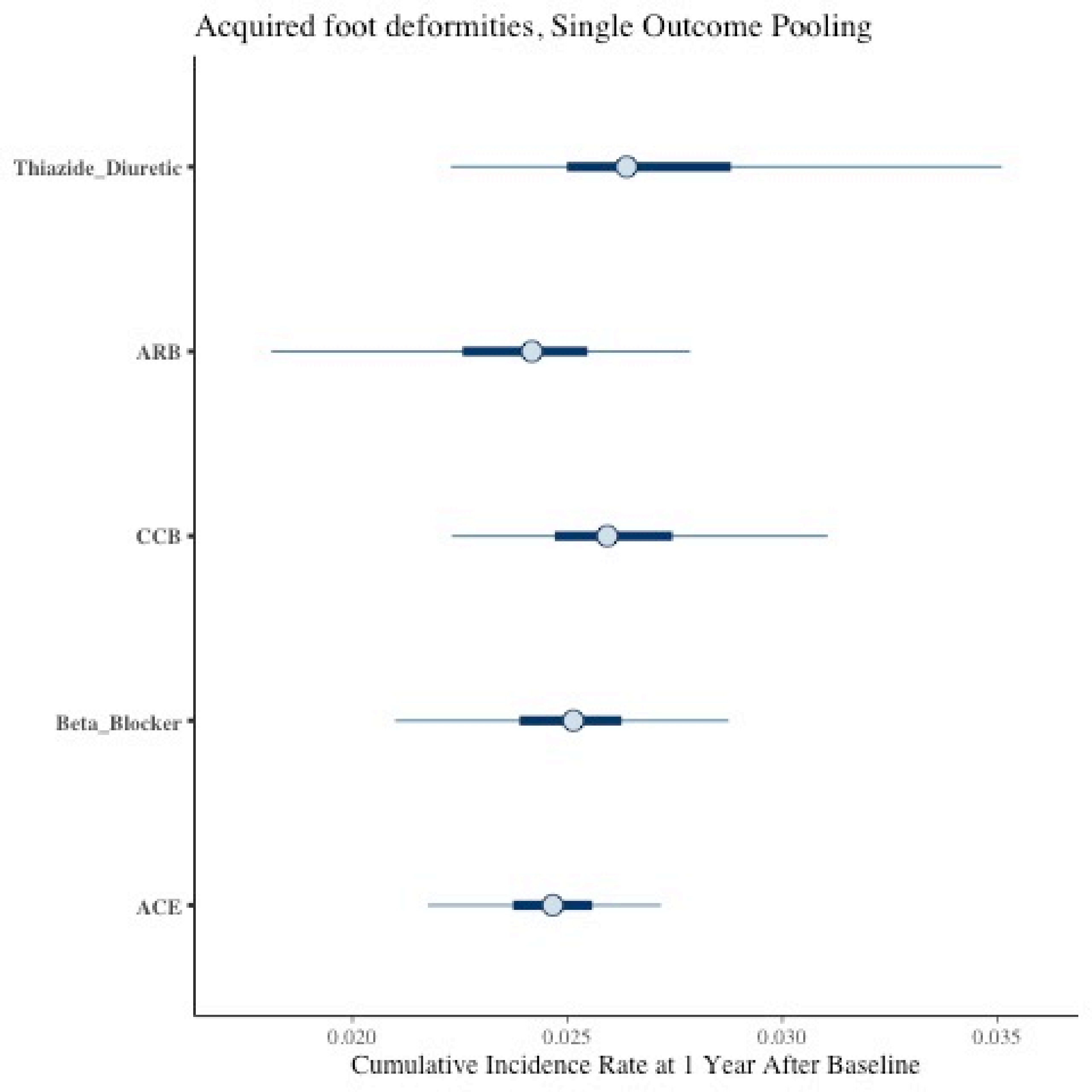

Acquired foot deformities, Single Outcome Pooling
Thiazide_Diuretic
ARB
CCB
Beta_Blocker
ACE
0.020
0.025
0.030
0.035
Cumulative Incidence Rate at 1 Year After Baseline

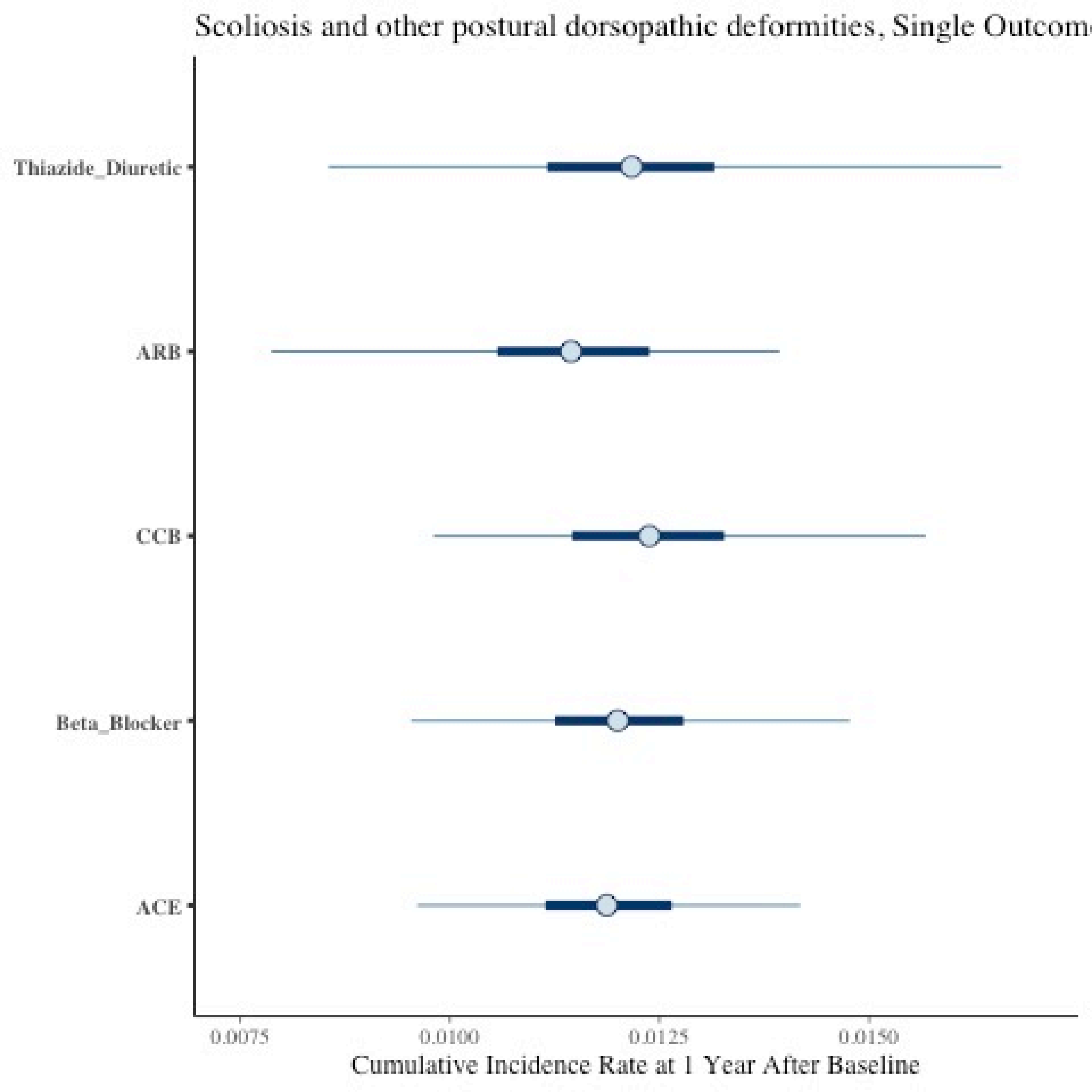

Scoliosis and other postural dorsopathic deformities, Single Outcome
Thiazide_Diuretic
ARB
CCB
Beta_Blocker
ACE
0.0075
0.0100
0.0125
0.0150
Cumulative Incidence Rate at 1 Year After Baseline

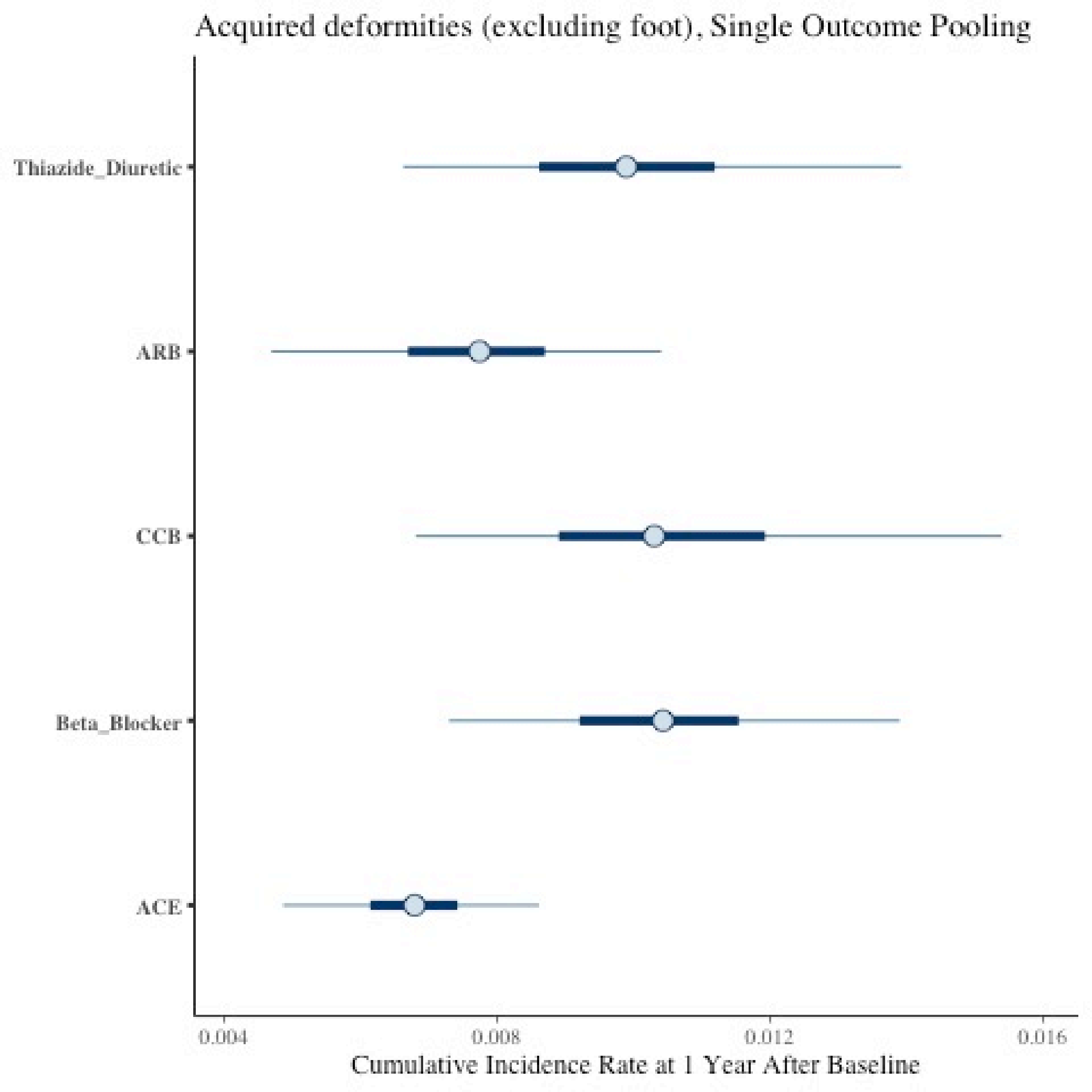

Acquired deformities (excluding foot), Single Outcome Pooling
Thiazide_Diuretic
ARB
CCB
Beta_Blocker
ACE
0.004
0.008
0.012
0.016
Cumulative Incidence Rate at 1 Year After Baseline

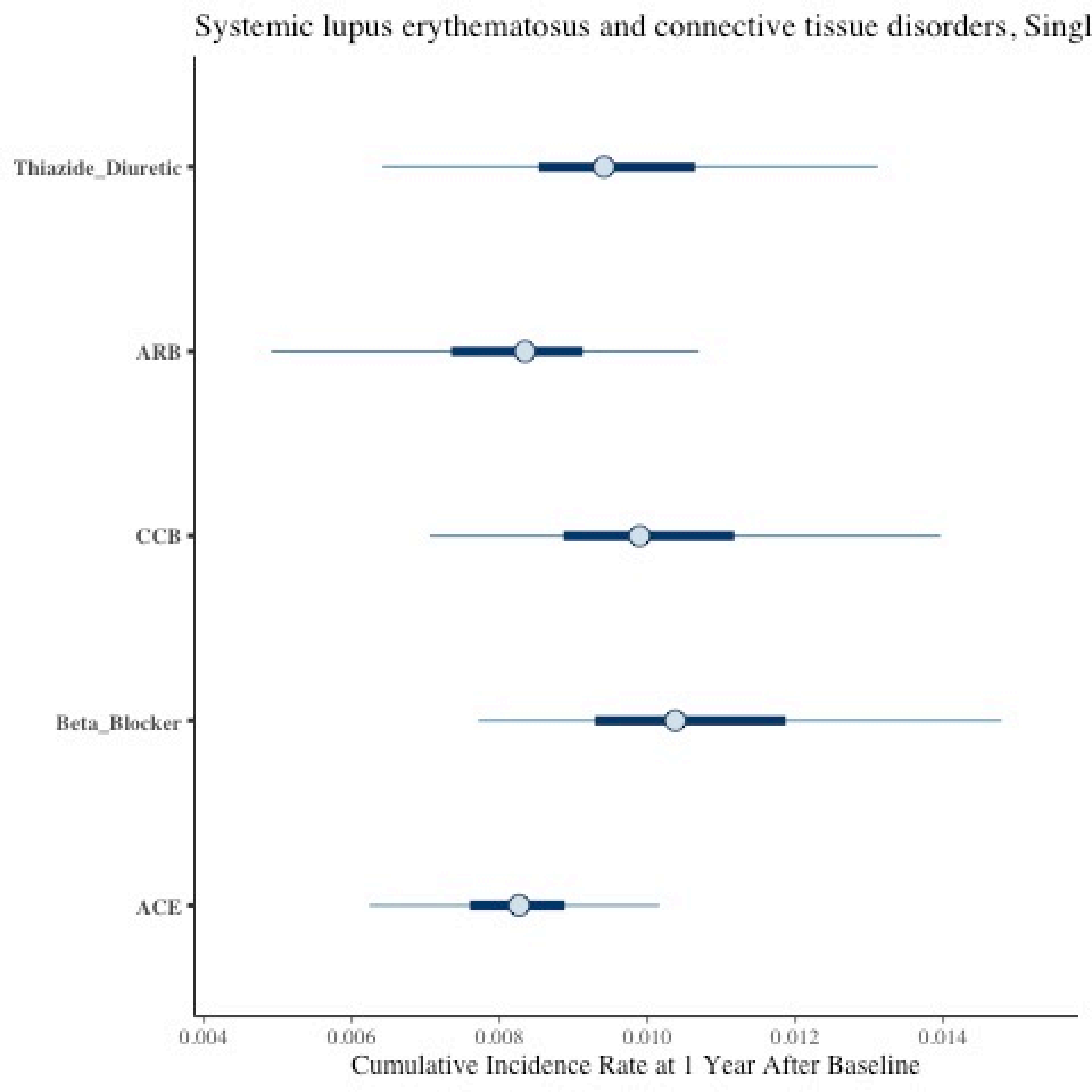
Systemic lupus erythematosus and connective tissue disorders, Singl
Thiazide_Diuretic
ARB
CCB
Beta_Blocker
ACE
0.004
0.006
0.008
0.010
0.012
0.014
Cumulative Incidence Rate at 1 Year After Baseline

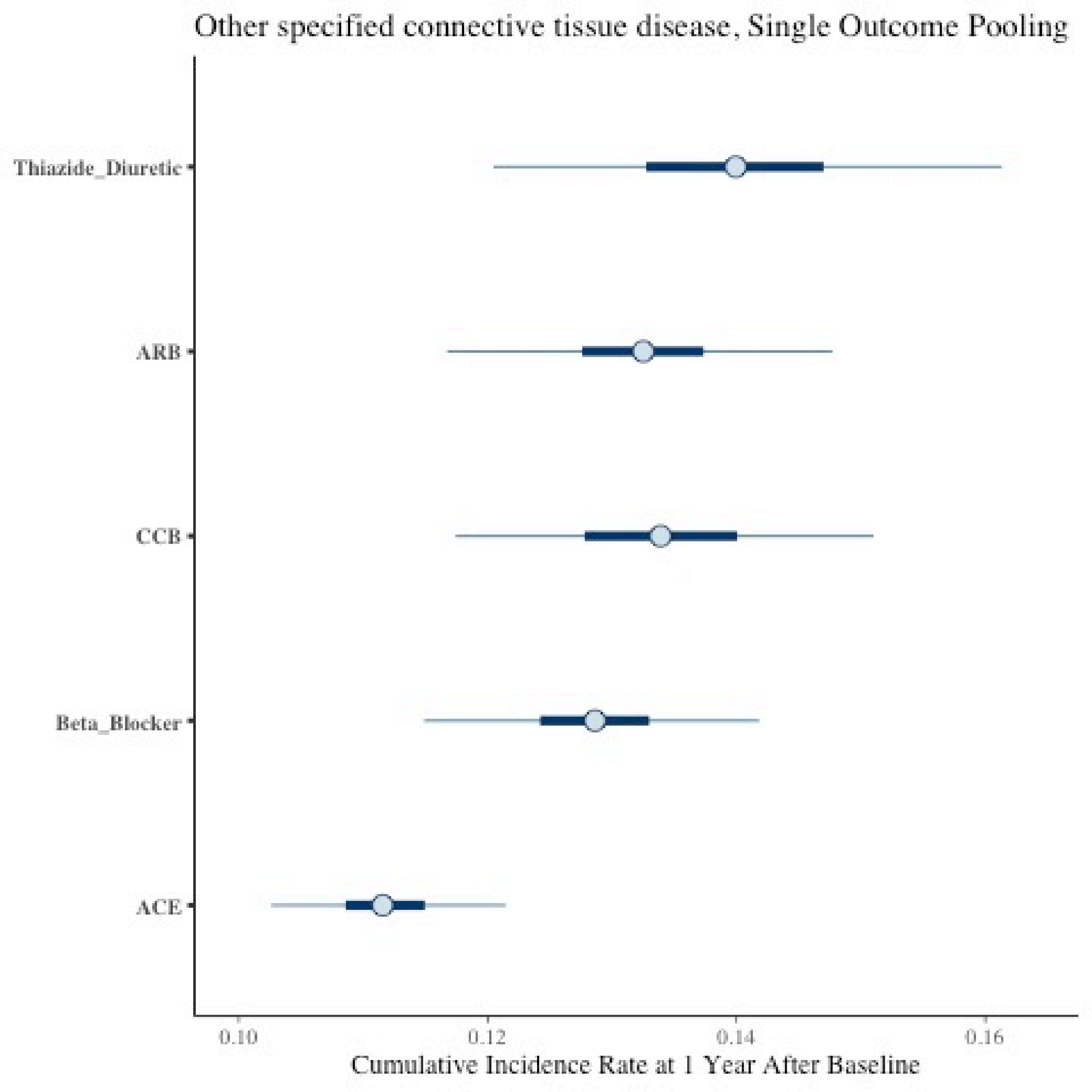

Other specified connective tissue disease, Single Outcome Pooling
Thiazide_Diuretic
ARB
CCB
Beta_Blocker
ACE
0.10
0.12
0.14
0.16
Cumulative Incidence Rate at 1 Year After Baseline

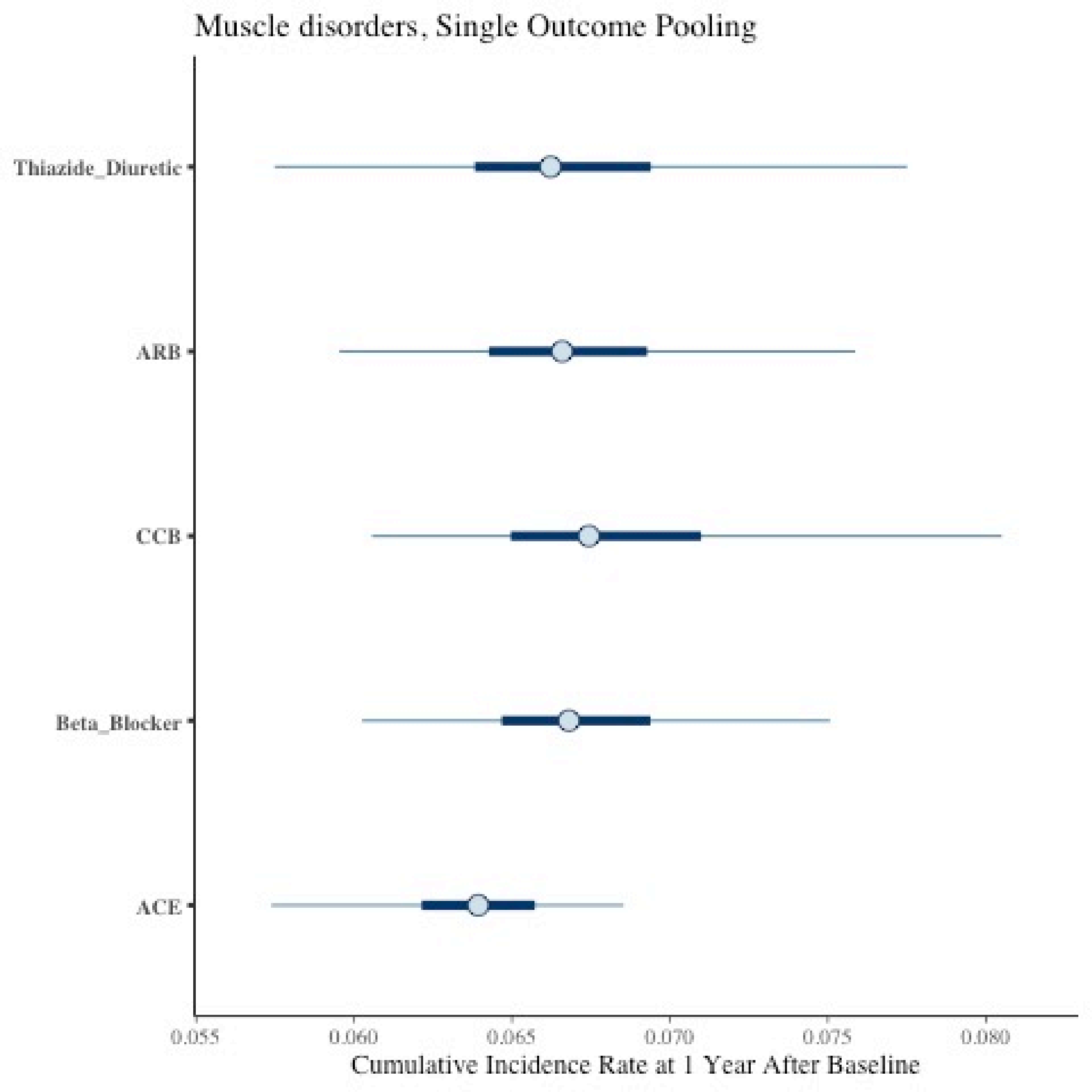

Muscle disorders, Single Outcome Pooling
Thiazide_Diuretic
ARB
CCB
Beta_Blocker
ACE
0.055
0.060
0.065
0.070
0.075
0.080
Cumulative Incidence Rate at 1 Year After Baseline

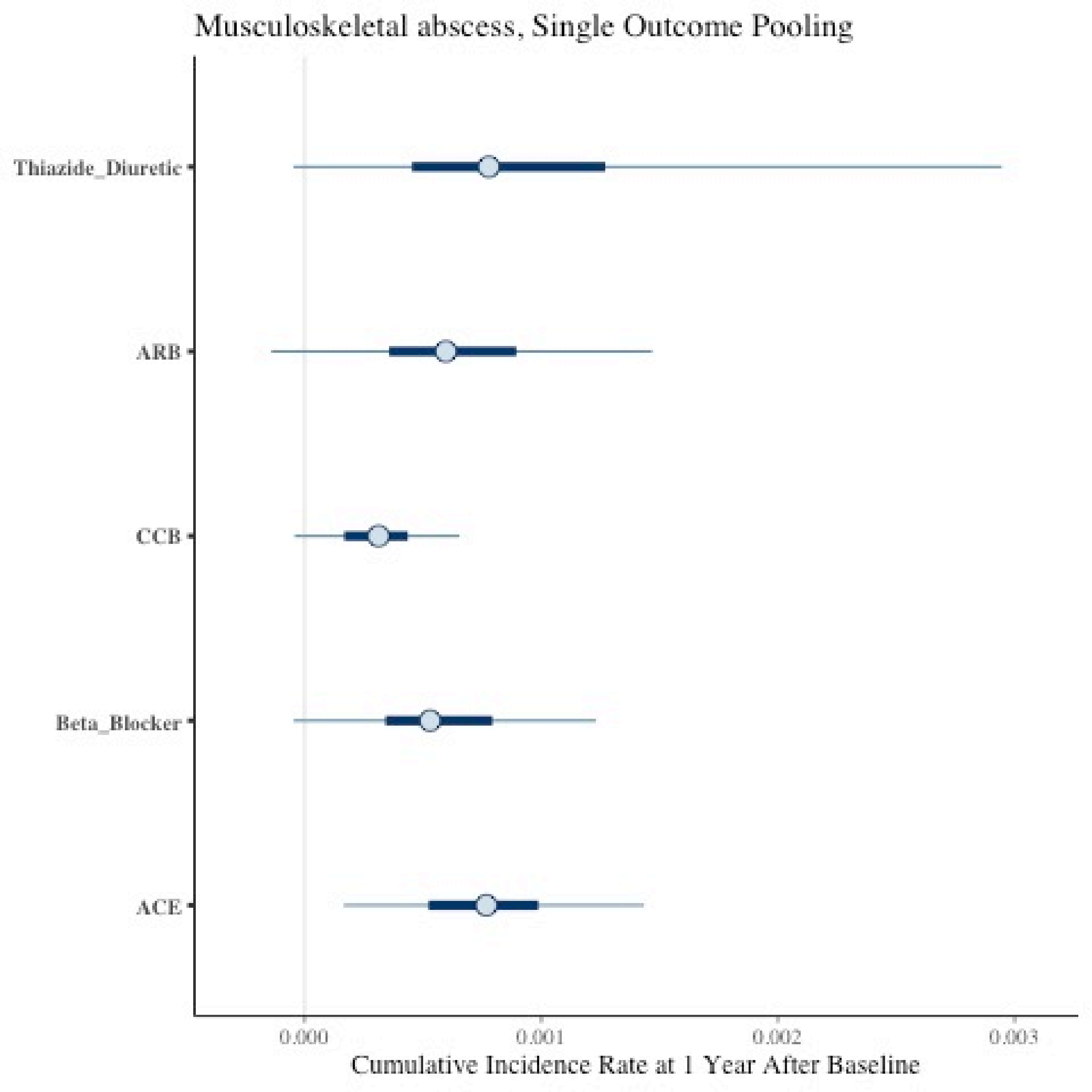

Musculoskeletal abscess, Single Outcome Pooling
Thiazide_Diuretic
ARB
CCB
Beta_Blocker
ACE
0.000
0.001
0.002
0.003
Cumulative Incidence Rate at 1 Year After Baseline

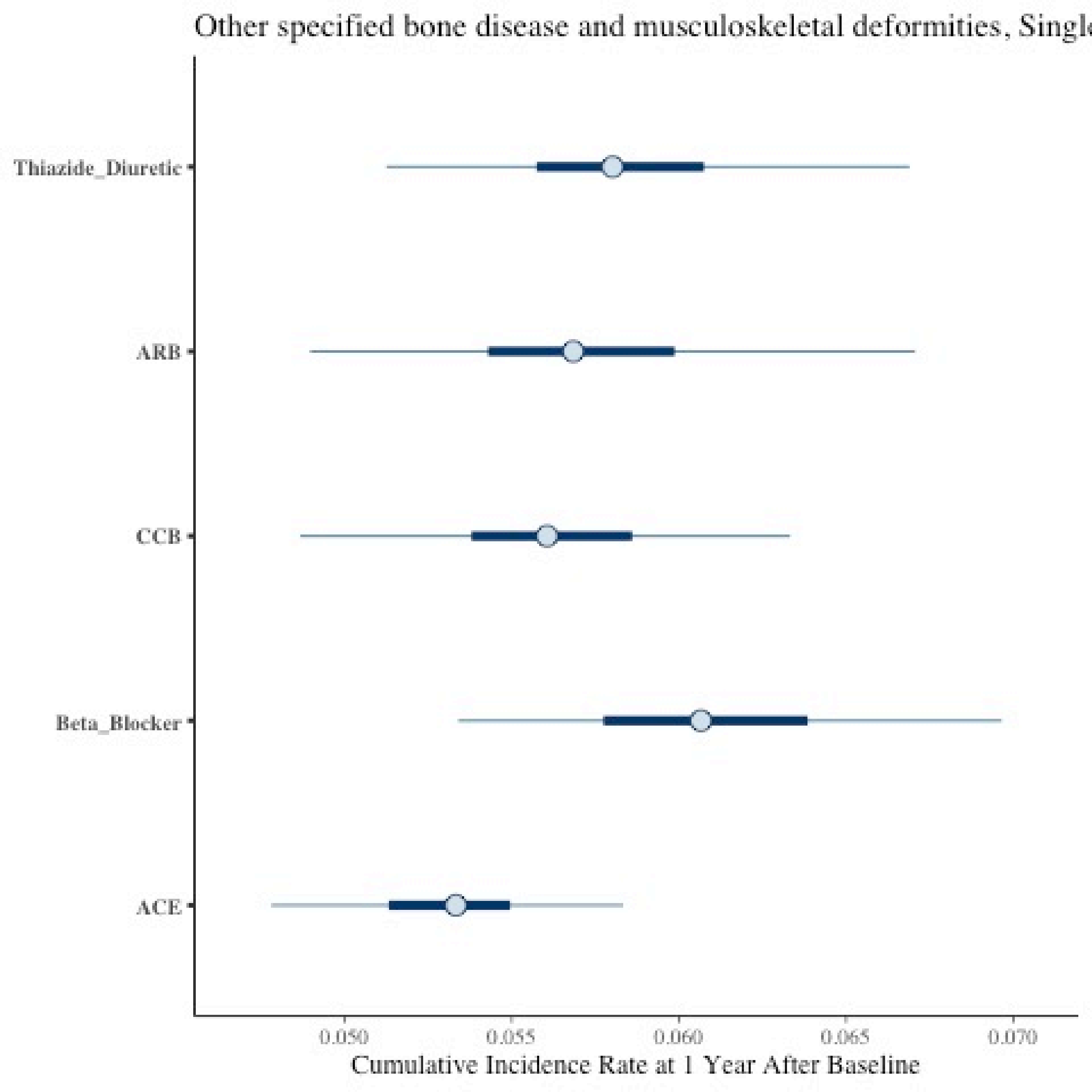

Other specified bone disease and musculoskeletal deformities, Singl
Thiazide_Diuretic
ARB
CCB
Beta_Blocker
ACE
0.050
0.055
0.060
0.065
0.070
Cumulative Incidence Rate at 1 Year After Baseline

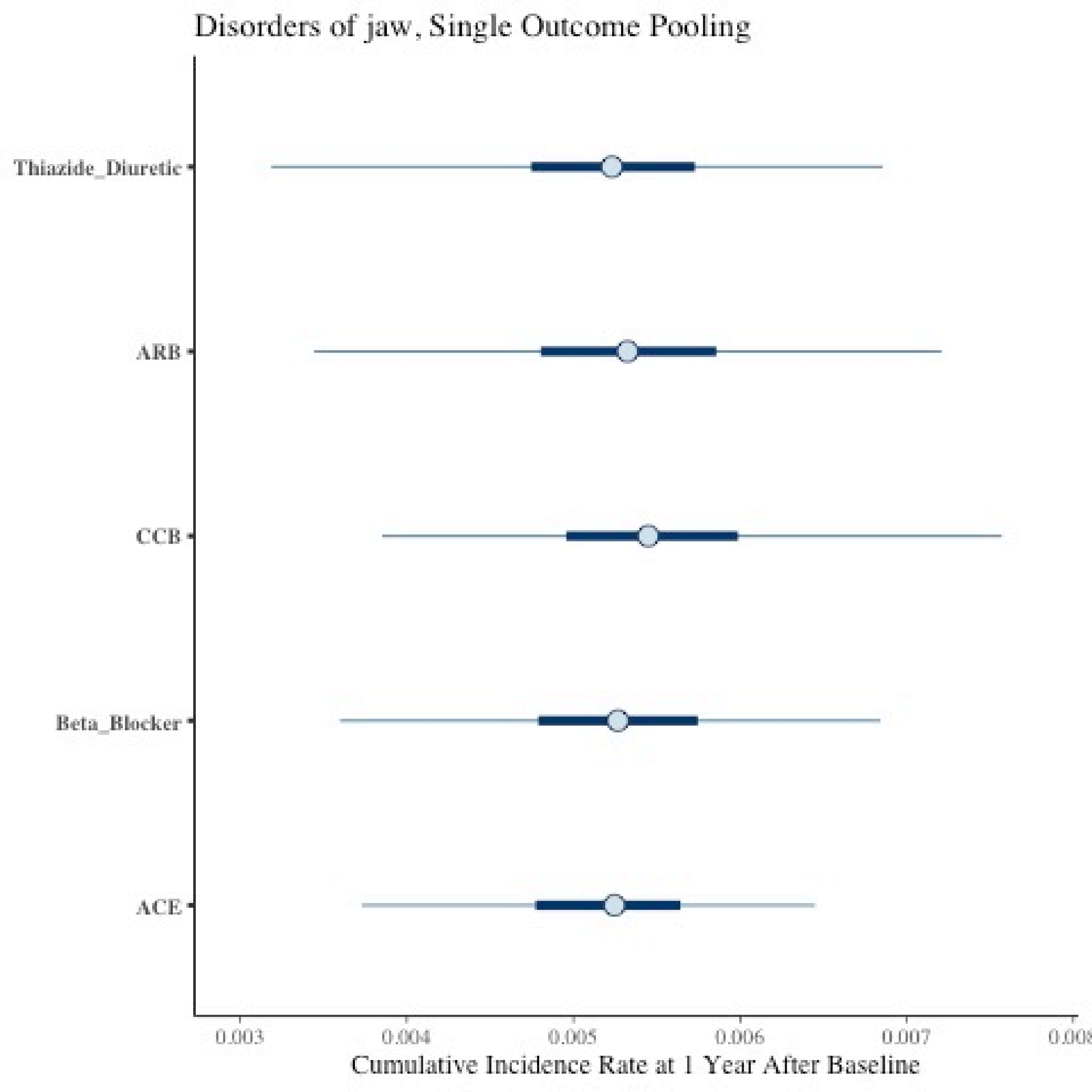

Disorders of jaw, Single Outcome Pooling
Thiazide_Diuretic
ARB
CCB
Beta_Blocker
ACE
0.003
0.004
0.005
0.006
0.007
0.008
Cumulative Incidence Rate at 1 Year After Baseline

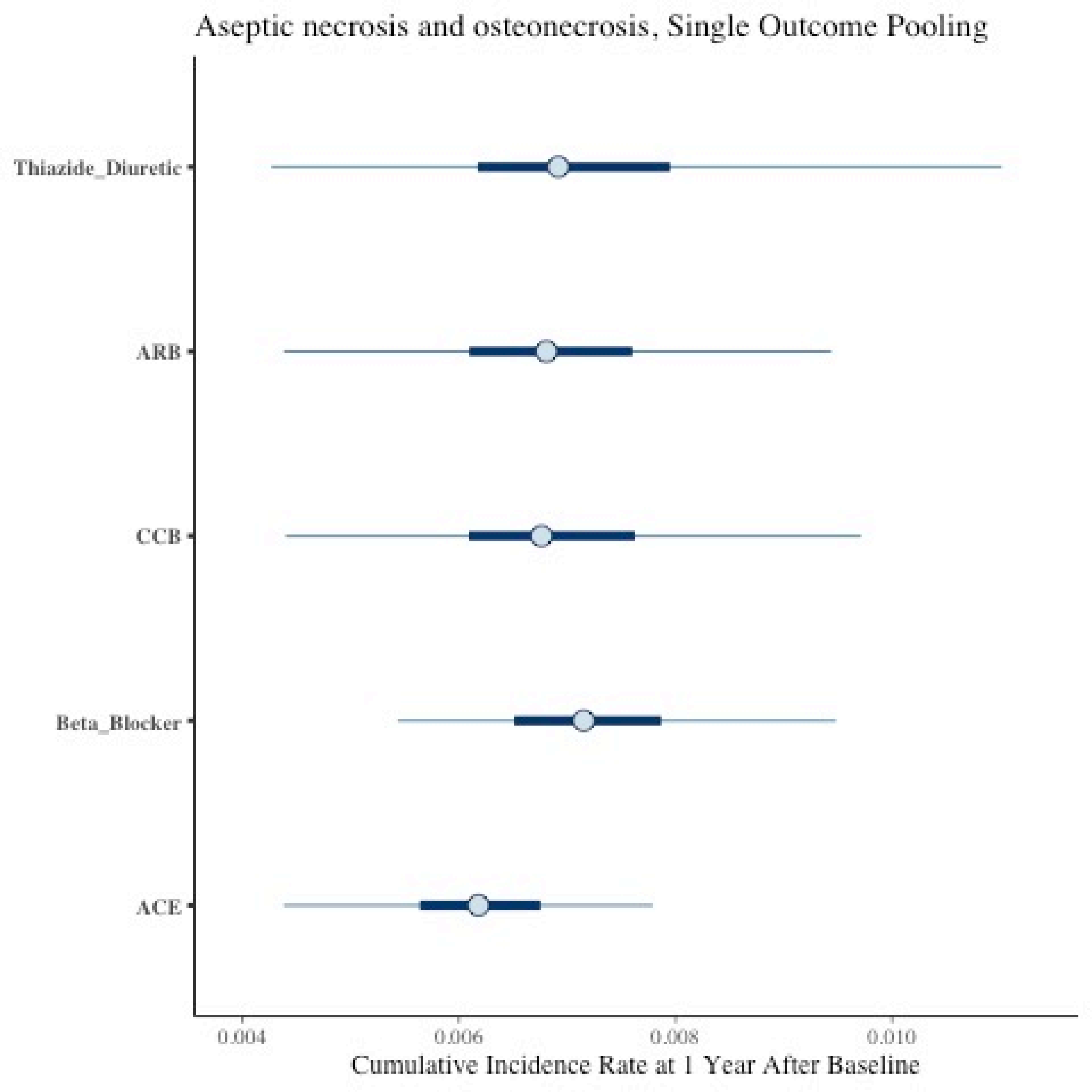

Aseptic necrosis and osteonecrosis, Single Outcome Pooling
Thiazide_Diuretic
ARB
CCB
Beta_Blocker
ACE
0.004
0.006
0.008
0.010
Cumulative Incidence Rate at 1 Year After Baseline

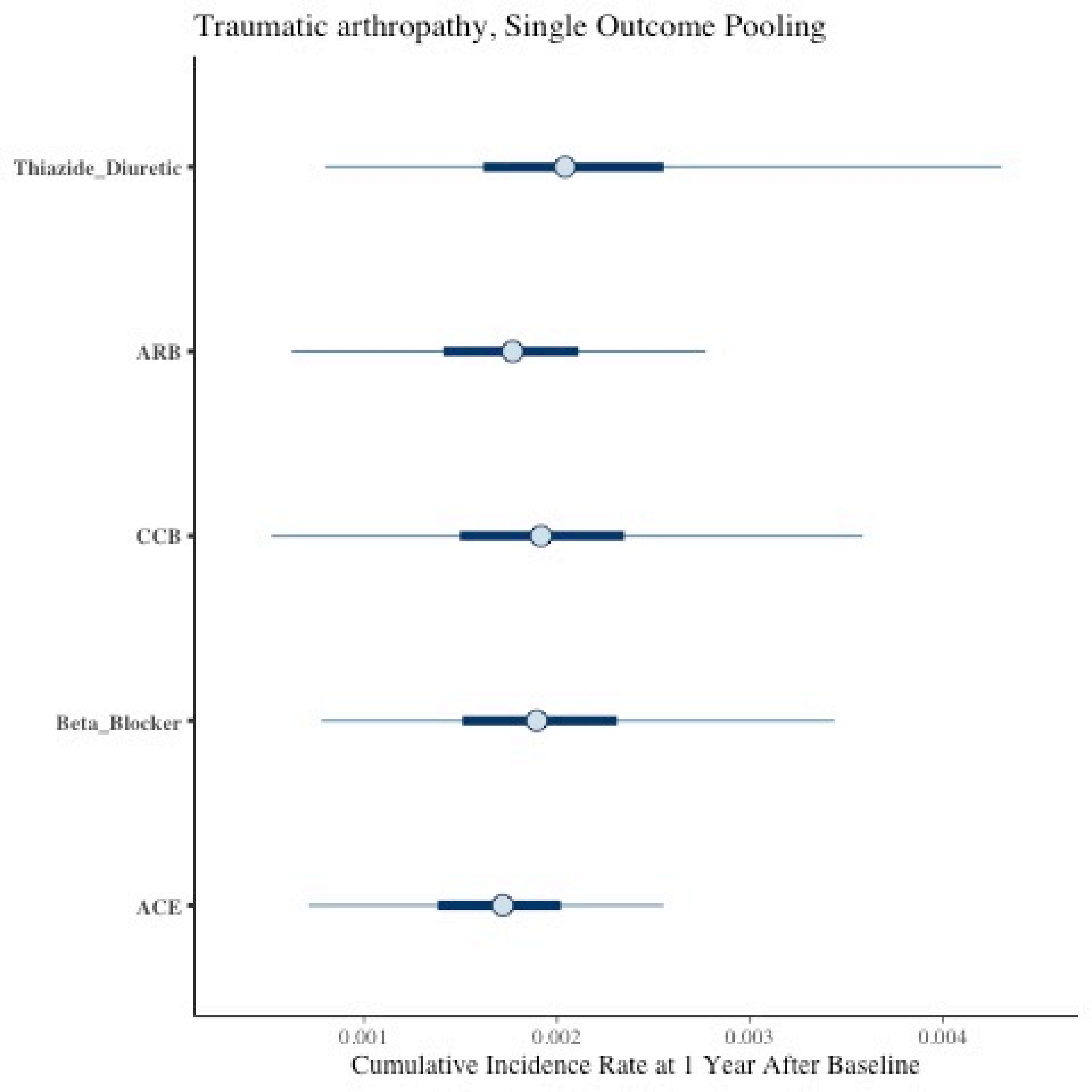

Traumatic arthropathy, Single Outcome Pooling
Thiazide_Diuretic
ARB
CCB
Beta_Blocker
ACE
0.001
0.002
0.003
0.004
Cumulative Incidence Rate at 1 Year After Baseline

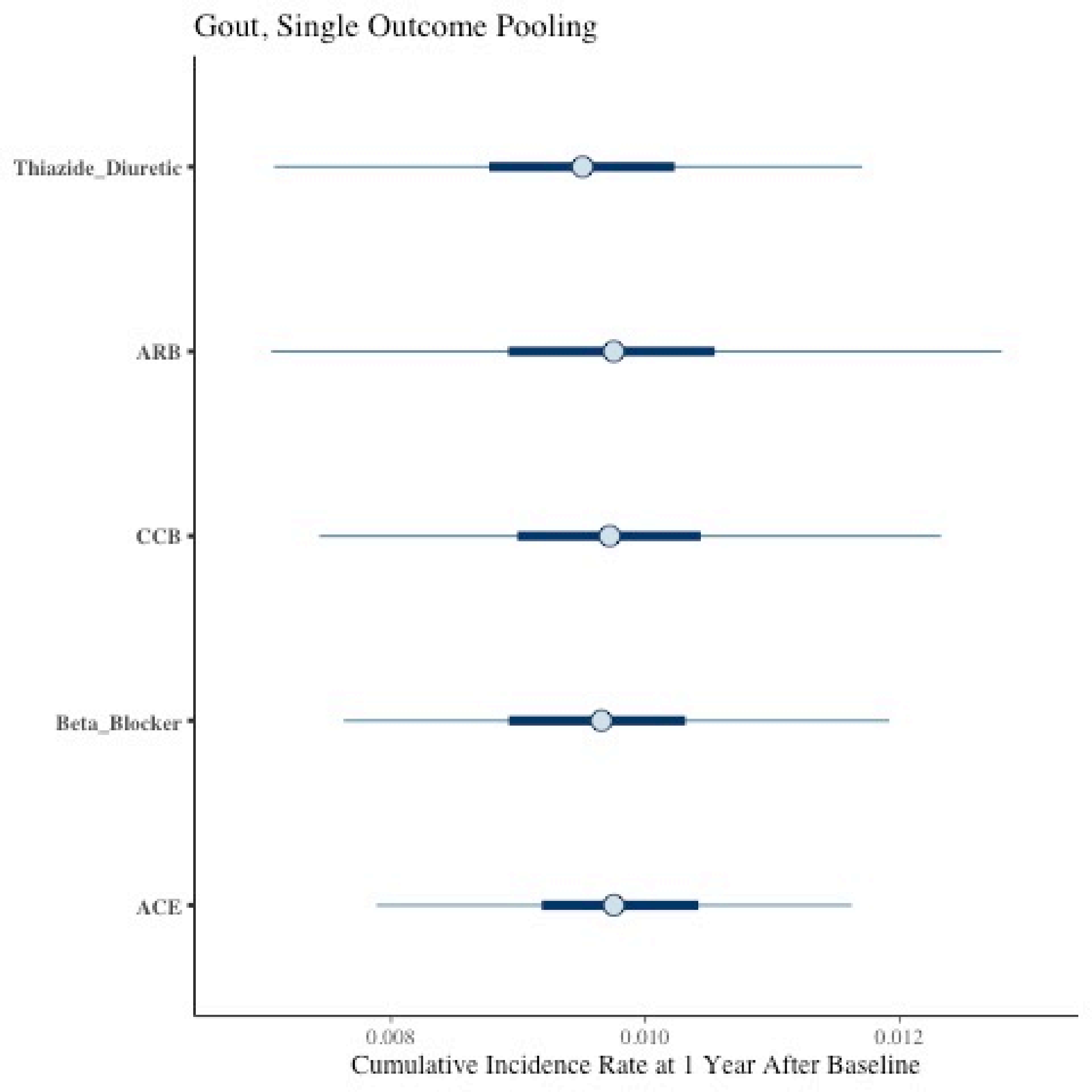
Gout, Single Outcome Pooling
Thiazide_Diuretic
ARB
CCB
Beta_Blocker
ACE
0.008
0.010
0.012
Cumulative Incidence Rate at 1 Year After Baseline

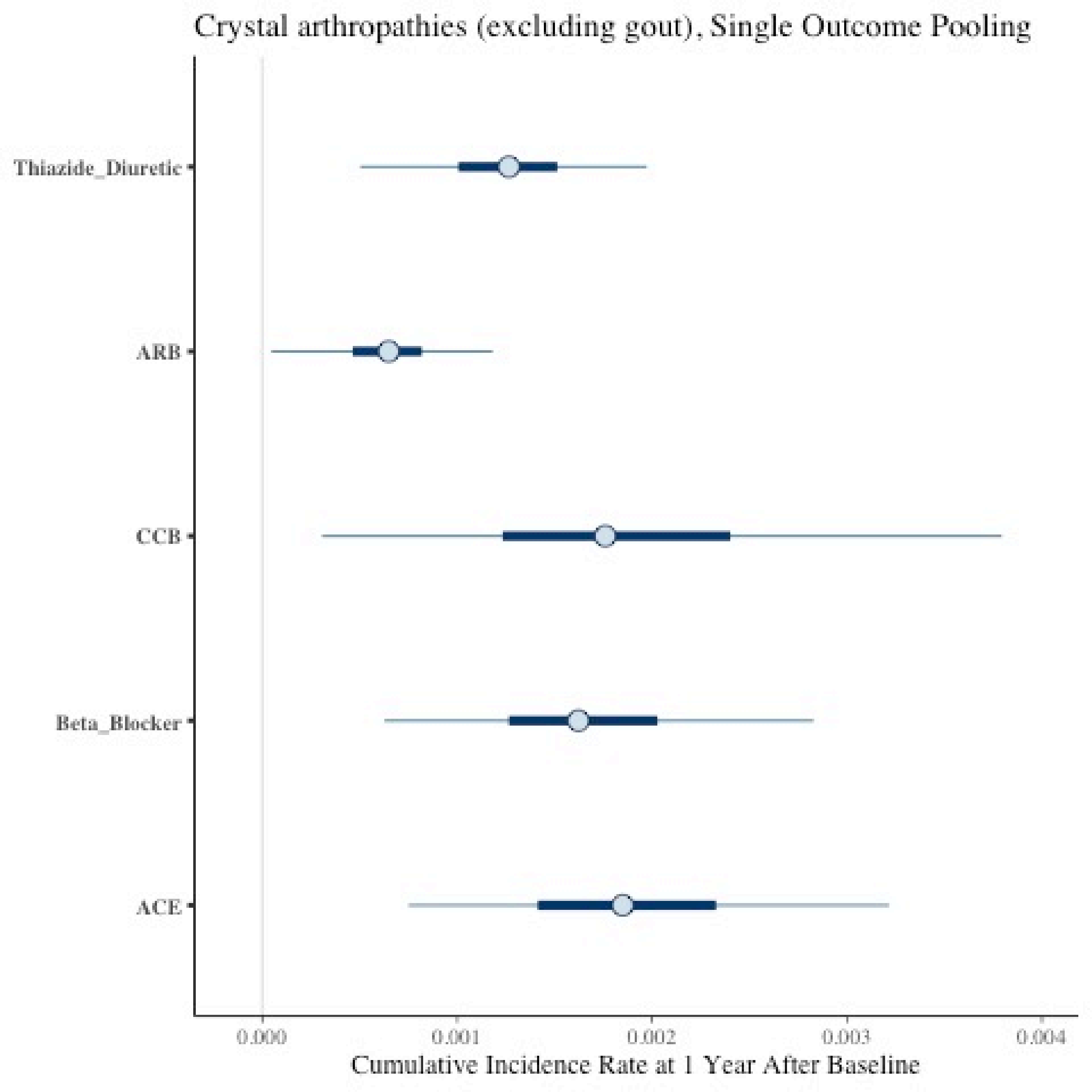
Crystal arthropathies (excluding gout), Single Outcome Pooling
Thiazide_Diuretic
ARB
CCB
Beta_Blocker
ACE
0.000
0.001
0.002
0.003
0.004
Cumulative Incidence Rate at 1 Year After Baseline

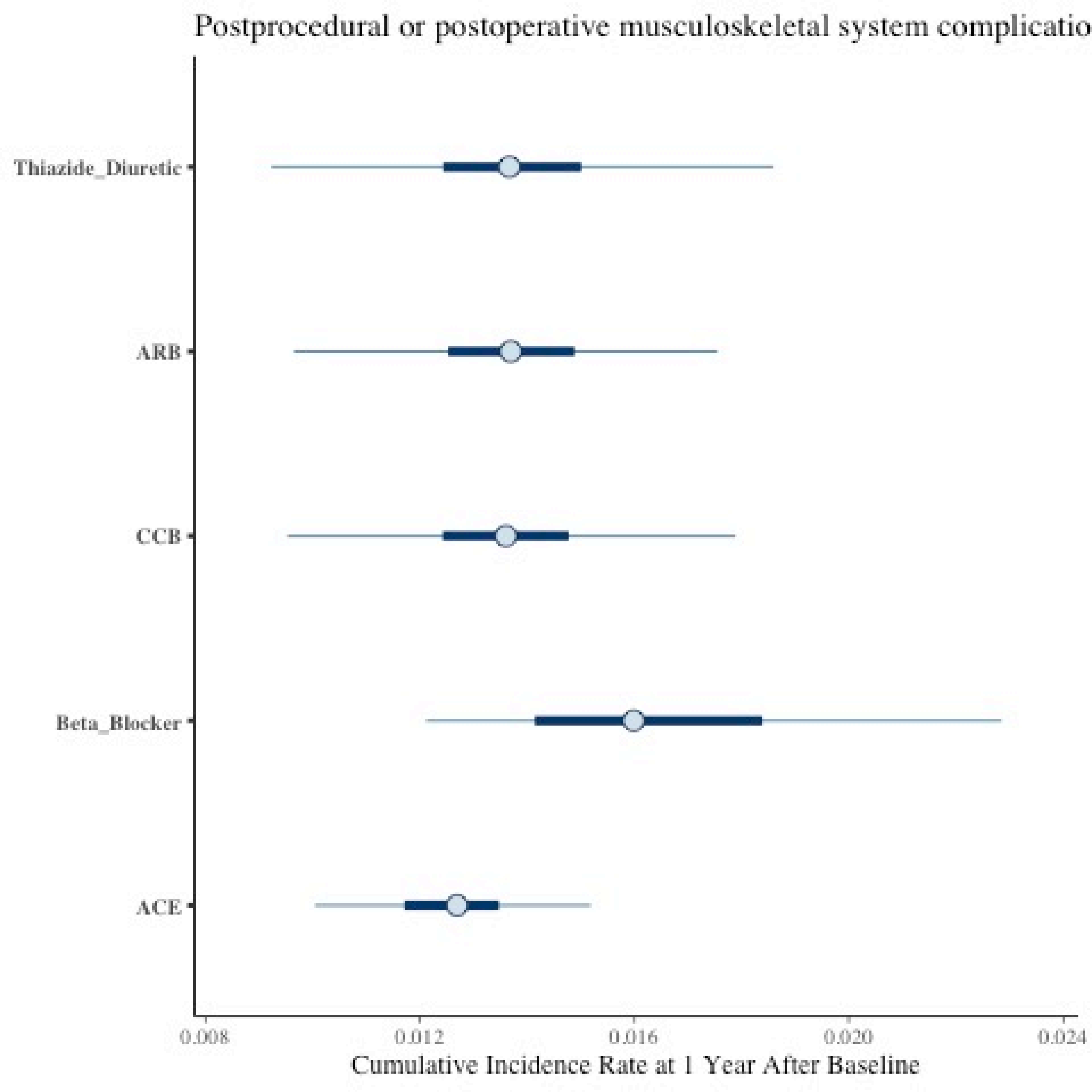

Postprocedural or postoperative musculoskeletal system complicatio
Thiazide_Diuretic
ARB
CCB
Beta_Blocker
ACE
0.008
0.012
0.016
0.020
0.024
Cumulative Incidence Rate at 1 Year After Baseline

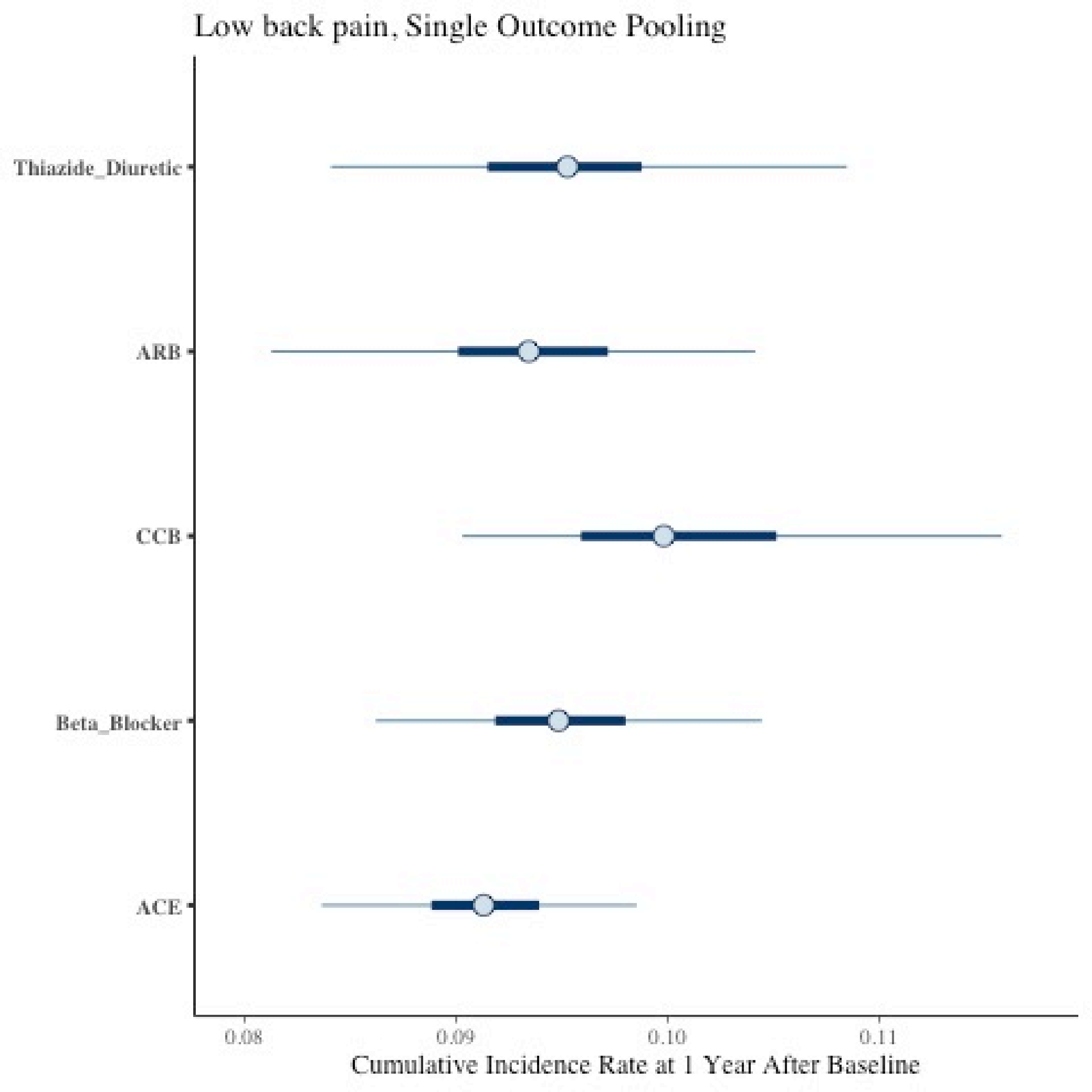

Low back pain, Single Outcome Pooling
Thiazide_Diuretic
ARB
CCB
Beta_Blocker
ACE
0.08
0.09
0.10
0.11
Cumulative Incidence Rate at 1 Year After Baseline

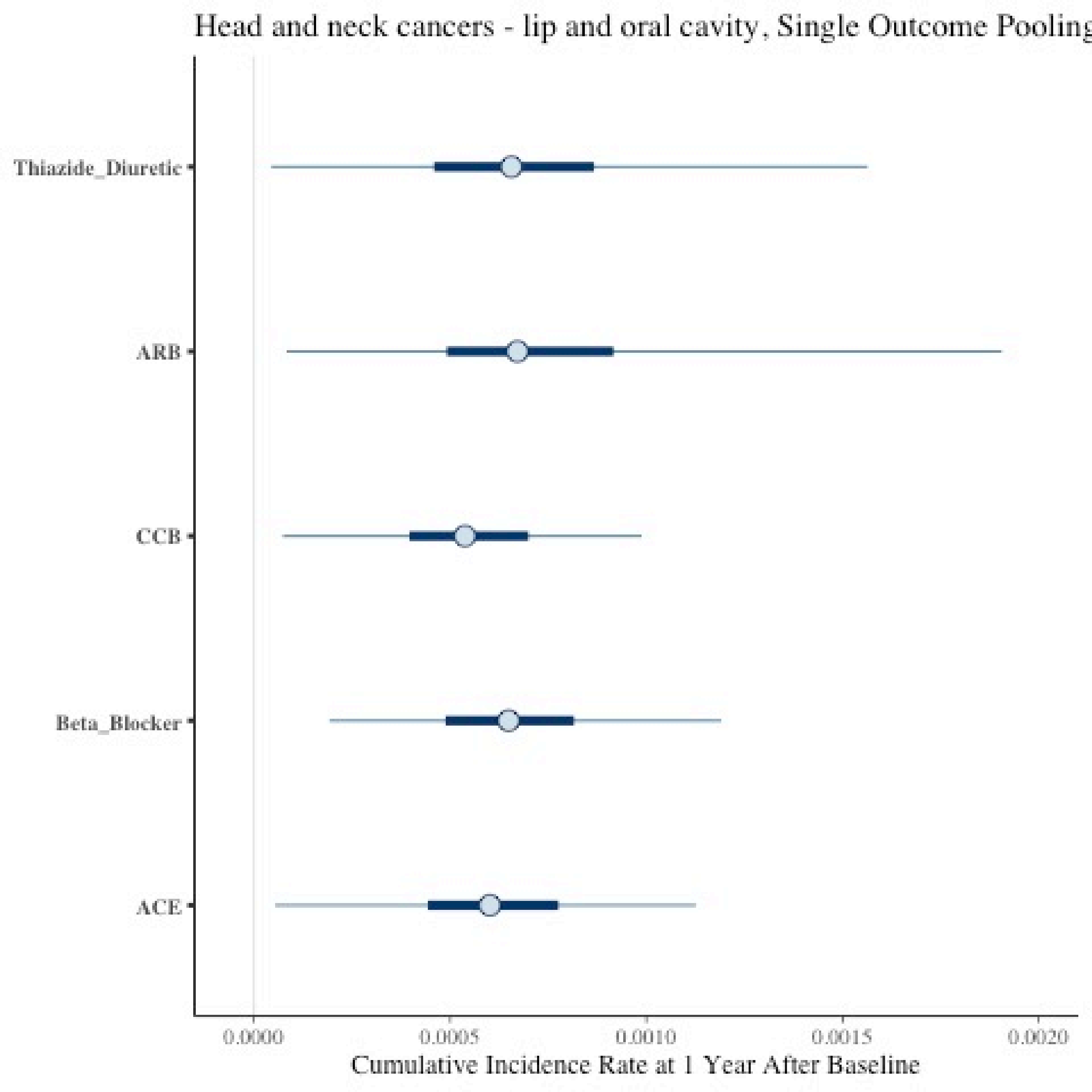

Head and neck cancers - lip and oral cavity, Single Outcome Pooling
Thiazide_Diuretic
ARB
CCB
Beta_Blocker
ACE
0.0000
0.0005
0.0010
0.0015
0.0020
Cumulative Incidence Rate at 1 Year After Baseline

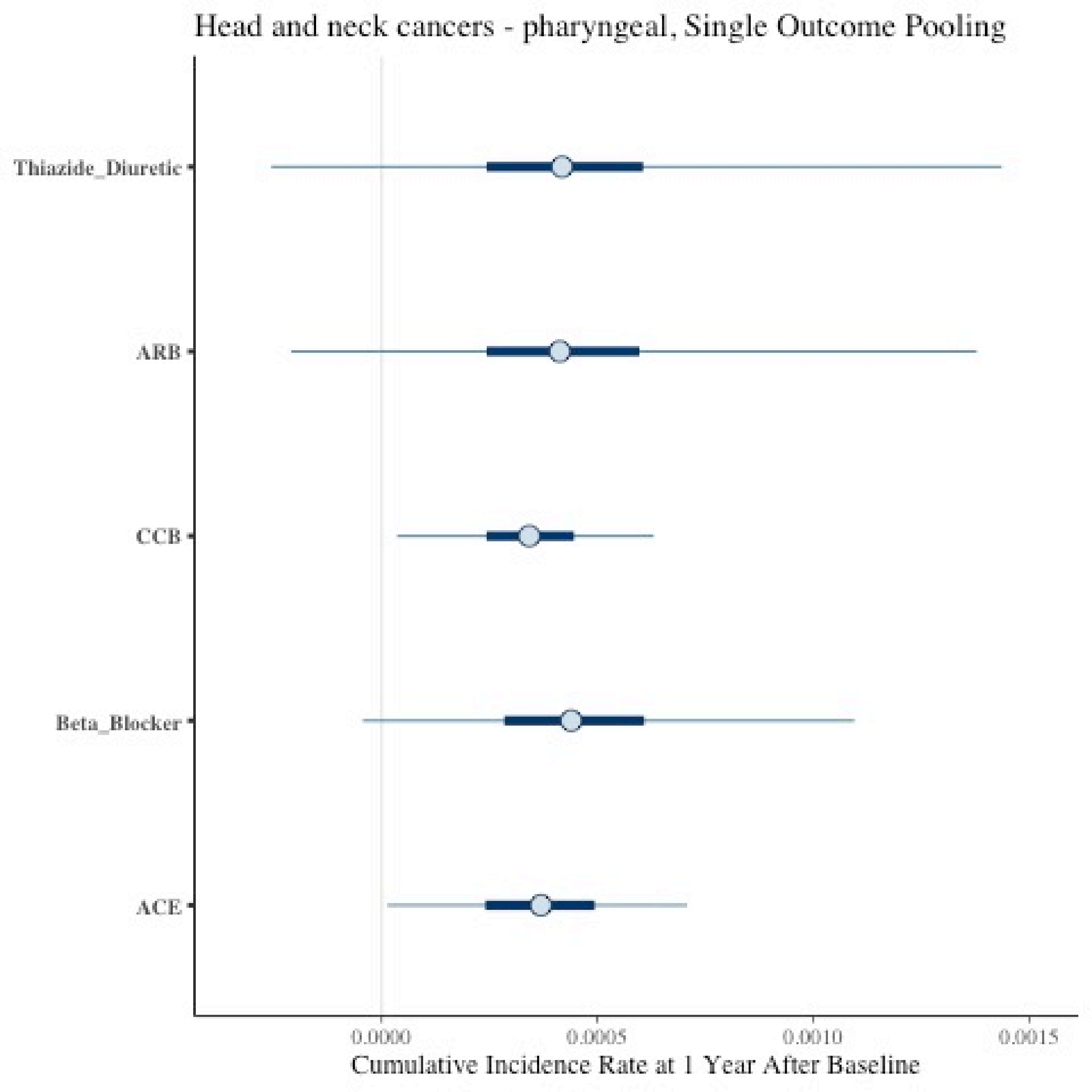

Head and neck cancers - pharyngeal, Single Outcome Pooling
Thiazide_Diuretic
ARB
CCB
Beta_Blocker
ACE
0.0000
0.0005
0.0010
0.0015
Cumulative Incidence Rate at 1 Year After Baseline

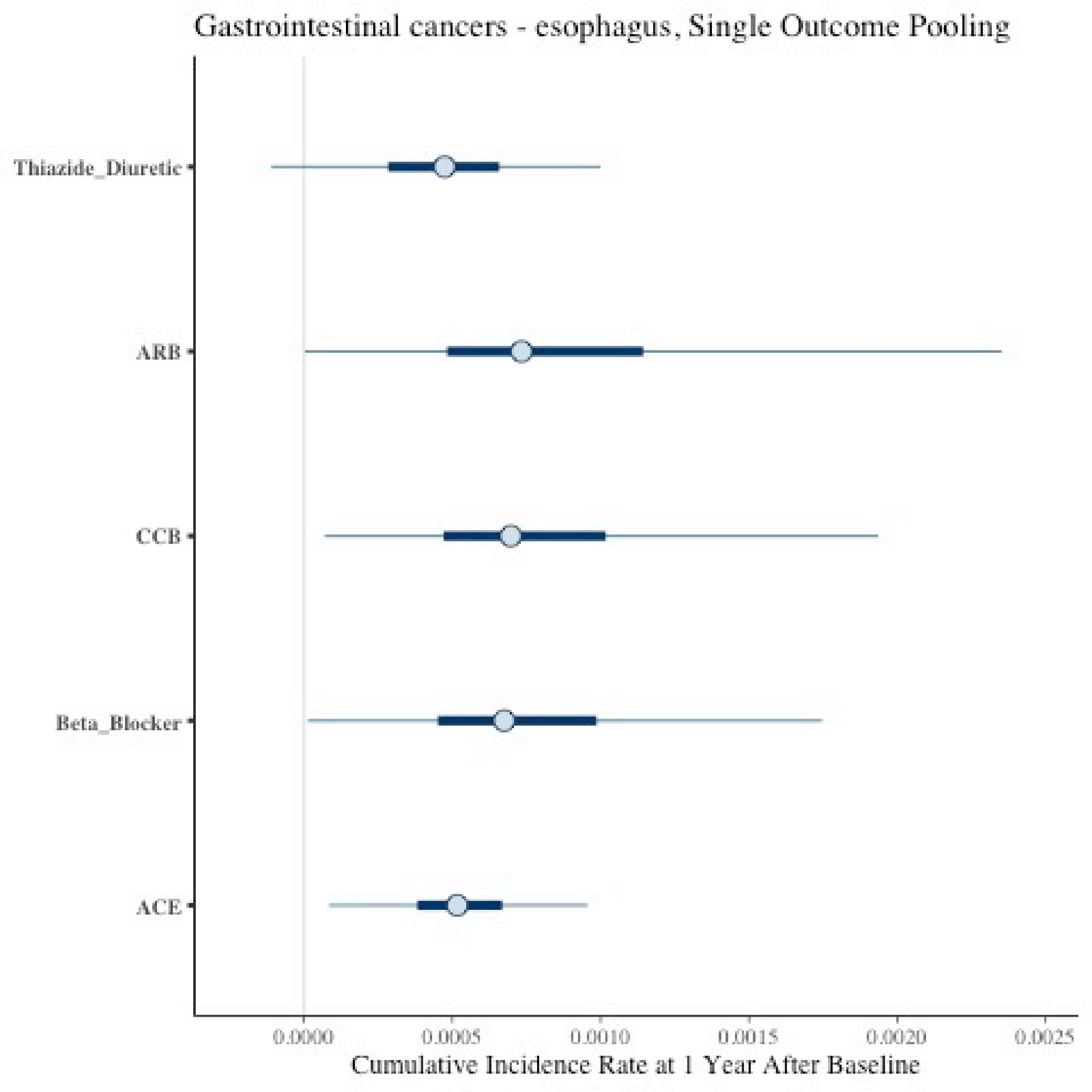

Gastrointestinal cancers - esophagus, Single Outcome Pooling
Thiazide_Diuretic
ARB
CCB
Beta_Blocker
ACE
0.0000
0.0005
0.0010
0.0015
0.0020
0.0025
Cumulative Incidence Rate at 1 Year After Baseline

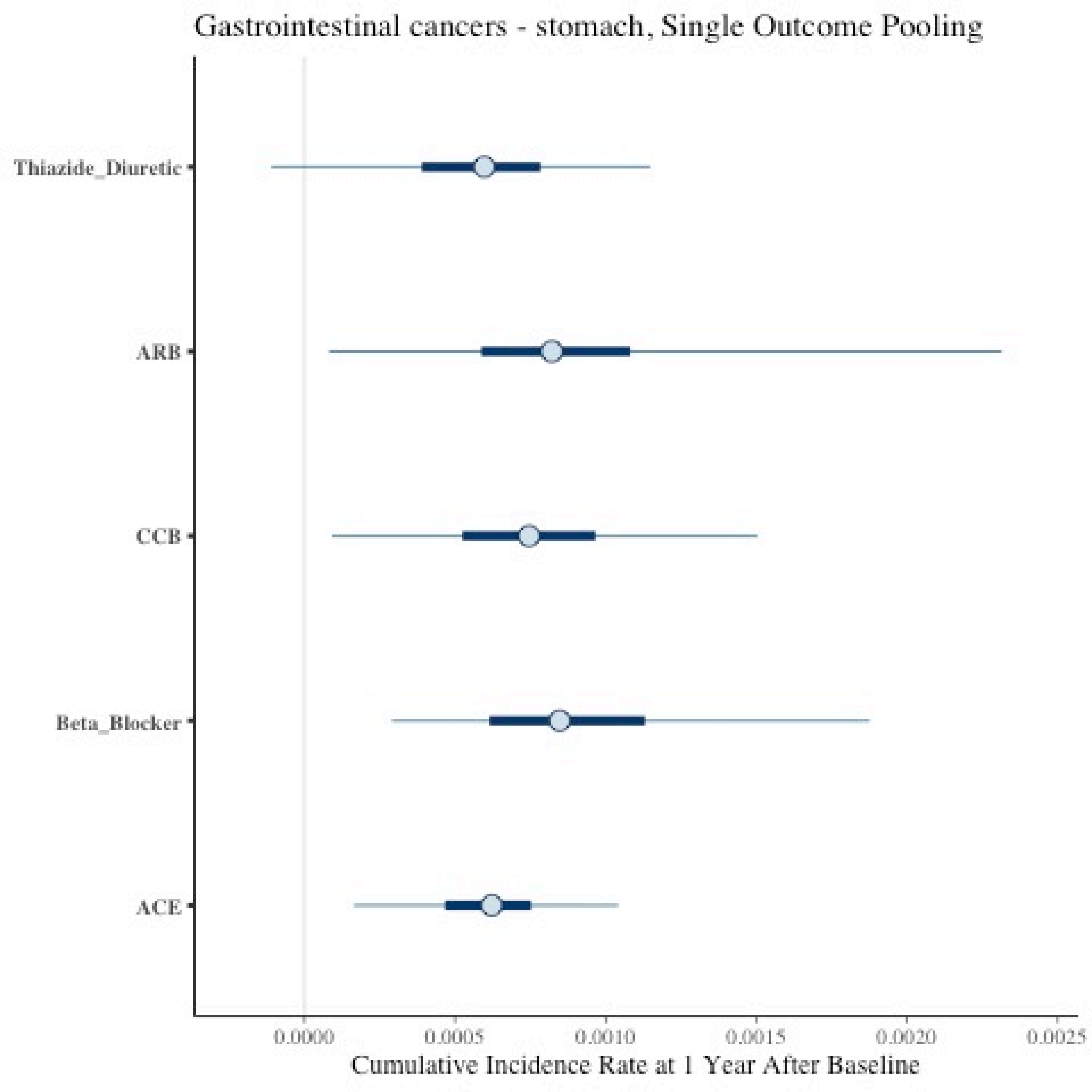

Gastrointestinal cancers - stomach, Single Outcome Pooling
Thiazide_Diuretic
ARB
CCB
Beta_Blocker
ACE
0.0000
0.0005
0.0010
0.0015
0.0020
0.0025
Cumulative Incidence Rate at 1 Year After Baseline

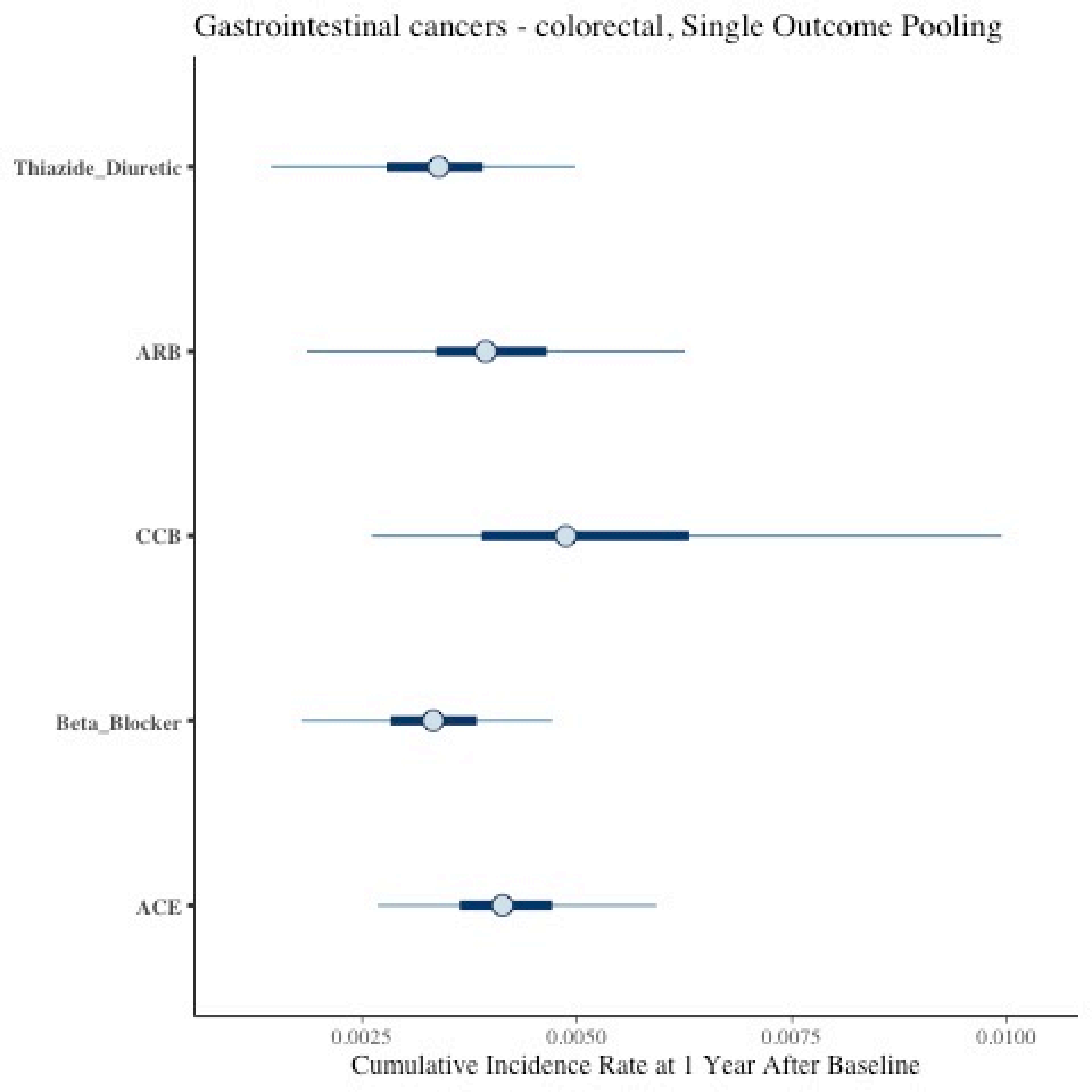

Gastrointestinal cancers - colorectal, Single Outcome Pooling
Thiazide_Diuretic
ARB
CCB
Beta_Blocker
ACE
0.0025
0.0050
0.0075
0.0100
Cumulative Incidence Rate at 1 Year After Baseline

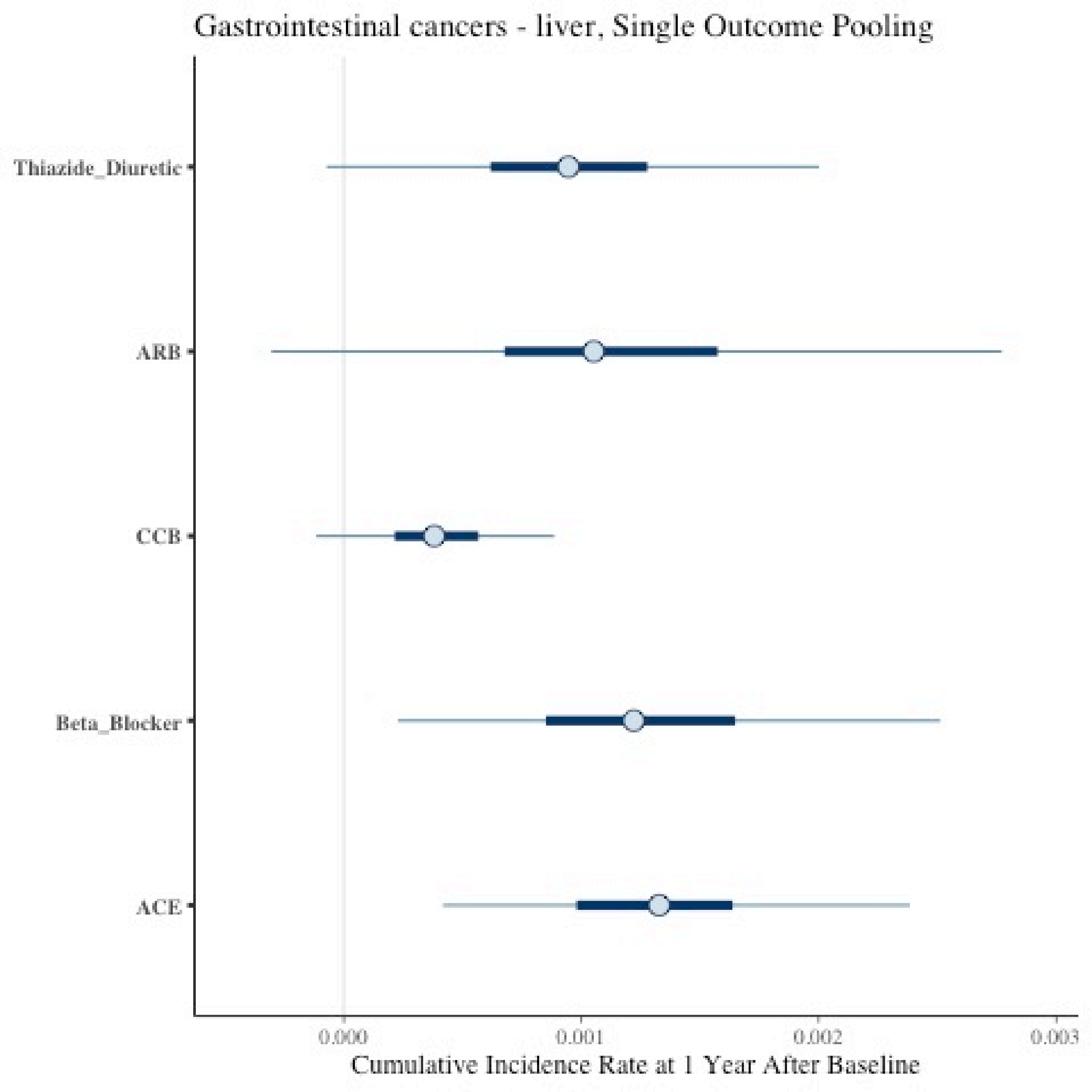
Gastrointestinal cancers - liver, Single Outcome Pooling
Thiazide_Diuretic
ARB
CCB
Beta_Blocker
ACE
0.000
0.001
0.002
0.003
Cumulative Incidence Rate at 1 Year After Baseline

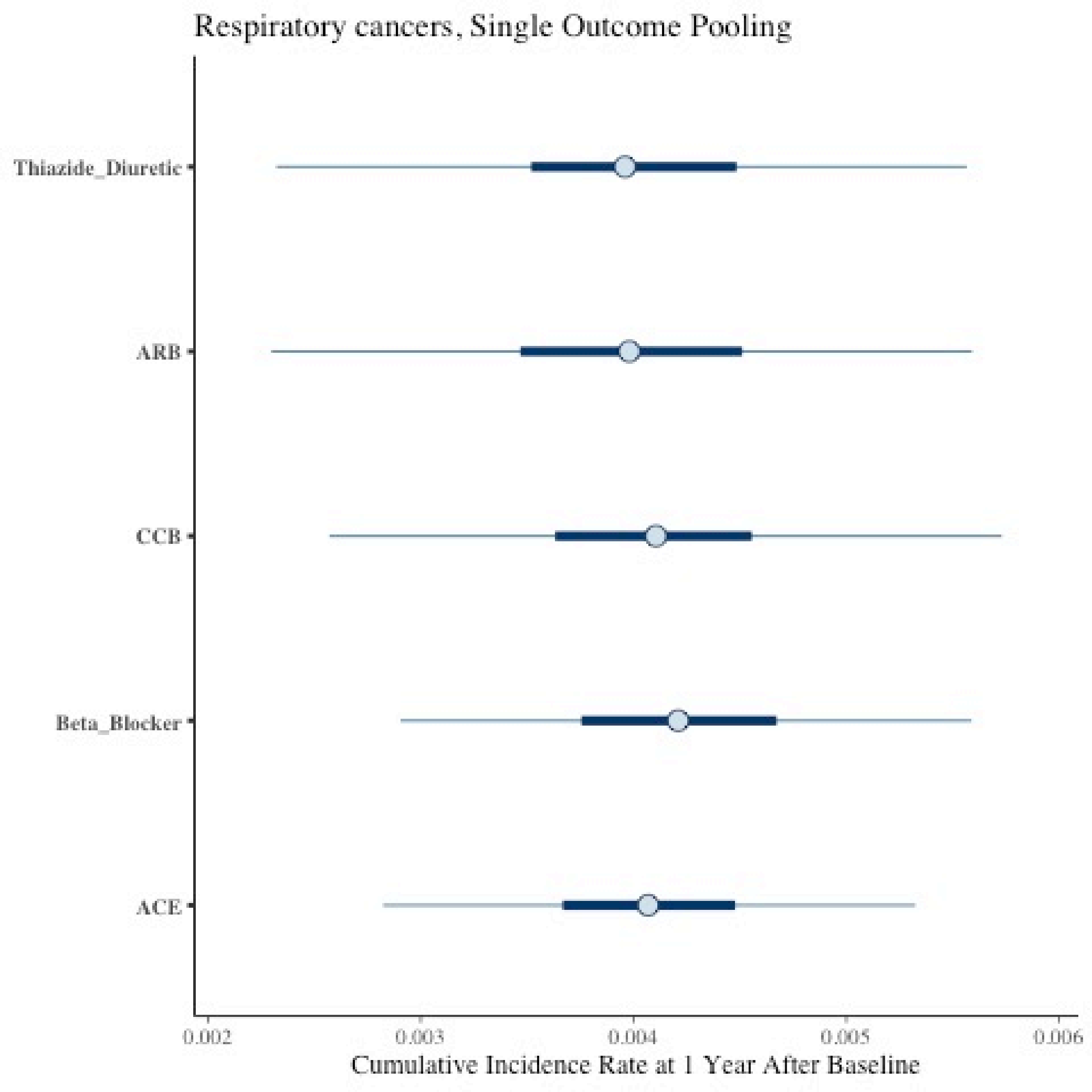

Respiratory cancers, Single Outcome Pooling
Thiazide_Diuretic
ARB
CCB
Beta_Blocker
ACE
0.002
0.003
0.004
0.005
0.006
Cumulative Incidence Rate at 1 Year After Baseline

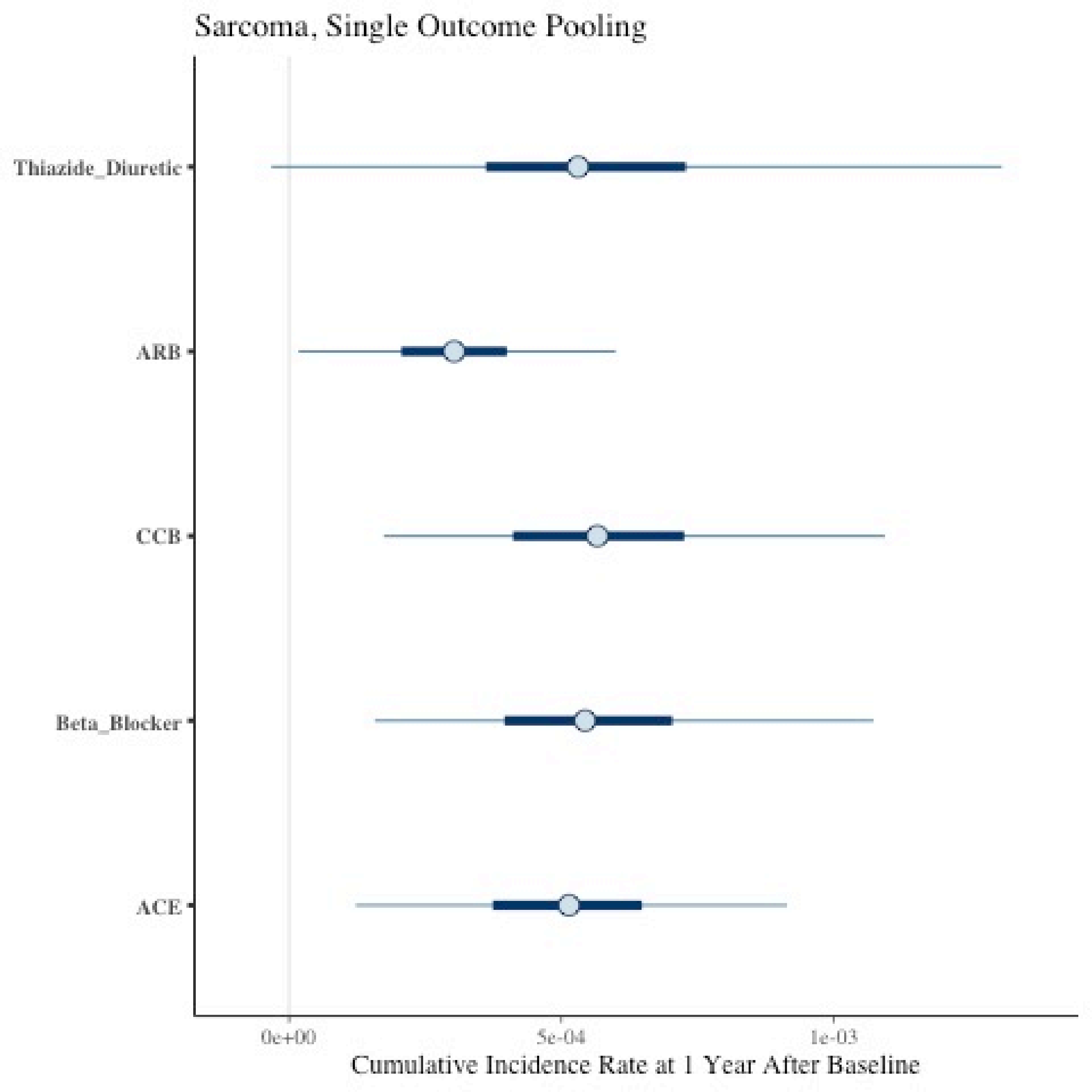

Sarcoma, Single Outcome Pooling
Thiazide_Diuretic
ARB
CCB
Beta_Blocker
ACE
0e+00
5e-04
1e-03
Cumulative Incidence Rate at 1 Year After Baseline

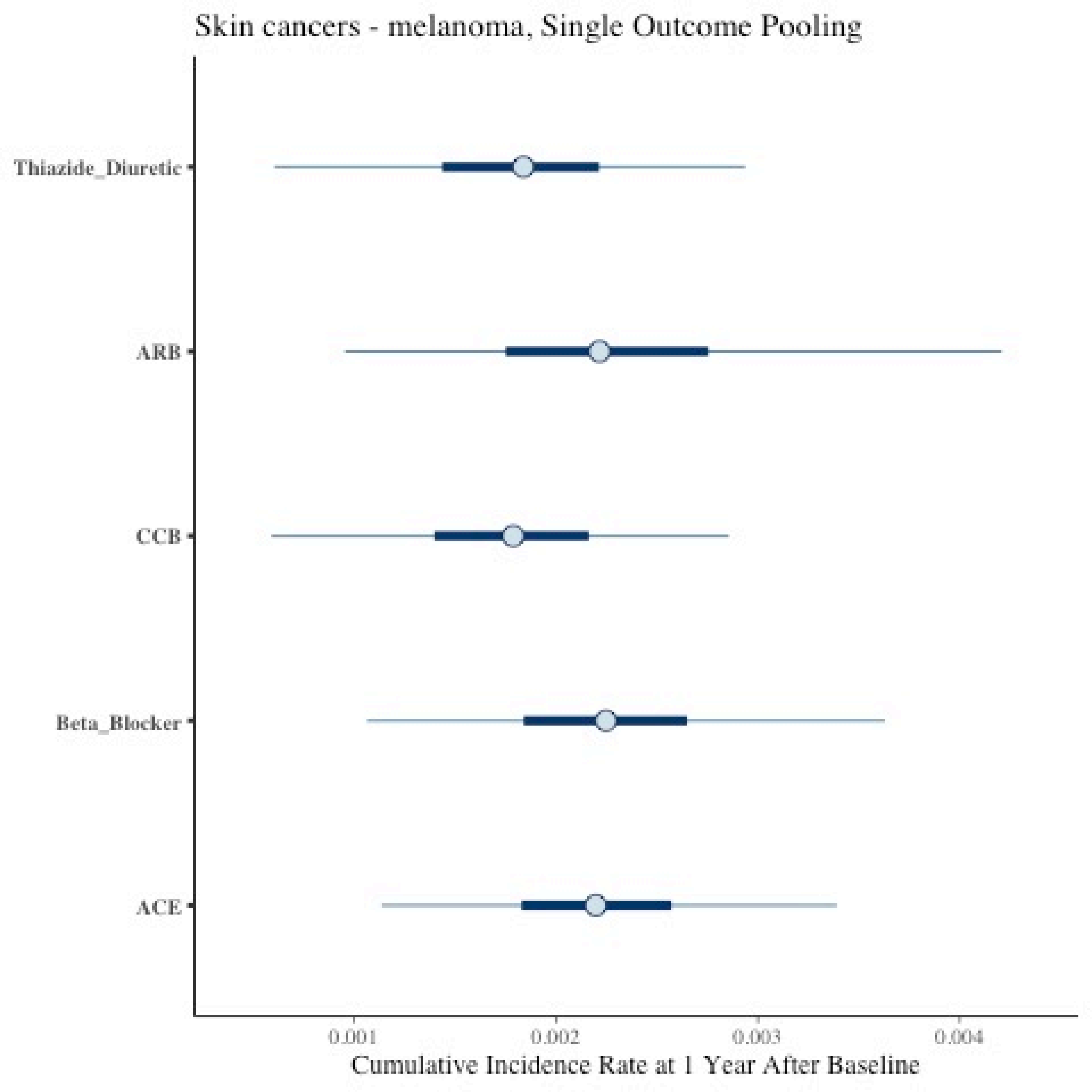
Skin cancers - melanoma, Single Outcome Pooling
Thiazide_Diuretic
ARB
CCB
Beta_Blocker
ACE
0.001
0.002
0.003
0.004
Cumulative Incidence Rate at 1 Year After Baseline

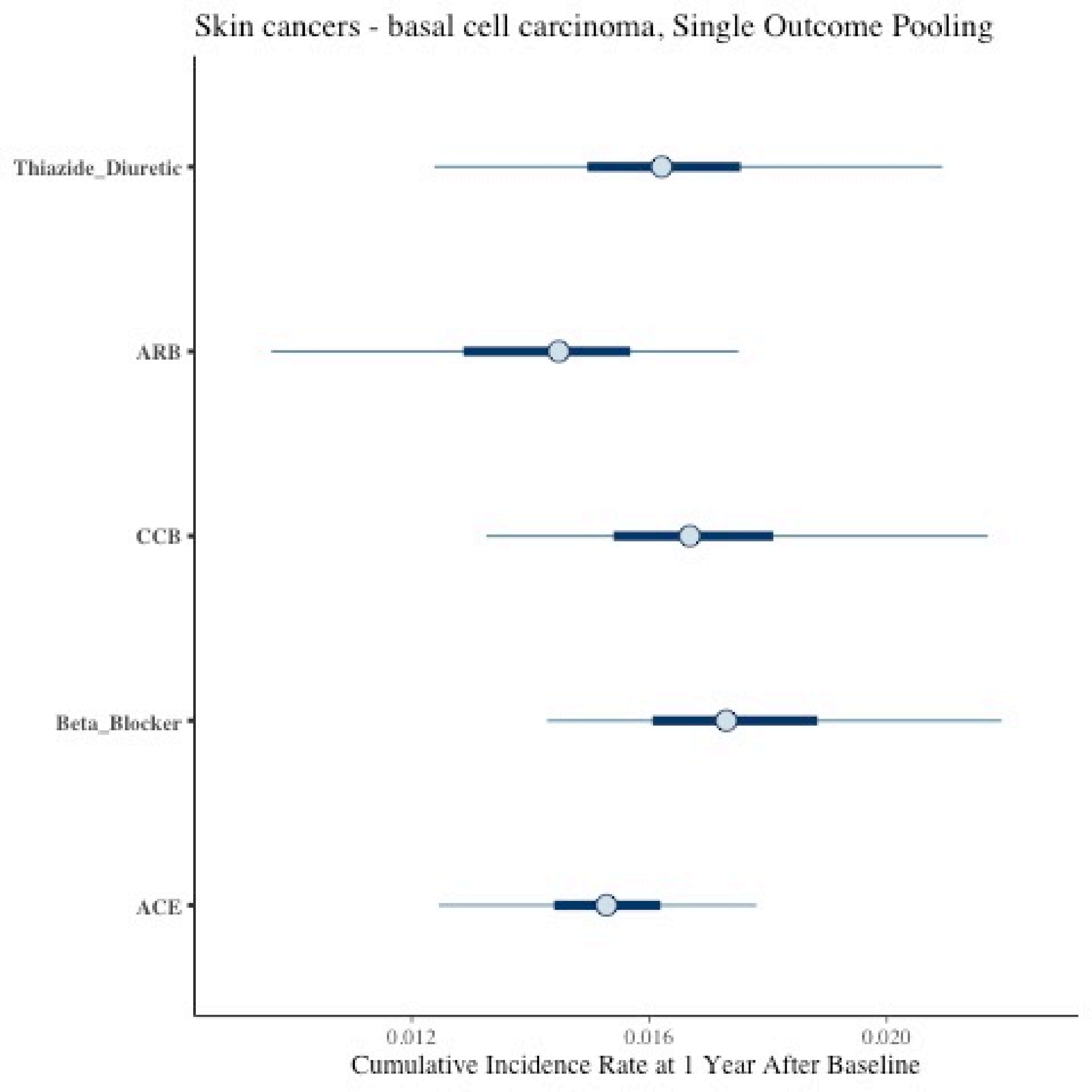

Skin cancers - basal cell carcinoma, Single Outcome Pooling
Thiazide_Diuretic
ARB
CCB
Beta_Blocker
ACE
0.012
0.016
0.020
Cumulative Incidence Rate at 1 Year After Baseline

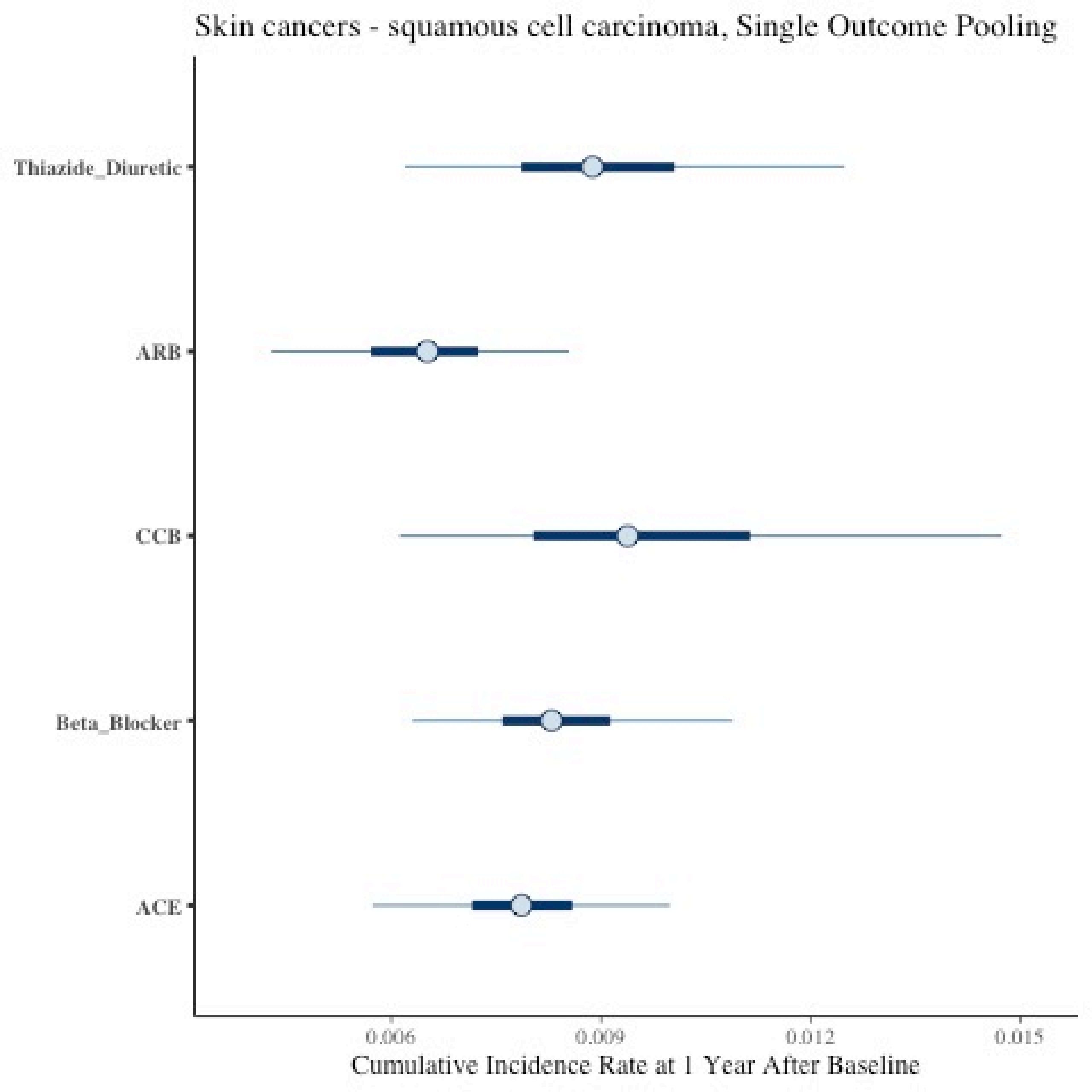

Skin cancers - squamous cell carcinoma, Single Outcome Pooling
Thiazide_Diuretic
ARB
CCB
Beta_Blocker
ACE
0.006
0.009
0.012
0.015
Cumulative Incidence Rate at 1 Year After Baseline

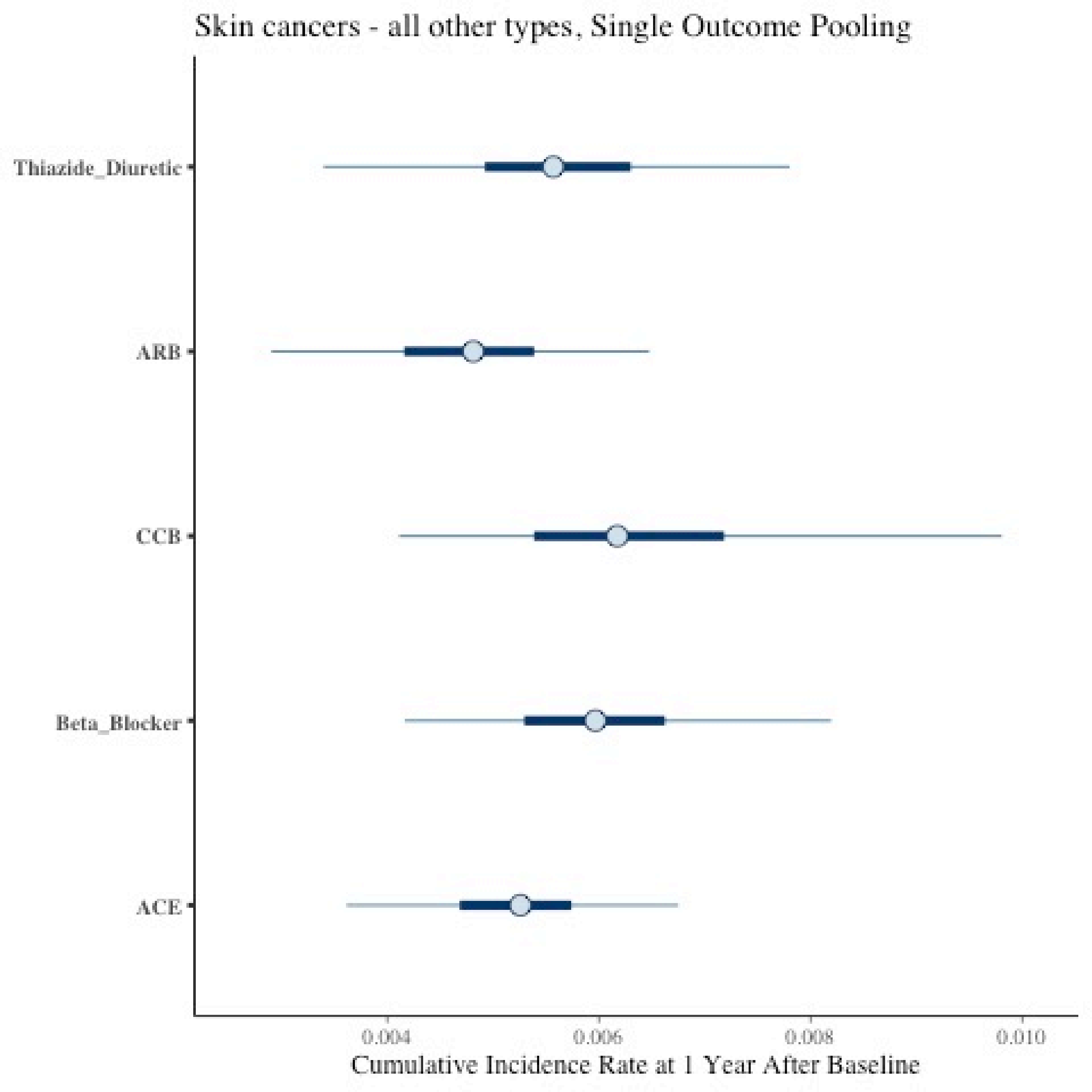

Skin cancers - all other types, Single Outcome Pooling
Thiazide_Diuretic
ARB
CCB
Beta_Blocker
ACE
0.004
0.006
0.008
0.010
Cumulative Incidence Rate at 1 Year After Baseline

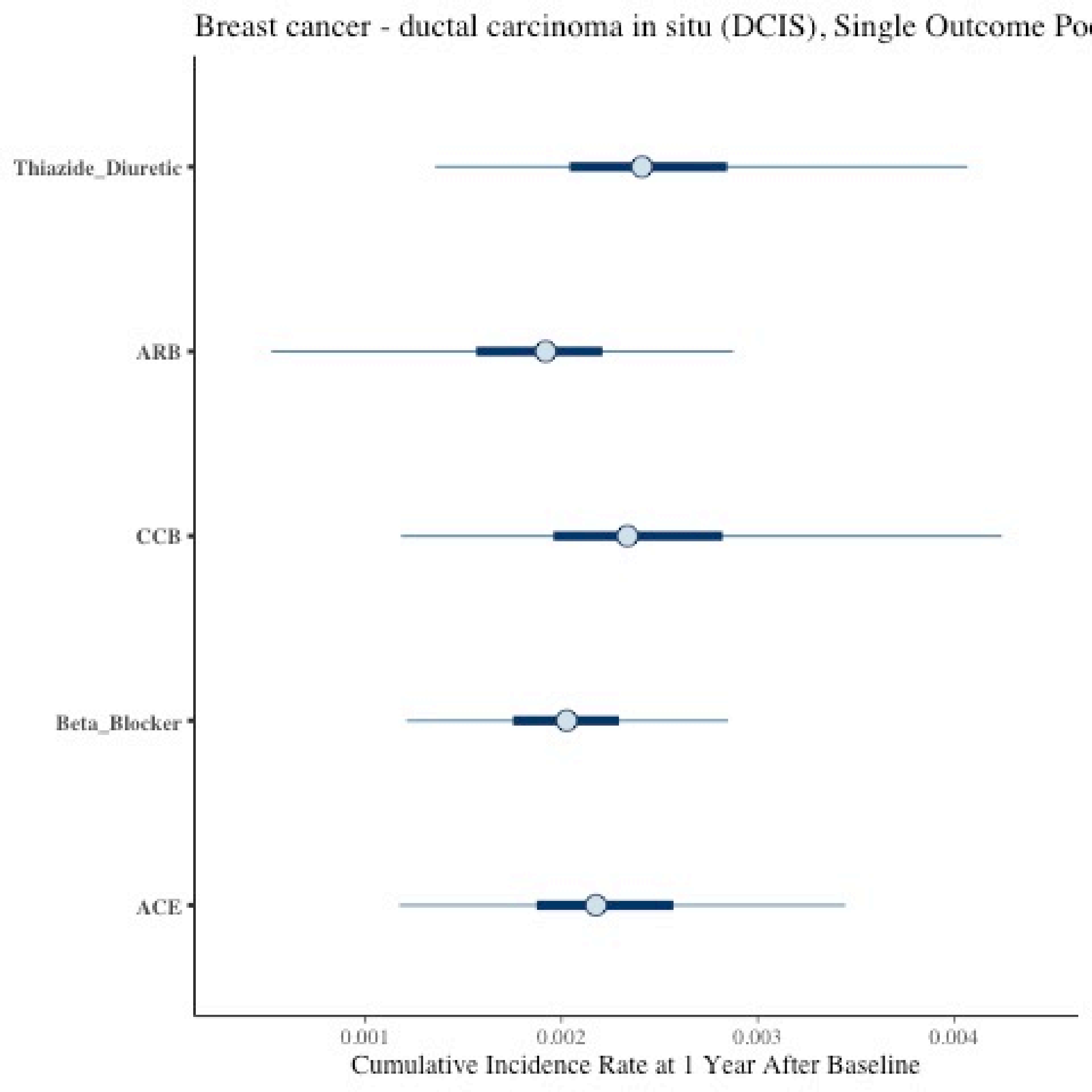

Breast cancer - ductal carcinoma in situ (DCIS), Single Outcome Po
Thiazide_Diuretic
ARB
CCB
Beta_Blocker
ACE
0.001
0.002
0.003
0.004
Cumulative Incidence Rate at 1 Year After Baseline

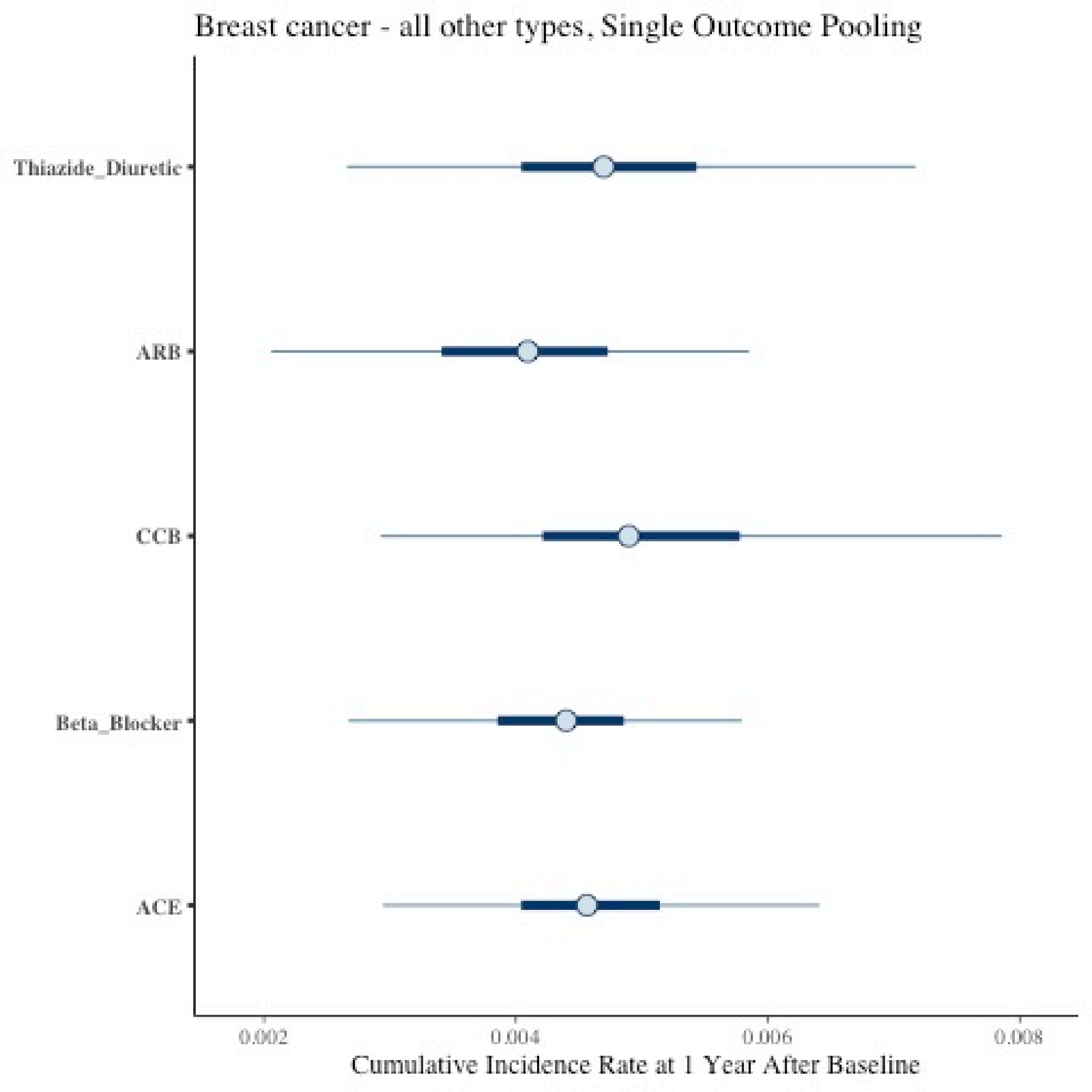

Breast cancer - all other types, Single Outcome Pooling
Thiazide_Diuretic
ARB
CCB
Beta_Blocker
ACE
0.002
0.004
0.006
0.008
Cumulative Incidence Rate at 1 Year After Baseline

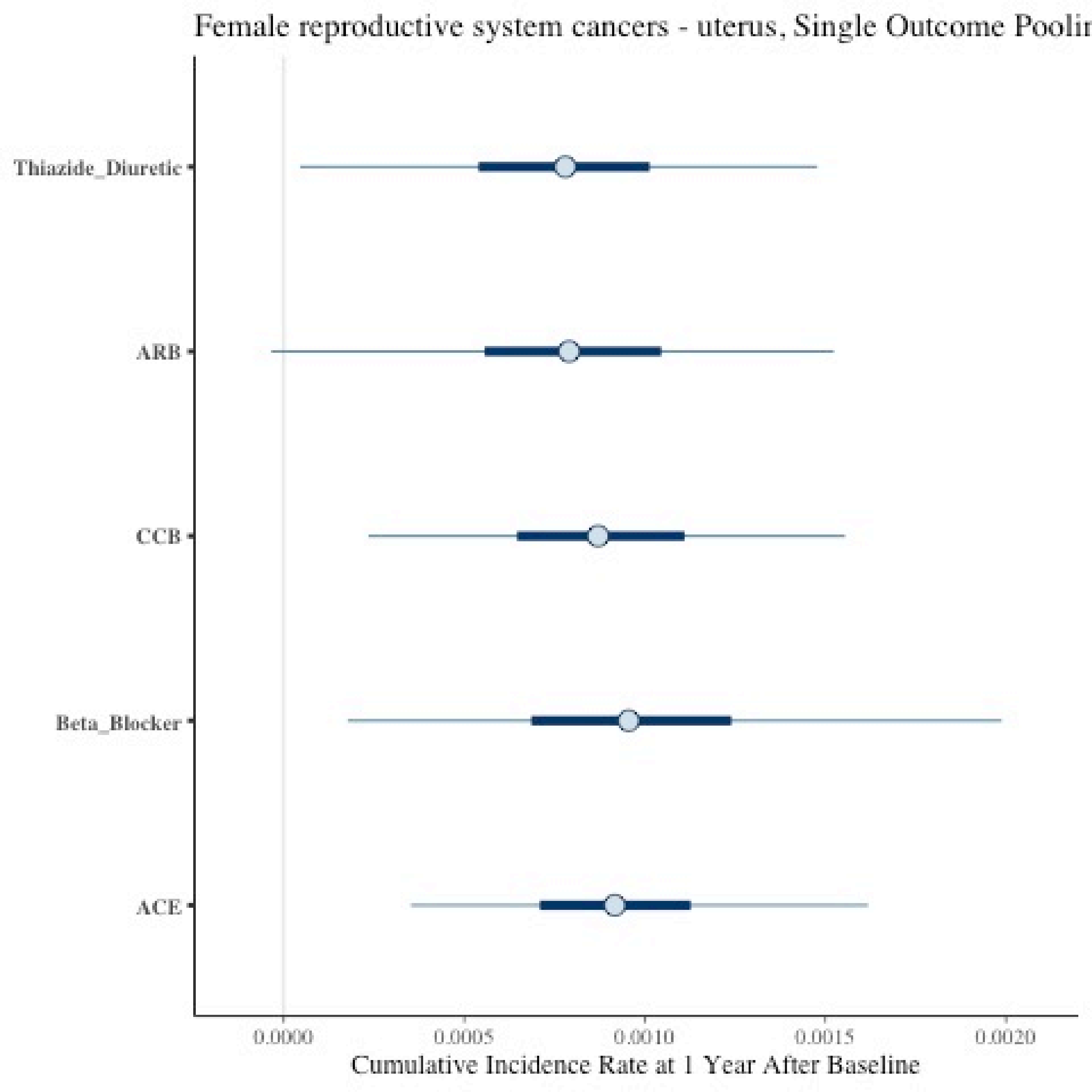

Female reproductive system cancers - uterus, Single Outcome Poolin
Thiazide_Diuretic
ARB
CCB
Beta_Blocker
ACE
0.0000
0.0005
0.0010
0.0015
0.0020
Cumulative Incidence Rate at 1 Year After Baseline

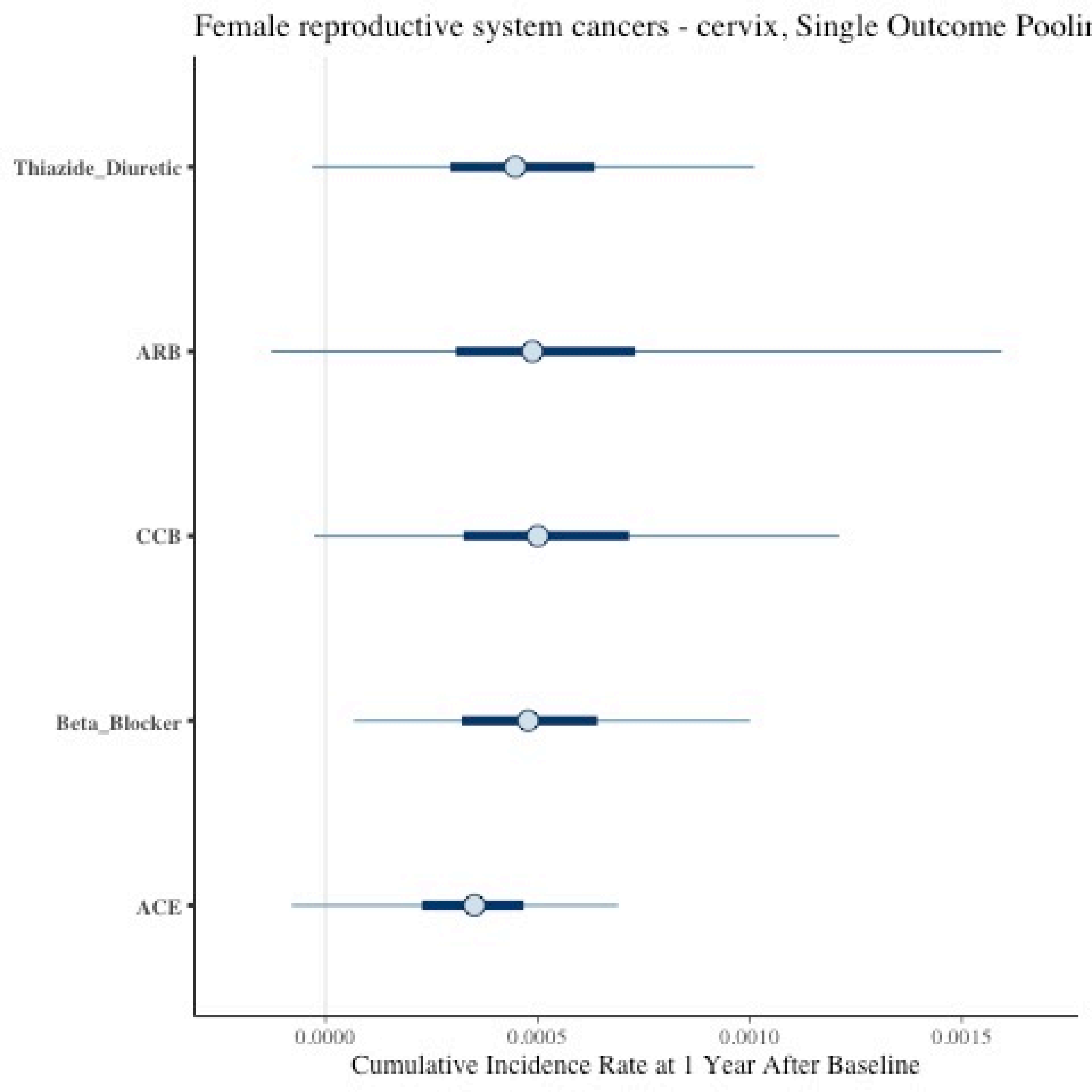
Female reproductive system cancers - cervix, Single Outcome Poolin
Thiazide_Diuretic
ARB
CCB
Beta_Blocker
ACE
0.0000
0.0005
0.0010
0.0015
Cumulative Incidence Rate at 1 Year After Baseline

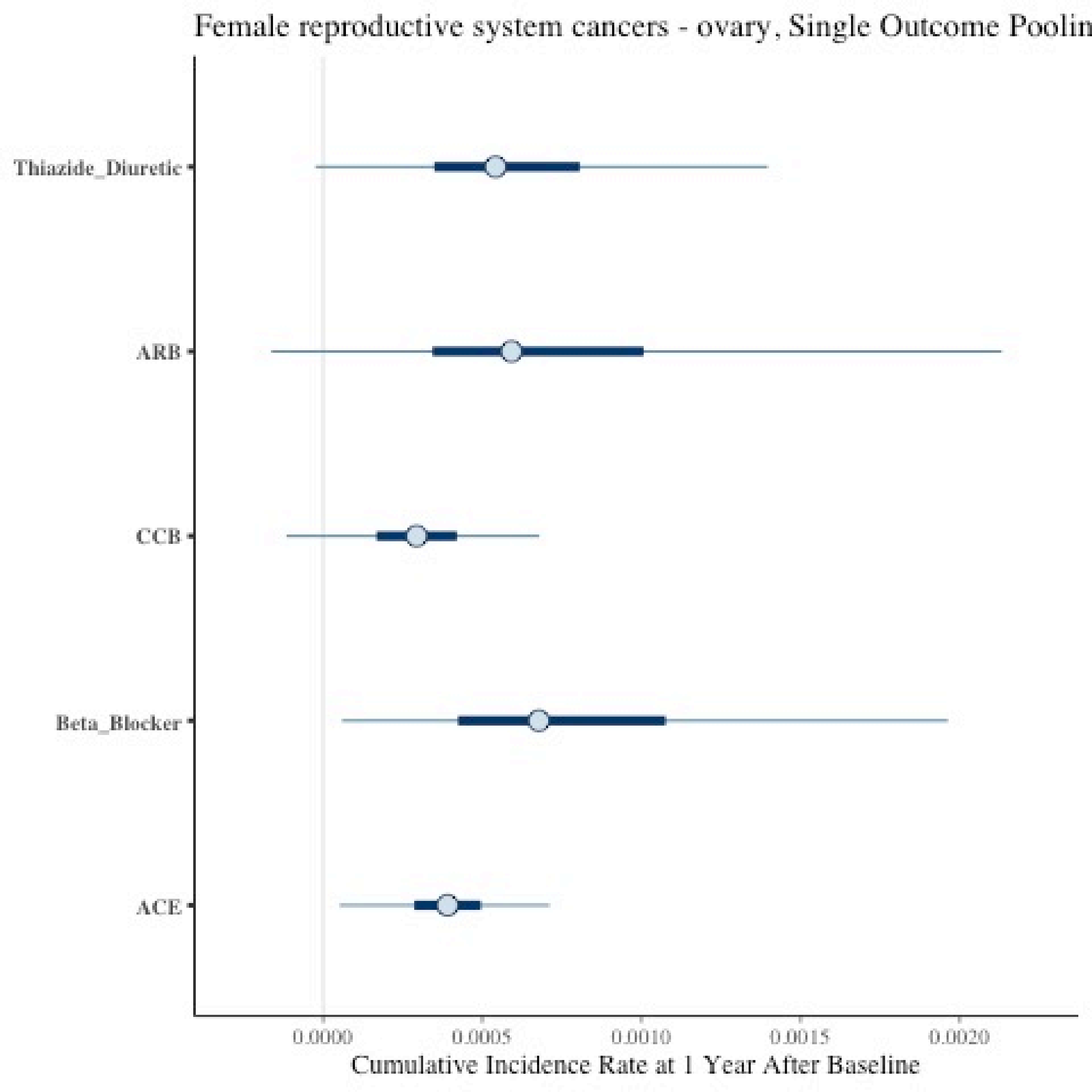
Female reproductive system cancers - ovary, Single Outcome Poolin
Thiazide_Diuretic
ARB
CCB
Beta_Blocker
ACE
0.0000
0.0005
0.0010
0.0015
0.0020
Cumulative Incidence Rate at 1 Year After Baseline

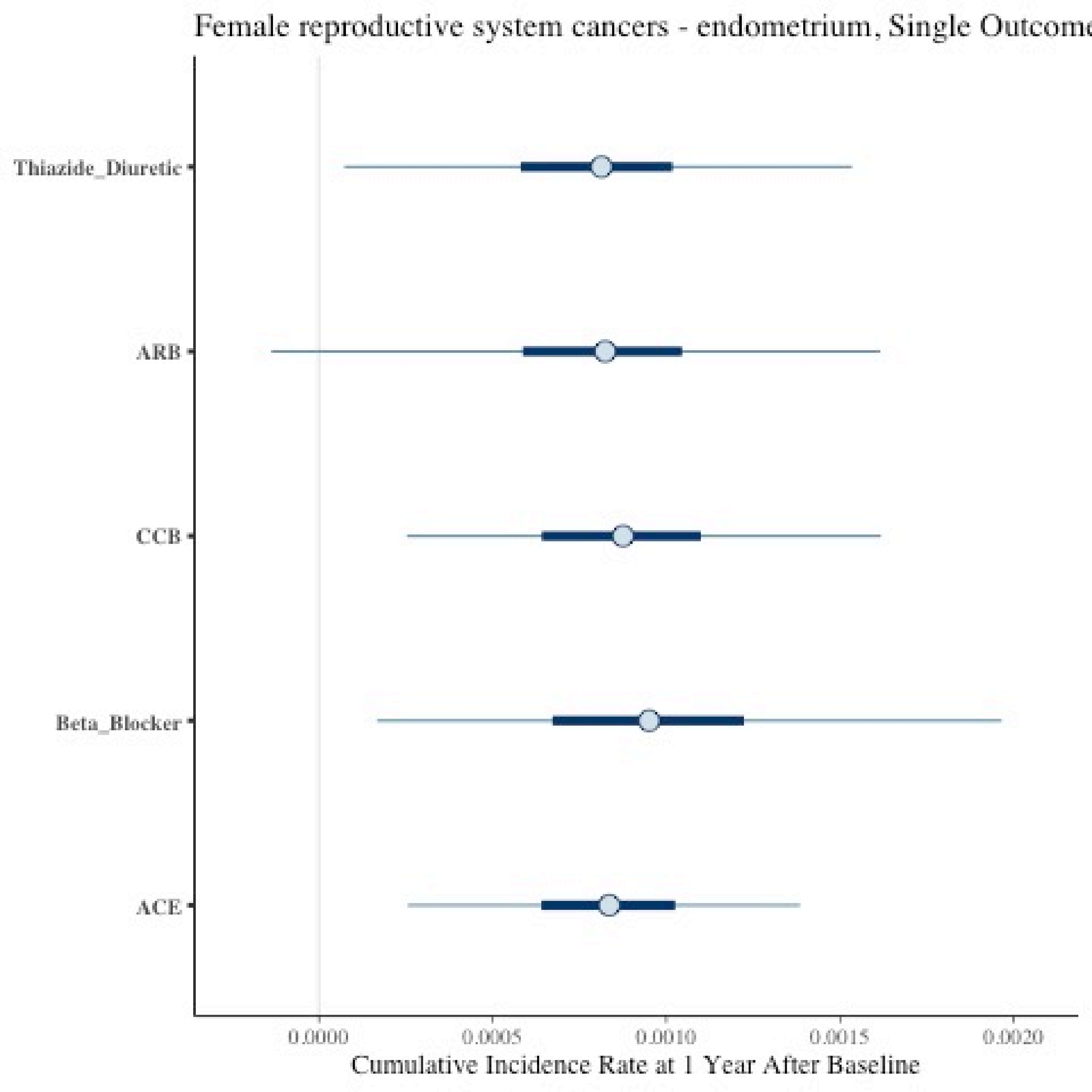
Female reproductive system cancers - endometrium, Single Outcome
Thiazide_Diuretic
ARB
CCB
Beta_Blocker
ACE
0.0000
0.0005
0.0010
0.0015
0.0020
Cumulative Incidence Rate at 1 Year After Baseline

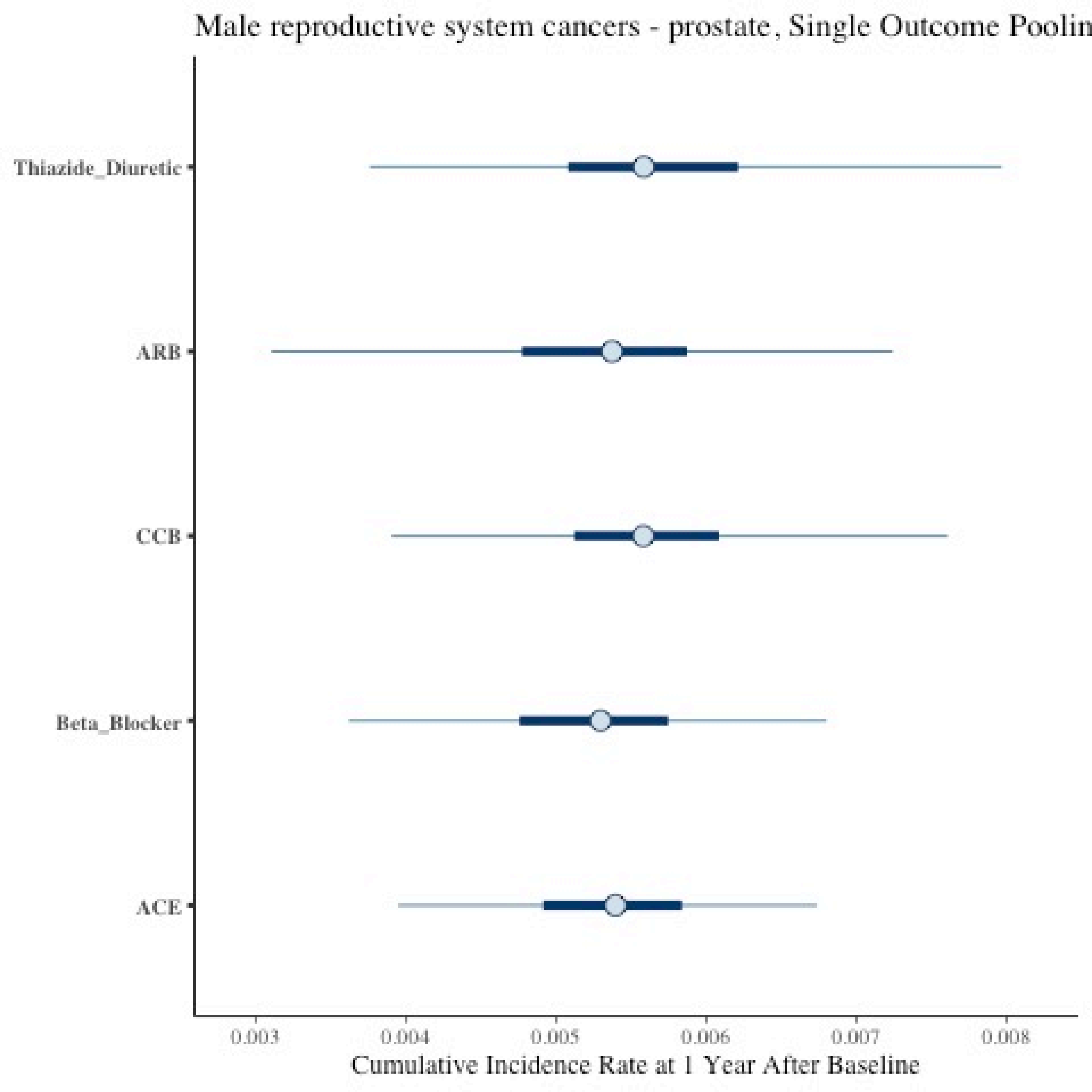
Male reproductive system cancers - prostate, Single Outcome Poolin
Thiazide_Diuretic
ARB
CCB
Beta_Blocker
ACE
0.003
0.004
0.005
0.006
0.007
0.008
Cumulative Incidence Rate at 1 Year After Baseline

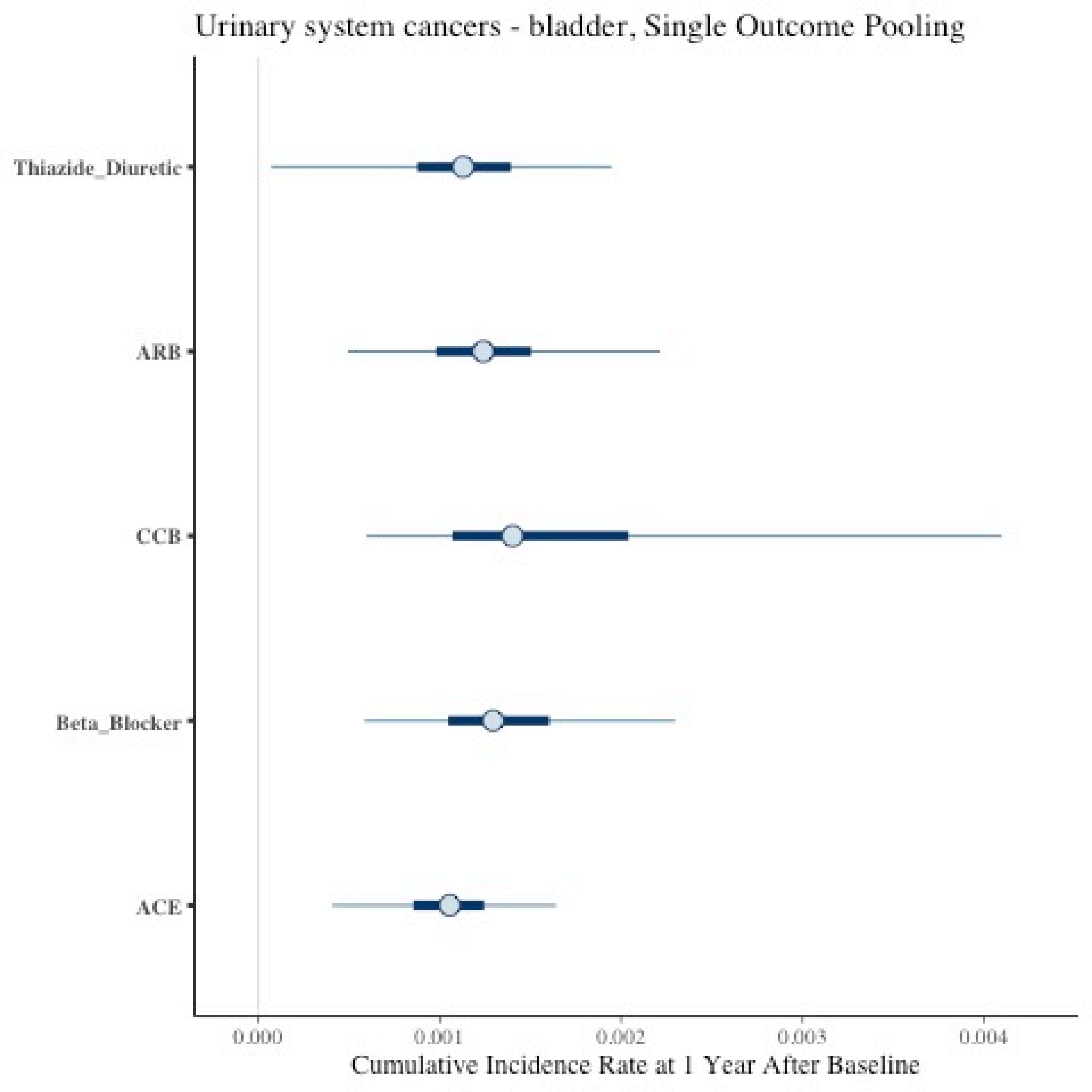
Urinary system cancers - bladder, Single Outcome Pooling
Thiazide_Diuretic
ARB
CCB
Beta_Blocker
ACE
0.000
0.001
0.002
0.003
0.004
Cumulative Incidence Rate at 1 Year After Baseline

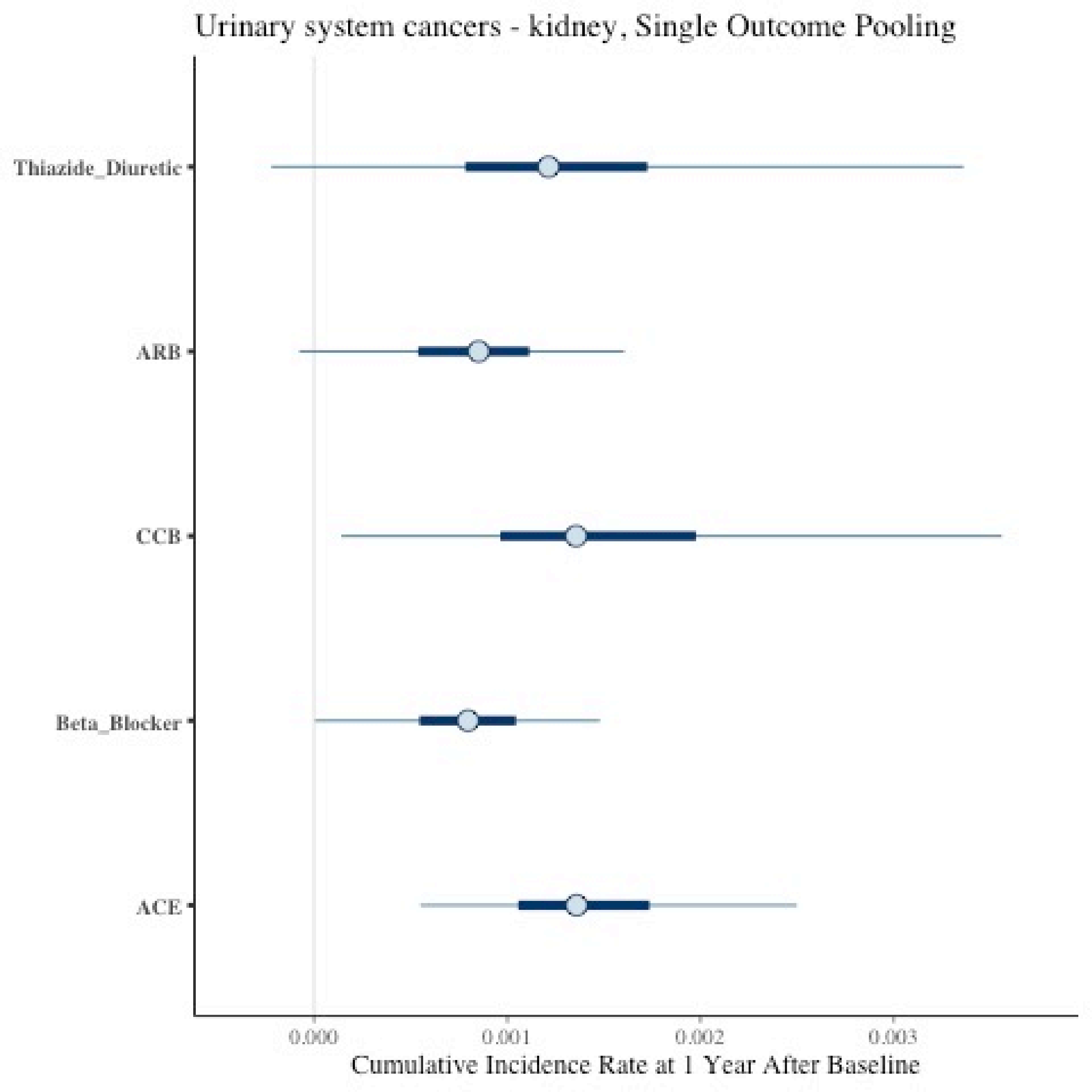

Urinary system cancers - kidney, Single Outcome Pooling
Thiazide_Diuretic
ARB
CCB
Beta_Blocker
ACE
0.000
0.001
0.002
0.003
Cumulative Incidence Rate at 1 Year After Baseline

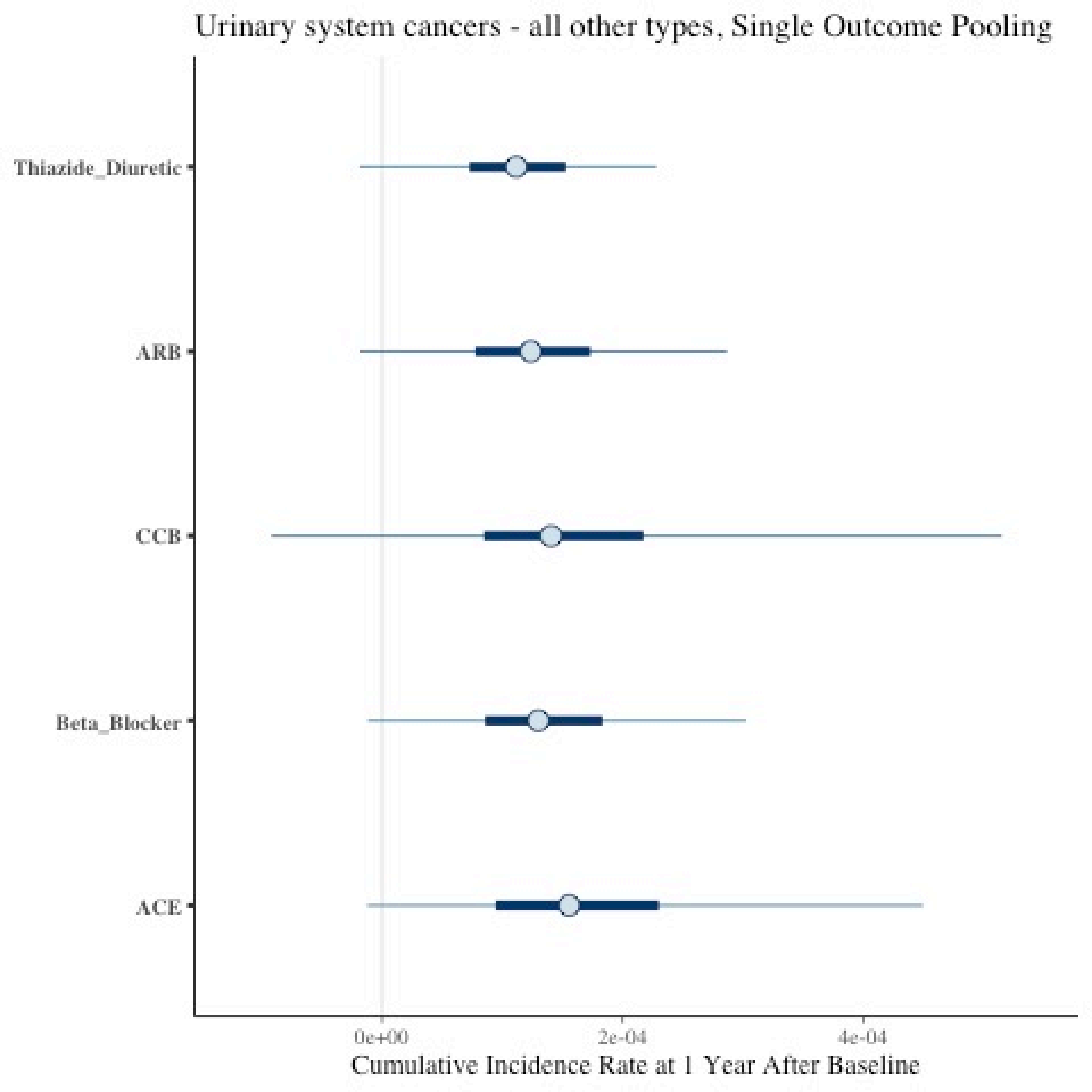
Urinary system cancers - all other types, Single Outcome Pooling
Thiazide_Diuretic
ARB
CCB
Beta_Blocker
ACE
0e+00
2e-04
4e-04
Cumulative Incidence Rate at 1 Year After Baseline

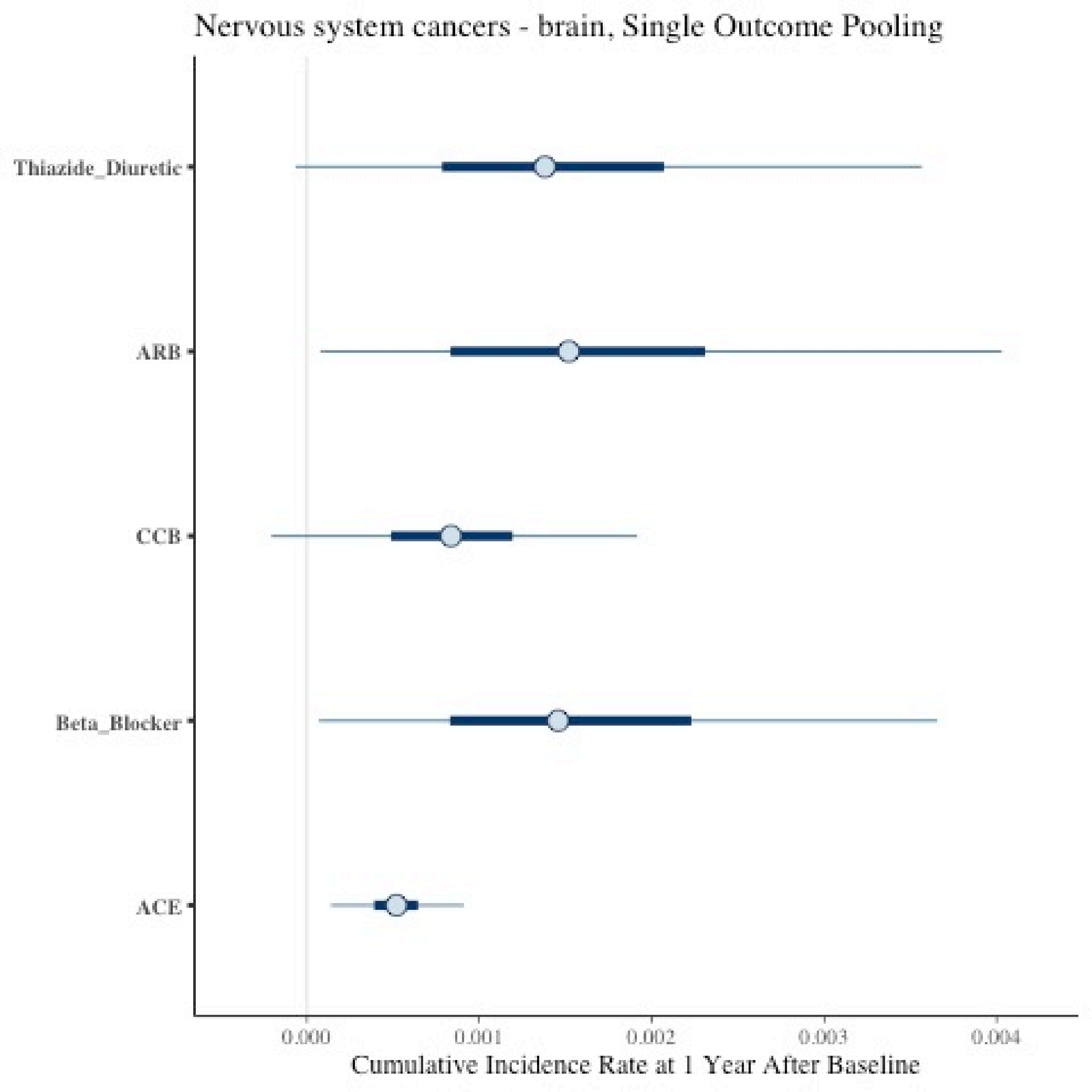
Nervous system cancers - brain, Single Outcome Pooling
Thiazide_Diuretic
ARB
CCB
Beta_Blocker
ACE
0.000
0.001
0.002
0.003
0.004
Cumulative Incidence Rate at 1 Year After Baseline

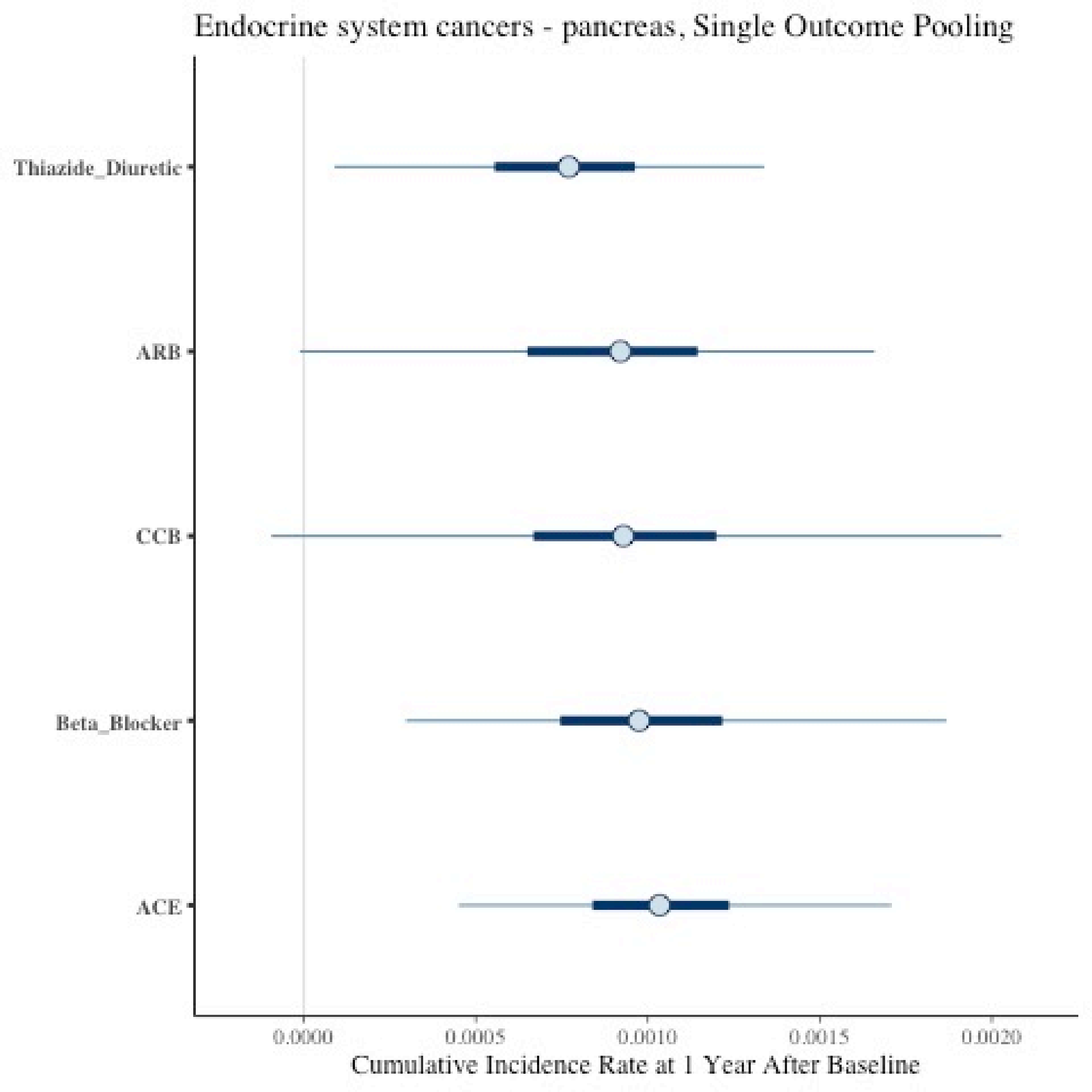
Endocrine system cancers - pancreas, Single Outcome Pooling
Thiazide_Diuretic
ARB
CCB
Beta_Blocker
ACE
0.0000
0.0005
0.0010
0.0015
0.0020
Cumulative Incidence Rate at 1 Year After Baseline

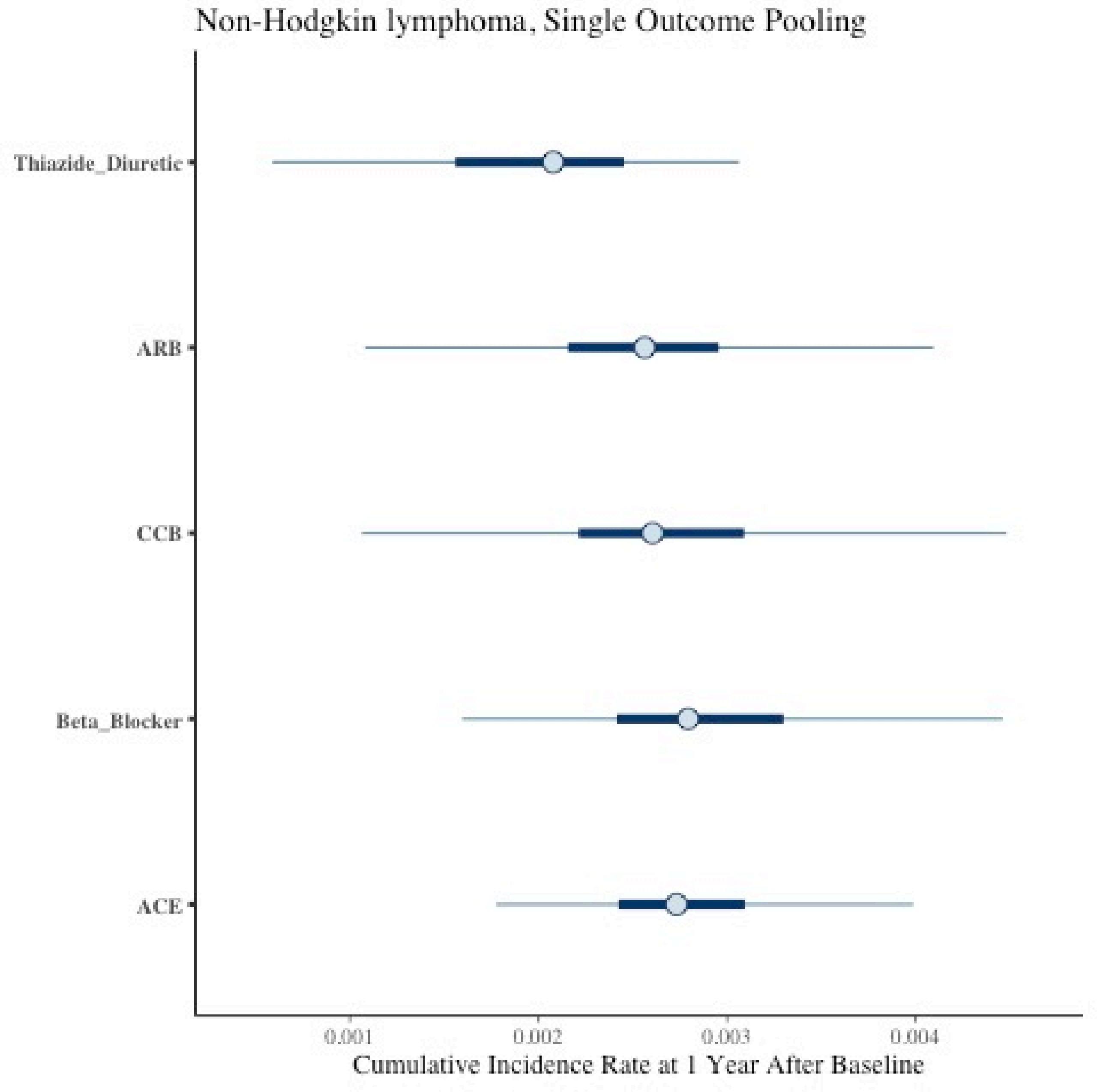

Non-Hodgkin lymphoma, Single Outcome Pooling
Thiazide_Diuretic
ARB
CCB
Beta_Blocker
ACE
0.001
0.002
0.003
0.004
Cumulative Incidence Rate at 1 Year After Baseline

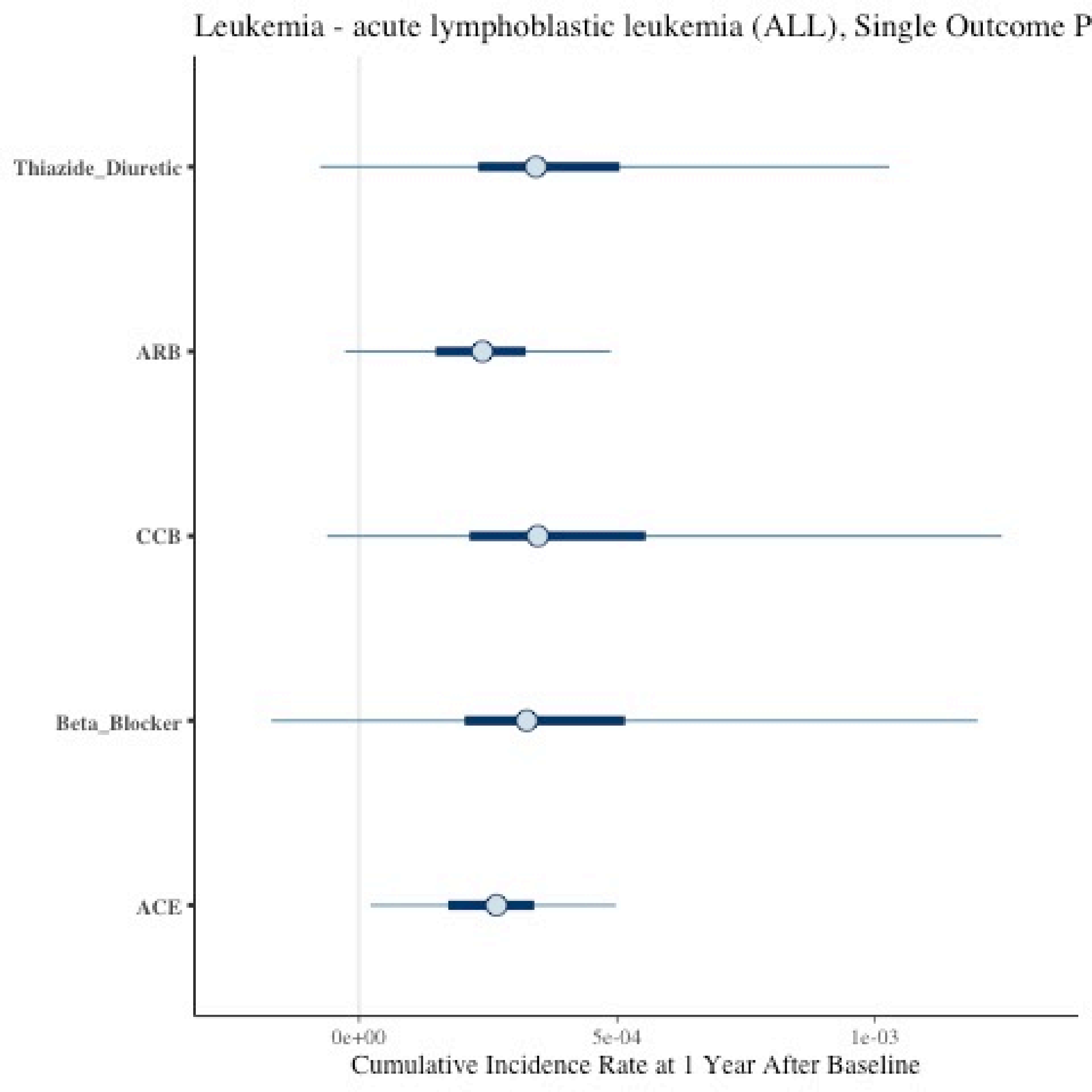
Leukemia - acute lymphoblastic leukemia (ALL), Single Outcome P
Thiazide_Diuretic
ARB
CCB
Beta_Blocker
ACE
0e+00
5e-04
1e-03
Cumulative Incidence Rate at 1 Year After Baseline

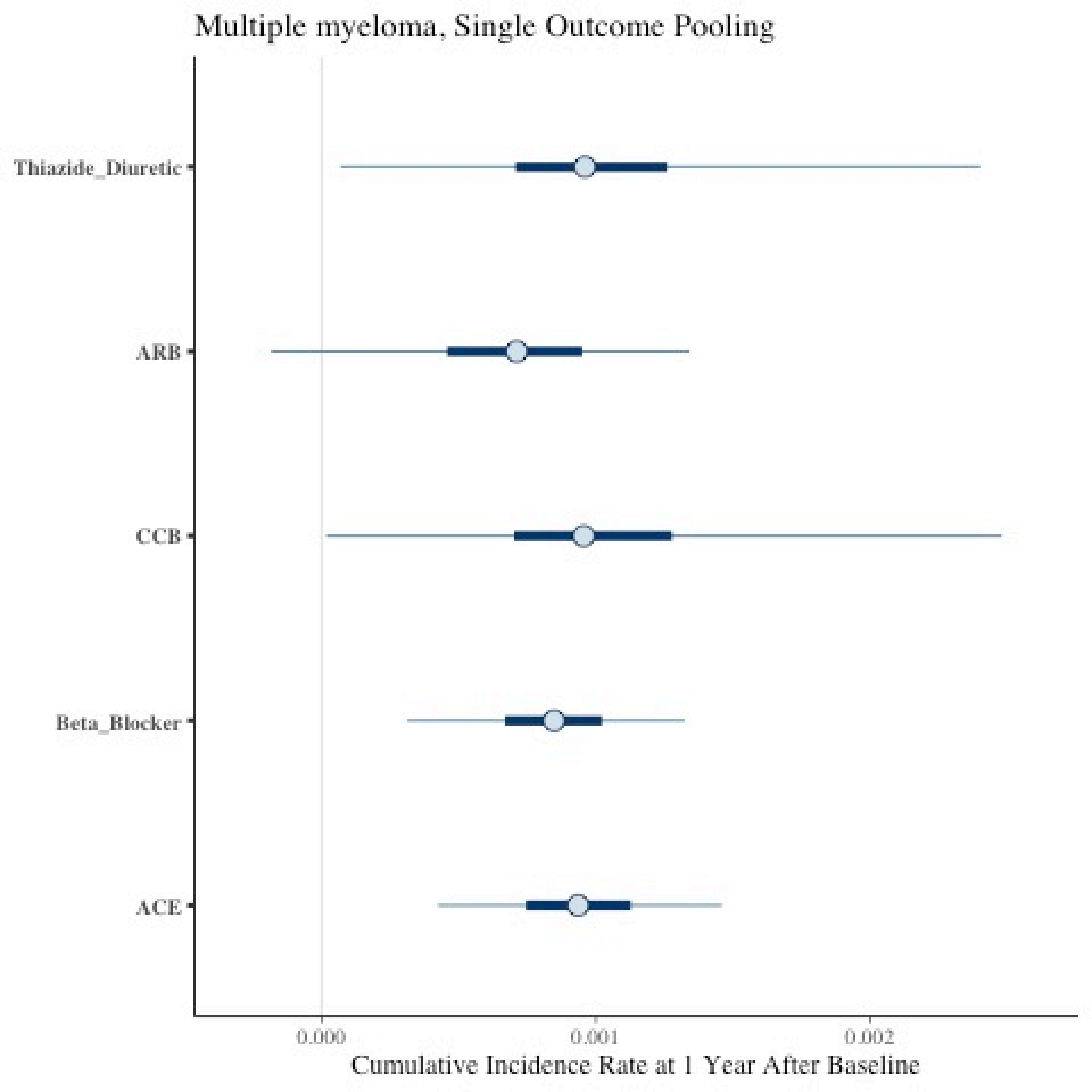

Multiple myeloma, Single Outcome Pooling
Thiazide_Diuretic
ARB
CCB
Beta_Blocker
ACE
0.000
0.001
0.002
Cumulative Incidence Rate at 1 Year After Baseline

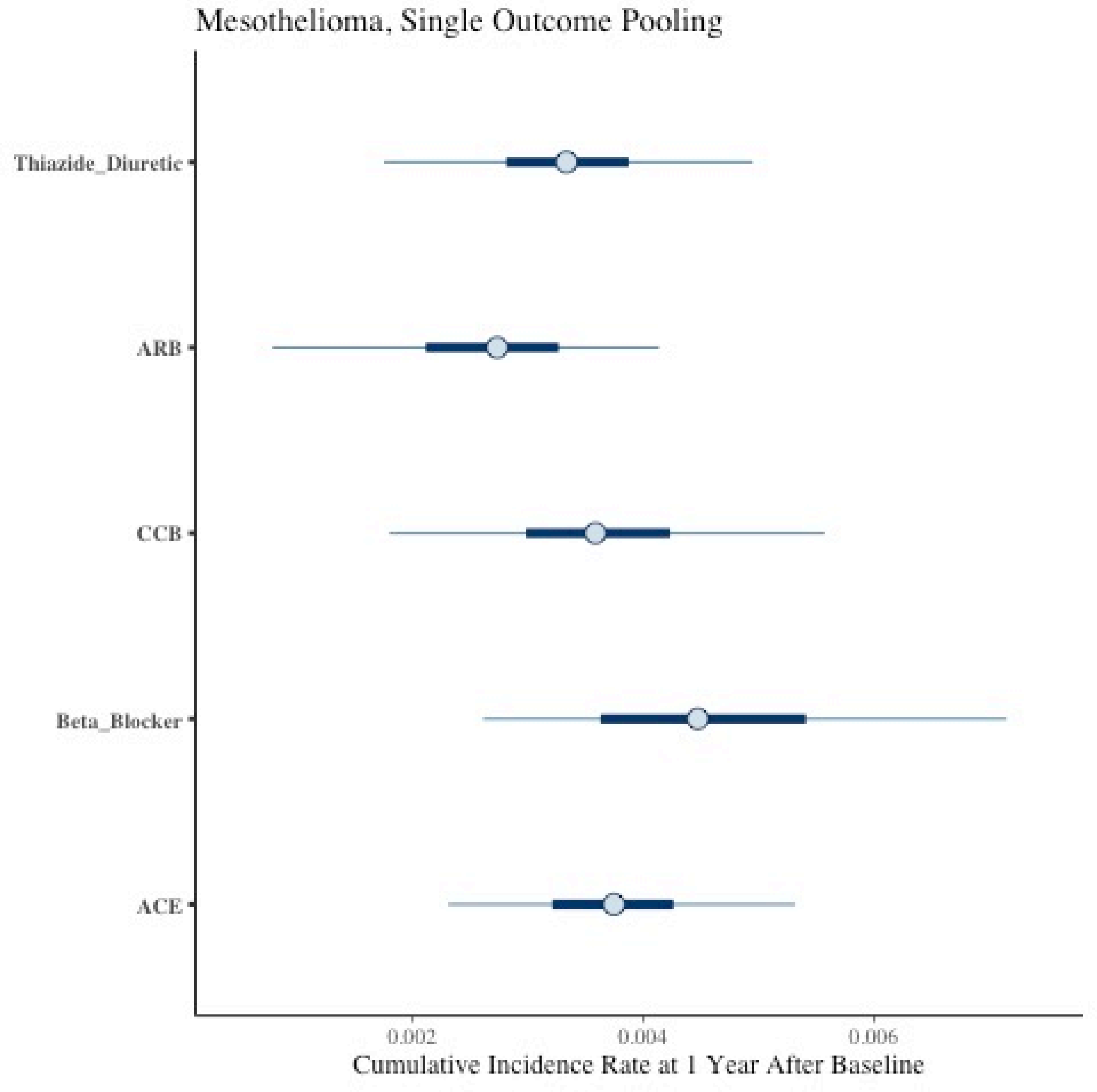
Mesothelioma, Single Outcome Pooling
Thiazide_Diuretic
ARB
CCB
Beta_Blocker
ACE
0.002
0.004
0.006
Cumulative Incidence Rate at 1 Year After Baseline

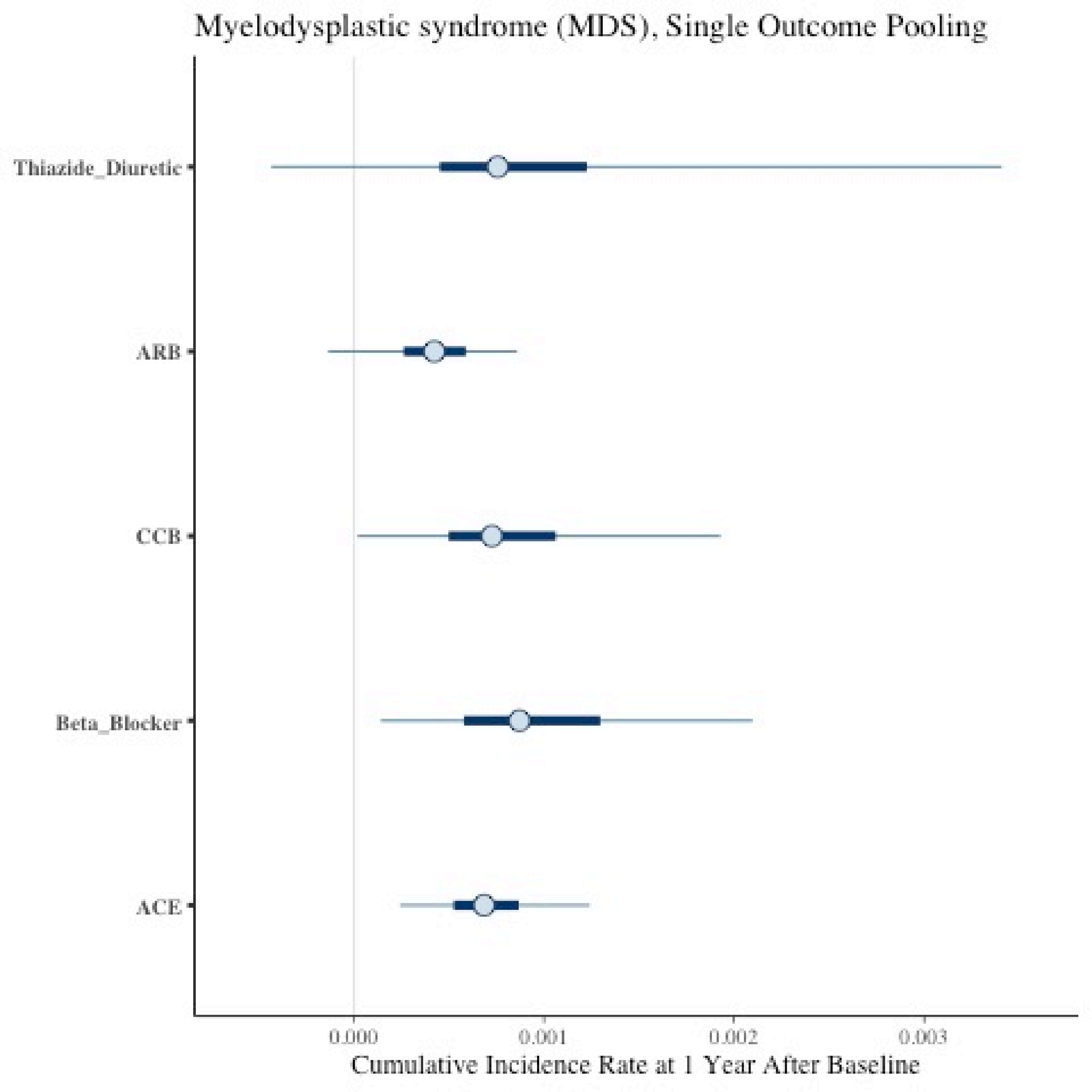

Myelodysplastic syndrome (MDS), Single Outcome Pooling
Thiazide_Diuretic
ARB
CCB
Beta_Blocker
ACE
0.000
0.001
0.002
0.003
Cumulative Incidence Rate at 1 Year After Baseline

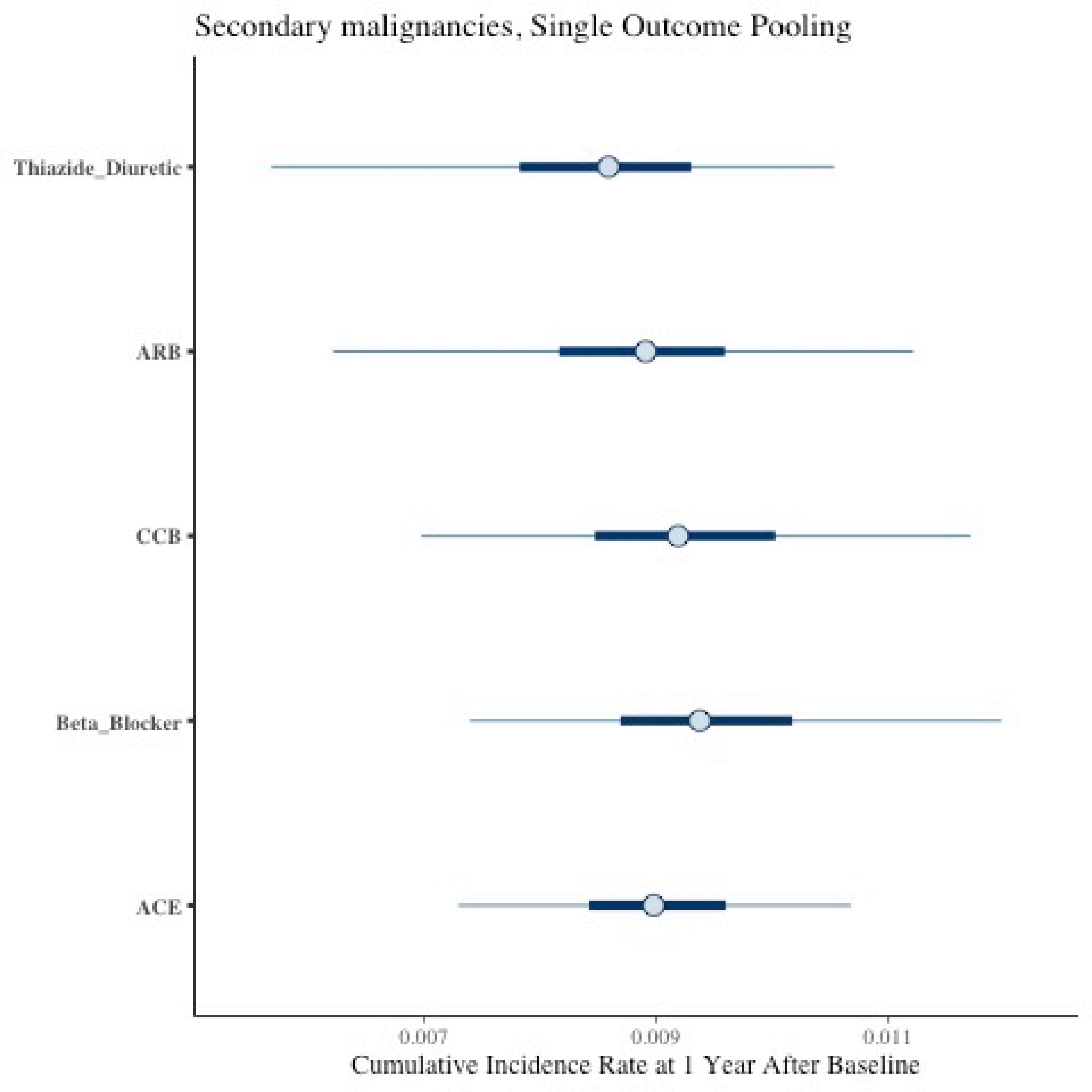
Secondary malignancies, Single Outcome Pooling
Thiazide_Diuretic
ARB
CCB
Beta_Blocker
ACE
0.007
0.009
0.011
Cumulative Incidence Rate at 1 Year After Baseline

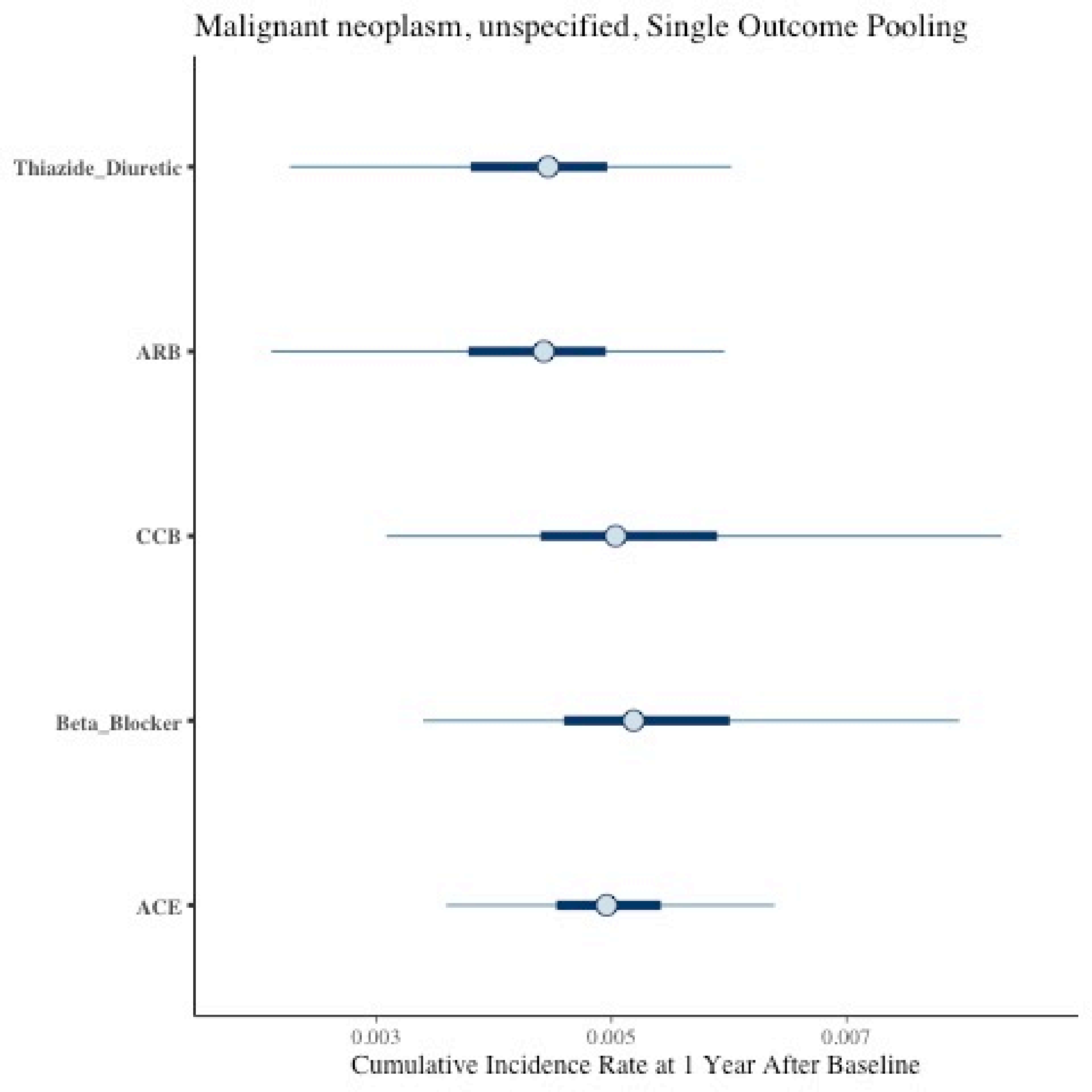

Malignant neoplasm, unspecified, Single Outcome Pooling
Thiazide_Diuretic
ARB
CCB
Beta_Blocker
ACE
0.003
0.005
0.007
Cumulative Incidence Rate at 1 Year After Baseline

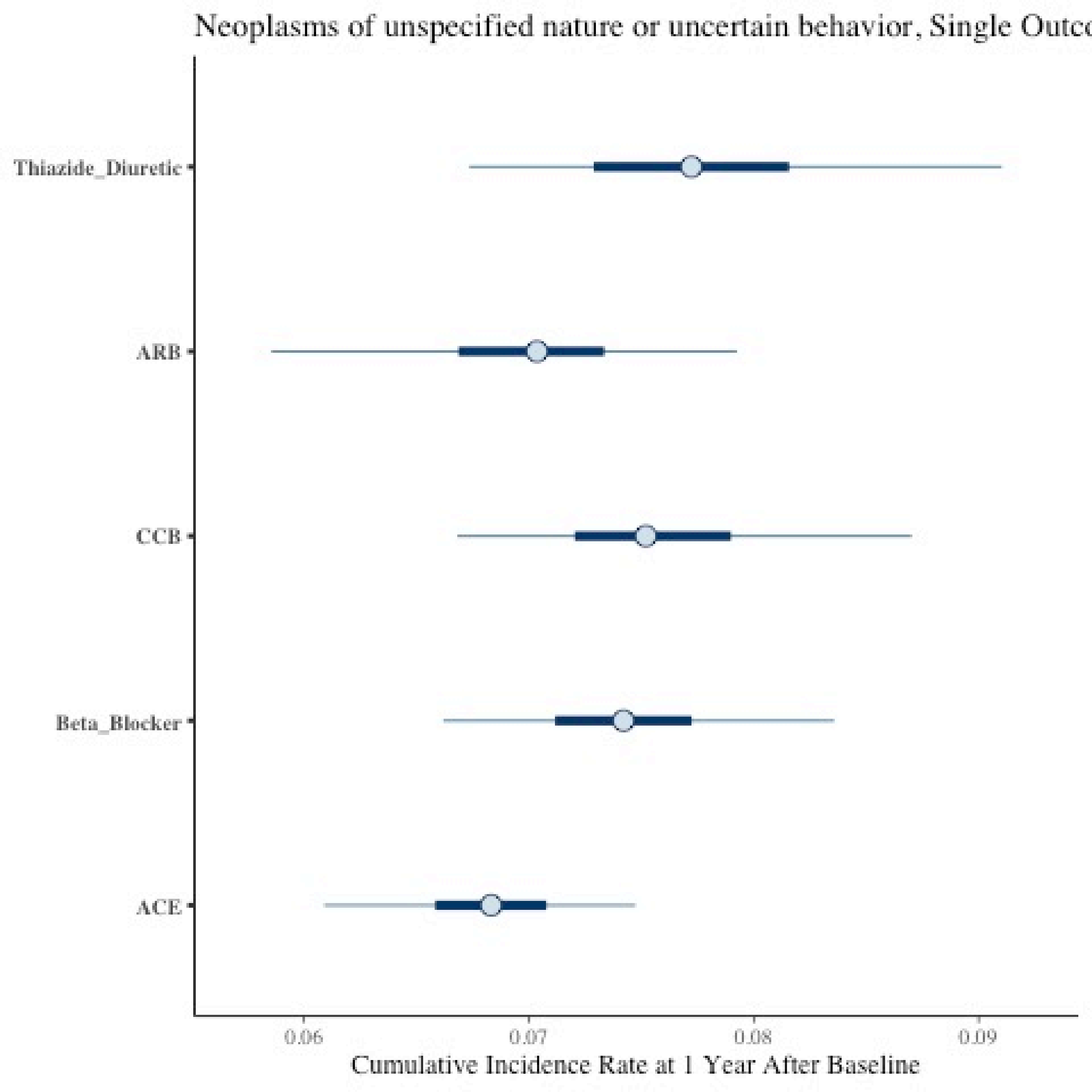

Neoplasms of unspecified nature or uncertain behavior, Single Outco
Thiazide_Diuretic
ARB
CCB
Beta_Blocker
ACE
0.06
0.07
0.08
0.09
Cumulative Incidence Rate at 1 Year After Baseline

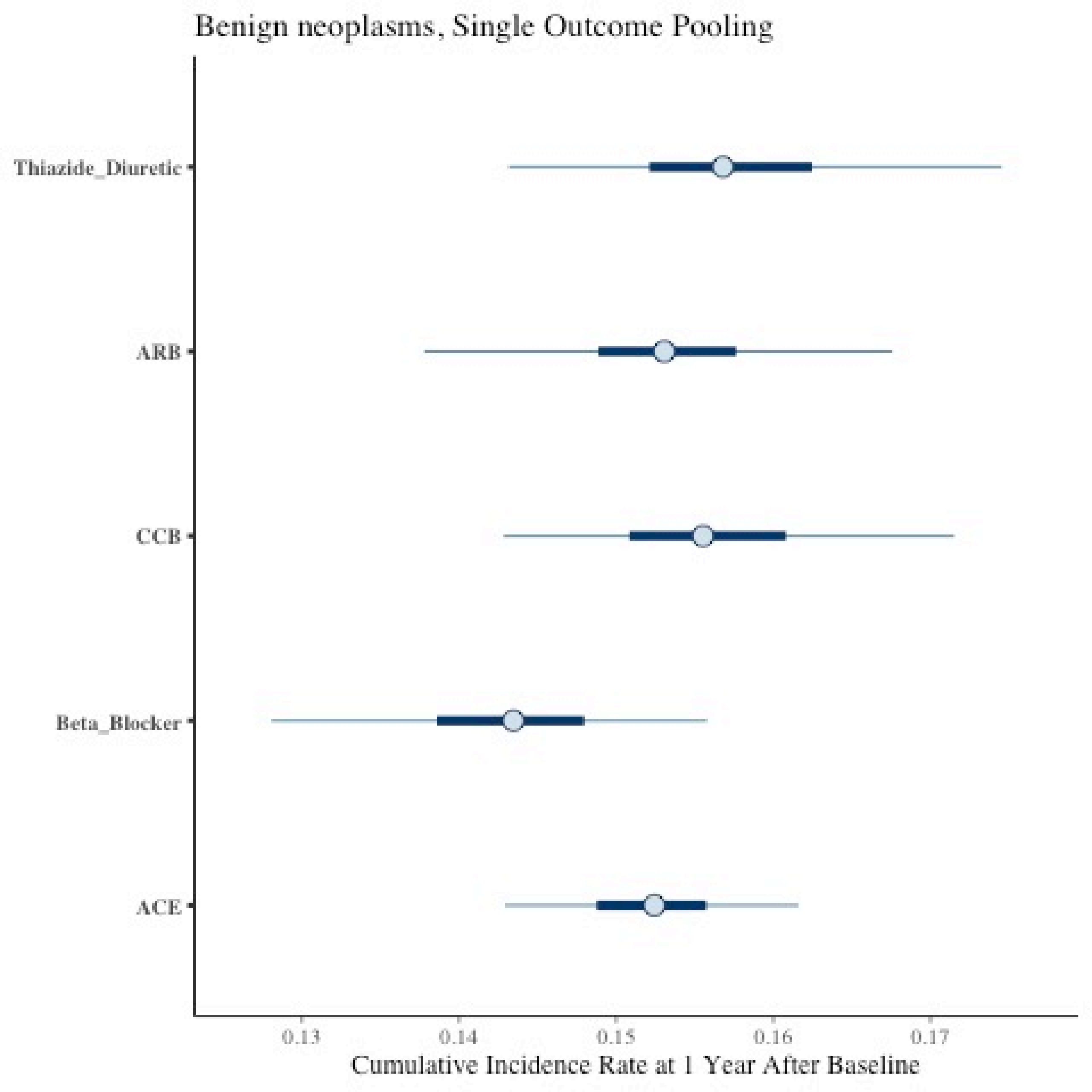

Benign neoplasms, Single Outcome Pooling
Thiazide_Diuretic
ARB
CCB
Beta_Blocker
ACE
0.13
0.14
0.15
0.16
0.17
Cumulative Incidence Rate at 1 Year After Baseline

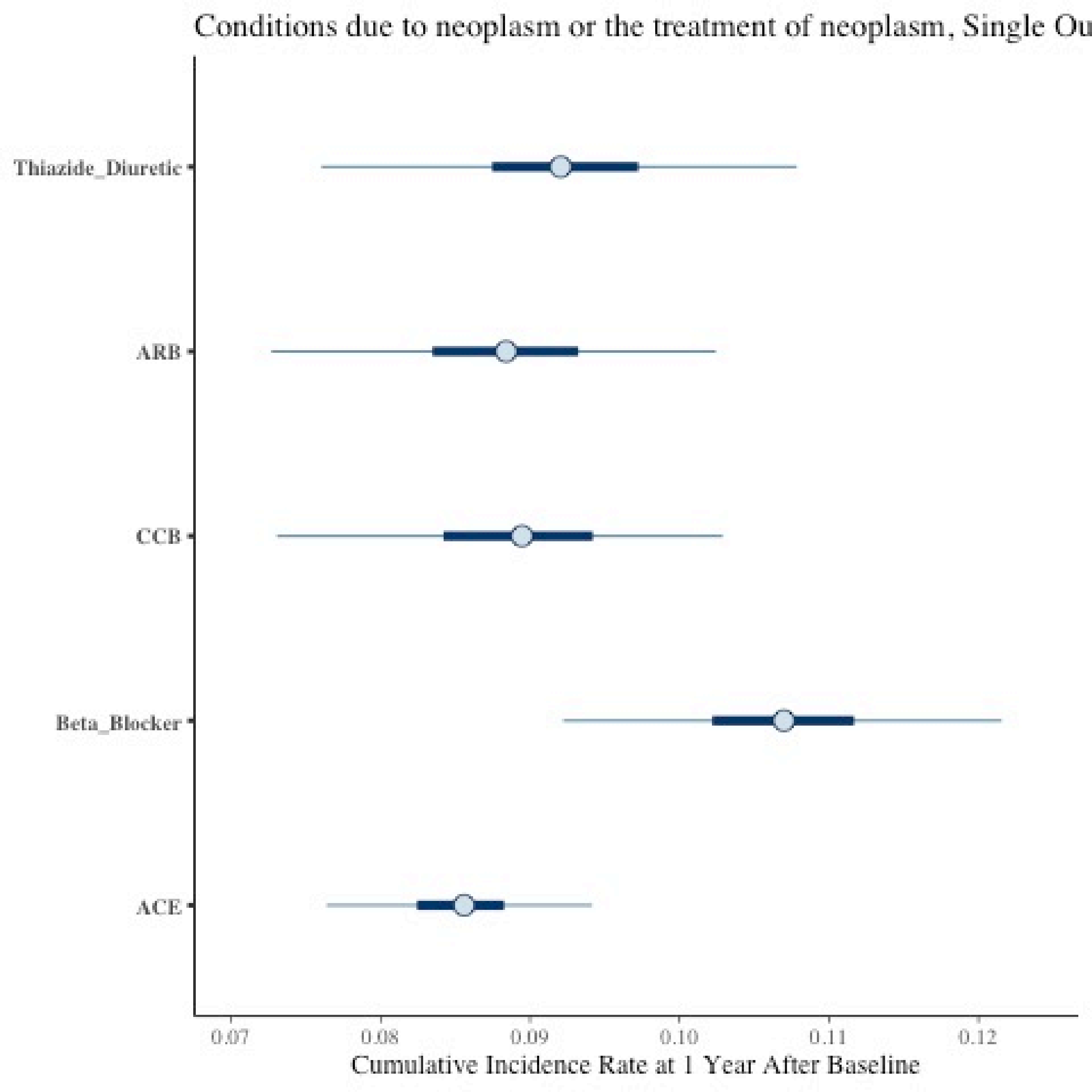

Conditions due to neoplasm or the treatment of neoplasm, Single Ou
Thiazide_Diuretic
ARB
CCB
Beta_Blocker
ACE
0.07
0.08
0.09
0.10
0.11
0.12
Cumulative Incidence Rate at 1 Year After Baseline

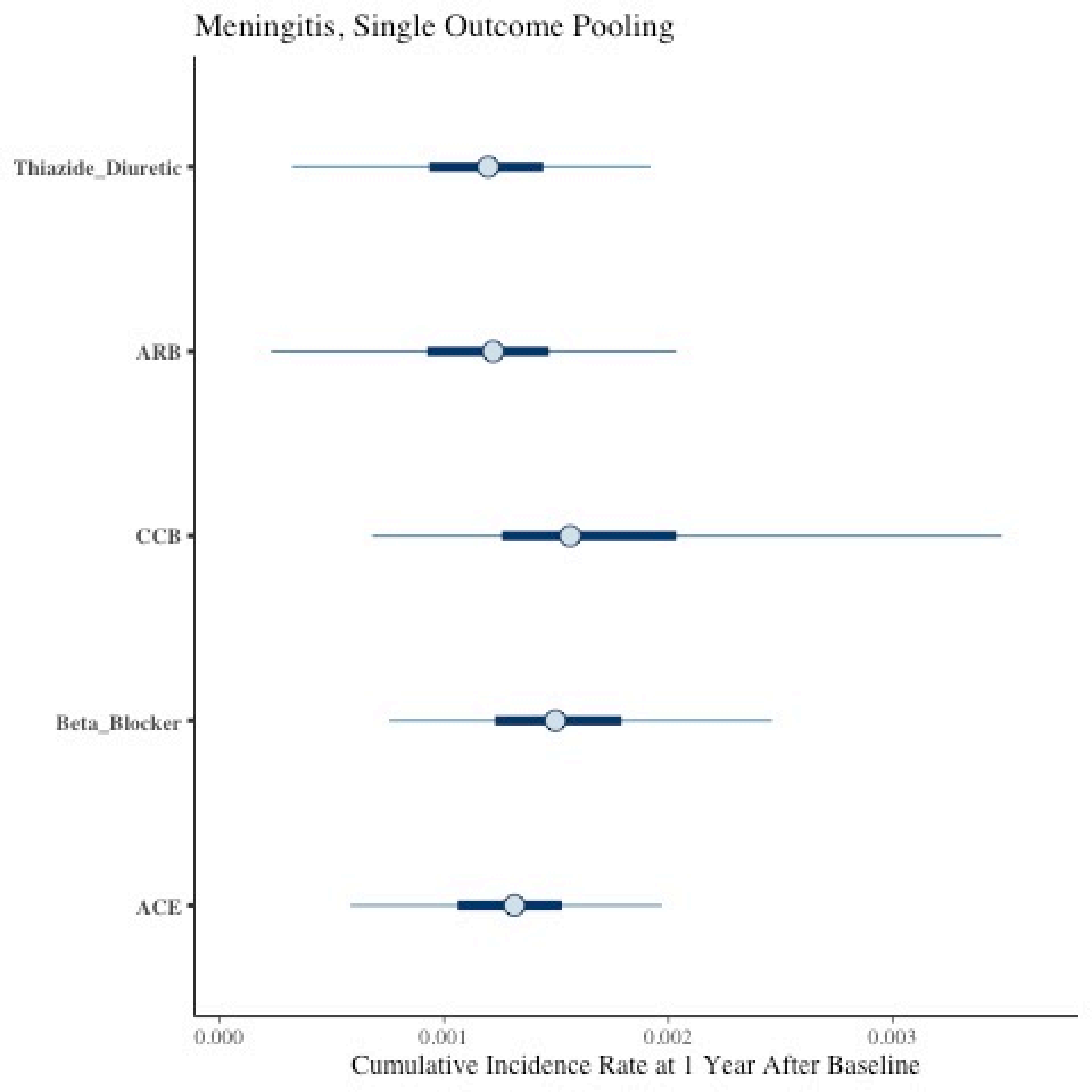

Meningitis, Single Outcome Pooling
Thiazide_Diuretic
ARB
CCB
Beta_Blocker
ACE
0.000
0.001
0.002
0.003
Cumulative Incidence Rate at 1 Year After Baseline

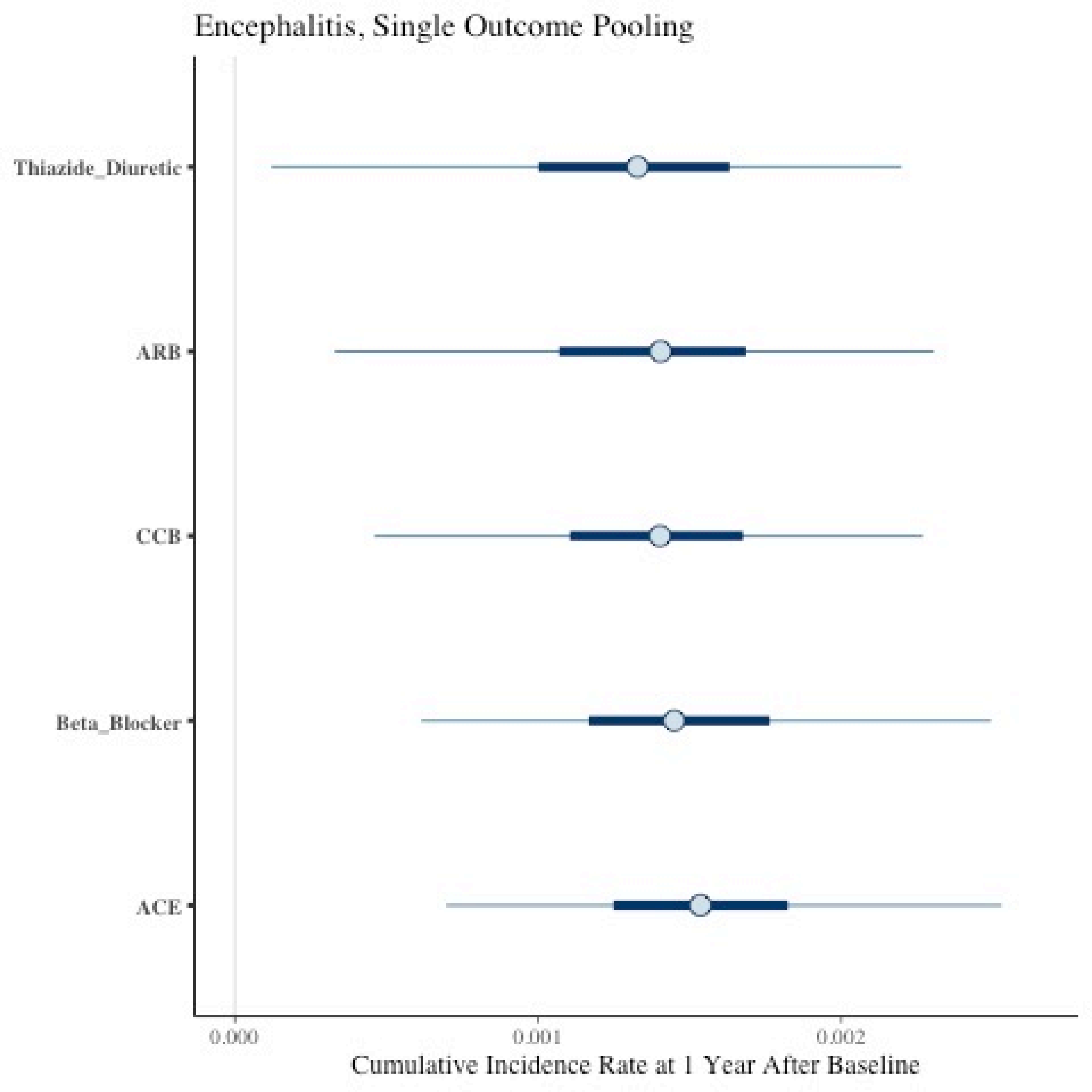

Encephalitis, Single Outcome Pooling
Thiazide_Diuretic
ARB
CCB
Beta_Blocker
ACE
0.000
0.001
0.002
Cumulative Incidence Rate at 1 Year After Baseline

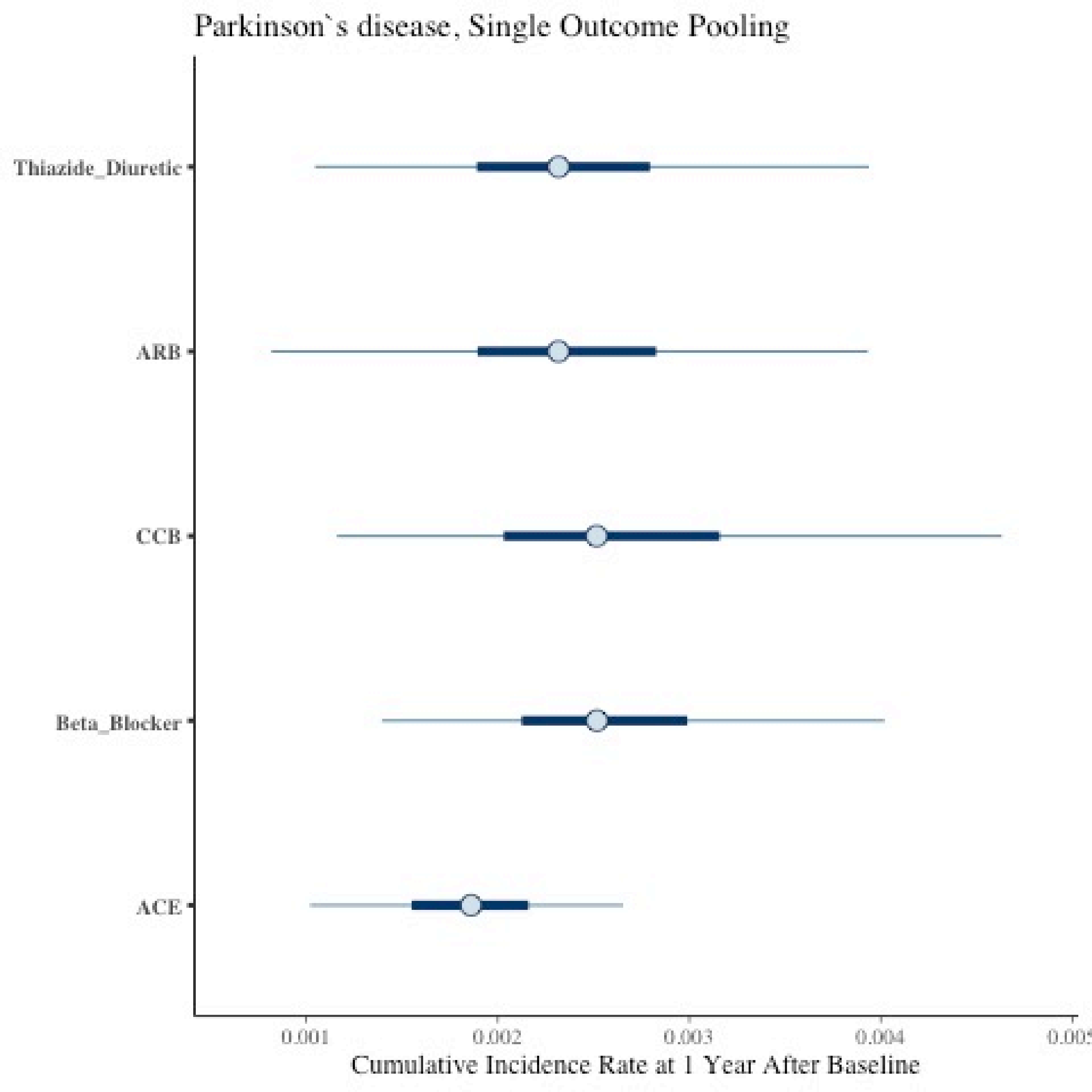

Parkinson`s disease, Single Outcome Pooling
Thiazide_Diuretic
ARB
CCB
Beta_Blocker
ACE
0.001
0.002
0.003
0.004
0.005
Cumulative Incidence Rate at 1 Year After Baseline

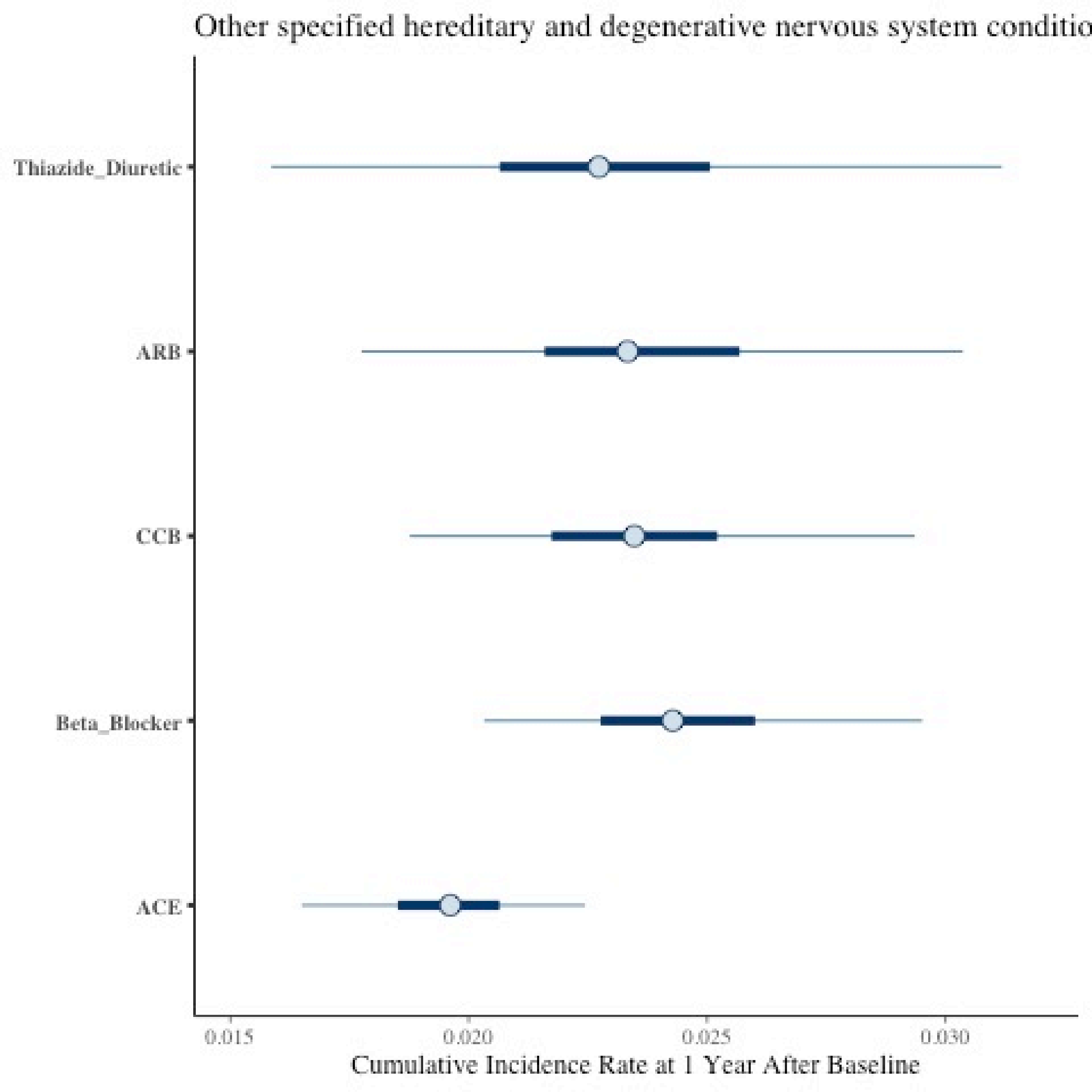

Other specified hereditary and degenerative nervous system conditio
Thiazide_Diuretic
ARB
CCB
Beta_Blocker
ACE
0.015
0.020
0.025
0.030
Cumulative Incidence Rate at 1 Year After Baseline

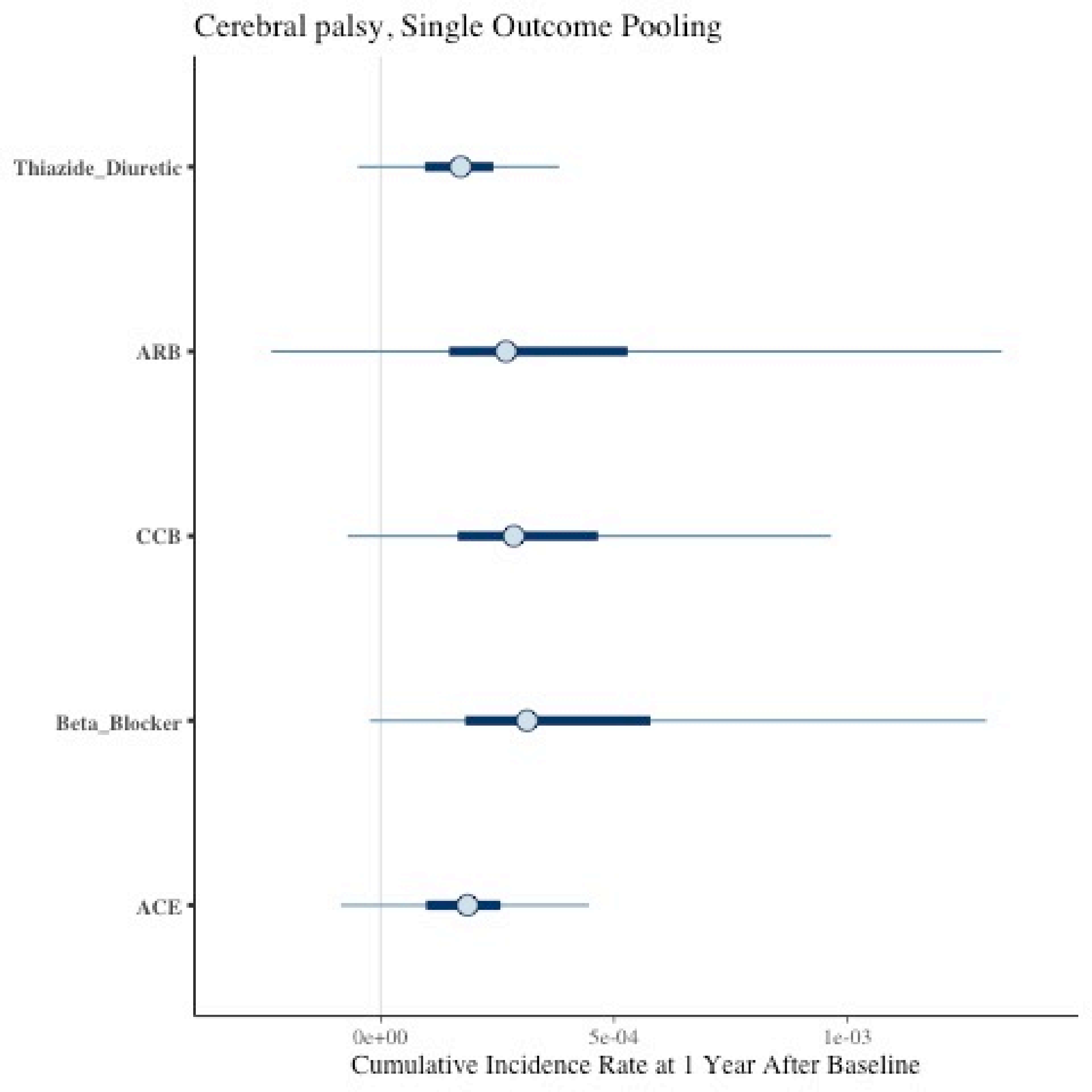

Cerebral palsy, Single Outcome Pooling
Thiazide_Diuretic
ARB
CCB
Beta_Blocker
ACE
0e+00
5e-04
1e-03
Cumulative Incidence Rate at 1 Year After Baseline

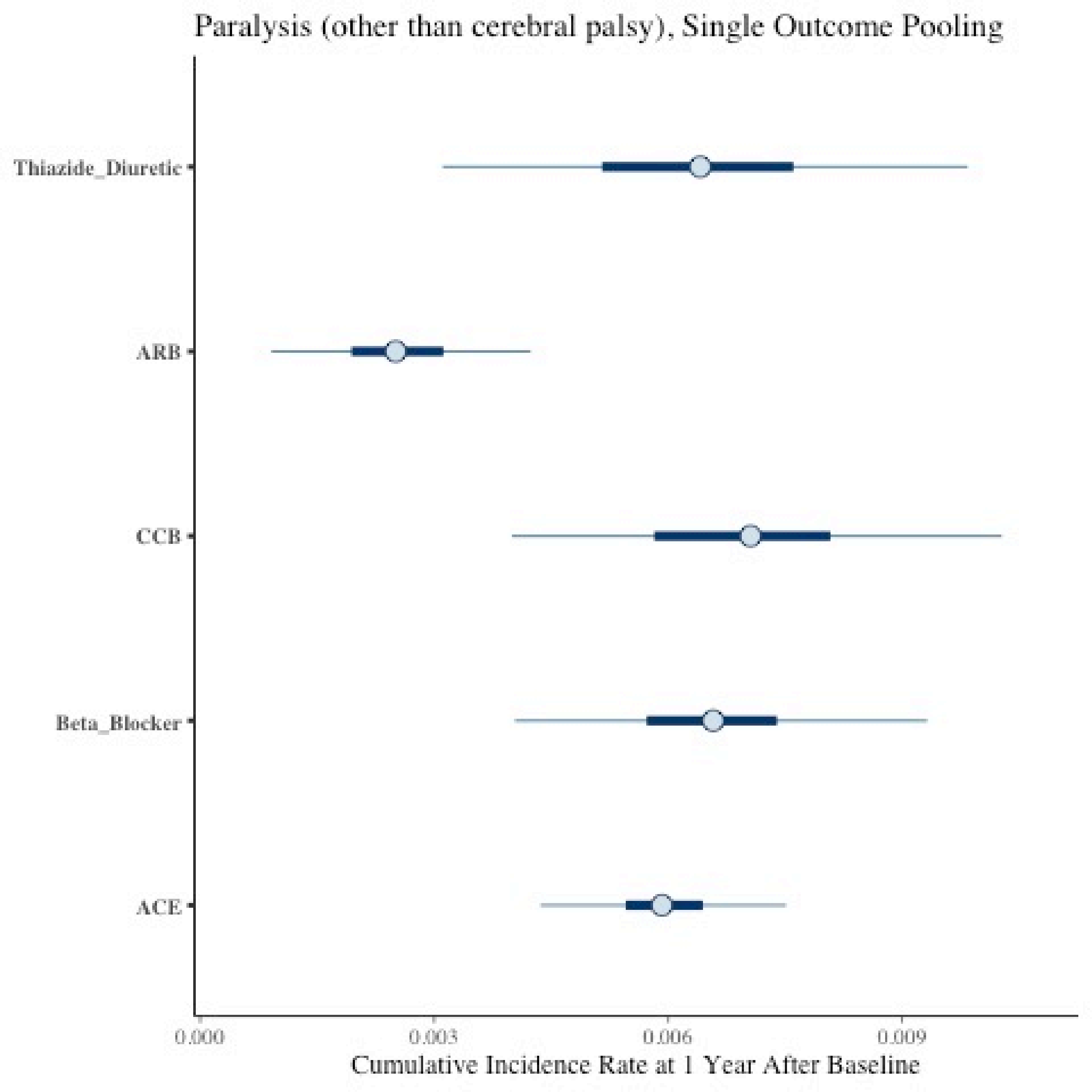

Paralysis (other than cerebral palsy), Single Outcome Pooling
Thiazide_Diuretic
ARB
CCB
Beta_Blocker
ACE
0.000
0.003
0.006
0.009
Cumulative Incidence Rate at 1 Year After Baseline

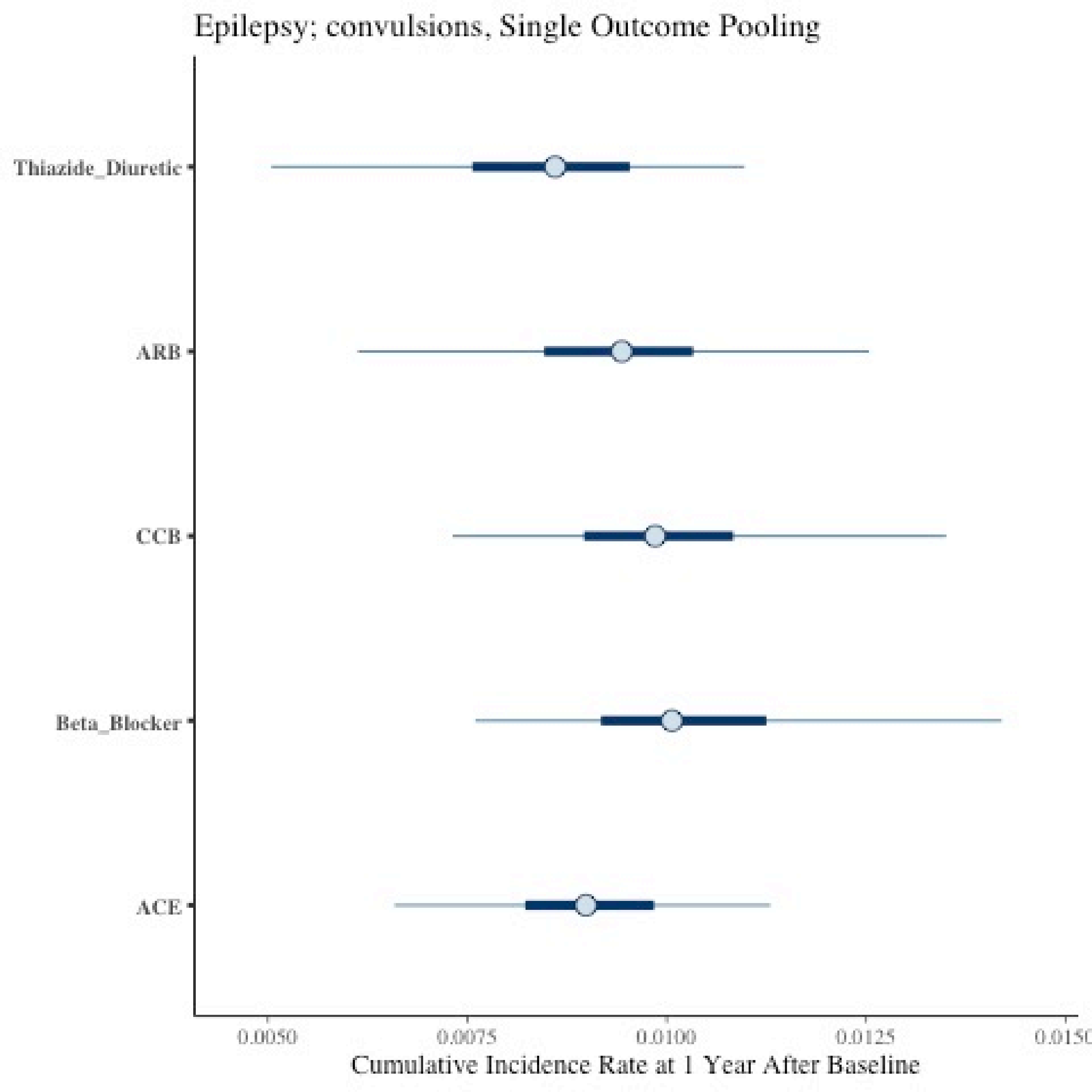
Epilepsy; convulsions, Single Outcome Pooling
Thiazide_Diuretic
ARB
CCB
Beta_Blocker
ACE
0.0050
0.0075
0.0100
0.0125
0.0150
Cumulative Incidence Rate at 1 Year After Baseline

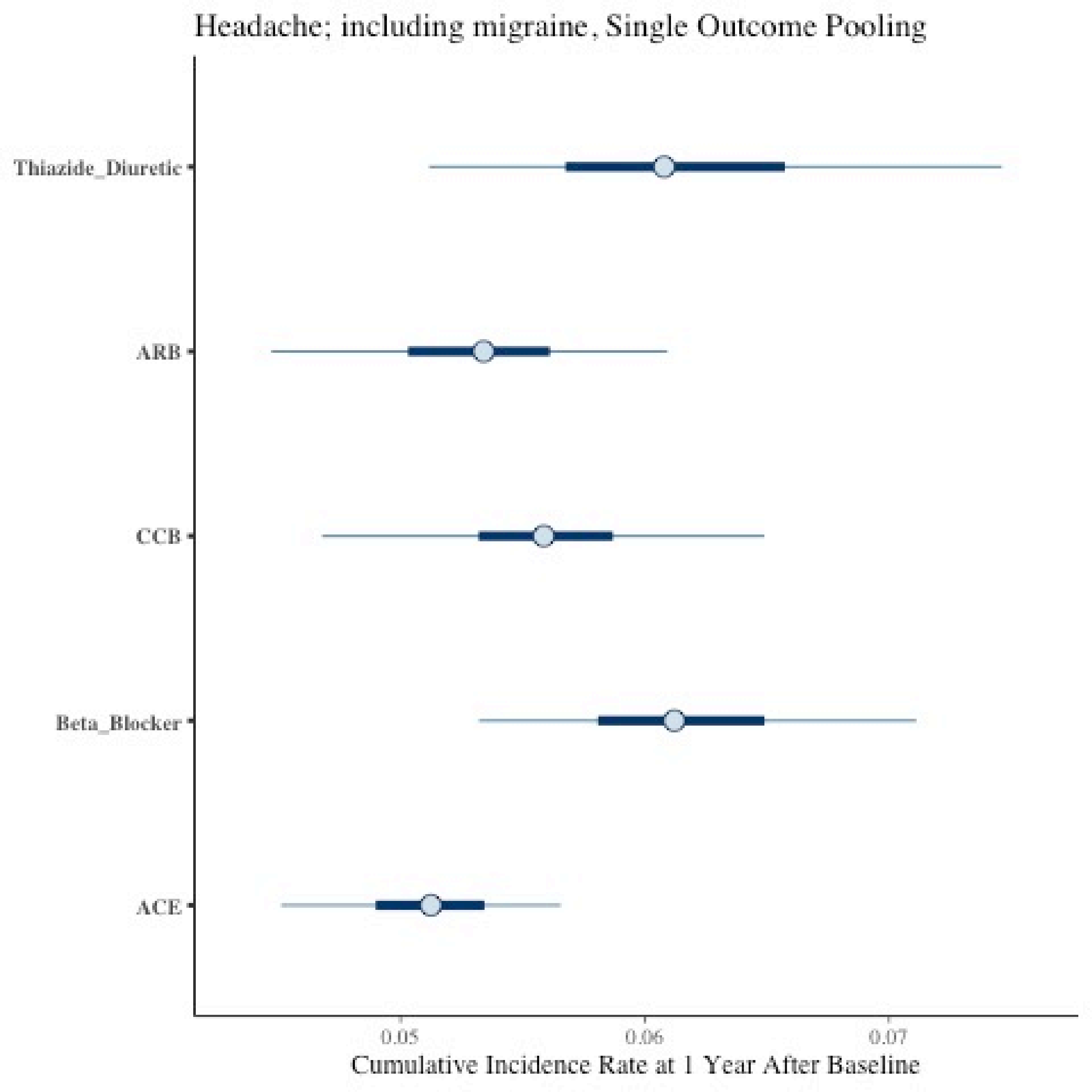
Headache; including migraine, Single Outcome Pooling
Thiazide_Diuretic
ARB
CCB
Beta_Blocker
ACE
0.05
0.06
0.07
Cumulative Incidence Rate at 1 Year After Baseline

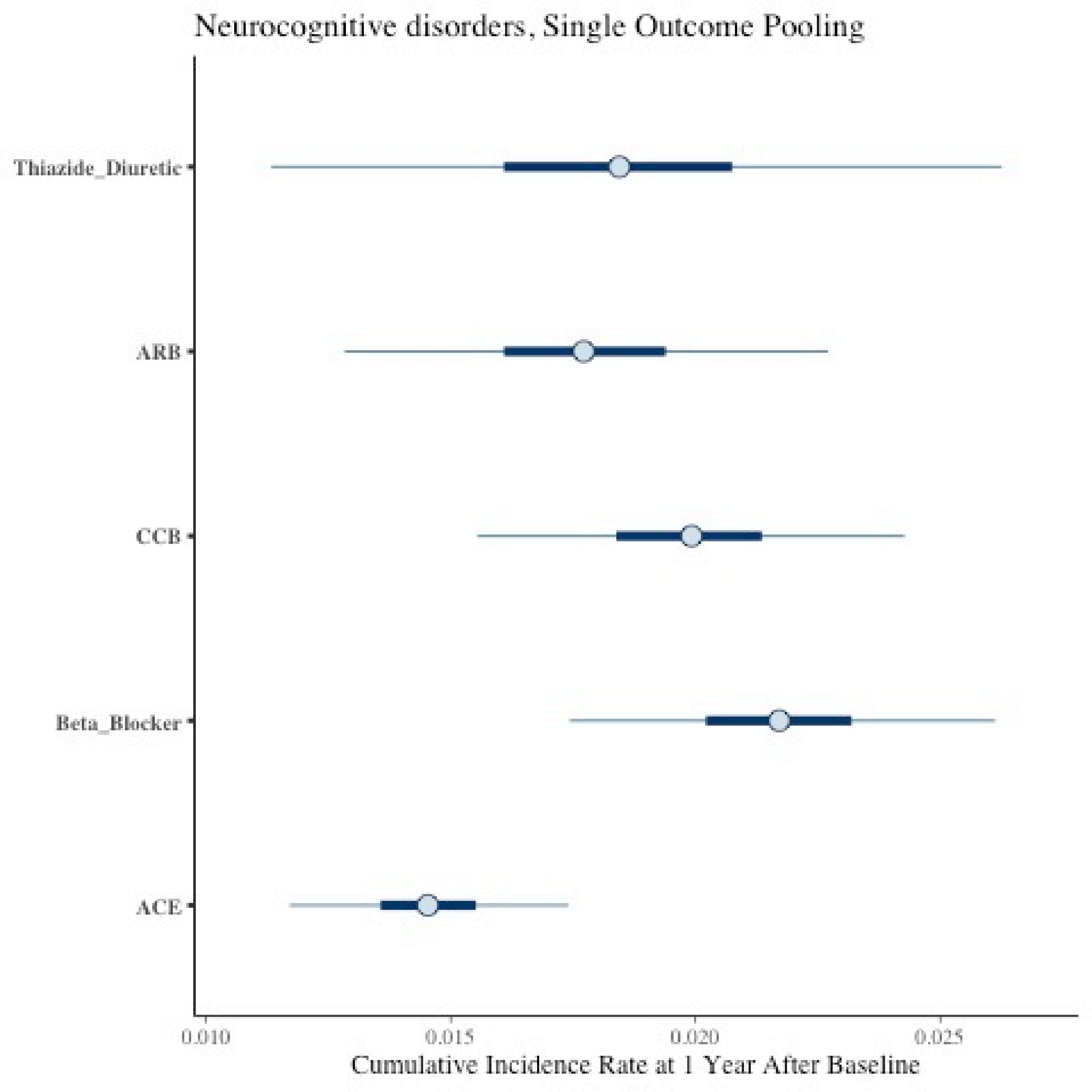
Neurocognitive disorders, Single Outcome Pooling
Thiazide_Diuretic
ARB
CCB
Beta_Blocker
ACE
0.010
0.015
0.020
0.025
Cumulative Incidence Rate at 1 Year After Baseline

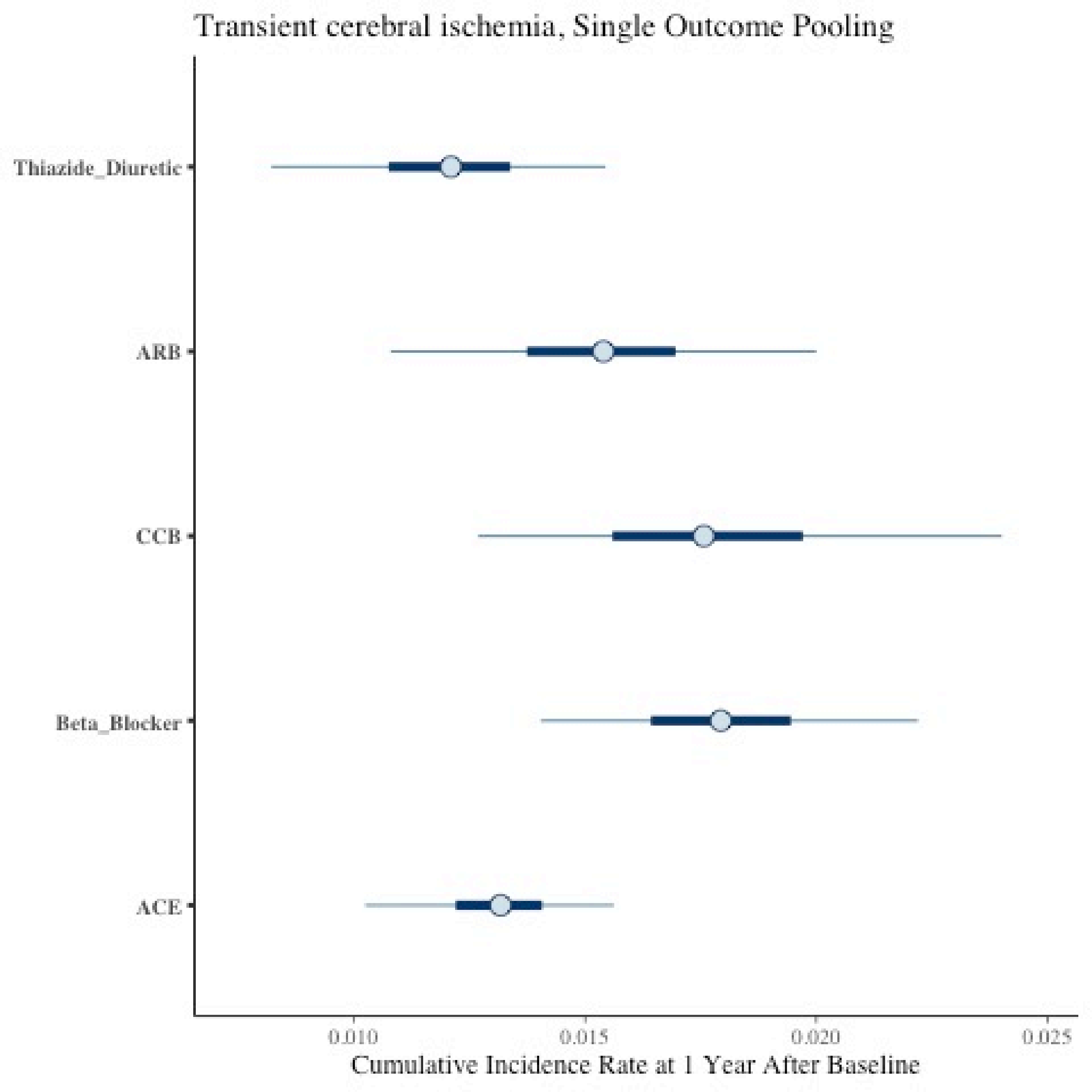

Transient cerebral ischemia, Single Outcome Pooling
Thiazide_Diuretic
ARB
CCB
Beta_Blocker
ACE
0.010
0.015
0.020
0.025
Cumulative Incidence Rate at 1 Year After Baseline

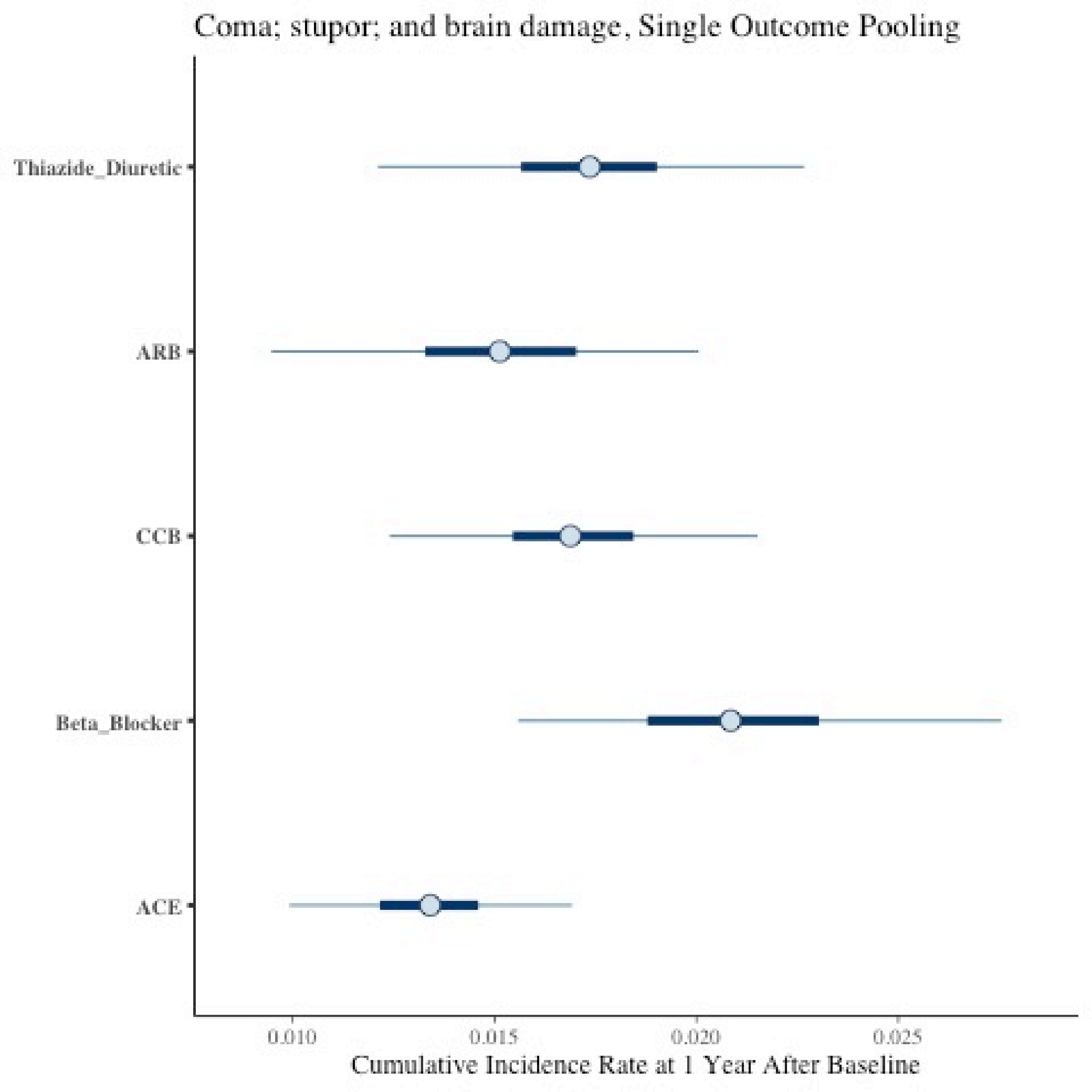
Coma; stupor; and brain damage, Single Outcome Pooling
Thiazide_Diuretic
ARB
CCB
Beta_Blocker
ACE
0.010
0.015
0.020
0.025
Cumulative Incidence Rate at 1 Year After Baseline

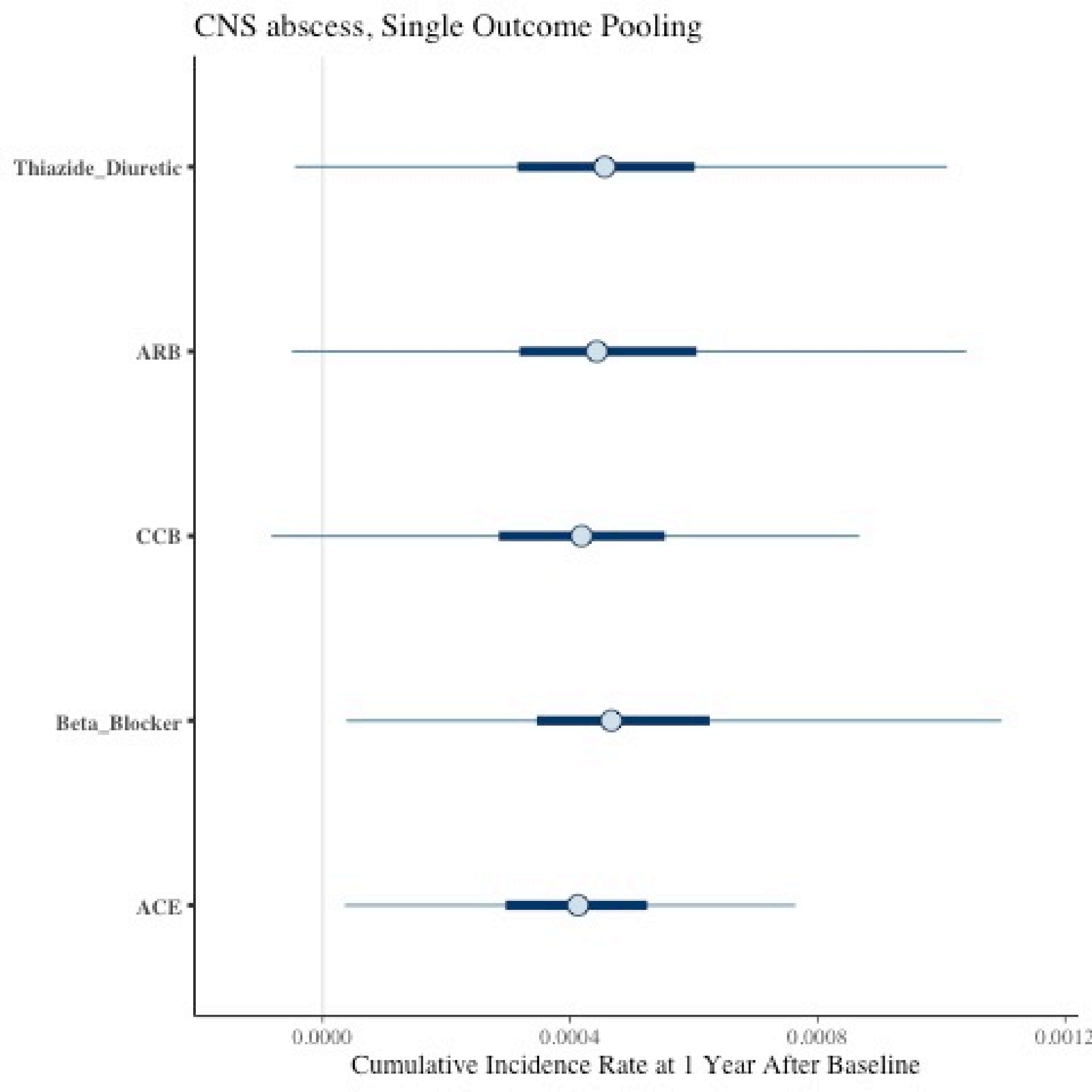
CNS abscess, Single Outcome Pooling
Thiazide_Diuretic
ARB
CCB
Beta_Blocker
ACE
0.0000
0.0004
0.0008
0.0012
Cumulative Incidence Rate at 1 Year After Baseline

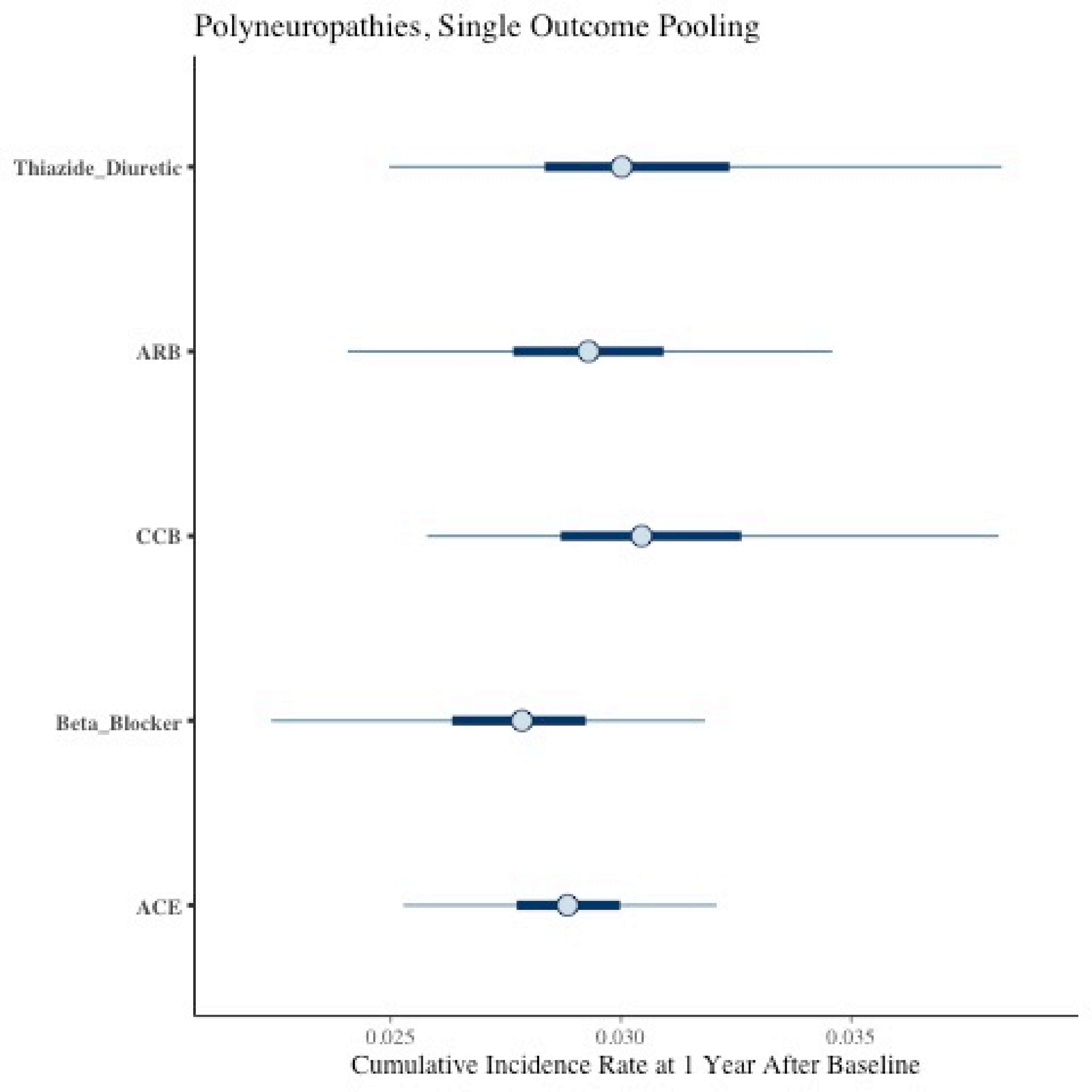

Polyneuropathies, Single Outcome Pooling
Thiazide_Diuretic
ARB
CCB
Beta_Blocker
ACE
0.025
0.030
0.035
Cumulative Incidence Rate at 1 Year After Baseline

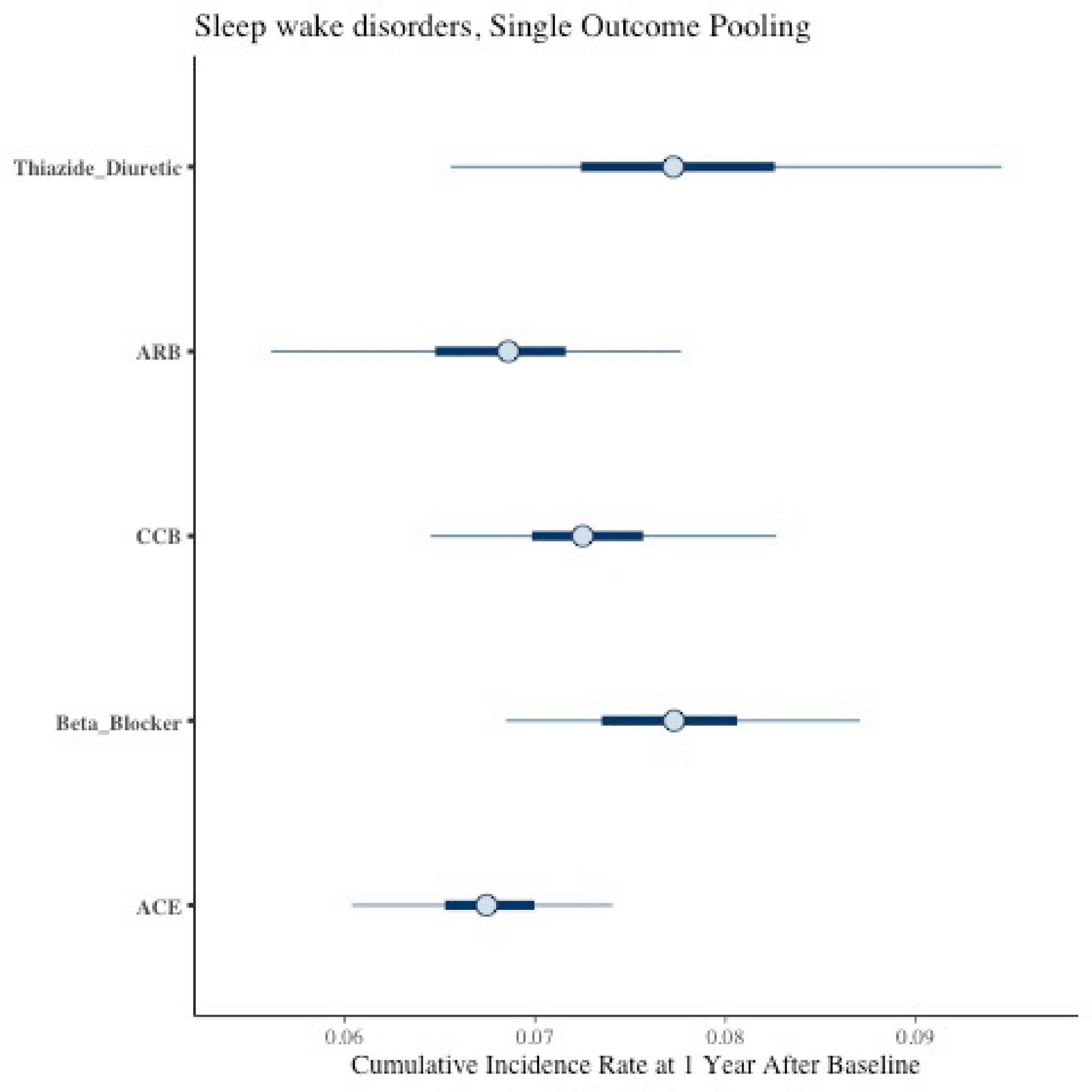

Sleep wake disorders, Single Outcome Pooling
Thiazide_Diuretic
ARB
CCB
Beta_Blocker
ACE
0.06
0.07
0.08
0.09
Cumulative Incidence Rate at 1 Year After Baseline

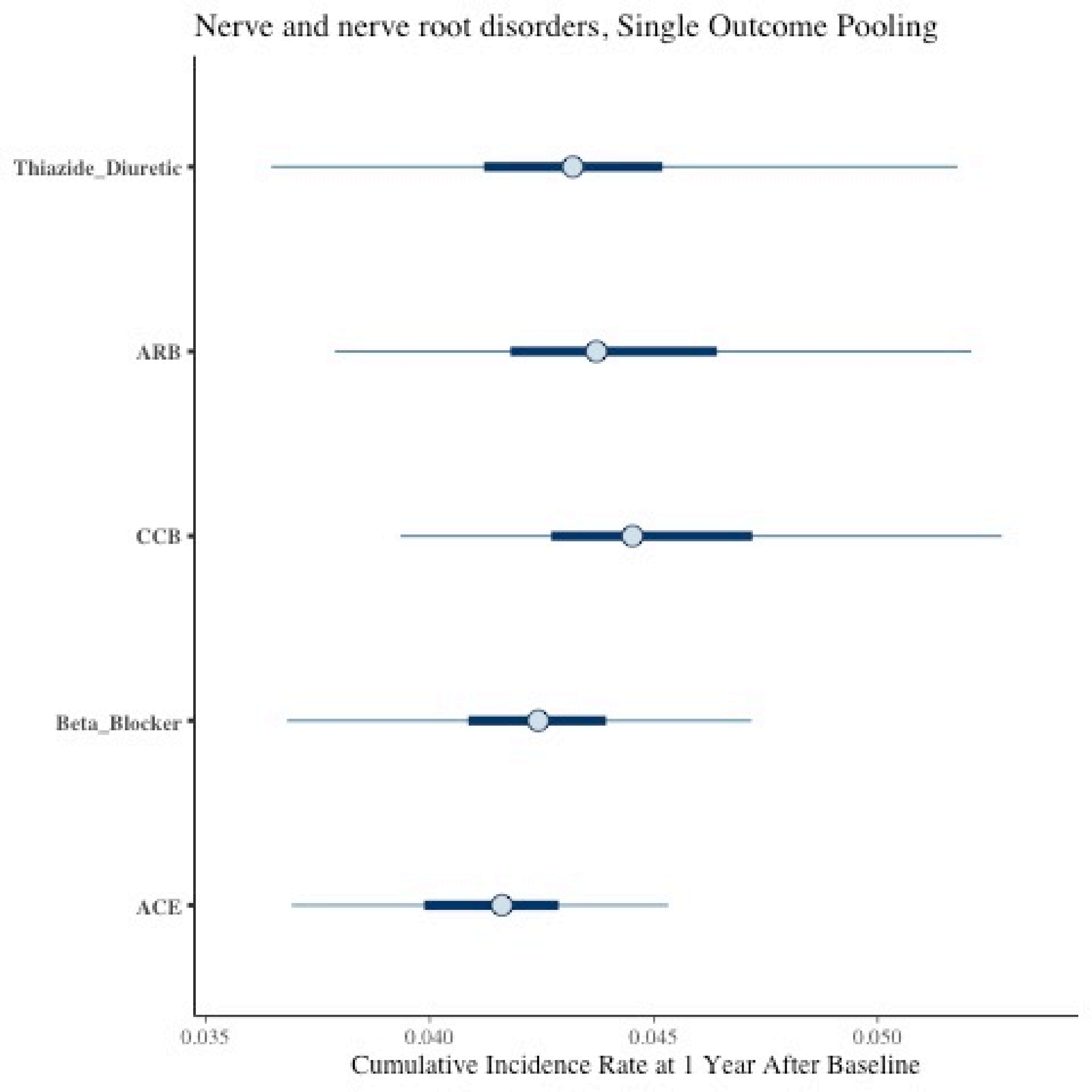

Nerve and nerve root disorders, Single Outcome Pooling
Thiazide_Diuretic
ARB
CCB
Beta_Blocker
ACE
0.035
0.040
0.045
0.050
Cumulative Incidence Rate at 1 Year After Baseline

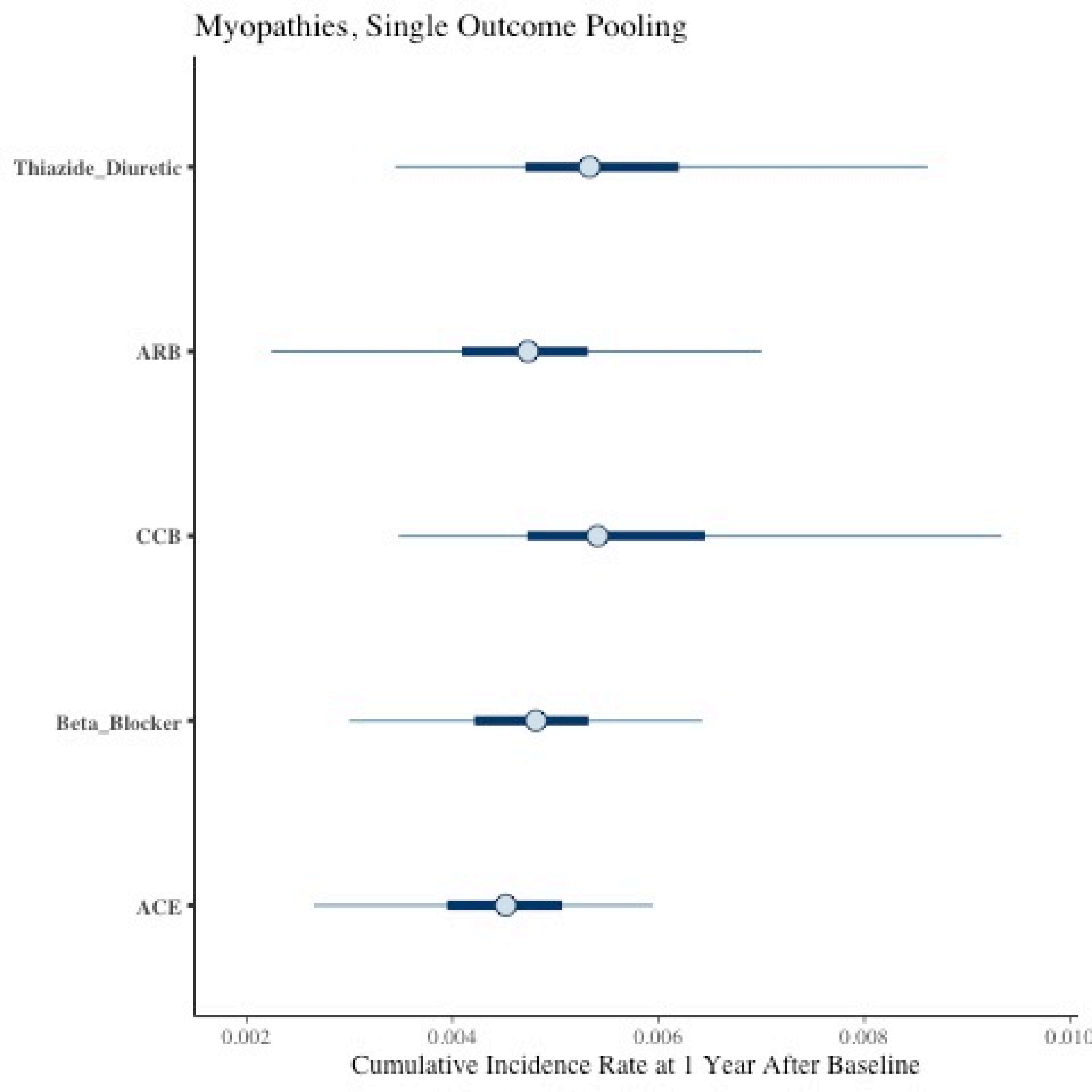

Myopathies, Single Outcome Pooling
Thiazide_Diuretic
ARB
CCB
Beta_Blocker
ACE
0.002
0.004
0.006
0.008
0.010
Cumulative Incidence Rate at 1 Year After Baseline

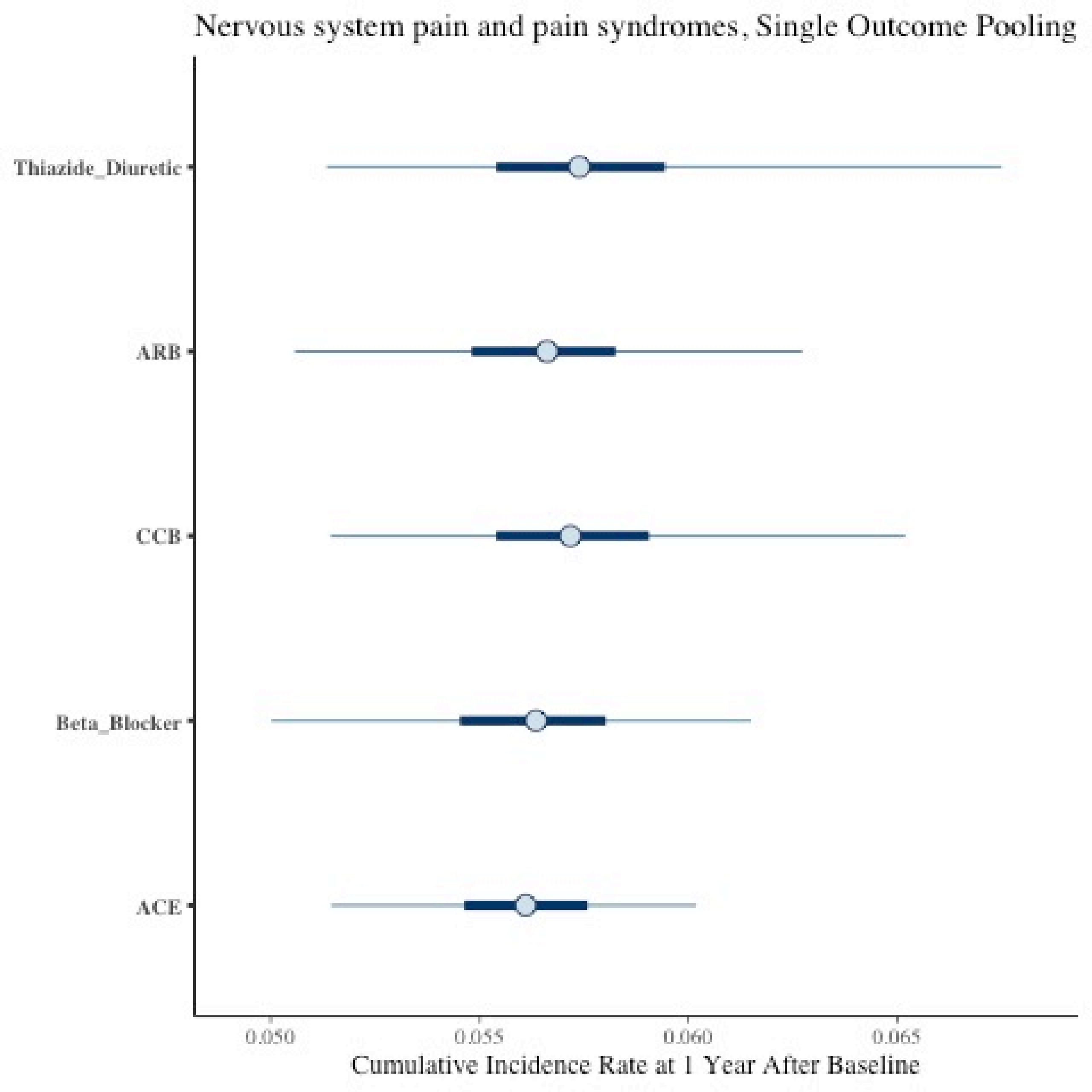

Nervous system pain and pain syndromes, Single Outcome Pooling
Thiazide_Diuretic
ARB
CCB
Beta_Blocker
ACE
0.050
0.055
0.060
0.065
Cumulative Incidence Rate at 1 Year After Baseline

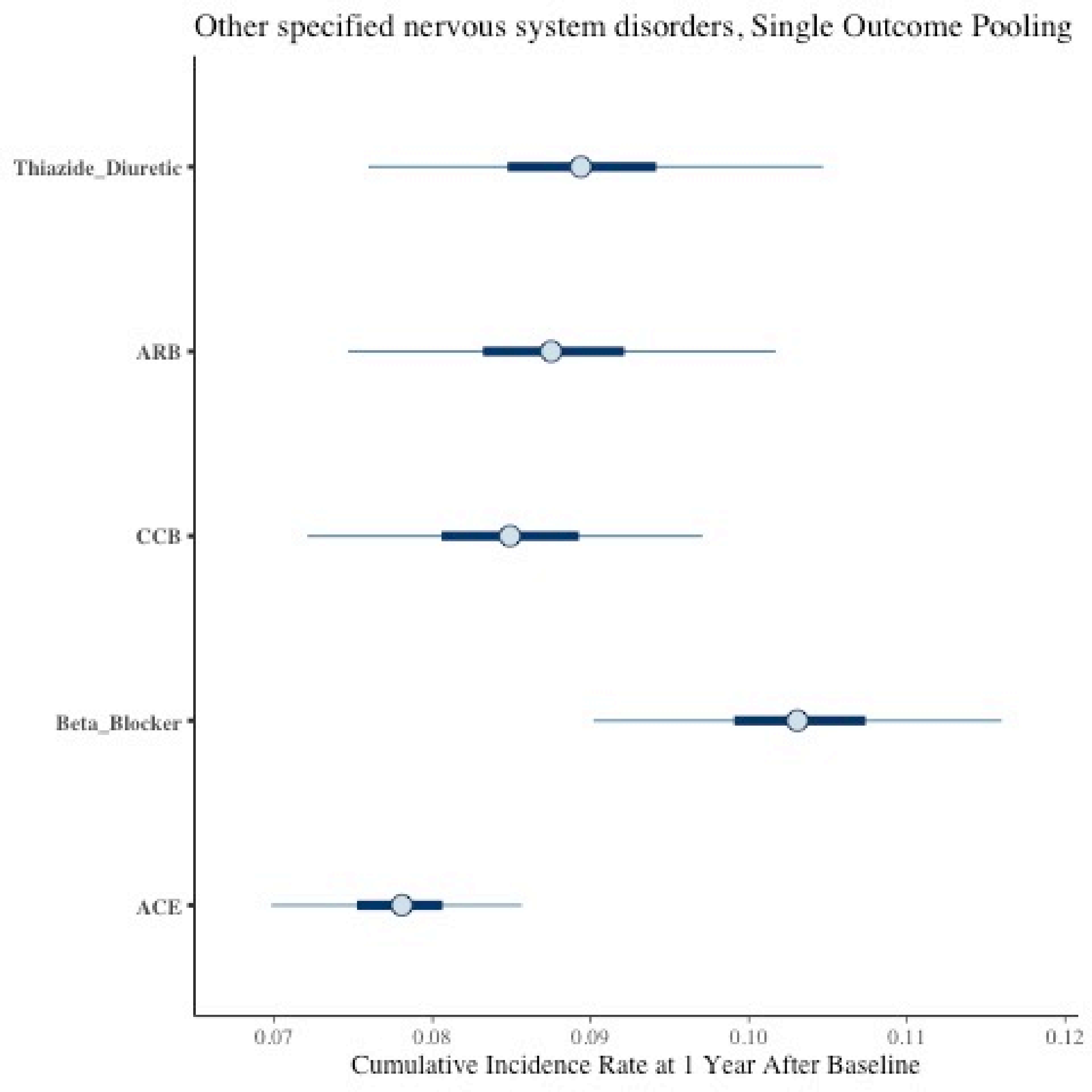

Other specified nervous system disorders, Single Outcome Pooling
Thiazide_Diuretic
ARB
CCB
Beta_Blocker
ACE
0.07
0.08
0.09
0.10
0.11
0.12
Cumulative Incidence Rate at 1 Year After Baseline

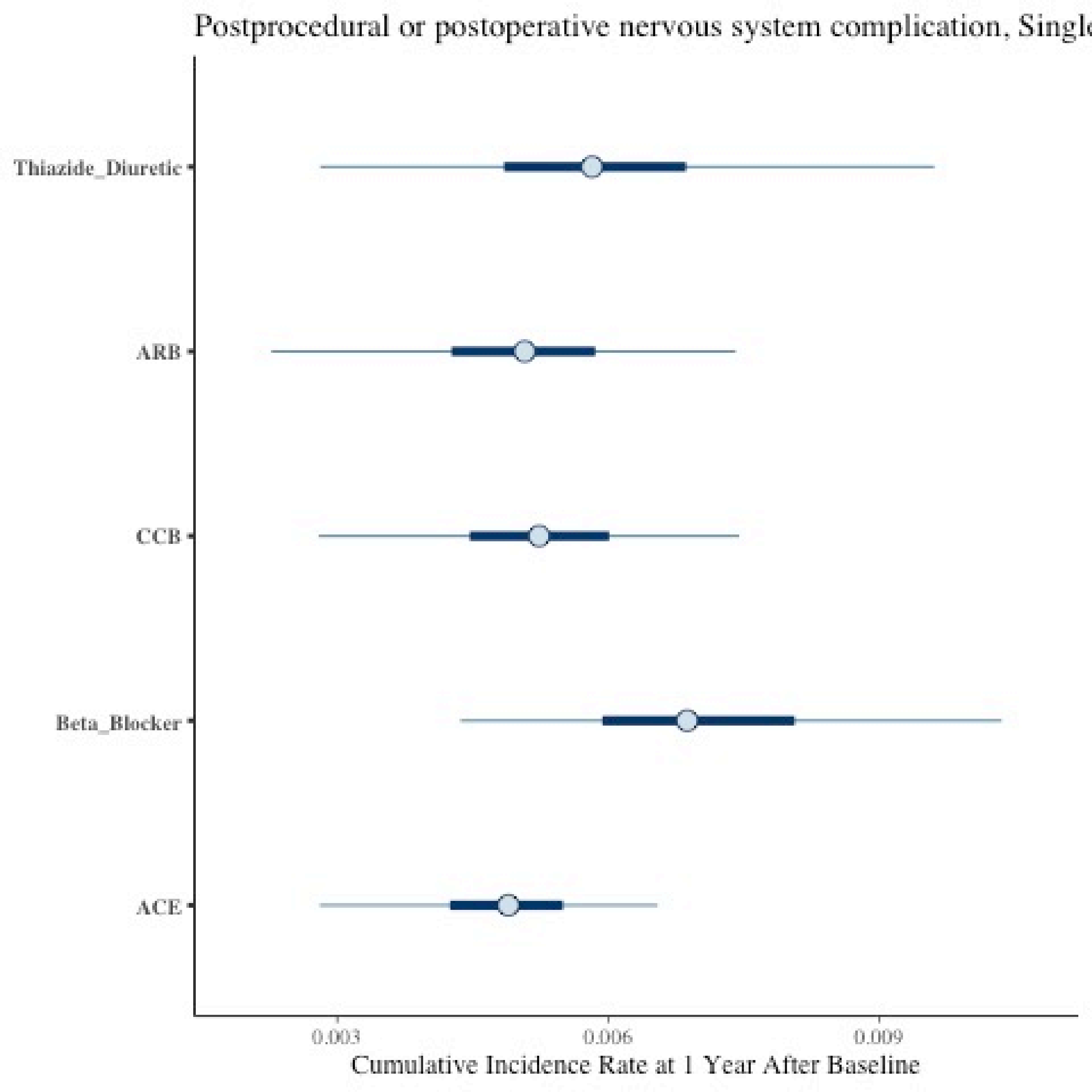

Postprocedural or postoperative nervous system complication, Single
Thiazide_Diuretic
ARB
CCB
Beta_Blocker
ACE
0.003
0.006
0.009
Cumulative Incidence Rate at 1 Year After Baseline

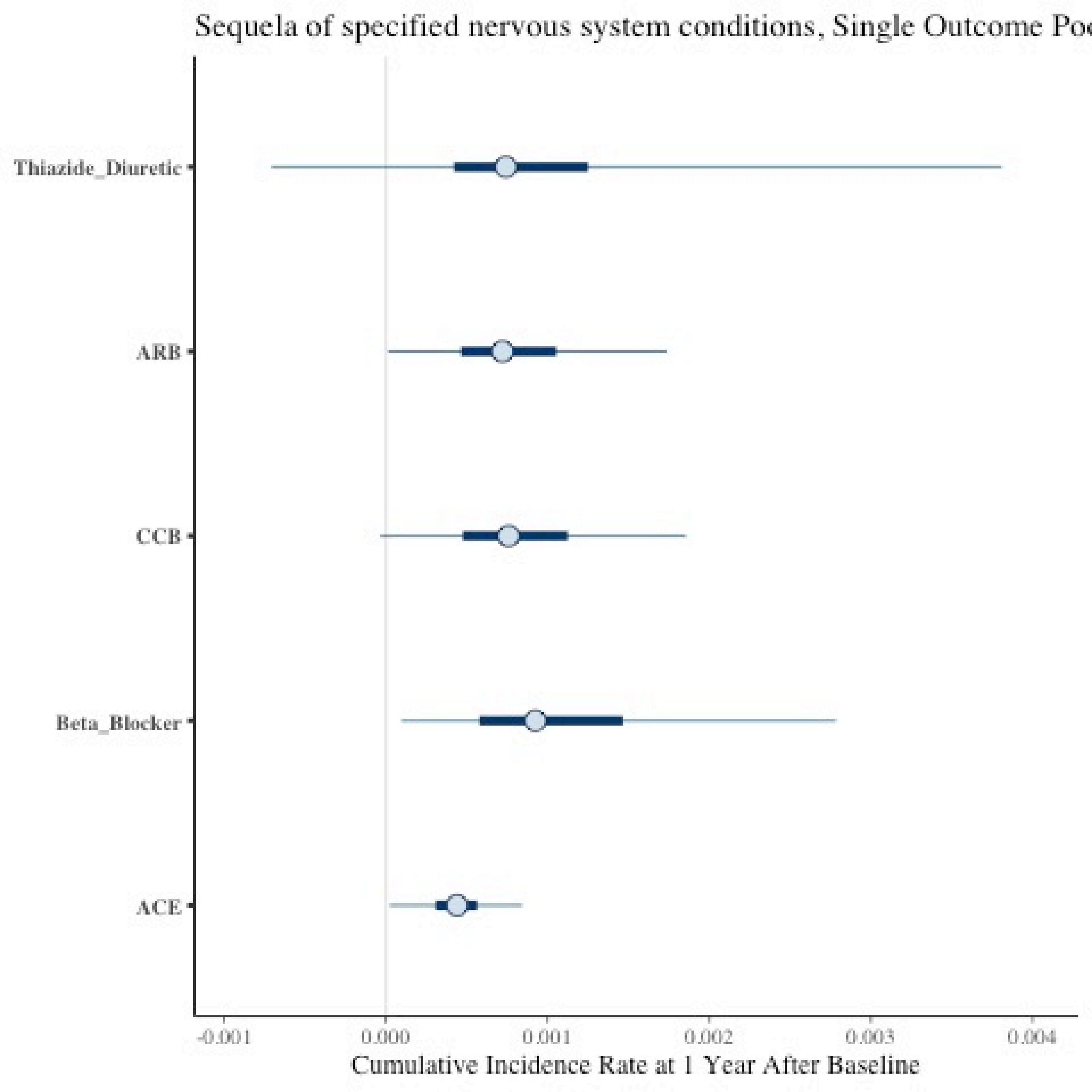

Sequela of specified nervous system conditions, Single Outcome Poo
Thiazide_Diuretic
ARB
CCB
Beta_Blocker
ACE
-0.001
0.000
0.001
0.002
0.003
0.004
Cumulative Incidence Rate at 1 Year After Baseline

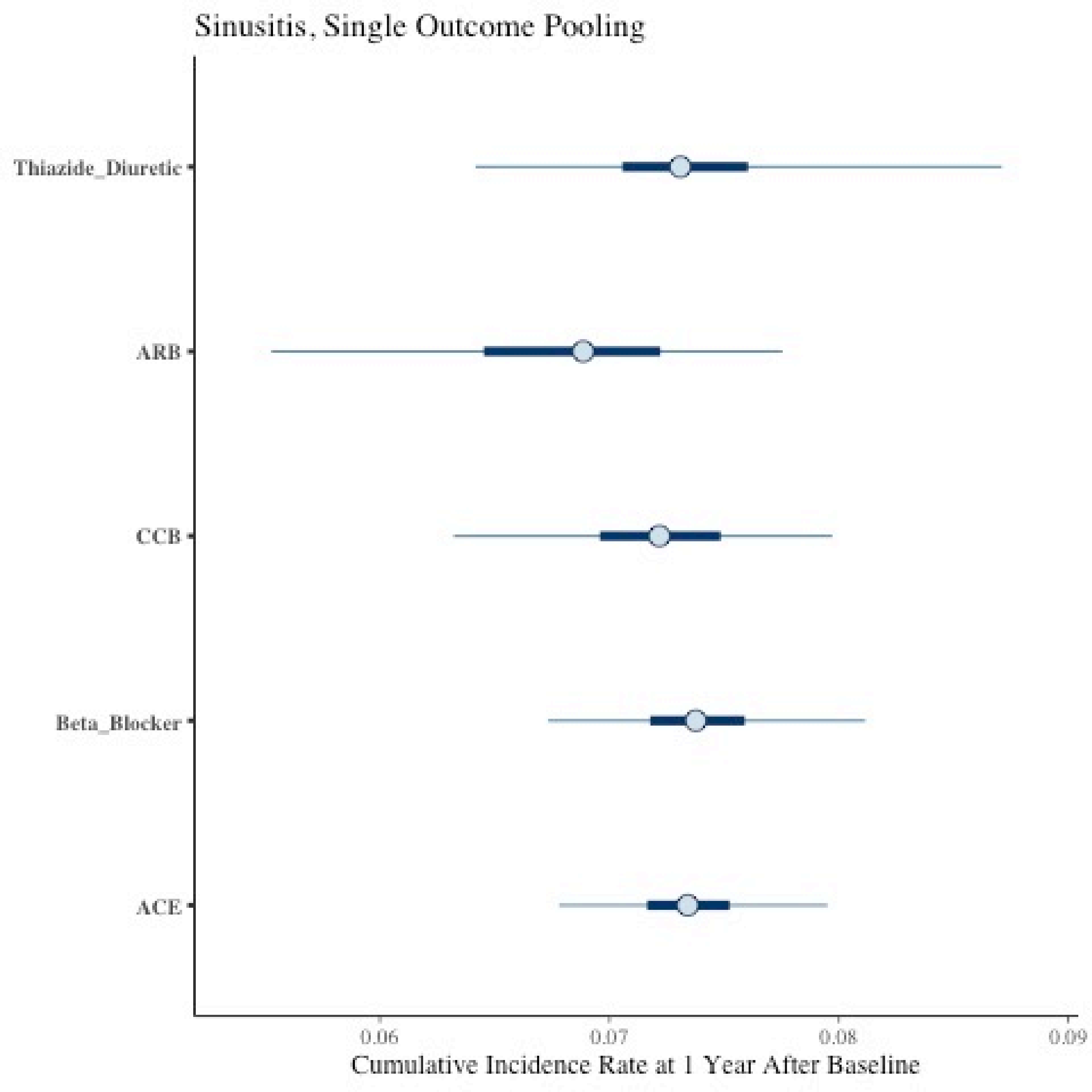

Sinusitis, Single Outcome Pooling
Thiazide_Diuretic
ARB
CCB
Beta_Blocker
ACE
0.06
0.07
0.08
0.09
Cumulative Incidence Rate at 1 Year After Baseline

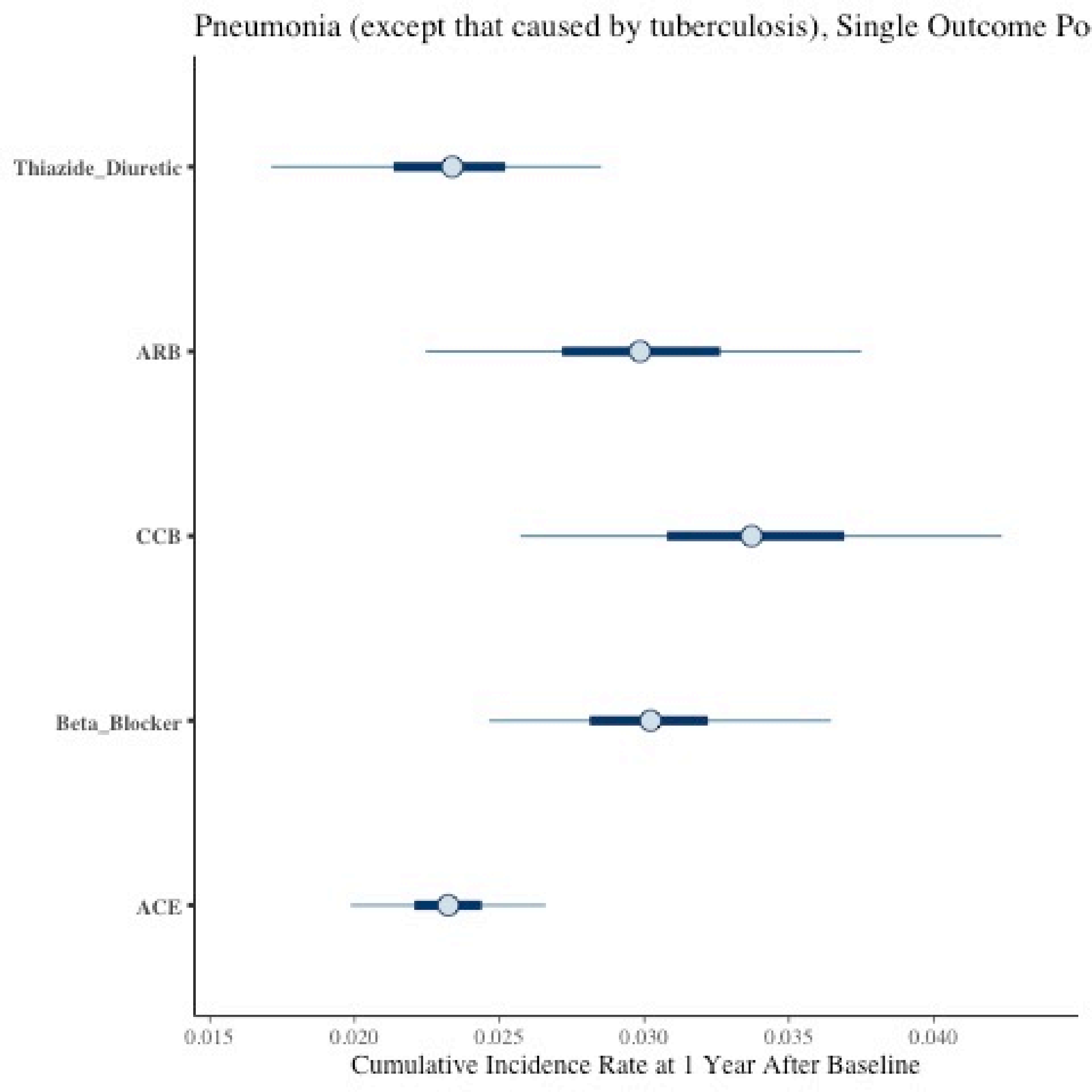
Pneumonia (except that caused by tuberculosis), Single Outcome Po
Thiazide_Diuretic
ARB
CCB
Beta_Blocker
ACE
0.015
0.020
0.025
0.030
0.035
0.040
Cumulative Incidence Rate at 1 Year After Baseline

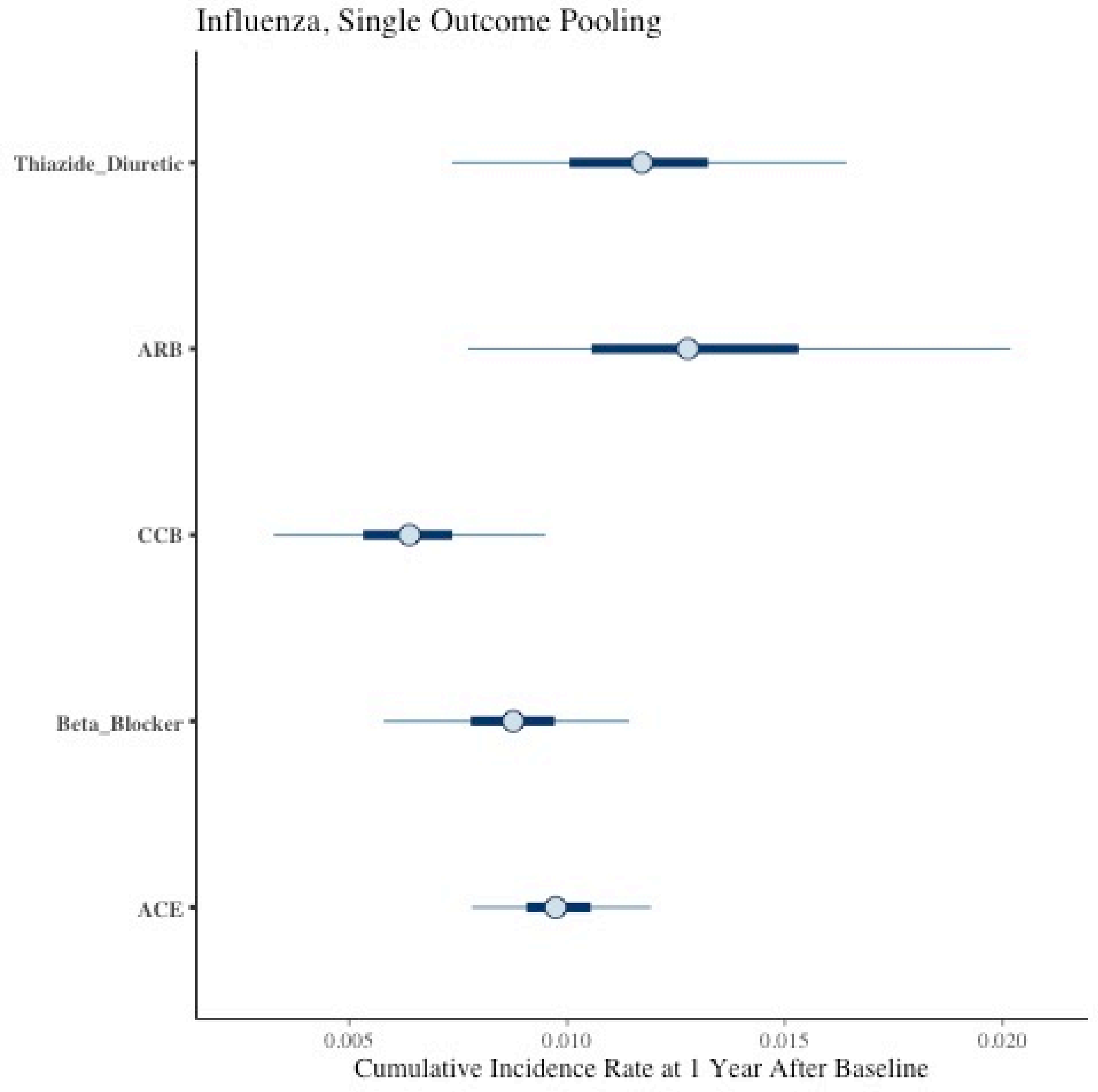

Influenza, Single Outcome Pooling
Thiazide_Diuretic
ARB
CCB
Beta_Blocker
ACE
0.005
0.010
0.015
0.020
Cumulative Incidence Rate at 1 Year After Baseline

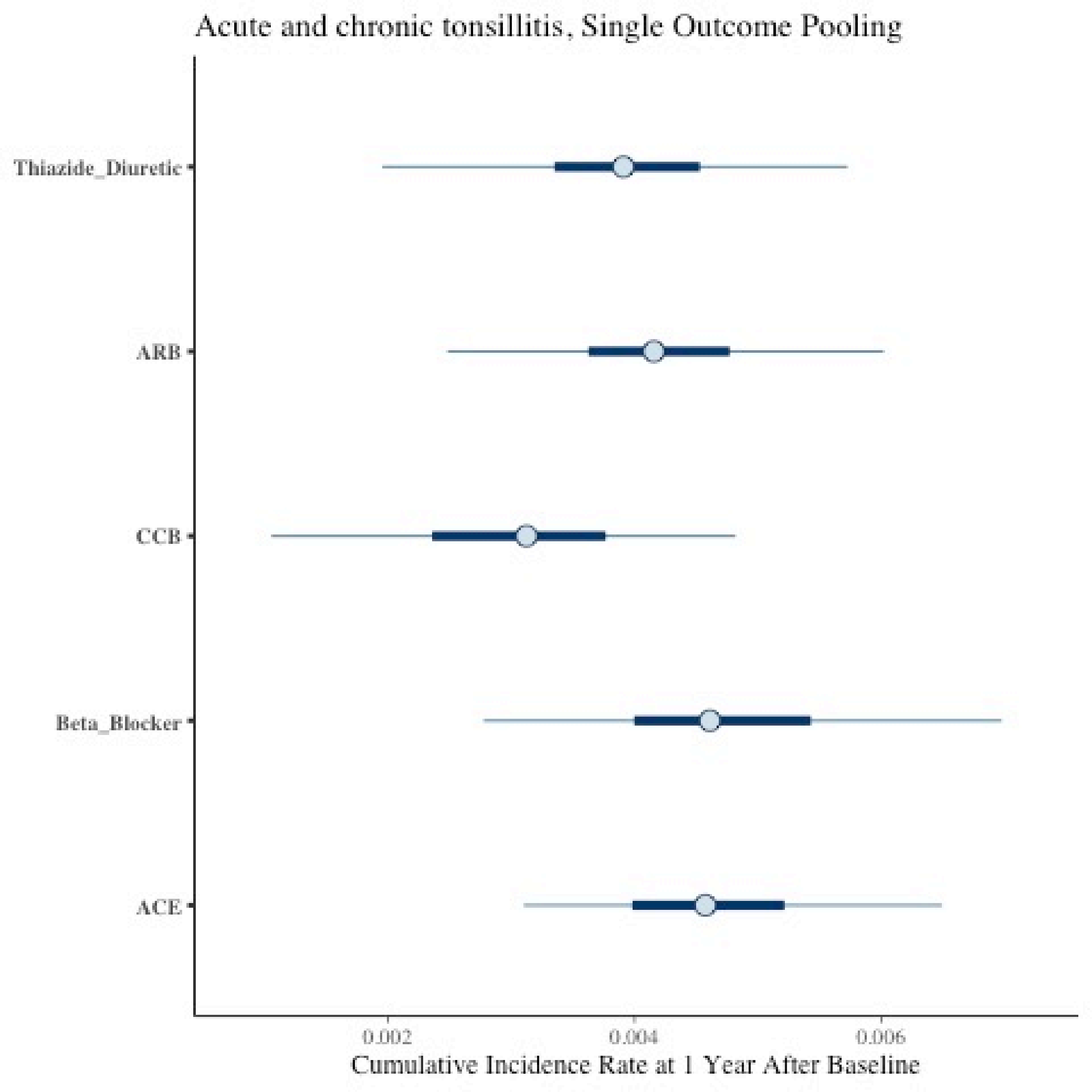
Acute and chronic tonsillitis, Single Outcome Pooling
Thiazide_Diuretic
ARB
CCB
Beta_Blocker
ACE
0.002
0.004
0.006
Cumulative Incidence Rate at 1 Year After Baseline

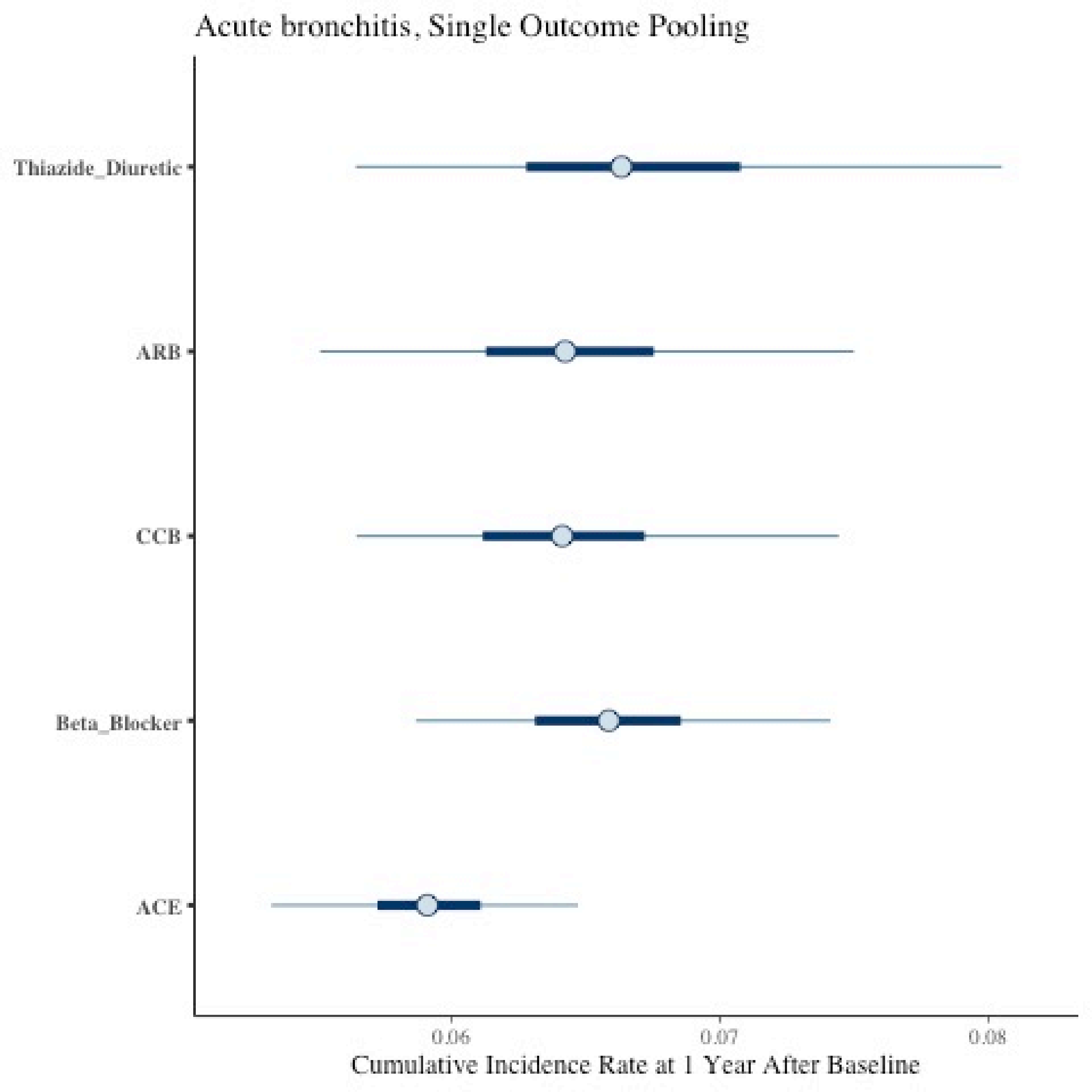

Acute bronchitis, Single Outcome Pooling
Thiazide_Diuretic
ARB
CCB
Beta_Blocker
ACE
0.06
0.07
0.08
Cumulative Incidence Rate at 1 Year After Baseline

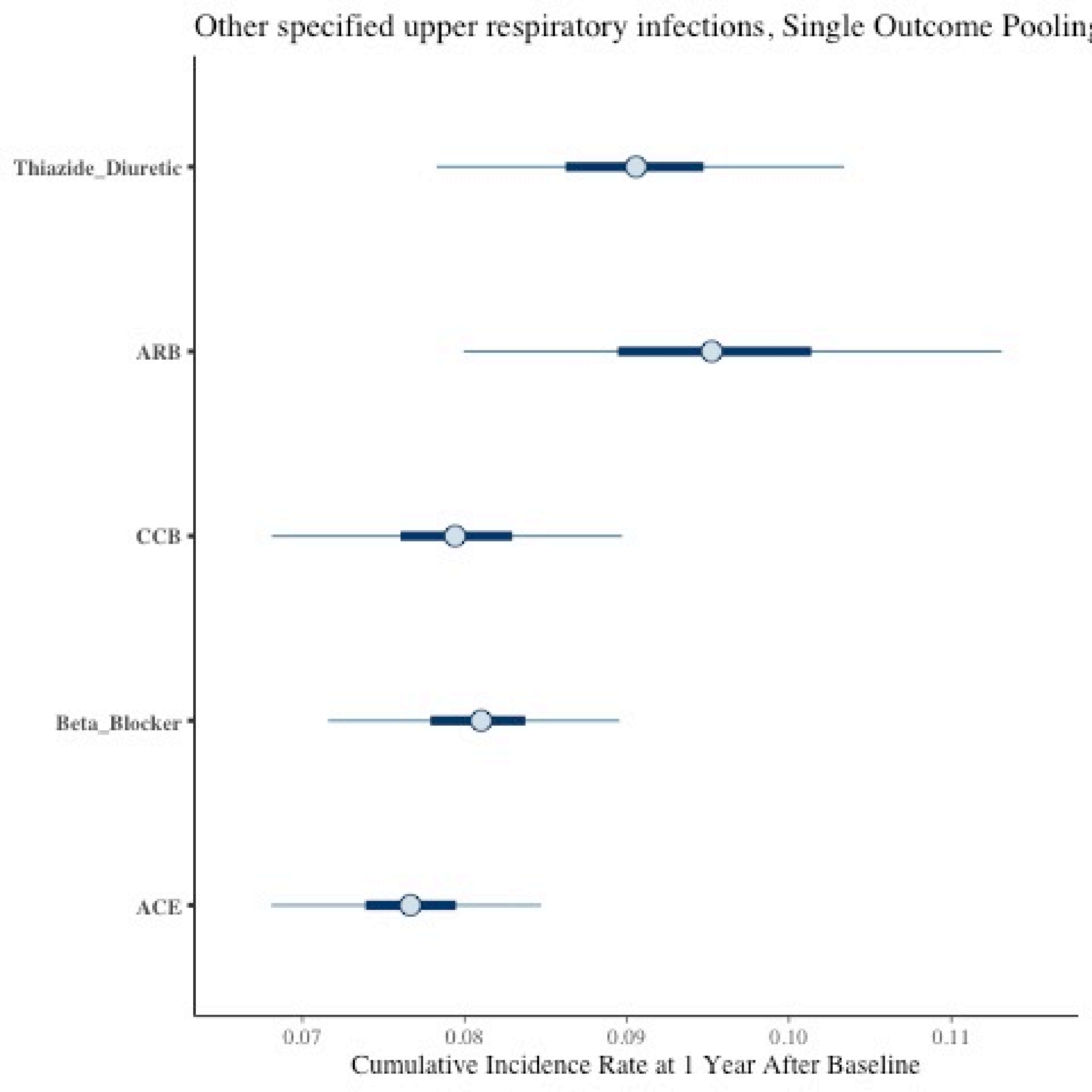

Other specified upper respiratory infections, Single Outcome Poolin
Thiazide_Diuretic
ARB
CCB
Beta_Blocker
ACE
0.07
0.08
0.09
0.10
0.11
Cumulative Incidence Rate at 1 Year After Baseline

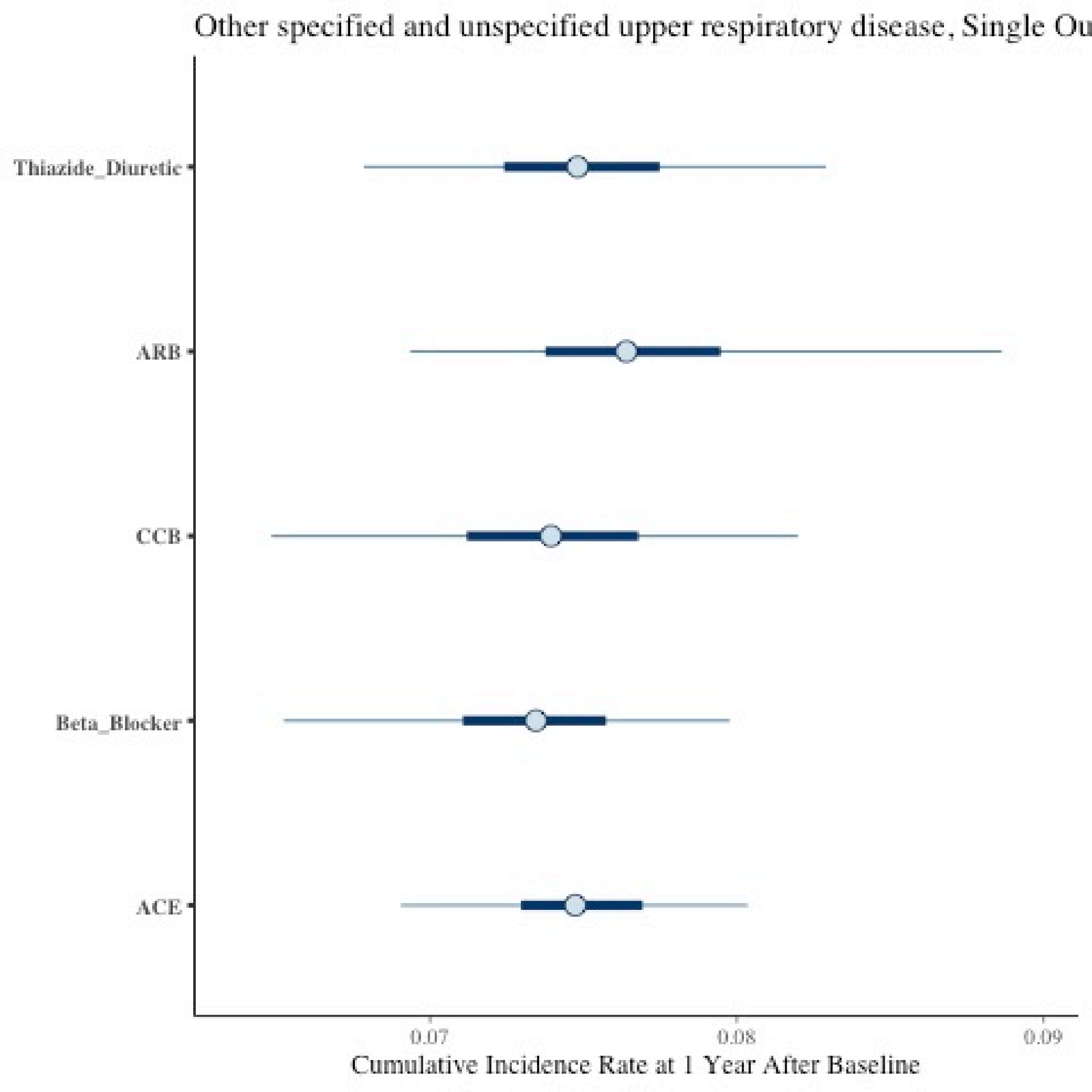

Other specified and unspecified upper respiratory disease, Single Ou
Thiazide_Diuretic
ARB
CCB
Beta_Blocker
ACE
0.07
0.08
0.09
Cumulative Incidence Rate at 1 Year After Baseline

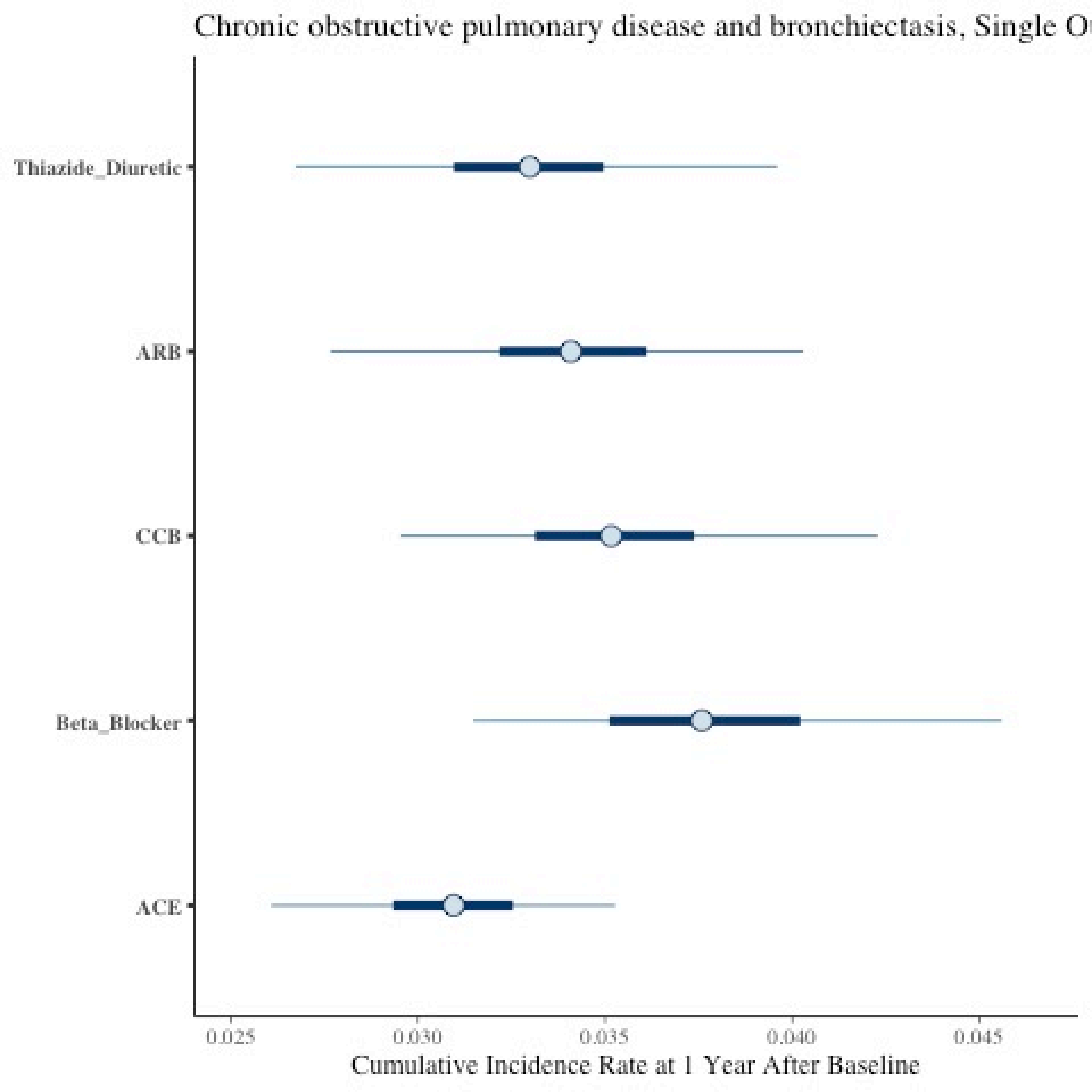

Chronic obstructive pulmonary disease and bronchiectasis, Single O
Thiazide_Diuretic
ARB
CCB
Beta_Blocker
ACE
0.025
0.030
0.035
0.040
0.045
Cumulative Incidence Rate at 1 Year After Baseline

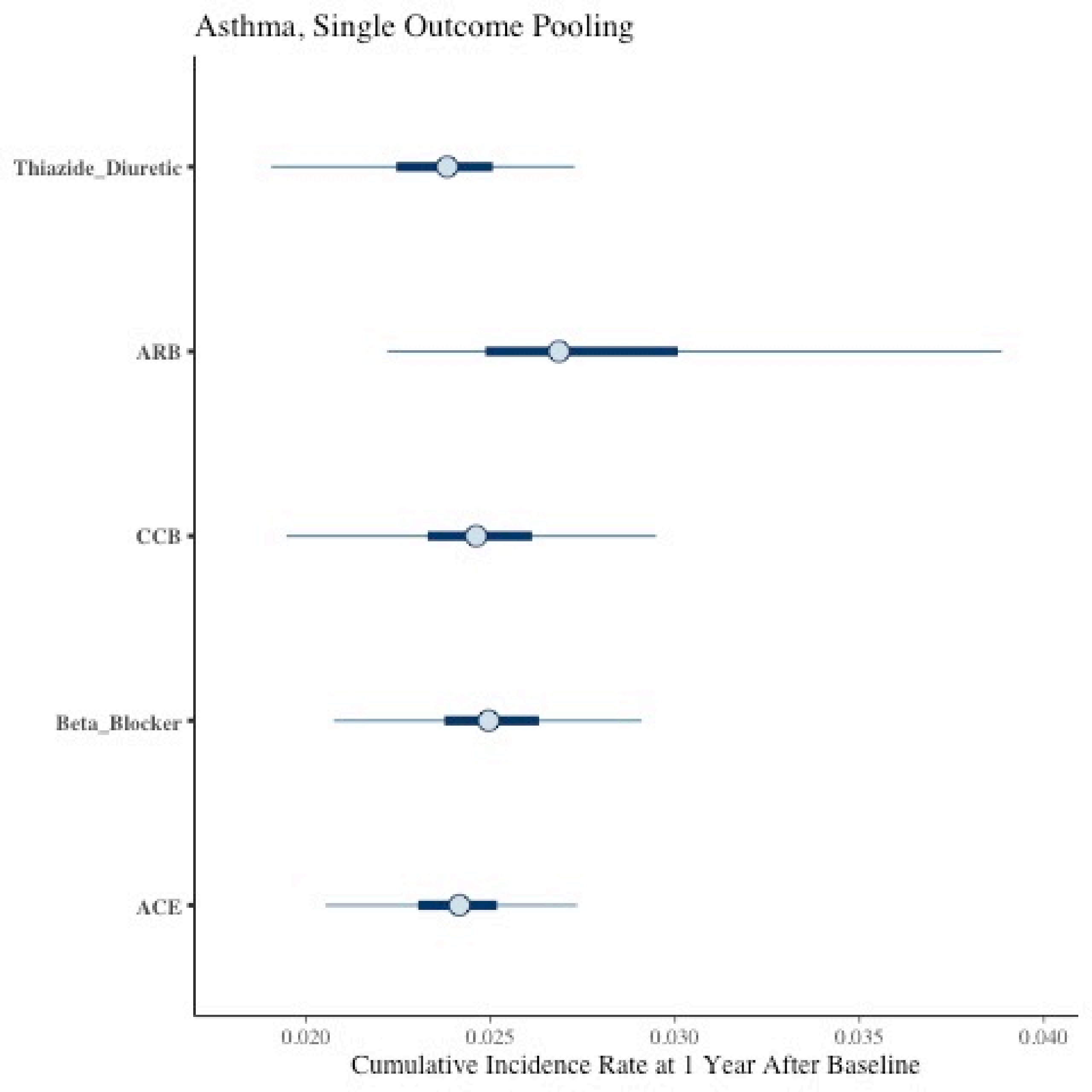
Asthma, Single Outcome Pooling
Thiazide_Diuretic
ARB
CCB
Beta_Blocker
ACE
0.020
0.025
0.030
0.035
0.040
Cumulative Incidence Rate at 1 Year After Baseline

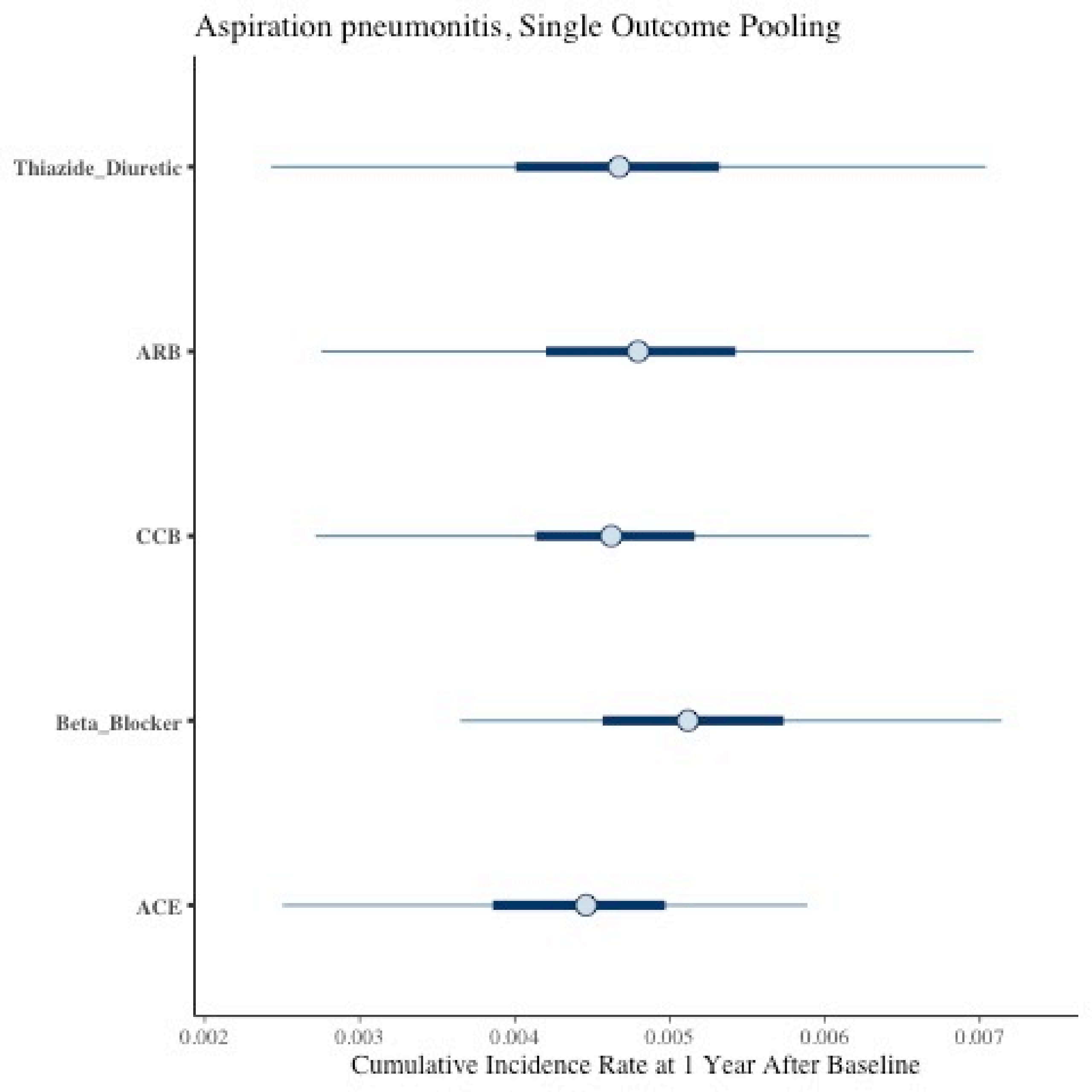

Aspiration pneumonitis, Single Outcome Pooling
Thiazide_Diuretic
ARB
CCB
Beta_Blocker
ACE
0.002
0.003
0.004
0.005
0.006
0.007
Cumulative Incidence Rate at 1 Year After Baseline

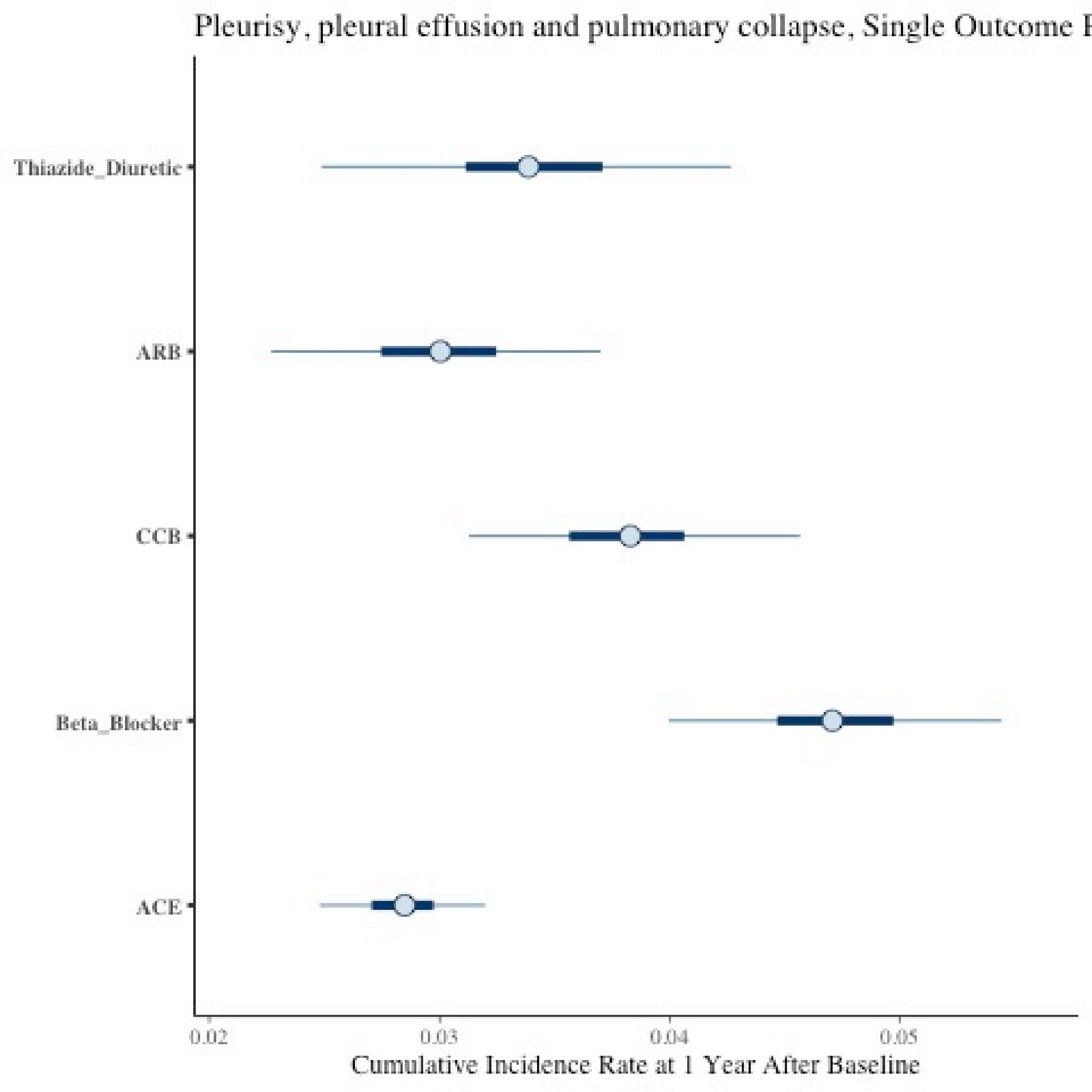

Pleurisy, pleural effusion and pulmonary collapse, Single Outcome F
Thiazide_Diuretic
ARB
CCB
Beta_Blocker
ACE
0.02
0.03
0.04
0.05
Cumulative Incidence Rate at 1 Year After Baseline

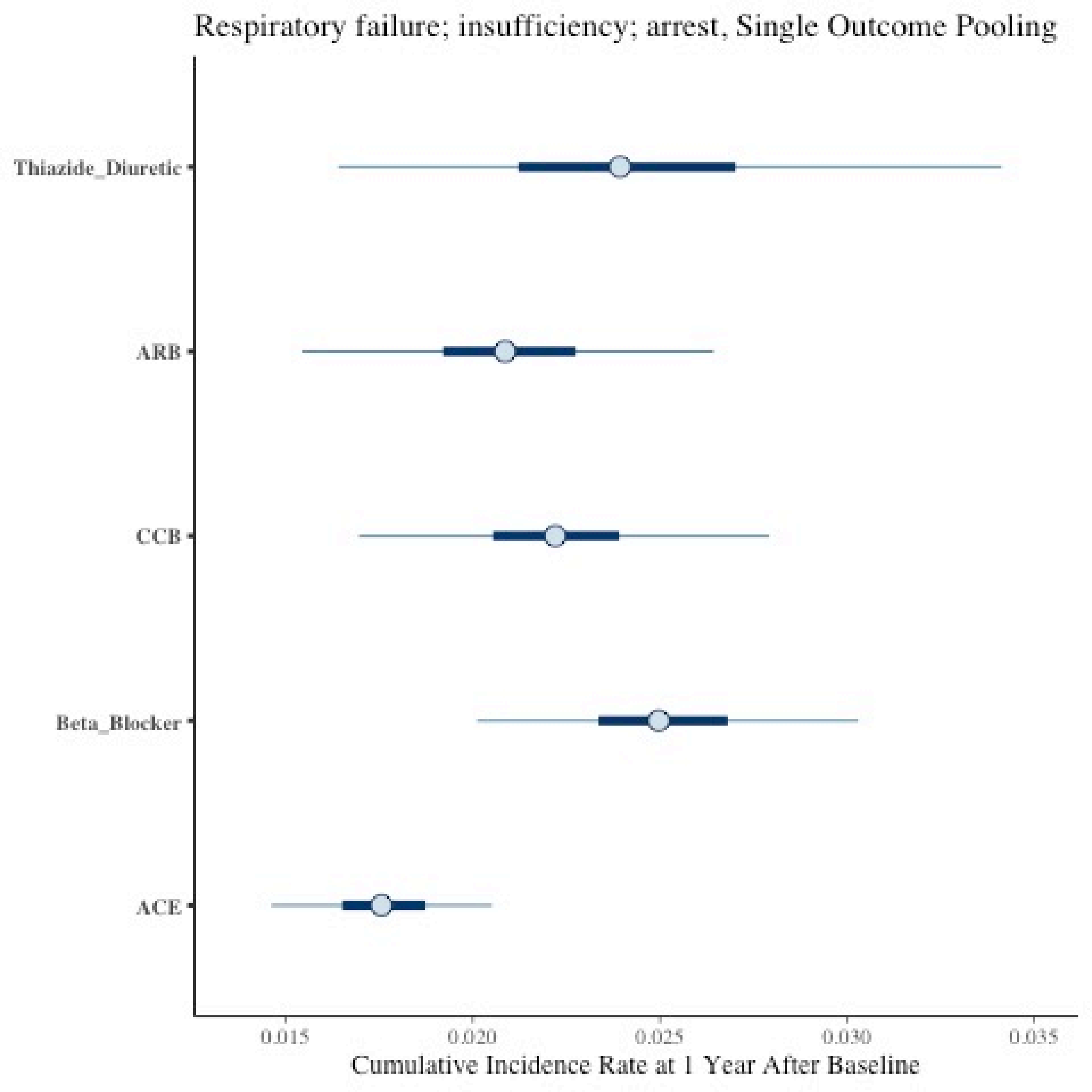

Respiratory failure; insufficiency; arrest, Single Outcome Pooling
Thiazide_Diuretic
ARB
CCB
Beta_Blocker
ACE
0.015
0.020
0.025
0.030
0.035
Cumulative Incidence Rate at 1 Year After Baseline

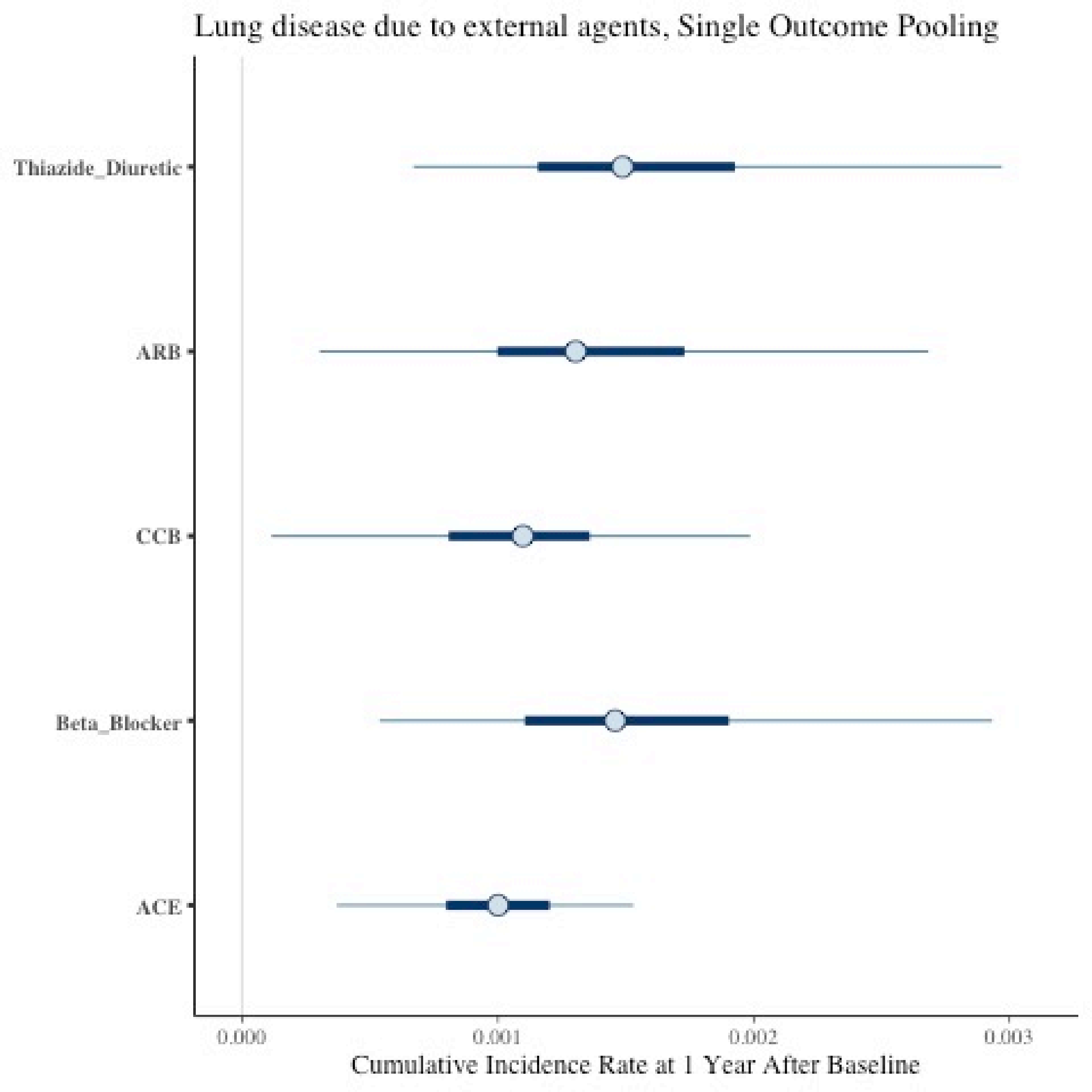

Lung disease due to external agents, Single Outcome Pooling
Thiazide_Diuretic
ARB
CCB
Beta_Blocker
ACE
0.000
0.001
0.002
0.003
Cumulative Incidence Rate at 1 Year After Baseline

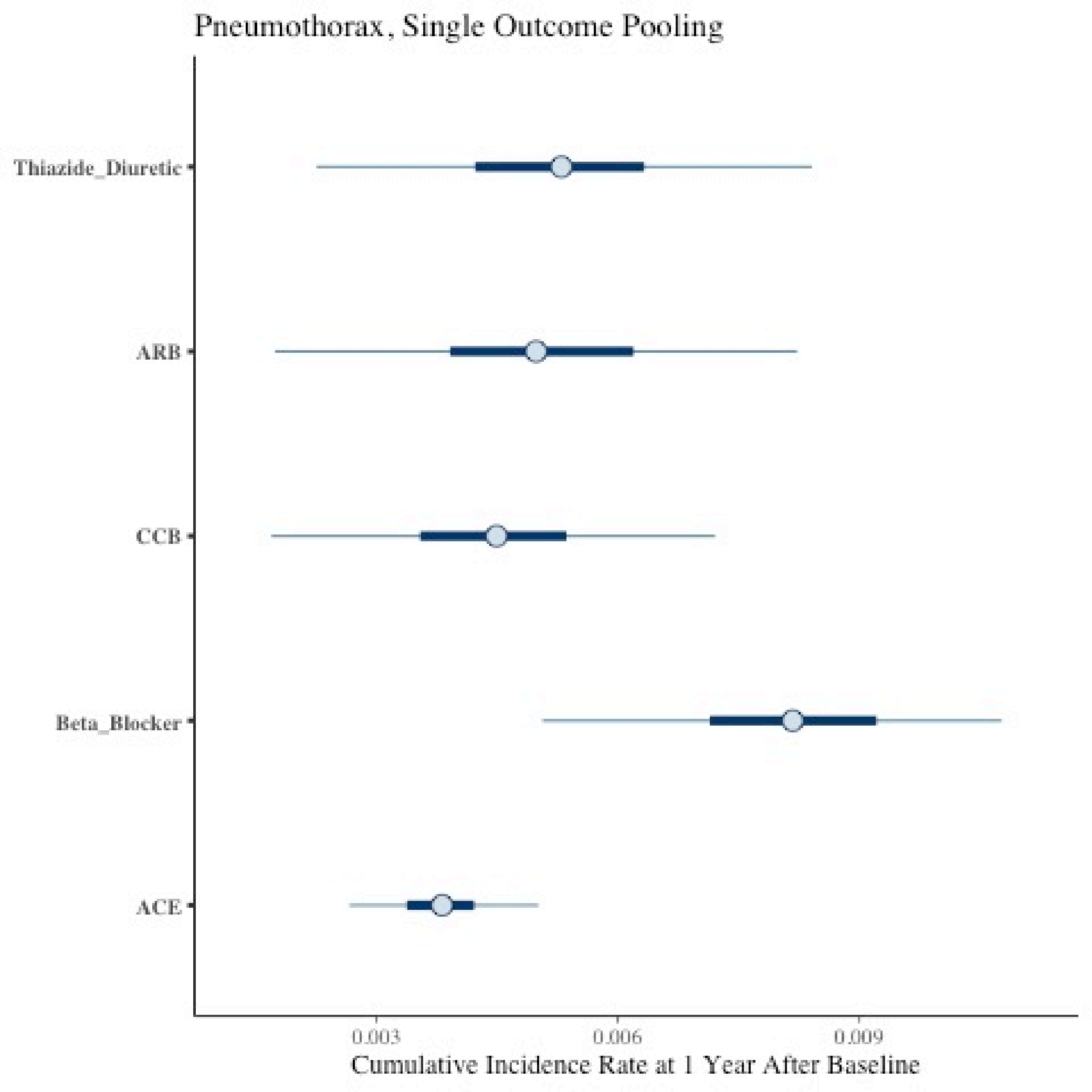
Pneumothorax, Single Outcome Pooling
Thiazide_Diuretic
ARB
CCB
Beta_Blocker
ACE
0.003
0.006
0.009
Cumulative Incidence Rate at 1 Year After Baseline

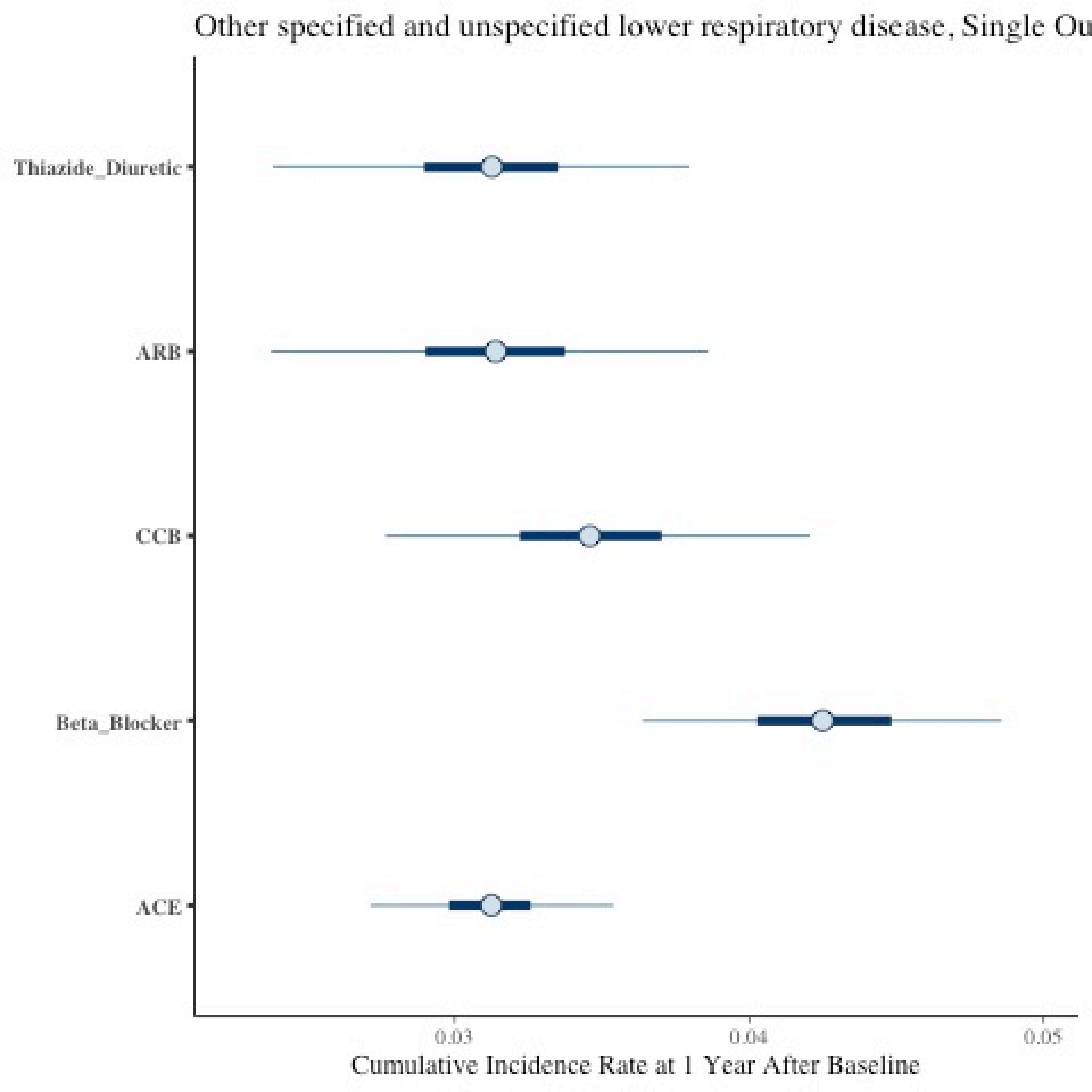
Other specified and unspecified lower respiratory disease, Single Ou
Thiazide_Diuretic
ARB
CCB
Beta_Blocker
ACE
0.03
0.04
0.05
Cumulative Incidence Rate at 1 Year After Baseline

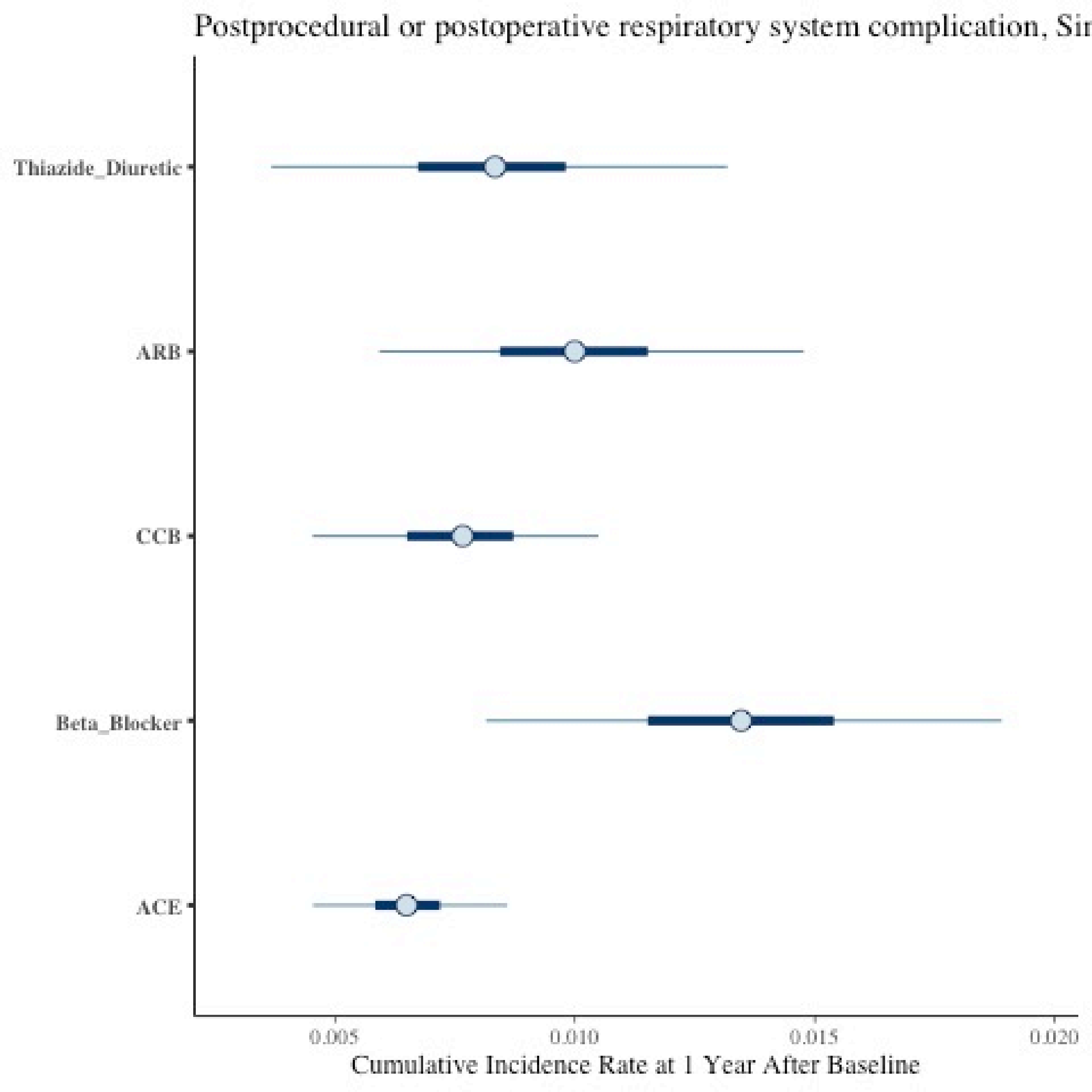
Postprocedural or postoperative respiratory system complication, Si
Thiazide_Diuretic
ARB
CCB
Beta_Blocker
ACE
0.005
0.010
0.015
0.020
Cumulative Incidence Rate at 1 Year After Baseline

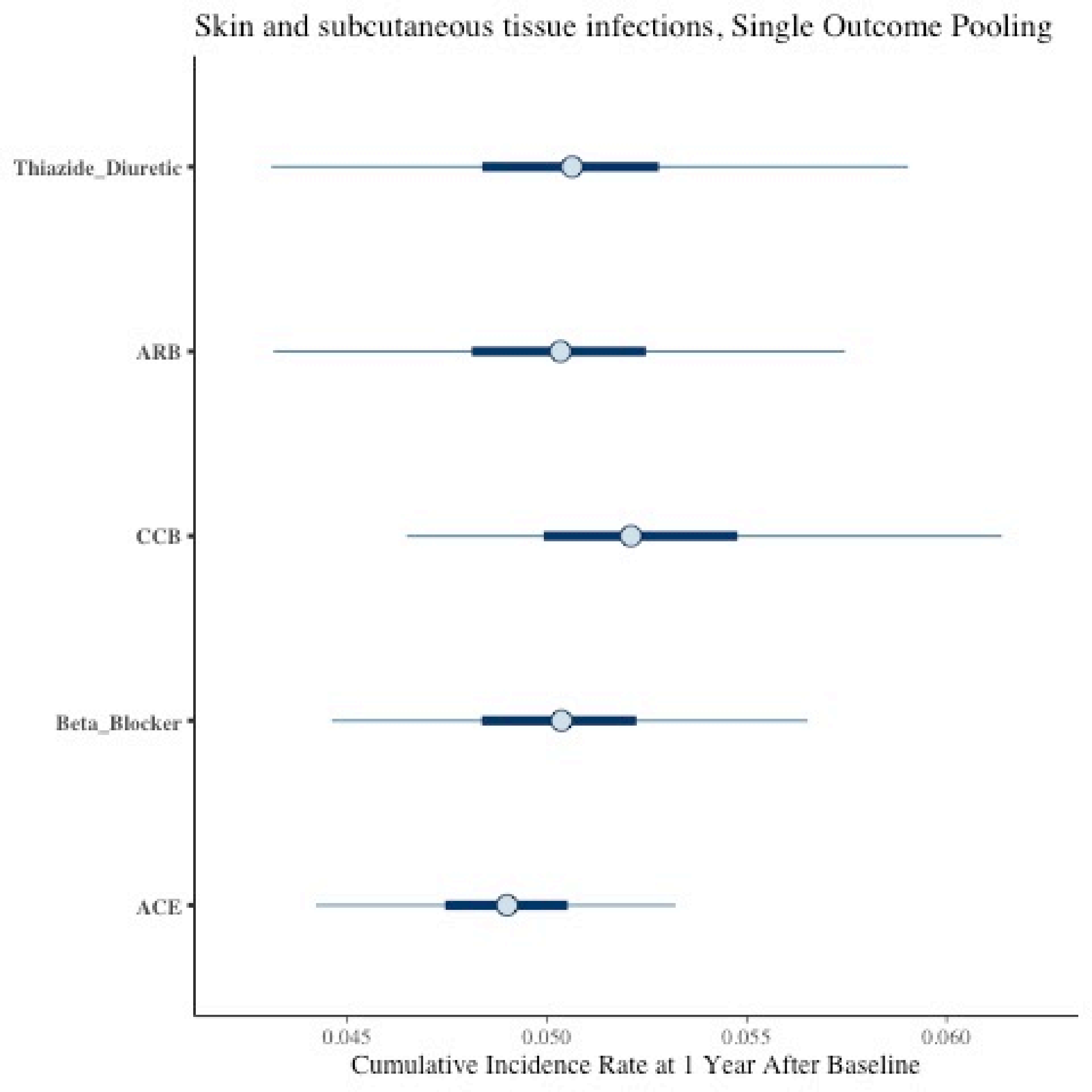

Skin and subcutaneous tissue infections, Single Outcome Pooling
Thiazide_Diuretic
ARB
CCB
Beta_Blocker
ACE
0.045
0.050
0.055
0.060
Cumulative Incidence Rate at 1 Year After Baseline

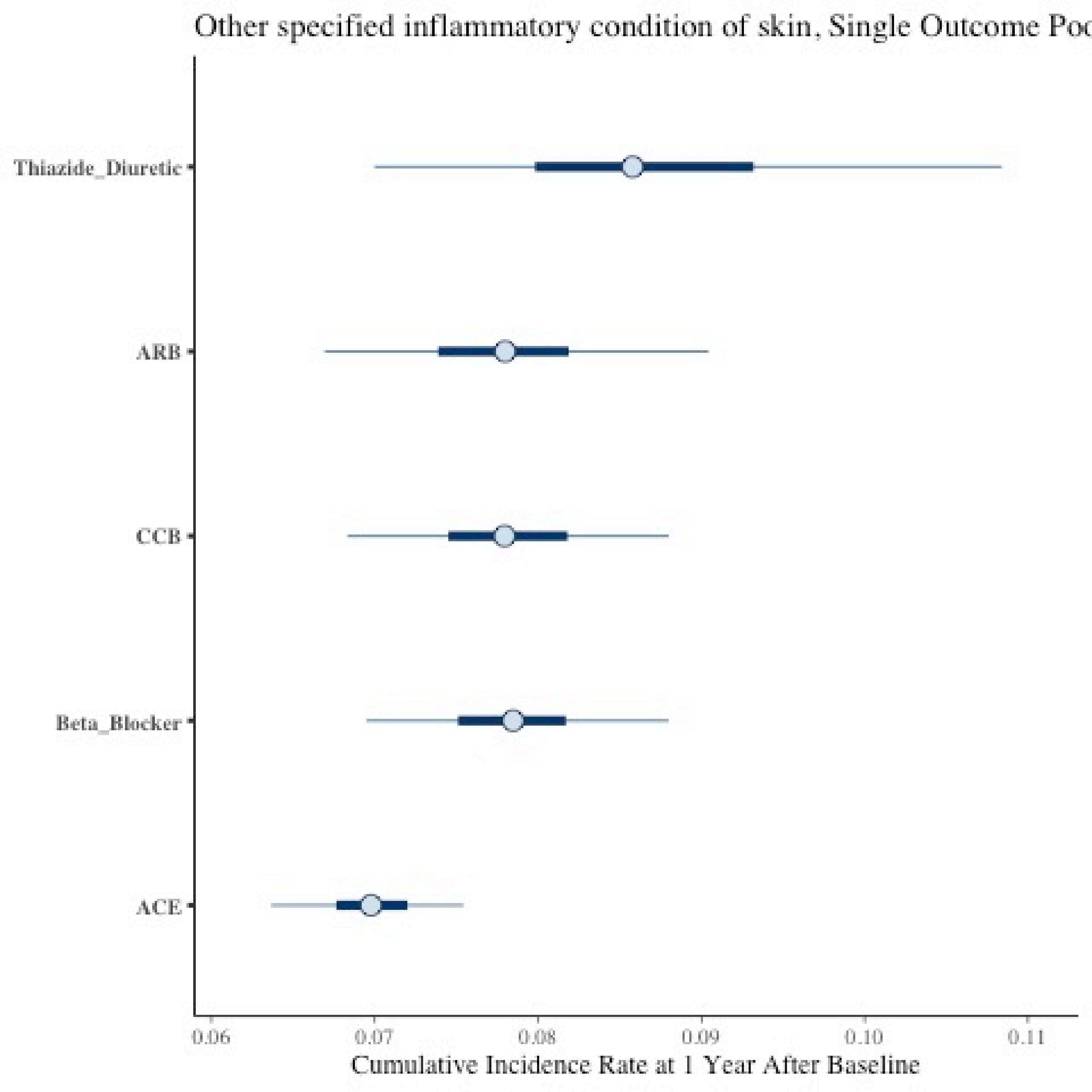

Other specified inflammatory condition of skin, Single Outcome Poo
Thiazide_Diuretic
ARB
CCB
Beta_Blocker
ACE
0.06
0.07
0.08
0.09
0.10
0.11
Cumulative Incidence Rate at 1 Year After Baseline

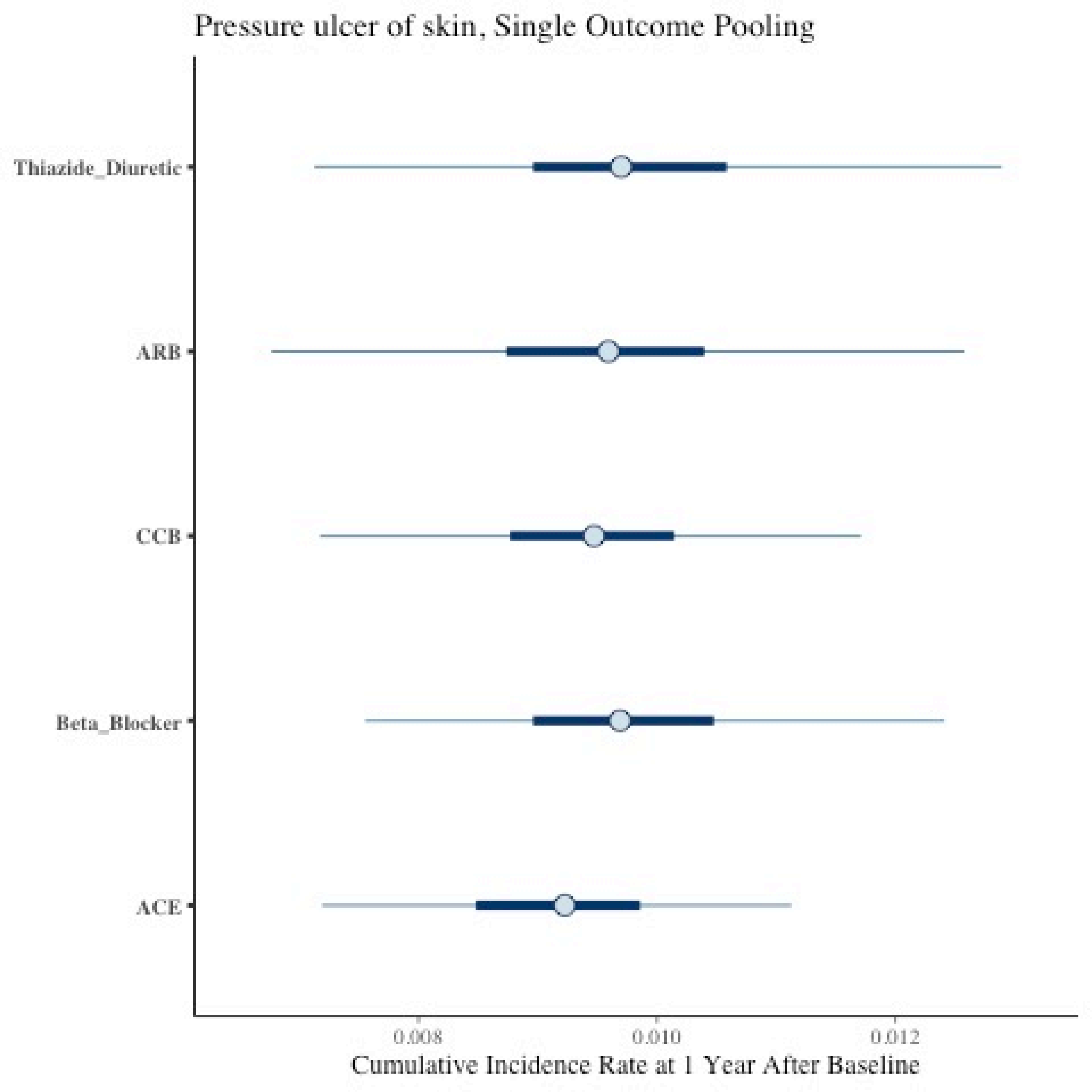
Pressure ulcer of skin, Single Outcome Pooling
Thiazide_Diuretic
ARB
CCB
Beta_Blocker
ACE
0.008
0.010
0.012
Cumulative Incidence Rate at 1 Year After Baseline

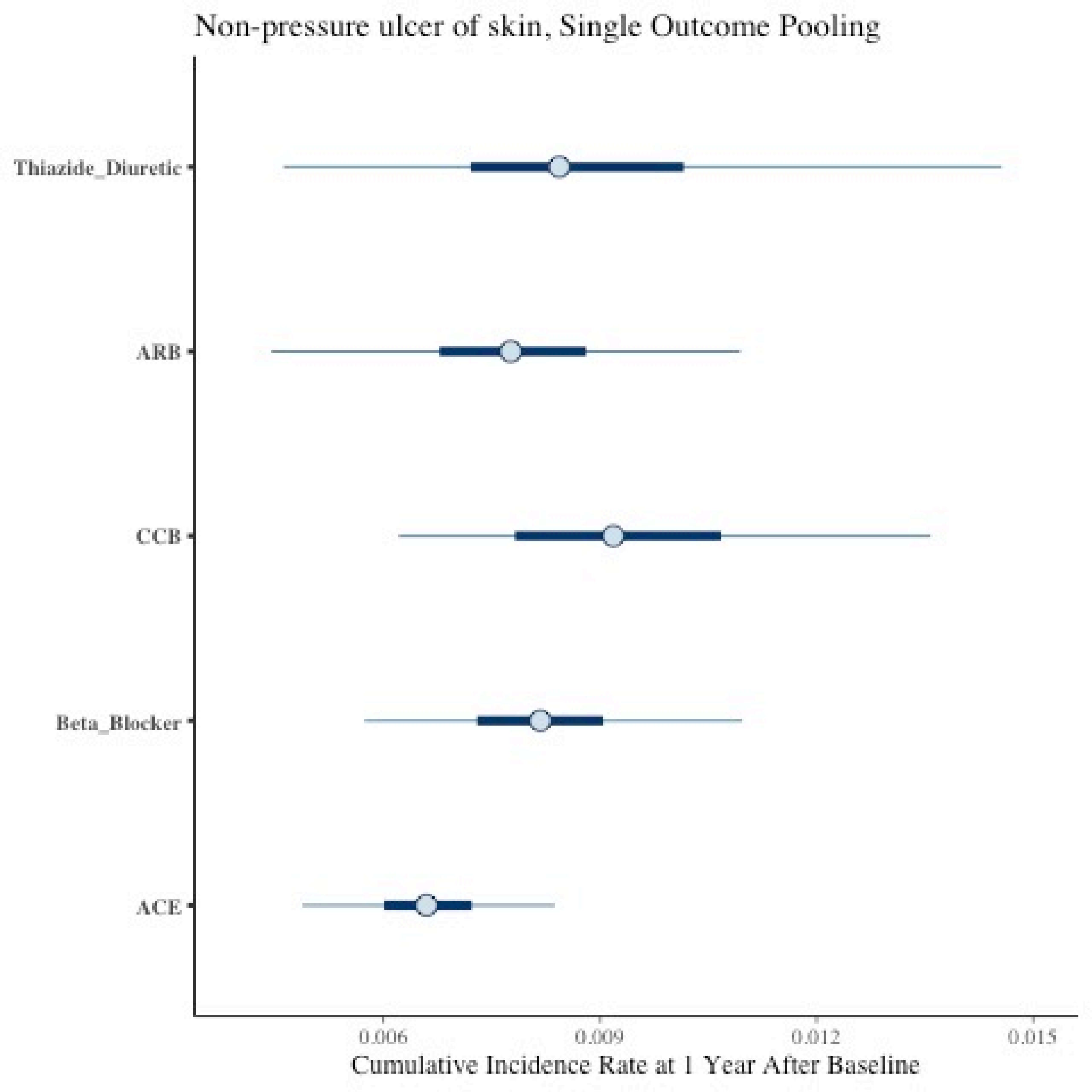

Non-pressure ulcer of skin, Single Outcome Pooling
Thiazide_Diuretic
ARB
CCB
Beta_Blocker
ACE
0.006
0.009
0.012
0.015
Cumulative Incidence Rate at 1 Year After Baseline

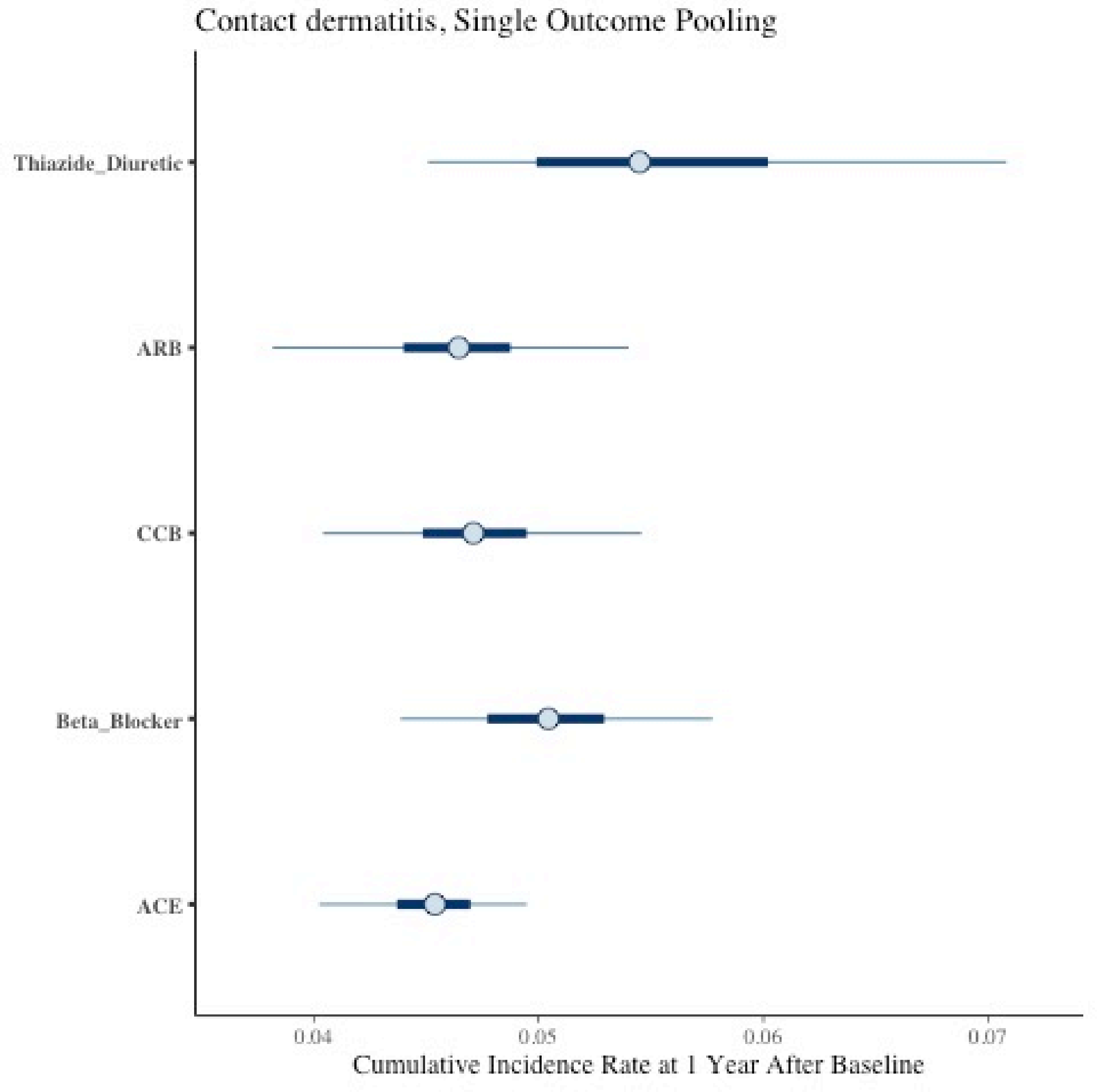
Contact dermatitis, Single Outcome Pooling
Thiazide_Diuretic
ARB
CCB
Beta_Blocker
ACE
0.04
0.05
0.06
0.07
Cumulative Incidence Rate at 1 Year After Baseline

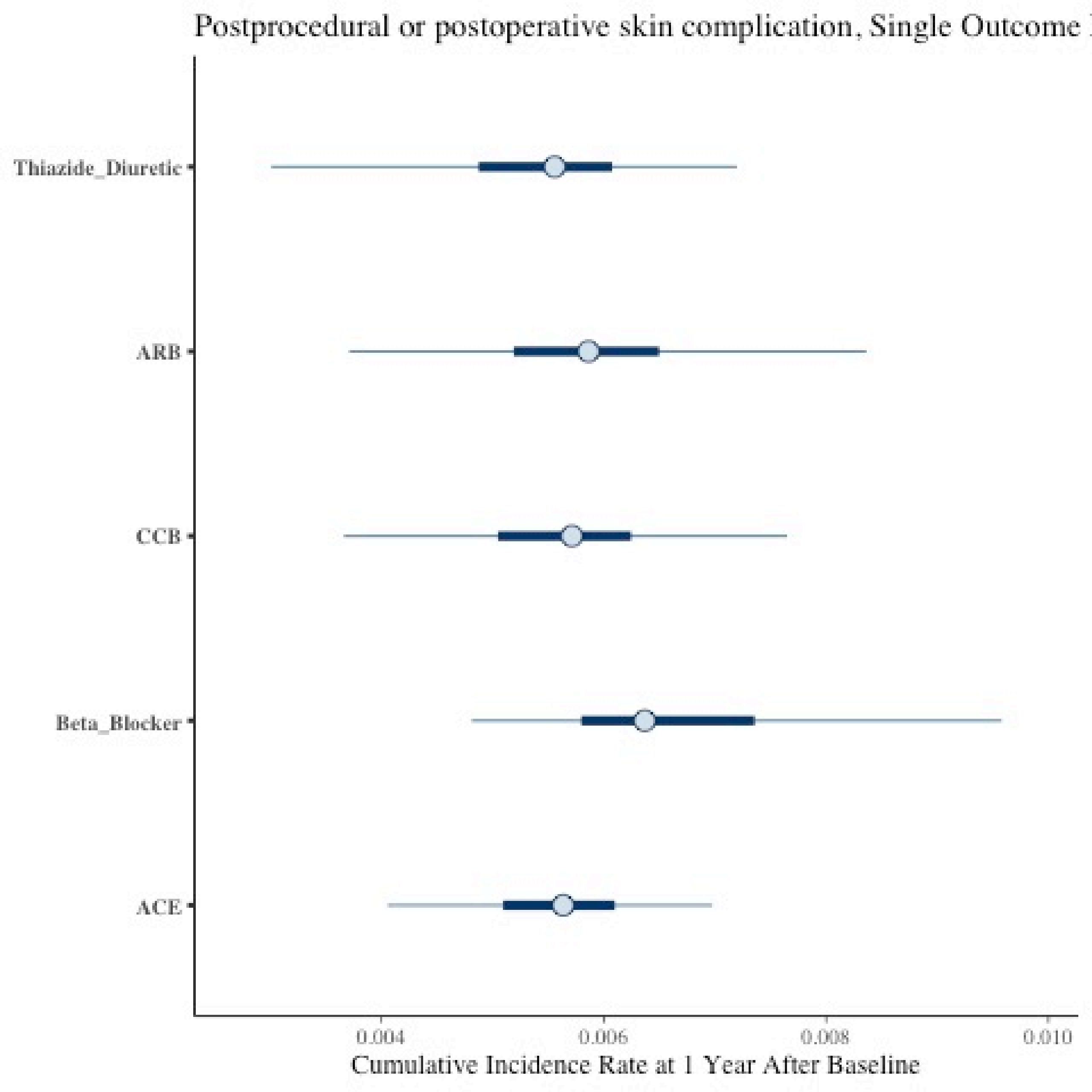

Postprocedural or postoperative skin complication, Single Outcome
Thiazide_Diuretic
ARB
CCB
Beta_Blocker
ACE
0.004
0.006
0.008
0.010
Cumulative Incidence Rate at 1 Year After Baseline

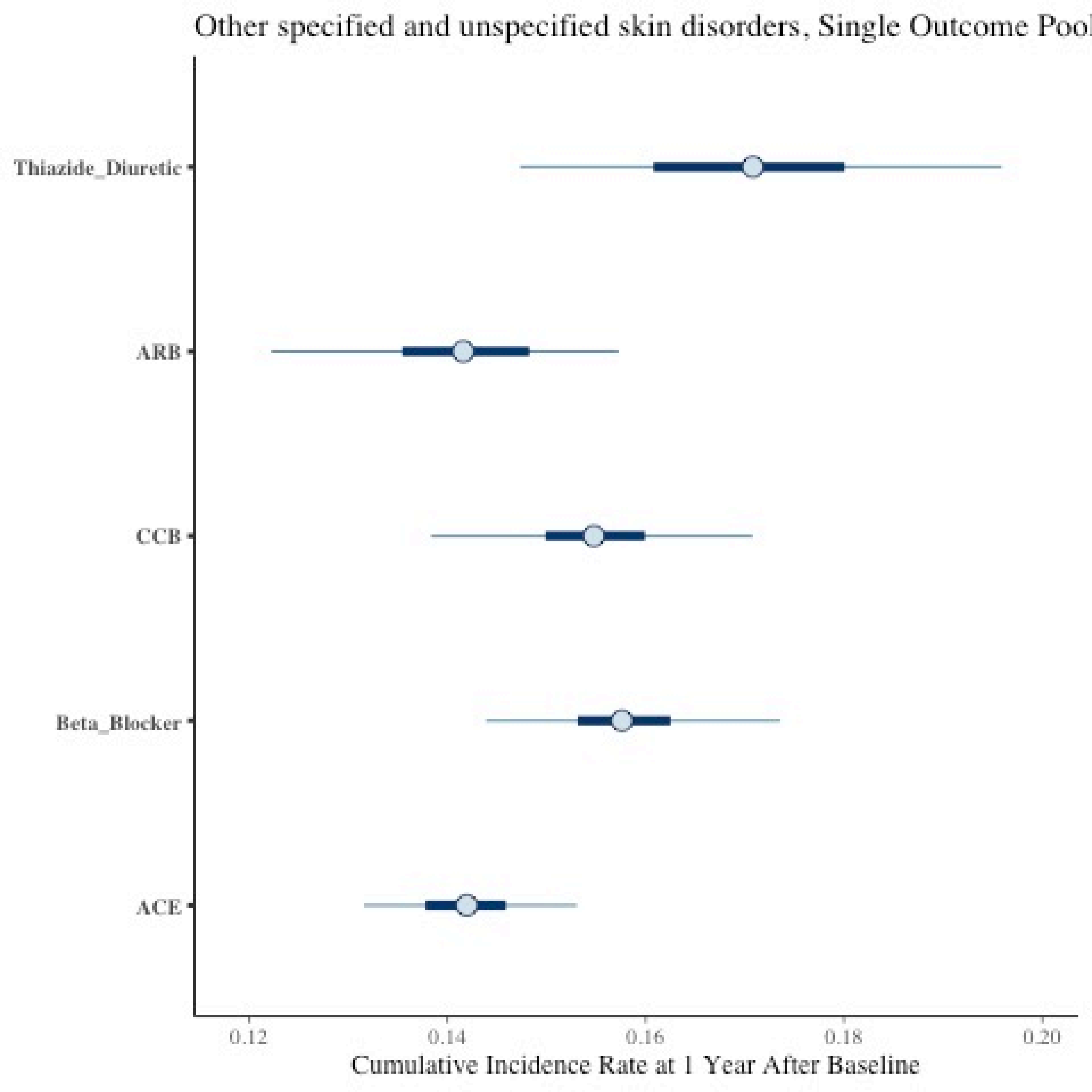

Other specified and unspecified skin disorders, Single Outcome Pool
Thiazide_Diuretic
ARB
CCB
Beta_Blocker
ACE
0.12
0.14
0.16
0.18
0.20
Cumulative Incidence Rate at 1 Year After Baseline

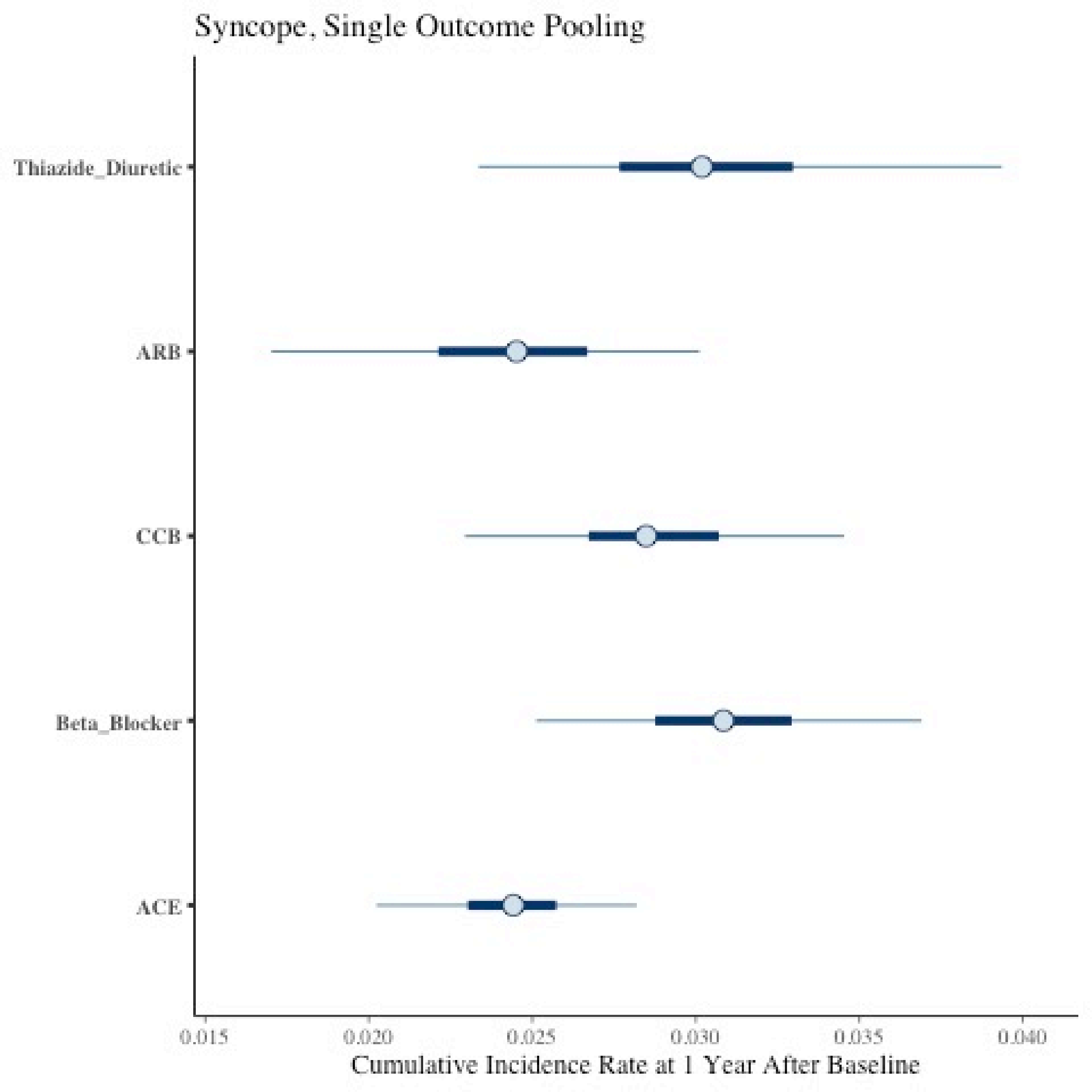

Syncope, Single Outcome Pooling
Thiazide_Diuretic
ARB
CCB
Beta_Blocker
ACE
0.015
0.020
0.025
0.030
0.035
0.040
Cumulative Incidence Rate at 1 Year After Baseline

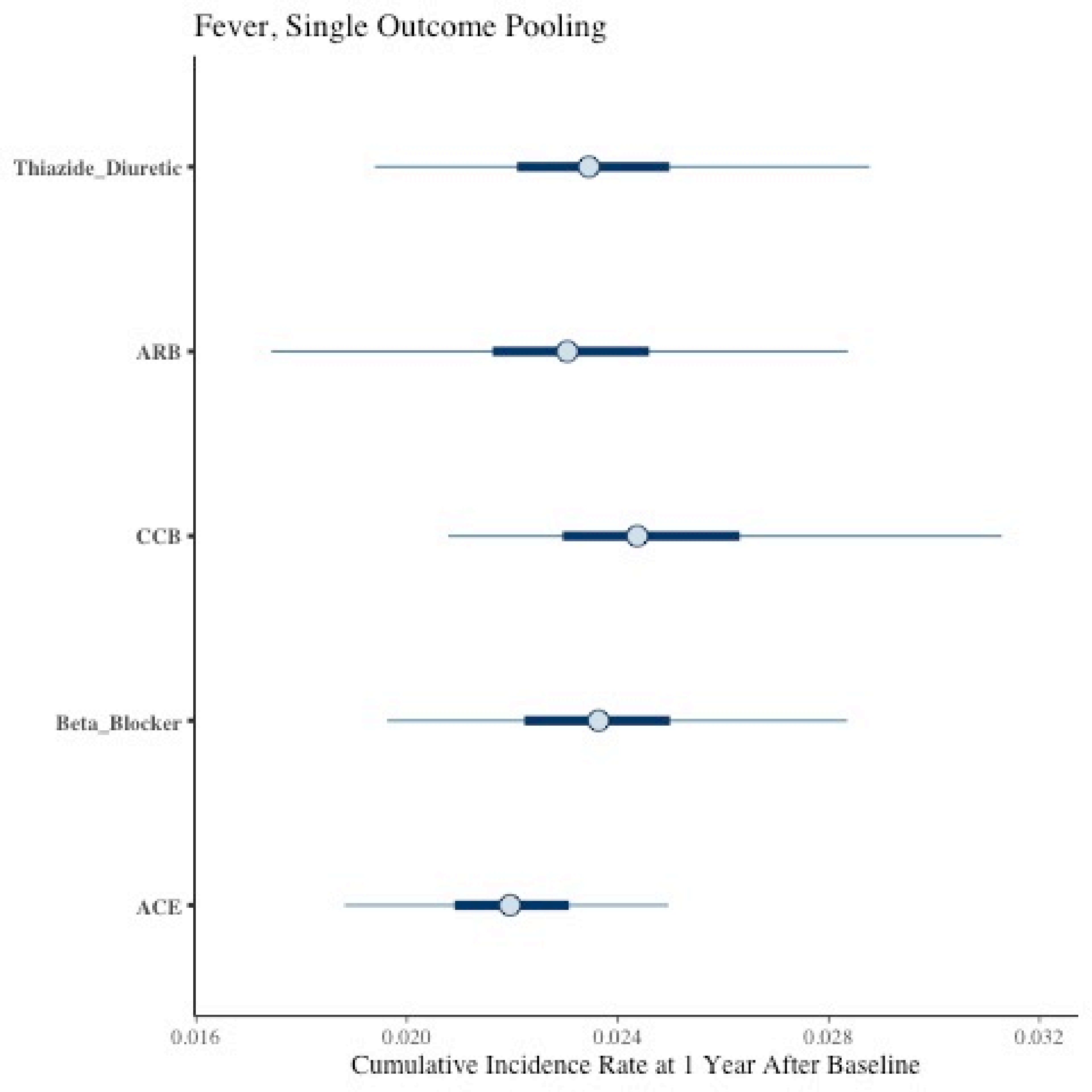
Fever, Single Outcome Pooling
Thiazide_Diuretic
ARB
CCB
Beta_Blocker
ACE
0.016
0.020
0.024
0.028
0.032
Cumulative Incidence Rate at 1 Year After Baseline

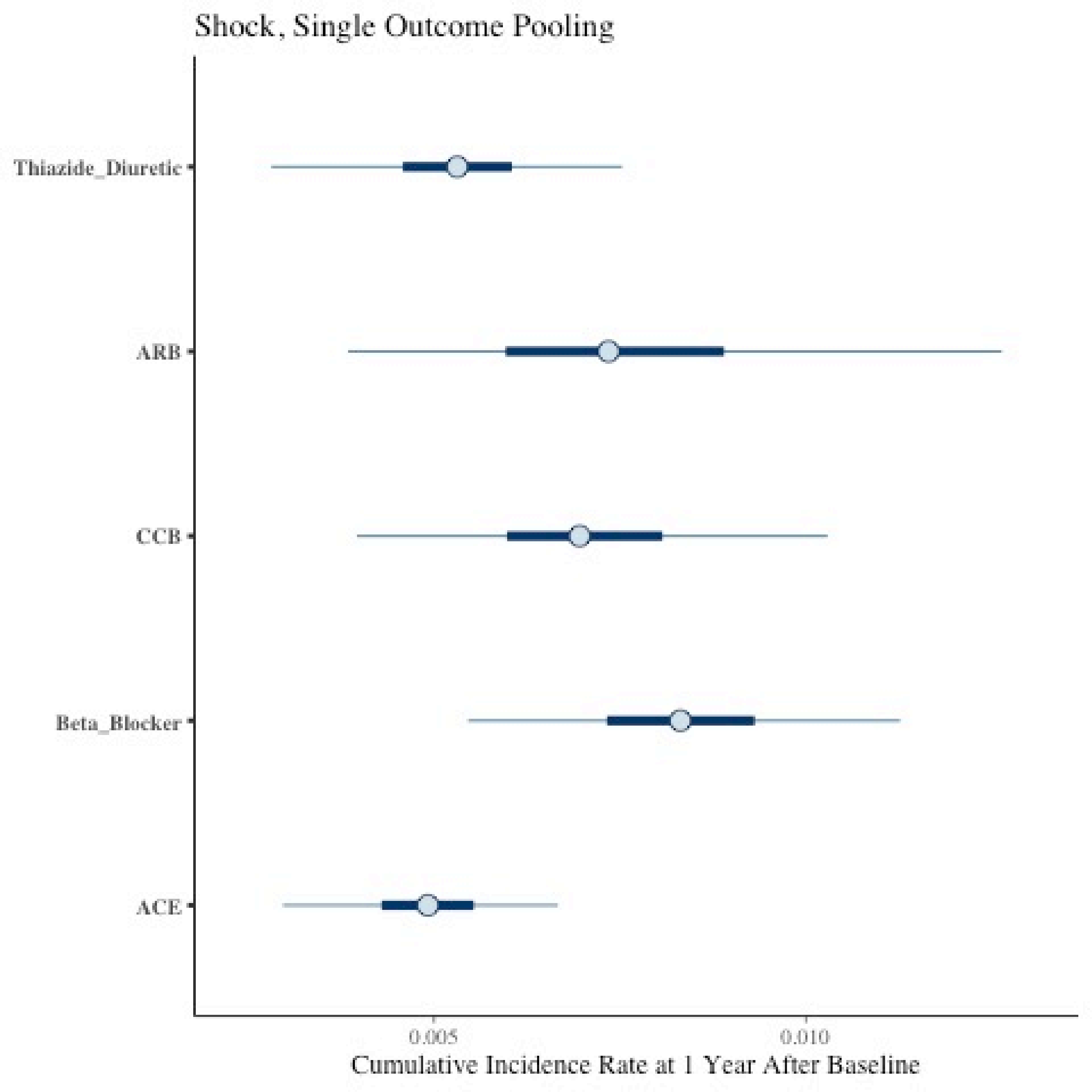

Shock, Single Outcome Pooling
Thiazide_Diuretic
ARB
CCB
Beta_Blocker
ACE
0.005
0.010
Cumulative Incidence Rate at 1 Year After Baseline

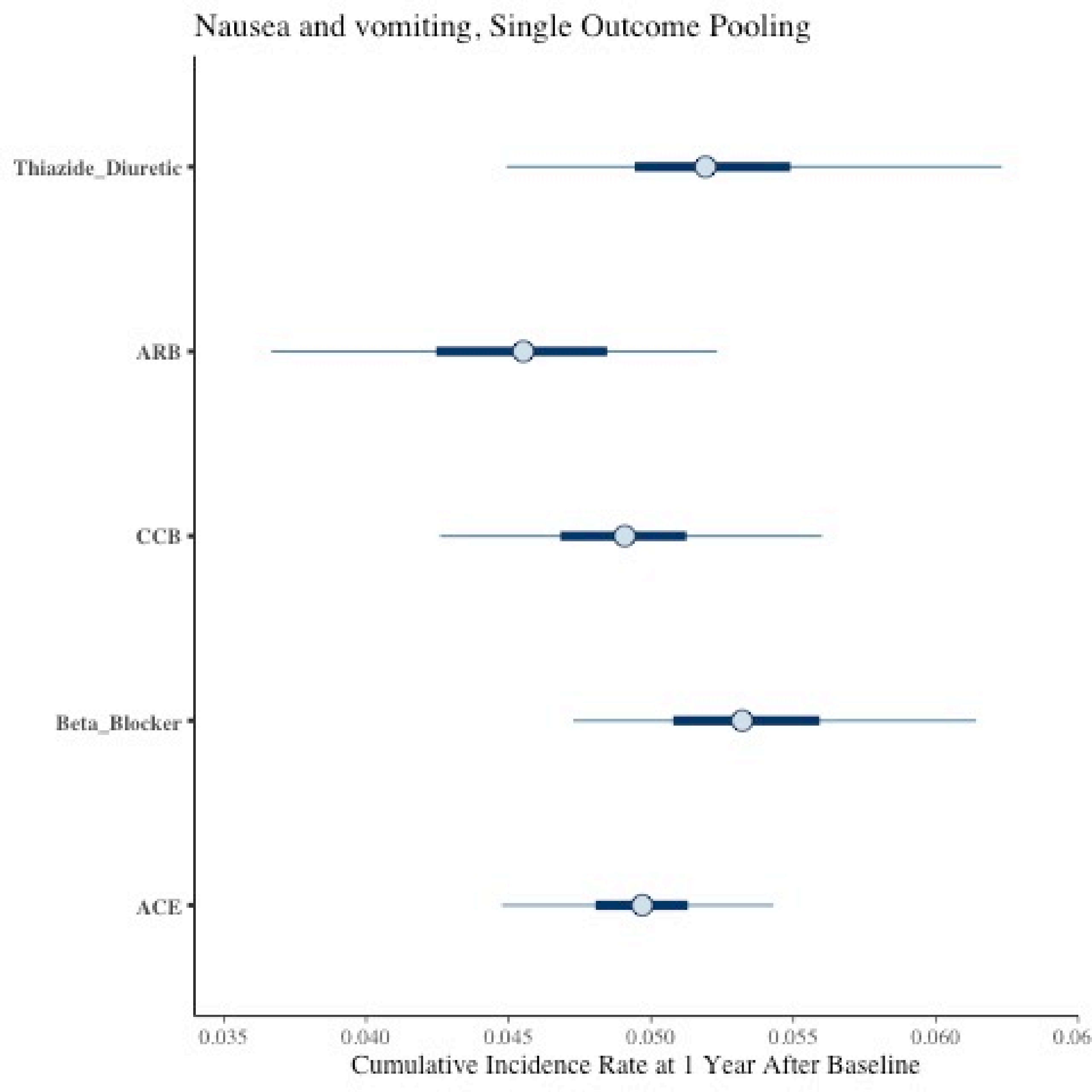

Nausea and vomiting, Single Outcome Pooling
Thiazide_Diuretic
ARB
CCB
Beta_Blocker
ACE
0.035
0.040
0.045
0.050
0.055
0.060
Cumulative Incidence Rate at 1 Year After Baseline

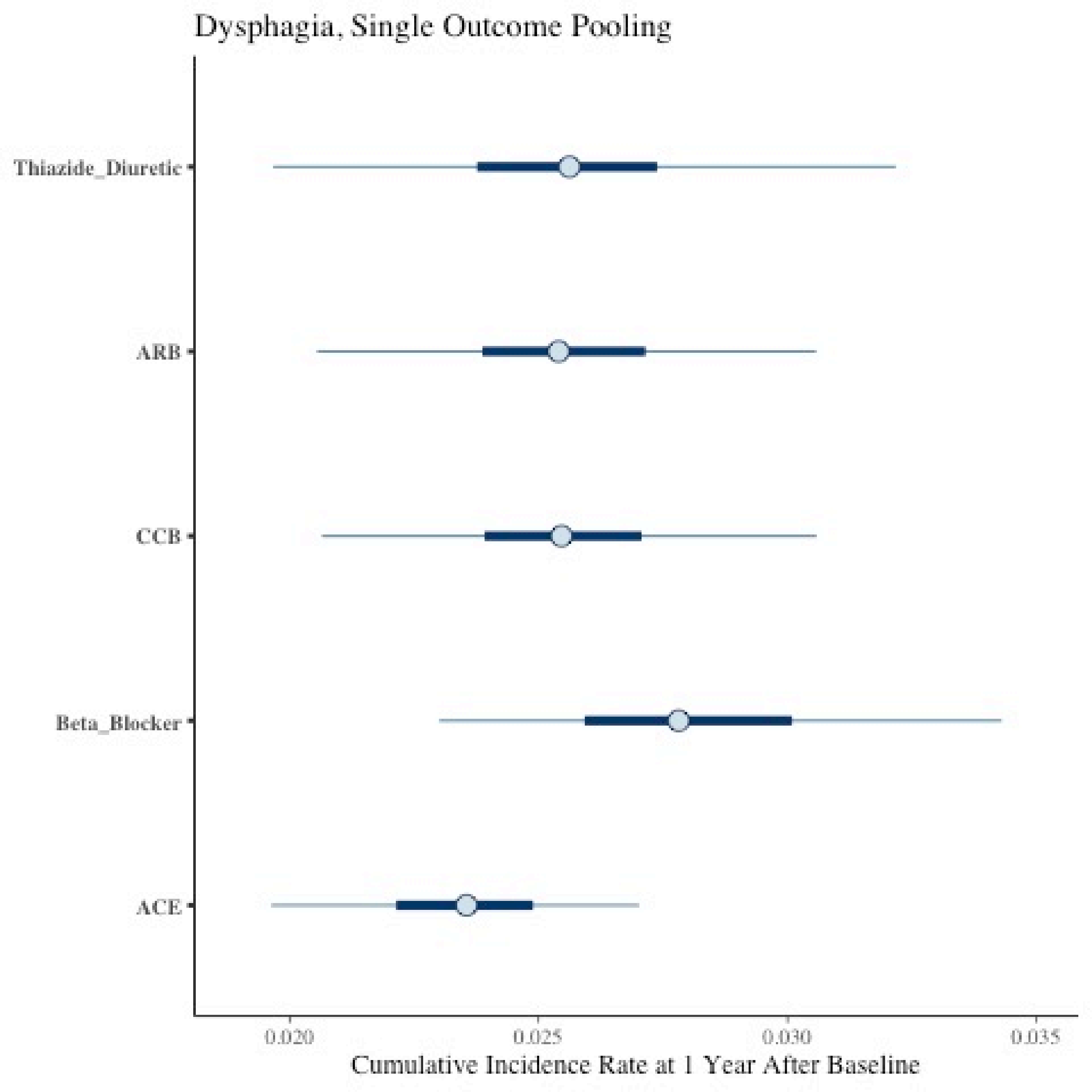
Dysphagia, Single Outcome Pooling
Thiazide_Diuretic
ARB
CCB
Beta_Blocker
ACE
0.020
0.025
0.030
0.035
Cumulative Incidence Rate at 1 Year After Baseline

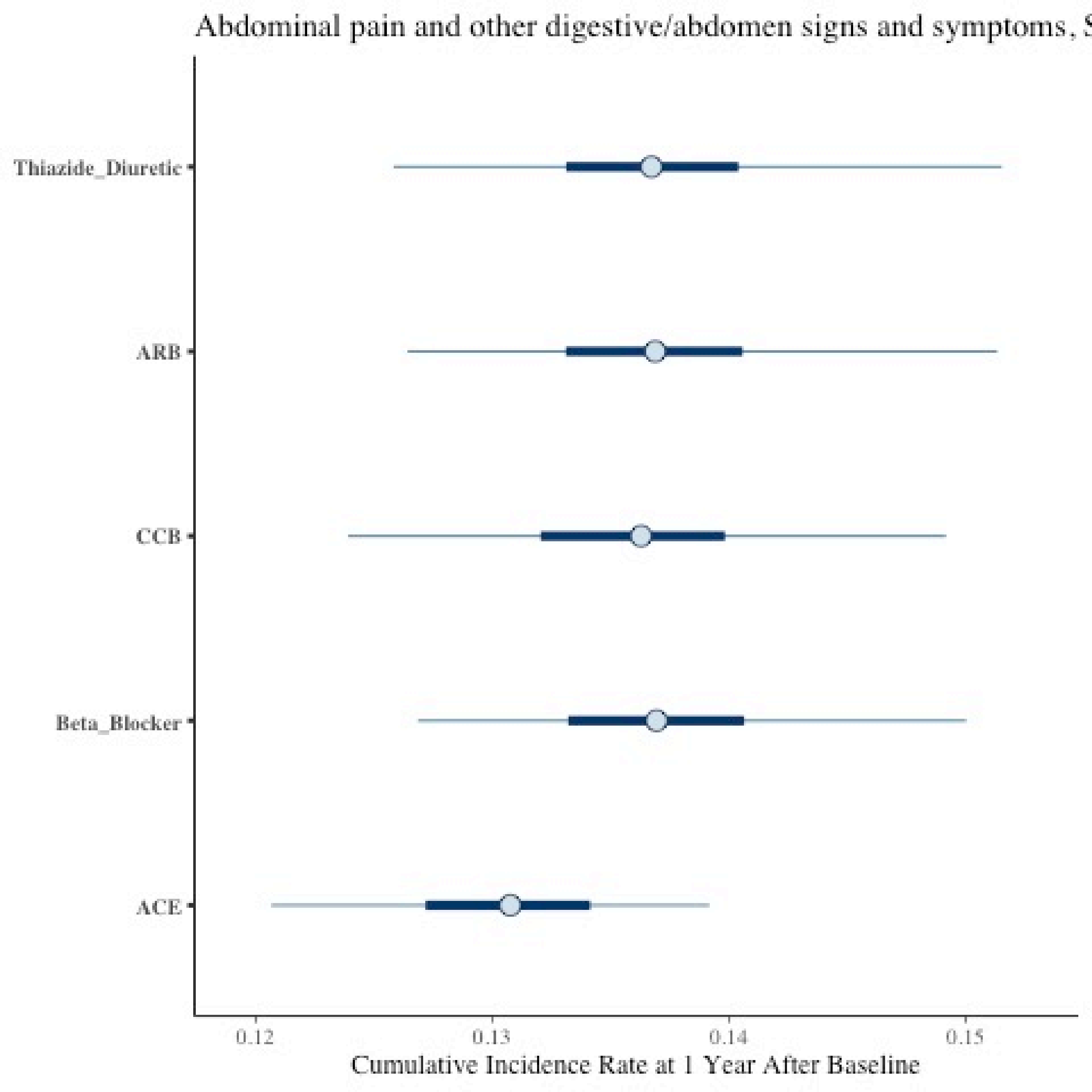

Abdominal pain and other digestive/abdomen signs and symptoms, S
Thiazide_Diuretic
ARB
CCB
Beta_Blocker
ACE
0.12
0.13
0.14
0.15
Cumulative Incidence Rate at 1 Year After Baseline

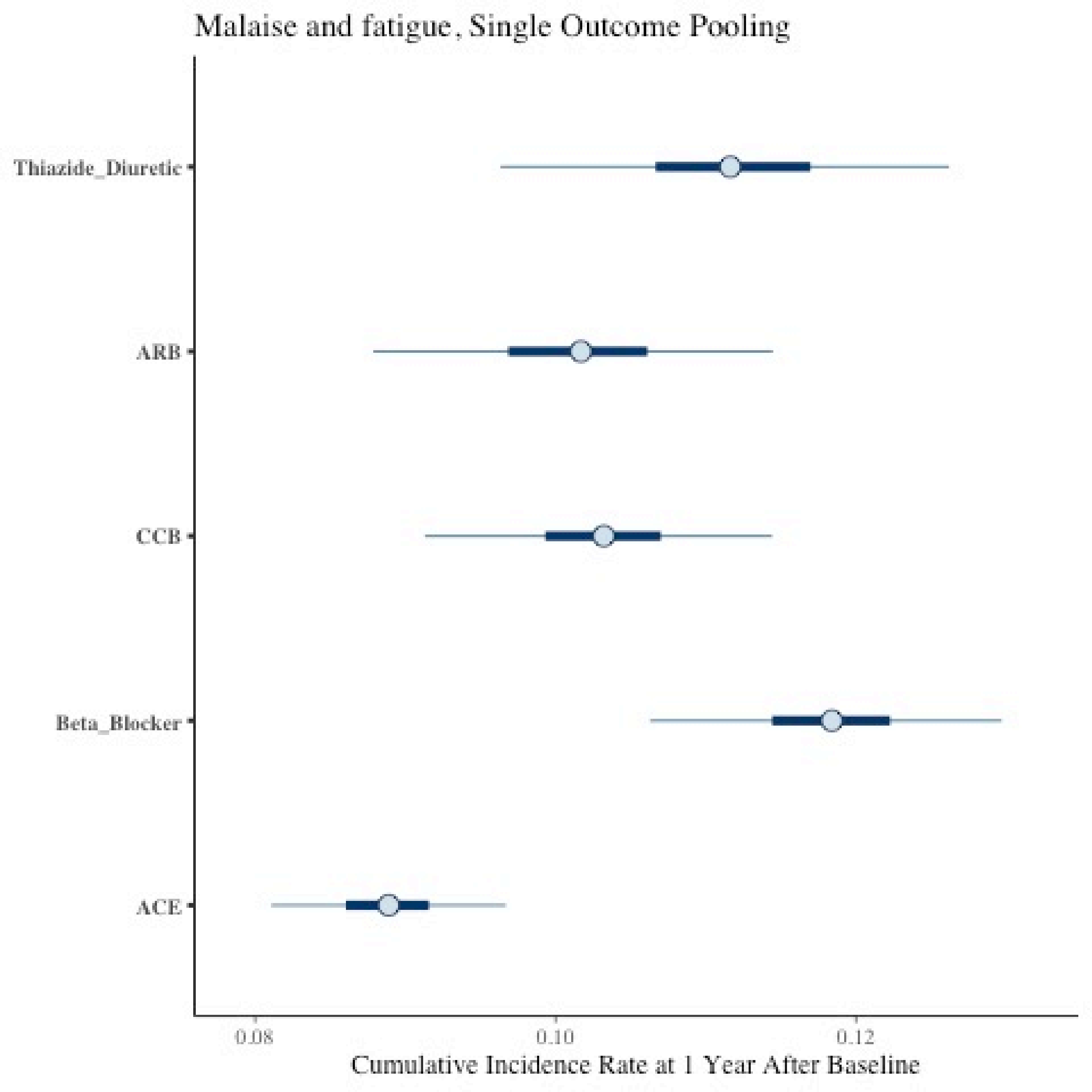
Malaise and fatigue, Single Outcome Pooling
Thiazide_Diuretic
ARB
CCB
Beta_Blocker
ACE
0.08
0.10
0.12
Cumulative Incidence Rate at 1 Year After Baseline

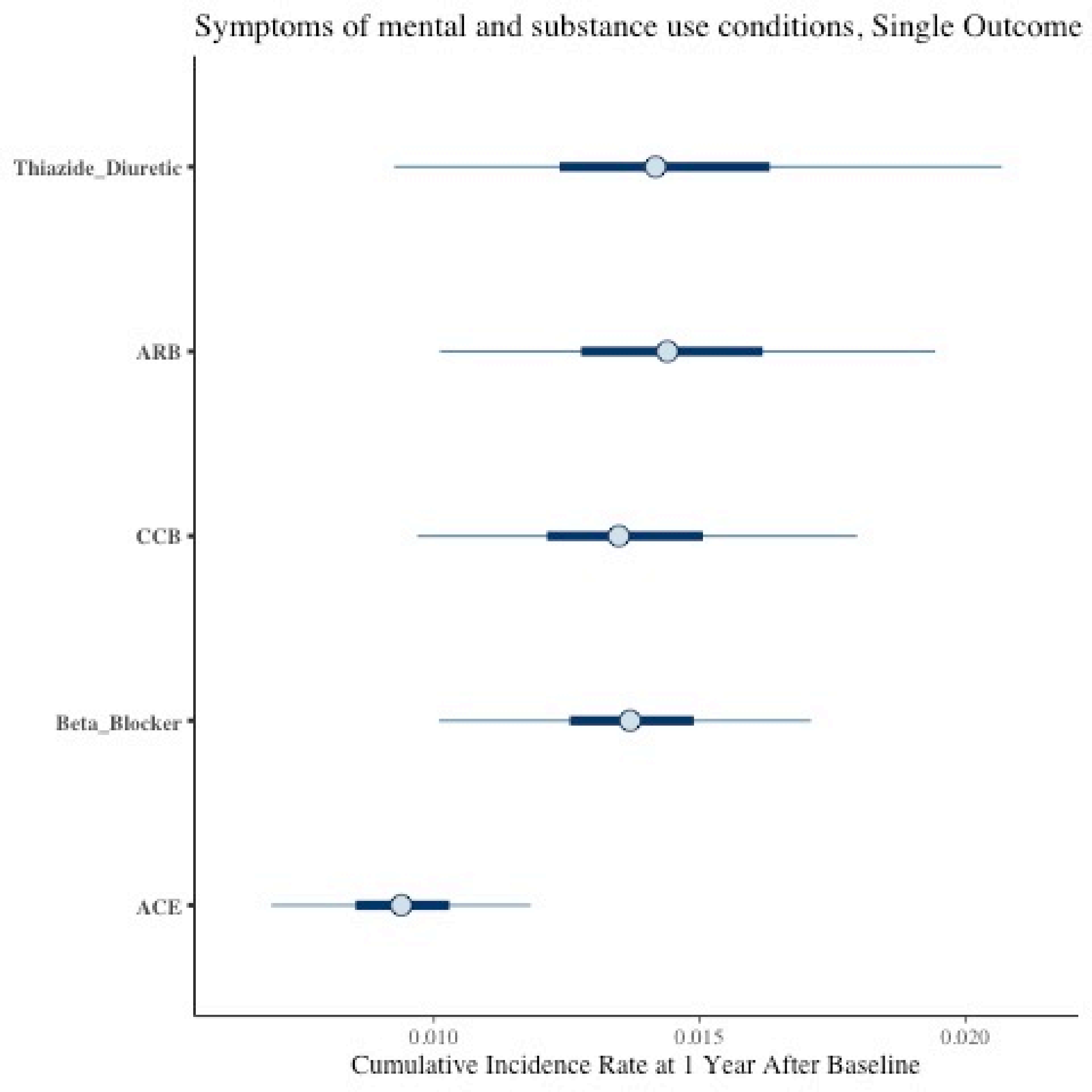

Symptoms of mental and substance use conditions, Single Outcome
Thiazide_Diuretic
ARB
CCB
Beta_Blocker
ACE
0.010
0.015
0.020
Cumulative Incidence Rate at 1 Year After Baseline

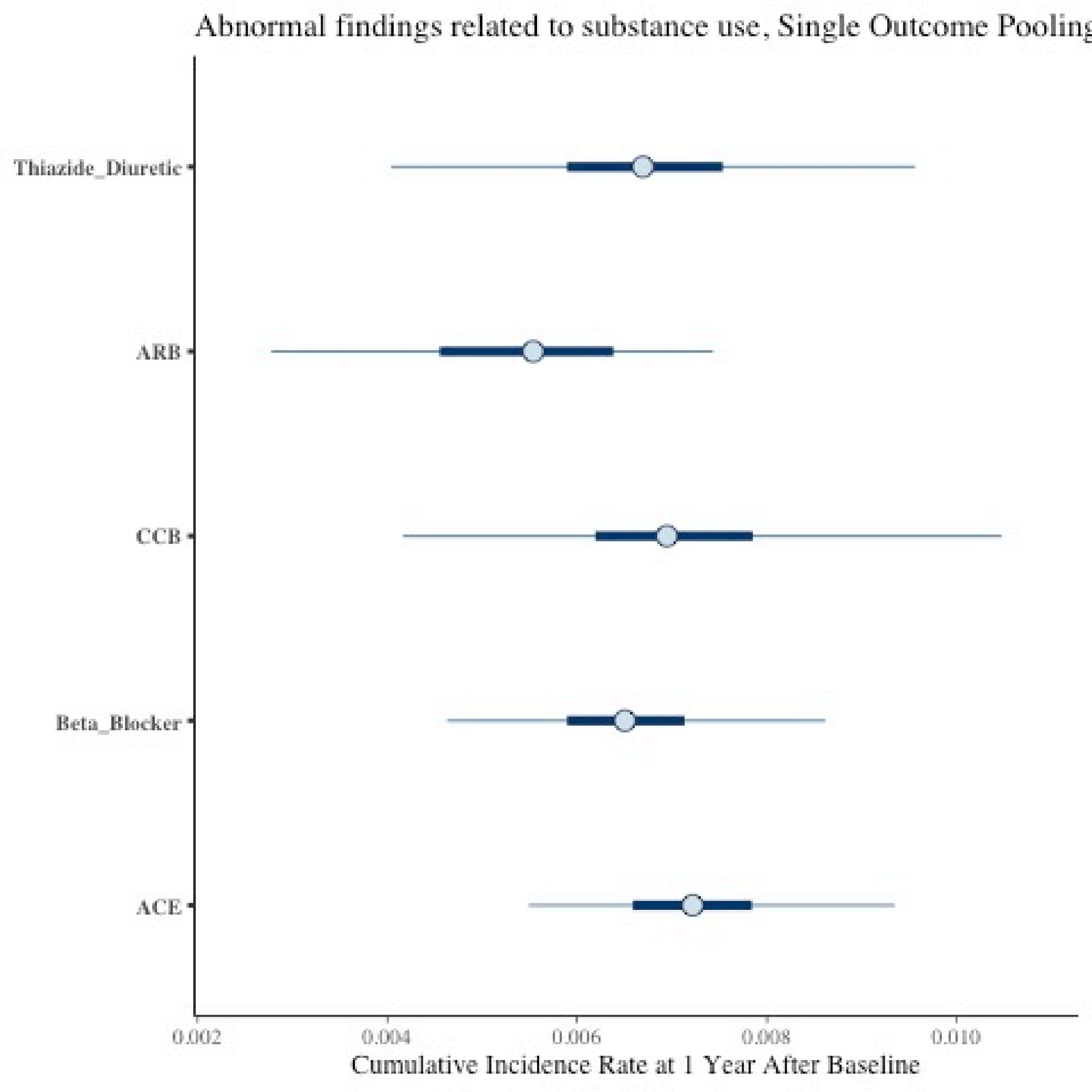

Abnormal findings related to substance use, Single Outcome Pooling
Thiazide_Diuretic
ARB
CCB
Beta_Blocker
ACE
0.002
0.004
0.006
0.008
0.010
Cumulative Incidence Rate at 1 Year After Baseline

APPENDIX 3

Forest Plots of Counterfactual Cumulative Incidence Rate Ratio Estimates Under the All Outcome Pooling Model From Section 2.3

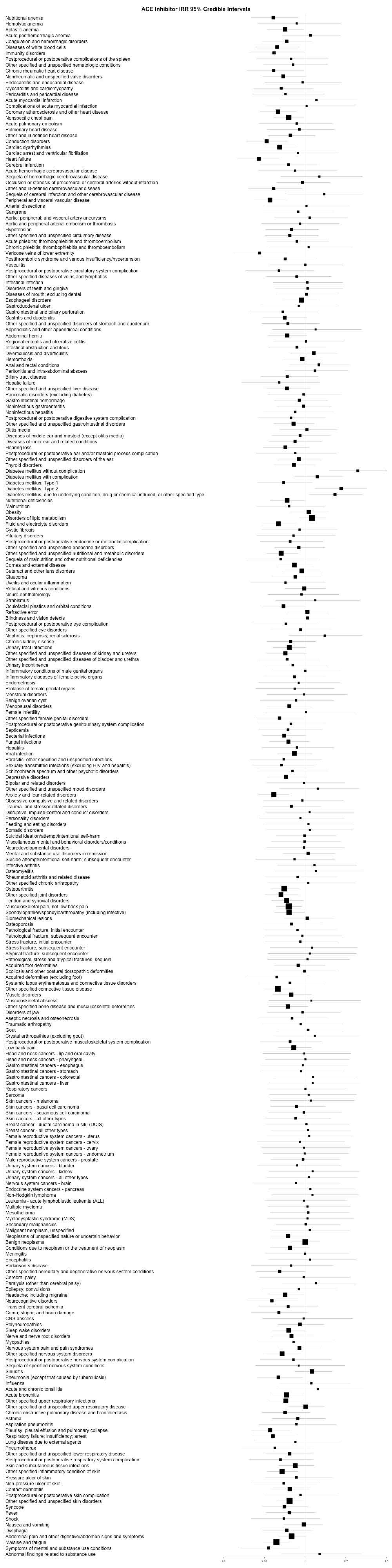
ACE Inhibitor IRR 95% Credible Intervals
Nutritional anemia
Hemolytic anemia
Aplastic anemia
Acute posthemorrhagic anemia
Coagulation and hemorrhagic disorders
Diseases of white blood cells
Immunity disorders
Postprocedural or postoperative complications of the spleen
Other specified and unspecified hematologic conditions
Chronic rheumatic heart disease
Nonrheumatic and unspecified valve disorders
Endocarditis and endocardial disease
Myocarditis and cardiomyopathy
Pericarditis and pericardial disease
Acute myocardial infarction
Complications of acute myocardial infarction
Coronary atherosclerosis and other heart disease
Nonspecific chest pain
Acute pulmonary embolism
Pulmonary heart disease
Other and ill-defined heart disease
Conduction disorders
Cardiac dysrhythmias
Cardiac arrest and ventricular fibrillation
Heart failure
Cerebral infarction
Acute hemorrhagic cerebrovascular disease
Sequela of hemorrhagic cerebrovascular disease
Occlusion or stenosis of precerebral or cerebral arteries without infarction
Other and ill-defined cerebrovascular disease
Sequela of cerebral infarction and other cerebrovascular disease
Peripheral and visceral vascular disease
Arterial dissections
Gangrene
Aortic; peripheral; and visceral artery aneurysms
Aortic and peripheral arterial embolism or thrombosis
Hypotension
Other specified and unspecified circulatory disease
Acute phlebitis; thrombophlebitis and thromboembolism
Chronic phlebitis; thrombophlebitis and thromboembolism
Varicose veins of lower extremity
Postthrombotic syndrome and venous insufficiency/hypertension
Vasculitis
Postprocedural or postoperative circulatory system complication
Other specified diseases of veins and lymphatics
Intestinal infection
Disorders of teeth and gingiva
Diseases of mouth; excluding dental
Esophageal disorders
Gastroduodenal ulcer
Gastrointestinal and biliary perforation
Gastritis and duodenitis
Other specified and unspecified disorders of stomach and duodenum
Appendicitis and other appendiceal conditions
Abdominal hernia
Regional enteritis and ulcerative colitis
Intestinal obstruction and ileus
Diverticulosis and diverticulitis
Hemorrhoids
Anal and rectal conditions
Peritonitis and intra-abdominal abscess
Biliary tract disease
Hepatic failure
Other specified and unspecified liver disease
Pancreatic disorders (excluding diabetes)
Gastrointestinal hemorrhage
Noninfectious gastroenteritis
Noninfectious hepatitis
Postprocedural or postoperative digestive system complication
Other specified and unspecified gastrointestinal disorders
Otitis media
Diseases of middle ear and mastoid (except otitis media)
Diseases of inner ear and related conditions
Hearing loss
Postprocedural or postoperative ear and/or mastoid process complication
Other specified and unspecified disorders of the ear
Thyroid disorders
Diabetes mellitus without complication
Diabetes mellitus with complication
Diabetes mellitus, Type 1
Diabetes mellitus, Type 2
Diabetes mellitus, due to underlying condition, drug or chemical induced, or other specified type
Nutritional deficiencies
Malnutrition
Obesity
Disorders of lipid metabolism
Fluid and electrolyte disorders
Cystic fibrosis
Pituitary disorders
Postprocedural or postoperative endocrine or metabolic complication
Other specified and unspecified endocrine disorders
Other specified and unspecified nutritional and metabolic disorders
Sequela of malnutrition and other nutritional deficiencies
Cornea and external disease
Cataract and other lens disorders
Glaucoma
Uveitis and ocular inflammation
Retinal and vitreous conditions
Neuro-ophthalmology
Strabismus
Oculofacial plastics and orbital conditions
Refractive error
Blindness and vision defects
Postprocedural or postoperative eye complication
Other specified eye disorders
Nephritis; nephrosis; renal sclerosis
Chronic kidney disease
Urinary tract infections
Other specified and unspecified diseases of kidney and ureters
Other specified and unspecified diseases of bladder and urethra
Urinary incontinence
Inflammatory conditions of male genital organs
Inflammatory diseases of female pelvic organs
Endometriosis
Prolapse of female genital organs
Menstrual disorders
Benign ovarian cyst
Menopausal disorders
Female infertility
Other specified female genital disorders
Postprocedural or postoperative genitourinary system complication
Septicemia
Bacterial infections
Fungal infections
Hepatitis
Viral infection
Parasitic, other specified and unspecified infections
Sexually transmitted infections (excluding HIV and hepatitis)
Schizophrenia spectrum and other psychotic disorders
Depressive disorders
Bipolar and related disorders
Other specified and unspecified mood disorders
Anxiety and fear-related disorders
Obsessive-compulsive and related disorders
Trauma- and stressor-related disorders
Disruptive, impulse-control and conduct disorders
Personality disorders
Feeding and eating disorders
Somatic disorders
Suicidal ideation/attempt/intentional self-harm
Miscellaneous mental and behavioral disorders/conditions
Neurodevelopmental disorders
Mental and substance use disorders in remission
Suicide attempt/intentional self-harm; subsequent encounter
Infective arthritis
Osteomyelitis
Rheumatoid arthritis and related disease
Other specified chronic arthropathy
Osteoarthritis
Other specified joint disorders
Tendon and synovial disorders
Musculoskeletal pain, not low back pain
Spondylopathies/spondyloarthropathy (including infective)
Biomechanical lesions
Osteoporosis
Pathological fracture, initial encounter
Pathological fracture, subsequent encounter
Stress fracture, initial encounter
Stress fracture, subsequent encounter
Atypical fracture, subsequent encounter
Pathological, stress and atypical fractures, sequela
Acquired foot deformities
Scoliosis and other postural dorsopathic deformities
Acquired deformities (excluding foot)
Systemic lupus erythematosus and connective tissue disorders
Other specified connective tissue disease
Muscle disorders
Musculoskeletal abscess
Other specified bone disease and musculoskeletal deformities
Disorders of jaw
Aseptic necrosis and osteonecrosis
Traumatic arthropathy
Gout
Crystal arthropathies (excluding gout)
Postprocedural or postoperative musculoskeletal system complication
Low back pain
Head and neck cancers - lip and oral cavity
Head and neck cancers - pharyngeal
Gastrointestinal cancers - esophagus
Gastrointestinal cancers - stomach
Gastrointestinal cancers - colorectal
Gastrointestinal cancers - liver
Respiratory cancers
Sarcoma
Skin cancers - melanoma
Skin cancers - basal cell carcinoma
Skin cancers - squamous cell carcinoma
Skin cancers - all other types
Breast cancer - ductal carcinoma in situ (DCIS)
Breast cancer - all other types
Female reproductive system cancers - uterus
Female reproductive system cancers - cervix
Female reproductive system cancers - ovary
Female reproductive system cancers - endometrium
Male reproductive system cancers - prostate
Urinary system cancers - bladder
Urinary system cancers - kidney
Urinary system cancers - all other types
Nervous system cancers - brain
Endocrine system cancers - pancreas
Non-Hodgkin lymphoma
Leukemia - acute lymphoblastic leukemia (ALL)
Multiple myeloma
Mesothelioma
Myelodysplastic syndrome (MDS)
Secondary malignancies
Malignant neoplasm, unspecified
Neoplasms of unspecified nature or uncertain behavior
Benign neoplasms
Conditions due to neoplasm or the treatment of neoplasm
Meningitis
Encephalitis
Parkinson`s disease
Other specified hereditary and degenerative nervous system conditions
Cerebral palsy
Paralysis (other than cerebral palsy)
Epilepsy; convulsions
Headache; including migraine
Neurocognitive disorders
Transient cerebral ischemia
Coma; stupor; and brain damage
CNS abscess
Polyneuropathies
Sleep wake disorders
Nerve and nerve root disorders
Myopathies
Nervous system pain and pain syndromes
Other specified nervous system disorders
Postprocedural or postoperative nervous system complication
Sequela of specified nervous system conditions
Sinusitis
Pneumonia (except that caused by tuberculosis)
Influenza
Acute and chronic tonsillitis
Acute bronchitis
Other specified upper respiratory infections
Other specified and unspecified upper respiratory disease
Chronic obstructive pulmonary disease and bronchiectasis
Asthma
Aspiration pneumonitis
Pleurisy, pleural effusion and pulmonary collapse
Respiratory failure; insufficiency; arrest
Lung disease due to external agents
Pneumothorax
Other specified and unspecified lower respiratory disease
Postprocedural or postoperative respiratory system complication
Skin and subcutaneous tissue infections
Other specified inflammatory condition of skin
Pressure ulcer of skin
Non-pressure ulcer of skin
Contact dermatitis
Postprocedural or postoperative skin complication
Other specified and unspecified skin disorders
Syncope
Fever
Shock
Nausea and vomiting
Dysphagia
Abdominal pain and other digestive/abdomen signs and symptoms
Malaise and fatigue
Symptoms of mental and substance use conditions
Abnormal findings related to substance use
0.5
0.75
1
1.25
1.5

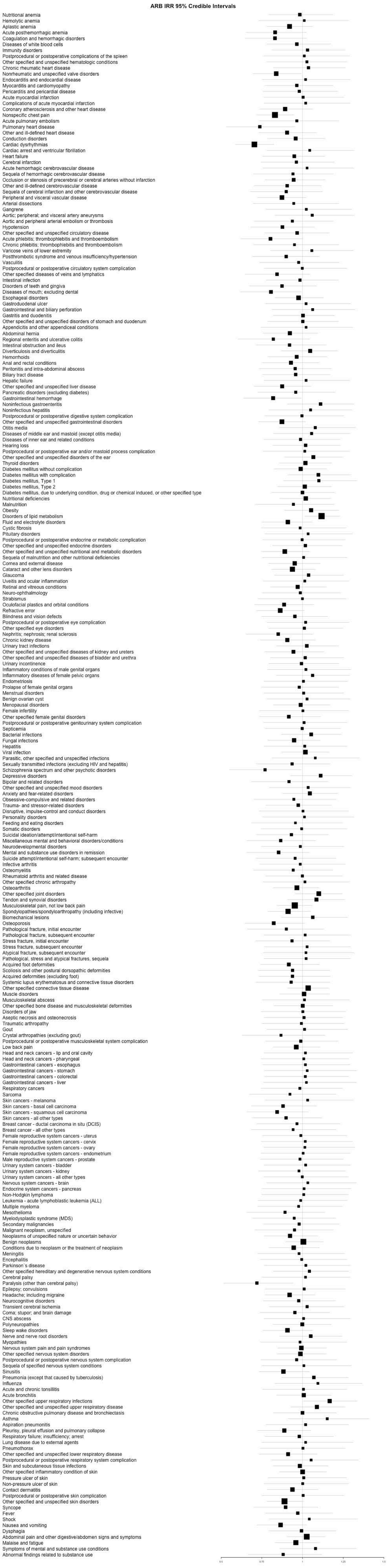
ARB IRR 95% Credible Intervals
Nutritional anemia
Hemolytic anemia
Aplastic anemia
Acute posthemorrhagic anemia
Coagulation and hemorrhagic disorders
Diseases of white blood cells
Immunity disorders
Postprocedural or postoperative complications of the spleen
Other specified and unspecified hematologic conditions
Chronic rheumatic heart disease
Nonrheumatic and unspecified valve disorders
Endocarditis and endocardial disease
Myocarditis and cardiomyopathy
Pericarditis and pericardial disease
Acute myocardial infarction
Complications of acute myocardial infarction
Coronary atherosclerosis and other heart disease
Nonspecific chest pain
Acute pulmonary embolism
Pulmonary heart disease
Other and ill-defined heart disease
Conduction disorders
Cardiac dysrhythmias
Cardiac arrest and ventricular fibrillation
Heart failure
Cerebral infarction
Acute hemorrhagic cerebrovascular disease
Sequela of hemorrhagic cerebrovascular disease
Occlusion or stenosis of precerebral or cerebral arteries without infarction
Other and ill-defined cerebrovascular disease
Sequela of cerebral infarction and other cerebrovascular disease
Peripheral and visceral vascular disease
Arterial dissections
Gangrene
Aortic; peripheral; and visceral artery aneurysms
Aortic and peripheral arterial embolism or thrombosis
Hypotension
Other specified and unspecified circulatory disease
Acute phlebitis; thrombophlebitis and thromboembolism
Chronic phlebitis; thrombophlebitis and thromboembolism
Varicose veins of lower extremity
Postthrombotic syndrome and venous insufficiency/hypertension
Vasculitis
Postprocedural or postoperative circulatory system complication
Other specified diseases of veins and lymphatics
Intestinal infection
Disorders of teeth and gingiva
Diseases of mouth; excluding dental
Esophageal disorders
Gastroduodenal ulcer
Gastrointestinal and biliary perforation
Gastritis and duodenitis
Other specified and unspecified disorders of stomach and duodenum
Appendicitis and other appendiceal conditions
Abdominal hernia
Regional enteritis and ulcerative colitis
Intestinal obstruction and ileus
Diverticulosis and diverticulitis
Hemorrhoids
Anal and rectal conditions
Peritonitis and intra-abdominal abscess
Biliary tract disease
Hepatic failure
Other specified and unspecified liver disease
Pancreatic disorders (excluding diabetes)
Gastrointestinal hemorrhage
Noninfectious gastroenteritis
Noninfectious hepatitis
Postprocedural or postoperative digestive system complication
Other specified and unspecified gastrointestinal disorders
Otitis media
Diseases of middle ear and mastoid (except otitis media)
Diseases of inner ear and related conditions
Hearing loss
Postprocedural or postoperative ear and/or mastoid process complication
Other specified and unspecified disorders of the ear
Thyroid disorders
Diabetes mellitus without complication
Diabetes mellitus with complication
Diabetes mellitus, Type 1
Diabetes mellitus, Type 2
Diabetes mellitus, due to underlying condition, drug or chemical induced, or other specified type
Nutritional deficiencies
Malnutrition
Obesity
Disorders of lipid metabolism
Fluid and electrolyte disorders
Cystic fibrosis
Pituitary disorders
Postprocedural or postoperative endocrine or metabolic complication
Other specified and unspecified endocrine disorders
Other specified and unspecified nutritional and metabolic disorders
Sequela of malnutrition and other nutritional deficiencies
Cornea and external disease
Cataract and other lens disorders
Glaucoma
Uveitis and ocular inflammation
Retinal and vitreous conditions
Neuro-ophthalmology
Strabismus
Oculofacial plastics and orbital conditions
Refractive error
Blindness and vision defects
Postprocedural or postoperative eye complication
Other specified eye disorders
Nephritis; nephrosis; renal sclerosis
Chronic kidney disease
Urinary tract infections
Other specified and unspecified diseases of kidney and ureters
Other specified and unspecified diseases of bladder and urethra
Urinary incontinence
Inflammatory conditions of male genital organs
Inflammatory diseases of female pelvic organs
Endometriosis
Prolapse of female genital organs
Menstrual disorders
Benign ovarian cyst
Menopausal disorders
Female infertility
Other specified female genital disorders
Postprocedural or postoperative genitourinary system complication
Septicemia
Bacterial infections
Fungal infections
Hepatitis
Viral infection
Parasitic, other specified and unspecified infections
Sexually transmitted infections (excluding HIV and hepatitis)
Schizophrenia spectrum and other psychotic disorders
Depressive disorders
Bipolar and related disorders
Other specified and unspecified mood disorders
Anxiety and fear-related disorders
Obsessive-compulsive and related disorders
Trauma- and stressor-related disorders
Disruptive, impulse-control and conduct disorders
Personality disorders
Feeding and eating disorders
Somatic disorders
Suicidal ideation/attempt/intentional self-harm
Miscellaneous mental and behavioral disorders/conditions
Neurodevelopmental disorders
Mental and substance use disorders in remission
Suicide attempt/intentional self-harm; subsequent encounter
Infective arthritis
Osteomyelitis
Rheumatoid arthritis and related disease
Other specified chronic arthropathy
Osteoarthritis
Other specified joint disorders
Tendon and synovial disorders
Musculoskeletal pain, not low back pain
Spondylopathies/spondyloarthropathy (including infective)
Biomechanical lesions
Osteoporosis
Pathological fracture, initial encounter
Pathological fracture, subsequent encounter
Stress fracture, initial encounter
Stress fracture, subsequent encounter
Atypical fracture, subsequent encounter
Pathological, stress and atypical fractures, sequela
Acquired foot deformities
Scoliosis and other postural dorsopathic deformities
Acquired deformities (excluding foot)
Systemic lupus erythematosus and connective tissue disorders
Other specified connective tissue disease
Muscle disorders
Musculoskeletal abscess
Other specified bone disease and musculoskeletal deformities
Disorders of jaw
Aseptic necrosis and osteonecrosis
Traumatic arthropathy
Gout
Crystal arthropathies (excluding gout)
Postprocedural or postoperative musculoskeletal system complication
Low back pain
Head and neck cancers - lip and oral cavity
Head and neck cancers - pharyngeal
Gastrointestinal cancers - esophagus
Gastrointestinal cancers - stomach
Gastrointestinal cancers - colorectal
Gastrointestinal cancers - liver
Respiratory cancers
Sarcoma
Skin cancers - melanoma
Skin cancers - basal cell carcinoma
Skin cancers - squamous cell carcinoma
Skin cancers - all other types
Breast cancer - ductal carcinoma in situ (DCIS)
Breast cancer - all other types
Female reproductive system cancers - uterus
Female reproductive system cancers - cervix
Female reproductive system cancers - ovary
Female reproductive system cancers - endometrium
Male reproductive system cancers - prostate
Urinary system cancers - bladder
Urinary system cancers - kidney
Urinary system cancers - all other types
Nervous system cancers - brain
Endocrine system cancers - pancreas
Non-Hodgkin lymphoma
Leukemia - acute lymphoblastic leukemia (ALL)
Multiple myeloma
Mesothelioma
Myelodysplastic syndrome (MDS)
Secondary malignancies
Malignant neoplasm, unspecified
Neoplasms of unspecified nature or uncertain behavior
Benign neoplasms
Conditions due to neoplasm or the treatment of neoplasm
Meningitis
Encephalitis
Parkinson`s disease
Other specified hereditary and degenerative nervous system conditions
Cerebral palsy
Paralysis (other than cerebral palsy)
Epilepsy; convulsions
Headache; including migraine
Neurocognitive disorders
Transient cerebral ischemia
Coma; stupor; and brain damage
CNS abscess
Polyneuropathies
Sleep wake disorders
Nerve and nerve root disorders
Myopathies
Nervous system pain and pain syndromes
Other specified nervous system disorders
Postprocedural or postoperative nervous system complication
Sequela of specified nervous system conditions
Sinusitis
Pneumonia (except that caused by tuberculosis)
Influenza
Acute and chronic tonsillitis
Acute bronchitis
Other specified upper respiratory infections
Other specified and unspecified upper respiratory disease
Chronic obstructive pulmonary disease and bronchiectasis
Asthma
Aspiration pneumonitis
Pleurisy, pleural effusion and pulmonary collapse
Respiratory failure; insufficiency; arrest
Lung disease due to external agents
Pneumothorax
Other specified and unspecified lower respiratory disease
Postprocedural or postoperative respiratory system complication
Skin and subcutaneous tissue infections
Other specified inflammatory condition of skin
Pressure ulcer of skin
Non-pressure ulcer of skin
Contact dermatitis
Postprocedural or postoperative skin complication
Other specified and unspecified skin disorders
Syncope
Fever
Shock
Nausea and vomiting
Dysphagia
Abdominal pain and other digestive/abdomen signs and symptoms
Malaise and fatigue
Symptoms of mental and substance use conditions
Abnormal findings related to substance use

Beta Blocker IRR 95% Credible Intervals

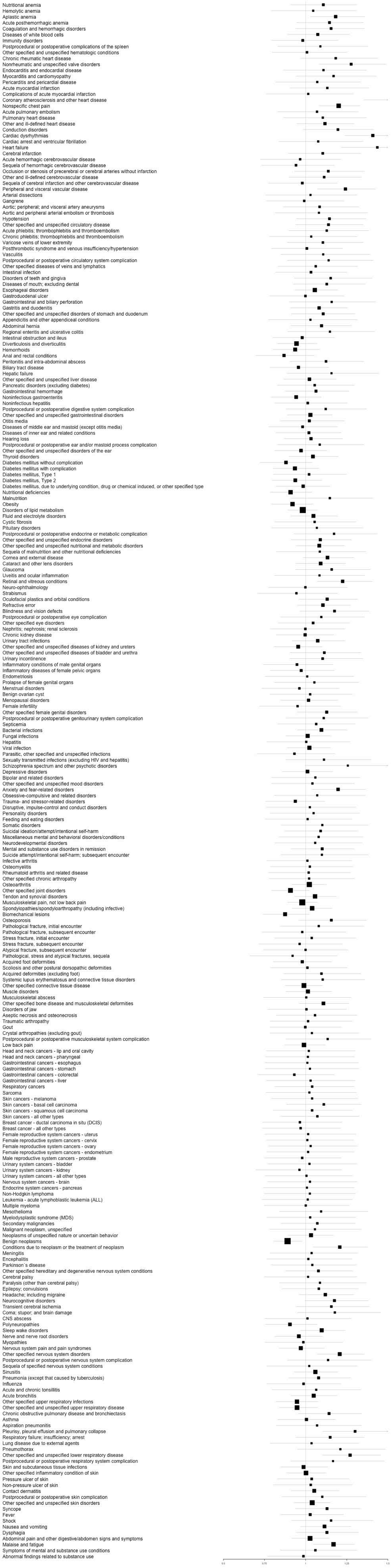

CCB IRR 95% Credible Intervals

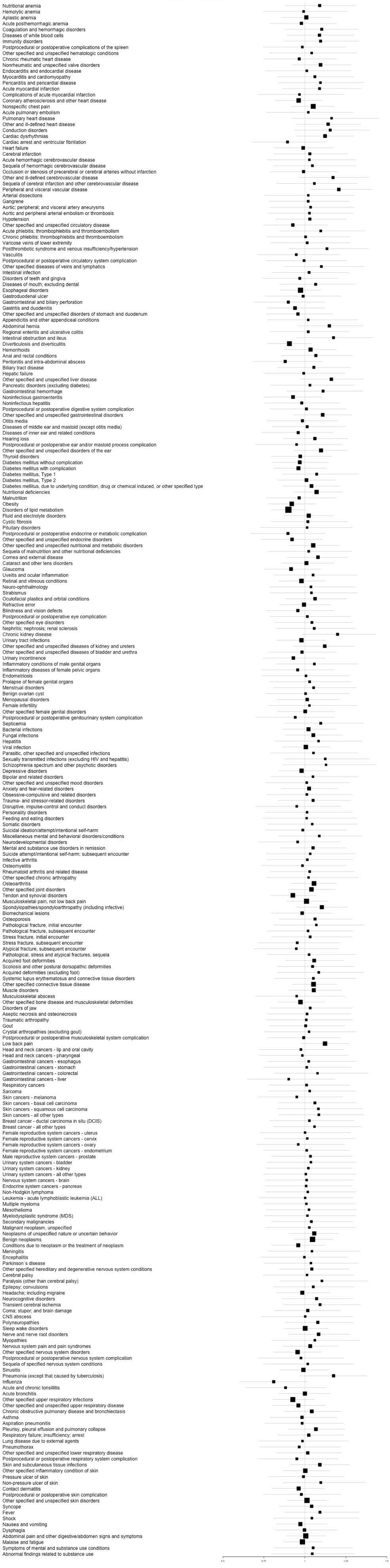

Thiazide IRR 95% Credible Intervals

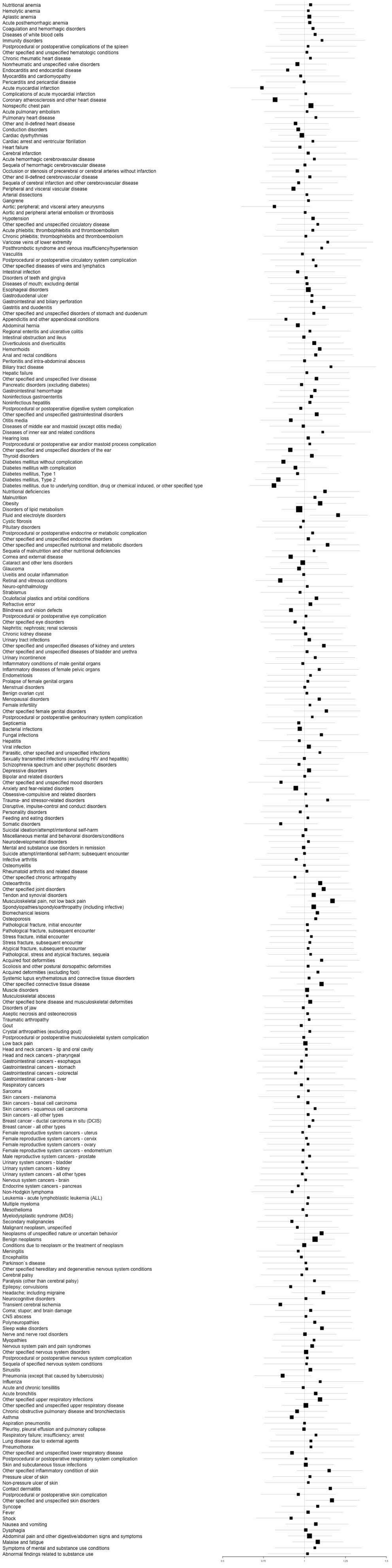

Supplement: Supplementary file 1 — Appendix S1 Supporting Information [file PDS-31-944-s001.pdf]
